# Exploring the Far Side of Mobile Health: Information Security and Privacy of Mobile Health Apps on iOS and Android

**DOI:** 10.2196/mhealth.3672

**Published:** 2015-01-19

**Authors:** Tobias Dehling, Fangjian Gao, Stephan Schneider, Ali Sunyaev

**Affiliations:** ^1^Department of Information SystemsFaculty of Management, Economics and Social SciencesUniversity of CologneCologneGermany

**Keywords:** mobile health, mobile apps, data security, software and application security, patient privacy, health information technology

## Abstract

**Background:**

Mobile health (mHealth) apps aim at providing seamless access to tailored health information technology and have the potential to alleviate global health burdens. Yet, they bear risks to information security and privacy because users need to reveal private, sensitive medical information to redeem certain benefits. Due to the plethora and diversity of available mHealth apps, implications for information security and privacy are unclear and complex.

**Objective:**

The objective of this study was to establish an overview of mHealth apps offered on iOS and Android with a special focus on potential damage to users through information security and privacy infringements.

**Methods:**

We assessed apps available in English and offered in the categories “Medical” and “Health & Fitness” in the iOS and Android App Stores. Based on the information retrievable from the app stores, we established an overview of available mHealth apps, tagged apps to make offered information machine-readable, and clustered the discovered apps to identify and group similar apps. Subsequently, information security and privacy implications were assessed based on health specificity of information available to apps, potential damage through information leaks, potential damage through information manipulation, potential damage through information loss, and potential value of information to third parties.

**Results:**

We discovered 24,405 health-related apps (iOS; 21,953; Android; 2452). Absence or scarceness of ratings for 81.36% (17,860/21,953) of iOS and 76.14% (1867/2452) of Android apps indicates that less than a quarter of mHealth apps are in more or less widespread use. Clustering resulted in 245 distinct clusters, which were consolidated into 12 app archetypes grouping clusters with similar assessments of potential damage through information security and privacy infringements. There were 6426 apps that were excluded during clustering. The majority of apps (95.63%, 17,193/17,979; of apps) pose at least some potential damage through information security and privacy infringements. There were 11.67% (2098/17,979) of apps that scored the highest assessments of potential damages.

**Conclusions:**

Various kinds of mHealth apps collect and offer critical, sensitive, private medical information, calling for a special focus on information security and privacy of mHealth apps. In order to foster user acceptance and trust, appropriate security measures and processes need to be devised and employed so that users can benefit from seamlessly accessible, tailored mHealth apps without exposing themselves to the serious repercussions of information security and privacy infringements.

##  Introduction

### mHealth Apps

Mobile health (mHealth) leverages various wireless technologies to provide health-related information and services on diverse mobile devices and is a promising subset of health information technology (IT) [1-6]. mHealth has the potential to alleviate global health burdens due to rising dissemination of mobile devices, standardized and easy access to cloud or Internet services, and the possibility of affordable global deployment [4,7-9]. mHealth apps target, for instance, prevalent global diseases [10,11] and offer vital health information at an individual as well as population level [12]. On the other hand, users, albeit deeming access to health information and related services beneficial, are concerned with information security and privacy issues, and want to control access to their information [13-15].

Information security and privacy issues impede users’ willingness to share information [16,17], and render thus the promising benefits to be reaped from mHealth apps moot, in order to tailor offered information and services to users’ needs, mHealth apps require access to relevant personal health information. Thus, mHealth apps will only offer more general services or cannot be used at all if users are not willing to share their health information. Moreover, infringements of information security and privacy lead not only to leakage or manipulation of private, sensitive information, but make also serious consequences like worsened morbidity or death more likely [18].

### Mobile Devices for mHealth

Typical mobile devices for mHealth are smartphones and tablets [11], which are characterized by a rapidly rising market penetration and access to a wide range of embedded technology like sensors for audio, video, location, orientation, and acceleration [8,11,19,20]. The main platforms for mobile devices are Google’s Android and Apple’s iOS [8]. The associated app stores (Apple iTunes, Google Play) [21,22] offer a vast amount of mHealth apps. These mHealth apps provide a variety of functionality requiring access to different kinds of information and supporting users in different ways, for example, support for weight management, tracking of workouts or medication regimens, facilitation of physician patient communication, management of chronic diseases, or implementation of Web-based interventions [23].

Mobile devices and apps have been addressed from various perspectives, for instance, security aspects [24-26], privacy [18,27-29], software engineering [30-32], medical implications [33,34], hardware [19,35], or user implications [20,36,37]. In contrast, pertinent governmental regulations, for example, [38,39], and extant reviews of mHealth apps, for example, [10,11,40-55], focus mostly on functional aspects and utility of apps for specific diseases or health conditions. Information security and privacy of mHealth apps is only scarcely addressed by extant research. With respect to information security and privacy, extant research offers, to the best of our knowledge, neither clear analysis of the peculiarities that distinguish mHealth apps from “common apps” (eg, weather apps or games), nor of the differences distinguishing apps available from each other. In short, understanding of information security and privacy implications of mHealth apps is lacking and hard to grasp due to the diversity and range of mHealth apps available. In order to address this gap, the objective of our research is to establish an overview of mHealth apps offered on iOS and Android, with a special focus on potential damage to users through information security and privacy infringements.

Our research contributes to practice and the knowledge base by shedding light on information security and privacy of mHealth apps. Aside from providing an overview of available mHealth apps, we contribute to the scientific knowledge base by deepening the understanding of information security and privacy of mHealth apps. Instead of treating mHealth apps as a monolithic technology, we focus on the multi-facetted nature of mHealth apps and identify different mHealth app archetypes with respect to information security and privacy. For practical audiences, our work fosters awareness of information security and privacy implications of mHealth apps. Besides substantiating the need for attention to information security and privacy of mHealth apps, our work demonstrates that mHealth apps are of a diverse nature and require tailored attention to information security and privacy. For developers and end users of mHealth apps, the identification of mHealth app archetypes is especially useful to recognize where and understand when attention to information security and privacy is of particular importance. Deepening the understanding of information security and privacy of mHealth apps is an important step toward realization of the promising potential of mHealth apps to transform and improve the health care environment [2].

## Methods

### App Discovery

We surveyed English language mHealth apps in the official iOS and Android App Stores. App stores organize their offerings in categories (eg, Books, Games, and News). We selected apps from the Medical and Health & Fitness categories, offered in both stores in May 2013. The iOS app store lists all apps by category and offers the desired information in plain hypertext markup language (HTML), enabling us to automatically parse app information to extract data. The Android App Store employs dynamically generated HTML pages so that the HTML texts displayed in the browser do not convey useful information, which is dynamically loaded from an underlying database. Hence, we used a third party open-source interface for retrieving app information [56]. However, Google imposes various constraints on app store access [8,57]; for instance, only a maximum of 500 apps is returned per search request, even if more apps match the query. Our approach for Android app discovery builds search queries based on words from a publicly available English word list [58] appended once with the string “medical” and once with the string “health”. Supplemented with missing health-related words and phrases identified during app tagging (see next paragraph), the word list consists of 111,632 distinct words and phrases (see Multimedia Appendices 1 or 2).

Apps that were not available in English, did not have an English description, or were not health-related, despite being offered in the categories Medical or Health & Fitness (eg, apps offering wallpapers), were excluded from further assessment. We employed tagging, that is, assignment of arbitrary terms describing an object to that object, to filter the initially discovered apps (iOS, 32,614; Android, 4632). Instead of assigning tags directly to an app, we assigned tags to corresponding strings in app descriptions. Only tags referring to health-related information collected by apps, health-related app purposes, handling of information, or other health-related app characteristics were used. For example, apps that provide medication-related functionality should be tagged with the tag “Medication”. Yet, app descriptions use different wording (eg, medication, pharmaceutical, or drug). Assigning tags to all encountered strings referring to medication reduces the number of redundant tags and establishes a corpus of string tag relationships that facilitates automated tagging of apps. Since extant research offered no clear guidance to determine cut-off points for manual tagging or the number of required tag matches, cut-off points were determined according to the available data in group discussions of the authors. We manually tagged 200 frequently rated apps (100 Health & Fitness, 100 Medical). Based on this initial tag corpus, we employed string matching [59] to automatically tag the remaining apps. With this approach, apps that do not offer English descriptions or health-related functionality are not assigned any or assigned only a small number of tags, because tags are assigned based on English, health-related words. Apps not matched by at least four distinct tags were excluded from further assessment.

### App Clustering

#### Clustering Approach

App tagging created a machine-readable description of app functionality. Since all apps were tagged based on the same tag corpus, apps with similar characteristics are assigned similar tags. We clustered [60] apps based on their tags to aggregate the data and identify the various kinds of apps in our sample. We used a graph—a set of vertices that are connected by a set of edges [61]—to represent the apps and their tag relationships. Vertices represent apps and edges represent tags both vertices have in common.

For identification of clusters, we used a heuristic by Blondel et al [62], called Louvain method, which is based on modularity optimization. Modularity is a measure for cluster quality introduced by Newman and Girvan [63]. Basically, modularity measures the fraction of edges in the graph that connect vertices within the same cluster minus the expected value of connections within a cluster if edges were inserted at random. Hence, a higher modularity value indicates that detected clusters are less random. The Louvain method performed well in comparative analyses of clustering algorithms [64,65], has low runtime so that it breaks our dense app tag graph down into clusters within a feasible amount of time, and does not require a priori determination of the number of clusters to be discovered, which is unfeasible due to the large numbers of apps, tags, and possible combinations. The Louvain method is an agglomerative clustering algorithm [60] that runs in multiple iterations until a maximum of modularity is reached [62]. Required algorithms were implemented in the programming languages PHP and Java. The Java library JGraphT [66] was used to represent graphs. The relational database management system MySQL was used for data management.

#### Cluster Assessment

Health IT faces various threats, for instance, intentional and unintentional disclosure or manipulation of information through insiders or outsiders, user errors, maintenance errors, software failures, or hardware failures, as well as environmental threats [67-70]. If such threats materialize, users will be in harms’ way. Based on extant research on information security and privacy in health care [68,71-79], we assess information security and privacy implications according to five characteristics: (1) health specificity of information available to apps, (2) potential damage through information leaks, (3) potential damage through information manipulation (change), (4) potential damage through information loss, and (5) potential value of information to third parties (Table 1). Cluster assessment is focused on risks specific to mHealth apps. Hence, risks associated with information ordinarily available to apps [24,27], like location information or device identifier, do not contribute to a more grave assessment.

Characteristic 1, health specificity of information available to apps, assesses whether the app has access to medical user information, access to other nonstandard information, or only access to standard information ordinarily available to apps like location information or device identifiers [24,27]. Characteristic 2 assesses the potential damage through information leaks, which can be classified as none, low, or high. Depending on offered functionality, health IT has access to information with low sensitivity like users’ height, weight, or common past illnesses and treatments like a cough or broken bones [71,72]. Other health IT offerings have, however, access to information with high sensitivity like abortions, mental illness, sexually transmitted diseases, HIV status, substance abuse, or genetic predispositions to disease [71-73]. Leaks of such information increase the likelihood of potential damage to users through socioeconomic repercussions [74], embarrassment or damage of reputation [68,71-73,75,76], social stigma [75], loss of affection or respect of family members [77], monetary repercussions through medical fraud (billing for treatments never rendered) or medical identity theft (obtainment of medical services with a fake medical identity) [68,73,74], more expensive insurance coverage or problems to obtain insurance coverage [71,72,75,77,78], or lessened employment possibilities [68,71,72,75,77]. Characteristic 3 assesses potential damage through information manipulation (change), possible values are none, low, or high. Potential damage through information manipulation was, for instance, assessed as low for information on eating patterns or past workouts. Manipulation of such information is inconvenient and undesirable, but poses only low potential damage. Potential damage through information manipulation was assessed as high for apps where information manipulation causes greater harm to users. If, for example, erroneous information is added to users’ information due to medical fraud, medical identity theft, negligence, malicious intent, or other threats, treatment can be based on erroneous information [68,73]. In addition, users’ quality of care is affected, potential for harm to health or death is increased, and later efforts to obtain medical, life, or disability insurance are impeded [68,73,74,76]. Potential damage through loss of information is assessed with characteristic 4, possible values are none, low, or high. Loss of uncritical information or information that can be restored was assessed as low. Loss of information was assessed as high in cases where, for instance, important information required for users’ care is no longer available [71,75,76]. Finally, the potential value of information for third parties is assessed by characteristic 5, possible values are none, low, or high. If apps have access to information valuable to third parties, infringements of information security and privacy are more likely because they are more rewarding for third parties. For mHealth apps that have only access to information commonly available to mobile apps, value was assessed as none. Value was assessed as low for collected information that is not directly useful to third parties, like unspecific information or information not attributable to users. On the other hand, information like insurance policy information, date of birth, or social security numbers is highly valuable to third parties; for instance, to commit medical identity theft or medical fraud [68,71,73]. Further uses of others’ private medical information that are not in the best interest of the data subject include the selling of medical information of celebrities [71], better fitting of insurance policies to insurees’ risks and selection of insurees [71,78,79], selection of healthy employees [68,71,78,79], or targeted marketing [71,72,78].

**Table 1:** Cluster assessment characteristics.

#	Name	Definition	Possible values
1	Specificity	Health specificity of information available to apps (eg, phone identifiers, eating habits, disease history)	Standard, nonstandard, medical
2	Leaks	Potential damage through leaks of information (eg, embarrassment, lessened employment prospects)	None, low, high
3	Change	Potential damage through manipulation (change) of information (eg, treatment errors)	None, low, high
4	Loss	Potential damage through loss of information (eg, loss of information important for treatment)	None, low, high
5	Value	Value of information to third parties (eg, medical identity theft, selection of employees)	None, low, high

### Assessing Discovered Clusters

There were two researchers that assessed all discovered clusters. To maintain a consistent interpretation of clusters during assessment, each rater annotated each cluster with a short description based on connotation and prevalence of tags assigned to the cluster. These descriptions were verified through comparison to apps contained in the respective cluster. Subsequently, clusters were assessed according to the five characteristics addressing information security and privacy implications. Reliability assessment with Janson’s and Olsson’s ι, an multivariate extension of Cohen’s κ for multiple judges on the same scale [80], led to a “substantial” [81] agreement score of ι=0.71. All remaining differences were resolved by discussion; if necessary, a third researcher was consulted for dispute resolution.

mHealth app archetypes (AT), with respect to information security and privacy are identified by grouping clusters with identical assessments in a final aggregation step. An archetype is “the original pattern or model of which all things of the same type are representations or copies” [82]. Hence, archetypes constitute underlying or core conceptions of objects observed in the real world. Real-world representations of archetypes may, however, materialize in different forms. For example, from an information security and privacy perspective, a medication reminder, as well as a patient interaction app are real-world representations of the same archetype; they both have access to sensitive medical information that should not be leaked to third parties, must remain accurate, and is of value to third parties. Yet, there is only a low demand for data preservation; medication reminders only need to store information until they have reminded users to take their medication, and patient interaction apps only need to store the data until the interaction has happened. Identification of mHealth app archetypes, with respect to information security and privacy, establishes, thus, a graspable overview of the thousands of mHealth apps offered in the app stores. To foster interpretability of app archetypes, identified app archetypes are numbered and additionally characterized by a natural language descriptor. The medication reminder and patient interaction app from the previous example are, for instance, both representations of the archetype AT 11 (Treatment Reminders). Due to the large diversity of possible real-world representations of mHealth app archetypes, it is unfeasible to identify meaningful descriptors capturing all facets of functionality offered by real-world archetype representations. The final descriptors were determined in group discussions of the authors. Hence, the archetype descriptors characterize exemplary functionality of real-world representations to foster archetype interpretability.

## Results

### Discovered Apps

**Figure 1:**
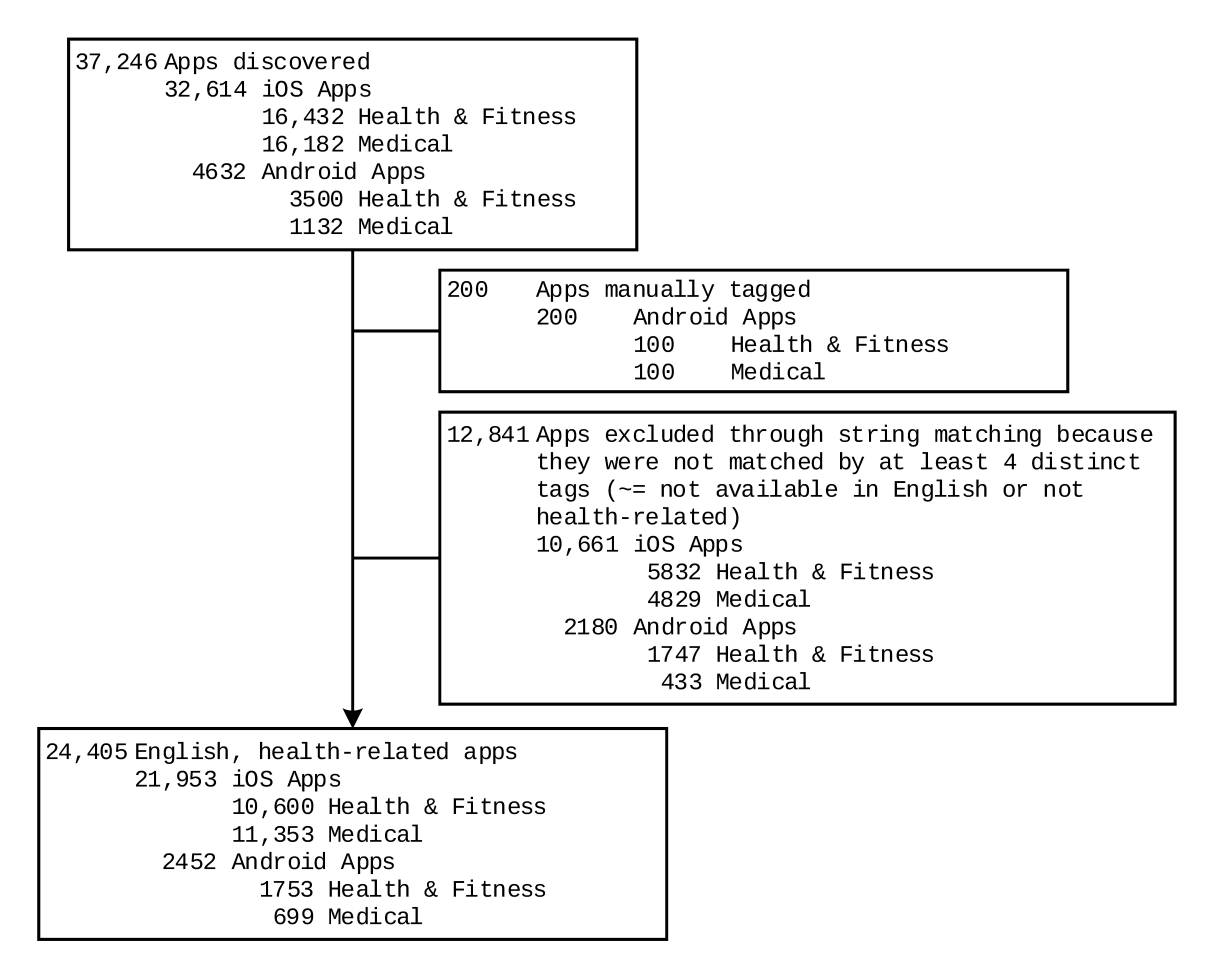
Flow chart of apps selection.

We discovered a total of 37,246 apps (iOS, 32,614; Android, 4632) in the categories Medical and Health & Fitness (Figure 1 shows this). After automatic tagging, 34.48% of apps (12,841/37,246; iOS, 32.69%, 10,661/32,614; Android, 47.06%, 2180/4632) were excluded from further assessment. The ratio of iOS mHealth apps to Android mHealth apps is 8.95 (21,953 to 2452).

**Figure 2:**
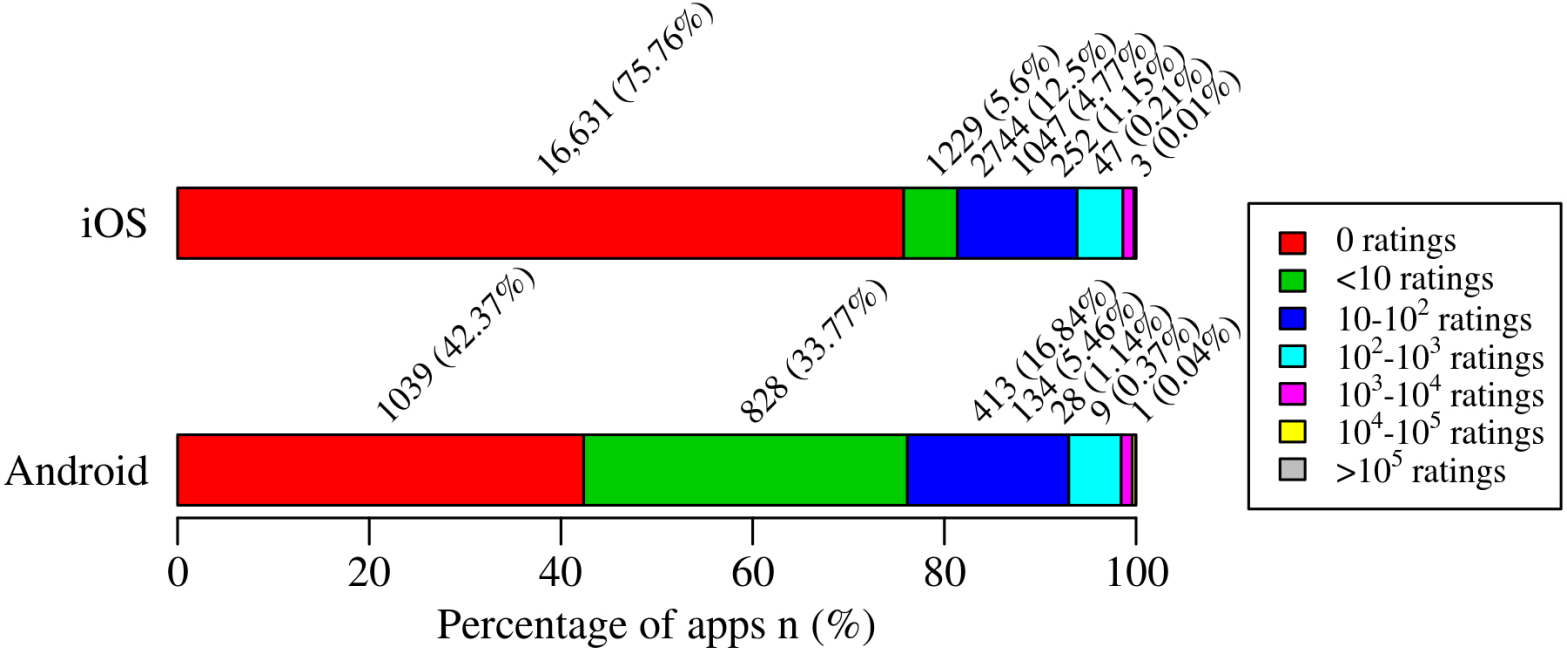
Rating count of mHealth apps by store. Number of ratings increases from left to right.

**Figure 3:**
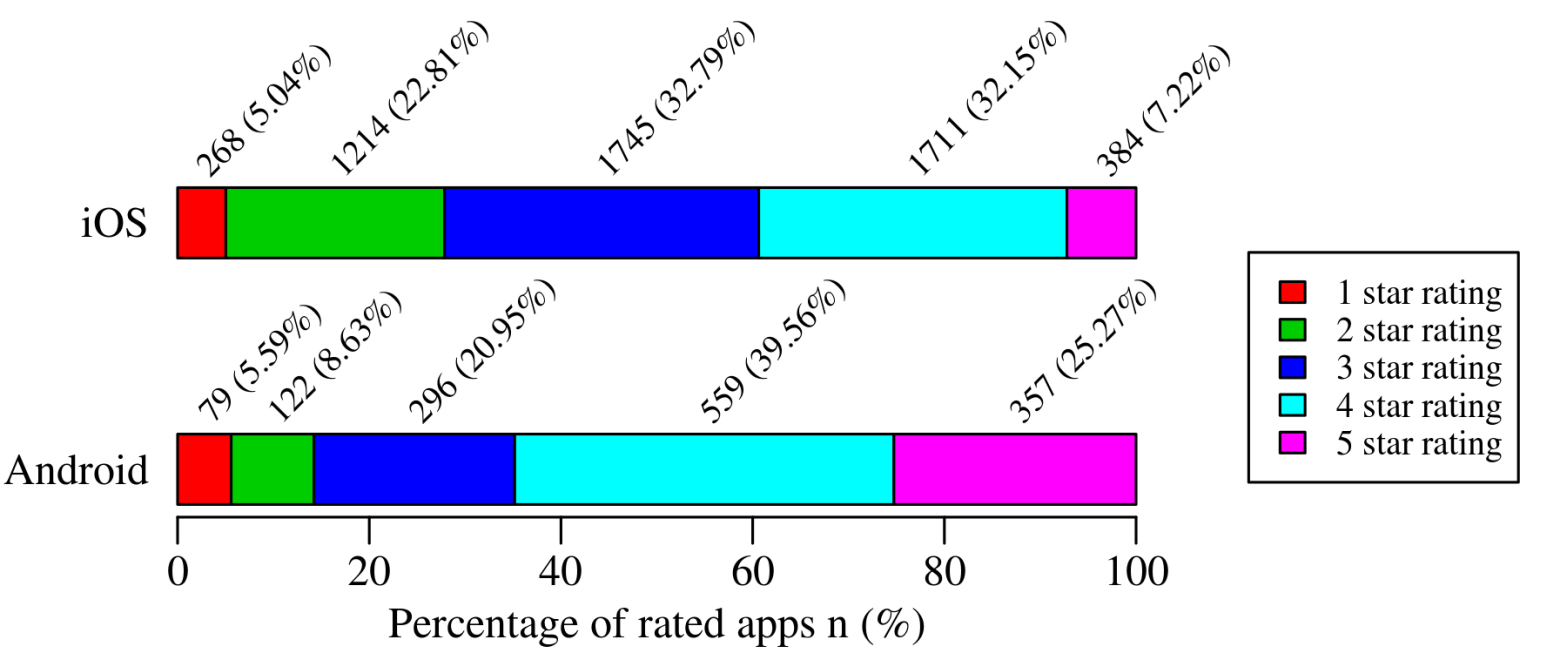
Rating of rated mHealth apps by store.

In both stores, users rate apps on 5-star integer rating scales, ranging from 1 to 5 stars. Mean rating scores of rated iOS and Android mHealth apps are 3.1 (median 3, SD 1.01) and 3.7 (median 3.92, SD 1.08), respectively. Figures 2 and 3 illustrate app ratings and rating counts in more detail. There are 81.36% (17,860/21,953) of iOS and 76.14% (1867/2452) of Android apps that have been rated less than 10 times. There are 75.76% (16,631/21,953) of iOS and 42.37% (1039/2452) of Android apps that have not been rated. There are 1.38% (302/21,953) of iOS and 1.55% (38/2452) of Android apps that have been rated more than 1000 times. There are 39.36% (2095/5322) of rated iOS apps that are rated four stars or more and 27.85% (1482/5322) of rated iOS apps are rated two stars or less. On Android, 64.83% (916/1413) of rated apps are rated four stars or more and 14.23% (201/1413) of rated apps are rated two stars or less. As illustrated in Figure 2, Android mHealth apps are rated higher than iOS mHealth apps (Mann Whitney U(6733)=2,592,190; *P*<.001; *r*=0.31; 95% CI 0.99997-0.99998). App category has no significant influence on app rating (iOS, Mann Whitney U(5320)=3,516,696; *P*=.92; *r*=0.002; Android, Mann Whitney U(1411)=203,559.5; *P*=.13; *r*=0.05).

**Figure 4:**
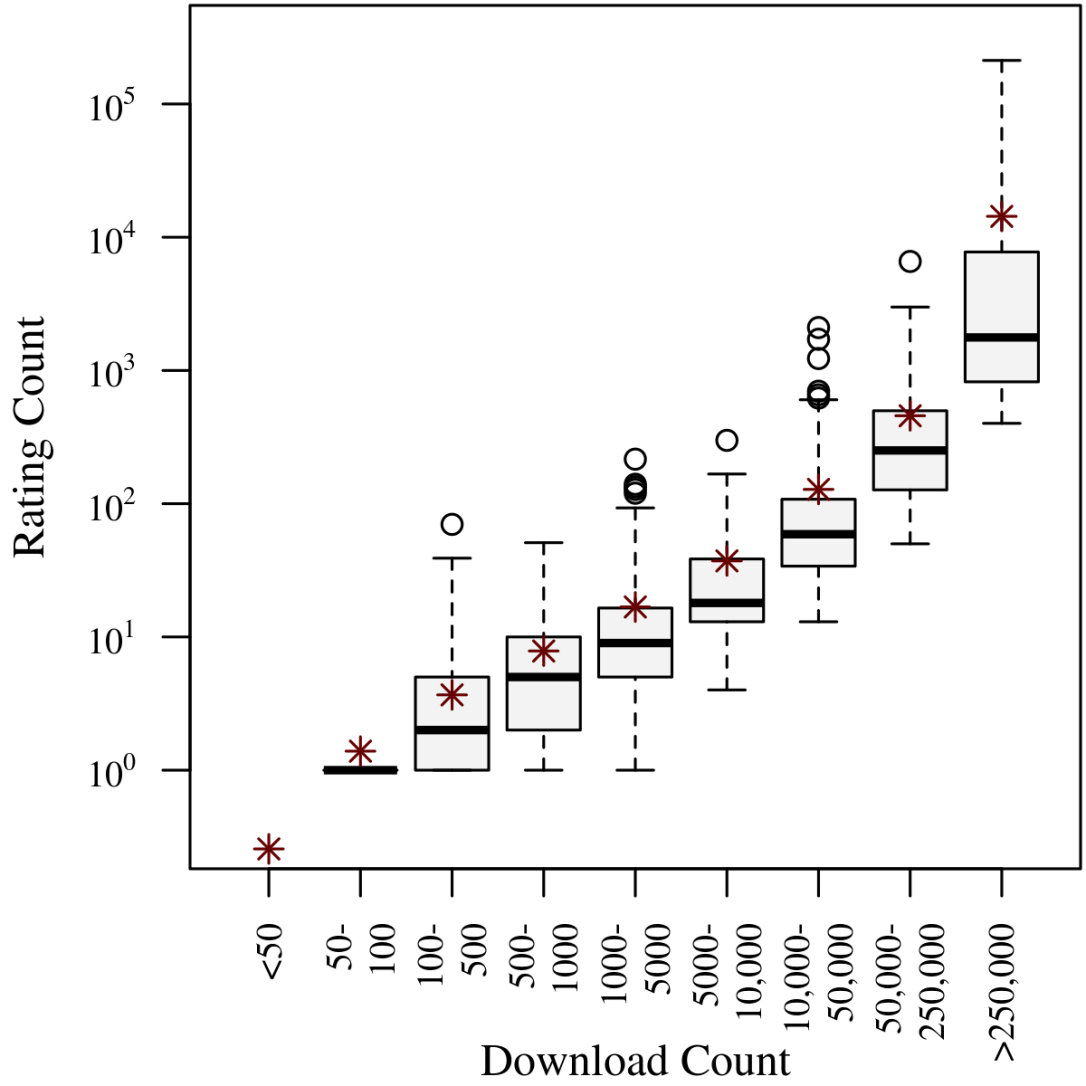
Boxplot of Android app rating count (log-scaled) and download count. Mean values are indicated with asterisks.

For Android apps, rating count and download count are strongly positively correlated (Spearman ρ=0.89, n=2452, *P*<.001), indicating that rating count is a good proxy for download count (Figure 4 shows this).

### App Clustering

**Figure 5:**
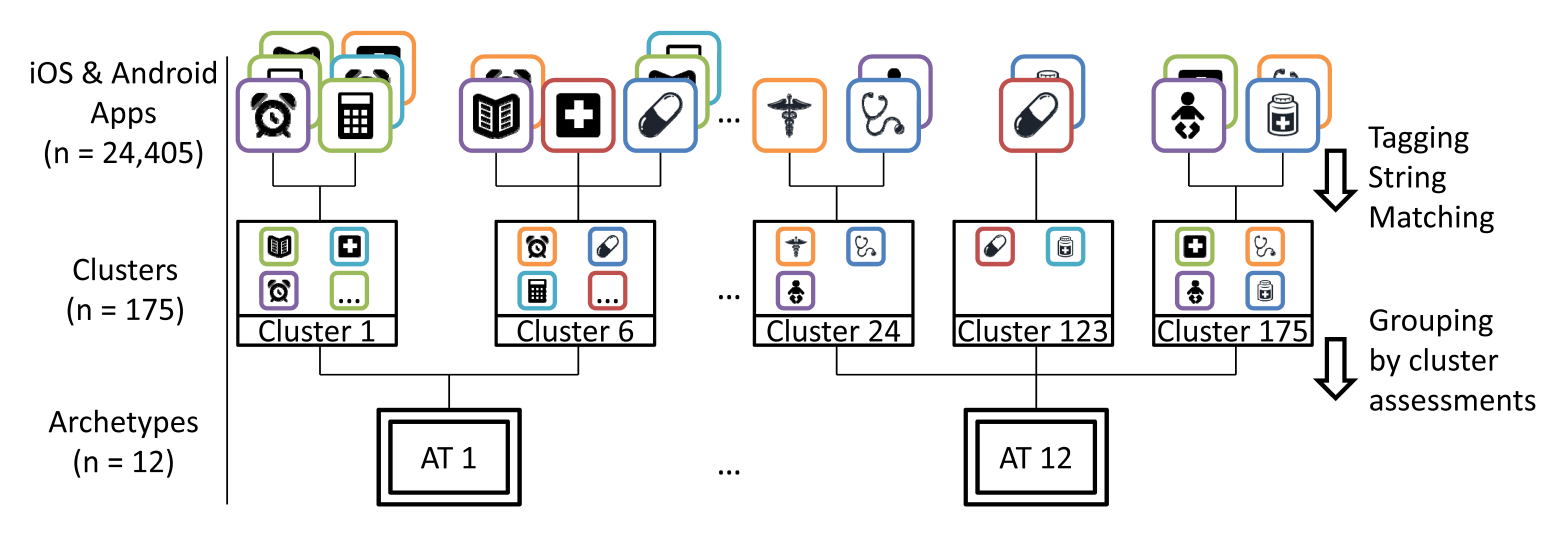
Outline of clustering process (AT = archetype).

Application of the Louvain method [62] grouped the 24,405 apps applicable for clustering into 245 distinct clusters with a modularity score of 0.47, which indicates a good division of the graph [63,83]. Discovered clusters have a mean size of 99.6 apps (minimum 2; maximum 910; median 90; SD 113.6). There are 28.6% (70/245) of clusters containing 26.33% (6426/24,405) of apps that conveyed no information relevant to our research scope and were excluded from further assessment. Some clusters are, for instance, too ambiguous because contained apps match mainly a single tag (eg, “Pain” or “Care Giver”) that is uninformative on its own with respect to our research scope. Cluster assessment, according to the five characteristics, led to further consolidation of the 175 informative clusters into 12 app archetypes, grouping clusters with identical characteristic assessments. The 12 app archetypes have a mean size of 14.6 clusters (minimum 3; maximum 58; median 8; SD 4.6) and 1498.25 apps (minimum 60; maximum 5603; median 615; SD 506.18). Figure 5 shows the clustering process.

Table 2 provides an overview of the cluster assessments with respect to health specificity of information, potential damage through leaks, manipulation, loss of information, and value of collected information to third parties. Medical information is available to apps in 33.7% (59/175) of clusters. There are 16.0% (28/175) of clusters that have access to information not available to ordinary apps [24,27], and apps in 50.3% (88/175) of clusters do not have access to more information than ordinary apps. Apps in 73.7% (129/175) of clusters have no or low potential damage through leaks of information. There are 39.4% (69/175) of clusters that are comprised of apps with high potential damage through manipulation of information. There is no potential damage through loss of information in 67.4% (118/175) of clusters. There are 77.7% (136/175) of clusters that consist of apps that have only access to information with no or low value for third parties.

**Table 2:** Cluster assessments with respect to the five information security and privacy characteristics.

		Clusters n (%)^a^ N=175	Apps n (%)^a^ N=17,979
**Specificity** ^b^			
	Standard^c^	88 (50.3)	8463 (47.07)
	Nonstandard^d^	28 (16.0)	4818 (26.80)
	Medical^e^	59 (33.7)	4698 (26.13)
**Leaks** ^f^			
	None	88 (50.3)	8463 (47.07)
	Low	41 (23.4)	5388 (29.97)
	High	46 (26.3)	4128 (22.96)
**Change** ^g^			
	None	9 (5.1)	786 (4.37)
	Low	97 (55.4)	11,641 (64.75)
	High	69 (39.4)	5552 (30.88)
**Loss** ^h^			
	None	118 (67.4)	10,049 (55.89)
	Low	32 (18.3)	5832 (32.44)
	High	25 (14.3)	2098 (11.67)
**Value** ^i^			
	None	88 (50.3)	8463 (47.07)
	Low	48 (27.4)	6108 (33.97)
	High	39 (22.3)	3408 (18.96)

^a^ Uninformative clusters are not included in percentages

^b^ Health specificity of information available to apps

^c^ Apps only have access to information ordinarily available to apps, for example, phone identifiers or location information

^d^ Apps have access to information not ordinarily available to apps, but no access to medical information, for example, workout history or eating habits

^e^ Apps have access to medical information, for example, disease history or health insurance information

^f^ Potential damage through leaks of information, for example, embarrassment, lessened employment possibilities

^g^ Potential damage through manipulation, change, of information, for example, treatment based on erroneous information

^h^ Potential damage through loss of information, for example, loss of information important for treatment

^i^ Value of information to third parties, for example, medical identity theft, selection of employees

### App Archetypes

Archetype descriptors and examples for functionality offered by apps of the different app archetypes are listed in Table 3. Table 4 illustrates the twelve discovered app archetypes with distinct value combinations according to the five characteristics. AT 1 (Casual Tools) represents 5.1% (9/175) of clusters and 4.37% (786/17,979) of apps. Apps of AT 1 only have access to information also available to ordinary apps and provide no critical functionality, so that their use cannot cause more damage than the use of any other app. Apps of AT 1 offer mostly generic information and are only marginally health-related. AT 2 (Common Knowledge Providers) is the archetype with the most representations in our sample (33.1%, 58/175 of clusters; 31.16%, 5603/17,979 of apps). Apps of AT 2 also have no access to other information than ordinary apps, so that there is no damage through leaks or loss of information. Apps of AT 2 have low potential damage through manipulation of information. More critical information is provided by apps of AT 3 (Treatment Guides), which provide information directly relevant for (self-)treatment or intended to guide users in emergency situations. Information provided by apps of AT 3 needs to be correct to serve as reliable foundation for (self-)treatment decisions; accidental or malicious provision of erroneous information promotes wrong or counterproductive treatment decisions. AT 3 represents 12.0% (21/175) of clusters and 11.54% (2074/17,979) of apps. AT 4 and AT 5 (Fitness Ad-Hoc Tools and Fitness Trackers; 16.0%, 28/175 of clusters; 26.80%, 4818/17,979 of apps) have access to more information than ordinary apps. Yet, they do not collect medical information, so that there is at most low potential damage because collected information is not sensitive, not crucial for provision of medical services, not important for future endeavors, and not valuable to third parties. The remaining seven app archetypes collect medical information (33.7%, 59/175 of clusters; 26.13%, 4698/17,979 of apps). AT 6 (Treatment Support Tools) is the only app archetype that collects medical information and has low potential damage through leaks of information. AT 6 represents calculators and tools for medical professionals or tools offering very specific functionality, so that collected information is either not attributable to patients or not informative. Hence, there is only low potential damage through leaks of information and low value of information to third parties. AT 3 (Treatment Guides), AT 6 (Treatment Support Tools), AT 10 (Health Monitors), AT 11 (Treatment Reminders), and AT 12 (Health Records) offer functionality directly relevant for treatment or decision making so that there is high potential damage through information manipulation. There are four app archetypes, AT 8 (State of Health Tests), AT 10 (Health Monitors), AT 11 (Treatment Reminders), and AT 12 (Health Records) that collect medical information detailed enough to be of high value to third parties (eg, blood test results, medication histories, or health records). While the other app archetypes do not require long storage times of collected information, apps of AT 12 (Health Records) collect medical information relevant for future decision making (eg, disease management tools, medication history, or health records), so that potential damage through loss of information is high. Since apps of AT 12 also tend to collect very detailed, personal information, potential damage through leaks or manipulation and value of information to third parties is high as well.

**Table 3:** Exemplary functionality of apps represented by the AT.

Archetype	Descriptor	Exemplary kinds of contained apps
AT 1	Casual Tools	Life improvement guides; mosquito repellents; brain fitness trainer
AT 2	Common Knowledge Providers	Information provision for education; alarm clocks; fitness guides
AT 3	Treatment Guides	First aid guides; home remedy guides; medication guides
AT 4	Fitness Ad-Hoc Tools	Diet calculators; weight control calculators; fitness calculators
AT 5	Fitness Trackers	Workout tracker; smoking cessation tools; diet tracker
AT 6	Treatment Support Tools	Diabetes calculators; dosage calculators; diagnosis support tools
AT 7	Intimate Ad-Hoc Tools	Fertility calculators; pregnancy calculators; physician finder
AT 8	State of Health Tests	Acuity tests; color vision tests; blood alcohol calculators
AT 9	Intimate Trackers	Menstruation, intercourse, fertility, and pregnancy tracker
AT 10	Health Monitors	Heart rate monitors; disease counseling; tools for blood test analysis
AT 11	Treatment Reminders	Medication reminder; patient interaction and communities
AT 12	Health Records	Health/emergency records; disease management tools; medication tracker

**Table 4:** AT with respective assessments of the five information security and privacy characteristics and contained clusters and apps.

AT	Specificity^a^	Leaks^e^	Change^f^	Loss^g^	Value^h^	Clusters n (%)^i^ N=175	Apps n (%)^i^ N=17,979
1	Standard^b^	None	None	None	None	9 (5.1)	786 (4.37)
2	Standard	None	Low	None	None	58 (33.1)	5603 (31.16)
3	Standard	None	High	None	None	21 (12.0)	2074 (11.54)
4	Nonstandard^c^	Low	Low	None	Low	7 (4.0)	216 (1.20)
5	Nonstandard	Low	Low	Low	Low	21 (12.0)	4602 (25.60)
6	Medical^d^	Low	High	None	Low	13 (7.4)	570 (3.17)
7	Medical	High	Low	None	Low	3 (1.7)	60 (0.33)
8	Medical	High	Low	None	High	4 (2.3)	500 (2.78)
9	Medical	High	Low	Low	Low	4 (2.3)	660 (3.67)
10	Medical	High	High	None	High	3 (1.7)	240 (1.33)
11	Medical	High	High	Low	High	7 (4.0)	570 (3.17)
12	Medical	High	High	High	High	25 (14.3)	2098 (11.67)

^a^ Health specificity of information available to apps

^b^ Apps only have access to information ordinarily available to apps, for example, phone identifiers or location information

^c^ Apps have access to information not ordinarily available to apps, but no access to medical information, for example, workout history or eating habits

^d^ Apps have access to medical information, for example, disease history or health insurance information

^e^ Potential damage through leaks of information, for example, embarrassment, lessened employment possibilities

^f^ Potential damage through manipulation, change, of information, for example, treatment based on erroneous information

^g^ Potential damage through loss of information, for example, loss of information important for treatment

^h^ Value of information to third parties, for example, medical identity theft, selection of employees

^i^ Uninformative clusters are not included in percentages

## Discussion

### Principal Results

#### Discovered Apps

Since their inception in 2008, the iOS and Android App Stores underwent a rapid development. After a few years, the app portfolios of both stores encompass hundreds of thousands of apps [8,29,57], which include thousands of mHealth apps. However, absence or scarceness of ratings for 81.36% (17,860/21,953) of iOS and 76.14% (1867/2452) of Android apps indicates that over three quarters of mHealth apps are not in widespread use. A fraction of users who download apps provide ratings [15,84]. Hence, apps less often rated are likely to be less often used than more often rated apps. An explanation for this is the increased visibility of better-rated apps [85], apps with higher and more ratings are more prominently displayed in app stores and thus more likely to be discovered by potential users. More ratings make the resulting app assessment also more reliable, which attracts more users. Furthermore, many apps offer similar or competing functionality (eg, calculation of the body mass index, tracking of workouts, or prediction of date of birth), so that only a few first-movers, heavily promoted apps, or high quality apps will gain a large user base. App ratings indicate that most users are not dissatisfied with rated apps, 72.15% (3840/5322) of iOS and 85.77% (1212/1413) of Android apps are rated average or above. Another impediment for more widespread use of mHealth apps might be users’ concerns about information security and privacy implications [15]. Our cluster analysis of mHealth apps sheds some light on the potential damage through information security and privacy infringements.

#### App Clustering

Since mHealth apps usually offer functionality related to users’ health, it is not a surprising finding that information security and privacy infringements cause potential damage for the majority of apps (94.9%, 166/175 of clusters; 95.63%, 17,193/17,979 of apps). mHealth apps offer, however, diverse functionality so that potential for damage through information security and privacy infringements differs. Manipulation of information is a threat common to most mHealth apps (94.9%, 166/175 of clusters; 95.63%, 17,193/17,979 of apps). Even apps that do not collect any medical information, like AT 2 (Common Knowledge Providers) or AT 3 (Treatment Guides), must ensure that information they provide is correct and stays correct because, at least some, users will act on offered information and base (self )treatment decisions on provided information. Apps offering information or functionality directly relevant for treatment or care must especially ensure that offered information is not accidentally or maliciously manipulated. mHealth apps that only provide information have, however, no information security and privacy implications through leaks or loss of collected information since no information is collected. About one half of the apps in our sample (50.3%, 88/175 of clusters; 47.07%, 8463/17,979 of apps) only provide information. Such apps are probably the most “pleasant” apps when it comes to protecting information security and privacy since no user-collected information must be protected. Thus, providers can focus on protection of integrity of information in rest and during transport, as well as offering accurate information from the onset. Still, extant research shows that information provided by some apps does not concur with current evidence and recommendations or is even contradicting [49,51].

There are 33.7% (59/175) of clusters and 26.13% (4698/17,979) of apps that have access to medical user information. All of these apps have high potential damage through information security and privacy infringements in at least one characteristic. Some apps, for example, AT 6 (Treatment Support Tools) do not collect detailed information or information attributable to users and do not retain entered information, so that there is no potential damage through loss of information, low potential damage through leaks of information, and low value of information for third parties. Yet, they serve as foundation for treatment decisions (eg, appropriate medication dosage), so that there is high potential damage through manipulation of information. Other apps collect information users want to keep private, for example, AT 9 (Intimate Trackers), so that there is high potential damage through leaks of information, but collected information is not directly relevant for treatment or state of health, so that the other characteristics pose only low potential damage. Potential damage of other apps, for example, AT 12 (Health Records) was rated with the most critical assessment in all five characteristics since contained information is sensitive and must be kept private, has to be accurate and accessible to inform treatment decisions, and allows for misuse motivated by financial gain. Consequentially, there is no one-size-fits-all approach for ensuring information security and privacy of mHealth apps. mHealth apps offer different functionality so that they are also subject to different threats. Accordingly, measures for protection of information security and privacy must be tailored to the app to be protected [70].

Our identification of the twelve mHealth app archetypes elucidates information security and privacy of mHealth apps, instead of a hazy collection comprised of the thousands of mHealth apps available in the app stores, the archetypes constitute a lucid, descriptive collection of twelve mHealth app archetypes with different information security and privacy characteristics. Future research can build on the archetypes, for instance, to prioritize information security and privacy requirements with respect to app type, devise collections of security measures ensuring sound protection of information security and privacy, analyze user perceptions of information security and privacy with respect to different kinds of apps, or to further theory and methodology for app development that takes information security and privacy implications into account. For example, potential damage through information security and privacy infringements would obviously be reduced if apps that mainly provide information did not store any user information and focused rather on secure interoperability with specialized storage apps. An overview of app archetypes with respect to information security is also helpful for practical audiences. Associating an mHealth app of interest with the respective archetype improves, for instance, the understanding of perks and perils associated with app use. The overview of the archetypes alone is useful to foster user comprehension and awareness of information security and privacy implications of mHealth app use. In order to continuously benefit from mHealth apps, users must be able to make informed decisions about mHealth app adoption and use.

The apps with the most serious assessment of potential damage through information security and privacy infractions (AT 12, Health Records; 14.3%, 25/175 of clusters; 11.67%, 2098/17,979 of apps) may also offer the most benefits to users [2]. AT 12 represents all the different facets of health records and disease management tools [86-89], which collect detailed health information, allowing them to offer functionality tailored to users’ needs and individual peculiarities or to provide other apps with the information required for tailoring offered functionality. Apps of AT 12 could rise to central hubs in the emerging mHealth environment if interoperability issues are solved [12,90] and information security and privacy is sufficiently addressed so that users can safely trust apps of AT 12 to protect their information [14,91,92].

It is noteworthy that some threats are common to all kinds of mHealth apps, even those without any data collection. Users’ behavior, or the sole fact that a guide for stress relief or fighting depression, a support tool for hypertension, or an app providing information on cancer, chronic diseases, infertility, or incontinence, is installed on a device reveals sensitive, private, or embarrassing information [93]. In the end, it is up to users which apps they use and what information they intend to share. To support users in this decision, it is important that they are sensitized to the risks associated with sharing private, sensitive, medical information [16,94] and offered means to gauge, configure, and control information security and privacy practices of mHealth apps [95,96]. Moreover, app stores need to establish processes that ensure protection of information security and privacy prior to making apps publicly accessible, at least, for apps with high potential damage and value to third parties. App developers and providers need to implement appropriate security measures to protect information security and privacy. While ease of app development, free access to helpful apps, and fast dissemination of innovations is desirable, it is imperative that these do not come at the price of lacking information security and privacy. Last, but not least, experienced users, researchers, and further independent entities need to contribute as well by identifying malicious and harmful apps, publishing their findings, and eliminating sources of harm and malice.

### Limitations

Since we established a broad overview of available mHealth apps and assessed all discovered apps fitting our selection criteria, it was unfeasible to install and test all apps, so that we focused on the information provided in app stores. This is, however, a common approach, for example, [8,40,51,52], which allowed us to analyze a large sample of over 30,000 apps. Moreover, we cannot ascertain how many of the English apps available on the Android App Store we discovered because the app store offers no complete listing of available apps and search results are limited to 500 apps. Extant reviews of apps in all categories offered in the Android App Store report around 20,000 apps offered in the categories Medial and Health & Fitness. However, these reviews collected apps independent of language and did not assess whether the apps actually offer functionality fitting the categories Medical or Health & Fitness. Our diverse wordlist, comprised of 111,632 distinct words and phrases (see Multimedia Appendices 1 or 2), introduced diversity to search queries and led to the discovery of a wide array of apps, while avoiding bias towards specific types of apps. Creation of search strings based on English words favored discovery of apps offered in English. While this may have reduced the number of discovered Android apps, it suits our research approach and objectives because apps not available in English were excluded from further assessment. Nevertheless, the reported difference in number of apps available on iOS and Android should be treated with care. For now, the iOS and Android App Stores offer far more apps than any other app store [8]. The dominant position of iOS and Android supports our focus on the iOS and Android App Store.

### Conclusions

The iOS and Android App Stores offer a wide selection of mHealth apps. Analysis of rating counts indicates, however, that less than a quarter of available apps are in more or less widespread use. An issue impeding app dissemination might be users’ information security and privacy concerns [15]. Our cluster analysis shows that most mHealth apps require access to sensitive personal information or offer other services potentially impacting users’ treatment or state of health, which increases the potential damage through information security and privacy infringements. The diversity of mHealth apps prevents, however, a one-size-fits-all approach to ensuring information security and privacy of mHealth apps. To address arising challenges, app providers, developers, stores, as well as users, must be sensitized to potential threats and further research and development efforts are required to facilitate protection from information security and privacy infringements. It would be undesirable to diminish or undermine the promising potential of mHealth apps to transform and improve the health care environment [2] through lacking attention to information security and privacy.

## Multimedia Appendix 1

Word list used for construction of search queries for Android app discovery.

## Multimedia Appendix 2

Word list used for construction of search queries for Android app discovery (alternate version in Microsoft Word format with new lines as seperator).

## Abbreviations

AT: app archetype
HTML: Hyper Text Markup Language
IT: information technology
mHealth: mobile health
