# Supplementary material for: Exploring the Far Side of Mobile Health: Information Security and Privacy of Mobile Health Apps on iOS and Android
Source: JMIR Mhealth Uhealth. 2015 Jan 19;3(1):e8. doi: 10.2196/mhealth.3672 (PMC4319144; doi:10.2196/mhealth.3672)
Supplement: Supplementary file 2 [file mhealth_v3i1e8_app2.doc]

""

"3D"

"7-day trial"

"9-week plan"

"a"

"aah"

"aahed"

"aahing"

"aahs"

"aam"

"aardvark"

"aardvarks"

"aardwolf"

"ab"

"abaci"

"aback"

"abacus"

"abacuses"

"abaft"

"abalone"

"abalones"

"abampere"

"abandon"

"abandoned"

"abandonedly"

"abandonee"

"abandoner"

"abandoners"

"abandoning"

"abandonment"

"abandonments"

"abandons"

"abase"

"abased"

"abasedly"

"abasement"

"abaser"

"abasers"

"abases"

"abash"

"abashed"

"abashedly"

"abashes"

"abashing"

"abashment"

"abashments"

"abasing"

"abatable"

"abate"

"abated"

"abatement"

"abatements"

"abater"

"abaters"

"abates"

"abating"

"abatis"

"abatises"

"abator"

"abattoir"

"abattoirs"

"abb"

"abbacies"

"abbacy"

"abbatial"

"abbe"

"abbes"

"abbess"

"abbesses"

"abbey"

"abbeys"

"abbot"

"abbotcies"

"abbotcy"

"abbots"

"abbotship"

"abbotships"

"abbott"

"abbr"

"abbrev"

"abbrevation"

"abbreviate"

"abbreviated"

"abbreviates"

"abbreviating"

"abbreviation"

"abbreviations"

"abbreviator"

"abbreviators"

"abc"

"abdicable"

"abdicate"

"abdicated"

"abdicates"

"abdicating"

"abdication"

"abdications"

"abdicator"

"abdomen"

"abdomens"

"abdominal"

"abdominally"

"abduct"

"abducted"

"abducting"

"abduction"

"abductions"

"abductor"

"abductors"

"abducts"

"abeam"

"abecedarian"

"abecedarians"

"abed"

"aberdeen"

"aberrance"

"aberrancies"

"aberrancy"

"aberrant"

"aberrantly"

"aberrants"

"aberration"

"aberrational"

"aberrations"

"abet"

"abetment"

"abets"

"abettal"

"abettals"

"abetted"

"abetter"

"abetters"

"abetting"

"abettor"

"abettors"

"abeyance"

"abeyances"

"abeyancies"

"abeyancy"

"abeyant"

"abg"

"abhor"

"abhorred"

"abhorrence"

"abhorrences"

"abhorrent"

"abhorrently"

"abhorrer"

"abhorrers"

"abhorring"

"abhors"

"abidance"

"abide"

"abided"

"abider"

"abiders"

"abides"

"abiding"

"abidingly"

"abidingness"

"abigail"

"abilene"

"abilities"

"ability"

"abiotic"

"abject"

"abjection"

"abjectly"

"abjectness"

"abjuration"

"abjurations"

"abjuratory"

"abjure"

"abjured"

"abjurer"

"abjurers"

"abjures"

"abjuring"

"ablate"

"ablated"

"ablates"

"ablating"

"ablation"

"ablations"

"ablatival"

"ablative"

"ablatively"

"ablatives"

"ablaze"

"able"

"ableness"

"abler"

"ables"

"ablest"

"ablings"

"abloom"

"ablush"

"abluted"

"ablution"

"ablutionary"

"ablutions"

"ablutomania"

"ably"

"abnegate"

"abnegated"

"abnegates"

"abnegating"

"abnegation"

"abnegations"

"abnegator"

"abnegators"

"abner"

"abnormal"

"abnormalities"

"abnormality"

"abnormally"

"abnormals"

"abo"

"aboard"

"abode"

"aboded"

"abodes"

"aboding"

"aboil"

"abolish"

"abolishable"

"abolished"

"abolisher"

"abolishers"

"abolishes"

"abolishing"

"abolishment"

"abolition"

"abolitionary"

"abolitionism"

"abolitionist"

"abolitionists"

"abominable"

"abominably"

"abominate"

"abominated"

"abominates"

"abominating"

"abomination"

"abominations"

"abominator"

"abominators"

"aboral"

"aboriginal"

"aboriginally"

"aborigine"

"aborigines"

"aborning"

"abort"

"aborted"

"aborter"

"aborters"

"abortifacient"

"aborting"

"abortion"

"abortional"

"abortionist"

"abortionists"

"abortions"

"abortive"

"abortively"

"abortiveness"

"abortogenic"

"aborts"

"aboulomania"

"abound"

"abounded"

"abounding"

"abounds"

"about"

"About how the human body works"

"above"

"aboveboard"

"aboveground"

"aboves"

"abracadabra"

"abradant"

"abradants"

"abrade"

"abraded"

"abrader"

"abraders"

"abrades"

"abrading"

"abraham"

"abrasion"

"abrasions"

"abrasive"

"abrasively"

"abrasiveness"

"abrasives"

"abreact"

"abreacted"

"abreacting"

"abreaction"

"abreacts"

"abreast"

"abridge"

"abridged"

"abridgement"

"abridgements"

"abridger"

"abridgers"

"abridges"

"abridging"

"abridgment"

"abridgments"

"abroad"

"abrogate"

"abrogated"

"abrogates"

"abrogating"

"abrogation"

"abrogations"

"abrogative"

"abrogator"

"abrogators"

"abrupt"

"abrupter"

"abruptest"

"abruptly"

"abruptness"

"abs"

"abscam"

"abscess"

"abscessed"

"abscesses"

"abscessing"

"abscise"

"abscised"

"abscises"

"abscising"

"abscissa"

"abscissae"

"abscissas"

"abscission"

"abscissions"

"abscond"

"absconded"

"absconder"

"absconders"

"absconding"

"absconds"

"absence"

"absences"

"absent"

"absented"

"absentee"

"absenteeism"

"absentees"

"absenter"

"absenters"

"absentia"

"absenting"

"absently"

"absentminded"

"absentmindedly"

"absentmindedness"

"absents"

"absinth"

"absinthe"

"absinthes"

"absinths"

"absolute"

"absolutely"

"absoluteness"

"absoluter"

"absolutes"

"absolutest"

"absolution"

"absolutions"

"absolutism"

"absolutist"

"absolutistic"

"absolutists"

"absolvable"

"absolve"

"absolved"

"absolver"

"absolvers"

"absolves"

"absolving"

"absorb"

"absorbability"

"absorbable"

"absorbed"

"absorbencies"

"absorbency"

"absorbent"

"absorbents"

"absorber"

"absorbers"

"absorbing"

"absorbingly"

"absorbs"

"absorptiometer"

"absorption"

"absorptions"

"absorptive"

"abstain"

"abstained"

"abstainer"

"abstainers"

"abstaining"

"abstains"

"abstemious"

"abstemiously"

"abstemiousness"

"abstention"

"abstentionism"

"abstentionist"

"abstentions"

"abstentious"

"abstinence"

"abstinent"

"abstinently"

"abstract"

"abstracted"

"abstractedly"

"abstractedness"

"abstracter"

"abstracters"

"abstracting"

"abstraction"

"abstractionism"

"abstractionist"

"abstractionists"

"abstractions"

"abstractly"

"abstractness"

"abstractor"

"abstractors"

"abstracts"

"abstricts"

"abstruse"

"abstrusely"

"abstruseness"

"abstruser"

"abstrusest"

"absurd"

"absurder"

"absurdest"

"absurdities"

"absurdity"

"absurdly"

"absurdness"

"absurds"

"absurdum"

"abt"

"abubble"

"abundance"

"abundances"

"abundant"

"abundantly"

"abusable"

"abusage"

"abuse"

"abused"

"abuser"

"abusers"

"abuses"

"abusing"

"abusive"

"abusively"

"abusiveness"

"abut"

"abutment"

"abutments"

"abuts"

"abuttal"

"abuttals"

"abutted"

"abutter"

"abutters"

"abutting"

"abuzz"

"abvolt"

"aby"

"abyes"

"abysm"

"abysmal"

"abysmally"

"abysms"

"abyss"

"abyssal"

"abysses"

"abyssinia"

"abyssinian"

"abyssinians"

"ac"

"acacia"

"acacias"

"academe"

"academes"

"academia"

"academias"

"academic"

"academical"

"academically"

"academician"

"academicians"

"academicianship"

"academicism"

"academics"

"academies"

"academy"

"acadia"

"acanthi"

"acanthus"

"acanthuses"

"acapulco"

"acarology"

"acarophobia"

"ACC"

"accede"

"acceded"

"accedence"

"acceder"

"acceders"

"accedes"

"acceding"

"accelerable"

"accelerando"

"accelerant"

"accelerate"

"accelerated"

"accelerates"

"accelerating"

"acceleration"

"accelerations"

"accelerative"

"accelerator"

"accelerators"

"accelerometer"

"accelerometers"

"accent"

"accented"

"accenting"

"accents"

"accentual"

"accentuate"

"accentuated"

"accentuates"

"accentuating"

"accentuation"

"accentuator"

"accept"

"acceptability"

"acceptable"

"acceptableness"

"acceptably"

"acceptance"

"acceptances"

"acceptant"

"acceptation"

"accepted"

"acceptedly"

"acceptee"

"acceptees"

"accepter"

"accepters"

"accepting"

"acceptive"

"acceptor"

"accepts"

"access"

"accessability"

"accessed"

"accesses"

"accessibility"

"accessible"

"accessibleness"

"accessibly"

"accessing"

"accession"

"accessions"

"accessories"

"accessorily"

"accessoriness"

"accessors"

"accessory"

"accidence"

"accident"

"accidental"

"accidentally"

"accidentalness"

"accidentals"

"accidents"

"accidie"

"accidies"

"acclaim"

"acclaimed"

"acclaimer"

"acclaimers"

"acclaiming"

"acclaims"

"acclamation"

"acclamations"

"acclimate"

"acclimated"

"acclimates"

"acclimating"

"acclimation"

"acclimatization"

"acclimatize"

"acclimatized"

"acclimatizer"

"acclimatizes"

"acclimatizing"

"acclivities"

"acclivitous"

"acclivity"

"accolade"

"accolades"

"accommodate"

"accommodated"

"accommodates"

"accommodating"

"accommodatingly"

"accommodation"

"accommodational"

"accommodations"

"accommodative"

"accommodatively"

"accommodativeness"

"accommodator"

"accommodators"

"accompanied"

"accompanies"

"accompaniment"

"accompaniments"

"accompanist"

"accompanists"

"accompany"

"accompanying"

"accompanyist"

"accompli"

"accomplice"

"accomplices"

"accomplis"

"accomplish"

"accomplishable"

"accomplished"

"accomplisher"

"accomplishers"

"accomplishes"

"accomplishing"

"accomplishment"

"accomplishments"

"accord"

"accordable"

"accordance"

"accordant"

"accordantly"

"accorded"

"accorder"

"accorders"

"according"

"accordingly"

"accordion"

"accordionist"

"accordionists"

"accordions"

"accords"

"accost"

"accostable"

"accosted"

"accosting"

"accosts"

"account"

"accountability"

"accountable"

"accountableness"

"accountably"

"accountancy"

"accountant"

"accountants"

"accountantship"

"accounted"

"accounter"

"accounters"

"accounting"

"accounts"

"accouter"

"accoutered"

"accoutering"

"accouterment"

"accouterments"

"accouters"

"accoutred"

"accoutrement"

"accoutres"

"accoutring"

"accredit"

"accreditation"

"accredited"

"accreditee"

"accrediting"

"accreditment"

"accredits"

"accrete"

"accreted"

"accretes"

"accreting"

"accretion"

"accretionary"

"accretions"

"accruable"

"accrual"

"accruals"

"accrue"

"accrued"

"accruement"

"accrues"

"accruing"

"acct"

"accts"

"acculturate"

"acculturation"

"acculturational"

"acculturative"

"accumulable"

"accumulate"

"accumulated"

"accumulates"

"accumulating"

"accumulation"

"accumulations"

"accumulative"

"accumulatively"

"accumulativeness"

"accumulator"

"accumulators"

"accuracies"

"accuracy"

"accurate"

"accurate sources"

"accurately"

"accurateness"

"accurse"

"accursed"

"accursedly"

"accursedness"

"accurst"

"accusable"

"accusal"

"accusals"

"accusant"

"accusation"

"accusations"

"accusative"

"accusatively"

"accusativeness"

"accusatives"

"accusatorial"

"accusatorially"

"accusatory"

"accusatrix"

"accusatrixes"

"accuse"

"accused"

"accuser"

"accusers"

"accuses"

"accusing"

"accusingly"

"accusive"

"accusor"

"accustom"

"accustomed"

"accustoming"

"accustoms"

"ace"

"aced"

"aceology"

"acerb"

"acerbate"

"acerbated"

"acerbates"

"acerbating"

"acerber"

"acerbest"

"acerbic"

"acerbities"

"acerbity"

"acerola"

"acerose"

"acerous"

"aces"

"acetaldehyde"

"acetaminophen"

"acetanilide"

"acetate"

"acetates"

"acetic"

"acetified"

"acetifies"

"acetify"

"acetifying"

"acetimeter"

"acetone"

"acetones"

"acetonic"

"acetylcholine"

"acetylene"

"acetylsalicylic"

"ache"

"ached"

"achene"

"achenes"

"achenial"

"aches"

"achier"

"achiest"

"achievable"

"achieve"

"achieved"

"achievement"

"achievements"

"achiever"

"achievers"

"achieves"

"achieving"

"achilles"

"achiness"

"aching"

"achingly"

"achoo"

"achordate"

"achromat"

"achromatic"

"achromatically"

"achromatism"

"achromats"

"achy"

"acid"

"acidhead"

"acidheads"

"acidic"

"acidifiable"

"acidification"

"acidified"

"acidifier"

"acidifiers"

"acidifies"

"acidify"

"acidifying"

"acidimeter"

"acidities"

"acidity"

"acidly"

"acidness"

"acidophilus"

"acidoses"

"acidosis"

"acidotic"

"acids"

"acidulate"

"acidulated"

"acidulates"

"acidulating"

"acidulation"

"acidulous"

"acidulously"

"acidulousness"

"acidy"

"acing"

"acknowledge"

"acknowledgeable"

"acknowledged"

"acknowledgedly"

"acknowledgement"

"acknowledgements"

"acknowledger"

"acknowledgers"

"acknowledges"

"acknowledging"

"acknowledgment"

"acknowledgments"

"aclu"

"acme"

"acmes"

"acne"

"acned"

"acnes"

"acoin"

"acology"

"acolyte"

"acolytes"

"aconite"

"aconites"

"acorn"

"acorns"

"acoustic"

"acoustical"

"acoustically"

"acoustics"

"acquaint"

"acquaintance"

"acquaintances"

"acquaintanceship"

"acquaintanceships"

"acquainted"

"acquainting"

"acquaints"

"acquiesce"

"acquiesced"

"acquiescence"

"acquiescent"

"acquiescently"

"acquiesces"

"acquiescing"

"acquiesence"

"acquirable"

"acquire"

"acquired"

"acquirement"

"acquirements"

"acquirer"

"acquirers"

"acquires"

"acquiring"

"acquisition"

"acquisitions"

"acquisitive"

"acquisitively"

"acquisitiveness"

"acquit"

"acquits"

"acquittal"

"acquittals"

"acquitted"

"acquitter"

"acquitting"

"acre"

"acreage"

"acreages"

"acred"

"acres"

"acrid"

"acrider"

"acridest"

"acridities"

"acridity"

"acridly"

"acridness"

"acrimonies"

"acrimonious"

"acrimoniously"

"acrimoniousness"

"acrimony"

"acrobat"

"acrobatic"

"acrobatically"

"acrobatics"

"acrobats"

"acroliths"

"acromegalic"

"acromegalies"

"acromegaly"

"acronym"

"acronyms"

"acrophobia"

"acropolis"

"acropolises"

"across"

"acrostic"

"acrostically"

"acrostics"

"acrylate"

"acrylic"

"acrylics"

"act"

"actable"

"acted"

"actin"

"acting"

"actings"

"actinic"

"actinically"

"actinide"

"actinides"

"actinism"

"actinium"

"actiniums"

"actinograph"

"actinometer"

"action"

"actionability"

"actionable"

"actions"

"activate"

"activated"

"activates"

"activating"

"activation"

"activations"

"activator"

"activators"

"active"

"actively"

"activeness"

"actives"

"activism"

"activisms"

"activist"

"activistic"

"activists"

"activities"

"activity"

"actomyosin"

"actor"

"actorish"

"actors"

"actress"

"actresses"

"acts"

"actual"

"actualities"

"actuality"

"actualization"

"actualize"

"actualized"

"actualizes"

"actualizing"

"actually"

"actuarial"

"actuaries"

"actuary"

"actuate"

"actuated"

"actuates"

"actuating"

"actuation"

"actuator"

"actuators"

"acuities"

"acuity"

"acumen"

"acumens"

"acupressure point massage"

"acupuncture"

"acupuncturist"

"acupuncturists"

"acute"

"acutely"

"acuteness"

"acuter"

"acutes"

"acutest"

"ad"

"adage"

"adages"

"adagial"

"adagio"

"adagios"

"adam"

"adamance"

"adamances"

"adamancies"

"adamancy"

"adamant"

"adamantine"

"adamantly"

"adamants"

"adams"

"adapt"

"adaptability"

"adaptable"

"adaptableness"

"adaptation"

"adaptations"

"adapted"

"adapter"

"adapters"

"adapting"

"adaption"

"adaptions"

"adaptive"

"adaptively"

"adaptiveness"

"adaptometer"

"adaptor"

"adaptors"

"adapts"

"add"

"addable"

"addax"

"added"

"addedly"

"addend"

"addenda"

"addends"

"addendum"

"adder"

"adders"

"addible"

"addict"

"addicted"

"addicting"

"addiction"

"addictions"

"addictive"

"addictively"

"addictiveness"

"addictives"

"addicts"

"adding"

"addison"

"addition"

"additional"

"additionally"

"additions"

"additive"

"additives"

"addle"

"addled"

"addles"

"addling"

"address"

"addressability"

"addressable"

"addressed"

"addressee"

"addressees"

"addresser"

"addressers"

"addresses"

"addressing"

"addrest"

"adds"

"adduce"

"adduceable"

"adduced"

"adducers"

"adduces"

"adducing"

"adduct"

"adducted"

"adducting"

"adduction"

"adductor"

"adductors"

"adenine"

"adenoid"

"adenoidal"

"adenoidectomy"

"adenoidism"

"adenoiditis"

"adenoids"

"adenology"

"adenose"

"adenosine"

"adept"

"adepter"

"adeptest"

"adeptly"

"adeptness"

"adepts"

"adequacies"

"adequacy"

"adequate"

"adequately"

"adequateness"

"adequation"

"adeste"

"ADHD"

"adhere"

"adhered"

"adherence"

"adherent"

"adherents"

"adherer"

"adherers"

"adheres"

"adhering"

"adhesion"

"adhesional"

"adhesions"

"adhesive"

"adhesively"

"adhesiveness"

"adhesives"

"adiabatic"

"adiabatically"

"adiathermancy"

"adieu"

"adieus"

"adieux"

"adios"

"adipose"

"adiposeness"

"adiposis"

"adiposities"

"adiposity"

"adit"

"adits"

"adj"

"adjacency"

"adjacent"

"adjacently"

"adjectival"

"adjectivally"

"adjective"

"adjectives"

"adjoin"

"adjoined"

"adjoining"

"adjoins"

"adjoint"

"adjoints"

"adjourn"

"adjourned"

"adjourning"

"adjournment"

"adjournments"

"adjourns"

"adjudge"

"adjudged"

"adjudges"

"adjudging"

"adjudicate"

"adjudicated"

"adjudicates"

"adjudicating"

"adjudication"

"adjudications"

"adjudicative"

"adjudicator"

"adjudicators"

"adjudicatory"

"adjudicature"

"adjunct"

"adjunctive"

"adjunctly"

"adjuncts"

"adjuration"

"adjurations"

"adjuratory"

"adjure"

"adjured"

"adjurer"

"adjurers"

"adjures"

"adjuring"

"adjuror"

"adjurors"

"adjust"

"adjustable"

"adjusted"

"adjuster"

"adjusters"

"adjusting"

"adjustment"

"adjustments"

"adjustor"

"adjustors"

"adjusts"

"adjutancy"

"adjutant"

"adjutants"

"adman"

"admen"

"admin"

"administer"

"administered"

"administerial"

"administering"

"administerings"

"administers"

"administrable"

"administrant"

"administrants"

"administrate"

"administrated"

"administrates"

"administrating"

"administration"

"administrational"

"administrations"

"administrative"

"administratively"

"administrator"

"administrators"

"administratrices"

"administratrix"

"adminstration"

"admirable"

"admirably"

"admiral"

"admirals"

"admiralship"

"admiralships"

"admiralties"

"admiralty"

"admiration"

"admirations"

"admire"

"admired"

"admirer"

"admirers"

"admires"

"admiring"

"admiringly"

"admissability"

"admissable"

"admissibility"

"admissible"

"admissibly"

"admission"

"admissions"

"admissive"

"admit"

"admits"

"admittance"

"admittances"

"admitted"

"admittedly"

"admitter"

"admitters"

"admitting"

"admix"

"admixed"

"admixes"

"admixing"

"admixt"

"admixture"

"admixtures"

"admonish"

"admonished"

"admonisher"

"admonishes"

"admonishing"

"admonishment"

"admonishments"

"admonition"

"admonitions"

"admonitory"

"ado"

"adobe"

"adobes"

"adolescence"

"adolescent"

"adolescently"

"adolescents"

"adolf"

"adolph"

"adonis"

"adopt"

"adoptabilities"

"adoptability"

"adoptable"

"adopted"

"adoptee"

"adoptees"

"adopter"

"adopters"

"adopting"

"adoption"

"adoptions"

"adoptive"

"adoptively"

"adopts"

"adorability"

"adorable"

"adorableness"

"adorably"

"adoration"

"adore"

"adored"

"adorer"

"adorers"

"adores"

"adoring"

"adorn"

"adorned"

"adorner"

"adorners"

"adorning"

"adornment"

"adornments"

"adorns"

"ados"

"adoze"

"adrenal"

"adrenalin"

"adrenaline"

"adrenals"

"adrenocortical"

"adriatic"

"adrift"

"adroit"

"adroiter"

"adroitest"

"adroitly"

"adroitness"

"ads"

"adsorb"

"adsorbable"

"adsorbate"

"adsorbates"

"adsorbed"

"adsorbent"

"adsorbents"

"adsorbing"

"adsorbs"

"adsorption"

"adsorptive"

"adsorptively"

"adsorptiveness"

"adulate"

"adulated"

"adulates"

"adulating"

"adulation"

"adulator"

"adulators"

"adulatory"

"adult"

"adulterant"

"adulterants"

"adulterate"

"adulterated"

"adulterates"

"adulterating"

"adulteration"

"adulterator"

"adulterators"

"adulterer"

"adulterers"

"adulteress"

"adulteresses"

"adulteries"

"adulterous"

"adulterously"

"adulterousness"

"adultery"

"adulthood"

"adultly"

"adultness"

"adults"

"adumbrate"

"adumbrated"

"adumbrates"

"adumbrating"

"adumbration"

"adumbrations"

"adumbrative"

"adumbratively"

"adv"

"Adv Direct"

"advance"

"advanced"

"advancement"

"advancements"

"advancer"

"advancers"

"advances"

"advancing"

"advantage"

"advantaged"

"advantageous"

"advantageously"

"advantageousness"

"advantages"

"advantaging"

"advent"

"adventitious"

"adventitiously"

"adventitiousness"

"advents"

"adventure"

"adventured"

"adventurer"

"adventurers"

"adventures"

"adventuresome"

"adventuress"

"adventuresses"

"adventuring"

"adventurous"

"adventurously"

"adventurousness"

"adverb"

"adverbial"

"adverbially"

"adverbs"

"adversaries"

"adversary"

"adversative"

"adversatively"

"adverse"

"adversely"

"adverseness"

"adversities"

"adversity"

"advert"

"adverted"

"advertent"

"advertently"

"adverting"

"advertise"

"advertised"

"advertisement"

"advertisements"

"advertiser"

"advertisers"

"advertises"

"advertising"

"advertize"

"advertized"

"advertizement"

"advertizer"

"advertizes"

"advertizing"

"adverts"

"advice"

"advices"

"advisability"

"advisable"

"advisatory"

"advise"

"advised"

"advisedly"

"advisedness"

"advisee"

"advisees"

"advisement"

"adviser"

"advisers"

"advises"

"advising"

"advisor"

"advisories"

"advisors"

"advisory"

"advocacies"

"advocacy"

"advocate"

"advocated"

"advocates"

"advocating"

"advocator"

"advocatory"

"advt"

"adyta"

"adytum"

"adz"

"adzes"

"AED"

"aedoeology"

"aegis"

"aegises"

"aelurophobia"

"aeolian"

"aeon"

"aeonian"

"aeonic"

"aeons"

"aerate"

"aerated"

"aerates"

"aerating"

"aeration"

"aerations"

"aerator"

"aerators"

"aerial"

"aerialist"

"aerialists"

"aerially"

"aerials"

"aerie"

"aeried"

"aerier"

"aeries"

"aeriest"

"aerified"

"aerifies"

"aeriform"

"aerify"

"aerifying"

"aerily"

"aerobatics"

"aerobe"

"aerobes"

"aerobia"

"aerobic"

"aerobically"

"aerobics"

"aerobiology"

"aerodonetics"

"aerodrome"

"aerodromes"

"aerodynamic"

"aerodynamical"

"aerodynamically"

"aerodynamics"

"aerodyne"

"aerofoil"

"aerofoils"

"aerogels"

"aerogram"

"aerograms"

"aerolite"

"aerolites"

"aerolith"

"aerolithology"

"aeroliths"

"aerological"

"aerologist"

"aerologists"

"aerology"

"aerometer"

"aeronaut"

"aeronautic"

"aeronautical"

"aeronautically"

"aeronautics"

"aeronauts"

"aerophilately"

"aerophobia"

"aeroplane"

"aerosol"

"aerosolization"

"aerosolize"

"aerosolized"

"aerosolizing"

"aerosols"

"aerospace"

"aerostat"

"aerostatics"

"aery"

"aeschylus"

"aesop"

"aesopian"

"aesthesia"

"aesthete"

"aesthetes"

"aesthetic"

"aesthetically"

"aesthetics"

"aestivate"

"aestivated"

"aestivates"

"aestivating"

"aether"

"aetheric"

"aethers"

"aethrioscope"

"afar"

"afars"

"afb"

"afeard"

"afeared"

"affability"

"affable"

"affably"

"affair"

"affaire"

"affaires"

"affairs"

"affect"

"affectation"

"affectations"

"affected"

"affectedly"

"affectedness"

"affecter"

"affecters"

"affecting"

"affectingly"

"affection"

"affectionate"

"affectionately"

"affectionless"

"affections"

"affective"

"affectively"

"affectivity"

"affects"

"afferent"

"afferently"

"affiance"

"affianced"

"affiances"

"affiancing"

"affiant"

"affidavit"

"affidavits"

"affiliate"

"affiliated"

"affiliates"

"affiliating"

"affiliation"

"affiliations"

"affinities"

"affinity"

"affirm"

"affirmable"

"affirmably"

"affirmance"

"affirmation"

"affirmations"

"affirmative"

"affirmatively"

"affirmativeness"

"affirmatives"

"affirmed"

"affirmer"

"affirmers"

"affirming"

"affirms"

"affix"

"affixal"

"affixation"

"affixed"

"affixer"

"affixers"

"affixes"

"affixing"

"affixion"

"afflatus"

"afflict"

"afflicted"

"afflicting"

"affliction"

"afflictions"

"afflictive"

"afflictively"

"afflicts"

"affluence"

"affluent"

"affluently"

"affluents"

"afflux"

"affluxes"

"afford"

"affordable"

"afforded"

"affording"

"affords"

"afforest"

"afforestation"

"afforested"

"afforesting"

"afforests"

"affray"

"affrayed"

"affrayer"

"affrayers"

"affraying"

"affrays"

"affright"

"affrighted"

"affrights"

"affront"

"affronted"

"affronting"

"affronts"

"affusions"

"afghan"

"afghani"

"afghanis"

"afghanistan"

"afghans"

"aficionado"

"aficionados"

"afield"

"afire"

"aflame"

"aflatoxin"

"afloat"

"aflutter"

"afoot"

"afore"

"aforementioned"

"aforesaid"

"aforethought"

"afoul"

"afraid"

"afreet"

"afreets"

"afresh"

"africa"

"african"

"africans"

"afrikaans"

"afrit"

"afrits"

"afro"

"afros"

"aft"

"after"

"afterbirth"

"afterbirths"

"afterburner"

"afterburners"

"aftercare"

"afterdeck"

"afterdecks"

"afterdischarge"

"aftereffect"

"aftereffects"

"afterglow"

"afterglows"

"afterimage"

"afterimages"

"afterimpression"

"afterlife"

"afterlives"

"aftermarket"

"aftermath"

"aftermaths"

"aftermost"

"afternoon"

"afternoons"

"afterpotential"

"afters"

"aftershave"

"aftershaves"

"aftertaste"

"aftertastes"

"afterthought"

"afterthoughts"

"afterward"

"afterwards"

"aftmost"

"again"

"against"

"agama"

"agamas"

"agamic"

"agapae"

"agape"

"agapeic"

"agar"

"agarics"

"agars"

"agate"

"agates"

"agatize"

"agave"

"agaves"

"agaze"

"age"

"AGE-SPECIFIC PEDIATRIC ECG VALUES"

"aged"

"agedly"

"agedness"

"ageing"

"ageings"

"ageism"

"ageist"

"ageists"

"ageless"

"agelessly"

"agelessness"

"agelong"

"agencies"

"agency"

"agenda"

"agendas"

"agendum"

"agendums"

"agent"

"agentry"

"agents"

"ageratum"

"ageratums"

"agers"

"ages"

"aggie"

"aggies"

"agglomerate"

"agglomerated"

"agglomerates"

"agglomerating"

"agglomeration"

"agglomerations"

"agglutinate"

"agglutinated"

"agglutinates"

"agglutinating"

"agglutination"

"agglutinations"

"agglutinative"

"agglutinatively"

"agglutinin"

"agglutinins"

"aggrandize"

"aggrandized"

"aggrandizement"

"aggrandizements"

"aggrandizer"

"aggrandizers"

"aggrandizes"

"aggrandizing"

"aggravate"

"aggravated"

"aggravates"

"aggravating"

"aggravation"

"aggravations"

"aggregate"

"aggregated"

"aggregates"

"aggregating"

"aggregation"

"aggregational"

"aggregations"

"aggregative"

"aggress"

"aggressed"

"aggresses"

"aggressing"

"aggression"

"aggressions"

"aggressive"

"aggressively"

"aggressiveness"

"aggressor"

"aggressors"

"aggrieve"

"aggrieved"

"aggrieves"

"aggrieving"

"agha"

"aghas"

"aghast"

"agile"

"agilely"

"agileness"

"agilities"

"agility"

"agin"

"aging"

"agings"

"agist"

"agists"

"agitable"

"agitate"

"agitated"

"agitatedly"

"agitates"

"agitating"

"agitation"

"agitations"

"agitato"

"agitator"

"agitators"

"agitprop"

"agitprops"

"aglare"

"agleam"

"aglee"

"aglet"

"aglets"

"agley"

"aglimmer"

"aglitter"

"aglow"

"agnizing"

"agnostic"

"agnosticism"

"agnostics"

"ago"

"agog"

"agonal"

"agone"

"agonic"

"agonies"

"agonise"

"agonised"

"agonises"

"agonist"

"agonistic"

"agonistics"

"agonists"

"agonize"

"agonized"

"agonizes"

"agonizing"

"agonizingly"

"agons"

"agony"

"agora"

"agorae"

"agoraphobia"

"agoraphobic"

"agoras"

"agouti"

"agouties"

"agouty"

"agrarian"

"agrarianism"

"agrarians"

"agree"

"agreeability"

"agreeable"

"agreeableness"

"agreeably"

"agreed"

"agreeing"

"agreement"

"agreements"

"agrees"

"agribusiness"

"agribusinesses"

"agric"

"agricultural"

"agriculturalist"

"agriculturalists"

"agriculturally"

"agriculture"

"agricultures"

"agriculturist"

"agriculturists"

"agrimony"

"agriology"

"agrobiology"

"agrology"

"agromania"

"agronomic"

"agronomics"

"agronomies"

"agronomist"

"agronomists"

"agronomy"

"agrostology"

"aground"

"ague"

"agues"

"agueweed"

"aguishly"

"agyiophobia"

"ah"

"aha"

"ahchoo"

"AHDI"

"ahead"

"ahem"

"ahems"

"ahimsa"

"ahimsas"

"ahold"

"ahorse"

"ahoy"

"ahs"

"ai"

"aichmophobia"

"aid"

"aide"

"aided"

"aider"

"aiders"

"aides"

"aidful"

"aiding"

"aidless"

"aidman"

"aidmen"

"aids"

"aiglets"

"aigrets"

"aigrette"

"aigrettes"

"aikido"

"aikidos"

"ail"

"ailanthus"

"ailanthuses"

"ailed"

"aileron"

"ailerons"

"ailing"

"ailment"

"ailments"

"ails"

"ailurophobe"

"ailurophobia"

"aim"

"aimed"

"aimer"

"aimers"

"aimful"

"aimfully"

"aiming"

"aimless"

"aimlessly"

"aimlessness"

"aims"

"ainus"

"air"

"airbill"

"airbills"

"airboat"

"airboats"

"airborne"

"airbrush"

"airbrushed"

"airbrushes"

"airbrushing"

"airbursts"

"airbus"

"airbusses"

"aircraft"

"aircrew"

"airdrome"

"airdromes"

"airdrop"

"airdropped"

"airdropping"

"airdrops"

"aired"

"airedale"

"airedales"

"airer"

"airest"

"airfare"

"airfares"

"airfield"

"airfields"

"airflow"

"airflows"

"airfoil"

"airfoils"

"airframe"

"airframes"

"airfreight"

"airglow"

"airhead"

"airheads"

"airier"

"airiest"

"airily"

"airiness"

"airing"

"airings"

"airless"

"airlessly"

"airlessness"

"airlift"

"airlifted"

"airlifting"

"airlifts"

"airlike"

"airline"

"airliner"

"airliners"

"airlines"

"airlock"

"airmail"

"airmailed"

"airmailing"

"airmails"

"airman"

"airmanship"

"airmen"

"airmobile"

"airplane"

"airplanes"

"airport"

"airports"

"airproofed"

"airs"

"airscrew"

"airscrews"

"airship"

"airships"

"airsick"

"airsickness"

"airspace"

"airspaces"

"airspeed"

"airspeeds"

"airstream"

"airstrip"

"airstrips"

"airtight"

"airwave"

"airwaves"

"airway"

"airways"

"airwoman"

"airwomen"

"airworthier"

"airworthiest"

"airworthiness"

"airworthy"

"airy"

"aisle"

"aisled"

"aisles"

"ait"

"aitch"

"aitches"

"ajar"

"ajiva"

"ajowans"

"akenes"

"akimbo"

"akin"

"akron"

"akvavit"

"akvavits"

"al"

"ala"

"alabama"

"alabamian"

"alabamians"

"alabaster"

"alack"

"alacrities"

"alacrity"

"aladdin"

"alai"

"alameda"

"alamedas"

"alamo"

"alamode"

"alamodes"

"alamos"

"alan"

"alans"

"alar"

"alarm"

"alarm clock"

"alarm sounds"

"alarm system"

"alarmclock"

"alarmed"

"alarming"

"alarmingly"

"alarmism"

"alarmisms"

"alarmist"

"alarmists"

"alarms"

"alarum"

"alarumed"

"alaruming"

"alarums"

"alary"

"alas"

"alaska"

"alaskan"

"alaskans"

"alaskas"

"alate"

"alated"

"alb"

"alba"

"albacore"

"albacores"

"albania"

"albanian"

"albanians"

"albany"

"albatross"

"albatrosses"

"albedo"

"albedos"

"albeit"

"albert"

"alberta"

"albinism"

"albinisms"

"albino"

"albinoism"

"albinos"

"albs"

"album"

"albumen"

"albumens"

"albumin"

"albuminous"

"albumins"

"albums"

"albuquerque"

"alcalde"

"alcaldes"

"alcazar"

"alcazars"

"alchemic"

"alchemical"

"alchemies"

"alchemist"

"alchemists"

"alchemy"

"alchymies"

"alcohol"

"alcoholic"

"alcoholically"

"alcoholics"

"alcoholism"

"alcoholization"

"alcoholized"

"alcoholizing"

"alcoholometer"

"alcohols"

"alcove"

"alcoved"

"alcoves"

"alcovinometer"

"aldehyde"

"aldehydes"

"alder"

"alderman"

"aldermanic"

"aldermanry"

"aldermen"

"alders"

"alderwoman"

"alderwomen"

"aldrin"

"aldrins"

"ale"

"aleatory"

"alecs"

"alee"

"alefs"

"alehouse"

"alehouses"

"alembic"

"alembics"

"aleph"

"alephs"

"alert"

"alert card"

"alerted"

"alerter"

"alerters"

"alertest"

"alerting"

"alertly"

"alertness"

"alerts"

"ales"

"alestinian"

"alethiology"

"aleuron"

"aleutian"

"aleutians"

"alewife"

"alewives"

"alexander"

"alexandria"

"alexandrian"

"alexandrine"

"alexandrines"

"alexia"

"alfa"

"alfalfa"

"alfalfas"

"alfas"

"alfred"

"alfresco"

"alga"

"algae"

"algal"

"algas"

"algebra"

"algebraic"

"algebraically"

"algebras"

"algedonics"

"algeria"

"algerian"

"algerians"

"algicide"

"algicides"

"algid"

"algiers"

"algin"

"alginate"

"alginates"

"algins"

"algoid"

"algology"

"algometer"

"algonquian"

"algonquians"

"algonquin"

"algonquins"

"algophobia"

"algorism"

"algorisms"

"algorithm"

"algorithmic"

"algorithms"

"alias"

"aliases"

"alibi"

"alibied"

"alibies"

"alibiing"

"alibis"

"alice"

"alien"

"alienabilities"

"alienability"

"alienable"

"alienage"

"alienages"

"alienate"

"alienated"

"alienates"

"alienating"

"alienation"

"alienator"

"aliened"

"alienee"

"alienees"

"aliener"

"alieners"

"aliening"

"alienism"

"alienisms"

"alienist"

"alienists"

"alienly"

"alienors"

"aliens"

"alight"

"alighted"

"alighting"

"alights"

"align"

"aligned"

"aligner"

"aligners"

"aligning"

"alignment"

"alignments"

"aligns"

"alii"

"alike"

"alikeness"

"aliment"

"alimentary"

"alimentation"

"alimented"

"alimenting"

"aliments"

"alimonies"

"alimony"

"aline"

"alined"

"alinement"

"aliner"

"aliners"

"alines"

"alining"

"aliphatic"

"aliquant"

"aliquot"

"aliquots"

"alit"

"aliter"

"alive"

"aliveness"

"alizarin"

"alizarine"

"alizarins"

"alk"

"alkali"

"alkalic"

"alkalies"

"alkalify"

"alkalimeter"

"alkalin"

"alkaline"

"alkalinities"

"alkalinity"

"alkalinization"

"alkalinize"

"alkalinized"

"alkalinizes"

"alkalinizing"

"alkalis"

"alkalise"

"alkalization"

"alkalize"

"alkalized"

"alkalizes"

"alkalizing"

"alkaloid"

"alkaloids"

"alkalosis"

"alkyd"

"alkyds"

"alkyl"

"alkyls"

"all"

"all-natural method"

"allah"

"allay"

"allayed"

"allayer"

"allayers"

"allaying"

"allayment"

"allays"

"allegation"

"allegations"

"allegator"

"allege"

"allegeable"

"alleged"

"allegedly"

"allegement"

"alleger"

"allegers"

"alleges"

"allegheny"

"allegiance"

"allegiances"

"allegiant"

"allegiantly"

"alleging"

"allegoric"

"allegorical"

"allegorically"

"allegories"

"allegorist"

"allegorists"

"allegory"

"allegretto"

"allegro"

"allegros"

"allele"

"alleles"

"allelic"

"alleluia"

"alleluias"

"allen"

"aller"

"allergen"

"allergenic"

"allergenicity"

"allergens"

"allergic"

"allergies"

"allergin"

"allergist"

"allergists"

"allergology"

"allergy"

"allergy alert"

"Allergy alert

weather channel pollen"

"alleviate"

"alleviated"

"alleviates"

"alleviating"

"alleviation"

"alleviations"

"alleviative"

"alleviator"

"alleviators"

"alleviatory"

"alley"

"alleys"

"alleyway"

"alleyways"

"allheal"

"allheals"

"alliable"

"alliance"

"alliances"

"allied"

"allies"

"alligator"

"alligators"

"alliterate"

"alliterated"

"alliterates"

"alliterating"

"alliteration"

"alliterations"

"alliterative"

"alliteratively"

"allium"

"alliums"

"allocability"

"allocable"

"allocate"

"allocated"

"allocatee"

"allocates"

"allocating"

"allocation"

"allocations"

"allocator"

"allocators"

"allogenic"

"allomorphism"

"allopathies"

"allopaths"

"allopathy"

"allot"

"alloted"

"allotment"

"allotments"

"allotrope"

"allotropes"

"allotrophic"

"allotropic"

"allotropically"

"allotropies"

"allotropism"

"allotropy"

"allots"

"allottable"

"allotted"

"allottee"

"allottees"

"allotter"

"allotters"

"allotting"

"allotypes"

"allotypic"

"allotypically"

"allover"

"allovers"

"allow"

"allowable"

"allowance"

"allowances"

"allowed"

"allowing"

"allows"

"alloy"

"alloyed"

"alloying"

"alloys"

"alls"

"allspice"

"allspices"

"allude"

"alluded"

"alludes"

"alluding"

"allure"

"allured"

"allurement"

"allurements"

"allurer"

"allurers"

"allures"

"alluring"

"alluringly"

"allusion"

"allusions"

"allusive"

"allusively"

"allusiveness"

"alluvia"

"alluvial"

"alluvials"

"alluvium"

"alluviums"

"ally"

"allying"

"allyls"

"alma"

"almanac"

"almanacs"

"almandine"

"almandines"

"almightily"

"almightiness"

"almighty"

"almner"

"almners"

"almond"

"almonds"

"almoner"

"almoners"

"almonry"

"almost"

"alms"

"almshouse"

"almshouses"

"almsman"

"almsmen"

"alnico"

"alnicoes"

"aloe"

"aloes"

"aloft"

"aloha"

"alohas"

"alone"

"aloneness"

"along"

"alongshore"

"alongside"

"aloof"

"aloofly"

"aloofness"

"alopecia"

"alopecias"

"alopecic"

"aloud"

"alp"

"alpaca"

"alpacas"

"alpenhorn"

"alpenhorns"

"alpenstock"

"alpenstocks"

"alpha"

"alphabet"

"alphabeted"

"alphabetic"

"alphabetical"

"alphabetically"

"alphabetization"

"alphabetize"

"alphabetized"

"alphabetizer"

"alphabetizers"

"alphabetizes"

"alphabetizing"

"alphabets"

"alphameric"

"alphanumeric"

"alphanumerics"

"alphas"

"alphorn"

"alphorns"

"alpine"

"alpinely"

"alpines"

"alpinism"

"alpinisms"

"alpinist"

"alpinists"

"alps"

"already"

"alright"

"also"

"alt"

"altar"

"altarpiece"

"altarpieces"

"altars"

"alter"

"alterability"

"alterable"

"alterably"

"alterant"

"alterants"

"alteration"

"alterations"

"alterative"

"alteratively"

"altercation"

"altercations"

"altered"

"alterer"

"alterers"

"altering"

"alternate"

"alternated"

"alternately"

"alternateness"

"alternates"

"alternatim"

"alternating"

"alternatingly"

"alternation"

"alternations"

"alternative"

"alternatively"

"alternativeness"

"alternatives"

"alternator"

"alternators"

"alters"

"althea"

"altho"

"althorn"

"althorns"

"although"

"altimeter"

"altimeters"

"altitude"

"altitudes"

"alto"

"altogether"

"altos"

"altruism"

"altruisms"

"altruist"

"altruistic"

"altruistically"

"altruists"

"alum"

"alumin"

"alumina"

"aluminas"

"alumine"

"alumines"

"aluminic"

"aluminize"

"aluminized"

"aluminizes"

"aluminizing"

"alumins"

"aluminum"

"aluminums"

"alumna"

"alumnae"

"alumni"

"alumnus"

"alumroot"

"alumroots"

"alums"

"alveolar"

"alveolars"

"alveolate"

"alveoli"

"alveolus"

"alway"

"always"

"alyssum"

"alyssums"

"alzheimer"

"am"

"ama"

"amah"

"amahs"

"amain"

"amalgam"

"amalgamate"

"amalgamated"

"amalgamates"

"amalgamating"

"amalgamation"

"amalgamative"

"amalgamator"

"amalgamators"

"amalgams"

"amandine"

"amanita"

"amanitas"

"amanuenses"

"amanuensis"

"amaranth"

"amaranthine"

"amaranths"

"amarettos"

"amarillo"

"amaryllis"

"amaryllises"

"amass"

"amassed"

"amasser"

"amassers"

"amasses"

"amassing"

"amassment"

"amassments"

"amateur"

"amateurish"

"amateurishly"

"amateurishness"

"amateurism"

"amateurs"

"amathophobia"

"amative"

"amatively"

"amativeness"

"amatorially"

"amatory"

"amaxophobia"

"amaze"

"amazed"

"amazedly"

"amazement"

"amazes"

"amazing"

"amazingly"

"amazon"

"amazonian"

"amazons"

"ambassador"

"ambassadorial"

"ambassadors"

"ambassadorship"

"ambassadorships"

"ambassadress"

"amber"

"ambergrease"

"ambergris"

"ambers"

"ambery"

"ambiance"

"ambidexter"

"ambidexterities"

"ambidexterity"

"ambidextrous"

"ambidextrously"

"ambidextrousness"

"ambience"

"ambience and white noise mixer"

"ambiences"

"ambient"

"ambients"

"ambiguities"

"ambiguity"

"ambiguous"

"ambiguously"

"ambiguousness"

"ambilateral"

"ambisexualities"

"ambisexuality"

"ambition"

"ambitioned"

"ambitions"

"ambitious"

"ambitiously"

"ambitiousness"

"ambivalence"

"ambivalent"

"ambivalently"

"ambivert"

"ambiverts"

"amble"

"ambled"

"ambler"

"amblers"

"ambles"

"ambling"

"ambrosia"

"ambrosial"

"ambrosially"

"ambrosias"

"ambulance"

"ambulances"

"ambulant"

"ambulate"

"ambulated"

"ambulates"

"ambulating"

"ambulation"

"ambulator"

"ambulatories"

"ambulators"

"ambulatory"

"ambulophobia"

"ambuscade"

"ambuscaded"

"ambuscades"

"ambuscading"

"ambush"

"ambushed"

"ambusher"

"ambushers"

"ambushes"

"ambushing"

"ambushment"

"ameba"

"amebae"

"ameban"

"amebas"

"amebean"

"amebic"

"ameboid"

"ameer"

"ameerate"

"ameers"

"ameliorate"

"ameliorated"

"ameliorates"

"ameliorating"

"amelioration"

"ameliorations"

"ameliorative"

"amen"

"amenability"

"amenable"

"amenably"

"amend"

"amendable"

"amendatory"

"amended"

"amender"

"amenders"

"amending"

"amendment"

"amendments"

"amends"

"amenities"

"amenity"

"amens"

"ament"

"aments"

"amerce"

"amerced"

"amercement"

"amercements"

"amerces"

"amercing"

"america"

"american"

"american measuring system"

"American Pregnancy Association"

"americana"

"americanism"

"americanisms"

"americanist"

"americanization"

"americanize"

"americanized"

"americanizes"

"americanizing"

"americans"

"americas"

"americium"

"amerind"

"amerindian"

"amerindians"

"amerinds"

"amerism"

"amethyst"

"amethysts"

"amex"

"amiability"

"amiable"

"amiableness"

"amiably"

"amias"

"amicabilities"

"amicability"

"amicable"

"amicableness"

"amicably"

"amice"

"amici"

"amicus"

"amid"

"amide"

"amides"

"amidic"

"amids"

"amidship"

"amidships"

"amidst"

"amies"

"amigas"

"amigo"

"amigos"

"amines"

"aminic"

"aminity"

"amino"

"aminoglycosides"

"amirate"

"amirates"

"amire"

"amirs"

"amis"

"amish"

"amiss"

"amities"

"amity"

"ammeter"

"ammeters"

"ammine"

"ammino"

"ammo"

"ammonia"

"ammoniac"

"ammoniacs"

"ammonias"

"ammoniate"

"ammoniating"

"ammonic"

"ammonify"

"ammonite"

"ammonites"

"ammonium"

"ammoniums"

"ammonoid"

"ammos"

"ammunition"

"amnesia"

"amnesiac"

"amnesiacs"

"amnesias"

"amnesic"

"amnesics"

"amnestic"

"amnestied"

"amnesties"

"amnesty"

"amnestying"

"amniocentesis"

"amnion"

"amnionic"

"amnions"

"amniote"

"amniotes"

"amniotic"

"amoeba"

"amoebae"

"amoeban"

"amoebas"

"amoebean"

"amoebic"

"amoeboid"

"amoebous"

"amok"

"amoks"

"amole"

"amoles"

"among"

"amongst"

"amontillado"

"amontillados"

"amoral"

"amorality"

"amorally"

"amoretti"

"amoretto"

"amorists"

"amoroso"

"amorous"

"amorously"

"amorousness"

"amorphous"

"amorphously"

"amorphousness"

"amort"

"amortise"

"amortizable"

"amortization"

"amortize"

"amortized"

"amortizement"

"amortizes"

"amortizing"

"amount"

"amounted"

"amounting"

"amounts"

"amour"

"amours"

"amove"

"amp"

"amperage"

"amperages"

"ampere"

"amperes"

"ampersand"

"ampersands"

"amphetamine"

"amphetamines"

"amphibia"

"amphibian"

"amphibians"

"amphibious"

"amphibiousness"

"amphibole"

"amphiboles"

"amphitheater"

"amphitheaters"

"amphora"

"amphorae"

"amphoral"

"amphoras"

"ampicillin"

"ampitheater"

"ample"

"ampleness"

"ampler"

"amplest"

"amplifiable"

"amplification"

"amplifications"

"amplified"

"amplifier"

"amplifiers"

"amplifies"

"amplify"

"amplifying"

"amplitude"

"amplitudes"

"amply"

"ampoule"

"ampoules"

"amps"

"ampul"

"ampule"

"ampules"

"ampulla"

"ampuls"

"amputate"

"amputated"

"amputates"

"amputating"

"amputation"

"amputations"

"amputator"

"amputee"

"amputees"

"amreeta"

"amreetas"

"amrita"

"amritas"

"amsterdam"

"amtrac"

"amtrack"

"amtracks"

"amtracs"

"amtrak"

"amu"

"amuck"

"amucks"

"amulet"

"amulets"

"amusable"

"amuse"

"amused"

"amusedly"

"amusement"

"amusements"

"amuser"

"amusers"

"amuses"

"amusing"

"amusingly"

"amyl"

"amylase"

"amylases"

"amyls"

"an"

"ana"

"anabolic"

"anabolism"

"anachronism"

"anachronisms"

"anachronistic"

"anachronistical"

"anachronistically"

"anaconda"

"anacondas"

"anadem"

"anadems"

"anadian"

"anaemia"

"anaemias"

"anaemic"

"anaerobe"

"anaerobes"

"anaerobic"

"anaerobically"

"anaesthesia"

"anaesthesiology"

"anaesthetic"

"anaesthetist"

"anaesthetization"

"anaesthetize"

"anaesthetized"

"anaesthetizing"

"anaglyptics"

"anagram"

"anagrammed"

"anagrams"

"anagraphy"

"anaheim"

"anal"

"analects"

"analemma"

"analemmas"

"analeptic"

"analgesia"

"analgesic"

"analgesics"

"analgia"

"anality"

"anally"

"analog"

"analogic"

"analogical"

"analogically"

"analogies"

"analogize"

"analogous"

"analogously"

"analogousness"

"analogs"

"analogue"

"analogues"

"analogy"

"analysand"

"analysands"

"analyse"

"analysed"

"analyser"

"analyses"

"analysis"

"analyst"

"analysts"

"analytic"

"analytical"

"analytically"

"analyzable"

"analyze"

"analyzed"

"analyzer"

"analyzers"

"analyzes"

"analyzing"

"anapest"

"anapestic"

"anapests"

"anarch"

"anarchic"

"anarchical"

"anarchically"

"anarchies"

"anarchism"

"anarchist"

"anarchistic"

"anarchists"

"anarchs"

"anarchy"

"anastigmatic"

"anastomoses"

"anastomosis"

"anatase"

"anathema"

"anathemas"

"anathemata"

"anathematize"

"anathematized"

"anathematizes"

"anathematizing"

"anatomic"

"anatomical"

"anatomically"

"anatomies"

"anatomist"

"anatomists"

"anatomize"

"anatomized"

"anatomizes"

"anatomizing"

"anatomy"

"anatomy images"

"anatto"

"anattos"

"ancestor"

"ancestors"

"ancestral"

"ancestrally"

"ancestress"

"ancestresses"

"ancestries"

"ancestry"

"anchor"

"anchorage"

"anchorages"

"anchored"

"anchoress"

"anchoresses"

"anchoring"

"anchorite"

"anchorites"

"anchoritic"

"anchors"

"anchovies"

"anchovy"

"ancien"

"anciens"

"ancient"

"ancienter"

"ancientest"

"anciently"

"ancientness"

"ancients"

"ancillaries"

"ancillary"

"and"

"andante"

"andantes"

"andantino"

"andantinos"

"andean"

"anderson"

"andes"

"andesite"

"andesyte"

"andiron"

"andirons"

"andor"

"andorra"

"andragogy"

"andre"

"andrew"

"androgen"

"androgenic"

"androgens"

"androgyne"

"androgynies"

"androgynism"

"androgynous"

"androgyny"

"android"

"Android application"

"androids"

"andromania"

"andromeda"

"ands"

"anear"

"anearing"

"anecdotal"

"anecdote"

"anecdotes"

"anecdotic"

"anecdotist"

"anecdotists"

"anechoic"

"anele"

"anemia"

"anemias"

"anemic"

"anemograph"

"anemology"

"anemometer"

"anemometers"

"anemone"

"anemones"

"anent"

"anergy"

"aneroid"

"aneroids"

"anesthesia"

"anesthesiologies"

"anesthesiologist"

"anesthesiologists"

"anesthesiology"

"anesthetic"

"anesthetically"

"anesthetics"

"anesthetist"

"anesthetists"

"anesthetization"

"anesthetize"

"anesthetized"

"anesthetizes"

"anesthetizing"

"aneurism"

"aneurisms"

"aneurysm"

"aneurysms"

"anew"

"angaries"

"angary"

"angas"

"angel"

"angeles"

"angelfish"

"angelfishes"

"angelic"

"angelica"

"angelical"

"angelically"

"angelicas"

"angelology"

"angels"

"angelus"

"angeluses"

"anger"

"angered"

"angering"

"angerly"

"angers"

"angina"

"anginal"

"anginas"

"anginous"

"angiogram"

"angiology"

"angiosperm"

"angiosperms"

"angle"

"angled"

"angler"

"anglers"

"angles"

"angleworm"

"angleworms"

"anglians"

"anglican"

"anglicanism"

"anglicans"

"anglicism"

"anglicisms"

"anglicization"

"anglicize"

"anglicized"

"anglicizes"

"anglicizing"

"angling"

"anglings"

"anglo"

"anglomania"

"anglophile"

"anglophiles"

"anglophilia"

"anglophobe"

"anglophobes"

"anglophobia"

"anglos"

"angola"

"angolan"

"angolans"

"angora"

"angoras"

"angostura"

"angrier"

"angriest"

"angrily"

"angry"

"angst"

"angstrom"

"angstroms"

"angsts"

"anguish"

"anguished"

"anguishes"

"anguishing"

"angular"

"angularities"

"angularity"

"angularly"

"angularness"

"angulating"

"angus"

"anguses"

"anhydride"

"anhydrides"

"anhydrous"

"anile"

"anilin"

"aniline"

"anilines"

"anilins"

"anilities"

"anility"

"anils"

"anima"

"animadversion"

"animadversions"

"animadvert"

"animadverted"

"animadverting"

"animadverts"

"animal"

"animal science"

"animalcule"

"animalcules"

"animalism"

"animalistic"

"animalities"

"animality"

"animally"

"animals"

"animas"

"animate"

"animated"

"animater"

"animaters"

"animates"

"animating"

"animation"

"animations"

"animato"

"animator"

"animators"

"animism"

"animisms"

"animist"

"animistic"

"animists"

"animo"

"animosities"

"animosity"

"animus"

"animuses"

"anion"

"anionic"

"anionically"

"anions"

"anis"

"anise"

"aniseed"

"aniseeds"

"anises"

"anisette"

"anisettes"

"anisic"

"anitinstitutionalism"

"ankara"

"ankh"

"ankhs"

"ankle"

"anklebone"

"anklebones"

"ankles"

"anklet"

"anklets"

"ankus"

"ankuses"

"ann"

"anna"

"annal"

"annalist"

"annalists"

"annals"

"annapolis"

"annas"

"annat"

"annatto"

"annattos"

"anne"

"anneal"

"annealed"

"annealer"

"annealers"

"annealing"

"anneals"

"annelid"

"annelids"

"annex"

"annexation"

"annexational"

"annexations"

"annexed"

"annexes"

"annexing"

"annexion"

"annexure"

"annie"

"annihilate"

"annihilated"

"annihilates"

"annihilating"

"annihilation"

"annihilator"

"annihilators"

"anniversaries"

"anniversary"

"anno"

"annotate"

"annotated"

"annotates"

"annotating"

"annotation"

"annotations"

"annotative"

"annotatively"

"annotativeness"

"annotator"

"annotators"

"announce"

"announced"

"announcement"

"announcements"

"announcer"

"announcers"

"announces"

"announcing"

"annoy"

"annoyance"

"annoyances"

"annoyed"

"annoyer"

"annoyers"

"annoying"

"annoyingly"

"annoys"

"annual"

"annualized"

"annually"

"annuals"

"annuitant"

"annuitants"

"annuities"

"annuity"

"annul"

"annular"

"annularity"

"annulate"

"annuler"

"annulet"

"annuli"

"annullable"

"annulled"

"annulling"

"annulment"

"annulments"

"annuls"

"annulus"

"annuluses"

"annum"

"annunciate"

"annunciated"

"annunciates"

"annunciating"

"annunciation"

"annunciations"

"annunciator"

"annunciators"

"annunciatory"

"anodal"

"anodally"

"anode"

"anodes"

"anodic"

"anodically"

"anodization"

"anodize"

"anodized"

"anodizes"

"anodizing"

"anodyne"

"anodynes"

"anodynic"

"anoia"

"anoint"

"anointed"

"anointer"

"anointers"

"anointing"

"anointment"

"anointments"

"anoints"

"anole"

"anoles"

"anomalies"

"anomalistic"

"anomalous"

"anomaly"

"anomia"

"anomic"

"anomie"

"anomies"

"anomy"

"anon"

"anonym"

"anonyma"

"anonymities"

"anonymity"

"anonymous"

"anonymously"

"anonymousness"

"anonyms"

"anopheles"

"anopia"

"anorak"

"anoraks"

"anorectic"

"anorexia"

"anorexias"

"anorexy"

"another"

"anoxia"

"anoxias"

"anoxic"

"anschluss"

"ansi"

"answer"

"answerability"

"answerable"

"answered"

"answerer"

"answerers"

"answering"

"answers"

"ant"

"antacid"

"antacids"

"antagonism"

"antagonisms"

"antagonist"

"antagonistic"

"antagonistically"

"antagonists"

"antagonize"

"antagonized"

"antagonizes"

"antagonizing"

"antarctic"

"antarctica"

"ante"

"anteater"

"anteaters"

"antebellum"

"antecede"

"anteceded"

"antecedence"

"antecedent"

"antecedental"

"antecedently"

"antecedents"

"antecedes"

"anteceding"

"antechamber"

"antechambers"

"antechoir"

"antechoirs"

"anted"

"antedate"

"antedated"

"antedates"

"antedating"

"antediluvian"

"anteed"

"antefix"

"anteing"

"antelope"

"antelopes"

"antemortem"

"antenna"

"antennae"

"antennal"

"antennas"

"antepartum"

"antepast"

"antepenult"

"antepenultimate"

"antepenults"

"anteposition"

"anterior"

"anteriorly"

"anteroom"

"anterooms"

"antes"

"anthem"

"anthemed"

"anthems"

"anther"

"antheral"

"anthers"

"anthill"

"anthills"

"anthologies"

"anthologist"

"anthologists"

"anthologize"

"anthologized"

"anthologizes"

"anthologizing"

"anthology"

"anthomania"

"anthony"

"anthraces"

"anthracite"

"anthracitic"

"anthralin"

"anthrax"

"anthrop"

"anthrophobia"

"anthropobiology"

"anthropocentric"

"anthropoid"

"anthropoidea"

"anthropoids"

"anthropologic"

"anthropological"

"anthropologically"

"anthropologies"

"anthropologist"

"anthropologists"

"anthropology"

"anthropomorphic"

"anthropomorphically"

"anthropomorphism"

"anthropomorphisms"

"anthropophagy"

"anthroposophy"

"anti"

"antiabortion"

"antiacid"

"antiaircraft"

"antibacterial"

"antibiotic"

"antibiotics"

"antibodies"

"antibody"

"antibusing"

"antic"

"anticancer"

"anticapitalist"

"anticapitalists"

"antichrist"

"antichrists"

"anticipate"

"anticipated"

"anticipates"

"anticipating"

"anticipation"

"anticipations"

"anticipative"

"anticipator"

"anticipators"

"anticipatory"

"anticked"

"anticlerical"

"anticlimactic"

"anticlimactically"

"anticlimax"

"anticlimaxes"

"anticlinal"

"anticline"

"anticlines"

"anticly"

"anticoagulant"

"anticoagulants"

"anticoagulating"

"anticommunism"

"anticommunist"

"anticommunists"

"anticonvulsant"

"anticonvulsive"

"anticorrosive"

"anticorrosives"

"antics"

"anticyclone"

"anticyclones"

"anticyclonic"

"antidemocratic"

"antidepressant"

"antidepressants"

"antidepressive"

"antidisestablishmentarian"

"antidisestablishmentarianism"

"antidotal"

"antidotally"

"antidote"

"antidotes"

"antielectron"

"antielectrons"

"antienvironmentalism"

"antienvironmentalist"

"antienvironmentalists"

"antifascism"

"antifascist"

"antifascists"

"antifertility"

"antifreeze"

"antifreezes"

"antifungal"

"antigen"

"antigene"

"antigenic"

"antigenically"

"antigenicity"

"antigens"

"antigravity"

"antihero"

"antiheroes"

"antiheroic"

"antihistamine"

"antihistamines"

"antihistaminic"

"antihumanism"

"antihypertensive"

"antihypertensives"

"antiinflammatories"

"antiinflammatory"

"antiinstitutionalist"

"antiinstitutionalists"

"antiinsurrectionally"

"antiinsurrectionists"

"antiknock"

"antiknocks"

"antilabor"

"antiliberal"

"antiliberals"

"antilles"

"antilogarithm"

"antilogarithms"

"antilogs"

"antimacassar"

"antimacassars"

"antimagnetic"

"antimalarial"

"antimatter"

"antimicrobial"

"antimilitarism"

"antimilitaristic"

"antimissile"

"antimonarchist"

"antimonarchists"

"antimonies"

"antimonopolistic"

"antimony"

"antinarcotic"

"antinarcotics"

"antinationalist"

"antinationalists"

"antineoplastic"

"antineutrino"

"antineutrinos"

"antineutron"

"antineutrons"

"anting"

"antings"

"antinoise"

"antinomian"

"antinomianism"

"antinomians"

"antinomies"

"antinomy"

"antinovel"

"antinovels"

"antinucleon"

"antinucleons"

"antioxidant"

"antioxidants"

"antipacifist"

"antipacifists"

"antiparliamentarian"

"antiparliamentarians"

"antiparticle"

"antiparticles"

"antipasti"

"antipasto"

"antipastos"

"antipathetic"

"antipathies"

"antipathy"

"antipersonnel"

"antiperspirant"

"antiperspirants"

"antiphon"

"antiphonal"

"antiphonally"

"antiphonic"

"antiphonically"

"antiphonies"

"antiphons"

"antiphony"

"antipodal"

"antipode"

"antipodean"

"antipodeans"

"antipodes"

"antipole"

"antipoles"

"antipollution"

"antipope"

"antipopes"

"antipoverty"

"antiprohibition"

"antiproton"

"antiprotons"

"antipyresis"

"antipyretic"

"antipyretics"

"antiquarian"

"antiquarianism"

"antiquarians"

"antiquaries"

"antiquary"

"antiquate"

"antiquated"

"antiquates"

"antiquating"

"antiquation"

"antique"

"antiqued"

"antiquely"

"antiqueness"

"antiquer"

"antiquers"

"antiques"

"antiquing"

"antiquities"

"antiquity"

"antiradical"

"antiradicals"

"antirational"

"antireligious"

"antirevolutionaries"

"antirevolutionary"

"antirust"

"antis"

"antisepsis"

"antiseptic"

"antiseptically"

"antisepticize"

"antisepticized"

"antisepticizing"

"antiseptics"

"antiserum"

"antiserums"

"antiskid"

"antislavery"

"antismog"

"antisocial"

"antisocially"

"antispasmodic"

"antispasmodics"

"antisubmarine"

"antitank"

"antitheses"

"antithesis"

"antithetic"

"antithetical"

"antithetically"

"antitoxin"

"antitoxins"

"antitrust"

"antiunion"

"antivenin"

"antivenins"

"antivivisectionist"

"antivivisectionists"

"antiwar"

"antler"

"antlered"

"antlers"

"antlike"

"antlion"

"antlions"

"antoinette"

"antonio"

"antony"

"antonym"

"antonymies"

"antonymous"

"antonyms"

"antonymy"

"antra"

"antral"

"antre"

"antrum"

"ants"

"antwerp"

"anuptaphobia"

"anus"

"anuses"

"anvil"

"anviled"

"anviling"

"anvilled"

"anvilling"

"anvils"

"anviltop"

"anviltops"

"anxieties"

"anxiety"

"anxious"

"anxiously"

"anxiousness"

"any"

"anybodies"

"anybody"

"anyhow"

"anymore"

"anyone"

"anyplace"

"anything"

"anythings"

"anytime"

"anyway"

"anyways"

"anywhere"

"anywheres"

"anywise"

"aorta"

"aortae"

"aortal"

"aortas"

"aortic"

"aouad"

"aouads"

"aoudad"

"aoudads"

"apace"

"apache"

"apaches"

"apanage"

"apanese"

"apart"

"apartheid"

"apartment"

"apartmental"

"apartments"

"apatetic"

"apathetic"

"apathetically"

"apathies"

"apathy"

"apatite"

"apatites"

"ape"

"apeak"

"aped"

"apeek"

"apelike"

"apennines"

"aper"

"apercu"

"apercus"

"aperient"

"aperies"

"aperiodic"

"aperitif"

"aperitifs"

"apers"

"apertural"

"aperture"

"apertures"

"apery"

"apes"

"apex"

"apexes"

"aphagia"

"aphanite"

"aphanites"

"aphasia"

"aphasiac"

"aphasiacs"

"aphasias"

"aphasic"

"aphasics"

"aphelia"

"aphelian"

"aphelion"

"aphid"

"aphids"

"aphis"

"aphnology"

"aphorise"

"aphorism"

"aphorisms"

"aphorist"

"aphoristic"

"aphoristically"

"aphorists"

"aphorize"

"aphorized"

"aphorizes"

"aphorizing"

"aphotic"

"aphrodisia"

"aphrodisiac"

"aphrodisiacal"

"aphrodisiacs"

"aphrodisiomania"

"aphrodite"

"apian"

"apiarian"

"apiaries"

"apiarist"

"apiarists"

"apiary"

"apical"

"apically"

"apices"

"apiece"

"aping"

"apiologies"

"apiology"

"apish"

"apishly"

"apium"

"aplenty"

"aplomb"

"aplombs"

"apnea"

"apneal"

"apneas"

"apneic"

"apnoea"

"apocalypse"

"apocalypses"

"apocalyptic"

"apocalyptical"

"apocalyptically"

"apocrypha"

"apocryphal"

"apocryphally"

"apocryphalness"

"apocynthion"

"apocynthions"

"apodal"

"apogeal"

"apogean"

"apogee"

"apogees"

"apogeic"

"apolitical"

"apolitically"

"apollo"

"apollos"

"apologal"

"apologetic"

"apologetically"

"apologia"

"apologias"

"apologies"

"apologist"

"apologists"

"apologize"

"apologized"

"apologizer"

"apologizers"

"apologizes"

"apologizing"

"apologue"

"apologues"

"apology"

"apolune"

"apolunes"

"apophthegm"

"apoplectic"

"apoplectically"

"apoplexies"

"apoplexy"

"aport"

"apostacies"

"apostacy"

"apostasies"

"apostasis"

"apostasy"

"apostate"

"apostates"

"apostatize"

"apostatized"

"apostatizes"

"apostatizing"

"apostle"

"apostles"

"apostleship"

"apostleships"

"apostolic"

"apostrophe"

"apostrophes"

"apostrophic"

"apostrophize"

"apostrophized"

"apostrophizes"

"apostrophizing"

"apothecaries"

"apothecary"

"apothegm"

"apothegms"

"apothem"

"apothems"

"apotheoses"

"apotheosis"

"app"

"App for monitoring fluids for kidney stones"

"app for symptoms"

"appal"

"appalachia"

"appalachian"

"appalachians"

"appall"

"appalled"

"appalling"

"appallingly"

"appalls"

"appaloosa"

"appaloosas"

"appals"

"appanage"

"appanages"

"apparat"

"apparats"

"apparatus"

"apparatuses"

"apparel"

"appareled"

"appareling"

"apparelled"

"apparelling"

"apparels"

"apparent"

"apparently"

"apparition"

"apparitions"

"appeal"

"appealability"

"appealable"

"appealed"

"appealer"

"appealers"

"appealing"

"appealingly"

"appeals"

"appear"

"appearance"

"appearances"

"appeared"

"appearers"

"appearing"

"appears"

"appease"

"appeased"

"appeasement"

"appeasements"

"appeaser"

"appeasers"

"appeases"

"appeasing"

"appellant"

"appellants"

"appellate"

"appellation"

"appellations"

"appellee"

"appellees"

"appellor"

"appellors"

"appels"

"append"

"appendage"

"appendages"

"appendant"

"appendectomies"

"appendectomy"

"appended"

"appendices"

"appendicitis"

"appending"

"appendix"

"appendixes"

"appends"

"apperceived"

"apperceiving"

"apperception"

"apperceptive"

"appertain"

"appertained"

"appertaining"

"appertains"

"appestat"

"appestats"

"appetencies"

"appetency"

"appetit"

"appetite"

"appetites"

"appetizer"

"appetizers"

"appetizing"

"appetizingly"

"applaud"

"applaudable"

"applaudably"

"applauded"

"applauder"

"applauders"

"applauding"

"applauds"

"applause"

"applauses"

"apple"

"applejack"

"apples"

"applesauce"

"appliance"

"appliances"

"applicabilities"

"applicability"

"applicable"

"applicably"

"applicant"

"applicants"

"application"

"applications"

"applicative"

"applicatively"

"applicator"

"applicators"

"applied"

"applier"

"appliers"

"applies"

"applique"

"appliqued"

"appliqueing"

"appliques"

"apply"

"applying"

"appoint"

"appointed"

"appointee"

"appointees"

"appointer"

"appointers"

"appointing"

"appointive"

"appointively"

"appointment"

"Appointment app"

"appointments"

"appoints"

"appomattox"

"apportion"

"apportioned"

"apportioning"

"apportionment"

"apportionments"

"apportions"

"apposable"

"appose"

"apposed"

"apposes"

"apposing"

"apposite"

"appositely"

"appositeness"

"apposition"

"appositions"

"appositive"

"appositively"

"appraisal"

"appraisals"

"appraise"

"appraised"

"appraisement"

"appraiser"

"appraisers"

"appraises"

"appraising"

"appraisingly"

"appreciable"

"appreciably"

"appreciate"

"appreciated"

"appreciates"

"appreciating"

"appreciation"

"appreciations"

"appreciative"

"appreciatively"

"appreciativeness"

"appreciator"

"appreciators"

"appreciatory"

"apprehend"

"apprehended"

"apprehending"

"apprehends"

"apprehensible"

"apprehensibly"

"apprehension"

"apprehensions"

"apprehensive"

"apprehensively"

"apprehensiveness"

"apprentice"

"apprenticed"

"apprentices"

"apprenticeship"

"apprenticeships"

"apprenticing"

"apprise"

"apprised"

"appriser"

"apprisers"

"apprises"

"apprising"

"apprize"

"apprized"

"apprizer"

"apprizes"

"approach"

"approachability"

"approachable"

"approached"

"approacher"

"approachers"

"approaches"

"approaching"

"approbate"

"approbated"

"approbating"

"approbation"

"approbations"

"approbative"

"appropriable"

"appropriate"

"appropriated"

"appropriately"

"appropriateness"

"Appropriateness Tool"

"appropriates"

"appropriating"

"appropriation"

"appropriations"

"appropriative"

"appropriator"

"appropriators"

"approval"

"approvals"

"approve"

"approved"

"approvement"

"approver"

"approvers"

"approves"

"approving"

"approvingly"

"approx"

"approximate"

"approximated"

"approximately"

"approximates"

"approximating"

"approximation"

"approximations"

"appurtenance"

"appurtenances"

"appurtenant"

"apres"

"apricot"

"apricots"

"april"

"apron"

"aproning"

"apronlike"

"aprons"

"apropos"

"apse"

"apses"

"apt"

"apter"

"apteryx"

"apteryxes"

"aptest"

"aptitude"

"aptitudes"

"aptly"

"aptness"

"aptnesses"

"aqua"

"aquacade"

"aquacades"

"aquaculture"

"aquae"

"aqualung"

"aquamarine"

"aquamarines"

"aquanaut"

"aquanauts"

"aquaphobia"

"aquaplane"

"aquaplaned"

"aquaplanes"

"aquaplaning"

"aquaria"

"aquarial"

"aquarian"

"aquarians"

"aquarist"

"aquarists"

"aquarium"

"aquariums"

"aquarius"

"aquas"

"aquatic"

"aquatics"

"aquatint"

"aquatinted"

"aquatints"

"aquatone"

"aquatones"

"aquavit"

"aquavits"

"aqueduct"

"aqueducts"

"aqueous"

"aqueously"

"aquiculture"

"aquifer"

"aquifers"

"aquiline"

"aquinas"

"aquiver"

"arab"

"arabesk"

"arabesks"

"arabesque"

"arabesques"

"arabia"

"arabian"

"arabians"

"arabic"

"arabize"

"arabizing"

"arable"

"arables"

"arabs"

"arachibutyrophobia"

"arachnid"

"arachnids"

"arachnoid"

"arachnology"

"arachnophobia"

"araks"

"aramaic"

"arapaho"

"arapahos"

"arbalest"

"arbalests"

"arbalist"

"arbiter"

"arbiters"

"arbitrable"

"arbitrage"

"arbitrager"

"arbitragers"

"arbitrages"

"arbitral"

"arbitrament"

"arbitraments"

"arbitrarily"

"arbitrariness"

"arbitrary"

"arbitrate"

"arbitrated"

"arbitrates"

"arbitrating"

"arbitration"

"arbitrational"

"arbitrations"

"arbitrative"

"arbitrator"

"arbitrators"

"arbor"

"arboreal"

"arbored"

"arbores"

"arborescent"

"arboreta"

"arboretum"

"arboretums"

"arborist"

"arborists"

"arborization"

"arborize"

"arborized"

"arborizes"

"arborizing"

"arborous"

"arbors"

"arborvitae"

"arborvitaes"

"arbour"

"arboured"

"arbours"

"arbutus"

"arbutuses"

"arc"

"arcade"

"arcaded"

"arcades"

"arcadia"

"arcadian"

"arcadians"

"arcadias"

"arcadings"

"arcana"

"arcane"

"arcanum"

"arced"

"arch"

"archaeologic"

"archaeological"

"archaeologically"

"archaeologist"

"archaeologists"

"archaeology"

"archaic"

"archaically"

"archaism"

"archaisms"

"archaist"

"archaistic"

"archaists"

"archaize"

"archaized"

"archaizes"

"archaizing"

"archangel"

"archangelic"

"archangels"

"archbishop"

"archbishopric"

"archbishoprics"

"archbishops"

"archdeacon"

"archdeacons"

"archdiocesan"

"archdiocese"

"archdioceses"

"archduchess"

"archduchesses"

"archduke"

"archdukes"

"arched"

"archelogy"

"archenemies"

"archenemy"

"archeological"

"archeology"

"archeozoic"

"archer"

"archeries"

"archers"

"archery"

"arches"

"archest"

"archetypal"

"archetype"

"archetypes"

"archetypic"

"archetypical"

"archfiend"

"archfiends"

"archiepiscopal"

"archimandrite"

"archimandrites"

"archimedean"

"archimedes"

"arching"

"archings"

"archipelago"

"archipelagoes"

"archipelagos"

"architect"

"architectonic"

"architectonics"

"architects"

"architectural"

"architecturally"

"architecture"

"architectures"

"architecure"

"architrave"

"architraves"

"archival"

"archive"

"archived"

"archives"

"archiving"

"archivist"

"archivists"

"archly"

"archness"

"archology"

"archon"

"archons"

"archonship"

"archonships"

"archway"

"archways"

"arcing"

"arcked"

"arcking"

"arco"

"arcs"

"arctic"

"arctics"

"arctophily"

"arcuate"

"arcus"

"ard"

"ardencies"

"ardency"

"ardent"

"ardently"

"ardor"

"ardors"

"ardour"

"ardours"

"arduous"

"arduously"

"arduousness"

"are"

"area"

"areal"

"areas"

"areaway"

"areaways"

"arena"

"arenas"

"areola"

"areolae"

"areolar"

"areolas"

"areolate"

"areole"

"areoles"

"areology"

"areometer"

"ares"

"aretaics"

"arete"

"aretes"

"arf"

"argal"

"argals"

"argent"

"argental"

"argentic"

"argentina"

"argentine"

"argentinean"

"argentineans"

"argentines"

"argentite"

"argents"

"argentum"

"argillaceous"

"argils"

"arginine"

"argle"

"argled"

"argles"

"argols"

"argon"

"argonaut"

"argonauts"

"argons"

"argosies"

"argosy"

"argot"

"argots"

"arguable"

"arguably"

"argue"

"argued"

"arguer"

"arguers"

"argues"

"argufied"

"argufiers"

"argufy"

"argufying"

"arguing"

"argument"

"argumentation"

"argumentative"

"argumentatively"

"argumentive"

"arguments"

"argus"

"arguses"

"argyle"

"argyles"

"argyll"

"argylls"

"arhat"

"arhats"

"arhythmias"

"aria"

"arias"

"arid"

"arider"

"aridest"

"aridities"

"aridity"

"aridly"

"aridness"

"ariel"

"aries"

"aright"

"arils"

"ariose"

"arioso"

"ariosos"

"arise"

"arisen"

"arises"

"arising"

"arisings"

"aristocracies"

"aristocracy"

"aristocrat"

"aristocratic"

"aristocratically"

"aristocrats"

"aristology"

"aristotelian"

"aristotle"

"arith"

"arithmetic"

"arithmetical"

"arithmetically"

"arithmetician"

"arithmeticians"

"arithmetics"

"arithmomania"

"arizona"

"arizonan"

"arizonans"

"arizonian"

"arizonians"

"ark"

"arkansan"

"arkansans"

"arkansas"

"arks"

"arlington"

"arm"

"armada"

"armadas"

"armadillo"

"armadillos"

"armageddon"

"armament"

"armaments"

"armature"

"armatured"

"armatures"

"armband"

"armbands"

"armchair"

"armchairs"

"armed"

"armenia"

"armenian"

"armenians"

"armer"

"armers"

"armful"

"armfuls"

"armhole"

"armholes"

"armies"

"armiger"

"armigers"

"arming"

"armings"

"armistice"

"armistices"

"armless"

"armlessly"

"armlessness"

"armlet"

"armlets"

"armload"

"armloads"

"armoire"

"armoires"

"armonica"

"armor"

"armored"

"armorer"

"armorers"

"armorial"

"armories"

"armoring"

"armors"

"armory"

"armour"

"armoured"

"armourer"

"armourers"

"armouries"

"armouring"

"armours"

"armoury"

"armpit"

"armpits"

"armrest"

"armrests"

"arms"

"armsful"

"army"

"armyworm"

"armyworms"

"arnica"

"arnicas"

"arnold"

"aroids"

"aroint"

"arointed"

"arointing"

"aroints"

"aroma"

"aromas"

"aromatic"

"aromatically"

"aromatics"

"aromatize"

"arose"

"around"

"arousal"

"arousals"

"arouse"

"aroused"

"arouser"

"arousers"

"arouses"

"arousing"

"aroynt"

"aroynts"

"arpeggio"

"arpeggios"

"arpent"

"arquebus"

"arquebuses"

"arrack"

"arracks"

"arraign"

"arraigned"

"arraigner"

"arraigning"

"arraignment"

"arraignments"

"arraigns"

"arrange"

"arranged"

"arrangement"

"arrangements"

"arranger"

"arrangers"

"arranges"

"arranging"

"arrant"

"arrantly"

"arras"

"arrases"

"array"

"arrayal"

"arrayals"

"arrayed"

"arrayer"

"arrayers"

"arraying"

"arrays"

"arrear"

"arrears"

"arrest"

"arrested"

"arrestee"

"arrestees"

"arrester"

"arresters"

"arresting"

"arrestment"

"arrestor"

"arrestors"

"arrests"

"arrhythmia"

"arrhythmias"

"arrhythmical"

"arrival"

"arrivals"

"arrive"

"arrived"

"arrivederci"

"arriver"

"arrivers"

"arrives"

"arriving"

"arrogance"

"arrogant"

"arrogantly"

"arrogate"

"arrogated"

"arrogates"

"arrogating"

"arrogation"

"arrogations"

"arrow"

"arrowed"

"arrowhead"

"arrowheads"

"arrowing"

"arrowroot"

"arrowroots"

"arrows"

"arrowy"

"arroyo"

"arroyos"

"ars"

"arse"

"arsenal"

"arsenals"

"arsenate"

"arsenates"

"arsenic"

"arsenical"

"arsenics"

"arsenides"

"arsenious"

"arsenites"

"arsenous"

"arses"

"arsis"

"arson"

"arsonic"

"arsonist"

"arsonists"

"arsonous"

"arsons"

"art"

"artefact"

"artemis"

"arterial"

"arterial blood gas"

"arterials"

"arteries"

"arteriocapillary"

"arteriogram"

"arteriography"

"arteriolar"

"arteriole"

"arterioles"

"arterioscleroses"

"arteriosclerosis"

"arteriosclerotic"

"artery"

"artful"

"artfully"

"artfulness"

"arthral"

"arthritic"

"arthritics"

"arthritis"

"arthritis pain"

"arthrography"

"arthrology"

"arthropod"

"arthropods"

"arthroscope"

"arthur"

"arthurian"

"artichoke"

"artichokes"

"article"

"articled"

"articles"

"articular"

"articulate"

"articulated"

"articulately"

"articulateness"

"articulates"

"articulating"

"articulation"

"articulationes"

"articulations"

"articulator"

"articulatory"

"artier"

"artiest"

"artifact"

"artifacts"

"artifice"

"artificer"

"artificers"

"artifices"

"artificial"

"artificiality"

"artificially"

"artificialness"

"artillerist"

"artillerists"

"artillery"

"artilleryman"

"artillerymen"

"artily"

"artiness"

"artisan"

"artisans"

"artisanship"

"artist"

"artiste"

"artistes"

"artistic"

"artistic temperament"

"artistically"

"artistries"

"artistry"

"artists"

"artless"

"artlessly"

"artlessness"

"arts"

"artwork"

"artworks"

"arty"

"arum"

"arums"

"aryan"

"aryans"

"aryls"

"arythmia"

"arythmic"

"as"

"asafetida"

"asap"

"asbestic"

"asbestos"

"asbestosis"

"ascend"

"ascendable"

"ascendance"

"ascendancy"

"ascendant"

"ascended"

"ascendence"

"ascendent"

"ascender"

"ascenders"

"ascending"

"ascends"

"ascension"

"ascensions"

"ascent"

"ascents"

"ascertain"

"ascertainable"

"ascertained"

"ascertaining"

"ascertainment"

"ascertains"

"ascetic"

"ascetically"

"asceticism"

"ascetics"

"ascii"

"ascorbate"

"ascorbic"

"ascot"

"ascots"

"ascribable"

"ascribe"

"ascribed"

"ascribes"

"ascribing"

"ascription"

"ascriptions"

"asea"

"asepses"

"asepsis"

"aseptic"

"aseptically"

"asexual"

"asexuality"

"asexually"

"asexuals"

"ash"

"ashamed"

"ashamedly"

"ashcan"

"ashcans"

"ashed"

"ashen"

"ashes"

"ashier"

"ashiest"

"ashiness"

"ashing"

"ashlar"

"ashlars"

"ashlers"

"ashless"

"ashman"

"ashmen"

"ashore"

"ashram"

"ashrams"

"ashtray"

"ashtrays"

"ashy"

"asia"

"asian"

"asians"

"asiatic"

"aside"

"asides"

"asinine"

"asininely"

"asininity"

"ask"

"askance"

"askant"

"asked"

"asker"

"askers"

"askew"

"asking"

"askings"

"asks"

"aslant"

"asleep"

"aslope"

"asocial"

"asp"

"asparagus"

"asparaguses"

"aspca"

"aspect"

"aspects"

"aspen"

"aspens"

"asper"

"asperges"

"asperities"

"asperity"

"aspers"

"asperse"

"aspersed"

"aspersers"

"asperses"

"aspersing"

"aspersion"

"aspersions"

"aspersors"

"asphalt"

"asphalted"

"asphaltic"

"asphalting"

"asphalts"

"asphaltum"

"aspheric"

"asphodel"

"asphodels"

"asphyxia"

"asphyxiant"

"asphyxiate"

"asphyxiated"

"asphyxiates"

"asphyxiating"

"asphyxiation"

"asphyxiator"

"asphyxy"

"aspic"

"aspics"

"aspidistra"

"aspidistras"

"aspirant"

"aspirants"

"aspirate"

"aspirated"

"aspirates"

"aspirating"

"aspiration"

"aspirations"

"aspirator"

"aspirators"

"aspire"

"aspired"

"aspirer"

"aspirers"

"aspires"

"aspirin"

"aspiring"

"aspiringly"

"aspirins"

"aspish"

"asps"

"asquint"

"asramas"

"ass"

"assafoetida"

"assagai"

"assagais"

"assail"

"assailable"

"assailant"

"assailants"

"assailed"

"assailer"

"assailers"

"assailing"

"assailment"

"assails"

"assam"

"assassin"

"assassinate"

"assassinated"

"assassinates"

"assassinating"

"assassination"

"assassinations"

"assassinator"

"assassins"

"assault"

"assaultable"

"assaulted"

"assaulter"

"assaulters"

"assaulting"

"assaultive"

"assaults"

"assay"

"assayed"

"assayer"

"assayers"

"assaying"

"assays"

"assegai"

"assegais"

"assemblage"

"assemblages"

"assemble"

"assembled"

"assembler"

"assemblers"

"assembles"

"assemblies"

"assembling"

"assembly"

"assemblyman"

"assemblymen"

"assemblywoman"

"assemblywomen"

"assent"

"assented"

"assenter"

"assenters"

"assenting"

"assentor"

"assentors"

"assents"

"assert"

"asserted"

"asserter"

"asserters"

"asserting"

"assertion"

"assertions"

"assertive"

"assertively"

"assertiveness"

"assertor"

"assertors"

"asserts"

"asses"

"assess"

"assessable"

"assessed"

"assessee"

"assesses"

"assessing"

"assessment"

"assessments"

"assessor"

"assessors"

"assessorship"

"asset"

"assets"

"asseverate"

"asseverated"

"asseverates"

"asseverating"

"asseveration"

"asseverations"

"asshole"

"assiduity"

"assiduous"

"assiduously"

"assiduousness"

"assign"

"assignability"

"assignable"

"assignat"

"assignation"

"assignations"

"assigned"

"assignee"

"assignees"

"assigner"

"assigners"

"assigning"

"assignment"

"assignments"

"assignor"

"assignors"

"assigns"

"assimilable"

"assimilate"

"assimilated"

"assimilates"

"assimilating"

"assimilation"

"assimilative"

"assimilator"

"assisi"

"assist"

"assistance"

"assistant"

"assistants"

"assisted"

"assister"

"assisters"

"assisting"

"assistor"

"assistors"

"assists"

"assize"

"assizer"

"assizes"

"asslike"

"assn"

"assoc"

"associate"

"associated"

"associates"

"associating"

"association"

"associations"

"associative"

"associatively"

"associativity"

"associator"

"associators"

"assonance"

"assonances"

"assonant"

"assonantly"

"assonants"

"assort"

"assorted"

"assorter"

"assorters"

"assorting"

"assortment"

"assortments"

"assorts"

"asst"

"assuagable"

"assuage"

"assuaged"

"assuagement"

"assuagements"

"assuages"

"assuaging"

"assuasive"

"assumable"

"assumably"

"assume"

"assumed"

"assumedly"

"assumer"

"assumers"

"assumes"

"assuming"

"assumption"

"assumptions"

"assumptive"

"assumptively"

"assumptiveness"

"assurance"

"assurances"

"assure"

"assured"

"assuredly"

"assureds"

"assurer"

"assurers"

"assures"

"assuring"

"assuror"

"assurors"

"assyria"

"assyrian"

"assyrians"

"astacology"

"astatine"

"astatines"

"aster"

"asterisk"

"asterisked"

"asterisks"

"asterism"

"asterisms"

"astern"

"asteroid"

"asteroidal"

"asteroids"

"asters"

"astheniology"

"asthma"

"asthmas"

"asthmatic"

"asthmatically"

"asthmatics"

"astigmatic"

"astigmatism"

"astir"

"astonish"

"astonished"

"astonishes"

"astonishing"

"astonishingly"

"astonishment"

"astonishments"

"astound"

"astounded"

"astounding"

"astoundingly"

"astounds"

"astraddle"

"astragal"

"astragals"

"astrakhan"

"astral"

"astrally"

"astrals"

"astraphobia"

"astrapophobia"

"astray"

"astride"

"astringe"

"astringed"

"astringency"

"astringent"

"astringents"

"astringes"

"astringing"

"astrobiological"

"astrobiologically"

"astrobiologies"

"astrobiologist"

"astrobiologists"

"astrobiology"

"astrodome"

"astrodynamic"

"astrodynamics"

"astrogeology"

"astroid"

"astrolabe"

"astrolabes"

"astrologer"

"astrologers"

"astrologic"

"astrological"

"astrologically"

"astrologist"

"astrologists"

"astrology"

"astrometeorology"

"astronaut"

"astronautic"

"astronautical"

"astronautically"

"astronautics"

"astronauts"

"astronomer"

"astronomers"

"astronomic"

"astronomical"

"astronomically"

"astronomy"

"astrophysical"

"astrophysicist"

"astrophysicists"

"astrophysics"

"astroseismology"

"astute"

"astutely"

"astuteness"

"asunder"

"aswarm"

"aswirl"

"aswoon"

"asyla"

"asylum"

"asylums"

"asymmetric"

"asymmetrical"

"asymmetrically"

"asymmetries"

"asymmetry"

"asymptomatic"

"asymptote"

"asymptotes"

"asymptotic"

"asymptotical"

"asymptotically"

"async"

"asynchronous"

"asyndeta"

"asystematic"

"at"

"atavic"

"atavism"

"atavisms"

"atavist"

"atavistic"

"atavists"

"ataxia"

"ataxic"

"ataxy"

"ate"

"atelier"

"ateliers"

"atheism"

"atheisms"

"atheist"

"atheistic"

"atheistical"

"atheistically"

"atheists"

"atheling"

"athelings"

"athena"

"athenaeum"

"athenaeums"

"atheneum"

"atheneums"

"athenian"

"athenians"

"athens"

"atheroscleroses"

"atherosclerosis"

"atherosclerotic"

"athirst"

"athlete"

"athletes"

"athletic"

"athletically"

"athletics"

"atholic"

"athwart"

"atilt"

"atin"

"atingle"

"atlanta"

"atlantic"

"atlas"

"atlases"

"atma"

"atman"

"atmans"

"atmas"

"atmology"

"atmometer"

"atmosphere"

"atmospheres"

"atmospheric"

"atmospherical"

"atmospherically"

"atmospherics"

"atoll"

"atolls"

"atom"

"atomic"

"atomical"

"atomically"

"atomics"

"atomies"

"atomise"

"atomised"

"atomises"

"atomising"

"atomism"

"atomisms"

"atomist"

"atomistic"

"atomists"

"atomization"

"atomize"

"atomized"

"atomizer"

"atomizers"

"atomizes"

"atomizing"

"atoms"

"atomy"

"atonable"

"atonal"

"atonality"

"atonally"

"atone"

"atoneable"

"atoned"

"atonement"

"atonements"

"atoner"

"atoners"

"atones"

"atonics"

"atonies"

"atoning"

"atoningly"

"atop"

"atopic"

"atremble"

"atria"

"atrial"

"atrial enlargement"

"atrip"

"atrium"

"atriums"

"atrocious"

"atrociously"

"atrociousness"

"atrocities"

"atrocity"

"atrophic"

"atrophied"

"atrophies"

"atrophy"

"atrophying"

"atropine"

"atropins"

"atropism"

"attach"

"attachable"

"attache"

"attached"

"attacher"

"attachers"

"attaches"

"attaching"

"attachment"

"attachments"

"attack"

"attacked"

"attacker"

"attackers"

"attacking"

"attackingly"

"attacks"

"attain"

"attainability"

"attainable"

"attainableness"

"attainably"

"attainder"

"attainders"

"attained"

"attainer"

"attainers"

"attaining"

"attainment"

"attainments"

"attains"

"attaint"

"attainted"

"attainting"

"attaints"

"attar"

"attars"

"attemper"

"attempered"

"attempt"

"attemptable"

"attempted"

"attempter"

"attempters"

"attempting"

"attempts"

"attend"

"attendance"

"attendances"

"attendant"

"attendantly"

"attendants"

"attended"

"attendee"

"attendees"

"attender"

"attenders"

"attending"

"attends"

"attention"

"Attention Deficit Disorder"

"Attention deficit hyperactivity disorder"

"Attention Increase"

"attentions"

"attentive"

"attentively"

"attentiveness"

"attenuate"

"attenuated"

"attenuates"

"attenuating"

"attenuation"

"attenuations"

"attermined"

"attest"

"attestable"

"attestant"

"attestation"

"attestations"

"attestator"

"attested"

"attester"

"attesters"

"attesting"

"attestor"

"attestors"

"attests"

"attic"

"attics"

"attila"

"attire"

"attired"

"attires"

"attiring"

"attitude"

"attitudes"

"attitudinal"

"attitudinize"

"attitudinized"

"attitudinizes"

"attitudinizing"

"attn"

"attorney"

"attorneys"

"attorning"

"attract"

"attractable"

"attractant"

"attractants"

"attracted"

"attracting"

"attraction"

"attractions"

"attractive"

"attractively"

"attractiveness"

"attracts"

"attrib"

"attributable"

"attribute"

"attributed"

"attributes"

"attributing"

"attribution"

"attributions"

"attributive"

"attributively"

"attributives"

"attrition"

"attritional"

"attune"

"attuned"

"attunes"

"attuning"

"atty"

"atwain"

"atween"

"atwitter"

"atypic"

"atypical"

"atypically"

"aubade"

"aubades"

"auberge"

"auberges"

"auburn"

"auburns"

"auction"

"auctioned"

"auctioneer"

"auctioneers"

"auctioning"

"auctions"

"auctorial"

"auctors"

"aud"

"audacious"

"audaciously"

"audaciousness"

"audacities"

"audacity"

"audad"

"audads"

"audibility"

"audible"

"audibles"

"audibly"

"audience"

"audiences"

"audient"

"audio"

"audio feedback"

"audio signal"

"audiogram"

"audiological"

"audiologies"

"audiologist"

"audiologists"

"audiology"

"audiometer"

"audiometers"

"audiometric"

"audiometries"

"audiometrist"

"audiometry"

"audiophile"

"audiophiles"

"audios"

"audiotape"

"audiotapes"

"audiovisual"

"audiovisuals"

"audit"

"audited"

"auditing"

"audition"

"auditioned"

"auditioning"

"auditions"

"auditive"

"auditives"

"auditor"

"auditoria"

"auditorial"

"auditories"

"auditorium"

"auditoriums"

"auditors"

"auditory"

"audits"

"auf"

"augend"

"augends"

"auger"

"augers"

"aught"

"aughts"

"augment"

"augmentation"

"augmentations"

"augmented"

"augmenter"

"augmenters"

"augmenting"

"augments"

"augur"

"augural"

"augured"

"augurer"

"augurers"

"auguries"

"auguring"

"augurs"

"augury"

"august"

"augusta"

"auguster"

"augustest"

"augustine"

"augustinian"

"augustly"

"augustness"

"auk"

"auklets"

"auks"

"auld"

"aulder"

"auldest"

"aunt"

"aunthood"

"aunthoods"

"auntie"

"aunties"

"auntliest"

"aunts"

"aunty"

"aura"

"aurae"

"aural"

"aurally"

"auras"

"aurate"

"aurated"

"aureate"

"aureately"

"aureateness"

"aureola"

"aureolae"

"aureolas"

"aureole"

"aureoled"

"aureoles"

"aureomycin"

"aureus"

"auric"

"auricle"

"auricled"

"auricles"

"auricular"

"auricularly"

"auriferous"

"auriform"

"auriscope"

"aurist"

"aurochs"

"aurochses"

"aurora"

"aurorae"

"auroral"

"auroras"

"aurorean"

"aurous"

"aurum"

"aurums"

"ausculation"

"auscultate"

"auscultated"

"auscultates"

"auscultating"

"auscultation"

"auscultations"

"auspice"

"auspices"

"auspicious"

"auspiciously"

"auspiciousness"

"aussie"

"aussies"

"austere"

"austerely"

"austereness"

"austerest"

"austerities"

"austerity"

"austin"

"austral"

"australia"

"australian"

"australians"

"australis"

"austria"

"austrian"

"austrians"

"autarchies"

"autarchy"

"autarky"

"autecology"

"authentic"

"authentic sounds"

"authentically"

"authenticate"

"authenticated"

"authenticates"

"authenticating"

"authentication"

"authentications"

"authenticator"

"authenticators"

"authenticities"

"authenticity"

"author"

"authored"

"authoress"

"authoresses"

"authoring"

"authoritarian"

"authoritarianism"

"authoritarianisms"

"authoritarians"

"authoritative"

"authoritatively"

"authoritativeness"

"authorities"

"authority"

"authorization"

"authorizations"

"authorize"

"authorized"

"authorizer"

"authorizers"

"authorizes"

"authorizing"

"authors"

"authorship"

"autism"

"autisms"

"autistic"

"auto"

"autobahn"

"autobahnen"

"autobahns"

"autobiographer"

"autobiographers"

"autobiographic"

"autobiographical"

"autobiographically"

"autobiographies"

"autobiography"

"autobus"

"autobuses"

"autobusses"

"autocade"

"autocades"

"autochthonous"

"autoclave"

"autoclaves"

"autocracies"

"autocracy"

"autocrat"

"autocratic"

"autocratically"

"autocrats"

"autodial"

"autodialed"

"autodialer"

"autodialers"

"autodialing"

"autodialled"

"autodialling"

"autodials"

"autodidact"

"autodidactic"

"autodidacts"

"autoed"

"autoeroticism"

"autoerotism"

"autogeneses"

"autogenesis"

"autogenetic"

"autogiro"

"autogiros"

"autograph"

"autographed"

"autographic"

"autographing"

"autographs"

"autogyro"

"autogyros"

"autohypnosis"

"autoimmunities"

"autoimmunity"

"autoimmunization"

"autoimmunize"

"autoimmunized"

"autoimmunizing"

"autoinfection"

"autoing"

"autoinoculation"

"autointoxication"

"autology"

"autolyze"

"automanipulation"

"automanipulative"

"automat"

"automata"

"automate"

"automated"

"automates"

"automatic"

"automatic update"

"automatically"

"automatics"

"automating"

"automation"

"automatism"

"automatization"

"automatize"

"automatized"

"automatizes"

"automatizing"

"automaton"

"automatons"

"automats"

"automobile"

"automobiles"

"automobilist"

"automobilists"

"automotive"

"automysophobia"

"autonomic"

"autonomies"

"autonomous"

"autonomously"

"autonomy"

"autophagy"

"autophobia"

"autopilot"

"autopilots"

"autopsic"

"autopsied"

"autopsies"

"autopsy"

"autopsying"

"autoregulation"

"autoregulative"

"autoregulatory"

"autos"

"autostrada"

"autostradas"

"autosuggestion"

"autosuggestions"

"autotherapy"

"autotransplant"

"autre"

"autumn"

"autumnal"

"autumns"

"aux"

"auxanometer"

"auxiliaries"

"auxiliary"

"auxillary"

"auxin"

"auxins"

"auxology"

"auxometer"

"avail"

"availabilities"

"availability"

"available"

"availed"

"availing"

"avails"

"avalanche"

"avalanches"

"avantgarde"

"avarice"

"avarices"

"avaricious"

"avariciously"

"avascular"

"avast"

"avatar"

"avatars"

"avaunt"

"avdp"

"ave"

"avenge"

"avenged"

"avenger"

"avengers"

"avenges"

"avenging"

"avengingly"

"avenses"

"avenue"

"avenues"

"aver"

"average"

"averaged"

"averages"

"averaging"

"averment"

"averments"

"averred"

"averring"

"avers"

"averse"

"aversely"

"aversion"

"aversions"

"aversive"

"avert"

"averted"

"averting"

"averts"

"aves"

"avg"

"avian"

"avianize"

"avianized"

"avianizes"

"avians"

"aviaries"

"aviarist"

"aviarists"

"aviary"

"aviate"

"aviated"

"aviates"

"aviating"

"aviation"

"aviations"

"aviator"

"aviators"

"aviatrices"

"aviatrix"

"aviatrixes"

"avid"

"avidities"

"avidity"

"avidly"

"avidness"

"avifauna"

"avion"

"avionic"

"avionics"

"avions"

"avis"

"aviso"

"avitaminoses"

"avitaminosis"

"avitaminotic"

"avocado"

"avocadoes"

"avocados"

"avocation"

"avocational"

"avocations"

"avocet"

"avocets"

"avogadro"

"avoid"

"avoidable"

"avoidably"

"avoidance"

"avoidances"

"avoidant"

"avoided"

"avoider"

"avoiders"

"avoiding"

"avoids"

"avoirdupois"

"avouch"

"avouched"

"avoucher"

"avouchers"

"avouches"

"avouching"

"avow"

"avowable"

"avowably"

"avowal"

"avowals"

"avowed"

"avowedly"

"avower"

"avowers"

"avowing"

"avows"

"avulsions"

"avuncular"

"aw"

"awacs"

"await"

"awaited"

"awaiter"

"awaiters"

"awaiting"

"awaits"

"awake"

"awaked"

"awaken"

"awakened"

"awakener"

"awakeners"

"awakening"

"awakenings"

"awakens"

"awakes"

"awaking"

"awakings"

"award"

"award winning"

"awarded"

"awardee"

"awardees"

"awarder"

"awarders"

"awarding"

"awards"

"aware"

"awareness"

"awash"

"away"

"awayness"

"awe"

"aweary"

"aweather"

"awed"

"aweigh"

"aweing"

"aweless"

"awes"

"awesome"

"awesomely"

"awesomeness"

"awful"

"awfuller"

"awfullest"

"awfully"

"awfulness"

"awhile"

"awhirl"

"awing"

"awkward"

"awkwarder"

"awkwardest"

"awkwardly"

"awkwardness"

"awl"

"awless"

"awls"

"awn"

"awned"

"awning"

"awninged"

"awnings"

"awns"

"awoke"

"awoken"

"awol"

"awols"

"awry"

"ax"

"axe"

"axed"

"axel"

"axels"

"axeman"

"axemen"

"axes"

"axial"

"axiality"

"axially"

"axil"

"axillae"

"axillar"

"axillaries"

"axillary"

"axillas"

"axils"

"axing"

"axiology"

"axiom"

"axiomatic"

"axiomatically"

"axioms"

"axis"

"axises"

"axle"

"axled"

"axles"

"axletree"

"axletrees"

"axlike"

"axman"

"axmen"

"axolotl"

"axolotls"

"axon"

"axonal"

"axone"

"axones"

"axonic"

"axons"

"axseed"

"ay"

"ayah"

"ayahs"

"ayatollah"

"ayatollahs"

"aye"

"ayes"

"Ayurveda"

"Ayurvedic herbal"

"ayurvedic pharmacology"

"azalea"

"azaleas"

"azide"

"azido"

"azimuth"

"azimuthal"

"azimuths"

"azine"

"azoic"

"azole"

"azons"

"azores"

"azote"

"azoth"

"aztec"

"aztecan"

"aztecs"

"azure"

"azures"

"azurite"

"azurites"

"ba"

"baa"

"baaed"

"baaing"

"baal"

"baalism"

"baalisms"

"baals"

"baas"

"baba"

"babas"

"babbitting"

"babble"

"babbled"

"babbler"

"babblers"

"babbles"

"babbling"

"babblings"

"babcock"

"babe"

"babel"

"babels"

"babes"

"babied"

"babies"

"babka"

"babkas"

"baboo"

"baboon"

"baboonish"

"baboons"

"baboos"

"babu"

"babul"

"babuls"

"babus"

"babushka"

"babushkas"

"baby"

"baby drawings"

"babyÂ´s name"

"babyhood"

"babyhoods"

"babying"

"babyish"

"babylon"

"babylonia"

"babylonian"

"babylonians"

"babysitting"

"bacca"

"baccalaureate"

"baccalaureates"

"baccarat"

"baccarats"

"bacchanal"

"bacchanalia"

"bacchanalian"

"bacchanalias"

"bacchanals"

"bacchant"

"bacchantes"

"bacchants"

"bacchic"

"bacchus"

"bach"

"bachelor"

"bachelorhood"

"bachelors"

"bachelorship"

"bacillary"

"bacilli"

"bacillus"

"back"

"backache"

"backaches"

"backbencher"

"backbenchers"

"backbend"

"backbends"

"backbit"

"backbite"

"backbiter"

"backbiters"

"backbites"

"backbiting"

"backbitten"

"backboard"

"backboards"

"backbone"

"backbones"

"backbreaking"

"backcourt"

"backcross"

"backdate"

"backdated"

"backdates"

"backdating"

"backdoor"

"backdrop"

"backdrops"

"backed"

"backed up"

"backer"

"backers"

"backfield"

"backfields"

"backfill"

"backfilled"

"backfills"

"backfire"

"backfired"

"backfires"

"backfiring"

"backgammon"

"background"

"backgrounds"

"backhand"

"backhanded"

"backhanding"

"backhands"

"backhoe"

"backhoes"

"backing"

"backings"

"backlash"

"backlashed"

"backlashes"

"backless"

"backlist"

"backlists"

"backlit"

"backlog"

"backlogged"

"backlogging"

"backlogs"

"backmost"

"backpack"

"backpacked"

"backpacker"

"backpackers"

"backpacking"

"backpacks"

"backrest"

"backrests"

"backround"

"backs"

"backsaw"

"backsaws"

"backseat"

"backseats"

"backside"

"backsides"

"backslap"

"backslapper"

"backslappers"

"backslapping"

"backslaps"

"backslid"

"backslidden"

"backslide"

"backslider"

"backsliders"

"backslides"

"backsliding"

"backspace"

"backspaced"

"backspaces"

"backspacing"

"backspin"

"backspins"

"backstage"

"backstairs"

"backstay"

"backstitching"

"backstop"

"backstops"

"backstretch"

"backstretches"

"backstroke"

"backstrokes"

"backstroking"

"backswept"

"backtrack"

"backtracked"

"backtracking"

"backtracks"

"backup"

"backups"

"backward"

"backwardly"

"backwardness"

"backwards"

"backwash"

"backwashes"

"backwater"

"backwaters"

"backwood"

"backwoods"

"backwoodsman"

"backwoodsmen"

"backyard"

"backyards"

"bacon"

"bacons"

"bacteria"

"bacterial"

"bacterial infection"

"bacterially"

"bactericidal"

"bactericidally"

"bactericide"

"bactericides"

"bacteriocidal"

"bacteriologic"

"bacteriological"

"bacteriologically"

"bacteriologies"

"bacteriologist"

"bacteriologists"

"bacteriology"

"bacteriophage"

"bacteriophages"

"bacteriotoxin"

"bacterium"

"bacteroidal"

"bad"

"bad habitual behaviors"

"baddie"

"baddies"

"baddy"

"bade"

"badge"

"badged"

"badger"

"badgered"

"badgering"

"badgerly"

"badgers"

"badges"

"badging"

"badinage"

"badinaged"

"badinages"

"badinaging"

"badland"

"badlands"

"badly"

"badman"

"badmen"

"badminton"

"badmouth"

"badmouthed"

"badmouthing"

"badmouths"

"badness"

"badnesses"

"bads"

"baedeker"

"baedekers"

"baffle"

"baffled"

"bafflement"

"bafflements"

"baffler"

"bafflers"

"baffles"

"baffling"

"bag"

"bagasse"

"bagatelle"

"bagatelles"

"bagel"

"bagels"

"bagful"

"bagfuls"

"baggage"

"baggages"

"bagged"

"baggie"

"baggier"

"baggies"

"baggiest"

"baggily"

"bagginess"

"bagging"

"baggings"

"baggy"

"baghdad"

"bagman"

"bagmen"

"bagnio"

"bagnios"

"bagpipe"

"bagpiper"

"bagpipers"

"bagpipes"

"bags"

"bagsful"

"baguet"

"baguets"

"baguette"

"baguettes"

"bagwig"

"bagwigs"

"bagworm"

"bagworms"

"bah"

"bahamas"

"bahamian"

"bahamians"

"baht"

"bahts"

"bail"

"bailable"

"bailed"

"bailee"

"bailer"

"bailers"

"bailey"

"baileys"

"bailie"

"bailies"

"bailiff"

"bailiffs"

"bailing"

"bailiwick"

"bailiwicks"

"bailment"

"bailor"

"bailors"

"bailout"

"bailouts"

"bails"

"bailsman"

"bailsmen"

"bairn"

"bairns"

"bait"

"baited"

"baiter"

"baiters"

"baiting"

"baits"

"baize"

"baizes"

"bake"

"baked"

"bakemeats"

"baker"

"bakeries"

"bakers"

"bakersfield"

"bakery"

"bakes"

"bakeshop"

"bakeshops"

"baking"

"bakings"

"baklava"

"baklavas"

"baksheesh"

"baksheeshes"

"bakshish"

"balalaika"

"balalaikas"

"balance"

"balanced"

"balancer"

"balancers"

"balances"

"balancing"

"balboa"

"balboas"

"balbriggan"

"balconies"

"balcony"

"bald"

"baldachin"

"baldachins"

"balded"

"balder"

"balderdash"

"baldest"

"baldhead"

"baldheads"

"balding"

"baldish"

"baldly"

"baldness"

"baldpate"

"baldpates"

"baldric"

"baldrick"

"baldricks"

"baldrics"

"balds"

"bale"

"baled"

"baleen"

"baleens"

"balefire"

"balefires"

"baleful"

"balefully"

"balefulness"

"baler"

"balers"

"bales"

"bali"

"balinese"

"baling"

"balk"

"balkan"

"balkans"

"balked"

"balker"

"balkers"

"balkier"

"balkiest"

"balkily"

"balkiness"

"balking"

"balks"

"balky"

"ball"

"ballad"

"balladeer"

"balladeers"

"ballades"

"balladic"

"balladries"

"balladry"

"ballads"

"ballast"

"ballasted"

"ballasting"

"ballasts"

"balled"

"baller"

"ballerina"

"ballerinas"

"ballers"

"ballet"

"balletic"

"balletomane"

"balletomanes"

"balletomania"

"ballets"

"balling"

"ballista"

"ballistae"

"ballistic"

"ballistically"

"ballistician"

"ballisticians"

"ballistics"

"ballistocardiograph"

"ballistophobia"

"ballo"

"balloon"

"ballooned"

"ballooner"

"ballooners"

"ballooning"

"balloonist"

"balloonlike"

"balloons"

"ballot"

"balloted"

"balloter"

"balloters"

"balloting"

"ballots"

"ballottable"

"ballplayer"

"ballplayers"

"ballpoint"

"ballpoints"

"ballroom"

"ballrooms"

"balls"

"ballute"

"ballutes"

"bally"

"ballyhoo"

"ballyhooed"

"ballyhooing"

"ballyhoos"

"ballyrag"

"balm"

"balmier"

"balmiest"

"balmily"

"balminess"

"balmoral"

"balmorals"

"balms"

"balmy"

"balneology"

"baloney"

"baloneys"

"balsa"

"balsam"

"balsamed"

"balsamic"

"balsaming"

"balsams"

"balsas"

"baltic"

"baltimore"

"baluster"

"balustered"

"balusters"

"balustrade"

"balustrades"

"bambino"

"bambinos"

"bamboo"

"bamboos"

"bamboozle"

"bamboozled"

"bamboozler"

"bamboozlers"

"bamboozles"

"bamboozling"

"ban"

"banal"

"banalities"

"banality"

"banally"

"banana"

"bananas"

"banco"

"band"

"bandage"

"bandaged"

"bandager"

"bandagers"

"bandages"

"bandaging"

"bandana"

"bandanas"

"bandanna"

"bandannas"

"bandbox"

"bandboxes"

"bandeau"

"bandeaus"

"bandeaux"

"banded"

"bander"

"banderole"

"banderoles"

"banders"

"bandicoot"

"bandicoots"

"bandied"

"bandies"

"banding"

"bandit"

"banditries"

"banditry"

"bandits"

"banditti"

"bandmaster"

"bandmasters"

"bandoleer"

"bandoleers"

"bands"

"bandsman"

"bandsmen"

"bandstand"

"bandstands"

"bandwagon"

"bandwagons"

"bandwidth"

"bandwidths"

"bandy"

"bandying"

"bane"

"baned"

"baneful"

"banes"

"bang"

"banged"

"banger"

"bangers"

"banging"

"bangkok"

"bangkoks"

"bangle"

"bangles"

"bangs"

"bangtail"

"bangtails"

"banish"

"banished"

"banisher"

"banishers"

"banishes"

"banishing"

"banishment"

"banishments"

"banister"

"banisters"

"banjo"

"banjoes"

"banjoist"

"banjoists"

"banjos"

"bank"

"bankable"

"bankbook"

"bankbooks"

"banked"

"banker"

"bankers"

"banking"

"bankings"

"banknote"

"banknotes"

"bankroll"

"bankrolled"

"bankrolling"

"bankrolls"

"bankrupt"

"bankruptcies"

"bankruptcy"

"bankrupted"

"bankrupting"

"bankrupts"

"banks"

"bankside"

"banksides"

"banned"

"banner"

"banners"

"banning"

"bannister"

"bannock"

"bannocks"

"banns"

"banquet"

"banqueted"

"banqueter"

"banqueters"

"banqueting"

"banquets"

"banquette"

"banquettes"

"bans"

"banshee"

"banshees"

"banshie"

"banshies"

"bantam"

"bantams"

"bantamweight"

"bantamweights"

"banter"

"bantered"

"banterer"

"banterers"

"bantering"

"banteringly"

"banters"

"banting"

"bantling"

"bantu"

"bantus"

"banyan"

"banyans"

"banzai"

"banzais"

"baobab"

"baobabs"

"baptise"

"baptised"

"baptises"

"baptism"

"baptismal"

"baptismally"

"baptisms"

"baptist"

"baptisteries"

"baptistery"

"baptists"

"baptize"

"baptized"

"baptizer"

"baptizers"

"baptizes"

"baptizing"

"bar"

"barb"

"barbados"

"barbara"

"barbarian"

"barbarianism"

"barbarians"

"barbaric"

"barbarically"

"barbarious"

"barbarism"

"barbarisms"

"barbarities"

"barbarity"

"barbarization"

"barbarize"

"barbarized"

"barbarizes"

"barbarizing"

"barbarous"

"barbarously"

"barbarousness"

"barbecue"

"barbecued"

"barbecues"

"barbecuing"

"barbed"

"barbel"

"barbell"

"barbells"

"barbels"

"barber"

"barbered"

"barbering"

"barberries"

"barberry"

"barbers"

"barbershop"

"barbershops"

"barbets"

"barbican"

"barbicans"

"barbing"

"barbital"

"barbiturate"

"barbiturates"

"barbituric"

"barbless"

"barbs"

"barbwire"

"barbwires"

"barcarole"

"barcaroles"

"barcelona"

"Barcode"

"Barcode scanner"

"bard"

"barded"

"bardes"

"bardic"

"barding"

"bards"

"bare"

"bareback"

"bared"

"barefaced"

"barefit"

"barefoot"

"barehanded"

"barehead"

"bareheaded"

"barelegged"

"barely"

"bareness"

"barer"

"bares"

"barest"

"barf"

"barfed"

"barfing"

"barflies"

"barfly"

"barfs"

"bargain"

"bargainable"

"bargained"

"bargainee"

"bargainer"

"bargainers"

"bargaining"

"bargains"

"barge"

"barged"

"bargee"

"bargees"

"bargeman"

"bargemen"

"barges"

"barging"

"barhop"

"barhopped"

"barhopping"

"barhops"

"bariatrician"

"baric"

"baring"

"barite"

"baritone"

"baritones"

"barium"

"bariums"

"bark"

"barked"

"barkeep"

"barkeeper"

"barkeepers"

"barkeeps"

"barkentine"

"barkentines"

"barker"

"barkers"

"barkier"

"barking"

"barkless"

"barks"

"barky"

"barless"

"barley"

"barleycorn"

"barleys"

"barlow"

"barlows"

"barmaid"

"barmaids"

"barman"

"barmen"

"barmie"

"barmier"

"barmiest"

"barmy"

"barn"

"barnacle"

"barnacled"

"barnacles"

"barnier"

"barns"

"barnstorm"

"barnstormed"

"barnstormer"

"barnstormers"

"barnstorming"

"barnstorms"

"barny"

"barnyard"

"barnyards"

"barodynamics"

"barogram"

"barograms"

"barograph"

"barographic"

"barographs"

"barology"

"barometer"

"barometers"

"barometric"

"barometrical"

"barometrically"

"barometrograph"

"barometry"

"baron"

"baronage"

"baronages"

"baroness"

"baronesses"

"baronet"

"baronetcies"

"baronetcy"

"baronets"

"baronial"

"baronies"

"barons"

"barony"

"baroque"

"baroques"

"baroscope"

"barouche"

"barouches"

"barque"

"barquentine"

"barques"

"barrable"

"barrack"

"barracked"

"barracking"

"barracks"

"barracuda"

"barracudas"

"barrage"

"barraged"

"barrages"

"barraging"

"barratrous"

"barratry"

"barre"

"barred"

"barrel"

"barreled"

"barreling"

"barrelled"

"barrelling"

"barrels"

"barren"

"barrener"

"barrenest"

"barrenly"

"barrenness"

"barrens"

"barrets"

"barrette"

"barrettes"

"barricade"

"barricaded"

"barricader"

"barricaders"

"barricades"

"barricading"

"barrier"

"barriers"

"barring"

"barrio"

"barrios"

"barrister"

"barristerial"

"barristers"

"barroom"

"barrooms"

"barrow"

"barrows"

"bars"

"barstool"

"barstools"

"bartend"

"bartended"

"bartender"

"bartenders"

"bartending"

"bartends"

"barter"

"bartered"

"barterer"

"barterers"

"bartering"

"barters"

"bartisans"

"bartizan"

"bartizans"

"bartlett"

"bartletts"

"barware"

"barwares"

"baryon"

"baryonic"

"baryons"

"barytone"

"bas"

"basal"

"Basal Metabolic Index"

"basally"

"basalt"

"basaltic"

"basalts"

"base"

"baseball"

"baseballs"

"baseboard"

"baseboards"

"baseborn"

"based"

"baseless"

"baselessly"

"baselessness"

"baseline"

"baselines"

"basely"

"baseman"

"basemen"

"basement"

"basements"

"baseness"

"baseplate"

"baser"

"bases"

"basest"

"bash"

"bashed"

"basher"

"bashers"

"bashes"

"bashful"

"bashfully"

"bashfulness"

"bashing"

"basic"

"basic contact information"

"basic life support"

"basically"

"basicity"

"basics"

"basified"

"basifier"

"basifiers"

"basifies"

"basify"

"basifying"

"basil"

"basilar"

"basilica"

"basilicas"

"basilisk"

"basilisks"

"basils"

"basin"

"basined"

"basinet"

"basinets"

"basing"

"basins"

"basis"

"bask"

"basked"

"basket"

"basketball"

"basketballs"

"basketful"

"basketfuls"

"basketlike"

"basketries"

"basketry"

"baskets"

"basketwork"

"basking"

"basks"

"basque"

"basques"

"bass"

"basses"

"basset"

"basseted"

"bassets"

"bassetting"

"bassi"

"bassinet"

"bassinets"

"bassist"

"bassists"

"bassly"

"bassness"

"basso"

"bassoon"

"bassoonist"

"bassoonists"

"bassoons"

"bassos"

"basswood"

"basswoods"

"bassy"

"bast"

"bastard"

"bastardies"

"bastardization"

"bastardizations"

"bastardize"

"bastardized"

"bastardizes"

"bastardizing"

"bastardly"

"bastards"

"bastardy"

"baste"

"basted"

"baster"

"basters"

"bastes"

"bastian"

"bastiles"

"bastille"

"bastilles"

"bastinado"

"bastinadoes"

"basting"

"bastings"

"bastion"

"bastioned"

"bastions"

"basts"

"bat"

"batboy"

"batboys"

"batch"

"batched"

"batcher"

"batchers"

"batches"

"batching"

"bate"

"bateau"

"bateaux"

"bated"

"bates"

"batfish"

"bath"

"bathe"

"bathed"

"bather"

"bathers"

"bathes"

"bathetic"

"bathetically"

"bathhouse"

"bathhouses"

"bathing"

"bathless"

"batholith"

"batholithic"

"batholiths"

"bathophobia"

"bathos"

"bathoses"

"bathrobe"

"bathrobes"

"bathroom"

"bathrooms"

"baths"

"bathtub"

"bathtubs"

"bathymeter"

"bathyscaph"

"bathyscaphe"

"bathyscaphes"

"bathysphere"

"bathyspheres"

"bathythermograph"

"batik"

"batiks"

"bating"

"batiste"

"batistes"

"batman"

"batmen"

"batology"

"baton"

"batons"

"batophobia"

"batrachian"

"batrachians"

"batrachophobia"

"bats"

"batsman"

"batsmen"

"battalion"

"battalions"

"batteau"

"batteaux"

"batted"

"batten"

"battened"

"battener"

"batteners"

"battening"

"battens"

"batter"

"battered"

"batteries"

"battering"

"batters"

"battery"

"battier"

"battiest"

"battiks"

"battiness"

"batting"

"battings"

"battle"

"battled"

"battledore"

"battledores"

"battlefield"

"battlefields"

"battlefront"

"battleground"

"battlegrounds"

"battlement"

"battlemented"

"battlements"

"battler"

"battlers"

"battles"

"battleship"

"battleships"

"battlewagon"

"battling"

"batts"

"batty"

"batwing"

"batwoman"

"batwomen"

"bauble"

"baubles"

"baud"

"bauds"

"baulk"

"baulked"

"baulkier"

"baulkiest"

"baulking"

"baulks"

"baulky"

"bauxite"

"bauxites"

"bavarian"

"bawd"

"bawdier"

"bawdies"

"bawdiest"

"bawdily"

"bawdiness"

"bawdric"

"bawdrics"

"bawdries"

"bawdry"

"bawds"

"bawdy"

"bawl"

"bawled"

"bawler"

"bawlers"

"bawling"

"bawls"

"bay"

"bayberries"

"bayberry"

"bayed"

"baying"

"bayonet"

"bayoneted"

"bayoneting"

"bayonets"

"bayonetted"

"bayonetting"

"bayou"

"bayous"

"bays"

"baywood"

"baywoods"

"bazaar"

"bazaars"

"bazar"

"bazars"

"bazooka"

"bazookas"

"bb"

"bbl"

"bdrm"

"be"

"beach"

"beachboy"

"beachboys"

"beachcomber"

"beachcombers"

"beached"

"beaches"

"beachhead"

"beachheads"

"beachier"

"beachiest"

"beaching"

"beachy"

"beacon"

"beaconed"

"beaconing"

"beaconless"

"beacons"

"bead"

"beaded"

"beadier"

"beadiest"

"beadily"

"beading"

"beadings"

"beadle"

"beadles"

"beadlike"

"beadman"

"beadmen"

"beadroll"

"beadrolls"

"beads"

"beadsman"

"beadsmen"

"beadwork"

"beadworks"

"beady"

"beagle"

"beagles"

"beak"

"beaked"

"beaker"

"beakers"

"beakier"

"beakiest"

"beakless"

"beaklike"

"beaks"

"beaky"

"beam"

"beamed"

"beamier"

"beamily"

"beaming"

"beamish"

"beamless"

"beams"

"beamy"

"bean"

"beanbag"

"beanbags"

"beanball"

"beanballs"

"beaned"

"beaneries"

"beanery"

"beanie"

"beanies"

"beaning"

"beanlike"

"beano"

"beanpole"

"beanpoles"

"beans"

"beanstalk"

"beanstalks"

"bear"

"bearable"

"bearably"

"bearberries"

"bearberry"

"bearcat"

"bearcats"

"beard"

"bearded"

"bearding"

"beardless"

"beards"

"bearer"

"bearers"

"bearing"

"bearings"

"bearish"

"bears"

"bearskin"

"bearskins"

"beast"

"beastie"

"beasties"

"beastlier"

"beastliest"

"beastliness"

"beastly"

"beasts"

"beat"

"beatable"

"beaten"

"beater"

"beaters"

"beatific"

"beatifically"

"beatification"

"beatified"

"beatifies"

"beatify"

"beatifying"

"beating"

"beatings"

"beatitude"

"beatitudes"

"beatles"

"beatnik"

"beatniks"

"beats"

"beats per minute"

"beau"

"beaucoup"

"beaufort"

"beauish"

"beaujolais"

"beaumont"

"beaus"

"beaut"

"beauteous"

"beauteously"

"beautician"

"beauticians"

"beauties"

"beautification"

"beautified"

"beautifier"

"beautifiers"

"beautifies"

"beautiful"

"beautifully"

"beautify"

"beautifying"

"beauts"

"beauty"

"beaux"

"beaver"

"beavered"

"beavering"

"beavers"

"bebop"

"bebopper"

"beboppers"

"bebops"

"becalm"

"becalmed"

"becalming"

"becalms"

"became"

"because"

"bechamel"

"bechamels"

"beck"

"becked"

"becking"

"beckon"

"beckoned"

"beckoner"

"beckoners"

"beckoning"

"beckoningly"

"beckons"

"becks"

"becloud"

"beclouded"

"beclouding"

"beclouds"

"become"

"becomes"

"becometh"

"becoming"

"becomingly"

"becomings"

"becurse"

"becurst"

"bed"

"bedamn"

"bedamned"

"bedamns"

"bedaub"

"bedaubed"

"bedaubing"

"bedaubs"

"bedazzle"

"bedazzled"

"bedazzlement"

"bedazzles"

"bedazzling"

"bedbug"

"bedbugs"

"bedchair"

"bedchairs"

"bedclothes"

"bedcover"

"bedcovers"

"beddable"

"bedded"

"bedder"

"bedders"

"bedding"

"beddings"

"bedeck"

"bedecked"

"bedecking"

"bedecks"

"bedevil"

"bedeviled"

"bedeviling"

"bedevilled"

"bedevilling"

"bedevilment"

"bedevils"

"bedew"

"bedewed"

"bedewing"

"bedews"

"bedfast"

"bedfellow"

"bedfellows"

"bedframe"

"bedframes"

"bedgown"

"bedgowns"

"bedight"

"bedighted"

"bedim"

"bedimmed"

"bedimming"

"bedims"

"bedizen"

"bedizened"

"bedizening"

"bedizens"

"bedlam"

"bedlamp"

"bedlamps"

"bedlams"

"bedmaker"

"bedmakers"

"bedmate"

"bedmates"

"bednighted"

"bednights"

"bedouin"

"bedouins"

"bedpan"

"bedpans"

"bedplates"

"bedpost"

"bedposts"

"bedquilt"

"bedquilts"

"bedraggle"

"bedraggled"

"bedraggles"

"bedraggling"

"bedrail"

"bedrails"

"bedrid"

"bedridden"

"bedrock"

"bedrocks"

"bedroll"

"bedrolls"

"bedroom"

"bedrooms"

"bedrug"

"beds"

"bedside"

"bedsides"

"bedsore"

"bedsores"

"bedspread"

"bedspreads"

"bedspring"

"bedsprings"

"bedstand"

"bedstands"

"bedstead"

"bedsteads"

"bedstraw"

"bedstraws"

"bedtime"

"bedtimes"

"beduins"

"bedumb"

"bedumbs"

"bedwarf"

"bedwarfs"

"bee"

"beebee"

"beebees"

"beebread"

"beebreads"

"beech"

"beechen"

"beeches"

"beechier"

"beechiest"

"beechnut"

"beechnuts"

"beechy"

"beef"

"beefburger"

"beefburgers"

"beefcake"

"beefcakes"

"beefeater"

"beefeaters"

"beefed"

"beefier"

"beefiest"

"beefily"

"beefing"

"beefless"

"beefs"

"beefsteak"

"beefsteaks"

"beefy"

"beehive"

"beehives"

"beekeeper"

"beekeepers"

"beekeeping"

"beelike"

"beeline"

"beelines"

"beelzebub"

"been"

"beep"

"beeped"

"beeper"

"beepers"

"beeping"

"beeps"

"beer"

"beerier"

"beeriest"

"beers"

"beery"

"bees"

"beeswax"

"beeswaxes"

"beeswings"

"beet"

"beethoven"

"beetle"

"beetled"

"beetles"

"beetling"

"beetroot"

"beetroots"

"beets"

"beeves"

"befall"

"befallen"

"befalling"

"befalls"

"befell"

"befit"

"befits"

"befitted"

"befitting"

"beflags"

"befog"

"befogged"

"befogging"

"befogs"

"befool"

"befooled"

"befooling"

"befools"

"before"

"beforehand"

"befoul"

"befouled"

"befoulier"

"befouling"

"befouls"

"befriend"

"befriended"

"befriending"

"befriends"

"befuddle"

"befuddled"

"befuddlement"

"befuddlements"

"befuddler"

"befuddlers"

"befuddles"

"befuddling"

"beg"

"began"

"begat"

"beget"

"begets"

"begetter"

"begetters"

"begetting"

"beggar"

"beggared"

"beggaries"

"beggaring"

"beggarliness"

"beggarly"

"beggars"

"beggary"

"begged"

"begging"

"begin"

"beginner"

"beginners"

"beginning"

"beginnings"

"begins"

"begird"

"begirt"

"begone"

"begonia"

"begonias"

"begorah"

"begorra"

"begorrah"

"begot"

"begotten"

"begrime"

"begrimed"

"begrimes"

"begriming"

"begrimmed"

"begrudge"

"begrudged"

"begrudges"

"begrudging"

"begrudgingly"

"begs"

"beguile"

"beguiled"

"beguilement"

"beguilements"

"beguiler"

"beguilers"

"beguiles"

"beguiling"

"beguine"

"beguines"

"begum"

"begums"

"begun"

"behalf"

"behave"

"behaved"

"behaver"

"behavers"

"behaves"

"behaving"

"behavior"

"behavioral"

"behaviorism"

"behaviorist"

"behavioristic"

"behaviorists"

"behaviors"

"behead"

"beheaded"

"beheading"

"beheads"

"beheld"

"behemoth"

"behemoths"

"behest"

"behests"

"behind"

"behindhand"

"behinds"

"behold"

"beholden"

"beholder"

"beholders"

"beholding"

"beholds"

"behoof"

"behoove"

"behooved"

"behooves"

"behooving"

"behove"

"behoved"

"behoves"

"beige"

"beiges"

"beigy"

"being"

"being more active"

"beings"

"beirut"

"bejewel"

"bejeweled"

"bejeweling"

"bejewelled"

"bejewelling"

"bejewels"

"beknighted"

"bel"

"belabor"

"belabored"

"belaboring"

"belabors"

"belabour"

"belaboured"

"belabours"

"belated"

"belatedly"

"belay"

"belayed"

"belaying"

"belays"

"belch"

"belched"

"belcher"

"belchers"

"belches"

"belching"

"beldam"

"beldame"

"beldames"

"beldams"

"beleaguer"

"beleaguered"

"beleaguering"

"beleaguers"

"beleaps"

"beleapt"

"belfast"

"belfries"

"belfry"

"belgian"

"belgians"

"belgium"

"belgrade"

"belie"

"belied"

"belief"

"beliefs"

"belier"

"beliers"

"belies"

"believability"

"believable"

"believably"

"believe"

"believed"

"believer"

"believers"

"believes"

"believeth"

"believing"

"belike"

"belittle"

"belittled"

"belittlement"

"belittler"

"belittlers"

"belittles"

"belittling"

"bell"

"belladonna"

"bellboy"

"bellboys"

"belle"

"belled"

"belles"

"belletrist"

"belletristic"

"belletrists"

"bellevue"

"bellhop"

"bellhops"

"belli"

"bellicose"

"bellicosely"

"bellicoseness"

"bellicosities"

"bellicosity"

"bellied"

"bellies"

"belligerence"

"belligerencies"

"belligerency"

"belligerent"

"belligerently"

"belligerents"

"belling"

"bellman"

"bellmen"

"bello"

"bellow"

"bellowed"

"bellower"

"bellowers"

"bellowing"

"bellows"

"bellpull"

"bellpulls"

"bells"

"bellum"

"bellweather"

"bellwether"

"bellwethers"

"bellworts"

"belly"

"bellyache"

"bellyached"

"bellyaches"

"bellyaching"

"bellybutton"

"bellybuttons"

"bellyful"

"bellyfull"

"bellyfulls"

"bellyfuls"

"bellying"

"belonephobia"

"belong"

"belonged"

"belonging"

"belongings"

"belongs"

"beloved"

"beloveds"

"below"

"belows"

"belt"

"belted"

"belting"

"beltings"

"beltless"

"beltline"

"beltlines"

"belts"

"beltway"

"beltways"

"beluga"

"belugas"

"belvedere"

"belvederes"

"belying"

"bema"

"bemas"

"bemata"

"bemire"

"bemired"

"bemires"

"bemiring"

"bemix"

"bemoan"

"bemoaned"

"bemoaning"

"bemoans"

"bemuse"

"bemused"

"bemuses"

"bemusing"

"ben"

"bench"

"benched"

"bencher"

"benchers"

"benches"

"benching"

"benchmark"

"benchmarked"

"benchmarking"

"benchmarks"

"bend"

"bendable"

"bended"

"bendee"

"bender"

"benders"

"bending"

"bends"

"bendy"

"bene"

"beneath"

"benedict"

"benediction"

"benedictions"

"benedicts"

"benefact"

"benefaction"

"benefactions"

"benefactive"

"benefactor"

"benefactors"

"benefactress"

"benefactresses"

"benefactrices"

"benefactrix"

"benefactrixes"

"benefic"

"benefice"

"beneficence"

"beneficent"

"beneficently"

"benefices"

"beneficial"

"beneficially"

"beneficialness"

"beneficiaries"

"beneficiary"

"beneficiate"

"beneficiated"

"beneficiating"

"beneficing"

"benefit"

"benefit system"

"benefited"

"benefiting"

"benefits"

"benefitted"

"benefitting"

"benes"

"benevolence"

"benevolent"

"benevolently"

"bengal"

"bengals"

"benighted"

"benightedly"

"benightedness"

"benign"

"benignancies"

"benignancy"

"benignant"

"benignantly"

"benignities"

"benignity"

"benignly"

"benin"

"benison"

"benisons"

"benjamin"

"bennets"

"bennies"

"benny"

"bens"

"bent"

"benthal"

"benthic"

"benthos"

"bentonite"

"bentonitic"

"bents"

"bentwood"

"bentwoods"

"benumb"

"benumbed"

"benumbedness"

"benumbing"

"benumbs"

"benzedrine"

"benzene"

"benzenes"

"benzin"

"benzine"

"benzines"

"benzoate"

"benzoates"

"benzocaine"

"benzoic"

"benzoin"

"benzoins"

"benzol"

"benzyl"

"bequeath"

"bequeathal"

"bequeathed"

"bequeathing"

"bequeathment"

"bequeaths"

"bequest"

"bequests"

"berate"

"berated"

"berates"

"berating"

"berber"

"berbers"

"berceuse"

"berceuses"

"bereave"

"bereaved"

"bereavement"

"bereavements"

"bereaver"

"bereavers"

"bereaves"

"bereaving"

"bereft"

"beret"

"berets"

"beretta"

"berettas"

"berg"

"bergamot"

"bergamots"

"bergh"

"bergman"

"bergs"

"berhymed"

"berhymes"

"beriberi"

"beriberis"

"bering"

"berkeley"

"berkelium"

"berlin"

"berliners"

"berlins"

"berm"

"berms"

"bermuda"

"bermudian"

"bermudians"

"bernard"

"berobed"

"berrettas"

"berried"

"berries"

"berry"

"berrying"

"berrylike"

"berserk"

"berserks"

"berth"

"bertha"

"berthas"

"berthed"

"berthing"

"berths"

"beryl"

"beryline"

"beryllium"

"beryls"

"beseech"

"beseeched"

"beseecher"

"beseechers"

"beseeches"

"beseeching"

"beseechingly"

"beseem"

"beseemed"

"beseeming"

"beseems"

"beset"

"besets"

"besetter"

"besetters"

"besetting"

"beshrew"

"beshrewed"

"beshrews"

"beside"

"besides"

"besiege"

"besieged"

"besiegement"

"besieger"

"besiegers"

"besieges"

"besieging"

"beslime"

"besmear"

"besmeared"

"besmearing"

"besmears"

"besmile"

"besmirch"

"besmirched"

"besmircher"

"besmirchers"

"besmirches"

"besmirching"

"besmoke"

"besmuts"

"besnows"

"besom"

"besoms"

"besot"

"besots"

"besotted"

"besotting"

"besought"

"bespake"

"bespangle"

"bespangled"

"bespangles"

"bespangling"

"bespatter"

"bespattered"

"bespattering"

"bespatters"

"bespeak"

"bespeaking"

"bespeaks"

"bespectacled"

"bespoke"

"bespoken"

"bespread"

"bespreading"

"bespreads"

"besprinkle"

"besprinkled"

"besprinkles"

"besprinkling"

"bess"

"bessemer"

"best"

"bested"

"bestial"

"bestialities"

"bestiality"

"bestialize"

"bestialized"

"bestializes"

"bestializing"

"bestially"

"bestiaries"

"bestiary"

"besting"

"bestir"

"bestirred"

"bestirring"

"bestirs"

"bestow"

"bestowal"

"bestowals"

"bestowed"

"bestowing"

"bestows"

"bestrew"

"bestrewed"

"bestrewing"

"bestrewn"

"bestrews"

"bestridden"

"bestride"

"bestrides"

"bestriding"

"bestrode"

"bests"

"bestseller"

"bestselling"

"bet"

"beta"

"betake"

"betaken"

"betakes"

"betaking"

"betas"

"betatron"

"betatrons"

"bete"

"betel"

"betelnut"

"betelnuts"

"betels"

"betes"

"bethel"

"bethels"

"bethink"

"bethinks"

"bethlehem"

"bethought"

"beths"

"betide"

"betided"

"betides"

"betiding"

"betime"

"betimes"

"betoken"

"betokened"

"betokening"

"betokens"

"betonies"

"betony"

"betook"

"betray"

"betrayal"

"betrayals"

"betrayed"

"betrayer"

"betrayers"

"betraying"

"betrays"

"betroth"

"betrothal"

"betrothals"

"betrothed"

"betrothing"

"betrothment"

"betroths"

"bets"

"betta"

"bettas"

"betted"

"better"

"bettered"

"bettering"

"betterment"

"betters"

"betting"

"bettor"

"bettors"

"betty"

"between"

"betweenbrain"

"betweens"

"betwixt"

"bevatron"

"bevatrons"

"bevel"

"beveled"

"beveler"

"bevelers"

"beveling"

"bevelled"

"beveller"

"bevellers"

"bevelling"

"bevels"

"beverage"

"beverages"

"bevies"

"bevy"

"bewail"

"bewailed"

"bewailer"

"bewailers"

"bewailing"

"bewails"

"beware"

"bewared"

"bewares"

"bewaring"

"bewig"

"bewigged"

"bewigs"

"bewilder"

"bewildered"

"bewildering"

"bewilderingly"

"bewilderment"

"bewilders"

"bewitch"

"bewitched"

"bewitches"

"bewitching"

"bewitchment"

"bewitchments"

"bewrayed"

"bewrayer"

"bewrays"

"bey"

"beyond"

"beyonds"

"beys"

"bezel"

"bezels"

"bezils"

"bezique"

"bezoar"

"bhakta"

"bhaktas"

"bhakti"

"bhaktis"

"bhang"

"bhangs"

"bhutan"

"bhutanese"

"bialy"

"bialys"

"biannual"

"biannually"

"bias"

"biased"

"biasedly"

"biases"

"biasing"

"biasness"

"biassed"

"biasses"

"biassing"

"biathlon"

"biathlons"

"biaxal"

"biaxial"

"bib"

"bibasic"

"bibbed"

"bibber"

"bibberies"

"bibbers"

"bibbery"

"bibbing"

"bibbs"

"bibelot"

"bibelots"

"bible"

"bibles"

"bibless"

"biblical"

"biblically"

"bibliog"

"bibliographer"

"bibliographers"

"bibliographic"

"bibliographical"

"bibliographically"

"bibliographies"

"bibliography"

"bibliology"

"bibliomania"

"bibliophile"

"bibliophiles"

"bibliophobia"

"bibliotherapies"

"bibliotherapist"

"bibliotherapy"

"bibliotics"

"bibs"

"bibulosities"

"bibulosity"

"bibulous"

"bicameral"

"bicarb"

"bicarbonate"

"bicarbonates"

"bicarbs"

"bicentenaries"

"bicentenary"

"bicentennial"

"bicentennially"

"bicentennials"

"biceps"

"bicepses"

"bichloride"

"bichlorides"

"bichrome"

"bicker"

"bickered"

"bickerer"

"bickerers"

"bickering"

"bickers"

"bicolor"

"bicolors"

"bicolour"

"bicolours"

"biconcave"

"biconcavity"

"biconvex"

"biconvexity"

"bicorn"

"bicornes"

"bicorporal"

"bicorporeal"

"bicultural"

"biculturalism"

"bicuspid"

"bicuspids"

"bicycle"

"bicycled"

"bicycler"

"bicyclers"

"bicycles"

"bicyclic"

"bicycling"

"bicyclist"

"bicyclists"

"bid"

"biddable"

"biddably"

"bidden"

"bidder"

"bidders"

"biddies"

"bidding"

"biddings"

"biddy"

"bide"

"bided"

"bider"

"biders"

"bides"

"bidet"

"bidets"

"biding"

"bidirectional"

"bids"

"biennia"

"biennial"

"biennially"

"biennials"

"biennium"

"bienniums"

"biens"

"bier"

"biers"

"biff"

"biffed"

"biffies"

"biffing"

"biffins"

"biffs"

"biffy"

"bifid"

"biflex"

"bifocal"

"bifocals"

"bifold"

"biforked"

"biform"

"bifurcate"

"bifurcated"

"bifurcates"

"bifurcating"

"bifurcation"

"bifurcations"

"big"

"bigamies"

"bigamist"

"bigamistic"

"bigamists"

"bigamize"

"bigamized"

"bigamizing"

"bigamous"

"bigamously"

"bigamy"

"bigeye"

"bigeyes"

"bigfoot"

"bigger"

"biggest"

"biggie"

"biggies"

"bigging"

"biggings"

"biggish"

"bighead"

"bigheaded"

"bigheads"

"bighearted"

"bigheartedly"

"bighorn"

"bighorns"

"bight"

"bighted"

"bights"

"bigly"

"bigmouth"

"bigmouthed"

"bigmouths"

"bigness"

"bignesses"

"bigot"

"bigoted"

"bigotedly"

"bigotries"

"bigotry"

"bigots"

"bigwig"

"bigwigs"

"bihourly"

"bijou"

"bijous"

"bijoux"

"bike"

"Bike computer"

"biked"

"biker"

"bikers"

"bikes"

"bikeway"

"bikeways"

"biking"

"bikini"

"bikinied"

"bikinis"

"bilabial"

"bilabials"

"bilateral"

"bilateralism"

"bilateralistic"

"bilateralities"

"bilaterality"

"bilaterally"

"bilberry"

"bilbo"

"bilbos"

"bile"

"biles"

"bilge"

"bilged"

"bilges"

"bilgier"

"bilgiest"

"bilging"

"bilgy"

"bilinear"

"bilingual"

"bilingually"

"bilious"

"biliousness"

"bilk"

"bilked"

"bilker"

"bilkers"

"bilking"

"bilks"

"bill"

"billable"

"billboard"

"billboards"

"billed"

"biller"

"billers"

"billet"

"billeted"

"billeter"

"billeters"

"billeting"

"billets"

"billfold"

"billfolds"

"billhead"

"billheads"

"billhook"

"billhooks"

"billiard"

"billiards"

"billie"

"billies"

"billing"

"billings"

"billings method"

"billingsgate"

"billion"

"billionaire"

"billionaires"

"billions"

"billionth"

"billionths"

"billow"

"billowed"

"billowier"

"billowiest"

"billowing"

"billows"

"billowy"

"bills"

"billy"

"billycan"

"billycans"

"bilobed"

"bimah"

"bimahs"

"bimanual"

"bimester"

"bimesters"

"bimetal"

"bimetallic"

"bimetallism"

"bimetallist"

"bimetallists"

"bimetals"

"bimethyls"

"bimodal"

"bimolecular"

"bimonthlies"

"bimonthly"

"bin"

"binal"

"binaries"

"binary"

"binaural"

"binaurally"

"bind"

"bindable"

"binder"

"binderies"

"binders"

"bindery"

"binding"

"bindings"

"bindle"

"bindles"

"binds"

"bindweed"

"bindweeds"

"bines"

"binge"

"binges"

"bingo"

"bingos"

"binnacle"

"binnacles"

"binned"

"binocular"

"binocularly"

"binoculars"

"binomial"

"binomials"

"bins"

"bints"

"bio"

"bioacoustics"

"bioactivities"

"bioactivity"

"bioassayed"

"bioassays"

"bioastronautical"

"bioastronautics"

"biocatalyst"

"biochemic"

"biochemical"

"biochemically"

"biochemist"

"biochemistries"

"biochemistry"

"biochemists"

"biocidal"

"biocide"

"biocides"

"bioclean"

"bioclimatologies"

"bioclimatology"

"biocycle"

"biocycles"

"biodegradability"

"biodegradable"

"biodegradation"

"biodegrade"

"biodegraded"

"biodegrading"

"biodynamics"

"bioecologies"

"bioecology"

"bioelectric"

"bioelectrical"

"bioelectricities"

"bioelectricity"

"bioelectronics"

"bioenergetics"

"bioengineering"

"bioenvironmental"

"bioenvironmentaly"

"biofeedback"

"bioflavonoid"

"biogenic"

"biogenies"

"biogeochemistry"

"biogeographer"

"biogeographers"

"biogeographic"

"biogeographical"

"biogeography"

"biographer"

"biographers"

"biographic"

"biographical"

"biographies"

"biography"

"biohazard"

"biokinetics"

"biol"

"biologic"

"biological"

"biologically"

"biologics"

"biologies"

"biologist"

"biologists"

"biology"

"bioluminescence"

"biomass"

"biomasses"

"biomaterial"

"biomathematics"

"biome"

"biomechanics"

"biomedical"

"biomedicine"

"biomes"

"biometer"

"biometric sensors"

"biometrics"

"biometries"

"biometry"

"biomicroscope"

"biomicroscopies"

"biomicroscopy"

"bionic"

"bionics"

"bionomics"

"biont"

"biophotometer"

"biophysical"

"biophysicist"

"biophysicists"

"biophysics"

"biophysiography"

"biopsies"

"biopsy"

"biopsychologies"

"biopsychology"

"bioptic"

"bioresearch"

"biorhythm"

"biorhythmic"

"biorhythmicities"

"biorhythmicity"

"biorythmic"

"bios"

"biosatellite"

"biosatellites"

"bioscience"

"biosciences"

"bioscientist"

"bioscope"

"bioscopes"

"bioscopy"

"biosensor"

"biosphere"

"biospheres"

"biostatistics"

"biosyntheses"

"biosynthesis"

"biota"

"biotas"

"biotechnological"

"biotechnologicaly"

"biotechnologies"

"biotechnology"

"biotelemetric"

"biotelemetries"

"biotelemetry"

"biotic"

"biotical"

"biotically"

"biotics"

"biotin"

"biotins"

"biotite"

"biotype"

"biotypes"

"biparental"

"biparted"

"bipartisan"

"bipartisanship"

"bipartite"

"bipartition"

"biparty"

"biped"

"bipedal"

"bipeds"

"biplane"

"biplanes"

"bipod"

"bipods"

"bipolar"

"bipolar disorder"

"bipolarity"

"bipotentialities"

"bipotentiality"

"biracial"

"biracialism"

"birch"

"birched"

"birchen"

"bircher"

"birchers"

"birches"

"birching"

"birchism"

"bird"

"Bird Chirping"

"birdbath"

"birdbaths"

"birdbrain"

"birdbrains"

"birdcage"

"birdcages"

"birdcall"

"birdcalls"

"birded"

"birder"

"birders"

"birdhouse"

"birdhouses"

"birdie"

"birdied"

"birdieing"

"birdies"

"birding"

"birdlime"

"birdlimed"

"birdlimes"

"birdliming"

"birdman"

"birdmen"

"birds"

"birdseed"

"birdseeds"

"birdseye"

"birdseyes"

"birefractive"

"bireme"

"biremes"

"biretta"

"birettas"

"birmingham"

"birretta"

"birrettas"

"birth"

"Birth control"

"birthday"

"birthdays"

"birthed"

"birthing"

"birthmark"

"birthmarks"

"birthplace"

"birthplaces"

"birthrate"

"birthrates"

"birthright"

"birthrights"

"births"

"birthstone"

"birthstones"

"bis"

"biscuit"

"biscuits"

"bisect"

"bisected"

"bisecting"

"bisection"

"bisectional"

"bisectionally"

"bisections"

"bisector"

"bisectors"

"bisects"

"bisexed"

"bisexual"

"bisexualism"

"bisexuality"

"bisexually"

"bisexuals"

"bishop"

"bishoped"

"bishoping"

"bishopric"

"bishoprics"

"bishops"

"bismarck"

"bismark"

"bismuth"

"bismuthal"

"bismuthic"

"bismuths"

"bison"

"bisons"

"bisque"

"bisques"

"bistable"

"bistate"

"bistro"

"bistros"

"bisulfate"

"bisulfide"

"bisulfite"

"bit"

"bitable"

"bitch"

"bitched"

"bitchery"

"bitches"

"bitchier"

"bitchiest"

"bitchily"

"bitching"

"bitchy"

"bite"

"biteable"

"biter"

"biters"

"bites"

"biting"

"bitingly"

"bits"

"bitsy"

"bitte"

"bitted"

"bitten"

"bitter"

"bittered"

"bitterer"

"bitterest"

"bittering"

"bitterly"

"bittern"

"bitterness"

"bitterns"

"bitters"

"bittersweet"

"bittersweets"

"bittier"

"bittiest"

"bitting"

"bitts"

"bitty"

"bitumen"

"bitumens"

"bituminous"

"bivalencies"

"bivalent"

"bivalve"

"bivalves"

"bivouac"

"bivouacked"

"bivouacking"

"bivouacks"

"bivouacs"

"biweeklies"

"biweekly"

"biyearly"

"bizarre"

"bizarrely"

"bizarreness"

"bizarres"

"bizonal"

"bizones"

"bks"

"blab"

"blabbed"

"blabber"

"blabbered"

"blabbering"

"blabbermouth"

"blabbers"

"blabbing"

"blabby"

"blabs"

"black"

"blackamoor"

"blackamoors"

"blackball"

"blackballed"

"blackballing"

"blackballs"

"blackberries"

"blackberry"

"blackbird"

"blackbirds"

"blackboard"

"blackboards"

"blacked"

"blacken"

"blackened"

"blackener"

"blackeners"

"blackening"

"blackens"

"blacker"

"blackest"

"blackfeet"

"blackfoot"

"blackguard"

"blackguards"

"blackhead"

"blackheads"

"blacking"

"blackings"

"blackish"

"blackjack"

"blackjacked"

"blackjacking"

"blackjacks"

"blacklight"

"blacklist"

"blacklisted"

"blacklisting"

"blacklists"

"blackly"

"blackmail"

"blackmailed"

"blackmailer"

"blackmailers"

"blackmailing"

"blackmails"

"blackness"

"blackout"

"blackouts"

"blacks"

"blacksmith"

"blacksmiths"

"blackthorn"

"blackthorns"

"blacktop"

"blacktopped"

"blacktopping"

"blacktops"

"bladder"

"bladder infection"

"bladders"

"bladdery"

"blade"

"bladed"

"blades"

"blah"

"blahs"

"blain"

"blains"

"blamable"

"blamableness"

"blamably"

"blame"

"blameable"

"blamed"

"blameful"

"blameless"

"blamelessly"

"blamelessness"

"blamer"

"blamers"

"blames"

"blameworthiness"

"blameworthy"

"blaming"

"blanc"

"blanch"

"blanche"

"blanched"

"blancher"

"blanchers"

"blanches"

"blanching"

"blancmange"

"blancmanges"

"bland"

"blander"

"blandest"

"blandish"

"blandished"

"blandisher"

"blandishers"

"blandishes"

"blandishing"

"blandishment"

"blandishments"

"blandly"

"blandness"

"blank"

"blanked"

"blanker"

"blankest"

"blanket"

"blanketed"

"blanketing"

"blankets"

"blanking"

"blankly"

"blankness"

"blanks"

"blare"

"blared"

"blares"

"blaring"

"blarney"

"blarneyed"

"blarneying"

"blarneys"

"blase"

"blaspheme"

"blasphemed"

"blasphemer"

"blasphemers"

"blasphemes"

"blasphemies"

"blaspheming"

"blasphemous"

"blasphemously"

"blasphemy"

"blast"

"blasted"

"blaster"

"blasters"

"blastier"

"blasting"

"blastings"

"blastoff"

"blastoffs"

"blasts"

"blasty"

"blat"

"blatancies"

"blatancy"

"blatant"

"blatantly"

"blather"

"blathered"

"blathering"

"blathers"

"blatherskite"

"blatherskites"

"blats"

"blatted"

"blatter"

"blattering"

"blatters"

"blatting"

"blaze"

"blazed"

"blazer"

"blazers"

"blazes"

"blazing"

"blazon"

"blazoned"

"blazoner"

"blazoners"

"blazoning"

"blazonry"

"blazons"

"bldg"

"bleach"

"bleached"

"bleacher"

"bleachers"

"bleaches"

"bleaching"

"bleachs"

"bleak"

"bleaker"

"bleakest"

"bleakish"

"bleakly"

"bleakness"

"bleaks"

"blear"

"bleared"

"blearier"

"bleariest"

"blearily"

"blearing"

"blears"

"bleary"

"bleat"

"bleated"

"bleater"

"bleaters"

"bleating"

"bleats"

"bled"

"bleed"

"bleeder"

"bleeders"

"bleeding"

"bleedings"

"bleeds"

"bleep"

"bleeped"

"bleeping"

"bleeps"

"blemish"

"blemished"

"blemishes"

"blemishing"

"blench"

"blenched"

"blencher"

"blenchers"

"blenches"

"blenching"

"blenchingly"

"blend"

"blended"

"blender"

"blenders"

"blending"

"blends"

"blennies"

"blennophobia"

"blenny"

"blent"

"blepharitis"

"bless"

"blessed"

"blesseder"

"blessedest"

"blessedly"

"blessedness"

"blesser"

"blessers"

"blesses"

"blessing"

"blessings"

"blest"

"blether"

"blethered"

"blethers"

"blew"

"blight"

"blighted"

"blighter"

"blighters"

"blighties"

"blighting"

"blightingly"

"blights"

"blighty"

"blimey"

"blimp"

"blimpish"

"blimps"

"blimy"

"blind"

"blindage"

"blindages"

"blinded"

"blinder"

"blinders"

"blindest"

"blindfold"

"blindfolded"

"blindfolding"

"blindfolds"

"blinding"

"blindly"

"blindness"

"blinds"

"blini"

"blinis"

"blink"

"blinked"

"blinker"

"blinkered"

"blinkering"

"blinkers"

"blinking"

"blinks"

"blintz"

"blintze"

"blintzes"

"blip"

"blipped"

"blippers"

"blipping"

"blips"

"bliss"

"blisses"

"blissful"

"blissfully"

"blissfulness"

"blister"

"blistered"

"blistering"

"blisters"

"blistery"

"blithe"

"blithely"

"blitheness"

"blither"

"blithered"

"blithering"

"blithers"

"blithesome"

"blithest"

"blitz"

"blitzed"

"blitzes"

"blitzing"

"blitzkrieg"

"blitzkrieged"

"blitzkrieging"

"blitzkriegs"

"blizzard"

"blizzards"

"bloat"

"bloated"

"bloater"

"bloaters"

"bloating"

"bloats"

"blob"

"blobbed"

"blobbing"

"blobs"

"bloc"

"block"

"blockade"

"blockaded"

"blockader"

"blockaders"

"blockades"

"blockading"

"blockage"

"blockages"

"blockbuster"

"blockbusters"

"blockbusting"

"blocked"

"blocker"

"blockers"

"blockhead"

"blockheads"

"blockhouse"

"blockhouses"

"blockier"

"blockiest"

"blocking"

"blockish"

"blocks"

"blocky"

"blocs"

"bloke"

"blokes"

"blond"

"blonde"

"blonder"

"blondes"

"blondest"

"blondish"

"blondness"

"blonds"

"blood"

"Blood Alcohol"

"blood circulation"

"blood flow"

"blood glucose"

"blood pressure"

"blood pressure control"

"Blood sugar"

"blood test"

"Blood work"

"bloodbath"

"bloodcurdling"

"bloodcurdlingly"

"blooded"

"bloodedness"

"bloodfin"

"bloodfins"

"bloodhound"

"bloodhounds"

"bloodied"

"bloodier"

"bloodies"

"bloodiest"

"bloodily"

"bloodiness"

"blooding"

"bloodings"

"bloodless"

"bloodletting"

"bloodlettings"

"bloodline"

"bloodlines"

"bloodmobile"

"bloodmobiles"

"bloodred"

"bloodroot"

"bloodroots"

"bloods"

"bloodshed"

"bloodshedder"

"bloodshedding"

"bloodshot"

"bloodstain"

"bloodstained"

"bloodstone"

"bloodstones"

"bloodstream"

"bloodstreams"

"bloodsucker"

"bloodsuckers"

"bloodsucking"

"bloodtest"

"bloodthirstier"

"bloodthirstiest"

"bloodthirstily"

"bloodthirstiness"

"bloodthirsty"

"bloodworm"

"bloody"

"bloodying"

"bloom"

"bloomed"

"bloomer"

"bloomers"

"bloomery"

"bloomier"

"bloomiest"

"blooming"

"blooms"

"bloomy"

"bloop"

"blooped"

"blooper"

"bloopers"

"blooping"

"bloops"

"blossom"

"blossomed"

"blossoming"

"blossoms"

"blossomy"

"blot"

"blotch"

"blotched"

"blotches"

"blotchier"

"blotchiest"

"blotching"

"blotchy"

"blots"

"blotted"

"blotter"

"blotters"

"blottier"

"blottiest"

"blotting"

"blotto"

"blotty"

"blouse"

"bloused"

"blouses"

"blousier"

"blousiest"

"blousily"

"blousing"

"blouson"

"blousons"

"blousy"

"blow"

"blowback"

"blowby"

"blowbys"

"blower"

"blowers"

"blowfish"

"blowfishes"

"blowflies"

"blowfly"

"blowgun"

"blowguns"

"blowhard"

"blowhards"

"blowhole"

"blowholes"

"blowier"

"blowiest"

"blowiness"

"blowing"

"blowjob"

"blown"

"blowoff"

"blowoffs"

"blowout"

"blowouts"

"blowpipe"

"blowpipes"

"blows"

"blowsed"

"blowsier"

"blowsiest"

"blowsily"

"blowsy"

"blowtorch"

"blowtorches"

"blowtube"

"blowtubes"

"blowup"

"blowups"

"blowy"

"blowzier"

"blowziest"

"blowzy"

"blubber"

"blubbered"

"blubberer"

"blubberers"

"blubbering"

"blubbers"

"blubbery"

"blucher"

"bluchers"

"bludgeon"

"bludgeoned"

"bludgeoning"

"bludgeons"

"blue"

"blueball"

"blueballs"

"bluebeard"

"bluebell"

"bluebells"

"blueberries"

"blueberry"

"bluebills"

"bluebird"

"bluebirds"

"blueblack"

"bluebonnet"

"bluebonnets"

"bluebook"

"bluebooks"

"bluebottle"

"bluebottles"

"bluecap"

"bluecoat"

"bluecoats"

"blued"

"bluefin"

"bluefins"

"bluefish"

"bluefishes"

"bluegill"

"bluegills"

"bluegrass"

"bluegum"

"bluegums"

"blueing"

"blueings"

"blueish"

"bluejacket"

"bluejackets"

"bluejay"

"bluejays"

"bluely"

"blueness"

"bluenose"

"bluenoses"

"bluepoint"

"bluepoints"

"blueprint"

"blueprinted"

"blueprinting"

"blueprints"

"bluer"

"blues"

"bluesman"

"bluesmen"

"bluest"

"bluestocking"

"bluestockings"

"bluesy"

"bluet"

"bluetooth"

"bluetooth glucose meter"

"bluey"

"blueys"

"bluff"

"bluffed"

"bluffer"

"bluffers"

"bluffest"

"bluffing"

"bluffly"

"bluffs"

"bluing"

"bluings"

"bluish"

"blunder"

"blunderbuss"

"blunderbusses"

"blundered"

"blunderer"

"blunderers"

"blundering"

"blunders"

"blunge"

"blunged"

"blunger"

"blungers"

"blunges"

"blunging"

"blunt"

"blunted"

"blunter"

"bluntest"

"blunting"

"bluntly"

"bluntness"

"blunts"

"blur"

"blurb"

"blurbs"

"blurred"

"blurrier"

"blurriest"

"blurrily"

"blurring"

"blurry"

"blurs"

"blurt"

"blurted"

"blurter"

"blurters"

"blurting"

"blurts"

"blush"

"blushed"

"blusher"

"blushers"

"blushes"

"blushful"

"blushfully"

"blushing"

"bluster"

"blustered"

"blusterer"

"blusterers"

"blustering"

"blusters"

"blustery"

"blvd"

"BMI"

"BMI calculation"

"boa"

"boar"

"board"

"boarded"

"boarder"

"boarders"

"boarding"

"boardinghouse"

"boardinghouses"

"boardings"

"boardman"

"boardmen"

"boards"

"boardwalk"

"boardwalks"

"boarish"

"boars"

"boas"

"boast"

"boasted"

"boaster"

"boasters"

"boastful"

"boastfully"

"boastfulness"

"boasting"

"boastingly"

"boasts"

"boat"

"boatable"

"boatbill"

"boatbills"

"boated"

"boatel"

"boatels"

"boater"

"boaters"

"boating"

"boatings"

"boatload"

"boatloads"

"boatman"

"boatmen"

"boats"

"boatsman"

"boatsmen"

"boatswain"

"boatswains"

"boatyard"

"boatyards"

"bob"

"bobbed"

"bobber"

"bobbers"

"bobbery"

"bobbies"

"bobbin"

"bobbinets"

"bobbing"

"bobbins"

"bobble"

"bobbled"

"bobbles"

"bobbling"

"bobby"

"bobbysocks"

"bobbysoxer"

"bobbysoxers"

"bobcat"

"bobcats"

"bobolink"

"bobolinks"

"bobs"

"bobsled"

"bobsledded"

"bobsledder"

"bobsledders"

"bobsledding"

"bobsleds"

"bobtail"

"bobtailed"

"bobtailing"

"bobtails"

"bobwhite"

"bobwhites"

"boca"

"bocaccio"

"bocce"

"bocces"

"bocci"

"boccie"

"boccies"

"boche"

"boches"

"bock"

"bocks"

"bod"

"bode"

"boded"

"bodega"

"bodegas"

"bodes"

"bodice"

"bodices"

"bodied"

"bodies"

"bodiless"

"bodily"

"boding"

"bodingly"

"bodings"

"bodkin"

"bodkins"

"bods"

"body"

"body area"

"Body fat"

"body mass index"

"Body Mass Indicator"

"Body Structure"

"body temperature"

"body view"

"body weight"

"body works"

"bodybuilder"

"bodybuilders"

"bodybuilding"

"bodyguard"

"bodyguards"

"bodying"

"bodysurf"

"bodysurfed"

"bodysurfs"

"bodyweight"

"bodywork"

"bodyworks"

"boeing"

"boer"

"boers"

"boff"

"boffin"

"boffins"

"boffo"

"boffola"

"boffolas"

"boffos"

"boffs"

"bog"

"bogart"

"bogey"

"bogeying"

"bogeyman"

"bogeymen"

"bogeys"

"bogged"

"boggier"

"boggiest"

"bogging"

"boggish"

"boggle"

"boggled"

"boggler"

"bogglers"

"boggles"

"boggling"

"boggy"

"bogie"

"bogies"

"bogle"

"bogled"

"bogles"

"bogota"

"bogs"

"bogus"

"bogy"

"bogyism"

"bogyman"

"bogymen"

"bohemia"

"bohemian"

"bohemians"

"bohemias"

"bohunk"

"bohunks"

"boil"

"boilable"

"boiled"

"boiler"

"boilermaker"

"boilermakers"

"boilers"

"boiling"

"boils"

"boise"

"boisterous"

"boisterously"

"boisterousness"

"bola"

"bolas"

"bold"

"bolded"

"bolder"

"boldest"

"boldface"

"boldfaced"

"boldfaces"

"boldfacing"

"bolding"

"boldly"

"boldness"

"bole"

"bolero"

"boleros"

"boles"

"bolide"

"bolides"

"bolivar"

"bolivars"

"bolivia"

"bolivian"

"bolivians"

"bolivias"

"boll"

"bollard"

"bollards"

"bolled"

"bolling"

"bollix"

"bollixed"

"bollixes"

"bollixing"

"bolloxed"

"bolloxes"

"bolls"

"bolo"

"bologna"

"bolognas"

"bolometer"

"boloney"

"boloneys"

"bolos"

"bolshevik"

"bolsheviks"

"bolshevism"

"bolshevist"

"bolshevists"

"bolster"

"bolstered"

"bolsterer"

"bolsterers"

"bolstering"

"bolsters"

"bolt"

"bolted"

"bolter"

"bolters"

"bolthead"

"boltheads"

"bolting"

"bolts"

"bolus"

"boluses"

"bomb"

"bombard"

"bombarded"

"bombardier"

"bombardiers"

"bombarding"

"bombardment"

"bombardments"

"bombards"

"bombast"

"bombastic"

"bombastically"

"bombasts"

"bombay"

"bombazine"

"bombe"

"bombed"

"bomber"

"bombers"

"bombes"

"bombing"

"bombings"

"bombload"

"bombloads"

"bombproof"

"bombs"

"bombshell"

"bombshells"

"bombsight"

"bombsights"

"bon"

"bona"

"bonanza"

"bonanzas"

"bonbon"

"bonbons"

"bond"

"bondable"

"bondage"

"bondages"

"bonded"

"bonder"

"bonders"

"bondholder"

"bondholders"

"bonding"

"bondless"

"bondmaid"

"bondmaids"

"bondman"

"bondmen"

"bonds"

"bondsman"

"bondsmen"

"bondwoman"

"bondwomen"

"bone"

"boneblack"

"boned"

"bonefish"

"bonefishes"

"bonehead"

"boneheads"

"boneless"

"bonelet"

"boner"

"boners"

"bones"

"boneset"

"bonesets"

"bonesetter"

"boney"

"boneyard"

"boneyards"

"bonfire"

"bonfires"

"bong"

"bonged"

"bonging"

"bongo"

"bongoes"

"bongoist"

"bongoists"

"bongos"

"bongs"

"bonhomie"

"bonhomies"

"bonier"

"boniest"

"boniface"

"bonifaces"

"boniness"

"boning"

"bonita"

"bonitas"

"bonito"

"bonitoes"

"bonitos"

"bonjour"

"bonkers"

"bonnet"

"bonneted"

"bonneting"

"bonnets"

"bonnie"

"bonnier"

"bonniest"

"bonnily"

"bonniness"

"bonny"

"bonnyclabber"

"bono"

"bonos"

"bons"

"bonsai"

"bonsoir"

"bonum"

"bonus"

"bonuses"

"bony"

"bonze"

"bonzer"

"bonzes"

"boo"

"boob"

"boobies"

"booboo"

"booboos"

"boobs"

"booby"

"boodle"

"boodled"

"boodler"

"boodlers"

"boodles"

"boodling"

"booed"

"booger"

"boogers"

"boogie"

"boogies"

"boogyman"

"boogymen"

"boohoo"

"boohooed"

"boohooing"

"boohoos"

"booing"

"book"

"bookbinder"

"bookbinders"

"bookbinding"

"bookcase"

"bookcases"

"booked"

"bookend"

"bookends"

"booker"

"bookers"

"bookie"

"bookies"

"booking"

"bookings"

"bookish"

"bookkeeper"

"bookkeepers"

"bookkeeping"

"booklet"

"booklets"

"booklists"

"booklore"

"booklores"

"bookmaker"

"bookmakers"

"bookmaking"

"bookman"

"bookmark"

"bookmarks"

"bookmen"

"bookmobile"

"bookmobiles"

"bookplate"

"bookplates"

"bookrack"

"bookracks"

"bookrest"

"bookrests"

"books"

"bookseller"

"booksellers"

"bookshelf"

"bookshelves"

"bookshop"

"bookshops"

"bookstore"

"bookstores"

"bookworm"

"bookworms"

"boolean"

"boom"

"boomage"

"boomed"

"boomer"

"boomerang"

"boomeranged"

"boomeranging"

"boomerangs"

"boomers"

"boomier"

"booming"

"boomkin"

"boomlet"

"booms"

"boomtown"

"boomtowns"

"boomy"

"boon"

"boondocks"

"boondoggle"

"boondoggled"

"boondoggler"

"boondogglers"

"boondoggles"

"boondoggling"

"boonies"

"boons"

"boor"

"boorish"

"boorishly"

"boorishness"

"boors"

"boos"

"boost"

"boost your unconscious determination"

"boosted"

"booster"

"boosters"

"boosting"

"boosts"

"boot"

"bootblack"

"bootblacks"

"booted"

"bootee"

"bootees"

"booteries"

"bootery"

"booth"

"booths"

"bootie"

"booties"

"booting"

"bootjack"

"bootjacks"

"bootlace"

"bootlaces"

"bootleg"

"bootlegged"

"bootlegger"

"bootleggers"

"bootlegging"

"bootlegs"

"bootless"

"bootlessly"

"bootlick"

"bootlicked"

"bootlicker"

"bootlickers"

"bootlicking"

"bootlicks"

"boots"

"bootstrap"

"bootstrapped"

"bootstrapping"

"bootstraps"

"booty"

"bootyless"

"booze"

"boozed"

"boozer"

"boozers"

"boozes"

"boozier"

"booziest"

"boozily"

"boozing"

"boozy"

"bop"

"bopped"

"bopper"

"boppers"

"bopping"

"bops"

"borage"

"borages"

"boranes"

"borate"

"borated"

"borates"

"borax"

"boraxes"

"borborygmatic"

"borborygmies"

"borborygmus"

"bordello"

"bordellos"

"bordels"

"border"

"bordereau"

"bordered"

"borderer"

"borderers"

"bordering"

"borderings"

"borderland"

"borderlands"

"borderline"

"borderlines"

"borders"

"bordures"

"bore"

"boreal"

"borealis"

"bored"

"boredom"

"boredoms"

"bores"

"boric"

"boring"

"boringly"

"borings"

"born"

"borne"

"borneo"

"boron"

"boronic"

"borons"

"borough"

"boroughs"

"borrow"

"borrowed"

"borrower"

"borrowers"

"borrowing"

"borrows"

"borsch"

"borscht"

"borschts"

"borsht"

"borshts"

"borstal"

"borstals"

"bort"

"borts"

"borty"

"bortz"

"borzoi"

"borzois"

"bosh"

"boskages"

"boskier"

"boskiest"

"bosks"

"bosky"

"bosom"

"bosomed"

"bosoming"

"bosoms"

"bosomy"

"boson"

"bosons"

"bosque"

"bosques"

"bosquet"

"boss"

"bossa"

"bossdom"

"bossed"

"bosses"

"bossier"

"bossies"

"bossiest"

"bossily"

"bossiness"

"bossing"

"bossism"

"bossisms"

"bossy"

"boston"

"bostonian"

"bostonians"

"bostons"

"bosun"

"bosuns"

"bot"

"botanic"

"botanical"

"botanies"

"botanist"

"botanists"

"botanize"

"botanized"

"botanizes"

"botanizing"

"botany"

"botch"

"botched"

"botcher"

"botchers"

"botchery"

"botches"

"botchier"

"botchiest"

"botchily"

"botching"

"botchy"

"botfly"

"both"

"bother"

"bothered"

"bothering"

"bothers"

"bothersome"

"botswana"

"botticelli"

"bottle"

"bottled"

"bottleful"

"bottlefuls"

"bottleneck"

"bottlenecks"

"bottler"

"bottlers"

"bottles"

"bottlesful"

"bottling"

"bottom"

"bottomed"

"bottomer"

"bottomers"

"bottoming"

"bottomless"

"bottommost"

"bottoms"

"botulin"

"botulins"

"botulism"

"botulisms"

"boucle"

"boudoir"

"boudoirs"

"bouffant"

"bouffants"

"bouffe"

"bouffes"

"bougainvillaea"

"bougainvillaeas"

"bougainvillea"

"bough"

"boughed"

"boughs"

"bought"

"boughten"

"bouillabaisse"

"bouillon"

"bouillons"

"boulder"

"boulders"

"bouldery"

"boule"

"boules"

"boulevard"

"boulevards"

"boulimia"

"bounce"

"bounced"

"bouncer"

"bouncers"

"bounces"

"bouncier"

"bounciest"

"bouncily"

"bouncing"

"bouncingly"

"bouncy"

"bound"

"boundaries"

"boundary"

"bounded"

"bounden"

"bounder"

"bounders"

"bounding"

"boundless"

"boundlessly"

"boundlessness"

"bounds"

"bounteous"

"bounteously"

"bounteousness"

"bountied"

"bounties"

"bountiful"

"bountifully"

"bountifulness"

"bounty"

"bountyless"

"bouquet"

"bouquets"

"bourbon"

"bourbons"

"bourg"

"bourgeois"

"bourgeoisie"

"bourgeon"

"bourgeoned"

"bourgeons"

"bourgs"

"bourn"

"bourne"

"bournes"

"bourns"

"bourree"

"bourrees"

"bourse"

"bouse"

"boused"

"bouses"

"bousy"

"bout"

"boutique"

"boutiques"

"boutonniere"

"boutonnieres"

"bouts"

"bouzouki"

"bouzoukia"

"bouzoukis"

"bovid"

"bovine"

"bovinely"

"bovines"

"bovinity"

"bow"

"bowdlerism"

"bowdlerization"

"bowdlerizations"

"bowdlerize"

"bowdlerized"

"bowdlerizes"

"bowdlerizing"

"bowed"

"bowedness"

"bowel"

"boweled"

"boweling"

"bowelled"

"bowelling"

"bowels"

"bower"

"bowered"

"boweries"

"bowering"

"bowerlike"

"bowers"

"bowery"

"bowfin"

"bowfins"

"bowfront"

"bowhead"

"bowheads"

"bowie"

"bowing"

"bowingly"

"bowings"

"bowknot"

"bowknots"

"bowl"

"bowlder"

"bowlders"

"bowled"

"bowleg"

"bowlegged"

"bowlegs"

"bowler"

"bowlers"

"bowless"

"bowlful"

"bowlfuls"

"bowlike"

"bowline"

"bowlines"

"bowling"

"bowlings"

"bowls"

"bowman"

"bowmen"

"bows"

"bowse"

"bowsed"

"bowses"

"bowshot"

"bowshots"

"bowsprit"

"bowsprits"

"bowstring"

"bowstrings"

"bowwow"

"bowwows"

"bowyer"

"box"

"boxcar"

"boxcars"

"boxed"

"boxer"

"boxers"

"boxes"

"boxfish"

"boxful"

"boxfuls"

"boxier"

"boxiest"

"boxiness"

"boxing"

"boxings"

"boxlike"

"boxwood"

"boxwoods"

"boxy"

"boy"

"boycott"

"boycotted"

"boycotting"

"boycotts"

"boyfriend"

"boyfriends"

"boyhood"

"boyhoods"

"boyish"

"boyishly"

"boyishness"

"boyo"

"boyos"

"boys"

"boysenberries"

"boysenberry"

"bozo"

"bozos"

"BPM"

"bps"

"br"

"bra"

"brace"

"braced"

"bracelet"

"bracelets"

"bracer"

"bracero"

"braceros"

"bracers"

"braces"

"brachial"

"brachiate"

"brachiating"

"brachiation"

"brachium"

"brachycephalic"

"brachycephalies"

"brachycephalism"

"brachycephaly"

"brachydactylia"

"brachydactylous"

"brachydactyly"

"bracing"

"bracings"

"bracken"

"brackens"

"bracket"

"bracketed"

"bracketing"

"brackets"

"brackish"

"brackishness"

"bract"

"bracted"

"bractlets"

"bracts"

"brad"

"bradawls"

"bradded"

"bradding"

"brads"

"brae"

"braes"

"brag"

"braggadocio"

"braggadocios"

"braggart"

"braggarts"

"bragged"

"bragger"

"braggers"

"braggest"

"braggier"

"braggiest"

"bragging"

"braggy"

"brags"

"brahma"

"brahman"

"brahmanism"

"brahmanist"

"brahmanists"

"brahmans"

"brahmas"

"brahmin"

"brahminism"

"brahminist"

"brahminists"

"brahmins"

"brahms"

"braid"

"braided"

"braider"

"braiders"

"braiding"

"braidings"

"braids"

"brail"

"brailed"

"brailing"

"braille"

"brailled"

"brailles"

"braillewriter"

"brailling"

"brails"

"brain"

"Brain Booster"

"brain development"

"Brain massage"

"brain state"

"Brain trainer"

"brain-state"

"braincase"

"brainchild"

"brainchildren"

"brained"

"brainier"

"brainiest"

"brainily"

"braininess"

"braining"

"brainish"

"brainless"

"brainlessly"

"brainlessness"

"brainpan"

"brainpans"

"brainpower"

"brains"

"brainsick"

"brainstorm"

"brainstorming"

"brainstorms"

"brainteaser"

"brainteasers"

"brainwash"

"brainwashed"

"brainwasher"

"brainwashers"

"brainwashes"

"brainwashing"

"brainwave"

"brainwaves"

"brainy"

"braise"

"braised"

"braises"

"braising"

"braize"

"braizes"

"brake"

"brakeage"

"brakeages"

"braked"

"brakeless"

"brakeman"

"brakemen"

"brakes"

"brakier"

"braking"

"braky"

"braless"

"bramble"

"brambled"

"brambles"

"bramblier"

"brambliest"

"brambling"

"brambly"

"bran"

"branch"

"branched"

"branches"

"branchier"

"branchiest"

"branching"

"branchings"

"branchless"

"branchlet"

"branchlike"

"branchy"

"brand"

"brand name drugs"

"branded"

"brander"

"branders"

"brandied"

"brandies"

"branding"

"brandish"

"brandished"

"brandisher"

"brandishers"

"brandishes"

"brandishing"

"brands"

"brandy"

"brandying"

"brans"

"bras"

"brash"

"brasher"

"brashes"

"brashest"

"brashier"

"brashiest"

"brashly"

"brashness"

"brashy"

"brasiers"

"brasil"

"brasilia"

"brasils"

"brass"

"brassage"

"brassard"

"brassards"

"brasserie"

"brasseries"

"brasses"

"brassica"

"brassicas"

"brassie"

"brassier"

"brassiere"

"brassieres"

"brassies"

"brassiest"

"brassily"

"brassish"

"brassy"

"brat"

"brats"

"brattier"

"brattiest"

"brattiness"

"brattish"

"brattling"

"bratty"

"bratwurst"

"braunschweiger"

"bravado"

"bravadoes"

"bravados"

"brave"

"braved"

"bravely"

"braveness"

"braver"

"braveries"

"bravers"

"bravery"

"braves"

"bravest"

"braving"

"bravo"

"bravoed"

"bravoes"

"bravoing"

"bravos"

"bravura"

"bravuras"

"bravure"

"braw"

"brawl"

"brawled"

"brawler"

"brawlers"

"brawlier"

"brawliest"

"brawling"

"brawlingly"

"brawls"

"brawn"

"brawnier"

"brawniest"

"brawnily"

"brawniness"

"brawns"

"brawny"

"bray"

"brayed"

"brayer"

"brayers"

"braying"

"brays"

"braze"

"brazed"

"brazee"

"brazen"

"brazened"

"brazening"

"brazenly"

"brazenness"

"brazens"

"brazer"

"brazers"

"brazes"

"brazier"

"braziers"

"brazil"

"brazilian"

"brazilians"

"brazils"

"brazing"

"breach"

"breached"

"breacher"

"breachers"

"breaches"

"breaching"

"bread"

"breadbasket"

"breadbaskets"

"breadboard"

"breadboards"

"breaded"

"breadfruit"

"breadfruits"

"breading"

"breadless"

"breads"

"breadstuff"

"breadstuffs"

"breadth"

"breadths"

"breadwinner"

"breadwinners"

"breadwinning"

"break"

"breakable"

"breakables"

"breakage"

"breakages"

"breakaway"

"breakdown"

"breakdowns"

"breaker"

"breakers"

"breakfast"

"breakfasted"

"breakfasting"

"breakfasts"

"breakfront"

"breakfronts"

"breaking"

"breakings"

"breakneck"

"breakout"

"breakouts"

"breakpoint"

"breakpoints"

"breaks"

"breakthrough"

"breakthroughs"

"breakup"

"breakups"

"breakwater"

"breakwaters"

"bream"

"breams"

"breast"

"breastbone"

"breastbones"

"breasted"

"breastfeeding"

"breasting"

"breastplate"

"breastplates"

"breasts"

"breaststroke"

"breaststrokes"

"breastwork"

"breastworks"

"breath"

"breathable"

"breathe"

"breathed"

"breather"

"breathers"

"breathes"

"breathier"

"breathiest"

"breathing"

"breathless"

"breathlessly"

"breathlessness"

"breaths"

"breathtaking"

"breathtakingly"

"breathy"

"breccia"

"bred"

"brede"

"breech"

"breechcloth"

"breechcloths"

"breeched"

"breeches"

"breeching"

"breed"

"breeder"

"breeders"

"breeding"

"breedings"

"breeds"

"breeze"

"breezed"

"breezes"

"breezeway"

"breezeways"

"breezier"

"breeziest"

"breezily"

"breeziness"

"breezing"

"breezy"

"brent"

"brethren"

"breton"

"bretons"

"breve"

"breves"

"brevet"

"brevetcies"

"breveted"

"breveting"

"brevets"

"brevetted"

"brevetting"

"brevi"

"breviaries"

"breviary"

"breviate"

"brevier"

"brevities"

"brevity"

"brew"

"brewage"

"brewages"

"brewed"

"brewer"

"breweries"

"brewers"

"brewery"

"brewing"

"brewings"

"brews"

"brezhnev"

"brian"

"briar"

"briars"

"briary"

"bribable"

"bribe"

"bribeable"

"bribed"

"bribee"

"briber"

"briberies"

"bribers"

"bribery"

"bribes"

"bribing"

"brick"

"brickbat"

"brickbats"

"bricked"

"brickier"

"brickiest"

"bricking"

"bricklayer"

"bricklayers"

"bricklaying"

"brickle"

"bricks"

"bricktop"

"brickwork"

"bricky"

"brickyard"

"bridal"

"bridally"

"bridals"

"bride"

"bridegroom"

"bridegrooms"

"brides"

"bridesmaid"

"bridesmaids"

"bridewell"

"bridge"

"bridgeable"

"bridged"

"bridgehead"

"bridgeheads"

"bridgeport"

"bridges"

"bridgework"

"bridging"

"bridgings"

"bridle"

"bridled"

"bridler"

"bridlers"

"bridles"

"bridling"

"brie"

"brief"

"briefcase"

"briefcases"

"briefed"

"briefer"

"briefest"

"briefing"

"briefings"

"briefless"

"briefly"

"briefness"

"briefs"

"brier"

"briers"

"briery"

"bries"

"brig"

"brigade"

"brigaded"

"brigades"

"brigadier"

"brigading"

"brigand"

"brigandage"

"brigands"

"brigantine"

"brigantines"

"bright"

"brighten"

"brightened"

"brightener"

"brighteners"

"brightening"

"brightens"

"brighter"

"brightest"

"brightly"

"brightness"

"brights"

"brigs"

"brill"

"brilliance"

"brilliancies"

"brilliancy"

"brilliant"

"brilliantine"

"brilliantly"

"brilliants"

"brim"

"brimful"

"brimfull"

"brimless"

"brimmed"

"brimmer"

"brimmers"

"brimming"

"brims"

"brimstone"

"brin"

"brindle"

"brindled"

"brindles"

"brine"

"brined"

"briner"

"brines"

"bring"

"bringer"

"bringers"

"bringeth"

"bringing"

"brings"

"brinier"

"brinies"

"briniest"

"brininess"

"brining"

"brinish"

"brink"

"brinkmanship"

"brinks"

"briny"

"brio"

"brioche"

"brioches"

"briony"

"brios"

"briquet"

"briquets"

"briquette"

"briquetted"

"briquettes"

"brisbane"

"brisk"

"brisked"

"brisker"

"briskest"

"brisket"

"briskets"

"brisking"

"briskly"

"briskness"

"brisks"

"brisling"

"brislings"

"bristle"

"bristled"

"bristles"

"bristlier"

"bristliest"

"bristling"

"bristly"

"bristol"

"bristols"

"brit"

"britain"

"britannia"

"britannic"

"britannica"

"britches"

"briticism"

"british"

"britisher"

"britishers"

"briton"

"britons"

"brittle"

"brittled"

"brittleness"

"brittler"

"brittles"

"brittlest"

"brittling"

"bro"

"broach"

"broached"

"broacher"

"broachers"

"broaches"

"broaching"

"broad"

"broadax"

"broadaxe"

"broadaxes"

"broadband"

"broadcast"

"broadcasted"

"broadcaster"

"broadcasters"

"broadcasting"

"broadcastings"

"broadcasts"

"broadcloth"

"broaden"

"broadened"

"broadening"

"broadenings"

"broadens"

"broader"

"broadest"

"broadish"

"broadloom"

"broadlooms"

"broadly"

"broadness"

"broads"

"broadside"

"broadsides"

"broadsword"

"broadswords"

"broadtail"

"broadway"

"brocade"

"brocaded"

"brocades"

"brocading"

"broccoli"

"broccolis"

"brochette"

"brochettes"

"brochure"

"brochures"

"brock"

"brocket"

"brockets"

"brocks"

"brocoli"

"brogan"

"brogans"

"brogue"

"broguery"

"brogues"

"broguish"

"broider"

"broidered"

"broideries"

"broidering"

"broiders"

"broidery"

"broil"

"broiled"

"broiler"

"broilers"

"broiling"

"broils"

"brokage"

"brokages"

"broke"

"broken"

"brokenhearted"

"brokenly"

"brokenness"

"broker"

"brokerage"

"brokerages"

"brokerly"

"brokers"

"brollies"

"brolly"

"bromate"

"bromatology"

"bromide"

"bromides"

"bromidic"

"bromine"

"bromines"

"bromo"

"bromos"

"bronc"

"bronchi"

"bronchia"

"bronchial"

"bronchially"

"bronchitic"

"bronchitis"

"broncho"

"bronchodilator"

"bronchopneumonia"

"bronchopulmonary"

"bronchos"

"bronchoscope"

"bronchoscopy"

"bronchus"

"bronco"

"broncobuster"

"broncobusters"

"broncos"

"broncs"

"brontology"

"brontophobia"

"brontosaur"

"brontosaurs"

"brontosaurus"

"brontosauruses"

"bronx"

"bronze"

"bronzed"

"bronzer"

"bronzers"

"bronzes"

"bronzier"

"bronziest"

"bronzing"

"bronzings"

"bronzy"

"brooch"

"brooches"

"brood"

"brooded"

"brooder"

"brooders"

"broodier"

"broodiest"

"brooding"

"broods"

"broody"

"brook"

"brooked"

"brooking"

"brooklet"

"brooklets"

"brooklyn"

"brooks"

"broom"

"broomed"

"broomier"

"broomiest"

"brooming"

"brooms"

"broomstick"

"broomsticks"

"broomy"

"bros"

"broth"

"brothel"

"brothels"

"brother"

"brotherhood"

"brothering"

"brotherliness"

"brotherly"

"brothers"

"brothier"

"brothiest"

"broths"

"brothy"

"brougham"

"broughams"

"brought"

"brouhaha"

"brouhahas"

"brow"

"browbeat"

"browbeaten"

"browbeating"

"browbeats"

"browless"

"brown"

"browned"

"browner"

"brownest"

"brownie"

"brownier"

"brownies"

"browniest"

"browning"

"brownish"

"brownout"

"brownouts"

"browns"

"brownstone"

"brownstones"

"browny"

"brows"

"browse"

"browsed"

"browser"

"browsers"

"browses"

"browsing"

"bruce"

"brucellosis"

"bruin"

"bruins"

"bruise"

"bruised"

"bruiser"

"bruisers"

"bruises"

"bruising"

"bruit"

"bruited"

"bruiter"

"bruiters"

"bruiting"

"bruits"

"brunch"

"brunched"

"brunches"

"brunching"

"brunet"

"brunets"

"brunette"

"brunettes"

"brunswick"

"brunt"

"brunts"

"brush"

"brushed"

"brusher"

"brushers"

"brushes"

"brushfire"

"brushier"

"brushiest"

"brushing"

"brushoff"

"brushoffs"

"brushup"

"brushups"

"brushwood"

"brushy"

"brusk"

"brusker"

"bruskest"

"bruskly"

"bruskness"

"brusque"

"brusquely"

"brusqueness"

"brusquer"

"brusquest"

"brussels"

"brut"

"brutal"

"brutalities"

"brutality"

"brutalization"

"brutalize"

"brutalized"

"brutalizes"

"brutalizing"

"brutally"

"brutalness"

"brute"

"bruted"

"brutely"

"brutes"

"brutified"

"brutifies"

"brutify"

"brutifying"

"bruting"

"brutish"

"brutishly"

"brutishness"

"brutism"

"brutisms"

"bruxomania"

"bryan"

"bryology"

"bryony"

"bub"

"bubbies"

"bubble"

"bubbled"

"bubbler"

"bubblers"

"bubbles"

"bubbletop"

"bubbletops"

"bubblier"

"bubblies"

"bubbliest"

"bubbling"

"bubbly"

"bubby"

"bubo"

"buboes"

"bubonic"

"bubs"

"buccaneer"

"buccaneers"

"buchanan"

"bucharest"

"buchu"

"buck"

"buckaroo"

"buckaroos"

"buckbean"

"buckbeans"

"buckboard"

"buckboards"

"bucked"

"bucker"

"buckeroo"

"buckeroos"

"buckers"

"bucket"

"bucketed"

"bucketer"

"bucketful"

"bucketfuls"

"bucketing"

"buckets"

"buckeye"

"buckeyes"

"buckhound"

"buckhounds"

"bucking"

"buckish"

"buckishly"

"buckle"

"buckled"

"buckleless"

"buckler"

"bucklered"

"bucklers"

"buckles"

"buckling"

"bucko"

"buckoes"

"buckra"

"buckram"

"buckramed"

"buckrams"

"buckras"

"bucks"

"bucksaw"

"bucksaws"

"buckshot"

"buckshots"

"buckskin"

"buckskins"

"bucktail"

"bucktails"

"buckteeth"

"buckthorn"

"bucktooth"

"bucktoothed"

"buckwheat"

"buckwheats"

"bucolic"

"bucolically"

"bucolics"

"bud"

"budapest"

"budded"

"budder"

"budders"

"buddha"

"buddhism"

"buddhist"

"buddhists"

"buddies"

"budding"

"buddles"

"buddy"

"budge"

"budged"

"budger"

"budgerigar"

"budgerigars"

"budgers"

"budges"

"budget"

"budgetary"

"budgeted"

"budgeter"

"budgeters"

"budgeting"

"budgets"

"budgie"

"budgies"

"budging"

"budless"

"budlike"

"buds"

"buenas"

"buenos"

"buff"

"buffable"

"buffalo"

"buffaloed"

"buffaloes"

"buffaloing"

"buffalos"

"buffed"

"buffer"

"buffered"

"buffering"

"buffers"

"buffet"

"buffeted"

"buffeter"

"buffeters"

"buffeting"

"buffets"

"buffier"

"buffing"

"buffo"

"buffoon"

"buffoonery"

"buffoonish"

"buffoons"

"buffos"

"buffs"

"buffy"

"bufotoxin"

"bug"

"bugaboo"

"bugaboos"

"bugbane"

"bugbanes"

"bugbear"

"bugbearish"

"bugbears"

"bugeye"

"bugeyes"

"bugged"

"bugger"

"buggered"

"buggeries"

"buggering"

"buggers"

"buggery"

"buggier"

"buggies"

"buggiest"

"bugging"

"buggy"

"bughouse"

"bughouses"

"bugle"

"bugled"

"bugler"

"buglers"

"bugles"

"bugling"

"bugs"

"bugseeds"

"buick"

"buicks"

"build"

"builded"

"builder"

"builders"

"building"

"buildings"

"builds"

"buildup"

"buildups"

"built"

"bulb"

"bulbar"

"bulbed"

"bulbous"

"bulbs"

"bulbul"

"bulbuls"

"bulgaria"

"bulgarian"

"bulgarians"

"bulge"

"bulged"

"bulger"

"bulgers"

"bulges"

"bulgier"

"bulgiest"

"bulging"

"bulgur"

"bulgurs"

"bulgy"

"bulimia"

"bulimiac"

"bulimias"

"bulimic"

"bulk"

"bulkage"

"bulkages"

"bulked"

"bulkhead"

"bulkheads"

"bulkier"

"bulkiest"

"bulkily"

"bulkiness"

"bulking"

"bulks"

"bulky"

"bull"

"bulldog"

"bulldogged"

"bulldogging"

"bulldogs"

"bulldoze"

"bulldozed"

"bulldozer"

"bulldozers"

"bulldozes"

"bulldozing"

"bulled"

"bullet"

"bulleted"

"bulletin"

"bulleting"

"bulletins"

"bulletproof"

"bulletproofed"

"bulletproofing"

"bulletproofs"

"bullets"

"bullfight"

"bullfighter"

"bullfighters"

"bullfighting"

"bullfights"

"bullfinch"

"bullfinches"

"bullfrog"

"bullfrogs"

"bullhead"

"bullheaded"

"bullheadedness"

"bullheads"

"bullhorn"

"bullhorns"

"bullied"

"bullier"

"bullies"

"bulling"

"bullion"

"bullions"

"bullish"

"bullneck"

"bullnecks"

"bullnose"

"bullnoses"

"bullock"

"bullocks"

"bullpen"

"bullpens"

"bullring"

"bullrings"

"bullrush"

"bullrushes"

"bulls"

"bullshit"

"bullshits"

"bullweed"

"bullweeds"

"bullwhip"

"bullwhips"

"bully"

"bullyboy"

"bullyboys"

"bullying"

"bullyrag"

"bullyrags"

"bulrush"

"bulrushes"

"bulwark"

"bulwarked"

"bulwarking"

"bulwarks"

"bum"

"bumble"

"bumblebee"

"bumblebees"

"bumbled"

"bumbler"

"bumblers"

"bumbles"

"bumbling"

"bumblings"

"bumboat"

"bumboats"

"bumkin"

"bumkins"

"bummed"

"bummer"

"bummers"

"bummest"

"bumming"

"bump"

"bumped"

"bumper"

"bumpered"

"bumpering"

"bumpers"

"bumpier"

"bumpiest"

"bumpily"

"bumpiness"

"bumping"

"bumpkin"

"bumpkinish"

"bumpkins"

"bumps"

"bumptious"

"bumptiously"

"bumptiousness"

"bumpy"

"bums"

"bun"

"bunch"

"bunched"

"bunches"

"bunchier"

"bunchiest"

"bunchily"

"bunching"

"bunchy"

"bunco"

"buncoed"

"buncoing"

"buncombe"

"buncos"

"bund"

"bundle"

"bundled"

"bundler"

"bundlers"

"bundles"

"bundling"

"bundlings"

"bunds"

"bung"

"bungalow"

"bungalows"

"bunged"

"bunghole"

"bungholes"

"bunging"

"bungle"

"bungled"

"bungler"

"bunglers"

"bungles"

"bungling"

"bunglings"

"bungs"

"bunion"

"bunions"

"bunk"

"bunked"

"bunker"

"bunkerage"

"bunkered"

"bunkering"

"bunkers"

"bunkhouse"

"bunkhouses"

"bunking"

"bunkmate"

"bunkmates"

"bunko"

"bunkoed"

"bunkoing"

"bunkos"

"bunks"

"bunkum"

"bunkums"

"bunn"

"bunnies"

"bunns"

"bunny"

"buns"

"bunsen"

"bunt"

"bunted"

"bunter"

"bunters"

"bunting"

"buntings"

"bunts"

"bunyan"

"buoy"

"buoyage"

"buoyages"

"buoyance"

"buoyances"

"buoyancies"

"buoyancy"

"buoyant"

"buoyantly"

"buoyed"

"buoying"

"buoys"

"bur"

"burble"

"burbled"

"burbler"

"burblers"

"burbles"

"burblier"

"burbliest"

"burbling"

"burbly"

"burden"

"burdened"

"burdener"

"burdeners"

"burdening"

"burdens"

"burdensome"

"burdies"

"burdock"

"burdocks"

"bureau"

"bureaucracies"

"bureaucracy"

"bureaucrat"

"bureaucratic"

"bureaucratically"

"bureaucratism"

"bureaucratization"

"bureaucratize"

"bureaucratized"

"bureaucratizes"

"bureaucratizing"

"bureaucrats"

"bureaus"

"bureaux"

"burette"

"burettes"

"burg"

"burgee"

"burgees"

"burgeon"

"burgeoned"

"burgeoning"

"burgeons"

"burger"

"burgers"

"burgess"

"burgesses"

"burgh"

"burgher"

"burghers"

"burghs"

"burglar"

"burglaries"

"burglarious"

"burglariously"

"burglarize"

"burglarized"

"burglarizes"

"burglarizing"

"burglarproof"

"burglars"

"burglary"

"burgle"

"burgled"

"burgles"

"burgling"

"burgomaster"

"burgomasters"

"burgoo"

"burgoos"

"burgouts"

"burgs"

"burgundies"

"burgundy"

"burial"

"burials"

"buried"

"burier"

"buriers"

"buries"

"burin"

"burins"

"burke"

"burl"

"burlap"

"burlaps"

"burled"

"burler"

"burlesk"

"burlesks"

"burlesque"

"burlesqued"

"burlesques"

"burlesquing"

"burley"

"burleys"

"burlier"

"burliest"

"burlily"

"burliness"

"burling"

"burls"

"burly"

"burma"

"burmese"

"burn"

"burnable"

"burned"

"burner"

"burners"

"burnet"

"burnets"

"burnie"

"burnies"

"burning"

"burnings"

"burnish"

"burnished"

"burnisher"

"burnishers"

"burnishes"

"burnishing"

"burnoose"

"burnooses"

"burnouses"

"burnout"

"burnouts"

"burns"

"burns extra fats"

"burnt"

"burp"

"burped"

"burping"

"burps"

"burr"

"burred"

"burrer"

"burrers"

"burrier"

"burring"

"burro"

"burros"

"burroughs"

"burrow"

"burrowed"

"burrower"

"burrowers"

"burrowing"

"burrows"

"burrs"

"burry"

"burs"

"bursa"

"bursae"

"bursal"

"bursar"

"bursarial"

"bursaries"

"bursars"

"bursarship"

"bursary"

"bursas"

"burse"

"burseeds"

"burses"

"bursitis"

"bursitises"

"burst"

"bursted"

"burster"

"bursters"

"bursting"

"bursts"

"burthen"

"burthens"

"burton"

"burtons"

"burundi"

"burundians"

"burweed"

"burweeds"

"bury"

"burying"

"bus"

"busbies"

"busboy"

"busboys"

"busby"

"bused"

"buses"

"bush"

"bushed"

"bushel"

"busheled"

"busheler"

"bushelers"

"busheling"

"bushelled"

"bushels"

"busher"

"bushers"

"bushes"

"bushfire"

"bushfires"

"bushido"

"bushidos"

"bushier"

"bushiest"

"bushily"

"bushing"

"bushings"

"bushman"

"bushmaster"

"bushmasters"

"bushmen"

"bushtit"

"bushtits"

"bushwack"

"bushwhack"

"bushwhacked"

"bushwhacker"

"bushwhackers"

"bushwhacking"

"bushwhacks"

"bushy"

"busied"

"busier"

"busies"

"busiest"

"busily"

"business"

"businesses"

"businesslike"

"businessman"

"businessmen"

"businesswoman"

"businesswomen"

"busing"

"busings"

"buskin"

"buskined"

"buskins"

"busman"

"busmen"

"buss"

"bussed"

"busses"

"bussing"

"bust"

"bustard"

"bustards"

"busted"

"buster"

"busters"

"bustier"

"bustiest"

"busting"

"bustle"

"bustled"

"bustler"

"bustlers"

"bustles"

"bustling"

"busts"

"busty"

"busy"

"busybodies"

"busybody"

"busying"

"busyness"

"busywork"

"busyworks"

"but"

"butane"

"butanes"

"butch"

"butcher"

"butchered"

"butcheries"

"butchering"

"butchers"

"butchery"

"butches"

"butler"

"butleries"

"butlers"

"butlery"

"buts"

"butt"

"butte"

"butted"

"butter"

"buttercup"

"buttercups"

"buttered"

"butterfat"

"butterfingered"

"butterfingers"

"butterfish"

"butterfishes"

"butterflies"

"butterfly"

"butterier"

"butteries"

"butteriest"

"buttering"

"buttermilk"

"butternut"

"butternuts"

"butters"

"butterscotch"

"buttery"

"buttes"

"butting"

"buttock"

"buttocks"

"button"

"buttoned"

"buttoner"

"buttoners"

"buttonhole"

"buttonholed"

"buttonholer"

"buttonholes"

"buttonholing"

"buttonhook"

"buttoning"

"buttons"

"buttony"

"buttress"

"buttressed"

"buttresses"

"buttressing"

"butts"

"butty"

"butyl"

"butyls"

"buxom"

"buxomer"

"buxomest"

"buxomly"

"buxomness"

"buy"

"buyable"

"buyer"

"buyers"

"buying"

"buying quality cannabis"

"buys"

"buzz"

"buzzard"

"buzzards"

"buzzed"

"buzzer"

"buzzers"

"buzzes"

"buzzing"

"buzzword"

"buzzwords"

"bwana"

"bwanas"

"by"

"bye"

"byelorussia"

"byelorussian"

"byelorussians"

"byes"

"bygone"

"bygones"

"bylaw"

"bylaws"

"byline"

"bylined"

"byliner"

"byliners"

"bylines"

"bylining"

"bypass"

"bypassed"

"bypasses"

"bypassing"

"bypath"

"bypaths"

"byplay"

"byplays"

"byproduct"

"byproducts"

"byre"

"byres"

"byroad"

"byroads"

"byron"

"byronic"

"bystander"

"bystanders"

"bystreet"

"bystreets"

"byte"

"bytes"

"byway"

"byways"

"byword"

"bywords"

"byzantine"

"byzantium"

"ca"

"cab"

"cabal"

"cabala"

"cabalas"

"cabalism"

"cabalist"

"cabalistic"

"cabalists"

"caballed"

"caballero"

"caballeros"

"caballing"

"cabals"

"cabana"

"cabanas"

"cabaret"

"cabarets"

"cabbage"

"cabbaged"

"cabbages"

"cabbaging"

"cabbala"

"cabbalah"

"cabbalahs"

"cabbalas"

"cabbie"

"cabbies"

"cabby"

"cabdriver"

"caber"

"cabers"

"cabin"

"cabined"

"cabinet"

"cabinetmaker"

"cabinetmakers"

"cabinetmaking"

"cabinets"

"cabinetwork"

"cabining"

"cabins"

"cable"

"cabled"

"cablegram"

"cablegrams"

"cables"

"cablets"

"cableway"

"cableways"

"cabling"

"cabman"

"cabmen"

"cabob"

"cabobs"

"cabochon"

"cabochons"

"caboodle"

"caboodles"

"caboose"

"cabooses"

"cabot"

"cabriolet"

"cabriolets"

"cabs"

"cabstand"

"cabstands"

"cacao"

"cacaos"

"cacciatore"

"cachalot"

"cachalots"

"cache"

"cached"

"cachepot"

"cachepots"

"caches"

"cachet"

"cacheted"

"cacheting"

"cachets"

"caching"

"caciques"

"cackle"

"cackled"

"cackler"

"cacklers"

"cackles"

"cackling"

"cacodemomania"

"cacodemonia"

"cacogenics"

"cacophonies"

"cacophonous"

"cacophonously"

"cacophony"

"cacti"

"cactoid"

"cactus"

"cactuses"

"cad"

"cadaver"

"cadaveric"

"cadaverous"

"cadaverously"

"cadavers"

"caddie"

"caddied"

"caddies"

"caddis"

"caddises"

"caddish"

"caddishly"

"caddishness"

"caddy"

"caddying"

"cadence"

"cadenced"

"cadences"

"cadencies"

"cadencing"

"cadency"

"cadent"

"cadenza"

"cadenzas"

"cades"

"cadet"

"cadets"

"cadetship"

"cadette"

"cadettes"

"cadge"

"cadged"

"cadger"

"cadgers"

"cadges"

"cadging"

"cadgy"

"cadillac"

"cadillacs"

"cadis"

"cadmic"

"cadmium"

"cadmiums"

"cadre"

"cadres"

"cads"

"caducei"

"caduceus"

"caduciaries"

"caecum"

"caesar"

"caesarean"

"caesareans"

"caesarists"

"caesium"

"caesura"

"caesurae"

"caesural"

"caesuras"

"caesuric"

"cafe"

"cafes"

"cafeteria"

"cafeterias"

"caffein"

"caffeine"

"caffeines"

"caffeinic"

"caffeins"

"caftan"

"caftans"

"cage"

"caged"

"cageling"

"cagelings"

"cager"

"cagers"

"cages"

"cagey"

"cageyness"

"cagier"

"cagiest"

"cagily"

"caginess"

"caging"

"cagy"

"cahoot"

"cahoots"

"caiman"

"caimans"

"cains"

"cairn"

"cairned"

"cairns"

"cairo"

"caisson"

"caissons"

"caitiff"

"caitiffs"

"cajaput"

"cajaputs"

"cajole"

"cajoled"

"cajolement"

"cajolements"

"cajoler"

"cajoleries"

"cajolers"

"cajolery"

"cajoles"

"cajoling"

"cajolingly"

"cajon"

"cajun"

"cajuns"

"cake"

"caked"

"cakes"

"cakewalk"

"cakewalked"

"cakewalker"

"cakewalks"

"cakier"

"cakiest"

"caking"

"caky"

"cal"

"calabash"

"calabashes"

"calaboose"

"calabooses"

"caladium"

"caladiums"

"calamar"

"calamaries"

"calamars"

"calamary"

"calamine"

"calamines"

"calamint"

"calamities"

"calamitous"

"calamitously"

"calamitousness"

"calamity"

"calc"

"calcalute"

"calcareous"

"calcareously"

"calcareousness"

"calcaria"

"calcic"

"calciferous"

"calcific"

"calcification"

"calcified"

"calcifies"

"calcify"

"calcifying"

"calcimine"

"calcimined"

"calcimines"

"calcimining"

"calcination"

"calcine"

"calcined"

"calcines"

"calcining"

"calcite"

"calcites"

"calcitic"

"calcium"

"calciums"

"calcspar"

"calculabilities"

"calculability"

"calculable"

"calculableness"

"calculably"

"calculate"

"calculated"

"calculatedly"

"calculates"

"calculating"

"calculatingly"

"calculation"

"calculational"

"calculations"

"calculative"

"calculator"

"calculators"

"calculi"

"calculous"

"calculus"

"calculuses"

"calcutta"

"caldera"

"calderas"

"calderon"

"caldron"

"caldrons"

"calefacient"

"calendal"

"calendar"

"calendar method"

"calendared"

"calendaring"

"calendars"

"calender"

"calendered"

"calendering"

"calenders"

"calends"

"calendula"

"calendulas"

"calf"

"calfs"

"calfskin"

"calfskins"

"calgary"

"caliber"

"calibers"

"calibrate"

"calibrated"

"calibrates"

"calibrating"

"calibration"

"calibrations"

"calibrator"

"calibrators"

"calibre"

"calibred"

"calibres"

"calico"

"calicoes"

"calicos"

"calif"

"califate"

"california"

"californian"

"californians"

"californium"

"califs"

"caliology"

"caliper"

"calipered"

"calipering"

"calipers"

"caliph"

"caliphal"

"caliphate"

"caliphates"

"caliphs"

"calisthenic"

"calisthenics"

"calix"

"calk"

"calked"

"calker"

"calkers"

"calking"

"calks"

"call"

"calla"

"callable"

"callas"

"callback"

"callbacks"

"callboy"

"callboys"

"called"

"caller"

"callers"

"callets"

"calli"

"calligrapher"

"calligraphers"

"calligraphic"

"calligraphy"

"calling"

"callings"

"calliope"

"calliopes"

"calliper"

"callosities"

"callosity"

"callous"

"calloused"

"callouses"

"callousing"

"callously"

"callousness"

"callow"

"callower"

"callowest"

"callowness"

"calls"

"callus"

"callused"

"calluses"

"callusing"

"calm"

"calmant"

"calmative"

"calmed"

"calmer"

"calmest"

"calming"

"Calming Music"

"calmingly"

"calmly"

"calmness"

"calms"

"calomel"

"calomels"

"calor"

"caloric"

"calorically"

"calorics"

"calorie"

"calorie counter"

"calories"

"calories export"

"calorific"

"calorifics"

"calorimeter"

"calorimeters"

"calorimetric"

"calorimetrically"

"calorimetry"

"calory"

"calotte"

"calpack"

"calpacs"

"calthrops"

"caltrap"

"caltraps"

"caltrop"

"caltrops"

"calumet"

"calumets"

"calumniate"

"calumniated"

"calumniates"

"calumniating"

"calumniation"

"calumniations"

"calumniator"

"calumniators"

"calumnies"

"calumnious"

"calumniously"

"calumny"

"calvary"

"calve"

"calved"

"calves"

"calvin"

"calving"

"calvinism"

"calvinist"

"calvinistic"

"calvinists"

"calvities"

"calx"

"calxes"

"calyces"

"calycle"

"calypso"

"calypsoes"

"calypsos"

"calyx"

"calyxes"

"cam"

"camaraderie"

"camass"

"camber"

"cambered"

"cambering"

"cambers"

"cambia"

"cambial"

"cambism"

"cambist"

"cambistry"

"cambium"

"cambiums"

"cambodia"

"cambodian"

"cambodians"

"cambrian"

"cambric"

"cambrics"

"cambridge"

"camden"

"came"

"camel"

"camelback"

"cameleer"

"cameleers"

"camelia"

"camelias"

"camellia"

"camellias"

"camelopard"

"camelopards"

"camels"

"camembert"

"cameo"

"cameoed"

"cameoing"

"cameos"

"camera"

"cameral"

"cameralism"

"cameralist"

"cameralistic"

"cameraman"

"cameramen"

"cameras"

"cameroon"

"cameroonian"

"cameroonians"

"camisole"

"camisoles"

"camomile"

"camomiles"

"camouflage"

"camouflaged"

"camouflager"

"camouflagers"

"camouflages"

"camouflaging"

"camp"

"campagne"

"campaign"

"campaigned"

"campaigner"

"campaigners"

"campaigning"

"campaigns"

"campanile"

"campaniles"

"campanili"

"campanologist"

"campanologists"

"campanology"

"campbell"

"campcraft"

"camped"

"camper"

"campers"

"campfire"

"campfires"

"campground"

"campgrounds"

"camphor"

"camphorate"

"camphorated"

"camphorates"

"camphorating"

"camphoric"

"camphors"

"campi"

"campier"

"campiest"

"campily"

"campiness"

"camping"

"campings"

"campo"

"camporee"

"camporees"

"campos"

"camps"

"campsite"

"campsites"

"campstool"

"campstools"

"campus"

"campuses"

"campy"

"cams"

"camshaft"

"camshafts"

"can"

"canaan"

"canaanite"

"canaanites"

"canada"

"canadian"

"canadianisms"

"canadians"

"canaille"

"canal"

"canalboat"

"canaled"

"canaling"

"canalise"

"canalization"

"canalizations"

"canalize"

"canalized"

"canalizes"

"canalizing"

"canalled"

"canaller"

"canallers"

"canalling"

"canals"

"canape"

"canapes"

"canard"

"canards"

"canaries"

"canary"

"canasta"

"canastas"

"canberra"

"cancan"

"cancans"

"cancel"

"cancelable"

"canceled"

"canceler"

"cancelers"

"canceling"

"cancellation"

"cancellations"

"cancelled"

"canceller"

"cancelling"

"cancels"

"cancer"

"cancerophobia"

"cancerous"

"cancerously"

"cancers"

"candela"

"candelabra"

"candelabrum"

"candelabrums"

"candescence"

"candescent"

"candid"

"candidacies"

"candidacy"

"candidate"

"candidates"

"candidature"

"candidatures"

"candide"

"candider"

"candidest"

"candidly"

"candidness"

"candidnesses"

"candids"

"candied"

"candies"

"candle"

"candled"

"candlelight"

"candlepin"

"candlepins"

"candlepower"

"candler"

"candlers"

"candles"

"candlestick"

"candlesticks"

"candlewick"

"candlewicks"

"candling"

"candor"

"candors"

"candour"

"candours"

"candy"

"candying"

"cane"

"canebrake"

"canebrakes"

"caned"

"caner"

"caners"

"canes"

"caneware"

"canewares"

"canfield"

"canfuls"

"cangues"

"canine"

"canines"

"caning"

"caninity"

"canister"

"canisters"

"canker"

"cankered"

"cankering"

"cankerous"

"cankers"

"cankerworm"

"cankerworms"

"canna"

"cannabic"

"cannabin"

"cannabinoids"

"cannabinol"

"cannabis"

"cannabises"

"cannabism"

"cannalling"

"cannas"

"canned"

"cannel"

"cannelon"

"canner"

"canneries"

"canners"

"cannery"

"cannibal"

"cannibalism"

"cannibalistic"

"cannibalization"

"cannibalize"

"cannibalized"

"cannibalizes"

"cannibalizing"

"cannibals"

"cannie"

"cannier"

"canniest"

"cannily"

"canniness"

"canning"

"cannings"

"cannon"

"cannonade"

"cannonaded"

"cannonades"

"cannonading"

"cannonball"

"cannonballed"

"cannonballing"

"cannonballs"

"cannoned"

"cannoneer"

"cannoneers"

"cannoning"

"cannonism"

"cannonry"

"cannons"

"cannot"

"cannula"

"cannulae"

"cannulas"

"canny"

"canoe"

"canoed"

"canoeing"

"canoeist"

"canoeists"

"canoes"

"canon"

"canonic"

"canonical"

"canonically"

"canonicals"

"canonicity"

"canonise"

"canonist"

"canonistic"

"canonists"

"canonization"

"canonizations"

"canonize"

"canonized"

"canonizes"

"canonizing"

"canonry"

"canons"

"canopied"

"canopies"

"canopy"

"canopying"

"cans"

"cansful"

"canst"

"cant"

"cantabile"

"cantaloupe"

"cantaloupes"

"cantankerous"

"cantankerously"

"cantankerousness"

"cantata"

"cantatas"

"canted"

"canteen"

"canteens"

"canter"

"canterbury"

"cantered"

"cantering"

"canters"

"canthal"

"cantharides"

"cantharis"

"canthi"

"canthus"

"canticle"

"canticles"

"cantilever"

"cantilevered"

"cantilevering"

"cantilevers"

"cantina"

"cantinas"

"canting"

"cantingly"

"cantle"

"cantles"

"canto"

"canton"

"cantonal"

"cantoned"

"cantonese"

"cantoning"

"cantonment"

"cantonments"

"cantons"

"cantor"

"cantors"

"cantos"

"cantrap"

"cantraps"

"cantrip"

"cantrips"

"cants"

"canty"

"canvas"

"canvasback"

"canvasbacks"

"canvased"

"canvaser"

"canvases"

"canvaslike"

"canvass"

"canvassed"

"canvasser"

"canvassers"

"canvasses"

"canvassing"

"canyon"

"canyons"

"canzona"

"canzonas"

"canzone"

"canzones"

"canzonet"

"canzoni"

"caoutchouc"

"cap"

"capabilities"

"capability"

"capable"

"capableness"

"capabler"

"capablest"

"capably"

"capacious"

"capaciously"

"capaciousness"

"capacitance"

"capacitances"

"capacitate"

"capacitated"

"capacitates"

"capacitating"

"capacitation"

"capacitations"

"capacities"

"capacitive"

"capacitively"

"capacitor"

"capacitors"

"capacity"

"caparison"

"caparisoned"

"caparisoning"

"caparisons"

"cape"

"caped"

"capelan"

"capelet"

"capelets"

"caper"

"capered"

"caperer"

"caperers"

"capering"

"capers"

"capes"

"capeskin"

"capetown"

"capework"

"capful"

"capfuls"

"capillaries"

"capillarity"

"capillary"

"capita"

"capital"

"capitalism"

"capitalist"

"capitalistic"

"capitalistically"

"capitalists"

"capitalization"

"capitalizations"

"capitalize"

"capitalized"

"capitalizer"

"capitalizers"

"capitalizes"

"capitalizing"

"capitally"

"capitals"

"capitate"

"capitation"

"capitations"

"capitol"

"capitols"

"capitulary"

"capitulate"

"capitulated"

"capitulates"

"capitulating"

"capitulation"

"capitulations"

"capitulator"

"capitulatory"

"capless"

"capmaker"

"capmakers"

"capon"

"capone"

"caponization"

"caponize"

"caponized"

"caponizes"

"caponizing"

"capons"

"capos"

"capote"

"capotes"

"capped"

"cappella"

"capper"

"cappers"

"capping"

"cappings"

"cappy"

"capric"

"capriccio"

"capriccios"

"caprice"

"caprices"

"capricious"

"capriciously"

"capriciousness"

"capricorn"

"capricorns"

"caprine"

"capriole"

"caprioles"

"caps"

"capsicum"

"capsicums"

"capsize"

"capsized"

"capsizes"

"capsizing"

"capstan"

"capstans"

"capstone"

"capstones"

"capsular"

"capsulate"

"capsulation"

"capsule"

"capsuled"

"capsules"

"capsuling"

"captain"

"captaincies"

"captaincy"

"captained"

"captaining"

"captains"

"captainship"

"captainships"

"captans"

"caption"

"captioned"

"captioning"

"captions"

"captious"

"captiously"

"captiousness"

"captivate"

"captivated"

"captivates"

"captivating"

"captivation"

"captivator"

"captivators"

"captive"

"captives"

"captivities"

"captivity"

"captor"

"captors"

"captress"

"capture"

"captured"

"capturer"

"capturers"

"captures"

"capturing"

"capuchin"

"capuchins"

"caput"

"capybara"

"capybaras"

"car"

"carabao"

"carabaos"

"carabineer"

"caracal"

"caracals"

"caracas"

"caracol"

"caracole"

"caracoles"

"caracols"

"caracul"

"caraculs"

"carafe"

"carafes"

"carageen"

"caramel"

"caramelize"

"caramelized"

"caramelizes"

"caramelizing"

"caramels"

"carapace"

"carapaces"

"carat"

"carate"

"carats"

"caravan"

"caravaning"

"caravanned"

"caravans"

"caravansaries"

"caravansary"

"caravel"

"caravels"

"caraway"

"caraways"

"carbarn"

"carbarns"

"carbide"

"carbides"

"carbine"

"carbineer"

"carbineers"

"carbines"

"carbo"

"carbohydrate"

"carbohydrates"

"carbolated"

"carbolic"

"carbon"

"carbonaceous"

"carbonate"

"carbonated"

"carbonates"

"carbonating"

"carbonation"

"carbonator"

"carbonators"

"carbondale"

"carbonic"

"carboniferous"

"carbonization"

"carbonize"

"carbonized"

"carbonizing"

"carbonless"

"carbons"

"carboras"

"carborundum"

"carboxyl"

"carboy"

"carboyed"

"carboys"

"carbuncle"

"carbuncles"

"carbuncular"

"carburetor"

"carburetors"

"carburets"

"carburization"

"carburize"

"carburized"

"carburizes"

"carburizing"

"carcase"

"carcases"

"carcass"

"carcasses"

"carcinogen"

"carcinogeneses"

"carcinogenesis"

"carcinogenic"

"carcinogenicity"

"carcinogens"

"carcinology"

"carcinoma"

"carcinomas"

"carcinomata"

"carcinomatous"

"card"

"cardamom"

"cardamoms"

"cardamon"

"cardamons"

"cardamum"

"cardamums"

"cardboard"

"cardcase"

"cardcases"

"carded"

"carder"

"carders"

"cardholder"

"cardholders"

"cardia"

"cardiac"

"cardiac dysrhythmias"

"cardiacs"

"cardias"

"cardiectomy"

"cardigan"

"cardigans"

"cardinal"

"cardinalate"

"cardinalates"

"cardinalities"

"cardinality"

"cardinally"

"cardinals"

"carding"

"cardings"

"Cardio"

"cardio fitness"

"cardiogram"

"cardiograms"

"cardiograph"

"cardiographer"

"cardiographic"

"cardiographies"

"cardiographs"

"cardiography"

"cardioid"

"cardioids"

"cardiologic"

"cardiological"

"cardiologies"

"cardiologist"

"cardiologists"

"cardiology"

"cardiometer"

"cardiometry"

"cardiopulmonary"

"cardioscope"

"CardioSource"

"cardiotherapies"

"cardiotherapy"

"CardioTrainer"

"cardiovascular"

"cardiovascular care"

"cardiovascular disease"

"Cardiovascular System"

"cardoon"

"cardoons"

"cardroom"

"cards"

"cardsharp"

"cardsharper"

"cardsharps"

"care"

"care-givers"

"cared"

"careen"

"careened"

"careener"

"careeners"

"careening"

"careens"

"career"

"careered"

"careerer"

"careerers"

"careering"

"careers"

"carefree"

"careful"

"carefuller"

"carefully"

"carefulness"

"careless"

"carelessly"

"carelessness"

"carer"

"carers"

"cares"

"caress"

"caressed"

"caresser"

"caressers"

"caresses"

"caressing"

"caret"

"caretaker"

"caretakers"

"caretaking"

"carets"

"careworn"

"carfare"

"carfares"

"carful"

"carfuls"

"cargo"

"cargoes"

"cargos"

"carhop"

"carhops"

"caribbean"

"caribes"

"caribou"

"caribous"

"caricature"

"caricatured"

"caricatures"

"caricaturing"

"caricaturist"

"caricaturists"

"caricology"

"caries"

"carillon"

"carillonneur"

"carillonneurs"

"carillons"

"carina"

"carinae"

"carinas"

"caring"

"carioca"

"cariocas"

"cariole"

"carious"

"carl"

"carless"

"carlo"

"carload"

"carloads"

"carlot"

"carmaker"

"carmakers"

"carman"

"carmen"

"carminative"

"carminatives"

"carmine"

"carmines"

"carnage"

"carnages"

"carnal"

"carnalities"

"carnality"

"carnally"

"carnation"

"carnations"

"carnauba"

"carnaubas"

"carne"

"carnegie"

"carnelian"

"carnelians"

"carney"

"carneys"

"carnie"

"carnies"

"carnify"

"carnifying"

"carnival"

"carnivals"

"carnivore"

"carnivores"

"carnivorous"

"carnivorously"

"carnivorousness"

"carny"

"carob"

"carobs"

"carol"

"caroled"

"caroler"

"carolers"

"carolina"

"carolinas"

"caroling"

"carolinian"

"carolinians"

"carolled"

"caroller"

"carollers"

"carolling"

"carols"

"carolyn"

"carom"

"caromed"

"caroming"

"caroms"

"carotene"

"carotenes"

"carotid"

"carotidal"

"carotids"

"carotin"

"carotins"

"carousal"

"carousals"

"carouse"

"caroused"

"carousel"

"carousels"

"carouser"

"carousers"

"carouses"

"carousing"

"carousingly"

"carp"

"carpal"

"carpals"

"carpe"

"carped"

"carpel"

"carpels"

"carpenter"

"carpenters"

"carpentry"

"carper"

"carpers"

"carpet"

"carpetbag"

"carpetbagged"

"carpetbagger"

"carpetbaggers"

"carpetbaggery"

"carpetbagging"

"carpetbags"

"carpeted"

"carpeting"

"carpets"

"carpi"

"carping"

"carpings"

"carpology"

"carport"

"carports"

"carps"

"carpus"

"carracks"

"carrageen"

"carrageenan"

"carrageenin"

"carrel"

"carrell"

"carrells"

"carrels"

"carriage"

"carriageable"

"carriages"

"carriageway"

"carried"

"carrier"

"carriers"

"carries"

"carrion"

"carrions"

"carroll"

"carrom"

"carromed"

"carroming"

"carroms"

"carrot"

"carrotier"

"carrotiest"

"carrots"

"carroty"

"carrousel"

"carrousels"

"carry"

"carryall"

"carryalls"

"carrying"

"carryings"

"carryon"

"carryons"

"carryout"

"carryouts"

"carryover"

"carryovers"

"cars"

"carsick"

"carsickness"

"carson"

"cart"

"cartable"

"cartage"

"cartages"

"carte"

"carted"

"cartel"

"cartels"

"carter"

"carters"

"cartes"

"cartesian"

"cartilage"

"cartilages"

"cartilaginous"

"carting"

"cartload"

"cartloads"

"cartographer"

"cartographers"

"cartographic"

"cartographies"

"cartography"

"cartomancies"

"cartomancy"

"carton"

"cartoned"

"cartoning"

"cartons"

"cartoon"

"cartooned"

"cartooning"

"cartoonist"

"cartoonists"

"cartoons"

"cartop"

"cartophily"

"cartridge"

"cartridges"

"carts"

"cartway"

"cartwheel"

"cartwheels"

"carve"

"carved"

"carven"

"carver"

"carvers"

"carves"

"carving"

"carvings"

"carwash"

"carwashes"

"caryatid"

"caryatides"

"caryatids"

"casa"

"casaba"

"casabas"

"casablanca"

"casanova"

"casas"

"casava"

"casavas"

"casbah"

"cascabel"

"cascade"

"cascaded"

"cascades"

"cascading"

"cascara"

"cascaras"

"case"

"casebook"

"casebooks"

"cased"

"caseharden"

"casehardened"

"casehardening"

"casehardens"

"casein"

"caseins"

"caseload"

"caseloads"

"casement"

"casements"

"cases"

"casette"

"casettes"

"casework"

"caseworker"

"caseworkers"

"caseworks"

"cash"

"cashable"

"cashbook"

"cashbooks"

"cashbox"

"cashboxes"

"cashed"

"casher"

"cashers"

"cashes"

"cashew"

"cashews"

"cashier"

"cashiered"

"cashiering"

"cashiers"

"cashing"

"cashless"

"cashmere"

"cashmeres"

"cashoo"

"cashoos"

"casing"

"casings"

"casino"

"casinos"

"cask"

"casked"

"casket"

"casketed"

"casketing"

"caskets"

"casking"

"casks"

"casper"

"caspian"

"casque"

"casqued"

"casques"

"cassaba"

"cassabas"

"cassandra"

"cassandras"

"cassava"

"cassavas"

"casserole"

"casseroles"

"cassette"

"cassettes"

"cassia"

"cassias"

"cassino"

"cassinos"

"cassis"

"cassiterite"

"cassock"

"cassocks"

"cassowaries"

"cassowary"

"cast"

"castanet"

"castanets"

"castaway"

"castaways"

"caste"

"casted"

"casteism"

"casteisms"

"casteless"

"castellan"

"castellans"

"castellated"

"caster"

"casters"

"castes"

"castigate"

"castigated"

"castigates"

"castigating"

"castigation"

"castigations"

"castigator"

"castigators"

"castigatory"

"castile"

"casting"

"castings"

"castle"

"castled"

"castles"

"castling"

"castoff"

"castoffs"

"castor"

"castors"

"castrametation"

"castrate"

"castrated"

"castrates"

"castrati"

"castrating"

"castration"

"castrations"

"castrato"

"castrator"

"castrators"

"castro"

"casts"

"casual"

"casually"

"casualness"

"casuals"

"casualties"

"casualty"

"casuist"

"casuistic"

"casuistical"

"casuistries"

"casuistry"

"casuists"

"casus"

"cat"

"catabolic"

"catabolically"

"catabolism"

"catabolize"

"catabolized"

"catabolizing"

"cataclysm"

"cataclysmal"

"cataclysmic"

"cataclysms"

"catacomb"

"catacombs"

"catacoustics"

"catafalque"

"catafalques"

"catagories"

"catalactics"

"catalepsies"

"catalepsy"

"cataleptic"

"cataleptically"

"cataleptics"

"cataleptoid"

"catalog"

"cataloged"

"cataloger"

"catalogers"

"cataloging"

"catalogs"

"catalogue"

"catalogued"

"cataloguer"

"catalogues"

"cataloguing"

"catalos"

"catalpa"

"catalpas"

"catalyses"

"catalysis"

"catalyst"

"catalysts"

"catalytic"

"catalytically"

"catalyze"

"catalyzed"

"catalyzer"

"catalyzers"

"catalyzes"

"catalyzing"

"catamaran"

"catamarans"

"catamite"

"catamites"

"catamount"

"catamounts"

"catapedamania"

"catapult"

"catapulted"

"catapulting"

"catapults"

"cataract"

"cataracts"

"catarrh"

"catarrhal"

"catarrhally"

"catarrhous"

"catarrhs"

"catastrophe"

"catastrophes"

"catastrophic"

"catastrophical"

"catastrophically"

"catatonia"

"catatonias"

"catatonic"

"catatonics"

"catatony"

"catawba"

"catawbas"

"catbird"

"catbirds"

"catboat"

"catboats"

"catcall"

"catcalled"

"catcalling"

"catcalls"

"catch"

"catchall"

"catchalls"

"catcher"

"catchers"

"catches"

"catchier"

"catchiest"

"catching"

"catchment"

"catchments"

"catchpenny"

"catchup"

"catchups"

"catchword"

"catchwords"

"catchy"

"catechectics"

"catechism"

"catechisms"

"catechist"

"catechists"

"catechize"

"catechized"

"catechizes"

"catechizing"

"catechumen"

"catechumens"

"categoric"

"categorical"

"categorically"

"categoricalness"

"categories"

"categorization"

"categorizations"

"categorize"

"categorized"

"categorizer"

"categorizers"

"categorizes"

"categorizing"

"category"

"catenaries"

"catenary"

"catenas"

"catenating"

"cater"

"catered"

"caterer"

"caterers"

"cateress"

"cateresses"

"catering"

"caterpillar"

"caterpillars"

"caters"

"caterwaul"

"caterwauled"

"caterwauling"

"caterwauls"

"cates"

"catfish"

"catfishes"

"catgut"

"catguts"

"catharine"

"catharses"

"catharsis"

"cathartic"

"cathartically"

"cathartics"

"cathect"

"cathects"

"cathedra"

"cathedral"

"cathedrals"

"catherine"

"catheter"

"catheterize"

"catheterized"

"catheterizes"

"catheterizing"

"catheters"

"cathetometer"

"cathexes"

"cathexis"

"cathisophobia"

"cathode"

"cathodes"

"cathodic"

"catholic"

"catholically"

"catholicism"

"catholicity"

"catholics"

"cathouse"

"cathouses"

"cathy"

"cation"

"cations"

"catkin"

"catkins"

"catlike"

"catling"

"catmint"

"catmints"

"catnap"

"catnaper"

"catnapers"

"catnapped"

"catnapping"

"catnaps"

"catnip"

"catnips"

"cats"

"catskill"

"catspaw"

"catspaws"

"catsup"

"catsups"

"cattail"

"cattails"

"catted"

"cattier"

"catties"

"cattiest"

"cattily"

"cattiness"

"catting"

"cattish"

"cattle"

"cattleman"

"cattlemen"

"catty"

"catwalk"

"catwalks"

"caucasian"

"caucasians"

"caucasoid"

"caucasoids"

"caucasus"

"caucus"

"caucused"

"caucuses"

"caucusing"

"caucussed"

"caucussing"

"caudal"

"caudally"

"caudate"

"caudated"

"caudexes"

"caudices"

"caudillo"

"caudillos"

"caught"

"caul"

"cauldron"

"cauldrons"

"cauliflower"

"cauliflowers"

"caulk"

"caulked"

"caulker"

"caulkers"

"caulking"

"caulkings"

"caulks"

"cauls"

"causable"

"causal"

"causalities"

"causality"

"causally"

"causals"

"causation"

"causative"

"cause"

"caused"

"causeless"

"causelessly"

"causer"

"causerie"

"causeries"

"causers"

"causes"

"causeway"

"causewayed"

"causeways"

"causeys"

"causing"

"caustic"

"caustically"

"causticity"

"caustics"

"cauterization"

"cauterize"

"cauterized"

"cauterizes"

"cauterizing"

"cautery"

"caution"

"cautionary"

"cautioned"

"cautioner"

"cautioning"

"cautions"

"cautious"

"cautiously"

"cautiousness"

"cavalcade"

"cavalcades"

"cavalier"

"cavaliered"

"cavalierly"

"cavalierness"

"cavaliers"

"cavalries"

"cavalry"

"cavalryman"

"cavalrymen"

"cave"

"caveat"

"caveated"

"caveatee"

"caveator"

"caveats"

"caved"

"cavefish"

"caveman"

"cavemen"

"caver"

"cavern"

"caverned"

"caverning"

"cavernous"

"cavernously"

"caverns"

"cavers"

"caves"

"caviar"

"caviare"

"caviares"

"caviars"

"cavie"

"cavies"

"cavil"

"caviled"

"caviler"

"cavilers"

"caviling"

"cavilled"

"caviller"

"cavillers"

"cavilling"

"cavils"

"caving"

"cavitate"

"cavitated"

"cavitates"

"cavitation"

"cavitations"

"cavitied"

"cavities"

"cavity"

"cavort"

"cavorted"

"cavorter"

"cavorters"

"cavorting"

"cavorts"

"cavy"

"caw"

"cawed"

"cawing"

"caws"

"cay"

"cayenne"

"cayenned"

"cayennes"

"cayman"

"caymans"

"cays"

"cayugas"

"cayuse"

"cayuses"

"cc"

"cd"

"cease"

"ceased"

"ceaseless"

"ceaselessly"

"ceaselessness"

"ceases"

"ceasing"

"ceca"

"cecal"

"cecil"

"cecropia"

"cecum"

"cedar"

"cedars"

"cedarwood"

"cede"

"ceded"

"ceder"

"ceders"

"cedes"

"cedilla"

"cedillas"

"ceding"

"cedulas"

"cees"

"Ceftazidime model"

"ceil"

"ceiled"

"ceiler"

"ceilers"

"ceiling"

"ceilings"

"ceilometer"

"ceils"

"ceinture"

"celadon"

"celadons"

"celandine"

"celandines"

"celeb"

"celebrant"

"celebrants"

"celebrate"

"celebrated"

"celebrates"

"celebrating"

"celebration"

"celebrationis"

"celebrations"

"celebrator"

"celebrators"

"celebre"

"celebres"

"celebrities"

"celebrity"

"celebs"

"celeriac"

"celeries"

"celerities"

"celerity"

"celery"

"celesta"

"celestas"

"celeste"

"celestes"

"celestial"

"celestially"

"celiac"

"celibacies"

"celibacy"

"celibate"

"celibates"

"cell"

"cellar"

"cellarage"

"cellared"

"cellarer"

"cellarers"

"cellaret"

"cellarets"

"cellaring"

"cellars"

"cellblock"

"cellblocks"

"celled"

"celli"

"celling"

"cellist"

"cellists"

"cello"

"cellophane"

"cellos"

"cells"

"cellular"

"cellulitis"

"celluloid"

"cellulose"

"cellulosic"

"celsius"

"celt"

"celtic"

"celts"

"cembali"

"cembalo"

"cembalos"

"cement"

"cementation"

"cemented"

"cementer"

"cementers"

"cementing"

"cements"

"cementum"

"cemetaries"

"cemetary"

"cemeteries"

"cemetery"

"cenacle"

"cenacles"

"cenobite"

"cenobites"

"cenobitic"

"cenobitical"

"cenophobia"

"cenotaph"

"cenotaphic"

"cenotaphs"

"cenozoic"

"cense"

"censed"

"censer"

"censers"

"censes"

"censing"

"censor"

"censorable"

"censored"

"censorial"

"censoring"

"censorious"

"censoriously"

"censoriousness"

"censors"

"censorship"

"censurable"

"censure"

"censured"

"censureless"

"censurer"

"censurers"

"censures"

"censuring"

"census"

"censused"

"censuses"

"censusing"

"cent"

"centare"

"centares"

"centaur"

"centaurs"

"centaury"

"centavo"

"centavos"

"centenarian"

"centenarians"

"centenaries"

"centenary"

"centennial"

"centennials"

"center"

"centerboard"

"centerboards"

"centered"

"centeredly"

"centeredness"

"centerfold"

"centerfolds"

"centering"

"centerline"

"centerpiece"

"centerpieces"

"centers"

"centesimal"

"centigrade"

"centigram"

"centigrams"

"centile"

"centiliter"

"centiliters"

"centillion"

"centime"

"centimes"

"centimeter"

"centimeters"

"centimo"

"centimos"

"centipede"

"centipedes"

"centner"

"cento"

"centra"

"central"

"centralest"

"centralism"

"centralist"

"centralistic"

"centralists"

"centralities"

"centrality"

"centralization"

"centralize"

"centralized"

"centralizer"

"centralizers"

"centralizes"

"centralizing"

"centrally"

"centrals"

"centre"

"centred"

"centres"

"centric"

"centrifugal"

"centrifugalize"

"centrifugally"

"centrifugation"

"centrifuge"

"centrifuged"

"centrifuges"

"centrifuging"

"centring"

"centripetal"

"centripetally"

"centrism"

"centrist"

"centrists"

"centroid"

"centroids"

"centrum"

"centrums"

"cents"

"centum"

"centums"

"centuple"

"centupled"

"centuples"

"centupling"

"centuries"

"centurion"

"centurions"

"century"

"cep"

"cephalic"

"cephalically"

"ceramic"

"ceramicist"

"ceramicists"

"ceramics"

"ceramist"

"ceramists"

"cerated"

"cerates"

"ceraunograph"

"cereal"

"cereals"

"cerebella"

"cerebellar"

"cerebellum"

"cerebellums"

"cerebra"

"cerebral"

"cerebrally"

"cerebrals"

"cerebrate"

"cerebrated"

"cerebrates"

"cerebrating"

"cerebration"

"cerebrations"

"cerebri"

"cerebric"

"cerebroid"

"cerebrospinal"

"cerebrovascular"

"cerebrum"

"cerebrums"

"cerecloth"

"cerecloths"

"cered"

"cerement"

"cerements"

"ceremonial"

"ceremonialism"

"ceremonialist"

"ceremonialists"

"ceremonially"

"ceremonials"

"ceremonies"

"ceremonious"

"ceremoniously"

"ceremoniousness"

"ceremony"

"cerenkov"

"ceres"

"cereus"

"cereuses"

"ceria"

"cerias"

"ceriphs"

"cerise"

"cerises"

"cerites"

"cerium"

"ceriums"

"cermet"

"cermets"

"cert"

"certain"

"certainest"

"certainly"

"certainness"

"certainties"

"certainty"

"certes"

"certifiable"

"certifiably"

"certificate"

"certificated"

"certificates"

"certificating"

"certification"

"certification exams"

"certifications"

"certified"

"Certified Medical Transcriptionist"

"certified personal trainer"

"certifier"

"certifiers"

"certifies"

"certify"

"certifying"

"certitude"

"certitudes"

"cerulean"

"ceruleans"

"cerumen"

"ceruminous"

"cervantes"

"cervical"

"cervices"

"cervicitis"

"cervine"

"cervix"

"cervixes"

"cesarean"

"cesareans"

"cesarian"

"cesium"

"cesiums"

"cess"

"cessation"

"cessed"

"cesses"

"cessing"

"cession"

"cessions"

"cesspit"

"cesspits"

"cesspool"

"cesspools"

"cesura"

"cesurae"

"cesuras"

"cetacean"

"cetaceans"

"cetera"

"cetologies"

"cetology"

"ceylon"

"ceylonese"

"cgs"

"chablis"

"chaconne"

"chaconnes"

"chad"

"chadarim"

"chads"

"chafe"

"chafed"

"chafer"

"chafers"

"chafes"

"chaff"

"chaffed"

"chaffer"

"chaffered"

"chafferer"

"chafferers"

"chaffering"

"chaffers"

"chaffier"

"chaffiest"

"chaffinch"

"chaffinches"

"chaffing"

"chaffs"

"chaffy"

"chafing"

"chagrin"

"chagrined"

"chagrining"

"chagrinned"

"chagrinning"

"chagrins"

"chain"

"chained"

"chaines"

"chaining"

"chainlike"

"chainman"

"chainmen"

"chains"

"chair"

"chaired"

"chairing"

"chairladies"

"chairlady"

"chairman"

"chairmaned"

"chairmanned"

"chairmanning"

"chairmans"

"chairmanship"

"chairmanships"

"chairmen"

"chairperson"

"chairpersons"

"chairs"

"chairwoman"

"chairwomen"

"chaise"

"chaises"

"chakra"

"chalah"

"chalcedonic"

"chalcedonies"

"chalcedony"

"chalcography"

"chalcopyrite"

"chalcotriptics"

"chaldron"

"chalet"

"chalets"

"chalice"

"chalices"

"chalk"

"chalkboard"

"chalkboards"

"chalked"

"chalkier"

"chalkiest"

"chalkiness"

"chalking"

"chalks"

"chalky"

"challah"

"challahs"

"challenge"

"challengeable"

"challenged"

"challenger"

"challengers"

"challenges"

"challenging"

"challengingly"

"challie"

"challies"

"challis"

"challises"

"challot"

"cham"

"chamber"

"chambered"

"chamberlain"

"chamberlains"

"chambermaid"

"chambermaids"

"chambers"

"chambray"

"chambrays"

"chameleon"

"chameleons"

"chamfer"

"chamfered"

"chamfering"

"chamfers"

"chamise"

"chamises"

"chamiso"

"chamisos"

"chammied"

"chammies"

"chamois"

"chamoised"

"chamoises"

"chamoising"

"chamoix"

"chamomile"

"champ"

"champagne"

"champagnes"

"champaign"

"champed"

"champer"

"champers"

"champing"

"champion"

"championed"

"championing"

"champions"

"championship"

"championships"

"champs"

"champy"

"chams"

"chance"

"chanced"

"chancel"

"chancelleries"

"chancellery"

"chancellor"

"chancellors"

"chancellorship"

"chancellorships"

"chancels"

"chanceman"

"chancemen"

"chancer"

"chanceries"

"chancering"

"chancery"

"chances"

"chancier"

"chanciest"

"chancily"

"chancing"

"chancre"

"chancres"

"chancroid"

"chancroids"

"chancy"

"chandelier"

"chandeliers"

"chandler"

"chandleries"

"chandlers"

"chandlery"

"chang"

"change"

"changeable"

"changed"

"changeful"

"changeless"

"changeling"

"changelings"

"changeover"

"changeovers"

"changer"

"changers"

"changes"

"changing"

"channel"

"channeled"

"channeling"

"channelization"

"channelize"

"channelized"

"channelizes"

"channelizing"

"channelled"

"channelling"

"channels"

"chanson"

"chansons"

"chant"

"chantage"

"chantages"

"chanted"

"chanter"

"chanters"

"chanteuse"

"chanteuses"

"chantey"

"chanteys"

"chanticleer"

"chanticleers"

"chanties"

"chanting"

"chantor"

"chantors"

"chantries"

"chantry"

"chants"

"chanty"

"chaology"

"chaos"

"chaoses"

"chaotic"

"chaotically"

"chaoticness"

"chap"

"chaparral"

"chaparrals"

"chapbook"

"chapbooks"

"chapeau"

"chapeaus"

"chapeaux"

"chapel"

"chapels"

"chaperon"

"chaperonage"

"chaperoned"

"chaperoning"

"chaperons"

"chapfallen"

"chaplain"

"chaplaincies"

"chaplaincy"

"chaplains"

"chaplet"

"chapleted"

"chaplets"

"chaplin"

"chapman"

"chapmen"

"chapped"

"chapping"

"chaps"

"chapt"

"chapter"

"chaptered"

"chaptering"

"chapters"

"char"

"character"

"characteristic"

"characteristically"

"characteristics"

"characterization"

"characterizations"

"characterize"

"characterized"

"characterizes"

"characterizing"

"characterless"

"characterology"

"characters"

"charactery"

"charade"

"charades"

"charbroil"

"charbroiled"

"charbroiling"

"charbroils"

"charcoal"

"charcoaled"

"charcoals"

"chard"

"chards"

"chare"

"chared"

"chares"

"charge"

"chargeable"

"charged"

"chargee"

"charger"

"chargers"

"charges"

"charging"

"charier"

"chariest"

"charily"

"chariness"

"charing"

"chariot"

"charioteer"

"charioteers"

"charioting"

"chariots"

"charism"

"charisma"

"charismas"

"charismatic"

"charisms"

"charitable"

"charitableness"

"charitably"

"charities"

"charity"

"charladies"

"charlady"

"charlatan"

"charlatanic"

"charlatanish"

"charlatanism"

"charlatanries"

"charlatanry"

"charlatans"

"charlemagne"

"charles"

"charleston"

"charlestons"

"charley"

"charlie"

"charlotte"

"charlottesville"

"charm"

"charmed"

"charmer"

"charmers"

"charming"

"charminger"

"charmingly"

"charms"

"charnel"

"charnels"

"charon"

"charred"

"charrier"

"charring"

"charros"

"charry"

"chars"

"chart"

"charted"

"charter"

"chartered"

"charterer"

"charterers"

"chartering"

"charters"

"charting"

"chartings"

"chartist"

"chartists"

"chartreuse"

"charts"

"charwoman"

"charwomen"

"chary"

"chase"

"chased"

"chaser"

"chasers"

"chases"

"chasing"

"chasings"

"chasm"

"chasmal"

"chasmed"

"chasmic"

"chasms"

"chasmy"

"chassed"

"chasses"

"chassis"

"chaste"

"chastely"

"chasten"

"chastened"

"chastener"

"chasteners"

"chasteness"

"chastening"

"chastens"

"chaster"

"chastest"

"chastise"

"chastised"

"chastisement"

"chastiser"

"chastisers"

"chastises"

"chastising"

"chastities"

"chastity"

"chasuble"

"chasubles"

"chat"

"chateau"

"chateaus"

"chateaux"

"chatelaine"

"chatelaines"

"chats"

"chattanooga"

"chatted"

"chattel"

"chattels"

"chatter"

"chatterbox"

"chatterboxes"

"chattered"

"chatterer"

"chatterers"

"chattering"

"chatters"

"chattery"

"chattier"

"chattiest"

"chattily"

"chattiness"

"chatting"

"chatty"

"chaucer"

"chaucerian"

"chaufers"

"chauffer"

"chauffers"

"chauffeur"

"chauffeured"

"chauffeuring"

"chauffeurs"

"chauffeuse"

"chaunters"

"chaunting"

"chauvinism"

"chauvinist"

"chauvinistic"

"chauvinistically"

"chauvinists"

"chaw"

"chawed"

"chawer"

"chawers"

"chawing"

"chaws"

"chayote"

"chayotes"

"cheap"

"cheapen"

"cheapened"

"cheapening"

"cheapens"

"cheaper"

"cheapest"

"cheapie"

"cheapies"

"cheapish"

"cheaply"

"cheapness"

"cheaps"

"cheapskate"

"cheapskates"

"cheat"

"cheated"

"cheater"

"cheateries"

"cheaters"

"cheatery"

"cheating"

"cheatingly"

"cheats"

"check"

"checkable"

"checkbook"

"checkbooks"

"checkbox"

"checked"

"checker"

"checkerboard"

"checkerboards"

"checkered"

"checkering"

"checkers"

"checking"

"checkless"

"checklist"

"checklists"

"checkmate"

"checkmated"

"checkmates"

"checkmating"

"checkoff"

"checkoffs"

"checkout"

"checkouts"

"checkpoint"

"checkpoints"

"checkroom"

"checkrooms"

"checkrowed"

"checks"

"checksum"

"checksums"

"checkup"

"checkups"

"chedar"

"cheddar"

"cheddars"

"cheek"

"cheekbone"

"cheekbones"

"cheeked"

"cheekful"

"cheekfuls"

"cheekier"

"cheekiest"

"cheekily"

"cheekiness"

"cheeking"

"cheeks"

"cheeky"

"cheep"

"cheeped"

"cheeper"

"cheepers"

"cheeping"

"cheeps"

"cheer"

"cheered"

"cheerer"

"cheerers"

"cheerful"

"cheerfully"

"cheerfulness"

"cheerier"

"cheeriest"

"cheerily"

"cheeriness"

"cheering"

"cheerio"

"cheerios"

"cheerleader"

"cheerleaders"

"cheerless"

"cheerlessly"

"cheerlessness"

"cheers"

"cheery"

"cheese"

"cheeseburger"

"cheeseburgers"

"cheesecake"

"cheesecakes"

"cheesecloth"

"cheesecloths"

"cheesed"

"cheeseparing"

"cheeses"

"cheesier"

"cheesiest"

"cheesily"

"cheesiness"

"cheesing"

"cheesy"

"cheetah"

"cheetahs"

"chef"

"chefdom"

"chefdoms"

"chefs"

"chekhov"

"chela"

"chelas"

"chelate"

"chelated"

"chelates"

"chelating"

"chelation"

"chelator"

"chelators"

"chem"

"chemical"

"chemically"

"chemicals"

"chemics"

"chemin"

"chemins"

"chemise"

"chemises"

"chemism"

"chemisms"

"chemist"

"chemistries"

"chemistry"

"chemists"

"chemoreception"

"chemoreceptive"

"chemoreceptivities"

"chemoreceptivity"

"chemoreceptor"

"chemosensitive"

"chemosensitivities"

"chemosensitivity"

"chemosterilant"

"chemosterilants"

"chemosurgery"

"chemotherapeutic"

"chemotherapeutical"

"chemotherapeutically"

"chemotherapeuticness"

"chemotherapeutics"

"chemotherapies"

"chemotherapist"

"chemotherapists"

"chemotherapy"

"chemotropism"

"chemurgic"

"chemurgy"

"chenille"

"chenilles"

"cheque"

"chequer"

"chequered"

"chequering"

"chequers"

"cheques"

"cherchez"

"cherenkov"

"cherish"

"cherished"

"cherisher"

"cherishers"

"cherishes"

"cherishing"

"cherokee"

"cherokees"

"cheroot"

"cheroots"

"cherries"

"cherry"

"cherrystone"

"cherrystones"

"chert"

"chertier"

"cherty"

"cherub"

"cherubic"

"cherubical"

"cherubically"

"cherubim"

"cherubs"

"chervil"

"chervils"

"chesapeake"

"chess"

"chessboard"

"chessboards"

"chesses"

"chessman"

"chessmen"

"chest"

"chest compressions"

"chested"

"chesterfield"

"chesterfields"

"chestful"

"chestfuls"

"chestier"

"chestiest"

"chestnut"

"chestnuts"

"chests"

"chesty"

"cheval"

"chevalier"

"chevaliers"

"chevaux"

"chevied"

"chevies"

"cheviot"

"chevrolet"

"chevrolets"

"chevron"

"chevrons"

"chevy"

"chevying"

"chew"

"chewable"

"chewed"

"chewer"

"chewers"

"chewier"

"chewiest"

"chewing"

"chews"

"chewy"

"cheyenne"

"cheyennes"

"chez"

"CHF"

"chi"

"chia"

"chianti"

"chiao"

"chiaroscuro"

"chiaroscuros"

"chias"

"chiasma"

"chiasms"

"chic"

"chicago"

"chicagoan"

"chicagoans"

"chicane"

"chicaned"

"chicaner"

"chicaneries"

"chicaners"

"chicanery"

"chicanes"

"chicaning"

"chicano"

"chicanos"

"chiccory"

"chichi"

"chichis"

"chick"

"chickadee"

"chickadees"

"chickasaw"

"chickasaws"

"chicken"

"chickened"

"chickening"

"chickens"

"chickpea"

"chickpeas"

"chicks"

"chickweed"

"chickweeds"

"chicle"

"chicles"

"chicly"

"chicness"

"chico"

"chicories"

"chicory"

"chicos"

"chics"

"chid"

"chidden"

"chide"

"chided"

"chider"

"chiders"

"chides"

"chiding"

"chidingly"

"chief"

"chiefdom"

"chiefdoms"

"chiefer"

"chiefest"

"chiefly"

"chiefs"

"chieftain"

"chieftaincies"

"chieftaincy"

"chieftains"

"chieftainship"

"chieftainships"

"chiel"

"chields"

"chiels"

"chiffon"

"chiffonier"

"chiffoniers"

"chiffonnier"

"chiffonniers"

"chiffons"

"chifforobe"

"chifforobes"

"chigger"

"chiggers"

"chignon"

"chignons"

"chigoe"

"chigoes"

"chihuahua"

"chihuahuas"

"chilblain"

"chilblains"

"child"

"childbearing"

"childbed"

"childbeds"

"childbirth"

"childbirths"

"childhood"

"childhoods"

"childing"

"childish"

"childishly"

"childishness"

"childless"

"childlessness"

"childliest"

"childlike"

"childly"

"childproof"

"children"

"chile"

"chilean"

"chileans"

"chiles"

"chili"

"chilies"

"chill"

"chilled"

"chiller"

"chillers"

"chillest"

"chilli"

"chillier"

"chillies"

"chilliest"

"chillily"

"chilliness"

"chilling"

"chillingly"

"chillness"

"chills"

"chillum"

"chillums"

"chilly"

"chimaera"

"chimaeras"

"chimbley"

"chimbly"

"chime"

"chimed"

"chimer"

"chimera"

"chimeras"

"chimeric"

"chimerical"

"chimers"

"chimes"

"chiming"

"chimley"

"chimney"

"chimneys"

"chimp"

"chimpanzee"

"chimpanzees"

"chimps"

"chin"

"china"

"chinamania"

"chinas"

"chinatown"

"chinaware"

"chinbone"

"chinch"

"chinches"

"chinchiest"

"chinchilla"

"chinchillas"

"chinchy"

"chine"

"chines"

"chinese"

"Chinese culture"

"Chinese instruments"

"Chinese medicine"

"Chinese Tai Chi way"

"chining"

"chink"

"chinked"

"chinkier"

"chinkiest"

"chinking"

"chinks"

"chinky"

"chinless"

"chinned"

"chinning"

"chino"

"chinone"

"chinook"

"chinooks"

"chinos"

"chins"

"chints"

"chintz"

"chintzes"

"chintzier"

"chintziest"

"chintzy"

"chip"

"chipmunk"

"chipmunks"

"chipped"

"chipper"

"chippered"

"chippering"

"chippers"

"chippewa"

"chippewas"

"chippie"

"chippies"

"chipping"

"chippy"

"chips"

"chirk"

"chirked"

"chirker"

"chirks"

"chirocosmetics"

"chirographer"

"chirographers"

"chirographic"

"chirographical"

"chirography"

"chirologies"

"chirology"

"chiromancy"

"chiropodist"

"chiropodists"

"chiropody"

"chiropractic"

"chiropractor"

"chiropractors"

"chiropraxis"

"chirp"

"chirped"

"chirper"

"chirpers"

"chirpier"

"chirpiest"

"chirpily"

"chirping"

"chirps"

"chirpy"

"chirrup"

"chirruped"

"chirruping"

"chirrups"

"chirrupy"

"chisel"

"chiseled"

"chiseler"

"chiselers"

"chiseling"

"chiselled"

"chiseller"

"chisellers"

"chiselling"

"chisels"

"chit"

"chitchat"

"chitchats"

"chitin"

"chitinous"

"chitins"

"chitlin"

"chitling"

"chitlings"

"chitlins"

"chiton"

"chitons"

"chits"

"chitter"

"chittered"

"chittering"

"chitterlings"

"chitters"

"chitties"

"chivalric"

"chivalries"

"chivalrous"

"chivalrously"

"chivalrousness"

"chivalry"

"chivaree"

"chive"

"chives"

"chivied"

"chivies"

"chivvied"

"chivvies"

"chivvy"

"chivvying"

"chivy"

"chivying"

"chloral"

"chlorals"

"chlorate"

"chlorates"

"chlordane"

"chloric"

"chlorid"

"chloride"

"chlorides"

"chlorin"

"chlorinate"

"chlorinated"

"chlorinates"

"chlorinating"

"chlorination"

"chlorinator"

"chlorinators"

"chlorine"

"chlorines"

"chlorite"

"chlorites"

"chloroform"

"chloroformed"

"chloroforming"

"chloroforms"

"chlorometer"

"chlorophyll"

"chloroplast"

"chlorosis"

"chlorotic"

"chlorous"

"chlorpromazine"

"chm"

"chock"

"chocked"

"chocking"

"chocks"

"chocolate"

"chocolates"

"choctaw"

"choctaws"

"choice"

"choicely"

"choiceness"

"choicer"

"choices"

"choicest"

"choir"

"choirboy"

"choirboys"

"choired"

"choiring"

"choirmaster"

"choirmasters"

"choirs"

"choke"

"choked"

"choker"

"chokers"

"chokes"

"chokey"

"chokier"

"choking"

"choking victim"

"choky"

"choler"

"cholera"

"choleras"

"choleric"

"cholers"

"cholesterol"

"choline"

"cholla"

"chollas"

"chomp"

"chomped"

"chomping"

"chomps"

"chondrite"

"chondrites"

"chondrule"

"chondrules"

"choose"

"chooser"

"choosers"

"chooses"

"choosey"

"choosier"

"choosiest"

"choosiness"

"choosing"

"choosy"

"chop"

"chophouse"

"chophouses"

"chopin"

"chopins"

"chopped"

"chopper"

"choppers"

"choppier"

"choppiest"

"choppily"

"choppiness"

"chopping"

"choppy"

"chops"

"chopstick"

"chopsticks"

"choral"

"chorale"

"chorales"

"chorally"

"chorals"

"chord"

"chordal"

"chordate"

"chordates"

"chorded"

"chording"

"chords"

"chore"

"chorea"

"choreal"

"choreas"

"chored"

"choreic"

"choreman"

"choremen"

"choreograph"

"choreographed"

"choreographer"

"choreographers"

"choreographic"

"choreographically"

"choreographing"

"choreographs"

"choreography"

"choreomania"

"chores"

"chorial"

"choric"

"chorine"

"chorines"

"choring"

"chorion"

"chorister"

"choristers"

"chorizo"

"chorizos"

"choroid"

"choroids"

"chorology"

"chortle"

"chortled"

"chortler"

"chortlers"

"chortles"

"chortling"

"chorus"

"chorused"

"choruses"

"chorusing"

"chorussed"

"chorusses"

"chorussing"

"chose"

"chosen"

"choses"

"chou"

"chow"

"chowchow"

"chowchows"

"chowder"

"chowdered"

"chowdering"

"chowders"

"chowed"

"chowing"

"chows"

"chowtime"

"chowtimes"

"chrematistics"

"chrematophobia"

"chrism"

"chrisms"

"christ"

"christen"

"christendom"

"christened"

"christener"

"christeners"

"christening"

"christens"

"christian"

"christianity"

"christianize"

"christianized"

"christianizes"

"christianizing"

"christians"

"christie"

"christies"

"christine"

"christly"

"christmas"

"christmases"

"christmastide"

"christopher"

"christs"

"christy"

"chroma"

"chromas"

"chromate"

"chromatic"

"chromatically"

"chromaticism"

"chromaticity"

"chromatogram"

"chromatograph"

"chromatographic"

"chromatographically"

"chromatography"

"chromatoptometer"

"chrome"

"chromed"

"chromes"

"chromic"

"chromide"

"chroming"

"chromite"

"chromium"

"chromiums"

"chromize"

"chromized"

"chromizes"

"chromizing"

"chromo"

"chromos"

"chromosomal"

"chromosomally"

"chromosome"

"chromosomes"

"chromosomic"

"chromosphere"

"chromospheres"

"chromospheric"

"chronaxy"

"chronic"

"chronic condition"

"chronic renal failure"

"chronically"

"chronicity"

"chronicle"

"chronicled"

"chronicler"

"chroniclers"

"chronicles"

"chronicling"

"chronics"

"chronobiology"

"chronograph"

"chronographic"

"chronographs"

"chronography"

"chronol"

"chronological"

"chronologically"

"chronologies"

"chronologist"

"chronologists"

"chronology"

"chronometer"

"chronometers"

"chronon"

"chronons"

"chronoscope"

"chrysalides"

"chrysalis"

"chrysalises"

"chrysanthemum"

"chrysanthemums"

"chrysler"

"chryslers"

"chrysolite"

"chrysology"

"chthonic"

"chub"

"chubbier"

"chubbiest"

"chubbily"

"chubbiness"

"chubby"

"chubs"

"chuck"

"chucked"

"chuckfull"

"chuckhole"

"chuckholes"

"chuckies"

"chucking"

"chuckle"

"chuckled"

"chuckler"

"chucklers"

"chuckles"

"chuckling"

"chucks"

"chucky"

"chuff"

"chuffed"

"chuffer"

"chuffing"

"chuffs"

"chuffy"

"chug"

"chugged"

"chugger"

"chuggers"

"chugging"

"chugs"

"chukka"

"chukkas"

"chukker"

"chukkers"

"chum"

"chummed"

"chummier"

"chummiest"

"chummily"

"chumminess"

"chumming"

"chummy"

"chump"

"chumped"

"chumping"

"chumps"

"chums"

"chumship"

"chumships"

"chungking"

"chunk"

"chunked"

"chunkier"

"chunkiest"

"chunkily"

"chunkiness"

"chunking"

"chunks"

"chunky"

"chunter"

"church"

"churched"

"churches"

"churchgoer"

"churchgoers"

"churchgoing"

"churchier"

"churchiest"

"churchill"

"churching"

"churchless"

"churchlier"

"churchly"

"churchman"

"churchmen"

"churchwarden"

"churchwardens"

"churchwoman"

"churchwomen"

"churchy"

"churchyard"

"churchyards"

"churl"

"churlish"

"churlishly"

"churlishness"

"churls"

"churn"

"churned"

"churner"

"churners"

"churning"

"churns"

"churrs"

"chute"

"chuted"

"chutes"

"chuting"

"chutist"

"chutists"

"chutnees"

"chutney"

"chutneys"

"chutzpa"

"chutzpah"

"chutzpahs"

"chutzpas"

"chyme"

"chymics"

"chymist"

"chymists"

"cia"

"ciao"

"cibophobia"

"cicada"

"cicadae"

"cicadas"

"cicatrices"

"cicatrix"

"cicatrixes"

"cicatrize"

"cicatrized"

"cicelies"

"cicely"

"cicero"

"cicerone"

"cicerones"

"ciceros"

"cichlid"

"cichlidae"

"cichlids"

"cider"

"ciders"

"cigar"

"cigaret"

"cigarets"

"cigarette"

"cigarettes"

"cigarillo"

"cigarillos"

"cigars"

"cilantro"

"cilantros"

"cilia"

"ciliary"

"ciliata"

"ciliate"

"ciliated"

"ciliates"

"cilium"

"cinch"

"cinched"

"cinches"

"cinching"

"cinchona"

"cinchonas"

"cincinnati"

"cincture"

"cinctured"

"cinctures"

"cincturing"

"cinder"

"cindered"

"cindering"

"cinderous"

"cinders"

"cindery"

"cine"

"cinema"

"cinemas"

"cinematheque"

"cinematheques"

"cinematic"

"cinematically"

"cinematograph"

"cinematographer"

"cinematographers"

"cinematographic"

"cinematographies"

"cinematography"

"cinerama"

"cineraria"

"cinerarium"

"cinerary"

"cinereal"

"cines"

"cinnabar"

"cinnabars"

"cinnamon"

"cinnamons"

"cinquain"

"cinquains"

"cinque"

"cinquefoil"

"cinquefoils"

"cinques"

"cions"

"cipher"

"ciphered"

"ciphering"

"ciphers"

"ciphonies"

"circ"

"circa"

"circadian"

"circe"

"circiter"

"circle"

"circled"

"circler"

"circlers"

"circles"

"circlet"

"circlets"

"circling"

"circuit"

"circuital"

"circuited"

"circuiteer"

"circuiter"

"circuities"

"circuiting"

"circuitous"

"circuitously"

"circuitry"

"circuits"

"circuity"

"circular"

"circularity"

"circularization"

"circularizations"

"circularize"

"circularized"

"circularizer"

"circularizers"

"circularizes"

"circularizing"

"circularly"

"circularness"

"circulars"

"circulate"

"circulated"

"circulates"

"circulating"

"circulation"

"circulations"

"circulative"

"circulator"

"circulators"

"circulatory"

"circum"

"circumambulate"

"circumambulated"

"circumambulates"

"circumambulating"

"circumambulation"

"circumambulations"

"circumcise"

"circumcised"

"circumcises"

"circumcising"

"circumcision"

"circumcisions"

"circumference"

"circumferences"

"circumflex"

"circumflexes"

"circumlocution"

"circumlocutions"

"circumlocutory"

"circumlunar"

"circumnavigate"

"circumnavigated"

"circumnavigates"

"circumnavigating"

"circumnavigation"

"circumnavigations"

"circumpolar"

"circumscribe"

"circumscribed"

"circumscribes"

"circumscribing"

"circumscription"

"circumscriptions"

"circumsolar"

"circumspect"

"circumspection"

"circumstance"

"circumstanced"

"circumstances"

"circumstantial"

"circumstantially"

"circumstantiate"

"circumstantiated"

"circumstantiates"

"circumstantiating"

"circumstantiation"

"circumstantiations"

"circumvent"

"circumventable"

"circumvented"

"circumventing"

"circumvention"

"circumventions"

"circumvents"

"circus"

"circuses"

"circusy"

"cirque"

"cirques"

"cirrhosis"

"cirrhotic"

"cirrocumulus"

"cirrose"

"cirrostratus"

"cirrous"

"cirrus"

"ciscoes"

"ciscos"

"ciselure"

"cislunar"

"cistern"

"cisternal"

"cisterns"

"cists"

"cit"

"citable"

"citadel"

"citadels"

"citation"

"citations"

"citator"

"citatory"

"citatum"

"cite"

"citeable"

"cited"

"citer"

"citers"

"cites"

"cithara"

"cithern"

"citherns"

"cithers"

"citicorp"

"citied"

"cities"

"citification"

"citified"

"citifies"

"citify"

"citifying"

"citing"

"citizen"

"citizenly"

"citizenries"

"citizenry"

"citizens"

"citizenship"

"citrate"

"citrates"

"citric"

"citrine"

"citrines"

"citrins"

"citron"

"citronella"

"citrons"

"citrous"

"citrus"

"citruses"

"cittern"

"city"

"cityfied"

"cityward"

"citywide"

"civet"

"civets"

"civic"

"civically"

"civicism"

"civicisms"

"civics"

"civies"

"civil"

"civiler"

"civilest"

"civilian"

"civilians"

"civilise"

"civilising"

"civilities"

"civility"

"civilizable"

"civilization"

"civilizations"

"civilize"

"civilized"

"civilizer"

"civilizers"

"civilizes"

"civilizing"

"civilly"

"civilness"

"civisms"

"civitas"

"civvies"

"civvy"

"cl"

"clabber"

"clabbered"

"clabbering"

"clabbers"

"clack"

"clacked"

"clacker"

"clackers"

"clacking"

"clacks"

"clad"

"cladding"

"claddings"

"clads"

"clagging"

"clags"

"claim"

"claimable"

"claimant"

"claimants"

"claimed"

"claimer"

"claimers"

"claiming"

"claimless"

"claims"

"clair"

"clairvoyance"

"clairvoyances"

"clairvoyancies"

"clairvoyancy"

"clairvoyant"

"clairvoyantly"

"clairvoyants"

"clam"

"clambake"

"clambakes"

"clamber"

"clambered"

"clambering"

"clambers"

"clammed"

"clammier"

"clammiest"

"clammily"

"clamminess"

"clamming"

"clammy"

"clamor"

"clamored"

"clamorer"

"clamorers"

"clamoring"

"clamorous"

"clamorously"

"clamorousness"

"clamors"

"clamour"

"clamoured"

"clamouring"

"clamours"

"clamp"

"clamped"

"clamper"

"clampers"

"clamping"

"clamps"

"clams"

"clamshell"

"clamshells"

"clamworm"

"clan"

"clandestine"

"clandestinely"

"clandestineness"

"clandestinity"

"clang"

"clanged"

"clanging"

"clangor"

"clangored"

"clangoring"

"clangorous"

"clangorously"

"clangors"

"clangour"

"clangoured"

"clangours"

"clangs"

"clank"

"clanked"

"clanking"

"clanks"

"clannish"

"clannishly"

"clannishness"

"clans"

"clansman"

"clansmen"

"clanswoman"

"clanswomen"

"clap"

"clapboard"

"clapboards"

"clapped"

"clapper"

"clappers"

"clapping"

"claps"

"clapt"

"claptrap"

"claptraps"

"claque"

"claques"

"clarence"

"claret"

"clarets"

"clarifiable"

"clarification"

"clarifications"

"clarified"

"clarifier"

"clarifiers"

"clarifies"

"clarify"

"clarifying"

"clarinet"

"clarinetist"

"clarinetists"

"clarinets"

"clarinettist"

"clarinettists"

"clarion"

"clarioned"

"clarioning"

"clarions"

"clarities"

"clarity"

"clark"

"clarke"

"clarkia"

"clarkias"

"clarksville"

"clash"

"clashed"

"clasher"

"clashers"

"clashes"

"clashing"

"clasp"

"clasped"

"clasper"

"claspers"

"clasping"

"clasps"

"claspt"

"class"

"classed"

"classer"

"classers"

"classes"

"classic"

"classical"

"classicalism"

"classically"

"classicism"

"classicist"

"classicists"

"classics"

"classier"

"classiest"

"classifiable"

"classification"

"classifications"

"classified"

"classifier"

"classifiers"

"classifies"

"classify"

"classifying"

"classily"

"classing"

"classless"

"classlessness"

"classmate"

"classmates"

"classroom"

"classrooms"

"classy"

"clastic"

"clatter"

"clattered"

"clatterer"

"clattering"

"clatters"

"clattery"

"claudius"

"claus"

"clausal"

"clause"

"clauses"

"claustrophobe"

"claustrophobia"

"claustrophobiac"

"claustrophobic"

"clave"

"claver"

"clavichord"

"clavichordist"

"clavichordists"

"clavichords"

"clavicle"

"clavicles"

"clavicular"

"clavier"

"clavierist"

"clavierists"

"claviers"

"claw"

"clawed"

"clawer"

"clawers"

"clawing"

"clawless"

"claws"

"claxon"

"claxons"

"clay"

"claybank"

"claybanks"

"clayed"

"clayey"

"clayier"

"claying"

"clayish"

"claymore"

"claymores"

"clays"

"clayware"

"claywares"

"clean"

"cleanable"

"cleaned"

"cleaner"

"cleaners"

"cleanest"

"cleaning"

"cleanlier"

"cleanliest"

"cleanliness"

"cleanly"

"cleanness"

"cleans"

"cleanse"

"cleansed"

"cleanser"

"cleansers"

"cleanses"

"cleansing"

"cleanup"

"cleanups"

"clear"

"clearable"

"clearance"

"clearances"

"cleared"

"clearer"

"clearest"

"clearheaded"

"clearheadedly"

"clearheadedness"

"clearing"

"clearinghouse"

"clearinghouses"

"clearings"

"clearly"

"clearness"

"clears"

"clearwater"

"cleat"

"cleated"

"cleating"

"cleats"

"cleavage"

"cleavages"

"cleave"

"cleaved"

"cleaver"

"cleavers"

"cleaves"

"cleaving"

"clef"

"clefs"

"cleft"

"clefts"

"clematis"

"clematises"

"clemencies"

"clemency"

"clement"

"clemently"

"clench"

"clenched"

"clenches"

"clenching"

"cleopatra"

"clepe"

"clept"

"clerestories"

"clerestory"

"clergies"

"clergy"

"clergyman"

"clergymen"

"clergywoman"

"clergywomen"

"cleric"

"clerical"

"clericalism"

"clericalist"

"clericalists"

"clericals"

"clerics"

"clerihews"

"clerk"

"clerkdom"

"clerkdoms"

"clerked"

"clerking"

"clerkish"

"clerklier"

"clerkliest"

"clerkly"

"clerks"

"clerkship"

"clerkships"

"cleveland"

"clever"

"cleverer"

"cleverest"

"cleverish"

"cleverly"

"cleverness"

"clevis"

"clevises"

"clew"

"clewed"

"clews"

"cliche"

"cliched"

"cliches"

"click"

"clicked"

"clicker"

"clickers"

"clicking"

"clicks"

"client"

"cliental"

"clientele"

"clienteles"

"clientless"

"clients"

"cliff"

"cliffhanger"

"cliffhangers"

"cliffhanging"

"cliffier"

"cliffiest"

"cliffs"

"cliffy"

"clift"

"clifts"

"climacophobia"

"climacteric"

"climacterics"

"climactic"

"climactically"

"climatal"

"climate"

"climates"

"climatic"

"climatical"

"climatically"

"climatologic"

"climatological"

"climatologically"

"climatologist"

"climatologists"

"climatology"

"climatotherapies"

"climatotherapy"

"climax"

"climaxed"

"climaxes"

"climaxing"

"climb"

"climbable"

"climbed"

"climber"

"climbers"

"climbing"

"climbs"

"clime"

"climes"

"clinch"

"clinched"

"clincher"

"clinchers"

"clinches"

"clinching"

"cline"

"cling"

"clinged"

"clinger"

"clingers"

"clingier"

"clingiest"

"clinging"

"clings"

"clingstone"

"clingstones"

"clingy"

"clinic"

"clinical"

"clinical tool"

"clinical trial"

"clinically"

"clinician"

"clinicians"

"clinics"

"clink"

"clinked"

"clinker"

"clinkered"

"clinkering"

"clinkers"

"clinking"

"clinks"

"clinology"

"clinomania"

"clinometer"

"clinophobia"

"clip"

"clipboard"

"clipboards"

"clipped"

"clipper"

"clippers"

"clipping"

"clippings"

"clips"

"clipsheet"

"clipsheets"

"clipt"

"clique"

"cliqued"

"cliques"

"cliquey"

"cliquier"

"cliquiest"

"cliquing"

"cliquish"

"cliquishly"

"cliquishness"

"cliquy"

"clitoral"

"clitoric"

"clitoridean"

"clitoridectomies"

"clitoridectomy"

"clitoris"

"clitorises"

"cloaca"

"cloacal"

"cloak"

"cloaked"

"cloaking"

"cloakroom"

"cloakrooms"

"cloaks"

"clobber"

"clobbered"

"clobbering"

"clobbers"

"cloche"

"cloches"

"clock"

"clock display"

"clocked"

"clocker"

"clockers"

"clocking"

"clockings"

"clocks"

"clockwise"

"clockwork"

"clockworks"

"clod"

"cloddier"

"cloddiest"

"cloddish"

"cloddishness"

"cloddy"

"clodhopper"

"clodhoppers"

"clodhopping"

"clodpate"

"clodpole"

"clodpoll"

"clods"

"clog"

"clogged"

"cloggier"

"cloggiest"

"clogging"

"cloggy"

"clogs"

"cloisonne"

"cloister"

"cloistered"

"cloistering"

"cloisters"

"cloistral"

"clomb"

"clomp"

"clomped"

"clomping"

"clomps"

"clonal"

"clonally"

"clone"

"cloned"

"clones"

"clonic"

"cloning"

"clonism"

"clonk"

"clonked"

"clonking"

"clonks"

"clop"

"clopped"

"clopping"

"clops"

"closable"

"close"

"Close your eyes"

"closeable"

"closed"

"closefisted"

"closefitting"

"closely"

"closemouthed"

"closeness"

"closeout"

"closeouts"

"closer"

"closers"

"closes"

"closest"

"closet"

"closeted"

"closeting"

"closets"

"closeup"

"closeups"

"closing"

"closings"

"closure"

"closured"

"closures"

"closuring"

"clot"

"cloth"

"clothbound"

"clothe"

"clothed"

"clothes"

"clotheshorse"

"clotheshorses"

"clothesline"

"clotheslines"

"clothespin"

"clothespins"

"clothespress"

"clothespresses"

"clothier"

"clothiers"

"clothing"

"clothings"

"cloths"

"clots"

"clotted"

"clotting"

"clotty"

"cloture"

"clotured"

"clotures"

"cloturing"

"cloud"

"cloud-distributed"

"cloudburst"

"cloudbursts"

"clouded"

"cloudier"

"cloudiest"

"cloudily"

"cloudiness"

"clouding"

"cloudless"

"cloudlet"

"cloudlets"

"cloudlike"

"clouds"

"cloudy"

"cloudy urine"

"clout"

"clouted"

"clouter"

"clouters"

"clouting"

"clouts"

"clove"

"cloven"

"clover"

"cloverleaf"

"cloverleaves"

"clovers"

"cloves"

"clown"

"clowned"

"clowneries"

"clownery"

"clowning"

"clownish"

"clownishly"

"clownishness"

"clowns"

"cloy"

"cloyed"

"cloying"

"cloys"

"club"

"clubable"

"clubbed"

"clubber"

"clubbers"

"clubbier"

"clubbiest"

"clubbing"

"clubby"

"clubfeet"

"clubfoot"

"clubfooted"

"clubhand"

"clubhauled"

"clubhouse"

"clubhouses"

"clubman"

"clubmen"

"clubrooms"

"clubroots"

"clubs"

"cluck"

"clucked"

"clucking"

"clucks"

"clue"

"clued"

"clueing"

"clues"

"cluing"

"clump"

"clumped"

"clumpier"

"clumpiest"

"clumping"

"clumpish"

"clumps"

"clumpy"

"clumsier"

"clumsiest"

"clumsily"

"clumsiness"

"clumsy"

"clung"

"clunk"

"clunked"

"clunker"

"clunkers"

"clunking"

"clunks"

"cluster"

"clustered"

"clustering"

"clusters"

"clustery"

"clutch"

"clutched"

"clutches"

"clutching"

"clutchy"

"clutter"

"cluttered"

"cluttering"

"clutters"

"clyster"

"cmdg"

"CMT"

"co"

"coach"

"coached"

"coacher"

"coachers"

"coaches"

"coaching"

"coachman"

"coachmen"

"coachwork"

"coact"

"coacted"

"coacting"

"coaction"

"coacts"

"coadjutor"

"coadjutors"

"coadmit"

"coaeval"

"coaevals"

"coagency"

"coagent"

"coagents"

"coagula"

"coagulability"

"coagulable"

"coagulant"

"coagulants"

"coagulate"

"coagulated"

"coagulates"

"coagulating"

"coagulation"

"coagulations"

"coagulative"

"coagulator"

"coagulators"

"coagulometer"

"coagulum"

"coal"

"coalbin"

"coalbins"

"coalbox"

"coalboxes"

"coaled"

"coaler"

"coalers"

"coalesce"

"coalesced"

"coalescence"

"coalescent"

"coalesces"

"coalescing"

"coalfish"

"coalhole"

"coalholes"

"coalified"

"coalifies"

"coalify"

"coaling"

"coalition"

"coalitional"

"coalitioner"

"coalitionist"

"coalitions"

"coalless"

"coalpit"

"coalpits"

"coals"

"coalsack"

"coalsacks"

"coalshed"

"coalsheds"

"coalyard"

"coalyards"

"coaming"

"coamings"

"coapts"

"coarse"

"coarsely"

"coarsen"

"coarsened"

"coarseness"

"coarsening"

"coarsens"

"coarser"

"coarsest"

"coast"

"coastal"

"coasted"

"coaster"

"coasters"

"coastguardsman"

"coastguardsmen"

"coasting"

"coastings"

"coastline"

"coastlines"

"coasts"

"coastward"

"coastwise"

"coat"

"coated"

"coatee"

"coater"

"coaters"

"coati"

"coating"

"coatings"

"coatis"

"coatless"

"coatrack"

"coatracks"

"coatroom"

"coatrooms"

"coats"

"coattail"

"coattails"

"coauthered"

"coauthor"

"coauthors"

"coax"

"coaxal"

"coaxed"

"coaxer"

"coaxers"

"coaxes"

"coaxial"

"coaxially"

"coaxing"

"coaxingly"

"cob"

"cobalt"

"cobaltic"

"cobalts"

"cobber"

"cobbers"

"cobbier"

"cobble"

"cobbled"

"cobbler"

"cobblers"

"cobbles"

"cobblestone"

"cobblestones"

"cobbling"

"cobby"

"cobnut"

"cobol"

"cobra"

"cobras"

"cobs"

"cobweb"

"cobwebbed"

"cobwebbier"

"cobwebbing"

"cobwebby"

"cobwebs"

"cocain"

"cocaine"

"cocaines"

"cocainism"

"cocainize"

"cocainized"

"cocains"

"cocas"

"cocci"

"coccus"

"coccygeal"

"coccyges"

"coccyx"

"coccyxes"

"cochaired"

"cochairing"

"cochairman"

"cochairmen"

"cochairs"

"cochineal"

"cochlea"

"cochleae"

"cochlear"

"cochleas"

"cock"

"cockade"

"cockaded"

"cockades"

"cockamamie"

"cockatoo"

"cockatoos"

"cockatrice"

"cockatrices"

"cockbilled"

"cockcrow"

"cockcrows"

"cocked"

"cocker"

"cockerel"

"cockerels"

"cockers"

"cockeye"

"cockeyed"

"cockeyes"

"cockfight"

"cockfights"

"cockhorse"

"cockhorses"

"cockier"

"cockiest"

"cockily"

"cockiness"

"cocking"

"cockish"

"cockle"

"cockled"

"cockles"

"cockleshell"

"cockleshells"

"cockney"

"cockneys"

"cockpit"

"cockpits"

"cockroach"

"cockroaches"

"cocks"

"cockscomb"

"cockscombs"

"cockspurs"

"cocksure"

"cocktail"

"cocktailed"

"cocktails"

"cockup"

"cockups"

"cocky"

"coco"

"cocoa"

"cocoanut"

"cocoanuts"

"cocoas"

"cocobolo"

"cocomat"

"cocomats"

"coconspirator"

"coconut"

"coconuts"

"cocoon"

"cocooned"

"cocooning"

"cocoons"

"cocos"

"cod"

"coda"

"codable"

"codal"

"codas"

"codder"

"codders"

"coddle"

"coddled"

"coddler"

"coddlers"

"coddles"

"coddling"

"code"

"coded"

"codefendant"

"codefendants"

"codein"

"codeine"

"codeines"

"codeins"

"codeless"

"coder"

"coders"

"codes"

"codeword"

"codex"

"codfish"

"codfishes"

"codger"

"codgers"

"codices"

"codicil"

"codicils"

"codicology"

"codification"

"codifications"

"codified"

"codifier"

"codifiers"

"codifies"

"codify"

"codifying"

"coding"

"codings"

"codling"

"codlings"

"codon"

"codons"

"codpiece"

"codpieces"

"cods"

"coed"

"coeditor"

"coeditors"

"coeds"

"coeducation"

"coeducational"

"coeducationally"

"coefficient"

"coefficients"

"coelenterate"

"coelenterates"

"coempt"

"coempts"

"coenact"

"coenamored"

"coenzyme"

"coequal"

"coequality"

"coequally"

"coequals"

"coequate"

"coequating"

"coerce"

"coerced"

"coercer"

"coercers"

"coerces"

"coercible"

"coercimeter"

"coercing"

"coercion"

"coercions"

"coercive"

"coercively"

"coerciveness"

"coeval"

"coevally"

"coevals"

"coexist"

"coexisted"

"coexistence"

"coexistent"

"coexisting"

"coexists"

"coextended"

"coextensive"

"coextensively"

"cofeature"

"cofeatures"

"coffee"

"coffeecake"

"coffeecakes"

"coffeehouse"

"coffeehouses"

"coffeepot"

"coffeepots"

"coffees"

"coffer"

"cofferdam"

"cofferdams"

"coffered"

"coffering"

"coffers"

"coffin"

"coffined"

"coffing"

"coffining"

"coffins"

"coffs"

"cog"

"cogence"

"cogences"

"cogencies"

"cogency"

"cogent"

"cogently"

"cogged"

"cogging"

"cogitate"

"cogitated"

"cogitates"

"cogitating"

"cogitation"

"cogitations"

"cogitative"

"cogitator"

"cogitators"

"cogito"

"cogitos"

"cognac"

"cognacs"

"cognate"

"cognates"

"cognati"

"cognation"

"cognisable"

"cognisance"

"cognise"

"cognised"

"cognises"

"cognising"

"cognition"

"cognitional"

"cognitive"

"cognitive abilities"

"cognitive behavioral therapy"

"cognizable"

"cognizably"

"cognizance"

"cognizant"

"cognize"

"cognized"

"cognizer"

"cognizers"

"cognizes"

"cognizing"

"cognomen"

"cognomens"

"cognomina"

"cognoscente"

"cognoscenti"

"cognoscing"

"cogs"

"cogway"

"cogwheel"

"cogwheels"

"cohabit"

"cohabitant"

"cohabitation"

"cohabited"

"cohabiting"

"cohabits"

"coheir"

"coheirs"

"cohen"

"cohere"

"cohered"

"coherence"

"coherency"

"coherent"

"coherently"

"coherer"

"coherers"

"coheres"

"cohering"

"cohesion"

"cohesions"

"cohesive"

"cohesively"

"cohesiveness"

"coho"

"cohort"

"cohorts"

"cohos"

"cohosh"

"cohoshes"

"coif"

"coifed"

"coiffed"

"coiffes"

"coiffeur"

"coiffeurs"

"coiffeuse"

"coiffeuses"

"coiffing"

"coiffure"

"coiffured"

"coiffures"

"coiffuring"

"coifing"

"coifs"

"coign"

"coigne"

"coigns"

"coil"

"coiled"

"coiler"

"coilers"

"coiling"

"coils"

"coin"

"coinable"

"coinage"

"coinages"

"coincide"

"coincided"

"coincidence"

"coincidences"

"coincident"

"coincidental"

"coincidentally"

"coincides"

"coinciding"

"coined"

"coiner"

"coiners"

"coinferred"

"coinhering"

"coining"

"coins"

"coinsurance"

"coinsured"

"coinsurer"

"coinsures"

"coinsuring"

"cointerred"

"coir"

"coirs"

"coital"

"coitally"

"coition"

"coitional"

"coitions"

"coitophobia"

"coitus"

"coituses"

"coke"

"coked"

"cokes"

"coking"

"col"

"cola"

"colander"

"colanders"

"colas"

"cold"

"colder"

"coldest"

"coldish"

"coldly"

"coldness"

"colds"

"cole"

"coleopterology"

"coles"

"coleslaw"

"coleslaws"

"coleus"

"coleuses"

"colewort"

"colic"

"colicky"

"colics"

"coliform"

"coliforms"

"colin"

"colinear"

"coliseum"

"coliseums"

"colitic"

"colitis"

"colitises"

"coll"

"collaborate"

"collaborated"

"collaborates"

"collaborating"

"collaboration"

"collaborationism"

"collaborationist"

"collaborationists"

"collaborations"

"collaborative"

"collaborator"

"collaborators"

"collage"

"collagen"

"collagens"

"collages"

"collapse"

"collapsed"

"collapses"

"collapsibility"

"collapsible"

"collapsing"

"collar"

"collarbone"

"collarbones"

"collard"

"collards"

"collared"

"collaring"

"collarless"

"collars"

"collat"

"collate"

"collated"

"collateral"

"collateralizing"

"collaterally"

"collaterals"

"collates"

"collating"

"collation"

"collations"

"collator"

"collators"

"colleague"

"colleagues"

"collect"

"collectable"

"collectables"

"collected"

"collectedly"

"collectible"

"collectibles"

"collecting"

"collection"

"collections"

"collective"

"collectively"

"collectives"

"collectivism"

"collectivist"

"collectivists"

"collectivize"

"collectivized"

"collectivizes"

"collectivizing"

"collector"

"collectors"

"collects"

"colleen"

"colleens"

"college"

"colleger"

"colleges"

"collegia"

"collegial"

"collegiality"

"collegially"

"collegian"

"collegians"

"collegiate"

"collegium"

"collegiums"

"colleted"

"collets"

"collide"

"collided"

"collides"

"colliding"

"collie"

"collied"

"collier"

"collieries"

"colliers"

"colliery"

"collies"

"collimate"

"collimating"

"collimation"

"collinear"

"collins"

"collinses"

"collision"

"collisions"

"collocate"

"collocated"

"collocates"

"collocating"

"collocation"

"collocations"

"collodion"

"collodium"

"colloid"

"colloidal"

"colloids"

"collop"

"collops"

"colloq"

"colloquia"

"colloquial"

"colloquialism"

"colloquialisms"

"colloquially"

"colloquies"

"colloquium"

"colloquiums"

"colloquy"

"collude"

"colluded"

"colluder"

"colluders"

"colludes"

"colluding"

"collusion"

"collusive"

"collusively"

"colluvial"

"colluvium"

"colly"

"colocate"

"cologne"

"cologned"

"colognes"

"cologs"

"colombia"

"colombian"

"colombians"

"colombo"

"colon"

"colonel"

"colonelcies"

"colonelcy"

"colonels"

"colonelship"

"colonelships"

"colones"

"colonial"

"colonialism"

"colonialist"

"colonialists"

"colonially"

"colonials"

"colonic"

"colonies"

"colonise"

"colonist"

"colonists"

"colonization"

"colonizationist"

"colonizations"

"colonize"

"colonized"

"colonizer"

"colonizers"

"colonizes"

"colonizing"

"colonnade"

"colonnaded"

"colonnades"

"colonoscope"

"colons"

"colony"

"colophon"

"colophons"

"color"

"Color Test"

"colorable"

"colorably"

"coloradan"

"coloradans"

"colorado"

"colorant"

"colorants"

"coloration"

"colorations"

"coloratura"

"coloraturas"

"colorblind"

"colorcast"

"colorcasting"

"colorcasts"

"colored"

"coloreds"

"colorer"

"colorers"

"colorfast"

"colorfastness"

"colorful"

"colorfully"

"colorfulness"

"colorimeter"

"colorimetry"

"coloring"

"colorings"

"colorism"

"colorisms"

"colorist"

"colorists"

"colorless"

"colors"

"colossal"

"colossally"

"colosseum"

"colossi"

"colossians"

"colossus"

"colossuses"

"colostomies"

"colostomy"

"colostrum"

"colour"

"coloured"

"colourer"

"colourers"

"colouring"

"colours"

"colporteur"

"colporteurs"

"colposcope"

"colt"

"colters"

"coltish"

"colts"

"columbia"

"columbian"

"columbic"

"columbine"

"columbines"

"columbium"

"columbus"

"column"

"columnal"

"columnar"

"columned"

"columnist"

"columnists"

"columns"

"colure"

"colures"

"com"

"coma"

"comanche"

"comanches"

"comas"

"comatose"

"comb"

"combat"

"combat zones"

"combatant"

"combatants"

"combated"

"combater"

"combaters"

"combating"

"combative"

"combatively"

"combativeness"

"combats"

"combattant"

"combatted"

"combatting"

"combe"

"combed"

"comber"

"combers"

"combes"

"combination"

"combinations"

"combine"

"combined"

"combiner"

"combiners"

"combines"

"combing"

"combings"

"combining"

"combo"

"combos"

"combs"

"combust"

"combusted"

"combustibilities"

"combustibility"

"combustible"

"combustibles"

"combustibly"

"combusting"

"combustion"

"combustive"

"combustively"

"combusts"

"come"

"comeback"

"comebacks"

"comedian"

"comedians"

"comedic"

"comedienne"

"comediennes"

"comedies"

"comedo"

"comedones"

"comedos"

"comedown"

"comedowns"

"comedy"

"comelier"

"comeliest"

"comeliness"

"comely"

"comer"

"comers"

"comes"

"comestible"

"comestibles"

"comet"

"cometary"

"cometh"

"cometic"

"cometology"

"comets"

"comeuppance"

"comeuppances"

"comfier"

"comfiest"

"comfit"

"comfits"

"comfort"

"comfortable"

"comfortableness"

"comfortably"

"comforted"

"comforter"

"comforters"

"comforting"

"comfortingly"

"comfortless"

"comforts"

"comfrey"

"comfreys"

"comfy"

"comic"

"comical"

"comicality"

"comically"

"comics"

"coming"

"comings"

"comities"

"comity"

"comma"

"command"

"commandant"

"commandants"

"commanded"

"commandeer"

"commandeered"

"commandeering"

"commandeers"

"commander"

"commanders"

"commanding"

"commandment"

"commandments"

"commando"

"commandoes"

"commandos"

"commands"

"commas"

"comme"

"commemorate"

"commemorated"

"commemorates"

"commemorating"

"commemoration"

"commemorations"

"commemorative"

"commemoratively"

"commemorator"

"commemorators"

"commence"

"commenced"

"commencement"

"commencements"

"commences"

"commencing"

"commend"

"commendable"

"commendably"

"commendation"

"commendations"

"commendatorily"

"commendatory"

"commended"

"commending"

"commends"

"commensurable"

"commensurably"

"commensurate"

"commensurately"

"commensuration"

"commensurations"

"comment"

"commentaries"

"commentary"

"commentate"

"commentator"

"commentators"

"commented"

"commenting"

"comments"

"commerce"

"commerced"

"commerces"

"commercial"

"commercialism"

"commercialist"

"commercialists"

"commercialization"

"commercializations"

"commercialize"

"commercialized"

"commercializes"

"commercializing"

"commercially"

"commercials"

"commercing"

"commie"

"commies"

"commination"

"comminatory"

"commingle"

"commingled"

"commingles"

"commingling"

"comminute"

"commiserate"

"commiserated"

"commiserates"

"commiserating"

"commiseration"

"commiserations"

"commiserative"

"commiseratively"

"commissar"

"commissariat"

"commissariats"

"commissaries"

"commissars"

"commissary"

"commission"

"commissioned"

"commissioner"

"commissioners"

"commissionership"

"commissionerships"

"commissioning"

"commissions"

"commit"

"commitment"

"commitments"

"commits"

"committable"

"committal"

"committals"

"committed"

"committee"

"committeeman"

"committeemen"

"committees"

"committeewoman"

"committeewomen"

"committing"

"commix"

"commixed"

"commixes"

"commixing"

"commixt"

"commode"

"commodes"

"commodious"

"commodiously"

"commodiousness"

"commodities"

"commodity"

"commodore"

"commodores"

"common"

"common people"

"commonable"

"commonalities"

"commonality"

"commonalties"

"commonalty"

"commoner"

"commoners"

"commonest"

"commonly"

"commonly used"

"commonness"

"commonplace"

"commonplaces"

"commons"

"commonsensical"

"commonweal"

"commonweals"

"commonwealth"

"commonwealths"

"commorancies"

"commotion"

"commotions"

"communal"

"communalism"

"communalist"

"communality"

"communalization"

"communalize"

"communalized"

"communally"

"communard"

"commune"

"communed"

"communes"

"communicability"

"communicable"

"communicableness"

"communicably"

"communicant"

"communicants"

"communicate"

"communicated"

"communicates"

"communicating"

"communication"

"communications"

"communicative"

"communicatively"

"communicativeness"

"communicator"

"communicators"

"communing"

"communion"

"communions"

"communique"

"communiques"

"communism"

"communist"

"communistic"

"communistically"

"communists"

"communities"

"community"

"commutable"

"commutation"

"commutations"

"commutative"

"commutatively"

"commutator"

"commutators"

"commute"

"commuted"

"commuter"

"commuters"

"commutes"

"commuting"

"commy"

"comp"

"compact"

"compacted"

"compacter"

"compactest"

"compacting"

"compaction"

"compactions"

"compactly"

"compactness"

"compactor"

"compactors"

"compacts"

"compadre"

"compadres"

"companied"

"companies"

"companion"

"companionable"

"companionably"

"companionless"

"companions"

"companionship"

"companionway"

"companionways"

"company"

"companying"

"comparability"

"comparable"

"comparably"

"comparative"

"comparatively"

"comparativeness"

"comparatives"

"compare"

"compared"

"comparer"

"comparers"

"compares"

"comparing"

"Comparing prescriptions"

"comparison"

"comparisons"

"compartment"

"compartmental"

"compartmentalize"

"compartmentalized"

"compartmentalizes"

"compartmentalizing"

"compartmentally"

"compartmented"

"compartments"

"comparts"

"compass"

"compassed"

"compasses"

"compassing"

"compassion"

"compassionate"

"compassionately"

"compatibilities"

"compatibility"

"compatible"

"compatible with Jelly Bean"

"compatibleness"

"compatibles"

"compatibly"

"compatriot"

"compatriots"

"comped"

"compeer"

"compeers"

"compel"

"compellable"

"compelled"

"compeller"

"compellers"

"compelling"

"compellingly"

"compels"

"compendia"

"compendium"

"compendiums"

"compends"

"compensability"

"compensable"

"compensate"

"compensated"

"compensates"

"compensating"

"compensation"

"compensations"

"compensative"

"compensatively"

"compensator"

"compensators"

"compensatory"

"compere"

"compered"

"comperes"

"compete"

"competed"

"competence"

"competencies"

"competency"

"competent"

"competently"

"competes"

"competing"

"competition"

"competitions"

"competitive"

"competitively"

"competitiveness"

"competitor"

"competitors"

"compilable"

"compilation"

"compilations"

"compile"

"compiled"

"compiler"

"compilers"

"compiles"

"compiling"

"comping"

"complacence"

"complacency"

"complacent"

"complacently"

"complain"

"complainant"

"complainants"

"complained"

"complainer"

"complainers"

"complaining"

"complains"

"complaint"

"complaints"

"complaisance"

"complaisant"

"complaisantly"

"compleat"

"complect"

"complected"

"complement"

"complemental"

"complementarily"

"complementariness"

"complementary"

"complemented"

"complementing"

"complements"

"complete"

"completed"

"completely"

"completeness"

"completer"

"completers"

"completes"

"completest"

"completing"

"completion"

"completions"

"complex"

"complexer"

"complexes"

"complexest"

"complexing"

"complexion"

"complexional"

"complexioned"

"complexions"

"complexities"

"complexity"

"complexness"

"compliance"

"compliances"

"compliancies"

"compliancy"

"compliant"

"compliantly"

"complicate"

"complicated"

"complicatedly"

"complicatedness"

"complicates"

"complicating"

"complication"

"complications"

"complicator"

"complicities"

"complicity"

"complied"

"complier"

"compliers"

"complies"

"compliment"

"complimentarily"

"complimentary"

"complimented"

"complimenter"

"complimenters"

"complimenting"

"compliments"

"complots"

"comply"

"complying"

"component"

"componential"

"components"

"comport"

"comported"

"comporting"

"comportment"

"comports"

"compos"

"compose"

"composed"

"composedly"

"composedness"

"composer"

"composers"

"composes"

"composing"

"composite"

"compositely"

"composites"

"composition"

"compositions"

"compositor"

"compositors"

"compost"

"composted"

"composting"

"composts"

"composure"

"compote"

"compotes"

"compound"

"compoundable"

"compounded"

"compounder"

"compounders"

"compounding"

"compounds"

"comprehend"

"comprehended"

"comprehendible"

"comprehending"

"comprehends"

"comprehensibility"

"comprehensible"

"comprehensibleness"

"comprehensibly"

"comprehension"

"comprehensive"

"comprehensively"

"comprehensiveness"

"compress"

"compressed"

"compressedly"

"compresses"

"compressibility"

"compressible"

"compressing"

"compression"

"compressional"

"compressions"

"compressive"

"compressively"

"compressor"

"compressors"

"comprise"

"comprised"

"comprises"

"comprising"

"comprize"

"comprized"

"comprizes"

"comprizing"

"compromisable"

"compromise"

"compromised"

"compromiser"

"compromisers"

"compromises"

"compromising"

"comps"

"compt"

"compte"

"compted"

"compting"

"comptroller"

"comptrollers"

"compts"

"compulsion"

"compulsions"

"compulsive"

"compulsively"

"compulsiveness"

"compulsives"

"compulsorily"

"compulsory"

"compunction"

"compunctions"

"computability"

"computable"

"computation"

"computational"

"computations"

"compute"

"computed"

"computer"

"computerese"

"computerization"

"computerize"

"computerized"

"computerizes"

"computerizing"

"computers"

"computes"

"computing"

"comrade"

"comradely"

"comrades"

"comradeship"

"comsat"

"comte"

"comtes"

"con"

"conation"

"conative"

"concatenate"

"concatenated"

"concatenates"

"concatenating"

"concatenation"

"concatenations"

"concave"

"concaved"

"concaveness"

"concaves"

"concaving"

"concavities"

"concavity"

"concavo"

"conceal"

"concealable"

"concealed"

"concealer"

"concealers"

"concealing"

"concealment"

"conceals"

"concede"

"conceded"

"concededly"

"conceder"

"conceders"

"concedes"

"conceding"

"conceit"

"conceited"

"conceitedly"

"conceitedness"

"conceiting"

"conceits"

"conceivability"

"conceivable"

"conceivableness"

"conceivably"

"conceive"

"conceived"

"conceiver"

"conceivers"

"conceives"

"conceiving"

"conceiving a baby"

"concelebrate"

"concelebrated"

"concelebrates"

"concelebrating"

"concelebration"

"concelebrations"

"concentrate"

"concentrated"

"concentrates"

"concentrating"

"concentration"

"concentrations"

"concentrative"

"concentrator"

"concentrators"

"concentric"

"concentrically"

"concentricity"

"concents"

"concept"

"conception"

"conceptional"

"conceptions"

"conceptive"

"concepts"

"conceptual"

"conceptualism"

"conceptualist"

"conceptualistic"

"conceptualists"

"conceptualization"

"conceptualizations"

"conceptualize"

"conceptualized"

"conceptualizes"

"conceptualizing"

"conceptually"

"concern"

"concerned"

"concerning"

"concernment"

"concerns"

"concert"

"concerted"

"concertedly"

"concerti"

"concertina"

"concertinas"

"concerting"

"concertize"

"concertized"

"concertizes"

"concertizing"

"concertmaster"

"concertmasters"

"concerto"

"concertos"

"concerts"

"concession"

"concessionaire"

"concessionaires"

"concessions"

"concessive"

"conch"

"conches"

"conchoid"

"conchology"

"conchs"

"conchy"

"concierge"

"concierges"

"conciliar"

"conciliate"

"conciliated"

"conciliates"

"conciliating"

"conciliation"

"conciliations"

"conciliator"

"conciliators"

"conciliatory"

"concise"

"concisely"

"conciseness"

"conciser"

"concisest"

"conclave"

"conclaves"

"conclude"

"concluded"

"concluder"

"concluders"

"concludes"

"concluding"

"conclusion"

"conclusions"

"conclusive"

"conclusively"

"conclusiveness"

"concoct"

"concocted"

"concocting"

"concoction"

"concoctions"

"concocts"

"concomitance"

"concomitant"

"concomitantly"

"concord"

"concordance"

"concordances"

"concordant"

"concordantly"

"concordat"

"concordats"

"concords"

"concourse"

"concourses"

"concrescence"

"concrescences"

"concrescent"

"concrete"

"concreted"

"concretely"

"concreteness"

"concretes"

"concreting"

"concretion"

"concretions"

"concubinage"

"concubine"

"concubines"

"concupiscence"

"concupiscent"

"concur"

"concurred"

"concurrence"

"concurrences"

"concurrent"

"concurrently"

"concurring"

"concurs"

"concuss"

"concussed"

"concusses"

"concussing"

"concussion"

"concussions"

"concussive"

"concussively"

"condemn"

"condemnable"

"condemnation"

"condemnations"

"condemnatory"

"condemned"

"condemner"

"condemners"

"condemning"

"condemnor"

"condemns"

"condensate"

"condensates"

"condensation"

"condensations"

"condense"

"condensed"

"condenser"

"condensers"

"condenses"

"condensing"

"condescend"

"condescended"

"condescendence"

"condescending"

"condescendingly"

"condescends"

"condescension"

"condign"

"condignly"

"condiment"

"condiments"

"condition"

"conditional"

"conditionalities"

"conditionality"

"conditionally"

"conditionals"

"conditione"

"conditioned"

"conditioner"

"conditioners"

"conditioning"

"conditions"

"condo"

"condole"

"condoled"

"condolence"

"condolences"

"condoler"

"condolers"

"condoles"

"condoling"

"condom"

"condominium"

"condominiums"

"condoms"

"condonable"

"condonation"

"condonations"

"condone"

"condoned"

"condoner"

"condoners"

"condones"

"condoning"

"condor"

"condores"

"condors"

"condos"

"conduce"

"conduced"

"conducer"

"conducers"

"conduces"

"conducing"

"conducive"

"conduciveness"

"conduct"

"conductance"

"conductances"

"conducted"

"conductibility"

"conductible"

"conducting"

"conduction"

"conductive"

"conductivities"

"conductivity"

"conductor"

"conductors"

"conducts"

"conduit"

"conduits"

"condyle"

"condyles"

"cone"

"coned"

"conelrad"

"conelrads"

"cones"

"conestoga"

"coney"

"coneys"

"conf"

"confab"

"confabbed"

"confabbing"

"confabs"

"confabulate"

"confabulated"

"confabulates"

"confabulating"

"confabulation"

"confabulations"

"confect"

"confecting"

"confection"

"confectioner"

"confectioneries"

"confectioners"

"confectionery"

"confectiones"

"confections"

"confects"

"confederacies"

"confederacy"

"confederate"

"confederated"

"confederates"

"confederating"

"confederation"

"confederations"

"confederative"

"confer"

"conferee"

"conferees"

"conference"

"conferences"

"conferment"

"conferred"

"conferrer"

"conferrers"

"conferring"

"confers"

"confess"

"confessable"

"confessed"

"confessedly"

"confesses"

"confessing"

"confession"

"confessional"

"confessionals"

"confessions"

"confessor"

"confessors"

"confetti"

"confetto"

"confidant"

"confidante"

"confidantes"

"confidants"

"confide"

"confided"

"confidence"

"confidences"

"confident"

"confidential"

"confidentiality"

"confidentially"

"confidentialness"

"confidently"

"confider"

"confiders"

"confides"

"confiding"

"configurable"

"configuration"

"configurational"

"configurations"

"configurative"

"configure"

"configured"

"configuring"

"confine"

"confined"

"confinement"

"confinements"

"confiner"

"confiners"

"confines"

"confining"

"confirm"

"confirmable"

"confirmation"

"confirmations"

"confirmatory"

"confirmed"

"confirming"

"confirmor"

"confirms"

"confiscate"

"confiscated"

"confiscates"

"confiscating"

"confiscation"

"confiscations"

"confiscator"

"confiscators"

"confiscatory"

"conflagration"

"conflagrations"

"conflict"

"conflicted"

"conflicting"

"conflictive"

"conflicts"

"confluence"

"confluences"

"confluent"

"conflux"

"confocal"

"conform"

"conformable"

"conformably"

"conformation"

"conformational"

"conformationally"

"conformations"

"conformed"

"conformer"

"conformers"

"conforming"

"conformism"

"conformist"

"conformists"

"conformities"

"conformity"

"conforms"

"confound"

"confounded"

"confoundedly"

"confounder"

"confounders"

"confounding"

"confounds"

"confraternities"

"confraternity"

"confrere"

"confreres"

"confront"

"confrontation"

"confrontations"

"confronted"

"confronting"

"confronts"

"confucian"

"confucianism"

"confucians"

"confucius"

"confuse"

"confused"

"confusedly"

"confuses"

"confusing"

"confusingly"

"confusion"

"confusional"

"confusions"

"confutable"

"confutation"

"confutations"

"confutative"

"confutator"

"confute"

"confuted"

"confuter"

"confuters"

"confutes"

"confuting"

"conga"

"congaed"

"congaing"

"congas"

"congeal"

"congealable"

"congealed"

"congealing"

"congealment"

"congeals"

"congee"

"congeed"

"congees"

"congener"

"congeneric"

"congeners"

"congenial"

"congeniality"

"congenially"

"congenital"

"congenitally"

"conger"

"congeries"

"congers"

"congest"

"congested"

"congesting"

"congestion"

"congestions"

"congestive"

"congests"

"conglomerate"

"conglomerated"

"conglomerates"

"conglomerating"

"conglomeration"

"conglomerations"

"congo"

"congoes"

"congolese"

"congos"

"congratulate"

"congratulated"

"congratulates"

"congratulating"

"congratulation"

"congratulations"

"congratulatory"

"congregant"

"congregants"

"congregate"

"congregated"

"congregates"

"congregating"

"congregation"

"congregational"

"congregations"

"congress"

"congressed"

"congresses"

"congressional"

"congressionally"

"congressman"

"congressmen"

"congresswoman"

"congresswomen"

"congruence"

"congruences"

"congruencies"

"congruency"

"congruent"

"congruently"

"congruities"

"congruity"

"congruous"

"congruously"

"conic"

"conical"

"conically"

"conicity"

"conics"

"conies"

"conifer"

"coniferous"

"conifers"

"conj"

"conjecturable"

"conjectural"

"conjecture"

"conjectured"

"conjectures"

"conjecturing"

"conjoin"

"conjoined"

"conjoining"

"conjoins"

"conjoint"

"conjointly"

"conjoints"

"conjugal"

"conjugality"

"conjugally"

"conjugant"

"conjugate"

"conjugated"

"conjugates"

"conjugating"

"conjugation"

"conjugational"

"conjugations"

"conjugator"

"conjugators"

"conjunct"

"conjunction"

"conjunctions"

"conjunctiva"

"conjunctivae"

"conjunctival"

"conjunctivas"

"conjunctive"

"conjunctives"

"conjunctivitis"

"conjuncts"

"conjuncture"

"conjunctures"

"conjuration"

"conjurations"

"conjure"

"conjured"

"conjurer"

"conjurers"

"conjures"

"conjuring"

"conjuror"

"conjurors"

"conk"

"conked"

"conker"

"conkers"

"conking"

"conks"

"conky"

"conn"

"connate"

"connect"

"connected"

"connectedly"

"connecter"

"connecters"

"connecticut"

"connecting"

"connection"

"connections"

"connective"

"connectively"

"connectives"

"connector"

"connectors"

"connects"

"conned"

"conner"

"conners"

"connie"

"conning"

"conniption"

"conniptions"

"connivance"

"connive"

"connived"

"conniver"

"connivers"

"connivery"

"connives"

"conniving"

"connoisseur"

"connoisseurs"

"connotation"

"connotations"

"connotative"

"connote"

"connoted"

"connotes"

"connoting"

"conns"

"connubial"

"conoid"

"conoidal"

"conoids"

"conquer"

"conquerable"

"conquered"

"conquering"

"conqueror"

"conquerors"

"conquers"

"conquest"

"conquests"

"conquian"

"conquistador"

"conquistadors"

"conrail"

"cons"

"consanguine"

"consanguineous"

"consanguinities"

"consanguinity"

"conscience"

"conscienceless"

"consciences"

"conscientious"

"conscientiously"

"conscientiousness"

"conscious"

"consciously"

"consciousness"

"conscript"

"conscripted"

"conscripting"

"conscription"

"conscripts"

"conscripttion"

"consecrate"

"consecrated"

"consecrates"

"consecrating"

"consecration"

"consecrations"

"consecrative"

"consecrator"

"consecratory"

"consecutive"

"consecutively"

"consecutiveness"

"consensual"

"consensually"

"consensus"

"consensuses"

"consent"

"consented"

"consenter"

"consenters"

"consenting"

"consents"

"consequence"

"consequences"

"consequent"

"consequential"

"consequentially"

"consequently"

"conservable"

"conservancy"

"conservation"

"conservational"

"conservationism"

"conservationist"

"conservationists"

"conservatism"

"conservative"

"conservatively"

"conservatives"

"conservator"

"conservatories"

"conservators"

"conservatorship"

"conservatory"

"conserve"

"conserved"

"conserves"

"conserving"

"consider"

"considerable"

"considerably"

"considerate"

"considerately"

"consideration"

"considerations"

"considered"

"considering"

"considers"

"consign"

"consignataries"

"consigned"

"consignee"

"consignees"

"consigning"

"consignment"

"consignments"

"consignor"

"consignors"

"consigns"

"consist"

"consisted"

"consistence"

"consistences"

"consistencies"

"consistency"

"consistent"

"consistently"

"consisting"

"consistorial"

"consistories"

"consistory"

"consists"

"consitutional"

"consolation"

"consolations"

"consolatory"

"console"

"consoled"

"consoler"

"consolers"

"consoles"

"consolidate"

"consolidated"

"consolidates"

"consolidating"

"consolidation"

"consolidations"

"consolidator"

"consolidators"

"consoling"

"consolingly"

"consomme"

"consommes"

"consonance"

"consonances"

"consonant"

"consonantal"

"consonantly"

"consonants"

"consort"

"consorted"

"consortia"

"consorting"

"consortium"

"consortiums"

"consorts"

"consortship"

"conspectus"

"conspectuses"

"conspicuous"

"conspicuously"

"conspicuousness"

"conspiracies"

"conspiracy"

"conspirator"

"conspiratorial"

"conspiratorially"

"conspirators"

"conspire"

"conspired"

"conspirer"

"conspirers"

"conspires"

"conspiring"

"conspiringly"

"constable"

"constables"

"constabularies"

"constabulary"

"constance"

"constancy"

"constant"

"constantinople"

"constantly"

"constants"

"constellation"

"constellations"

"consternate"

"consternation"

"constipate"

"constipated"

"constipates"

"constipating"

"constipation"

"constituencies"

"constituency"

"constituent"

"constituently"

"constituents"

"constitute"

"constituted"

"constitutes"

"constituting"

"constitution"

"constitutional"

"constitutionality"

"constitutionally"

"constitutionals"

"constitutions"

"constitutive"

"constrain"

"constrainable"

"constrained"

"constrainedly"

"constrainer"

"constrainers"

"constraining"

"constrainment"

"constrains"

"constraint"

"constraints"

"constrict"

"constricted"

"constricting"

"constriction"

"constrictions"

"constrictive"

"constrictor"

"constrictors"

"constricts"

"construable"

"construct"

"constructed"

"constructing"

"construction"

"constructionism"

"constructionist"

"constructionists"

"constructions"

"constructive"

"constructively"

"constructiveness"

"constructor"

"constructors"

"constructs"

"construe"

"construed"

"construer"

"construers"

"construes"

"construing"

"consubstantiation"

"consul"

"consular"

"consulate"

"consulates"

"consulating"

"consuls"

"consulship"

"consulships"

"consult"

"consult record"

"consultant"

"consultants"

"consultation"

"consultations"

"consultative"

"consultatory"

"consulted"

"consulter"

"consulting"

"consultive"

"consults"

"consumable"

"consume"

"consumed"

"consumer"

"consumerism"

"consumers"

"consumes"

"consuming"

"consummate"

"consummated"

"consummately"

"consummates"

"consummating"

"consummation"

"consummations"

"consummator"

"consummatory"

"consumption"

"consumptions"

"consumptive"

"consumptively"

"consumptiveness"

"consumptives"

"cont"

"contact"

"contacted"

"contacting"

"contacts"

"contagion"

"contagions"

"contagious"

"contagiously"

"contagiousness"

"contain"

"containable"

"contained"

"container"

"containerization"

"containerize"

"containerized"

"containerizes"

"containerizing"

"containers"

"containership"

"containerships"

"containing"

"containment"

"containments"

"contains"

"contaminant"

"contaminants"

"contaminate"

"contaminated"

"contaminates"

"contaminating"

"contamination"

"contaminations"

"contaminative"

"contaminator"

"conte"

"contemn"

"contemned"

"contemner"

"contemnor"

"contemns"

"contemplate"

"contemplated"

"contemplates"

"contemplating"

"contemplation"

"contemplations"

"contemplative"

"contemplatively"

"contemplator"

"contemplators"

"contemporaneous"

"contemporaneously"

"contemporaries"

"contemporarily"

"contemporary"

"contempt"

"contemptible"

"contemptibly"

"contempts"

"contemptuous"

"contemptuously"

"contemptuousness"

"contend"

"contended"

"contender"

"contendere"

"contenders"

"contending"

"contends"

"content"

"contented"

"contentedly"

"contentedness"

"contenting"

"contention"

"contentional"

"contentions"

"contentious"

"contentiously"

"contentiousness"

"contently"

"contentment"

"contents"

"conterminous"

"conterminously"

"conterminousness"

"contes"

"contest"

"contestable"

"contestably"

"contestant"

"contestants"

"contestation"

"contested"

"contestee"

"contesting"

"contests"

"context"

"contexts"

"contextual"

"contextually"

"contiguities"

"contiguity"

"contiguous"

"contiguously"

"contiguousness"

"continence"

"continent"

"continental"

"continentally"

"continents"

"contingence"

"contingencies"

"contingency"

"contingent"

"contingentiam"

"contingently"

"contingents"

"continua"

"continuable"

"continual"

"continually"

"continuance"

"continuances"

"continuant"

"continuation"

"continuations"

"continue"

"continued"

"continuer"

"continuers"

"continues"

"continuing"

"continuities"

"continuity"

"continuo"

"continuos"

"continuous"

"continuously"

"continuousness"

"continuum"

"conto"

"contort"

"contorted"

"contorting"

"contortion"

"contortionist"

"contortionistic"

"contortionists"

"contortions"

"contortive"

"contorts"

"contour"

"contoured"

"contouring"

"contours"

"contra"

"contraband"

"contraception"

"contraceptive"

"contraceptives"

"contract"

"contracted"

"contractibility"

"contractible"

"contractile"

"contractility"

"contracting"

"contraction"

"contractions"

"contractive"

"contractor"

"contractors"

"contracts"

"contractual"

"contractually"

"contracture"

"contradict"

"contradicted"

"contradicting"

"contradiction"

"contradictions"

"contradictive"

"contradictively"

"contradictorily"

"contradictory"

"contradicts"

"contradistinction"

"contradistinctions"

"contradistinctive"

"contrail"

"contrails"

"contraindicate"

"contraindicated"

"contraindicates"

"contraindicating"

"contraindication"

"contraindications"

"contraindicative"

"contraire"

"contralto"

"contraltos"

"contraption"

"contraptions"

"contrapuntal"

"contraries"

"contrarieties"

"contrariety"

"contrarily"

"contrariness"

"contrariwise"

"contrary"

"contrast"

"contrastable"

"contrasted"

"contrasting"

"contrastingly"

"contrasts"

"contravene"

"contravened"

"contravenes"

"contravening"

"contravention"

"contretemps"

"contribute"

"contributed"

"contributes"

"contributing"

"contribution"

"contributions"

"contributor"

"contributories"

"contributorily"

"contributors"

"contributory"

"contrite"

"contritely"

"contriteness"

"contrition"

"contrivance"

"contrivances"

"contrive"

"contrived"

"contrivedly"

"contriver"

"contrivers"

"contrives"

"contriving"

"control"

"control of insomnia"

"control of your cravings"

"controllability"

"controllable"

"controllably"

"controlled"

"controller"

"controllers"

"controlling"

"controls"

"controversial"

"controversially"

"controversies"

"controversy"

"controvert"

"controverted"

"controvertible"

"controverting"

"controverts"

"contumacious"

"contumaciously"

"contumacy"

"contumelies"

"contumelious"

"contumely"

"contuse"

"contused"

"contuses"

"contusing"

"contusion"

"contusions"

"conundrum"

"conundrums"

"conurbation"

"conurbations"

"conus"

"convalesce"

"convalesced"

"convalescence"

"convalescent"

"convalescents"

"convalesces"

"convalescing"

"convect"

"convected"

"convecting"

"convection"

"convectional"

"convective"

"convects"

"convene"

"convened"

"convener"

"conveners"

"convenes"

"convenience"

"conveniences"

"convenient"

"conveniently"

"convening"

"convent"

"convented"

"conventicle"

"conventicles"

"conventing"

"convention"

"conventional"

"conventionalism"

"conventionality"

"conventionalize"

"conventionalized"

"conventionalizes"

"conventionalizing"

"conventionally"

"conventionary"

"conventioneer"

"conventioneers"

"conventions"

"convents"

"conventual"

"converge"

"converged"

"convergence"

"convergency"

"convergent"

"converges"

"converging"

"conversant"

"conversation"

"conversational"

"conversationalist"

"conversationalists"

"conversationally"

"conversations"

"converse"

"conversed"

"conversely"

"converses"

"conversing"

"conversion"

"conversions"

"convert"

"converted"

"converter"

"converters"

"convertible"

"convertibles"

"converting"

"convertor"

"convertors"

"converts"

"convex"

"convexes"

"convexities"

"convexity"

"convexly"

"convexo"

"convey"

"conveyable"

"conveyance"

"conveyancer"

"conveyances"

"conveyancing"

"conveyed"

"conveyer"

"conveyers"

"conveying"

"conveyor"

"conveyors"

"conveys"

"convict"

"convicted"

"convicting"

"conviction"

"convictions"

"convicts"

"convince"

"convinced"

"convincer"

"convincers"

"convinces"

"convincing"

"convincingly"

"convivial"

"conviviality"

"convivially"

"convocation"

"convocations"

"convoke"

"convoked"

"convoker"

"convokers"

"convokes"

"convoking"

"convoluted"

"convolutely"

"convoluting"

"convolution"

"convolutions"

"convolvulus"

"convolvuluses"

"convoy"

"convoyed"

"convoying"

"convoys"

"convulsant"

"convulse"

"convulsed"

"convulses"

"convulsing"

"convulsion"

"convulsions"

"convulsive"

"convulsively"

"cony"

"coo"

"cooch"

"cooed"

"cooee"

"cooeeing"

"cooees"

"cooer"

"cooers"

"cooey"

"cooeyed"

"cooeying"

"cooeys"

"cooing"

"cooingly"

"cook"

"cookable"

"cookbook"

"cookbooks"

"cooked"

"cooker"

"cookeries"

"cookers"

"cookery"

"cookey"

"cookeys"

"cookie"

"cookies"

"cooking"

"cooking directions"

"cooking timer"

"cookings"

"cookout"

"cookouts"

"cooks"

"cookshop"

"cookshops"

"cookware"

"cookwares"

"cooky"

"cool"

"coolant"

"coolants"

"cooled"

"cooler"

"coolers"

"coolest"

"cooley"

"coolidge"

"coolie"

"coolies"

"cooling"

"coolish"

"coolly"

"coolness"

"cools"

"cooly"

"coomb"

"coombe"

"coombes"

"coombs"

"coon"

"cooncan"

"coonhound"

"coonhounds"

"coons"

"coonskin"

"coonskins"

"coop"

"cooped"

"cooper"

"cooperage"

"cooperate"

"cooperated"

"cooperates"

"cooperating"

"cooperation"

"cooperations"

"cooperative"

"cooperatively"

"cooperativeness"

"cooperatives"

"cooperator"

"cooperators"

"coopered"

"coopering"

"coopers"

"coopery"

"cooping"

"coops"

"coopt"

"coopted"

"coopting"

"cooption"

"coopts"

"coordinate"

"coordinated"

"coordinately"

"coordinates"

"coordinating"

"coordination"

"coordinations"

"coordinative"

"coordinator"

"coordinators"

"coos"

"coot"

"cootie"

"cooties"

"coots"

"cop"

"copal"

"copals"

"coparent"

"coparents"

"copartner"

"copartners"

"copartnership"

"cope"

"copeck"

"coped"

"copenhagen"

"copepod"

"copepods"

"coper"

"copernican"

"copernicus"

"copers"

"copes"

"copied"

"copier"

"copiers"

"copies"

"copilot"

"copilots"

"coping"

"copings"

"copious"

"copiously"

"copiousness"

"coplanar"

"coplot"

"coplots"

"copolymer"

"copolymeric"

"copolymerization"

"copolymerizations"

"copolymerize"

"copolymerized"

"copolymerizing"

"copolymers"

"copout"

"copouts"

"copped"

"copper"

"copperas"

"coppered"

"copperhead"

"copperheads"

"coppering"

"copperplate"

"coppers"

"coppersmith"

"coppery"

"coppice"

"coppiced"

"coppices"

"copping"

"copra"

"copras"

"coprocessing"

"coprocessor"

"coprocessors"

"coprolith"

"coprology"

"copromania"

"cops"

"copse"

"copses"

"copter"

"copters"

"copula"

"copulae"

"copular"

"copulas"

"copulate"

"copulated"

"copulates"

"copulating"

"copulation"

"copulations"

"copulative"

"copulatively"

"copulatory"

"copy"

"copybook"

"copybooks"

"copyboy"

"copyboys"

"copycat"

"copycats"

"copycatted"

"copydesks"

"copyholder"

"copyholders"

"copying"

"copyist"

"copyists"

"copyreader"

"copyreaders"

"copyright"

"copyrightable"

"copyrighted"

"copyrighting"

"copyrights"

"copywriter"

"copywriters"

"coquet"

"coquetries"

"coquetry"

"coquets"

"coquette"

"coquetted"

"coquettes"

"coquetting"

"coquettish"

"coquettishly"

"coquinas"

"coracle"

"coracles"

"coral"

"corals"

"coram"

"corbel"

"corbeled"

"corbels"

"cord"

"cordage"

"cordages"

"cordate"

"corded"

"corder"

"corders"

"cordial"

"cordiality"

"cordially"

"cordialness"

"cordials"

"cordillera"

"cordilleran"

"cordilleras"

"cording"

"cordite"

"cordites"

"cordless"

"cordlessly"

"cordoba"

"cordobas"

"cordon"

"cordoned"

"cordoning"

"cordons"

"cordovan"

"cordovans"

"cords"

"corduroy"

"corduroys"

"cordwains"

"cordwood"

"cordwoods"

"core"

"core routine"

"cored"

"coredeemed"

"coreigns"

"corelate"

"corelating"

"coreless"

"corer"

"corers"

"cores"

"corespondent"

"corespondents"

"corgi"

"corgis"

"coriander"

"corianders"

"coring"

"corinthian"

"corinthians"

"cork"

"corkage"

"corkages"

"corked"

"corker"

"corkers"

"corkier"

"corkiest"

"corking"

"corks"

"corkscrew"

"corkscrewed"

"corkscrewing"

"corkscrews"

"corkwood"

"corkwoods"

"corky"

"corm"

"cormorant"

"cormorants"

"corms"

"corn"

"cornball"

"cornballs"

"cornbread"

"corncake"

"corncakes"

"corncob"

"corncobs"

"corncrib"

"corncribs"

"cornea"

"corneal"

"corneas"

"corned"

"cornel"

"cornell"

"cornels"

"corneous"

"corner"

"cornerback"

"cornered"

"cornering"

"corners"

"cornerstone"

"cornerstones"

"cornet"

"cornetist"

"cornetists"

"cornets"

"cornfed"

"cornfield"

"cornflower"

"cornflowers"

"cornhusk"

"cornhusks"

"cornice"

"corniced"

"cornices"

"corniche"

"cornier"

"corniest"

"cornify"

"cornily"

"corniness"

"corning"

"cornmeal"

"cornmeals"

"cornrow"

"cornrows"

"corns"

"cornstalk"

"cornstalks"

"cornstarch"

"cornu"

"cornucopia"

"cornucopian"

"cornucopias"

"cornucopiate"

"cornute"

"corny"

"corolla"

"corollaries"

"corollary"

"corollas"

"corona"

"coronach"

"coronachs"

"coronae"

"coronagraph"

"coronal"

"coronals"

"coronaries"

"coronary"

"coronary artery disease"

"coronas"

"coronation"

"coronations"

"coronels"

"coroner"

"coroners"

"coronet"

"coronets"

"corotate"

"corp"

"corpora"

"corporal"

"corporally"

"corporals"

"corporate"

"corporately"

"corporation"

"corporations"

"corporative"

"corpore"

"corporeal"

"corporeality"

"corporeally"

"corps"

"corpse"

"corpses"

"corpsman"

"corpsmen"

"corpulence"

"corpulences"

"corpulencies"

"corpulency"

"corpulent"

"corpulently"

"corpus"

"corpuscle"

"corpuscles"

"corpuscular"

"corral"

"corralled"

"corralling"

"corrals"

"correality"

"correct"

"correctable"

"corrected"

"correcter"

"correctest"

"correcting"

"correction"

"correctional"

"corrections"

"corrective"

"correctives"

"correctly"

"correctness"

"corrector"

"corrects"

"correl"

"correlatable"

"correlate"

"correlated"

"correlates"

"correlating"

"correlation"

"correlations"

"correlative"

"correlatives"

"correspond"

"corresponded"

"correspondence"

"correspondences"

"correspondent"

"correspondents"

"corresponding"

"correspondingly"

"corresponds"

"corrida"

"corridas"

"corridor"

"corridors"

"corrigenda"

"corrigendum"

"corrigibility"

"corrigible"

"corrigibly"

"corroborate"

"corroborated"

"corroborates"

"corroborating"

"corroboration"

"corroborations"

"corroborative"

"corroboratively"

"corroborator"

"corroborators"

"corroboratory"

"corrode"

"corroded"

"corroder"

"corroders"

"corrodes"

"corrodibility"

"corrodible"

"corroding"

"corrosion"

"corrosive"

"corrosively"

"corrosiveness"

"corrosives"

"corrugate"

"corrugated"

"corrugates"

"corrugating"

"corrugation"

"corrugations"

"corrugator"

"corrugators"

"corrupt"

"corrupted"

"corrupter"

"corruptest"

"corruptibilities"

"corruptibility"

"corruptible"

"corruptibleness"

"corruptibly"

"corrupting"

"corruption"

"corruptionist"

"corruptions"

"corruptive"

"corruptly"

"corruptness"

"corruptor"

"corrupts"

"corsage"

"corsages"

"corsair"

"corsairs"

"corse"

"corselet"

"corselets"

"corses"

"corset"

"corseted"

"corseting"

"corsets"

"corslet"

"corslets"

"cortege"

"corteges"

"cortex"

"cortexes"

"cortical"

"cortically"

"cortices"

"cortin"

"cortisone"

"corundum"

"corundums"

"coruscate"

"coruscated"

"coruscates"

"coruscating"

"coruscation"

"coruscations"

"coruscative"

"corvee"

"corvees"

"corves"

"corvet"

"corvets"

"corvette"

"corvettes"

"corvine"

"corymbs"

"coryza"

"coryzal"

"coryzas"

"cosec"

"cosecant"

"cosecants"

"cosecs"

"coset"

"cosets"

"cosey"

"coseys"

"cosh"

"coshed"

"cosher"

"coshered"

"coshers"

"coshes"

"coshing"

"cosie"

"cosier"

"cosies"

"cosiest"

"cosign"

"cosignatories"

"cosignatory"

"cosigned"

"cosigner"

"cosigners"

"cosigning"

"cosigns"

"cosily"

"cosine"

"cosines"

"cosiness"

"cosmetic"

"cosmetically"

"cosmetician"

"cosmetics"

"cosmetologist"

"cosmetologists"

"cosmetology"

"cosmic"

"cosmical"

"cosmically"

"cosmism"

"cosmisms"

"cosmist"

"cosmists"

"cosmo"

"cosmochemical"

"cosmochemistry"

"cosmogonic"

"cosmogonies"

"cosmogonist"

"cosmogonists"

"cosmogony"

"cosmological"

"cosmologist"

"cosmologists"

"cosmology"

"cosmonaut"

"cosmonauts"

"cosmopolis"

"cosmopolises"

"cosmopolitan"

"cosmopolitanism"

"cosmopolitans"

"cosmos"

"cosmoses"

"cosponsor"

"cosponsored"

"cosponsoring"

"cosponsors"

"cosponsorship"

"cosponsorships"

"cossack"

"cossacks"

"cosset"

"cosseted"

"cosseting"

"cossets"

"cost"

"costar"

"costard"

"costards"

"costarred"

"costarring"

"costed"

"coster"

"costers"

"costing"

"costive"

"costively"

"costiveness"

"costless"

"costlier"

"costliest"

"costliness"

"costly"

"costs"

"costum foods"

"costume"

"costumed"

"costumer"

"costumers"

"costumes"

"costumey"

"costumier"

"costumiers"

"costuming"

"cosy"

"cot"

"cotan"

"cotangent"

"cotangents"

"cotans"

"cote"

"coted"

"coterie"

"coteries"

"coterminous"

"cotes"

"cotillion"

"cotillions"

"cotillon"

"cots"

"cotta"

"cottage"

"cottager"

"cottagers"

"cottages"

"cottagey"

"cotter"

"cotters"

"cottiers"

"cotton"

"cottoned"

"cottoning"

"cottonmouth"

"cottonmouths"

"cottons"

"cottonseed"

"cottonseeds"

"cottontail"

"cottontails"

"cottonwood"

"cottonwoods"

"cottony"

"cotyledon"

"cotyledonal"

"cotyledonary"

"cotyledonous"

"cotyledons"

"couch"

"couchant"

"couchantly"

"couched"

"coucher"

"couchers"

"couches"

"couching"

"couchings"

"cougar"

"cougars"

"cough"

"coughed"

"cougher"

"coughers"

"coughing"

"coughs"

"could"

"couldest"

"couldst"

"coulee"

"coulees"

"coulomb"

"coulombmeter"

"coulombs"

"coulometer"

"coulter"

"coulters"

"council"

"councillor"

"councillorship"

"councilman"

"councilmen"

"councilor"

"councilors"

"councils"

"councilwoman"

"councilwomen"

"counsel"

"counselable"

"counseled"

"counselee"

"counseling"

"counsellable"

"counselled"

"counselling"

"counsellor"

"counsellors"

"counselor"

"counselors"

"counsels"

"count"

"countability"

"countable"

"countdown"

"countdown timer"

"countdowns"

"counted"

"countenance"

"countenanced"

"countenances"

"countenancing"

"counter"

"counteract"

"counteracted"

"counteracting"

"counteraction"

"counteractions"

"counteractive"

"counteractively"

"counteracts"

"counterattack"

"counterattacked"

"counterattacking"

"counterattacks"

"counterbalance"

"counterbalanced"

"counterbalances"

"counterbalancing"

"counterblow"

"counterclaim"

"counterclaimed"

"counterclaiming"

"counterclaims"

"counterclassification"

"counterclassifications"

"counterclockwise"

"counterculture"

"countercultures"

"countercurrent"

"countered"

"counterespionage"

"counterfeit"

"counterfeited"

"counterfeiter"

"counterfeiters"

"counterfeiting"

"counterfeitly"

"counterfeitness"

"counterfeits"

"countering"

"counterinsurgencies"

"counterinsurgency"

"counterinsurgent"

"counterinsurgents"

"counterintelligence"

"countermaid"

"counterman"

"countermand"

"countermanded"

"countermanding"

"countermands"

"countermeasure"

"countermeasures"

"countermen"

"counteroffensive"

"counteroffensives"

"counteroffer"

"counteropening"

"counterpane"

"counterpanes"

"counterpart"

"counterparts"

"counterphobic"

"counterplea"

"counterplot"

"counterplotted"

"counterplotting"

"counterpoint"

"counterpointed"

"counterpointing"

"counterpoints"

"counterpoise"

"counterpoised"

"counterpoises"

"counterpoising"

"counterproductive"

"counterrevolution"

"counterrevolutionaries"

"counterrevolutionary"

"counterrevolutions"

"counters"

"countersank"

"countershock"

"countersign"

"countersignature"

"countersignatures"

"countersigned"

"countersigning"

"countersigns"

"countersink"

"countersinking"

"countersinks"

"counterspies"

"counterspy"

"countersunk"

"countertenor"

"countertenors"

"countervail"

"countervailed"

"countervailing"

"countervails"

"counterweight"

"counterweights"

"countess"

"countesses"

"countian"

"counties"

"counting"

"countless"

"countries"

"countrified"

"country"

"countryman"

"countrymen"

"countryside"

"countrywide"

"countrywoman"

"countrywomen"

"counts"

"county"

"coup"

"coupe"

"couped"

"coupes"

"couping"

"couple"

"coupled"

"coupler"

"couplers"

"couples"

"couplet"

"couplets"

"coupling"

"couplings"

"coupon"

"coupons"

"coups"

"courage"

"courageous"

"courageously"

"courageousness"

"courages"

"courant"

"courante"

"courants"

"courier"

"couriers"

"course"

"coursed"

"courser"

"coursers"

"courses"

"coursing"

"coursings"

"court"

"courted"

"courteous"

"courteously"

"courteousness"

"courter"

"courters"

"courtesan"

"courtesans"

"courtesied"

"courtesies"

"courtesy"

"courthouse"

"courthouses"

"courtier"

"courtiers"

"courting"

"courtlier"

"courtliest"

"courtliness"

"courtly"

"courtroom"

"courtrooms"

"courts"

"courtship"

"courtships"

"courtyard"

"courtyards"

"couscous"

"couscouses"

"cousin"

"cousinly"

"cousinry"

"cousins"

"couth"

"couther"

"couthest"

"couthier"

"couths"

"couture"

"coutures"

"couturier"

"couturiere"

"couturieres"

"couturiers"

"covalence"

"covalences"

"covalent"

"covalently"

"cove"

"coved"

"coven"

"covenant"

"covenanted"

"covenantee"

"covenanting"

"covenantor"

"covenants"

"covens"

"cover"

"coverage"

"coverages"

"coverall"

"coveralls"

"covered"

"coverer"

"coverers"

"covering"

"coverings"

"coverlet"

"coverlets"

"coverlid"

"coverlids"

"covers"

"coverslip"

"covert"

"covertly"

"covertness"

"coverts"

"coverture"

"coverup"

"coverups"

"coves"

"covet"

"coveted"

"coveter"

"coveters"

"coveting"

"covetous"

"covetously"

"covetousness"

"covets"

"covey"

"coveys"

"coving"

"covings"

"cow"

"cowages"

"coward"

"cowardice"

"cowardliness"

"cowardly"

"cowards"

"cowbane"

"cowbell"

"cowbells"

"cowbird"

"cowbirds"

"cowboy"

"cowboys"

"cowcatcher"

"cowcatchers"

"cowed"

"cowedly"

"cower"

"cowered"

"cowering"

"cowers"

"cowfish"

"cowgirl"

"cowgirls"

"cowhand"

"cowhands"

"cowherb"

"cowherd"

"cowherds"

"cowhide"

"cowhided"

"cowhides"

"cowier"

"cowiest"

"cowing"

"cowkine"

"cowl"

"cowled"

"cowlick"

"cowlicks"

"cowling"

"cowlings"

"cowls"

"cowman"

"cowmen"

"coworker"

"coworkers"

"cowpat"

"cowpats"

"cowpea"

"cowpeas"

"cowpoke"

"cowpokes"

"cowpox"

"cowpoxes"

"cowpuncher"

"cowpunchers"

"cowrie"

"cowries"

"cowry"

"cows"

"cowshed"

"cowsheds"

"cowskin"

"cowskins"

"cowslip"

"cowslips"

"coxcomb"

"coxcombs"

"coxswain"

"coxswains"

"coxwain"

"coxwaining"

"coxwains"

"coy"

"coyer"

"coyest"

"coyish"

"coyly"

"coyness"

"coynesses"

"coyote"

"coyotes"

"coypu"

"coypus"

"cozen"

"cozenage"

"cozened"

"cozener"

"cozeners"

"cozening"

"cozens"

"cozes"

"cozey"

"cozeys"

"cozie"

"cozier"

"cozies"

"coziest"

"cozily"

"coziness"

"cozy"

"cpi"

"cpl"

"CPR"

"cps"

"cpu"

"cr"

"craal"

"craals"

"crab"

"crabapple"

"crabbed"

"crabbedness"

"crabber"

"crabbers"

"crabbier"

"crabbiest"

"crabbily"

"crabbiness"

"crabbing"

"crabby"

"crabgrass"

"crabs"

"crabwise"

"crack"

"crackdown"

"crackdowns"

"cracked"

"cracker"

"crackerjack"

"crackerjacks"

"crackers"

"cracking"

"crackings"

"crackle"

"crackled"

"crackles"

"cracklier"

"crackliest"

"crackling"

"crackly"

"cracknel"

"cracknels"

"crackpot"

"crackpots"

"cracks"

"cracksman"

"crackup"

"crackups"

"cracky"

"cradle"

"cradled"

"cradler"

"cradlers"

"cradles"

"cradlesong"

"cradlesongs"

"cradling"

"craft"

"crafted"

"craftier"

"craftiest"

"craftily"

"craftiness"

"crafting"

"crafts"

"craftsman"

"craftsmanly"

"craftsmanship"

"craftsmen"

"crafty"

"crag"

"cragged"

"craggier"

"craggiest"

"craggily"

"cragginess"

"craggy"

"crags"

"cragsman"

"cragsmen"

"cram"

"crambos"

"crammed"

"crammer"

"crammers"

"cramming"

"cramp"

"cramped"

"cramping"

"crampon"

"crampons"

"cramps"

"crams"

"cranberries"

"cranberry"

"cranched"

"cranches"

"cranching"

"crane"

"craned"

"cranes"

"crania"

"cranial"

"cranially"

"craniate"

"craning"

"craniofacial"

"craniology"

"craniometer"

"cranium"

"craniums"

"crank"

"crankcase"

"crankcases"

"cranked"

"cranker"

"crankest"

"crankier"

"crankiest"

"crankily"

"crankiness"

"cranking"

"crankpin"

"crankpins"

"cranks"

"crankshaft"

"crankshafts"

"cranky"

"crannied"

"crannies"

"cranny"

"crap"

"crape"

"craped"

"crapes"

"craping"

"crapped"

"crapper"

"crappers"

"crappie"

"crappier"

"crappies"

"crappiest"

"crappiness"

"crapping"

"crappy"

"craps"

"crapshooter"

"crapshooters"

"crapulence"

"crapulent"

"crapulous"

"crash"

"crashed"

"crasher"

"crashers"

"crashes"

"crashing"

"crass"

"crasser"

"crassest"

"crassly"

"crassness"

"crate"

"crated"

"crater"

"cratered"

"cratering"

"craters"

"crates"

"crating"

"cratometer"

"craton"

"cratons"

"cravat"

"cravats"

"crave"

"craved"

"craven"

"cravened"

"cravenly"

"cravenness"

"cravens"

"craver"

"cravers"

"craves"

"craving"

"cravingly"

"cravings"

"craw"

"crawdad"

"crawdads"

"crawfish"

"crawfished"

"crawfishes"

"crawl"

"crawled"

"crawler"

"crawlers"

"crawlier"

"crawliest"

"crawling"

"crawls"

"crawlspace"

"crawlway"

"crawlways"

"crawly"

"craws"

"crayfish"

"crayfishes"

"crayon"

"crayoned"

"crayoning"

"crayonist"

"crayonists"

"crayons"

"craze"

"crazed"

"crazes"

"crazier"

"crazies"

"craziest"

"crazily"

"craziness"

"crazing"

"crazy"

"crc"

"creak"

"creaked"

"creakier"

"creakiest"

"creakily"

"creakiness"

"creaking"

"creaks"

"creaky"

"cream"

"creamed"

"creamer"

"creameries"

"creamers"

"creamery"

"creamier"

"creamiest"

"creamily"

"creaminess"

"creaming"

"creams"

"creamy"

"crease"

"creased"

"creaser"

"creasers"

"creases"

"creasier"

"creasiest"

"creasing"

"creasy"

"create"

"created"

"creates"

"creating"

"creation"

"creations"

"creative"

"creative cells"

"creatively"

"creativeness"

"creativity"

"Creativity enhancing"

"creator"

"creators"

"creature"

"creatures"

"creche"

"creches"

"credence"

"credences"

"credential"

"credentialed"

"credentials"

"credenza"

"credenzas"

"credibilities"

"credibility"

"credible"

"credibleness"

"credibly"

"credit"

"creditabilities"

"creditability"

"creditable"

"creditableness"

"creditably"

"credited"

"crediting"

"creditor"

"creditors"

"credits"

"credo"

"credos"

"credulity"

"credulous"

"credulously"

"cree"

"creed"

"creedal"

"creeds"

"creek"

"creeks"

"creel"

"creels"

"creep"

"creepage"

"creepages"

"creeper"

"creepers"

"creepie"

"creepier"

"creepies"

"creepiest"

"creepily"

"creepiness"

"creeping"

"creeps"

"creepy"

"crees"

"cremate"

"cremated"

"cremates"

"cremating"

"cremation"

"cremations"

"cremator"

"crematoria"

"crematories"

"crematorium"

"crematoriums"

"cremators"

"crematory"

"creme"

"cremes"

"cremnophobia"

"crenate"

"crenated"

"crenation"

"crenel"

"crenelate"

"crenelated"

"crenelates"

"crenelating"

"crenelation"

"crenelations"

"creneled"

"crenels"

"creole"

"creoles"

"creosote"

"creosoted"

"creosotes"

"creosoting"

"crepe"

"creped"

"crepes"

"crepey"

"crepier"

"creping"

"crepitant"

"crepitation"

"crepitus"

"crept"

"crepuscular"

"crepy"

"crescendo"

"crescendos"

"crescent"

"crescentic"

"crescents"

"crescograph"

"cress"

"cresses"

"cresset"

"cressets"

"crest"

"crestal"

"crested"

"crestfallen"

"crestfallenly"

"cresting"

"crestings"

"crestless"

"crests"

"cretaceous"

"crete"

"cretic"

"cretin"

"cretinism"

"cretinize"

"cretinized"

"cretinizing"

"cretinous"

"cretins"

"cretonne"

"crevasse"

"crevasses"

"crevassing"

"crevice"

"creviced"

"crevices"

"crew"

"crewcut"

"crewed"

"crewel"

"crewels"

"crewelwork"

"crewing"

"crewless"

"crewman"

"crewmen"

"crews"

"crib"

"cribbage"

"cribbages"

"cribbed"

"cribber"

"cribbers"

"cribbing"

"cribbings"

"cribs"

"cribwork"

"cribworks"

"crick"

"cricked"

"cricket"

"cricketer"

"cricketers"

"cricketing"

"crickets"

"cricking"

"cricks"

"cried"

"crier"

"criers"

"cries"

"crime"

"crimea"

"crimean"

"crimeless"

"crimes"

"criminal"

"criminalities"

"criminality"

"criminally"

"criminalness"

"criminals"

"criminated"

"criminologic"

"criminological"

"criminologically"

"criminologies"

"criminologist"

"criminologists"

"criminology"

"crimp"

"crimped"

"crimper"

"crimpers"

"crimpier"

"crimpiest"

"crimping"

"crimps"

"crimpy"

"crimson"

"crimsoned"

"crimsoning"

"crimsons"

"cringe"

"cringed"

"cringer"

"cringers"

"cringes"

"cringing"

"cringles"

"crinites"

"crinkle"

"crinkled"

"crinkles"

"crinklier"

"crinkliest"

"crinkliness"

"crinkling"

"crinkly"

"crinoids"

"crinoline"

"crinolines"

"cripple"

"crippled"

"crippler"

"cripplers"

"cripples"

"crippling"

"crises"

"crisic"

"crisis"

"crisp"

"crisped"

"crispen"

"crispened"

"crispening"

"crispens"

"crisper"

"crispers"

"crispest"

"crispier"

"crispiest"

"crispily"

"crispiness"

"crisping"

"crisply"

"crispness"

"crisps"

"crispy"

"crisscross"

"crisscrossed"

"crisscrosses"

"crisscrossing"

"criteria"

"Criteria Tool"

"criterion"

"criterions"

"critic"

"critical"

"critical care"

"criticality"

"critically"

"criticalness"

"criticism"

"criticisms"

"criticizable"

"criticize"

"criticized"

"criticizer"

"criticizers"

"criticizes"

"criticizing"

"critics"

"critique"

"critiqued"

"critiques"

"critiquing"

"critter"

"critters"

"crittur"

"critturs"

"croak"

"croaked"

"croaker"

"croakers"

"croakier"

"croakiest"

"croakily"

"croakiness"

"croaking"

"croaks"

"croaky"

"crochet"

"crocheted"

"crocheter"

"crocheters"

"crocheting"

"crochets"

"croci"

"crock"

"crocked"

"crockeries"

"crockery"

"crocket"

"crockets"

"crocking"

"crocks"

"crocodile"

"crocodiles"

"crocus"

"crocuses"

"croft"

"crofter"

"crofters"

"crofts"

"croissant"

"croissants"

"cromwell"

"cromwellian"

"crone"

"crones"

"cronies"

"crony"

"cronyism"

"cronyisms"

"crook"

"crooked"

"crookeder"

"crookedest"

"crookedly"

"crookedness"

"crookeries"

"crookery"

"crooking"

"crookneck"

"crooknecks"

"crooks"

"croon"

"crooned"

"crooner"

"crooners"

"crooning"

"croons"

"crop"

"cropland"

"croplands"

"cropless"

"cropped"

"cropper"

"croppers"

"cropping"

"crops"

"croquet"

"croqueted"

"croqueting"

"croquets"

"croquette"

"croquettes"

"crosby"

"crosier"

"crosiers"

"cross"

"crossability"

"crossarm"

"crossbar"

"crossbars"

"crossbeam"

"crossbeams"

"crossbones"

"crossbow"

"crossbows"

"crossbred"

"crossbreed"

"crossbreeding"

"crossbreeds"

"crosscurrent"

"crosscurrents"

"crosscut"

"crosscuts"

"crosscutting"

"crosse"

"crossed"

"crosser"

"crossers"

"crosses"

"crossest"

"crosshatch"

"crosshatched"

"crosshatches"

"crosshatching"

"crossing"

"crossings"

"crosslet"

"crossly"

"crossness"

"crossover"

"crossovers"

"crosspatch"

"crosspatches"

"crosspiece"

"crosspieces"

"crossroad"

"crossroads"

"crosstalk"

"crosstie"

"crossties"

"crosstown"

"crosswalk"

"crosswalks"

"crossway"

"crossways"

"crosswise"

"crossword"

"crosswords"

"crotch"

"crotched"

"crotches"

"crotchet"

"crotchetiness"

"crotchets"

"crotchety"

"crouch"

"crouched"

"crouches"

"crouching"

"croup"

"croupier"

"croupiers"

"croupiest"

"croupily"

"croups"

"croupy"

"crouton"

"croutons"

"crow"

"crowbar"

"crowbars"

"crowd"

"crowded"

"crowdedness"

"crowder"

"crowders"

"crowdies"

"crowding"

"crowds"

"crowdy"

"crowed"

"crower"

"crowers"

"crowfeet"

"crowfoot"

"crowfoots"

"crowing"

"crown"

"crowned"

"crowner"

"crowners"

"crownets"

"crowning"

"crowns"

"crows"

"crowsteps"

"crozier"

"croziers"

"crucial"

"crucially"

"crucialness"

"cruciate"

"crucible"

"crucibles"

"crucifer"

"crucified"

"crucifies"

"crucifix"

"crucifixes"

"crucifixion"

"crucifixions"

"cruciform"

"crucify"

"crucifying"

"crud"

"crudded"

"crudding"

"cruddy"

"crude"

"crudely"

"crudeness"

"cruder"

"crudes"

"crudest"

"crudities"

"crudity"

"cruds"

"cruel"

"crueler"

"cruelest"

"crueller"

"cruellest"

"cruelly"

"cruelness"

"cruelties"

"cruelty"

"cruet"

"cruets"

"cruise"

"cruised"

"cruiser"

"cruisers"

"cruises"

"cruising"

"cruller"

"crullers"

"crumb"

"crumbed"

"crumber"

"crumbers"

"crumbier"

"crumbiest"

"crumbing"

"crumble"

"crumbled"

"crumbles"

"crumblier"

"crumbliest"

"crumbliness"

"crumbling"

"crumblings"

"crumbly"

"crumbs"

"crumby"

"crummie"

"crummier"

"crummies"

"crummiest"

"crummy"

"crump"

"crumped"

"crumpet"

"crumpets"

"crumping"

"crumple"

"crumpled"

"crumples"

"crumpling"

"crumply"

"crumps"

"crunch"

"crunched"

"cruncher"

"crunchers"

"crunches"

"crunchier"

"crunchiest"

"crunching"

"crunchy"

"crupper"

"cruppers"

"crusade"

"crusaded"

"crusader"

"crusaders"

"crusades"

"crusading"

"crusados"

"cruse"

"crush"

"crushable"

"crushed"

"crusher"

"crushers"

"crushes"

"crushing"

"crushproof"

"crust"

"crustacea"

"crustacean"

"crustaceans"

"crustal"

"crusted"

"crustier"

"crustiest"

"crustily"

"crusting"

"crusts"

"crusty"

"crutch"

"crutched"

"crutches"

"crux"

"cruxes"

"cruzados"

"cruzeiro"

"cruzeiros"

"cry"

"crybabies"

"crybaby"

"crying"

"cryingly"

"cryobiologically"

"cryobiology"

"cryogen"

"cryogenic"

"cryogenically"

"cryogenics"

"cryogenies"

"cryogens"

"cryogeny"

"cryolite"

"cryometer"

"cryonic"

"cryonics"

"cryoscope"

"cryostat"

"cryostats"

"cryosurgeon"

"cryosurgery"

"cryosurgical"

"cryotherapies"

"cryotherapy"

"cryotron"

"cryotrons"

"crypt"

"cryptal"

"cryptic"

"cryptically"

"crypto"

"cryptogam"

"cryptogram"

"cryptograms"

"cryptograph"

"cryptographer"

"cryptographers"

"cryptographic"

"cryptography"

"cryptology"

"cryptos"

"cryptozoology"

"crypts"

"crystal"

"crystalize"

"crystalline"

"crystallization"

"crystallize"

"crystallized"

"crystallizer"

"crystallizes"

"crystallizing"

"crystallogram"

"crystallographer"

"crystallographers"

"crystallographic"

"crystallography"

"crystalloid"

"crystalloidal"

"crystals"

"cs"

"csp"

"cst"

"ct"

"ctetology"

"ctg"

"ctrl"

"cts"

"cub"

"cuba"

"cubage"

"cubages"

"cuban"

"cubans"

"cubature"

"cubbies"

"cubbish"

"cubby"

"cubbyhole"

"cubbyholes"

"cube"

"cubebs"

"cubed"

"cuber"

"cubers"

"cubes"

"cubic"

"cubical"

"cubicity"

"cubicle"

"cubicles"

"cubicly"

"cubics"

"cubiform"

"cubing"

"cubism"

"cubisms"

"cubist"

"cubistic"

"cubists"

"cubit"

"cubital"

"cubits"

"cuboid"

"cuboidal"

"cuboids"

"cubs"

"cuckold"

"cuckolded"

"cuckolding"

"cuckoldry"

"cuckolds"

"cuckoo"

"cuckooed"

"cuckooing"

"cuckoos"

"cucumber"

"cucumbers"

"cucurbit"

"cud"

"cudbears"

"cuddies"

"cuddle"

"cuddled"

"cuddles"

"cuddlesome"

"cuddlier"

"cuddliest"

"cuddling"

"cuddly"

"cuddy"

"cudgel"

"cudgeled"

"cudgeler"

"cudgelers"

"cudgeling"

"cudgelled"

"cudgelling"

"cudgels"

"cuds"

"cudweed"

"cudweeds"

"cue"

"cued"

"cueing"

"cues"

"cuesta"

"cuestas"

"cuff"

"cuffed"

"cuffing"

"cuffless"

"cufflinks"

"cuffs"

"cuing"

"cuirass"

"cuirassed"

"cuirasses"

"cuirassing"

"cuish"

"cuishes"

"cuisine"

"cuisines"

"cuke"

"cukes"

"culinary"

"cull"

"culled"

"cullender"

"culler"

"cullers"

"cullet"

"cullets"

"cullied"

"cullies"

"culling"

"culls"

"cully"

"culminate"

"culminated"

"culminates"

"culminating"

"culmination"

"culminations"

"culms"

"culotte"

"culottes"

"culpa"

"culpability"

"culpable"

"culpableness"

"culpably"

"culpae"

"culpas"

"culprit"

"culprits"

"cult"

"cultic"

"cultigen"

"cultism"

"cultisms"

"cultist"

"cultists"

"cultivable"

"cultivar"

"cultivatable"

"cultivate"

"cultivated"

"cultivates"

"cultivating"

"cultivation"

"cultivations"

"cultivator"

"cultivators"

"cults"

"cultural"

"culturally"

"culture"

"cultured"

"cultures"

"cultures and regions"

"culturing"

"culver"

"culvers"

"culvert"

"culverts"

"cum"

"cumber"

"cumbered"

"cumberer"

"cumberers"

"cumbering"

"cumbers"

"cumbersome"

"cumbersomeness"

"cumbrous"

"cumbrously"

"cumin"

"cumins"

"cummerbund"

"cummerbunds"

"cummers"

"cummin"

"cumquat"

"cumquats"

"cumshaw"

"cumshaws"

"cumulate"

"cumulated"

"cumulates"

"cumulating"

"cumulative"

"cumulatively"

"cumuli"

"cumulonimbus"

"cumulous"

"cumulus"

"cuneate"

"cuneiform"

"cuniform"

"cunner"

"cunners"

"cunni"

"cunnilinctus"

"cunnilinguism"

"cunnilingus"

"cunning"

"cunninger"

"cunningest"

"cunningly"

"cunningness"

"cunnings"

"cunt"

"cunts"

"cup"

"cupbearer"

"cupbearers"

"cupboard"

"cupboards"

"cupcake"

"cupcakes"

"cupful"

"cupfuls"

"cupholder"

"cupid"

"cupidities"

"cupidity"

"cupids"

"cupola"

"cupolaed"

"cupolas"

"cuppa"

"cuppas"

"cupped"

"cupper"

"cuppers"

"cuppier"

"cupping"

"cuppings"

"cuppy"

"cupreous"

"cupric"

"cuprite"

"cuprites"

"cupronickel"

"cuprous"

"cuprums"

"cups"

"cupsful"

"cur"

"curability"

"curable"

"curableness"

"curably"

"curacao"

"curacaos"

"curacies"

"curacy"

"curara"

"curare"

"curares"

"curari"

"curarization"

"curate"

"curates"

"curative"

"curatively"

"curatives"

"curator"

"curatorial"

"curators"

"curatorship"

"curatrices"

"curatrix"

"curb"

"curbable"

"curbed"

"curber"

"curbers"

"curbing"

"curbings"

"curbs"

"curbside"

"curbstone"

"curbstones"

"curd"

"curded"

"curdier"

"curding"

"curdle"

"curdled"

"curdler"

"curdlers"

"curdles"

"curdling"

"curds"

"curdy"

"cure"

"cured"

"cureless"

"curer"

"curers"

"cures"

"curets"

"curettage"

"curette"

"curetted"

"curettes"

"curetting"

"curfew"

"curfewed"

"curfewing"

"curfews"

"curia"

"curiae"

"curial"

"curie"

"curies"

"curing"

"curio"

"curios"

"curiosa"

"curiosities"

"curiosity"

"curious"

"curiouser"

"curiousest"

"curiously"

"curiousness"

"curium"

"curiums"

"curl"

"curled"

"curler"

"curlers"

"curlew"

"curlews"

"curlicue"

"curlicued"

"curlicues"

"curlicuing"

"curlier"

"curliest"

"curlily"

"curliness"

"curling"

"curlings"

"curls"

"curly"

"curlycue"

"curlycues"

"curmudgeon"

"curmudgeons"

"curran"

"currant"

"currants"

"curred"

"currencies"

"currency"

"current"

"currently"

"currentness"

"currents"

"curricula"

"curricular"

"curriculum"

"curriculums"

"currie"

"curried"

"currier"

"curriers"

"curriery"

"curries"

"curring"

"currish"

"curry"

"currycomb"

"currycombed"

"currycombing"

"currycombs"

"currying"

"curs"

"curse"

"cursed"

"curseder"

"cursedest"

"cursedly"

"cursedness"

"curser"

"cursers"

"curses"

"cursing"

"cursive"

"cursively"

"cursiveness"

"cursives"

"cursor"

"cursorily"

"cursoriness"

"cursors"

"cursory"

"curst"

"curt"

"curtail"

"curtailed"

"curtailing"

"curtailment"

"curtailments"

"curtails"

"curtain"

"curtained"

"curtaining"

"curtains"

"curter"

"curtesies"

"curtest"

"curtesy"

"curtly"

"curtness"

"curtsey"

"curtseyed"

"curtseying"

"curtseys"

"curtsied"

"curtsies"

"curtsy"

"curtsying"

"curvaceous"

"curvaceously"

"curvature"

"curvatures"

"curve"

"curved"

"curvedly"

"curves"

"curvet"

"curveted"

"curvets"

"curvetting"

"curvey"

"curvier"

"curviest"

"curviness"

"curving"

"curvy"

"cuscus"

"cusec"

"cushier"

"cushiest"

"cushily"

"cushiness"

"cushing"

"cushion"

"cushioned"

"cushioning"

"cushions"

"cushiony"

"cushy"

"cusp"

"cuspated"

"cusped"

"cuspid"

"cuspidal"

"cuspidated"

"cuspidor"

"cuspidors"

"cuspids"

"cusps"

"cuss"

"cussed"

"cussedly"

"cusser"

"cussers"

"cusses"

"cussing"

"cussword"

"cusswords"

"custard"

"custards"

"custodial"

"custodian"

"custodians"

"custodianship"

"custodies"

"custody"

"custom"

"custom sounds"

"customarily"

"customary"

"customer"

"customer service"

"Customer Support team"

"customers"

"customhouse"

"customhouses"

"Customizable"

"customization"

"customize"

"customized"

"customizes"

"customizing"

"customs"

"customshouse"

"cut"

"cutaneous"

"cutaneously"

"cutaway"

"cutaways"

"cutback"

"cutbacks"

"cutcheries"

"cutdown"

"cutdowns"

"cute"

"cutely"

"cuteness"

"cuter"

"cutes"

"cutesier"

"cutesiest"

"cutest"

"cutesy"

"cutey"

"cuteys"

"cuticle"

"cuticles"

"cuticular"

"cutie"

"cuties"

"cutin"

"cutinizing"

"cutins"

"cutis"

"cutlas"

"cutlases"

"cutlass"

"cutlasses"

"cutler"

"cutleries"

"cutlers"

"cutlery"

"cutlet"

"cutlets"

"cutlines"

"cutoff"

"cutoffs"

"cutout"

"cutouts"

"cutpurse"

"cutpurses"

"cuts"

"cuttable"

"cuttages"

"cutter"

"cutters"

"cutthroat"

"cutthroats"

"cutting"

"cuttings"

"cuttle"

"cuttlebone"

"cuttlebones"

"cuttled"

"cuttlefish"

"cuttlefishes"

"cuttles"

"cuttling"

"cutty"

"cutup"

"cutups"

"cutworm"

"cutworms"

"cwm"

"cwt"

"cyan"

"cyanic"

"cyanide"

"cyanided"

"cyanides"

"cyanin"

"cyanitic"

"cyanoacrylate"

"cyanogen"

"cyanometer"

"cyanosed"

"cyanoses"

"cyanosis"

"cyanotic"

"cyans"

"cybercultural"

"cyberculture"

"cybernated"

"cybernation"

"cybernetic"

"cybernetical"

"cybernetically"

"cybernetician"

"cyberneticist"

"cyberneticists"

"cybernetics"

"cyberphobia"

"cyborg"

"cyborgs"

"cycad"

"cycads"

"cyclamate"

"cyclamates"

"cyclamen"

"cyclamens"

"cyclazocine"

"cycle"

"cyclecar"

"cyclecars"

"cycled"

"cycler"

"cyclers"

"cycles"

"cyclic"

"cyclical"

"cyclically"

"cyclicly"

"cycling"

"cyclings"

"cyclist"

"cyclists"

"cyclized"

"cyclizes"

"cyclizing"

"cyclo"

"cyclograph"

"cycloid"

"cycloidal"

"cycloids"

"cyclometer"

"cyclometers"

"cyclonal"

"cyclone"

"cyclones"

"cyclonic"

"cyclonically"

"cyclopedia"

"cyclopedias"

"cyclopes"

"cyclops"

"cyclos"

"cyclotron"

"cyclotrons"

"cygnet"

"cygnets"

"cylinder"

"cylindered"

"cylinders"

"cylindrical"

"cylindrically"

"cymbal"

"cymbaler"

"cymbalers"

"cymbalist"

"cymbalists"

"cymbals"

"cymbling"

"cyme"

"cymes"

"cymograph"

"cymometer"

"cymose"

"cynic"

"cynical"

"cynically"

"cynicism"

"cynicisms"

"cynics"

"cynology"

"cynophobia"

"cynosure"

"cynosures"

"cypher"

"cyphered"

"cyphering"

"cyphers"

"cypres"

"cypreses"

"cypress"

"cypresses"

"cyprian"

"cyprians"

"cypriot"

"cypriote"

"cypriotes"

"cypriots"

"cyprus"

"cypruses"

"cyst"

"cystectomies"

"cystic"

"cystitis"

"cystoscope"

"cysts"

"cytheromania"

"cytologic"

"cytological"

"cytologically"

"cytologies"

"cytologist"

"cytologists"

"cytology"

"cytometer"

"cytoplasm"

"cytoplasmic"

"cytosine"

"czar"

"czardas"

"czardases"

"czardom"

"czardoms"

"czarevna"

"czarevnas"

"czarina"

"czarinas"

"czarism"

"czarisms"

"czarist"

"czarists"

"czaritza"

"czaritzas"

"czars"

"czech"

"czechoslovak"

"czechoslovakia"

"czechoslovakian"

"czechoslovakians"

"czechoslovaks"

"czechs"

"dab"

"dabbed"

"dabbing"

"dabble"

"dabbled"

"dabbler"

"dabblers"

"dabbles"

"dabbling"

"dabblings"

"dabs"

"dace"

"daces"

"dacha"

"dachas"

"dachshund"

"dachshunds"

"dacnomania"

"dacoit"

"dacoits"

"dacron"

"dactyl"

"dactylic"

"dactyliology"

"dactylography"

"dactylology"

"dactyls"

"dactylus"

"dad"

"dada"

"dadaism"

"dadaisms"

"dadaist"

"dadaists"

"dadas"

"daddies"

"daddling"

"daddy"

"dado"

"dadoed"

"dadoes"

"dadoing"

"dados"

"dads"

"daemon"

"daemonic"

"daemons"

"daffier"

"daffiest"

"daffiness"

"daffodil"

"daffodils"

"daffy"

"daft"

"dafter"

"daftest"

"daftly"

"daftness"

"dag"

"dagger"

"daggered"

"daggers"

"dago"

"dagoba"

"dagobas"

"dagoes"

"dagos"

"daguerreotype"

"daguerreotypes"

"dah"

"dahlia"

"dahlias"

"dahomey"

"dailies"

"daily"

"daimon"

"daimonic"

"daimons"

"daimyo"

"daimyos"

"daintier"

"dainties"

"daintiest"

"daintily"

"daintiness"

"dainty"

"daiquiri"

"daiquiris"

"dairies"

"dairy"

"dairying"

"dairymaid"

"dairymaids"

"dairyman"

"dairymen"

"dais"

"daises"

"daisied"

"daisies"

"daisy"

"dakoit"

"dakoits"

"dakota"

"dakotan"

"dakotans"

"dakotas"

"dal"

"dale"

"dales"

"dalesman"

"dalesmen"

"daleth"

"daleths"

"dallas"

"dalles"

"dalliance"

"dalliances"

"dallied"

"dallier"

"dalliers"

"dallies"

"dally"

"dallying"

"dalmatian"

"dalmatians"

"dalton"

"dam"

"damage"

"damageable"

"damaged"

"damager"

"damagers"

"damages"

"damaging"

"damagingly"

"damascene"

"damascened"

"damascenes"

"damascus"

"damask"

"damasked"

"damasks"

"dame"

"dames"

"dammed"

"dammer"

"dammers"

"damming"

"damn"

"damnabilities"

"damnability"

"damnable"

"damnableness"

"damnably"

"damnation"

"damndest"

"damned"

"damneder"

"damnedest"

"damner"

"damners"

"damnification"

"damnify"

"damnifying"

"damning"

"damnit"

"damns"

"damocles"

"damosel"

"damosels"

"damozels"

"damp"

"damped"

"dampen"

"dampened"

"dampener"

"dampeners"

"dampening"

"dampens"

"damper"

"dampers"

"dampest"

"damping"

"dampish"

"damply"

"dampness"

"damps"

"dams"

"damsel"

"damselflies"

"damselfly"

"damsels"

"damson"

"damsons"

"dan"

"dana"

"dance"

"danced"

"dancer"

"dancers"

"dances"

"dancing"

"dancingly"

"dandelion"

"dandelions"

"dander"

"dandered"

"danders"

"dandier"

"dandies"

"dandiest"

"dandification"

"dandified"

"dandifies"

"dandify"

"dandifying"

"dandily"

"dandle"

"dandled"

"dandler"

"dandlers"

"dandles"

"dandling"

"dandruff"

"dandy"

"dandyish"

"dandyism"

"dandyisms"

"dane"

"danegeld"

"danegelds"

"danes"

"daneweed"

"danewort"

"dang"

"danged"

"danger"

"dangered"

"dangerous"

"dangerously"

"dangerousness"

"dangers"

"danging"

"dangle"

"dangled"

"dangler"

"danglers"

"dangles"

"dangling"

"dangs"

"daniel"

"danish"

"dank"

"danker"

"dankest"

"dankly"

"dankness"

"danseur"

"danseurs"

"danseuse"

"danseuses"

"dante"

"danube"

"dap"

"daphnia"

"daphnias"

"dapper"

"dapperer"

"dapperest"

"dapperly"

"dapperness"

"dapping"

"dapple"

"dappled"

"dapples"

"dappling"

"darcy"

"dare"

"dared"

"daredevil"

"daredevils"

"dareful"

"darer"

"darers"

"dares"

"daresay"

"daring"

"daringly"

"daringness"

"darings"

"dark"

"darked"

"darken"

"darkened"

"darkener"

"darkeners"

"darkening"

"darkens"

"darker"

"darkest"

"darkey"

"darkeys"

"darkhaired"

"darkie"

"darkies"

"darking"

"darkish"

"darkle"

"darkled"

"darkles"

"darklier"

"darkliest"

"darkling"

"darkly"

"darkness"

"darkroom"

"darkrooms"

"darks"

"darksome"

"darky"

"darling"

"darlings"

"darn"

"darndest"

"darndests"

"darned"

"darneder"

"darnedest"

"darnel"

"darnels"

"darner"

"darners"

"darning"

"darnings"

"darns"

"dart"

"darted"

"darter"

"darters"

"darting"

"darts"

"darvon"

"darwin"

"darwinian"

"darwinians"

"darwinism"

"darwinist"

"darwinists"

"darwinite"

"dash"

"dashboard"

"dashboards"

"dashed"

"dasher"

"dashers"

"dashes"

"dashier"

"dashiki"

"dashikis"

"dashing"

"dashingly"

"dashpot"

"dashpots"

"dashy"

"dastard"

"dastardliness"

"dastardly"

"dastards"

"data"

"data set"

"data-encrypted"

"database"

"databases"

"datable"

"dataflow"

"datamation"

"datary"

"datcha"

"datchas"

"date"

"dateable"

"dated"

"datedly"

"datedness"

"dateless"

"dateline"

"datelined"

"datelines"

"datelining"

"dater"

"daters"

"dates"

"dating"

"dative"

"datively"

"datives"

"datsun"

"datsuns"

"datum"

"datums"

"datura"

"daturas"

"daub"

"daubed"

"dauber"

"dauberies"

"daubers"

"daubery"

"daubes"

"daubier"

"daubing"

"daubs"

"dauby"

"daughter"

"daughterly"

"daughters"

"daunt"

"daunted"

"daunter"

"daunters"

"daunting"

"dauntless"

"dauntlessly"

"dauntlessness"

"daunts"

"dauphin"

"dauphine"

"dauphins"

"dave"

"davenport"

"davenports"

"david"

"davies"

"davis"

"davit"

"davits"

"daw"

"dawdle"

"dawdled"

"dawdler"

"dawdlers"

"dawdles"

"dawdling"

"dawn"

"dawned"

"dawning"

"dawns"

"day"

"daybed"

"daybeds"

"daybook"

"daybooks"

"daybreak"

"daybreaks"

"daydream"

"daydreamed"

"daydreamer"

"daydreamers"

"daydreaming"

"daydreams"

"daydreamt"

"dayflies"

"dayflower"

"dayflowers"

"dayfly"

"dayglow"

"dayglows"

"daylight"

"daylighted"

"daylights"

"daylilies"

"daylily"

"daylit"

"daylong"

"daymare"

"dayroom"

"dayrooms"

"days"

"dayside"

"daysides"

"daystar"

"daystars"

"daytime"

"daytimes"

"dayton"

"daze"

"dazed"

"dazedly"

"dazedness"

"dazes"

"dazing"

"dazzle"

"dazzled"

"dazzler"

"dazzlers"

"dazzles"

"dazzling"

"dazzlingly"

"db"

"dbl"

"dbms"

"dc"

"de"

"deaccession"

"deaccessioned"

"deaccessioning"

"deaccessions"

"deacidification"

"deacidified"

"deacidifying"

"deacon"

"deaconed"

"deaconess"

"deaconesses"

"deaconing"

"deaconries"

"deaconry"

"deacons"

"deactivate"

"deactivated"

"deactivates"

"deactivating"

"deactivation"

"deactivations"

"deactivator"

"deactivators"

"dead"

"deadbeat"

"deadbeats"

"deaden"

"deadened"

"deadener"

"deadeners"

"deadening"

"deadens"

"deader"

"deadest"

"deadeye"

"deadeyes"

"deadfall"

"deadfalls"

"deadhead"

"deadheaded"

"deadheads"

"deadlier"

"deadliest"

"deadline"

"deadlines"

"deadliness"

"deadlock"

"deadlocked"

"deadlocking"

"deadlocks"

"deadly"

"deadman"

"deadness"

"deadpan"

"deadpanned"

"deadpans"

"deads"

"deadweight"

"deadwood"

"deadwoods"

"deaf"

"deafen"

"deafened"

"deafening"

"deafens"

"deafer"

"deafest"

"deafish"

"deafly"

"deafness"

"deair"

"deairs"

"deal"

"dealcoholization"

"dealer"

"dealers"

"dealership"

"dealerships"

"dealing"

"dealings"

"deals"

"dealt"

"dean"

"deaneries"

"deanery"

"deaning"

"deans"

"deanship"

"deanships"

"dear"

"dearer"

"dearest"

"dearie"

"dearies"

"dearly"

"dearness"

"dears"

"dearth"

"dearths"

"deary"

"deash"

"death"

"deathbed"

"deathbeds"

"deathblow"

"deathblows"

"deathcup"

"deathcups"

"deathful"

"deathless"

"deathlessly"

"deathlessness"

"deathlike"

"deathly"

"deathrate"

"deaths"

"deathtrap"

"deathtraps"

"deathwatch"

"deathwatches"

"deathy"

"deb"

"debacle"

"debacles"

"debar"

"debark"

"debarkation"

"debarkations"

"debarked"

"debarking"

"debarks"

"debarment"

"debarred"

"debarring"

"debars"

"debase"

"debased"

"debasedness"

"debasement"

"debaser"

"debasers"

"debases"

"debasing"

"debatable"

"debatably"

"debate"

"debateable"

"debated"

"debater"

"debaters"

"debates"

"debating"

"debauch"

"debauched"

"debauchedly"

"debauchedness"

"debauchee"

"debauchees"

"debaucher"

"debaucheries"

"debauchery"

"debauches"

"debauching"

"debbie"

"debenture"

"debentures"

"debilitant"

"debilitate"

"debilitated"

"debilitates"

"debilitating"

"debilitation"

"debilitations"

"debilitative"

"debilities"

"debility"

"debit"

"debitable"

"debited"

"debiting"

"debits"

"debonair"

"debonairly"

"debonairness"

"debone"

"debouch"

"debouche"

"debouched"

"debouches"

"debouching"

"debrided"

"debrief"

"debriefed"

"debriefing"

"debriefings"

"debriefs"

"debris"

"debruising"

"debs"

"debt"

"debtee"

"debtless"

"debtor"

"debtors"

"debts"

"debug"

"debugged"

"debugger"

"debuggers"

"debugging"

"debugs"

"debunk"

"debunked"

"debunker"

"debunkers"

"debunking"

"debunks"

"debussy"

"debut"

"debutant"

"debutante"

"debutantes"

"debutants"

"debuted"

"debuting"

"debuts"

"dec"

"decade"

"decadence"

"decadent"

"decadently"

"decadents"

"decades"

"decaffeinate"

"decaffeinated"

"decaffeinates"

"decaffeinating"

"decagon"

"decagons"

"decagram"

"decahedra"

"decahedron"

"decahedrons"

"decal"

"decalcification"

"decalcified"

"decalcifies"

"decalcify"

"decalcifying"

"decalcomania"

"decalcomanias"

"decaliters"

"decals"

"decameter"

"decameters"

"decamp"

"decamped"

"decamping"

"decampment"

"decamps"

"decant"

"decanted"

"decanter"

"decanters"

"decanting"

"decants"

"decapitate"

"decapitated"

"decapitates"

"decapitating"

"decapitation"

"decapitations"

"decapitator"

"decapod"

"decapods"

"decapsulate"

"decares"

"decasyllabic"

"decasyllable"

"decasyllables"

"decathlon"

"decathlons"

"decay"

"decayable"

"decayed"

"decayedness"

"decayer"

"decayers"

"decaying"

"decays"

"decease"

"deceased"

"deceases"

"deceasing"

"decedent"

"decedents"

"deceit"

"deceitful"

"deceitfully"

"deceitfulness"

"deceits"

"deceivable"

"deceive"

"deceived"

"deceiver"

"deceivers"

"deceives"

"deceiving"

"deceivingly"

"decelerate"

"decelerated"

"decelerates"

"decelerating"

"deceleration"

"decelerations"

"decelerator"

"decelerators"

"decelerometer"

"december"

"decemvir"

"decenaries"

"decenary"

"decencies"

"decency"

"decennia"

"decennial"

"decennially"

"decennials"

"decenniums"

"decent"

"decenter"

"decentered"

"decentest"

"decently"

"decentralism"

"decentralist"

"decentralization"

"decentralizations"

"decentralize"

"decentralized"

"decentralizes"

"decentralizing"

"decentring"

"deception"

"deceptions"

"deceptive"

"deceptively"

"deceptiveness"

"decertification"

"decertified"

"decertifying"

"dechlorinate"

"dechlorinated"

"dechlorinating"

"dechlorination"

"deciare"

"deciares"

"decibel"

"decibels"

"decidable"

"decide"

"decided"

"decidedly"

"decider"

"deciders"

"decides"

"deciding"

"decidua"

"decidual"

"deciduous"

"deciduously"

"deciduousness"

"decigram"

"decigrams"

"decile"

"deciliter"

"deciliters"

"decimal"

"decimalization"

"decimalize"

"decimalized"

"decimalizes"

"decimalizing"

"decimally"

"decimals"

"decimate"

"decimated"

"decimates"

"decimating"

"decimation"

"decimeter"

"decimeters"

"decipher"

"decipherable"

"deciphered"

"deciphering"

"deciphers"

"decision"

"decision making"

"decision support"

"decisional"

"decisions"

"decisive"

"decisively"

"decisiveness"

"decistere"

"decisteres"

"deck"

"decked"

"decker"

"deckers"

"deckhand"

"deckhands"

"decking"

"deckings"

"deckle"

"deckles"

"decks"

"declaim"

"declaimed"

"declaimer"

"declaimers"

"declaiming"

"declaims"

"declamation"

"declamations"

"declamatory"

"declarable"

"declarant"

"declaration"

"declarations"

"declarative"

"declaratively"

"declarator"

"declaratory"

"declare"

"declared"

"declarer"

"declarers"

"declares"

"declaring"

"declasse"

"declassification"

"declassifications"

"declassified"

"declassifies"

"declassify"

"declassifying"

"declassing"

"declension"

"declensions"

"declinable"

"declination"

"declinational"

"declinations"

"declinatory"

"declinature"

"decline"

"declined"

"decliner"

"decliners"

"declines"

"declining"

"declinometer"

"declivities"

"declivity"

"deco"

"decoct"

"decocted"

"decocting"

"decoction"

"decocts"

"decode"

"decoded"

"decoder"

"decoders"

"decodes"

"decoding"

"decodings"

"decollated"

"decollete"

"decolonization"

"decolonize"

"decolonized"

"decolonizes"

"decolonizing"

"decommission"

"decommissioned"

"decommissioning"

"decommissions"

"decompensate"

"decompensated"

"decompensates"

"decompensating"

"decompensation"

"decompensations"

"decomposability"

"decomposable"

"decompose"

"decomposed"

"decomposer"

"decomposers"

"decomposes"

"decomposing"

"decomposition"

"decompositions"

"decompress"

"decompressed"

"decompresses"

"decompressing"

"decompression"

"decompressions"

"decompressive"

"decongest"

"decongestant"

"decongestants"

"decongested"

"decongesting"

"decongestion"

"decongestive"

"decongests"

"decontaminate"

"decontaminated"

"decontaminates"

"decontaminating"

"decontamination"

"decontaminations"

"decontaminator"

"decontaminators"

"decontrol"

"decontrolled"

"decontrolling"

"decontrols"

"decor"

"decorate"

"decorated"

"decorates"

"decorating"

"decoration"

"decorations"

"decorative"

"decoratively"

"decorativeness"

"decorator"

"decorators"

"decorous"

"decorously"

"decorousness"

"decors"

"decorticate"

"decorum"

"decorums"

"decoupage"

"decouple"

"decoy"

"decoyed"

"decoyer"

"decoyers"

"decoying"

"decoys"

"decrease"

"decreased"

"decreases"

"decreasing"

"decreasingly"

"decree"

"decreed"

"decreeing"

"decreer"

"decreers"

"decrees"

"decrement"

"decrements"

"decrepit"

"decrepitly"

"decrepitude"

"decrescendo"

"decrescendos"

"decrial"

"decrials"

"decried"

"decrier"

"decriers"

"decries"

"decriminalization"

"decriminalize"

"decriminalized"

"decriminalizes"

"decriminalizing"

"decrowns"

"decry"

"decrying"

"decrypt"

"decrypted"

"decrypting"

"decryption"

"decryptions"

"decrypts"

"dedicate"

"dedicated"

"dedicatee"

"dedicates"

"dedicating"

"dedication"

"dedicational"

"dedications"

"dedicator"

"dedicators"

"dedicatory"

"deduce"

"deduced"

"deduces"

"deducible"

"deducing"

"deduct"

"deducted"

"deductibility"

"deductible"

"deductibles"

"deducting"

"deduction"

"deductions"

"deductive"

"deductively"

"deducts"

"deed"

"deedbox"

"deeded"

"deedier"

"deeding"

"deedless"

"deeds"

"deedy"

"deejay"

"deejays"

"deem"

"deemed"

"deeming"

"deemphasis"

"deemphasize"

"deemphasized"

"deemphasizes"

"deemphasizing"

"deems"

"deep"

"deepen"

"deepened"

"deepener"

"deepeners"

"deepening"

"deepens"

"deeper"

"deepest"

"deeply"

"deepness"

"deeps"

"deer"

"deerfly"

"deerflys"

"deers"

"deerskin"

"deerskins"

"deerstalker"

"deerstalkers"

"deerweed"

"deerweeds"

"deeryard"

"dees"

"deescalate"

"deescalated"

"deescalates"

"deescalating"

"deescalation"

"deescalations"

"deface"

"defaced"

"defacement"

"defacements"

"defacer"

"defacers"

"defaces"

"defacing"

"defacto"

"defalcate"

"defalcated"

"defalcates"

"defalcating"

"defalcation"

"defalcations"

"defamation"

"defamations"

"defamatory"

"defame"

"defamed"

"defamer"

"defamers"

"defames"

"defaming"

"defamingly"

"defat"

"defats"

"defatted"

"default"

"defaulted"

"defaulter"

"defaulters"

"defaulting"

"defaults"

"defeat"

"defeated"

"defeater"

"defeaters"

"defeating"

"defeatism"

"defeatist"

"defeatists"

"defeats"

"defecate"

"defecated"

"defecates"

"defecating"

"defecation"

"defect"

"defected"

"defecter"

"defecters"

"defecting"

"defection"

"defections"

"defective"

"defectively"

"defectiveness"

"defector"

"defectors"

"defects"

"defeminize"

"defeminized"

"defeminizing"

"defence"

"defences"

"defend"

"defendable"

"defendant"

"defendants"

"defended"

"defender"

"defenders"

"defending"

"defends"

"defense"

"defensed"

"defenseless"

"defenselessly"

"defenselessness"

"defenses"

"defensibility"

"defensible"

"defensibly"

"defensing"

"defensive"

"defensively"

"defensiveness"

"defer"

"deference"

"deferent"

"deferential"

"deferentially"

"deferment"

"deferments"

"deferrable"

"deferral"

"deferrals"

"deferred"

"deferrer"

"deferrers"

"deferring"

"defers"

"defiance"

"defiances"

"defiant"

"defiantly"

"defibrillate"

"deficiencies"

"deficiency"

"deficient"

"deficiently"

"deficit"

"deficits"

"defied"

"defier"

"defiers"

"defies"

"defile"

"defiled"

"defilement"

"defilements"

"defiler"

"defilers"

"defiles"

"defiling"

"defilingly"

"definable"

"definably"

"define"

"defined"

"definement"

"definer"

"definers"

"defines"

"defining"

"definite"

"definitely"

"definiteness"

"definition"

"definitions"

"definitive"

"definitively"

"definitiveness"

"defintion"

"deflagrate"

"deflagrated"

"deflagrates"

"deflagrating"

"deflagration"

"deflagrations"

"deflate"

"deflated"

"deflates"

"deflating"

"deflation"

"deflationary"

"deflations"

"deflator"

"deflators"

"deflea"

"deflect"

"deflectable"

"deflected"

"deflecting"

"deflection"

"deflections"

"deflective"

"deflector"

"deflectors"

"deflects"

"defloration"

"deflorations"

"deflorescence"

"deflower"

"deflowered"

"deflowering"

"deflowers"

"defoam"

"defoamed"

"defoamer"

"defog"

"defogged"

"defogger"

"defoggers"

"defogging"

"defogs"

"defoliant"

"defoliants"

"defoliate"

"defoliated"

"defoliates"

"defoliating"

"defoliation"

"defoliations"

"defoliator"

"defoliators"

"deforest"

"deforestation"

"deforested"

"deforesting"

"deforests"

"deform"

"deformable"

"deformation"

"deformations"

"deformative"

"deformed"

"deformer"

"deformers"

"deforming"

"deformities"

"deformity"

"deforms"

"defraud"

"defraudation"

"defrauded"

"defrauder"

"defrauders"

"defrauding"

"defrauds"

"defray"

"defrayable"

"defrayal"

"defrayals"

"defrayed"

"defrayer"

"defrayers"

"defraying"

"defrayment"

"defrays"

"defrock"

"defrocked"

"defrocking"

"defrocks"

"defrost"

"defrosted"

"defroster"

"defrosters"

"defrosting"

"defrosts"

"deft"

"defter"

"deftest"

"deftly"

"deftness"

"defunct"

"defunctive"

"defunctness"

"defuse"

"defused"

"defuses"

"defusing"

"defuze"

"defuzed"

"defuzes"

"defuzing"

"defy"

"defying"

"degas"

"degass"

"degassed"

"degasses"

"degassing"

"degauss"

"degaussed"

"degausses"

"degaussing"

"degeneracies"

"degeneracy"

"degenerate"

"degenerated"

"degenerately"

"degenerateness"

"degenerates"

"degenerating"

"degeneration"

"degenerations"

"degenerative"

"degerm"

"degermed"

"degradable"

"degradation"

"degradations"

"degrade"

"degraded"

"degradedly"

"degradedness"

"degrader"

"degraders"

"degrades"

"degrading"

"degrease"

"degreased"

"degreases"

"degreasing"

"degree"

"degreed"

"degrees"

"degum"

"degummed"

"degumming"

"degums"

"dehorn"

"dehorned"

"dehorner"

"dehorning"

"dehorns"

"dehumanization"

"dehumanize"

"dehumanized"

"dehumanizes"

"dehumanizing"

"dehumidification"

"dehumidified"

"dehumidifier"

"dehumidifiers"

"dehumidifies"

"dehumidify"

"dehumidifying"

"dehydrate"

"dehydrated"

"dehydrates"

"dehydrating"

"dehydration"

"dehydrator"

"dehydrators"

"dehydrogenate"

"dehydrogenated"

"dehydrogenates"

"dehydrogenating"

"dehydrogenation"

"dehypnotize"

"dehypnotized"

"dehypnotizing"

"dei"

"deice"

"deiced"

"deicer"

"deicers"

"deices"

"deicidal"

"deicide"

"deicides"

"deicing"

"deific"

"deifical"

"deification"

"deifications"

"deified"

"deifier"

"deifiers"

"deifies"

"deiform"

"deify"

"deifying"

"deign"

"deigned"

"deigning"

"deigns"

"deionization"

"deionizations"

"deionize"

"deionized"

"deionizes"

"deionizing"

"deism"

"deisms"

"deist"

"deistic"

"deists"

"deities"

"deity"

"deja"

"deject"

"dejected"

"dejectedly"

"dejectedness"

"dejecting"

"dejection"

"dejections"

"dejects"

"dekagram"

"dekagrams"

"dekaliter"

"dekaliters"

"dekameter"

"dekameters"

"dekares"

"del"

"delaware"

"delawarean"

"delay"

"delayed"

"delayer"

"delayers"

"delaying"

"delays"

"dele"

"delead"

"delectable"

"delectably"

"delectation"

"delectations"

"deled"

"delegacies"

"delegacy"

"delegalizing"

"delegant"

"delegate"

"delegated"

"delegatee"

"delegates"

"delegati"

"delegating"

"delegation"

"delegations"

"delegatory"

"deleing"

"deles"

"delete"

"deleted"

"deleterious"

"deleteriously"

"deleteriousness"

"deletes"

"deleting"

"deletion"

"deletions"

"delft"

"delfts"

"delhi"

"deli"

"deliberate"

"deliberated"

"deliberately"

"deliberateness"

"deliberates"

"deliberating"

"deliberation"

"deliberations"

"deliberative"

"deliberatively"

"deliberator"

"delicacies"

"delicacy"

"delicate"

"delicately"

"delicateness"

"delicates"

"delicatessen"

"delicatessens"

"delicious"

"deliciously"

"deliciousness"

"delict"

"delicti"

"delicto"

"delight"

"delighted"

"delightedly"

"delightful"

"delightfully"

"delightfulness"

"delighting"

"delights"

"delime"

"deliming"

"delimit"

"delimitating"

"delimitation"

"delimitations"

"delimitative"

"delimited"

"delimiter"

"delimiters"

"delimiting"

"delimits"

"delineate"

"delineated"

"delineates"

"delineating"

"delineation"

"delineations"

"delineative"

"delinquencies"

"delinquency"

"delinquent"

"delinquently"

"delinquents"

"deliquesce"

"deliquesced"

"deliquescence"

"deliquescent"

"deliquesces"

"deliquescing"

"deliria"

"deliriant"

"delirifacient"

"delirious"

"deliriously"

"deliriousness"

"delirium"

"deliriums"

"delis"

"delist"

"deliver"

"deliverable"

"deliverables"

"deliverance"

"delivered"

"deliverer"

"deliverers"

"deliveries"

"delivering"

"delivers"

"delivery"

"delivery date"

"dell"

"dells"

"delly"

"delouse"

"deloused"

"delouses"

"delousing"

"delphinia"

"delphinium"

"delphiniums"

"delta"

"deltaic"

"deltas"

"deltic"

"deltiology"

"deltoid"

"deltoids"

"delude"

"deluded"

"deluder"

"deluders"

"deludes"

"deluding"

"deludingly"

"deluge"

"deluged"

"deluges"

"deluging"

"delusion"

"delusional"

"delusionary"

"delusionist"

"delusions"

"delusive"

"delusively"

"delusiveness"

"delusory"

"deluxe"

"delve"

"delved"

"delver"

"delvers"

"delves"

"delving"

"demagnetization"

"demagnetize"

"demagnetized"

"demagnetizes"

"demagnetizing"

"demagnification"

"demagog"

"demagogic"

"demagogies"

"demagogs"

"demagogue"

"demagoguery"

"demagogues"

"demagogy"

"demand"

"demandable"

"demanded"

"demander"

"demanders"

"demanding"

"demandingly"

"demands"

"demarcate"

"demarcated"

"demarcates"

"demarcating"

"demarcation"

"demarcations"

"demarcator"

"demarcators"

"demarche"

"demarches"

"demarking"

"demasculinize"

"demasculinized"

"demasculinizing"

"demean"

"demeaned"

"demeaning"

"demeanor"

"demeanors"

"demeans"

"dement"

"demented"

"dementedly"

"dementia"

"dementias"

"dementing"

"dements"

"demerit"

"demerited"

"demeriting"

"demerits"

"demesne"

"demesnes"

"demeter"

"demigod"

"demigods"

"demijohn"

"demijohns"

"demilitarization"

"demilitarize"

"demilitarized"

"demilitarizes"

"demilitarizing"

"demimondain"

"demimondaine"

"demimondaines"

"demimonde"

"demineralization"

"demineralize"

"demineralized"

"demineralizes"

"demineralizing"

"demise"

"demised"

"demises"

"demising"

"demit"

"demitasse"

"demitasses"

"demits"

"demitted"

"demiurge"

"demiurges"

"demo"

"demob"

"demobbed"

"demobbing"

"demobilization"

"demobilizations"

"demobilize"

"demobilized"

"demobilizes"

"demobilizing"

"demobs"

"democracies"

"democracy"

"democrat"

"democratic"

"democratical"

"democratically"

"democratism"

"democratization"

"democratize"

"democratized"

"democratizes"

"democratizing"

"democrats"

"demode"

"demodulate"

"demodulated"

"demodulates"

"demodulating"

"demodulation"

"demodulations"

"demographer"

"demographers"

"demographic"

"demographically"

"demographics"

"demographies"

"demography"

"demoiselle"

"demoiselles"

"demolish"

"demolished"

"demolisher"

"demolishes"

"demolishing"

"demolition"

"demolitionist"

"demolitions"

"demology"

"demon"

"demoness"

"demonetization"

"demonetize"

"demonetized"

"demonetizes"

"demonetizing"

"demoniac"

"demoniacal"

"demoniacs"

"demonian"

"demonic"

"demonical"

"demonise"

"demonism"

"demonisms"

"demonist"

"demonists"

"demonize"

"demonized"

"demonizes"

"demonizing"

"demonologies"

"demonology"

"demonomania"

"demons"

"demonstrable"

"demonstrably"

"demonstrandum"

"demonstrate"

"demonstrated"

"demonstrates"

"demonstrating"

"demonstration"

"demonstrational"

"demonstrationist"

"demonstrationists"

"demonstrations"

"demonstrative"

"demonstratively"

"demonstrativeness"

"demonstrator"

"demonstrators"

"demoralization"

"demoralize"

"demoralized"

"demoralizer"

"demoralizers"

"demoralizes"

"demoralizing"

"demos"

"demote"

"demoted"

"demotes"

"demotic"

"demotics"

"demoting"

"demotion"

"demotions"

"demotist"

"demount"

"demountable"

"demounted"

"demounting"

"demounts"

"dempster"

"demulcent"

"demulcents"

"demultiplexes"

"demur"

"demure"

"demurely"

"demureness"

"demurer"

"demurest"

"demurrable"

"demurrage"

"demurrages"

"demurral"

"demurrals"

"demurred"

"demurrer"

"demurrers"

"demurring"

"demurs"

"demythologization"

"demythologizations"

"demythologize"

"demythologized"

"demythologizes"

"demythologizing"

"den"

"denarii"

"denarius"

"denationalizing"

"denaturant"

"denaturants"

"denaturation"

"denature"

"denatured"

"denatures"

"denaturing"

"denazified"

"denazifies"

"denazify"

"dendrite"

"dendrites"

"dendritic"

"dendrochronology"

"dendroid"

"dendrologic"

"dendrological"

"dendrologist"

"dendrologists"

"dendrology"

"dendrometer"

"dendrons"

"dengue"

"dengues"

"deniable"

"deniably"

"denial"

"denials"

"denicotinize"

"denicotinized"

"denicotinizes"

"denicotinizing"

"denied"

"denier"

"deniers"

"denies"

"denigrate"

"denigrated"

"denigrates"

"denigrating"

"denigration"

"denigrations"

"denigrator"

"denigrators"

"denigratory"

"denim"

"denims"

"denizen"

"denizens"

"denmark"

"denned"

"denning"

"dennis"

"denominate"

"denominated"

"denominates"

"denominating"

"denomination"

"denominational"

"denominationally"

"denominations"

"denominator"

"denominators"

"denotation"

"denotations"

"denotative"

"denote"

"denoted"

"denotes"

"denoting"

"denotive"

"denouement"

"denouements"

"denounce"

"denounced"

"denouncement"

"denouncements"

"denouncer"

"denouncers"

"denounces"

"denouncing"

"dens"

"dense"

"densely"

"denseness"

"denser"

"densest"

"densified"

"densifies"

"densify"

"densifying"

"densimeter"

"densities"

"densitometer"

"densitometers"

"density"

"dent"

"dental"

"dentally"

"dentals"

"dentate"

"dented"

"dentifrice"

"dentifrices"

"dentin"

"dentinal"

"dentine"

"dentines"

"denting"

"dentins"

"dentist"

"dentistries"

"dentistry"

"dentists"

"dentition"

"dents"

"denture"

"dentures"

"denuclearization"

"denuclearize"

"denuclearized"

"denuclearizes"

"denuclearizing"

"denudate"

"denudation"

"denudations"

"denude"

"denuded"

"denuder"

"denuders"

"denudes"

"denuding"

"denunciate"

"denunciation"

"denunciations"

"denunciatory"

"denver"

"deny"

"denying"

"deodar"

"deodars"

"deodorant"

"deodorants"

"deodorize"

"deodorized"

"deodorizer"

"deodorizers"

"deodorizes"

"deodorizing"

"deontology"

"deoxidation"

"deoxidization"

"deoxidize"

"deoxidized"

"deoxidizer"

"deoxidizers"

"deoxidizes"

"deoxidizing"

"deoxygenate"

"deoxygenated"

"deoxygenating"

"deoxygenation"

"deoxyribonucleic"

"depart"

"departed"

"departing"

"department"

"departmental"

"departmentalism"

"departmentalization"

"departmentalize"

"departmentalized"

"departmentalizes"

"departmentalizing"

"departmentally"

"departments"

"departs"

"departure"

"departures"

"depend"

"dependabilities"

"dependability"

"dependable"

"dependableness"

"dependably"

"dependance"

"dependant"

"depended"

"dependence"

"dependencies"

"dependency"

"dependent"

"dependently"

"dependents"

"depending"

"depends"

"depersonalize"

"depersonalized"

"depersonalizes"

"depersonalizing"

"depict"

"depicted"

"depicter"

"depicters"

"depicting"

"depiction"

"depictions"

"depictor"

"depictors"

"depicts"

"depilate"

"depilated"

"depilates"

"depilating"

"depilation"

"depilatories"

"depilatory"

"deplane"

"deplaned"

"deplanes"

"deplaning"

"depletable"

"deplete"

"depleted"

"depletes"

"depleting"

"depletion"

"depletions"

"deplorable"

"deplorableness"

"deplorably"

"deplore"

"deplored"

"deplorer"

"deplorers"

"deplores"

"deploring"

"deploy"

"deployed"

"deploying"

"deployment"

"deployments"

"deploys"

"depolarization"

"depolarize"

"depolarized"

"depolarizer"

"depolarizers"

"depolarizes"

"depolarizing"

"depolished"

"depolishes"

"depoliticize"

"depoliticized"

"depoliticizes"

"depoliticizing"

"deponent"

"deponents"

"deponing"

"depopulate"

"depopulated"

"depopulates"

"depopulating"

"depopulation"

"depopulations"

"depopulator"

"depopulators"

"deport"

"deportability"

"deportable"

"deportation"

"deportations"

"deported"

"deportee"

"deportees"

"deporting"

"deportment"

"deports"

"deposable"

"deposal"

"deposals"

"depose"

"deposed"

"deposer"

"deposers"

"deposes"

"deposing"

"deposit"

"deposited"

"depositing"

"deposition"

"depositional"

"depositions"

"depositor"

"depositories"

"depositors"

"depository"

"deposits"

"depot"

"depots"

"deprave"

"depraved"

"depravedly"

"depravedness"

"depraver"

"depraves"

"depraving"

"depravities"

"depravity"

"deprecate"

"deprecated"

"deprecates"

"deprecating"

"deprecatingly"

"deprecation"

"deprecations"

"deprecative"

"deprecator"

"deprecators"

"deprecatory"

"depreciable"

"depreciate"

"depreciated"

"depreciates"

"depreciating"

"depreciatingly"

"depreciation"

"depreciations"

"depreciative"

"depreciatively"

"depreciator"

"depreciators"

"depreciatory"

"depredate"

"depredated"

"depredating"

"depredation"

"depredations"

"depredator"

"depredatory"

"deprehension"

"depress"

"depressant"

"depressants"

"depressed"

"depresses"

"depressibilities"

"depressibility"

"depressible"

"depressing"

"depressingly"

"depression"

"depressional"

"depressionary"

"depressions"

"depressive"

"depressively"

"depressives"

"depressor"

"depressors"

"deprival"

"deprivals"

"deprivation"

"deprivations"

"deprive"

"deprived"

"depriver"

"deprivers"

"deprives"

"depriving"

"deprogram"

"deprogrammed"

"deprogrammer"

"deprogrammers"

"deprogramming"

"deprogrammings"

"deprograms"

"dept"

"depth"

"depths"

"deputation"

"deputational"

"deputations"

"deputative"

"depute"

"deputed"

"deputes"

"deputies"

"deputing"

"deputize"

"deputized"

"deputizes"

"deputizing"

"deputy"

"der"

"derail"

"derailed"

"derailing"

"derailleur"

"derailleurs"

"derailment"

"derailments"

"derails"

"derange"

"deranged"

"derangement"

"derangements"

"deranges"

"deranging"

"derat"

"derats"

"deray"

"derbies"

"derby"

"deregulate"

"deregulated"

"deregulates"

"deregulating"

"deregulation"

"deregulations"

"derelict"

"dereliction"

"derelictions"

"derelicts"

"derestrict"

"deride"

"derided"

"derider"

"deriders"

"derides"

"deriding"

"deringer"

"derisible"

"derision"

"derisions"

"derisive"

"derisively"

"derisiveness"

"derisory"

"derivate"

"derivation"

"derivations"

"derivative"

"derivatives"

"derive"

"derived"

"deriver"

"derivers"

"derives"

"deriving"

"derm"

"derma"

"dermabrasion"

"dermal"

"dermas"

"dermatitis"

"dermatitises"

"dermatoglyphics"

"dermatological"

"dermatologies"

"dermatologist"

"dermatologists"

"dermatology"

"dermic"

"dermis"

"dermises"

"dermopathy"

"derms"

"dernier"

"derogate"

"derogated"

"derogates"

"derogating"

"derogation"

"derogations"

"derogatorily"

"derogatoriness"

"derogatory"

"derrick"

"derricks"

"derriere"

"derrieres"

"derries"

"derringer"

"derringers"

"derris"

"derrises"

"dervish"

"dervishes"

"des"

"desalinate"

"desalinated"

"desalinates"

"desalinating"

"desalination"

"desalinization"

"desalinize"

"desalinized"

"desalinizes"

"desalinizing"

"desalt"

"desalted"

"desalter"

"desalters"

"desalting"

"desalts"

"desand"

"descant"

"descanted"

"descanting"

"descants"

"descartes"

"descend"

"descendance"

"descendant"

"descendants"

"descended"

"descendence"

"descendent"

"descending"

"descends"

"descent"

"descents"

"describable"

"describe"

"described"

"describer"

"describers"

"describes"

"describing"

"descried"

"descrier"

"descriers"

"descries"

"description"

"descriptions"

"descriptive"

"descriptively"

"descriptiveness"

"descry"

"descrying"

"descString"

"desecrate"

"desecrated"

"desecrates"

"desecrating"

"desecration"

"desecrations"

"desecrator"

"desegregate"

"desegregated"

"desegregates"

"desegregating"

"desegregation"

"deselect"

"deselected"

"deselecting"

"deselects"

"desensitization"

"desensitizations"

"desensitize"

"desensitized"

"desensitizer"

"desensitizers"

"desensitizes"

"desensitizing"

"desert"

"deserted"

"deserter"

"deserters"

"desertic"

"deserting"

"desertion"

"desertions"

"deserts"

"deserve"

"deserved"

"deservedly"

"deserver"

"deservers"

"deserves"

"deserving"

"deservingly"

"desex"

"desexed"

"desexes"

"desexing"

"desexualization"

"desexualize"

"desexualized"

"desexualizing"

"desiccant"

"desiccants"

"desiccate"

"desiccated"

"desiccates"

"desiccating"

"desiccation"

"desiccations"

"desiccative"

"desiccator"

"desiccators"

"desiccatory"

"desiderata"

"desideratum"

"design"

"designate"

"designated"

"designates"

"designating"

"designation"

"designations"

"designative"

"designator"

"designed"

"designedly"

"designee"

"designees"

"designer"

"designers"

"designing"

"designment"

"designs"

"desilvered"

"desirability"

"desirable"

"desirably"

"desire"

"desireable"

"desired"

"desirer"

"desirers"

"desires"

"desiring"

"desirous"

"desist"

"desisted"

"desisting"

"desists"

"desk"

"deskman"

"deskmen"

"desks"

"desktop"

"desmology"

"desolate"

"desolated"

"desolately"

"desolateness"

"desolates"

"desolating"

"desolation"

"desolations"

"desoxyribonucleic"

"despair"

"despaired"

"despairing"

"despairingly"

"despairs"

"despatch"

"despatched"

"despatcher"

"despatchers"

"despatches"

"despatching"

"desperado"

"desperadoes"

"desperados"

"desperate"

"desperately"

"desperateness"

"desperation"

"despicable"

"despicably"

"despise"

"despised"

"despiser"

"despisers"

"despises"

"despising"

"despite"

"despited"

"despiteful"

"despitefully"

"despites"

"despiting"

"despoil"

"despoiled"

"despoiler"

"despoilers"

"despoiling"

"despoilment"

"despoilments"

"despoils"

"despoliation"

"despoliations"

"despond"

"desponded"

"despondence"

"despondencies"

"despondency"

"despondent"

"despondently"

"desponding"

"despondingly"

"desponds"

"despot"

"despotic"

"despotically"

"despotism"

"despotisms"

"despots"

"dessert"

"desserts"

"destain"

"destaining"

"destination"

"destinations"

"destine"

"destined"

"destines"

"destinies"

"destining"

"destiny"

"destitute"

"destitutely"

"destituteness"

"destitution"

"destressed"

"destrier"

"destriers"

"destroy"

"destroyable"

"destroyed"

"destroyer"

"destroyers"

"destroying"

"destroyingly"

"destroys"

"destruct"

"destructed"

"destructibility"

"destructible"

"destructing"

"destruction"

"destructions"

"destructive"

"destructively"

"destructiveness"

"destructor"

"destructors"

"destructs"

"desuetude"

"desuetudes"

"desugar"

"desugaring"

"desulfured"

"desultory"

"desynchronizing"

"detach"

"detachability"

"detachable"

"detachably"

"detached"

"detacher"

"detachers"

"detaches"

"detaching"

"detachment"

"detachments"

"detail"

"detailed"

"detailer"

"detailers"

"detailing"

"details"

"detain"

"detained"

"detainee"

"detainees"

"detainer"

"detainers"

"detaining"

"detainment"

"detains"

"detect"

"detectable"

"detectably"

"detected"

"detecter"

"detecters"

"detectible"

"detecting"

"detection"

"detections"

"detective"

"detectives"

"detector"

"detectors"

"detects"

"detent"

"detente"

"detentes"

"detention"

"detents"

"deter"

"deterge"

"deterged"

"detergent"

"detergents"

"deterger"

"deterges"

"deteriorate"

"deteriorated"

"deteriorates"

"deteriorating"

"deterioration"

"deteriorations"

"deteriorative"

"determent"

"determents"

"determinability"

"determinable"

"determinableness"

"determinably"

"determinacy"

"determinant"

"determinants"

"determinate"

"determinateness"

"determination"

"determinations"

"determinative"

"determine"

"determined"

"determinedly"

"determinedness"

"determines"

"determining"

"determinism"

"determinist"

"deterministic"

"determinists"

"deterred"

"deterrence"

"deterrent"

"deterrents"

"deterrer"

"deterrers"

"deterring"

"deters"

"detest"

"detestable"

"detestably"

"detestation"

"detestations"

"detested"

"detester"

"detesters"

"detesting"

"detests"

"dethrone"

"dethroned"

"dethronement"

"dethronements"

"dethroner"

"dethrones"

"dethroning"

"detonable"

"detonate"

"detonated"

"detonates"

"detonating"

"detonation"

"detonations"

"detonator"

"detonators"

"detour"

"detoured"

"detouring"

"detournement"

"detours"

"detoxicated"

"detoxicating"

"detoxication"

"detoxicator"

"detoxification"

"detoxified"

"detoxifier"

"detoxifies"

"detoxify"

"detoxifying"

"detract"

"detracted"

"detracting"

"detraction"

"detractions"

"detractive"

"detractor"

"detractors"

"detracts"

"detrain"

"detrained"

"detraining"

"detrains"

"detriment"

"detrimental"

"detrimentally"

"detrimentalness"

"detriments"

"detrital"

"detritus"

"detroit"

"detumescence"

"detumescent"

"deuce"

"deuced"

"deucedly"

"deuces"

"deucing"

"deus"

"deuterium"

"deuteron"

"deuteronomy"

"deuterons"

"deutsche"

"deutschland"

"deux"

"deva"

"devaluate"

"devaluated"

"devaluates"

"devaluating"

"devaluation"

"devaluations"

"devalue"

"devalued"

"devalues"

"devaluing"

"devas"

"devastate"

"devastated"

"devastates"

"devastating"

"devastatingly"

"devastation"

"devastations"

"devastative"

"devastator"

"devastators"

"devein"

"deveined"

"deveining"

"deveins"

"develop"

"develope"

"developed"

"developer"

"developers"

"developes"

"developing"

"development"

"developmental"

"developmentally"

"developments"

"develops"

"devest"

"deviance"

"deviances"

"deviancies"

"deviancy"

"deviant"

"deviants"

"deviate"

"deviated"

"deviates"

"deviating"

"deviation"

"deviational"

"deviations"

"deviator"

"deviators"

"device"

"devices"

"devil"

"deviled"

"deviling"

"devilish"

"devilishly"

"devilishness"

"devilkin"

"devilled"

"devilling"

"devilment"

"devilments"

"devilries"

"devilry"

"devils"

"deviltries"

"deviltry"

"devious"

"deviously"

"deviousness"

"devisable"

"devisal"

"devisals"

"devise"

"devised"

"devisee"

"devisees"

"deviser"

"devisers"

"devises"

"devising"

"devisor"

"devisors"

"devitalize"

"devitalized"

"devitalizes"

"devitalizing"

"devoice"

"devoicing"

"devoid"

"devoir"

"devoirs"

"devolution"

"devolutionary"

"devolutive"

"devolve"

"devolved"

"devolvement"

"devolvements"

"devolves"

"devolving"

"devon"

"devonian"

"devote"

"devoted"

"devotedly"

"devotedness"

"devotee"

"devotees"

"devotes"

"devoting"

"devotion"

"devotional"

"devotions"

"devour"

"devoured"

"devourer"

"devourers"

"devouring"

"devours"

"devout"

"devoutly"

"devoutness"

"dew"

"dewatering"

"dewax"

"dewaxed"

"dewaxes"

"dewberries"

"dewberry"

"dewclaw"

"dewclaws"

"dewdrop"

"dewdrops"

"dewed"

"dewfall"

"dewfalls"

"dewier"

"dewiest"

"dewily"

"dewiness"

"dewing"

"dewlap"

"dewlapped"

"dewlaps"

"dewless"

"dewool"

"deworm"

"dews"

"dewy"

"dexes"

"dexies"

"dexter"

"dexterity"

"dexterous"

"dexterously"

"dexterousness"

"dextral"

"dextrin"

"dextrins"

"dextro"

"dextrorotary"

"dextrose"

"dextroses"

"dextrous"

"dezinc"

"dharma"

"dharmas"

"dharmic"

"dhole"

"dholes"

"dhoti"

"dhotis"

"dhow"

"dhows"

"dhyana"

"diabetes"

"Diabetes mellitus"

"diabetic"

"diabetic ketoacidosis"

"diabetics"

"diablery"

"diabolic"

"diabolical"

"diabolically"

"diabolo"

"diabology"

"diabolos"

"diacritic"

"diacritical"

"diacritics"

"diadem"

"diademed"

"diadems"

"diadic"

"diaeresis"

"diag"

"diagnosable"

"diagnose"

"diagnoseable"

"diagnosed"

"diagnoses"

"diagnosing"

"diagnosis"

"diagnosis guide"

"diagnosis of lesions"

"diagnostic"

"DIAGNOSTIC CRITERIA"

"diagnostically"

"diagnostician"

"diagnosticians"

"diagnostics"

"diagometer"

"diagonal"

"diagonally"

"diagonals"

"diagram"

"diagramed"

"diagraming"

"diagrammable"

"diagrammatic"

"diagrammatical"

"diagrammatically"

"diagrammed"

"diagrammer"

"diagramming"

"diagrams"

"diagraph"

"diagraphics"

"diagraphs"

"dial"

"dialect"

"dialectal"

"dialectic"

"dialectical"

"dialectics"

"dialectology"

"dialects"

"dialed"

"dialer"

"dialers"

"dialing"

"dialings"

"dialist"

"dialists"

"diallage"

"dialled"

"dialler"

"diallers"

"dialling"

"diallings"

"diallist"

"dialog"

"dialoger"

"dialogged"

"dialogic"

"dialogs"

"dialogue"

"dialogued"

"dialogues"

"dialoguing"

"dials"

"dialyse"

"dialysed"

"dialyser"

"dialyses"

"dialysis"

"dialytic"

"dialyze"

"dialyzed"

"dialyzer"

"dialyzes"

"diam"

"diamagnetic"

"diamagnetism"

"diameter"

"diameters"

"diametric"

"diametrical"

"diametrically"

"diamond"

"diamondback"

"diamondbacks"

"diamonding"

"diamonds"

"diana"

"diane"

"dianthus"

"dianthuses"

"diapason"

"diapasons"

"diaper"

"diapered"

"diapering"

"diapers"

"diaphanometer"

"diaphanous"

"diaphoretic"

"diaphoretics"

"diaphragm"

"diaphragmatic"

"diaphragms"

"diarchy"

"diaries"

"diarist"

"diarists"

"diarrhea"

"diarrheal"

"diarrheas"

"diarrhoeal"

"diarrhoeic"

"diary"

"dias"

"diaspora"

"diasporas"

"diaspore"

"diastole"

"diastoles"

"diastolic"

"diastrophic"

"diastrophism"

"diathermic"

"diathermies"

"diathermy"

"diatom"

"diatomic"

"diatomite"

"diatoms"

"diatonic"

"diatribe"

"diatribes"

"diazepam"

"diazo"

"dibbed"

"dibber"

"dibbers"

"dibbing"

"dibble"

"dibbled"

"dibbler"

"dibblers"

"dibbles"

"dibbling"

"dibbuk"

"dibbukim"

"dibbuks"

"dibs"

"dicasts"

"dice"

"diced"

"dicer"

"dicers"

"dices"

"dicey"

"dichotic"

"dichotomies"

"dichotomous"

"dichotomously"

"dichotomy"

"dichromatic"

"dichromatism"

"dichroscope"

"dicier"

"diciest"

"dicing"

"dick"

"dickens"

"dickenses"

"dickensian"

"dicker"

"dickered"

"dickering"

"dickers"

"dickey"

"dickeys"

"dickie"

"dickies"

"dicks"

"dicky"

"dicot"

"dicots"

"dicotyledon"

"dicotyledonous"

"dicotyledons"

"dict"

"dicta"

"dictaphone"

"dictaphones"

"dictate"

"dictated"

"dictates"

"dictating"

"dictation"

"dictations"

"dictator"

"dictatorial"

"dictatorially"

"dictatorialness"

"dictators"

"dictatorship"

"dictatorships"

"dictatory"

"diction"

"dictionaries"

"dictionary"

"dictions"

"dictronics"

"dictum"

"dictums"

"did"

"didactic"

"didactically"

"didacticism"

"didacts"

"diddle"

"diddled"

"diddler"

"diddlers"

"diddles"

"diddling"

"didies"

"dido"

"didoes"

"didos"

"didst"

"didy"

"die"

"dieback"

"diebacks"

"died"

"diehard"

"diehards"

"dieing"

"dieldrin"

"dielectric"

"dielectrics"

"diem"

"diemaker"

"diemakers"

"diereses"

"dieresis"

"dies"

"diesel"

"diesels"

"dieses"

"diestock"

"diestocks"

"diet"

"dietary"

"dietary methods"

"dieted"

"dieter"

"dieters"

"dietetic"

"dietetically"

"dietetics"

"diethylamide"

"dietician"

"dieticians"

"dieting"

"dietitian"

"dietitians"

"diets"

"differ"

"differed"

"difference"

"differences"

"different"

"differentia"

"differentiable"

"differentiae"

"differential"

"differentially"

"differentials"

"differentiate"

"differentiated"

"differentiates"

"differentiating"

"differentiation"

"differentiations"

"differently"

"differing"

"differs"

"difficult"

"difficulties"

"difficultly"

"difficulty"

"diffidence"

"diffident"

"diffidently"

"diffract"

"diffracted"

"diffraction"

"diffractions"

"diffractive"

"diffractometer"

"diffracts"

"diffuse"

"diffused"

"diffusely"

"diffuseness"

"diffuser"

"diffusers"

"diffuses"

"diffusing"

"diffusion"

"diffusions"

"diffusive"

"diffusor"

"diffusors"

"dig"

"digamy"

"digest"

"digestant"

"digested"

"digester"

"digesters"

"digestibility"

"digestible"

"digesting"

"digestion"

"digestive"

"Digestive System"

"digestively"

"digestiveness"

"digestor"

"digestors"

"digests"

"digged"

"digger"

"diggers"

"digging"

"diggings"

"dight"

"dighted"

"dights"

"digit"

"digital"

"digitalis"

"digitalization"

"digitalize"

"digitalized"

"digitalizing"

"digitally"

"digitals"

"digitate"

"digitization"

"digitize"

"digitized"

"digitizes"

"digitizing"

"digits"

"diglots"

"dignified"

"dignifiedly"

"dignifies"

"dignify"

"dignifying"

"dignitaries"

"dignitary"

"dignities"

"dignity"

"digraph"

"digraphs"

"digress"

"digressed"

"digresses"

"digressing"

"digression"

"digressions"

"digressive"

"digressively"

"digs"

"dihedral"

"dihedrals"

"dihedron"

"dikdik"

"dikdiks"

"dike"

"diked"

"diker"

"dikers"

"dikes"

"diking"

"dilantin"

"dilapidate"

"dilapidated"

"dilapidating"

"dilapidation"

"dilapidator"

"dilatant"

"dilatants"

"dilatate"

"dilatation"

"dilatations"

"dilatator"

"dilate"

"dilated"

"dilater"

"dilaters"

"dilates"

"dilating"

"dilation"

"dilations"

"dilative"

"dilatometer"

"dilator"

"dilatorily"

"dilatoriness"

"dilators"

"dilatory"

"dildo"

"dildoe"

"dildoes"

"dildos"

"dilemma"

"dilemmas"

"dilemmic"

"dilettante"

"dilettantes"

"dilettanti"

"dilettantish"

"dilettantism"

"diligence"

"diligent"

"diligently"

"dill"

"dillies"

"dills"

"dilly"

"dillydallied"

"dillydallies"

"dillydally"

"dillydallying"

"diluent"

"diluents"

"dilute"

"diluted"

"diluter"

"diluters"

"dilutes"

"diluting"

"dilution"

"dilutions"

"dilutive"

"dilutor"

"dilutors"

"diluvial"

"diluvian"

"diluvion"

"diluvium"

"dim"

"dime"

"dimension"

"dimensional"

"dimensionality"

"dimensions"

"dimer"

"dimers"

"dimes"

"diminish"

"diminished"

"diminishes"

"diminishing"

"diminishment"

"diminishments"

"diminuendo"

"diminuendos"

"diminution"

"diminutions"

"diminutive"

"dimities"

"dimity"

"dimly"

"dimmable"

"dimmed"

"dimmer"

"dimmers"

"dimmest"

"dimming"

"dimmock"

"dimness"

"dimorph"

"dimorphic"

"dimorphism"

"dimorphisms"

"dimorphous"

"dimout"

"dimouts"

"dimple"

"dimpled"

"dimples"

"dimpling"

"dimply"

"dims"

"dimwit"

"dimwits"

"dimwitted"

"dimwittedness"

"din"

"dinar"

"dinars"

"dine"

"dined"

"diner"

"dineros"

"diners"

"dines"

"dinette"

"dinettes"

"ding"

"dingbat"

"dingbats"

"dingdong"

"dingdonged"

"dingdongs"

"dinged"

"dingey"

"dingeys"

"dinghies"

"dinghy"

"dingier"

"dingiest"

"dingily"

"dinginess"

"dinging"

"dingle"

"dingles"

"dingo"

"dingoes"

"dings"

"dingus"

"dinguses"

"dingy"

"dining"

"dinkier"

"dinkies"

"dinkiest"

"dinking"

"dinkum"

"dinky"

"dinned"

"dinner"

"dinners"

"dinnertime"

"dinnerware"

"dinning"

"dinomania"

"dinosaur"

"dinosaurs"

"dins"

"dint"

"dinted"

"dinting"

"dints"

"diocesan"

"diocese"

"dioceses"

"diode"

"diodes"

"diogenes"

"dionysian"

"dionysus"

"diopter"

"diopters"

"dioptometer"

"dioptre"

"dioptrics"

"diorama"

"dioramas"

"dioramic"

"diorites"

"dioritic"

"dioxane"

"dioxide"

"dioxides"

"dioxids"

"dioxin"

"dip"

"diphtheria"

"diphtherial"

"diphtherian"

"diphtheric"

"diphtheritic"

"diphthong"

"diphthongs"

"dipleidoscope"

"diplex"

"diplograph"

"diploid"

"diploids"

"diploidy"

"diploma"

"diplomacies"

"diplomacy"

"diplomas"

"diplomat"

"diplomate"

"diplomates"

"diplomatic"

"diplomatically"

"diplomatics"

"diplomatique"

"diplomatist"

"diplomatists"

"diplomatology"

"diplomats"

"diplopod"

"dipody"

"dipole"

"dipoles"

"dippable"

"dipped"

"dipper"

"dippers"

"dippier"

"dippiest"

"dipping"

"dippings"

"dippy"

"dips"

"dipsomania"

"dipsomaniac"

"dipsomaniacal"

"dipsomaniacs"

"dipstick"

"dipsticks"

"dipt"

"diptera"

"dipterous"

"diptyca"

"diptych"

"diptychs"

"dire"

"direct"

"directed"

"directer"

"directest"

"directing"

"direction"

"directional"

"directionally"

"directions"

"directive"

"directives"

"directly"

"directness"

"director"

"directorate"

"directorates"

"directories"

"directors"

"directorship"

"directorships"

"directory"

"directs"

"direful"

"direfully"

"direly"

"direness"

"direr"

"direst"

"dirge"

"dirgeful"

"dirges"

"dirigible"

"dirigibles"

"dirk"

"dirked"

"dirking"

"dirks"

"dirndl"

"dirndls"

"dirt"

"dirtied"

"dirtier"

"dirties"

"dirtiest"

"dirtily"

"dirtiness"

"dirts"

"dirty"

"dirtying"

"dis"

"disabilities"

"disability"

"disable"

"disabled"

"disablement"

"disabler"

"disables"

"disabling"

"disabuse"

"disabused"

"disabuses"

"disabusing"

"disaccharide"

"disaccharides"

"disacknowledgements"

"disadvantage"

"disadvantaged"

"disadvantageous"

"disadvantageously"

"disadvantageousness"

"disadvantages"

"disaffect"

"disaffected"

"disaffectedly"

"disaffecting"

"disaffection"

"disaffections"

"disaffects"

"disaffiliate"

"disaffiliated"

"disaffiliates"

"disaffiliating"

"disaffiliation"

"disaffiliations"

"disaffirmance"

"disaffirmation"

"disaggregation"

"disagree"

"disagreeable"

"disagreeableness"

"disagreeably"

"disagreed"

"disagreeing"

"disagreement"

"disagreements"

"disagrees"

"disallow"

"disallowance"

"disallowances"

"disallowed"

"disallowing"

"disallows"

"disannul"

"disannulled"

"disannulling"

"disappear"

"disappearance"

"disappearances"

"disappeared"

"disappearing"

"disappears"

"disappoint"

"disappointed"

"disappointing"

"disappointment"

"disappointments"

"disappoints"

"disapprobation"

"disapprobations"

"disapproval"

"disapprovals"

"disapprove"

"disapproved"

"disapproves"

"disapproving"

"disapprovingly"

"disarm"

"disarmament"

"disarmed"

"disarmer"

"disarmers"

"disarming"

"disarmingly"

"disarms"

"disarrange"

"disarranged"

"disarrangement"

"disarrangements"

"disarranges"

"disarranging"

"disarray"

"disarrayed"

"disarraying"

"disarrays"

"disarticulate"

"disarticulated"

"disarticulating"

"disarticulation"

"disassemble"

"disassembled"

"disassembles"

"disassembling"

"disassembly"

"disassimilate"

"disassimilated"

"disassimilating"

"disassimilation"

"disassimilative"

"disassociate"

"disassociated"

"disassociates"

"disassociating"

"disassociation"

"disaster"

"disasters"

"disastrous"

"disastrously"

"disavow"

"disavowal"

"disavowals"

"disavowed"

"disavowing"

"disavows"

"disband"

"disbanded"

"disbanding"

"disbandment"

"disbandments"

"disbands"

"disbar"

"disbarment"

"disbarments"

"disbarred"

"disbarring"

"disbars"

"disbelief"

"disbeliefs"

"disbelieve"

"disbelieved"

"disbeliever"

"disbelievers"

"disbelieves"

"disbelieving"

"disbosom"

"disbound"

"disbowel"

"disburden"

"disburdened"

"disburdening"

"disburdens"

"disbursal"

"disburse"

"disbursed"

"disbursement"

"disbursements"

"disburser"

"disburses"

"disbursing"

"disc"

"discants"

"discard"

"discarded"

"discarding"

"discards"

"discase"

"discased"

"discases"

"disced"

"discern"

"discernable"

"discerned"

"discerner"

"discerners"

"discernible"

"discerning"

"discerningly"

"discernment"

"discerns"

"discharge"

"dischargeable"

"discharged"

"discharger"

"dischargers"

"discharges"

"discharging"

"discing"

"disciple"

"disciples"

"discipleship"

"disciplinarian"

"disciplinarians"

"disciplinary"

"discipline"

"disciplined"

"discipliner"

"discipliners"

"disciplines"

"discipling"

"disciplining"

"disclaim"

"disclaimant"

"disclaimed"

"disclaimer"

"disclaimers"

"disclaiming"

"disclaims"

"disclamation"

"disclamatory"

"disclose"

"disclosed"

"discloser"

"discloses"

"disclosing"

"disclosure"

"disclosures"

"disco"

"discoblastic"

"discographies"

"discography"

"discoid"

"discoids"

"discolor"

"discoloration"

"discolorations"

"discolored"

"discoloring"

"discolors"

"discomania"

"discombobulate"

"discombobulated"

"discombobulates"

"discombobulating"

"discombobulation"

"discomfit"

"discomfited"

"discomfiting"

"discomfits"

"discomfiture"

"discomfort"

"discomforted"

"discomforting"

"discomforts"

"discommode"

"discommoded"

"discommodes"

"discommoding"

"discompose"

"discomposed"

"discomposes"

"discomposing"

"discomposure"

"disconcert"

"disconcerted"

"disconcerting"

"disconcertingly"

"disconcertment"

"disconcerts"

"disconnect"

"disconnected"

"disconnecting"

"disconnection"

"disconnections"

"disconnects"

"disconsolate"

"disconsolately"

"disconsolateness"

"discontent"

"discontented"

"discontentedly"

"discontentedness"

"discontenting"

"discontentment"

"discontentments"

"discontents"

"discontinuance"

"discontinuances"

"discontinuation"

"discontinuations"

"discontinue"

"discontinued"

"discontinues"

"discontinuing"

"discontinuities"

"discontinuity"

"discontinuous"

"discontinuously"

"discord"

"discordance"

"discordant"

"discordantly"

"discording"

"discords"

"discos"

"discotheque"

"discotheques"

"discount"

"Discount Card"

"discountable"

"discounted"

"discountenance"

"discountenanced"

"discountenances"

"discountenancing"

"discounter"

"discounters"

"discounting"

"discountinuous"

"discounts"

"discourage"

"discouraged"

"discouragement"

"discouragements"

"discourages"

"discouraging"

"discouragingly"

"discourse"

"discoursed"

"discourser"

"discoursers"

"discourses"

"discoursing"

"discourteous"

"discourteously"

"discourtesies"

"discourtesy"

"discover"

"discoverable"

"discovered"

"discoverer"

"discoverers"

"discoveries"

"discovering"

"discovers"

"discovery"

"discredit"

"discreditable"

"discredited"

"discrediting"

"discredits"

"discreet"

"discreeter"

"discreetly"

"discrepancies"

"discrepancy"

"discrepant"

"discrepantly"

"discrete"

"discretely"

"discretion"

"discretional"

"discretionary"

"discriminate"

"discriminated"

"discriminately"

"discriminates"

"discriminating"

"discrimination"

"discriminational"

"discriminations"

"discriminator"

"discriminatory"

"discrown"

"discrowned"

"discs"

"discursive"

"discursively"

"discursiveness"

"discus"

"discuses"

"discuss"

"discussant"

"discussants"

"discussed"

"discusses"

"discussing"

"discussion"

"discussion forum"

"discussionis"

"discussions"

"disdain"

"disdained"

"disdainful"

"disdainfully"

"disdaining"

"disdains"

"disease"

"diseased"

"diseases"

"diseasing"

"disembark"

"disembarkation"

"disembarkations"

"disembarked"

"disembarking"

"disembarks"

"disembodied"

"disembodies"

"disembodiment"

"disembodiments"

"disembody"

"disembodying"

"disembowel"

"disemboweled"

"disemboweling"

"disembowelled"

"disembowelling"

"disembowelment"

"disembowelments"

"disembowels"

"disemploy"

"disemployed"

"disemploying"

"disemployment"

"disemploys"

"disenchant"

"disenchanted"

"disenchanting"

"disenchantingly"

"disenchantment"

"disenchantments"

"disenchants"

"disencumber"

"disencumbered"

"disencumbering"

"disencumbers"

"disenfranchise"

"disenfranchised"

"disenfranchisement"

"disenfranchisements"

"disenfranchises"

"disenfranchising"

"disengage"

"disengaged"

"disengagement"

"disengagements"

"disengages"

"disengaging"

"disentailment"

"disentangle"

"disentangled"

"disentanglement"

"disentanglements"

"disentangles"

"disentangling"

"disenthrall"

"disenthralled"

"disenthralling"

"disenthralls"

"disentitle"

"disentitling"

"disequilibria"

"disequilibrium"

"disequilibriums"

"disestablish"

"disestablished"

"disestablishes"

"disestablishing"

"disestablishment"

"disestablismentarian"

"disestablismentarianism"

"disesteem"

"disfavor"

"disfavored"

"disfavors"

"disfigure"

"disfigured"

"disfigurement"

"disfigurements"

"disfigurer"

"disfigures"

"disfiguring"

"disfiguringly"

"disfranchise"

"disfranchised"

"disfranchisement"

"disfranchisements"

"disfranchiser"

"disfranchisers"

"disfranchises"

"disfranchising"

"disfrocked"

"disfrocks"

"disfunction"

"disgorge"

"disgorged"

"disgorges"

"disgorging"

"disgrace"

"disgraced"

"disgraceful"

"disgracefully"

"disgracefulness"

"disgracer"

"disgracers"

"disgraces"

"disgracing"

"disgruntle"

"disgruntled"

"disgruntles"

"disgruntling"

"disguise"

"disguised"

"disguisement"

"disguisements"

"disguises"

"disguising"

"disgust"

"disgusted"

"disgustedly"

"disgusting"

"disgustingly"

"disgusts"

"dish"

"dishabille"

"disharmonies"

"disharmonious"

"disharmony"

"dishcloth"

"dishcloths"

"dishearten"

"disheartened"

"disheartening"

"dishearteningly"

"disheartenment"

"disheartens"

"dished"

"dishes"

"dishevel"

"disheveled"

"disheveling"

"dishevelled"

"dishevelling"

"dishevelment"

"dishevelments"

"dishevels"

"dishful"

"dishfuls"

"dishier"

"dishing"

"dishonest"

"dishonesties"

"dishonestly"

"dishonesty"

"dishonor"

"dishonorable"

"dishonorableness"

"dishonorably"

"dishonored"

"dishonoring"

"dishonors"

"dishpan"

"dishpans"

"dishrag"

"dishrags"

"dishtowel"

"dishtowels"

"dishware"

"dishwares"

"dishwasher"

"dishwashers"

"dishwater"

"dishy"

"disillusion"

"disillusioned"

"disillusioning"

"disillusionment"

"disillusionments"

"disillusions"

"disinclination"

"disinclinations"

"disincline"

"disinclined"

"disinclines"

"disinclining"

"disincorporate"

"disincorporated"

"disincorporating"

"disincorporation"

"disinfect"

"disinfectant"

"disinfectants"

"disinfected"

"disinfecting"

"disinfection"

"disinfections"

"disinfects"

"disinfestant"

"disinfestation"

"disinformation"

"disingenuous"

"disinherit"

"disinheritance"

"disinheritances"

"disinherited"

"disinheriting"

"disinherits"

"disintegrate"

"disintegrated"

"disintegrates"

"disintegrating"

"disintegration"

"disintegrations"

"disintegrative"

"disintegrator"

"disintegrators"

"disinter"

"disinterest"

"disinterested"

"disinterestedly"

"disinterestedness"

"disinterred"

"disinterring"

"disinters"

"disintoxication"

"disjoin"

"disjoined"

"disjoining"

"disjoins"

"disjoint"

"disjointed"

"disjointedly"

"disjointedness"

"disjointing"

"disjoints"

"disjunct"

"disjunctive"

"disjuncts"

"disk"

"disked"

"diskette"

"diskettes"

"disking"

"disks"

"dislike"

"disliked"

"disliker"

"dislikes"

"disliking"

"dislocate"

"dislocated"

"dislocates"

"dislocating"

"dislocation"

"dislocations"

"dislodge"

"dislodged"

"dislodges"

"dislodging"

"disloyal"

"disloyally"

"disloyalties"

"disloyalty"

"dismal"

"dismaler"

"dismalest"

"dismally"

"dismalness"

"dismals"

"dismantle"

"dismantled"

"dismantlement"

"dismantles"

"dismantling"

"dismast"

"dismasting"

"dismay"

"dismayed"

"dismaying"

"dismays"

"dismember"

"dismembered"

"dismembering"

"dismemberment"

"dismemberments"

"dismembers"

"dismes"

"dismiss"

"dismissal"

"dismissals"

"dismissed"

"dismisses"

"dismissing"

"dismortgage"

"dismortgaged"

"dismortgaging"

"dismount"

"dismountable"

"dismounted"

"dismounting"

"dismounts"

"disney"

"disneyland"

"disobedience"

"disobedient"

"disobediently"

"disobey"

"disobeyed"

"disobeyer"

"disobeyers"

"disobeying"

"disobeys"

"disoblige"

"disobliged"

"disobliges"

"disobliging"

"disorder"

"disordered"

"disordering"

"disorderliness"

"disorderly"

"disorders"

"disorganization"

"disorganize"

"disorganized"

"disorganizer"

"disorganizers"

"disorganizes"

"disorganizing"

"disorient"

"disorientate"

"disorientated"

"disorientates"

"disorientating"

"disorientation"

"disoriented"

"disorienting"

"disorients"

"disown"

"disowned"

"disowning"

"disownment"

"disowns"

"disparage"

"disparaged"

"disparagement"

"disparagements"

"disparages"

"disparaging"

"disparagingly"

"disparate"

"disparately"

"disparities"

"disparity"

"dispassion"

"dispassionate"

"dispassionately"

"dispatch"

"dispatched"

"dispatcher"

"dispatchers"

"dispatches"

"dispatching"

"dispel"

"dispelled"

"dispelling"

"dispels"

"dispending"

"dispensable"

"dispensaries"

"dispensary"

"dispensation"

"dispensations"

"dispensatory"

"dispense"

"dispensed"

"dispenser"

"dispensers"

"dispenses"

"dispensing"

"dispersal"

"dispersals"

"disperse"

"dispersed"

"dispersement"

"disperses"

"dispersing"

"dispersion"

"dispersions"

"dispirit"

"dispirited"

"dispiriting"

"dispirits"

"displace"

"displaced"

"displacement"

"displacements"

"displaces"

"displacing"

"displanted"

"display"

"displayable"

"displayed"

"displaying"

"displays"

"displease"

"displeased"

"displeases"

"displeasing"

"displeasure"

"displeasures"

"disport"

"disported"

"disporting"

"disports"

"disposable"

"disposal"

"disposals"

"dispose"

"disposed"

"disposer"

"disposers"

"disposes"

"disposing"

"disposition"

"dispositions"

"dispositive"

"dispossess"

"dispossessed"

"dispossesses"

"dispossessing"

"dispossession"

"dispossessor"

"dispossessory"

"dispraise"

"disproof"

"disproofs"

"disproportion"

"disproportional"

"disproportionate"

"disproportionately"

"disproportionates"

"disproportions"

"disprovable"

"disprove"

"disproved"

"disproven"

"disproves"

"disproving"

"disputability"

"disputable"

"disputably"

"disputant"

"disputants"

"disputation"

"disputations"

"disputatious"

"dispute"

"disputed"

"disputer"

"disputers"

"disputes"

"disputing"

"disqualification"

"disqualifications"

"disqualified"

"disqualifies"

"disqualify"

"disqualifying"

"disquiet"

"disquieted"

"disquieting"

"disquietingly"

"disquiets"

"disquietude"

"disquietudes"

"disquisition"

"disquisitions"

"disraeli"

"disregard"

"disregarded"

"disregardful"

"disregarding"

"disregards"

"disrepair"

"disreputability"

"disreputable"

"disreputably"

"disrepute"

"disrespect"

"disrespectable"

"disrespectful"

"disrespectfully"

"disrobe"

"disrobed"

"disrober"

"disrobers"

"disrobes"

"disrobing"

"disrupt"

"disrupted"

"disrupter"

"disrupting"

"disruption"

"disruptions"

"disruptive"

"disruptively"

"disruptiveness"

"disrupts"

"dissatisfaction"

"dissatisfactions"

"dissatisfied"

"dissatisfies"

"dissatisfy"

"dissatisfying"

"dissect"

"dissected"

"dissecting"

"dissection"

"dissections"

"dissector"

"dissectors"

"dissects"

"dissemblance"

"dissemble"

"dissembled"

"dissembler"

"dissemblers"

"dissembles"

"dissembling"

"dissemblingly"

"disseminate"

"disseminated"

"disseminates"

"disseminating"

"dissemination"

"disseminations"

"dissension"

"dissensions"

"dissent"

"dissented"

"dissenter"

"dissenters"

"dissentient"

"dissentients"

"dissenting"

"dissents"

"dissepimental"

"dissert"

"dissertation"

"dissertations"

"disserts"

"disserve"

"disservice"

"disservices"

"dissever"

"dissevered"

"dissevering"

"dissevers"

"dissidence"

"dissident"

"dissidently"

"dissidents"

"dissimilar"

"dissimilarities"

"dissimilarity"

"dissimilate"

"dissimilitude"

"dissimulate"

"dissimulated"

"dissimulates"

"dissimulating"

"dissimulation"

"dissimulations"

"dissimulator"

"dissimulators"

"dissipate"

"dissipated"

"dissipater"

"dissipaters"

"dissipates"

"dissipating"

"dissipation"

"dissipations"

"dissipator"

"dissipators"

"dissociate"

"dissociated"

"dissociates"

"dissociating"

"dissociation"

"dissociations"

"dissociative"

"dissolute"

"dissolutely"

"dissoluteness"

"dissolution"

"dissolutions"

"dissolutive"

"dissolvability"

"dissolvable"

"dissolve"

"dissolved"

"dissolves"

"dissolving"

"dissonance"

"dissonances"

"dissonant"

"dissonantly"

"dissuadable"

"dissuade"

"dissuaded"

"dissuader"

"dissuades"

"dissuading"

"dissuasion"

"dissuasions"

"dissuasive"

"dissuasively"

"dissuasiveness"

"distaff"

"distaffs"

"distal"

"distally"

"distance"

"Distance-based activity"

"distanced"

"distances"

"distancing"

"distant"

"distantly"

"distantness"

"distaste"

"distasted"

"distasteful"

"distastefully"

"distastefulness"

"distastes"

"distasting"

"distemper"

"distend"

"distended"

"distending"

"distends"

"distensibilities"

"distensibility"

"distensible"

"distension"

"distensions"

"distent"

"distention"

"distentions"

"distich"

"distichs"

"distill"

"distillable"

"distillate"

"distillates"

"distillation"

"distillations"

"distilled"

"distiller"

"distilleries"

"distillers"

"distillery"

"distilling"

"distills"

"distils"

"distinct"

"distincter"

"distinction"

"distinctions"

"distinctive"

"distinctively"

"distinctiveness"

"distinctly"

"distinctness"

"distinguish"

"distinguishable"

"distinguishably"

"distinguished"

"distinguishes"

"distinguishing"

"distort"

"distortable"

"distorted"

"distorter"

"distorters"

"distorting"

"distortion"

"distortional"

"distortions"

"distorts"

"distr"

"distract"

"distracted"

"distractedly"

"distractibility"

"distracting"

"distractingly"

"distraction"

"distractions"

"distractive"

"distracts"

"distrain"

"distraint"

"distrait"

"distraught"

"distress"

"distressed"

"distresses"

"distressful"

"distressfully"

"distressing"

"distressingly"

"distributable"

"distribute"

"distributed"

"distributee"

"distributer"

"distributes"

"distributing"

"distribution"

"distributions"

"distributive"

"distributively"

"distributor"

"distributors"

"distributorship"

"distributution"

"district"

"districted"

"districts"

"distrust"

"distrusted"

"distrustful"

"distrustfully"

"distrustfulness"

"distrusting"

"distrusts"

"disturb"

"disturbance"

"disturbances"

"disturbed"

"disturber"

"disturbers"

"disturbing"

"disturbingly"

"disturbs"

"disunion"

"disunite"

"disunited"

"disuniter"

"disuniters"

"disunites"

"disunities"

"disuniting"

"disunity"

"disuse"

"disused"

"disuses"

"disusing"

"disvaluing"

"disyoke"

"dit"

"ditch"

"ditched"

"ditcher"

"ditchers"

"ditches"

"ditching"

"ditchless"

"dites"

"dither"

"dithered"

"dithering"

"dithers"

"dithery"

"ditties"

"ditto"

"dittoed"

"dittoes"

"dittoing"

"dittos"

"ditty"

"diuretic"

"diuretically"

"diuretics"

"diurnal"

"diurnally"

"diurnals"

"diva"

"divagate"

"divagated"

"divagates"

"divagating"

"divagation"

"divagations"

"divalent"

"divan"

"divans"

"divas"

"dive"

"dived"

"diver"

"diverge"

"diverged"

"divergence"

"divergences"

"divergent"

"divergently"

"diverges"

"diverging"

"divers"

"diverse"

"diversely"

"diverseness"

"diversification"

"diversifications"

"diversified"

"diversifies"

"diversify"

"diversifying"

"diversion"

"diversionary"

"diversionist"

"diversions"

"diversities"

"diversity"

"divert"

"diverted"

"diverter"

"diverters"

"diverticula"

"diverticulitis"

"diverticulum"

"diverting"

"diverts"

"dives"

"divest"

"divested"

"divesting"

"divestitive"

"divestiture"

"divestitures"

"divestment"

"divests"

"divesture"

"dividable"

"divide"

"divided"

"dividend"

"dividends"

"divider"

"dividers"

"divides"

"dividing"

"divination"

"divinations"

"divine"

"divined"

"divinely"

"diviner"

"diviners"

"divines"

"divinest"

"diving"

"divining"

"divinise"

"divinities"

"divinity"

"divinize"

"divisibilities"

"divisibility"

"divisible"

"divisibleness"

"divisim"

"division"

"divisional"

"divisions"

"divisive"

"divisively"

"divisiveness"

"divisor"

"divisors"

"divorce"

"divorceable"

"divorced"

"divorcee"

"divorcees"

"divorcement"

"divorcements"

"divorcer"

"divorcers"

"divorces"

"divorcing"

"divot"

"divots"

"divulge"

"divulged"

"divulgement"

"divulgence"

"divulgences"

"divulger"

"divulgers"

"divulges"

"divulging"

"divvied"

"divvies"

"divvy"

"divvying"

"dixie"

"dixieland"

"dixit"

"dizzied"

"dizzier"

"dizzies"

"dizziest"

"dizzily"

"dizziness"

"dizzy"

"dizzying"

"djakarta"

"djellaba"

"djellabas"

"djibouti"

"djin"

"djinn"

"djinni"

"djinns"

"djinny"

"djins"

"DNA"

"dnieper"

"DNR"

"do"

"do not need networking"

"do not resuscitate"

"doable"

"dobber"

"dobbin"

"dobbins"

"doberman"

"dobermans"

"dobies"

"doblas"

"dobras"

"dobson"

"doc"

"docent"

"docents"

"docile"

"docilely"

"docilities"

"docility"

"docimasia"

"docimology"

"dock"

"dockage"

"dockages"

"docked"

"docker"

"dockers"

"docket"

"docketed"

"docketing"

"dockets"

"dockhand"

"dockhands"

"docking"

"docklands"

"docks"

"dockside"

"docksides"

"dockyard"

"dockyards"

"docs"

"doctor"

"doctor appointment"

"Doctor Mom"

"doctor of the family"

"doctoral"

"doctorate"

"doctorates"

"doctored"

"doctoring"

"doctors"

"doctorship"

"doctrinaire"

"doctrinairism"

"doctrinal"

"doctrinally"

"doctrine"

"doctrines"

"docudrama"

"docudramas"

"document"

"documentable"

"documental"

"documentaries"

"documentarily"

"documentary"

"documentation"

"documented"

"documenter"

"documenters"

"documenting"

"documents"

"dodder"

"doddered"

"dodderer"

"dodderers"

"doddering"

"dodders"

"doddery"

"dodge"

"dodged"

"dodger"

"dodgers"

"dodgery"

"dodges"

"dodgier"

"dodging"

"dodgy"

"dodo"

"dodoes"

"dodoism"

"dodoisms"

"dodos"

"doe"

"doer"

"doers"

"does"

"doeskin"

"doeskins"

"doest"

"doeth"

"doff"

"doffed"

"doffer"

"doffers"

"doffing"

"doffs"

"dog"

"dogbane"

"dogbanes"

"dogberries"

"dogberry"

"dogcart"

"dogcarts"

"dogcatcher"

"dogcatchers"

"dogdom"

"doge"

"dogear"

"dogeared"

"dogears"

"doges"

"dogey"

"dogeys"

"dogface"

"dogfaces"

"dogfight"

"dogfights"

"dogfish"

"dogfishes"

"dogged"

"doggedly"

"doggedness"

"dogger"

"doggerel"

"doggerels"

"doggers"

"doggery"

"doggie"

"doggier"

"doggies"

"dogging"

"doggish"

"doggo"

"doggone"

"doggoned"

"doggoner"

"doggones"

"doggonest"

"doggoning"

"doggrel"

"doggy"

"doghouse"

"doghouses"

"dogie"

"dogies"

"dogleg"

"doglegged"

"doglegging"

"doglegs"

"dogma"

"dogmas"

"dogmata"

"dogmatic"

"dogmatical"

"dogmatically"

"dogmatism"

"dogmatist"

"dogmatists"

"dognap"

"dognaped"

"dognaper"

"dognapers"

"dognaping"

"dognapped"

"dognapping"

"dognaps"

"dogs"

"dogsbodies"

"dogsbody"

"dogsled"

"dogsleds"

"dogteeth"

"dogtooth"

"dogtrot"

"dogtrots"

"dogtrotted"

"dogwatch"

"dogwatches"

"dogwood"

"dogwoods"

"dogy"

"doilies"

"doily"

"doing"

"doings"

"dojo"

"dojos"

"dol"

"dolce"

"dolci"

"doldrums"

"dole"

"doled"

"doleful"

"dolefuller"

"dolefully"

"dolefulness"

"doles"

"dolesome"

"doling"

"doll"

"dollar"

"dollars"

"dolled"

"dollied"

"dollies"

"dolling"

"dollish"

"dollishly"

"dollop"

"dollops"

"dolls"

"dolly"

"dollying"

"dolman"

"dolmen"

"dolmens"

"dolomite"

"dolomites"

"dolor"

"dolores"

"doloroso"

"dolorous"

"dolorously"

"dolorousness"

"dolors"

"dolour"

"dolours"

"dolphin"

"dolphins"

"dolt"

"doltish"

"doltishly"

"dolts"

"dom"

"domain"

"domains"

"dome"

"domed"

"domes"

"domestic"

"domestically"

"domesticate"

"domesticated"

"domesticates"

"domesticating"

"domestication"

"domestications"

"domesticator"

"domesticities"

"domesticity"

"domestics"

"domicil"

"domicile"

"domiciled"

"domiciles"

"domiciliary"

"domiciliated"

"domiciling"

"domicils"

"dominance"

"dominant"

"dominantly"

"dominants"

"dominate"

"dominated"

"dominates"

"dominating"

"domination"

"dominations"

"dominator"

"dominators"

"domineer"

"domineered"

"domineering"

"domineers"

"domines"

"doming"

"domini"

"dominica"

"dominican"

"dominicans"

"dominick"

"dominie"

"dominion"

"dominions"

"dominium"

"domino"

"dominoes"

"dominos"

"dominus"

"doms"

"don"

"dona"

"donald"

"donate"

"donated"

"donatee"

"donates"

"donating"

"donatio"

"donation"

"donationes"

"donations"

"donative"

"donatives"

"donator"

"donators"

"done"

"donee"

"donees"

"doneness"

"dong"

"dongs"

"donjon"

"donjons"

"donkey"

"donkeys"

"donna"

"donnas"

"donne"

"donned"

"donnees"

"donning"

"donnish"

"donnybrook"

"donnybrooks"

"donor"

"donors"

"donorship"

"donovan"

"dons"

"donut"

"donuts"

"doodad"

"doodads"

"doodle"

"doodled"

"doodler"

"doodlers"

"doodles"

"doodling"

"doolies"

"doom"

"doomed"

"doomful"

"dooming"

"dooms"

"doomsday"

"doomsdays"

"doomster"

"doomsters"

"door"

"doorbell"

"doorbells"

"doorjamb"

"doorjambs"

"doorkeeper"

"doorknob"

"doorknobs"

"doorless"

"doorman"

"doormat"

"doormats"

"doormen"

"doornail"

"doornails"

"doorplate"

"doorplates"

"doorpost"

"doorposts"

"doors"

"doorsill"

"doorsills"

"doorstep"

"doorsteps"

"doorstop"

"doorstops"

"doorway"

"doorways"

"dooryard"

"dooryards"

"doozer"

"doozies"

"doozy"

"dop"

"dopant"

"dopants"

"dope"

"doped"

"doper"

"dopers"

"dopes"

"dopester"

"dopey"

"dopier"

"dopiest"

"dopiness"

"doping"

"doppler"

"dopy"

"dorado"

"doramania"

"doric"

"dories"

"doris"

"dorm"

"dormancies"

"dormancy"

"dormant"

"dormer"

"dormers"

"dormice"

"dormitories"

"dormitory"

"dormouse"

"dorms"

"dormy"

"doromania"

"dorothy"

"dorp"

"dors"

"dorsa"

"dorsal"

"dorsally"

"dorsals"

"dorsi"

"dory"

"dos"

"dosage"

"dosages"

"dose"

"dosed"

"doser"

"dosers"

"doses"

"dosimeter"

"dosimeters"

"dosimetric"

"dosimetries"

"dosimetry"

"dosing"

"dosiology"

"doss"

"dossed"

"dosser"

"dossers"

"dosses"

"dossier"

"dossiers"

"dossing"

"dost"

"dostoevsky"

"dot"

"dotage"

"dotages"

"dotard"

"dotardly"

"dotards"

"dotation"

"dote"

"doted"

"doter"

"doters"

"dotes"

"doth"

"dotier"

"dotiest"

"doting"

"dotingly"

"dots"

"dotted"

"dottels"

"dotter"

"dotters"

"dottier"

"dottiest"

"dottily"

"dotting"

"dottle"

"dottles"

"dotty"

"doty"

"double"

"doubled"

"doubleheader"

"doubleheaders"

"doubleness"

"doubler"

"doublers"

"doubles"

"doublet"

"doublethink"

"doublets"

"doublewidth"

"doubling"

"doubloon"

"doubloons"

"doubly"

"doubt"

"doubtable"

"doubted"

"doubter"

"doubters"

"doubtful"

"doubtfully"

"doubtfulness"

"doubting"

"doubtingly"

"doubtless"

"doubtlessly"

"doubts"

"douce"

"douche"

"douched"

"douches"

"douching"

"dough"

"doughboy"

"doughboys"

"doughier"

"doughiest"

"doughnut"

"doughnuts"

"doughs"

"dought"

"doughtier"

"doughtiest"

"doughtily"

"doughtiness"

"doughty"

"doughy"

"douglas"

"dour"

"dourer"

"dourest"

"dourine"

"dourly"

"dourness"

"douse"

"doused"

"douser"

"dousers"

"douses"

"dousing"

"dove"

"dovecote"

"dovecotes"

"dovecots"

"dover"

"doves"

"dovetail"

"dovetailed"

"dovetailing"

"dovetails"

"dovish"

"dowager"

"dowagers"

"dowdier"

"dowdies"

"dowdiest"

"dowdily"

"dowdiness"

"dowdy"

"dowdyish"

"dowel"

"doweled"

"doweling"

"dowelled"

"dowelling"

"dowels"

"dower"

"dowered"

"doweries"

"dowering"

"dowers"

"dowery"

"dowing"

"dowitcher"

"dowitchers"

"down"

"downbeat"

"downbeats"

"downcast"

"downcasts"

"downcourt"

"downed"

"downer"

"downers"

"downfall"

"downfallen"

"downfalls"

"downgrade"

"downgraded"

"downgrades"

"downgrading"

"downhearted"

"downheartedly"

"downhill"

"downhills"

"downier"

"downiest"

"downing"

"downlink"

"downlinked"

"downlinking"

"downlinks"

"download"

"downloadable"

"downloaded"

"downloading"

"downloads"

"downplay"

"downplayed"

"downplays"

"downpour"

"downpours"

"downrange"

"downright"

"downs"

"downshift"

"downshifted"

"downshifting"

"downshifts"

"downsize"

"downsized"

"downsizes"

"downsizing"

"downstage"

"downstairs"

"downstate"

"downstream"

"downstroke"

"downstrokes"

"downswing"

"downswings"

"downtime"

"downtimes"

"downtown"

"downtowns"

"downtrend"

"downtrends"

"downtrod"

"downtrodden"

"downturn"

"downturns"

"downward"

"downwind"

"downy"

"dowries"

"dowry"

"dows"

"dowse"

"dowsed"

"dowser"

"dowsers"

"dowses"

"dowsing"

"doxie"

"doxies"

"doxologies"

"doxology"

"doxy"

"doyen"

"doyenne"

"doyennes"

"doyens"

"doylies"

"doyly"

"doz"

"doze"

"dozed"

"dozen"

"dozened"

"dozening"

"dozens"

"dozenth"

"dozenths"

"dozer"

"dozers"

"dozes"

"dozier"

"doziest"

"dozily"

"doziness"

"dozing"

"dozy"

"dp"

"drab"

"drabbed"

"drabber"

"drabbest"

"drabbets"

"drabbing"

"drabble"

"drably"

"drabness"

"drabs"

"drachm"

"drachma"

"drachmae"

"drachmas"

"drachms"

"draconian"

"draconic"

"draft"

"draftable"

"drafted"

"draftee"

"draftees"

"drafter"

"drafters"

"draftier"

"draftiest"

"draftily"

"draftiness"

"drafting"

"draftings"

"drafts"

"draftsman"

"draftsmanship"

"draftsmen"

"drafty"

"drag"

"dragged"

"dragger"

"draggers"

"draggier"

"draggiest"

"dragging"

"draggle"

"draggled"

"draggles"

"draggling"

"draggy"

"dragline"

"draglines"

"dragnet"

"dragnets"

"dragoman"

"dragomans"

"dragomen"

"dragon"

"dragonet"

"dragonflies"

"dragonfly"

"dragonhead"

"dragons"

"dragoon"

"dragooned"

"dragooning"

"dragoons"

"dragrope"

"dragropes"

"drags"

"dragster"

"dragsters"

"drain"

"drainage"

"drainages"

"drained"

"drainer"

"drainers"

"draining"

"drainpipe"

"drainpipes"

"drains"

"drake"

"drakes"

"dram"

"drama"

"dramamine"

"dramas"

"dramatic"

"dramatically"

"dramatics"

"dramatis"

"dramatist"

"dramatists"

"dramatization"

"dramatizations"

"dramatize"

"dramatized"

"dramatizes"

"dramatizing"

"dramaturgy"

"drams"

"dramshop"

"drang&drop"

"drank"

"drapable"

"drape"

"drapeable"

"draped"

"draper"

"draperies"

"drapers"

"drapery"

"drapes"

"drapetomania"

"draping"

"drastic"

"drastically"

"drat"

"drats"

"dratted"

"dratting"

"draught"

"draughtier"

"draughting"

"draughts"

"draughty"

"drave"

"draw"

"drawable"

"drawback"

"drawbacks"

"drawbar"

"drawbars"

"drawbore"

"drawbridge"

"drawbridges"

"drawdown"

"drawer"

"drawers"

"drawing"

"drawings"

"drawl"

"drawled"

"drawler"

"drawlers"

"drawlier"

"drawling"

"drawls"

"drawly"

"drawn"

"draws"

"drawstring"

"drawstrings"

"drawtube"

"dray"

"drayage"

"drayages"

"drayed"

"draying"

"drayman"

"draymen"

"drays"

"dread"

"dreaded"

"dreadful"

"dreadfully"

"dreadfulness"

"dreadfuls"

"dreading"

"dreadnought"

"dreadnoughts"

"dreads"

"dream"

"dreamed"

"dreamer"

"dreamers"

"dreamful"

"dreamier"

"dreamiest"

"dreamily"

"dreaminess"

"dreaming"

"dreamland"

"dreamless"

"dreamlike"

"dreams"

"dreamt"

"dreamy"

"drear"

"drearier"

"drearies"

"dreariest"

"drearily"

"dreariness"

"dreary"

"dreck"

"drecks"

"dredge"

"dredged"

"dredger"

"dredgers"

"dredges"

"dredging"

"dredgings"

"dreg"

"dreggier"

"dreggiest"

"dreggish"

"dreggy"

"dregs"

"dreidel"

"dreidels"

"dreidl"

"dreidls"

"drek"

"dreks"

"drench"

"drenched"

"drencher"

"drenchers"

"drenches"

"drenching"

"dress"

"dressage"

"dressages"

"dressed"

"dresser"

"dressers"

"dresses"

"dressier"

"dressiest"

"dressily"

"dressiness"

"dressing"

"dressings"

"dressmaker"

"dressmakers"

"dressmaking"

"dressy"

"drest"

"drew"

"drib"

"dribbed"

"dribbing"

"dribble"

"dribbled"

"dribbler"

"dribblers"

"dribbles"

"dribblet"

"dribblets"

"dribbling"

"driblet"

"driblets"

"dribs"

"dried"

"drier"

"driers"

"dries"

"driest"

"drift"

"driftage"

"driftages"

"drifted"

"drifter"

"drifters"

"driftier"

"driftiest"

"drifting"

"driftpin"

"driftpins"

"drifts"

"driftway"

"driftwood"

"drifty"

"drill"

"drilled"

"driller"

"drillers"

"drilling"

"drillings"

"drillmaster"

"drillmasters"

"drills"

"drily"

"drink"

"drinkable"

"drinker"

"drinkers"

"drinking"

"drinks"

"drip"

"dripless"

"dripped"

"dripper"

"drippers"

"drippier"

"drippiest"

"dripping"

"drippings"

"drippy"

"drips"

"dript"

"drivable"

"drive"

"drivel"

"driveled"

"driveler"

"drivelers"

"driveling"

"drivelled"

"driveller"

"drivellers"

"drivelling"

"drivels"

"driven"

"driver"

"driverless"

"drivers"

"drives"

"driveway"

"driveways"

"driving"

"drizzle"

"drizzled"

"drizzles"

"drizzlier"

"drizzliest"

"drizzling"

"drizzly"

"drogue"

"drogues"

"droit"

"droits"

"droll"

"droller"

"drolleries"

"drollery"

"drollest"

"drolling"

"drollness"

"drolls"

"drolly"

"dromedaries"

"dromedary"

"dromomania"

"dromometer"

"dromophobia"

"drone"

"droned"

"droner"

"droners"

"drones"

"drongo"

"drongos"

"droning"

"dronish"

"drool"

"drooled"

"drooling"

"drools"

"droop"

"drooped"

"droopier"

"droopiest"

"droopily"

"droopiness"

"drooping"

"droops"

"droopy"

"drop"

"dropkick"

"dropkicker"

"dropkicks"

"droplet"

"droplets"

"dropout"

"dropouts"

"dropped"

"dropper"

"droppers"

"dropping"

"droppings"

"drops"

"dropshots"

"dropsical"

"dropsied"

"dropsies"

"dropsy"

"dropt"

"dropworts"

"droshky"

"drosometer"

"dross"

"drosses"

"drossier"

"drossiest"

"drossiness"

"drossy"

"drought"

"droughts"

"droughty"

"drouthy"

"drove"

"droved"

"drover"

"drovers"

"droves"

"droving"

"drown"

"drownd"

"drownded"

"drownding"

"drownds"

"drowned"

"drowner"

"drowners"

"drowning"

"drowns"

"drowse"

"drowsed"

"drowses"

"drowsier"

"drowsiest"

"drowsily"

"drowsiness"

"drowsing"

"drowsy"

"drub"

"drubbed"

"drubber"

"drubbers"

"drubbing"

"drubbings"

"drubs"

"drudge"

"drudged"

"drudger"

"drudgeries"

"drudgers"

"drudgery"

"drudges"

"drudging"

"drug"

"drug discount card"

"drug dosing"

"drug error"

"drug guide"

"Drug guide and diagnosis guide"

"drug interaction"

"drug model"

"drug model database"

"drug name"

"drug prices"

"drugged"

"drugging"

"druggist"

"druggists"

"drugmaker"

"drugs"

"drugstore"

"drugstores"

"druid"

"druidess"

"druidesses"

"druidic"

"druidism"

"druidisms"

"druids"

"drum"

"drumbeat"

"drumbeats"

"drumhead"

"drumheads"

"drumlin"

"drumlins"

"drummed"

"drummer"

"drummers"

"drumming"

"drumroll"

"drumrolls"

"drums"

"drumstick"

"drumsticks"

"drunk"

"drunkard"

"drunkards"

"drunken"

"drunkenly"

"drunkenness"

"drunker"

"drunkest"

"drunkometer"

"drunks"

"drupe"

"drupelet"

"drupelets"

"drupes"

"druthers"

"dry"

"dryable"

"dryad"

"dryades"

"dryadic"

"dryads"

"dryer"

"dryers"

"dryest"

"drying"

"drylot"

"dryly"

"dryness"

"drynesses"

"drypoint"

"drypoints"

"dryrot"

"drys"

"drywall"

"drywalls"

"duad"

"duads"

"dual"

"dualism"

"dualisms"

"dualist"

"dualistic"

"dualists"

"dualities"

"duality"

"dualize"

"dualized"

"dualizes"

"dualizing"

"dually"

"duals"

"dub"

"dubbed"

"dubber"

"dubbers"

"dubbin"

"dubbing"

"dubbings"

"dubieties"

"dubiety"

"dubio"

"dubious"

"dubiously"

"dubiousness"

"dublin"

"dubonnet"

"dubonnets"

"dubs"

"ducal"

"ducally"

"ducat"

"ducats"

"duce"

"duces"

"duchess"

"duchesses"

"duchies"

"duchy"

"duck"

"duckbill"

"duckbills"

"duckboard"

"duckboards"

"ducked"

"ducker"

"duckers"

"duckie"

"duckier"

"duckies"

"duckiest"

"ducking"

"duckling"

"ducklings"

"duckpin"

"duckpins"

"ducks"

"ducktail"

"ducktails"

"duckweed"

"duckweeds"

"ducky"

"duct"

"ductal"

"ducted"

"ductile"

"ductility"

"ducting"

"ductings"

"ductless"

"ducts"

"dud"

"duddy"

"dude"

"dudes"

"dudgeon"

"dudgeons"

"dudish"

"dudishly"

"duds"

"due"

"duel"

"dueled"

"dueler"

"duelers"

"dueling"

"duelist"

"duelists"

"duelled"

"dueller"

"duellers"

"duelling"

"duellist"

"duellists"

"duello"

"duellos"

"duels"

"duenna"

"duennas"

"dues"

"duet"

"duets"

"duetted"

"duetting"

"duettist"

"duettists"

"duff"

"duffel"

"duffels"

"duffer"

"duffers"

"duffle"

"duffles"

"duffs"

"duffy"

"dug"

"dugong"

"dugongs"

"dugout"

"dugouts"

"dugs"

"duke"

"dukedom"

"dukedoms"

"dukes"

"dulcet"

"dulcetly"

"dulcets"

"dulcify"

"dulcimer"

"dulcimers"

"dull"

"dullard"

"dullards"

"dulled"

"duller"

"dullest"

"dulling"

"dullish"

"dullness"

"dulls"

"dully"

"dulness"

"dulse"

"dulses"

"duluth"

"duly"

"dumb"

"dumbbell"

"dumbbells"

"dumbed"

"dumber"

"dumbest"

"dumbing"

"dumbly"

"dumbness"

"dumbs"

"dumbstruck"

"dumbwaiter"

"dumbwaiters"

"dumdum"

"dumdums"

"dumfound"

"dumfounded"

"dumfounding"

"dumfounds"

"dummied"

"dummies"

"dummkopf"

"dummkopfs"

"dummy"

"dummying"

"dump"

"dumpcart"

"dumpcarts"

"dumped"

"dumper"

"dumpers"

"dumpier"

"dumpiest"

"dumpily"

"dumpiness"

"dumping"

"dumpings"

"dumpish"

"dumpling"

"dumplings"

"dumps"

"dumpy"

"dun"

"dunce"

"dunces"

"dundee"

"dunderhead"

"dunderheads"

"dunderpate"

"dunderpates"

"dune"

"dunes"

"dung"

"dungaree"

"dungarees"

"dunged"

"dungeon"

"dungeons"

"dunghill"

"dunghills"

"dungier"

"dunging"

"dungs"

"dungy"

"dunk"

"dunked"

"dunker"

"dunkers"

"dunking"

"dunks"

"dunnage"

"dunnages"

"dunned"

"dunner"

"dunning"

"duns"

"duo"

"duodecimal"

"duodecimals"

"duodena"

"duodenal"

"duodenum"

"duodenums"

"duologue"

"duologues"

"duos"

"duotones"

"dup"

"dupable"

"dupe"

"duped"

"duper"

"duperies"

"dupers"

"dupery"

"dupes"

"duping"

"duple"

"duplex"

"duplexed"

"duplexer"

"duplexers"

"duplexes"

"duplexing"

"duplexs"

"duplicate"

"duplicated"

"duplicates"

"duplicating"

"duplication"

"duplications"

"duplicator"

"duplicators"

"duplicities"

"duplicitous"

"duplicity"

"dupped"

"durabilities"

"durability"

"durable"

"durableness"

"durables"

"durably"

"dural"

"durance"

"durances"

"duration"

"duration of the snooze"

"durational"

"durations"

"durative"

"duratives"

"duress"

"duresses"

"during"

"durn"

"durndest"

"durned"

"durneder"

"durnedest"

"durning"

"durns"

"durometer"

"durra"

"durrs"

"durst"

"durum"

"durums"

"dusk"

"dusked"

"duskier"

"duskiest"

"duskily"

"duskiness"

"dusking"

"duskish"

"dusks"

"dusky"

"dust"

"dustbin"

"dustbins"

"dusted"

"duster"

"dusters"

"dustheap"

"dustheaps"

"dustier"

"dustiest"

"dustily"

"dustiness"

"dusting"

"dustless"

"dustman"

"dustmen"

"dustpan"

"dustpans"

"dustrag"

"dustrags"

"dusts"

"dustup"

"dustups"

"dusty"

"dutch"

"dutchess"

"dutchman"

"dutchmen"

"duteous"

"duteously"

"dutiable"

"duties"

"dutiful"

"dutifully"

"dutifulness"

"duty"

"duumvir"

"dvorak"

"dwarf"

"dwarfed"

"dwarfer"

"dwarfest"

"dwarfing"

"dwarfish"

"dwarfism"

"dwarfisms"

"dwarflike"

"dwarfs"

"dwarves"

"dwell"

"dwelled"

"dweller"

"dwellers"

"dwelling"

"dwellings"

"dwells"

"dwelt"

"dwight"

"dwindle"

"dwindled"

"dwindles"

"dwindling"

"dx"

"dyable"

"dyad"

"dyadic"

"dyadics"

"dyads"

"dyarchy"

"dybbuk"

"dybbukim"

"dybbuks"

"dye"

"dyeable"

"dyed"

"dyeing"

"dyeings"

"dyer"

"dyers"

"dyes"

"dyestuff"

"dyestuffs"

"dyeweed"

"dyewood"

"dying"

"dyings"

"dyke"

"dykes"

"dyking"

"dynamic"

"dynamical"

"dynamically"

"dynamics"

"dynamism"

"dynamisms"

"dynamist"

"dynamistic"

"dynamists"

"dynamite"

"dynamited"

"dynamiter"

"dynamiters"

"dynamites"

"dynamiting"

"dynamo"

"dynamograph"

"dynamometer"

"dynamometers"

"dynamos"

"dynamoscope"

"dynast"

"dynastic"

"dynasties"

"dynasts"

"dynasty"

"dynatrons"

"dyne"

"dynes"

"dynode"

"dysenteric"

"dysenteries"

"dysentery"

"dysesthesia"

"dysesthetic"

"dysfunction"

"dysfunctional"

"dysfunctions"

"dysgenics"

"dyslectic"

"dyslexia"

"dyslexias"

"dyslexic"

"dyslexics"

"dysmorphophobia"

"dyspepsia"

"dyspepsy"

"dyspeptic"

"dyspeptical"

"dyspeptically"

"dyspeptics"

"dysprosium"

"dysteleology"

"dystopia"

"dystopias"

"dystrophic"

"dystrophies"

"dystrophy"

"dysuria"

"each"

"eager"

"eagerer"

"eagerest"

"eagerly"

"eagerness"

"eagers"

"eagle"

"eagles"

"eaglet"

"eaglets"

"ear"

"earache"

"earaches"

"eardrop"

"eardrops"

"eardrum"

"eardrums"

"eared"

"earflap"

"earflaps"

"earful"

"earfuls"

"earing"

"earings"

"earl"

"earlaps"

"earldom"

"earldoms"

"earless"

"earlier"

"earliest"

"earliness"

"earlobe"

"earlobes"

"earlock"

"earlocks"

"earls"

"earlship"

"earlships"

"early"

"earmark"

"earmarked"

"earmarking"

"earmarks"

"earmuff"

"earmuffs"

"earn"

"earnable"

"earned"

"earner"

"earners"

"earnest"

"earnestly"

"earnestness"

"earnests"

"earning"

"earnings"

"earns"

"earphone"

"earphones"

"earpiece"

"earpieces"

"earplug"

"earplugs"

"earring"

"earrings"

"ears"

"earshot"

"earshots"

"earsplitting"

"earth"

"earthbound"

"earthed"

"earthen"

"earthenware"

"earthier"

"earthiest"

"earthily"

"earthiness"

"earthing"

"earthlier"

"earthliest"

"earthliness"

"earthling"

"earthlings"

"earthly"

"earthman"

"earthmen"

"earthmoving"

"earthquake"

"earthquakes"

"earths"

"earthsets"

"earthshaking"

"earthward"

"earthwork"

"earthworks"

"earthworm"

"earthworms"

"earthy"

"earwax"

"earwaxes"

"earwig"

"earwigged"

"earwigging"

"earwigs"

"earworm"

"earworms"

"ease"

"eased"

"easeful"

"easel"

"easels"

"easement"

"easements"

"easer"

"easers"

"eases"

"easier"

"easies"

"easiest"

"easily"

"easiness"

"easing"

"east"

"eastbound"

"easter"

"easterlies"

"easterly"

"eastern"

"easterner"

"easterners"

"easters"

"easting"

"eastings"

"eastman"

"easts"

"eastward"

"eastwardly"

"eastwards"

"easy"

"easygoing"

"eat"

"eat healthier"

"eatable"

"eatables"

"eaten"

"eater"

"eateries"

"eaters"

"eatery"

"eating"

"eating habit"

"eating pattern"

"eatings"

"eats"

"eau"

"eaux"

"eave"

"eaved"

"eaves"

"eavesdrop"

"eavesdropped"

"eavesdropper"

"eavesdroppers"

"eavesdropping"

"eavesdrops"

"ebb"

"ebbed"

"ebbing"

"ebbs"

"ebcdic"

"ebon"

"ebonies"

"ebonite"

"ebonites"

"ebonizing"

"ebons"

"ebony"

"ebullience"

"ebullient"

"ebulliently"

"ebullioscope"

"ebullition"

"ebullitions"

"ECC"

"eccentric"

"eccentrically"

"eccentricities"

"eccentricity"

"eccentrics"

"eccl"

"ecclesia"

"ecclesiastes"

"ecclesiastic"

"ecclesiastical"

"ecclesiastically"

"ecclesiasticalness"

"ecclesiastics"

"ecclesiology"

"eccrinology"

"ecdemomania"

"ecdysial"

"ecdysis"

"ECG"

"ECG abnormality"

"echelon"

"echeloned"

"echeloning"

"echelons"

"echidna"

"echidnae"

"echidnas"

"echinodermata"

"echo"

"echoed"

"echoer"

"echoers"

"echoes"

"echoey"

"echoic"

"echoing"

"echoism"

"echoisms"

"echolalia"

"echoless"

"echolocation"

"eclair"

"eclairs"

"eclampsia"

"eclamptic"

"eclat"

"eclats"

"eclectic"

"eclectically"

"eclecticism"

"eclectics"

"eclipse"

"eclipsed"

"eclipses"

"eclipsing"

"ecliptic"

"ecliptics"

"eclogue"

"eclogues"

"ecocide"

"ecol"

"ecole"

"ecoles"

"ecologic"

"ecological"

"ecologically"

"ecologies"

"ecologist"

"ecologists"

"ecology"

"econ"

"economic"

"economical"

"economically"

"economics"

"economies"

"economist"

"economists"

"economize"

"economized"

"economizer"

"economizers"

"economizes"

"economizing"

"economy"

"ecophobia"

"ecosystem"

"ecosystems"

"ecotype"

"ecotypes"

"ecotypic"

"ecru"

"ecrus"

"ecstasies"

"ecstasy"

"ecstatic"

"ecstatically"

"ecstatics"

"ectoderm"

"ectomorph"

"ectopic"

"ectoplasm"

"ectoplasmatic"

"ectoplasmic"

"ecuador"

"ecumenic"

"ecumenical"

"ecumenicalism"

"ecumenically"

"ecumenicism"

"ecumenicity"

"ecumenism"

"ecus"

"eczema"

"eczemas"

"eczematous"

"edam"

"edaphology"

"EDC"

"edda"

"eddied"

"eddies"

"eddy"

"eddying"

"edelweiss"

"edelweisses"

"edema"

"edemas"

"edemata"

"edematous"

"eden"

"edentates"

"edgar"

"edge"

"edged"

"edgeless"

"edger"

"edgers"

"edges"

"edgeways"

"edgewise"

"edgier"

"edgiest"

"edgily"

"edginess"

"edging"

"edgings"

"edgy"

"edibility"

"edible"

"edibleness"

"edibles"

"edict"

"edictally"

"edicts"

"edification"

"edifice"

"edifices"

"edified"

"edifier"

"edifiers"

"edifies"

"edify"

"edifying"

"edinburgh"

"edison"

"edit"

"editable"

"edited"

"edith"

"editing"

"edition"

"editions"

"editor"

"editorial"

"editorialist"

"editorialization"

"editorializations"

"editorialize"

"editorialized"

"editorializer"

"editorializers"

"editorializes"

"editorializing"

"editorially"

"editorials"

"editors"

"editorship"

"editorships"

"editress"

"editresses"

"edits"

"educability"

"educable"

"educate"

"educated"

"educates"

"educating"

"education"

"education tool"

"educational"

"educational purpose"

"Educational videos"

"educationally"

"educations"

"educative"

"educator"

"educators"

"educe"

"educed"

"educes"

"educing"

"educt"

"eduction"

"eductions"

"eductive"

"eductor"

"eductors"

"educts"

"edward"

"edwards"

"eel"

"eelgrass"

"eelgrasses"

"eelier"

"eeliest"

"eels"

"eelworm"

"eely"

"eerie"

"eerier"

"eeriest"

"eerily"

"eeriness"

"eery"

"effable"

"efface"

"effaceable"

"effaced"

"effacement"

"effacer"

"effacers"

"effaces"

"effacing"

"effect"

"effected"

"effecter"

"effecters"

"effecting"

"effective"

"effective contraception"

"effectively"

"effectiveness"

"effector"

"effectors"

"effects"

"effectual"

"effectuality"

"effectually"

"effectuate"

"effectuated"

"effectuates"

"effectuating"

"effectuation"

"effeminacy"

"effeminate"

"effeminately"

"effemination"

"effendi"

"effendis"

"efferent"

"efferents"

"effervesce"

"effervesced"

"effervescence"

"effervescent"

"effervescently"

"effervesces"

"effervescing"

"effete"

"effetely"

"effeteness"

"efficacies"

"efficacious"

"efficaciously"

"efficacy"

"efficiencies"

"efficiency"

"efficient"

"efficiently"

"effigies"

"effigy"

"effloresce"

"effloresced"

"efflorescence"

"efflorescent"

"effloresces"

"efflorescing"

"effluence"

"effluences"

"effluent"

"effluents"

"effluvia"

"effluvial"

"effluvias"

"effluvium"

"effluviums"

"efflux"

"effluxes"

"effort"

"effortless"

"effortlessly"

"effortlessness"

"efforts"

"effronteries"

"effrontery"

"effs"

"effulge"

"effulged"

"effulgence"

"effulgences"

"effulgent"

"effulgently"

"effulges"

"effulging"

"effuse"

"effused"

"effuses"

"effusing"

"effusiometer"

"effusion"

"effusions"

"effusive"

"effusively"

"effusiveness"

"eft"

"efts"

"eftsoon"

"eftsoons"

"egad"

"egads"

"egalitarian"

"egalitarianism"

"egalitarians"

"egalite"

"egalites"

"egestions"

"egg"

"egg diameter"

"eggbeater"

"eggbeaters"

"eggcup"

"eggcups"

"egged"

"egger"

"eggers"

"egghead"

"eggheads"

"egging"

"eggnog"

"eggnogs"

"eggplant"

"eggplants"

"eggs"

"eggshell"

"eggshells"

"egis"

"egises"

"eglantine"

"eglantines"

"ego"

"egocentric"

"egocentricities"

"egocentricity"

"egocentrism"

"egoism"

"egoisms"

"egoist"

"egoistic"

"egoistical"

"egoistically"

"egoists"

"egomania"

"egomaniac"

"egomaniacal"

"egomaniacally"

"egomanias"

"egos"

"egotism"

"egotisms"

"egotist"

"egotistic"

"egotistical"

"egotistically"

"egotists"

"egregious"

"egregiously"

"egregiousness"

"egress"

"egressed"

"egresses"

"egressing"

"egret"

"egrets"

"egypt"

"egyptian"

"egyptians"

"Egyptology"

"eh"

"eHealth"

"ehr"

"eider"

"eiderdown"

"eiders"

"eidetic"

"eidograph"

"eidola"

"eidolon"

"eidolons"

"eidos"

"eiffel"

"eight"

"eightball"

"eightballs"

"eighteen"

"eighteens"

"eighteenth"

"eighteenths"

"eighth"

"eighthly"

"eighths"

"eighties"

"eightieth"

"eightieths"

"eights"

"eighty"

"eikon"

"einstein"

"einsteinium"

"eire"

"eisenhower"

"eisteddfod"

"eisteddfods"

"either"

"ejacula"

"ejaculate"

"ejaculated"

"ejaculates"

"ejaculating"

"ejaculation"

"ejaculations"

"ejaculator"

"ejaculators"

"ejaculatory"

"ejaculum"

"eject"

"ejecta"

"ejectable"

"ejected"

"ejecting"

"ejection"

"ejections"

"ejective"

"ejectives"

"ejectment"

"ejector"

"ejectors"

"ejects"

"ejectum"

"eke"

"eked"

"ekes"

"EKG"

"eking"

"ekistic"

"ekistics"

"el"

"elaborate"

"elaborated"

"elaborately"

"elaborateness"

"elaborates"

"elaborating"

"elaboration"

"elaborations"

"elaborator"

"elaborators"

"elaine"

"elan"

"eland"

"elands"

"elans"

"elapse"

"elapsed"

"elapses"

"elapsing"

"elastic"

"elastically"

"elasticities"

"elasticity"

"elasticize"

"elasticized"

"elasticizes"

"elasticizing"

"elastics"

"elasticum"

"elastin"

"elastins"

"elastomer"

"elastomeric"

"elastomers"

"elate"

"elated"

"elatedly"

"elater"

"elaters"

"elates"

"elating"

"elation"

"elations"

"elative"

"elatives"

"elatrometer"

"elbow"

"elbowed"

"elbowing"

"elbowroom"

"elbows"

"eld"

"elder"

"elderberries"

"elderberry"

"elderly"

"elders"

"eldest"

"eldrich"

"eldritch"

"elds"

"eleanor"

"elect"

"elected"

"electee"

"electees"

"electing"

"election"

"electioneer"

"electioneered"

"electioneering"

"electioneers"

"elections"

"elective"

"electively"

"electives"

"elector"

"electoral"

"electorally"

"electorate"

"electorates"

"electorial"

"electors"

"electra"

"electrets"

"electric"

"electrical"

"electrically"

"electrician"

"electricians"

"electricity"

"electrics"

"electrification"

"electrified"

"electrifier"

"electrifiers"

"electrifies"

"electrify"

"electrifying"

"electro"

"electrocardiogram"

"electrocardiograms"

"electrocardiograph"

"electrocardiographic"

"electrocardiographs"

"electrocardiography"

"electrochemical"

"electrochemically"

"electrochemistry"

"electrocute"

"electrocuted"

"electrocutes"

"electrocuting"

"electrocution"

"electrocutional"

"electrocutions"

"electrode"

"electrodes"

"electrodynamic"

"electrodynamics"

"electrodynamometer"

"electroencephalogram"

"electroencephalograms"

"electroencephalograph"

"electroencephalographic"

"electroencephalographs"

"electroencephalography"

"Electroencephalography theory"

"electrogram"

"electrograph"

"electrologist"

"electrologists"

"electrology"

"electrolyses"

"electrolysis"

"electrolyte"

"electrolyte component"

"electrolytes"

"electrolytic"

"electrolytically"

"electrolyze"

"electrolyzed"

"electrolyzing"

"electromagnet"

"electromagnetic"

"electromagnetical"

"electromagnetically"

"electromagnetism"

"electromagnets"

"electrometer"

"electromotive"

"electromyograph"

"electron"

"electronarcosis"

"electronic"

"electronic health record"

"electronic medical record"

"electronically"

"electronics"

"electrons"

"electrophorese"

"electrophoresed"

"electrophoreses"

"electrophoresing"

"electrophoresis"

"electrophoretic"

"electroplate"

"electroplated"

"electroplates"

"electroplating"

"electropositive"

"electroretinograph"

"electroscope"

"electroscopes"

"electroshock"

"electroshocks"

"electrostatic"

"electrostatics"

"electrosurgeries"

"electrosurgery"

"electrosurgically"

"electrotherapies"

"electrotheraputic"

"electrotheraputical"

"electrotheraputically"

"electrotheraputics"

"electrotherapy"

"electrotype"

"electrotypes"

"electrum"

"electrums"

"elects"

"electuary"

"eleemosynary"

"elegance"

"elegances"

"elegancies"

"elegancy"

"elegant"

"eleganter"

"elegantly"

"elegiac"

"elegiacs"

"elegies"

"elegise"

"elegised"

"elegises"

"elegist"

"elegists"

"elegize"

"elegized"

"elegizes"

"elegizing"

"elegy"

"element"

"elemental"

"elementally"

"elementals"

"elementarily"

"elementariness"

"elementary"

"elements"

"elephant"

"elephantiases"

"elephantiasis"

"elephantine"

"elephants"

"eleutheromania"

"eleutherophobia"

"elevate"

"elevated"

"elevates"

"elevating"

"elevation"

"elevations"

"elevator"

"elevators"

"eleven"

"elevens"

"eleventh"

"elevenths"

"elevon"

"elevons"

"elf"

"elfin"

"elfins"

"elfish"

"elfishly"

"elfishness"

"elflock"

"elflocks"

"elhi"

"elicit"

"elicitation"

"elicited"

"eliciting"

"elicitor"

"elicitors"

"elicits"

"elide"

"elided"

"elides"

"elidible"

"eliding"

"eligibility"

"eligible"

"eligibles"

"eligibly"

"elijah"

"eliminant"

"eliminate"

"eliminated"

"eliminates"

"eliminating"

"elimination"

"eliminations"

"eliminative"

"eliminator"

"eliminators"

"eliminatory"

"elision"

"elisions"

"elite"

"elites"

"elitism"

"elitisms"

"elitist"

"elitists"

"elixir"

"elixirs"

"elizabeth"

"elizabethan"

"elizabethans"

"elk"

"elkhound"

"elkhounds"

"elks"

"ell"

"ellen"

"ellipse"

"ellipses"

"ellipsis"

"ellipsograph"

"ellipsoid"

"ellipsoidal"

"ellipsoids"

"elliptic"

"elliptical"

"elliptically"

"ells"

"elm"

"elmier"

"elmiest"

"elms"

"elmy"

"elocution"

"elocutionist"

"elocutionists"

"elongate"

"elongated"

"elongates"

"elongating"

"elongation"

"elongations"

"elope"

"eloped"

"elopement"

"elopements"

"eloper"

"elopers"

"elopes"

"eloping"

"eloquence"

"eloquent"

"eloquently"

"else"

"elses"

"elsewhere"

"elucidate"

"elucidated"

"elucidates"

"elucidating"

"elucidation"

"elucidations"

"elucidator"

"elucidators"

"elude"

"eluded"

"eluder"

"eluders"

"eludes"

"eluding"

"elusion"

"elusive"

"elusively"

"elusiveness"

"elusory"

"eluviating"

"elver"

"elvers"

"elves"

"elvis"

"elvish"

"elvishly"

"elysian"

"elysium"

"em"

"emaciate"

"emaciated"

"emaciates"

"emaciating"

"emaciation"

"email"

"emanate"

"emanated"

"emanates"

"emanating"

"emanation"

"emanations"

"emanative"

"emanator"

"emanators"

"emancipate"

"emancipated"

"emancipates"

"emancipating"

"emancipation"

"emancipations"

"emancipator"

"emancipators"

"emasculate"

"emasculated"

"emasculates"

"emasculating"

"emasculation"

"emasculations"

"emasculator"

"emasculators"

"embalm"

"embalmed"

"embalmer"

"embalmers"

"embalming"

"embalms"

"embank"

"embanked"

"embanking"

"embankment"

"embankments"

"embanks"

"embar"

"embargo"

"embargoed"

"embargoes"

"embargoing"

"embark"

"embarkation"

"embarkations"

"embarked"

"embarking"

"embarkment"

"embarks"

"embarrass"

"embarrassed"

"embarrassedly"

"embarrasses"

"embarrassing"

"embarrassingly"

"embarrassment"

"embarrassments"

"embarred"

"embarring"

"embars"

"embassador"

"embassadress"

"embassies"

"embassy"

"embattle"

"embattled"

"embattles"

"embattling"

"embay"

"embays"

"embed"

"embedded"

"embedding"

"embeds"

"embellish"

"embellished"

"embellisher"

"embellishers"

"embellishes"

"embellishing"

"embellishment"

"embellishments"

"ember"

"embers"

"embezzle"

"embezzled"

"embezzlement"

"embezzlements"

"embezzler"

"embezzlers"

"embezzles"

"embezzling"

"embitter"

"embittered"

"embittering"

"embitterment"

"embitterments"

"embitters"

"emblaze"

"emblazers"

"emblazing"

"emblazon"

"emblazoned"

"emblazoning"

"emblazonment"

"emblazonments"

"emblazons"

"emblem"

"emblematic"

"emblematical"

"emblements"

"embleming"

"emblems"

"embodied"

"embodier"

"embodiers"

"embodies"

"embodiment"

"embodiments"

"embody"

"embodying"

"embolden"

"emboldened"

"emboldening"

"emboldens"

"emboli"

"embolic"

"embolism"

"embolisms"

"embolization"

"embolus"

"embonpoint"

"embordered"

"emborders"

"embosomed"

"embosoming"

"embosoms"

"emboss"

"embossed"

"embosser"

"embossers"

"embosses"

"embossing"

"embossment"

"embossments"

"embouchure"

"embouchures"

"embow"

"emboweled"

"emboweling"

"embowelled"

"embower"

"embowered"

"embowering"

"embowers"

"embows"

"embrace"

"embraceable"

"embraced"

"embracer"

"embracers"

"embraces"

"embracing"

"embrasure"

"embrasures"

"embrocate"

"embrocated"

"embrocates"

"embrocating"

"embrocation"

"embrocations"

"embroglios"

"embroider"

"embroidered"

"embroiderer"

"embroiderers"

"embroideries"

"embroidering"

"embroiders"

"embroidery"

"embroil"

"embroiled"

"embroiling"

"embroilment"

"embroilments"

"embroils"

"embryo"

"embryogenic"

"embryoid"

"embryologic"

"embryological"

"embryologically"

"embryologies"

"embryologist"

"embryologists"

"embryology"

"embryonic"

"embryos"

"emcee"

"emceed"

"emceeing"

"emcees"

"emeer"

"emeerate"

"emeers"

"emend"

"emendable"

"emendating"

"emendation"

"emendations"

"emended"

"emender"

"emenders"

"emending"

"emends"

"emerald"

"emeralds"

"emerge"

"emerged"

"emergence"

"emergences"

"emergencies"

"emergency"

"emergency call"

"emergency care"

"emergency contact"

"emergency medical practioners"

"emergency medical records"

"Emergency Medical Services"

"Emergency Medicine"

"emergency numbers"

"emergency plan"

"Emergency Responders"

"emergency room"

"emergency situation"

"emergency system"

"emergent"

"emergents"

"emerges"

"emerging"

"emeries"

"emerita"

"emeriti"

"emeritus"

"emersion"

"emersions"

"emerson"

"emery"

"emetic"

"emetically"

"emetics"

"emetology"

"emf"

"emigrant"

"emigrants"

"emigrate"

"emigrated"

"emigrates"

"emigrating"

"emigration"

"emigrational"

"emigrations"

"emigre"

"emigres"

"emily"

"eminence"

"eminences"

"eminencies"

"eminency"

"eminent"

"eminently"

"emir"

"emirate"

"emirates"

"emirs"

"emissaries"

"emissary"

"emission"

"emissions"

"emissive"

"emissivity"

"emit"

"emits"

"emitted"

"emitter"

"emitters"

"emitting"

"emmenology"

"emmet"

"emmets"

"emmies"

"emmy"

"emocrat"

"emollient"

"emollients"

"emolument"

"emoluments"

"emote"

"emoted"

"emoter"

"emoters"

"emotes"

"emoting"

"emotion"

"emotional"

"emotionalism"

"emotionalist"

"emotionalistic"

"emotionality"

"emotionalize"

"emotionally"

"emotionless"

"emotionlessness"

"emotions"

"emotive"

"empalers"

"empaling"

"empanel"

"empaneled"

"empaneling"

"empanelled"

"empanels"

"empathetic"

"empathic"

"empathies"

"empathize"

"empathized"

"empathizes"

"empathizing"

"empathy"

"empennage"

"empennages"

"emperor"

"emperors"

"emphases"

"emphasis"

"emphasize"

"emphasized"

"emphasizes"

"emphasizing"

"emphatic"

"emphatically"

"emphysema"

"empire"

"empires"

"empiric"

"empirical"

"empirically"

"empiricism"

"empiricist"

"empiricists"

"empirics"

"emplace"

"emplaced"

"emplacement"

"emplacements"

"emplaces"

"emplacing"

"emplane"

"emplaning"

"empleomania"

"employ"

"employability"

"employable"

"employed"

"employee"

"employees"

"employer"

"employers"

"employing"

"employment"

"employments"

"employs"

"empoisoned"

"emporia"

"emporium"

"emporiums"

"empower"

"empowered"

"empowering"

"empowerment"

"empowers"

"empress"

"empresses"

"emptied"

"emptier"

"emptiers"

"empties"

"emptiest"

"emptily"

"emptiness"

"emptings"

"emptive"

"emptor"

"empty"

"emptying"

"empurple"

"empurpled"

"empurples"

"empurpling"

"empyreal"

"empyrean"

"empyreans"

"emr"

"ems"

"EMS personnel"

"emu"

"emulate"

"emulated"

"emulates"

"emulating"

"emulation"

"emulations"

"emulative"

"emulatively"

"emulator"

"emulators"

"emulous"

"emulsible"

"emulsifiable"

"emulsification"

"emulsifications"

"emulsified"

"emulsifier"

"emulsifiers"

"emulsifies"

"emulsify"

"emulsifying"

"emulsin"

"emulsion"

"emulsions"

"emulsive"

"emulsoid"

"emulsoids"

"emus"

"en"

"enable"

"enabled"

"enabler"

"enablers"

"enables"

"enabling"

"enact"

"enacted"

"enacting"

"enactive"

"enactment"

"enactments"

"enactor"

"enactors"

"enacts"

"enamel"

"enameled"

"enameler"

"enamelers"

"enameling"

"enamelled"

"enameller"

"enamellers"

"enamelling"

"enamels"

"enamelware"

"enamelwork"

"enamor"

"enamored"

"enamoring"

"enamors"

"enamour"

"enamoured"

"enamouring"

"enamours"

"enarthrodial"

"enate"

"enates"

"enatic"

"enc"

"encage"

"encaged"

"encages"

"encaging"

"encamp"

"encamped"

"encamping"

"encampment"

"encampments"

"encamps"

"encapsulate"

"encapsulated"

"encapsulates"

"encapsulating"

"encapsulation"

"encapsulations"

"encapsule"

"encapsuled"

"encapsules"

"encapsuling"

"encase"

"encased"

"encasement"

"encases"

"encasing"

"enceinte"

"encephala"

"encephalic"

"encephalitic"

"encephalitis"

"encephalogram"

"encephalograph"

"encephalographic"

"encephalography"

"encephalomyelitis"

"encephalon"

"enchain"

"enchained"

"enchaining"

"enchainment"

"enchainments"

"enchains"

"enchant"

"enchanted"

"enchanter"

"enchanters"

"enchanting"

"enchantingly"

"enchantment"

"enchantments"

"enchantress"

"enchantresses"

"enchants"

"enchilada"

"enchiladas"

"encina"

"encipher"

"enciphered"

"enciphering"

"encipherment"

"encipherments"

"enciphers"

"encircle"

"encircled"

"encirclement"

"encirclements"

"encircles"

"encircling"

"encl"

"enclasp"

"enclasping"

"enclave"

"enclaves"

"enclosable"

"enclose"

"enclosed"

"encloser"

"enclosers"

"encloses"

"enclosing"

"enclosure"

"enclosures"

"encode"

"encoded"

"encoder"

"encoders"

"encodes"

"encoding"

"encodings"

"encomia"

"encomium"

"encomiums"

"encompass"

"encompassed"

"encompasses"

"encompassing"

"encompassment"

"encore"

"encored"

"encores"

"encoring"

"encounter"

"encountered"

"encounterer"

"encounterers"

"encountering"

"encounters"

"encourage"

"encouraged"

"encouragement"

"encouragements"

"encourager"

"encouragers"

"encourages"

"encouraging"

"encouragingly"

"encroach"

"encroached"

"encroaches"

"encroaching"

"encroachment"

"encroachments"

"encrust"

"encrustation"

"encrusted"

"encrusting"

"encrypt"

"encrypted"

"encrypting"

"encryption"

"encryptions"

"encrypts"

"encumber"

"encumbered"

"encumbering"

"encumbers"

"encumbrance"

"encumbrancer"

"encumbrances"

"encyclic"

"encyclical"

"encyclicals"

"encyclics"

"encyclopedia"

"encyclopedias"

"encyclopedic"

"encyclopedically"

"encyst"

"encysted"

"encysting"

"encystment"

"encystments"

"encysts"

"end"

"endamaged"

"endamages"

"endamaging"

"endanger"

"endangered"

"endangering"

"endangerment"

"endangerments"

"endangers"

"endbrain"

"endbrains"

"endear"

"endeared"

"endearing"

"endearingly"

"endearment"

"endearments"

"endears"

"endeavor"

"endeavored"

"endeavoring"

"endeavors"

"endeavour"

"endeavoured"

"endeavouring"

"ended"

"endemic"

"endemics"

"endemiology"

"ender"

"endermic"

"enders"

"ending"

"ending position"

"endings"

"enditing"

"endive"

"endives"

"endleaf"

"endleaves"

"endless"

"endlessly"

"endlessness"

"endlong"

"endmost"

"endnote"

"endnotes"

"endocrine"

"Endocrine System"

"endocrinic"

"endocrinologic"

"endocrinological"

"endocrinologies"

"endocrinologist"

"endocrinologists"

"endocrinology"

"endocrinous"

"endoderms"

"endogamy"

"endogenous"

"endogenously"

"endogeny"

"Endomondo"

"endomorph"

"endomorphic"

"endomorphism"

"endorsable"

"endorse"

"endorsed"

"endorsee"

"endorsees"

"endorsement"

"endorsements"

"endorser"

"endorsers"

"endorses"

"endorsing"

"endorsor"

"endoscope"

"endoscopes"

"endoscopic"

"endoscopies"

"endoscopy"

"endoskeleton"

"endosmometer"

"endothermal"

"endothermic"

"endow"

"endowed"

"endower"

"endowers"

"endowing"

"endowment"

"endowments"

"endows"

"endozoic"

"endpaper"

"endpapers"

"endplate"

"endplates"

"endpoint"

"endpoints"

"endrin"

"ends"

"endue"

"endued"

"endues"

"enduing"

"endurable"

"endurance"

"endure"

"endured"

"endures"

"enduring"

"enduro"

"enduros"

"endways"

"endwise"

"enema"

"enemas"

"enemies"

"enemy"

"energetic"

"energetically"

"energetics"

"energies"

"energise"

"energize"

"energized"

"energizer"

"energizers"

"energizes"

"energizing"

"energy"

"energy levels"

"enervate"

"enervated"

"enervates"

"enervating"

"enervation"

"enervator"

"enervators"

"enface"

"enfant"

"enfants"

"enfeeble"

"enfeebled"

"enfeeblement"

"enfeeblements"

"enfeebles"

"enfeebling"

"enfeoffed"

"enfeoffing"

"enfeoffment"

"enfetter"

"enfettered"

"enfetters"

"enfever"

"enfevered"

"enfevering"

"enfevers"

"enfilade"

"enfiladed"

"enfilades"

"enfilading"

"enfin"

"enflame"

"enflamed"

"enflames"

"enflaming"

"enfold"

"enfolded"

"enfolder"

"enfolders"

"enfolding"

"enfoldings"

"enfolds"

"enforce"

"enforceability"

"enforceable"

"enforced"

"enforcement"

"enforcer"

"enforcers"

"enforces"

"enforcing"

"enframe"

"enframed"

"enframes"

"enframing"

"enfranchise"

"enfranchised"

"enfranchisement"

"enfranchisements"

"enfranchises"

"enfranchising"

"engage"

"engaged"

"engagement"

"engagements"

"engager"

"engagers"

"engages"

"engaging"

"engagingly"

"engender"

"engendered"

"engendering"

"engenders"

"engild"

"engilding"

"engilds"

"engine"

"engined"

"engineer"

"engineered"

"engineering"

"engineers"

"engineless"

"engineries"

"enginery"

"engines"

"engining"

"engird"

"engirded"

"engirding"

"engirdle"

"engirdled"

"engirdles"

"engirdling"

"engirds"

"engirt"

"england"

"englander"

"englanders"

"english"

"englished"

"englishes"

"englishing"

"englishman"

"englishmen"

"englishwoman"

"englishwomen"

"englobe"

"englobed"

"englobement"

"englobing"

"englutting"

"engorge"

"engorged"

"engorgement"

"engorges"

"engorging"

"engr"

"engraft"

"engrafted"

"engrafting"

"engrafts"

"engrailed"

"engrailing"

"engrained"

"engraining"

"engram"

"engramme"

"engrammes"

"engrams"

"engrave"

"engraved"

"engraver"

"engravers"

"engraves"

"engraving"

"engravings"

"engross"

"engrossed"

"engrosser"

"engrossers"

"engrosses"

"engrossing"

"engrossment"

"engulf"

"engulfed"

"engulfing"

"engulfment"

"engulfs"

"enhaloed"

"enhaloes"

"enhaloing"

"enhance"

"enhanced"

"enhancement"

"enhancements"

"enhancer"

"enhancers"

"enhances"

"enhancing"

"enigma"

"enigmas"

"enigmata"

"enigmatic"

"enigmatical"

"enigmatically"

"enigmatology"

"enjambment"

"enjambments"

"enjoin"

"enjoinder"

"enjoinders"

"enjoined"

"enjoiner"

"enjoiners"

"enjoining"

"enjoins"

"enjoy"

"enjoyable"

"enjoyably"

"enjoyed"

"enjoyer"

"enjoyers"

"enjoying"

"enjoyment"

"enjoyments"

"enjoys"

"enkindle"

"enkindled"

"enkindles"

"enkindling"

"enlace"

"enlacing"

"enlarge"

"enlarged"

"enlargement"

"enlargements"

"enlarger"

"enlargers"

"enlarges"

"enlarging"

"enlighten"

"enlightened"

"enlightener"

"enlighteners"

"enlightening"

"enlightenment"

"enlightenments"

"enlightens"

"enlist"

"enlisted"

"enlistee"

"enlistees"

"enlister"

"enlisters"

"enlisting"

"enlistment"

"enlistments"

"enlists"

"enliven"

"enlivened"

"enlivening"

"enlivenment"

"enlivenments"

"enlivens"

"enmesh"

"enmeshed"

"enmeshes"

"enmeshing"

"enmeshment"

"enmeshments"

"enmities"

"enmity"

"ennead"

"enneads"

"enneagons"

"ennoble"

"ennobled"

"ennoblement"

"ennoblements"

"ennobler"

"ennoblers"

"ennobles"

"ennobling"

"ennui"

"ennuis"

"enormities"

"enormity"

"enormous"

"enormously"

"enormousness"

"enosimania"

"enough"

"enoughs"

"enounced"

"enounces"

"enouncing"

"enow"

"enplane"

"enplaned"

"enplanes"

"enplaning"

"enqueue"

"enquire"

"enquired"

"enquirer"

"enquires"

"enquiries"

"enquiring"

"enquiry"

"enrage"

"enraged"

"enrages"

"enraging"

"enrapt"

"enrapture"

"enraptured"

"enraptures"

"enrapturing"

"enravish"

"enravished"

"enravishes"

"enrich"

"enriched"

"enricher"

"enrichers"

"enriches"

"enriching"

"enrichment"

"enrichments"

"enrobe"

"enrobed"

"enrober"

"enrobers"

"enrobes"

"enrobing"

"enrol"

"enroll"

"enrolled"

"enrollee"

"enrollees"

"enroller"

"enrollers"

"enrolling"

"enrollment"

"enrollments"

"enrolls"

"enrolment"

"enrols"

"enroot"

"ens"

"ensamples"

"ensconce"

"ensconced"

"ensconces"

"ensconcing"

"enscrolled"

"enscrolls"

"ensemble"

"ensembles"

"enserfing"

"ensheathe"

"ensheathed"

"ensheathes"

"ensheathing"

"ensheaths"

"enshrine"

"enshrined"

"enshrinement"

"enshrinements"

"enshrines"

"enshrining"

"enshroud"

"enshrouded"

"enshrouding"

"enshrouds"

"ensign"

"ensigncy"

"ensigns"

"ensilage"

"ensilaged"

"ensilages"

"ensilaging"

"ensile"

"ensiled"

"ensiles"

"ensiling"

"ensky"

"enskying"

"enslave"

"enslaved"

"enslavement"

"enslavements"

"enslaver"

"enslavers"

"enslaves"

"enslaving"

"ensnare"

"ensnared"

"ensnarement"

"ensnarements"

"ensnarer"

"ensnarers"

"ensnares"

"ensnaring"

"ensnarl"

"ensnarled"

"ensnarling"

"ensnarls"

"ensorcel"

"ensorceled"

"ensorcels"

"ensoul"

"ensouling"

"ensphered"

"enspheres"

"ensuant"

"ensue"

"ensued"

"ensues"

"ensuing"

"ensure"

"ensured"

"ensurer"

"ensurers"

"ensures"

"ensuring"

"enswathed"

"enswathes"

"entail"

"entailed"

"entailer"

"entailers"

"entailing"

"entailment"

"entailments"

"entails"

"entangle"

"entangled"

"entanglement"

"entanglements"

"entangler"

"entanglers"

"entangles"

"entangling"

"entendre"

"entendres"

"entente"

"ententes"

"enter"

"enterable"

"entered"

"enterer"

"enterers"

"entering"

"enteritis"

"enterprise"

"enterpriser"

"enterprises"

"enterprising"

"enterprisingly"

"enterprize"

"enters"

"entertain"

"entertained"

"entertainer"

"entertainers"

"entertaining"

"entertainingly"

"entertainment"

"entertainments"

"entertains"

"entheomania"

"enthrall"

"enthralled"

"enthralling"

"enthrallingly"

"enthrallment"

"enthrallments"

"enthralls"

"enthrone"

"enthroned"

"enthronement"

"enthronements"

"enthrones"

"enthroning"

"enthuse"

"enthused"

"enthuses"

"enthusiasm"

"enthusiasms"

"enthusiast"

"enthusiastic"

"enthusiastically"

"enthusiasts"

"enthusing"

"entice"

"enticed"

"enticement"

"enticements"

"enticer"

"enticers"

"entices"

"enticing"

"entire"

"entirely"

"entireness"

"entires"

"entireties"

"entirety"

"entities"

"entitle"

"entitled"

"entitlement"

"entitles"

"entitling"

"entity"

"entoiled"

"entoiling"

"entoils"

"entomb"

"entombed"

"entombing"

"entombment"

"entombments"

"entombs"

"entomological"

"entomologically"

"entomologies"

"entomologist"

"entomologists"

"entomology"

"entourage"

"entourages"

"entozoology"

"entrails"

"entrain"

"entrained"

"entraining"

"entrains"

"entrance"

"entranced"

"entrancement"

"entrancements"

"entrances"

"entrancing"

"entrancingly"

"entrant"

"entrants"

"entrap"

"entrapment"

"entrapments"

"entrapped"

"entrapping"

"entraps"

"entre"

"entreat"

"entreated"

"entreaties"

"entreating"

"entreatingly"

"entreats"

"entreaty"

"entree"

"entrees"

"entrench"

"entrenched"

"entrenches"

"entrenching"

"entrenchment"

"entrenchments"

"entrepreneur"

"entrepreneurial"

"entrepreneurs"

"entrepreneurship"

"entries"

"entropies"

"entropy"

"entrust"

"entrusted"

"entrusting"

"entrustment"

"entrusts"

"entry"

"entryway"

"entryways"

"entwine"

"entwined"

"entwines"

"entwining"

"entwist"

"entwisted"

"entwisting"

"entwists"

"enumerable"

"enumerate"

"enumerated"

"enumerates"

"enumerating"

"enumeration"

"enumerations"

"enumerator"

"enumerators"

"enunciate"

"enunciated"

"enunciates"

"enunciating"

"enunciation"

"enunciations"

"enunciator"

"enunciators"

"enure"

"enureses"

"enuresis"

"enuretic"

"envelop"

"envelope"

"enveloped"

"enveloper"

"envelopers"

"envelopes"

"enveloping"

"envelopment"

"envelopments"

"envelops"

"envenom"

"envenomation"

"envenomed"

"envenoming"

"envenomization"

"envenoms"

"enviable"

"enviably"

"envied"

"envier"

"enviers"

"envies"

"envious"

"enviously"

"enviousness"

"environ"

"environed"

"environing"

"environment"

"environmental"

"environmentalism"

"environmentalist"

"environmentalists"

"environmentally"

"environments"

"environs"

"envisage"

"envisaged"

"envisages"

"envisaging"

"envision"

"envisioned"

"envisioning"

"envisions"

"envoi"

"envois"

"envoy"

"envoys"

"envy"

"envying"

"envyingly"

"enwheeling"

"enwinding"

"enwombing"

"enwrap"

"enwrapped"

"enwrapping"

"enzymatic"

"enzymatically"

"enzyme"

"enzymes"

"enzymically"

"enzymologies"

"enzymologist"

"enzymology"

"EOB"

"eocene"

"eof"

"eohippus"

"eohippuses"

"eolian"

"eolipiles"

"eolith"

"eolithic"

"eoliths"

"eon"

"eonian"

"eons"

"eosophobia"

"epa"

"epaulet"

"epaulets"

"epaxial"

"epee"

"epeeist"

"epeeists"

"epees"

"epergne"

"epergnes"

"ephebiatrics"

"ephedra"

"ephedras"

"ephedrin"

"ephedrine"

"ephedrins"

"ephemera"

"ephemerae"

"ephemeral"

"ephemeras"

"ephesians"

"epic"

"epical"

"epically"

"epicalyces"

"epicalyxes"

"epicanthic"

"epicene"

"epicenes"

"epicenter"

"epicenters"

"epicentral"

"epics"

"epicure"

"epicurean"

"epicureans"

"epicures"

"epicycle"

"epicycles"

"epidemic"

"epidemically"

"epidemics"

"epidemiological"

"epidemiologies"

"epidemiologist"

"epidemiology"

"epidermal"

"epidermic"

"epidermis"

"epidermization"

"epidermoidal"

"epiderms"

"epidiascope"

"epiglottis"

"epiglottises"

"epigon"

"epigram"

"epigrammatic"

"epigrammatical"

"epigrammatically"

"epigrammatism"

"epigrammatist"

"epigrammatize"

"epigrammatizer"

"epigrams"

"epigraph"

"epigrapher"

"epigraphic"

"epigraphical"

"epigraphically"

"epigraphs"

"epigraphy"

"epilepsies"

"epilepsy"

"epileptic"

"epileptics"

"epileptoid"

"epileptology"

"epilog"

"epilogs"

"epilogue"

"epilogued"

"epilogues"

"epiloguing"

"epinephrine"

"epiphanies"

"epiphany"

"epiphenomena"

"epiphenomenalism"

"epiphenomenon"

"epiphytes"

"episcopacies"

"episcopacy"

"episcopal"

"episcopalian"

"episcopalians"

"episcopally"

"episcopate"

"episcopates"

"episcope"

"episcopes"

"episode"

"episodes"

"episodic"

"episodically"

"epistasies"

"epistemology"

"epistle"

"epistler"

"epistlers"

"epistles"

"epistolary"

"epitaph"

"epitaphs"

"epithalamia"

"epithalamion"

"epithalamium"

"epithelia"

"epithelial"

"epithelium"

"epitheliums"

"epithet"

"epithets"

"epitome"

"epitomes"

"epitomic"

"epitomize"

"epitomized"

"epitomizes"

"epitomizing"

"epizoa"

"epizootic"

"epoch"

"epochal"

"epochally"

"epochs"

"epode"

"epomania"

"eponym"

"eponymic"

"eponymies"

"eponyms"

"eponymy"

"epoxied"

"epoxies"

"epoxy"

"epoxyed"

"epoxying"

"epsilon"

"epsilons"

"epsom"

"epublican"

"equability"

"equable"

"equably"

"equal"

"equaled"

"equaling"

"equalise"

"equalised"

"equalises"

"equalising"

"equalities"

"equality"

"equalization"

"equalize"

"equalized"

"equalizer"

"equalizers"

"equalizes"

"equalizing"

"equalled"

"equalling"

"equally"

"equals"

"equanimity"

"equatable"

"equate"

"equated"

"equates"

"equating"

"equation"

"equational"

"equationally"

"equations"

"equator"

"equatorial"

"equators"

"equerries"

"equerry"

"equestrian"

"equestrianism"

"equestrians"

"equestrienne"

"equestriennes"

"equiangular"

"equidistance"

"equidistant"

"equidistantly"

"equilateral"

"equilibrate"

"equilibrated"

"equilibrates"

"equilibrating"

"equilibration"

"equilibrations"

"equilibrator"

"equilibria"

"equilibrium"

"equilibriums"

"equine"

"equinely"

"equines"

"equinities"

"equinity"

"equinoctial"

"equinox"

"equinoxes"

"equip"

"equipage"

"equipages"

"equipment"

"equipments"

"equipoise"

"equipoises"

"equipped"

"equipper"

"equippers"

"equipping"

"equips"

"equitable"

"equitably"

"equitant"

"equitation"

"equites"

"equities"

"equity"

"equivalence"

"equivalences"

"equivalencies"

"equivalency"

"equivalent"

"equivalently"

"equivalents"

"equivocacies"

"equivocacy"

"equivocal"

"equivocalities"

"equivocality"

"equivocally"

"equivocalness"

"equivocate"

"equivocated"

"equivocates"

"equivocating"

"equivocation"

"equivocations"

"equivocator"

"equivocators"

"equivoke"

"equivokes"

"era"

"eradicable"

"eradicate"

"eradicated"

"eradicates"

"eradicating"

"eradication"

"eradications"

"eradicator"

"eradicators"

"eras"

"erasable"

"erase"

"erased"

"eraser"

"erasers"

"erases"

"erasing"

"erasions"

"erasmus"

"erasure"

"erasures"

"erat"

"erbium"

"erbiums"

"ere"

"erect"

"erectable"

"erected"

"erecter"

"erecters"

"erectile"

"erectilities"

"erecting"

"erection"

"erections"

"erective"

"erectly"

"erectness"

"erector"

"erectors"

"erects"

"erelong"

"eremite"

"eremites"

"eremitic"

"eremology"

"erenow"

"erewhile"

"erg"

"ergasiomania"

"ergasiophobia"

"ergo"

"ergograph"

"ergology"

"ergomania"

"ergometer"

"ergonomic"

"ergonomically"

"ergonomics"

"ergophobia"

"ergosterol"

"ergot"

"ergotic"

"ergotisms"

"ergotized"

"ergots"

"ergs"

"erica"

"ericas"

"erie"

"erigerons"

"erin"

"eriometer"

"eristic"

"eristics"

"erman"

"ermine"

"ermined"

"ermines"

"erne"

"ernest"

"erns"

"erode"

"eroded"

"erodes"

"erodible"

"eroding"

"erogenous"

"eros"

"erose"

"erosely"

"eroses"

"erosible"

"erosion"

"erosional"

"erosions"

"erosive"

"erosiveness"

"erosivity"

"erotic"

"erotica"

"erotical"

"erotically"

"eroticism"

"eroticist"

"eroticization"

"eroticize"

"eroticizing"

"erotics"

"erotism"

"erotisms"

"erotization"

"erotize"

"erotized"

"erotizing"

"erotogeneses"

"erotogenesis"

"erotogenic"

"erotomania"

"erotophobia"

"err"

"errancies"

"errancy"

"errand"

"errands"

"errant"

"errantly"

"errantries"

"errantry"

"errants"

"errata"

"erratas"

"erratic"

"erratically"

"erratics"

"erratum"

"erred"

"erring"

"erringly"

"erroneous"

"erroneously"

"erroneousness"

"error"

"errorless"

"errors"

"errs"

"ersatz"

"ersatzes"

"ersian"

"erst"

"erstwhile"

"eruct"

"eructate"

"eructated"

"eructates"

"eructating"

"eructation"

"eructed"

"eructing"

"eructs"

"erudite"

"eruditely"

"erudition"

"erupt"

"erupted"

"erupting"

"eruption"

"eruptional"

"eruptions"

"eruptive"

"eruptively"

"eruptives"

"erupts"

"erysipelas"

"erythema"

"erythrocyte"

"erythrocytes"

"erythromycin"

"erythrophobia"

"es"

"esc"

"escalade"

"escaladed"

"escalades"

"escalading"

"escalate"

"escalated"

"escalates"

"escalating"

"escalation"

"escalations"

"escalator"

"escalators"

"escalatory"

"escallop"

"escalloped"

"escalloping"

"escallops"

"escaloped"

"escalops"

"escapable"

"escapade"

"escapades"

"escape"

"escaped"

"escapee"

"escapees"

"escapement"

"escapements"

"escaper"

"escapers"

"escapes"

"escapeway"

"escaping"

"escapism"

"escapisms"

"escapist"

"escapists"

"escapology"

"escargot"

"escargots"

"escarole"

"escaroles"

"escarp"

"escarped"

"escarping"

"escarpment"

"escarpments"

"escars"

"eschalot"

"eschalots"

"eschatology"

"escheated"

"Escherichia coli"

"eschew"

"eschewal"

"eschewals"

"eschewed"

"eschewer"

"eschewers"

"eschewing"

"eschews"

"escort"

"escorted"

"escorting"

"escorts"

"escoting"

"escritoire"

"escritoires"

"escrow"

"escrowed"

"escrowee"

"escrowing"

"escrows"

"escuages"

"escudo"

"escudos"

"esculent"

"esculents"

"escutcheon"

"escutcheons"

"eses"

"eskimo"

"eskimos"

"esophagal"

"esophageal"

"esophagi"

"esophagoscope"

"esophagus"

"esoteric"

"esp"

"espadrille"

"espadrilles"

"espalier"

"espaliered"

"espaliers"

"espanol"

"espanoles"

"especial"

"especially"

"esperanto"

"espial"

"espials"

"espied"

"espies"

"espionage"

"esplanade"

"esplanades"

"espousal"

"espousals"

"espouse"

"espoused"

"espouser"

"espousers"

"espouses"

"espousing"

"espresso"

"espressos"

"esprit"

"esprits"

"espy"

"espying"

"esquire"

"esquired"

"esquires"

"esquiring"

"ess"

"essay"

"essayed"

"essayer"

"essayers"

"essaying"

"essayist"

"essayists"

"essays"

"esse"

"essence"

"essences"

"essential"

"essentially"

"essentials"

"esses"

"establish"

"establishable"

"established"

"establisher"

"establishes"

"establishing"

"establishment"

"establishments"

"establismentarian"

"establismentarianism"

"estancias"

"estate"

"estated"

"estates"

"estating"

"esteem"

"esteemed"

"esteeming"

"esteems"

"ester"

"esters"

"esther"

"esthesias"

"esthete"

"esthetes"

"esthetic"

"esthetics"

"estimable"

"estimate"

"estimated"

"Estimated due date"

"estimates"

"estimating"

"estimation"

"estimations"

"estimator"

"estimators"

"estivate"

"estivated"

"estivates"

"estivating"

"estonia"

"estonian"

"estonians"

"estop"

"estoppage"

"estopped"

"estoppel"

"estoppels"

"estopping"

"estops"

"estradiol"

"estragons"

"estrange"

"estranged"

"estrangement"

"estrangements"

"estranges"

"estranging"

"estray"

"estraying"

"estreating"

"estrin"

"estrogen"

"estrogenic"

"estrogenicity"

"estrogens"

"estrous"

"estrum"

"estrus"

"estruses"

"estuaries"

"estuary"

"et"

"eta"

"etagere"

"etageres"

"etape"

"etatism"

"etatist"

"etc"

"etcetera"

"etceteras"

"etch"

"etched"

"etcher"

"etchers"

"etches"

"etching"

"etchings"

"eternal"

"eternally"

"eternalness"

"eternals"

"eterne"

"eternise"

"eternities"

"eternity"

"eternize"

"eternized"

"eternizes"

"eternizing"

"eth"

"ethane"

"ethanes"

"ethanol"

"ethanols"

"ethel"

"ethene"

"ethenes"

"ether"

"ethereal"

"ethereally"

"etherealness"

"etheric"

"etherification"

"etherified"

"etherifies"

"etherify"

"etherish"

"etherize"

"etherized"

"etherizes"

"etherizing"

"etheromania"

"ethers"

"ethic"

"ethical"

"ethicalities"

"ethically"

"ethicalness"

"ethicals"

"ethicians"

"ethicist"

"ethicists"

"ethicize"

"ethicized"

"ethicizes"

"ethicizing"

"ethics"

"ethiopia"

"ethiopian"

"ethiopians"

"ethnic"

"ethnical"

"ethnically"

"ethnicity"

"ethnics"

"ethnogeny"

"ethnologic"

"ethnological"

"ethnologist"

"ethnologists"

"ethnology"

"ethnomania"

"ethnomethodology"

"ethnomusicology"

"ethnoses"

"ethological"

"ethologies"

"ethologist"

"ethologists"

"ethology"

"ethonomics"

"ethos"

"ethoses"

"ethyl"

"ethylated"

"ethylates"

"ethylene"

"ethylenes"

"ethyls"

"etiolate"

"etiolated"

"etiolates"

"etiolating"

"etiologic"

"etiological"

"etiologically"

"etiologies"

"etiology"

"etiquette"

"etiquettes"

"etna"

"etnas"

"etoile"

"etoiles"

"etruria"

"etruscan"

"etruscans"

"etude"

"etudes"

"etym"

"etymological"

"etymologies"

"etymologist"

"etymologists"

"etymology"

"eucalypti"

"eucalyptus"

"eucalyptuses"

"eucharist"

"eucharistic"

"eucharistical"

"eucharists"

"euchre"

"euchred"

"euchres"

"euchring"

"euclid"

"euclidean"

"eudaemon"

"eudaemons"

"eudemons"

"eudiometer"

"eugene"

"eugenic"

"eugenical"

"eugenically"

"eugenicist"

"eugenicists"

"eugenics"

"eugenism"

"eugenist"

"eugenists"

"euglena"

"euglenas"

"euler"

"eulogia"

"eulogies"

"eulogise"

"eulogist"

"eulogistic"

"eulogists"

"eulogiums"

"eulogize"

"eulogized"

"eulogizer"

"eulogizers"

"eulogizes"

"eulogizing"

"eulogomania"

"eulogy"

"eumorphic"

"eunuch"

"eunuchism"

"eunuchoid"

"eunuchs"

"euphemism"

"euphemisms"

"euphemistic"

"euphemistically"

"euphenics"

"euphobia"

"euphonies"

"euphonious"

"euphony"

"euphoria"

"euphorias"

"euphoric"

"euphorically"

"euphrates"

"eurasia"

"eurasian"

"eurasians"

"eureka"

"euripides"

"eurodollar"

"eurodollars"

"europe"

"european"

"europeans"

"europium"

"europiums"

"eurythmics"

"eurythmies"

"eurythmy"

"eustachian"

"euthanasia"

"euthenics"

"eutrophic"

"eutrophication"

"eutrophies"

"eutrophy"

"evacuants"

"evacuate"

"evacuated"

"evacuates"

"evacuating"

"evacuation"

"evacuations"

"evacuator"

"evacuators"

"evacuee"

"evacuees"

"evadable"

"evade"

"evaded"

"evader"

"evaders"

"evades"

"evadible"

"evading"

"evaluate"

"evaluated"

"evaluates"

"evaluating"

"evaluation"

"evaluations"

"evaluator"

"evaluators"

"evanesce"

"evanesced"

"evanescence"

"evanescent"

"evanescently"

"evanesces"

"evanescing"

"evangelic"

"evangelical"

"evangelicalism"

"evangelically"

"evangelicals"

"evangelism"

"evangelist"

"evangelistic"

"evangelistically"

"evangelists"

"evangelize"

"evangelized"

"evangelizes"

"evangelizing"

"evangels"

"evanished"

"evanishes"

"evans"

"evaporate"

"evaporated"

"evaporates"

"evaporating"

"evaporation"

"evaporations"

"evaporative"

"evaporator"

"evaporators"

"evaporimeter"

"evaporite"

"evaporitic"

"evasion"

"evasions"

"evasive"

"evasively"

"evasiveness"

"eve"

"even"

"evened"

"evener"

"eveners"

"evenest"

"evenfall"

"evenfalls"

"evenhanded"

"evening"

"evenings"

"evenly"

"evenness"

"evens"

"evensong"

"evensongs"

"event"

"eventful"

"eventfully"

"eventfulness"

"eventide"

"eventides"

"events"

"eventual"

"eventualities"

"eventuality"

"eventually"

"eventuate"

"eventuated"

"eventuates"

"eventuating"

"eventuation"

"eventuations"

"ever"

"everblooming"

"everest"

"everglade"

"everglades"

"evergreen"

"evergreens"

"everlasting"

"everlastingly"

"evermore"

"Evernote"

"eversion"

"eversions"

"evert"

"everted"

"everting"

"evertor"

"evertors"

"everts"

"every"

"everybody"

"everyday"

"everyman"

"everymen"

"everyone"

"everyplace"

"everything"

"everyway"

"everywhere"

"eves"

"evict"

"evicted"

"evictee"

"evictees"

"evicting"

"eviction"

"evictions"

"evictor"

"evictors"

"evicts"

"evidence"

"evidence based medicine"

"evidenced"

"evidences"

"evidencing"

"evident"

"evidential"

"evidentiary"

"evidently"

"evil"

"evildoer"

"evildoers"

"eviler"

"evilest"

"eviller"

"evillest"

"evilly"

"evilness"

"evils"

"evince"

"evinced"

"evinces"

"evincible"

"evincing"

"evincive"

"eviscerate"

"eviscerated"

"eviscerates"

"eviscerating"

"evisceration"

"eviscerations"

"evitable"

"evocable"

"evocation"

"evocations"

"evocative"

"evocator"

"evocators"

"evoke"

"evoked"

"evoker"

"evokers"

"evokes"

"evoking"

"evolutes"

"evolution"

"evolutionary"

"evolutionism"

"evolutionist"

"evolutionists"

"evolutions"

"evolve"

"evolved"

"evolvement"

"evolvements"

"evolver"

"evolvers"

"evolves"

"evolving"

"evulsions"

"evzone"

"evzones"

"ew"

"ewe"

"ewer"

"ewers"

"ewes"

"ewing"

"ewish"

"ex"

"exacerbate"

"exacerbated"

"exacerbates"

"exacerbating"

"exacerbatingly"

"exacerbation"

"exacerbations"

"exact"

"exacta"

"exactas"

"exacted"

"exacter"

"exacters"

"exactest"

"exacting"

"exactingly"

"exactingness"

"exaction"

"exactions"

"exactitude"

"exactly"

"exactness"

"exactor"

"exactors"

"exacts"

"exaggerate"

"exaggerated"

"exaggeratedly"

"exaggerates"

"exaggerating"

"exaggeration"

"exaggerations"

"exaggerative"

"exaggerator"

"exaggerators"

"exalt"

"exaltation"

"exaltations"

"exalted"

"exalter"

"exalters"

"exalting"

"exalts"

"exam"

"examination"

"examinations"

"examine"

"examined"

"examinee"

"examinees"

"examiner"

"examiners"

"examines"

"examining"

"example"

"exampled"

"examples"

"exampling"

"exams"

"exarch"

"exarchies"

"exarchs"

"exarchy"

"exasperate"

"exasperated"

"exasperates"

"exasperating"

"exasperation"

"excavate"

"excavated"

"excavates"

"excavating"

"excavation"

"excavations"

"excavator"

"excavators"

"exceed"

"exceeded"

"exceeder"

"exceeders"

"exceeding"

"exceedingly"

"exceeds"

"excel"

"excelled"

"excellence"

"excellences"

"excellencies"

"excellency"

"excellent"

"excellently"

"excelling"

"excels"

"excelsior"

"except"

"excepted"

"excepting"

"exception"

"exceptionable"

"exceptional"

"exceptionality"

"exceptionally"

"exceptions"

"excepts"

"excerpt"

"excerpted"

"excerpting"

"excerpts"

"excess"

"excesses"

"excessive"

"excessively"

"excessiveness"

"exchange"

"exchangeable"

"exchanged"

"exchanger"

"exchanges"

"exchanging"

"exchequer"

"exchequers"

"excisable"

"excise"

"excised"

"exciseman"

"excisemen"

"excises"

"excising"

"excision"

"excisions"

"excitabilities"

"excitability"

"excitable"

"excitant"

"excitants"

"excitation"

"excitations"

"excitatory"

"excite"

"excited"

"excitedly"

"excitement"

"excitements"

"exciter"

"exciters"

"excites"

"exciting"

"excitons"

"excitor"

"excitors"

"exclaim"

"exclaimed"

"exclaimer"

"exclaimers"

"exclaiming"

"exclaims"

"exclamation"

"exclamations"

"exclamatory"

"exclave"

"exclaves"

"exclude"

"excluded"

"excluder"

"excluders"

"excludes"

"excluding"

"exclusion"

"exclusions"

"exclusive"

"exclusively"

"exclusiveness"

"exclusivity"

"excogitate"

"excogitated"

"excogitates"

"excogitating"

"excommunicate"

"excommunicated"

"excommunicates"

"excommunicating"

"excommunication"

"excommunications"

"excommunicator"

"excommunicators"

"excoriate"

"excoriated"

"excoriates"

"excoriating"

"excoriation"

"excoriations"

"excrement"

"excremental"

"excrements"

"excrescence"

"excrescences"

"excrescent"

"excreta"

"excretal"

"excrete"

"excreted"

"excreter"

"excreters"

"excretes"

"excreting"

"excretion"

"excretions"

"excretory"

"excruciate"

"excruciating"

"excruciatingly"

"exculpate"

"exculpated"

"exculpates"

"exculpating"

"exculpation"

"exculpations"

"excursion"

"excursionist"

"excursionists"

"excursions"

"excursive"

"excursively"

"excursiveness"

"excursus"

"excursuses"

"excusable"

"excusableness"

"excuse"

"excused"

"excuser"

"excusers"

"excuses"

"excusing"

"exec"

"execeptional"

"execrable"

"execrably"

"execrate"

"execrated"

"execrates"

"execrating"

"execration"

"execrations"

"execrator"

"execrators"

"execs"

"executable"

"execute"

"executed"

"executer"

"executers"

"executes"

"executing"

"execution"

"executional"

"executioner"

"executioners"

"executions"

"executive"

"executives"

"executor"

"executorial"

"executors"

"executorship"

"executory"

"executrices"

"executrix"

"executrixes"

"exedra"

"exegeses"

"exegesis"

"exegete"

"exegetes"

"exegetic"

"exempla"

"exemplar"

"exemplars"

"exemplary"

"exempli"

"exemplification"

"exemplifications"

"exemplified"

"exemplifies"

"exemplify"

"exemplifying"

"exemplum"

"exempt"

"exempted"

"exemptible"

"exempting"

"exemption"

"exemptions"

"exemptive"

"exempts"

"exercisable"

"exercise"

"exercise timer"

"exercised"

"exerciser"

"exercisers"

"exercises"

"exercising"

"exert"

"exerted"

"exerting"

"exertion"

"exertions"

"exertive"

"exerts"

"exes"

"exfoliate"

"exhalant"

"exhalants"

"exhalation"

"exhalations"

"exhale"

"exhaled"

"exhalent"

"exhales"

"exhaling"

"exhaust"

"exhausted"

"exhaustible"

"exhausting"

"exhaustion"

"exhaustive"

"exhaustless"

"exhausts"

"exhibit"

"exhibitant"

"exhibited"

"exhibiter"

"exhibiters"

"exhibiting"

"exhibition"

"exhibitioner"

"exhibitionism"

"exhibitionist"

"exhibitionists"

"exhibitions"

"exhibitor"

"exhibitors"

"exhibits"

"exhilarate"

"exhilarated"

"exhilarates"

"exhilarating"

"exhilaration"

"exhilarative"

"exhort"

"exhortation"

"exhortations"

"exhorted"

"exhorter"

"exhorters"

"exhorting"

"exhorts"

"exhumation"

"exhumations"

"exhume"

"exhumed"

"exhumer"

"exhumers"

"exhumes"

"exhuming"

"exhusband"

"exican"

"exigence"

"exigences"

"exigencies"

"exigency"

"exigent"

"exigible"

"exiguities"

"exiguity"

"exiguous"

"exile"

"exiled"

"exiles"

"exilic"

"exiling"

"exist"

"existed"

"existence"

"existences"

"existent"

"existential"

"existentialism"

"existentialist"

"existentialists"

"existents"

"existing"

"exists"

"exit"

"exited"

"exiting"

"exits"

"exobiological"

"exobiologist"

"exobiologists"

"exobiology"

"exocrine"

"exocrinologies"

"exodus"

"exoduses"

"exogamic"

"exogamies"

"exogamous"

"exogamy"

"exogenous"

"exogenously"

"exonerate"

"exonerated"

"exonerates"

"exonerating"

"exoneration"

"exonerations"

"exonerator"

"exonerators"

"exorbitance"

"exorbitant"

"exorbitantly"

"exorcise"

"exorcised"

"exorciser"

"exorcisers"

"exorcises"

"exorcising"

"exorcism"

"exorcisms"

"exorcist"

"exorcists"

"exorcize"

"exorcized"

"exorcizes"

"exorcizing"

"exordia"

"exordium"

"exordiums"

"exoskeleton"

"exosphere"

"exospheres"

"exospheric"

"exoteric"

"exoterically"

"exothermal"

"exothermic"

"exotic"

"exotica"

"exotically"

"exoticism"

"exotics"

"exotism"

"exotisms"

"exotoxic"

"exotoxin"

"expand"

"expandable"

"expanded"

"expander"

"expanders"

"expandible"

"expanding"

"expands"

"expanse"

"expanses"

"expansible"

"expansion"

"expansionary"

"expansionism"

"expansionist"

"expansionists"

"expansions"

"expansive"

"expansively"

"expansiveness"

"expatiate"

"expatiated"

"expatiates"

"expatiating"

"expatiation"

"expatiations"

"expatiator"

"expatiators"

"expatriate"

"expatriated"

"expatriates"

"expatriating"

"expatriation"

"expatriations"

"expect"

"expectable"

"expectance"

"expectancies"

"expectancy"

"expectant"

"expectantly"

"expectation"

"expectations"

"expectative"

"expected"

"expectedly"

"expecter"

"expecters"

"expecting"

"expectorant"

"expectorants"

"expectorate"

"expectorated"

"expectorates"

"expectorating"

"expectoration"

"expectorations"

"expectorator"

"expectorators"

"expects"

"expedience"

"expediences"

"expediencies"

"expediency"

"expedient"

"expediential"

"expediently"

"expedients"

"expedite"

"expedited"

"expediter"

"expediters"

"expedites"

"expediting"

"expedition"

"expeditionary"

"expeditions"

"expeditious"

"expeditiously"

"expeditiousness"

"expeditor"

"expel"

"expellable"

"expelled"

"expellee"

"expellees"

"expeller"

"expellers"

"expelling"

"expels"

"expend"

"expendability"

"expendable"

"expended"

"expender"

"expenders"

"expending"

"expenditure"

"expenditures"

"expends"

"expense"

"expensed"

"expenses"

"expensing"

"expensive"

"expensively"

"expensiveness"

"experience"

"experienced"

"experiences"

"experiencing"

"experiential"

"experiment"

"experimental"

"experimentalist"

"experimentally"

"experimentation"

"experimented"

"experimenter"

"experimenters"

"experimenting"

"experiments"

"expert"

"experted"

"experting"

"expertise"

"expertly"

"expertness"

"experts"

"experts answer"

"expiable"

"expiate"

"expiated"

"expiates"

"expiating"

"expiation"

"expiations"

"expiator"

"expiators"

"expiatory"

"expiration"

"expirations"

"expiratory"

"expire"

"expired"

"expirer"

"expirers"

"expires"

"expiries"

"expiring"

"explain"

"explainable"

"explained"

"explainer"

"explainers"

"explaining"

"explains"

"explanation"

"explanations"

"explanatory"

"explanted"

"explanting"

"expletive"

"expletives"

"explicable"

"explicate"

"explicated"

"explicates"

"explicating"

"explication"

"explications"

"explicator"

"explicators"

"explicit"

"explicitly"

"explicitness"

"explicits"

"explode"

"exploded"

"exploder"

"exploders"

"explodes"

"exploding"

"exploit"

"exploitable"

"exploitation"

"exploitations"

"exploitative"

"exploited"

"exploitee"

"exploiter"

"exploiters"

"exploiting"

"exploits"

"exploration"

"explorations"

"exploratory"

"explore"

"explored"

"explorer"

"explorers"

"explores"

"exploring"

"explosion"

"explosions"

"explosive"

"explosively"

"explosiveness"

"explosives"

"expo"

"exponent"

"exponential"

"exponentially"

"exponents"

"export"

"exportable"

"exportation"

"exportations"

"exported"

"exporter"

"exporters"

"exporting"

"exports"

"expos"

"exposal"

"exposals"

"expose"

"exposed"

"exposer"

"exposers"

"exposes"

"exposing"

"exposit"

"exposited"

"expositing"

"exposition"

"expositions"

"expositor"

"expositors"

"expository"

"exposits"

"expostulate"

"expostulated"

"expostulates"

"expostulating"

"expostulation"

"expostulations"

"exposure"

"exposures"

"expound"

"expounded"

"expounder"

"expounders"

"expounding"

"expounds"

"express"

"expressed"

"expresses"

"expressible"

"expressing"

"expression"

"expressionism"

"expressionist"

"expressionistic"

"expressionists"

"expressionless"

"expressions"

"expressive"

"expressively"

"expressiveness"

"expressly"

"expressway"

"expressways"

"expropriate"

"expropriated"

"expropriates"

"expropriating"

"expropriation"

"expropriations"

"expropriator"

"expulse"

"expulsed"

"expulses"

"expulsing"

"expulsion"

"expulsions"

"expunge"

"expunged"

"expunger"

"expungers"

"expunges"

"expunging"

"expurgate"

"expurgated"

"expurgates"

"expurgating"

"expurgation"

"expurgations"

"expurgator"

"expurgators"

"expwy"

"exquisite"

"exquisitely"

"exquisiteness"

"exsanguine"

"exscinding"

"exsert"

"exserted"

"exserting"

"exserts"

"ext"

"extant"

"extemporaneous"

"extemporaneously"

"extemporaneousness"

"extemporary"

"extempore"

"extemporize"

"extemporized"

"extemporizes"

"extemporizing"

"extend"

"extendability"

"extendable"

"extended"

"extender"

"extenders"

"extendibility"

"extendible"

"extending"

"extends"

"extensible"

"extension"

"extensions"

"extensive"

"extensively"

"extensiveness"

"extensometer"

"extensor"

"extensors"

"extent"

"extents"

"extenuate"

"extenuated"

"extenuates"

"extenuating"

"extenuation"

"extenuations"

"exterior"

"exteriorize"

"exteriorized"

"exteriorizing"

"exteriorly"

"exteriors"

"exterminate"

"exterminated"

"exterminates"

"exterminating"

"extermination"

"exterminations"

"exterminator"

"exterminators"

"extern"

"external"

"externalism"

"externalization"

"externalize"

"externalized"

"externalizes"

"externalizing"

"externally"

"externals"

"externs"

"exterritoriality"

"extinct"

"extincted"

"extincting"

"extinction"

"extinctions"

"extincts"

"extinguised"

"extinguish"

"extinguishable"

"extinguished"

"extinguisher"

"extinguishers"

"extinguishes"

"extinguishing"

"extinguishment"

"extirpate"

"extirpated"

"extirpates"

"extirpating"

"extirpation"

"extirpations"

"extirpator"

"extol"

"extoll"

"extolled"

"extoller"

"extollers"

"extolling"

"extolls"

"extols"

"extorsion"

"extorsive"

"extort"

"extorted"

"extorter"

"extorters"

"extorting"

"extortion"

"extortionate"

"extortionately"

"extortioner"

"extortioners"

"extortionist"

"extortionists"

"extortions"

"extorts"

"extra"

"extra content"

"extracellular"

"extract"

"extracted"

"extracting"

"extraction"

"extractions"

"extractive"

"extractor"

"extractors"

"extracts"

"extracurricular"

"extraditable"

"extradite"

"extradited"

"extradites"

"extraditing"

"extradition"

"extraditions"

"extrados"

"extradoses"

"extragalactic"

"extrajudicially"

"extralegal"

"extramarital"

"extramural"

"extraneous"

"extraneously"

"extraneousness"

"extranuclear"

"extraordinarily"

"extraordinary"

"extrapolate"

"extrapolated"

"extrapolates"

"extrapolating"

"extrapolation"

"extrapolations"

"extras"

"extrasensory"

"extraterrestrial"

"extraterrestrially"

"extraterrestrials"

"extraterritorial"

"extraterritoriality"

"extraterritorials"

"extrauterine"

"extravagance"

"extravagances"

"extravagant"

"extravagantly"

"extravagantness"

"extravaganza"

"extravaganzas"

"extravehicular"

"extravert"

"extreme"

"extremely"

"extremeness"

"extremer"

"extremes"

"extremest"

"extremis"

"extremism"

"extremist"

"extremists"

"extremities"

"extremity"

"extricable"

"extricate"

"extricated"

"extricates"

"extricating"

"extrication"

"extrications"

"extrinsic"

"extrinsically"

"extrospection"

"extroversion"

"extroversive"

"extrovert"

"extroverted"

"extroverts"

"extrude"

"extruded"

"extruder"

"extruders"

"extrudes"

"extruding"

"extrusion"

"extrusions"

"extrusive"

"exuberance"

"exuberant"

"exuberantly"

"exudate"

"exudates"

"exudation"

"exudations"

"exudative"

"exude"

"exuded"

"exudes"

"exuding"

"exult"

"exultant"

"exultantly"

"exultation"

"exulted"

"exulting"

"exultingly"

"exults"

"exurb"

"exurban"

"exurbanite"

"exurbanites"

"exurbia"

"exurbias"

"exurbs"

"exxon"

"eye"

"eyeable"

"eyeball"

"eyeballed"

"eyeballing"

"eyeballs"

"eyebeam"

"eyebeams"

"eyebolt"

"eyebolts"

"eyebrow"

"eyebrows"

"eyecup"

"eyecups"

"eyed"

"eyedness"

"eyedropper"

"eyedropperful"

"eyedroppers"

"eyeful"

"eyefuls"

"eyeglass"

"eyeglasses"

"eyehole"

"eyeholes"

"eyehook"

"eyehooks"

"eyeing"

"eyelash"

"eyelashes"

"eyeless"

"eyelet"

"eyelets"

"eyeletted"

"eyeletting"

"eyelid"

"eyelids"

"eyeliner"

"eyeliners"

"eyepiece"

"eyepieces"

"eyepoint"

"eyepoints"

"eyer"

"eyers"

"eyes"

"eyeshade"

"eyeshades"

"eyeshot"

"eyeshots"

"eyesight"

"eyesights"

"eyesore"

"eyesores"

"eyespots"

"eyestalk"

"eyestalks"

"eyestone"

"eyestones"

"eyestrain"

"eyeteeth"

"eyetooth"

"eyewash"

"eyewashes"

"eyewaters"

"eyewink"

"eyewinks"

"eyewitness"

"eyewitnesses"

"eying"

"eyrie"

"eyries"

"eyrir"

"ezekiel"

"fabaceous"

"fabian"

"fable"

"fabled"

"fabler"

"fablers"

"fables"

"fabling"

"fabric"

"fabricate"

"fabricated"

"fabricates"

"fabricating"

"fabrication"

"fabrications"

"fabricator"

"fabricators"

"fabrics"

"fabulist"

"fabulists"

"fabulous"

"fabulously"

"facade"

"facades"

"face"

"face whatever challenges"

"faceable"

"Facebook"

"faced"

"facedown"

"faceless"

"facelessness"

"facelift"

"facelifts"

"facer"

"facers"

"faces"

"facet"

"faceted"

"faceting"

"facetious"

"facetiously"

"facetiousness"

"facets"

"facetted"

"facetting"

"faceup"

"facia"

"facial"

"facially"

"facials"

"facias"

"facie"

"facies"

"facile"

"facilely"

"facileness"

"facilitate"

"facilitated"

"facilitates"

"facilitating"

"facilitation"

"facilities"

"facility"

"facing"

"facings"

"facsimile"

"facsimiles"

"fact"

"factful"

"faction"

"factional"

"factionalism"

"factions"

"factious"

"factiously"

"factiousness"

"factitious"

"factitiously"

"factitiousness"

"facto"

"factor"

"factorable"

"factorage"

"factored"

"factorial"

"factorials"

"factories"

"factoring"

"factorize"

"factorized"

"factors"

"factorship"

"factory"

"factotum"

"factotums"

"facts"

"factual"

"factualism"

"factually"

"facula"

"faculae"

"faculties"

"faculty"

"fad"

"fadable"

"faddier"

"faddish"

"faddism"

"faddisms"

"faddist"

"faddists"

"faddy"

"fade"

"fadeaway"

"fadeaways"

"faded"

"fadedly"

"fadeless"

"fadeout"

"fader"

"faders"

"fades"

"fading"

"fadings"

"fads"

"faeces"

"faerie"

"faeries"

"faery"

"fag"

"fagged"

"fagging"

"faggot"

"faggoting"

"faggots"

"fagot"

"fagoted"

"fagoter"

"fagoting"

"fagotings"

"fagots"

"fags"

"fahrenheit"

"faience"

"faiences"

"fail"

"failed"

"failing"

"failingly"

"failings"

"faille"

"fails"

"failsafe"

"failure"

"failures"

"fain"

"fainer"

"fainest"

"faint"

"fainted"

"fainter"

"fainters"

"faintest"

"fainthearted"

"faintheartedly"

"faintheartedness"

"fainting"

"faintish"

"faintly"

"faintness"

"faints"

"fair"

"faire"

"faired"

"fairer"

"fairest"

"fairground"

"fairgrounds"

"fairies"

"fairing"

"fairings"

"fairish"

"fairly"

"fairness"

"fairs"

"fairway"

"fairways"

"fairy"

"fairyism"

"fairyland"

"fairylands"

"fait"

"faith"

"faithed"

"faithful"

"faithfully"

"faithfulness"

"faithfuls"

"faithing"

"faithless"

"faithlessly"

"faithlessness"

"faiths"

"faits"

"fake"

"faked"

"fakeer"

"fakeers"

"faker"

"fakeries"

"fakers"

"fakery"

"fakes"

"faking"

"fakir"

"fakirs"

"falchion"

"falchions"

"falcon"

"falconer"

"falconers"

"falconet"

"falconets"

"falconries"

"falconry"

"falcons"

"fall"

"fallacies"

"fallacious"

"fallaciously"

"fallacy"

"fallback"

"fallbacks"

"fallen"

"faller"

"fallers"

"fallibility"

"fallible"

"fallibleness"

"fallibly"

"falling"

"fallings"

"falloff"

"falloffs"

"fallopian"

"fallout"

"fallouts"

"fallow"

"fallowed"

"fallowing"

"fallows"

"falls"

"false"

"falsehood"

"falsehoods"

"falsely"

"falseness"

"falser"

"falsest"

"falsetto"

"falsettos"

"falsie"

"falsies"

"falsifiability"

"falsifiable"

"falsification"

"falsifications"

"falsified"

"falsifier"

"falsifiers"

"falsifies"

"falsify"

"falsifying"

"falsities"

"falsity"

"faltboat"

"faltboats"

"falter"

"faltered"

"falterer"

"falterers"

"faltering"

"falteringly"

"falters"

"fame"

"famed"

"fameless"

"fames"

"familarity"

"familia"

"familial"

"familiar"

"familiarities"

"familiarity"

"familiarization"

"familiarizations"

"familiarize"

"familiarized"

"familiarizes"

"familiarizing"

"familiarly"

"familiarness"

"familiars"

"families"

"family"

"Family Doctor"

"family health record"

"family history"

"family planning"

"famine"

"famines"

"faming"

"famish"

"famished"

"famishes"

"famishing"

"famous"

"famously"

"fan"

"fanatic"

"fanatical"

"fanatically"

"fanaticism"

"fanaticize"

"fanaticized"

"fanatics"

"fancied"

"fancier"

"fanciers"

"fancies"

"fanciest"

"fanciful"

"fancifully"

"fancifulness"

"fancily"

"fanciness"

"fancy"

"fancying"

"fancywork"

"fandango"

"fandangos"

"fandom"

"fandoms"

"fanes"

"fanfare"

"fanfares"

"fanfarons"

"fanfold"

"fanfolds"

"fang"

"fanged"

"fangless"

"fangs"

"fanjet"

"fanjets"

"fanlight"

"fanlights"

"fanned"

"fanner"

"fanners"

"fannies"

"fanning"

"fanny"

"fans"

"fantail"

"fantailed"

"fantails"

"fantasia"

"fantasias"

"fantasie"

"fantasied"

"fantasies"

"fantasist"

"fantasists"

"fantasize"

"fantasized"

"fantasizes"

"fantasizing"

"fantasm"

"fantasms"

"fantast"

"fantastic"

"fantastical"

"fantastically"

"fantasticalness"

"fantasts"

"fantasy"

"fantasying"

"fantod"

"fantods"

"fantom"

"fantoms"

"fanwise"

"fanwort"

"fanworts"

"fanzine"

"fanzines"

"faqir"

"faqirs"

"faquir"

"far"

"farad"

"faraday"

"faradays"

"farads"

"faraway"

"farce"

"farced"

"farcer"

"farcers"

"farces"

"farceurs"

"farcical"

"farcies"

"farcing"

"farcy"

"fards"

"fare"

"fared"

"farer"

"farers"

"fares"

"farewell"

"farewelled"

"farewells"

"farfels"

"farfetched"

"farina"

"farinaceous"

"farinas"

"faring"

"farm"

"farmable"

"farmed"

"farmer"

"farmers"

"farmhand"

"farmhands"

"farmhouse"

"farmhouses"

"farming"

"farmings"

"farmland"

"farmlands"

"farms"

"farmstead"

"farmsteads"

"farmyard"

"farmyards"

"farness"

"faro"

"faroff"

"faros"

"farrago"

"farragoes"

"farrier"

"farriers"

"farriery"

"farrow"

"farrowed"

"farrowing"

"farrows"

"farseeing"

"farsighted"

"farsightedly"

"farsightedness"

"fart"

"farted"

"farther"

"farthermost"

"farthest"

"farthing"

"farthingale"

"farthingales"

"farthings"

"farting"

"farts"

"fasces"

"fascia"

"fasciae"

"fascial"

"fascias"

"fascicle"

"fascicled"

"fascicles"

"fascinate"

"fascinated"

"fascinates"

"fascinating"

"fascination"

"fascinations"

"fascism"

"fascisms"

"fascist"

"fascistic"

"fascists"

"fashed"

"fashes"

"fashion"

"fashionable"

"fashionableness"

"fashionably"

"fashioned"

"fashioner"

"fashioners"

"fashioning"

"fashions"

"fast"

"fastback"

"fastbacks"

"fastball"

"fastballs"

"fasted"

"fasten"

"fastened"

"fastener"

"fasteners"

"fastening"

"fastenings"

"fastens"

"faster"

"fastest"

"fastidious"

"fastidiously"

"fastidiousness"

"fasting"

"fastings"

"fastness"

"fastnesses"

"fasts"

"fat"

"fatal"

"fatale"

"fatales"

"fatalism"

"fatalisms"

"fatalist"

"fatalistic"

"fatalistically"

"fatalists"

"fatalities"

"fatality"

"fatally"

"fatalness"

"fatback"

"fatbacks"

"fate"

"fated"

"fateful"

"fatefully"

"fatefulness"

"fates"

"fathead"

"fatheaded"

"fatheads"

"father"

"fathered"

"fatherhood"

"fathering"

"fatherland"

"fatherlands"

"fatherless"

"fatherliness"

"fatherly"

"fathers"

"fathom"

"fathomable"

"fathomed"

"fathometer"

"fathoming"

"fathomless"

"fathoms"

"fatigability"

"fatigable"

"fatiguabilities"

"fatiguability"

"fatiguable"

"fatigue"

"fatigued"

"fatigueless"

"fatigues"

"fatiguing"

"fating"

"fatless"

"fatly"

"fatness"

"fatnesses"

"fats"

"fatso"

"fatsoes"

"fatsos"

"fatstocks"

"fatted"

"fatten"

"fattened"

"fattener"

"fatteners"

"fattening"

"fattens"

"fatter"

"fattest"

"fattier"

"fatties"

"fattiest"

"fattily"

"fatting"

"fattish"

"fatty"

"fatuities"

"fatuity"

"fatuous"

"fatuously"

"fatuousness"

"fatuus"

"faubourg"

"faubourgs"

"fauces"

"faucet"

"faucets"

"faugh"

"faulkner"

"fault"

"faulted"

"faultfinder"

"faultfinders"

"faultfinding"

"faultier"

"faultiest"

"faultily"

"faultiness"

"faulting"

"faultless"

"faultlessly"

"faultlessness"

"faults"

"faulty"

"faun"

"fauna"

"faunae"

"faunal"

"faunally"

"faunas"

"fauns"

"faust"

"faustian"

"faut"

"fauve"

"fauves"

"fauvism"

"fauvisms"

"fauvist"

"fauvists"

"faux"

"favor"

"favorable"

"favorableness"

"favorably"

"favored"

"favorer"

"favorers"

"favoring"

"favorite"

"favorites"

"favoritism"

"favors"

"favour"

"favoured"

"favourer"

"favourers"

"favouring"

"favours"

"fawn"

"fawned"

"fawner"

"fawners"

"fawnier"

"fawning"

"fawningly"

"fawns"

"fawny"

"fax"

"faxed"

"faxes"

"faxing"

"fay"

"faying"

"fays"

"faze"

"fazed"

"fazes"

"fazing"

"fbi"

"FDA"

"fealties"

"fealty"

"fear"

"feared"

"fearer"

"fearers"

"fearful"

"fearfuller"

"fearfully"

"fearfulness"

"fearing"

"fearless"

"fearlessly"

"fearlessness"

"fears"

"fearsome"

"fearsomely"

"feasance"

"feasances"

"feasant"

"fease"

"feasibility"

"feasible"

"feasibleness"

"feasibly"

"feast"

"feasted"

"feaster"

"feasters"

"feastful"

"feasting"

"feasts"

"feat"

"feater"

"featest"

"feather"

"featherbed"

"featherbedded"

"featherbedding"

"featherbrain"

"featherbrained"

"feathered"

"featheredge"

"featheredges"

"featherier"

"featheriness"

"feathering"

"featherless"

"feathers"

"featherweight"

"featherweights"

"feathery"

"featlier"

"featliest"

"featly"

"feats"

"feature"

"feature limit"

"feature point"

"featured"

"featureless"

"features"

"featuring"

"feaze"

"febrifuge"

"febrifuges"

"febrile"

"february"

"fecal"

"feces"

"feckless"

"fecklessly"

"feculent"

"fecund"

"fecundate"

"fecundated"

"fecundates"

"fecundating"

"fecundation"

"fecundations"

"fecundity"

"fed"

"fedayeen"

"federacy"

"federal"

"federalism"

"federalist"

"federalists"

"federalization"

"federalizations"

"federalize"

"federalized"

"federalizes"

"federalizing"

"federally"

"federals"

"federate"

"federated"

"federates"

"federating"

"federation"

"federational"

"federations"

"federative"

"federatively"

"federator"

"fedora"

"fedoras"

"feds"

"fee"

"feeble"

"feebleminded"

"feeblemindedly"

"feeblemindedness"

"feebleness"

"feebler"

"feeblest"

"feeblish"

"feebly"

"feed"

"feed back"

"feedable"

"feedback"

"feedbacks"

"feedbag"

"feedbags"

"feedbox"

"feedboxes"

"feeder"

"feeders"

"feeding"

"feedings"

"feedlot"

"feedlots"

"feeds"

"feedstuff"

"feedstuffs"

"feeing"

"feel"

"feeler"

"feelers"

"feeless"

"feeling"

"feeling rejuvenated"

"feelingly"

"feelings"

"feels"

"fees"

"feet"

"feetless"

"feign"

"feigned"

"feignedly"

"feigner"

"feigners"

"feigning"

"feigns"

"feinschmecker"

"feinschmeckers"

"feint"

"feinted"

"feinting"

"feints"

"feist"

"feistier"

"feistiest"

"feists"

"feisty"

"feldspar"

"feldspars"

"felicitate"

"felicitated"

"felicitates"

"felicitating"

"felicitation"

"felicitations"

"felicitator"

"felicitators"

"felicities"

"felicitous"

"felicitously"

"felicity"

"feline"

"felinely"

"felines"

"felinities"

"felinity"

"felix"

"fell"

"fella"

"fellable"

"fellah"

"fellaheen"

"fellahin"

"fellahs"

"fellas"

"fellate"

"fellated"

"fellatee"

"fellating"

"fellatio"

"fellation"

"fellations"

"fellatios"

"fellator"

"fellatrice"

"fellatrices"

"fellatrix"

"fellatrixes"

"felled"

"feller"

"fellers"

"fellest"

"fellies"

"felling"

"fellness"

"felloe"

"felloes"

"fellow"

"fellowed"

"fellowing"

"fellowly"

"fellowman"

"fellowmen"

"fellows"

"fellowship"

"fellowships"

"fells"

"felly"

"felon"

"felonies"

"felonious"

"feloniously"

"feloniousness"

"felonries"

"felons"

"felony"

"felt"

"felted"

"felting"

"feltings"

"felts"

"feltwork"

"feluccas"

"fem"

"female"

"female body"

"femaleness"

"females"

"feminacies"

"feminacy"

"feminine"

"femininely"

"feminines"

"femininity"

"feminise"

"feminism"

"feminisms"

"feminist"

"feministic"

"feminists"

"feminities"

"feminity"

"feminization"

"feminize"

"feminized"

"feminizes"

"feminizing"

"femme"

"femmes"

"femora"

"femoral"

"femur"

"femurs"

"fen"

"fence"

"fenced"

"fenceless"

"fencepost"

"fencer"

"fencers"

"fences"

"fencible"

"fencibles"

"fencing"

"fencings"

"fend"

"fended"

"fender"

"fendered"

"fenders"

"fending"

"fends"

"fenestrae"

"fenestration"

"fennec"

"fennecs"

"fennel"

"fennels"

"fenny"

"fens"

"fenugreek"

"feoff"

"feoffment"

"feral"

"ferlies"

"fermata"

"fermatas"

"ferment"

"fermentable"

"fermentation"

"fermentations"

"fermentative"

"fermented"

"fermenting"

"ferments"

"fermi"

"fermis"

"fermium"

"fern"

"ferneries"

"fernery"

"ferniest"

"fernless"

"ferns"

"ferny"

"ferocious"

"ferociously"

"ferociousness"

"ferocities"

"ferocity"

"ferret"

"ferreted"

"ferreter"

"ferreters"

"ferreting"

"ferrets"

"ferrety"

"ferriage"

"ferric"

"ferried"

"ferries"

"ferris"

"ferrite"

"ferrites"

"ferromagnetic"

"ferromagnetism"

"ferrotype"

"ferrotypes"

"ferrous"

"ferrule"

"ferruled"

"ferrules"

"ferruling"

"ferrum"

"ferrums"

"ferry"

"ferryage"

"ferryboat"

"ferryboats"

"ferrying"

"ferryman"

"ferrymen"

"fertile"

"fertile period"

"fertilely"

"fertileness"

"fertilisation"

"fertilities"

"fertility"

"Fertility Awareness"

"fertility awareness methods"

"fertility tracking app"

"fertilizable"

"fertilization"

"fertilizations"

"fertilize"

"fertilized"

"fertilizer"

"fertilizers"

"fertilizes"

"fertilizing"

"ferule"

"feruled"

"ferules"

"feruling"

"fervencies"

"fervency"

"fervent"

"fervently"

"fervid"

"fervidly"

"fervidness"

"fervor"

"fervors"

"fervour"

"fervours"

"fescue"

"fescues"

"fess"

"fesse"

"fessed"

"fesses"

"fessing"

"festal"

"fester"

"festered"

"festering"

"festers"

"festival"

"festivals"

"festive"

"festively"

"festiveness"

"festivities"

"festivity"

"festoon"

"festooned"

"festooning"

"festoons"

"feta"

"fetal"

"fetas"

"fetch"

"fetched"

"fetcher"

"fetchers"

"fetches"

"fetching"

"fetchingly"

"fete"

"feted"

"fetes"

"feticide"

"feticides"

"fetid"

"fetidly"

"fetidness"

"feting"

"fetish"

"fetishes"

"fetishism"

"fetishist"

"fetishistic"

"fetishists"

"fetlock"

"fetlocks"

"fetor"

"fetors"

"fetted"

"fetter"

"fettered"

"fetterer"

"fetterers"

"fettering"

"fetters"

"fettle"

"fettles"

"fettlings"

"fettucini"

"fetus"

"fetuses"

"feud"

"feudal"

"feudalism"

"feudalist"

"feudalistic"

"feudalists"

"feudally"

"feudary"

"feudatories"

"feudatory"

"feuded"

"feuding"

"feudist"

"feudists"

"feuds"

"fever"

"fevered"

"feverfew"

"feverfews"

"fevering"

"feverish"

"feverishly"

"feverishness"

"feverous"

"fevers"

"few"

"fewer"

"fewest"

"fewness"

"fewnesses"

"fey"

"feyer"

"feyest"

"feyness"

"feynesses"

"fez"

"fezes"

"fezzed"

"fezzes"

"fiance"

"fiancee"

"fiancees"

"fiances"

"fiasco"

"fiascoes"

"fiascos"

"fiat"

"fiats"

"fib"

"fibbed"

"fibber"

"fibbers"

"fibbing"

"fiber"

"fiberboard"

"fibered"

"fiberfill"

"fiberglass"

"fiberize"

"fiberized"

"fiberizes"

"fiberizing"

"fibers"

"fiberscope"

"fibre"

"fibres"

"fibril"

"fibrillate"

"fibrillation"

"fibrillations"

"fibrils"

"fibrin"

"fibrinogen"

"fibrinous"

"fibrins"

"fibroid"

"fibroids"

"fibroin"

"fibroma"

"fibrose"

"fibrosis"

"fibrous"

"fibs"

"fibula"

"fibulae"

"fibular"

"fibulas"

"fica"

"fiche"

"fiches"

"fichu"

"fichus"

"fickle"

"fickleness"

"fickler"

"ficklest"

"fiction"

"fictional"

"fictionalize"

"fictionalized"

"fictionalizes"

"fictionalizing"

"fictionally"

"fictions"

"fictitious"

"fictitiously"

"fictive"

"fid"

"fiddle"

"fiddled"

"fiddler"

"fiddlers"

"fiddles"

"fiddlestick"

"fiddlesticks"

"fiddling"

"fide"

"fidel"

"fideles"

"fidelis"

"fidelities"

"fidelity"

"fides"

"fidget"

"fidgeted"

"fidgeter"

"fidgeters"

"fidgetiness"

"fidgeting"

"fidgets"

"fidgety"

"fido"

"fidos"

"fids"

"fiducial"

"fiducially"

"fiduciaries"

"fiduciarily"

"fiduciary"

"fie"

"fief"

"fiefdom"

"fiefdoms"

"fiefs"

"field"

"fielded"

"fielder"

"fielders"

"fielding"

"fieldleft"

"fieldmice"

"fieldpiece"

"fieldpieces"

"fields"

"fieldstone"

"fieldwork"

"fiend"

"fiendish"

"fiendishly"

"fiendishness"

"fiends"

"fierce"

"fiercely"

"fierceness"

"fiercer"

"fiercest"

"fierier"

"fieriest"

"fierily"

"fieriness"

"fiery"

"fiesta"

"fiestas"

"fife"

"fifed"

"fifer"

"fifers"

"fifes"

"fifing"

"fifteen"

"fifteens"

"fifteenth"

"fifteenths"

"fifth"

"fifthly"

"fifths"

"fifties"

"fiftieth"

"fiftieths"

"fifty"

"fig"

"figeater"

"figeaters"

"figged"

"figging"

"fight"

"fighter"

"fighters"

"fighting"

"fightings"

"fights"

"figment"

"figments"

"figs"

"figurant"

"figurants"

"figurate"

"figuration"

"figurations"

"figurative"

"figuratively"

"figurativeness"

"figure"

"figured"

"figurehead"

"figureheads"

"figurer"

"figurers"

"figures"

"figurine"

"figurines"

"figuring"

"figurings"

"figwort"

"figworts"

"fiji"

"filagree"

"filagreed"

"filagrees"

"filament"

"filamentary"

"filamentous"

"filaments"

"filar"

"filaree"

"filarees"

"filbert"

"filberts"

"filch"

"filched"

"filcher"

"filchers"

"filches"

"filching"

"file"

"fileable"

"filed"

"filename"

"filenames"

"filer"

"filers"

"files"

"filespec"

"filet"

"fileted"

"fileting"

"filets"

"filial"

"filially"

"filiated"

"filiates"

"filibuster"

"filibustered"

"filibusterer"

"filibusterers"

"filibustering"

"filibusters"

"filicide"

"filicides"

"filigree"

"filigreed"

"filigreeing"

"filigrees"

"filii"

"filing"

"filings"

"filipino"

"filipinos"

"filisters"

"filius"

"fill"

"fillable"

"fille"

"filled"

"filler"

"fillers"

"filles"

"fillet"

"filleted"

"filleting"

"fillets"

"fillies"

"filling"

"fillings"

"fillip"

"filliped"

"filliping"

"fillips"

"fillmore"

"fills"

"filly"

"film"

"filmcards"

"filmdom"

"filmdoms"

"filmed"

"filmgoer"

"filmgoers"

"filmic"

"filmier"

"filmiest"

"filmily"

"filminess"

"filming"

"filmland"

"filmlands"

"filmographies"

"filmography"

"films"

"filmsets"

"filmstrip"

"filmstrips"

"filmy"

"filter"

"filterability"

"filterable"

"filtered"

"filterer"

"filterers"

"filtering"

"filters"

"filth"

"filthier"

"filthiest"

"filthily"

"filthiness"

"filths"

"filthy"

"filtrable"

"filtrate"

"filtrated"

"filtrates"

"filtrating"

"filtration"

"fin"

"finable"

"finagle"

"finagled"

"finagler"

"finaglers"

"finagles"

"finagling"

"final"

"finale"

"finales"

"finalis"

"finalism"

"finalisms"

"finalist"

"finalists"

"finalities"

"finality"

"finalization"

"finalizations"

"finalize"

"finalized"

"finalizes"

"finalizing"

"finally"

"finals"

"finance"

"financed"

"finances"

"financial"

"financial difficulties"

"financially"

"financier"

"financiers"

"financing"

"finbacks"

"finch"

"finches"

"find"

"Find sleep"

"findable"

"finder"

"finders"

"finding"

"findings"

"finds"

"fine"

"fineable"

"fined"

"finely"

"fineness"

"finer"

"fineries"

"finery"

"fines"

"finespun"

"finesse"

"finessed"

"finesses"

"finessing"

"finest"

"finfishes"

"finfoots"

"finger"

"fingerboard"

"fingerboards"

"fingered"

"fingerer"

"fingerers"

"fingering"

"fingerings"

"fingerling"

"fingerlings"

"fingernail"

"fingernails"

"fingerprint"

"fingerprinted"

"fingerprinting"

"fingerprints"

"fingers"

"fingertip"

"fingertips"

"finial"

"finialed"

"finials"

"finical"

"finickier"

"finickiest"

"finicky"

"fining"

"finings"

"finis"

"finises"

"finish"

"finished"

"finisher"

"finishers"

"finishes"

"finishing"

"finite"

"finitely"

"finiteness"

"finites"

"finitude"

"finitudes"

"fink"

"finked"

"finking"

"finks"

"finland"

"finless"

"finmark"

"finn"

"finnan"

"finned"

"finnickier"

"finnicky"

"finnier"

"finniest"

"finning"

"finnmark"

"finnmarks"

"finns"

"finny"

"finochio"

"finochios"

"fins"

"fiord"

"fir"

"fire"

"firearm"

"firearms"

"fireball"

"fireballs"

"firebase"

"firebases"

"firebird"

"firebirds"

"fireboat"

"fireboats"

"firebomb"

"firebombed"

"firebombing"

"firebombs"

"firebox"

"fireboxes"

"firebrand"

"firebrands"

"firebreak"

"firebreaks"

"firebrick"

"firebricks"

"firebug"

"firebugs"

"fireclays"

"firecracker"

"firecrackers"

"fired"

"firedamp"

"firedamps"

"firedog"

"firedogs"

"firefanged"

"fireflies"

"firefly"

"firehalls"

"firehouse"

"firehouses"

"fireless"

"firelight"

"fireman"

"firemen"

"firepan"

"firepans"

"fireplace"

"fireplaces"

"fireplug"

"fireplugs"

"firepower"

"fireproof"

"firer"

"firers"

"fires"

"fireside"

"firesides"

"firetrap"

"firetraps"

"firewater"

"fireweed"

"fireweeds"

"firewood"

"firewoods"

"firework"

"fireworks"

"fireworm"

"fireworms"

"firing"

"firings"

"firkin"

"firkins"

"firm"

"firma"

"firmament"

"firmed"

"firmer"

"firmers"

"firmest"

"firming"

"firmly"

"firmness"

"firms"

"firry"

"firs"

"first"

"First aid"

"first aid training"

"first cycle"

"firstborn"

"firsthand"

"firstling"

"firstlings"

"firstly"

"firsts"

"firth"

"firths"

"fiscal"

"fiscally"

"fiscals"

"fish"

"fishable"

"fishbone"

"fishbowl"

"fishbowls"

"fished"

"fisher"

"fisheries"

"fisherman"

"fishermen"

"fishers"

"fishery"

"fishes"

"fisheye"

"fisheyes"

"fishhook"

"fishhooks"

"fishier"

"fishiest"

"fishily"

"fishiness"

"fishing"

"fishings"

"fishless"

"fishline"

"fishlines"

"fishmeal"

"fishnet"

"fishnets"

"fishpole"

"fishpoles"

"fishpond"

"fishponds"

"fishskin"

"fishtail"

"fishtailed"

"fishtailing"

"fishtails"

"fishways"

"fishwife"

"fishwives"

"fishy"

"fissile"

"fissility"

"fission"

"fissionable"

"fissioned"

"fissioning"

"fissions"

"fissure"

"fissured"

"fissures"

"fissuring"

"fist"

"fisted"

"fistful"

"fistfuls"

"fistic"

"fisticuff"

"fisticuffs"

"fisting"

"fists"

"fistula"

"fistulae"

"fistular"

"fistulas"

"fistulous"

"fit"

"fitchews"

"fitful"

"fitfully"

"fitfulness"

"fitly"

"fitments"

"fitness"

"fitness activity"

"fitnesses"

"fits"

"fittable"

"fitted"

"fitter"

"fitters"

"fittest"

"fitting"

"fittingly"

"fittingness"

"fittings"

"five"

"fivefold"

"fivepins"

"fiver"

"fivers"

"fives"

"fix"

"fixable"

"fixate"

"fixated"

"fixates"

"fixating"

"fixation"

"fixations"

"fixative"

"fixatives"

"fixe"

"fixed"

"fixedly"

"fixedness"

"fixer"

"fixers"

"fixes"

"fixing"

"fixings"

"fixities"

"fixity"

"fixture"

"fixtures"

"fixup"

"fixups"

"fixures"

"fizgig"

"fizgigs"

"fizz"

"fizzed"

"fizzer"

"fizzers"

"fizzes"

"fizzier"

"fizziest"

"fizzing"

"fizzle"

"fizzled"

"fizzles"

"fizzling"

"fizzy"

"fjord"

"fjords"

"flab"

"flabbergast"

"flabbergasted"

"flabbergasting"

"flabbergasts"

"flabbier"

"flabbiest"

"flabbily"

"flabbiness"

"flabby"

"flabs"

"flaccid"

"flaccidities"

"flaccidity"

"flack"

"flacks"

"flacon"

"flacons"

"flag"

"flagella"

"flagellant"

"flagellants"

"flagellate"

"flagellated"

"flagellates"

"flagellating"

"flagellation"

"flagellations"

"flagellator"

"flagellators"

"flagellomania"

"flagellum"

"flagellums"

"flageolet"

"flageolets"

"flagged"

"flagger"

"flaggers"

"flaggier"

"flaggiest"

"flagging"

"flaggings"

"flaggy"

"flagitious"

"flagless"

"flagman"

"flagmen"

"flagon"

"flagons"

"flagpole"

"flagpoles"

"flagrance"

"flagrancy"

"flagrant"

"flagrante"

"flagrantly"

"flags"

"flagship"

"flagships"

"flagstaff"

"flagstaffs"

"flagstone"

"flagstones"

"flail"

"flailed"

"flailing"

"flails"

"flair"

"flairs"

"flak"

"flake"

"flaked"

"flaker"

"flakers"

"flakes"

"flakier"

"flakiest"

"flakily"

"flakiness"

"flaking"

"flaky"

"flambe"

"flambeau"

"flambeaus"

"flambeaux"

"flambee"

"flambeed"

"flambeing"

"flambes"

"flamboyance"

"flamboyancy"

"flamboyant"

"flamboyantly"

"flame"

"flamed"

"flamenco"

"flamencos"

"flameout"

"flameouts"

"flameproof"

"flamer"

"flamers"

"flames"

"flamethrower"

"flamethrowers"

"flamier"

"flamines"

"flaming"

"flamingly"

"flamingo"

"flamingoes"

"flamingos"

"flammability"

"flammable"

"flammably"

"flammed"

"flamming"

"flams"

"flamy"

"flan"

"flanders"

"flange"

"flanged"

"flanger"

"flangers"

"flanges"

"flanging"

"flank"

"flanked"

"flanker"

"flankers"

"flanking"

"flanks"

"flannel"

"flanneled"

"flannelet"

"flanneling"

"flannelled"

"flannelly"

"flannels"

"flans"

"flap"

"flapjack"

"flapjacks"

"flapless"

"flappable"

"flapped"

"flapper"

"flappers"

"flappier"

"flappiest"

"flapping"

"flappy"

"flaps"

"flare"

"flared"

"flares"

"flaring"

"flash"

"flashback"

"flashbacks"

"flashbulb"

"flashbulbs"

"flashcard"

"flashcube"

"flashcubes"

"flashed"

"flasher"

"flashers"

"flashes"

"flashflood"

"flashforward"

"flashforwards"

"flashgun"

"flashguns"

"flashier"

"flashiest"

"flashily"

"flashiness"

"flashing"

"flashings"

"flashlamp"

"flashlamps"

"flashlight"

"flashlights"

"flashtube"

"flashtubes"

"flashy"

"flask"

"flasks"

"flat"

"flatbed"

"flatbeds"

"flatboat"

"flatboats"

"flatcar"

"flatcars"

"flatfeet"

"flatfish"

"flatfishes"

"flatfoot"

"flatfooted"

"flatfoots"

"flathead"

"flatheads"

"flatiron"

"flatirons"

"flatland"

"flatlands"

"flatly"

"flatness"

"flats"

"flatted"

"flatten"

"flattened"

"flattener"

"flatteners"

"flattening"

"flattens"

"flatter"

"flattered"

"flatterer"

"flatterers"

"flatteries"

"flattering"

"flatteringly"

"flatters"

"flattery"

"flattest"

"flatting"

"flattish"

"flattop"

"flattops"

"flatulence"

"flatulences"

"flatulencies"

"flatulency"

"flatulent"

"flatulently"

"flatus"

"flatuses"

"flatware"

"flatwares"

"flatways"

"flatwise"

"flatwork"

"flatworks"

"flatworm"

"flatworms"

"flaunt"

"flaunted"

"flaunter"

"flaunters"

"flauntier"

"flauntiest"

"flaunting"

"flauntingly"

"flaunts"

"flaunty"

"flautist"

"flautists"

"flavedos"

"flavonoid"

"flavonol"

"flavonols"

"flavor"

"flavored"

"flavorer"

"flavorers"

"flavorful"

"flavorfully"

"flavoring"

"flavorings"

"flavorless"

"flavors"

"flavorsome"

"flavory"

"flavour"

"flavoured"

"flavouring"

"flavours"

"flavoury"

"flaw"

"flawed"

"flawier"

"flawing"

"flawless"

"flawlessly"

"flawlessness"

"flaws"

"flawy"

"flax"

"flaxen"

"flaxes"

"flaxier"

"flaxseed"

"flaxseeds"

"flaxy"

"flay"

"flayed"

"flayer"

"flayers"

"flaying"

"flays"

"flea"

"fleabag"

"fleabags"

"fleabane"

"fleabanes"

"fleabite"

"fleabites"

"fleabitten"

"fleas"

"fleawort"

"fleche"

"fleches"

"fleck"

"flecked"

"flecking"

"flecks"

"flecky"

"fled"

"fledge"

"fledged"

"fledges"

"fledgier"

"fledging"

"fledgling"

"fledglings"

"fledgy"

"flee"

"fleece"

"fleeced"

"fleecer"

"fleecers"

"fleeces"

"fleecier"

"fleeciest"

"fleecily"

"fleeciness"

"fleecing"

"fleecy"

"fleeing"

"fleer"

"fleered"

"fleering"

"fleers"

"flees"

"fleet"

"fleeted"

"fleeter"

"fleetest"

"fleeting"

"fleetingly"

"fleetingness"

"fleetly"

"fleetness"

"fleets"

"fleming"

"flemings"

"flemish"

"flemished"

"flemishes"

"flenched"

"flenches"

"flenching"

"flense"

"flensed"

"flenser"

"flensers"

"flenses"

"flensing"

"flesh"

"fleshed"

"flesher"

"fleshers"

"fleshes"

"fleshier"

"fleshiest"

"fleshiness"

"fleshing"

"fleshings"

"fleshlier"

"fleshliest"

"fleshly"

"fleshpot"

"fleshpots"

"fleshy"

"fletch"

"fletched"

"fletcher"

"fletchers"

"fletches"

"fletching"

"fleury"

"flew"

"flews"

"flex"

"flexed"

"flexes"

"flexibility"

"flexible"

"flexibly"

"flexile"

"flexing"

"flexion"

"flexions"

"flexitime"

"flexor"

"flexors"

"flexure"

"flexures"

"fleyed"

"flibbertigibbet"

"flibbertigibbets"

"flick"

"flicked"

"flicker"

"flickered"

"flickering"

"flickers"

"flickery"

"flicking"

"flicks"

"flied"

"flier"

"fliers"

"flies"

"fliest"

"flight"

"Flight Mode"

"flighted"

"flightier"

"flightiest"

"flightiness"

"flighting"

"flightless"

"flights"

"flighty"

"flimflam"

"flimflammer"

"flimflams"

"flimsier"

"flimsies"

"flimsiest"

"flimsily"

"flimsiness"

"flimsy"

"flinch"

"flinched"

"flincher"

"flinchers"

"flinches"

"flinching"

"flinchingly"

"flinder"

"flinders"

"fling"

"flinger"

"flingers"

"flinging"

"flings"

"flint"

"flinted"

"flintier"

"flintiest"

"flintily"

"flinting"

"flintlike"

"flintlock"

"flintlocks"

"flints"

"flinty"

"flip"

"flippancies"

"flippancy"

"flippant"

"flippantly"

"flipped"

"flipper"

"flippers"

"flippest"

"flipping"

"flips"

"flirt"

"flirtation"

"flirtations"

"flirtatious"

"flirtatiously"

"flirtatiousness"

"flirted"

"flirter"

"flirters"

"flirtier"

"flirtiest"

"flirting"

"flirtingly"

"flirts"

"flirty"

"flit"

"flitch"

"flitched"

"flitches"

"flitching"

"flite"

"flites"

"flits"

"flitted"

"flitter"

"flittered"

"flittering"

"flitters"

"flitting"

"flivver"

"flivvers"

"float"

"floatability"

"floatable"

"floatage"

"floatages"

"floatation"

"floated"

"floater"

"floaters"

"floatier"

"floatiest"

"floating"

"floats"

"floaty"

"floccular"

"floccules"

"flocculus"

"flock"

"flocked"

"flockier"

"flockiest"

"flocking"

"flockings"

"flocks"

"flocky"

"floe"

"floes"

"flog"

"flogged"

"flogger"

"floggers"

"flogging"

"floggings"

"flogs"

"flood"

"flooded"

"flooder"

"flooders"

"floodgate"

"floodgates"

"flooding"

"floodlight"

"floodlighted"

"floodlighting"

"floodlights"

"floodlit"

"floodplain"

"floods"

"floodwater"

"floodway"

"floodways"

"flooey"

"floor"

"floorages"

"floorboard"

"floorboards"

"floored"

"floorer"

"floorers"

"flooring"

"floorings"

"floors"

"floorshift"

"floorshifts"

"floorshow"

"floorthrough"

"floorwalker"

"floorwalkers"

"floosies"

"floozie"

"floozies"

"floozy"

"flop"

"flophouse"

"flophouses"

"flopover"

"flopovers"

"flopped"

"flopper"

"floppers"

"floppier"

"floppies"

"floppiest"

"floppily"

"flopping"

"floppy"

"flops"

"flora"

"florae"

"floral"

"florally"

"floras"

"florence"

"florences"

"florentine"

"florentines"

"florescence"

"florescent"

"floret"

"florets"

"florid"

"florida"

"floridan"

"floridans"

"floridian"

"floridians"

"floridly"

"florimania"

"florin"

"florins"

"floriscope"

"florist"

"floristry"

"florists"

"floss"

"flossed"

"flosses"

"flossie"

"flossier"

"flossies"

"flossiest"

"flossing"

"flossy"

"flotation"

"flotations"

"flotilla"

"flotillas"

"flotsam"

"flotsams"

"flounce"

"flounced"

"flounces"

"flouncier"

"flounciest"

"flouncing"

"flouncy"

"flounder"

"floundered"

"floundering"

"flounderingly"

"flounders"

"flour"

"floured"

"flouring"

"flourish"

"flourished"

"flourishes"

"flourishing"

"flourishingly"

"flours"

"floury"

"flout"

"flouted"

"flouter"

"flouters"

"flouting"

"flouts"

"flow"

"flowage"

"flowages"

"flowchart"

"flowcharted"

"flowcharting"

"flowcharts"

"flowed"

"flower"

"flowered"

"flowerer"

"flowerers"

"floweret"

"flowerets"

"flowerier"

"floweriest"

"floweriness"

"flowering"

"flowerless"

"flowerpot"

"flowerpots"

"flowers"

"flowery"

"flowing"

"flowingly"

"flowmeter"

"flown"

"flows"

"flu"

"flub"

"flubbed"

"flubbing"

"flubdubs"

"flubs"

"fluctuate"

"fluctuated"

"fluctuates"

"fluctuating"

"fluctuation"

"fluctuational"

"fluctuations"

"flue"

"flued"

"fluencies"

"fluency"

"fluent"

"fluently"

"flues"

"fluff"

"fluffed"

"fluffier"

"fluffiest"

"fluffily"

"fluffiness"

"fluffing"

"fluffs"

"fluffy"

"fluid"

"fluid infusion"

"fluidal"

"fluidic"

"fluidics"

"fluidities"

"fluidity"

"fluidize"

"fluidized"

"fluidizes"

"fluidizing"

"fluidly"

"fluidness"

"fluidrams"

"fluids"

"fluke"

"fluked"

"flukes"

"flukey"

"flukier"

"flukiest"

"fluking"

"fluky"

"flume"

"flumed"

"flumes"

"fluming"

"flummeries"

"flummery"

"flummox"

"flummoxed"

"flummoxes"

"flummoxing"

"flump"

"flumped"

"flung"

"flunk"

"flunked"

"flunker"

"flunkers"

"flunkey"

"flunkeys"

"flunkies"

"flunking"

"flunks"

"flunky"

"fluor"

"fluorenes"

"fluoresce"

"fluoresced"

"fluorescence"

"fluorescent"

"fluoresces"

"fluorescing"

"fluoridate"

"fluoridated"

"fluoridates"

"fluoridating"

"fluoridation"

"fluoridations"

"fluoride"

"fluorides"

"fluorimeter"

"fluorinate"

"fluorinated"

"fluorinates"

"fluorinating"

"fluorination"

"fluorinations"

"fluorine"

"fluorines"

"fluorite"

"fluorites"

"fluorocarbon"

"fluorocarbons"

"fluorophosphate"

"fluoroscope"

"fluoroscopes"

"fluoroscopic"

"fluoroscopically"

"fluoroscopies"

"fluoroscopist"

"fluoroscopists"

"fluoroscopy"

"fluorosis"

"fluors"

"flurried"

"flurries"

"flurry"

"flurrying"

"flus"

"flush"

"flushable"

"flushed"

"flusher"

"flushers"

"flushes"

"flushest"

"flushing"

"flushness"

"fluster"

"flustered"

"flustering"

"flusters"

"flute"

"fluted"

"fluter"

"fluters"

"flutes"

"flutier"

"flutiest"

"fluting"

"flutings"

"flutist"

"flutists"

"flutter"

"fluttered"

"flutterer"

"flutterers"

"fluttering"

"flutters"

"fluttery"

"fluty"

"fluviology"

"flux"

"fluxed"

"fluxes"

"fluxing"

"fluxions"

"fly"

"flyable"

"flyaway"

"flyaways"

"flyblown"

"flyblows"

"flyby"

"flybys"

"flycatcher"

"flycatchers"

"flyer"

"flyers"

"flying"

"flyings"

"flyleaf"

"flyleaves"

"flyman"

"flymen"

"flyover"

"flyovers"

"flypaper"

"flypapers"

"flyspeck"

"flyspecked"

"flyspecks"

"flytrap"

"flytraps"

"flyway"

"flyways"

"flyweight"

"flyweights"

"flywheel"

"flywheels"

"foal"

"foaled"

"foaling"

"foals"

"foam"

"foamed"

"foamer"

"foamers"

"foamier"

"foamiest"

"foamily"

"foaminess"

"foaming"

"foamless"

"foams"

"foamy"

"fob"

"fobbed"

"fobbing"

"fobs"

"focal"

"focalised"

"focalises"

"focalize"

"focalized"

"focalizes"

"focalizing"

"focally"

"foci"

"focimeter"

"focus"

"focused"

"focuser"

"focusers"

"focuses"

"focusing"

"focussed"

"focusses"

"focussing"

"fodder"

"foddered"

"foddering"

"fodders"

"foe"

"foehn"

"foehns"

"foeman"

"foemen"

"foes"

"foetal"

"foeti"

"foetid"

"foetor"

"foetors"

"foetus"

"foetuses"

"fog"

"fogbound"

"fogey"

"fogeys"

"foggages"

"fogged"

"fogger"

"foggers"

"foggier"

"foggiest"

"foggily"

"fogginess"

"fogging"

"foggy"

"foghorn"

"foghorns"

"fogie"

"fogies"

"fogless"

"fogs"

"fogy"

"fogyish"

"fogyism"

"fogyisms"

"foible"

"foibles"

"foil"

"foilable"

"foiled"

"foiling"

"foils"

"foilsman"

"foilsmen"

"foins"

"foist"

"foisted"

"foisting"

"foists"

"fold"

"foldable"

"foldage"

"foldaway"

"foldboat"

"foldboats"

"folded"

"folder"

"folderol"

"folderols"

"folders"

"folding"

"foldout"

"foldouts"

"folds"

"folia"

"foliage"

"foliaged"

"foliages"

"foliar"

"foliate"

"foliated"

"foliates"

"foliating"

"foliation"

"folic"

"folio"

"folioed"

"folioing"

"folios"

"folk"

"folkish"

"folklore"

"folklores"

"folkloric"

"folklorist"

"folkloristics"

"folklorists"

"folkmoots"

"folkmotes"

"folks"

"folksier"

"folksiest"

"folksily"

"folksongs"

"folksy"

"folktale"

"folktales"

"folkway"

"folkways"

"follicle"

"follicles"

"follicular"

"follies"

"follow"

"followed"

"follower"

"followers"

"followeth"

"following"

"followings"

"follows"

"followup"

"folly"

"foment"

"fomentation"

"fomentations"

"fomented"

"fomenter"

"fomenters"

"fomenting"

"foments"

"fond"

"fondant"

"fondants"

"fonded"

"fonder"

"fondest"

"fonding"

"fondle"

"fondled"

"fondler"

"fondlers"

"fondles"

"fondling"

"fondlings"

"fondly"

"fondness"

"fonds"

"fondu"

"fondue"

"fondues"

"font"

"fontal"

"fontanelle"

"fontanels"

"fontina"

"fontinas"

"fonts"

"food"

"Food and Drug Administration"

"food database"

"food items"

"foodless"

"foods"

"foodservices"

"foodstuff"

"foodstuffs"

"foofaraw"

"foofaraws"

"fool"

"fooled"

"fooleries"

"foolery"

"foolfish"

"foolhardier"

"foolhardiest"

"foolhardily"

"foolhardiness"

"foolhardy"

"fooling"

"foolish"

"foolisher"

"foolishest"

"foolishly"

"foolishness"

"foolproof"

"fools"

"foolscap"

"foolscaps"

"foot"

"footage"

"footages"

"football"

"footballs"

"footbath"

"footbaths"

"footboard"

"footboards"

"footboy"

"footbridge"

"footbridges"

"footed"

"footer"

"footers"

"footfall"

"footfalls"

"footgear"

"footgears"

"foothill"

"foothills"

"foothold"

"footholds"

"footier"

"footing"

"footings"

"footlambert"

"footless"

"footlessness"

"footlight"

"footlights"

"footlocker"

"footlockers"

"footloose"

"footman"

"footmark"

"footmarks"

"footmen"

"footnote"

"footnoted"

"footnotes"

"footnoting"

"footpace"

"footpaces"

"footpad"

"footpads"

"footpath"

"footpaths"

"footprint"

"footprints"

"footrace"

"footraces"

"footrest"

"footrests"

"footrope"

"footropes"

"foots"

"footsie"

"footsies"

"footslog"

"footslogs"

"footsore"

"footsoreness"

"footstep"

"footsteps"

"footstool"

"footstools"

"footway"

"footways"

"footwear"

"footwears"

"footwork"

"footworks"

"footworn"

"footy"

"foozle"

"foozlers"

"foozling"

"fop"

"fopped"

"fopperies"

"foppery"

"fopping"

"foppish"

"fops"

"for"

"for prescriptions"

"fora"

"forage"

"foraged"

"forager"

"foragers"

"forages"

"foraging"

"foramen"

"foramina"

"forasmuch"

"foray"

"forayed"

"forayer"

"forayers"

"foraying"

"forays"

"forbad"

"forbade"

"forbear"

"forbearance"

"forbearer"

"forbearers"

"forbearing"

"forbearingly"

"forbears"

"forbid"

"forbidals"

"forbiddance"

"forbidden"

"forbidder"

"forbidding"

"forbiddingly"

"forbids"

"forbode"

"forboded"

"forbodes"

"forboding"

"forbore"

"forborne"

"force"

"forced"

"forcedly"

"forceful"

"forcefully"

"forcefulness"

"forceless"

"forceps"

"forcer"

"forcers"

"forces"

"forcible"

"forcibleness"

"forcibly"

"forcing"

"ford"

"fordable"

"forded"

"fordid"

"fording"

"fordless"

"fords"

"fore"

"forearm"

"forearmed"

"forearming"

"forearms"

"forebay"

"forebear"

"forebearing"

"forebears"

"forebode"

"foreboded"

"foreboder"

"forebodes"

"forebodies"

"foreboding"

"forebodings"

"forebrain"

"foreby"

"forebye"

"forecast"

"forecasted"

"forecaster"

"forecasters"

"forecasting"

"forecastle"

"forecastles"

"forecasts"

"foreclose"

"foreclosed"

"forecloses"

"foreclosing"

"foreclosure"

"foreclosures"

"foreconscious"

"forecourt"

"foredate"

"foredated"

"foredates"

"foredating"

"foredeck"

"foredecks"

"foredid"

"foredo"

"foredoing"

"foredoom"

"foredoomed"

"foredooming"

"foredooms"

"forefather"

"forefathers"

"forefeels"

"forefeet"

"forefend"

"forefended"

"forefends"

"forefinger"

"forefingers"

"forefoot"

"forefront"

"foregather"

"forego"

"foregoer"

"foregoers"

"foregoes"

"foregoing"

"foregone"

"foreground"

"foregrounds"

"foregut"

"forehand"

"forehanded"

"forehandedly"

"forehandedness"

"forehands"

"forehead"

"foreheads"

"forehoof"

"forehoofs"

"forehooves"

"foreign"

"foreigner"

"foreigners"

"foreignness"

"forejudge"

"forejudger"

"forejudgment"

"foreknew"

"foreknow"

"foreknowing"

"foreknowledge"

"foreknown"

"foreknows"

"foreladies"

"forelady"

"foreland"

"forelands"

"foreleg"

"forelegs"

"forelimb"

"forelimbs"

"forelock"

"forelocks"

"foreman"

"foremanship"

"foremast"

"foremasts"

"foremen"

"foremost"

"foremother"

"forename"

"forenamed"

"forenames"

"forenoon"

"forenoons"

"forensic"

"forensically"

"forensics"

"foreordain"

"foreordained"

"foreordaining"

"foreordainment"

"foreordainments"

"foreordains"

"foreordination"

"forepart"

"foreparts"

"forepaw"

"forepaws"

"forepeaks"

"foreplay"

"foreplays"

"forepleasure"

"forequarter"

"forequarters"

"foreran"

"forerun"

"forerunner"

"forerunners"

"foreruns"

"fores"

"foresaid"

"foresail"

"foresails"

"foresaw"

"foresee"

"foreseeability"

"foreseeable"

"foreseeing"

"foreseen"

"foreseer"

"foreseers"

"foresees"

"foreshadow"

"foreshadowed"

"foreshadower"

"foreshadowing"

"foreshadows"

"foresheet"

"foresheets"

"foreshore"

"foreshorten"

"foreshortened"

"foreshortening"

"foreshortens"

"foreshowed"

"foreshown"

"foreshows"

"foreside"

"foresides"

"foresight"

"foresighted"

"foresightedly"

"foresightedness"

"foreskin"

"foreskins"

"forest"

"forestall"

"forestalled"

"forestaller"

"forestalling"

"forestalls"

"forestation"

"forestay"

"forestays"

"forested"

"forester"

"foresters"

"forestery"

"foresting"

"forestry"

"forests"

"foreswear"

"foreswearing"

"foreswore"

"foresworn"

"foretaste"

"foretasted"

"foretastes"

"foretasting"

"foretell"

"foreteller"

"foretellers"

"foretelling"

"foretells"

"forethought"

"forethoughtful"

"foretime"

"foretimes"

"foretoken"

"foretokened"

"foretokening"

"foretokens"

"foretold"

"foretop"

"foretops"

"forever"

"forevermore"

"forevers"

"forewarn"

"forewarned"

"forewarning"

"forewarns"

"forewent"

"forewing"

"forewings"

"forewoman"

"forewomen"

"foreword"

"forewords"

"foreworn"

"foreyard"

"forfeit"

"forfeitable"

"forfeitableness"

"forfeited"

"forfeiting"

"forfeits"

"forfeiture"

"forfeitures"

"forfend"

"forfended"

"forfending"

"forfends"

"forgather"

"forgathered"

"forgathering"

"forgathers"

"forgave"

"forge"

"forged"

"forger"

"forgeries"

"forgers"

"forgery"

"forges"

"forget"

"forgetful"

"forgetfully"

"forgetfulness"

"forgets"

"forgettable"

"forgetting"

"forging"

"forgings"

"forgivable"

"forgive"

"forgiven"

"forgiveness"

"forgiver"

"forgivers"

"forgives"

"forgiving"

"forgo"

"forgoer"

"forgoers"

"forgoes"

"forgoing"

"forgone"

"forgot"

"forgotten"

"forint"

"forints"

"forjudge"

"forjudged"

"forjudger"

"forjudges"

"forjudging"

"fork"

"forked"

"forkedly"

"forker"

"forkers"

"forkful"

"forkfuls"

"forkier"

"forking"

"forkless"

"forklift"

"forklifts"

"forklike"

"forks"

"forksful"

"forky"

"forlorn"

"forlorner"

"forlornest"

"forlornly"

"form"

"forma"

"formable"

"formal"

"formaldehyde"

"formalin"

"formalins"

"formalism"

"formalist"

"formalistic"

"formalistically"

"formalities"

"formality"

"formalization"

"formalize"

"formalized"

"formalizer"

"formalizes"

"formalizing"

"formally"

"formals"

"formant"

"format"

"formated"

"formating"

"formation"

"formations"

"formative"

"formats"

"formatted"

"formatter"

"formatters"

"formatting"

"formed"

"former"

"formerly"

"formers"

"formfeed"

"formfeeds"

"formfitting"

"formful"

"formic"

"formica"

"formidable"

"formidably"

"forming"

"formless"

"formlessly"

"formlessness"

"forms"

"formula"

"formulae"

"formulary"

"formulas"

"formulate"

"formulated"

"formulates"

"formulating"

"formulation"

"formulations"

"formulator"

"formulators"

"fornicate"

"fornicated"

"fornicates"

"fornicating"

"fornication"

"fornications"

"fornicator"

"fornicators"

"fornicatrices"

"fornicatrix"

"forsake"

"forsaken"

"forsaker"

"forsakers"

"forsakes"

"forsaking"

"forsee"

"forseeable"

"forseen"

"forsook"

"forsooth"

"forspent"

"forswear"

"forswearing"

"forswears"

"forswore"

"forsworn"

"forsythia"

"forsythias"

"fort"

"forte"

"fortes"

"forth"

"forthcoming"

"forthright"

"forthrightly"

"forthrightness"

"forthwith"

"forties"

"fortieth"

"fortieths"

"fortification"

"fortifications"

"fortified"

"fortifier"

"fortifiers"

"fortifies"

"fortify"

"fortifying"

"fortiori"

"fortis"

"fortissimo"

"fortitude"

"fortnight"

"fortnightly"

"fortnights"

"fortran"

"fortress"

"fortressed"

"fortresses"

"forts"

"fortuities"

"fortuitous"

"fortuitously"

"fortuitus"

"fortuity"

"fortunate"

"fortunately"

"fortunateness"

"fortune"

"fortuned"

"fortunes"

"fortuneteller"

"fortunetellers"

"fortunetelling"

"fortuning"

"forty"

"fortyfive"

"fortyfives"

"forum"

"forums"

"forward"

"forwarded"

"forwarder"

"forwarders"

"forwardest"

"forwarding"

"forwardly"

"forwardness"

"forwards"

"forwardsearch"

"forwent"

"forwhy"

"forworn"

"forzando"

"forzandos"

"fossa"

"fossae"

"fossate"

"fosse"

"fosses"

"fossil"

"fossilization"

"fossilize"

"fossilized"

"fossilizes"

"fossilizing"

"fossillike"

"fossils"

"foster"

"fosterage"

"fostered"

"fosterer"

"fosterers"

"fostering"

"fosterling"

"fosterlings"

"fosters"

"fought"

"foul"

"foulard"

"foulards"

"fouled"

"fouler"

"foulest"

"fouling"

"foulings"

"foully"

"foulmouthed"

"foulness"

"fouls"

"found"

"foundation"

"foundational"

"foundations"

"founded"

"founder"

"foundered"

"foundering"

"founders"

"founding"

"foundling"

"foundlings"

"foundress"

"foundries"

"foundry"

"founds"

"fount"

"fountain"

"fountained"

"fountainhead"

"fountainheads"

"fountains"

"founts"

"four"

"fourflusher"

"fourflushers"

"fourfold"

"fourpenny"

"fourposter"

"fourposters"

"fours"

"fourscore"

"foursome"

"foursomes"

"foursquare"

"fourteen"

"fourteens"

"fourteenth"

"fourteenths"

"fourth"

"fourthly"

"fourths"

"fovea"

"foveae"

"foveal"

"foveate"

"fowl"

"fowled"

"fowler"

"fowlers"

"fowling"

"fowlings"

"fowlpox"

"fowls"

"fox"

"foxed"

"foxes"

"foxfire"

"foxfires"

"foxfish"

"foxglove"

"foxgloves"

"foxhole"

"foxholes"

"foxhound"

"foxhounds"

"foxier"

"foxiest"

"foxily"

"foxiness"

"foxing"

"foxings"

"foxskin"

"foxskins"

"foxtail"

"foxtails"

"foxtrot"

"foxy"

"foyer"

"foyers"

"fps"

"frabjous"

"fracas"

"fracases"

"fraction"

"fractional"

"fractionalize"

"fractionalized"

"fractionalizing"

"fractionally"

"fractioned"

"fractions"

"fractious"

"fractiously"

"fracture"

"fractured"

"fractures"

"fracturing"

"frag"

"fragged"

"fragging"

"fraggings"

"fragile"

"fragileness"

"fragilities"

"fragility"

"fragment"

"fragmental"

"fragmentally"

"fragmentarily"

"fragmentariness"

"fragmentary"

"fragmentate"

"fragmentation"

"fragmented"

"fragmenting"

"fragments"

"fragrance"

"fragrances"

"fragrancy"

"fragrant"

"fragrantly"

"frags"

"frail"

"frailer"

"frailest"

"frailly"

"frailness"

"frails"

"frailties"

"frailty"

"framable"

"frambesia"

"frame"

"framed"

"framer"

"framers"

"frames"

"framework"

"frameworks"

"framing"

"franc"

"franca"

"francas"

"france"

"frances"

"franchise"

"franchised"

"franchisee"

"franchisees"

"franchiser"

"franchisers"

"franchises"

"franchising"

"francis"

"franciscan"

"franciscans"

"francisco"

"francium"

"franciums"

"franco"

"francomania"

"Francophobia"

"francs"

"frangibility"

"frangible"

"frank"

"franked"

"frankenstein"

"frankensteins"

"franker"

"frankers"

"frankest"

"frankfort"

"frankfurt"

"frankfurter"

"frankfurters"

"frankincense"

"franking"

"franklin"

"franklins"

"frankly"

"frankness"

"franks"

"frantic"

"frantically"

"franz"

"frappe"

"frapped"

"frappes"

"frapping"

"fraps"

"frat"

"frater"

"fraternal"

"fraternalism"

"fraternally"

"fraternities"

"fraternity"

"fraternization"

"fraternize"

"fraternized"

"fraternizer"

"fraternizes"

"fraternizing"

"fratriage"

"fratricidal"

"fratricide"

"fratricides"

"frats"

"frau"

"fraud"

"frauds"

"fraudulence"

"fraudulent"

"fraudulently"

"fraudulentness"

"frauen"

"fraught"

"fraughted"

"fraughts"

"fraulein"

"frauleins"

"fraus"

"fray"

"frayed"

"fraying"

"frayings"

"frays"

"frazzle"

"frazzled"

"frazzles"

"frazzling"

"freak"

"freaked"

"freakier"

"freakiest"

"freakily"

"freaking"

"freakish"

"freakishly"

"freakishness"

"freakout"

"freakouts"

"freaks"

"freaky"

"freckle"

"freckled"

"freckles"

"frecklier"

"freckliest"

"freckling"

"freckly"

"fred"

"frederick"

"free"

"free app"

"free of charge"

"free trial"

"free version"

"freebee"

"freebees"

"freebie"

"freebies"

"freeboard"

"freeboot"

"freebooted"

"freebooter"

"freebooters"

"freeboots"

"freeborn"

"freed"

"freedman"

"freedmen"

"freedom"

"freedoms"

"freeform"

"freehand"

"freehanded"

"freehandedly"

"freehearted"

"freeheartedly"

"freehold"

"freeholder"

"freeholders"

"freeholds"

"freeing"

"freeing your endorphin"

"freelance"

"freelanced"

"freelances"

"freelancing"

"freeload"

"freeloaded"

"freeloader"

"freeloaders"

"freeloading"

"freeloads"

"freely"

"freeman"

"freemason"

"freemasonry"

"freemasons"

"freemen"

"freeness"

"freeport"

"freer"

"frees"

"freest"

"freestanding"

"freestone"

"freestones"

"freethinker"

"freethinkers"

"freethinking"

"freeway"

"freeways"

"freewheel"

"freewheelers"

"freewheeling"

"freewill"

"freezable"

"freeze"

"freezed"

"freezer"

"freezers"

"freezes"

"freezing"

"freight"

"freightage"

"freighted"

"freighter"

"freighters"

"freighting"

"freights"

"freightyard"

"french"

"frenched"

"frenches"

"frenching"

"frenchman"

"frenchmen"

"frenchwoman"

"frenchwomen"

"frenetic"

"frenetically"

"frenetics"

"frenum"

"frenzied"

"frenzies"

"frenzily"

"frenzy"

"frenzying"

"freon"

"frequencies"

"frequency"

"frequent"

"frequent urination"

"frequentation"

"frequented"

"frequenter"

"frequenters"

"frequenting"

"frequently"

"frequentness"

"frequents"

"frere"

"freres"

"fresco"

"frescoed"

"frescoer"

"frescoers"

"frescoes"

"frescoing"

"frescoist"

"frescoists"

"frescos"

"fresh"

"freshed"

"freshen"

"freshened"

"freshener"

"fresheners"

"freshening"

"freshens"

"fresher"

"freshes"

"freshest"

"freshet"

"freshets"

"freshing"

"freshly"

"freshman"

"freshmen"

"freshness"

"freshwater"

"fresnel"

"fresnels"

"fresno"

"fret"

"fretful"

"fretfully"

"fretfulness"

"fretless"

"frets"

"fretsaw"

"fretsaws"

"fretsome"

"fretted"

"fretter"

"fretters"

"frettier"

"frettiest"

"fretting"

"fretwork"

"fretworks"

"freud"

"freudian"

"freudianism"

"freudians"

"friability"

"friable"

"friableness"

"friar"

"friaries"

"friarly"

"friars"

"friary"

"frican"

"fricanmerican"

"fricassee"

"fricasseed"

"fricasseeing"

"fricassees"

"fricative"

"friction"

"frictional"

"frictionless"

"frictions"

"friday"

"fridays"

"fridge"

"fridges"

"fried"

"friedman"

"friend"

"friended"

"friending"

"friendless"

"friendlessness"

"friendlier"

"friendlies"

"friendliest"

"friendliness"

"friendly"

"friends"

"friendship"

"friendships"

"frier"

"friers"

"fries"

"frieze"

"friezes"

"frig"

"frigate"

"frigates"

"frigged"

"frigging"

"fright"

"frighted"

"frighten"

"frightened"

"frightening"

"frighteningly"

"frightens"

"frightful"

"frightfully"

"frightfulness"

"frighting"

"frights"

"frigid"

"frigidities"

"frigidity"

"frigidly"

"frigs"

"frijole"

"frijoles"

"frill"

"frilled"

"friller"

"frillers"

"frillier"

"frilliest"

"frilliness"

"frilling"

"frillings"

"frills"

"frilly"

"fringe"

"fringed"

"fringeless"

"fringelike"

"fringes"

"fringier"

"fringiest"

"fringing"

"fringy"

"fripperies"

"frippery"

"frisbee"

"frisbees"

"frisian"

"frisk"

"frisked"

"frisker"

"friskers"

"friskets"

"friskier"

"friskiest"

"friskily"

"friskiness"

"frisking"

"frisks"

"frisky"

"frisson"

"frissons"

"fritted"

"fritter"

"frittered"

"fritterer"

"fritterers"

"frittering"

"fritters"

"fritting"

"frivol"

"frivoled"

"frivoler"

"frivolers"

"frivoling"

"frivolities"

"frivolity"

"frivolled"

"frivolling"

"frivolous"

"frivolously"

"friz"

"frizettes"

"frizz"

"frizzed"

"frizzer"

"frizzers"

"frizzes"

"frizzier"

"frizziest"

"frizzily"

"frizziness"

"frizzing"

"frizzle"

"frizzled"

"frizzler"

"frizzlers"

"frizzles"

"frizzlier"

"frizzliest"

"frizzling"

"frizzly"

"frizzy"

"fro"

"frock"

"frocked"

"frocking"

"frocks"

"froes"

"frog"

"frogeye"

"frogeyed"

"frogeyes"

"frogfishes"

"frogged"

"froggier"

"froggiest"

"frogging"

"froggy"

"frogman"

"frogmen"

"frogs"

"frolic"

"frolicked"

"frolicker"

"frolickers"

"frolicking"

"frolicky"

"frolics"

"frolicsome"

"from"

"fromage"

"fromages"

"frond"

"fronds"

"front"

"front-end"

"frontage"

"frontager"

"frontages"

"frontal"

"frontally"

"frontals"

"fronted"

"fronter"

"frontier"

"frontiers"

"frontiersman"

"frontiersmen"

"fronting"

"frontispiece"

"frontispieces"

"frontlets"

"fronts"

"frontward"

"frosh"

"frost"

"frostbit"

"frostbite"

"frostbites"

"frostbiting"

"frostbitten"

"frosted"

"frosteds"

"frostier"

"frostiest"

"frostily"

"frostiness"

"frosting"

"frostings"

"frostlike"

"frosts"

"frostwork"

"frosty"

"froth"

"frothed"

"frothier"

"frothiest"

"frothily"

"frothiness"

"frothing"

"froths"

"frothy"

"froufrou"

"froufrous"

"frouncing"

"frow"

"froward"

"frowardness"

"frown"

"frowned"

"frowner"

"frowners"

"frowning"

"frowningly"

"frowns"

"frowsier"

"frowstier"

"frowstiest"

"frowsty"

"frowsy"

"frowzier"

"frowziest"

"frowzily"

"frowziness"

"frowzy"

"froze"

"frozen"

"frozenly"

"frozenness"

"fructified"

"fructifies"

"fructify"

"fructifying"

"fructose"

"fructoses"

"fructuary"

"frug"

"frugal"

"frugalities"

"frugality"

"frugally"

"frugged"

"frugging"

"frugs"

"fruit"

"fruitages"

"fruitcake"

"fruitcakes"

"fruited"

"fruiter"

"fruiterer"

"fruiterers"

"fruiters"

"fruitful"

"fruitfully"

"fruitfulness"

"fruitier"

"fruitiest"

"fruitiness"

"fruiting"

"fruition"

"fruitions"

"fruitless"

"fruitlessly"

"fruitlessness"

"fruitlet"

"fruitlets"

"fruits"

"fruity"

"frumenties"

"frumenty"

"frump"

"frumpier"

"frumpiest"

"frumpily"

"frumpish"

"frumps"

"frumpy"

"frusta"

"frustrate"

"frustrated"

"frustrates"

"frustrating"

"frustratingly"

"frustration"

"frustrations"

"frustum"

"frustums"

"fry"

"fryer"

"fryers"

"frying"

"frypan"

"frypans"

"fub"

"fubbed"

"fubbing"

"fubsier"

"fuchsia"

"fuchsias"

"fuck"

"fucked"

"fucking"

"fucks"

"fuddle"

"fuddled"

"fuddles"

"fuddling"

"fudge"

"fudged"

"fudges"

"fudging"

"fuds"

"fuehrer"

"fuehrers"

"fuel"

"fueled"

"fueler"

"fuelers"

"fueling"

"fuelled"

"fueller"

"fuellers"

"fuelling"

"fuels"

"fug"

"fugal"

"fugally"

"fugatos"

"fugged"

"fuggier"

"fugging"

"fuggy"

"fugit"

"fugitive"

"fugitively"

"fugitives"

"fugs"

"fugue"

"fugued"

"fugues"

"fuguing"

"fuguist"

"fuguists"

"fuhrer"

"fuhrers"

"fuji"

"fujis"

"fulcra"

"fulcrum"

"fulcrums"

"fulfil"

"fulfill"

"fulfilled"

"fulfiller"

"fulfillers"

"fulfilling"

"fulfilling moment"

"fulfillment"

"fulfillments"

"fulfills"

"fulfils"

"fulgent"

"fulgurant"

"fulgurate"

"full"

"full term"

"Full version"

"fullback"

"fullbacks"

"fulled"

"fuller"

"fullered"

"fulleries"

"fullering"

"fullers"

"fullery"

"fullest"

"fullface"

"fullfil"

"fulling"

"fullness"

"fulls"

"fullterm"

"fulltime"

"fully"

"fulminant"

"fulminate"

"fulminated"

"fulminates"

"fulminating"

"fulmination"

"fulminations"

"fulminator"

"fulness"

"fulnesses"

"fulsome"

"fulsomely"

"fulsomeness"

"fulvous"

"fumaric"

"fumarole"

"fumaroles"

"fumarolic"

"fumatory"

"fumble"

"fumbled"

"fumbler"

"fumblers"

"fumbles"

"fumbling"

"fume"

"fumed"

"fumeless"

"fumer"

"fumers"

"fumes"

"fumet"

"fumets"

"fumettes"

"fumier"

"fumiest"

"fumigant"

"fumigants"

"fumigate"

"fumigated"

"fumigates"

"fumigating"

"fumigation"

"fumigations"

"fumigator"

"fumigators"

"fuming"

"fumitory"

"fumy"

"fun"

"function"

"functional"

"functionalist"

"functionalistic"

"functionalities"

"functionality"

"functionally"

"functionaries"

"functionary"

"functioned"

"functioning"

"functionless"

"functions"

"fund"

"fundament"

"fundamental"

"fundamentalism"

"fundamentalist"

"fundamentalists"

"fundamentally"

"fundamentals"

"funded"

"fundi"

"funding"

"funds"

"funeral"

"funerals"

"funerary"

"funereal"

"funereally"

"funfair"

"funfairs"

"fungal"

"fungi"

"fungic"

"fungicidal"

"fungicidally"

"fungicide"

"fungicides"

"fungiform"

"fungitoxic"

"fungoid"

"fungoids"

"fungosity"

"fungous"

"fungus"

"funguses"

"funicular"

"funiculars"

"funk"

"funked"

"funker"

"funkers"

"funkier"

"funkiest"

"funking"

"funks"

"funky"

"funned"

"funnel"

"funneled"

"funneling"

"funnelled"

"funnelling"

"funnels"

"funnier"

"funnies"

"funniest"

"funnily"

"funniness"

"funning"

"funny"

"funnyman"

"funnymen"

"fur"

"furbelow"

"furbelows"

"furbish"

"furbished"

"furbishes"

"furbishing"

"furcated"

"furcates"

"furcula"

"furculae"

"furcular"

"furies"

"furioso"

"furious"

"furiously"

"furl"

"furlable"

"furled"

"furler"

"furlers"

"furless"

"furling"

"furlong"

"furlongs"

"furlough"

"furloughed"

"furloughing"

"furloughs"

"furls"

"furnace"

"furnaced"

"furnaces"

"furnacing"

"furnish"

"furnished"

"furnisher"

"furnishes"

"furnishing"

"furnishings"

"furniture"

"furor"

"furore"

"furores"

"furors"

"furred"

"furrier"

"furrieries"

"furriers"

"furriery"

"furriest"

"furrily"

"furriner"

"furriners"

"furriness"

"furring"

"furrings"

"furrow"

"furrowed"

"furrower"

"furrowers"

"furrowing"

"furrows"

"furrowy"

"furry"

"furs"

"further"

"furtherance"

"furthered"

"furthering"

"furthermore"

"furthermost"

"furthers"

"furthest"

"furtive"

"furtively"

"furtiveness"

"furuncle"

"furuncles"

"fury"

"furze"

"furzes"

"furzier"

"furzy"

"fuse"

"fused"

"fusee"

"fusees"

"fusel"

"fuselage"

"fuselages"

"fuseless"

"fusels"

"fuses"

"fusible"

"fusibleness"

"fusibly"

"fusiform"

"fusil"

"fusile"

"fusileer"

"fusileers"

"fusilier"

"fusiliers"

"fusillade"

"fusillades"

"fusils"

"fusing"

"fusion"

"fusional"

"fusions"

"fuss"

"fussbudget"

"fussbudgets"

"fussed"

"fusser"

"fussers"

"fusses"

"fussier"

"fussiest"

"fussily"

"fussiness"

"fussing"

"fusspot"

"fusspots"

"fussy"

"fustian"

"fustians"

"fustic"

"fustics"

"fustier"

"fustiest"

"fustily"

"fustiness"

"fusty"

"futhermore"

"futile"

"futilely"

"futileness"

"futilities"

"futility"

"futural"

"future"

"future menstruation"

"futureless"

"futures"

"futurism"

"futurisms"

"futurist"

"futuristic"

"futuristically"

"futurists"

"futurities"

"futurity"

"futurologist"

"futurologists"

"futurology"

"fuze"

"fuzed"

"fuzee"

"fuzees"

"fuzes"

"fuzil"

"fuzils"

"fuzing"

"fuzz"

"fuzzed"

"fuzzes"

"fuzzier"

"fuzziest"

"fuzzily"

"fuzziness"

"fuzzing"

"fuzzy"

"fwd"

"fylfot"

"fylfots"

"g spot"

"ga"

"gab"

"gabardine"

"gabardines"

"gabbed"

"gabber"

"gabbers"

"gabbier"

"gabbiest"

"gabbiness"

"gabbing"

"gabble"

"gabbled"

"gabbler"

"gabblers"

"gabbles"

"gabbling"

"gabbro"

"gabbroic"

"gabbros"

"gabby"

"gaberdine"

"gaberdines"

"gabfest"

"gabfests"

"gable"

"gabled"

"gables"

"gabling"

"gabon"

"gabriel"

"gabs"

"gad"

"gadabout"

"gadabouts"

"gadded"

"gadder"

"gadders"

"gadding"

"gadflies"

"gadfly"

"gadget"

"gadgeteer"

"gadgeteers"

"gadgetries"

"gadgetry"

"gadgets"

"gadgety"

"gadolinium"

"gads"

"gadzooks"

"gaelic"

"gaels"

"gaff"

"gaffe"

"gaffed"

"gaffer"

"gaffers"

"gaffes"

"gaffing"

"gaffs"

"gag"

"gaga"

"gage"

"gaged"

"gager"

"gagers"

"gages"

"gagged"

"gagger"

"gaggers"

"gagging"

"gaggle"

"gaggled"

"gaggles"

"gaggling"

"gaging"

"gagman"

"gagmen"

"gags"

"gagster"

"gagsters"

"gaieties"

"gaiety"

"gaily"

"gain"

"gainable"

"gained"

"gainer"

"gainers"

"gainful"

"gainfully"

"gainfulness"

"gaining"

"gainless"

"gainlier"

"gainliest"

"gainly"

"gains"

"gainsaid"

"gainsay"

"gainsayer"

"gainsayers"

"gainsaying"

"gainsays"

"gainst"

"gait"

"gaited"

"gaiter"

"gaiters"

"gaiting"

"gaits"

"gal"

"gala"

"galactic"

"galactometer"

"galactoscope"

"galactose"

"galahad"

"galahads"

"galas"

"galatea"

"galateas"

"galatians"

"galax"

"galaxies"

"galaxy"

"gale"

"galena"

"galenas"

"galenic"

"galenite"

"gales"

"galilean"

"galilee"

"galilees"

"galilei"

"galileo"

"galipot"

"galivant"

"gall"

"gallamine"

"gallant"

"gallanted"

"gallanting"

"gallantly"

"gallantries"

"gallantry"

"gallants"

"gallbladder"

"gallbladders"

"galled"

"galleon"

"galleons"

"galleried"

"galleries"

"gallery"

"gallerying"

"galley"

"galleys"

"gallflies"

"galliard"

"galliards"

"gallic"

"gallicism"

"gallicisms"

"gallied"

"gallies"

"gallimaufries"

"gallimaufry"

"galling"

"gallingly"

"gallinule"

"gallinules"

"gallium"

"galliums"

"gallivant"

"gallivanted"

"gallivanter"

"gallivanters"

"gallivanting"

"gallivants"

"gallnuts"

"gallomania"

"gallon"

"gallons"

"galloot"

"galloots"

"gallop"

"galloped"

"galloper"

"gallopers"

"gallophobia"

"galloping"

"gallops"

"gallows"

"gallowses"

"galls"

"gallstone"

"gallstones"

"gallup"

"gallus"

"galluses"

"gally"

"galoot"

"galoots"

"galop"

"galops"

"galore"

"galores"

"galosh"

"galoshed"

"galoshes"

"gals"

"galumph"

"galumphed"

"galumphing"

"galumphs"

"galvanic"

"galvanically"

"galvanism"

"galvanization"

"galvanizations"

"galvanize"

"galvanized"

"galvanizer"

"galvanizers"

"galvanizes"

"galvanizing"

"galvanometer"

"galvanometers"

"galvanometric"

"galvanoscope"

"gam"

"gamba"

"gambas"

"gambian"

"gambians"

"gambias"

"gambit"

"gambits"

"gamble"

"gambled"

"gambler"

"gamblers"

"gambles"

"gambling"

"gambol"

"gamboled"

"gamboling"

"gambolled"

"gambolling"

"gambols"

"gambrel"

"gambusias"

"game"

"gamecock"

"gamecocks"

"gamed"

"gamekeeper"

"gamekeepers"

"gamelan"

"gamelans"

"gamely"

"gameness"

"gamer"

"games"

"gamesmanship"

"gamesome"

"gamesomely"

"gamest"

"gamester"

"gamesters"

"gamete"

"gametes"

"gametic"

"gamey"

"gamic"

"gamier"

"gamiest"

"gamily"

"gamin"

"gamine"

"gamines"

"gaminess"

"gaming"

"gamings"

"gamins"

"gamma"

"gammas"

"gammer"

"gammon"

"gammons"

"gamomania"

"gamophobia"

"gams"

"gamut"

"gamuts"

"gamy"

"gander"

"gandered"

"gandering"

"ganders"

"gandhi"

"ganef"

"ganefs"

"ganev"

"ganevs"

"gang"

"ganged"

"ganger"

"gangers"

"ganges"

"ganging"

"gangland"

"ganglands"

"ganglia"

"ganglial"

"gangliar"

"gangliate"

"ganglier"

"gangliest"

"gangling"

"ganglion"

"ganglionic"

"ganglions"

"gangly"

"gangplank"

"gangplanks"

"gangplow"

"gangplows"

"gangrel"

"gangrene"

"gangrened"

"gangrenes"

"gangrening"

"gangrenous"

"gangs"

"gangster"

"gangsterism"

"gangsters"

"gangues"

"gangway"

"gangways"

"ganja"

"gannet"

"gannets"

"ganser"

"gantlet"

"gantleted"

"gantleting"

"gantlets"

"gantries"

"gantry"

"ganymede"

"ganymedes"

"gaol"

"gaoled"

"gaoler"

"gaolers"

"gaoling"

"gaols"

"gap"

"gape"

"gaped"

"gaper"

"gapers"

"gapes"

"gaping"

"gapingly"

"gaposis"

"gapped"

"gappier"

"gapping"

"gappy"

"gaps"

"gapy"

"gar"

"garage"

"garaged"

"garages"

"garaging"

"garb"

"garbage"

"garbages"

"garbanzo"

"garbanzos"

"garbed"

"garbing"

"garble"

"garbled"

"garbler"

"garblers"

"garbles"

"garbless"

"garbling"

"garbo"

"garbology"

"garbs"

"garcon"

"garcons"

"garde"

"garden"

"gardened"

"gardener"

"gardeners"

"gardenia"

"gardenias"

"gardening"

"gardens"

"garfield"

"garfish"

"garfishes"

"gargantua"

"gargantuan"

"gargle"

"gargled"

"gargler"

"garglers"

"gargles"

"gargling"

"gargoyle"

"gargoyled"

"gargoyles"

"garibaldi"

"garish"

"garishly"

"garishness"

"garland"

"garlanded"

"garlanding"

"garlands"

"garlic"

"garlicky"

"garlics"

"garment"

"garmented"

"garmenting"

"garments"

"garner"

"garnered"

"garnering"

"garners"

"garnet"

"garnetlike"

"garnets"

"garnish"

"garnishable"

"garnished"

"garnishee"

"garnisheed"

"garnisheeing"

"garnishees"

"garnishes"

"garnishing"

"garnishment"

"garnishments"

"garniture"

"garnitures"

"garoted"

"garotes"

"garoting"

"garotte"

"garotted"

"garotter"

"garotters"

"garottes"

"garotting"

"garret"

"garrets"

"garrison"

"garrisoned"

"garrisoning"

"garrisons"

"garrote"

"garroted"

"garroter"

"garroters"

"garrotes"

"garroting"

"garrotte"

"garrotted"

"garrotter"

"garrottes"

"garrotting"

"garrulity"

"garrulous"

"garrulously"

"garrulousness"

"gars"

"garter"

"gartered"

"gartering"

"garters"

"garth"

"garths"

"gary"

"gas"

"gasbag"

"gasbags"

"gaseous"

"gaseously"

"gaseousness"

"gases"

"gash"

"gashed"

"gasher"

"gashes"

"gashing"

"gashouse"

"gashouses"

"gasified"

"gasifier"

"gasifies"

"gasiform"

"gasify"

"gasifying"

"gasket"

"gaskets"

"gasless"

"gaslight"

"gaslights"

"gaslit"

"gasman"

"gasmen"

"gasogenes"

"gasohol"

"gasoliers"

"gasoline"

"gasolines"

"gasometer"

"gasp"

"gasped"

"gasper"

"gaspers"

"gasping"

"gasps"

"gassed"

"gasser"

"gassers"

"gasses"

"gassier"

"gassiest"

"gassiness"

"gassing"

"gassings"

"gassy"

"gastight"

"gastrectomies"

"gastrectomy"

"gastric"

"gastritis"

"gastroenteric"

"gastroenteritis"

"gastroenterological"

"gastroenterologically"

"gastroenterologist"

"gastroenterologists"

"gastroenterology"

"gastrointestinal"

"gastrolavage"

"gastrologist"

"gastrologists"

"gastrology"

"gastronome"

"gastronomes"

"gastronomic"

"gastronomical"

"gastronomically"

"gastronomy"

"gastropod"

"gastropods"

"gastroscope"

"gastroscopic"

"gastroscopy"

"gastrostomy"

"gastrulas"

"gasworks"

"gat"

"gate"

"gatecrasher"

"gatecrashers"

"gated"

"gatefold"

"gatefolds"

"gatekeeper"

"gatekeepers"

"gateless"

"gateman"

"gatemen"

"gatepost"

"gateposts"

"gates"

"gateway"

"gateways"

"gather"

"gathered"

"gatherer"

"gatherers"

"gathering"

"gatherings"

"gathers"

"gating"

"gator"

"gats"

"gatsby"

"gauche"

"gauchely"

"gaucheness"

"gaucher"

"gaucherie"

"gaucheries"

"gauchest"

"gaucho"

"gauchos"

"gaud"

"gauderies"

"gaudery"

"gaudier"

"gaudies"

"gaudiest"

"gaudily"

"gaudiness"

"gauds"

"gaudy"

"gauge"

"gaugeable"

"gauged"

"gauger"

"gaugers"

"gauges"

"gauging"

"gauls"

"gaunt"

"gaunter"

"gauntest"

"gauntlet"

"gauntleted"

"gauntlets"

"gauntly"

"gauntness"

"gauntries"

"gauss"

"gausses"

"gauze"

"gauzes"

"gauzier"

"gauziest"

"gauzily"

"gauziness"

"gauzy"

"gavage"

"gave"

"gavel"

"gaveled"

"gaveler"

"gaveling"

"gavelled"

"gaveller"

"gavelling"

"gavels"

"gavot"

"gavots"

"gavotte"

"gavotted"

"gavottes"

"gavotting"

"gawk"

"gawked"

"gawker"

"gawkers"

"gawkier"

"gawkies"

"gawkiest"

"gawkily"

"gawking"

"gawkish"

"gawks"

"gawky"

"gay"

"gayer"

"gayest"

"gayeties"

"gayety"

"gayly"

"gayness"

"gaynesses"

"gays"

"gaze"

"gazebo"

"gazeboes"

"gazebos"

"gazed"

"gazelle"

"gazelles"

"gazer"

"gazers"

"gazes"

"gazette"

"gazetted"

"gazetteer"

"gazetteers"

"gazettes"

"gazetting"

"gazing"

"gazpacho"

"gazpachos"

"gds"

"gear"

"gearbox"

"gearboxes"

"gearcase"

"gearcases"

"geared"

"gearing"

"gearings"

"gearless"

"gears"

"gearshift"

"gearshifts"

"gearwheel"

"gearwheels"

"gecko"

"geckoes"

"geckos"

"gecks"

"gee"

"geed"

"geegaw"

"geegaws"

"geeing"

"geek"

"geeks"

"gees"

"geese"

"geezer"

"geezers"

"gefilte"

"geiger"

"geisha"

"geishas"

"gel"

"gelable"

"gelatin"

"gelatine"

"gelatines"

"gelating"

"gelatinization"

"gelatinize"

"gelatinized"

"gelatinizing"

"gelatinous"

"gelatinously"

"gelatins"

"geld"

"gelded"

"gelder"

"gelders"

"gelding"

"geldings"

"gelds"

"gelee"

"gelees"

"gelid"

"gelidity"

"gelidly"

"gelignite"

"gelled"

"gelling"

"gels"

"gelt"

"gelts"

"gem"

"geminate"

"geminated"

"geminates"

"geminating"

"gemination"

"geminations"

"gemini"

"geminis"

"gemmier"

"gemmiest"

"gemmily"

"gemmological"

"gemmologist"

"gemmologists"

"gemmology"

"gemmy"

"gemological"

"gemologies"

"gemologist"

"gemologists"

"gemology"

"gems"

"gemsbok"

"gemsbucks"

"gemstone"

"gemstones"

"gemutlich"

"gemutlichkeit"

"gen"

"genal"

"gendarme"

"gendarmerie"

"gendarmes"

"gender"

"gendered"

"gendering"

"genders"

"gene"

"genealogical"

"genealogically"

"genealogies"

"genealogist"

"genealogists"

"genealogy"

"genera"

"general"

"generalissimo"

"generalissimos"

"generalists"

"generalities"

"generality"

"generalizable"

"generalization"

"generalizations"

"generalize"

"generalized"

"generalizer"

"generalizes"

"generalizing"

"generally"

"generals"

"generalship"

"generalships"

"generate"

"generated"

"generates"

"generating"

"generation"

"generational"

"generations"

"generative"

"generatively"

"generator"

"generators"

"generic"

"generic alternatives"

"generic drugs"

"generically"

"generics"

"generis"

"generosities"

"generosity"

"generous"

"generously"

"generousness"

"genes"

"geneses"

"genesiology"

"genesis"

"genet"

"genethlialogy"

"genetic"

"genetic defect"

"genetically"

"geneticist"

"geneticists"

"genetics"

"geneva"

"genevas"

"genghis"

"genial"

"geniality"

"genially"

"genic"

"genie"

"genies"

"genii"

"geniophobia"

"genital"

"genitalia"

"genitalic"

"genitals"

"genitive"

"genitives"

"genitors"

"genitourinary"

"geniture"

"genitures"

"genius"

"geniuses"

"genoa"

"genocidal"

"genocide"

"genocides"

"genome"

"genomes"

"genomic"

"genophobia"

"genotype"

"genotypes"

"genotypic"

"genotypical"

"genre"

"genres"

"gens"

"gent"

"genteel"

"genteeler"

"genteelest"

"genteelly"

"genteelness"

"gentian"

"gentians"

"gentil"

"gentile"

"gentiles"

"gentility"

"gentle"

"gentled"

"gentlefolk"

"gentlefolks"

"gentleman"

"gentlemanlike"

"gentlemanly"

"gentlemen"

"gentleness"

"gentler"

"gentles"

"gentlest"

"gentlewoman"

"gentlewomen"

"gentling"

"gently"

"gentries"

"gentrification"

"gentry"

"gents"

"genuflect"

"genuflected"

"genuflecting"

"genuflection"

"genuflections"

"genuflects"

"genuine"

"genuinely"

"genuineness"

"genus"

"genuses"

"geocentric"

"geocentrically"

"geochemical"

"geochemist"

"geochemistry"

"geochemists"

"geochronology"

"geocoding"

"geode"

"geodes"

"geodesic"

"geodesics"

"geodesist"

"geodesists"

"geodesy"

"geodetic"

"geodic"

"geoduck"

"geoducks"

"geog"

"geogeny"

"geogony"

"geographer"

"geographers"

"geographic"

"geographical"

"geographically"

"geographies"

"geography"

"geoid"

"geoidal"

"geoids"

"geol"

"geologer"

"geologers"

"geologic"

"geological"

"geologically"

"geologies"

"geologist"

"geologists"

"geology"

"geom"

"geomagnetic"

"geomagnetism"

"geomancies"

"geomancy"

"geomedicine"

"geometer"

"geometers"

"geometric"

"geometrical"

"geometrically"

"geometrician"

"geometricians"

"geometries"

"geometry"

"geomorphogeny"

"geomorphology"

"geophones"

"geophysical"

"geophysicist"

"geophysicists"

"geophysics"

"geophytes"

"geopolitics"

"geoponics"

"george"

"georgia"

"georgian"

"georgians"

"georgic"

"geoscientist"

"geoscientists"

"geostationary"

"geosynchronous"

"geosynclinal"

"geosyncline"

"geosynclines"

"geotaxy"

"geotechnics"

"geothermal"

"geothermic"

"geothermometer"

"geotropic"

"geotropically"

"gerald"

"geranium"

"geraniums"

"gerardias"

"gerascophobia"

"geratology"

"gerbil"

"gerbils"

"geriatric"

"geriatrician"

"geriatrics"

"geriatrist"

"germ"

"german"

"germane"

"germanely"

"germaneness"

"germanic"

"germanies"

"germanium"

"germaniums"

"germanized"

"germans"

"germantown"

"germany"

"germen"

"germfree"

"germicidal"

"germicide"

"germicides"

"germier"

"germiest"

"germinal"

"germinate"

"germinated"

"germinates"

"germinating"

"germination"

"germinations"

"germproof"

"germs"

"germy"

"gerocomy"

"gerontic"

"gerontological"

"gerontologies"

"gerontologist"

"gerontologists"

"gerontology"

"gerontotherapies"

"gerontotherapy"

"gerrymander"

"gerrymandered"

"gerrymandering"

"gerrymanders"

"gertrude"

"gerund"

"gerunds"

"GES"

"gesso"

"gessoes"

"gestalt"

"gestalten"

"gestalts"

"gestapo"

"gestapos"

"gestate"

"gestated"

"gestates"

"gestating"

"gestation"

"gestational"

"gestational age"

"Gestational diabetes"

"gestations"

"geste"

"gestes"

"gesticulate"

"gesticulated"

"gesticulates"

"gesticulating"

"gesticulation"

"gesticulations"

"gests"

"gestural"

"gesture"

"gestured"

"gesturer"

"gesturers"

"gestures"

"gesturing"

"gesundheit"

"get"

"get fit"

"get up"

"getable"

"getaway"

"getaways"

"gets"

"gettable"

"getter"

"gettered"

"getters"

"getting"

"gettysburg"

"getup"

"getups"

"geum"

"geums"

"gewgaw"

"gewgaws"

"geyser"

"geysers"

"ghana"

"ghanaians"

"ghanian"

"ghast"

"ghastful"

"ghastlier"

"ghastliest"

"ghastliness"

"ghastly"

"ghat"

"ghats"

"ghee"

"ghees"

"gherkin"

"gherkins"

"ghetto"

"ghettoed"

"ghettoes"

"ghettoing"

"ghettoize"

"ghettoized"

"ghettoizes"

"ghettoizing"

"ghettos"

"ghost"

"ghosted"

"ghostier"

"ghostiest"

"ghosting"

"ghostlier"

"ghostliest"

"ghostlike"

"ghostliness"

"ghostly"

"ghosts"

"ghostwrite"

"ghostwriter"

"ghostwriters"

"ghostwrites"

"ghostwriting"

"ghostwritten"

"ghostwrote"

"ghosty"

"ghoul"

"ghoulish"

"ghoulishly"

"ghoulishness"

"ghouls"

"giant"

"giantess"

"giantesses"

"giantism"

"giantisms"

"giants"

"gib"

"gibbed"

"gibber"

"gibbered"

"gibbering"

"gibberish"

"gibbers"

"gibbet"

"gibbeted"

"gibbeting"

"gibbets"

"gibbetted"

"gibbing"

"gibbon"

"gibbons"

"gibbosities"

"gibbosity"

"gibbous"

"gibbously"

"gibbousness"

"gibe"

"gibed"

"giber"

"gibers"

"gibes"

"gibing"

"gibingly"

"giblet"

"giblets"

"gibraltar"

"gibs"

"gibsons"

"gid"

"giddap"

"giddied"

"giddier"

"giddies"

"giddiest"

"giddily"

"giddiness"

"giddy"

"giddying"

"gift"

"gifted"

"giftedly"

"gifting"

"giftless"

"gifts"

"gig"

"gigabit"

"gigabits"

"gigabyte"

"gigabytes"

"gigantic"

"gigantically"

"gigantism"

"gigantology"

"gigas"

"gigaton"

"gigatons"

"gigawatt"

"gigawatts"

"gigged"

"gigging"

"giggle"

"giggled"

"giggler"

"gigglers"

"giggles"

"gigglier"

"giggliest"

"giggling"

"gigglingly"

"giggly"

"gigolo"

"gigolos"

"gigs"

"gigue"

"gigues"

"gila"

"gilbert"

"gilberts"

"gild"

"gilded"

"gilder"

"gilders"

"gildhall"

"gilding"

"gildings"

"gilds"

"gill"

"gilled"

"giller"

"gillers"

"gillie"

"gillied"

"gillies"

"gilling"

"gillnet"

"gillnets"

"gills"

"gilly"

"gilt"

"gilts"

"gimbal"

"gimbaled"

"gimbaling"

"gimballed"

"gimballing"

"gimbals"

"gimcrack"

"gimcrackery"

"gimcracks"

"gimel"

"gimels"

"gimlet"

"gimleted"

"gimleting"

"gimlets"

"gimmick"

"gimmicked"

"gimmicking"

"gimmickry"

"gimmicks"

"gimmicky"

"gimp"

"gimped"

"gimpier"

"gimpiest"

"gimping"

"gimps"

"gimpy"

"gin"

"ginger"

"gingerbread"

"gingered"

"gingering"

"gingerly"

"gingers"

"gingersnap"

"gingersnaps"

"gingery"

"gingham"

"ginghams"

"gingivae"

"gingival"

"gingivitis"

"gingko"

"ginkgo"

"ginkgoes"

"ginks"

"ginned"

"ginner"

"ginners"

"ginnier"

"ginning"

"ginnings"

"ginny"

"gins"

"ginseng"

"ginsengs"

"gip"

"gipped"

"gipper"

"gippers"

"gipping"

"gips"

"gipsied"

"gipsies"

"gipsy"

"gipsying"

"giraffe"

"giraffes"

"girasol"

"girasoles"

"gird"

"girded"

"girder"

"girders"

"girding"

"girdle"

"girdled"

"girdler"

"girdlers"

"girdles"

"girdling"

"girds"

"girl"

"girlfriend"

"girlfriends"

"girlhood"

"girlhoods"

"girlie"

"girlies"

"girlish"

"girlishness"

"girls"

"girly"

"girns"

"giros"

"girt"

"girted"

"girth"

"girthed"

"girthing"

"girths"

"girting"

"girts"

"gismo"

"gismos"

"gist"

"gists"

"git"

"gitanos"

"giuseppe"

"give"

"give up"

"giveable"

"giveaway"

"giveaways"

"given"

"givens"

"giver"

"givers"

"gives"

"givin"

"giving"

"gizmo"

"gizmos"

"gizzard"

"gizzards"

"gjetost"

"gjetosts"

"glabrous"

"glace"

"glaceed"

"glaceing"

"glaces"

"glacial"

"glacially"

"glaciate"

"glaciated"

"glaciates"

"glaciating"

"glacier"

"glaciered"

"glaciers"

"glaciologist"

"glaciologists"

"glaciology"

"glacises"

"glad"

"gladded"

"gladden"

"gladdened"

"gladdening"

"gladdens"

"gladder"

"gladdest"

"gladding"

"glade"

"gladelike"

"glades"

"gladiate"

"gladiator"

"gladiatorial"

"gladiators"

"gladier"

"gladiola"

"gladiolas"

"gladioli"

"gladiolus"

"gladioluses"

"gladlier"

"gladliest"

"gladly"

"gladness"

"glads"

"gladsome"

"gladsomely"

"gladstone"

"glady"

"glaive"

"glaives"

"glamor"

"glamorization"

"glamorizations"

"glamorize"

"glamorized"

"glamorizer"

"glamorizes"

"glamorizing"

"glamorous"

"glamorously"

"glamorousness"

"glamors"

"glamour"

"glamoured"

"glamouring"

"glamourize"

"glamourous"

"glamours"

"glance"

"glanced"

"glances"

"glancing"

"glancingly"

"gland"

"glanders"

"glandes"

"glands"

"glandular"

"glandularly"

"glans"

"glare"

"glared"

"glares"

"glarier"

"glaring"

"glaringly"

"glary"

"glasgow"

"glass"

"glassblower"

"glassblowers"

"glassblowing"

"glassed"

"glasser"

"glasses"

"glassful"

"glassfuls"

"glassie"

"glassier"

"glassies"

"glassiest"

"glassily"

"glassine"

"glassines"

"glassiness"

"glassing"

"glassman"

"glassmen"

"glassware"

"glasswork"

"glassworker"

"glassy"

"glaucoma"

"glaucomas"

"glaucomatous"

"glaucous"

"glaze"

"glazed"

"glazer"

"glazers"

"glazes"

"glazier"

"glazieries"

"glaziers"

"glaziery"

"glazing"

"glazings"

"glazy"

"gleam"

"gleamed"

"gleamier"

"gleamiest"

"gleaming"

"gleams"

"gleamy"

"glean"

"gleanable"

"gleaned"

"gleaner"

"gleaners"

"gleaning"

"gleanings"

"gleans"

"gleba"

"glebe"

"glee"

"gleeful"

"gleefully"

"gleefulness"

"gleeman"

"gleemen"

"glees"

"gleesome"

"glen"

"glendale"

"glengarries"

"glengarry"

"glens"

"glenwood"

"glib"

"glibber"

"glibbest"

"glibly"

"glibness"

"glide"

"glided"

"glider"

"gliders"

"glides"

"gliding"

"glim"

"glimmer"

"glimmered"

"glimmering"

"glimmerings"

"glimmers"

"glimpse"

"glimpsed"

"glimpser"

"glimpsers"

"glimpses"

"glimpsing"

"glims"

"glint"

"glinted"

"glinting"

"glints"

"glissade"

"glissaded"

"glissades"

"glissading"

"glissandi"

"glissando"

"glisten"

"glistened"

"glistening"

"glistens"

"glister"

"glistered"

"glistering"

"glisters"

"glitch"

"glitches"

"glitter"

"glittered"

"glittering"

"glitters"

"glittery"

"glitzy"

"gloam"

"gloaming"

"gloamings"

"gloat"

"gloated"

"gloater"

"gloaters"

"gloating"

"gloats"

"glob"

"global"

"globalism"

"globalist"

"globalists"

"globalization"

"globalize"

"globalized"

"globalizing"

"globally"

"globate"

"globe"

"globed"

"globes"

"globetrotter"

"globetrotters"

"globetrotting"

"globing"

"globoid"

"globoids"

"globose"

"globosities"

"globous"

"globs"

"globular"

"globularity"

"globularly"

"globularness"

"globule"

"globules"

"globulin"

"globulins"

"glockenspiel"

"glockenspiels"

"glogg"

"gloggs"

"glom"

"glommed"

"glomming"

"gloms"

"gloom"

"gloomed"

"gloomful"

"gloomier"

"gloomiest"

"gloomily"

"gloominess"

"glooming"

"gloomings"

"glooms"

"gloomy"

"glop"

"glops"

"gloria"

"gloriam"

"glorias"

"gloried"

"glories"

"glorification"

"glorifications"

"glorified"

"glorifier"

"glorifiers"

"glorifies"

"glorify"

"glorifying"

"glorious"

"gloriously"

"gloriousness"

"glory"

"glorying"

"gloss"

"glossal"

"glossarial"

"glossaries"

"glossary"

"glossed"

"glosser"

"glossers"

"glosses"

"glossier"

"glossies"

"glossiest"

"glossily"

"glossiness"

"glossing"

"glossolalia"

"glossologies"

"glossology"

"glossy"

"glottal"

"glottic"

"glottides"

"glottis"

"glottises"

"glottologies"

"glove"

"gloved"

"glover"

"glovers"

"gloves"

"gloving"

"glow"

"glowed"

"glower"

"glowered"

"glowering"

"glowers"

"glowflies"

"glowfly"

"glowing"

"glows"

"glowworm"

"glowworms"

"gloxinia"

"gloxinias"

"gloze"

"glucose"

"glucose level"

"glucose units"

"glucoses"

"glucosic"

"glue"

"glued"

"glueing"

"gluer"

"gluers"

"glues"

"gluey"

"gluier"

"gluiest"

"gluily"

"gluing"

"glum"

"glumly"

"glummer"

"glummest"

"glumness"

"glut"

"glutamate"

"glutamates"

"glutamine"

"gluteal"

"glutei"

"gluten"

"gluteus"

"glutinous"

"glutinously"

"gluts"

"glutted"

"glutting"

"glutton"

"gluttonies"

"gluttonous"

"gluttonously"

"gluttons"

"gluttony"

"glycemia"

"glyceraldehyde"

"glyceride"

"glycerin"

"glycerine"

"glycerins"

"glycerol"

"glycerols"

"glycerose"

"glyceryl"

"glyceryls"

"glycogen"

"glycogenic"

"glycogens"

"glycol"

"glycols"

"glycoside"

"glycosides"

"glycosidic"

"glycosyls"

"glyoxylic"

"glyph"

"glyphic"

"glyphs"

"glyptic"

"glyptics"

"glyptography"

"glyptology"

"gnarl"

"gnarled"

"gnarlier"

"gnarliest"

"gnarling"

"gnarls"

"gnarly"

"gnars"

"gnash"

"gnashed"

"gnashes"

"gnashing"

"gnat"

"gnats"

"gnattier"

"gnaw"

"gnawable"

"gnawed"

"gnawer"

"gnawers"

"gnawing"

"gnawingly"

"gnawings"

"gnawn"

"gnaws"

"gneiss"

"gneisses"

"gneissic"

"gnocchi"

"gnome"

"gnomes"

"gnomic"

"gnomical"

"gnomish"

"gnomist"

"gnomists"

"gnomon"

"gnomonic"

"gnomonics"

"gnomons"

"gnoses"

"gnosiology"

"gnosis"

"gnostic"

"gnotobiologies"

"gnotobiology"

"gnotobiotic"

"gnotobiotically"

"gnotobiotics"

"gnu"

"gnus"

"go"

"goad"

"goaded"

"goading"

"goads"

"goal"

"goaled"

"goalie"

"goalies"

"goaling"

"goalkeeper"

"goalkeepers"

"goalless"

"goalpost"

"goalposts"

"goals"

"goaltender"

"goaltenders"

"goat"

"goatee"

"goateed"

"goatees"

"goatfish"

"goatherd"

"goatherds"

"goatish"

"goats"

"goatskin"

"goatskins"

"gob"

"gobbed"

"gobbet"

"gobbets"

"gobbing"

"gobble"

"gobbled"

"gobbledegook"

"gobbledygook"

"gobbler"

"gobblers"

"gobbles"

"gobbling"

"gobies"

"goblet"

"goblets"

"goblin"

"goblins"

"goboes"

"gobos"

"gobs"

"goby"

"god"

"godchild"

"godchildren"

"goddam"

"goddamn"

"goddamned"

"goddamning"

"goddamns"

"goddams"

"goddard"

"goddaughter"

"goddaughters"

"goddess"

"goddesses"

"godding"

"godfather"

"godfathers"

"godhead"

"godheads"

"godhood"

"godhoods"

"godless"

"godlessly"

"godlessness"

"godlier"

"godliest"

"godlike"

"godlily"

"godliness"

"godling"

"godlings"

"godly"

"godmother"

"godmothers"

"godowns"

"godparent"

"godparents"

"gods"

"godsend"

"godsends"

"godship"

"godships"

"godson"

"godsons"

"godspeed"

"godwit"

"godwits"

"goebbels"

"goer"

"goers"

"goes"

"goethe"

"gofer"

"gofers"

"goffer"

"goffered"

"goggle"

"goggled"

"goggler"

"gogglers"

"goggles"

"gogglier"

"goggliest"

"goggling"

"goggly"

"goglets"

"gogo"

"gogos"

"going"

"goings"

"goiter"

"goiters"

"goitre"

"goitres"

"goitrous"

"golcondas"

"gold"

"goldarn"

"goldarns"

"goldbrick"

"goldbricker"

"goldbrickers"

"goldbricks"

"goldbugs"

"golden"

"goldener"

"goldenest"

"goldenly"

"goldenrod"

"goldenrods"

"golder"

"goldest"

"goldeyes"

"goldfield"

"goldfinch"

"goldfinches"

"goldfish"

"goldfishes"

"golds"

"goldsmith"

"goldsmiths"

"goldurn"

"goldurns"

"golem"

"golems"

"golf"

"golfed"

"golfer"

"golfers"

"golfing"

"golfings"

"golfs"

"golgotha"

"golgothas"

"goliaths"

"golliwog"

"golliwogs"

"golly"

"gombo"

"gombos"

"gombroons"

"gomorrah"

"gonad"

"gonadal"

"gonadectomies"

"gonadectomized"

"gonadectomizing"

"gonadectomy"

"gonadial"

"gonadic"

"gonads"

"gondola"

"gondolas"

"gondolier"

"gondoliers"

"gone"

"goneness"

"goner"

"goners"

"gonfalon"

"gonfalons"

"gong"

"gonged"

"gonging"

"gongs"

"gonif"

"gonifs"

"goniometer"

"gonococcal"

"gonococci"

"gonococcic"

"gonococcus"

"gonocytes"

"gonof"

"gonofs"

"gonoph"

"gonophore"

"gonophs"

"gonopores"

"gonorrhea"

"gonorrheal"

"gonorrhoea"

"goo"

"goober"

"goobers"

"good"

"good night sleep"

"goodby"

"goodbye"

"goodbyes"

"goodbys"

"gooder"

"gooders"

"goodie"

"goodies"

"goodish"

"goodlier"

"goodliest"

"goodly"

"goodman"

"goodmen"

"goodness"

"goodnight"

"goodrich"

"goods"

"goodwife"

"goodwill"

"goodwills"

"goodwives"

"goody"

"goodyear"

"gooey"

"goof"

"goofball"

"goofballs"

"goofed"

"goofier"

"goofiest"

"goofily"

"goofiness"

"goofing"

"goofs"

"goofy"

"Google"

"Google Earth"

"google map"

"googlies"

"googly"

"googol"

"googols"

"gooier"

"gooiest"

"gook"

"gooks"

"gooky"

"goon"

"gooney"

"gooneys"

"goonie"

"goonies"

"goons"

"goony"

"goop"

"goops"

"goos"

"goose"

"gooseberries"

"gooseberry"

"goosed"

"gooses"

"goosey"

"goosier"

"goosiest"

"goosing"

"goosy"

"gopher"

"gophers"

"gorals"

"gorblimy"

"gore"

"gored"

"gores"

"gorge"

"gorged"

"gorgedly"

"gorgeous"

"gorgeously"

"gorgeousness"

"gorger"

"gorgers"

"gorges"

"gorget"

"gorgets"

"gorging"

"gorgon"

"gorgons"

"gorgonzola"

"gorier"

"goriest"

"gorilla"

"gorillas"

"gorily"

"goriness"

"goring"

"gorki"

"gormand"

"gormandize"

"gormandized"

"gormandizer"

"gormandizers"

"gormandizes"

"gormandizing"

"gormands"

"gorse"

"gorses"

"gorsier"

"gorsy"

"gory"

"gosh"

"goshawk"

"goshawks"

"gosling"

"goslings"

"gospel"

"gospelers"

"gospels"

"gossamer"

"gossamers"

"gossip"

"gossiped"

"gossiper"

"gossipers"

"gossiping"

"gossipped"

"gossipping"

"gossipry"

"gossips"

"gossipy"

"gossoon"

"got"

"goth"

"gothic"

"gothically"

"gothicism"

"gothicist"

"gothicize"

"gothics"

"goths"

"gotten"

"gouache"

"gouaches"

"gouda"

"gouge"

"gouged"

"gouger"

"gougers"

"gouges"

"gouging"

"goulash"

"goulashes"

"gourami"

"gouramis"

"gourd"

"gourde"

"gourdes"

"gourds"

"gourmand"

"gourmandize"

"gourmands"

"gourmet"

"gourmets"

"gout"

"goutier"

"goutiest"

"goutily"

"goutiness"

"gouts"

"gouty"

"gov"

"govern"

"governability"

"governable"

"governableness"

"governance"

"governed"

"governess"

"governesses"

"governing"

"government"

"governmental"

"governments"

"governor"

"governorate"

"governors"

"governorship"

"governorships"

"governs"

"govt"

"gown"

"gowned"

"gowning"

"gowns"

"gownsman"

"gownsmen"

"goy"

"goyim"

"goyish"

"goys"

"gps"

"GPS location"

"GPS mode"

"graal"

"graals"

"grab"

"grabbed"

"grabber"

"grabbers"

"grabbier"

"grabbiest"

"grabbing"

"grabby"

"graben"

"grabens"

"grabs"

"grace"

"graced"

"graceful"

"gracefully"

"gracefulness"

"graceless"

"gracelessly"

"gracelessness"

"graces"

"gracile"

"graciles"

"gracilis"

"gracing"

"gracioso"

"graciosos"

"gracious"

"graciously"

"graciousness"

"grackle"

"grackles"

"grad"

"gradable"

"gradate"

"gradated"

"gradates"

"gradatim"

"gradating"

"gradation"

"gradational"

"gradations"

"grade"

"graded"

"grader"

"graders"

"grades"

"gradient"

"gradients"

"grading"

"gradiometer"

"grads"

"gradual"

"gradualism"

"gradually"

"graduals"

"graduand"

"graduands"

"graduate"

"graduated"

"graduates"

"graduating"

"graduation"

"graduations"

"graduator"

"graduators"

"graecize"

"graecized"

"graecizes"

"graecizing"

"Graecomania"

"graffiti"

"graffito"

"graft"

"graftage"

"graftages"

"grafted"

"grafter"

"grafters"

"grafting"

"grafts"

"graham"

"grail"

"grails"

"grain"

"grained"

"grainer"

"grainers"

"grainfield"

"grainier"

"grainiest"

"graininess"

"graining"

"grains"

"grainy"

"gram"

"gramarye"

"gramercy"

"graminology"

"grammar"

"Grammar Rules"

"grammarian"

"grammarians"

"grammars"

"grammatical"

"grammatically"

"grammatology"

"gramme"

"grammes"

"grammies"

"grammy"

"gramophone"

"gramophones"

"gramp"

"gramps"

"grampus"

"grampuses"

"grams"

"grana"

"granaries"

"granary"

"grand"

"grandad"

"grandads"

"grandam"

"grandame"

"grandames"

"grandams"

"grandaunt"

"grandaunts"

"grandbaby"

"grandchild"

"grandchildren"

"granddad"

"granddads"

"granddaughter"

"granddaughters"

"grande"

"grandee"

"grandees"

"grander"

"grandest"

"grandeur"

"grandeurs"

"grandfather"

"grandfathers"

"grandiloquence"

"grandiloquent"

"grandiloquently"

"grandiose"

"grandiosely"

"grandioseness"

"grandiosity"

"grandly"

"grandma"

"grandmas"

"grandmaster"

"grandmaternal"

"grandmother"

"grandmothers"

"grandnephew"

"grandnephews"

"grandness"

"grandniece"

"grandnieces"

"grandpa"

"grandparent"

"grandparents"

"grandpas"

"grands"

"grandsir"

"grandsirs"

"grandson"

"grandsons"

"grandstand"

"grandstander"

"grandstands"

"grandtotal"

"granduncle"

"granduncles"

"grange"

"granger"

"grangers"

"granges"

"granite"

"granites"

"graniteware"

"granitic"

"grannie"

"grannies"

"granny"

"granola"

"grant"

"grantable"

"granted"

"grantee"

"grantees"

"granter"

"granters"

"granting"

"grantor"

"grantors"

"grants"

"grantsman"

"grantsmanship"

"grantsmen"

"granular"

"granularity"

"granularly"

"granulate"

"granulated"

"granulates"

"granulating"

"granulation"

"granulations"

"granulator"

"granulators"

"granule"

"granules"

"granulose"

"grape"

"grapefruit"

"grapefruits"

"graperies"

"grapery"

"grapes"

"grapeshot"

"grapevine"

"grapevines"

"graph"

"graphed"

"graphemes"

"graphemics"

"graphic"

"graphical"

"graphically"

"graphicness"

"graphics"

"graphie"

"graphing"

"graphite"

"graphites"

"graphitic"

"graphological"

"graphologies"

"graphologist"

"graphologists"

"graphology"

"graphomania"

"graphophobia"

"graphs"

"graphy"

"grapier"

"grapnel"

"grapnels"

"grapple"

"grappled"

"grappler"

"grapplers"

"grapples"

"grappling"

"grapy"

"gras"

"grasp"

"graspable"

"grasped"

"grasper"

"graspers"

"grasping"

"graspingly"

"graspingness"

"grasps"

"grass"

"grass and hash"

"grassed"

"grassers"

"grasses"

"grassfire"

"grasshopper"

"grasshoppers"

"grassier"

"grassiest"

"grassily"

"grassing"

"grassland"

"grasslands"

"grassplot"

"grassroots"

"grassy"

"grata"

"gratae"

"grate"

"grated"

"grateful"

"gratefully"

"gratefulness"

"grater"

"graters"

"grates"

"gratia"

"gratias"

"gratification"

"gratifications"

"gratified"

"gratifies"

"gratify"

"gratifying"

"gratifyingly"

"gratin"

"grating"

"gratingly"

"gratings"

"gratins"

"gratis"

"gratitude"

"gratuities"

"gratuitous"

"gratuitously"

"gratuitousness"

"gratuity"

"graupel"

"gravamen"

"gravamina"

"grave"

"graveclothes"

"graved"

"gravel"

"graveled"

"graveless"

"graveling"

"gravelled"

"gravelling"

"gravelly"

"gravels"

"gravely"

"graven"

"graveness"

"graver"

"gravers"

"graves"

"gravest"

"gravestone"

"gravestones"

"graveyard"

"graveyards"

"gravid"

"gravidity"

"gravidly"

"gravidness"

"gravies"

"gravimeter"

"gravimeters"

"gravimetric"

"graving"

"gravitate"

"gravitated"

"gravitates"

"gravitating"

"gravitation"

"gravitational"

"gravitationally"

"gravitations"

"gravitative"

"gravitic"

"gravities"

"graviton"

"gravitons"

"gravity"

"gravure"

"gravures"

"gravy"

"gray"

"graybacks"

"graybeard"

"graybeards"

"grayed"

"grayer"

"grayest"

"graying"

"grayish"

"grayling"

"graylings"

"grayly"

"grayness"

"grayouts"

"grays"

"grazable"

"graze"

"grazed"

"grazer"

"grazers"

"grazes"

"grazier"

"graziers"

"grazing"

"grazingly"

"grazings"

"grazioso"

"grease"

"greased"

"greasepaint"

"greaser"

"greasers"

"greases"

"greasewood"

"greasier"

"greasiest"

"greasily"

"greasiness"

"greasing"

"greasy"

"great"

"greatcoat"

"greatcoated"

"greatcoats"

"greaten"

"greatened"

"greatening"

"greatens"

"greater"

"greatest"

"greathearted"

"greatheartedly"

"greatheartedness"

"greatly"

"greatness"

"greats"

"greave"

"greaved"

"greaves"

"grebe"

"grebes"

"grecian"

"grecians"

"grecized"

"grecizes"

"greco"

"greece"

"greed"

"greedier"

"greediest"

"greedily"

"greediness"

"greeds"

"greedy"

"greek"

"greeks"

"green"

"greenback"

"greenbacks"

"greenbelt"

"greened"

"greener"

"greeneries"

"greenery"

"greenest"

"greenflies"

"greengrocer"

"greengrocers"

"greenhorn"

"greenhorns"

"greenhouse"

"greenhouses"

"greenier"

"greeniest"

"greening"

"greenings"

"greenish"

"greenishness"

"greenland"

"greenly"

"greenness"

"greenroom"

"greenrooms"

"greens"

"greenstick"

"greensward"

"greenthumbed"

"greenwich"

"greenwood"

"greenwoods"

"greeny"

"greet"

"greeted"

"greeter"

"greeters"

"greeting"

"greetings"

"greets"

"gregarious"

"gregariously"

"gregariousness"

"gregatim"

"gregorian"

"gregory"

"gremlin"

"gremlins"

"gremmie"

"gremmies"

"gremmy"

"grenada"

"grenade"

"grenades"

"grenadier"

"grenadiers"

"grenadine"

"grenadines"

"greta"

"grew"

"grey"

"greyed"

"greyer"

"greyest"

"greyhound"

"greyhounds"

"greying"

"greyish"

"greyly"

"greyness"

"greys"

"grid"

"griddle"

"griddlecake"

"griddlecakes"

"griddled"

"griddles"

"griddling"

"grided"

"grides"

"gridiron"

"gridirons"

"gridlock"

"grids"

"grief"

"griefs"

"grievance"

"grievances"

"grievant"

"grieve"

"grieved"

"griever"

"grievers"

"grieves"

"grieving"

"grievingly"

"grievous"

"grievously"

"grievousness"

"griffin"

"griffins"

"griffon"

"griffons"

"grift"

"grifted"

"grifter"

"grifters"

"grifting"

"grifts"

"grigs"

"grill"

"grillades"

"grillage"

"grillages"

"grille"

"grilled"

"griller"

"grillers"

"grilles"

"grillework"

"grilling"

"grills"

"grillwork"

"grim"

"grimace"

"grimaced"

"grimacer"

"grimacers"

"grimaces"

"grimacing"

"grime"

"grimed"

"grimes"

"grimier"

"grimiest"

"grimily"

"griminess"

"griming"

"grimly"

"grimm"

"grimmer"

"grimmest"

"grimness"

"grimy"

"grin"

"grind"

"grinded"

"grinder"

"grinders"

"grindery"

"grinding"

"grindingly"

"grindings"

"grinds"

"grindstone"

"grindstones"

"gringo"

"gringos"

"grinned"

"grinner"

"grinners"

"grinning"

"grins"

"griot"

"griots"

"grip"

"gripe"

"griped"

"griper"

"gripers"

"gripes"

"gripey"

"gripier"

"gripiest"

"griping"

"grippe"

"gripped"

"gripper"

"grippers"

"grippes"

"grippier"

"grippiest"

"gripping"

"grippingly"

"gripple"

"grippy"

"grips"

"gripsack"

"gript"

"gripy"

"grislier"

"grisliest"

"grisly"

"grist"

"gristle"

"gristles"

"gristlier"

"gristliest"

"gristly"

"gristmill"

"grists"

"grit"

"grits"

"gritted"

"grittier"

"grittiest"

"grittily"

"grittiness"

"gritting"

"gritty"

"grizzle"

"grizzled"

"grizzler"

"grizzlers"

"grizzles"

"grizzlier"

"grizzlies"

"grizzliest"

"grizzling"

"grizzly"

"groan"

"groaned"

"groaner"

"groaners"

"groaning"

"groans"

"groat"

"groats"

"grocer"

"groceries"

"grocers"

"grocery"

"grog"

"groggery"

"groggier"

"groggiest"

"groggily"

"grogginess"

"groggy"

"grogram"

"grograms"

"grogs"

"grogshop"

"grogshops"

"groin"

"groined"

"groining"

"groins"

"gromatics"

"grommet"

"grommets"

"groom"

"groomed"

"groomer"

"groomers"

"grooming"

"grooms"

"groomsman"

"groomsmen"

"groove"

"grooved"

"groover"

"groovers"

"grooves"

"groovier"

"grooviest"

"grooving"

"groovy"

"grope"

"groped"

"groper"

"gropers"

"gropes"

"groping"

"gropingly"

"grosbeak"

"grosbeaks"

"groschen"

"grosgrain"

"grosgrains"

"gross"

"grossed"

"grosser"

"grossers"

"grosses"

"grossest"

"grossing"

"grossly"

"grossness"

"grosz"

"grot"

"grotesque"

"grotesquely"

"grotesqueness"

"grotesques"

"grots"

"grotto"

"grottoes"

"grottos"

"grouch"

"grouched"

"grouches"

"grouchier"

"grouchiest"

"grouchily"

"grouchiness"

"grouching"

"groucho"

"grouchy"

"ground"

"groundage"

"grounded"

"grounder"

"grounders"

"groundhog"

"grounding"

"groundless"

"groundlessly"

"groundlessness"

"groundling"

"groundlings"

"groundmass"

"groundnut"

"grounds"

"groundsheet"

"groundswell"

"groundswells"

"groundwater"

"groundwave"

"groundwork"

"group"

"grouped"

"grouper"

"groupers"

"groupie"

"groupies"

"grouping"

"groupings"

"groups"

"grouse"

"groused"

"grouser"

"grousers"

"grouses"

"grousing"

"grout"

"grouted"

"grouter"

"grouters"

"groutier"

"groutiest"

"grouting"

"grouts"

"grouty"

"grove"

"groved"

"grovel"

"groveled"

"groveler"

"grovelers"

"groveling"

"grovelled"

"grovelling"

"grovels"

"groves"

"grow"

"grow tool"

"growable"

"grower"

"growers"

"growing"

"growl"

"growled"

"growler"

"growlers"

"growlier"

"growliest"

"growling"

"growlingly"

"growls"

"growly"

"grown"

"grownup"

"grownups"

"grows"

"growth"

"growths"

"grub"

"grubbed"

"grubber"

"grubbers"

"grubbier"

"grubbiest"

"grubbily"

"grubbiness"

"grubbing"

"grubby"

"grubs"

"grubstake"

"grubstaked"

"grubstaker"

"grubstakes"

"grubstaking"

"grubworm"

"grubworms"

"grudge"

"grudged"

"grudger"

"grudgers"

"grudges"

"grudging"

"grudgingly"

"gruel"

"grueled"

"grueler"

"gruelers"

"grueling"

"gruelingly"

"gruelings"

"gruelled"

"grueller"

"gruellers"

"gruelling"

"gruellings"

"gruels"

"gruesome"

"gruesomely"

"gruesomeness"

"gruesomer"

"gruesomest"

"gruff"

"gruffed"

"gruffer"

"gruffest"

"gruffish"

"gruffly"

"gruffness"

"gruffs"

"gruffy"

"grumble"

"grumbled"

"grumbler"

"grumblers"

"grumbles"

"grumbling"

"grumbly"

"grump"

"grumped"

"grumpier"

"grumpiest"

"grumpily"

"grumpiness"

"grumping"

"grumpish"

"grumps"

"grumpy"

"grungier"

"grungiest"

"grungy"

"grunion"

"grunions"

"grunt"

"grunted"

"grunter"

"grunters"

"grunting"

"gruntingly"

"gruntle"

"gruntled"

"gruntles"

"grunts"

"grutten"

"gryphon"

"gryphons"

"guacamole"

"guaco"

"guam"

"guanaco"

"guanacos"

"guanin"

"guanine"

"guano"

"guanos"

"guar"

"guarani"

"guaranies"

"guaranis"

"guarantee"

"guaranteed"

"guaranteeing"

"guarantees"

"guarantied"

"guaranties"

"guarantor"

"guarantors"

"guaranty"

"guarantying"

"guard"

"guardant"

"guardants"

"guarded"

"guardedly"

"guarder"

"guarders"

"guardhouse"

"guardhouses"

"guardian"

"guardians"

"guardianship"

"guardianships"

"guarding"

"guardrail"

"guards"

"guardsman"

"guardsmen"

"guars"

"guatemala"

"guatemalan"

"guatemalans"

"guava"

"guavas"

"gubernative"

"gubernatorial"

"guck"

"gucks"

"gudgeon"

"gudgeons"

"guerdon"

"guerdons"

"guerilla"

"guerillas"

"guernsey"

"guernseys"

"guerre"

"guerrilla"

"guerrillas"

"guess"

"guessed"

"guesser"

"guessers"

"guesses"

"guessing"

"guesstimate"

"guesstimates"

"guesswork"

"guest"

"guested"

"guesting"

"guests"

"guff"

"guffaw"

"guffawed"

"guffawing"

"guffaws"

"guffs"

"guiana"

"guidable"

"guidance"

"guidances"

"guide"

"guidebook"

"guidebooks"

"guided"

"guideline"

"guidelines"

"guideposts"

"guider"

"guiders"

"guides"

"guiding"

"guidon"

"guidons"

"guild"

"guilder"

"guilders"

"guildhall"

"guildry"

"guilds"

"guile"

"guiled"

"guileful"

"guileless"

"guilelessly"

"guilelessness"

"guiles"

"guiling"

"guillotine"

"guillotined"

"guillotines"

"guillotining"

"guilt"

"guiltier"

"guiltiest"

"guiltily"

"guiltiness"

"guiltless"

"guiltlessly"

"guiltlessness"

"guilts"

"guilty"

"guinea"

"guinean"

"guineas"

"guinnes"

"guiro"

"guise"

"guised"

"guises"

"guising"

"guitar"

"guitarist"

"guitarists"

"guitars"

"gulch"

"gulches"

"gulden"

"guldens"

"gulf"

"gulfed"

"gulfier"

"gulfing"

"gulflike"

"gulfs"

"gulfweed"

"gulfy"

"gull"

"gullable"

"gullably"

"gulled"

"gullet"

"gullets"

"gulley"

"gulleys"

"gullibility"

"gullible"

"gullibly"

"gullied"

"gullies"

"gulling"

"gulls"

"gully"

"gullying"

"gulp"

"gulped"

"gulper"

"gulpers"

"gulpier"

"gulping"

"gulps"

"gulpy"

"gum"

"gumbo"

"gumboil"

"gumboils"

"gumbos"

"gumdrop"

"gumdrops"

"gumless"

"gumlike"

"gummed"

"gummer"

"gummers"

"gummier"

"gummiest"

"gumming"

"gummites"

"gummy"

"gumption"

"gumptions"

"gums"

"gumshoe"

"gumshoed"

"gumshoes"

"gumtree"

"gumtrees"

"gumweed"

"gumweeds"

"gumwood"

"gumwoods"

"gun"

"gunbarrel"

"gunboat"

"gunboats"

"guncotton"

"gundog"

"gunfight"

"gunfighter"

"gunfighters"

"gunfights"

"gunfire"

"gunfires"

"gung"

"gunk"

"gunks"

"gunless"

"gunlock"

"gunlocks"

"gunman"

"gunmen"

"gunmetal"

"gunmetals"

"gunned"

"gunnel"

"gunnels"

"gunner"

"gunneries"

"gunners"

"gunnery"

"gunnies"

"gunning"

"gunnings"

"gunny"

"gunnysack"

"gunnysacks"

"gunpapers"

"gunplay"

"gunplays"

"gunpoint"

"gunpoints"

"gunpowder"

"gunroom"

"gunrooms"

"gunrunner"

"gunrunning"

"guns"

"gunsel"

"gunsels"

"gunship"

"gunships"

"gunshot"

"gunshots"

"gunslinger"

"gunslingers"

"gunslinging"

"gunsmith"

"gunsmiths"

"gunstock"

"gunstocks"

"gunwale"

"gunwales"

"gunwhale"

"guppies"

"guppy"

"gurgle"

"gurgled"

"gurgles"

"gurgling"

"gurney"

"gurneys"

"guru"

"gurus"

"gush"

"gushed"

"gusher"

"gushers"

"gushes"

"gushier"

"gushiest"

"gushily"

"gushing"

"gushy"

"gusset"

"gusseted"

"gusseting"

"gussets"

"gussied"

"gussies"

"gussy"

"gussying"

"gust"

"gustable"

"gustation"

"gustative"

"gustatorial"

"gustatorially"

"gustatorily"

"gustatory"

"gusted"

"gustier"

"gustiest"

"gustily"

"gusting"

"gustless"

"gusto"

"gustoes"

"gusts"

"gusty"

"gut"

"gutless"

"gutlessness"

"gutlike"

"guts"

"gutsier"

"gutsiest"

"gutsy"

"gutta"

"guttatim"

"gutted"

"gutter"

"guttered"

"guttering"

"gutters"

"guttersnipe"

"guttersnipes"

"guttery"

"guttier"

"guttiest"

"gutting"

"guttural"

"gutturally"

"gutturals"

"gutty"

"guy"

"guyana"

"guyed"

"guying"

"guys"

"guzzle"

"guzzled"

"guzzler"

"guzzlers"

"guzzles"

"guzzling"

"gweduc"

"gweduck"

"gweducks"

"gweducs"

"gym"

"gymkhana"

"gymkhanas"

"gymnasia"

"gymnasium"

"gymnasiums"

"gymnast"

"gymnastic"

"gymnastically"

"gymnastics"

"gymnasts"

"gymnophobia"

"gymnosperm"

"gymnosperms"

"gyms"

"gynaecology"

"gynaecomania"

"gynarchy"

"gynecologic"

"gynecological"

"gynecologies"

"gynecologist"

"gynecologists"

"gynecology"

"gyp"

"gypped"

"gypper"

"gyppers"

"gypping"

"gyps"

"gypsied"

"gypsies"

"gypsum"

"gypsums"

"gypsy"

"gypsydom"

"gypsydoms"

"gypsying"

"gypsyish"

"gypsyism"

"gypsyisms"

"gyral"

"gyrate"

"gyrated"

"gyrates"

"gyrating"

"gyration"

"gyrations"

"gyrator"

"gyrators"

"gyratory"

"gyre"

"gyred"

"gyres"

"gyrfalcon"

"gyrfalcons"

"gyring"

"gyro"

"gyrocompass"

"gyrocompasses"

"gyrograph"

"gyroidal"

"gyromagnetic"

"gyros"

"gyroscope"

"gyroscopes"

"gyroscopic"

"gyroscopically"

"gyrose"

"gyrostatics"

"gyrus"

"gyve"

"gyved"

"gyves"

"gyving"

"ha"

"habanera"

"habaneras"

"habeas"

"haberdasher"

"haberdasheries"

"haberdashers"

"haberdashery"

"habile"

"habiliment"

"habiliments"

"habilitate"

"habilitation"

"habit"

"habitability"

"habitable"

"habitableness"

"habitably"

"habitancies"

"habitancy"

"habitant"

"habitants"

"habitat"

"habitation"

"habitations"

"habitats"

"habited"

"habiting"

"habits"

"habitual"

"habituality"

"habitually"

"habitualness"

"habituate"

"habituated"

"habituates"

"habituating"

"habituation"

"habituations"

"habitude"

"habitue"

"habitues"

"habromania"

"hacienda"

"haciendas"

"hack"

"hackamore"

"hackberry"

"hackbut"

"hacked"

"hackee"

"hacker"

"hackers"

"hackie"

"hackies"

"hacking"

"hackle"

"hackled"

"hackler"

"hacklers"

"hackles"

"hacklier"

"hackling"

"hackly"

"hackman"

"hackmen"

"hackney"

"hackneyed"

"hackneying"

"hackneys"

"hacks"

"hacksaw"

"hacksaws"

"hackwork"

"hackworks"

"had"

"haddie"

"haddock"

"haddocks"

"hades"

"hading"

"hadj"

"hadjee"

"hadjees"

"hadjes"

"hadji"

"hadjis"

"hadron"

"hadronic"

"hadrons"

"hadst"

"haemataulics"

"haematin"

"haemoglobin"

"haets"

"hafnium"

"hafniums"

"haft"

"hafted"

"hafter"

"hafting"

"haftorah"

"haftorahs"

"hafts"

"hag"

"hagadists"

"hagborn"

"hagbuts"

"hagfish"

"haggard"

"haggardly"

"haggards"

"hagged"

"hagging"

"haggis"

"haggises"

"haggish"

"haggle"

"haggled"

"haggler"

"hagglers"

"haggles"

"haggling"

"hagiographer"

"hagiographers"

"hagiography"

"hagiology"

"hagiomania"

"hagridden"

"hagride"

"hagrides"

"hagriding"

"hagrode"

"hags"

"hague"

"hah"

"hahnium"

"hahs"

"haiku"

"hail"

"hailed"

"hailer"

"hailers"

"hailing"

"hails"

"hailstone"

"hailstones"

"hailstorm"

"hailstorms"

"hair"

"hairball"

"hairballs"

"hairband"

"hairbands"

"hairbreadth"

"hairbreadths"

"hairbrush"

"hairbrushes"

"haircaps"

"haircloth"

"haircloths"

"haircut"

"haircuts"

"haircutter"

"haircutting"

"hairdo"

"hairdos"

"hairdresser"

"hairdressers"

"hairdressing"

"haired"

"hairier"

"hairiest"

"hairiness"

"hairless"

"hairlessness"

"hairlike"

"hairline"

"hairlines"

"hairlock"

"hairlocks"

"hairpiece"

"hairpieces"

"hairpin"

"hairpins"

"hairs"

"hairsbreadth"

"hairsbreadths"

"hairsplitter"

"hairsplitters"

"hairsplitting"

"hairspray"

"hairsprays"

"hairspring"

"hairsprings"

"hairstreak"

"hairstyle"

"hairstyles"

"hairstyling"

"hairstylist"

"hairstylists"

"hairweaver"

"hairweavers"

"hairweaving"

"hairwork"

"hairworks"

"hairworm"

"hairy"

"haiti"

"haitian"

"haitians"

"haji"

"hajis"

"hajj"

"hajjes"

"hajji"

"hajjis"

"hake"

"hakeems"

"hakes"

"halavah"

"halavahs"

"halberd"

"halberds"

"halcyon"

"halcyons"

"hale"

"haled"

"haleness"

"haler"

"halers"

"hales"

"halest"

"half"

"halfback"

"halfbacks"

"halfbeak"

"halfbeaks"

"halfhearted"

"halfheartedly"

"halfheartedness"

"halflife"

"halflives"

"halfpence"

"halfpennies"

"halfpenny"

"halftime"

"halftimes"

"halftone"

"halftones"

"halfway"

"halibut"

"halibuts"

"halide"

"halides"

"halidom"

"halidome"

"halidomes"

"halidoms"

"halieutics"

"halifax"

"haling"

"halite"

"halitoses"

"halitosis"

"hall"

"hallah"

"hallelujah"

"hallelujahs"

"hallmark"

"hallmarked"

"hallmarks"

"hallo"

"halloa"

"halloaing"

"halloas"

"halloed"

"halloes"

"halloo"

"hallooed"

"hallooing"

"halloos"

"hallos"

"hallow"

"hallowed"

"halloween"

"halloweens"

"hallower"

"hallowers"

"hallowing"

"hallows"

"halls"

"hallucinate"

"hallucinated"

"hallucinates"

"hallucinating"

"hallucination"

"hallucinational"

"hallucinations"

"hallucinative"

"hallucinatory"

"hallucinogen"

"hallucinogenic"

"hallucinogens"

"hallucinoses"

"hallucinosis"

"hallway"

"hallways"

"halo"

"haloed"

"haloes"

"halogen"

"halogenating"

"halogenoid"

"halogenous"

"halogens"

"haloids"

"haloing"

"halometer"

"halos"

"halt"

"halted"

"halter"

"haltered"

"haltering"

"halters"

"halting"

"haltingly"

"halts"

"halva"

"halvah"

"halvahs"

"halvas"

"halve"

"halved"

"halvers"

"halves"

"halving"

"halyard"

"halyards"

"ham"

"hamadryad"

"hamartiology"

"hamburg"

"hamburger"

"hamburgers"

"hamburgs"

"hamilton"

"hamiltonian"

"hamlet"

"hamlets"

"hammed"

"hammer"

"hammered"

"hammerer"

"hammerers"

"hammerhead"

"hammerheaded"

"hammerheads"

"hammering"

"hammerless"

"hammerlock"

"hammerlocks"

"hammers"

"hammertoe"

"hammertoes"

"hammier"

"hammiest"

"hammily"

"hamming"

"hammock"

"hammocks"

"hammy"

"hamper"

"hampered"

"hamperer"

"hamperers"

"hampering"

"hampers"

"hampshire"

"hampshireman"

"hampshiremen"

"hampshirite"

"hampshirites"

"hams"

"hamster"

"hamsters"

"hamstring"

"hamstringing"

"hamstrings"

"hamstrung"

"hance"

"hand"

"handbag"

"handbags"

"handball"

"handballs"

"handbarrow"

"handbarrows"

"handbill"

"handbills"

"handbook"

"handbooks"

"handbreadth"

"handcar"

"handcars"

"handcart"

"handcarts"

"handclasp"

"handclasps"

"handcraft"

"handcrafted"

"handcrafting"

"handcrafts"

"handcuff"

"handcuffed"

"handcuffing"

"handcuffs"

"handed"

"handedly"

"handedness"

"handel"

"handfast"

"handfasted"

"handfasts"

"handful"

"handfuls"

"handgrip"

"handgrips"

"handgun"

"handguns"

"handhold"

"handholds"

"handicap"

"handicapped"

"handicapper"

"handicappers"

"handicapping"

"handicaps"

"handicraft"

"handicrafts"

"handicraftsman"

"handicraftsmen"

"handier"

"handiest"

"handily"

"handiness"

"handing"

"handiwork"

"handkerchief"

"handkerchiefs"

"handle"

"handlebar"

"handlebars"

"handled"

"handler"

"handlers"

"handles"

"handless"

"handling"

"handlings"

"handlists"

"handloom"

"handlooms"

"handmade"

"handmaid"

"handmaiden"

"handmaidens"

"handmaids"

"handoff"

"handoffs"

"handout"

"handouts"

"handpick"

"handpicked"

"handpicking"

"handpicks"

"handpiece"

"handrail"

"handrails"

"hands"

"handsaw"

"handsaws"

"handsbreadth"

"handselling"

"handset"

"handsets"

"handsewn"

"handsful"

"handshake"

"handshakes"

"handshaking"

"handsome"

"handsomely"

"handsomeness"

"handsomer"

"handsomest"

"handspring"

"handsprings"

"handstand"

"handstands"

"handwheel"

"handwork"

"handworks"

"handwoven"

"handwrit"

"handwrite"

"handwrites"

"handwriting"

"handwritings"

"handwritten"

"handwrote"

"handy"

"handyman"

"handymen"

"hang"

"hangable"

"hangar"

"hangared"

"hangaring"

"hangars"

"hangdog"

"hangdogs"

"hanged"

"hanger"

"hangers"

"hangfire"

"hanging"

"hangings"

"hangman"

"hangmen"

"hangnail"

"hangnails"

"hangout"

"hangouts"

"hangover"

"hangovers"

"hangs"

"hangtag"

"hangup"

"hangups"

"hank"

"hanked"

"hanker"

"hankered"

"hankerer"

"hankerers"

"hankering"

"hankerings"

"hankers"

"hankie"

"hankies"

"hanking"

"hanks"

"hanksgiving"

"hanky"

"hanoi"

"hansel"

"hansom"

"hansoms"

"hants"

"hanukkah"

"hanuman"

"hanumans"

"haole"

"haoles"

"hap"

"haphazard"

"haphazardly"

"haphazardness"

"hapless"

"haplessly"

"haplessness"

"haploid"

"haploids"

"haploidy"

"haply"

"happen"

"happened"

"happening"

"happenings"

"happens"

"happenstance"

"happier"

"happiest"

"happily"

"happiness"

"happing"

"happy"

"haps"

"haptometer"

"harangue"

"harangued"

"haranguer"

"haranguers"

"harangues"

"haranguing"

"harass"

"harassed"

"harasser"

"harassers"

"harasses"

"harassing"

"harassingly"

"harassment"

"harassments"

"harbinger"

"harbingers"

"harbor"

"harborage"

"harbored"

"harborer"

"harborers"

"harboring"

"harborless"

"harbors"

"harbour"

"harboured"

"harbouring"

"harbours"

"hard"

"hardback"

"hardbacks"

"hardball"

"hardballs"

"hardboard"

"hardboiled"

"hardboots"

"hardbought"

"hardbound"

"hardcase"

"hardcore"

"hardcover"

"hardcovers"

"harden"

"hardened"

"hardener"

"hardeners"

"hardening"

"hardens"

"harder"

"hardest"

"hardhacks"

"hardhanded"

"hardhandedness"

"hardhat"

"hardhats"

"hardhead"

"hardheaded"

"hardheadedly"

"hardheadedness"

"hardheads"

"hardhearted"

"hardheartedly"

"hardheartedness"

"hardier"

"hardies"

"hardiest"

"hardihood"

"hardily"

"hardiness"

"harding"

"hardly"

"hardness"

"hardpan"

"hardpans"

"hards"

"hardset"

"hardshell"

"hardship"

"hardships"

"hardstand"

"hardstands"

"hardtack"

"hardtacks"

"hardtop"

"hardtops"

"hardware"

"hardwares"

"hardwired"

"hardwood"

"hardwoods"

"hardworking"

"hardy"

"hare"

"harebell"

"harebells"

"harebrained"

"hared"

"hareem"

"hareems"

"harelike"

"harelip"

"harelipped"

"harelips"

"harem"

"harems"

"hares"

"haring"

"hark"

"harked"

"harken"

"harkened"

"harkener"

"harkeners"

"harkening"

"harkens"

"harking"

"harks"

"harlem"

"harlequin"

"harlequins"

"harlot"

"harlotries"

"harlotry"

"harlots"

"harm"

"harmed"

"harmer"

"harmers"

"harmful"

"harmfully"

"harmfulness"

"harming"

"harmless"

"harmlessly"

"harmlessness"

"harmonic"

"harmonica"

"harmonically"

"harmonicas"

"harmonics"

"harmonies"

"harmonious"

"harmoniously"

"harmoniousness"

"harmonium"

"harmoniums"

"harmonization"

"harmonizations"

"harmonize"

"harmonized"

"harmonizer"

"harmonizers"

"harmonizes"

"harmonizing"

"harmonograph"

"harmonometer"

"harmony"

"harms"

"harness"

"harnessed"

"harnesser"

"harnessers"

"harnesses"

"harnessing"

"harold"

"harp"

"harped"

"harper"

"harpers"

"harpies"

"harping"

"harpings"

"harpist"

"harpists"

"harpoon"

"harpooned"

"harpooner"

"harpooners"

"harpooning"

"harpoons"

"harps"

"harpsichord"

"harpsichordist"

"harpsichords"

"harpy"

"harridan"

"harridans"

"harried"

"harrier"

"harriers"

"harries"

"harriet"

"harris"

"Harris-Benedict formula"

"harrison"

"harrow"

"harrowed"

"harrower"

"harrowers"

"harrowing"

"harrows"

"harrumph"

"harrumphed"

"harrumphs"

"harry"

"harrying"

"harsh"

"harshen"

"harshened"

"harshening"

"harshens"

"harsher"

"harshest"

"harshly"

"harshness"

"hart"

"hartebeest"

"hartford"

"harts"

"hartshorn"

"haruspex"

"harvard"

"harvest"

"harvestable"

"harvested"

"harvester"

"harvesters"

"harvesting"

"harvestman"

"harvests"

"has"

"hasenpfeffer"

"hash"

"hashed"

"hasheesh"

"hasheeshes"

"hashes"

"hashhead"

"hashheads"

"hashing"

"hashish"

"hashishes"

"hasid"

"hasidic"

"hasidim"

"hasp"

"hasped"

"hasping"

"hasps"

"hassels"

"hassle"

"hassled"

"hassles"

"hassling"

"hassock"

"hassocks"

"hast"

"hasta"

"haste"

"hasted"

"hasteful"

"hasten"

"hastened"

"hastener"

"hasteners"

"hastening"

"hastens"

"hastes"

"hastier"

"hastiest"

"hastily"

"hastiness"

"hasting"

"hasty"

"hat"

"hatable"

"hatband"

"hatbands"

"hatbox"

"hatboxes"

"hatch"

"hatchable"

"hatchback"

"hatchbacks"

"hatcheck"

"hatched"

"hatcheling"

"hatchelled"

"hatcher"

"hatcheries"

"hatchers"

"hatchery"

"hatches"

"hatchet"

"hatchetlike"

"hatchets"

"hatching"

"hatchings"

"hatchment"

"hatchway"

"hatchways"

"hate"

"hateable"

"hated"

"hateful"

"hatefully"

"hatefulness"

"hatemonger"

"hatemongering"

"hater"

"haters"

"hates"

"hatful"

"hatfuls"

"hath"

"hating"

"hatless"

"hatmaker"

"hatmakers"

"hatpin"

"hatpins"

"hatrack"

"hatracks"

"hatred"

"hatreds"

"hats"

"hatsful"

"hatted"

"hatter"

"hatters"

"hatting"

"hauberk"

"hauberks"

"haugh"

"haughtier"

"haughtiest"

"haughtily"

"haughtiness"

"haughty"

"haul"

"haulage"

"hauled"

"hauler"

"haulers"

"haulier"

"hauling"

"hauls"

"haulyard"

"haulyards"

"haunch"

"haunched"

"haunches"

"haunt"

"haunted"

"haunter"

"haunters"

"haunting"

"hauntingly"

"haunts"

"hausfrau"

"hausfrauen"

"hausfraus"

"hautbois"

"hautboy"

"hautboys"

"haute"

"hauteur"

"hauteurs"

"havana"

"have"

"haven"

"havened"

"havening"

"havens"

"haver"

"havers"

"haversack"

"haversacks"

"haves"

"having"

"haviors"

"haviour"

"haviours"

"havoc"

"havocked"

"havocker"

"havockers"

"havocking"

"havocs"

"haw"

"hawaii"

"hawaiian"

"hawaiians"

"hawed"

"hawing"

"hawk"

"hawkbill"

"hawkbills"

"hawked"

"hawker"

"hawkers"

"hawkeye"

"hawkeys"

"hawkies"

"hawking"

"hawkings"

"hawkish"

"hawkmoth"

"hawkmoths"

"hawknose"

"hawknoses"

"hawks"

"hawkshaw"

"hawkshaws"

"hawkweed"

"hawkweeds"

"haws"

"hawse"

"hawser"

"hawsers"

"hawses"

"hawthorn"

"hawthorne"

"hawthorns"

"hay"

"haycock"

"haycocks"

"haydn"

"hayed"

"hayer"

"hayers"

"hayes"

"hayfields"

"hayfork"

"hayforks"

"haying"

"hayings"

"hayloft"

"haylofts"

"haymaker"

"haymakers"

"haymow"

"haymows"

"hayrack"

"hayracks"

"hayrick"

"hayricks"

"hayride"

"hayrides"

"hays"

"hayseed"

"hayseeds"

"haystack"

"haystacks"

"hayward"

"haywards"

"haywire"

"haywires"

"hazard"

"hazarded"

"hazarding"

"hazardless"

"hazardous"

"hazardously"

"hazardousness"

"hazards"

"haze"

"hazed"

"hazel"

"hazelnut"

"hazelnuts"

"hazels"

"hazer"

"hazers"

"hazes"

"hazier"

"haziest"

"hazily"

"haziness"

"hazing"

"hazings"

"hazy"

"hdqrs"

"he"

"head"

"headache"

"headaches"

"headachier"

"headachy"

"headband"

"headbands"

"headboard"

"headboards"

"headcheese"

"headdress"

"headdresses"

"headed"

"header"

"headers"

"headfirst"

"headforemost"

"headgear"

"headgears"

"headhunt"

"headhunted"

"headhunter"

"headhunters"

"headhunting"

"headhunts"

"headier"

"headiest"

"headily"

"headiness"

"heading"

"headings"

"headlamp"

"headlamps"

"headland"

"headlands"

"headless"

"headlight"

"headlights"

"headline"

"headlined"

"headlines"

"headlining"

"headlock"

"headlocks"

"headlong"

"headman"

"headmaster"

"headmasters"

"headmen"

"headmistress"

"headmistresses"

"headmost"

"headnote"

"headnotes"

"headphone"

"headphones"

"headpiece"

"headpieces"

"headpin"

"headpins"

"headquarter"

"headquartered"

"headquartering"

"headquarters"

"headrest"

"headrests"

"headroom"

"headrooms"

"heads"

"headset"

"headsets"

"headship"

"headshrinker"

"headsman"

"headsmen"

"headspring"

"headstall"

"headstalls"

"headstand"

"headstands"

"headstay"

"headstone"

"headstones"

"headstrong"

"headwaiter"

"headwaiters"

"headwater"

"headwaters"

"headway"

"headways"

"headwind"

"headwinds"

"headword"

"headwords"

"headwork"

"headworks"

"heady"

"heal"

"healable"

"healed"

"healer"

"healers"

"healing"

"healing art"

"healing melodies"

"heals"

"health"

"health application"

"Health Care Business"

"health care professional"

"Health Care Provider"

"Health Care Record"

"Health Guarantees"

"health history"

"health information"

"health insurance"

"Health insurance app"

"health knowledge"

"health plan"

"health policy"

"health questions and answers"

"health record"

"health summary"

"health system"

"health tracker"

"healthcare"

"healthcare professionals"

"healthcare worker"

"healthful"

"healthfully"

"healthfulness"

"healthier"

"healthiest"

"healthily"

"healthiness"

"healths"

"healthy"

"heap"

"heaped"

"heaping"

"heaps"

"hear"

"hearable"

"heard"

"hearer"

"hearers"

"hearing"

"hearings"

"hearken"

"hearkened"

"hearkening"

"hearkens"

"hears"

"hearsay"

"hearsays"

"hearse"

"hearsed"

"hearses"

"hearsing"

"heart"

"heart beats"

"Heart disease"

"heart failure"

"heart frequency"

"heart health"

"heart model"

"Heart Rate"

"heart rate monitor"

"heart rate monitoring"

"Heart Sound"

"heartache"

"heartaches"

"heartbeat"

"heartbeats"

"heartbreak"

"heartbreaker"

"heartbreaking"

"heartbreaks"

"heartbroke"

"heartbroken"

"heartburn"

"heartburns"

"hearted"

"hearten"

"heartened"

"heartening"

"heartens"

"heartfelt"

"hearth"

"hearths"

"hearthside"

"hearthsides"

"hearthstone"

"hearthstones"

"heartier"

"hearties"

"heartiest"

"heartily"

"heartiness"

"hearting"

"heartland"

"heartlands"

"heartless"

"heartlessly"

"heartlessness"

"heartrending"

"hearts"

"heartsick"

"heartsickness"

"heartsore"

"heartstring"

"heartstrings"

"heartthrob"

"heartthrobs"

"heartwarming"

"heartwood"

"heartworm"

"heat"

"heatable"

"heated"

"heatedly"

"heater"

"heaters"

"heath"

"heathen"

"heathendom"

"heathenish"

"heathenism"

"heathens"

"heather"

"heathered"

"heathers"

"heathery"

"heathier"

"heathiest"

"heaths"

"heathy"

"heating"

"heatless"

"heats"

"heatstroke"

"heatstrokes"

"heave"

"heaved"

"heaven"

"heavenlier"

"heavenly"

"heavens"

"heavenward"

"heaver"

"heavers"

"heaves"

"heavier"

"heavies"

"heaviest"

"heavily"

"heaviness"

"heaving"

"heavy"

"heavyhearted"

"heavyheartedness"

"heavyset"

"heavyweight"

"heavyweights"

"hebephrenia"

"hebephrenic"

"hebraic"

"hebraism"

"hebraist"

"hebraists"

"hebraized"

"hebraizes"

"hebraizing"

"hebrew"

"hebrews"

"hecatomb"

"hecatombs"

"heck"

"heckle"

"heckled"

"heckler"

"hecklers"

"heckles"

"heckling"

"hecks"

"hectare"

"hectares"

"hectic"

"hectical"

"hectically"

"hecticly"

"hectogram"

"hectograms"

"hectoliter"

"hectoliters"

"hectometer"

"hectometers"

"hector"

"hectored"

"hectoring"

"hectors"

"hedge"

"hedged"

"hedgehog"

"hedgehogs"

"hedgehop"

"hedgehopped"

"hedgehopper"

"hedgehopping"

"hedgehops"

"hedgepig"

"hedgepigs"

"hedger"

"hedgerow"

"hedgerows"

"hedgers"

"hedges"

"hedgier"

"hedgiest"

"hedging"

"hedgy"

"hedonic"

"hedonically"

"hedonics"

"hedonism"

"hedonisms"

"hedonist"

"hedonistic"

"hedonists"

"hee"

"heed"

"heeded"

"heeder"

"heeders"

"heedful"

"heedfully"

"heedfulness"

"heeding"

"heedless"

"heedlessly"

"heedlessness"

"heeds"

"heehaw"

"heehawed"

"heehawing"

"heehaws"

"heel"

"heelballs"

"heeled"

"heeler"

"heelers"

"heeling"

"heelings"

"heelless"

"heelpost"

"heelposts"

"heels"

"heeltap"

"heeltaps"

"heft"

"hefted"

"hefter"

"hefters"

"heftier"

"heftiest"

"heftily"

"heftiness"

"hefting"

"hefts"

"hefty"

"hegemon"

"hegemonic"

"hegemonical"

"hegemonies"

"hegemony"

"hegira"

"hegiras"

"heifer"

"heifers"

"heigh"

"height"

"height father"

"height mother"

"heighten"

"heightened"

"heightening"

"heightens"

"heighth"

"heighths"

"heights"

"heil"

"heiled"

"heiling"

"heils"

"heinie"

"heinies"

"heinous"

"heinously"

"heinousness"

"heir"

"heirdom"

"heirdoms"

"heired"

"heiress"

"heiresses"

"heiring"

"heirless"

"heirloom"

"heirlooms"

"heirs"

"heirship"

"heirships"

"heist"

"heisted"

"heister"

"heisters"

"heisting"

"heists"

"hejira"

"hektare"

"hektares"

"helcology"

"held"

"helen"

"helical"

"helices"

"helicograph"

"helicoid"

"helicoidal"

"helicoids"

"helicon"

"helicons"

"helicopter"

"helicopters"

"helicopts"

"helio"

"heliocentric"

"heliocentrically"

"heliocentricity"

"heliograph"

"heliographs"

"heliology"

"heliometer"

"heliophobia"

"helios"

"helioscope"

"helioseismology"

"heliotherapies"

"heliotherapy"

"heliotrope"

"heliotropes"

"heliotropic"

"heliotropically"

"heliotropism"

"helipad"

"helipads"

"heliport"

"heliports"

"helistop"

"helistops"

"helium"

"heliums"

"helix"

"helixes"

"hell"

"hellbent"

"hellbox"

"hellboxes"

"hellcat"

"hellcats"

"hellebore"

"hellebores"

"helled"

"hellene"

"hellenes"

"hellenic"

"hellenism"

"hellenist"

"hellenistic"

"hellenists"

"Hellenomania"

"heller"

"hellers"

"hellfire"

"hellfires"

"hellgrammite"

"hellgrammites"

"hellhole"

"hellholes"

"helling"

"hellion"

"hellions"

"hellish"

"hellishly"

"hellishness"

"hello"

"helloed"

"helloes"

"helloing"

"hellos"

"hells"

"helluva"

"helm"

"helmed"

"helmet"

"helmeted"

"helmeting"

"helmets"

"helming"

"helminthology"

"helmless"

"helms"

"helmsman"

"helmsmen"

"helot"

"helotry"

"helots"

"help"

"help messages"

"help section"

"helpable"

"helped"

"helper"

"helpers"

"helpful"

"helpfully"

"helpfulness"

"helping"

"helpings"

"helpless"

"helplessly"

"helplessness"

"helpmate"

"helpmates"

"helpmeet"

"helpmeets"

"helps"

"helsinki"

"helve"

"helved"

"helves"

"helving"

"hem"

"hemacytometer"

"heman"

"hematic"

"hematin"

"hematinic"

"hematite"

"hematites"

"hematologic"

"hematological"

"hematologies"

"hematologist"

"hematologists"

"hematology"

"hematoma"

"hematomas"

"hematozoa"

"hematuria"

"heme"

"hemingway"

"hemiola"

"hemiolas"

"hemiplegic"

"hemisection"

"hemisphere"

"hemispheres"

"hemispheric"

"hemispherical"

"hemistich"

"hemistichs"

"hemline"

"hemlines"

"hemlock"

"hemlocks"

"hemmed"

"hemmer"

"hemmers"

"hemming"

"hemodialyses"

"hemodialysis"

"hemoglobin"

"hemoglobinic"

"hemogram"

"hemokonia"

"hemolyze"

"hemophilia"

"hemophiliac"

"hemophiliacs"

"hemophilic"

"hemorrhage"

"hemorrhaged"

"hemorrhages"

"hemorrhagic"

"hemorrhaging"

"hemorrhoid"

"hemorrhoidal"

"hemorrhoidectomies"

"hemorrhoidectomy"

"hemorrhoids"

"hemostat"

"hemostats"

"hemotoxin"

"hemp"

"hempen"

"hempier"

"hemps"

"hempseed"

"hempseeds"

"hempweed"

"hempweeds"

"hempy"

"hems"

"hemstitch"

"hemstitched"

"hemstitches"

"hemstitching"

"hen"

"henbane"

"henbanes"

"henbit"

"henbits"

"hence"

"henceforth"

"henceforward"

"henchman"

"henchmen"

"hencoop"

"hencoops"

"henhouse"

"henhouses"

"henna"

"hennaed"

"hennaing"

"hennas"

"henneries"

"hennery"

"henpeck"

"henpecked"

"henpecking"

"henpecks"

"henries"

"henry"

"henrys"

"hens"

"henting"

"hents"

"heortology"

"hep"

"heparin"

"hepatic"

"hepatica"

"hepaticas"

"hepatics"

"hepatitis"

"hepatize"

"hepatized"

"hepatizes"

"hepatology"

"hepburn"

"hepcat"

"hepcats"

"heptad"

"heptads"

"heptagon"

"heptagons"

"heptameter"

"heptameters"

"heptanes"

"heptarch"

"heptarchs"

"heptoses"

"her"

"herald"

"heralded"

"heraldic"

"heralding"

"heraldist"

"heraldists"

"heraldries"

"heraldry"

"heralds"

"herb"

"herbaceous"

"herbage"

"herbages"

"herbal"

"Herbal Medicine"

"herbal section"

"herbalist"

"herbalists"

"herbals"

"herbaria"

"herbarium"

"herbariums"

"herbert"

"herbicidal"

"herbicidally"

"herbicide"

"herbicides"

"herbier"

"herbivore"

"herbivores"

"herbivorous"

"herbivorously"

"herbless"

"herbs"

"herby"

"herculean"

"hercules"

"herculeses"

"herd"

"herded"

"herder"

"herders"

"herding"

"herdman"

"herdmen"

"herds"

"herdsman"

"herdsmen"

"herdswoman"

"herdswomen"

"here"

"hereabout"

"hereafter"

"hereat"

"hereby"

"hereditarily"

"hereditariness"

"hereditary"

"heredities"

"heredity"

"hereford"

"herefords"

"herein"

"hereinafter"

"hereinto"

"hereof"

"hereon"

"heres"

"heresies"

"heresiology"

"heresy"

"heretic"

"heretical"

"heretically"

"heretics"

"hereto"

"heretofore"

"heretrix"

"hereunder"

"hereunto"

"hereupon"

"herewith"

"heritabilities"

"heritability"

"heritable"

"heritably"

"heritage"

"heritages"

"heritor"

"heritors"

"heritrix"

"herman"

"hermaphrodism"

"hermaphrodite"

"hermaphrodites"

"hermaphroditic"

"hermaphroditically"

"hermaphroditism"

"hermeneutic"

"hermeneutical"

"hermeneutically"

"hermeneutics"

"hermes"

"hermetic"

"hermetical"

"hermetically"

"hermit"

"hermitage"

"hermitages"

"hermitic"

"hermitry"

"hermits"

"hernia"

"herniae"

"hernial"

"hernias"

"herniate"

"herniated"

"herniates"

"herniating"

"herniation"

"herniations"

"hero"

"heroes"

"heroic"

"heroical"

"heroically"

"heroicalness"

"heroics"

"heroin"

"heroine"

"heroines"

"heroinism"

"heroins"

"heroism"

"heroisms"

"heroize"

"heroized"

"heroizes"

"heroizing"

"heron"

"herons"

"heros"

"herpes"

"herpeses"

"herpetic"

"herpetologic"

"herpetological"

"herpetologist"

"herpetologists"

"herpetology"

"herpetophobia"

"herr"

"herring"

"herringbone"

"herringbones"

"herrings"

"hers"

"herself"

"hershey"

"hertz"

"hertzes"

"hesitance"

"hesitancies"

"hesitancy"

"hesitant"

"hesitantly"

"hesitate"

"hesitated"

"hesitater"

"hesitaters"

"hesitates"

"hesitating"

"hesitatingly"

"hesitation"

"hesitations"

"hesitator"

"hessian"

"hessians"

"hest"

"hetaera"

"hetaerae"

"hetaeras"

"hetaeric"

"hetero"

"heterodox"

"heterodoxies"

"heterodoxy"

"heteroerotic"

"heterogeneity"

"heterogeneous"

"heterogeneously"

"heterogeneousness"

"heterogenous"

"heteronomous"

"heteronomy"

"heteronymous"

"heterophile"

"heteros"

"heteroses"

"heterosexual"

"heterosexuality"

"heterosexually"

"heterosexuals"

"heterosis"

"heterotic"

"heuristic"

"heuristics"

"hew"

"hewable"

"hewed"

"hewer"

"hewers"

"hewing"

"hewn"

"hews"

"hex"

"hexad"

"hexadecimal"

"hexads"

"hexagon"

"hexagonal"

"hexagons"

"hexagram"

"hexagrams"

"hexahedra"

"hexahedral"

"hexahedron"

"hexahedrons"

"hexameter"

"hexameters"

"hexametromania"

"hexane"

"hexaploid"

"hexapod"

"hexapodies"

"hexapods"

"hexapody"

"hexarchies"

"hexed"

"hexer"

"hexers"

"hexes"

"hexing"

"hexone"

"hexose"

"hexyl"

"hexylresorcinol"

"hexyls"

"hey"

"heyday"

"heydays"

"heydey"

"heydeys"

"hi"

"hiatal"

"hiatus"

"hiatuses"

"hibachi"

"hibachis"

"hibernal"

"hibernate"

"hibernated"

"hibernates"

"hibernating"

"hibernation"

"hibernator"

"hibernators"

"hibiscus"

"hibiscuses"

"hic"

"hiccough"

"hiccoughed"

"hiccoughs"

"hiccup"

"hiccuped"

"hiccuping"

"hiccupped"

"hiccupping"

"hiccups"

"hick"

"hickey"

"hickeys"

"hickories"

"hickory"

"hicks"

"hid"

"hidable"

"hidalgo"

"hidalgos"

"hidden"

"hiddenly"

"hide"

"hide ads"

"hideaway"

"hideaways"

"hidebound"

"hided"

"hideless"

"hideous"

"hideously"

"hideousness"

"hideout"

"hideouts"

"hider"

"hiders"

"hides"

"hiding"

"hidings"

"hie"

"hied"

"hieing"

"hierarch"

"hierarchal"

"hierarchial"

"hierarchic"

"hierarchical"

"hierarchically"

"hierarchies"

"hierarchism"

"hierarchs"

"hierarchy"

"hieratic"

"hieratically"

"hieroglyphic"

"hieroglyphics"

"hierology"

"hieromania"

"hierophant"

"hierophants"

"hierophobia"

"hies"

"higgle"

"high"

"high blood sugar"

"high definition"

"High Maturity"

"high-dpi"

"high-resolution"

"highball"

"highballed"

"highballs"

"highbinder"

"highboard"

"highborn"

"highboy"

"highboys"

"highbred"

"highbrow"

"highbrows"

"higher"

"highest"

"highfalutin"

"highhanded"

"highhandedly"

"highhandedness"

"highhatting"

"highjack"

"highjacked"

"highjacks"

"highland"

"highlander"

"highlanders"

"highlands"

"highlight"

"highlighted"

"highlighting"

"highlights"

"highly"

"highness"

"highnesses"

"highroad"

"highroads"

"highs"

"highschool"

"hight"

"hightail"

"hightailed"

"hightailing"

"hightails"

"hightech"

"highted"

"highth"

"highths"

"highting"

"hights"

"highway"

"highwayman"

"highwaymen"

"highways"

"hijack"

"hijacked"

"hijacker"

"hijackers"

"hijacking"

"hijacks"

"hijinks"

"hike"

"hiked"

"hiker"

"hikers"

"hikes"

"hiking"

"hilarious"

"hilariously"

"hilariousness"

"hilarities"

"hilarity"

"hill"

"hillbillies"

"hillbilly"

"hilled"

"hiller"

"hillers"

"hillier"

"hilliest"

"hilliness"

"hilling"

"hillock"

"hillocks"

"hillocky"

"hills"

"hillside"

"hillsides"

"hilltop"

"hilltops"

"hilly"

"hilt"

"hilted"

"hilting"

"hiltless"

"hilts"

"him"

"himalayan"

"himalayas"

"himself"

"hind"

"hindbrain"

"hinder"

"hinderance"

"hindered"

"hinderer"

"hinderers"

"hindering"

"hindermost"

"hinders"

"hindgut"

"hindguts"

"hindi"

"hindmost"

"hindquarter"

"hindquarters"

"hindrance"

"hindrances"

"hinds"

"hindsight"

"hindu"

"hinduism"

"hindus"

"hindustan"

"hindustani"

"hinese"

"hinge"

"hinged"

"hingeless"

"hinger"

"hingers"

"hinges"

"hinging"

"hinnied"

"hinnies"

"hinny"

"hint"

"hinted"

"hinter"

"hinterland"

"hinterlands"

"hinters"

"hinting"

"hints"

"hip"

"hipbone"

"hipbones"

"hiphuggers"

"hipless"

"hipline"

"hipness"

"hipnesses"

"hipparchs"

"hipped"

"hipper"

"hippest"

"hippiatrics"

"hippie"

"hippiedom"

"hippier"

"hippies"

"hipping"

"hippish"

"hippo"

"hippocampus"

"hippocrates"

"hippocratic"

"hippocratism"

"hippodrome"

"hippodromes"

"hippology"

"hippomania"

"hippometer"

"hippopotami"

"hippopotamus"

"hippopotamuses"

"hippos"

"hippy"

"hips"

"hipshot"

"hipster"

"hipsters"

"hirable"

"hiragana"

"hiraganas"

"hire"

"hireable"

"hired"

"hireling"

"hirelings"

"hirer"

"hirers"

"hires"

"hiring"

"hiroshima"

"hirsute"

"hirsuteness"

"hirsutism"

"his"

"hisn"

"hispanic"

"hispanics"

"hispaniola"

"hispano"

"hispid"

"hiss"

"hissed"

"hisself"

"hisser"

"hissers"

"hisses"

"hissing"

"hissings"

"hist"

"histamin"

"histamine"

"histamines"

"histaminic"

"histamins"

"histed"

"histing"

"histogram"

"histograms"

"histologist"

"histologists"

"histology"

"histolysis"

"histolytic"

"histopathology"

"historian"

"historians"

"historic"

"historical"

"historical data"

"historically"

"historicity"

"histories"

"historiographer"

"historiographers"

"historiography"

"historiology"

"history"

"history of workouts"

"histrionic"

"histrionically"

"histrionics"

"hists"

"hit"

"hitch"

"hitched"

"hitcher"

"hitchers"

"hitches"

"hitchhike"

"hitchhiked"

"hitchhiker"

"hitchhikers"

"hitchhikes"

"hitchhiking"

"hitching"

"hither"

"hitherto"

"hitler"

"hitlerism"

"hitless"

"hits"

"hitter"

"hitters"

"hitting"

"hive"

"hived"

"hives"

"hiving"

"ho"

"hoagie"

"hoagies"

"hoagy"

"hoar"

"hoard"

"hoarded"

"hoarder"

"hoarders"

"hoarding"

"hoardings"

"hoards"

"hoarfrost"

"hoarfrosts"

"hoarier"

"hoariest"

"hoarily"

"hoariness"

"hoarse"

"hoarsely"

"hoarsen"

"hoarsened"

"hoarseness"

"hoarsening"

"hoarsens"

"hoarser"

"hoarsest"

"hoary"

"hoatzin"

"hoatzins"

"hoax"

"hoaxed"

"hoaxer"

"hoaxers"

"hoaxes"

"hoaxing"

"hob"

"hobbesian"

"hobbet"

"hobbies"

"hobbit"

"hobble"

"hobbled"

"hobbledehoy"

"hobbledehoys"

"hobbler"

"hobblers"

"hobbles"

"hobbling"

"hobby"

"hobbyhorse"

"hobbyhorses"

"hobbyist"

"hobbyists"

"hobgoblin"

"hobgoblins"

"hobnail"

"hobnailed"

"hobnails"

"hobnob"

"hobnobbed"

"hobnobbing"

"hobnobs"

"hobo"

"hoboed"

"hoboes"

"hoboing"

"hoboism"

"hoboisms"

"hobos"

"hobs"

"hoc"

"hock"

"hocked"

"hocker"

"hockers"

"hockey"

"hockeys"

"hocking"

"hocks"

"hockshop"

"hockshops"

"hocus"

"hocused"

"hocuses"

"hocusing"

"hocussed"

"hocusses"

"hocussing"

"hod"

"hodad"

"hodaddy"

"hodads"

"hodgepodge"

"hodgepodges"

"hodoscope"

"hods"

"hoe"

"hoecake"

"hoecakes"

"hoed"

"hoedown"

"hoedowns"

"hoeing"

"hoer"

"hoers"

"hoes"

"hog"

"hogan"

"hogans"

"hogback"

"hogbacks"

"hogfish"

"hogfishes"

"hogged"

"hogger"

"hoggers"

"hogging"

"hoggish"

"hoggishly"

"hoggs"

"hognose"

"hognoses"

"hognut"

"hognuts"

"hogs"

"hogshead"

"hogsheads"

"hogtie"

"hogtied"

"hogtieing"

"hogties"

"hogtying"

"hogwash"

"hogwashes"

"hogweed"

"hogweeds"

"hoi"

"hoise"

"hoist"

"hoisted"

"hoister"

"hoisters"

"hoisting"

"hoists"

"hoke"

"hokey"

"hokier"

"hokiest"

"hoking"

"hokum"

"hokums"

"hokypokies"

"hokypoky"

"hold"

"holdable"

"holdall"

"holdalls"

"holdback"

"holdbacks"

"holden"

"holder"

"holders"

"holdfast"

"holdfasts"

"holding"

"holdings"

"holdout"

"holdouts"

"holdover"

"holdovers"

"holds"

"holdup"

"holdups"

"hole"

"holed"

"holeless"

"holeproof"

"holer"

"holes"

"holey"

"holiday"

"holidayed"

"holidaying"

"holidays"

"holier"

"holies"

"holiest"

"holily"

"holiness"

"holing"

"holism"

"holisms"

"holist"

"holistic"

"holistic medicine"

"holistically"

"holists"

"holland"

"hollandaise"

"hollander"

"hollanders"

"hollands"

"holler"

"hollered"

"hollering"

"hollers"

"hollies"

"hollo"

"holloaing"

"hollooing"

"hollow"

"hollowed"

"hollower"

"hollowest"

"hollowing"

"hollowly"

"hollowness"

"hollows"

"hollowware"

"holly"

"hollyhock"

"hollyhocks"

"hollywood"

"holmes"

"holmium"

"holmiums"

"holocaust"

"holocausts"

"holocene"

"holocrine"

"hologram"

"holograms"

"holograph"

"holographic"

"holographies"

"holographs"

"holography"

"holotypes"

"holstein"

"holsteins"

"holster"

"holstered"

"holsters"

"holt"

"holts"

"holy"

"holyday"

"holydays"

"holystone"

"holystones"

"holytide"

"homage"

"homaged"

"homager"

"homagers"

"homages"

"homaging"

"hombre"

"hombres"

"homburg"

"homburgs"

"home"

"Home Remedies"

"home screen"

"homebodies"

"homebody"

"homebound"

"homebred"

"homebreds"

"homebuilders"

"homebuilding"

"homecoming"

"homecomings"

"homed"

"homefolk"

"homegrown"

"homeland"

"homelands"

"homeless"

"homelier"

"homeliest"

"homelike"

"homeliness"

"homely"

"homemade"

"homemaker"

"homemakers"

"homemaking"

"homeomorphous"

"homeopath"

"homeopathic"

"homeopathically"

"homeopathies"

"homeopathy"

"homeostases"

"homeostasis"

"homeostatic"

"homeowner"

"homeowners"

"homer"

"homeric"

"homering"

"homeroom"

"homerooms"

"homers"

"homes"

"homesick"

"homesickness"

"homesite"

"homespun"

"homespuns"

"homestead"

"homesteader"

"homesteaders"

"homesteads"

"homestretch"

"homestretches"

"hometown"

"hometowns"

"homeward"

"homework"

"homeworker"

"homeworks"

"homey"

"homeyness"

"homichlophobia"

"homicidal"

"homicidally"

"homicide"

"homicides"

"homier"

"homiest"

"homiletic"

"homiletics"

"homilies"

"homilist"

"homilists"

"homily"

"hominem"

"hominess"

"homing"

"hominid"

"hominidae"

"hominids"

"hominies"

"hominized"

"hominoid"

"hominoids"

"hominy"

"homo"

"homocentric"

"homoerotic"

"homoeroticism"

"homoerotism"

"homogeneity"

"homogeneous"

"homogeneously"

"homogeneousness"

"homogenization"

"homogenize"

"homogenized"

"homogenizer"

"homogenizers"

"homogenizes"

"homogenizing"

"homograph"

"homographic"

"homographs"

"homolog"

"homologies"

"homologous"

"homologue"

"homology"

"homonym"

"homonymic"

"homonymies"

"homonyms"

"homonymy"

"homophile"

"homophiles"

"homophobia"

"homophone"

"homophones"

"homos"

"homosexual"

"homosexuality"

"homosexually"

"homosexuals"

"homotype"

"homunculi"

"homy"

"hon"

"honan"

"honcho"

"honchos"

"honda"

"hondas"

"honduran"

"hondurans"

"honduras"

"hone"

"honed"

"honer"

"honers"

"hones"

"honest"

"honester"

"honestest"

"honesties"

"honestly"

"honestness"

"honesty"

"honeworts"

"honey"

"honeybee"

"honeybees"

"honeybun"

"honeybuns"

"honeycomb"

"honeycombed"

"honeycombs"

"honeydew"

"honeydews"

"honeyed"

"honeyful"

"honeying"

"honeymoon"

"honeymooned"

"honeymooner"

"honeymooners"

"honeymooning"

"honeymoons"

"honeys"

"honeysuckle"

"honeysuckles"

"hongkong"

"honied"

"honing"

"honk"

"honked"

"honker"

"honkers"

"honkey"

"honkeys"

"honkie"

"honkies"

"honking"

"honks"

"honky"

"honkytonks"

"honolulu"

"honor"

"honorable"

"honorableness"

"honorables"

"honorably"

"honorands"

"honoraria"

"honoraries"

"honorarily"

"honorarium"

"honorariums"

"honorary"

"honored"

"honoree"

"honorees"

"honorer"

"honorers"

"honorific"

"honorifically"

"honorifics"

"honoring"

"honorless"

"honors"

"honour"

"honoured"

"honourer"

"honourers"

"honouring"

"honours"

"hooch"

"hooches"

"hood"

"hooded"

"hooding"

"hoodless"

"hoodlum"

"hoodlums"

"hoodoo"

"hoodooed"

"hoodooing"

"hoodoos"

"hoods"

"hoodwink"

"hoodwinked"

"hoodwinking"

"hoodwinks"

"hooey"

"hooeys"

"hoof"

"hoofbeat"

"hoofbeats"

"hoofbound"

"hoofed"

"hoofer"

"hoofers"

"hoofing"

"hoofless"

"hoofmarks"

"hoofs"

"hook"

"hooka"

"hookah"

"hookahs"

"hookas"

"hooked"

"hookedness"

"hooker"

"hookers"

"hookey"

"hookeys"

"hookier"

"hookies"

"hooking"

"hookless"

"hooklets"

"hooknose"

"hooknoses"

"hooks"

"hookup"

"hookups"

"hookworm"

"hookworms"

"hooky"

"hooligan"

"hooliganism"

"hooligans"

"hoop"

"hooped"

"hooper"

"hoopers"

"hooping"

"hoopla"

"hooplas"

"hoopless"

"hoops"

"hoopster"

"hoopsters"

"hoorah"

"hoorahed"

"hoorahing"

"hoorahs"

"hooray"

"hoorayed"

"hooraying"

"hoorays"

"hoosegow"

"hoosegows"

"hoosgow"

"hoosgows"

"hoosier"

"hoosiers"

"hoot"

"hootch"

"hootches"

"hooted"

"hootenannies"

"hootenanny"

"hooter"

"hooters"

"hooting"

"hoots"

"hoover"

"hooves"

"hop"

"hope"

"hoped"

"hopeful"

"hopefully"

"hopefulness"

"hopefuls"

"hopeless"

"hopelessly"

"hopelessness"

"hoper"

"hopers"

"hopes"

"hophead"

"hopheads"

"hopi"

"hoping"

"hopis"

"hoplite"

"hoplology"

"hopped"

"hopper"

"hoppers"

"hopping"

"hops"

"hopsack"

"hopsacking"

"hopsacks"

"hopscotch"

"hoptoad"

"hoptoads"

"hor"

"hora"

"horace"

"horah"

"horal"

"horary"

"horas"

"horde"

"horded"

"hordes"

"hording"

"horehound"

"horehounds"

"horizon"

"horizons"

"horizontal"

"horizontally"

"hormonal"

"hormonally"

"hormone"

"hormones"

"hormonic"

"horn"

"hornbeam"

"hornbill"

"hornbills"

"hornbook"

"hornbooks"

"horned"

"horner"

"hornet"

"hornets"

"hornier"

"horniest"

"hornily"

"horning"

"hornless"

"hornlike"

"hornpipe"

"hornpipes"

"horns"

"hornswoggle"

"hornswoggled"

"hornswoggling"

"horny"

"horography"

"horologe"

"horologes"

"horological"

"horologies"

"horologist"

"horologists"

"horology"

"horoscope"

"horoscopes"

"horrendous"

"horrendously"

"horrible"

"horribleness"

"horribles"

"horribly"

"horrid"

"horridly"

"horridness"

"horrific"

"horrified"

"horrifies"

"horrify"

"horrifying"

"horripilation"

"horror"

"horrors"

"hors"

"horse"

"horseback"

"horsecar"

"horsed"

"horsefeathers"

"horseflesh"

"horseflies"

"horsefly"

"horsehair"

"horsehide"

"horsehides"

"horselaugh"

"horselaughs"

"horseless"

"horseman"

"horsemanship"

"horsemen"

"horseplay"

"horseplayer"

"horseplayers"

"horsepower"

"horsepowers"

"horsepox"

"horseradish"

"horseradishes"

"horses"

"horseshoe"

"horseshoer"

"horseshoers"

"horseshoes"

"horsetail"

"horsetails"

"horsewhip"

"horsewhipped"

"horsewhipping"

"horsewhips"

"horsewoman"

"horsewomen"

"horsey"

"horsier"

"horsiest"

"horsily"

"horsing"

"horst"

"horsy"

"hortative"

"hortatory"

"horticultural"

"horticulture"

"horticulturist"

"horticulturists"

"hosanna"

"hosannaed"

"hosannas"

"hose"

"hosed"

"hoses"

"hosier"

"hosieries"

"hosiers"

"hosiery"

"hosing"

"hosp"

"hospice"

"hospices"

"hospitable"

"hospitableness"

"hospitably"

"hospital"

"Hospital Management"

"hospitalism"

"hospitalities"

"hospitality"

"hospitalization"

"hospitalizations"

"hospitalize"

"hospitalized"

"hospitalizes"

"hospitalizing"

"hospitals"

"hospitium"

"host"

"hostage"

"hostages"

"hosted"

"hostel"

"hosteled"

"hosteler"

"hostelers"

"hosteling"

"hostelries"

"hostelry"

"hostels"

"hostess"

"hostessed"

"hostesses"

"hostessing"

"hostile"

"hostilely"

"hostiles"

"hostilities"

"hostility"

"hosting"

"hostler"

"hostlers"

"hostly"

"hosts"

"hot"

"hotbed"

"hotbeds"

"hotblood"

"hotblooded"

"hotbox"

"hotboxes"

"hotcake"

"hotcakes"

"hotchpotch"

"hotdog"

"hotdogged"

"hotdogging"

"hotdogs"

"hotel"

"hotelier"

"hoteliers"

"hotelkeeper"

"hotelman"

"hotelmen"

"hotels"

"hotfoot"

"hotfooted"

"hotfooting"

"hotfoots"

"hothead"

"hotheaded"

"hotheadedly"

"hotheadedness"

"hotheads"

"hothouse"

"hothouses"

"hotkey"

"hotline"

"hotly"

"hotness"

"hotnesses"

"hotrod"

"hotrods"

"hots"

"hotshot"

"hotshots"

"hotspur"

"hotspurs"

"hotted"

"hotter"

"hottest"

"hotting"

"hottish"

"hotzone"

"hound"

"hounded"

"hounder"

"hounders"

"hounding"

"hounds"

"hour"

"hourglass"

"hourglasses"

"houri"

"houris"

"hourly"

"hours"

"house"

"houseboat"

"houseboats"

"houseboy"

"houseboys"

"housebreak"

"housebreaker"

"housebreakers"

"housebreaking"

"housebroken"

"houseclean"

"housecleaned"

"housecleaning"

"housecleans"

"housecoat"

"housecoats"

"housed"

"houseflies"

"housefly"

"houseful"

"housefuls"

"household"

"householder"

"householders"

"households"

"househusband"

"househusbands"

"housekeeper"

"housekeepers"

"housekeeping"

"houseless"

"houselights"

"housemaid"

"housemaids"

"houseman"

"housemaster"

"housemen"

"housemother"

"housemothers"

"housepaint"

"houser"

"housers"

"houses"

"housesat"

"housesit"

"housesits"

"housesitting"

"housetop"

"housetops"

"housewares"

"housewarming"

"housewarmings"

"housewife"

"housewifeliness"

"housewifely"

"housewifery"

"housewives"

"housework"

"houseworker"

"houseworkers"

"housing"

"housings"

"houston"

"hove"

"hovel"

"hovelling"

"hovels"

"hover"

"hovercraft"

"hovercrafts"

"hovered"

"hoverer"

"hoverers"

"hovering"

"hovers"

"how"

"howbeit"

"howdah"

"howdahs"

"howdie"

"howdies"

"howdy"

"howe"

"howes"

"however"

"howitzer"

"howitzers"

"howl"

"howled"

"howler"

"howlers"

"howlet"

"howling"

"howls"

"hows"

"howsabout"

"howsoever"

"hoy"

"hoyden"

"hoydening"

"hoydens"

"hoyle"

"hoyles"

"hp"

"hr"

"hristian"

"hristianity"

"hristmas"

"hrs"

"hts"

"huarache"

"huaraches"

"hub"

"hubbies"

"hubbub"

"hubbubs"

"hubby"

"hubcap"

"hubcaps"

"hubris"

"hubrises"

"hubs"

"huck"

"huckleberries"

"huckleberry"

"hucks"

"huckster"

"huckstered"

"huckstering"

"hucksters"

"huddle"

"huddled"

"huddler"

"huddlers"

"huddles"

"huddling"

"hudson"

"hue"

"hued"

"hueless"

"hues"

"huff"

"huffed"

"huffier"

"huffiest"

"huffily"

"huffiness"

"huffing"

"huffish"

"huffs"

"huffy"

"hug"

"huge"

"hugely"

"hugeness"

"huger"

"hugest"

"huggable"

"hugged"

"hugger"

"huggermugger"

"huggers"

"hugging"

"hugs"

"huguenot"

"huguenots"

"huh"

"hula"

"hulas"

"hulk"

"hulked"

"hulkier"

"hulking"

"hulks"

"hulky"

"hull"

"hullabaloo"

"hulled"

"huller"

"hullers"

"hulling"

"hullo"

"hulloaed"

"hulloaing"

"hulloed"

"hulloes"

"hulloing"

"hullos"

"hulls"

"hum"

"human"

"human body"

"human body works"

"human brain"

"human ear"

"human value"

"humane"

"humanely"

"humaneness"

"humaner"

"humanest"

"humanism"

"humanisms"

"humanist"

"humanistic"

"humanistically"

"humanists"

"humanitarian"

"humanitarianism"

"humanitarians"

"humanities"

"humanity"

"humanization"

"humanize"

"humanized"

"humanizer"

"humanizers"

"humanizes"

"humanizing"

"humankind"

"humanly"

"humanness"

"humanoid"

"humanoids"

"humans"

"humble"

"humbled"

"humbleness"

"humbler"

"humblers"

"humbles"

"humblest"

"humbling"

"humbly"

"humbug"

"humbugged"

"humbugger"

"humbuggers"

"humbugging"

"humbugs"

"humdinger"

"humdingers"

"humdrum"

"humdrums"

"humectant"

"humeral"

"humeri"

"humerus"

"humid"

"humidfied"

"humidfies"

"humidification"

"humidified"

"humidifier"

"humidifiers"

"humidifies"

"humidify"

"humidifying"

"humidistat"

"humidities"

"humidity"

"humidly"

"humidor"

"humidors"

"humiliate"

"humiliated"

"humiliates"

"humiliating"

"humiliatingly"

"humiliation"

"humiliations"

"humilities"

"humility"

"hummable"

"hummed"

"hummer"

"hummers"

"humming"

"hummingbird"

"hummingbirds"

"hummock"

"hummocks"

"hummocky"

"humongous"

"humor"

"humoral"

"humored"

"humorer"

"humorers"

"humorful"

"humoring"

"humorist"

"humorists"

"humorless"

"humorlessly"

"humorlessness"

"humorous"

"humorously"

"humorousness"

"humors"

"humour"

"humoured"

"humouring"

"humours"

"hump"

"humpback"

"humpbacked"

"humpbacks"

"humped"

"humph"

"humphed"

"humphing"

"humphs"

"humpier"

"humping"

"humps"

"humpy"

"hums"

"humus"

"humuses"

"hun"

"hunch"

"hunchback"

"hunchbacked"

"hunchbacks"

"hunched"

"hunches"

"hunching"

"hundred"

"hundredfold"

"hundreds"

"hundredth"

"hundredths"

"hundredweight"

"hundredweights"

"hung"

"hungarian"

"hungarians"

"hungary"

"hunger"

"hungered"

"hungering"

"hungerless"

"hungers"

"hungrier"

"hungriest"

"hungrily"

"hungry"

"hunk"

"hunker"

"hunkered"

"hunkering"

"hunkers"

"hunks"

"hunky"

"hunnish"

"hunnishness"

"huns"

"hunt"

"huntable"

"hunted"

"huntedly"

"hunter"

"hunters"

"hunting"

"huntings"

"huntley"

"huntress"

"huntresses"

"hunts"

"huntsman"

"huntsmen"

"hup"

"hurdle"

"hurdled"

"hurdler"

"hurdlers"

"hurdles"

"hurdling"

"hurl"

"hurled"

"hurler"

"hurlers"

"hurling"

"hurlings"

"hurls"

"hurly"

"huron"

"hurrah"

"hurrahed"

"hurrahing"

"hurrahs"

"hurray"

"hurrayed"

"hurraying"

"hurrays"

"hurricane"

"hurricanes"

"hurried"

"hurriedly"

"hurriedness"

"hurrier"

"hurriers"

"hurries"

"hurry"

"hurrying"

"hurt"

"hurter"

"hurters"

"hurtful"

"hurting"

"hurtle"

"hurtled"

"hurtles"

"hurtless"

"hurtling"

"hurts"

"husband"

"husbanded"

"husbander"

"husbanding"

"husbandlike"

"husbandly"

"husbandman"

"husbandmen"

"husbandry"

"husbands"

"hush"

"hushaby"

"hushed"

"hushedly"

"hushes"

"hushful"

"hushing"

"husk"

"husked"

"husker"

"huskers"

"huskier"

"huskies"

"huskiest"

"huskily"

"huskiness"

"husking"

"huskings"

"husks"

"husky"

"hussar"

"hussars"

"hussies"

"hussy"

"hustings"

"hustle"

"hustled"

"hustler"

"hustlers"

"hustles"

"hustling"

"hut"

"hutch"

"hutched"

"hutches"

"hutching"

"hutment"

"hutments"

"huts"

"hutted"

"hutting"

"hutzpa"

"hutzpah"

"hutzpahs"

"hutzpas"

"huzza"

"huzzaed"

"huzzah"

"huzzahed"

"huzzahing"

"huzzahs"

"huzzaing"

"huzzas"

"hwy"

"hyacinth"

"hyacinthine"

"hyacinths"

"hyaena"

"hyaenas"

"hyaenic"

"hybrid"

"hybridism"

"hybridization"

"hybridizations"

"hybridize"

"hybridized"

"hybridizer"

"hybridizers"

"hybridizes"

"hybridizing"

"hybrids"

"hyde"

"hydra"

"hydrae"

"hydrangea"

"hydrangeas"

"hydrant"

"hydrants"

"hydrargyrum"

"hydras"

"hydrate"

"hydrated"

"hydrates"

"hydrating"

"hydration"

"hydrations"

"hydrator"

"hydrators"

"hydraulic"

"hydraulically"

"hydraulics"

"hydric"

"hydride"

"hydrides"

"hydro"

"hydrobiology"

"hydrocarbon"

"hydrocarbons"

"hydrocephali"

"hydrocephalic"

"hydrocephalies"

"hydrocephaloid"

"hydrocephalus"

"hydrocephaly"

"hydrochloric"

"hydrochloride"

"hydrodynamic"

"hydrodynamics"

"hydroelectric"

"hydroelectricity"

"hydrofluoric"

"hydrofoil"

"hydrofoils"

"hydrogen"

"hydrogenate"

"hydrogenated"

"hydrogenates"

"hydrogenating"

"hydrogenation"

"hydrogenations"

"hydrogenous"

"hydrogens"

"hydrogeology"

"hydrographer"

"hydrographers"

"hydrographic"

"hydrography"

"hydrokinetics"

"hydrologic"

"hydrological"

"hydrologist"

"hydrologists"

"hydrology"

"hydrolyses"

"hydrolysis"

"hydrolytic"

"hydrolyze"

"hydromania"

"hydromassage"

"hydrometeorology"

"hydrometer"

"hydrometers"

"hydropathically"

"hydropathy"

"hydrophobia"

"hydrophobic"

"hydrophobicity"

"hydrophone"

"hydrophones"

"hydroplane"

"hydroplanes"

"hydroponic"

"hydroponics"

"hydropower"

"hydros"

"hydroscope"

"hydrosphere"

"hydrospheres"

"hydrostatic"

"hydrostatical"

"hydrostatics"

"hydrotherapeutic"

"hydrotherapeutical"

"hydrotherapeutically"

"hydrotherapeutician"

"hydrotherapeuticians"

"hydrotherapeutics"

"hydrotherapies"

"hydrotherapist"

"hydrotherapy"

"hydrothermal"

"hydrothermally"

"hydrotimeter"

"hydrotropism"

"hydrous"

"hydroxide"

"hydroxides"

"hydroxy"

"hydrozoan"

"hydrozoon"

"hyena"

"hyenas"

"hyetograph"

"hyetology"

"hyetometer"

"hyetometrograph"

"hygeist"

"hygiastics"

"hygieists"

"hygiene"

"hygienes"

"hygienic"

"hygienical"

"hygienically"

"hygienics"

"hygienist"

"hygienists"

"hygiology"

"hygrograph"

"hygrology"

"hygrometer"

"hygrometers"

"hygrometries"

"hygrometry"

"hygroscope"

"hygroscopic"

"hying"

"hylomania"

"hymen"

"hymenal"

"hymeneal"

"hymenoptera"

"hymenopteran"

"hymenopteron"

"hymenopterous"

"hymens"

"hymn"

"hymnal"

"hymnals"

"hymnaries"

"hymnary"

"hymnbook"

"hymnbooks"

"hymned"

"hymning"

"hymnist"

"hymnists"

"hymnodies"

"hymnody"

"hymnography"

"hymnology"

"hymns"

"hyoglossi"

"hype"

"hyped"

"hyper"

"hyperacid"

"hyperacidity"

"hyperactive"

"hyperactivities"

"hyperactivity"

"hyperbaric"

"hyperbarically"

"hyperbola"

"hyperbolas"

"hyperbole"

"hyperboles"

"hyperbolic"

"hyperbolically"

"hyperborean"

"hypercritical"

"hypercritically"

"hyperexcitable"

"hyperextension"

"hyperglycemia"

"hyperglycemic"

"hypericum"

"hyperinflation"

"hyperion"

"hyperirritable"

"hyperkinesia"

"hyperkinesis"

"hyperkinetic"

"hypermania"

"hyperons"

"hyperopia"

"hyperopic"

"hyperpituitary"

"hypersensitive"

"hypersensitiveness"

"hypersensitivities"

"hypersensitivity"

"hypersensitize"

"hypersensitized"

"hypersensitizing"

"hypersexual"

"hypersexualities"

"hypersexuality"

"hypersonic"

"hypertension"

"hypertensive"

"hyperthyroid"

"hyperthyroidism"

"hyperthyroids"

"hypertonicity"

"hypertrophic"

"hypertrophied"

"hypertrophies"

"hypertrophy"

"hypertrophying"

"hyperventilation"

"hypes"

"hyphen"

"hyphenate"

"hyphenated"

"hyphenates"

"hyphenating"

"hyphenation"

"hyphenations"

"hyphened"

"hyphening"

"hyphens"

"hyping"

"hypnic"

"hypnoanalyses"

"hypnoanalysis"

"hypnogogic"

"hypnoid"

"hypnoidal"

"hypnology"

"hypnophobia"

"hypnophobias"

"hypnoses"

"hypnosis"

"hypnosis technologies"

"Hypnotherapist"

"hypnotherapy"

"hypnotherapy session"

"hypnotic"

"hypnotic sessions"

"hypnotically"

"hypnotics"

"hypnotism"

"hypnotist"

"hypnotists"

"hypnotizable"

"hypnotize"

"hypnotized"

"hypnotizes"

"hypnotizing"

"hypo"

"hypocenter"

"hypocenters"

"hypochondria"

"hypochondriac"

"hypochondriacal"

"hypochondriacs"

"hypochondriasis"

"hypocrisies"

"hypocrisy"

"hypocrite"

"hypocrites"

"hypocritic"

"hypocritical"

"hypocritically"

"hypoderm"

"hypodermatically"

"hypodermic"

"hypodermically"

"hypodermics"

"hypoed"

"hypoergic"

"hypoglycemia"

"hypoglycemic"

"hypoing"

"hypomania"

"hypos"

"hyposensitive"

"hyposensitivity"

"hyposensitize"

"hyposensitized"

"hyposensitizing"

"hypotension"

"hypotensive"

"hypotenuse"

"hypotenuses"

"hypothecate"

"hypothecated"

"hypothecates"

"hypothecating"

"hypothermal"

"hypothermia"

"hypothermic"

"hypotheses"

"hypothesi"

"hypothesis"

"hypothesist"

"hypothesists"

"hypothesize"

"hypothesized"

"hypothesizer"

"hypothesizers"

"hypothesizes"

"hypothesizing"

"hypothetical"

"hypothetically"

"hypothyroid"

"hypothyroidism"

"hypothyroids"

"hypotonic"

"hypotrophies"

"hypoxemia"

"hypoxemic"

"hypoxia"

"hypoxic"

"hypsography"

"hypsometer"

"hypsophobia"

"hyrax"

"hyraxes"

"hyson"

"hysons"

"hyssop"

"hyssops"

"hysterectomies"

"hysterectomize"

"hysterectomized"

"hysterectomizes"

"hysterectomizing"

"hysterectomy"

"hysteria"

"hysterias"

"hysteric"

"hysterical"

"hysterically"

"hysterics"

"hystericus"

"hysteromania"

"i"

"iamatology"

"iamb"

"iambi"

"iambic"

"iambics"

"iambs"

"iambus"

"iambuses"

"iatrogenic"

"iatrology"

"iatromathematics"

"iatrophobia"

"iberia"

"iberian"

"iberians"

"ibex"

"ibexes"

"ibices"

"ibid"

"ibidem"

"ibis"

"ibises"

"ible"

"ibm"

"ic"

"icbm"

"ice"

"Ice Cream Sandwich"

"iceberg"

"icebergs"

"iceboat"

"iceboats"

"icebound"

"icebox"

"iceboxes"

"icebreaker"

"icebreakers"

"icecap"

"icecaps"

"iced"

"icefall"

"icefalls"

"icehouse"

"icehouses"

"iceland"

"icelander"

"icelanders"

"icelandic"

"iceless"

"iceman"

"icemen"

"ices"

"ichnography"

"ichnology"

"ichor"

"ichorous"

"ichors"

"ichthyic"

"ichthyism"

"ichthyisms"

"ichthyoid"

"ichthyologist"

"ichthyologists"

"ichthyology"

"ichthyophagous"

"ichthyosiform"

"icicle"

"icicled"

"icicles"

"icier"

"iciest"

"icily"

"iciness"

"icinesses"

"icing"

"icings"

"icker"

"ickier"

"ickiest"

"icky"

"icon"

"iconic"

"iconical"

"iconoclasm"

"iconoclast"

"iconoclastic"

"iconoclasts"

"iconography"

"iconology"

"iconomania"

"iconometer"

"iconophobia"

"icons"

"ICS"

"ictus"

"ictuses"

"icy"

"id"

"idaho"

"idahoan"

"idahoans"

"idea"

"ideal"

"ideal weight"

"idealism"

"idealisms"

"idealist"

"idealistic"

"idealistically"

"idealists"

"idealities"

"ideality"

"idealization"

"idealizations"

"idealize"

"idealized"

"idealizes"

"idealizing"

"ideally"

"idealogies"

"idealogue"

"idealogy"

"ideals"

"ideas"

"ideate"

"ideated"

"ideates"

"ideation"

"ideational"

"ideations"

"idee"

"idem"

"identical"

"identically"

"identicalness"

"identifer"

"identifers"

"identifiability"

"identifiable"

"identifiably"

"identification"

"identification data"

"identifications"

"identified"

"identifier"

"identifiers"

"identifies"

"identify"

"identities"

"identity"

"ideo"

"ideogenetic"

"ideogeny"

"ideogram"

"ideograms"

"ideograph"

"ideographs"

"ideokinetic"

"ideologic"

"ideological"

"ideologically"

"ideologies"

"ideologist"

"ideologize"

"ideologized"

"ideologizing"

"ideologue"

"ideology"

"ideomotor"

"ides"

"idiocies"

"idiocratic"

"idiocy"

"idiogram"

"idiom"

"idiomatic"

"idiomatically"

"idiometer"

"idiomology"

"idioms"

"idiopathic"

"idiopathy"

"idiopsychology"

"idiosyncracies"

"idiosyncracy"

"idiosyncrasies"

"idiosyncrasy"

"idiosyncratic"

"idiot"

"idiotic"

"idiotical"

"idiotically"

"idiotisms"

"idiots"

"idle"

"idled"

"idleness"

"idler"

"idlers"

"idles"

"idlesses"

"idlest"

"idling"

"idly"

"idol"

"idolater"

"idolaters"

"idolatries"

"idolatrous"

"idolatry"

"idolise"

"idolised"

"idoliser"

"idolises"

"idolism"

"idolisms"

"idolization"

"idolize"

"idolized"

"idolizer"

"idolizers"

"idolizes"

"idolizing"

"idolomania"

"idols"

"ids"

"idyl"

"idylist"

"idylists"

"idyll"

"idyllic"

"idyllist"

"idyllists"

"idylls"

"idyls"

"ie"

"ieee"

"if"

"ife"

"iff"

"iffier"

"iffiest"

"iffiness"

"iffy"

"ifs"

"igloo"

"igloos"

"igneous"

"ignified"

"ignifies"

"ignifying"

"ignis"

"ignitable"

"ignite"

"ignited"

"igniter"

"igniters"

"ignites"

"ignitible"

"igniting"

"ignition"

"ignitions"

"ignitors"

"ignobility"

"ignoble"

"ignobly"

"ignominies"

"ignominious"

"ignominiously"

"ignominy"

"ignoramus"

"ignoramuses"

"ignorance"

"ignorant"

"ignorantly"

"ignorantness"

"ignore"

"ignored"

"ignorer"

"ignorers"

"ignores"

"ignoring"

"iguana"

"iguanas"

"iguanians"

"ii"

"iii"

"ikebana"

"ikebanas"

"ikon"

"ikons"

"ileal"

"ileitis"

"ileum"

"ilia"

"iliad"

"iliads"

"ilium"

"ilk"

"ilks"

"ill"

"illegal"

"illegalities"

"illegality"

"illegalization"

"illegalize"

"illegalized"

"illegalizing"

"illegally"

"illegibility"

"illegible"

"illegibly"

"illegitimacies"

"illegitimacy"

"illegitimate"

"illegitimated"

"illegitimately"

"illegitimating"

"illegitimation"

"iller"

"illest"

"illiberal"

"illicit"

"illicitly"

"illicitness"

"illimitable"

"illimitably"

"illinois"

"illinoisan"

"illiteracies"

"illiteracy"

"illiterate"

"illiterately"

"illiterateness"

"illiterates"

"illness"

"illnesses"

"illogic"

"illogical"

"illogicality"

"illogically"

"illogics"

"ills"

"illume"

"illumed"

"illumes"

"illuminable"

"illuminance"

"illuminate"

"illuminated"

"illuminates"

"illuminating"

"illuminatingly"

"illumination"

"illuminations"

"illuminative"

"illuminator"

"illuminators"

"illumine"

"illumined"

"illumines"

"illuming"

"illumining"

"illuminist"

"illusion"

"illusional"

"illusionary"

"illusionism"

"illusionist"

"illusionists"

"illusions"

"illusive"

"illusiveness"

"illusory"

"illustrate"

"illustrated"

"illustrated instruction"

"illustrates"

"illustrating"

"illustration"

"illustrations"

"illustrative"

"illustratively"

"illustrator"

"illustrators"

"illustrious"

"illustriously"

"illustriousness"

"illy"

"image"

"imaged"

"imageries"

"imagery"

"images"

"imaginable"

"imaginably"

"imaginal"

"imaginarily"

"imaginary"

"imagination"

"imaginations"

"imaginative"

"imaginatively"

"imagine"

"imagined"

"imaginer"

"imaginers"

"imagines"

"imaging"

"imagining"

"imaginings"

"imagism"

"imagisms"

"imagist"

"imagists"

"imago"

"imagoes"

"imam"

"imamates"

"imams"

"imaums"

"imbalance"

"imbalances"

"imbalm"

"imbalmed"

"imbalmer"

"imbalmers"

"imbalming"

"imbark"

"imbarked"

"imbecile"

"imbeciles"

"imbecilic"

"imbecilities"

"imbecility"

"imbed"

"imbedded"

"imbedding"

"imbeds"

"imbibe"

"imbibed"

"imbiber"

"imbibers"

"imbibes"

"imbibing"

"imbibition"

"imbibitional"

"imbibitions"

"imbody"

"imbricate"

"imbrication"

"imbrications"

"imbrium"

"imbroglio"

"imbroglios"

"imbrue"

"imbrued"

"imbrues"

"imbruing"

"imbue"

"imbued"

"imbues"

"imbuing"

"imburse"

"imitable"

"imitate"

"imitated"

"imitatee"

"imitates"

"imitating"

"imitation"

"imitational"

"imitations"

"imitative"

"imitatively"

"imitativeness"

"imitator"

"imitators"

"immaculacy"

"immaculate"

"immaculately"

"immaculateness"

"immanence"

"immanency"

"immanent"

"immanently"

"immaterial"

"immaterialities"

"immateriality"

"immaterially"

"immaterialness"

"immature"

"immaturely"

"immatures"

"immaturities"

"immaturity"

"immeasurable"

"immeasurably"

"immediacies"

"immediacy"

"immediate"

"immediately"

"immediateness"

"immedicable"

"immemorial"

"immemorially"

"immense"

"immensely"

"immenser"

"immensest"

"immensities"

"immensity"

"immerge"

"immerse"

"immersed"

"immerses"

"immersing"

"immersion"

"immersions"

"immesh"

"immeshing"

"immies"

"immigrant"

"immigrants"

"immigrate"

"immigrated"

"immigrates"

"immigrating"

"immigration"

"immigrations"

"imminence"

"imminent"

"imminently"

"immiscibility"

"immiscible"

"immitigable"

"immix"

"immixed"

"immixes"

"immixing"

"immobile"

"immobilities"

"immobility"

"immobilization"

"immobilize"

"immobilized"

"immobilizer"

"immobilizes"

"immobilizing"

"immoderacy"

"immoderate"

"immoderately"

"immoderateness"

"immoderation"

"immodest"

"immodestly"

"immodesty"

"immolate"

"immolated"

"immolates"

"immolating"

"immolation"

"immolations"

"immoral"

"immoralities"

"immorality"

"immorally"

"immortal"

"immortalities"

"immortality"

"immortalize"

"immortalized"

"immortalizes"

"immortalizing"

"immortally"

"immortals"

"immotile"

"immotility"

"immovability"

"immovable"

"immovably"

"immoveable"

"immune"

"immune function"

"immunes"

"immunities"

"immunity"

"immunization"

"immunizations"

"immunize"

"immunized"

"immunizes"

"immunizing"

"immunochemistry"

"immunogen"

"immunogenetics"

"immunoglobulin"

"immunologic"

"immunological"

"immunologically"

"immunologies"

"immunologist"

"immunologists"

"immunology"

"immunopathology"

"immunoreactive"

"immunosuppressant"

"immunosuppressants"

"immunosuppressive"

"immunotherapies"

"immunotherapy"

"immure"

"immured"

"immures"

"immuring"

"immutability"

"immutable"

"immutableness"

"immutably"

"imp"

"impact"

"impacted"

"impacter"

"impacters"

"impacting"

"impaction"

"impactor"

"impactors"

"impacts"

"impainted"

"impair"

"impaired"

"impairer"

"impairers"

"impairing"

"impairment"

"impairments"

"impairs"

"impala"

"impalas"

"impale"

"impaled"

"impalement"

"impalements"

"impaler"

"impalers"

"impales"

"impaling"

"impalpability"

"impalpable"

"impalpably"

"impanel"

"impaneled"

"impaneling"

"impanelled"

"impanelling"

"impanels"

"imparity"

"impart"

"imparted"

"imparter"

"imparters"

"impartial"

"impartiality"

"impartially"

"impartialness"

"impartible"

"impartibly"

"imparting"

"imparts"

"impassability"

"impassable"

"impasse"

"impasses"

"impassibility"

"impassible"

"impassibly"

"impassion"

"impassionate"

"impassioned"

"impassioning"

"impassive"

"impassively"

"impassiveness"

"impassivity"

"impasto"

"impatience"

"impatiens"

"impatient"

"impatiently"

"impeach"

"impeachable"

"impeached"

"impeacher"

"impeachers"

"impeaches"

"impeaching"

"impeachment"

"impeachments"

"impearl"

"impearled"

"impearling"

"impearls"

"impeccability"

"impeccable"

"impeccably"

"impecuniosity"

"impecunious"

"impecuniously"

"impecuniousness"

"imped"

"impedance"

"impedances"

"impede"

"impeded"

"impeder"

"impeders"

"impedes"

"impedient"

"impediment"

"impedimenta"

"impediments"

"impeding"

"impel"

"impelled"

"impeller"

"impellers"

"impelling"

"impellor"

"impellors"

"impels"

"impend"

"impended"

"impending"

"impends"

"impenetrability"

"impenetrable"

"impenetrableness"

"impenetrably"

"impenitence"

"impenitent"

"impenitently"

"imper"

"imperative"

"imperatively"

"imperatives"

"imperceivable"

"imperceptibility"

"imperceptible"

"imperceptibleness"

"imperceptibly"

"imperception"

"imperceptive"

"imperceptiveness"

"impercipient"

"imperfect"

"imperfectability"

"imperfection"

"imperfections"

"imperfectly"

"imperfectness"

"imperfects"

"imperforate"

"imperforates"

"imperia"

"imperial"

"imperialism"

"imperialist"

"imperialistic"

"imperialists"

"imperially"

"imperialness"

"imperials"

"imperii"

"imperil"

"imperiled"

"imperiling"

"imperilled"

"imperilling"

"imperilment"

"imperilments"

"imperils"

"imperious"

"imperiously"

"imperiousness"

"imperishable"

"imperishably"

"imperium"

"imperiums"

"impermanence"

"impermanent"

"impermanently"

"impermeabilities"

"impermeability"

"impermeable"

"impermeably"

"impermissible"

"impersonal"

"impersonality"

"impersonalize"

"impersonalized"

"impersonally"

"impersonate"

"impersonated"

"impersonates"

"impersonating"

"impersonation"

"impersonations"

"impersonator"

"impersonators"

"impertinence"

"impertinences"

"impertinencies"

"impertinency"

"impertinent"

"impertinently"

"imperturbability"

"imperturbable"

"imperturbably"

"impervious"

"imperviously"

"imperviousness"

"impetigo"

"impetigos"

"impetuosity"

"impetuous"

"impetuously"

"impetuousness"

"impetus"

"impetuses"

"impieties"

"impiety"

"imping"

"impinge"

"impinged"

"impingement"

"impingements"

"impinger"

"impingers"

"impinges"

"impinging"

"impings"

"impious"

"impiously"

"impiousness"

"impish"

"impishly"

"impishness"

"implacability"

"implacable"

"implacably"

"implacentalia"

"implant"

"implantation"

"implanted"

"implanter"

"implanting"

"implants"

"implausibility"

"implausible"

"implausibleness"

"implausibly"

"implement"

"implementable"

"implementation"

"implementations"

"implemented"

"implementing"

"implementor"

"implementors"

"implements"

"implicate"

"implicated"

"implicates"

"implicating"

"implication"

"implications"

"implicit"

"implicitly"

"implicitness"

"implied"

"impliedly"

"implies"

"implode"

"imploded"

"implodes"

"imploding"

"imploration"

"implorations"

"implore"

"implored"

"implorer"

"implorers"

"implores"

"imploring"

"imploringly"

"implosion"

"implosions"

"implosive"

"imply"

"implying"

"impolite"

"impolitely"

"impoliteness"

"impolitic"

"impolitical"

"impolitically"

"impoliticly"

"imponderability"

"imponderable"

"imponderableness"

"imponderables"

"imponderably"

"import"

"importable"

"importance"

"important"

"importantly"

"importation"

"importations"

"imported"

"importer"

"importers"

"importing"

"imports"

"importunate"

"importunately"

"importunateness"

"importune"

"importuned"

"importunes"

"importuning"

"importunities"

"importunity"

"impose"

"imposed"

"imposer"

"imposers"

"imposes"

"imposing"

"imposingly"

"imposition"

"impositions"

"impossibilities"

"impossibility"

"impossible"

"impossibleness"

"impossibly"

"impost"

"imposted"

"imposter"

"imposters"

"imposting"

"impostor"

"impostors"

"imposts"

"imposture"

"impostures"

"impotence"

"impotences"

"impotencies"

"impotency"

"impotent"

"impotently"

"impotents"

"impound"

"impoundable"

"impounded"

"impounding"

"impoundment"

"impoundments"

"impounds"

"impoverish"

"impoverished"

"impoverisher"

"impoverishes"

"impoverishing"

"impoverishment"

"impowers"

"impracticability"

"impracticable"

"impractical"

"impracticalities"

"impracticality"

"imprecate"

"imprecated"

"imprecates"

"imprecating"

"imprecation"

"imprecations"

"imprecator"

"imprecators"

"imprecise"

"imprecisely"

"impreciseness"

"imprecision"

"imprecisions"

"impregnability"

"impregnable"

"impregnably"

"impregnate"

"impregnated"

"impregnates"

"impregnating"

"impregnation"

"impregnations"

"impresario"

"impresarios"

"impress"

"impressed"

"impresser"

"impressers"

"impresses"

"impressibility"

"impressible"

"impressing"

"impression"

"impressionable"

"impressionably"

"impressionis"

"impressionism"

"impressionist"

"impressionistic"

"impressionists"

"impressions"

"impressive"

"impressively"

"impressiveness"

"impressment"

"impressments"

"imprest"

"imprests"

"imprimatur"

"imprimaturs"

"imprimis"

"imprint"

"imprinted"

"imprinter"

"imprinters"

"imprinting"

"imprints"

"imprison"

"imprisoned"

"imprisoning"

"imprisonment"

"imprisonments"

"imprisons"

"improbabilities"

"improbability"

"improbable"

"improbably"

"impromptu"

"improper"

"improperly"

"improperness"

"improprieties"

"impropriety"

"improvability"

"improvable"

"improve"

"improved"

"improvement"

"improvements"

"improver"

"improvers"

"improves"

"improvidence"

"improvident"

"improvidently"

"improving"

"improvisation"

"improvisational"

"improvisations"

"improvise"

"improvised"

"improviser"

"improvisers"

"improvises"

"improvising"

"improvisor"

"improvisors"

"imprudence"

"imprudent"

"imprudently"

"imps"

"impudence"

"impudent"

"impudently"

"impugn"

"impugnable"

"impugned"

"impugner"

"impugners"

"impugning"

"impugnment"

"impugns"

"impuissance"

"impulse"

"impulsed"

"impulses"

"impulsing"

"impulsion"

"impulsions"

"impulsive"

"impulsively"

"impulsiveness"

"impunities"

"impunity"

"impure"

"impurely"

"impureness"

"impurities"

"impurity"

"imputable"

"imputation"

"imputations"

"impute"

"imputed"

"imputer"

"imputers"

"imputes"

"imputing"

"imroving"

"in"

"in the area"

"inabilities"

"inability"

"inaccessibility"

"inaccessible"

"inaccuracies"

"inaccuracy"

"inaccurate"

"inaction"

"inactions"

"inactivate"

"inactivated"

"inactivates"

"inactivating"

"inactivation"

"inactivations"

"inactive"

"inactively"

"inactivities"

"inactivity"

"inadequacies"

"inadequacy"

"inadequate"

"inadequately"

"inadequateness"

"inadmissability"

"inadmissable"

"inadmissibility"

"inadmissible"

"inadmissibly"

"inadvertence"

"inadvertency"

"inadvertent"

"inadvertently"

"inadvisability"

"inadvisable"

"inadvisably"

"inalienability"

"inalienable"

"inalienably"

"inalterability"

"inalterable"

"inalterableness"

"inalterably"

"inamorata"

"inamoratas"

"inane"

"inanely"

"inaner"

"inaners"

"inanes"

"inanimate"

"inanimately"

"inanimateness"

"inanities"

"inanity"

"inapplicability"

"inapplicable"

"inapplicably"

"inapposite"

"inappositeness"

"inappreciable"

"inappreciably"

"inappreciative"

"inappreciatively"

"inapproachable"

"inappropriate"

"inappropriately"

"inappropriateness"

"inapt"

"inaptitude"

"inaptly"

"inaptness"

"inarguable"

"inarm"

"inarticulate"

"inarticulately"

"inarticulateness"

"inartistic"

"inartistically"

"inasmuch"

"inassimilable"

"inattention"

"inattentive"

"inattentively"

"inattentiveness"

"inaudibility"

"inaudible"

"inaudibly"

"inaugural"

"inaugurals"

"inaugurate"

"inaugurated"

"inaugurates"

"inaugurating"

"inauguration"

"inaugurations"

"inaugurator"

"inauspicious"

"inauspiciously"

"inauspiciousness"

"inboard"

"inboards"

"inborn"

"inbound"

"inbounds"

"inbreathe"

"inbreathing"

"inbred"

"inbreed"

"inbreeder"

"inbreeding"

"inbreeds"

"inbuilt"

"inc"

"inca"

"incaged"

"incages"

"incalculable"

"incalculableness"

"incalculably"

"incandescence"

"incandescent"

"incandescently"

"incantation"

"incantations"

"incapabilities"

"incapability"

"incapable"

"incapably"

"incapacious"

"incapacitant"

"incapacitate"

"incapacitated"

"incapacitates"

"incapacitating"

"incapacitation"

"incapacitator"

"incapacities"

"incapacity"

"incarcerate"

"incarcerated"

"incarcerates"

"incarcerating"

"incarceration"

"incarcerations"

"incarcerator"

"incarcerators"

"incarnadine"

"incarnadined"

"incarnadines"

"incarnadining"

"incarnate"

"incarnated"

"incarnates"

"incarnating"

"incarnation"

"incarnations"

"incas"

"incase"

"incased"

"incases"

"incautious"

"incautiously"

"incendiaries"

"incendiarism"

"incendiarist"

"incendiary"

"incense"

"incensed"

"incenses"

"incensing"

"incentive"

"incentives"

"incept"

"incepting"

"inception"

"inceptions"

"inceptive"

"inceptors"

"incepts"

"incertitude"

"incessant"

"incessantly"

"incest"

"incests"

"incestuous"

"incestuously"

"incestuousness"

"inch"

"inched"

"inches"

"inching"

"inchoate"

"inchoately"

"inchworm"

"inchworms"

"incidence"

"incident"

"incidental"

"incidentally"

"incidentals"

"incidentless"

"incidently"

"incidents"

"incinerate"

"incinerated"

"incinerates"

"incinerating"

"incineration"

"incinerations"

"incinerator"

"incinerators"

"incipience"

"incipiencies"

"incipiency"

"incipient"

"incise"

"incised"

"incises"

"incising"

"incision"

"incisions"

"incisive"

"incisively"

"incisiveness"

"incisor"

"incisors"

"incisory"

"incitant"

"incitants"

"incitation"

"incitations"

"incite"

"incited"

"incitement"

"incitements"

"inciter"

"inciters"

"incites"

"inciting"

"incitingly"

"incitive"

"incitory"

"incivil"

"incivilities"

"incivility"

"inclemency"

"inclement"

"inclinable"

"inclination"

"inclinations"

"incline"

"inclined"

"incliner"

"incliners"

"inclines"

"inclining"

"inclinometer"

"inclose"

"inclosed"

"incloser"

"inclosers"

"incloses"

"inclosing"

"inclosure"

"include"

"included"

"includes"

"including"

"inclusion"

"inclusions"

"inclusive"

"inclusively"

"inclusiveness"

"incog"

"incognita"

"incognito"

"incognitos"

"incognizant"

"incoherence"

"incoherences"

"incoherent"

"incoherently"

"incoincidence"

"incoincident"

"incombustible"

"income"

"incomes"

"incoming"

"incomings"

"incommensurable"

"incommensurate"

"incommensurately"

"incommode"

"incommoded"

"incommodes"

"incommoding"

"incommodious"

"incommunicable"

"incommunicably"

"incommunicado"

"incommunicative"

"incommutable"

"incommutably"

"incomparability"

"incomparable"

"incomparably"

"incompatibilities"

"incompatibility"

"incompatible"

"incompatibly"

"incompensation"

"incompetence"

"incompetencies"

"incompetency"

"incompetent"

"incompetently"

"incompetents"

"incomplete"

"incompletely"

"incompleteness"

"incompliance"

"incompliancies"

"incompliancy"

"incompliant"

"incomprehensible"

"incomprehensibleness"

"incomprehensiblies"

"incomprehensibly"

"incomprehension"

"incompressable"

"incompressibility"

"incompressible"

"incompressibly"

"incomputable"

"incomputably"

"inconcealable"

"inconceivabilities"

"inconceivability"

"inconceivable"

"inconceivably"

"inconclusive"

"inconclusively"

"inconclusiveness"

"incongruence"

"incongruent"

"incongruently"

"incongruities"

"incongruity"

"incongruous"

"incongruously"

"incongruousness"

"inconsequent"

"inconsequential"

"inconsequentially"

"inconsiderable"

"inconsiderate"

"inconsiderately"

"inconsiderateness"

"inconsistences"

"inconsistencies"

"inconsistency"

"inconsistent"

"inconsistently"

"inconsistentness"

"inconsolable"

"inconsolably"

"inconsonant"

"inconspicuous"

"inconspicuously"

"inconspicuousness"

"inconstancy"

"inconstant"

"inconstantly"

"inconsumable"

"inconsumably"

"incontestabilities"

"incontestability"

"incontestable"

"incontestably"

"incontinence"

"incontinencies"

"incontinency"

"incontinent"

"incontinently"

"incontrovertible"

"incontrovertibly"

"inconvenience"

"inconvenienced"

"inconveniences"

"inconveniencing"

"inconvenient"

"inconveniently"

"inconvertibilities"

"inconvertibility"

"incoordination"

"incorporate"

"incorporated"

"incorporatedness"

"incorporates"

"incorporating"

"incorporation"

"incorporations"

"incorporator"

"incorporators"

"incorporatorship"

"incorporeal"

"incorporeality"

"incorrect"

"incorrectly"

"incorrectness"

"incorrigibility"

"incorrigible"

"incorrigibleness"

"incorrigibly"

"incorrupt"

"incorrupted"

"incorruptibilities"

"incorruptibility"

"incorruptible"

"incorruptibly"

"incorruption"

"incorruptly"

"increasable"

"increase"

"increase IQ points"

"increased"

"increased hunger"

"increased thirst"

"increaser"

"increasers"

"increases"

"increasing"

"increasingly"

"incredibilities"

"incredibility"

"incredible"

"incredibleness"

"incredibly"

"incredulity"

"incredulous"

"incredulously"

"increment"

"incremental"

"incremented"

"incrementing"

"increments"

"incretory"

"incriminate"

"incriminated"

"incriminates"

"incriminating"

"incrimination"

"incriminator"

"incriminatory"

"incrust"

"incrustation"

"incrustations"

"incrusted"

"incrusting"

"incrusts"

"incubate"

"incubated"

"incubates"

"incubating"

"incubation"

"incubational"

"incubations"

"incubative"

"incubator"

"incubators"

"incubi"

"incubus"

"incubuses"

"inculcate"

"inculcated"

"inculcates"

"inculcating"

"inculcation"

"inculpability"

"inculpable"

"inculpate"

"inculpated"

"inculpates"

"inculpating"

"incumbencies"

"incumbency"

"incumbent"

"incumbently"

"incumbents"

"incumber"

"incumbered"

"incumbering"

"incumbers"

"incumbrance"

"incunabula"

"incunabulum"

"incur"

"incurability"

"incurable"

"incurably"

"incurious"

"incuriously"

"incurrable"

"incurred"

"incurring"

"incurs"

"incursion"

"incursions"

"incurve"

"incurving"

"incus"

"indebted"

"indebtedness"

"indecencies"

"indecency"

"indecent"

"indecenter"

"indecently"

"indeciduous"

"indecipherable"

"indecision"

"indecisive"

"indecisively"

"indecisiveness"

"indecorous"

"indecorously"

"indecorousness"

"indeed"

"indefatigability"

"indefatigable"

"indefatigably"

"indefeasible"

"indefeasibly"

"indefensibility"

"indefensible"

"indefensibly"

"indefinable"

"indefinably"

"indefinite"

"indefinitely"

"indefiniteness"

"indelible"

"indelibly"

"indelicacy"

"indelicate"

"indelicately"

"indemnification"

"indemnifications"

"indemnificator"

"indemnificatory"

"indemnified"

"indemnifier"

"indemnifies"

"indemnify"

"indemnifying"

"indemnitee"

"indemnities"

"indemnitor"

"indemnity"

"indemnization"

"indemonstrable"

"indent"

"indentation"

"indentations"

"indented"

"indenter"

"indenters"

"indenting"

"indention"

"indentions"

"indentor"

"indentors"

"indents"

"indenture"

"indentured"

"indentures"

"indenturing"

"independence"

"independent"

"independently"

"independents"

"indescribabilities"

"indescribability"

"indescribable"

"indescribably"

"indestructibility"

"indestructible"

"indestructibleness"

"indestructibly"

"indeterminable"

"indeterminacy"

"indeterminate"

"indeterminately"

"indeterminateness"

"indetermination"

"index"

"indexable"

"indexation"

"indexed"

"indexer"

"indexers"

"indexes"

"indexing"

"india"

"indian"

"Indian medicine"

"indiana"

"indianan"

"indianans"

"indianapolis"

"indianian"

"indianians"

"indians"

"indicants"

"indicate"

"indicated"

"indicates"

"indicating"

"indication"

"indications"

"indicative"

"indicatively"

"indicatives"

"indicator"

"indicators"

"indices"

"indicia"

"indicium"

"indict"

"indictable"

"indictably"

"indicted"

"indictee"

"indictees"

"indicter"

"indicters"

"indicting"

"indictment"

"indictments"

"indictor"

"indictors"

"indicts"

"indies"

"indifference"

"indifferent"

"indifferently"

"indigence"

"indigene"

"indigenes"

"indigenous"

"indigens"

"indigent"

"indigently"

"indigents"

"indigestibility"

"indigestibilty"

"indigestible"

"indigestion"

"indigestive"

"indign"

"indignant"

"indignantly"

"indignation"

"indignities"

"indignity"

"indigo"

"indigoes"

"indigos"

"indirect"

"indirection"

"indirections"

"indirectly"

"indirectness"

"indiscernible"

"indiscoverable"

"indiscreet"

"indiscreetly"

"indiscreetness"

"indiscrete"

"indiscretion"

"indiscretions"

"indiscriminantly"

"indiscriminate"

"indiscriminately"

"indiscriminateness"

"indiscriminating"

"indiscrimination"

"indispensabilities"

"indispensability"

"indispensable"

"indispensableness"

"indispensably"

"indispensible"

"indisposed"

"indisposition"

"indispositions"

"indisputable"

"indisputableness"

"indisputably"

"indissolubility"

"indissoluble"

"indissolubly"

"indistinct"

"indistinctly"

"indistinctness"

"indistinguishable"

"indite"

"indited"

"inditer"

"inditers"

"indites"

"inditing"

"indium"

"indiums"

"individual"

"individualism"

"individualist"

"individualistic"

"individualists"

"individualities"

"individuality"

"individualization"

"individualize"

"individualized"

"individualizes"

"individualizing"

"individually"

"individuals"

"individuate"

"individuated"

"individuates"

"individuating"

"individuation"

"indivisibility"

"indivisible"

"indivisibly"

"indochina"

"indochinese"

"indoctrinate"

"indoctrinated"

"indoctrinates"

"indoctrinating"

"indoctrination"

"indoctrinations"

"indol"

"indolence"

"indolent"

"indolently"

"indomitable"

"indomitably"

"indonesia"

"indonesian"

"indonesians"

"indoor"

"indoors"

"indorse"

"indorsed"

"indorsee"

"indorsees"

"indorsement"

"indorser"

"indorses"

"indorsing"

"indorsor"

"indorsors"

"indow"

"indowed"

"indows"

"indraft"

"indrawn"

"indubitable"

"indubitably"

"induce"

"induced"

"inducement"

"inducements"

"inducer"

"inducers"

"induces"

"inducible"

"inducing"

"induct"

"inductance"

"inductances"

"inducted"

"inductee"

"inductees"

"inducting"

"induction"

"inductions"

"inductive"

"inductively"

"inductiveness"

"inductor"

"inductors"

"inducts"

"indue"

"indued"

"indues"

"indulge"

"indulged"

"indulgence"

"indulgences"

"indulgent"

"indulgently"

"indulger"

"indulgers"

"indulges"

"indulging"

"indurate"

"indurated"

"indurates"

"indurating"

"induration"

"indurations"

"indurative"

"industrial"

"industrialism"

"industrialist"

"industrialists"

"industrialization"

"industrialize"

"industrialized"

"industrializes"

"industrializing"

"industrially"

"industrials"

"industries"

"industrious"

"industriously"

"industriousness"

"industry"

"indwell"

"indwelling"

"indwells"

"indwelt"

"inearthed"

"inebriant"

"inebriate"

"inebriated"

"inebriates"

"inebriating"

"inebriation"

"inebriety"

"inebrious"

"inedible"

"inedited"

"ineducability"

"ineducable"

"ineffable"

"ineffably"

"ineffaceable"

"ineffective"

"ineffectively"

"ineffectiveness"

"ineffectual"

"ineffectually"

"ineffectualness"

"inefficacious"

"inefficaciously"

"inefficacy"

"inefficiencies"

"inefficiency"

"inefficient"

"inefficiently"

"inelastic"

"inelasticity"

"inelegance"

"inelegant"

"inelegantly"

"ineligibility"

"ineligible"

"ineligibles"

"ineligibly"

"ineloquent"

"ineloquently"

"ineluctable"

"ineluctably"

"inept"

"ineptitude"

"ineptly"

"ineptness"

"inequable"

"inequalities"

"inequality"

"inequitable"

"inequitableness"

"inequitably"

"inequities"

"inequity"

"ineradicable"

"inerrant"

"inert"

"inertia"

"inertial"

"inertias"

"inertly"

"inertness"

"inerts"

"inescapable"

"inescapably"

"inessential"

"inestimable"

"inestimably"

"inevitabilities"

"inevitability"

"inevitable"

"inevitableness"

"inevitably"

"inexact"

"inexactitude"

"inexactly"

"inexactness"

"inexcusability"

"inexcusable"

"inexcusableness"

"inexcusably"

"inexecutable"

"inexecution"

"inexhaustible"

"inexhaustibly"

"inexorable"

"inexorably"

"inexpedient"

"inexpensive"

"inexpensively"

"inexpensiveness"

"inexperience"

"inexperienced"

"inexpert"

"inexpertly"

"inexpiable"

"inexplicable"

"inexplicably"

"inexpressibilities"

"inexpressibility"

"inexpressible"

"inexpressibly"

"inexpressive"

"inexpressiveness"

"inextinguishable"

"inextinguishables"

"inextinguishably"

"inextricability"

"inextricable"

"inextricably"

"infallibility"

"infallible"

"infallibleness"

"infallibly"

"infamies"

"infamous"

"infamously"

"infamy"

"infancies"

"infancy"

"infant"

"infanticidal"

"infanticide"

"infanticides"

"infantile"

"infantilism"

"infantility"

"infantries"

"infantry"

"infantryman"

"infantrymen"

"infants"

"infarct"

"infarcted"

"infarction"

"infarctions"

"infarcts"

"infatuate"

"infatuated"

"infatuates"

"infatuating"

"infatuation"

"infatuations"

"infeasible"

"infect"

"infected"

"infecter"

"infecters"

"infecting"

"infection"

"infections"

"infectious"

"infectiously"

"infectiousness"

"infective"

"infector"

"infectors"

"infects"

"infecund"

"infelicitous"

"infelicity"

"infeoffed"

"infer"

"inferable"

"inference"

"inferences"

"inferential"

"inferentially"

"inferior"

"inferiorities"

"inferiority"

"inferiors"

"infernal"

"infernally"

"inferno"

"infernos"

"inferred"

"inferrer"

"inferrers"

"inferrible"

"inferring"

"infers"

"infertile"

"infertilely"

"infertility"

"infest"

"infestation"

"infestations"

"infested"

"infester"

"infesters"

"infesting"

"infests"

"infidel"

"infidelities"

"infidelity"

"infidels"

"infield"

"infielder"

"infielders"

"infields"

"infighter"

"infighters"

"infighting"

"infiltrate"

"infiltrated"

"infiltrates"

"infiltrating"

"infiltration"

"infiltrations"

"infiltrator"

"infiltrators"

"infinite"

"infinitely"

"infiniteness"

"infinites"

"infinitesimal"

"infinitesimally"

"infinitesimals"

"infinities"

"infinitive"

"infinitives"

"infinitude"

"infinitum"

"infinity"

"infirm"

"infirmable"

"infirmaries"

"infirmary"

"infirmed"

"infirming"

"infirmities"

"infirmity"

"infirmly"

"infirmness"

"infirms"

"infix"

"infixed"

"infixes"

"inflame"

"inflamed"

"inflamer"

"inflamers"

"inflames"

"inflaming"

"inflammabilities"

"inflammability"

"inflammable"

"inflammation"

"inflammations"

"inflammative"

"inflammatorily"

"inflammatory"

"inflatable"

"inflate"

"inflated"

"inflater"

"inflaters"

"inflates"

"inflating"

"inflation"

"inflationary"

"inflationism"

"inflationist"

"inflationists"

"inflations"

"inflator"

"inflators"

"inflect"

"inflected"

"inflecting"

"inflection"

"inflectional"

"inflections"

"inflects"

"inflexed"

"inflexibility"

"inflexible"

"inflexibleness"

"inflexibly"

"inflict"

"inflictable"

"inflicted"

"inflicter"

"inflicting"

"infliction"

"inflictions"

"inflictive"

"inflictor"

"inflicts"

"inflight"

"inflorescence"

"inflow"

"inflows"

"influence"

"influenceabilities"

"influenceability"

"influenceable"

"influenced"

"influencer"

"influences"

"influencing"

"influent"

"influential"

"influents"

"influenza"

"influenzas"

"influx"

"influxes"

"info"

"infold"

"infolded"

"infolder"

"infolders"

"infolding"

"infolds"

"infomania"

"inform"

"informal"

"informalities"

"informality"

"informally"

"informant"

"informants"

"information"

"informational"

"informational purposes only"

"informative"

"informatively"

"informativeness"

"informed"

"informer"

"informers"

"informing"

"informs"

"infos"

"infra"

"infract"

"infracted"

"infraction"

"infractions"

"infractor"

"infrangible"

"infrared"

"infrareds"

"infrasonic"

"infrastructure"

"infrastructures"

"infrequence"

"infrequency"

"infrequent"

"infrequently"

"infringe"

"infringed"

"infringement"

"infringements"

"infringer"

"infringers"

"infringes"

"infringing"

"infundibula"

"infundibular"

"infundibuliform"

"infundibulum"

"infuriate"

"infuriated"

"infuriates"

"infuriating"

"infuriatingly"

"infuriation"

"infuse"

"infused"

"infuser"

"infusers"

"infuses"

"infusibility"

"infusible"

"infusing"

"infusion"

"infusions"

"infusive"

"infusoria"

"ingate"

"ingather"

"ingathered"

"ingathers"

"ingenious"

"ingeniously"

"ingeniousness"

"ingenue"

"ingenues"

"ingenuity"

"ingenuous"

"ingenuously"

"ingenuousness"

"ingest"

"ingestant"

"ingested"

"ingestible"

"ingesting"

"ingestion"

"ingestive"

"ingests"

"ingle"

"ingles"

"inglorious"

"ingloriously"

"ingloriousness"

"ingoing"

"ingot"

"ingots"

"ingraft"

"ingrafted"

"ingrafting"

"ingrain"

"ingrained"

"ingraining"

"ingrains"

"ingrate"

"ingrates"

"ingratiate"

"ingratiated"

"ingratiates"

"ingratiating"

"ingratiation"

"ingratitude"

"ingredient"

"ingredients"

"ingress"

"ingresses"

"ingression"

"ingressive"

"ingroup"

"ingroups"

"ingrowing"

"ingrown"

"ingrowths"

"inguinal"

"ingulf"

"ingulfing"

"ingulfs"

"inhabit"

"inhabitability"

"inhabitable"

"inhabitance"

"inhabitancies"

"inhabitancy"

"inhabitant"

"inhabitants"

"inhabitation"

"inhabited"

"inhabiter"

"inhabiting"

"inhabitress"

"inhabits"

"inhalant"

"inhalants"

"inhalation"

"inhalations"

"inhalator"

"inhalators"

"inhale"

"inhaled"

"inhaler"

"inhalers"

"inhales"

"inhaling"

"inharmonic"

"inharmonious"

"inhaul"

"inhaulers"

"inhere"

"inhered"

"inherence"

"inherent"

"inherently"

"inherently inaccurate"

"inheres"

"inhering"

"inherit"

"inheritabilities"

"inheritability"

"inheritable"

"inheritably"

"inheritance"

"inheritances"

"inherited"

"inheriting"

"inheritor"

"inheritors"

"inheritress"

"inherits"

"inhesions"

"inhibit"

"inhibited"

"inhibiter"

"inhibiting"

"inhibition"

"inhibitions"

"inhibitive"

"inhibitor"

"inhibitors"

"inhibitory"

"inhibits"

"inholding"

"inhomogeneities"

"inhospitable"

"inhospitably"

"inhospitality"

"inhuman"

"inhumane"

"inhumanely"

"inhumanities"

"inhumanity"

"inhumanly"

"inhume"

"inhumed"

"inhumer"

"inhumes"

"inimicability"

"inimical"

"inimically"

"inimitable"

"inimitably"

"iniquities"

"iniquitous"

"iniquitously"

"iniquity"

"initial"

"initial load"

"initialed"

"initialing"

"initialization"

"initialize"

"initialized"

"initializing"

"initialled"

"initialling"

"initially"

"initials"

"initiate"

"initiated"

"initiates"

"initiating"

"initiation"

"initiations"

"initiative"

"initiatives"

"initiator"

"initiators"

"initiatory"

"inject"

"injectant"

"injected"

"injecting"

"injection"

"injections"

"injector"

"injectors"

"injects"

"injudicious"

"injudiciously"

"injudiciousness"

"injunction"

"injunctions"

"injure"

"injured"

"injurer"

"injurers"

"injures"

"injuries"

"injuring"

"injurious"

"injuriously"

"injuriousness"

"injury"

"injustice"

"injustices"

"ink"

"inkblot"

"inkblots"

"inked"

"inker"

"inkers"

"inkhorn"

"inkhorns"

"inkier"

"inkiest"

"inkiness"

"inking"

"inkle"

"inkles"

"inkless"

"inkling"

"inklings"

"inkpot"

"inkpots"

"inks"

"inkstand"

"inkstands"

"inkwell"

"inkwells"

"inky"

"inlaid"

"inland"

"inlander"

"inlanders"

"inlands"

"inlay"

"inlayer"

"inlayers"

"inlaying"

"inlays"

"inlet"

"inlets"

"inletting"

"inlier"

"inliers"

"inly"

"inmate"

"inmates"

"inmesh"

"inmeshing"

"inmost"

"inn"

"innards"

"innate"

"innately"

"innateness"

"inned"

"inner"

"inner calm"

"innerly"

"innermost"

"inners"

"innersole"

"innerspring"

"innervate"

"innervated"

"innervates"

"innervating"

"innervation"

"innervational"

"innervations"

"innerving"

"innholder"

"inning"

"innings"

"innkeeper"

"innkeepers"

"innocence"

"innocency"

"innocent"

"innocenter"

"innocently"

"innocents"

"innocuous"

"innocuously"

"innocuousness"

"innominate"

"innovate"

"innovated"

"innovates"

"innovating"

"innovation"

"innovations"

"innovative"

"innovator"

"innovators"

"innoxious"

"inns"

"innuendo"

"innuendoes"

"innuendos"

"innumerable"

"inoculant"

"inoculate"

"inoculated"

"inoculates"

"inoculating"

"inoculation"

"inoculations"

"inoculative"

"inoculums"

"inoffensive"

"inoffensively"

"inoffensiveness"

"inofficial"

"inofficious"

"inoperable"

"inoperative"

"inopportune"

"inopportunely"

"inordinate"

"inordinately"

"inorganic"

"inorganically"

"inositols"

"inpatient"

"inpatients"

"inphase"

"inpouring"

"inpours"

"input"

"inputs"

"inputted"

"inputting"

"inquest"

"inquests"

"inquieting"

"inquietude"

"inquire"

"inquired"

"inquirer"

"inquirers"

"inquires"

"inquiries"

"inquiring"

"inquiringly"

"inquiry"

"inquisition"

"inquisitional"

"inquisitions"

"inquisitive"

"inquisitively"

"inquisitiveness"

"inquisitor"

"inquisitorial"

"inquisitorially"

"inquisitors"

"inquisitory"

"inroad"

"inroads"

"inrush"

"inrushes"

"inrushing"

"ins"

"insalivating"

"insalivation"

"insalubrious"

"insalubrities"

"insalubrity"

"insane"

"insanely"

"insaner"

"insanest"

"insanitary"

"insanitation"

"insanities"

"insanity"

"insatiability"

"insatiable"

"insatiably"

"insatiate"

"inscribe"

"inscribed"

"inscriber"

"inscribers"

"inscribes"

"inscribing"

"inscription"

"inscriptions"

"inscrolls"

"inscrutability"

"inscrutable"

"inscrutableness"

"inscrutably"

"inseam"

"inseams"

"insect"

"insecticidal"

"insecticide"

"insecticides"

"insectifuge"

"insectivorous"

"insectology"

"insects"

"insecure"

"insecurely"

"insecureness"

"insecurities"

"insecurity"

"inseminate"

"inseminated"

"inseminates"

"inseminating"

"insemination"

"inseminations"

"inseminator"

"inseminators"

"insensate"

"insensately"

"insensateness"

"insensibility"

"insensible"

"insensibly"

"insensitive"

"insensitively"

"insensitivities"

"insensitivity"

"insentience"

"insentient"

"inseparability"

"inseparable"

"inseparableness"

"inseparably"

"insert"

"inserted"

"inserter"

"inserters"

"inserting"

"insertion"

"insertions"

"inserts"

"inset"

"insets"

"insetters"

"insetting"

"insheathe"

"insheathed"

"insheathing"

"insheaths"

"inshore"

"inshrined"

"inshrines"

"inshrining"

"inside"

"insider"

"insiders"

"insides"

"insidious"

"insidiously"

"insidiousness"

"insight"

"insightful"

"insights"

"insigne"

"insignia"

"insignias"

"insignificance"

"insignificant"

"insincere"

"insincerely"

"insincerities"

"insincerity"

"insinuate"

"insinuated"

"insinuates"

"insinuating"

"insinuation"

"insinuations"

"insinuator"

"insinuators"

"insipid"

"insipidity"

"insipidly"

"insist"

"insisted"

"insistence"

"insistency"

"insistent"

"insistently"

"insister"

"insisters"

"insisting"

"insistingly"

"insists"

"insobriety"

"insofar"

"insolation"

"insole"

"insolence"

"insolent"

"insolently"

"insolents"

"insoles"

"insolubilities"

"insolubility"

"insoluble"

"insolubly"

"insolvable"

"insolvencies"

"insolvency"

"insolvent"

"insomnia"

"insomniac"

"insomniacs"

"insomnias"

"insomuch"

"insouciance"

"insouciant"

"insoul"

"inspect"

"inspected"

"inspecting"

"inspection"

"inspections"

"inspector"

"inspectorate"

"inspectorial"

"inspectors"

"inspects"

"insphering"

"inspiration"

"inspirational"

"inspirationally"

"inspirations"

"inspiratory"

"inspire"

"inspired"

"inspirer"

"inspirers"

"inspires"

"inspiring"

"inspirit"

"inspirited"

"inspiriting"

"inspirits"

"inst"

"instabilities"

"instability"

"instal"

"install"

"installant"

"installation"

"installations"

"installed"

"installer"

"installers"

"installing"

"installment"

"installments"

"installs"

"instalment"

"instals"

"instance"

"instanced"

"instances"

"instancing"

"instant"

"instantaneous"

"instantaneously"

"instanter"

"instantly"

"instants"

"instarred"

"instate"

"instated"

"instatement"

"instates"

"instating"

"instead"

"instep"

"insteps"

"instigate"

"instigated"

"instigates"

"instigating"

"instigatingly"

"instigation"

"instigative"

"instigator"

"instigators"

"instil"

"instill"

"instillation"

"instilled"

"instiller"

"instillers"

"instilling"

"instillment"

"instills"

"instils"

"instinct"

"instinctive"

"instinctively"

"instincts"

"instinctual"

"institute"

"instituted"

"instituter"

"instituters"

"institutes"

"instituting"

"institution"

"institutional"

"institutionalism"

"institutionalist"

"institutionalists"

"institutionalization"

"institutionalize"

"institutionalized"

"institutionalizes"

"institutionalizing"

"institutionally"

"institutions"

"institutor"

"institutors"

"instr"

"instrokes"

"instruct"

"instructed"

"instructing"

"instruction"

"instructional"

"instructions"

"instructive"

"instructor"

"instructors"

"instructorship"

"instructorships"

"instructs"

"instrument"

"instrumental"

"instrumentalist"

"instrumentalists"

"instrumentalities"

"instrumentality"

"instrumentally"

"instrumentary"

"instrumentation"

"instrumentations"

"instrumented"

"instrumenting"

"instruments"

"insubmissive"

"insubordinate"

"insubordinately"

"insubordination"

"insubstantial"

"insufferable"

"insufferably"

"insufficiencies"

"insufficiency"

"insufficient"

"insufficiently"

"insulants"

"insular"

"insularity"

"insulars"

"insulate"

"insulated"

"insulates"

"insulating"

"insulation"

"insulations"

"insulator"

"insulators"

"insulin"

"Insulin calculator"

"insulin-dependent diabetes mellitus"

"insulins"

"insult"

"insulted"

"insulter"

"insulters"

"insulting"

"insultingly"

"insults"

"insuperable"

"insuperably"

"insupportable"

"insupportably"

"insuppressible"

"insurability"

"insurable"

"insurance"

"Insurance group"

"insurance information"

"Insurance tracker"

"insurant"

"insurants"

"insure"

"insured"

"insureds"

"insurer"

"insurers"

"insures"

"insurgence"

"insurgences"

"insurgencies"

"insurgency"

"insurgent"

"insurgents"

"insurgescence"

"insuring"

"insurmountable"

"insurmountably"

"insurrect"

"insurrection"

"insurrectional"

"insurrectionally"

"insurrectionaries"

"insurrectionary"

"insurrectionist"

"insurrectionists"

"insurrections"

"insusceptibilities"

"insusceptibility"

"insusceptible"

"int"

"intact"

"intactness"

"intagli"

"intaglio"

"intaglios"

"intake"

"intakes"

"intangibilities"

"intangibility"

"intangible"

"intangibles"

"intangibly"

"intarsias"

"integer"

"integers"

"integral"

"integrally"

"integrals"

"integrate"

"integrated"

"integrates"

"integrating"

"integration"

"integrationist"

"integrations"

"integrative"

"integrator"

"integrities"

"integrity"

"integument"

"integumental"

"integumentary"

"Integumentary System"

"integuments"

"intel"

"intellect"

"intellects"

"intellectual"

"intellectualism"

"intellectualist"

"intellectualization"

"intellectualizations"

"intellectualize"

"intellectualized"

"intellectualizes"

"intellectualizing"

"intellectually"

"intellectuals"

"intelligence"

"intelligences"

"intelligent"

"Intelligent Increase"

"intelligently"

"intelligentsia"

"intelligibility"

"intelligible"

"intelligibly"

"intemperance"

"intemperances"

"intemperate"

"intemperately"

"intemperateness"

"intend"

"intended"

"intendeds"

"intender"

"intenders"

"intending"

"intendment"

"intends"

"intense"

"intensely"

"intenseness"

"intenser"

"intensest"

"intensification"

"intensifications"

"intensified"

"intensifier"

"intensifiers"

"intensifies"

"intensify"

"intensifying"

"intensities"

"intensity"

"intensive"

"intensive care"

"intensively"

"intensiveness"

"intensives"

"intent"

"intention"

"intentional"

"intentionally"

"intentioned"

"intentions"

"intently"

"intentness"

"intents"

"inter"

"interacademic"

"interacinous"

"interact"

"interacted"

"interacting"

"interaction"

"interactions"

"interactive"

"interactive reference"

"interactively"

"interacts"

"interagency"

"interagent"

"interatomic"

"interbank"

"interbanking"

"interborough"

"interbranch"

"interbred"

"interbreed"

"interbreeding"

"interbreeds"

"intercalary"

"intercalate"

"intercalated"

"intercalates"

"intercalating"

"intercalation"

"intercalations"

"intercapillary"

"intercede"

"interceded"

"interceder"

"intercedes"

"interceding"

"intercellular"

"intercept"

"intercepted"

"intercepting"

"interception"

"interceptions"

"interceptive"

"interceptor"

"interceptors"

"intercepts"

"intercession"

"intercessional"

"intercessions"

"intercessor"

"intercessors"

"intercessory"

"interchange"

"interchangeable"

"interchangeably"

"interchanged"

"interchanges"

"interchanging"

"intercity"

"interclass"

"intercollegiate"

"intercom"

"intercommunicate"

"intercommunicated"

"intercommunicates"

"intercommunicating"

"intercommunication"

"intercommunications"

"intercompany"

"intercoms"

"interconnect"

"interconnected"

"interconnecting"

"interconnection"

"interconnections"

"interconnects"

"intercontinental"

"intercostal"

"intercounty"

"intercourse"

"intercultural"

"intercuts"

"interdenominational"

"interdepartmental"

"interdependence"

"interdependency"

"interdependent"

"interdict"

"interdicted"

"interdicting"

"interdiction"

"interdictions"

"interdictive"

"interdictor"

"interdictory"

"interdicts"

"interdictum"

"interdisciplinary"

"interdistrict"

"interest"

"interested"

"interesting"

"interestingly"

"interests"

"interface"

"interfaced"

"interfaces"

"interfacial"

"interfacing"

"interfactional"

"interfaith"

"interfere"

"interfered"

"interference"

"interferences"

"interferer"

"interferers"

"interferes"

"interfering"

"interferometer"

"interferometers"

"interferometries"

"interferometry"

"interferon"

"interfertile"

"interfile"

"interfiled"

"interfiles"

"interfiling"

"interfirm"

"intergalactic"

"intergovernmental"

"intergroup"

"interhemispheric"

"interim"

"interims"

"interior"

"interiorly"

"interiors"

"interject"

"interjected"

"interjecting"

"interjection"

"interjectional"

"interjectionally"

"interjections"

"interjector"

"interjectors"

"interjectory"

"interjects"

"interlace"

"interlaced"

"interlaces"

"interlacing"

"interlaid"

"interlard"

"interlarded"

"interlarding"

"interlards"

"interlays"

"interleaf"

"interleave"

"interleaved"

"interleaves"

"interleaving"

"interlibrary"

"interline"

"interlinear"

"interlined"

"interlines"

"interlining"

"interlock"

"interlocked"

"interlocking"

"interlocks"

"interlocution"

"interlocutor"

"interlocutors"

"interlocutory"

"interlocutress"

"interlocutresses"

"interlocutrice"

"interlocutrices"

"interlope"

"interloped"

"interloper"

"interlopers"

"interlopes"

"interloping"

"interlude"

"interludes"

"interlunar"

"intermarriage"

"intermarriages"

"intermarried"

"intermarries"

"intermarry"

"intermarrying"

"intermediacy"

"intermediaries"

"intermediary"

"intermediate"

"intermediated"

"intermediately"

"intermediateness"

"intermediates"

"intermediating"

"intermediation"

"intermediator"

"intermediatory"

"intermenstrual"

"interment"

"interments"

"intermesh"

"intermeshed"

"intermeshes"

"intermeshing"

"intermezzi"

"intermezzo"

"intermezzos"

"interminable"

"interminableness"

"interminably"

"intermingle"

"intermingled"

"intermingles"

"intermingling"

"intermission"

"intermissions"

"intermit"

"intermits"

"intermitted"

"intermittence"

"intermittencies"

"intermittency"

"intermittent"

"intermittently"

"intermitting"

"intermix"

"intermixed"

"intermixes"

"intermixing"

"intermixture"

"intermixtures"

"intermolecular"

"intermuscular"

"intern"

"internal"

"internal medicine"

"internality"

"internalization"

"internalize"

"internalized"

"internalizing"

"internally"

"internals"

"international"

"internationalism"

"internationalist"

"internationalists"

"internationalization"

"internationalizations"

"internationalize"

"internationalized"

"internationalizes"

"internationalizing"

"internationally"

"internationals"

"internecine"

"interned"

"internee"

"internees"

"internes"

"Internet"

"Internet Access"

"interning"

"internist"

"internists"

"internment"

"internments"

"internodal"

"internode"

"internodes"

"interns"

"internship"

"internships"

"internuclear"

"internuncio"

"internuncios"

"interoceanic"

"interoffice"

"interorbital"

"interorbitally"

"interpersonal"

"interpersonally"

"interphone"

"interphones"

"interplanetary"

"interplant"

"interplay"

"interplays"

"interplead"

"interpol"

"interpolar"

"interpolate"

"interpolated"

"interpolates"

"interpolating"

"interpolation"

"interpolations"

"interpolator"

"interpolators"

"interpose"

"interposed"

"interposer"

"interposers"

"interposes"

"interposing"

"interposition"

"interpositions"

"interpret"

"interpretable"

"interpretation"

"interpretational"

"interpretations"

"interpretative"

"interpretatively"

"interpreted"

"interpreter"

"interpreters"

"interpreting"

"interpretive"

"interprets"

"interprofessional"

"interrace"

"interracial"

"interred"

"interregional"

"interregna"

"interregnal"

"interregnum"

"interregnums"

"interrelate"

"interrelated"

"interrelatedness"

"interrelates"

"interrelating"

"interrelation"

"interrelations"

"interrelationship"

"interrelationships"

"interreligious"

"interring"

"interrogable"

"interrogant"

"interrogate"

"interrogated"

"interrogates"

"interrogating"

"interrogation"

"interrogational"

"interrogations"

"interrogative"

"interrogatively"

"interrogator"

"interrogatories"

"interrogatorily"

"interrogators"

"interrogatory"

"interrogee"

"interrupt"

"interrupted"

"interrupter"

"interrupters"

"interrupting"

"interruption"

"interruptions"

"interruptive"

"interrupts"

"inters"

"interscholastic"

"interschool"

"intersect"

"intersected"

"intersecting"

"intersection"

"intersectional"

"intersections"

"intersects"

"intersession"

"intersessions"

"intersex"

"intersexual"

"intersexualism"

"intersexualities"

"intersexuality"

"intersexually"

"intersocietal"

"intersperse"

"interspersed"

"intersperses"

"interspersing"

"interspersion"

"interspersions"

"interstate"

"interstates"

"interstellar"

"interstice"

"interstices"

"intersticial"

"interstitial"

"interstitially"

"intertangle"

"intertangled"

"intertangles"

"intertangling"

"interterritorial"

"intertidal"

"interties"

"intertribal"

"intertropical"

"intertwine"

"intertwined"

"intertwinement"

"intertwinements"

"intertwines"

"intertwining"

"interuniversity"

"interurban"

"interval"

"intervals"

"intervarsity"

"intervene"

"intervened"

"intervener"

"interveners"

"intervenes"

"intervening"

"intervention"

"interventionism"

"interventionist"

"interventionists"

"interventions"

"intervertebral"

"interview"

"interviewed"

"interviewee"

"interviewees"

"interviewer"

"interviewers"

"interviewing"

"interviews"

"intervocalic"

"interweave"

"interweaved"

"interweaves"

"interweaving"

"interwove"

"interwoven"

"interwrought"

"intestacy"

"intestate"

"intestinal"

"intestinally"

"intestine"

"intestines"

"intimacies"

"intimacy"

"intimate"

"intimated"

"intimately"

"intimateness"

"intimater"

"intimaters"

"intimates"

"intimating"

"intimation"

"intimations"

"intimidate"

"intimidated"

"intimidates"

"intimidating"

"intimidation"

"intimidations"

"intimidator"

"intimidatory"

"intitling"

"intl"

"into"

"intolerable"

"intolerably"

"intolerance"

"intolerant"

"intomb"

"intombing"

"intombs"

"intonating"

"intonation"

"intonations"

"intone"

"intoned"

"intoner"

"intoners"

"intones"

"intoning"

"intoxicant"

"intoxicants"

"intoxicate"

"intoxicated"

"intoxicatedly"

"intoxicates"

"intoxicating"

"intoxication"

"intoxications"

"intoxicative"

"intr"

"intra"

"intracity"

"intractable"

"intradermal"

"intramolecular"

"intramural"

"intramurally"

"intrans"

"intransigence"

"intransigent"

"intransigently"

"intransigents"

"intransitive"

"intransitively"

"intransitiveness"

"intransitives"

"intrastate"

"intrauterine"

"intravaginal"

"intravenous"

"intravenously"

"intreating"

"intrench"

"intrenched"

"intrenches"

"intrepid"

"intrepidity"

"intrepidly"

"intricacies"

"intricacy"

"intricate"

"intricately"

"intricateness"

"intrigue"

"intrigued"

"intriguer"

"intriguers"

"intrigues"

"intriguing"

"intriguingly"

"intrinsic"

"intrinsically"

"intro"

"introduce"

"introduced"

"introducer"

"introducers"

"introduces"

"introducible"

"introducing"

"introduction"

"introductions"

"introductory"

"introit"

"introits"

"introject"

"introjection"

"intromission"

"intromit"

"intromits"

"intromitted"

"intromittent"

"intromitter"

"intromitting"

"intros"

"introspection"

"introspections"

"introspective"

"introspectively"

"introspectiveness"

"introversion"

"introversions"

"introversive"

"introvert"

"introverted"

"introverts"

"intrude"

"intruded"

"intruder"

"intruders"

"intrudes"

"intruding"

"intrudingly"

"intrusion"

"intrusions"

"intrusive"

"intrusively"

"intrusiveness"

"intrust"

"intrusted"

"intrusting"

"intrusts"

"intuit"

"intuited"

"intuiting"

"intuition"

"intuitions"

"intuitive"

"intuitively"

"intuitiveness"

"intuito"

"intuits"

"intumesce"

"inturn"

"inturned"

"intwined"

"intwines"

"intwining"

"intwisted"

"intwists"

"inundant"

"inundate"

"inundated"

"inundates"

"inundating"

"inundation"

"inundations"

"inure"

"inured"

"inurement"

"inurements"

"inures"

"inuring"

"inurn"

"inurns"

"inutile"

"invadable"

"invade"

"invaded"

"invader"

"invaders"

"invades"

"invading"

"invagination"

"invalid"

"invalidate"

"invalidated"

"invalidates"

"invalidating"

"invalidation"

"invalidations"

"invalidator"

"invalided"

"invaliding"

"invalidism"

"invalidity"

"invalidly"

"invalidness"

"invalids"

"invaluable"

"invaluably"

"invariability"

"invariable"

"invariably"

"invariant"

"invasion"

"invasions"

"invasive"

"invasiveness"

"invected"

"invective"

"invectives"

"inveigh"

"inveighed"

"inveighing"

"inveighs"

"inveigle"

"inveigled"

"inveiglement"

"inveigler"

"inveiglers"

"inveigles"

"inveigling"

"invent"

"invented"

"inventer"

"inventers"

"inventing"

"invention"

"inventions"

"inventive"

"inventively"

"inventiveness"

"inventor"

"inventoried"

"inventories"

"inventors"

"inventory"

"inventorying"

"invents"

"inverness"

"invernesses"

"inverse"

"inversely"

"inverses"

"inversion"

"inversions"

"inversive"

"invert"

"invertase"

"invertebrate"

"invertebrates"

"inverted"

"inverter"

"inverters"

"invertible"

"inverting"

"invertor"

"invertors"

"inverts"

"invest"

"investable"

"invested"

"investible"

"investigatable"

"investigate"

"investigated"

"investigates"

"investigating"

"investigation"

"investigational"

"investigations"

"investigative"

"investigator"

"investigators"

"investigatory"

"investing"

"investiture"

"investitures"

"investment"

"investments"

"investor"

"investors"

"invests"

"inveteracy"

"inveterate"

"inveterately"

"inviabilities"

"inviable"

"inviably"

"invidious"

"invidiously"

"invidiousness"

"invigorate"

"invigorated"

"invigorates"

"invigorating"

"invigoration"

"invigorations"

"invigorator"

"invincibility"

"invincible"

"invincibly"

"inviolability"

"inviolable"

"inviolably"

"inviolacy"

"inviolate"

"inviolated"

"inviolately"

"inviolateness"

"invisibility"

"invisible"

"invisibleness"

"invisibly"

"invitation"

"invitational"

"invitations"

"invite"

"invited"

"invitee"

"invitees"

"inviter"

"inviters"

"invites"

"inviting"

"invocable"

"invocate"

"invocated"

"invocates"

"invocating"

"invocation"

"invocational"

"invocations"

"invocator"

"invoice"

"invoiced"

"invoices"

"invoicing"

"invoke"

"invoked"

"invoker"

"invokers"

"invokes"

"invoking"

"involucre"

"involucres"

"involuntarily"

"involuntariness"

"involuntary"

"involute"

"involuted"

"involutes"

"involuting"

"involution"

"involutions"

"involve"

"involved"

"involvement"

"involvements"

"involver"

"involvers"

"involves"

"involving"

"invulnerability"

"invulnerable"

"invulnerably"

"inward"

"inwardly"

"inwards"

"inweave"

"inweaved"

"inweaves"

"inweaving"

"inwinding"

"inwinds"

"inwrapped"

"inwrought"

"iodide"

"iodides"

"iodin"

"iodinating"

"iodine"

"iodines"

"iodize"

"iodized"

"iodizer"

"iodizers"

"iodizes"

"iodizing"

"iodoform"

"iodoforms"

"ion"

"ionic"

"ionicity"

"ionics"

"ionise"

"ionised"

"ionises"

"ionising"

"ionium"

"ioniums"

"ionizable"

"ionization"

"ionizations"

"ionize"

"ionized"

"ionizer"

"ionizers"

"ionizes"

"ionizing"

"ionosphere"

"ionospheres"

"ionospheric"

"ions"

"iota"

"iotas"

"iou"

"iowa"

"iowan"

"iowans"

"ipecac"

"ipecacs"

"ipso"

"iqs"

"ira"

"irades"

"iran"

"iranian"

"iranians"

"iraq"

"iraqi"

"iraqis"

"irascibility"

"irascible"

"irate"

"irately"

"irateness"

"irater"

"iratest"

"ire"

"ired"

"ireful"

"irefully"

"ireland"

"ireless"

"irene"

"irenic"

"irenology"

"ires"

"iridectomies"

"irides"

"iridescence"

"iridescences"

"iridescent"

"iridic"

"iridium"

"iridiums"

"iridology"

"iring"

"iris"

"iriscope"

"irised"

"irises"

"irish"

"irishman"

"irishmen"

"irishwoman"

"irishwomen"

"irising"

"irk"

"irked"

"irking"

"irks"

"irksome"

"irksomely"

"iron"

"ironbark"

"ironbound"

"ironclad"

"ironclads"

"ironed"

"ironer"

"ironers"

"irones"

"ironic"

"ironical"

"ironically"

"ironies"

"ironing"

"ironings"

"ironist"

"ironists"

"irons"

"ironside"

"ironsides"

"ironstone"

"ironstones"

"ironware"

"ironwares"

"ironweed"

"ironwood"

"ironwoods"

"ironwork"

"ironworker"

"ironworkers"

"ironworks"

"irony"

"iroquoian"

"iroquoians"

"iroquois"

"irradiant"

"irradiate"

"irradiated"

"irradiates"

"irradiating"

"irradiation"

"irradiations"

"irrational"

"irrationalities"

"irrationality"

"irrationally"

"irrationalness"

"irreal"

"irrebuttable"

"irreclaimable"

"irreclaimably"

"irreconcilability"

"irreconcilable"

"irreconcilably"

"irrecoverable"

"irrecoverably"

"irredeemability"

"irredeemable"

"irredeemably"

"irredentism"

"irredentist"

"irredentists"

"irreducibilities"

"irreducibility"

"irreducible"

"irreducibly"

"irreformable"

"irrefragable"

"irrefutability"

"irrefutable"

"irrefutably"

"irregardless"

"irregular"

"irregularities"

"irregularity"

"irregularly"

"irregulars"

"irrelevance"

"irrelevances"

"irrelevancies"

"irrelevancy"

"irrelevant"

"irrelevantly"

"irreligious"

"irreligiousness"

"irremediable"

"irremediableness"

"irremediably"

"irremovable"

"irremovably"

"irreparable"

"irreparableness"

"irreparably"

"irrepatriable"

"irreplaceable"

"irreplaceably"

"irrepressible"

"irrepressibly"

"irreproachable"

"irreproachably"

"irresistible"

"irresistibly"

"irresolute"

"irresolutely"

"irresolution"

"irrespective"

"irrespectively"

"irresponsibilities"

"irresponsibility"

"irresponsible"

"irresponsibleness"

"irresponsibly"

"irresuscitable"

"irretrievability"

"irretrievable"

"irretrievably"

"irreverence"

"irreverences"

"irreverent"

"irreverently"

"irreversibility"

"irreversible"

"irreversibly"

"irrevocability"

"irrevocable"

"irrevocableness"

"irrevocably"

"irrigable"

"irrigate"

"irrigated"

"irrigates"

"irrigating"

"irrigation"

"irrigations"

"irrigator"

"irrigators"

"irritabilities"

"irritability"

"irritable"

"irritableness"

"irritably"

"irritancies"

"irritancy"

"irritant"

"irritants"

"irritate"

"irritated"

"irritates"

"irritating"

"irritatingly"

"irritation"

"irritations"

"irritative"

"irrupt"

"irrupted"

"irrupting"

"irruption"

"irruptions"

"irruptive"

"irrupts"

"irs"

"is"

"isaac"

"isaiah"

"iscariot"

"iscose"

"isinglass"

"isis"

"islam"

"islamic"

"island"

"islanded"

"islander"

"islanders"

"islanding"

"islands"

"isle"

"isled"

"isles"

"islet"

"islets"

"isling"

"islomania"

"ism"

"isms"

"isobar"

"isobaric"

"isobars"

"Isochronic Tones"

"isocline"

"isoclines"

"isogamy"

"isogon"

"isolable"

"isolate"

"isolated"

"isolates"

"isolating"

"isolation"

"isolationism"

"isolationist"

"isolationists"

"isolator"

"isolators"

"isolog"

"isologs"

"isomer"

"isomeric"

"isomerism"

"isomerization"

"isomerize"

"isomerizing"

"isomerous"

"isomers"

"isometric"

"isometrical"

"isometrically"

"isometrics"

"isometries"

"isometry"

"isomorph"

"isomorphism"

"isomorphs"

"isopod"

"isoprene"

"isopropanol"

"isopropyl"

"isosceles"

"isostasy"

"isostatic"

"isostatically"

"isotherm"

"isothermal"

"isotherms"

"isotonic"

"isotonically"

"isotope"

"isotopes"

"isotopic"

"isotopically"

"isotopy"

"isotropic"

"ispanic"

"israel"

"israeli"

"israelis"

"israelite"

"israelites"

"issei"

"isseis"

"issuable"

"issuably"

"issuance"

"issuances"

"issuant"

"issue"

"issued"

"issueless"

"issuer"

"issuers"

"issues"

"issuing"

"ist"

"istanbul"

"isthmi"

"isthmian"

"isthmic"

"isthmus"

"isthmuses"

"istle"

"it"

"ital"

"italian"

"italians"

"italic"

"italicize"

"italicized"

"italicizes"

"italicizing"

"italics"

"Italomania"

"italy"

"itch"

"itched"

"itches"

"itchier"

"itchiest"

"itchiness"

"itching"

"itchings"

"itchy"

"item"

"itemed"

"iteming"

"itemization"

"itemizations"

"itemize"

"itemized"

"itemizer"

"itemizers"

"itemizes"

"itemizing"

"items"

"iterances"

"iterant"

"iterate"

"iterated"

"iterates"

"iterating"

"iteration"

"iterations"

"iterative"

"iterum"

"itinerant"

"itinerants"

"itineraries"

"itinerary"

"its"

"itself"

"iud"

"iuds"

"iv"

"ivi"

"ivied"

"ivies"

"ivories"

"ivory"

"ivy"

"ixia"

"ixias"

"ixtles"

"izar"

"izzard"

"izzards"

"jab"

"jabbed"

"jabber"

"jabbered"

"jabberer"

"jabberers"

"jabbering"

"jabbers"

"jabbing"

"jabbingly"

"jabot"

"jabots"

"jabs"

"jacal"

"jacals"

"jacaranda"

"jacarandas"

"jacinth"

"jacinthe"

"jacinths"

"jack"

"jackal"

"jackals"

"jackanapes"

"jackanapeses"

"jackass"

"jackasses"

"jackboot"

"jackboots"

"jackdaw"

"jackdaws"

"jacked"

"jacker"

"jackeroo"

"jackeroos"

"jackers"

"jacket"

"jacketed"

"jacketing"

"jacketless"

"jackets"

"jackfish"

"jackfishes"

"jackhammer"

"jackhammers"

"jackie"

"jackies"

"jacking"

"jackknife"

"jackknifed"

"jackknifes"

"jackknifing"

"jackknives"

"jackleg"

"jacklegs"

"jackpot"

"jackpots"

"jackrabbit"

"jackroll"

"jacks"

"jackscrew"

"jackscrews"

"jackson"

"jacksonian"

"jacksonville"

"jackstraw"

"jackstraws"

"jacky"

"jacob"

"jacobean"

"jacobin"

"jacobins"

"jacobus"

"jacquard"

"jacquards"

"jacqueline"

"jade"

"jaded"

"jadedly"

"jadedness"

"jadeite"

"jadeites"

"jades"

"jading"

"jadish"

"jadishly"

"jaegars"

"jag"

"jagged"

"jaggeder"

"jaggedest"

"jaggedly"

"jaggedness"

"jagger"

"jaggers"

"jaggery"

"jaggier"

"jaggiest"

"jagging"

"jaggs"

"jaggy"

"jagless"

"jags"

"jaguar"

"jaguars"

"jai"

"jail"

"jailbait"

"jailbird"

"jailbirds"

"jailbreak"

"jailbreaker"

"jailbreaks"

"jailed"

"jailer"

"jailers"

"jailhouse"

"jailing"

"jailkeeper"

"jailor"

"jailors"

"jails"

"jakarta"

"jake"

"jakes"

"jalap"

"jalopies"

"jaloppy"

"jalopy"

"jalousie"

"jalousies"

"jam"

"jamaica"

"jamaican"

"jamaicans"

"jamb"

"jambed"

"jambing"

"jamboree"

"jamborees"

"jambs"

"james"

"jamestown"

"jammed"

"jammer"

"jammers"

"jamming"

"jams"

"jane"

"janeiro"

"janes"

"janet"

"jangle"

"jangled"

"jangler"

"janglers"

"jangles"

"jangling"

"jangly"

"janisary"

"janitor"

"janitorial"

"janitors"

"janitress"

"janitresses"

"janizary"

"jansky"

"january"

"janus"

"jap"

"japan"

"japanese"

"japanize"

"japanized"

"japanizes"

"japanizing"

"japanned"

"japanner"

"japanners"

"japanning"

"japans"

"jape"

"japed"

"japer"

"japeries"

"japers"

"japery"

"japes"

"japing"

"japingly"

"japonica"

"japonicas"

"jar"

"jardiniere"

"jardinieres"

"jarful"

"jarfuls"

"jargon"

"jargoned"

"jargoning"

"jargonize"

"jargonized"

"jargonizing"

"jargons"

"jarred"

"jarring"

"jars"

"jarsful"

"jasmine"

"jasmines"

"jason"

"jasper"

"jaspers"

"jaspery"

"jato"

"jatos"

"jaundice"

"jaundiced"

"jaundices"

"jaundicing"

"jaunt"

"jaunted"

"jauntier"

"jauntiest"

"jauntily"

"jauntiness"

"jaunting"

"jaunts"

"jaunty"

"java"

"javanese"

"javas"

"javelin"

"javelined"

"javelins"

"jaw"

"jawbone"

"jawboned"

"jawbones"

"jawboning"

"jawbreaker"

"jawbreakers"

"jawed"

"jawing"

"jawless"

"jawline"

"jawlines"

"jaws"

"jay"

"jaybird"

"jaybirds"

"jaycee"

"jaycees"

"jaygee"

"jaygees"

"jays"

"jayvee"

"jayvees"

"jaywalk"

"jaywalked"

"jaywalker"

"jaywalkers"

"jaywalking"

"jaywalks"

"jazz"

"jazzed"

"jazzer"

"jazzers"

"jazzes"

"jazzier"

"jazziest"

"jazzily"

"jazziness"

"jazzing"

"jazzman"

"jazzmen"

"jazzy"

"jct"

"jealous"

"jealousies"

"jealously"

"jealousness"

"jealousy"

"jean"

"jeannette"

"jeans"

"jeep"

"jeepers"

"jeeps"

"jeer"

"jeered"

"jeerer"

"jeerers"

"jeering"

"jeeringly"

"jeers"

"jeez"

"jefe"

"jefes"

"jefferson"

"jeffersonian"

"jeffersonians"

"jehad"

"jehus"

"jejunal"

"jejune"

"jejunely"

"jejunity"

"jejunum"

"jejunums"

"jekyll"

"jell"

"jelled"

"jellied"

"jellies"

"jellified"

"jellifies"

"jellify"

"jellifying"

"jelling"

"jells"

"jelly"

"jellybean"

"jellybeans"

"jellyfish"

"jellyfishes"

"jellying"

"jellylike"

"jemmied"

"jemmies"

"jemmy"

"jennet"

"jennets"

"jennies"

"jenny"

"jeopard"

"jeopardied"

"jeopardies"

"jeoparding"

"jeopardize"

"jeopardized"

"jeopardizes"

"jeopardizing"

"jeopardous"

"jeopardy"

"jerboa"

"jerboas"

"jeremiad"

"jeremiads"

"jeremiah"

"jerk"

"jerked"

"jerker"

"jerkers"

"jerkier"

"jerkies"

"jerkiest"

"jerkily"

"jerkin"

"jerkiness"

"jerking"

"jerkins"

"jerks"

"jerkwater"

"jerky"

"jeroboam"

"jeroboams"

"jerries"

"jerry"

"jerrycan"

"jerrycans"

"jersey"

"jerseyed"

"jerseyite"

"jerseyites"

"jerseys"

"jerusalem"

"jess"

"jesse"

"jessed"

"jesses"

"jest"

"jested"

"jester"

"jesters"

"jestful"

"jesting"

"jestings"

"jests"

"jesuit"

"jesuitic"

"jesuitical"

"jesuitries"

"jesuitry"

"jesuits"

"jesus"

"jet"

"jetliner"

"jetliners"

"jetport"

"jetports"

"jets"

"jetsam"

"jetsams"

"jetsom"

"jetsoms"

"jetted"

"jettied"

"jetties"

"jetting"

"jettison"

"jettisoned"

"jettisoning"

"jettisons"

"jetty"

"jettying"

"jeu"

"jeux"

"jew"

"jewed"

"jewel"

"jeweled"

"jeweler"

"jewelers"

"jeweling"

"jewelled"

"jeweller"

"jewellers"

"jewelling"

"jewelries"

"jewelry"

"jewels"

"jewelweed"

"jewelweeds"

"jewfish"

"jewfishes"

"jewing"

"jewish"

"jewishness"

"jewry"

"jews"

"jezebel"

"jezebels"

"jib"

"jibbed"

"jibber"

"jibbers"

"jibbing"

"jibe"

"jibed"

"jiber"

"jibers"

"jibes"

"jibing"

"jibingly"

"jibs"

"jiff"

"jiffies"

"jiffs"

"jiffy"

"jig"

"jigaboo"

"jigaboos"

"jigged"

"jigger"

"jiggered"

"jiggers"

"jigging"

"jiggle"

"jiggled"

"jiggles"

"jigglier"

"jiggliest"

"jiggling"

"jiggly"

"jigs"

"jigsaw"

"jigsawed"

"jigsawing"

"jigsawn"

"jigsaws"

"jihad"

"jihads"

"jill"

"jillion"

"jillions"

"jills"

"jilt"

"jilted"

"jilter"

"jilters"

"jilting"

"jilts"

"jim"

"jiminy"

"jimjams"

"jimmied"

"jimmies"

"jimminy"

"jimmy"

"jimmying"

"jimsonweed"

"jingle"

"jingled"

"jingler"

"jinglers"

"jingles"

"jinglier"

"jingliest"

"jingling"

"jingly"

"jingo"

"jingoes"

"jingoish"

"jingoism"

"jingoisms"

"jingoist"

"jingoistic"

"jingoists"

"jinn"

"jinnee"

"jinni"

"jinns"

"jinrikisha"

"jinrikishas"

"jins"

"jinx"

"jinxed"

"jinxes"

"jinxing"

"jitney"

"jitneys"

"jitter"

"jitterbug"

"jitterbugged"

"jitterbugging"

"jitterbugs"

"jittered"

"jittering"

"jitters"

"jittery"

"jiujitsu"

"jiujitsus"

"jiujutsu"

"jiujutsus"

"jive"

"jived"

"jives"

"jiving"

"jnana"

"jnanas"

"job"

"jobbed"

"jobber"

"jobbers"

"jobbing"

"jobholder"

"jobholders"

"jobless"

"joblessness"

"joblots"

"jobs"

"jock"

"jockey"

"jockeyed"

"jockeying"

"jockeys"

"jocko"

"jockos"

"jocks"

"jockstrap"

"jockstraps"

"jocose"

"jocosely"

"jocoseness"

"jocosities"

"jocosity"

"jocular"

"jocularity"

"jocund"

"jocundities"

"jocundity"

"jocundly"

"jodhpur"

"jodhpurs"

"joe"

"joes"

"joey"

"joeys"

"jog"

"jogged"

"jogger"

"joggers"

"jogging"

"joggle"

"joggled"

"joggler"

"jogglers"

"joggles"

"joggling"

"jogs"

"johannes"

"johannesburg"

"john"

"johnnie"

"johnnies"

"johnny"

"johns"

"johnson"

"joie"

"join"

"joinable"

"joined"

"joiner"

"joineries"

"joiners"

"joinery"

"joining"

"joinings"

"joins"

"joint"

"jointed"

"jointer"

"jointers"

"jointing"

"jointly"

"joints"

"jointure"

"jointuring"

"joist"

"joisted"

"joisting"

"joists"

"jojoba"

"jojobas"

"joke"

"joked"

"joker"

"jokers"

"jokes"

"jokester"

"jokesters"

"joking"

"jokingly"

"jollied"

"jollier"

"jollies"

"jolliest"

"jollification"

"jollifications"

"jollified"

"jollifies"

"jollify"

"jollifying"

"jollily"

"jolliness"

"jollities"

"jollity"

"jolly"

"jollying"

"jolt"

"jolted"

"jolter"

"jolters"

"joltier"

"joltily"

"jolting"

"jolts"

"jolty"

"jonah"

"jonahs"

"jonathan"

"jones"

"joneses"

"jongleur"

"jongleurs"

"jonquil"

"jonquils"

"joram"

"jordan"

"jordanian"

"jordanians"

"jordans"

"jorum"

"jose"

"joseph"

"josephine"

"josephs"

"josh"

"joshed"

"josher"

"joshers"

"joshes"

"joshing"

"joshua"

"joss"

"josses"

"jostle"

"jostled"

"jostler"

"jostlers"

"jostles"

"jostling"

"jot"

"jota"

"jots"

"jotted"

"jotter"

"jotters"

"jotting"

"jottings"

"jotty"

"joule"

"joules"

"jounce"

"jounced"

"jounces"

"jouncier"

"jounciest"

"jouncing"

"jouncy"

"jour"

"journal"

"journalese"

"journalism"

"journalist"

"journalistic"

"journalistically"

"journalists"

"journalize"

"journalized"

"journalizing"

"journals"

"journey"

"journeyed"

"journeyer"

"journeyers"

"journeying"

"journeyman"

"journeymen"

"journeys"

"joust"

"jousted"

"jouster"

"jousters"

"jousting"

"jousts"

"jovial"

"joviality"

"jovially"

"jow"

"jowl"

"jowled"

"jowlier"

"jowliest"

"jowls"

"jowly"

"joy"

"joyance"

"joyce"

"joyed"

"joyful"

"joyfuller"

"joyfullest"

"joyfully"

"joyfulness"

"joying"

"joyless"

"joylessness"

"joyous"

"joyously"

"joyousness"

"joyridden"

"joyride"

"joyrider"

"joyriders"

"joyrides"

"joyriding"

"joyrode"

"joys"

"joystick"

"joysticks"

"juan"

"juans"

"jubilant"

"jubilantly"

"jubilate"

"jubilated"

"jubilates"

"jubilating"

"jubilation"

"jubilations"

"jubile"

"jubilee"

"jubilees"

"jubiles"

"jud"

"judaic"

"judaica"

"judaical"

"judaism"

"judas"

"judases"

"judder"

"judge"

"judged"

"judgelike"

"judgement"

"judger"

"judgers"

"judges"

"judgeship"

"judgeships"

"judging"

"judgmatic"

"judgment"

"judgmental"

"judgments"

"judicatories"

"judicatory"

"judicature"

"judicatures"

"judice"

"judicial"

"judicialized"

"judicializing"

"judicially"

"judiciaries"

"judiciary"

"judicious"

"judiciously"

"judiciousness"

"judith"

"judo"

"judoist"

"judoists"

"judos"

"judy"

"jug"

"jugful"

"jugfuls"

"jugged"

"juggernaut"

"juggernauts"

"jugging"

"juggle"

"juggled"

"juggler"

"juggleries"

"jugglers"

"jugglery"

"juggles"

"juggling"

"jugglingly"

"jugglings"

"jughead"

"jugheads"

"jugs"

"jugsful"

"jugula"

"jugular"

"jugulars"

"jugulate"

"jugulated"

"jugulates"

"juice"

"juiced"

"juiceless"

"juicer"

"juicers"

"juices"

"juicier"

"juiciest"

"juicily"

"juiciness"

"juicing"

"juicy"

"jujitsu"

"jujitsus"

"juju"

"jujube"

"jujubes"

"jujuism"

"jujuist"

"jujus"

"jujutsu"

"jujutsus"

"juke"

"jukebox"

"jukeboxes"

"juked"

"jukes"

"juking"

"julep"

"juleps"

"julienne"

"juliennes"

"julius"

"july"

"jumble"

"jumbled"

"jumbler"

"jumblers"

"jumbles"

"jumbling"

"jumbo"

"jumbos"

"jumbuck"

"jumbucks"

"jump"

"jumpable"

"jumped"

"jumper"

"jumpers"

"jumpier"

"jumpiest"

"jumpily"

"jumpiness"

"jumping"

"jumpingly"

"jumpoff"

"jumpoffs"

"jumps"

"jumpy"

"junco"

"juncoes"

"juncos"

"junction"

"junctional"

"junctions"

"juncture"

"junctures"

"june"

"juneau"

"jungian"

"jungle"

"jungles"

"junglier"

"jungliest"

"jungly"

"junior"

"juniors"

"juniper"

"junipers"

"junk"

"junked"

"junker"

"junkers"

"junket"

"junketed"

"junketeer"

"junketeers"

"junketer"

"junketers"

"junketing"

"junkets"

"junkie"

"junkier"

"junkies"

"junkiest"

"junking"

"junkman"

"junkmen"

"junks"

"junky"

"junkyard"

"junkyards"

"juno"

"junta"

"juntas"

"junto"

"juntos"

"jupe"

"jupiter"

"jurassic"

"jurator"

"juratory"

"jure"

"juridic"

"juridical"

"juridically"

"juries"

"jurisdiction"

"jurisdictional"

"jurisdictionally"

"jurisdictions"

"jurisdictive"

"jurisprudence"

"jurisprudent"

"jurisprudential"

"jurist"

"juristic"

"juristically"

"jurists"

"juror"

"jurors"

"jury"

"juryless"

"juryman"

"jurymen"

"jurywoman"

"jurywomen"

"jus"

"just"

"justed"

"juster"

"justers"

"justest"

"justice"

"justices"

"justiceship"

"justiciable"

"justiciary"

"justifiable"

"justifiably"

"justification"

"justifications"

"justified"

"justifier"

"justifiers"

"justifies"

"justify"

"justifying"

"justing"

"justinian"

"justle"

"justly"

"justness"

"justs"

"jut"

"jute"

"jutes"

"juts"

"jutted"

"jutting"

"juttingly"

"jutty"

"juvenal"

"juvenile"

"juveniles"

"juvenilities"

"juvenility"

"juxta"

"juxtapose"

"juxtaposed"

"juxtaposes"

"juxtaposing"

"juxtaposition"

"juxtapositions"

"kabala"

"kabalas"

"kabbala"

"kabbalah"

"kabbalahs"

"kabbalas"

"kabob"

"kabobs"

"kabuki"

"kabukis"

"kachina"

"kachinas"

"kaddish"

"kaddishes"

"kadis"

"kadish"

"kadishim"

"kaffir"

"kaffirs"

"kafir"

"kafirs"

"kafka"

"kaftan"

"kaftans"

"kahuna"

"kahunas"

"kaiak"

"kainotophobia"

"kaiser"

"kaisers"

"kajeput"

"kajeputs"

"kaka"

"kakas"

"kakemono"

"kakemonos"

"kakis"

"kakistocracies"

"kakistocracy"

"kakogenic"

"kakorrhaphiophobia"

"kale"

"kaleidoscope"

"kaleidoscopes"

"kaleidoscopic"

"kaleidoscopically"

"kalends"

"kales"

"kaleyards"

"kalif"

"kalifate"

"kalifs"

"kalimba"

"kalimbas"

"kaliph"

"kaliphs"

"kalium"

"kaliums"

"kalology"

"kalpa"

"kalpas"

"kamaaina"

"kamaainas"

"kame"

"kames"

"kamikaze"

"kamikazes"

"kampuchea"

"kanas"

"kangaroo"

"kangaroos"

"kanji"

"kanjis"

"kansan"

"kansans"

"kansas"

"kantian"

"kantians"

"kaolin"

"kaons"

"kapok"

"kapoks"

"kappa"

"kappas"

"kaput"

"kaputt"

"karakul"

"karakuls"

"karat"

"karate"

"karates"

"karats"

"karen"

"karma"

"karmas"

"karmic"

"karst"

"karsts"

"kart"

"karts"

"karyocyte"

"karyology"

"karyotype"

"kasha"

"kashas"

"kashmir"

"kashmirs"

"katabolism"

"katakana"

"katakanas"

"katathermometer"

"katharine"

"katharometer"

"katharses"

"katharsis"

"kathartic"

"katherine"

"kathy"

"katrina"

"katydid"

"katydids"

"katzenjammer"

"kayak"

"kayaker"

"kayakers"

"kayaks"

"kayo"

"kayoed"

"kayoes"

"kayoing"

"kayos"

"kays"

"kazoo"

"kazoos"

"kb"

"keats"

"keb"

"kebab"

"kebabs"

"kebob"

"kebobs"

"ked"

"kedge"

"kedged"

"kedges"

"kedging"

"keefs"

"keel"

"keelage"

"keelboats"

"keeled"

"keeler"

"keelhaul"

"keelhauled"

"keelhauls"

"keeling"

"keelless"

"keels"

"keen"

"keened"

"keener"

"keeners"

"keenest"

"keening"

"keenly"

"keenness"

"keens"

"keep"

"keepable"

"keeper"

"keepers"

"keeping"

"keepings"

"keeps"

"keepsake"

"keepsakes"

"keester"

"keesters"

"kef"

"kefir"

"kefirs"

"keg"

"kegler"

"keglers"

"kegs"

"keister"

"keisters"

"keloid"

"keloidal"

"keloids"

"kelp"

"kelped"

"kelpie"

"kelpies"

"kelping"

"kelps"

"kelpy"

"keltic"

"keltics"

"kelts"

"kelvin"

"kelvins"

"kempt"

"ken"

"kendo"

"kendos"

"kenned"

"kennedy"

"kennel"

"kenneled"

"kenneling"

"kennelled"

"kennelling"

"kennels"

"kenning"

"kennings"

"kenny"

"keno"

"kenophobia"

"kenos"

"kenosis"

"kenosises"

"kens"

"kent"

"kentuckian"

"kentuckians"

"kentucky"

"kenya"

"kenyans"

"kep"

"kepi"

"kepis"

"kept"

"keratin"

"keratins"

"keratitis"

"keratoid"

"keratomas"

"keratometer"

"keratoses"

"keratosis"

"keratotic"

"keratotomies"

"keraunograph"

"kerb"

"kerbed"

"kerbing"

"kerbs"

"kerchief"

"kerchiefs"

"kerchieves"

"kerchoo"

"kerf"

"kerfed"

"kerfing"

"kerfs"

"kern"

"kerned"

"kernel"

"kerneled"

"kerneling"

"kernelled"

"kernelling"

"kernels"

"kerning"

"kerns"

"kerosene"

"kerosenes"

"kerosine"

"kerplunk"

"kerry"

"kestrel"

"kestrels"

"ket"

"ketch"

"ketches"

"ketchup"

"ketchups"

"ketone"

"ketones"

"ketonuria"

"kettle"

"kettledrum"

"kettledrums"

"kettles"

"kevels"

"kevils"

"kex"

"key"

"key point"

"key words"

"keyage"

"keyboard"

"keyboarded"

"keyboards"

"keyed"

"keyhole"

"keyholes"

"keying"

"keyless"

"keyman"

"keynote"

"keynoted"

"keynoter"

"keynoters"

"keynotes"

"keynoting"

"keypad"

"keypunch"

"keypunched"

"keypuncher"

"keypunchers"

"keypunches"

"keypunching"

"keys"

"keyset"

"keysets"

"keyster"

"keysters"

"keystone"

"keystones"

"keystroke"

"keystrokes"

"keyway"

"keyways"

"keyword"

"keywords"

"khaki"

"khakis"

"khalif"

"khalifa"

"khalifs"

"khan"

"khanate"

"khanates"

"khans"

"khartoum"

"khats"

"khedive"

"khedives"

"khrushchev"

"kibble"

"kibbled"

"kibbles"

"kibbling"

"kibbutz"

"kibbutzim"

"kibitz"

"kibitzed"

"kibitzer"

"kibitzers"

"kibitzes"

"kibitzing"

"kibosh"

"kiboshed"

"kiboshes"

"kiboshing"

"kick"

"kickback"

"kickbacks"

"kicked"

"kicker"

"kickers"

"kickier"

"kickiest"

"kicking"

"kickoff"

"kickoffs"

"kicks"

"kickshaw"

"kickshaws"

"kickstand"

"kickstands"

"kickup"

"kickups"

"kicky"

"kid"

"kidded"

"kidder"

"kidders"

"kiddie"

"kiddies"

"kidding"

"kiddingly"

"kiddish"

"kiddo"

"kiddoes"

"kiddos"

"kiddy"

"kidnap"

"kidnaped"

"kidnapee"

"kidnaper"

"kidnapers"

"kidnaping"

"kidnapped"

"kidnapper"

"kidnappers"

"kidnapping"

"kidnaps"

"kidney"

"kidney stones"

"kidneys"

"kidology"

"kids"

"Kidsâ€™ illnesses"

"kidskin"

"kidskins"

"kidvid"

"kiefs"

"kielbasa"

"kielbasas"

"kielbasy"

"kieselguhr"

"kiester"

"kiesters"

"kiev"

"kif"

"kike"

"kikes"

"kilderkin"

"kill"

"killdee"

"killdeer"

"killdeers"

"killdees"

"killed"

"killer"

"killers"

"killing"

"killings"

"killjoy"

"killjoys"

"kills"

"kiln"

"kilned"

"kilning"

"kilns"

"kilo"

"kilobar"

"kilobase"

"kilobit"

"kilobits"

"kilobyte"

"kilobytes"

"kilocycle"

"kilocycles"

"kilogram"

"kilograms"

"kilohertz"

"kiloliter"

"kilometer"

"kilometers"

"kilorad"

"kilorads"

"kilos"

"kiloton"

"kilotons"

"kilovolt"

"kilovolts"

"kilowatt"

"kilowatts"

"kilt"

"kilted"

"kilter"

"kilters"

"kiltie"

"kilties"

"kilting"

"kilts"

"kilty"

"kimono"

"kimonoed"

"kimonos"

"kin"

"kinaestheic"

"kinaesthesia"

"kinaesthesias"

"kinaesthetic"

"kinaesthetically"

"kind"

"kind of stress"

"kinder"

"kindergarten"

"kindergartens"

"kindergartner"

"kindergartners"

"kindest"

"kindhearted"

"kindheartedly"

"kindheartedness"

"kindle"

"kindled"

"kindler"

"kindlers"

"kindles"

"kindlier"

"kindliest"

"kindliness"

"kindling"

"kindlings"

"kindly"

"kindness"

"kindnesses"

"kindred"

"kindredless"

"kindredness"

"kindreds"

"kindredship"

"kinds"

"kine"

"kinema"

"kinemas"

"kinematic"

"kinematical"

"kinematically"

"kinematics"

"kinematograph"

"kineplasty"

"kines"

"kinescope"

"kinescopes"

"kineses"

"kinesic"

"kinesics"

"kinesiologic"

"kinesiological"

"kinesiologies"

"kinesiology"

"kinesis"

"kinesthesia"

"kinesthesias"

"kinesthetic"

"kinesthetically"

"kinetic"

"kinetics"

"kinetins"

"kinetoscope"

"kinfolk"

"kinfolks"

"king"

"kingdom"

"kingdoms"

"kinged"

"kingfish"

"kingfisher"

"kingfishers"

"kingfishes"

"kinghoods"

"kinging"

"kingless"

"kinglet"

"kinglets"

"kinglier"

"kingliest"

"kingliness"

"kingly"

"kingpin"

"kingpins"

"kings"

"kingship"

"kingships"

"kingside"

"kingwood"

"kinhin"

"kink"

"kinkajou"

"kinkajous"

"kinked"

"kinkier"

"kinkiest"

"kinkily"

"kinkiness"

"kinking"

"kinks"

"kinky"

"kinless"

"kins"

"kinsfolk"

"kinship"

"kinships"

"kinsman"

"kinsmanship"

"kinsmen"

"kinspeople"

"kinswoman"

"kinswomen"

"kiosk"

"kiosks"

"kiowa"

"kip"

"kipper"

"kippered"

"kippering"

"kippers"

"kippur"

"kips"

"kipskins"

"kir"

"kirigami"

"kirigamis"

"kirk"

"kirkman"

"kirkmen"

"kirks"

"kirned"

"kirsch"

"kirsches"

"kirtle"

"kirtled"

"kirtles"

"kishka"

"kishkas"

"kishkes"

"kismet"

"kismetic"

"kismets"

"kiss"

"kissable"

"kissably"

"kissed"

"kisser"

"kissers"

"kisses"

"kissing"

"kist"

"kit"

"kitchen"

"kitchenette"

"kitchenettes"

"kitchens"

"kitchenware"

"kite"

"kited"

"kiter"

"kiters"

"kites"

"kith"

"kithara"

"kitharas"

"kithing"

"kiths"

"kiting"

"kitling"

"kitlings"

"kits"

"kitsch"

"kitsches"

"kitschy"

"kitted"

"kitten"

"kittened"

"kittening"

"kittenish"

"kittenishly"

"kittens"

"kitties"

"kitting"

"kitty"

"kivas"

"kiwi"

"kiwis"

"kl"

"klanism"

"klans"

"klatch"

"klatches"

"klatsch"

"klatsches"

"klaxon"

"klaxons"

"kleig"

"kleptomania"

"kleptomaniac"

"kleptomaniacs"

"klieg"

"klopemania"

"kludge"

"kludged"

"kludges"

"kludging"

"klutz"

"klutzes"

"klutzier"

"klutziest"

"klutzy"

"klystron"

"klystrons"

"knack"

"knacked"

"knacker"

"knackeries"

"knackers"

"knackery"

"knacking"

"knacks"

"knackwurst"

"knackwursts"

"knapped"

"knapper"

"knappers"

"knapping"

"knaps"

"knapsack"

"knapsacks"

"knapweeds"

"knave"

"knaveries"

"knavery"

"knaves"

"knavish"

"knavishly"

"knavishness"

"knead"

"kneaded"

"kneader"

"kneaders"

"kneading"

"kneads"

"knee"

"kneecap"

"kneecapping"

"kneecappings"

"kneecaps"

"kneed"

"kneehole"

"kneeholes"

"kneeing"

"kneel"

"kneeled"

"kneeler"

"kneelers"

"kneeling"

"kneels"

"kneepad"

"kneepads"

"kneepan"

"knees"

"knell"

"knelled"

"knelling"

"knells"

"knelt"

"knew"

"knickerbockers"

"knickers"

"knickknack"

"knickknacks"

"knife"

"knifed"

"knifer"

"knifers"

"knifes"

"knifing"

"knifings"

"knight"

"knighted"

"knighthood"

"knighthoods"

"knighting"

"knightly"

"knights"

"knish"

"knishes"

"knit"

"knits"

"knitted"

"knitter"

"knitters"

"knitting"

"knittings"

"knitwear"

"knitwears"

"knives"

"knob"

"knobbed"

"knobbier"

"knobbiest"

"knobbiness"

"knobby"

"knobs"

"knock"

"knockdown"

"knockdowns"

"knocked"

"knocker"

"knockers"

"knocking"

"knockoff"

"knockoffs"

"knockout"

"knockouts"

"knocks"

"knockwurst"

"knockwursts"

"knoll"

"knolls"

"knolly"

"knot"

"knothole"

"knotholes"

"knots"

"knotted"

"knotter"

"knotters"

"knottier"

"knottiest"

"knottily"

"knottiness"

"knotting"

"knotty"

"knotweed"

"knotweeds"

"knout"

"knouted"

"knouting"

"knouts"

"know"

"knowable"

"knower"

"knowers"

"knowhow"

"knowhows"

"knowing"

"knowinger"

"knowingest"

"knowingly"

"knowingness"

"knowings"

"knowledge"

"knowledgeability"

"knowledgeable"

"knowledgeably"

"knowledged"

"knowledgeless"

"known"

"known issue"

"knowns"

"knows"

"knox"

"knoxville"

"knuckle"

"knuckleball"

"knucklebone"

"knucklebones"

"knuckled"

"knucklehead"

"knuckleheads"

"knuckler"

"knucklers"

"knuckles"

"knucklier"

"knuckliest"

"knuckling"

"knuckly"

"knucks"

"knurl"

"knurled"

"knurlier"

"knurliest"

"knurling"

"knurls"

"knurly"

"koala"

"koalas"

"koan"

"koans"

"kobold"

"kobolds"

"kodak"

"kodiak"

"kohl"

"kohlrabi"

"kohlrabies"

"kohls"

"kola"

"kolas"

"kolinskies"

"kolinsky"

"kolkhoz"

"komondors"

"kong"

"konimeter"

"koniology"

"koniscope"

"koodoos"

"kook"

"kookaburra"

"kookie"

"kookier"

"kookiest"

"kookiness"

"kooks"

"kooky"

"kop"

"kopeck"

"kopecks"

"kopek"

"kopeks"

"kophs"

"kopje"

"kopjes"

"koppies"

"koran"

"korea"

"korean"

"koreans"

"korsakoff"

"korsakow"

"koruna"

"korunas"

"koruny"

"kosher"

"koshered"

"koshering"

"koshers"

"koto"

"kotos"

"kowtow"

"kowtowed"

"kowtower"

"kowtowers"

"kowtowing"

"kowtows"

"kraal"

"kraals"

"kraft"

"krafts"

"krait"

"kraits"

"kraken"

"krakens"

"kraut"

"krauts"

"krebs"

"kremlin"

"kremlinologist"

"kremlinologists"

"kremlinology"

"kremlins"

"kreutzer"

"kreuzers"

"krill"

"krills"

"kris"

"krises"

"krishna"

"krispies"

"krona"

"krone"

"kronen"

"kroner"

"kronor"

"kronur"

"kryolites"

"kryoliths"

"krypton"

"kryptonite"

"kryptons"

"ktenology"

"kuchen"

"kuchens"

"kudo"

"kudos"

"kudu"

"kudus"

"kudzu"

"kudzus"

"kulak"

"kulaks"

"kultur"

"kulturs"

"kumiss"

"kummels"

"kumquat"

"kumquats"

"kumshaw"

"kung"

"kuwait"

"kvetch"

"kvetched"

"kvetches"

"kvetching"

"kwacha"

"kwachas"

"kwashiorkor"

"kwhr"

"kyanising"

"kyanizing"

"kyat"

"kyats"

"kymatology"

"kymograms"

"kymograph"

"kynurenic"

"kyoto"

"kyrie"

"kyries"

"la"

"laager"

"lab"

"label"

"label and short descriptions"

"labeled"

"labeler"

"labelers"

"labeling"

"labella"

"labelled"

"labeller"

"labellers"

"labelling"

"labels"

"labeorphily"

"labia"

"labial"

"labially"

"labials"

"labiate"

"labidometer"

"labile"

"labium"

"labor"

"laboratorial"

"laboratorially"

"laboratorian"

"laboratories"

"laboratory"

"labored"

"laboredly"

"laborer"

"laborers"

"laboring"

"laboringly"

"laborings"

"laborious"

"laboriously"

"laboriousness"

"laborite"

"laborites"

"labors"

"laborsaving"

"labour"

"laboured"

"labourer"

"labourers"

"labouring"

"labours"

"labrador"

"labradorite"

"labs"

"laburnum"

"laburnums"

"labyrinth"

"labyrinthine"

"labyrinths"

"lac"

"laccolith"

"laccoliths"

"lace"

"laced"

"laceier"

"lacer"

"lacerable"

"lacerate"

"lacerated"

"lacerates"

"lacerating"

"laceration"

"lacerations"

"lacerative"

"lacers"

"laces"

"lacewing"

"lacewings"

"lacework"

"laceworks"

"lacey"

"lachrymal"

"lachrymation"

"lachrymator"

"lachrymatory"

"lachrymose"

"lacier"

"laciest"

"lacily"

"laciness"

"lacing"

"lacings"

"lack"

"lackadaisical"

"lackadaisically"

"lackaday"

"lacked"

"lacker"

"lackers"

"lackey"

"lackeyed"

"lackeying"

"lackeys"

"lacking"

"lackluster"

"lacks"

"laconic"

"laconically"

"laconism"

"lacquer"

"lacquered"

"lacquerer"

"lacquerers"

"lacquering"

"lacquers"

"lacrimal"

"lacrimation"

"lacrimatory"

"lacrosse"

"lacrosses"

"lactate"

"lactated"

"lactates"

"lactating"

"lactation"

"lactational"

"lactationally"

"lactations"

"lacteal"

"lacteally"

"lactic"

"lactobacilli"

"lactobacillus"

"lactometer"

"lactoprotein"

"lactoscope"

"lactose"

"lactoses"

"lactovegetarian"

"lacuna"

"lacunae"

"lacunal"

"lacunar"

"lacunary"

"lacunas"

"lacy"

"lad"

"ladanum"

"ladanums"

"ladder"

"laddered"

"laddering"

"ladders"

"laddie"

"laddies"

"lade"

"laded"

"laden"

"ladened"

"ladens"

"lader"

"laders"

"lades"

"ladies"

"lading"

"ladings"

"ladle"

"ladled"

"ladleful"

"ladlefuls"

"ladler"

"ladlers"

"ladles"

"ladling"

"ladron"

"ladrone"

"ladrons"

"lads"

"lady"

"ladybird"

"ladybirds"

"ladybug"

"ladybugs"

"ladyfinger"

"ladyfingers"

"ladyish"

"ladykin"

"ladylike"

"ladylove"

"ladyloves"

"ladyship"

"ladyships"

"laetrile"

"lafayette"

"lag"

"lager"

"lagers"

"laggard"

"laggardly"

"laggardness"

"laggards"

"lagged"

"lagger"

"laggers"

"lagging"

"laggings"

"lagniappe"

"lagniappes"

"lagoon"

"lagoonal"

"lagoons"

"lags"

"laguna"

"lagunas"

"lahore"

"laical"

"laicisms"

"laicized"

"laicizes"

"laicizing"

"laid"

"lain"

"lair"

"laird"

"lairdly"

"lairds"

"laired"

"lairing"

"lairs"

"laissez"

"lait"

"laities"

"laity"

"lake"

"laked"

"lakeport"

"lakeports"

"laker"

"lakers"

"lakes"

"lakeside"

"lakesides"

"lakier"

"lakiest"

"laking"

"lakings"

"laky"

"lallygag"

"lallygagged"

"lallygagging"

"lallygags"

"lam"

"lama"

"lamaism"

"lamas"

"lamaseries"

"lamasery"

"lamb"

"lambast"

"lambaste"

"lambasted"

"lambastes"

"lambasting"

"lambasts"

"lambda"

"lambdas"

"lambed"

"lambencies"

"lambency"

"lambent"

"lambently"

"lamber"

"lambers"

"lambert"

"lambie"

"lambies"

"lambing"

"lambkin"

"lambkins"

"lambs"

"lambskin"

"lambskins"

"lame"

"lamebrain"

"lamebrains"

"lamed"

"lamedhs"

"lameds"

"lamella"

"lamellae"

"lamellas"

"lamely"

"lameness"

"lament"

"lamentable"

"lamentably"

"lamentation"

"lamentations"

"lamented"

"lamenter"

"lamenters"

"lamenting"

"laments"

"lamer"

"lames"

"lamest"

"lamia"

"lamias"

"lamina"

"laminae"

"laminal"

"laminar"

"laminary"

"laminas"

"laminate"

"laminated"

"laminates"

"laminating"

"lamination"

"laminator"

"laming"

"lammed"

"lamming"

"lamp"

"lampblack"

"lamped"

"lampers"

"lamping"

"lamplight"

"lamplighter"

"lampoon"

"lampooned"

"lampooner"

"lampooners"

"lampoonery"

"lampooning"

"lampoonist"

"lampoonists"

"lampoons"

"lamppost"

"lampposts"

"lamprey"

"lampreys"

"lamps"

"lams"

"lanai"

"lanais"

"lanameter"

"lance"

"lanced"

"lancelets"

"lancelot"

"lancer"

"lancers"

"lances"

"lancet"

"lanceted"

"lancets"

"lanciers"

"lancinate"

"lancing"

"land"

"landau"

"landaus"

"landed"

"lander"

"landers"

"landfall"

"landfalls"

"landfill"

"landfills"

"landform"

"landforms"

"landholder"

"landholders"

"landholding"

"landing"

"landings"

"landladies"

"landlady"

"landless"

"landlessness"

"landlocked"

"landlord"

"landlordism"

"landlordly"

"landlords"

"landlordship"

"landlubber"

"landlubbers"

"landmark"

"landmarks"

"landmass"

"landmasses"

"landocracies"

"landowner"

"landowners"

"landownership"

"landowning"

"landright"

"lands"

"landsat"

"landscape"

"landscaped"

"landscaper"

"landscapers"

"landscapes"

"landscaping"

"landsides"

"landskips"

"landslid"

"landslide"

"landslides"

"landslip"

"landslips"

"landsman"

"landsmen"

"landward"

"lane"

"lanes"

"langauge"

"langley"

"langsynes"

"language"

"languages"

"langues"

"languid"

"languidly"

"languidness"

"languish"

"languished"

"languisher"

"languishers"

"languishes"

"languishing"

"languor"

"languorous"

"languorously"

"languorousness"

"languors"

"langur"

"laniard"

"lank"

"lanker"

"lankest"

"lankier"

"lankiest"

"lankily"

"lankiness"

"lankly"

"lankness"

"lanky"

"lanolin"

"lanoline"

"lanolines"

"lanolins"

"lansing"

"lantana"

"lantanas"

"lantern"

"lanterns"

"lanthanum"

"lanthorns"

"lanyard"

"lanyards"

"laos"

"laotian"

"laotians"

"lap"

"lap timer"

"laparorrhaphy"

"laparoscope"

"laparotomies"

"laparotomy"

"lapboard"

"lapboards"

"lapdog"

"lapdogs"

"lapel"

"lapels"

"lapful"

"lapfuls"

"lapidaries"

"lapidary"

"lapidated"

"lapidates"

"lapidating"

"lapidists"

"lapin"

"lapinized"

"lapis"

"lapises"

"lapland"

"laplander"

"laplanders"

"lapp"

"lapped"

"lapper"

"lappering"

"lappers"

"lappet"

"lappets"

"lapping"

"lapps"

"laps"

"lapse"

"lapsed"

"lapser"

"lapsers"

"lapses"

"lapsing"

"lapsus"

"laptop"

"lapwing"

"lapwings"

"lar"

"larboard"

"larboards"

"larcenable"

"larcener"

"larceners"

"larcenies"

"larcenist"

"larcenists"

"larcenous"

"larcenously"

"larceny"

"larch"

"larches"

"lard"

"larded"

"larder"

"larders"

"lardier"

"lardiest"

"larding"

"lards"

"lardy"

"lares"

"large"

"large clock display"

"largehearted"

"largely"

"largeness"

"larger"

"larges"

"largess"

"largesse"

"largesses"

"largest"

"largish"

"largo"

"largos"

"lariat"

"lariated"

"lariating"

"lariats"

"larithmics"

"lark"

"larked"

"larker"

"larkers"

"larkier"

"larking"

"larks"

"larkspur"

"larkspurs"

"larky"

"larrup"

"larruped"

"larruper"

"larrupers"

"larruping"

"larrups"

"larry"

"larums"

"larva"

"larvae"

"larval"

"larvas"

"larvicide"

"laryngal"

"laryngeal"

"laryngectomies"

"laryngectomize"

"laryngectomy"

"larynges"

"laryngitic"

"laryngitis"

"laryngology"

"laryngoscope"

"laryngoscopy"

"laryngotracheal"

"larynx"

"larynxes"

"lasagna"

"lasagnas"

"lasagne"

"lasagnes"

"lascar"

"lascars"

"lascivious"

"lasciviously"

"lasciviousness"

"lased"

"laser"

"laserdisk"

"laserdisks"

"laserjet"

"lasers"

"lases"

"lash"

"lashed"

"lasher"

"lashers"

"lashes"

"lashing"

"lashings"

"lasing"

"lass"

"lasses"

"lassie"

"lassies"

"lassitude"

"lassitudes"

"lasso"

"lassoed"

"lassoer"

"lassoers"

"lassoes"

"lassoing"

"lassos"

"last"

"Last menstrual period"

"lasted"

"laster"

"lasters"

"lasting"

"lastingly"

"lastingness"

"lastings"

"lastly"

"lasts"

"latch"

"latched"

"latches"

"latchets"

"latching"

"latchkey"

"latchkeys"

"latchstring"

"latchstrings"

"late"

"latecomer"

"latecomers"

"lated"

"lateen"

"lateens"

"lately"

"laten"

"latencies"

"latency"

"latened"

"lateness"

"latening"

"latens"

"latent"

"latently"

"latents"

"later"

"lateral"

"lateraled"

"lateralities"

"laterally"

"laterals"

"latest"

"latests"

"latex"

"latexes"

"lath"

"lathe"

"lathed"

"lather"

"lathered"

"latherer"

"latherers"

"lathering"

"lathers"

"lathery"

"lathes"

"lathier"

"lathing"

"lathings"

"laths"

"lathwork"

"lathworks"

"lathy"

"latin"

"latinize"

"latinized"

"latinizes"

"latinizing"

"latino"

"latinos"

"latins"

"latish"

"latissimi"

"latissimus"

"latitude"

"latitudes"

"latitudinal"

"latitudinally"

"latitudinarian"

"latitudinarianism"

"latitudinarians"

"latrine"

"latrines"

"latten"

"latter"

"latterly"

"lattice"

"latticed"

"lattices"

"latticework"

"latticing"

"latvia"

"latvian"

"latvians"

"laud"

"laudability"

"laudable"

"laudably"

"laudanum"

"laudanums"

"laudation"

"laudator"

"laudatorily"

"laudators"

"laudatory"

"laude"

"lauded"

"lauder"

"lauderdale"

"lauders"

"lauding"

"lauds"

"laugh"

"laughable"

"laughably"

"laughed"

"laugher"

"laughers"

"laughing"

"laughingly"

"laughings"

"laughingstock"

"laughingstocks"

"laughs"

"laughter"

"laughters"

"launch"

"launched"

"launcher"

"launchers"

"launches"

"launching"

"launchings"

"launder"

"laundered"

"launderer"

"launderers"

"launderette"

"laundering"

"launders"

"laundress"

"laundresses"

"laundries"

"laundromat"

"laundromats"

"laundry"

"laundryman"

"laundrymen"

"laundrywoman"

"laundrywomen"

"laura"

"lauras"

"laureate"

"laureated"

"laureates"

"laureateship"

"laureateships"

"laureating"

"laurel"

"laureled"

"laureling"

"laurelled"

"laurelling"

"laurels"

"lava"

"lavabo"

"lavaboes"

"lavage"

"lavages"

"lavalava"

"lavalavas"

"lavalier"

"lavaliere"

"lavalieres"

"lavaliers"

"lavas"

"lavation"

"lavations"

"lavatories"

"lavatory"

"lave"

"laved"

"lavender"

"lavendered"

"lavenders"

"laver"

"lavers"

"laves"

"laving"

"lavish"

"lavished"

"lavisher"

"lavishers"

"lavishes"

"lavishest"

"lavishing"

"lavishly"

"lavishness"

"law"

"law texts"

"lawbook"

"lawbreaker"

"lawbreakers"

"lawbreaking"

"lawcourt"

"lawed"

"lawful"

"lawfully"

"lawfulness"

"lawgiver"

"lawgivers"

"lawgiving"

"lawing"

"lawings"

"lawless"

"lawlessly"

"lawlessness"

"lawmaker"

"lawmakers"

"lawmaking"

"lawman"

"lawmen"

"lawn"

"lawnmower"

"lawns"

"lawny"

"lawrence"

"lawrencium"

"laws"

"lawsuit"

"lawsuits"

"lawyer"

"lawyeress"

"lawyeresses"

"lawyering"

"lawyerlike"

"lawyerly"

"lawyers"

"lax"

"laxative"

"laxatives"

"laxer"

"laxest"

"laxities"

"laxity"

"laxly"

"laxness"

"laxnesses"

"lay"

"layabout"

"layabouts"

"layaway"

"layaways"

"layed"

"layer"

"layered"

"layering"

"layerings"

"layers"

"layette"

"layettes"

"laying"

"layman"

"laymen"

"layoff"

"layoffs"

"layout"

"layouts"

"layover"

"layovers"

"lays"

"laywoman"

"laywomen"

"lazar"

"lazaret"

"lazarette"

"lazaretto"

"lazarettos"

"lazars"

"lazarus"

"laze"

"lazed"

"lazes"

"lazied"

"lazier"

"lazies"

"laziest"

"lazily"

"laziness"

"lazing"

"lazuli"

"lazulis"

"lazy"

"lazybones"

"lazying"

"lazyish"

"lbs"

"LCD screen"

"lea"

"leach"

"leached"

"leacher"

"leachers"

"leaches"

"leachier"

"leachiest"

"leaching"

"leachy"

"lead"

"leaded"

"leaden"

"leadenly"

"leader"

"leaderless"

"leaders"

"leadership"

"leadier"

"leading"

"leadings"

"leadoff"

"leadoffs"

"leads"

"leadworks"

"leady"

"leaf"

"leafage"

"leafed"

"leafhopper"

"leafhoppers"

"leafier"

"leafiest"

"leafing"

"leafless"

"leaflet"

"leaflets"

"leafs"

"leafstalk"

"leafstalks"

"leafworm"

"leafworms"

"leafy"

"league"

"leagued"

"leaguer"

"leaguered"

"leaguering"

"leaguers"

"leagues"

"leaguing"

"leak"

"leakage"

"leakages"

"leaked"

"leaker"

"leakers"

"leakier"

"leakiest"

"leakily"

"leakiness"

"leaking"

"leaks"

"leaky"

"leal"

"lean"

"leaned"

"leaner"

"leanest"

"leaning"

"leanings"

"leanly"

"leanness"

"leans"

"leant"

"leap"

"leaped"

"leaper"

"leapers"

"leapfrog"

"leapfrogged"

"leapfrogging"

"leapfrogs"

"leaping"

"leaps"

"leapt"

"lear"

"learn"

"learnable"

"learned"

"learnedness"

"learner"

"learners"

"learning"

"learning modules"

"learning tool"

"learnings"

"learns"

"learnt"

"leary"

"leas"

"leasable"

"lease"

"leaseback"

"leased"

"leasehold"

"leaseholder"

"leaseholders"

"leaseholds"

"leaseless"

"leaser"

"leasers"

"leases"

"leash"

"leashed"

"leashes"

"leashing"

"leasing"

"leasings"

"least"

"leasts"

"leastwise"

"leather"

"leathered"

"leatheriness"

"leathering"

"leathern"

"leatherneck"

"leathernecks"

"leathers"

"leathery"

"leave"

"leaved"

"leaven"

"leavened"

"leavening"

"leavens"

"leaver"

"leavers"

"leaves"

"leaves in the wind"

"leavier"

"leaving"

"leavings"

"lebanese"

"lebanon"

"lech"

"lechayim"

"lecher"

"lechered"

"lecheries"

"lechering"

"lecherous"

"lecherously"

"lecherousness"

"lechers"

"lechery"

"leches"

"lecithin"

"lecithins"

"lect"

"lectern"

"lecterns"

"lections"

"lector"

"lectors"

"lecture"

"lectured"

"lecturer"

"lecturers"

"lectures"

"lectureship"

"lectureships"

"lecturing"

"led"

"ledge"

"ledgeless"

"ledger"

"ledgers"

"ledges"

"ledgier"

"ledgy"

"lee"

"leeboard"

"leeboards"

"leech"

"leeched"

"leeches"

"leeching"

"leeds"

"leek"

"leeks"

"leer"

"leered"

"leerier"

"leeriest"

"leerily"

"leeriness"

"leering"

"leeringly"

"leers"

"leery"

"lees"

"leeward"

"leewardly"

"leewards"

"leeway"

"leeways"

"left"

"lefter"

"leftest"

"lefties"

"leftism"

"leftisms"

"leftist"

"leftists"

"leftover"

"leftovers"

"lefts"

"leftward"

"leftwing"

"lefty"

"leg"

"legacies"

"legacy"

"legal"

"legal code"

"legal document"

"legal information"

"legalese"

"legaleses"

"legalism"

"legalisms"

"legalist"

"legalistic"

"legalistically"

"legalists"

"legalities"

"legality"

"legalization"

"legalizations"

"legalize"

"legalized"

"legalizes"

"legalizing"

"legally"

"legalness"

"legals"

"legate"

"legated"

"legatee"

"legatees"

"legates"

"legateship"

"legateships"

"legation"

"legationary"

"legations"

"legato"

"legator"

"legators"

"legatos"

"legend"

"legendarily"

"legendary"

"legendry"

"legends"

"leger"

"legerdemain"

"legged"

"leggier"

"leggiest"

"legging"

"legginged"

"leggings"

"leggins"

"leggy"

"leghorn"

"leghorns"

"legibilities"

"legibility"

"legible"

"legibleness"

"legibly"

"legion"

"legionaries"

"legionary"

"legionnaire"

"legionnaires"

"legions"

"legislate"

"legislated"

"legislates"

"legislating"

"legislation"

"legislative"

"legislatively"

"legislator"

"legislatorial"

"legislators"

"legislatorship"

"legislatress"

"legislatresses"

"legislatrices"

"legislatrix"

"legislatrixes"

"legislature"

"legislatures"

"legists"

"legit"

"legitimacies"

"legitimacy"

"legitimate"

"legitimated"

"legitimately"

"legitimateness"

"legitimating"

"legitimation"

"legitimatize"

"legitimatized"

"legitimatizing"

"legitimism"

"legitimist"

"legitimization"

"legitimizations"

"legitimize"

"legitimized"

"legitimizer"

"legitimizes"

"legitimizing"

"legits"

"legless"

"legman"

"legmen"

"legroom"

"legrooms"

"legs"

"legume"

"legumes"

"leguminous"

"legumins"

"legwork"

"legworks"

"lehayim"

"lei"

"leipzig"

"leis"

"leister"

"leisure"

"leisure light"

"leisured"

"leisureless"

"leisurely"

"leisures"

"leitmotif"

"leitmotifs"

"lek"

"leks"

"leman"

"lemans"

"lemma"

"lemmas"

"lemming"

"lemmings"

"lemon"

"lemonade"

"lemonades"

"lemonish"

"lemons"

"lemony"

"lempira"

"lempiras"

"lemur"

"lemuroids"

"lemurs"

"lend"

"lender"

"lenders"

"lending"

"lends"

"length"

"lengthen"

"lengthened"

"lengthener"

"lengtheners"

"lengthening"

"lengthens"

"lengthier"

"lengthiest"

"lengthily"

"lengthiness"

"lengths"

"lengthwise"

"lengthy"

"lenience"

"leniencies"

"leniency"

"lenient"

"leniently"

"lenin"

"leningrad"

"leninism"

"leninist"

"leninists"

"lenities"

"lenitive"

"lenity"

"lens"

"lense"

"lensed"

"lenses"

"lent"

"lentando"

"lenten"

"lentic"

"lenticular"

"lentiform"

"lentil"

"lentils"

"lento"

"lentos"

"leo"

"leon"

"leonard"

"leonardo"

"leone"

"leones"

"leonine"

"leopard"

"leopards"

"leos"

"leotard"

"leotards"

"leper"

"lepers"

"lepidoptera"

"lepidopteran"

"lepidopterology"

"leprechaun"

"leprechauns"

"leprology"

"leprosaria"

"leprosarium"

"leprosariums"

"leprose"

"leprosies"

"leprosy"

"leprous"

"leptometer"

"lepton"

"leptonic"

"leptons"

"lesbian"

"lesbianism"

"lesbians"

"lese"

"lesion"

"lesions"

"less"

"lessee"

"lessees"

"lessen"

"lessened"

"lessening"

"lessens"

"lesser"

"lesson"

"lessoned"

"lessoning"

"lessons"

"lessor"

"lessors"

"lest"

"let"

"letch"

"letches"

"letdown"

"letdowns"

"lethal"

"lethalities"

"lethality"

"lethally"

"lethals"

"lethargic"

"lethargically"

"lethargies"

"lethargy"

"lethe"

"lethean"

"lethes"

"lets"

"letted"

"letter"

"lettered"

"letterer"

"letterers"

"letterhead"

"letterheads"

"lettering"

"letterings"

"letterman"

"lettermen"

"letterpress"

"letters"

"letting"

"lettuce"

"lettuces"

"letup"

"letups"

"leu"

"leucocyte"

"leucoma"

"leukaemia"

"leukaemic"

"leukemia"

"leukemias"

"leukemic"

"leukemics"

"leukemoid"

"leukocyte"

"leukocytes"

"leukoma"

"lev"

"leva"

"levant"

"levants"

"levator"

"levators"

"levee"

"leveed"

"leveeing"

"levees"

"level"

"leveled"

"leveler"

"levelers"

"levelheaded"

"levelheadedness"

"leveling"

"levelled"

"leveller"

"levellers"

"levelling"

"levelly"

"levelness"

"levels"

"lever"

"leverage"

"leveraged"

"leverages"

"leveraging"

"levered"

"leveret"

"leverets"

"levering"

"levers"

"levi"

"leviathan"

"leviathans"

"levied"

"levier"

"leviers"

"levies"

"levin"

"levis"

"levitate"

"levitated"

"levitates"

"levitating"

"levitation"

"levitations"

"levitical"

"leviticus"

"levities"

"levity"

"levo"

"levulose"

"levuloses"

"levy"

"levying"

"lewd"

"lewder"

"lewdest"

"lewdly"

"lewdness"

"lewis"

"lewises"

"lex"

"lexical"

"lexically"

"lexicographer"

"lexicographers"

"lexicographic"

"lexicographical"

"lexicographically"

"lexicography"

"lexicology"

"lexicon"

"lexicons"

"lexigraphy"

"ley"

"leys"

"lf"

"lh"

"liabilities"

"liability"

"liable"

"liaise"

"liaised"

"liaises"

"liaising"

"liaison"

"liaisons"

"liana"

"lianas"

"lianes"

"liar"

"liars"

"lib"

"libation"

"libationary"

"libations"

"libbed"

"libber"

"libbers"

"libbing"

"libel"

"libelant"

"libelants"

"libeled"

"libelee"

"libelees"

"libeler"

"libelers"

"libeling"

"libelist"

"libelists"

"libellant"

"libelled"

"libellee"

"libellees"

"libeller"

"libellers"

"libelling"

"libellous"

"libellously"

"libelous"

"libelously"

"libels"

"liber"

"liberal"

"liberalism"

"liberalities"

"liberality"

"liberalization"

"liberalizations"

"liberalize"

"liberalized"

"liberalizes"

"liberalizing"

"liberally"

"liberalness"

"liberals"

"liberate"

"liberated"

"liberates"

"liberating"

"liberation"

"liberationist"

"liberationists"

"liberations"

"liberator"

"liberators"

"liberia"

"liberian"

"liberians"

"libers"

"libertarian"

"libertarianism"

"libertarians"

"liberties"

"libertine"

"libertines"

"liberty"

"libidinal"

"libidinally"

"libidinization"

"libidinized"

"libidinizing"

"libidinous"

"libidinously"

"libido"

"libidos"

"libitum"

"libra"

"librarian"

"librarians"

"libraries"

"library"

"libras"

"librate"

"librated"

"librates"

"librating"

"libre"

"libretti"

"librettist"

"librettists"

"libretto"

"librettos"

"libris"

"libs"

"libya"

"libyans"

"lice"

"licence"

"licencees"

"licencers"

"licencing"

"licensable"

"license"

"licensed"

"licensee"

"licensees"

"licenseless"

"licenser"

"licensers"

"licenses"

"licensing"

"licensor"

"licensors"

"licensure"

"licentiate"

"licentiates"

"licentious"

"licentiously"

"licentiousness"

"lichee"

"lichees"

"lichen"

"lichened"

"lichening"

"lichenins"

"lichenoid"

"lichenology"

"lichenous"

"lichens"

"lichi"

"lichis"

"licht"

"lichting"

"licit"

"licitation"

"licitly"

"lick"

"licked"

"licker"

"lickers"

"lickety"

"licking"

"lickings"

"licks"

"licorice"

"licorices"

"lictor"

"lictors"

"lid"

"lidar"

"lidded"

"lidding"

"lidless"

"lido"

"lidos"

"lids"

"lie"

"liechtenstein"

"lied"

"lieder"

"lief"

"liefer"

"liefest"

"liefly"

"liege"

"liegeman"

"liegemen"

"lieges"

"lien"

"lienable"

"lienal"

"lienee"

"lienholder"

"lienor"

"liens"

"lienteries"

"lier"

"liers"

"lies"

"lieu"

"lieut"

"lieutenancies"

"lieutenancy"

"lieutenant"

"lieutenants"

"life"

"life enhancing"

"life purpose"

"life science"

"lifeblood"

"lifeboat"

"lifeboats"

"lifebuoy"

"lifeful"

"lifeguard"

"lifeguards"

"lifeless"

"lifelessly"

"lifelessness"

"lifelike"

"lifelikeness"

"lifeline"

"lifelines"

"lifelong"

"lifer"

"lifers"

"lifesaver"

"lifesavers"

"lifesaving"

"lifespan"

"lifestyle"

"lifestyles"

"lifetime"

"lifetimes"

"lifeway"

"lifework"

"lifeworks"

"lift"

"liftable"

"lifted"

"lifter"

"lifters"

"lifting"

"liftman"

"liftmen"

"liftoff"

"liftoffs"

"lifts"

"ligament"

"ligamentary"

"ligamentous"

"ligaments"

"ligate"

"ligated"

"ligates"

"ligating"

"ligation"

"ligations"

"ligature"

"ligatured"

"ligatures"

"ligaturing"

"liger"

"light"

"light system"

"lighted"

"lighten"

"lightened"

"lightener"

"lighteners"

"lightening"

"lightens"

"lighter"

"lighterage"

"lightered"

"lightering"

"lighters"

"lightest"

"lightface"

"lightfaced"

"lightfingered"

"lightfooted"

"lightful"

"lightheaded"

"lighthearted"

"lightheartedly"

"lightheartedness"

"lighthouse"

"lighthouses"

"lighting"

"lightings"

"lightish"

"lightly"

"lightmindedness"

"lightness"

"lightning"

"Lightning Bug"

"lightnings"

"lights"

"lightship"

"lightships"

"lightsome"

"lightweight"

"lightweights"

"lightyears"

"ligneous"

"lignification"

"lignifications"

"lignified"

"lignifies"

"lignify"

"lignifying"

"lignin"

"lignins"

"lignite"

"lignites"

"lignitic"

"lignum"

"lignums"

"ligyrophobia"

"likability"

"likable"

"likableness"

"like"

"likeable"

"liked"

"likelier"

"likeliest"

"likelihood"

"likelihoods"

"likely"

"liken"

"likened"

"likeness"

"likenesses"

"likening"

"likens"

"liker"

"likers"

"likes"

"likest"

"likewise"

"liking"

"likings"

"lilac"

"lilacs"

"lilied"

"lilies"

"lilliput"

"lilliputian"

"lilliputians"

"lilliputs"

"lilly"

"lilt"

"lilted"

"lilting"

"lilts"

"lily"

"lim"

"lima"

"limacology"

"limacons"

"limas"

"limb"

"limbeck"

"limbed"

"limber"

"limbered"

"limberer"

"limberest"

"limbering"

"limberly"

"limberness"

"limbers"

"limbic"

"limbier"

"limbing"

"limbless"

"limbo"

"limbos"

"limbs"

"limburger"

"limby"

"lime"

"limeade"

"limeades"

"limed"

"limekiln"

"limekilns"

"limelight"

"limelights"

"limens"

"limerick"

"limericks"

"limes"

"limestone"

"limestones"

"limewater"

"limey"

"limeys"

"limier"

"limiest"

"liminal"

"liminess"

"liming"

"limit"

"limitable"

"limitation"

"limitations"

"limitative"

"limited"

"limited number"

"limitedly"

"limitedness"

"limiteds"

"limiter"

"limiters"

"limiting"

"limitless"

"limitlessly"

"limits"

"limn"

"limned"

"limner"

"limners"

"limning"

"limnobiology"

"limnology"

"limns"

"limo"

"limonite"

"limonitic"

"limos"

"limousine"

"limousines"

"limp"

"limped"

"limper"

"limpers"

"limpest"

"limpet"

"limpets"

"limpid"

"limpidity"

"limpidly"

"limpidness"

"limping"

"limply"

"limpness"

"limps"

"limy"

"linable"

"linac"

"linacs"

"linage"

"linages"

"linchpin"

"linchpins"

"lincoln"

"linda"

"lindane"

"lindanes"

"linden"

"lindens"

"lindies"

"lindy"

"line"

"lineable"

"lineage"

"lineages"

"lineal"

"lineally"

"lineament"

"lineaments"

"linear"

"linearly"

"lineate"

"linebacker"

"linebackers"

"linecut"

"lined"

"linefeed"

"lineless"

"lineman"

"linemen"

"linen"

"linens"

"lineny"

"liner"

"liners"

"lines"

"linesman"

"linesmen"

"lineup"

"lineups"

"liney"

"ling"

"lingam"

"lingams"

"lingas"

"linger"

"lingered"

"lingerer"

"lingerers"

"lingerie"

"lingeries"

"lingering"

"lingeringly"

"lingers"

"lingier"

"lingo"

"lingoes"

"lings"

"lingua"

"lingual"

"lingually"

"linguals"

"linguine"

"linguines"

"linguini"

"linguinis"

"linguist"

"linguistic"

"linguistically"

"linguistics"

"linguists"

"lingula"

"linier"

"liniest"

"liniment"

"liniments"

"lining"

"linings"

"link"

"linkable"

"linkage"

"linkages"

"linkboy"

"linked"

"linker"

"linkers"

"linking"

"linkman"

"linkmen"

"links"

"linkup"

"linkups"

"linky"

"linnet"

"linnets"

"lino"

"linoleum"

"linoleums"

"linonophobia"

"linos"

"linotype"

"linotypes"

"lins"

"linseed"

"linseeds"

"linsey"

"linseys"

"lint"

"lintel"

"lintels"

"linter"

"linters"

"lintier"

"lintiest"

"lints"

"linty"

"linum"

"liny"

"lion"

"lioness"

"lionesses"

"lionhearted"

"lionise"

"lionization"

"lionize"

"lionized"

"lionizer"

"lionizers"

"lionizes"

"lionizing"

"lions"

"lip"

"lipase"

"lipectomies"

"lipid"

"lipids"

"lipless"

"lipolyses"

"lipoprotein"

"liposoluble"

"lipped"

"lipper"

"lippers"

"lippier"

"lippiest"

"lippiness"

"lipping"

"lippy"

"lipreading"

"lips"

"lipstick"

"lipsticks"

"liq"

"liquate"

"liquefacient"

"liquefaction"

"liquefactions"

"liquefactive"

"liquefiable"

"liquefied"

"liquefier"

"liquefiers"

"liquefies"

"liquefy"

"liquefying"

"liquescent"

"liqueur"

"liqueurs"

"liquid"

"liquidate"

"liquidated"

"liquidates"

"liquidating"

"liquidation"

"liquidations"

"liquidator"

"liquidators"

"liquidities"

"liquidity"

"liquidize"

"liquidized"

"liquidizes"

"liquidizing"

"liquidly"

"liquids"

"liquify"

"liquor"

"liquored"

"liquorice"

"liquoring"

"liquors"

"lira"

"liras"

"lire"

"lisbon"

"lisle"

"lisles"

"lisp"

"lisped"

"lisper"

"lispers"

"lisping"

"lispingly"

"lisps"

"lissom"

"lissome"

"lissomely"

"lissomeness"

"lissomly"

"list"

"listable"

"listed"

"listen"

"listened"

"listener"

"listeners"

"listening"

"listenings"

"listens"

"lister"

"listers"

"listing"

"listings"

"listless"

"listlessly"

"listlessness"

"lists"

"liszt"

"lit"

"litanies"

"litany"

"litchi"

"litchis"

"lite"

"Lite version"

"liter"

"literacies"

"literacy"

"literal"

"literalism"

"literally"

"literalness"

"literals"

"literariness"

"literary"

"literate"

"literately"

"literates"

"literati"

"literatim"

"literature"

"literatures"

"liters"

"lites"

"lith"

"lithe"

"lithely"

"litheness"

"lither"

"lithesome"

"lithest"

"lithias"

"lithic"

"lithium"

"lithiums"

"litho"

"lithograph"

"lithographed"

"lithographer"

"lithographers"

"lithographic"

"lithographically"

"lithographing"

"lithographs"

"lithography"

"lithologic"

"lithology"

"lithos"

"lithosphere"

"lithotome"

"lithotomy"

"lithuania"

"lithuanian"

"lithuanians"

"litigable"

"litigant"

"litigants"

"litigate"

"litigated"

"litigates"

"litigating"

"litigation"

"litigations"

"litigator"

"litigators"

"litigiosity"

"litigious"

"litigiously"

"litigiousness"

"litmus"

"litmuses"

"litoral"

"litre"

"litres"

"lits"

"litten"

"litter"

"litterateur"

"litterateurs"

"litterbug"

"litterbugs"

"littered"

"litterer"

"litterers"

"littering"

"litters"

"littery"

"little"

"littleneck"

"littlenecks"

"littleness"

"littler"

"littles"

"littlest"

"littlish"

"littoral"

"littorals"

"liturgic"

"liturgical"

"liturgically"

"liturgies"

"liturgiology"

"liturgist"

"liturgists"

"liturgy"

"livability"

"livable"

"live"

"live joyfully"

"liveability"

"liveable"

"lived"

"livelier"

"liveliest"

"livelihood"

"livelihoods"

"livelily"

"liveliness"

"livelong"

"lively"

"liven"

"livened"

"livener"

"liveners"

"liveness"

"livening"

"livens"

"liver"

"livered"

"liveried"

"liveries"

"liverish"

"liverishness"

"liverpool"

"livers"

"liverwort"

"liverworts"

"liverwurst"

"liverwursts"

"livery"

"liveryman"

"liverymen"

"lives"

"livest"

"livestock"

"livetrap"

"livetraps"

"livid"

"lividities"

"lividity"

"lividly"

"lividness"

"liviers"

"living"

"livingly"

"livings"

"livlihood"

"livre"

"livres"

"lizard"

"lizards"

"ll"

"llama"

"llamas"

"llano"

"llanos"

"LMP"

"lo"

"loach"

"loaches"

"load"

"loadable"

"loaded"

"loader"

"loaders"

"loading"

"loadings"

"loads"

"loadstar"

"loadstone"

"loadstones"

"loaf"

"loafed"

"loafer"

"loafers"

"loafing"

"loafs"

"loam"

"loamed"

"loamier"

"loamiest"

"loaming"

"loams"

"loamy"

"loan"

"loanable"

"loaned"

"loaner"

"loaners"

"loaning"

"loanings"

"loans"

"loanshark"

"loansharking"

"loanword"

"loanwords"

"loath"

"loathe"

"loathed"

"loather"

"loathers"

"loathes"

"loathful"

"loathing"

"loathings"

"loathly"

"loathness"

"loathsome"

"loathsomely"

"loathsomeness"

"loaves"

"lob"

"lobar"

"lobbed"

"lobber"

"lobbers"

"lobbied"

"lobbies"

"lobbing"

"lobby"

"lobbyer"

"lobbyers"

"lobbying"

"lobbyism"

"lobbyisms"

"lobbyist"

"lobbyists"

"lobe"

"lobed"

"lobefin"

"lobelia"

"lobelias"

"lobes"

"loblollies"

"loblolly"

"lobo"

"lobos"

"lobotomies"

"lobotomize"

"lobotomized"

"lobotomizing"

"lobotomy"

"lobs"

"lobster"

"lobsters"

"lobular"

"lobule"

"lobules"

"loc"

"local"

"locale"

"locales"

"localising"

"localism"

"localisms"

"localist"

"localists"

"localite"

"localites"

"localities"

"locality"

"localization"

"localizations"

"localize"

"localized"

"localizer"

"localizes"

"localizing"

"locally"

"locals"

"locate"

"located"

"locater"

"locaters"

"locates"

"locating"

"location"

"Location course"

"locations"

"locative"

"locatives"

"locator"

"locators"

"loch"

"lochs"

"loci"

"lock"

"lockable"

"lockage"

"lockages"

"lockbox"

"lockboxes"

"locked"

"locker"

"lockers"

"locket"

"lockets"

"locking"

"lockjaw"

"lockjaws"

"lockless"

"locknut"

"locknuts"

"lockout"

"lockouts"

"locks"

"locksmith"

"locksmiths"

"lockstep"

"locksteps"

"lockup"

"lockups"

"loco"

"locoed"

"locoes"

"locoing"

"locoism"

"locoisms"

"locomote"

"locomoted"

"locomotes"

"locomoting"

"locomotion"

"locomotive"

"locomotives"

"locomotor"

"locos"

"locoweed"

"locoweeds"

"locus"

"locust"

"locusts"

"locution"

"locutions"

"locutory"

"lode"

"loden"

"lodes"

"lodestar"

"lodestars"

"lodestone"

"lodge"

"lodgeable"

"lodged"

"lodgement"

"lodgements"

"lodger"

"lodgers"

"lodges"

"lodging"

"lodgings"

"lodgment"

"lodgments"

"lodicules"

"loess"

"loesses"

"loessial"

"loft"

"lofted"

"lofter"

"lofters"

"loftier"

"loftiest"

"loftily"

"loftiness"

"lofting"

"loftless"

"lofts"

"lofty"

"log"

"log book"

"logan"

"loganberries"

"loganberry"

"logans"

"logarithm"

"logarithmic"

"logarithmical"

"logarithms"

"logbook"

"logbooks"

"loge"

"loges"

"logged"

"logger"

"loggerhead"

"loggerheads"

"loggers"

"loggia"

"loggias"

"loggie"

"loggier"

"logging"

"loggings"

"loggy"

"logia"

"logic"

"logical"

"logically"

"logician"

"logicians"

"logicize"

"logicized"

"logicizes"

"logicizing"

"logics"

"logier"

"logiest"

"logily"

"login"

"login areas"

"loginess"

"logistic"

"logistical"

"logistically"

"logistician"

"logisticians"

"logistics"

"logjam"

"logjams"

"logo"

"logogram"

"logomania"

"logorrhea"

"logos"

"logotype"

"logotypes"

"logotypies"

"logroll"

"logrolled"

"logrolling"

"logrolls"

"logs"

"logway"

"logways"

"logwood"

"logwoods"

"logy"

"loimology"

"loin"

"loincloth"

"loincloths"

"loins"

"loiter"

"loitered"

"loiterer"

"loiterers"

"loitering"

"loiteringly"

"loiters"

"loll"

"lolled"

"loller"

"lollers"

"lollies"

"lolling"

"lollipop"

"lollipops"

"lollop"

"lolloped"

"lolloping"

"lollops"

"lolls"

"lolly"

"lollygag"

"lollygags"

"lollypop"

"lollypops"

"london"

"londoner"

"londoners"

"lone"

"lonelier"

"loneliest"

"lonelily"

"loneliness"

"lonely"

"loneness"

"loner"

"loners"

"lonesome"

"lonesomely"

"lonesomeness"

"lonesomes"

"long"

"longboat"

"longboats"

"longbow"

"longbows"

"longed"

"longer"

"longers"

"longes"

"longest"

"longevities"

"longevity"

"longhair"

"longhaired"

"longhairs"

"longhand"

"longhorn"

"longhorns"

"longing"

"longingly"

"longings"

"longish"

"longitude"

"longitudes"

"longitudinal"

"longitudinally"

"longline"

"longlines"

"longly"

"longness"

"longrun"

"longs"

"longship"

"longships"

"longshoreman"

"longshoremen"

"longshot"

"longstanding"

"longsuffering"

"longterm"

"longtime"

"longue"

"longues"

"longways"

"longwise"

"loo"

"loofa"

"loofah"

"loofahs"

"loofas"

"loofs"

"look"

"look for"

"looked"

"looker"

"lookers"

"looking"

"lookout"

"lookouts"

"looks"

"lookup"

"lookups"

"loom"

"loomed"

"looming"

"looms"

"loon"

"looney"

"loonier"

"loonies"

"looniest"

"looniness"

"loons"

"loony"

"loop"

"looped"

"looper"

"loopers"

"loophole"

"loopholes"

"loopholing"

"loopier"

"looping"

"loops"

"loopy"

"loos"

"loose"

"loosed"

"loosely"

"loosen"

"loosened"

"loosener"

"looseners"

"looseness"

"loosening"

"loosens"

"looser"

"looses"

"loosest"

"loosing"

"loot"

"looted"

"looter"

"looters"

"looting"

"loots"

"lop"

"lope"

"loped"

"loper"

"lopers"

"lopes"

"loping"

"lopped"

"lopper"

"loppers"

"loppier"

"lopping"

"loppy"

"lops"

"lopsided"

"lopsidedly"

"lopsidedness"

"loquacious"

"loquaciously"

"loquaciousness"

"loquacity"

"loquat"

"loquats"

"loran"

"lorans"

"lord"

"lorded"

"lording"

"lordings"

"lordlier"

"lordliest"

"lordliness"

"lordling"

"lordlings"

"lordly"

"lords"

"lordship"

"lordships"

"lore"

"lores"

"lorgnette"

"lorgnettes"

"lories"

"loris"

"lorises"

"lorn"

"lornness"

"lorries"

"lorry"

"lory"

"losable"

"lose"

"lose weight"

"loser"

"losers"

"loses"

"losing"

"losingly"

"losings"

"loss"

"losses"

"lossy"

"lost"

"lostness"

"lot"

"loth"

"lothario"

"lotharios"

"lothsome"

"lotion"

"lotions"

"lotos"

"lots"

"lotted"

"lotteries"

"lottery"

"lotting"

"lotto"

"lottos"

"lotus"

"lotuses"

"loud"

"louden"

"loudened"

"loudening"

"loudens"

"louder"

"loudest"

"loudish"

"loudlier"

"loudliest"

"loudly"

"loudmouth"

"loudmouthed"

"loudmouths"

"loudness"

"loudspeaker"

"loudspeakers"

"lough"

"louie"

"louies"

"louis"

"louise"

"louisiana"

"louisianan"

"louisianans"

"louisianian"

"louisianians"

"louisville"

"lounge"

"lounged"

"lounger"

"loungers"

"lounges"

"lounging"

"loungy"

"loup"

"loupe"

"louped"

"loupes"

"louping"

"loups"

"lour"

"lours"

"loury"

"louse"

"loused"

"louses"

"lousier"

"lousiest"

"lousily"

"lousiness"

"lousing"

"lousy"

"lout"

"louted"

"louting"

"loutish"

"loutishly"

"loutishness"

"louts"

"louver"

"louvered"

"louvers"

"louvre"

"louvres"

"lovable"

"lovableness"

"lovably"

"lovage"

"lovages"

"love"

"loveable"

"loveably"

"lovebird"

"lovebirds"

"loved"

"loveless"

"lovelessly"

"lovelessness"

"lovelier"

"lovelies"

"loveliest"

"lovelily"

"loveliness"

"lovelorn"

"lovely"

"lovemaking"

"lover"

"loverly"

"lovers"

"loves"

"lovesick"

"lovesickness"

"lovevines"

"loving"

"lovingly"

"low"

"Low Maturity"

"lowborn"

"lowboy"

"lowboys"

"lowbred"

"lowbrow"

"lowbrows"

"lowdown"

"lowdowns"

"lowed"

"lower"

"lowercase"

"lowerclassman"

"lowered"

"lowering"

"loweringly"

"lowermost"

"lowers"

"lowery"

"lowest"

"lowing"

"lowings"

"lowish"

"lowland"

"lowlander"

"lowlands"

"lowlier"

"lowliest"

"lowlife"

"lowlifes"

"lowliness"

"lowly"

"lowness"

"lownesses"

"lows"

"lox"

"loxes"

"loxing"

"loxodograph"

"loxodromy"

"loyal"

"loyaler"

"loyalest"

"loyalism"

"loyalisms"

"loyalist"

"loyalists"

"loyally"

"loyalness"

"loyalties"

"loyalty"

"lozenge"

"lozenges"

"lpm"

"lr"

"luau"

"luaus"

"lubber"

"lubberly"

"lubbers"

"lube"

"lubes"

"lubricant"

"lubricants"

"lubricate"

"lubricated"

"lubricates"

"lubricating"

"lubrication"

"lubrications"

"lubricator"

"lubricators"

"lubricity"

"lubricous"

"lucence"

"lucencies"

"lucency"

"lucent"

"lucently"

"lucern"

"lucerne"

"luces"

"lucia"

"lucid"

"lucid dreaming"

"lucidities"

"lucidity"

"lucidly"

"lucidness"

"lucifer"

"lucifers"

"lucille"

"lucimeter"

"lucite"

"luck"

"lucked"

"luckie"

"luckier"

"luckies"

"luckiest"

"luckily"

"luckiness"

"lucking"

"luckless"

"lucks"

"lucky"

"lucrative"

"lucratively"

"lucrativeness"

"lucre"

"lucres"

"lucubrate"

"lucubrated"

"lucubrates"

"lucubrating"

"lucubration"

"lucubrations"

"lucy"

"ludicrous"

"ludicrously"

"ludicrousness"

"ludwig"

"luff"

"luffed"

"luffing"

"luffs"

"lug"

"luge"

"luges"

"luggage"

"luggages"

"lugged"

"lugger"

"luggers"

"luggies"

"lugging"

"lugs"

"lugubrious"

"lugubriously"

"lugubriousness"

"luke"

"lukewarm"

"lukewarmly"

"lukewarmness"

"lull"

"lullabied"

"lullabies"

"lullaby"

"lullabying"

"lulled"

"lulling"

"lullingly"

"lulls"

"lulu"

"lulus"

"lumbago"

"lumbagos"

"lumbar"

"lumbars"

"lumber"

"lumbered"

"lumberer"

"lumberers"

"lumbering"

"lumberjack"

"lumberjacks"

"lumberman"

"lumbermen"

"lumbers"

"lumberyard"

"lumberyards"

"lumen"

"lumens"

"lumina"

"luminal"

"luminance"

"luminaries"

"luminary"

"luminesce"

"luminesced"

"luminescence"

"luminescent"

"luminesces"

"luminescing"

"luminiferous"

"luminists"

"luminosity"

"luminous"

"luminously"

"lummox"

"lummoxes"

"lump"

"lumped"

"lumpen"

"lumpens"

"lumper"

"lumpers"

"lumpfish"

"lumpier"

"lumpiest"

"lumpily"

"lumpiness"

"lumping"

"lumpish"

"lumps"

"lumpy"

"luna"

"lunacies"

"lunacy"

"lunar"

"lunaria"

"lunarian"

"lunarians"

"lunars"

"lunas"

"lunate"

"lunatic"

"lunatically"

"lunatics"

"lunation"

"lunations"

"lunch"

"lunched"

"luncheon"

"luncheonette"

"luncheonettes"

"luncheons"

"luncher"

"lunchers"

"lunches"

"lunching"

"lunchroom"

"lunchrooms"

"lunchtime"

"lune"

"lunes"

"lunet"

"lunets"

"lunette"

"lunettes"

"lung"

"Lung Cancer"

"lung sound"

"lunge"

"lunged"

"lungee"

"lunger"

"lungers"

"lunges"

"lungfish"

"lungfishes"

"lunging"

"lungs"

"lunier"

"lunies"

"luniest"

"lunk"

"lunker"

"lunkers"

"lunkhead"

"lunkheads"

"lunks"

"luny"

"lupin"

"lupine"

"lupines"

"lupins"

"lupus"

"lupuses"

"lurch"

"lurched"

"lurcher"

"lurchers"

"lurches"

"lurching"

"lure"

"lured"

"lurer"

"lurers"

"lures"

"lurid"

"luridly"

"luridness"

"luring"

"luringly"

"lurk"

"lurked"

"lurker"

"lurkers"

"lurking"

"lurks"

"luscious"

"lusciously"

"lusciousness"

"lush"

"lushed"

"lusher"

"lushes"

"lushest"

"lushing"

"lushly"

"lushness"

"lust"

"lusted"

"luster"

"lustered"

"lustering"

"lusterless"

"lusters"

"lustful"

"lustfully"

"lustfulness"

"lustier"

"lustiest"

"lustily"

"lustiness"

"lusting"

"lustral"

"lustre"

"lustred"

"lustres"

"lustring"

"lustrous"

"lustrum"

"lusts"

"lusty"

"lutanist"

"lutanists"

"lute"

"luteal"

"luteal phase"

"luted"

"lutenist"

"lutenists"

"lutes"

"lutetium"

"luteum"

"luther"

"lutheran"

"lutheranism"

"lutherans"

"luting"

"lutings"

"lutist"

"lutists"

"lux"

"luxations"

"luxe"

"luxembourg"

"luxes"

"luxmeter"

"luxuriance"

"luxuriant"

"luxuriantly"

"luxuriate"

"luxuriated"

"luxuriates"

"luxuriating"

"luxuriation"

"luxuries"

"luxurious"

"luxuriously"

"luxuriousness"

"luxury"

"lycanthrope"

"lycanthropies"

"lycanthropy"

"lycee"

"lycees"

"lyceum"

"lyceums"

"lychee"

"lychees"

"lye"

"lyes"

"lygophobia"

"lying"

"lyingly"

"lyings"

"lymph"

"lymphatic"

"Lymphatic System"

"lymphatically"

"lymphocyte"

"lymphocytes"

"lymphocytic"

"lymphoid"

"lymphomas"

"lymphosarcoma"

"lymphosarcomas"

"lymphs"

"lympic"

"lympics"

"lynch"

"lynched"

"lyncher"

"lynchers"

"lynches"

"lynching"

"lynchings"

"lynx"

"lynxes"

"lyonnaise"

"lypemania"

"lyrate"

"lyrated"

"lyrately"

"lyre"

"lyrebird"

"lyrebirds"

"lyres"

"lyric"

"lyrical"

"lyrically"

"lyricism"

"lyricisms"

"lyricist"

"lyricists"

"lyricize"

"lyricized"

"lyricizes"

"lyricizing"

"lyrics"

"lyriform"

"lyrism"

"lyrisms"

"lyrist"

"lyrists"

"lysed"

"lysergic"

"lyses"

"lysimeter"

"lysin"

"lysine"

"lysing"

"lysins"

"lyssophobia"

"ma"

"mac"

"macabre"

"macadam"

"macadamize"

"macadamized"

"macadamizes"

"macadamizing"

"macadams"

"macaque"

"macaques"

"macaroni"

"macaronies"

"macaronis"

"macaroon"

"macaroons"

"macaw"

"macaws"

"mace"

"maced"

"macedonia"

"macedonian"

"macedonians"

"macer"

"macerate"

"macerated"

"macerater"

"maceraters"

"macerates"

"macerating"

"maceration"

"macerator"

"macerators"

"macers"

"maces"

"mach"

"machete"

"machetes"

"machiavellian"

"machiavellianism"

"machiavellians"

"machicolation"

"machicolations"

"machina"

"machinability"

"machinable"

"machinate"

"machinated"

"machination"

"machinations"

"machinator"

"machine"

"machineable"

"machined"

"machinelike"

"machineries"

"machinery"

"machines"

"machining"

"machinist"

"machinists"

"machinize"

"machinized"

"machinizing"

"machismo"

"machismos"

"macho"

"machos"

"machree"

"machrees"

"machs"

"macing"

"macintosh"

"macintoshes"

"mack"

"mackerel"

"mackerels"

"mackinaw"

"mackinaws"

"mackintosh"

"mackintoshes"

"macks"

"macle"

"macrame"

"macrames"

"macro"

"macrobiotic"

"macrobiotics"

"macrocephalic"

"macrocephalous"

"macrocephaly"

"macrocosm"

"macrocosmic"

"macrocosms"

"macrocyte"

"macroeconomic"

"macroeconomics"

"macromania"

"macromolecule"

"macron"

"macrons"

"macrophobia"

"macros"

"macroscopic"

"macroscopical"

"macroscopically"

"macrostructural"

"macrostructure"

"macs"

"macula"

"macular"

"maculas"

"maculate"

"maculated"

"maculates"

"maculating"

"maculation"

"maculations"

"mad"

"madagascar"

"madam"

"madame"

"madames"

"madams"

"madcap"

"madcaply"

"madcaps"

"madded"

"madden"

"maddened"

"maddening"

"maddeningly"

"maddens"

"madder"

"madders"

"maddest"

"madding"

"maddish"

"made"

"made up"

"madeira"

"madeiras"

"mademoiselle"

"mademoiselles"

"madhouse"

"madhouses"

"madison"

"madly"

"madman"

"madmen"

"madness"

"madnesses"

"madonna"

"madonnas"

"madras"

"madrases"

"madre"

"madres"

"madrid"

"madrigal"

"madrigals"

"madrone"

"madrones"

"mads"

"madwoman"

"madwomen"

"madwort"

"maelstrom"

"maelstroms"

"maenad"

"maenades"

"maenadic"

"maenadism"

"maenads"

"maestoso"

"maestosos"

"maestri"

"maestro"

"maestros"

"maffia"

"maffias"

"mafia"

"mafias"

"mafiosi"

"mafioso"

"mag"

"magazine"

"magazines"

"magdalen"

"magdalene"

"magdalenes"

"magdalens"

"mage"

"magellan"

"magenta"

"magentas"

"mages"

"maggie"

"maggot"

"maggots"

"maggoty"

"magi"

"magic"

"magical"

"magically"

"magician"

"magicians"

"magicked"

"magicking"

"magics"

"magirics"

"magister"

"magisterial"

"magisterially"

"magisterialness"

"magisters"

"magistery"

"magistracies"

"magistracy"

"magistral"

"magistrate"

"magistrates"

"magistrateship"

"magistrature"

"magma"

"magmas"

"magmatic"

"magnanerie"

"magnanimity"

"magnanimous"

"magnanimously"

"magnanimousness"

"magnate"

"magnates"

"magnateship"

"magnesia"

"magnesian"

"magnesias"

"magnesic"

"magnesium"

"magnet"

"magnetic"

"magnetic field"

"magnetically"

"magnetics"

"magnetism"

"magnetisms"

"magnetite"

"magnetizable"

"magnetization"

"magnetize"

"magnetized"

"magnetizer"

"magnetizers"

"magnetizes"

"magnetizing"

"magneto"

"magnetograph"

"magnetometer"

"magnetometers"

"magneton"

"magnetons"

"magnetos"

"magnets"

"magnific"

"magnification"

"magnifications"

"magnificence"

"magnificent"

"magnificently"

"magnifico"

"magnificoes"

"magnified"

"magnifier"

"magnifiers"

"magnifies"

"magnify"

"magnifying"

"magniloquence"

"magniloquent"

"magnitude"

"magnitudes"

"magnolia"

"magnolias"

"magnum"

"magnums"

"magpie"

"magpies"

"mags"

"maguey"

"magueys"

"magus"

"magyar"

"magyars"

"maharaja"

"maharajah"

"maharajahs"

"maharajas"

"maharanee"

"maharanees"

"maharani"

"maharanis"

"maharishi"

"maharishis"

"mahatma"

"mahatmas"

"mahjong"

"mahjongg"

"mahjonggs"

"mahjongs"

"mahoganies"

"mahogany"

"mahomet"

"mahonia"

"mahonias"

"mahout"

"mahouts"

"maid"

"maiden"

"maidenhair"

"maidenhairs"

"maidenhead"

"maidenheads"

"maidenhood"

"maidenliness"

"maidenly"

"maidens"

"maidhood"

"maidhoods"

"maidish"

"maids"

"maidservant"

"maidservants"

"mail"

"mailability"

"mailable"

"mailbag"

"mailbags"

"mailbox"

"mailboxes"

"mailed"

"mailer"

"mailers"

"mailing"

"mailings"

"maillot"

"maillots"

"mailman"

"mailmen"

"mails"

"mailwoman"

"mailwomen"

"maim"

"maimed"

"maimedness"

"maimer"

"maimers"

"maiming"

"maims"

"main"

"maine"

"mainframe"

"mainframes"

"mainland"

"mainlander"

"mainlanders"

"mainlands"

"mainline"

"mainlined"

"mainliner"

"mainliners"

"mainlines"

"mainlining"

"mainly"

"mainmast"

"mainmasts"

"mains"

"mainsail"

"mainsails"

"mainspring"

"mainsprings"

"mainstay"

"mainstays"

"mainstream"

"mainstreams"

"maintain"

"maintainability"

"maintainable"

"maintained"

"maintainer"

"maintainers"

"maintaining"

"maintains"

"maintenance"

"maintenances"

"maintop"

"maintops"

"maisonette"

"maisonettes"

"maist"

"maitre"

"maitres"

"maize"

"maizes"

"majestic"

"majestical"

"majestically"

"majesties"

"majesty"

"majolica"

"major"

"majora"

"majored"

"majorem"

"majorette"

"majorettes"

"majoring"

"majorities"

"majority"

"majors"

"majuscule"

"majuscules"

"makable"

"make"

"make proper decisions"

"makeable"

"maker"

"makers"

"makes"

"makeshift"

"makeshifts"

"makeup"

"makeups"

"makeweight"

"makework"

"making"

"makings"

"mal"

"mala"

"malachite"

"malacology"

"maladaptation"

"maladapted"

"maladies"

"maladjusted"

"maladjustive"

"maladjustment"

"maladjustments"

"maladminister"

"maladministered"

"maladministering"

"maladministers"

"maladministration"

"maladministrative"

"maladroit"

"maladroitly"

"maladroitness"

"malady"

"malagasy"

"malaise"

"malaises"

"malamute"

"malamutes"

"malapert"

"malapertly"

"malapertness"

"malaprop"

"malapropism"

"malapropisms"

"malapropos"

"malaprops"

"malaria"

"malarial"

"malarian"

"malarias"

"malariology"

"malarious"

"malarkey"

"malarkeys"

"malarky"

"malathion"

"malawi"

"malawians"

"malay"

"malaya"

"malayalam"

"malayan"

"malayans"

"malays"

"malaysia"

"malaysian"

"malaysians"

"malconduct"

"malconstruction"

"malcontent"

"malcontents"

"male"

"male body"

"maledict"

"maledicted"

"malediction"

"maledictions"

"maledictive"

"maledictory"

"maledicts"

"malefaction"

"malefactions"

"malefactor"

"malefactors"

"malefactress"

"malefactresses"

"malefic"

"maleficence"

"maleficent"

"maleficently"

"maleficio"

"malemutes"

"maleness"

"males"

"malevolence"

"malevolent"

"malevolently"

"malfeasance"

"malfeasant"

"malfeasantly"

"malfeasants"

"malformation"

"malformations"

"malformed"

"malfunction"

"malfunctioned"

"malfunctioning"

"malfunctions"

"mali"

"malice"

"malices"

"malicious"

"maliciously"

"maliciousness"

"malign"

"malignance"

"malignancies"

"malignancy"

"malignant"

"malignantly"

"maligned"

"maligner"

"maligners"

"maligning"

"malignities"

"malignity"

"malignly"

"maligns"

"maline"

"malines"

"malinger"

"malingered"

"malingerer"

"malingerers"

"malingering"

"malingers"

"malinvestment"

"mall"

"mallard"

"mallards"

"malleability"

"malleable"

"malleableness"

"malleably"

"malled"

"mallei"

"mallet"

"mallets"

"malleus"

"mallow"

"mallows"

"malls"

"malnourished"

"malnourishment"

"malnutrition"

"malocclusion"

"malocclusions"

"malodor"

"malodorous"

"malodorously"

"malodorousness"

"malodors"

"malpractice"

"malpracticed"

"malpracticing"

"malpractitioner"

"malpresentation"

"malt"

"malta"

"maltase"

"malted"

"malteds"

"maltese"

"malthus"

"malthusian"

"malthusianism"

"maltier"

"malting"

"maltose"

"maltreat"

"maltreated"

"maltreating"

"maltreatment"

"maltreatments"

"maltreats"

"malts"

"malty"

"mama"

"mamas"

"mamba"

"mambas"

"mambo"

"mamboed"

"mamboes"

"mamboing"

"mambos"

"mamelukes"

"mameyes"

"mameys"

"mamie"

"mamies"

"mamma"

"mammae"

"mammal"

"mammalia"

"mammalian"

"mammalians"

"mammalogy"

"mammals"

"mammary"

"mammas"

"mammate"

"mammee"

"mammey"

"mammeys"

"mammie"

"mammies"

"mammiform"

"mammogram"

"mammographic"

"mammographies"

"mammography"

"mammon"

"mammons"

"mammoth"

"mammoths"

"mammotomy"

"mammy"

"man"

"manacle"

"manacled"

"manacles"

"manacling"

"manage"

"manageability"

"manageable"

"manageableness"

"manageably"

"managed"

"management"

"managemental"

"managements"

"manager"

"manageress"

"managerial"

"managerially"

"managers"

"managership"

"manages"

"managing"

"manana"

"mananas"

"manas"

"manatee"

"manatees"

"manÃ¨ge"

"manchester"

"manchu"

"manchuria"

"manchurian"

"manchurians"

"manchus"

"mandala"

"mandalas"

"mandalic"

"mandamus"

"mandamuses"

"mandarin"

"mandarins"

"mandate"

"mandated"

"mandatee"

"mandates"

"mandating"

"mandator"

"mandatorily"

"mandators"

"mandatory"

"mandible"

"mandibles"

"mandibular"

"mandolin"

"mandolinist"

"mandolinists"

"mandolins"

"mandragora"

"mandrake"

"mandrakes"

"mandrel"

"mandrels"

"mandril"

"mandrill"

"mandrills"

"mane"

"maned"

"manege"

"manes"

"maneuver"

"maneuverability"

"maneuverable"

"maneuvered"

"maneuverer"

"maneuvering"

"maneuvers"

"manful"

"manfully"

"manfulness"

"manganese"

"manganesian"

"manganous"

"mange"

"mangels"

"manger"

"mangers"

"manges"

"mangey"

"mangier"

"mangiest"

"mangily"

"manginess"

"mangle"

"mangled"

"mangler"

"manglers"

"mangles"

"mangling"

"mango"

"mangoes"

"mangos"

"mangrove"

"mangroves"

"mangy"

"manhandle"

"manhandled"

"manhandles"

"manhandling"

"manhattan"

"manhattans"

"manhole"

"manholes"

"manhood"

"manhoods"

"manhours"

"manhunt"

"manhunts"

"mania"

"maniac"

"maniacal"

"maniacally"

"maniacs"

"manias"

"manic"

"manically"

"manics"

"manicure"

"manicured"

"manicures"

"manicuring"

"manicurist"

"manicurists"

"manifest"

"manifestable"

"manifestation"

"manifestations"

"manifestative"

"manifested"

"manifesting"

"manifestly"

"manifesto"

"manifestoed"

"manifestoes"

"manifestos"

"manifests"

"manifold"

"manifolded"

"manifolding"

"manifoldly"

"manifoldness"

"manifolds"

"manikin"

"manikins"

"manila"

"manilas"

"manilla"

"manillas"

"manioc"

"maniocas"

"maniocs"

"maniple"

"maniples"

"manipulability"

"manipulable"

"manipulatable"

"manipulate"

"manipulated"

"manipulates"

"manipulating"

"manipulation"

"manipulations"

"manipulative"

"manipulatively"

"manipulator"

"manipulators"

"manipulatory"

"manitoba"

"manitou"

"manitous"

"mankind"

"manless"

"manlier"

"manliest"

"manlike"

"manliness"

"manly"

"manmade"

"manna"

"mannas"

"manned"

"mannequin"

"mannequins"

"manner"

"mannered"

"mannerism"

"mannerisms"

"mannerless"

"mannerliness"

"mannerly"

"manners"

"mannikin"

"mannikins"

"manning"

"mannish"

"mannishly"

"mannishness"

"manoeuver"

"manoeuvered"

"manoeuvering"

"manoeuvre"

"manoeuvred"

"manoeuvreing"

"manometer"

"manometers"

"manometric"

"manometries"

"manometry"

"manor"

"manorial"

"manorialism"

"manors"

"manos"

"manpack"

"manpower"

"manpowers"

"manque"

"manrope"

"mans"

"mansard"

"mansards"

"manse"

"manservant"

"manses"

"mansion"

"mansions"

"manslaughter"

"manslaughters"

"manslayer"

"manslayers"

"mansuetude"

"manta"

"mantas"

"mantel"

"mantelet"

"mantelets"

"mantelpiece"

"mantelpieces"

"mantels"

"mantes"

"mantic"

"mantid"

"mantids"

"mantilla"

"mantillas"

"mantis"

"mantises"

"mantissa"

"mantissas"

"mantle"

"mantled"

"mantlepiece"

"mantlepieces"

"mantles"

"mantlet"

"mantling"

"mantlings"

"mantra"

"mantrap"

"mantraps"

"mantras"

"mantua"

"mantuas"

"manual"

"manually"

"manuals"

"manubrial"

"manubrium"

"manubriums"

"manuever"

"manueverable"

"manuevered"

"manuevers"

"manufactories"

"manufactory"

"manufacturable"

"manufacture"

"manufactured"

"manufacturer"

"manufacturers"

"manufactures"

"manufacturing"

"manumission"

"manumissions"

"manumit"

"manumits"

"manumitted"

"manumitting"

"manure"

"manured"

"manurer"

"manures"

"manuring"

"manus"

"manuscript"

"manuscription"

"manuscripts"

"manward"

"manwise"

"manx"

"many"

"manyfold"

"mao"

"maoism"

"maoist"

"maoists"

"maori"

"maoris"

"map"

"maple"

"maples"

"mapmaker"

"mapmakers"

"mappable"

"mapped"

"mapper"

"mappers"

"mapping"

"mappings"

"maps"

"maquette"

"maquettes"

"maqui"

"mar"

"marabou"

"marabous"

"marabouts"

"maraca"

"maracas"

"maraschino"

"maraschinos"

"marathon"

"marathons"

"maraud"

"marauded"

"marauder"

"marauders"

"marauding"

"marauds"

"marble"

"marbled"

"marbleization"

"marbleize"

"marbleized"

"marbleizes"

"marbleizing"

"marbler"

"marblers"

"marbles"

"marblier"

"marbliest"

"marbling"

"marblings"

"marbly"

"marc"

"marcel"

"marcelled"

"marcels"

"march"

"marched"

"marcher"

"marchers"

"marches"

"marchesa"

"marching"

"marchioness"

"marchionesses"

"marcs"

"mardi"

"mare"

"mares"

"margaret"

"margarine"

"margarins"

"margays"

"marge"

"margent"

"margented"

"margents"

"marges"

"margin"

"marginal"

"marginalia"

"marginality"

"marginally"

"marginate"

"margined"

"margining"

"margins"

"margrave"

"margraves"

"marguerite"

"marguerites"

"maria"

"mariachi"

"mariachis"

"marie"

"marigold"

"marigolds"

"marigraph"

"marihuana"

"marijuana"

"marijuana buyers"

"marilyn"

"marimba"

"marimbas"

"marina"

"marinade"

"marinaded"

"marinades"

"marinading"

"marinara"

"marinaras"

"marinas"

"marinate"

"marinated"

"marinates"

"marinating"

"marine"

"mariner"

"mariners"

"marines"

"Mariology"

"marionette"

"marionettes"

"mariposa"

"mariposas"

"marish"

"marital"

"maritally"

"maritime"

"marjoram"

"marjorams"

"marjorie"

"mark"

"markdown"

"markdowns"

"marked"

"markedly"

"marker"

"markers"

"market"

"marketability"

"marketable"

"marketed"

"marketeer"

"marketeers"

"marketer"

"marketers"

"marketing"

"marketings"

"marketplace"

"marketplaces"

"markets"

"marketwise"

"marking"

"markings"

"markka"

"markkaa"

"marks"

"marksman"

"marksmanship"

"marksmen"

"markswoman"

"markswomen"

"markup"

"markups"

"marl"

"marled"

"marlier"

"marlin"

"marline"

"marlinespike"

"marlinespikes"

"marling"

"marlins"

"marmalade"

"marmalades"

"marmite"

"marmites"

"marmoreal"

"marmoset"

"marmosets"

"marmot"

"marmots"

"maroon"

"marooned"

"marooning"

"maroons"

"marque"

"marquee"

"marquees"

"marques"

"marquess"

"marquesses"

"marquetry"

"marquis"

"marquise"

"marquises"

"marquisette"

"marquisettes"

"marred"

"marrer"

"marrers"

"marriage"

"marriageability"

"marriageable"

"marriages"

"married"

"marrieds"

"marrier"

"marriers"

"marries"

"marring"

"marron"

"marrons"

"marrow"

"marrowbone"

"marrowbones"

"marrowed"

"marrowing"

"marrows"

"marrowy"

"marry"

"marrying"

"mars"

"marse"

"marseillaise"

"marseille"

"marseilles"

"marses"

"marsh"

"marshal"

"marshalcies"

"marshalcy"

"marshaled"

"marshaling"

"marshall"

"marshalled"

"marshalling"

"marshalls"

"marshals"

"marshes"

"marshier"

"marshiest"

"marshiness"

"marshlands"

"marshmallow"

"marshmallows"

"marshs"

"marshy"

"marsupia"

"marsupial"

"marsupialization"

"marsupialize"

"marsupializing"

"marsupials"

"marsupium"

"mart"

"marted"

"marten"

"martens"

"martha"

"martial"

"martialed"

"martialing"

"martialism"

"martialist"

"martialists"

"martialled"

"martialling"

"martially"

"martials"

"martian"

"martians"

"martin"

"martinet"

"martinets"

"martinez"

"marting"

"martingale"

"martingales"

"martini"

"martinis"

"martins"

"martlets"

"marts"

"martyr"

"martyrdom"

"martyrdoms"

"martyred"

"martyries"

"martyring"

"martyrology"

"martyrs"

"martyry"

"marvel"

"marveled"

"marveling"

"marvelled"

"marvelling"

"marvelous"

"marvelously"

"marvelousness"

"marvels"

"marx"

"marxian"

"marxism"

"marxist"

"marxists"

"mary"

"maryland"

"marylander"

"marylanders"

"marzipan"

"marzipans"

"mas"

"mascara"

"mascaras"

"maschera"

"mascon"

"mascons"

"mascot"

"mascots"

"masculine"

"masculinely"

"masculineness"

"masculines"

"masculinities"

"masculinity"

"masculinization"

"masculinize"

"masculinized"

"masculinizing"

"maser"

"masers"

"mash"

"mashed"

"masher"

"mashers"

"mashes"

"mashie"

"mashies"

"mashing"

"mashy"

"mask"

"maskable"

"masked"

"masker"

"maskers"

"masking"

"maskings"

"masks"

"masochism"

"masochist"

"masochistic"

"masochistically"

"masochists"

"mason"

"masoned"

"masonic"

"masonries"

"masonry"

"masons"

"masonwork"

"masque"

"masquer"

"masquerade"

"masqueraded"

"masquerader"

"masqueraders"

"masquerades"

"masquerading"

"masquers"

"masques"

"mass"

"massa"

"massachusetts"

"massacre"

"massacred"

"massacrer"

"massacrers"

"massacres"

"massacring"

"massage"

"massaged"

"massager"

"massagers"

"massages"

"massaging"

"massagist"

"massagists"

"massas"

"masscult"

"masse"

"massed"

"massedly"

"masses"

"masseur"

"masseurs"

"masseuse"

"masseuses"

"massier"

"massiest"

"massif"

"massifs"

"massiness"

"massing"

"massive"

"massively"

"massiveness"

"massless"

"masslessness"

"massy"

"mast"

"mastectomies"

"mastectomy"

"masted"

"master"

"mastered"

"masterful"

"masterfully"

"masterfulness"

"masteries"

"mastering"

"masterly"

"mastermind"

"masterminded"

"masterminding"

"masterminds"

"masterpiece"

"masterpieces"

"masters"

"masterwork"

"masterworks"

"mastery"

"masthead"

"mastheaded"

"mastheads"

"mastic"

"masticate"

"masticated"

"masticates"

"masticating"

"mastication"

"mastications"

"masticatory"

"mastics"

"mastiff"

"mastiffs"

"mastless"

"mastodon"

"mastodonic"

"mastodons"

"mastoid"

"mastoidal"

"mastoiditis"

"mastoids"

"mastology"

"masts"

"masturbate"

"masturbated"

"masturbates"

"masturbating"

"masturbation"

"masturbator"

"masturbators"

"masturbatory"

"mat"

"matador"

"matadors"

"match"

"matchable"

"matchbook"

"matchbooks"

"matchbox"

"matchboxes"

"matched"

"matcher"

"matchers"

"matches"

"matching"

"matchings"

"matchless"

"matchlessly"

"matchlock"

"matchlocks"

"matchmaker"

"matchmakers"

"matchmaking"

"mate"

"mated"

"mateless"

"mater"

"materfamilias"

"materia"

"material"

"materialism"

"materialist"

"materialistic"

"materialistically"

"materialists"

"materialities"

"materiality"

"materialization"

"materializations"

"materialize"

"materialized"

"materializes"

"materializing"

"materially"

"materialness"

"materials"

"materiel"

"materiels"

"maternal"

"maternalism"

"maternally"

"maternities"

"maternity"

"maters"

"mates"

"mateship"

"matey"

"mateys"

"math"

"math problem"

"mathematic"

"mathematical"

"mathematically"

"mathematician"

"mathematicians"

"mathematics"

"maths"

"matilda"

"matildas"

"matin"

"matinal"

"matinee"

"matinees"

"mating"

"matings"

"matins"

"matless"

"matriarch"

"matriarchal"

"matriarchies"

"matriarchs"

"matriarchy"

"matrices"

"matricidal"

"matricide"

"matricides"

"matriculant"

"matriculants"

"matriculate"

"matriculated"

"matriculates"

"matriculating"

"matriculation"

"matriculations"

"matriline"

"matrilineage"

"matrilineal"

"matrilineally"

"matrilinear"

"matrilinearly"

"matrilinies"

"matriliny"

"matrimonial"

"matrimonially"

"matrimony"

"matrix"

"matrixes"

"matrixing"

"matron"

"matronal"

"matronliness"

"matronly"

"matrons"

"mats"

"matt"

"matte"

"matted"

"mattedly"

"matter"

"mattered"

"mattering"

"matters"

"mattery"

"mattes"

"matthew"

"matting"

"mattings"

"mattins"

"mattock"

"mattocks"

"mattress"

"mattresses"

"matts"

"maturate"

"maturated"

"maturates"

"maturating"

"maturation"

"maturational"

"maturations"

"maturative"

"mature"

"matured"

"maturely"

"matureness"

"maturer"

"matures"

"maturest"

"maturing"

"maturities"

"maturity"

"matutinal"

"matutinally"

"matzahs"

"matzo"

"matzoh"

"matzohs"

"matzos"

"matzoth"

"maudlin"

"maudlinly"

"maul"

"mauled"

"mauler"

"maulers"

"mauling"

"mauls"

"maunder"

"maundered"

"maunderer"

"maunderers"

"maundering"

"maunders"

"maundies"

"maundy"

"maupassant"

"mauritania"

"mauritanian"

"mauritanians"

"mausolea"

"mausoleum"

"mausoleums"

"maut"

"mauve"

"mauves"

"maven"

"mavens"

"maverick"

"mavericks"

"mavin"

"mavins"

"maw"

"mawkish"

"mawkishly"

"mawkishness"

"maws"

"max"

"maxi"

"maxicoats"

"maxilla"

"maxillae"

"maxillary"

"maxim"

"maxima"

"maximal"

"maximally"

"maximals"

"maximin"

"maximins"

"maximite"

"maximization"

"maximize"

"maximized"

"maximizer"

"maximizers"

"maximizes"

"maximizing"

"maxims"

"maximum"

"maximums"

"maxis"

"maxixe"

"maxwell"

"maxwells"

"may"

"maya"

"mayan"

"mayans"

"mayapple"

"mayapples"

"mayas"

"maybe"

"maybushes"

"mayday"

"maydays"

"mayest"

"mayflies"

"mayflower"

"mayflowers"

"mayfly"

"mayhap"

"mayhem"

"mayhemming"

"mayhems"

"maying"

"mayings"

"mayo"

"mayonnaise"

"mayor"

"mayoral"

"mayoralties"

"mayoralty"

"mayoress"

"mayoresses"

"mayors"

"mayorship"

"mayorships"

"maypole"

"maypoles"

"maypop"

"maypops"

"mays"

"mayst"

"mayvin"

"mayvins"

"mayweed"

"mayweeds"

"maze"

"mazed"

"mazedly"

"mazel"

"mazer"

"mazers"

"mazes"

"mazier"

"maziest"

"mazily"

"maziness"

"mazing"

"mazology"

"mazuma"

"mazurka"

"mazurkas"

"mazy"

"mb"

"mc"

"mcdonald"

"md"

"MD encyclopedia"

"me"

"mea"

"mead"

"meadow"

"meadowland"

"meadowlands"

"meadowlark"

"meadowlarks"

"meadows"

"meadowsweet"

"meadowsweets"

"meadowy"

"meads"

"meager"

"meagerly"

"meagerness"

"meal"

"mealie"

"mealier"

"mealies"

"mealiest"

"meals"

"mealtime"

"mealtimes"

"mealworm"

"mealworms"

"mealy"

"mealybug"

"mealybugs"

"mealymouthed"

"mean"

"meander"

"meandered"

"meanderer"

"meanderers"

"meandering"

"meanders"

"meaner"

"meaners"

"meanest"

"meanie"

"meanies"

"meaning"

"meaningful"

"meaningfully"

"meaningfulness"

"meaningless"

"meanings"

"meanly"

"meanness"

"means"

"meanspirited"

"meant"

"meantime"

"meantimes"

"meanwhile"

"meany"

"meas"

"measle"

"measled"

"measles"

"measlier"

"measliest"

"measly"

"measurability"

"measurable"

"measurably"

"measurage"

"measure"

"measured"

"measureless"

"measurement"

"measurements"

"measurer"

"measurers"

"measures"

"measuring"

"meat"

"meatball"

"meatballs"

"meathead"

"meatheads"

"meatier"

"meatiest"

"meatily"

"meatiness"

"meatless"

"meats"

"meaty"

"mecate"

"mecca"

"meccas"

"mech"

"mechanic"

"mechanical"

"mechanically"

"mechanics"

"mechanism"

"mechanisms"

"mechanisms of action"

"mechanist"

"mechanistic"

"mechanistically"

"mechanists"

"mechanization"

"mechanize"

"mechanized"

"mechanizer"

"mechanizers"

"mechanizes"

"mechanizing"

"mechanoreception"

"mechanoreceptive"

"mechanoreceptor"

"mechanotherapies"

"mechanotherapist"

"mechanotherapists"

"mechanotheraputic"

"mechanotheraputically"

"mechanotherapy"

"mecometer"

"meconology"

"mecum"

"mecums"

"medal"

"medaled"

"medalist"

"medalists"

"medalling"

"medallion"

"medallions"

"medals"

"meddle"

"meddled"

"meddler"

"meddlers"

"meddles"

"meddlesome"

"meddlesomely"

"meddling"

"medevac"

"medevacs"

"media"

"mediacy"

"medial"

"medially"

"medials"

"median"

"medianly"

"medians"

"medias"

"mediate"

"mediated"

"mediately"

"mediates"

"mediating"

"mediation"

"mediational"

"mediative"

"mediator"

"mediatorial"

"mediators"

"mediatorship"

"medic"

"medicable"

"medicably"

"medicaid"

"medicaids"

"medical"

"Medical Acronyms"

"medical and health field"

"medical calculator"

"medical card"

"medical care"

"medical check"

"medical condition"

"medical conditions"

"medical content"

"Medical Control Protocol"

"medical cosmetic"

"medical diagnosis"

"Medical diagnosis app"

"Medical Doctor"

"medical emergency"

"medical equipment"

"medical field"

"medical guide"

"Medical handbook"

"medical history"

"medical information"

"Medical Issues"

"medical knowledge"

"Medical Law"

"Medical marijuana"

"medical oversight"

"medical record"

"medical record system"

"medical reference"

"medical service"

"medical student"

"medical system"

"Medical Terminology"

"medical terms"

"Medical Transcription"

"medical user"

"medically"

"medicals"

"medicament"

"medicaments"

"medicant"

"medicare"

"medicares"

"medicate"

"medicated"

"medicates"

"medicating"

"medication"

"Medication management"

"medications"

"medicative"

"medicator"

"medicinable"

"medicinal"

"medicinally"

"medicine"

"Medicine details"

"medicined"

"medicines"

"medicining"

"medicks"

"medico"

"medicos"

"medics"

"medieval"

"medievalism"

"medievalist"

"medievalists"

"medievally"

"medievals"

"mediocre"

"mediocrities"

"mediocrity"

"Mediquation"

"meditate"

"meditated"

"meditates"

"meditating"

"meditatio"

"meditation"

"meditations"

"meditative"

"meditative states"

"meditative tunes"

"meditatively"

"mediterranean"

"medium"

"mediumistic"

"mediums"

"medius"

"medlars"

"medley"

"medleys"

"MedPage"

"meds"

"medulla"

"medullae"

"medullar"

"medullary"

"medullas"

"medusa"

"medusan"

"medusas"

"medusoid"

"medusoids"

"meed"

"meeds"

"meek"

"meeker"

"meekest"

"meekly"

"meekness"

"meerschaum"

"meerschaums"

"meet"

"meeter"

"meeters"

"meeting"

"meeting timer"

"meetinghouse"

"meetings"

"meetly"

"meetness"

"meets"

"meg"

"megabar"

"megabit"

"megabits"

"megabuck"

"megabucks"

"megabyte"

"megabytes"

"megacephalous"

"megacolon"

"megacycle"

"megacycles"

"megadeath"

"megadeaths"

"megadyne"

"megadynes"

"megahertz"

"megakaryocytic"

"megalith"

"megalithic"

"megaliths"

"megalomania"

"megalomaniac"

"megalomaniacal"

"megalomaniacally"

"megalomaniacs"

"megalopolis"

"megalopolises"

"megameter"

"megaphone"

"megaphones"

"megapod"

"megascope"

"megaton"

"megatons"

"megavitamin"

"megavolt"

"megavolts"

"megawatt"

"megawatts"

"megillah"

"megillahs"

"megohm"

"megohms"

"mein"

"meioses"

"meiosis"

"meiotic"

"mekometer"

"mekong"

"mel"

"melamine"

"melamines"

"melancholia"

"melancholiac"

"melancholiacs"

"melancholic"

"melancholically"

"melancholies"

"melancholy"

"melanesia"

"melanesian"

"melanesians"

"melange"

"melanges"

"melanic"

"melanin"

"melanins"

"melanism"

"melanisms"

"melanists"

"melanites"

"melanized"

"melanizes"

"melanocarcinoma"

"melanogen"

"melanoids"

"melanoma"

"melanomas"

"melanomata"

"melanophore"

"melanotic"

"melba"

"melbourne"

"melchizedek"

"meld"

"melded"

"melder"

"melders"

"melding"

"meldometer"

"melds"

"melee"

"melees"

"meliorate"

"meliorated"

"meliorates"

"meliorating"

"melioration"

"meliorations"

"meliorative"

"melittology"

"mellific"

"mellifluent"

"mellifluous"

"mellifluously"

"mellitus"

"mellow"

"mellowed"

"mellower"

"mellowest"

"mellowing"

"mellowly"

"mellowness"

"mellows"

"melodeon"

"melodeons"

"melodic"

"melodically"

"melodies"

"melodious"

"melodiously"

"melodiousness"

"melodist"

"melodists"

"melodize"

"melodized"

"melodizes"

"melodizing"

"melodrama"

"melodramas"

"melodramatic"

"melodramatically"

"melodramatics"

"melodramatist"

"melodramatists"

"melody"

"melomania"

"melon"

"melons"

"meloplasties"

"melt"

"meltable"

"meltage"

"meltages"

"meltdown"

"meltdowns"

"melted"

"melter"

"melters"

"melting"

"meltingly"

"melton"

"meltons"

"melts"

"meltwater"

"member"

"membered"

"members"

"membership"

"memberships"

"membranaceous"

"membranal"

"membrane"

"membranes"

"membranous"

"membranously"

"memento"

"mementoes"

"mementos"

"memo"

"memoir"

"memoirs"

"memorabilia"

"memorability"

"memorable"

"memorableness"

"memorably"

"memoranda"

"memorandum"

"memorandums"

"memorial"

"memorialist"

"memorialize"

"memorialized"

"memorializes"

"memorializing"

"memorials"

"memories"

"memorization"

"memorize"

"memorized"

"memorizer"

"memorizers"

"memorizes"

"memorizing"

"memory"

"memos"

"memphis"

"memsahib"

"memsahibs"

"men"

"menace"

"menaced"

"menacer"

"menacers"

"menaces"

"menacing"

"menacingly"

"menads"

"menage"

"menagerie"

"menageries"

"menages"

"menarche"

"menarches"

"mend"

"mendable"

"mendacious"

"mendaciously"

"mendacities"

"mendacity"

"mended"

"mendel"

"mendelevium"

"mendelian"

"mendelianism"

"mendelianist"

"mendelism"

"mendelist"

"mendelize"

"mendelssohn"

"mender"

"menders"

"mendicancies"

"mendicancy"

"mendicant"

"mendicants"

"mending"

"mendings"

"mends"

"menfolk"

"menfolks"

"menhaden"

"menhadens"

"menhir"

"menhirs"

"menial"

"menially"

"menials"

"meningeal"

"meninges"

"meningism"

"meningitic"

"meningitis"

"meninx"

"meniscal"

"meniscectomy"

"menisci"

"meniscoid"

"meniscus"

"meniscuses"

"mennonite"

"mennonites"

"menologies"

"menopausal"

"menopause"

"menorah"

"menorahs"

"menorrhea"

"mens"

"mensal"

"mensas"

"mensch"

"menschen"

"mensches"

"mensed"

"menservants"

"menses"

"mensing"

"menstrual"

"menstrual calendar"

"menstrual cycle"

"menstrual cycles"

"menstrual period"

"menstruant"

"menstruate"

"menstruated"

"menstruates"

"menstruating"

"menstruation"

"menstruations"

"menstruous"

"menstruum"

"mensurability"

"mensurable"

"mensural"

"mensuration"

"mensurative"

"menswear"

"menswears"

"mental"

"mental activity"

"mental clarity"

"Mental disorders"

"mental health"

"mental healthiness"

"mental refresher"

"mentalist"

"mentalists"

"mentalities"

"mentality"

"mentally"

"mentation"

"menthe"

"menthol"

"mentholated"

"menthols"

"mention"

"mentionable"

"mentioned"

"mentioner"

"mentioners"

"mentioning"

"mentions"

"mentis"

"mentor"

"mentors"

"menu"

"menus"

"meow"

"meowed"

"meowing"

"meows"

"mephistopheles"

"mephitic"

"mephitis"

"meprobamate"

"mer"

"mercantile"

"mercantilism"

"mercantilistic"

"mercaptan"

"mercenaries"

"mercenarily"

"mercenariness"

"mercenary"

"mercer"

"mercerize"

"mercerized"

"mercerizes"

"mercerizing"

"mercers"

"mercery"

"merchandisable"

"merchandise"

"merchandised"

"merchandiser"

"merchandisers"

"merchandises"

"merchandising"

"merchandized"

"merchant"

"merchantability"

"merchantable"

"merchanted"

"merchantman"

"merchantmen"

"merchantries"

"merchantry"

"merchants"

"merci"

"mercies"

"merciful"

"mercifully"

"mercifulness"

"merciless"

"mercilessly"

"mercurial"

"mercurialism"

"mercurialize"

"mercurially"

"mercurialness"

"mercuric"

"mercuries"

"mercurochrome"

"mercurous"

"mercury"

"mercy"

"mere"

"merely"

"merengue"

"merengues"

"mereology"

"merer"

"meres"

"merest"

"meretricious"

"meretriciously"

"meretriciousness"

"merganser"

"mergansers"

"merge"

"merged"

"mergence"

"mergences"

"merger"

"mergers"

"merges"

"merging"

"merican"

"meridian"

"meridians"

"meridiem"

"meringue"

"meringues"

"merino"

"merinos"

"merit"

"meritable"

"merited"

"meritedly"

"meriting"

"meritocracies"

"meritocracy"

"meritorious"

"meritoriously"

"meritoriousness"

"merits"

"merlin"

"merlins"

"merlon"

"merlons"

"mermaid"

"mermaids"

"merman"

"mermen"

"merrier"

"merriest"

"merrily"

"merriment"

"merriness"

"merry"

"merrymaker"

"merrymakers"

"merrymaking"

"mesa"

"mesalliance"

"mesalliances"

"mesas"

"mescal"

"mescaline"

"mescalism"

"mescals"

"mesdames"

"mesdemoiselles"

"meseemed"

"meseems"

"mesenteries"

"mesentery"

"mesh"

"meshed"

"meshes"

"meshier"

"meshing"

"meshwork"

"meshworks"

"meshy"

"mesmeric"

"mesmerism"

"mesmerist"

"mesmerists"

"mesmerization"

"mesmerize"

"mesmerized"

"mesmerizer"

"mesmerizers"

"mesmerizes"

"mesmerizing"

"mesology"

"mesomorph"

"mesomorphic"

"meson"

"mesonic"

"mesons"

"mesopotamia"

"mesopotamian"

"mesosphere"

"mesospheric"

"mesozoa"

"mesozoan"

"mesozoic"

"mesquit"

"mesquite"

"mesquites"

"mess"

"message"

"messages"

"messed"

"messenger"

"messengers"

"messes"

"messiah"

"messiahs"

"messianic"

"messier"

"messiest"

"messieurs"

"messily"

"messiness"

"messing"

"messman"

"messmate"

"messmates"

"messmen"

"messrs"

"messy"

"mestiza"

"mestizas"

"mestizo"

"mestizoes"

"mestizos"

"met"

"meta"

"metabases"

"metabasis"

"metabolic"

"metabolical"

"metabolically"

"metabolism"

"metabolite"

"metabolites"

"metabolizability"

"metabolizable"

"metabolize"

"metabolized"

"metabolizes"

"metabolizing"

"metacarpal"

"metacarpals"

"metacarpi"

"metacarpus"

"metagalaxy"

"metagenetically"

"metal"

"metalaw"

"metaled"

"metaling"

"metalist"

"metalists"

"metalize"

"metalized"

"metalizes"

"metalizing"

"metalled"

"metallic"

"metallically"

"metalliferous"

"metalling"

"metalloenzyme"

"metallogeny"

"metallography"

"metalloid"

"metalloidal"

"metallurgic"

"metallurgical"

"metallurgically"

"metallurgist"

"metallurgists"

"metallurgy"

"metals"

"metalware"

"metalwork"

"metalworker"

"metalworkers"

"metalworking"

"metamer"

"metameric"

"metamers"

"metamorphic"

"metamorphism"

"metamorphisms"

"metamorphose"

"metamorphosed"

"metamorphoses"

"metamorphosing"

"metamorphosis"

"metamorphous"

"metaphase"

"metaphor"

"metaphoric"

"metaphorical"

"metaphorically"

"metaphors"

"metaphysical"

"metaphysically"

"metaphysician"

"metaphysicians"

"metaphysics"

"metapolitics"

"metapsychology"

"metastases"

"metastasis"

"metastasize"

"metastasized"

"metastasizes"

"metastasizing"

"metastatic"

"metatarsal"

"metatarsally"

"metatarsi"

"metatarsus"

"metatheses"

"metathesis"

"metazoa"

"metazoan"

"metazoans"

"metazoic"

"mete"

"meted"

"metempsychoses"

"metempsychosis"

"meteor"

"meteoric"

"meteorically"

"meteorism"

"meteorite"

"meteorites"

"meteoritic"

"meteoritics"

"meteorograph"

"meteoroid"

"meteoroids"

"meteorological"

"meteorologist"

"meteorologists"

"meteorology"

"meteors"

"meter"

"meterage"

"meterages"

"metered"

"metering"

"meterological"

"meters"

"metes"

"methacrylate"

"methadone"

"methamphetamine"

"methane"

"methanes"

"methanol"

"methanols"

"methanometer"

"methaqualone"

"methinks"

"method"

"methodic"

"methodical"

"methodically"

"methodism"

"methodist"

"methodists"

"methodize"

"methodized"

"methodizes"

"methodizing"

"methodological"

"methodologically"

"methodologies"

"methodology"

"methods"

"methomania"

"methought"

"methyl"

"methylene"

"methylparaben"

"methyls"

"meticulosity"

"meticulous"

"meticulously"

"meticulousness"

"metier"

"metiers"

"meting"

"metonym"

"metonymies"

"metonyms"

"metonymy"

"metre"

"metred"

"metres"

"metric"

"metrical"

"metrically"

"metricate"

"metricated"

"metricates"

"metricating"

"metrication"

"metricize"

"metricized"

"metricizes"

"metricizing"

"metrics"

"metrified"

"metrifies"

"metrify"

"metrifying"

"metring"

"metrist"

"metrists"

"metro"

"metrography"

"metroliner"

"metroliners"

"metrologies"

"metrology"

"metromania"

"metronome"

"metronomes"

"metronomic"

"metrophobia"

"metropolis"

"metropolises"

"metropolitan"

"metropolitanize"

"metropolitanized"

"metros"

"mettle"

"mettled"

"mettles"

"mettlesome"

"meuniere"

"mew"

"mewed"

"mewing"

"mewl"

"mewled"

"mewler"

"mewlers"

"mewling"

"mewls"

"mews"

"mexican"

"mexicans"

"mexico"

"mezcal"

"mezcals"

"mezquit"

"mezquite"

"mezquites"

"mezuza"

"mezuzah"

"mezuzahs"

"mezuzas"

"mezzanine"

"mezzanines"

"mezzo"

"mezzos"

"mf"

"mfd"

"mfg"

"mg"

"mHealth"

"mho"

"miami"

"miaou"

"miaoued"

"miaouing"

"miaous"

"miaow"

"miaowed"

"miaowing"

"miaows"

"miasm"

"miasma"

"miasmal"

"miasmas"

"miasmata"

"miasmatic"

"miasmic"

"miasms"

"miaul"

"miauled"

"mibs"

"mica"

"micas"

"mice"

"michael"

"michelangelo"

"michigan"

"mick"

"mickey"

"mickeys"

"mickle"

"micks"

"micro"

"microanalyses"

"microanalysis"

"microanalytic"

"microanalytical"

"microanatomy"

"microbarograph"

"microbars"

"microbe"

"microbes"

"microbial"

"microbian"

"microbic"

"microbicidal"

"microbicide"

"microbiologic"

"microbiological"

"microbiologies"

"microbiologist"

"microbiologists"

"microbiology"

"microbiotic"

"microbus"

"microbuses"

"microcalorimeter"

"microcephalic"

"microcephalous"

"microcephalus"

"microcephaly"

"microchemistry"

"microclimate"

"microclimates"

"microclimatological"

"microclimatology"

"microcomputer"

"microcomputers"

"microcopies"

"microcopy"

"microcosm"

"microcosmic"

"microcosmical"

"microcosms"

"microdissection"

"microelectronic"

"microelectronics"

"microfiche"

"microfiches"

"microfilm"

"microfilmed"

"microfilmer"

"microfilming"

"microfilms"

"microform"

"microforms"

"microgram"

"microgramme"

"microgrammes"

"micrograms"

"micrograph"

"micrographs"

"micrography"

"microgroove"

"microgrooves"

"microhm"

"microinstruction"

"microinstructions"

"microlith"

"micrologic"

"micrology"

"micromania"

"micromanipulator"

"micromanipulators"

"micromeli"

"micrometer"

"micrometers"

"micromillimeter"

"microminiature"

"microminiaturization"

"microminiaturizations"

"microminiaturize"

"microminiaturized"

"micron"

"micronesia"

"micronesian"

"micronesians"

"micronometer"

"microns"

"micronutrient"

"microorganism"

"microorganisms"

"micropalaeontology"

"microphone"

"microphones"

"microphotograph"

"microphotographed"

"microphotographic"

"microphotographing"

"microphotographs"

"microphotography"

"microphysics"

"microphytology"

"micropipette"

"microprocessing"

"microprocessor"

"microprocessors"

"microprogram"

"microprogrammed"

"microprogramming"

"microradiographical"

"microradiographically"

"microradiography"

"micros"

"microscope"

"microscopes"

"microscopic"

"microscopical"

"microscopically"

"microscopies"

"microscopist"

"microscopy"

"microsecond"

"microseconds"

"microseismograph"

"microseismometer"

"microspace"

"microspacing"

"microstate"

"microstates"

"microstructural"

"microstructure"

"microsurgeon"

"microsurgeons"

"microsurgeries"

"microsurgery"

"microsurgical"

"microsystems"

"microtome"

"microtomy"

"microvasculature"

"microvolt"

"microwave"

"microwaves"

"microzoon"

"micturate"

"mid"

"midair"

"midairs"

"midas"

"midbody"

"midbrain"

"midbrains"

"midchannel"

"midday"

"middays"

"midden"

"middens"

"middies"

"middle"

"middlebrow"

"middlebrowism"

"middlebrows"

"middleclass"

"middled"

"middleman"

"middlemen"

"middlemost"

"middler"

"middlers"

"middles"

"middleweight"

"middleweights"

"middling"

"middlingly"

"middlings"

"middy"

"mideast"

"midfield"

"midge"

"midges"

"midget"

"midgets"

"midgut"

"midguts"

"midi"

"midiron"

"midis"

"midland"

"midlands"

"midleg"

"midlegs"

"midline"

"midlines"

"midmonth"

"midmonths"

"midmorning"

"midmost"

"midmosts"

"midnight"

"midnights"

"midpoint"

"midpoints"

"midrange"

"midranges"

"midrib"

"midribs"

"midriff"

"midriffs"

"mids"

"midsection"

"midship"

"midshipman"

"midshipmen"

"midships"

"midst"

"midstream"

"midsts"

"midsummer"

"midsummers"

"midterm"

"midterms"

"midtown"

"midtowns"

"midway"

"midways"

"midweek"

"midweekly"

"midweeks"

"midwest"

"midwestern"

"midwesterner"

"midwesterners"

"midwife"

"midwifed"

"midwiferies"

"midwifery"

"midwifes"

"midwifing"

"midwinter"

"midwinters"

"midwived"

"midwives"

"midwiving"

"midyear"

"midyears"

"mien"

"miens"

"miff"

"miffed"

"miffing"

"miffs"

"miffy"

"mig"

"miggs"

"might"

"mightier"

"mightiest"

"mightily"

"mightiness"

"mights"

"mighty"

"mignon"

"mignonette"

"mignonettes"

"mignonne"

"mignons"

"migraine"

"migraines"

"migrant"

"migrants"

"migrate"

"migrated"

"migrates"

"migrating"

"migration"

"migrational"

"migrations"

"migrator"

"migrators"

"migratory"

"migs"

"mijnheers"

"mikado"

"mikados"

"mike"

"mikes"

"mikrons"

"mikvah"

"mikveh"

"mil"

"miladies"

"miladis"

"milady"

"milage"

"milages"

"milan"

"milanese"

"milch"

"mild"

"milden"

"mildened"

"mildening"

"mildens"

"milder"

"mildest"

"mildew"

"mildewed"

"mildewing"

"mildews"

"mildewy"

"mildly"

"mildness"

"mildred"

"mile"

"mileage"

"mileages"

"mileometer"

"milepost"

"mileposts"

"miler"

"milers"

"miles"

"milestone"

"milestones"

"milfoil"

"milfoils"

"milieu"

"milieus"

"milieux"

"militancy"

"militant"

"militantly"

"militantness"

"militants"

"militaries"

"militarily"

"militarism"

"militarist"

"militaristic"

"militarists"

"militarize"

"militarized"

"militarizes"

"militarizing"

"military"

"militate"

"militated"

"militates"

"militating"

"militia"

"militiaman"

"militiamen"

"militias"

"milk"

"milked"

"milker"

"milkers"

"milkier"

"milkiest"

"milkily"

"milkiness"

"milking"

"milkmaid"

"milkmaids"

"milkman"

"milkmen"

"milks"

"milksop"

"milksops"

"milkweed"

"milkweeds"

"milkwood"

"milkwort"

"milky"

"mill"

"millable"

"millage"

"milldam"

"milldams"

"mille"

"milled"

"millennia"

"millennial"

"millennium"

"millenniums"

"miller"

"millers"

"milles"

"millet"

"millets"

"milliammeter"

"milliampere"

"milliamperes"

"milliard"

"milliards"

"millibar"

"millibars"

"millier"

"milligram"

"milligrams"

"milliliter"

"milliliters"

"millimeter"

"millimeters"

"millimetric"

"millimicron"

"milliner"

"milliners"

"millinery"

"milling"

"millings"

"milliohms"

"million"

"millionaire"

"millionaires"

"millions"

"millionth"

"millionths"

"millipede"

"millipedes"

"millirem"

"millirems"

"millisecond"

"milliseconds"

"millivolt"

"millivolts"

"millpond"

"millponds"

"millrace"

"millraces"

"millrun"

"millruns"

"mills"

"millstone"

"millstones"

"millstream"

"millstreams"

"millwork"

"millworks"

"millwright"

"millwrights"

"milord"

"milords"

"milos"

"milquetoast"

"milquetoasts"

"mils"

"milt"

"miltiest"

"milton"

"milwaukee"

"mim"

"mime"

"mimed"

"mimeo"

"mimeoed"

"mimeograph"

"mimeographed"

"mimeographing"

"mimeographs"

"mimeoing"

"mimeos"

"mimer"

"mimers"

"mimes"

"mimesis"

"mimetic"

"mimetically"

"mimic"

"mimical"

"mimicked"

"mimicker"

"mimickers"

"mimicking"

"mimicries"

"mimicry"

"mimics"

"miming"

"mimosa"

"mimosas"

"min"

"minable"

"minaciousness"

"minacity"

"minaret"

"minarets"

"minatory"

"mince"

"minced"

"mincemeat"

"mincer"

"mincers"

"minces"

"mincier"

"mincing"

"mincy"

"mind"

"minded"

"mindedly"

"mindedness"

"minder"

"minders"

"mindful"

"mindfully"

"mindfulness"

"minding"

"mindless"

"mindlessly"

"mindlessness"

"minds"

"mine"

"mineable"

"mined"

"minelayer"

"minelayers"

"miner"

"mineral"

"mineralization"

"mineralize"

"mineralized"

"mineralizes"

"mineralizing"

"mineralogic"

"mineralogical"

"mineralogically"

"mineralogist"

"mineralogists"

"mineralogy"

"minerals"

"miners"

"minerva"

"mines"

"minestrone"

"minesweeper"

"minesweepers"

"ming"

"mingle"

"mingled"

"mingler"

"minglers"

"mingles"

"mingling"

"mingy"

"mini"

"miniature"

"miniatures"

"miniaturist"

"miniaturists"

"miniaturization"

"miniaturizations"

"miniaturize"

"miniaturized"

"miniaturizes"

"miniaturizing"

"minibike"

"minibikes"

"minibus"

"minibuses"

"minibusses"

"minicab"

"minicabs"

"minicar"

"minicars"

"minicomputer"

"minicomputers"

"minidisk"

"minidisks"

"minifloppies"

"minifloppy"

"minify"

"minifying"

"minikin"

"minikins"

"minim"

"minima"

"minimal"

"minimalist"

"minimalists"

"minimally"

"minimals"

"minimax"

"minimaxes"

"minimization"

"minimize"

"minimized"

"minimizer"

"minimizers"

"minimizes"

"minimizing"

"minims"

"minimum"

"minimums"

"mining"

"minings"

"minion"

"minions"

"minis"

"miniscule"

"miniseries"

"miniskirt"

"miniskirted"

"miniskirts"

"ministate"

"ministates"

"minister"

"ministered"

"ministerial"

"ministerially"

"ministering"

"ministers"

"ministrant"

"ministrants"

"ministration"

"ministrations"

"ministries"

"ministry"

"mink"

"minks"

"minneapolis"

"minnesinger"

"minnesingers"

"minnesota"

"minnesotan"

"minnesotans"

"minnie"

"minnow"

"minnows"

"minny"

"minor"

"minora"

"minorca"

"minorcas"

"minored"

"minoring"

"minorities"

"minority"

"minors"

"mins"

"minster"

"minsters"

"minstrel"

"minstrels"

"minstrelsy"

"mint"

"mintage"

"minted"

"minter"

"minters"

"mintier"

"mintiest"

"minting"

"mintmark"

"mints"

"minty"

"minuend"

"minuends"

"minuet"

"minuets"

"minus"

"minuscule"

"minuscules"

"minuses"

"minute"

"minuted"

"minutely"

"minuteman"

"minutemen"

"minuteness"

"minuter"

"minutes"

"minutest"

"minutia"

"minutiae"

"minutial"

"minuting"

"minx"

"minxes"

"minxish"

"minyan"

"minyanim"

"minyans"

"miocene"

"miosis"

"miotic"

"mirabile"

"miracle"

"miracles"

"miraculous"

"miraculously"

"miraculousness"

"mirage"

"mirages"

"mire"

"mired"

"mires"

"miriam"

"mirier"

"miriest"

"miriness"

"miring"

"mirk"

"mirkest"

"mirkier"

"mirkily"

"mirks"

"mirky"

"mirror"

"mirrored"

"mirroring"

"mirrors"

"mirth"

"mirthful"

"mirthfully"

"mirthfulness"

"mirthless"

"mirths"

"mirv"

"mirvs"

"miry"

"misact"

"misadd"

"misadded"

"misaddress"

"misaddressed"

"misaddresses"

"misaddressing"

"misadjust"

"misadjusted"

"misadjusting"

"misadjusts"

"misadministration"

"misadventure"

"misadventures"

"misadvise"

"misadvised"

"misadvises"

"misadvising"

"misaim"

"misaimed"

"misaligned"

"misalignment"

"misalignments"

"misalleging"

"misalliance"

"misalliances"

"misalphabetize"

"misalphabetized"

"misalphabetizes"

"misalphabetizing"

"misanthrope"

"misanthropes"

"misanthropic"

"misanthropical"

"misanthropically"

"misanthropies"

"misanthropist"

"misanthropists"

"misanthropy"

"misapplication"

"misapplied"

"misapplier"

"misapplies"

"misapply"

"misapplying"

"misapprehend"

"misapprehended"

"misapprehending"

"misapprehends"

"misapprehension"

"misapprehensions"

"misappropriate"

"misappropriated"

"misappropriates"

"misappropriating"

"misappropriation"

"misappropriations"

"misarrange"

"misarranged"

"misarrangement"

"misarrangements"

"misarranges"

"misarranging"

"misbeget"

"misbegetting"

"misbegot"

"misbegotten"

"misbehave"

"misbehaved"

"misbehaver"

"misbehavers"

"misbehaves"

"misbehaving"

"misbehavior"

"misbelief"

"misbeliefs"

"misbestow"

"misbestowed"

"misbestowing"

"misbestows"

"misbiasing"

"misbiassed"

"misbilling"

"misbills"

"misc"

"miscalculate"

"miscalculated"

"miscalculates"

"miscalculating"

"miscalculation"

"miscalculations"

"miscall"

"miscalled"

"miscalling"

"miscalls"

"miscarriage"

"miscarriages"

"miscarried"

"miscarries"

"miscarry"

"miscarrying"

"miscast"

"miscasting"

"miscasts"

"miscegenation"

"miscegenational"

"miscegenations"

"miscellaneous"

"miscellaneously"

"miscellaneousness"

"miscellanies"

"miscellany"

"mischance"

"mischances"

"mischarge"

"mischarged"

"mischarges"

"mischarging"

"mischief"

"mischiefs"

"mischievous"

"mischievously"

"mischievousness"

"miscibilities"

"miscibility"

"miscible"

"misclassification"

"misclassifications"

"misclassified"

"misclassifies"

"misclassify"

"misclassifying"

"miscognizant"

"misconceive"

"misconceived"

"misconceives"

"misconceiving"

"misconception"

"misconceptions"

"misconduct"

"misconstruction"

"misconstructions"

"misconstrue"

"misconstrued"

"misconstrues"

"misconstruing"

"miscontinuance"

"miscopied"

"miscopies"

"miscopy"

"miscopying"

"miscount"

"miscounted"

"miscounting"

"miscounts"

"miscreant"

"miscreants"

"miscue"

"miscued"

"miscues"

"miscuing"

"miscut"

"misdated"

"misdates"

"misdeal"

"misdealing"

"misdeals"

"misdealt"

"misdeed"

"misdeeds"

"misdeems"

"misdefine"

"misdefined"

"misdefines"

"misdefining"

"misdeliveries"

"misdemeanant"

"misdemeanor"

"misdemeanors"

"misdescription"

"misdescriptive"

"misdiagnose"

"misdiagnosed"

"misdiagnoses"

"misdiagnosing"

"misdiagnosis"

"misdid"

"misdirect"

"misdirected"

"misdirecting"

"misdirection"

"misdirections"

"misdirects"

"misdo"

"misdoer"

"misdoers"

"misdoes"

"misdoing"

"misdoings"

"misdone"

"misdoubt"

"misdoubted"

"misdoubts"

"misdrawn"

"misdraws"

"mise"

"misedits"

"miseducate"

"miseducated"

"miseducates"

"miseducating"

"miseducation"

"misemploy"

"misemployed"

"misemploying"

"misemployment"

"misemploys"

"miser"

"miserabilia"

"miserable"

"miserableness"

"miserably"

"misereres"

"misericordia"

"miseries"

"miserliness"

"miserly"

"misers"

"misery"

"misfeasance"

"misfeasances"

"misfeasor"

"misfeasors"

"misfile"

"misfiled"

"misfiles"

"misfiling"

"misfire"

"misfired"

"misfires"

"misfiring"

"misfit"

"misfits"

"misfitted"

"misformed"

"misfortune"

"misfortunes"

"misgive"

"misgiving"

"misgivings"

"misgovern"

"misgoverned"

"misgoverning"

"misgovernment"

"misgoverns"

"misguidance"

"misguide"

"misguided"

"misguidedly"

"misguider"

"misguiders"

"misguides"

"misguiding"

"mishandle"

"mishandled"

"mishandles"

"mishandling"

"mishap"

"mishaps"

"mishear"

"misheard"

"mishearing"

"mishears"

"mishmash"

"mishmashes"

"mishmosh"

"mishmoshes"

"misidentification"

"misidentifications"

"misidentified"

"misidentifies"

"misinform"

"misinformant"

"misinformants"

"misinformation"

"misinformed"

"misinforming"

"misinforms"

"misinstruct"

"misinstructed"

"misinstructing"

"misinstruction"

"misinstructions"

"misinstructs"

"misintelligence"

"misinterpret"

"misinterpretation"

"misinterpretations"

"misinterpreted"

"misinterpreting"

"misinterprets"

"misjudge"

"misjudged"

"misjudges"

"misjudging"

"misjudgment"

"misjudgments"

"mislabel"

"mislabeled"

"mislabeling"

"mislabelled"

"mislabelling"

"mislabels"

"mislaid"

"mislain"

"mislay"

"mislayer"

"mislayers"

"mislaying"

"mislays"

"mislead"

"misleader"

"misleading"

"misleadingly"

"misleads"

"misled"

"mislies"

"mislike"

"mismanage"

"mismanaged"

"mismanagement"

"mismanager"

"mismanages"

"mismanaging"

"mismark"

"mismarked"

"mismarks"

"mismarriage"

"mismarriages"

"mismatch"

"mismatched"

"mismatches"

"mismatching"

"mismate"

"mismated"

"mismates"

"mismating"

"mismeeting"

"misname"

"misnamed"

"misnames"

"misnaming"

"misnomer"

"misnomers"

"misnumber"

"misnumbered"

"misnumbering"

"misnumbers"

"miso"

"misogamist"

"misogamists"

"misogamy"

"misogynic"

"misogynist"

"misogynistic"

"misogynists"

"misogynous"

"misogyny"

"misos"

"misplace"

"misplaced"

"misplacement"

"misplaces"

"misplacing"

"misplay"

"misplayed"

"misplaying"

"misplays"

"misprint"

"misprinted"

"misprinting"

"misprints"

"misprision"

"misprisions"

"misprize"

"mispronounce"

"mispronounced"

"mispronounces"

"mispronouncing"

"mispronunciation"

"mispronunciations"

"misproportion"

"misproportions"

"mispunctuate"

"misquotation"

"misquotations"

"misquote"

"misquoted"

"misquotes"

"misquoting"

"misread"

"misreading"

"misreads"

"misreport"

"misreported"

"misreporting"

"misreports"

"misrepresent"

"misrepresentation"

"misrepresentations"

"misrepresented"

"misrepresentee"

"misrepresenter"

"misrepresenting"

"misrepresents"

"misrule"

"misruled"

"misrules"

"misruling"

"miss"

"missaid"

"missal"

"missals"

"missed"

"misses"

"misshape"

"misshaped"

"misshapen"

"misshapes"

"misshaping"

"missies"

"missile"

"missilery"

"missiles"

"missilry"

"missing"

"mission"

"missionaries"

"missionary"

"missions"

"missis"

"mississippi"

"mississippian"

"mississippians"

"missive"

"missives"

"missort"

"missorted"

"missorting"

"missorts"

"missouri"

"missourian"

"missourians"

"misspeak"

"misspell"

"misspelled"

"misspelling"

"misspellings"

"misspells"

"misspelt"

"misspend"

"misspending"

"misspends"

"misspent"

"misspoke"

"misstate"

"misstated"

"misstatement"

"misstatements"

"misstates"

"misstating"

"misstep"

"missteps"

"missus"

"missy"

"mist"

"mistakable"

"mistake"

"mistaken"

"mistakenly"

"mistaker"

"mistakers"

"mistakes"

"mistaking"

"mistaught"

"mistbow"

"misted"

"mister"

"misterm"

"mistermed"

"misterming"

"misterms"

"misters"

"mistier"

"mistiest"

"mistily"

"mistime"

"mistimed"

"mistimes"

"mistiming"

"mistiness"

"misting"

"mistitle"

"mistitled"

"mistitles"

"mistitling"

"mistletoe"

"mistletoes"

"mistook"

"mistral"

"mistrals"

"mistranscribed"

"mistranscribing"

"mistranscription"

"mistranslate"

"mistranslated"

"mistranslates"

"mistranslating"

"mistranslation"

"mistreat"

"mistreated"

"mistreating"

"mistreatment"

"mistreats"

"mistress"

"mistresses"

"mistrial"

"mistrials"

"mistrust"

"mistrusted"

"mistrustful"

"mistrustfully"

"mistrustfulness"

"mistrusting"

"mistrustingly"

"mistrusts"

"mists"

"mistune"

"mistuned"

"mistunes"

"mistuning"

"misty"

"mistype"

"mistyped"

"mistypes"

"mistyping"

"mistypings"

"misunderstand"

"misunderstanding"

"misunderstandingly"

"misunderstandings"

"misunderstands"

"misunderstood"

"misusage"

"misuse"

"misused"

"misuser"

"misusers"

"misuses"

"misusing"

"miswording"

"mite"

"miter"

"mitered"

"miterer"

"miterers"

"mitering"

"miters"

"mites"

"mitier"

"mitiest"

"mitigate"

"mitigated"

"mitigates"

"mitigating"

"mitigation"

"mitigative"

"mitigator"

"mitigators"

"mitigatory"

"mitochondria"

"mitochondrion"

"mitoses"

"mitosis"

"mitotic"

"mitral"

"mitre"

"mitred"

"mitres"

"mitring"

"mitt"

"mitten"

"mittens"

"mitts"

"mitzvah"

"mitzvahs"

"mix"

"mixable"

"mixed"

"mixer"

"mixers"

"mixes"

"mixing"

"mixology"

"mixt"

"mixture"

"mixtures"

"mixup"

"mixups"

"mizens"

"mizzen"

"mizzenmast"

"mizzenmasts"

"mizzens"

"mizzle"

"mizzly"

"mkt"

"mktg"

"mmhmm"

"mn"

"mnemic"

"mnemon"

"mnemonic"

"mnemonically"

"mnemonics"

"mo"

"moan"

"moaned"

"moanful"

"moaning"

"moans"

"moas"

"moat"

"moated"

"moating"

"moats"

"mob"

"mobbed"

"mobber"

"mobbers"

"mobbing"

"mobbish"

"mobcap"

"mobcaps"

"mobil"

"mobile"

"Mobile Health Record"

"mobiles"

"mobilia"

"mobilities"

"mobility"

"mobilization"

"mobilizations"

"mobilize"

"mobilized"

"mobilizer"

"mobilizers"

"mobilizes"

"mobilizing"

"mobs"

"mobster"

"mobsters"

"moccasin"

"moccasins"

"mocha"

"mochas"

"mock"

"mockable"

"mocked"

"mocker"

"mockeries"

"mockers"

"mockery"

"mocking"

"mockingbird"

"mockingbirds"

"mockingly"

"mocks"

"mockup"

"mockups"

"mod"

"modal"

"modalities"

"modality"

"modally"

"mode"

"model"

"modeled"

"modeler"

"modelers"

"modeling"

"modelled"

"modeller"

"modellers"

"modelling"

"models"

"modem"

"modems"

"moderate"

"moderated"

"moderately"

"moderateness"

"moderates"

"moderating"

"moderation"

"moderato"

"moderator"

"moderatorial"

"moderators"

"moderatorship"

"moderatos"

"modern"

"moderner"

"modernest"

"modernism"

"modernist"

"modernistic"

"modernists"

"modernity"

"modernization"

"modernize"

"modernized"

"modernizer"

"modernizers"

"modernizes"

"modernizing"

"modernly"

"modernness"

"moderns"

"modes"

"modest"

"modester"

"modestest"

"modesties"

"modestly"

"modesty"

"modi"

"modicum"

"modicums"

"modifiable"

"modifiableness"

"modification"

"modifications"

"modified"

"modifier"

"modifiers"

"modifies"

"modify"

"modifying"

"modish"

"modishly"

"modishness"

"modiste"

"modistes"

"modo"

"mods"

"modula"

"modular"

"modularity"

"modulate"

"modulated"

"modulates"

"modulating"

"modulation"

"modulations"

"modulative"

"modulator"

"modulators"

"modulatory"

"module"

"modules"

"modulo"

"modulus"

"modus"

"mogul"

"moguls"

"mohair"

"mohairs"

"mohammed"

"mohawk"

"mohawks"

"moi"

"moieties"

"moiety"

"moil"

"moiled"

"moiler"

"moilers"

"moiling"

"moils"

"moire"

"moires"

"moist"

"moisten"

"moistened"

"moistener"

"moisteners"

"moistening"

"moistens"

"moister"

"moistest"

"moistful"

"moistly"

"moistness"

"moisture"

"moistureless"

"moistureproof"

"moistures"

"moisturize"

"moisturized"

"moisturizer"

"moisturizers"

"moisturizes"

"moisturizing"

"molar"

"molars"

"molasses"

"molasseses"

"mold"

"moldable"

"moldboard"

"moldboards"

"molded"

"molder"

"moldered"

"moldering"

"molders"

"moldier"

"moldiest"

"moldiness"

"molding"

"moldings"

"molds"

"moldy"

"mole"

"molecular"

"molecularly"

"molecule"

"molecules"

"molehill"

"molehills"

"moles"

"moleskin"

"moleskins"

"molest"

"molestation"

"molestations"

"molested"

"molester"

"molesters"

"molesting"

"molests"

"moliere"

"molies"

"moline"

"molinology"

"moll"

"mollie"

"mollies"

"mollification"

"mollified"

"mollifier"

"mollifiers"

"mollifies"

"mollify"

"mollifying"

"molls"

"mollusc"

"molluscan"

"molluscans"

"molluscs"

"mollusk"

"mollusks"

"molly"

"mollycoddle"

"mollycoddled"

"mollycoddler"

"mollycoddlers"

"mollycoddles"

"mollycoddling"

"moloch"

"molochs"

"molt"

"molted"

"molten"

"moltenly"

"molter"

"molters"

"molting"

"molto"

"molts"

"moly"

"molybdenum"

"molybdic"

"mom"

"moment"

"momentarily"

"momentariness"

"momentary"

"momently"

"momento"

"momentoes"

"momentos"

"momentous"

"momentously"

"momentousness"

"moments"

"momentum"

"momentums"

"momilogy"

"momism"

"momisms"

"momma"

"mommas"

"mommies"

"mommy"

"moms"

"mon"

"monaco"

"monad"

"monadal"

"monadic"

"monadism"

"monadisms"

"monads"

"monarch"

"monarchial"

"monarchic"

"monarchical"

"monarchies"

"monarchism"

"monarchist"

"monarchistic"

"monarchists"

"monarchs"

"monarchy"

"monasterial"

"monasteries"

"monastery"

"monastic"

"monastical"

"monastically"

"monasticism"

"monastics"

"monatomic"

"monaural"

"monaurally"

"monaxonic"

"monday"

"mondays"

"monde"

"mondo"

"mondos"

"monetarily"

"monetarism"

"monetarist"

"monetarists"

"monetary"

"monetize"

"monetized"

"monetizes"

"monetizing"

"money"

"moneybag"

"moneybags"

"moneychanger"

"moneychangers"

"moneyed"

"moneyer"

"moneyers"

"moneylender"

"moneylenders"

"moneymaker"

"moneymakers"

"moneymaking"

"moneys"

"mongeese"

"monger"

"mongering"

"mongers"

"mongol"

"mongolia"

"mongolian"

"mongolianism"

"mongolians"

"mongolism"

"mongoloid"

"mongoloids"

"mongols"

"mongoose"

"mongooses"

"mongrel"

"mongrels"

"mongst"

"monicker"

"monickers"

"monied"

"monies"

"moniker"

"monikers"

"monish"

"monism"

"monisms"

"monist"

"monistic"

"monistical"

"monists"

"monition"

"monitions"

"monitor"

"monitored"

"monitories"

"monitoring"

"monitoring fluids"

"monitors"

"monitory"

"monk"

"monkeries"

"monkery"

"monkey"

"monkeyed"

"monkeying"

"monkeys"

"monkeyshine"

"monkeyshines"

"monkhood"

"monkhoods"

"monkish"

"monkishly"

"monkishness"

"monks"

"monkshood"

"monkshoods"

"mono"

"monocellular"

"monochromatic"

"monochromatically"

"monochromaticity"

"monochrome"

"monochromes"

"monocle"

"monocled"

"monocles"

"monocot"

"monocots"

"monocotyledon"

"monocotyledonous"

"monocotyledons"

"monocrat"

"monocular"

"monocularly"

"monocyte"

"monocytes"

"monodic"

"monodies"

"monodist"

"monodists"

"monody"

"monofilament"

"monofuels"

"monogamic"

"monogamies"

"monogamist"

"monogamistic"

"monogamists"

"monogamous"

"monogamously"

"monogamousness"

"monogamy"

"monogram"

"monogramed"

"monogrammed"

"monogramming"

"monograms"

"monograph"

"monographer"

"monographers"

"monographic"

"monographs"

"monogyny"

"monolingual"

"monolith"

"monolithic"

"monoliths"

"monolog"

"monologist"

"monologists"

"monologs"

"monologue"

"monologues"

"monologuist"

"monologuists"

"monology"

"monomania"

"monomaniac"

"monomaniacal"

"monomaniacs"

"monomanias"

"monomer"

"monomeric"

"monomers"

"monomial"

"monomials"

"monomolecular"

"monomolecularly"

"mononucleoses"

"mononucleosis"

"monophobia"

"monophonic"

"monophonically"

"monoplane"

"monoplanes"

"monoploid"

"monopole"

"monopoles"

"monopolies"

"monopolism"

"monopolist"

"monopolistic"

"monopolistically"

"monopolists"

"monopolization"

"monopolize"

"monopolized"

"monopolizer"

"monopolizes"

"monopolizing"

"monopoly"

"monorail"

"monorails"

"monos"

"monosaccharide"

"monosexualities"

"monosexuality"

"monosodium"

"monosyllabic"

"monosyllabically"

"monosyllable"

"monosyllables"

"monotheism"

"monotheist"

"monotheistic"

"monotheists"

"monotone"

"monotones"

"monotonies"

"monotonous"

"monotonously"

"monotonousness"

"monotony"

"monotremata"

"monotreme"

"monoxide"

"monoxides"

"monozygotic"

"monroe"

"mons"

"monseigneur"

"monsieur"

"monsieurs"

"monsignor"

"monsignori"

"monsignors"

"monsoon"

"monsoonal"

"monsoons"

"monster"

"monsters"

"monstrance"

"monstrances"

"monstrosities"

"monstrosity"

"monstrous"

"monstrously"

"monstrousness"

"montage"

"montaged"

"montages"

"montaging"

"montana"

"montanan"

"montanans"

"montane"

"monte"

"monterey"

"montes"

"montessori"

"montevideo"

"montezuma"

"montgomery"

"month"

"monthlies"

"monthly"

"months"

"montpelier"

"montreal"

"monument"

"monumental"

"monumentally"

"monuments"

"mony"

"moo"

"mooch"

"mooched"

"moocher"

"moochers"

"mooches"

"mooching"

"mood"

"moodier"

"moodiest"

"moodily"

"moodiness"

"moods"

"moody"

"mooed"

"mooing"

"moola"

"moolah"

"moolahs"

"moolas"

"moon"

"moonbeam"

"moonbeams"

"moonbow"

"mooncalf"

"mooncalves"

"mooned"

"moonfish"

"moonie"

"moonier"

"mooniest"

"moonily"

"mooning"

"moonish"

"moonless"

"moonlet"

"moonlets"

"moonlight"

"moonlighted"

"moonlighter"

"moonlighters"

"moonlighting"

"moonlights"

"moonlit"

"moonrise"

"moonrises"

"moons"

"moonscape"

"moonscapes"

"moonset"

"moonsets"

"moonshine"

"moonshined"

"moonshiner"

"moonshiners"

"moonshining"

"moonshot"

"moonshots"

"moonstone"

"moonstones"

"moonstruck"

"moonwalk"

"moonwalks"

"moonward"

"moony"

"moor"

"moorage"

"moorages"

"moore"

"moored"

"moorier"

"mooring"

"moorings"

"moorish"

"moorland"

"moorlands"

"moors"

"moory"

"moos"

"moose"

"moot"

"mooted"

"mooter"

"mooters"

"mooting"

"moots"

"mop"

"mope"

"moped"

"mopeder"

"mopeders"

"mopeds"

"moper"

"mopers"

"mopes"

"mopey"

"mopier"

"mopiest"

"moping"

"mopingly"

"mopish"

"mopishly"

"mopped"

"mopper"

"moppers"

"moppet"

"moppets"

"mopping"

"mops"

"mopy"

"mor"

"moraine"

"moraines"

"moral"

"moral judgment"

"morale"

"morales"

"moralism"

"moralisms"

"moralist"

"moralistic"

"moralistically"

"moralists"

"moralities"

"morality"

"moralization"

"moralize"

"moralized"

"moralizer"

"moralizers"

"moralizes"

"moralizing"

"morally"

"morals"

"morass"

"morasses"

"morassy"

"moratoria"

"moratorium"

"moratoriums"

"moray"

"morays"

"morbid"

"morbidities"

"morbidity"

"morbidly"

"morbidness"

"mordacious"

"mordancy"

"mordant"

"mordanted"

"mordanting"

"mordantly"

"mordants"

"mordent"

"mordents"

"more"

"morel"

"morels"

"moreover"

"mores"

"morgan"

"morganatic"

"morgen"

"morgens"

"morgue"

"morgues"

"moribund"

"moribundity"

"moribundly"

"mormon"

"mormonism"

"mormons"

"morn"

"morning"

"mornings"

"morningstar"

"morns"

"moroccan"

"moroccans"

"morocco"

"moroccos"

"moron"

"moronic"

"moronically"

"moronism"

"moronisms"

"moronities"

"morons"

"morose"

"morosely"

"moroseness"

"morph"

"morpheme"

"morphemes"

"morphemic"

"morphia"

"morphic"

"morphin"

"morphine"

"morphines"

"morphinic"

"morphinomania"

"morpho"

"morphogeneses"

"morphogenesis"

"morphogenetic"

"morphogenic"

"morphologic"

"morphological"

"morphologically"

"morphologies"

"morphologist"

"morphologists"

"morphology"

"morphos"

"morphs"

"morris"

"morrow"

"morrows"

"morse"

"morsel"

"morseling"

"morselled"

"morsels"

"mort"

"mortal"

"mortalities"

"mortality"

"mortally"

"mortals"

"mortar"

"mortarboard"

"mortarboards"

"mortared"

"mortaring"

"mortarless"

"mortars"

"mortary"

"mortem"

"mortgage"

"mortgageable"

"mortgaged"

"mortgagee"

"mortgagees"

"mortgager"

"mortgagers"

"mortgages"

"mortgaging"

"mortgagor"

"mortgagors"

"mortice"

"mortician"

"morticians"

"mortification"

"mortifications"

"mortified"

"mortifies"

"mortify"

"mortifying"

"mortifyingly"

"mortis"

"mortise"

"mortised"

"mortiser"

"mortisers"

"mortises"

"mortising"

"mortuaries"

"mortuary"

"mosaic"

"mosaicism"

"mosaics"

"moscow"

"moses"

"mosey"

"moseyed"

"moseying"

"moseys"

"mosks"

"moslem"

"moslems"

"mosque"

"mosques"

"mosquito"

"mosquitoes"

"mosquitos"

"moss"

"mossback"

"mossbacks"

"mossed"

"mosser"

"mossers"

"mosses"

"mossier"

"mossiest"

"mossiness"

"mossy"

"most"

"mostly"

"mosts"

"mot"

"mote"

"motel"

"motels"

"motes"

"motet"

"motets"

"motey"

"moth"

"mothball"

"mothballed"

"mothballs"

"mother"

"mother-to-be"

"motherÂ´s food"

"motherboard"

"mothered"

"motherhood"

"mothering"

"motherland"

"motherlands"

"motherless"

"motherliness"

"motherly"

"mothers"

"mothery"

"mothier"

"mothproof"

"moths"

"mothy"

"motif"

"motifs"

"motile"

"motiles"

"motilities"

"motility"

"motion"

"motional"

"motioned"

"motioner"

"motioners"

"motioning"

"motionless"

"motionlessly"

"motionlessness"

"motions"

"motivate"

"motivated"

"motivates"

"motivating"

"motivation"

"motivational"

"motivationally"

"motivations"

"motivator"

"motive"

"motived"

"motiveless"

"motives"

"motivic"

"motivities"

"motley"

"motleyer"

"motleyest"

"motleys"

"motlier"

"motliest"

"motor"

"motorbike"

"motorbikes"

"motorboat"

"motorboats"

"motorbus"

"motorbuses"

"motorcade"

"motorcades"

"motorcar"

"motorcars"

"motorcycle"

"motorcycles"

"motorcyclist"

"motorcyclists"

"motordrome"

"motored"

"motoric"

"motoring"

"motorings"

"motorist"

"motorists"

"motorization"

"motorize"

"motorized"

"motorizes"

"motorizing"

"motorman"

"motormen"

"motors"

"motorscooters"

"motorship"

"motorships"

"motortruck"

"motortrucks"

"motorway"

"motorways"

"mots"

"mottle"

"mottled"

"mottler"

"mottlers"

"mottles"

"mottling"

"motto"

"mottoes"

"mottos"

"moue"

"moues"

"moujik"

"mould"

"moulded"

"moulder"

"mouldered"

"mouldering"

"moulders"

"mouldier"

"mouldiest"

"moulding"

"mouldings"

"moulds"

"mouldy"

"moulin"

"moulins"

"moult"

"moulted"

"moulter"

"moulters"

"moulting"

"moults"

"mound"

"mounded"

"mounding"

"mounds"

"mount"

"mountable"

"mountain"

"mountain spring"

"mountaineer"

"mountaineered"

"mountaineering"

"mountaineers"

"mountainous"

"mountains"

"mountainside"

"mountainsides"

"mountaintop"

"mountaintops"

"mountebank"

"mountebankeries"

"mountebankery"

"mountebanks"

"mounted"

"mounter"

"mounters"

"mountie"

"mounties"

"mounting"

"mountings"

"mounts"

"mourn"

"mourned"

"mourner"

"mourners"

"mournful"

"mournfully"

"mournfulness"

"mourning"

"mournings"

"mourns"

"mouse"

"moused"

"mouser"

"mousers"

"mouses"

"mousetrap"

"mousetraps"

"mousey"

"mousier"

"mousiest"

"mousily"

"mousiness"

"mousing"

"mousings"

"moussaka"

"moussakas"

"mousse"

"mousses"

"moustache"

"moustaches"

"mousy"

"mouth"

"mouthed"

"mouther"

"mouthers"

"mouthful"

"mouthfuls"

"mouthier"

"mouthiest"

"mouthily"

"mouthing"

"mouthpart"

"mouthparts"

"mouthpiece"

"mouthpieces"

"mouths"

"mouthwash"

"mouthwashes"

"mouthy"

"mouton"

"movability"

"movable"

"movableness"

"movables"

"movably"

"move"

"moveability"

"moveable"

"moveables"

"moveably"

"moved"

"moveless"

"movement"

"movements"

"mover"

"movers"

"moves"

"movie"

"moviedom"

"movies"

"moving"

"movingly"

"mow"

"mowed"

"mower"

"mowers"

"mowing"

"mown"

"mows"

"moxa"

"moxas"

"moxibustion"

"moxie"

"moxies"

"mozambique"

"mozart"

"Mozart's piano sonatas"

"mozzarella"

"mpg"

"mph"

"mr"

"ms"

"MS consult"

"msec"

"msg"

"mss"

"much"

"muches"

"muchness"

"mucilage"

"mucilages"

"mucilaginous"

"mucilaginously"

"muck"

"mucked"

"mucker"

"muckers"

"muckier"

"muckiest"

"muckily"

"mucking"

"muckles"

"muckluck"

"mucklucks"

"muckrake"

"muckraked"

"muckraker"

"muckrakers"

"muckrakes"

"muckraking"

"mucks"

"muckworms"

"mucky"

"mucosity"

"mucous"

"mucus"

"mucuses"

"mud"

"mudcap"

"mudcapped"

"mudcapping"

"mudcaps"

"mudded"

"mudder"

"mudders"

"muddied"

"muddier"

"muddies"

"muddiest"

"muddily"

"muddiness"

"mudding"

"muddle"

"muddled"

"muddleheaded"

"muddler"

"muddlers"

"muddles"

"muddling"

"muddy"

"muddying"

"mudfish"

"mudfishes"

"mudguard"

"mudguards"

"mudlark"

"mudlarks"

"mudpuppies"

"mudra"

"mudras"

"mudrocks"

"muds"

"mudsill"

"mudsills"

"mudslinger"

"mudslingers"

"mudslinging"

"mudstones"

"muenster"

"muensters"

"muezzin"

"muezzins"

"muff"

"muffed"

"muffin"

"muffing"

"muffins"

"muffle"

"muffled"

"muffler"

"mufflers"

"muffles"

"muffling"

"muffs"

"mufti"

"muftis"

"mug"

"mugged"

"mugger"

"muggered"

"muggering"

"muggers"

"muggier"

"muggiest"

"muggily"

"mugginess"

"mugging"

"muggings"

"muggins"

"muggs"

"muggy"

"mugs"

"mugwort"

"mugworts"

"mugwump"

"mugwumps"

"mujik"

"mukluk"

"mukluks"

"mulatto"

"mulattoes"

"mulattos"

"mulberries"

"mulberry"

"mulch"

"mulched"

"mulches"

"mulching"

"mulct"

"mulcted"

"mulcting"

"mulcts"

"mule"

"muled"

"mules"

"muleteer"

"muleteers"

"muley"

"muleys"

"mulier"

"muling"

"mulish"

"mulishly"

"mulishness"

"mull"

"mulla"

"mullah"

"mullahs"

"mulled"

"mullein"

"mulleins"

"mullen"

"mullens"

"muller"

"mullers"

"mullet"

"mullets"

"mulligan"

"mulligans"

"mulligatawny"

"mulling"

"mullion"

"mullioned"

"mullioning"

"mullions"

"mulls"

"multi"

"multi-choice"

"multicellular"

"multicellularity"

"multichannel"

"multicolored"

"multidimensional"

"multidirectional"

"multiengined"

"multiethnic"

"multifaced"

"multifaceted"

"multifactorial"

"multifamily"

"multifarious"

"multifariously"

"multifariousness"

"multiform"

"multifunction"

"multiinfection"

"multijet"

"multilateral"

"multilaterally"

"multilayer"

"multilayered"

"multilevel"

"multilineal"

"multilingual"

"multilingualization"

"multilingualizes"

"multimedia"

"multimillion"

"multimillionaire"

"multimillionaires"

"multimolecular"

"multimotored"

"multinational"

"multinationals"

"multipartite"

"multiparty"

"multipeds"

"multiphasic"

"multiple"

"multiple choice"

"Multiple Sclerosis"

"multiple-choice"

"multiples"

"multiplex"

"multiplexed"

"multiplexer"

"multiplexing"

"multiplicand"

"multiplicands"

"multiplication"

"multiplicational"

"multiplications"

"multiplicities"

"multiplicity"

"multiplied"

"multiplier"

"multipliers"

"multiplies"

"multiply"

"multiplying"

"multipolar"

"multipurpose"

"multiracial"

"multiradial"

"multistage"

"multistory"

"multitasking"

"multitude"

"multitudes"

"multitudinous"

"multitudinously"

"multivalence"

"multivalent"

"multivariate"

"multivariates"

"multiversities"

"multiversity"

"multivitamin"

"multivitamins"

"multo"

"mum"

"mumble"

"mumbled"

"mumbler"

"mumblers"

"mumbles"

"mumbletypeg"

"mumbling"

"mumbo"

"mumm"

"mummed"

"mummer"

"mummeries"

"mummers"

"mummery"

"mummied"

"mummies"

"mummification"

"mummified"

"mummifies"

"mummify"

"mummifying"

"mumming"

"mumms"

"mummy"

"mummying"

"mump"

"mumped"

"mumper"

"mumps"

"mums"

"munch"

"munched"

"muncher"

"munchers"

"munches"

"munchies"

"munching"

"munchy"

"mundane"

"mundanely"

"mungoose"

"munich"

"municipal"

"municipalities"

"municipality"

"municipally"

"munificence"

"munificent"

"munificently"

"munition"

"munitioned"

"munitions"

"munster"

"muon"

"muonic"

"muons"

"mural"

"muralist"

"muralists"

"murals"

"murder"

"murdered"

"murderee"

"murderees"

"murderer"

"murderers"

"murderess"

"murderesses"

"murdering"

"murderous"

"murderously"

"murderousness"

"murders"

"murex"

"murexes"

"muriate"

"muriatic"

"murine"

"murines"

"muring"

"muriphobia"

"murk"

"murker"

"murkest"

"murkier"

"murkiest"

"murkily"

"murkiness"

"murkly"

"murks"

"murky"

"murmur"

"murmured"

"murmurer"

"murmurers"

"murmuring"

"murmurous"

"murmurs"

"murphies"

"murphy"

"murrain"

"murrains"

"murther"

"murthered"

"muscat"

"muscatel"

"muscatels"

"muscats"

"muscel tension"

"muscle"

"muscle highlighting tool"

"muscle tension"

"musclebound"

"muscled"

"musclemen"

"muscles"

"muscling"

"muscly"

"muscology"

"muscovite"

"muscovites"

"muscular"

"muscular tension"

"muscularities"

"muscularity"

"muscularly"

"musculation"

"musculature"

"musculatures"

"musculoskeletal"

"Musculoskeletal System"

"muse"

"mused"

"museful"

"museology"

"muser"

"musers"

"muses"

"musette"

"musettes"

"museum"

"museums"

"mush"

"mushed"

"musher"

"mushers"

"mushes"

"mushier"

"mushiest"

"mushily"

"mushiness"

"mushing"

"mushroom"

"mushroomed"

"mushrooming"

"mushrooms"

"mushy"

"music"

"music pieces"

"music player"

"Music Therapy"

"music tracks"

"musical"

"musicale"

"musicales"

"musically"

"musicals"

"musician"

"musicianly"

"musicians"

"musicianship"

"musicological"

"musicologist"

"musicologists"

"musicology"

"musicotherapies"

"musicotherapy"

"musics"

"musing"

"musingly"

"musings"

"musk"

"muskeg"

"muskegs"

"muskellunge"

"musket"

"musketeer"

"musketeers"

"musketries"

"musketry"

"muskets"

"muskie"

"muskier"

"muskies"

"muskiest"

"muskily"

"muskiness"

"muskits"

"muskmelon"

"muskmelons"

"muskrat"

"muskrats"

"musks"

"musky"

"muslim"

"muslims"

"muslin"

"muslins"

"musomania"

"muss"

"mussed"

"mussel"

"mussels"

"musses"

"mussier"

"mussiest"

"mussily"

"mussiness"

"mussing"

"mussolini"

"mussy"

"must"

"mustache"

"mustached"

"mustaches"

"mustachio"

"mustachioed"

"mustang"

"mustangs"

"mustard"

"mustards"

"musted"

"muster"

"mustered"

"mustering"

"musters"

"mustier"

"mustiest"

"mustily"

"mustiness"

"musting"

"musts"

"musty"

"mutability"

"mutable"

"mutably"

"mutagen"

"mutagenesis"

"mutagenic"

"mutagenically"

"mutagenicities"

"mutagenicity"

"mutagens"

"mutandis"

"mutant"

"mutants"

"mutate"

"mutated"

"mutates"

"mutating"

"mutation"

"mutational"

"mutationally"

"mutations"

"mutative"

"mutator"

"mutchkin"

"mute"

"muted"

"mutedly"

"mutely"

"muteness"

"muter"

"mutes"

"mutest"

"mutilate"

"mutilated"

"mutilates"

"mutilating"

"mutilation"

"mutilations"

"mutilative"

"mutilator"

"mutilators"

"mutineer"

"mutineered"

"mutineers"

"muting"

"mutinied"

"mutinies"

"mutining"

"mutinous"

"mutinously"

"mutinousness"

"mutiny"

"mutinying"

"mutism"

"mutt"

"mutter"

"muttered"

"mutterer"

"mutterers"

"muttering"

"mutters"

"mutton"

"muttonchops"

"muttons"

"muttony"

"mutts"

"mutual"

"Mutual translation"

"mutualism"

"mutualist"

"mutualities"

"mutuality"

"mutualization"

"mutually"

"mutuals"

"mutuel"

"mutuels"

"muumuu"

"muumuus"

"mux"

"muzhik"

"muzhiks"

"muzjiks"

"muzzier"

"muzziest"

"muzzily"

"muzzle"

"muzzled"

"muzzler"

"muzzlers"

"muzzles"

"muzzling"

"muzzy"

"mw"

"my"

"my health"

"myasthenia"

"myasthenic"

"mycelial"

"mycelium"

"mycobacterium"

"mycological"

"mycologist"

"mycologists"

"mycology"

"mycotoxic"

"mycotoxin"

"myelitis"

"myeloma"

"mylar"

"myna"

"mynah"

"mynahs"

"mynas"

"mynheer"

"mynheers"

"myocardia"

"myocardial"

"myograph"

"myology"

"myope"

"myopes"

"myophobia"

"myopia"

"myopias"

"myopic"

"myopically"

"myopy"

"myosin"

"myriad"

"myriads"

"myriapod"

"myriapods"

"myringoscope"

"myrmecology"

"myrmidon"

"myrmidons"

"myrrh"

"myrrhic"

"myrrhs"

"myrtle"

"myrtles"

"myself"

"mysophobia"

"mysteries"

"mysterious"

"mysteriously"

"mysteriousness"

"mystery"

"mystic"

"mystical"

"mystically"

"mysticism"

"mysticisms"

"mysticly"

"mystics"

"mystification"

"mystifications"

"mystified"

"mystifier"

"mystifiers"

"mystifies"

"mystify"

"mystifying"

"mystifyingly"

"mystique"

"mystiques"

"myth"

"mythic"

"mythical"

"mythologic"

"mythological"

"mythologically"

"mythologies"

"mythologist"

"mythologists"

"mythology"

"mythomania"

"mythos"

"myths"

"na"

"nab"

"nabbed"

"nabbing"

"nabob"

"nabobery"

"nabobism"

"nabobisms"

"nabobs"

"nabs"

"nacelle"

"nacelles"

"nacre"

"nacred"

"nacreous"

"nacres"

"nadir"

"nadirs"

"nae"

"nag"

"nagasaki"

"nagged"

"nagger"

"naggers"

"nagging"

"nags"

"nahuatl"

"nahuatls"

"naiad"

"naiades"

"naiads"

"naif"

"naifs"

"nail"

"nailed"

"nailer"

"nailers"

"nailhead"

"nailheads"

"nailing"

"nails"

"nailset"

"nailsets"

"nainsook"

"nairobi"

"naive"

"naively"

"naiveness"

"naivest"

"naivete"

"naivetes"

"naiveties"

"naivety"

"naked"

"nakeder"

"nakedest"

"nakedly"

"nakedness"

"nam"

"namable"

"name"

"nameable"

"named"

"nameless"

"namelessly"

"namely"

"nameplate"

"nameplates"

"namer"

"namers"

"names"

"namesake"

"namesakes"

"naming"

"nan"

"nance"

"nances"

"nancy"

"nankeen"

"nankeens"

"nanking"

"nankins"

"nannie"

"nannies"

"nanny"

"nanograms"

"nanosecond"

"nanoseconds"

"nanowatt"

"nanowatts"

"naology"

"nap"

"napalm"

"napalmed"

"napalming"

"napalms"

"nape"

"naperies"

"napery"

"napes"

"naphtha"

"naphthalene"

"naphthas"

"naphthols"

"naphthous"

"napkin"

"napkins"

"naples"

"napless"

"napoleon"

"napoleonic"

"napoleons"

"napped"

"napper"

"nappers"

"nappes"

"nappie"

"nappier"

"nappies"

"napping"

"nappy"

"naps"

"narc"

"narcissi"

"narcissism"

"narcissist"

"narcissistic"

"narcissistically"

"narcissists"

"narcissus"

"narcissuses"

"narco"

"narcohypnoses"

"narcohypnosis"

"narcolepsies"

"narcolepsy"

"narcoleptic"

"narcomania"

"narcomata"

"narcos"

"narcosis"

"narcotherapies"

"narcotherapy"

"narcotic"

"narcotically"

"narcotics"

"narcotine"

"narcotism"

"narcotization"

"narcotize"

"narcotized"

"narcotizes"

"narcotizing"

"narcs"

"nard"

"nares"

"naris"

"nark"

"narked"

"narking"

"narks"

"narrate"

"narrated"

"narrater"

"narraters"

"narrates"

"narrating"

"narration"

"narrations"

"narrative"

"narratives"

"narrator"

"narrators"

"narrow"

"narrowed"

"narrower"

"narrowest"

"narrowing"

"narrowish"

"narrowly"

"narrowness"

"narrows"

"narthex"

"narthexes"

"narwal"

"narwals"

"narwhal"

"narwhales"

"narwhals"

"nary"

"nasa"

"nasal"

"nasalise"

"nasalities"

"nasality"

"nasalization"

"nasalize"

"nasalized"

"nasalizes"

"nasalizing"

"nasally"

"nasals"

"nascence"

"nascences"

"nascencies"

"nascency"

"nascent"

"nashville"

"nasology"

"nasoscope"

"nastier"

"nastiest"

"nastily"

"nastiness"

"nasturtium"

"nasturtiums"

"nasty"

"natal"

"natalities"

"natality"

"natally"

"natant"

"natantly"

"natation"

"natatory"

"nates"

"nathless"

"nation"

"national"

"nationalism"

"nationalist"

"nationalistic"

"nationalistically"

"nationalists"

"nationalities"

"nationality"

"nationalization"

"nationalizations"

"nationalize"

"nationalized"

"nationalizes"

"nationalizing"

"nationally"

"nationals"

"nationhood"

"nationless"

"nations"

"nationwide"

"native"

"natively"

"natives"

"nativism"

"nativisms"

"nativist"

"nativists"

"nativities"

"nativity"

"natl"

"nato"

"natriums"

"natron"

"natrons"

"natter"

"nattered"

"nattering"

"natters"

"nattier"

"nattiest"

"nattily"

"nattiness"

"natty"

"natural"

"Natural contraception"

"natural cure"

"natural method"

"natural sounds"

"natural workflow"

"naturalism"

"naturalist"

"naturalistic"

"naturalists"

"naturalization"

"naturalizations"

"naturalize"

"naturalized"

"naturalizes"

"naturalizing"

"naturally"

"naturalness"

"naturals"

"nature"

"Nature and Melodic Tunes"

"natured"

"naturedly"

"naturel"

"natureopathy"

"natures"

"naturopathic"

"naturopathy"

"naugahyde"

"naught"

"naughtier"

"naughtiest"

"naughtily"

"naughtiness"

"naughts"

"naughty"

"nausea"

"nauseam"

"nauseants"

"nauseas"

"nauseate"

"nauseated"

"nauseates"

"nauseating"

"nauseatingly"

"nauseation"

"nauseous"

"nauseously"

"nauseousness"

"naut"

"nautch"

"nautches"

"nautical"

"nautically"

"nautics"

"nautili"

"nautilus"

"nautiluses"

"navaho"

"navahoes"

"navahos"

"navajo"

"navajos"

"naval"

"nave"

"navel"

"navels"

"naves"

"navies"

"navigability"

"navigable"

"navigably"

"navigate"

"navigated"

"navigates"

"navigating"

"navigation"

"navigational"

"navigator"

"navigators"

"navvies"

"navvy"

"navy"

"nay"

"nays"

"nazareth"

"nazi"

"nazified"

"nazifies"

"nazify"

"nazifying"

"naziism"

"nazis"

"nazism"

"ndian"

"ne"

"neanderthal"

"neanderthals"

"neap"

"neapolitan"

"neapolitans"

"neaps"

"near"

"nearby"

"neared"

"nearer"

"nearest"

"nearing"

"nearliest"

"nearly"

"nearness"

"nears"

"nearsighted"

"nearsightedly"

"nearsightedness"

"neat"

"neaten"

"neatened"

"neatening"

"neatens"

"neater"

"neatest"

"neath"

"neatherd"

"neatherds"

"neatly"

"neatness"

"neats"

"neb"

"nebbish"

"nebbishes"

"nebraska"

"nebraskan"

"nebraskans"

"nebs"

"nebula"

"nebulae"

"nebulaphobia"

"nebular"

"nebulas"

"nebule"

"nebulise"

"nebulize"

"nebulized"

"nebulizer"

"nebulizers"

"nebulizes"

"nebulizing"

"nebulosities"

"nebulosity"

"nebulous"

"nebulously"

"necessaries"

"necessarily"

"necessariness"

"necessary"

"necessitate"

"necessitated"

"necessitates"

"necessitating"

"necessities"

"necessitous"

"necessitously"

"necessity"

"neck"

"neckband"

"neckbands"

"necked"

"neckerchief"

"neckerchiefs"

"neckerchieves"

"necking"

"neckings"

"necklace"

"necklaces"

"neckless"

"neckline"

"necklines"

"necks"

"necktie"

"neckties"

"neckwear"

"neckwears"

"necrologies"

"necrology"

"necromancer"

"necromancers"

"necromancy"

"necromania"

"necrophile"

"necrophilia"

"necrophilic"

"necrophilism"

"necrophilous"

"necrophobia"

"necropolis"

"necropolises"

"necrose"

"necrosis"

"necrotic"

"necrotically"

"necrotize"

"nectar"

"nectarine"

"nectarines"

"nectars"

"nectary"

"nee"

"need"

"needed"

"needer"

"needers"

"needful"

"needfulness"

"needfuls"

"needier"

"neediest"

"needily"

"neediness"

"needing"

"needle"

"needled"

"needlepoint"

"needlepoints"

"needler"

"needlers"

"needles"

"needless"

"needlessly"

"needlessness"

"needlework"

"needleworker"

"needling"

"needlings"

"needs"

"needy"

"nef"

"nefarious"

"nefariously"

"nefariousness"

"negate"

"negated"

"negater"

"negaters"

"negates"

"negating"

"negation"

"negations"

"negative"

"negatived"

"negatively"

"negativeness"

"negatives"

"negativing"

"negativism"

"negativistic"

"negativity"

"negator"

"negators"

"negatrons"

"neglect"

"neglected"

"neglecter"

"neglectful"

"neglectfully"

"neglectfulness"

"neglecting"

"neglector"

"neglects"

"negligee"

"negligees"

"negligence"

"negligent"

"negligently"

"negligible"

"negligibly"

"negotiability"

"negotiable"

"negotiant"

"negotiants"

"negotiate"

"negotiated"

"negotiates"

"negotiating"

"negotiation"

"negotiations"

"negotiator"

"negotiators"

"negotiatory"

"negotiatress"

"negotiatrix"

"negotiatrixes"

"negritude"

"negro"

"negroes"

"negroid"

"negroids"

"negrophobia"

"negus"

"neguses"

"nehemiah"

"nehru"

"neigh"

"neighbor"

"neighbored"

"neighborhood"

"neighborhoods"

"neighboring"

"neighborliness"

"neighborly"

"neighbors"

"neighed"

"neighing"

"neighs"

"neither"

"nelson"

"nelsons"

"nematode"

"nematodes"

"nematology"

"nembutal"

"nemeses"

"nemesis"

"neoclassic"

"neoclassical"

"neoclassically"

"neoclassicism"

"neocolonial"

"neocolonialism"

"neocolonialist"

"neocolonialists"

"neocolonially"

"neodymium"

"neolith"

"neoliths"

"neologic"

"neologies"

"neologism"

"neologisms"

"neology"

"neomorphs"

"neomycin"

"neomycins"

"neon"

"neonatal"

"neonatally"

"neonate"

"neonates"

"neonatology"

"neoned"

"neons"

"neophobia"

"neophobic"

"neophyte"

"neophytes"

"neoplasia"

"neoplasm"

"neoplasms"

"neoplastic"

"neoprene"

"neoprenes"

"neossology"

"neotenies"

"neoteny"

"neoteric"

"neoterics"

"nepal"

"nepalese"

"nepenthe"

"nepenthes"

"neper"

"nephelometer"

"nephew"

"nephews"

"nephograph"

"nephology"

"nephoscope"

"nephrectomy"

"nephrite"

"nephrites"

"nephritic"

"nephritis"

"nephritises"

"nephrology"

"nephron"

"nephrons"

"nepotic"

"nepotism"

"nepotisms"

"nepotist"

"nepotistic"

"nepotistical"

"nepotistically"

"nepotists"

"neptune"

"neptunian"

"neptunium"

"nerd"

"nerds"

"nereid"

"nereides"

"nereids"

"nereis"

"nerts"

"nertz"

"nervate"

"nervation"

"nerve"

"nerve functioning"

"nerved"

"nerveless"

"nervelessly"

"nervelessness"

"nerves"

"nervier"

"nerviest"

"nervily"

"nervine"

"nervines"

"nerviness"

"nerving"

"nervings"

"nervosa"

"nervosities"

"nervosity"

"nervous"

"Nervous System"

"nervously"

"nervousness"

"nervy"

"nescient"

"nescients"

"ness"

"nest"

"nested"

"nester"

"nesters"

"nesting"

"nestings"

"nestle"

"nestled"

"nestler"

"nestlers"

"nestles"

"nestlike"

"nestling"

"nestlings"

"nestor"

"nestors"

"nests"

"net"

"nether"

"netherlands"

"nethermost"

"netless"

"netlike"

"nets"

"netsuke"

"netsukes"

"nettable"

"nettably"

"netted"

"netter"

"nettier"

"netting"

"nettings"

"nettle"

"nettled"

"nettler"

"nettlers"

"nettles"

"nettlesome"

"nettlier"

"nettliest"

"nettling"

"nettly"

"netty"

"network"

"Network Communications"

"networked"

"networking"

"networks"

"neural"

"neuralgia"

"neuralgias"

"neuralgic"

"neurally"

"neurasthenia"

"neurasthenias"

"neurasthenic"

"neurasthenically"

"neurasthenics"

"neuritic"

"neuritis"

"neuritises"

"neurobiology"

"neurogram"

"neurological"

"neurologically"

"neurologies"

"neurologist"

"neurologists"

"neurologize"

"neurologized"

"neurology"

"neuromotor"

"neuromuscular"

"neuron"

"neuronal"

"neurone"

"neurones"

"neuronic"

"neurons"

"neuropath"

"neuropathy"

"neurophysiologic"

"neurophysiological"

"neurophysiologically"

"neurophysiology"

"neuropsychiatric"

"neuropsychiatry"

"neuropsychology"

"neuroscience"

"neurosensory"

"neuroses"

"neurosis"

"neurosurgeon"

"neurosurgeries"

"neurosurgery"

"neurosurgical"

"neurotic"

"neurotically"

"neuroticism"

"neurotics"

"neurotoxic"

"neurotoxicity"

"neurotoxin"

"neurotransmitter"

"neurotransmitters"

"neurovascular"

"neurypnology"

"neuter"

"neutered"

"neutering"

"neuters"

"neutral"

"neutralism"

"neutralist"

"neutralistic"

"neutralists"

"neutralities"

"neutrality"

"neutralization"

"neutralizations"

"neutralize"

"neutralized"

"neutralizer"

"neutralizers"

"neutralizes"

"neutralizing"

"neutrally"

"neutrals"

"neutrino"

"neutrinos"

"neutron"

"neutrons"

"neutrophil"

"neutrophils"

"neutrosophy"

"nevada"

"nevadan"

"nevadans"

"nevadians"

"never"

"nevermore"

"nevertheless"

"nevi"

"nevoid"

"nevus"

"new"

"newark"

"newborn"

"newborns"

"newcastle"

"newcomer"

"newcomers"

"newel"

"newels"

"newer"

"newest"

"newfangled"

"newfashioned"

"newfound"

"newfoundland"

"newish"

"newly"

"newlywed"

"newlyweds"

"newmown"

"newness"

"newnesses"

"newport"

"news"

"newsboy"

"newsboys"

"newsbreak"

"newscast"

"newscaster"

"newscasters"

"newscasts"

"newsdealer"

"newsdealers"

"newsgirl"

"newsgirls"

"newsier"

"newsies"

"newsiest"

"newsiness"

"newsletter"

"newsletters"

"newsman"

"newsmen"

"newspaper"

"newspaperman"

"newspapermen"

"newspapers"

"newspaperwoman"

"newspaperwomen"

"newspeak"

"newspeaks"

"newsprint"

"newsreel"

"newsreels"

"newsrooms"

"newsstand"

"newsstands"

"newsweek"

"newswoman"

"newswomen"

"newsworthiness"

"newsworthy"

"newsy"

"newt"

"newton"

"newtonian"

"newtons"

"newts"

"next"

"nextdoor"

"nextly"

"nexus"

"nexuses"

"NFP"

"nglish"

"niacin"

"niacinamide"

"niacins"

"niagara"

"nib"

"nibbed"

"nibble"

"nibbled"

"nibbler"

"nibblers"

"nibbles"

"nibbling"

"niblick"

"niblicks"

"nibs"

"nicaragua"

"nicaraguan"

"nicaraguans"

"nice"

"nicely"

"niceness"

"nicer"

"nicest"

"niceties"

"nicety"

"niche"

"niched"

"niches"

"niching"

"nicholas"

"nick"

"nicked"

"nickel"

"nickeled"

"nickeling"

"nickelled"

"nickelodeon"

"nickelodeons"

"nickels"

"nicker"

"nickered"

"nickering"

"nickers"

"nicking"

"nickle"

"nickles"

"nicknack"

"nicknacks"

"nickname"

"nicknamed"

"nicknames"

"nicknaming"

"nicks"

"nicotine"

"nicotines"

"nicotinic"

"nictate"

"nictated"

"nictates"

"nictating"

"nictation"

"nictitate"

"nictitated"

"nictitates"

"nictitating"

"nictitation"

"nidology"

"niece"

"nieces"

"nielsen"

"nietzsche"

"niftier"

"niftiest"

"nifty"

"nigeria"

"nigerian"

"nigerians"

"niggard"

"niggarded"

"niggarding"

"niggardliness"

"niggardly"

"niggards"

"nigger"

"niggers"

"niggle"

"niggled"

"niggler"

"nigglers"

"niggles"

"niggling"

"nigglingly"

"nigglings"

"nigh"

"nighed"

"nigher"

"nighest"

"nighing"

"nighness"

"nighs"

"night"

"nightcap"

"nightcaps"

"nightclothes"

"nightclub"

"nightclubs"

"nightcrawler"

"nightcrawlers"

"nightdress"

"nighter"

"nighters"

"nightfall"

"nightfalls"

"nightgown"

"nightgowns"

"nighthawk"

"nighthawks"

"nightie"

"nighties"

"nightingale"

"nightingales"

"nightjar"

"nightjars"

"nightlong"

"nightly"

"nightman"

"nightmare"

"nightmares"

"nightmarish"

"nightmen"

"nightrider"

"nightriders"

"nights"

"nightshade"

"nightshades"

"nightshirt"

"nightshirts"

"nightspot"

"nightspots"

"nightstand"

"nightstands"

"nightstick"

"nighttime"

"nighttimes"

"nightwalker"

"nightwalkers"

"nightwear"

"nighty"

"nigritude"

"nihil"

"nihilism"

"nihilisms"

"nihilist"

"nihilistic"

"nihilistically"

"nihilists"

"nihilities"

"nihility"

"nihils"

"nijinsky"

"nil"

"nile"

"nill"

"nilled"

"nilling"

"nills"

"nils"

"nim"

"nimbi"

"nimble"

"nimbleness"

"nimbler"

"nimblest"

"nimbly"

"nimbus"

"nimbused"

"nimbuses"

"nimrods"

"nims"

"nincompoop"

"nincompoops"

"nine"

"ninefold"

"ninepin"

"ninepins"

"nines"

"nineteen"

"nineteens"

"nineteenth"

"nineteenths"

"nineties"

"ninetieth"

"ninetieths"

"ninety"

"ninnies"

"ninny"

"ninnyish"

"ninon"

"ninth"

"ninthly"

"ninths"

"niobium"

"niobiums"

"nip"

"nipped"

"nipper"

"nippers"

"nippier"

"nippiest"

"nippily"

"nipping"

"nipple"

"nipples"

"nippon"

"nipponese"

"nippy"

"nips"

"nirvana"

"nirvanas"

"nirvanic"

"nisei"

"niseis"

"nisi"

"nit"

"nited"

"niter"

"niters"

"nitpick"

"nitpicked"

"nitpicker"

"nitpickers"

"nitpicking"

"nitpicks"

"nitrate"

"nitrated"

"nitrates"

"nitrating"

"nitration"

"nitrators"

"nitre"

"nitres"

"nitric"

"nitride"

"nitrification"

"nitrified"

"nitrifies"

"nitrify"

"nitrifying"

"nitrile"

"nitrite"

"nitrites"

"nitritoid"

"nitro"

"nitrocellulose"

"nitrocellulosic"

"nitrogen"

"nitrogenous"

"nitrogens"

"nitroglycerin"

"nitroglycerine"

"nitrometer"

"nitros"

"nitrous"

"nits"

"nittier"

"nitty"

"nitwit"

"nitwits"

"nix"

"nixed"

"nixes"

"nixie"

"nixies"

"nixing"

"nixon"

"nixy"

"nj"

"nm"

"no"

"no ads"

"No data connection"

"No health insurance"

"No web account"

"noah"

"nob"

"nobbier"

"nobbily"

"nobble"

"nobbled"

"nobbler"

"nobblers"

"nobbles"

"nobbling"

"nobby"

"nobel"

"nobelist"

"nobelists"

"nobelium"

"nobeliums"

"nobilities"

"nobility"

"noble"

"nobleman"

"noblemen"

"nobleness"

"nobler"

"nobles"

"noblesse"

"noblesses"

"noblest"

"noblewoman"

"noblewomen"

"nobly"

"nobodies"

"nobody"

"nobs"

"nock"

"nocked"

"nocking"

"nocks"

"noctambulation"

"noctambulism"

"noctambulist"

"noctambulistic"

"nocturn"

"nocturnal"

"nocturnally"

"nocturne"

"nocturnes"

"nocturns"

"nocuous"

"nod"

"nodal"

"nodally"

"nodded"

"nodder"

"nodders"

"noddies"

"nodding"

"noddle"

"noddles"

"noddy"

"node"

"nodes"

"nods"

"nodular"

"nodule"

"nodules"

"nodus"

"noel"

"noels"

"noes"

"noesis"

"noetic"

"nog"

"noggin"

"noggings"

"noggins"

"noggs"

"nogs"

"nohow"

"noir"

"noire"

"noires"

"noise"

"noised"

"noiseless"

"noiselessly"

"noiselessness"

"noisemaker"

"noisemakers"

"noises"

"noisier"

"noisiest"

"noisily"

"noisiness"

"noising"

"noisome"

"noisomely"

"noisy"

"nolle"

"nolo"

"nom"

"nomad"

"nomadic"

"nomadically"

"nomadism"

"nomadisms"

"nomads"

"nome"

"nomenclature"

"nomenclatures"

"nominal"

"nominally"

"nominals"

"nominate"

"nominated"

"nominately"

"nominates"

"nominating"

"nomination"

"nominations"

"nominative"

"nominatively"

"nominatives"

"nominator"

"nominators"

"nominee"

"nominees"

"nomism"

"nomisms"

"nomogram"

"nomograms"

"nomograph"

"nomography"

"nomology"

"noms"

"non"

"non-insulin-dependent diabetes"

"nonabrasive"

"nonabrasively"

"nonabrasiveness"

"nonabsolute"

"nonabsolutely"

"nonabsoluteness"

"nonabsorbable"

"nonabsorbent"

"nonabsorbents"

"nonabstainer"

"nonabstainers"

"nonacademic"

"nonacademics"

"nonacceptance"

"nonacid"

"nonactive"

"nonactives"

"nonadaptive"

"nonaddicting"

"nonaddictive"

"nonadhesive"

"nonadjacent"

"nonadjustable"

"nonadministrative"

"nonadministratively"

"nonadmission"

"nonadmissions"

"nonadult"

"nonadults"

"nonadvantageous"

"nonadvantageously"

"nonaffiliated"

"nonaffilliated"

"nonage"

"nonagenarian"

"nonagenarians"

"nonages"

"nonaggression"

"nonagon"

"nonagons"

"nonagreement"

"nonagricultural"

"nonalcoholic"

"nonaligned"

"nonalignment"

"nonallergenic"

"nonanalytic"

"nonappearance"

"nonappearances"

"nonapplicable"

"nonaquatic"

"nonassertive"

"nonassertively"

"nonassimilation"

"nonathletic"

"nonattendance"

"nonattributive"

"nonattributively"

"nonauthoritative"

"nonauthoritatively"

"nonautomated"

"nonautomatic"

"nonbasic"

"nonbeing"

"nonbeings"

"nonbeliever"

"nonbelievers"

"nonbelligerent"

"nonbelligerents"

"nonbending"

"nonbreakable"

"noncancellable"

"noncancerous"

"noncarbonated"

"noncarnivorous"

"noncasual"

"noncausal"

"noncausally"

"nonce"

"noncelestial"

"noncellular"

"noncentral"

"noncentrally"

"nonces"

"nonchalance"

"nonchalant"

"nonchalantly"

"nonchargeable"

"noncivilized"

"nonclassical"

"nonclassically"

"nonclerical"

"nonclerically"

"nonclinical"

"nonclinically"

"noncoagulating"

"noncohesive"

"noncohesively"

"noncohesiveness"

"noncollapsable"

"noncollapsible"

"noncollectible"

"noncom"

"noncombat"

"noncombatant"

"noncombatants"

"noncombining"

"noncombustible"

"noncombustibles"

"noncommercial"

"noncommercially"

"noncommissioned"

"noncommittal"

"noncommittally"

"noncommunicable"

"noncommunicative"

"noncommunist"

"noncommunists"

"noncompeting"

"noncompetitive"

"noncompliance"

"noncomplying"

"noncompulsory"

"noncoms"

"nonconciliatory"

"nonconclusive"

"nonconclusively"

"nonconclusiveness"

"nonconcurrence"

"nonconcurrent"

"nonconcurrently"

"nonconducting"

"nonconductive"

"nonconductor"

"nonconductors"

"nonconfidence"

"nonconfidential"

"nonconflicting"

"nonconforming"

"nonconformism"

"nonconformist"

"nonconformists"

"nonconformity"

"noncongealing"

"nonconnective"

"nonconsecutive"

"nonconsecutively"

"nonconsenting"

"nonconstructive"

"nonconstructively"

"nonconsumption"

"noncontagious"

"noncontemporary"

"noncontiguous"

"noncontiguously"

"noncontinuance"

"noncontinuation"

"noncontinuous"

"noncontraband"

"noncontrabands"

"noncontradictory"

"noncontrastable"

"noncontributing"

"noncontributory"

"noncontrollable"

"noncontrollably"

"noncontroversial"

"noncontroversially"

"nonconventional"

"nonconvergent"

"nonconversant"

"nonconvertible"

"noncooperation"

"noncooperative"

"noncorroborative"

"noncorroding"

"noncorrosive"

"noncreative"

"noncriminal"

"noncritical"

"noncrystalline"

"noncumulative"

"noncyclical"

"nondairy"

"nondeductible"

"nondeliveries"

"nondelivery"

"nondemocratic"

"nondemonstrable"

"nondenominational"

"nondepartmental"

"nondependence"

"nondescript"

"nondescriptive"

"nondestructive"

"nondestructively"

"nondestructiveness"

"nondetachable"

"nondevelopment"

"nondifferentiation"

"nondiplomatic"

"nondirectional"

"nondisciplinary"

"nondisclosure"

"nondiscriminating"

"nondiscrimination"

"nondiscriminatory"

"nondistribution"

"nondivisible"

"nondomesticated"

"nondramatic"

"nondrinker"

"nondrinkers"

"nondrying"

"none"

"noneducable"

"noneducational"

"noneffective"

"noneffervescent"

"noneffervescently"

"nonego"

"nonegos"

"nonelastic"

"nonelection"

"nonelective"

"nonelectric"

"nonelectrically"

"noneligible"

"nonemotional"

"nonemotionally"

"nonempirical"

"nonempirically"

"nonempty"

"nonenforceable"

"nonenforcement"

"nonentities"

"nonentity"

"nonequal"

"nonequals"

"nonequivalent"

"nonequivalents"

"nones"

"nonessential"

"nonessentials"

"nonesuch"

"nonesuches"

"nonetheless"

"nonethical"

"nonethically"

"nonethicalness"

"nonevent"

"nonevents"

"nonexchangeable"

"nonexclusive"

"nonexempt"

"nonexistence"

"nonexistent"

"nonexisting"

"nonexpendable"

"nonexplosive"

"nonexplosives"

"nonexportable"

"nonextant"

"nonextraditable"

"nonfactual"

"nonfactually"

"nonfascist"

"nonfascists"

"nonfat"

"nonfatal"

"nonfatally"

"nonfederal"

"nonfederated"

"nonferrous"

"nonfiction"

"nonfictional"

"nonfilterable"

"nonflammable"

"nonflexible"

"nonflowering"

"nonfood"

"nonforfeitable"

"nonforfeiture"

"nonforfeitures"

"nonformation"

"nonfreezing"

"nonfulfillment"

"nonfunctional"

"nongaseous"

"nongovernmental"

"nongregarious"

"nonhabitable"

"nonhabitual"

"nonhabituating"

"nonhazardous"

"nonhereditary"

"nonhero"

"nonheroes"

"nonhistoric"

"nonhomogeneous"

"nonhuman"

"nonidentical"

"nonidentities"

"nonidentity"

"nonideological"

"nonidiomatic"

"nonimmunities"

"nonimmunity"

"noninclusive"

"nonincriminating"

"nonindependent"

"noninductive"

"nonindulgence"

"nonindustrial"

"noninfectious"

"noninflammable"

"noninflammatory"

"noninflected"

"noninflectional"

"noninformative"

"noninformatively"

"noninhabitable"

"noninheritable"

"noninjurious"

"noninjuriously"

"noninjuriousness"

"noninstinctive"

"noninstinctual"

"noninstitutional"

"nonintellectual"

"nonintellectually"

"nonintellectuals"

"noninterchangeable"

"noninterfaced"

"noninterference"

"nonintersecting"

"nonintervention"

"noninterventional"

"noninterventionist"

"noninterventionists"

"nonintoxicant"

"nonintoxicants"

"nonintoxicating"

"nonirritant"

"nonirritating"

"nonjudicial"

"nonketotic hyperosmolar coma"

"nonkosher"

"nonlegal"

"nonlethal"

"nonlife"

"nonlinear"

"nonliterary"

"nonliturgical"

"nonliturgically"

"nonliving"

"nonlocals"

"nonlogical"

"nonluminous"

"nonmagnetic"

"nonmalicious"

"nonmaliciously"

"nonmalignant"

"nonman"

"nonmaterial"

"nonmaterialistic"

"nonmathematical"

"nonmeasurable"

"nonmechanical"

"nonmechanically"

"nonmechanistic"

"nonmember"

"nonmembers"

"nonmembership"

"nonmen"

"nonmetal"

"nonmetallic"

"nonmetals"

"nonmigratory"

"nonmilitant"

"nonmilitantly"

"nonmilitants"

"nonmilitarily"

"nonmilitary"

"nonmoral"

"nonmotile"

"nonmystical"

"nonmystically"

"nonmythical"

"nonmythically"

"nonnative"

"nonnatives"

"nonnatural"

"nonnavigable"

"nonnegotiable"

"nonnitrogenous"

"nonnumeric"

"nonnutritious"

"nonobedience"

"nonobjective"

"nonobligatory"

"nonobservance"

"nonoccurrence"

"nonodorous"

"nonofficial"

"nonofficially"

"nonoperable"

"nonoperative"

"nonorganic"

"nonorthodox"

"nonowner"

"nonowners"

"nonparallel"

"nonparametric"

"nonparasitic"

"nonpareil"

"nonpareils"

"nonparliamentary"

"nonparticipant"

"nonparticipating"

"nonparticipation"

"nonpartisan"

"nonpartisans"

"nonpasserine"

"nonpaying"

"nonpayment"

"nonperformance"

"nonperishable"

"nonperishables"

"nonpermanent"

"nonpermeable"

"nonphysical"

"nonphysically"

"nonphysiological"

"nonphysiologically"

"nonpigmented"

"nonplus"

"nonplused"

"nonpluses"

"nonplusing"

"nonplussed"

"nonplusses"

"nonplussing"

"nonpoetic"

"nonpoisonous"

"nonpolitical"

"nonpolitically"

"nonporous"

"nonpossession"

"nonpossessive"

"nonpossessively"

"nonpossessiveness"

"nonprecious"

"nonpredatory"

"nonpredictable"

"nonprejudicial"

"nonprejudicially"

"nonprescriptive"

"nonpreservable"

"nonprocedural"

"nonproduction"

"nonproductive"

"nonprofessional"

"nonprofit"

"nonprofitable"

"nonproliferation"

"nonproportional"

"nonproportionally"

"nonproprietaries"

"nonproprietary"

"nonprotective"

"nonprotectively"

"nonproven"

"nonpunishable"

"nonracial"

"nonradical"

"nonradioactive"

"nonrational"

"nonrationally"

"nonreactive"

"nonreader"

"nonreaders"

"nonrealistic"

"nonreciprocal"

"nonreciprocally"

"nonreciprocals"

"nonrecognition"

"nonrecoverable"

"nonrecurrent"

"nonrecurring"

"nonredeemable"

"nonrefillable"

"nonreflective"

"nonregimented"

"nonregistered"

"nonrelational"

"nonreligious"

"nonremunerative"

"nonrenewable"

"nonrepresentational"

"nonrepresentative"

"nonresidence"

"nonresident"

"nonresidential"

"nonresidents"

"nonresidual"

"nonresistant"

"nonresistants"

"nonrestricted"

"nonrestrictive"

"nonreturnable"

"nonreversible"

"nonrhythmic"

"nonrigid"

"nonsalable"

"nonsalaried"

"nonscheduled"

"nonscholastic"

"nonscientific"

"nonseasonal"

"nonsecret"

"nonsecretly"

"nonsectarian"

"nonsecular"

"nonsegregated"

"nonselective"

"nonsense"

"nonsenses"

"nonsensical"

"nonsensically"

"nonsensitive"

"nonsexist"

"nonsexists"

"nonsexual"

"nonsexually"

"nonsignificant"

"nonsinkable"

"nonsked"

"nonskid"

"nonskilled"

"nonslip"

"nonsmoker"

"nonsmokers"

"nonsmoking"

"nonsocial"

"nonspeaking"

"nonspecialist"

"nonspecialists"

"nonspecialized"

"nonspecific"

"nonspiritual"

"nonsporting"

"nonstable"

"nonstaining"

"nonstandard"

"nonstandardized"

"nonstick"

"nonstimulating"

"nonstop"

"nonstrategic"

"nonstriker"

"nonstrikers"

"nonstriking"

"nonstructural"

"nonstructurally"

"nonsubmissive"

"nonsubmissively"

"nonsubmissiveness"

"nonsubscriber"

"nonsubscribers"

"nonsuccess"

"nonsuccessive"

"nonsuccessively"

"nonsuccessiveness"

"nonsuches"

"nonsupport"

"nonsuppression"

"nonsupression"

"nonsurgical"

"nonsusceptibility"

"nonsusceptible"

"nonsustaining"

"nonsymbolic"

"nonsystematic"

"nontaxable"

"nontechnical"

"nontechnically"

"nontemporal"

"nontemporally"

"nontenure"

"nontheatrical"

"nonthinking"

"nontoxic"

"nontraditional"

"nontraditionally"

"nontransferable"

"nontransparent"

"nontropical"

"nontruths"

"nontypical"

"nontypically"

"nonunified"

"nonuniform"

"nonunion"

"nonunionist"

"nonunions"

"nonunited"

"nonuple"

"nonuser"

"nonusers"

"nonvascular"

"nonvascularly"

"nonvenomous"

"nonverbal"

"nonviable"

"nonviolation"

"nonviolence"

"nonviolent"

"nonviolently"

"nonvirulent"

"nonvisible"

"nonvisual"

"nonvisually"

"nonvocal"

"nonvocational"

"nonvolatile"

"nonvoluntary"

"nonvoter"

"nonvoters"

"nonvoting"

"nonwhite"

"nonwhites"

"nonworker"

"nonworkers"

"nonworking"

"nonyielding"

"nonzebra"

"nonzero"

"noodle"

"noodled"

"noodles"

"noodling"

"nook"

"nookies"

"nooks"

"nooky"

"noology"

"noon"

"noonday"

"noondays"

"nooning"

"noonings"

"noons"

"noontide"

"noontides"

"noontime"

"noontimes"

"noose"

"noosed"

"nooser"

"noosers"

"nooses"

"noosing"

"nope"

"nor"

"nordic"

"norfolk"

"norm"

"norma"

"normal"

"normal meditates states"

"normalacy"

"normalcies"

"normalcy"

"normalities"

"normality"

"normalization"

"normalize"

"normalized"

"normalizer"

"normalizes"

"normalizing"

"normally"

"normals"

"norman"

"normandy"

"normans"

"normative"

"normatively"

"normativeness"

"normed"

"norms"

"norse"

"norseman"

"norsemen"

"north"

"northbound"

"northeast"

"northeaster"

"northeasterly"

"northeastern"

"northeasterner"

"northeasters"

"northeastward"

"northeastwardly"

"northeners"

"norther"

"northerly"

"northern"

"northerner"

"northerners"

"northernmost"

"northerns"

"northers"

"northings"

"norths"

"northward"

"northwardly"

"northwards"

"northwest"

"northwesterly"

"northwestern"

"northwestward"

"northwestwardly"

"norway"

"norwegian"

"norwegians"

"nos"

"nose"

"nosebag"

"nosebags"

"nosebands"

"nosebleed"

"nosebleeds"

"nosed"

"nosedive"

"nosegay"

"nosegays"

"nosepiece"

"noses"

"nosey"

"nosh"

"noshed"

"nosher"

"noshers"

"noshes"

"noshing"

"nosier"

"nosiest"

"nosily"

"nosiness"

"nosing"

"nosings"

"nosology"

"nosomania"

"nosophobia"

"nostalgia"

"nostalgic"

"nostalgically"

"noster"

"nostology"

"nostomania"

"nostril"

"nostrils"

"nostrum"

"nostrums"

"nosy"

"not"

"not approved"

"not clinically validated"

"not need networking"

"nota"

"notabilities"

"notability"

"notable"

"notables"

"notably"

"notal"

"notandum"

"notaphily"

"notarial"

"notarially"

"notaries"

"notarization"

"notarizations"

"notarize"

"notarized"

"notarizes"

"notarizing"

"notary"

"notaryship"

"notate"

"notated"

"notates"

"notating"

"notation"

"notational"

"notations"

"notch"

"notched"

"notcher"

"notchers"

"notches"

"notching"

"notchy"

"note"

"notebook"

"notebooks"

"notecases"

"noted"

"notedly"

"notepad"

"notepads"

"notepaper"

"noter"

"noters"

"notes"

"noteworthily"

"noteworthiness"

"noteworthy"

"nothing"

"nothingness"

"nothings"

"notice"

"noticeable"

"noticeably"

"noticed"

"notices"

"noticing"

"notifiable"

"notification"

"notification system"

"notifications"

"notified"

"notifier"

"notifiers"

"notifies"

"notify"

"notifying"

"noting"

"notion"

"notional"

"notionally"

"notions"

"notochord"

"notochordal"

"notorieties"

"notoriety"

"notorious"

"notoriously"

"notre"

"nots"

"notwithstanding"

"nougat"

"nougats"

"nought"

"noughts"

"noumena"

"noumenal"

"noumenon"

"noun"

"nounal"

"nounally"

"nouns"

"nourish"

"nourished"

"nourisher"

"nourishers"

"nourishes"

"nourishing"

"nourishment"

"nourishments"

"nous"

"nouveau"

"nouveaux"

"nouvelle"

"nova"

"novae"

"novas"

"novel"

"novelette"

"novelettes"

"novelising"

"novelist"

"novelistic"

"novelists"

"novelization"

"novelizations"

"novelize"

"novelized"

"novelizes"

"novelizing"

"novella"

"novellas"

"novelle"

"novelly"

"novels"

"novelties"

"novelty"

"november"

"novena"

"novenae"

"novenas"

"novercaphobia"

"novice"

"novices"

"novitiate"

"novitiates"

"novo"

"novocain"

"novocaine"

"now"

"nowadays"

"noway"

"noways"

"nowhere"

"nowheres"

"nowise"

"nows"

"noxious"

"noxiously"

"noxiousness"

"nozzle"

"nozzles"

"nt"

"nternet"

"nth"

"nu"

"nuance"

"nuanced"

"nuances"

"nub"

"nubbier"

"nubbiest"

"nubbin"

"nubbins"

"nubble"

"nubbles"

"nubblier"

"nubbly"

"nubby"

"nubia"

"nubias"

"nubile"

"nubilities"

"nubility"

"nubs"

"nucleal"

"nuclear"

"nucleate"

"nucleated"

"nucleates"

"nucleating"

"nucleation"

"nucleations"

"nucleator"

"nucleators"

"nuclei"

"nucleic"

"nuclein"

"nucleolar"

"nucleoli"

"nucleolus"

"nucleon"

"nucleonic"

"nucleonics"

"nucleons"

"nucleoplasm"

"nucleoplasmatic"

"nucleoprotein"

"nucleus"

"nucleuses"

"nude"

"nudely"

"nudeness"

"nuder"

"nudes"

"nudest"

"nudge"

"nudged"

"nudger"

"nudgers"

"nudges"

"nudging"

"nudie"

"nudies"

"nudism"

"nudisms"

"nudist"

"nudists"

"nudities"

"nudity"

"nudnick"

"nudnicks"

"nudnik"

"nudniks"

"nudum"

"nugatory"

"nugget"

"nuggets"

"nuggety"

"nuisance"

"nuisances"

"nuke"

"nukes"

"null"

"nulled"

"nullification"

"nullifications"

"nullificator"

"nullified"

"nullifier"

"nullifiers"

"nullifies"

"nullify"

"nullifying"

"nulling"

"nulliparous"

"nullities"

"nullity"

"nullo"

"nulls"

"numb"

"numbed"

"number"

"numberable"

"numbered"

"numberer"

"numberers"

"numbering"

"numberings"

"numberless"

"numbers"

"numbest"

"numbing"

"numbingly"

"numbly"

"numbness"

"numbs"

"numbskull"

"numerable"

"numerably"

"numeral"

"numerals"

"numerary"

"numerate"

"numerated"

"numerates"

"numerating"

"numeration"

"numerations"

"numerator"

"numerators"

"numeric"

"numerical"

"numerically"

"numerics"

"numerologist"

"numerologists"

"numerology"

"numerous"

"numerously"

"numerousness"

"numinous"

"numismatic"

"numismatics"

"numismatist"

"numismatists"

"nummary"

"nummular"

"numskull"

"numskulls"

"nun"

"nuncio"

"nuncios"

"nuncle"

"nuncles"

"nuncupative"

"nunneries"

"nunnery"

"nunnish"

"nunquam"

"nuns"

"nuptial"

"nuptially"

"nuptials"

"nurse"

"nursed"

"nurseling"

"nursemaid"

"nursemaids"

"nurser"

"nurseries"

"nursers"

"nursery"

"nurserymaid"

"nurserymaids"

"nurseryman"

"nurserymen"

"nurses"

"nursing"

"nursing home"

"nursings"

"nursling"

"nurslings"

"nurture"

"nurtured"

"nurturer"

"nurturers"

"nurtures"

"nurturing"

"nut"

"nutations"

"nutcracker"

"nutcrackers"

"nutgrasses"

"nuthatch"

"nuthatches"

"nuthouse"

"nuthouses"

"nutlet"

"nutlets"

"nutlike"

"nutmeat"

"nutmeats"

"nutmeg"

"nutmegs"

"nutpick"

"nutpicks"

"nutria"

"nutrias"

"nutrient"

"nutrients"

"nutriment"

"nutrimental"

"nutriments"

"nutrition"

"Nutrition Section"

"nutritional"

"nutritionally"

"nutritionist"

"nutritionists"

"nutritious"

"nutritiously"

"nutritiousness"

"nutritive"

"nutritively"

"nuts"

"nutshell"

"nutshells"

"nutted"

"nutter"

"nutters"

"nuttier"

"nuttiest"

"nuttily"

"nuttiness"

"nutting"

"nutty"

"nuzzle"

"nuzzled"

"nuzzler"

"nuzzlers"

"nuzzles"

"nuzzling"

"ny"

"nybble"

"nybbles"

"nybblize"

"nyctophobia"

"nylon"

"nylons"

"nymph"

"nymphal"

"nymphet"

"nymphets"

"nympho"

"nympholepsies"

"nympholeptic"

"nymphology"

"nymphomania"

"nymphomaniac"

"nymphomaniacal"

"nymphomaniacs"

"nymphos"

"nymphs"

"nystagmus"

"oaf"

"oafish"

"oafishly"

"oafishness"

"oafs"

"oak"

"oaken"

"oakland"

"oaks"

"oakum"

"oakums"

"oar"

"oared"

"oaring"

"oarless"

"oarlock"

"oarlocks"

"oars"

"oarsman"

"oarsmanship"

"oarsmen"

"oases"

"oasis"

"oasts"

"oat"

"oatcake"

"oatcakes"

"oaten"

"oater"

"oaters"

"oath"

"oaths"

"oatmeal"

"oatmeals"

"oats"

"ob"

"obbligati"

"obbligato"

"obbligatos"

"obduction"

"obduracies"

"obduracy"

"obdurate"

"obdurated"

"obdurately"

"obdurateness"

"obdurating"

"obduration"

"obeah"

"obeahisms"

"obeahs"

"obedience"

"obedient"

"obediential"

"obediently"

"obeisance"

"obeisances"

"obeisant"

"obeli"

"obelisk"

"obelisks"

"obese"

"obesely"

"obesities"

"obesity"

"obey"

"obeyable"

"obeyed"

"obeyer"

"obeyers"

"obeying"

"obeys"

"obfuscable"

"obfuscate"

"obfuscated"

"obfuscates"

"obfuscating"

"obfuscation"

"obfuscator"

"obfuscators"

"obfuscatory"

"obgyn"

"obi"

"obis"

"obit"

"obiter"

"obits"

"obituaries"

"obituary"

"object"

"objectant"

"objected"

"objecting"

"objection"

"objectionability"

"objectionable"

"objectional"

"objections"

"objective"

"objectively"

"objectiveness"

"objectives"

"objectivity"

"objector"

"objectors"

"objects"

"objicient"

"objuration"

"objurgate"

"objurgated"

"objurgates"

"objurgating"

"objurgation"

"objurgations"

"oblate"

"oblately"

"oblates"

"oblation"

"oblational"

"oblations"

"obligability"

"obligable"

"obligate"

"obligated"

"obligates"

"obligating"

"obligation"

"obligational"

"obligations"

"obligato"

"obligator"

"obligatorily"

"obligatory"

"oblige"

"obliged"

"obligee"

"obligement"

"obliger"

"obligers"

"obliges"

"obliging"

"obligingly"

"oblique"

"obliqued"

"obliquely"

"obliqueness"

"obliques"

"obliquities"

"obliquity"

"obliterate"

"obliterated"

"obliterates"

"obliterating"

"obliteration"

"obliterations"

"obliterative"

"obliterator"

"obliterators"

"oblivion"

"oblivions"

"oblivious"

"obliviously"

"obliviousness"

"oblong"

"oblongata"

"oblongatae"

"oblongatas"

"oblongish"

"oblongly"

"oblongness"

"oblongs"

"obloquies"

"obloquy"

"obnoxiety"

"obnoxious"

"obnoxiously"

"obnoxiousness"

"oboe"

"oboes"

"oboist"

"oboists"

"obol"

"obols"

"obovate"

"obovoid"

"obscene"

"obscenely"

"obscener"

"obscenest"

"obscenities"

"obscenity"

"obscura"

"obscurant"

"obscuranticism"

"obscurantism"

"obscurantist"

"obscurantists"

"obscuras"

"obscuration"

"obscurative"

"obscure"

"obscured"

"obscurely"

"obscurement"

"obscurer"

"obscurers"

"obscures"

"obscurest"

"obscuring"

"obscurities"

"obscurity"

"obsequies"

"obsequious"

"obsequiously"

"obsequiousness"

"obsequy"

"observable"

"observably"

"observance"

"observances"

"observant"

"observation"

"observational"

"observations"

"observatories"

"observatory"

"observe"

"observed"

"observer"

"observers"

"observes"

"observing"

"observingly"

"obsess"

"obsessed"

"obsesses"

"obsessing"

"obsessingly"

"obsession"

"obsessional"

"obsessions"

"obsessive"

"obsessive compulsive disorder"

"obsessively"

"obsessiveness"

"obsessor"

"obsessors"

"obsidian"

"obsidians"

"obsolescence"

"obsolescent"

"obsolescently"

"obsolete"

"obsoleted"

"obsoletely"

"obsoleteness"

"obsoletes"

"obsoleting"

"obstacle"

"obstacles"

"obstetric"

"obstetrical"

"obstetrically"

"obstetrician"

"obstetricians"

"obstetrics"

"obstetrics and gynecology"

"obstinacies"

"obstinacy"

"obstinate"

"obstinately"

"obstinateness"

"obstreperous"

"obstreperously"

"obstreperousness"

"obstruct"

"obstructed"

"obstructer"

"obstructers"

"obstructing"

"obstruction"

"obstructionism"

"obstructionist"

"obstructionists"

"obstructions"

"obstructive"

"obstructively"

"obstructiveness"

"obstructor"

"obstructors"

"obstructs"

"obtain"

"obtainable"

"obtained"

"obtainer"

"obtainers"

"obtaining"

"obtainment"

"obtains"

"obtrude"

"obtruded"

"obtruder"

"obtruders"

"obtrudes"

"obtruding"

"obtrusion"

"obtrusions"

"obtrusive"

"obtrusively"

"obtrusiveness"

"obtuse"

"obtusely"

"obtuseness"

"obtuser"

"obtusest"

"obverse"

"obverses"

"obverting"

"obverts"

"obviate"

"obviated"

"obviates"

"obviating"

"obviation"

"obviator"

"obviators"

"obvious"

"obviously"

"obviousness"

"obwheel"

"ocarina"

"ocarinas"

"occasion"

"occasional"

"occasional reminder"

"occasionally"

"occasioned"

"occasioning"

"occasions"

"occident"

"occidental"

"occidentals"

"occidents"

"occipital"

"occiputs"

"occlude"

"occluded"

"occludes"

"occluding"

"occlusal"

"occlusion"

"occlusions"

"occlusive"

"occult"

"occulted"

"occulter"

"occulters"

"occulting"

"occultism"

"occultist"

"occultists"

"occultly"

"occults"

"occupance"

"occupancies"

"occupancy"

"occupant"

"occupants"

"occupation"

"occupational"

"occupational diseases"

"occupationally"

"occupations"

"occupative"

"occupiable"

"occupied"

"occupier"

"occupiers"

"occupies"

"occupy"

"occupying"

"occur"

"occurred"

"occurrence"

"occurrences"

"occurrent"

"occurring"

"occurs"

"ocean"

"ocean scenes"

"ocean waves"

"oceanarium"

"oceanaut"

"oceanauts"

"oceangoing"

"oceanic"

"oceanid"

"oceanographer"

"oceanographers"

"oceanographic"

"oceanography"

"oceanologist"

"oceanologists"

"oceanology"

"oceans"

"oceanside"

"ocellus"

"ocelot"

"ocelots"

"ocher"

"ochered"

"ocherous"

"ochers"

"ochery"

"ochlophobia"

"ochre"

"ochred"

"ochreous"

"ochres"

"ochring"

"ochroid"

"ochrous"

"oclock"

"ocotillos"

"octad"

"octads"

"octagon"

"octagonal"

"octagonally"

"octagons"

"octal"

"octane"

"octanes"

"octangle"

"octangles"

"octant"

"octants"

"octaval"

"octave"

"octaves"

"octavo"

"octavos"

"octet"

"octets"

"octette"

"octettes"

"october"

"octogenarian"

"octogenarians"

"octopi"

"octopod"

"octopodes"

"octopods"

"octopus"

"octopuses"

"octoroon"

"octoroons"

"octothorpe"

"octuple"

"octupled"

"octuples"

"octuplet"

"octuplets"

"octupling"

"octuply"

"octyl"

"octyls"

"ocular"

"ocularly"

"oculars"

"oculi"

"oculist"

"oculists"

"oculus"

"ocurred"

"od"

"odalisk"

"odalisks"

"odalisque"

"odd"

"oddball"

"oddballs"

"odder"

"oddest"

"oddish"

"oddities"

"oddity"

"oddly"

"oddment"

"oddments"

"oddness"

"oddnesses"

"odds"

"ode"

"odeon"

"odeons"

"odes"

"odessa"

"odic"

"odin"

"odious"

"odiously"

"odiousness"

"odium"

"odiums"

"odograph"

"odology"

"odometer"

"odometers"

"odontograph"

"odontology"

"odor"

"odorant"

"odorants"

"odored"

"odorful"

"odoriferous"

"odoriferously"

"odoriferousness"

"odorize"

"odorized"

"odorizes"

"odorizing"

"odorless"

"odorous"

"odorously"

"odors"

"odour"

"odourful"

"odours"

"odyl"

"odysseus"

"odyssey"

"odysseys"

"oedipal"

"oedipus"

"oedipuses"

"oenology"

"oenomania"

"oenomel"

"oenometer"

"oenophile"

"oenophiles"

"oenophobia"

"oersted"

"oesophagus"

"oeuvre"

"oeuvres"

"of"

"ofay"

"ofays"

"off"

"offal"

"offals"

"offbeat"

"offbeats"

"offcast"

"offcut"

"offed"

"offence"

"offences"

"offend"

"offended"

"offender"

"offenders"

"offending"

"offends"

"offense"

"offenseless"

"offenses"

"offensive"

"offensively"

"offensiveness"

"offensives"

"offer"

"offerable"

"offered"

"offeree"

"offerer"

"offerers"

"offering"

"offerings"

"offeror"

"offerors"

"offers"

"offers several free and premium plugins"

"offertories"

"offertory"

"offhand"

"offhanded"

"offhandedly"

"offhandedness"

"office"

"officeholder"

"officeholders"

"officer"

"officered"

"officering"

"officers"

"offices"

"official"

"officialdom"

"officialism"

"officialities"

"officiality"

"officially"

"officials"

"officiant"

"officiants"

"officiary"

"officiate"

"officiated"

"officiates"

"officiating"

"officiation"

"officiator"

"officinal"

"officio"

"officious"

"officiously"

"officiousness"

"offing"

"offings"

"offish"

"offishness"

"offline"

"offload"

"offloaded"

"offloading"

"offloads"

"offpay"

"offprint"

"offprints"

"offs"

"offset"

"offsets"

"offsetting"

"offshoot"

"offshoots"

"offshore"

"offside"

"offspring"

"offsprings"

"offstage"

"offtrack"

"oft"

"often"

"oftener"

"oftenest"

"oftenness"

"ofter"

"oftest"

"ofttimes"

"ogee"

"ogees"

"ogham"

"oghamic"

"ogive"

"ogle"

"ogled"

"ogler"

"oglers"

"ogles"

"ogling"

"ogre"

"ogreish"

"ogreism"

"ogres"

"ogress"

"ogresses"

"ogrish"

"ogrishly"

"oh"

"ohed"

"ohing"

"ohio"

"ohioan"

"ohioans"

"ohm"

"ohmage"

"ohmages"

"ohmic"

"ohmmeter"

"ohmmeters"

"ohms"

"oho"

"ohs"

"oidium"

"oikology"

"oil"

"oilbirds"

"oilcan"

"oilcans"

"oilcloth"

"oilcloths"

"oilcup"

"oilcups"

"oiled"

"oiler"

"oilers"

"oilheating"

"oilhole"

"oilier"

"oiliest"

"oilily"

"oiliness"

"oiling"

"oilman"

"oilmen"

"oilpapers"

"oils"

"oilseed"

"oilseeds"

"oilskin"

"oilskins"

"oilstone"

"oilstones"

"oilway"

"oily"

"oink"

"oinked"

"oinking"

"oinks"

"ointment"

"ointments"

"ojibwa"

"ojibwas"

"ok"

"okapi"

"okapis"

"okay"

"okayed"

"okaying"

"okays"

"okeydoke"

"okie"

"okinawa"

"oklahoma"

"oklahoman"

"oklahomans"

"okra"

"okras"

"old"

"olden"

"older"

"oldest"

"oldfashioned"

"oldie"

"oldies"

"oldish"

"oldness"

"oldnesses"

"olds"

"oldsmobile"

"oldster"

"oldsters"

"oldstyles"

"ole"

"oleaginous"

"oleander"

"oleanders"

"oleo"

"oleomargarine"

"oleometer"

"oleoresin"

"oleos"

"oles"

"oleums"

"olfaction"

"olfactology"

"olfactometer"

"olfactometric"

"olfactometry"

"olfactory"

"olibanums"

"oligarch"

"oligarchic"

"oligarchical"

"oligarchies"

"oligarchs"

"oligarchy"

"oligocene"

"oligomania"

"oligopoly"

"olio"

"olios"

"olive"

"oliver"

"olives"

"olivia"

"olivine"

"olivines"

"olivinic"

"olla"

"ollas"

"ologies"

"ologist"

"ologists"

"olograph"

"ology"

"olympia"

"olympiad"

"olympiads"

"olympian"

"olympians"

"olympic"

"olympics"

"olympus"

"omaha"

"omahas"

"oman"

"ombre"

"ombres"

"ombrology"

"ombrometer"

"ombrophobia"

"ombudsman"

"ombudsmen"

"omega"

"omegas"

"omelet"

"omelets"

"omelette"

"omelettes"

"omen"

"omened"

"omens"

"omicron"

"omicrons"

"omikron"

"ominous"

"ominously"

"ominousness"

"omissible"

"omission"

"omissions"

"omissive"

"omit"

"omits"

"omittance"

"omitted"

"omitting"

"omniarchs"

"omnibus"

"omnibuses"

"omnicompetence"

"omnicompetent"

"omnific"

"omnipotence"

"omnipotent"

"omnipotently"

"omnipresence"

"omnipresent"

"omniscience"

"omniscient"

"omnisciently"

"omnium"

"omnivore"

"omnivores"

"omnivorous"

"omnivorously"

"omnivorousness"

"omphali"

"omphalos"

"oms"

"on"

"onager"

"onagers"

"onanism"

"onanisms"

"onanist"

"onanistic"

"onanists"

"onboard"

"once"

"onces"

"oncogenic"

"oncograph"

"oncologic"

"oncological"

"oncologies"

"oncology"

"oncometer"

"oncoming"

"oncomings"

"oncosimeter"

"ondograph"

"one"

"onefold"

"oneida"

"oneidas"

"oneirology"

"oneness"

"onenesses"

"onerosities"

"onerosity"

"onerous"

"onerously"

"onerousness"

"onery"

"ones"

"oneself"

"onethird"

"onetime"

"ongoing"

"ongress"

"oniomania"

"onion"

"onions"

"onionskin"

"onionskins"

"online"

"onlooker"

"onlookers"

"only"

"onomamania"

"onomasiology"

"onomastics"

"onomatomania"

"onomatophobia"

"onomatopoeia"

"onomatopoeic"

"onomatopoeically"

"onomatopoetic"

"onomatopoetically"

"onomatopoieses"

"onomatopoiesis"

"onondaga"

"onondagas"

"onrush"

"onrushes"

"onrushing"

"onset"

"onsets"

"onshore"

"onside"

"onslaught"

"onslaughts"

"onstage"

"ontario"

"onto"

"ontogeneses"

"ontogenesis"

"ontogenetic"

"ontogenetically"

"ontogenic"

"ontogenically"

"ontogenies"

"ontogeny"

"ontological"

"ontologies"

"ontology"

"onus"

"onuses"

"onward"

"onwards"

"onychotillomania"

"onyx"

"onyxes"

"oocyte"

"oodles"

"ooh"

"oohed"

"oohing"

"oohs"

"oolite"

"oolith"

"oology"

"oolong"

"oolongs"

"oometer"

"oomph"

"oomphs"

"oops"

"ooze"

"oozed"

"oozes"

"oozier"

"ooziest"

"oozily"

"ooziness"

"oozing"

"oozy"

"opacification"

"opacified"

"opacifies"

"opacify"

"opacifying"

"opacimeter"

"opacities"

"opacity"

"opal"

"opalesced"

"opalescence"

"opalescent"

"opalesces"

"opalescing"

"opaline"

"opals"

"opaque"

"opaqued"

"opaquely"

"opaqueness"

"opaquer"

"opaques"

"opaquest"

"opaquing"

"ope"

"opec"

"opeidoscope"

"open"

"openable"

"opened"

"openendedness"

"opener"

"openers"

"openest"

"openhanded"

"openhandedly"

"openhandedness"

"openhearted"

"openheartedly"

"openheartedness"

"opening"

"openings"

"openly"

"openmouthed"

"openness"

"opens"

"openwork"

"openworks"

"opera"

"operabilities"

"operability"

"operable"

"operably"

"operand"

"operandi"

"operands"

"operant"

"operants"

"operas"

"operate"

"operated"

"operates"

"operatic"

"operatically"

"operatics"

"operating"

"operation"

"operational"

"operationally"

"operations"

"operative"

"operatively"

"operatives"

"operator"

"operators"

"operculated"

"operetta"

"operettas"

"opes"

"ophidian"

"ophidians"

"ophidiophobia"

"ophiology"

"ophthalmic"

"ophthalmologic"

"ophthalmological"

"ophthalmologically"

"ophthalmologies"

"ophthalmologist"

"ophthalmologists"

"ophthalmology"

"ophthalmometer"

"ophthalmometry"

"ophthalmophobia"

"ophthalmoscope"

"ophthalmoscopes"

"ophthalmoscopic"

"ophthalmoscopies"

"ophthalmoscopy"

"opiate"

"opiated"

"opiates"

"opiating"

"opine"

"opined"

"opiner"

"opiners"

"opines"

"opining"

"opinion"

"opinionated"

"opinionatedly"

"opinions"

"opiomania"

"opisometer"

"opium"

"opiumisms"

"opiums"

"opossum"

"opossums"

"opp"

"opponent"

"opponents"

"opportune"

"opportunely"

"opportunism"

"opportunist"

"opportunistic"

"opportunists"

"opportunities"

"opportunity"

"opposabilities"

"opposability"

"opposable"

"oppose"

"opposed"

"opposer"

"opposers"

"opposes"

"opposing"

"opposite"

"oppositely"

"oppositeness"

"opposites"

"opposition"

"oppositional"

"oppositionist"

"oppositionists"

"oppress"

"oppressed"

"oppresses"

"oppressing"

"oppression"

"oppressive"

"oppressively"

"oppressiveness"

"oppressor"

"oppressors"

"opprobriate"

"opprobriated"

"opprobriating"

"opprobrious"

"opprobriously"

"opprobrium"

"opprobriums"

"oppugn"

"oppugns"

"ops"

"opsiometer"

"opsomania"

"opt"

"optative"

"optatives"

"opted"

"optic"

"optical"

"optically"

"optician"

"opticians"

"opticist"

"opticopupillary"

"optics"

"optima"

"optimal"

"optimally"

"optimeter"

"optimise"

"optimism"

"optimisms"

"optimist"

"optimistic"

"optimistical"

"optimistically"

"optimists"

"optimization"

"optimize"

"optimized"

"optimizes"

"optimizing"

"optimum"

"optimum state"

"optimums"

"opting"

"option"

"optional"

"optionally"

"optionals"

"optionees"

"optioning"

"options"

"optology"

"optometer"

"optometric"

"optometrical"

"optometries"

"optometrist"

"optometrists"

"optometry"

"optophobia"

"opts"

"opulence"

"opulences"

"opulencies"

"opulency"

"opulent"

"opulently"

"opus"

"opuses"

"or"

"oracle"

"oracles"

"oracular"

"oracularly"

"oral"

"oralities"

"orality"

"orally"

"oralogy"

"orals"

"orang"

"orange"

"Orange Book"

"orangeade"

"orangeades"

"orangeries"

"orangery"

"oranges"

"orangey"

"orangier"

"orangiest"

"orangish"

"orangs"

"orangutan"

"orangutans"

"orangy"

"orate"

"orated"

"orates"

"orating"

"oration"

"orations"

"orator"

"oratorian"

"oratorical"

"oratorically"

"oratories"

"oratorio"

"oratorios"

"orators"

"oratory"

"oratress"

"oratresses"

"oratrices"

"oratrix"

"orb"

"orbed"

"orbicular"

"orbing"

"orbit"

"orbital"

"orbitally"

"orbitals"

"orbited"

"orbiter"

"orbiters"

"orbiting"

"orbits"

"orbs"

"orc"

"orca"

"orcas"

"orch"

"orchard"

"orchardist"

"orchardists"

"orchardman"

"orchards"

"orchectomy"

"orchestra"

"orchestral"

"orchestrally"

"orchestras"

"orchestrate"

"orchestrated"

"orchestrates"

"orchestrating"

"orchestration"

"orchestrations"

"orchestrator"

"orchestrators"

"orchid"

"orchidology"

"orchidomania"

"orchidometer"

"orchids"

"orchis"

"orcs"

"ord"

"ordain"

"ordained"

"ordainer"

"ordainers"

"ordaining"

"ordainment"

"ordains"

"ordeal"

"ordeals"

"order"

"ordered"

"orderer"

"orderers"

"ordering"

"orderings"

"orderlies"

"orderliness"

"orderly"

"orders"

"ordinal"

"ordinals"

"ordinance"

"ordinances"

"ordinands"

"ordinarier"

"ordinaries"

"ordinarily"

"ordinariness"

"ordinarius"

"ordinary"

"ordinate"

"ordinates"

"ordination"

"ordinations"

"ordnance"

"ordnances"

"ordo"

"ordonnance"

"ordos"

"ordure"

"ordures"

"ordurous"

"ore"

"oread"

"orean"

"oregano"

"oreganos"

"oregon"

"oregonian"

"oregonians"

"ores"

"orf"

"organ"

"organa"

"organdie"

"organdies"

"organdy"

"organelle"

"organelles"

"organic"

"organically"

"organics"

"organism"

"organismal"

"organismic"

"organisms"

"organist"

"organists"

"organization"

"organizational"

"organizationally"

"organizations"

"organize"

"organized"

"organizer"

"organizers"

"organizes"

"organizing"

"organophosphate"

"organs"

"organza"

"orgasm"

"orgasmic"

"orgasms"

"orgastic"

"orgeat"

"orgeats"

"orgiac"

"orgiastic"

"orgiastical"

"orgic"

"orgies"

"orgy"

"oriel"

"oriels"

"orient"

"oriental"

"orientals"

"orientate"

"orientated"

"orientates"

"orientating"

"orientation"

"orientations"

"oriented"

"orienting"

"orients"

"orifice"

"orifices"

"orificial"

"orig"

"origami"

"origamis"

"origin"

"original"

"originalities"

"originality"

"originally"

"originals"

"originate"

"originated"

"originates"

"originating"

"origination"

"originator"

"originators"

"origins"

"oriole"

"orioles"

"orion"

"orison"

"orisons"

"orleans"

"orlon"

"ormolu"

"ormolus"

"ornament"

"ornamental"

"ornamentation"

"ornamentations"

"ornamented"

"ornamenting"

"ornaments"

"ornate"

"ornately"

"ornateness"

"ornerier"

"orneriest"

"orneriness"

"ornery"

"ornithological"

"ornithologist"

"ornithologists"

"ornithology"

"ornithophobia"

"orogenic"

"orogeny"

"orology"

"orotund"

"orotundity"

"orphan"

"orphanage"

"orphanages"

"orphaned"

"orphanhood"

"orphaning"

"orphans"

"orpheus"

"orphic"

"orpiments"

"orpines"

"orreries"

"orrery"

"orris"

"orrises"

"orrisroot"

"ors"

"ort"

"orth"

"ortho"

"orthodontia"

"orthodontic"

"orthodontics"

"orthodontist"

"orthodontists"

"orthodox"

"orthodoxes"

"orthodoxies"

"orthodoxy"

"orthoepist"

"orthoepists"

"orthoepy"

"orthographic"

"orthographically"

"orthography"

"orthomolecular"

"orthopaedic"

"orthopaedics"

"orthopaedist"

"orthopedic"

"orthopedically"

"orthopedics"

"orthopedist"

"orthopedists"

"orthopterology"

"ortolan"

"ortolans"

"orts"

"orwell"

"orwellian"

"oryctology"

"oryx"

"oryxes"

"os"

"osage"

"osages"

"osaka"

"oscar"

"oscars"

"oscillate"

"oscillated"

"oscillates"

"oscillating"

"oscillation"

"oscillations"

"oscillator"

"oscillators"

"oscillatory"

"oscillogram"

"oscillograph"

"oscillographic"

"oscillographies"

"oscillography"

"oscillometer"

"oscillometric"

"oscillometries"

"oscillometry"

"oscilloscope"

"oscilloscopes"

"oscilloscopic"

"oscilloscopically"

"oscula"

"osculant"

"oscular"

"osculate"

"osculated"

"osculates"

"osculating"

"osculation"

"osculations"

"oscule"

"oscules"

"osculum"

"osier"

"osiers"

"oslo"

"osmic"

"osmics"

"osmium"

"osmiums"

"osmol"

"osmology"

"osmometer"

"osmose"

"osmosed"

"osmoses"

"osmosing"

"osmosis"

"osmotic"

"osmotically"

"osphresiology"

"osprey"

"ospreys"

"ossea"

"osseous"

"osseously"

"ossia"

"ossification"

"ossifications"

"ossificatory"

"ossified"

"ossifier"

"ossifiers"

"ossifies"

"ossify"

"ossifying"

"ossuaries"

"ossuary"

"osteal"

"osteitic"

"osteitis"

"ostensibilities"

"ostensibility"

"ostensible"

"ostensibly"

"ostensive"

"ostentation"

"ostentatious"

"ostentatiously"

"osteoarthritic"

"osteoarthritis"

"osteological"

"osteologically"

"osteologies"

"osteologist"

"osteology"

"osteopath"

"osteopathic"

"osteopathically"

"osteopathies"

"osteopathist"

"osteopaths"

"osteopathy"

"osteoporosis"

"osteoscleroses"

"osteosclerosis"

"osteosclerotic"

"osteotome"

"osteotomy"

"ostia"

"ostinato"

"ostinatos"

"ostium"

"ostler"

"ostlers"

"ostmark"

"ostmarks"

"ostomy"

"ostracism"

"ostracize"

"ostracized"

"ostracizes"

"ostracizing"

"ostracods"

"ostrich"

"ostriches"

"oswego"

"other"

"other workout trainers"

"others"

"otherwise"

"otherworldliness"

"otherworldly"

"otic"

"otiose"

"otiosely"

"otiosity"

"otolaryngologies"

"otolaryngologist"

"otolaryngologists"

"otolaryngology"

"otolith"

"otolithic"

"otoliths"

"otologic"

"otological"

"otologically"

"otologies"

"otologist"

"otology"

"otorhinolaryngology"

"otoscope"

"otoscopes"

"otoscopic"

"otoscopies"

"otoscopy"

"ottawa"

"ottawas"

"otter"

"otters"

"otto"

"ottoman"

"ottomans"

"oubliette"

"oubliettes"

"ouch"

"ouches"

"ought"

"oughted"

"oughts"

"oui"

"ouija"

"ounce"

"ounces"

"our"

"ourangs"

"ours"

"ourself"

"ourselves"

"ousel"

"ousels"

"oust"

"ousted"

"ouster"

"ousters"

"ousting"

"ousts"

"out"

"outage"

"outages"

"outargue"

"outargued"

"outargues"

"outarguing"

"outback"

"outbacks"

"outbalance"

"outbalanced"

"outbalances"

"outbalancing"

"outbargain"

"outbargained"

"outbargaining"

"outbargains"

"outbid"

"outbidden"

"outbidding"

"outbids"

"outbluff"

"outbluffed"

"outbluffing"

"outbluffs"

"outboard"

"outboards"

"outboast"

"outboasted"

"outboasting"

"outboasts"

"outbound"

"outbox"

"outboxed"

"outboxes"

"outboxing"

"outbreak"

"outbreaks"

"outbuilding"

"outbuildings"

"outburst"

"outbursts"

"outcast"

"outcaste"

"outcastes"

"outcasts"

"outchiding"

"outclass"

"outclassed"

"outclasses"

"outclassing"

"outcome"

"outcomes"

"outcried"

"outcries"

"outcrop"

"outcropped"

"outcropping"

"outcroppings"

"outcrops"

"outcry"

"outdate"

"outdated"

"outdates"

"outdating"

"outdid"

"outdistance"

"outdistanced"

"outdistances"

"outdistancing"

"outdo"

"outdodge"

"outdodged"

"outdodges"

"outdodging"

"outdoer"

"outdoers"

"outdoes"

"outdoing"

"outdone"

"outdoor"

"outdoor activities"

"outdoor sport"

"outdoors"

"outdraw"

"outdrew"

"outed"

"outer"

"outermost"

"outers"

"outface"

"outfaced"

"outfaces"

"outfacing"

"outfield"

"outfielded"

"outfielder"

"outfielders"

"outfielding"

"outfields"

"outfight"

"outfighting"

"outfights"

"outfit"

"outfits"

"outfitted"

"outfitter"

"outfitters"

"outfitting"

"outfittings"

"outflank"

"outflanked"

"outflanker"

"outflanking"

"outflanks"

"outflew"

"outflow"

"outflowed"

"outflowing"

"outflows"

"outfought"

"outfox"

"outfoxed"

"outfoxes"

"outfoxing"

"outgas"

"outgassed"

"outgasses"

"outgassing"

"outgo"

"outgoes"

"outgoing"

"outgoings"

"outgrew"

"outgrow"

"outgrowing"

"outgrown"

"outgrows"

"outgrowth"

"outgrowths"

"outguess"

"outguessed"

"outguesses"

"outguessing"

"outgun"

"outgunned"

"outgunning"

"outguns"

"outgushes"

"outhit"

"outhits"

"outhitting"

"outhouse"

"outhouses"

"outing"

"outings"

"outjuts"

"outjutting"

"outland"

"outlandish"

"outlandishly"

"outlandishness"

"outlands"

"outlast"

"outlasted"

"outlasting"

"outlasts"

"outlaw"

"outlawed"

"outlawing"

"outlawries"

"outlawry"

"outlaws"

"outlay"

"outlaying"

"outlays"

"outleap"

"outleaped"

"outleaping"

"outleaps"

"outleapt"

"outlet"

"outlets"

"outlie"

"outlier"

"outliers"

"outlies"

"outline"

"outlined"

"outlines"

"outlining"

"outlive"

"outlived"

"outliver"

"outlivers"

"outlives"

"outliving"

"outlook"

"outlooks"

"outlying"

"outmaneuver"

"outmaneuvered"

"outmaneuvering"

"outmaneuvers"

"outmarch"

"outmarched"

"outmarches"

"outmarching"

"outmode"

"outmoded"

"outmodes"

"outmoved"

"outnumber"

"outnumbered"

"outnumbering"

"outnumbers"

"outpace"

"outpaced"

"outpaces"

"outpacing"

"outpatient"

"outpatients"

"outpayment"

"outperform"

"outperformed"

"outperforming"

"outperforms"

"outplay"

"outplayed"

"outplaying"

"outplays"

"outpost"

"outposts"

"outpour"

"outpoured"

"outpouring"

"outpourings"

"outproduce"

"outproduced"

"outproduces"

"outproducing"

"output"

"outputs"

"outputted"

"outputting"

"outrace"

"outraced"

"outraces"

"outracing"

"outrage"

"outraged"

"outrageous"

"outrageously"

"outrageousness"

"outrages"

"outraging"

"outran"

"outrange"

"outranged"

"outranges"

"outranging"

"outrank"

"outranked"

"outranking"

"outranks"

"outre"

"outreach"

"outreached"

"outreaches"

"outreaching"

"outreason"

"outreasoned"

"outreasoning"

"outreasons"

"outrider"

"outriders"

"outrides"

"outriding"

"outrigger"

"outriggers"

"outright"

"outrightness"

"outrooted"

"outrooting"

"outrun"

"outrunning"

"outruns"

"outrush"

"outs"

"outscore"

"outscored"

"outscores"

"outscoring"

"outsell"

"outselling"

"outsells"

"outset"

"outsets"

"outshine"

"outshined"

"outshines"

"outshining"

"outshone"

"outshout"

"outshouted"

"outshouting"

"outshouts"

"outside"

"outsider"

"outsiders"

"outsides"

"outsize"

"outsized"

"outsizes"

"outskirt"

"outskirts"

"outsmart"

"outsmarted"

"outsmarting"

"outsmarts"

"outsold"

"outspell"

"outspelled"

"outspelling"

"outspells"

"outspoke"

"outspoken"

"outspokenly"

"outspokenness"

"outspread"

"outspreading"

"outspreads"

"outstand"

"outstanding"

"outstandingly"

"outstandingness"

"outstands"

"outstare"

"outstared"

"outstares"

"outstaring"

"outstation"

"outstations"

"outstay"

"outstayed"

"outstaying"

"outstays"

"outstretch"

"outstretched"

"outstretches"

"outstretching"

"outstrip"

"outstripped"

"outstripping"

"outstrips"

"outstroke"

"outswam"

"outswim"

"outswimming"

"outswims"

"outswum"

"outtakes"

"outthink"

"outtrumped"

"outvote"

"outvoted"

"outvotes"

"outvoting"

"outwait"

"outwaited"

"outwaits"

"outwalk"

"outwalked"

"outwalking"

"outwalks"

"outward"

"outwardly"

"outwards"

"outwear"

"outwearing"

"outwears"

"outweigh"

"outweighed"

"outweighing"

"outweighs"

"outwit"

"outwits"

"outwitted"

"outwitting"

"outwore"

"outwork"

"outworked"

"outworker"

"outworkers"

"outworking"

"outworks"

"outworn"

"outyell"

"outyelled"

"outyelling"

"outyells"

"ouzel"

"ouzels"

"ouzo"

"ouzos"

"ova"

"oval"

"ovality"

"ovally"

"ovalness"

"ovals"

"ovarial"

"ovarian"

"ovaries"

"ovary"

"ovate"

"ovately"

"ovation"

"ovations"

"oven"

"ovenbird"

"ovens"

"ovenware"

"ovenwares"

"over"

"Over-the-counter"

"overabound"

"overabounded"

"overabounding"

"overabounds"

"overabundance"

"overabundant"

"overachieve"

"overachieved"

"overachiever"

"overachieving"

"overact"

"overacted"

"overacting"

"overactive"

"overacts"

"overadorned"

"overage"

"overages"

"overaggressive"

"overall"

"overalls"

"overambitious"

"overambitiously"

"overanalyze"

"overanalyzed"

"overanalyzes"

"overanalyzing"

"overanxious"

"overapprehensive"

"overapprehensively"

"overapprehensiveness"

"overarched"

"overarches"

"overargumentative"

"overarm"

"overassertive"

"overassertively"

"overassertiveness"

"overassessment"

"overassured"

"overate"

"overattached"

"overattentive"

"overattentively"

"overattentiveness"

"overawe"

"overawed"

"overawes"

"overawing"

"overbake"

"overbaked"

"overbakes"

"overbaking"

"overbalance"

"overbalanced"

"overbalances"

"overbalancing"

"overbear"

"overbearing"

"overbearingly"

"overbears"

"overbid"

"overbidden"

"overbidding"

"overbids"

"overbite"

"overbites"

"overblown"

"overblows"

"overboard"

"overbold"

"overbooked"

"overbooks"

"overbore"

"overborne"

"overbought"

"overburden"

"overburdened"

"overburdening"

"overburdens"

"overburdensome"

"overbuy"

"overbuying"

"overbuys"

"overcame"

"overcapacity"

"overcapitalize"

"overcapitalized"

"overcapitalizes"

"overcapitalizing"

"overcareful"

"overcast"

"overcasts"

"overcasual"

"overcautious"

"overcautiously"

"overcautiousness"

"overcharge"

"overcharged"

"overcharges"

"overcharging"

"overcloud"

"overclouded"

"overclouding"

"overclouds"

"overcoat"

"overcoats"

"overcome"

"overcomes"

"overcoming"

"overcommon"

"overcompensate"

"overcompensated"

"overcompensates"

"overcompensating"

"overcompensation"

"overcompensations"

"overcompensators"

"overcompetitive"

"overcomplacency"

"overcomplacent"

"overconcern"

"overconfidence"

"overconfident"

"overconfidently"

"overconscientious"

"overconservative"

"overconsiderate"

"overcook"

"overcooked"

"overcooking"

"overcooks"

"overcool"

"overcooled"

"overcooling"

"overcools"

"overcorrection"

"overcritical"

"overcritically"

"overcrowd"

"overcrowded"

"overcrowding"

"overcrowds"

"overcurious"

"overdecorate"

"overdecorated"

"overdecorates"

"overdecorating"

"overdefensive"

"overdelicate"

"overdependence"

"overdependent"

"overdesirous"

"overdetailed"

"overdevelop"

"overdeveloped"

"overdeveloping"

"overdevelopment"

"overdevelops"

"overdid"

"overdiligent"

"overdiligently"

"overdiversification"

"overdiversified"

"overdiversifies"

"overdiversify"

"overdiversifying"

"overdiversity"

"overdo"

"overdoes"

"overdoing"

"overdone"

"overdosage"

"overdose"

"overdosed"

"overdoses"

"overdosing"

"overdraft"

"overdrafts"

"overdramatize"

"overdramatized"

"overdramatizes"

"overdramatizing"

"overdrank"

"overdraw"

"overdrawing"

"overdrawn"

"overdraws"

"overdress"

"overdressed"

"overdresses"

"overdressing"

"overdrew"

"overdrink"

"overdrinking"

"overdrinks"

"overdrive"

"overdrives"

"overdrunk"

"overdue"

"overeager"

"overearnest"

"overeasy"

"overeat"

"overeaten"

"overeating"

"overeats"

"overed"

"overeducate"

"overeducated"

"overeducates"

"overeducating"

"overelaborate"

"overelaborated"

"overelaborates"

"overelaborating"

"overembellish"

"overembellished"

"overembellishes"

"overembellishing"

"overemotional"

"overemphasis"

"overemphasize"

"overemphasized"

"overemphasizes"

"overemphasizing"

"overemphatic"

"overenthusiastic"

"overenthusiastically"

"overestimate"

"overestimated"

"overestimates"

"overestimating"

"overestimation"

"overestimations"

"overexcitable"

"overexcitably"

"overexcite"

"overexcited"

"overexcites"

"overexciting"

"overexercise"

"overexercised"

"overexercises"

"overexercising"

"overexert"

"overexerted"

"overexerting"

"overexerts"

"overexpand"

"overexpanded"

"overexpanding"

"overexpands"

"overexpansion"

"overexpectant"

"overexplicit"

"overexpose"

"overexposed"

"overexposes"

"overexposing"

"overexposure"

"overextend"

"overextended"

"overextending"

"overextends"

"overextension"

"overfamiliar"

"overfamiliarity"

"overfanciful"

"overfastidious"

"overfatigue"

"overfatigued"

"overfatigues"

"overfatiguing"

"overfed"

"overfeed"

"overfeeding"

"overfeeds"

"overfill"

"overfilled"

"overfilling"

"overfills"

"overflew"

"overflies"

"overflight"

"overflights"

"overflow"

"overflowed"

"overflowing"

"overflown"

"overflows"

"overfly"

"overflying"

"overfond"

"overfull"

"overfurnish"

"overfurnished"

"overfurnishes"

"overfurnishing"

"overgarment"

"overgeneralization"

"overgeneralize"

"overgeneralized"

"overgeneralizes"

"overgeneralizing"

"overgenerous"

"overglaze"

"overglazes"

"overgraze"

"overgrazed"

"overgrazes"

"overgrazing"

"overgrew"

"overgrow"

"overgrowing"

"overgrown"

"overgrows"

"overgrowth"

"overhand"

"overhanded"

"overhands"

"overhang"

"overhanging"

"overhangs"

"overhastily"

"overhastiness"

"overhasty"

"overhaul"

"overhauled"

"overhauling"

"overhauls"

"overhead"

"overheads"

"overheaped"

"overheaps"

"overhear"

"overheard"

"overhearing"

"overhears"

"overheat"

"overheated"

"overheating"

"overheats"

"overhung"

"overhurried"

"overidealistic"

"overimaginative"

"overimpress"

"overimpressed"

"overimpresses"

"overimpressing"

"overincline"

"overinclined"

"overinclines"

"overinclining"

"overindulge"

"overindulged"

"overindulgence"

"overindulgent"

"overindulges"

"overindulging"

"overindustrialize"

"overindustrialized"

"overindustrializes"

"overindustrializing"

"overinflate"

"overinflated"

"overinflates"

"overinflating"

"overinfluential"

"overinsistence"

"overinsistent"

"overinsistently"

"overinsure"

"overinsured"

"overinsures"

"overinsuring"

"overintellectual"

"overintellectually"

"overintense"

"overintensely"

"overinterest"

"overinvest"

"overinvested"

"overinvesting"

"overinvests"

"overissue"

"overissues"

"overjoy"

"overjoyed"

"overjoying"

"overjoys"

"overkill"

"overkilled"

"overkills"

"overladed"

"overladen"

"overlades"

"overlaid"

"overlain"

"overland"

"overlands"

"overlap"

"overlapped"

"overlapping"

"overlaps"

"overlarge"

"overlavish"

"overlay"

"overlaying"

"overlays"

"overleaf"

"overleap"

"overleaped"

"overleaping"

"overleaps"

"overleapt"

"overlie"

"overlies"

"overload"

"overloaded"

"overloading"

"overloads"

"overlong"

"overlook"

"overlooked"

"overlooking"

"overlooks"

"overlord"

"overlorded"

"overlords"

"overlordship"

"overly"

"overlying"

"overmagnification"

"overmagnified"

"overmagnifies"

"overmagnify"

"overmagnifying"

"overman"

"overmans"

"overmaster"

"overmastered"

"overmastering"

"overmasters"

"overmatch"

"overmatched"

"overmatches"

"overmatching"

"overmen"

"overmodest"

"overmodestly"

"overmodified"

"overmodifies"

"overmodify"

"overmodifying"

"overmuch"

"overmuches"

"overnice"

"overnight"

"overnighters"

"overoptimism"

"overpaid"

"overparticular"

"overpass"

"overpassed"

"overpasses"

"overpast"

"overpay"

"overpaying"

"overpayment"

"overpays"

"overpessimistic"

"overplay"

"overplayed"

"overplaying"

"overplays"

"overpopulate"

"overpopulated"

"overpopulates"

"overpopulating"

"overpopulation"

"overpower"

"overpowered"

"overpowerful"

"overpowering"

"overpoweringly"

"overpowers"

"overpraise"

"overpraised"

"overpraises"

"overpraising"

"overprecise"

"overprecisely"

"overprice"

"overpriced"

"overprices"

"overpricing"

"overprint"

"overprinted"

"overprinting"

"overprints"

"overproduce"

"overproduced"

"overproduces"

"overproducing"

"overproduction"

"overprominent"

"overprompt"

"overpromptly"

"overproportion"

"overprotect"

"overprotected"

"overprotecting"

"overprotection"

"overprotects"

"overproud"

"overqualified"

"overran"

"overrank"

"overrate"

"overrated"

"overrates"

"overrating"

"overreach"

"overreached"

"overreacher"

"overreachers"

"overreaches"

"overreaching"

"overreact"

"overreacted"

"overreacting"

"overreaction"

"overreactions"

"overreacts"

"overrefine"

"overrefined"

"overrefinement"

"overrefines"

"overrefining"

"overridden"

"override"

"overrides"

"overriding"

"overrighteous"

"overrighteously"

"overrighteousness"

"overrigid"

"overripe"

"overroast"

"overroasted"

"overroasting"

"overroasts"

"overrode"

"overrule"

"overruled"

"overrules"

"overruling"

"overrun"

"overrunning"

"overruns"

"overs"

"oversalt"

"oversalted"

"oversalting"

"oversalts"

"oversaw"

"overscrupulous"

"overscrupulously"

"overscrupulousness"

"oversea"

"overseas"

"oversee"

"overseeing"

"overseen"

"overseer"

"overseers"

"overseership"

"oversees"

"oversell"

"overselling"

"oversells"

"oversensitive"

"oversensitively"

"oversensitiveness"

"oversensitivity"

"oversevere"

"oversexed"

"overshadow"

"overshadowed"

"overshadowing"

"overshadows"

"oversharp"

"overshoe"

"overshoes"

"overshoot"

"overshooting"

"overshoots"

"overshot"

"overshots"

"oversides"

"oversight"

"oversights"

"oversimple"

"oversimplification"

"oversimplifications"

"oversimplified"

"oversimplifies"

"oversimplify"

"oversimplifying"

"oversize"

"oversized"

"oversizes"

"overskeptical"

"overskirt"

"oversleep"

"oversleeping"

"oversleeps"

"overslept"

"overslips"

"overslipt"

"oversold"

"oversolicitous"

"oversolicitously"

"oversolicitousness"

"oversophisticated"

"oversoul"

"oversouls"

"oversparing"

"overspecialization"

"overspecialize"

"overspecialized"

"overspecializes"

"overspecializing"

"overspend"

"overspending"

"overspends"

"overspent"

"overspins"

"overspread"

"overspreading"

"overspreads"

"overstate"

"overstated"

"overstatement"

"overstatements"

"overstates"

"overstating"

"overstay"

"overstayed"

"overstaying"

"overstays"

"overstep"

"overstepped"

"overstepping"

"oversteps"

"overstimulate"

"overstimulated"

"overstimulates"

"overstimulating"

"overstimulation"

"overstock"

"overstocked"

"overstocking"

"overstocks"

"overstrain"

"overstretch"

"overstretched"

"overstretches"

"overstretching"

"overstrict"

"overstrike"

"overstuff"

"overstuffed"

"oversubscribe"

"oversubscribed"

"oversubscribes"

"oversubscribing"

"oversubscription"

"oversubtle"

"oversubtleties"

"oversubtlety"

"oversupplied"

"oversupplies"

"oversupply"

"oversupplying"

"oversuspicious"

"oversystematic"

"overt"

"overtake"

"overtaken"

"overtakes"

"overtaking"

"overtax"

"overtaxed"

"overtaxes"

"overtaxing"

"overtechnical"

"overthrew"

"overthrow"

"overthrower"

"overthrowers"

"overthrowing"

"overthrown"

"overthrows"

"overtime"

"overtire"

"overtired"

"overtires"

"overtiring"

"overtly"

"overtone"

"overtones"

"overtook"

"overtop"

"overtopped"

"overtopping"

"overtops"

"overtrain"

"overtrained"

"overtraining"

"overtrains"

"overture"

"overtured"

"overtures"

"overturing"

"overturn"

"overturned"

"overturning"

"overturns"

"overuse"

"overused"

"overuses"

"overusing"

"overvalue"

"overvalued"

"overvalues"

"overvaluing"

"overview"

"overviews"

"overviolent"

"overwealthy"

"overween"

"overweening"

"overweeningly"

"overweens"

"overweigh"

"overweighed"

"overweighing"

"overweighs"

"overweight"

"overwhelm"

"overwhelmed"

"overwhelming"

"overwhelmingly"

"overwhelms"

"overwilling"

"overwillingly"

"overwise"

"overwork"

"overworked"

"overworking"

"overworks"

"overwound"

"overwrite"

"overwrites"

"overwriting"

"overwritten"

"overwrote"

"overwrought"

"overzealous"

"overzealously"

"overzealousness"

"ovid"

"oviduct"

"oviducts"

"oviet"

"oviform"

"ovine"

"ovines"

"oviparity"

"oviparous"

"oviparously"

"oviposits"

"ovoid"

"ovoidal"

"ovoids"

"ovolo"

"ovular"

"ovulary"

"ovulate"

"ovulated"

"ovulates"

"ovulating"

"ovulation"

"ovulation dates"

"ovulation tracker"

"ovulations"

"ovulatory"

"ovule"

"ovules"

"ovum"

"ow"

"owe"

"owed"

"owes"

"owing"

"owl"

"owlet"

"owlets"

"owlish"

"owlishly"

"owllike"

"owls"

"own"

"ownable"

"owned"

"owner"

"ownerless"

"owners"

"ownership"

"ownerships"

"owning"

"owns"

"ox"

"oxalic"

"oxalis"

"oxalises"

"oxblood"

"oxbloods"

"oxbow"

"oxbows"

"oxcart"

"oxcarts"

"oxen"

"oxes"

"oxeye"

"oxeyes"

"oxford"

"oxfords"

"oxgall"

"oxheart"

"oxhearts"

"oxidant"

"oxidants"

"oxidate"

"oxidating"

"oxidation"

"oxidations"

"oxidative"

"oxidatively"

"oxide"

"oxides"

"oxidic"

"oxidise"

"oxidizable"

"oxidization"

"oxidizations"

"oxidize"

"oxidized"

"oxidizer"

"oxidizers"

"oxidizes"

"oxidizing"

"oxlip"

"oxlips"

"oxtail"

"oxtails"

"oxter"

"oxters"

"oxtongue"

"oxtongues"

"oxy"

"oxyacetylene"

"oxygen"

"oxygenate"

"oxygenated"

"oxygenates"

"oxygenating"

"oxygenation"

"oxygenic"

"oxygenize"

"oxygenizing"

"oxygenous"

"oxygens"

"oxyhydrogen"

"oxymoron"

"oyer"

"oyers"

"oyes"

"oyez"

"oyster"

"oystered"

"oysterer"

"oysterers"

"oysteries"

"oystering"

"oysterman"

"oystermen"

"oysters"

"oysterwoman"

"oysterwomen"

"oz"

"ozonator"

"ozone"

"ozones"

"ozonic"

"ozonise"

"ozonization"

"ozonize"

"ozonized"

"ozonizer"

"ozonizers"

"ozonizes"

"ozonizing"

"ozonous"

"pa"

"pablum"

"pabulum"

"pabulums"

"pac"

"pace"

"paced"

"pacemaker"

"pacemakers"

"pacemaking"

"pacer"

"pacers"

"paces"

"pacesetter"

"pacesetters"

"pacesetting"

"pachisi"

"pachyderm"

"pachydermatous"

"pachyderms"

"pachymeter"

"pachysandra"

"pachysandras"

"pacifiable"

"pacific"

"pacifica"

"pacifically"

"pacification"

"pacified"

"pacifier"

"pacifiers"

"pacifies"

"pacifism"

"pacifisms"

"pacifist"

"pacifists"

"pacify"

"pacifying"

"pacing"

"pack"

"packable"

"package"

"package inserts"

"packaged"

"packager"

"packagers"

"packages"

"packaging"

"packaging insert"

"packed"

"packer"

"packers"

"packet"

"packeted"

"packeting"

"packets"

"packhorse"

"packhorses"

"packing"

"packinghouse"

"packings"

"packman"

"packmen"

"packs"

"packsack"

"packsacks"

"packsaddle"

"packsaddles"

"packthread"

"packthreads"

"pacs"

"pact"

"pacta"

"pacts"

"pad"

"padded"

"paddies"

"padding"

"paddings"

"paddle"

"paddled"

"paddler"

"paddlers"

"paddles"

"paddling"

"paddlings"

"paddock"

"paddocked"

"paddocking"

"paddocks"

"paddy"

"padishah"

"padishahs"

"padlock"

"padlocked"

"padlocking"

"padlocks"

"padre"

"padres"

"padri"

"padrone"

"pads"

"padshah"

"paean"

"paeanisms"

"paeans"

"paedology"

"paedophobia"

"paedotrophy"

"paella"

"paellas"

"paeons"

"pagan"

"pagandom"

"pagandoms"

"paganish"

"paganism"

"paganisms"

"paganist"

"paganists"

"paganize"

"paganized"

"paganizer"

"paganizes"

"paganizing"

"pagans"

"page"

"pageant"

"pageantry"

"pageants"

"pageboy"

"pageboys"

"paged"

"pagers"

"pages"

"pagesize"

"paginal"

"paginate"

"paginated"

"paginates"

"paginating"

"pagination"

"paging"

"pagoda"

"pagodas"

"paid"

"paid account"

"paid version"

"paidonosology"

"pail"

"pailful"

"pailfuls"

"pails"

"pailsful"

"pain"

"paine"

"pained"

"painful"

"painfuller"

"painfully"

"painfulness"

"paining"

"painkiller"

"painkillers"

"painkilling"

"painless"

"painlessly"

"painlessness"

"pains"

"painstaking"

"painstakingly"

"paint"

"paintbrush"

"paintbrushes"

"painted"

"painter"

"painters"

"paintier"

"paintiest"

"painting"

"paintings"

"paints"

"painty"

"pair"

"paired"

"pairing"

"pairings"

"pairs"

"paisan"

"paisano"

"paisanos"

"paisans"

"paisley"

"paisleys"

"pajama"

"pajamaed"

"pajamas"

"pakistan"

"pakistani"

"pakistanis"

"pal"

"palace"

"palaced"

"palaces"

"paladin"

"paladins"

"palaeoanthropology"

"palaeobiology"

"palaeoclimatology"

"palaeolimnology"

"palaeontology"

"palaeopedology"

"palanquin"

"palanquins"

"palatability"

"palatable"

"palatably"

"palatal"

"palate"

"palates"

"palatial"

"palatinate"

"palatinates"

"palatine"

"palatines"

"palaver"

"palavered"

"palavering"

"palavers"

"palazzi"

"palazzo"

"pale"

"paled"

"paleface"

"palefaces"

"palely"

"paleness"

"paleo"

"paleobotany"

"paleocene"

"paleographer"

"paleographers"

"paleographic"

"paleographical"

"paleography"

"paleontologist"

"paleontologists"

"paleontology"

"paleozoic"

"paler"

"pales"

"palest"

"palestine"

"palestinian"

"palestinians"

"palets"

"palette"

"palettes"

"palfrey"

"palfreys"

"palier"

"palimpsest"

"palimpsests"

"palindrome"

"palindromes"

"palindromic"

"palindromically"

"paling"

"palings"

"palinode"

"palinodes"

"palisade"

"palisaded"

"palisades"

"palisading"

"palish"

"pall"

"palladia"

"palladium"

"palladiums"

"pallbearer"

"pallbearers"

"palled"

"pallet"

"pallets"

"pallette"

"pallettes"

"palliate"

"palliated"

"palliates"

"palliating"

"palliation"

"palliations"

"palliative"

"palliatively"

"pallid"

"pallidly"

"pallier"

"palling"

"pallograph"

"pallor"

"pallors"

"palls"

"pally"

"palm"

"palmate"

"palmature"

"palmed"

"palmer"

"palmers"

"palmettes"

"palmetto"

"palmettoes"

"palmettos"

"palmier"

"palmiest"

"palming"

"palmist"

"palmistry"

"palmists"

"palmitate"

"palms"

"palmy"

"palmyra"

"palmyras"

"palomino"

"palominos"

"palooka"

"palookas"

"palpability"

"palpable"

"palpably"

"palpal"

"palpate"

"palpated"

"palpates"

"palpating"

"palpation"

"palpations"

"palpator"

"palpators"

"palpitate"

"palpitated"

"palpitates"

"palpitating"

"palpitation"

"palpitations"

"palps"

"palpus"

"pals"

"palsied"

"palsies"

"palsy"

"palsying"

"palter"

"paltered"

"paltering"

"palters"

"paltrier"

"paltriest"

"paltrily"

"paltriness"

"paltry"

"palynology"

"pam"

"pampa"

"pampas"

"pampean"

"pamper"

"pampered"

"pamperer"

"pamperers"

"pampering"

"pampers"

"pamphlet"

"pamphleteer"

"pamphleteers"

"pamphlets"

"pan"

"panacea"

"panacean"

"panaceas"

"panache"

"panaches"

"panama"

"panamanian"

"panamanians"

"panamas"

"panatella"

"panatellas"

"pancake"

"pancaked"

"pancakes"

"pancaking"

"panchromatic"

"pancreas"

"pancreases"

"pancreatic"

"panda"

"pandas"

"pandemic"

"pandemics"

"pandemonium"

"pander"

"pandered"

"panderer"

"panderers"

"pandering"

"panders"

"pandit"

"pandits"

"pandora"

"pandoras"

"pandowdies"

"pandowdy"

"pane"

"paned"

"panegyric"

"panegyrical"

"panegyrics"

"panegyrist"

"panegyrists"

"panegyrize"

"panegyrized"

"panegyrizes"

"panegyrizing"

"panel"

"paneled"

"paneling"

"panelings"

"panelist"

"panelists"

"panelled"

"panelling"

"panels"

"panes"

"panful"

"panfuls"

"pang"

"panga"

"panged"

"panging"

"pangolin"

"pangolins"

"pangs"

"panhandle"

"panhandled"

"panhandler"

"panhandlers"

"panhandles"

"panhandling"

"panic"

"Panic attack"

"panic attacks"

"panicked"

"panickier"

"panickiest"

"panicking"

"panicky"

"panicle"

"panicled"

"panicles"

"panics"

"panier"

"panish"

"panjandrum"

"panjandrums"

"panned"

"pannier"

"panniers"

"pannikin"

"pannikins"

"panning"

"panocha"

"panophobia"

"panoplies"

"panoply"

"panorama"

"panoramas"

"panoramic"

"panoramically"

"panpipe"

"panpipes"

"pans"

"pansies"

"pansophies"

"pansy"

"pant"

"pantaloons"

"panted"

"pantheism"

"pantheist"

"pantheistic"

"pantheistical"

"pantheists"

"pantheon"

"pantheons"

"panther"

"panthers"

"pantie"

"panties"

"panting"

"pantingly"

"pantochronometer"

"pantograph"

"pantomime"

"pantomimed"

"pantomimes"

"pantomimic"

"pantomiming"

"pantomimist"

"pantomimists"

"pantophobia"

"pantries"

"pantry"

"pants"

"pantsuit"

"pantsuits"

"panty"

"pantywaist"

"pantywaists"

"panzer"

"panzers"

"pap"

"papa"

"papacies"

"papacy"

"papain"

"papains"

"papal"

"papally"

"papas"

"papaw"

"papaws"

"papaya"

"papayan"

"papayas"

"paper"

"paper medical record"

"paperback"

"paperbacks"

"paperboard"

"paperboards"

"paperboy"

"paperboys"

"papered"

"paperer"

"paperers"

"paperhanger"

"paperhangers"

"paperhanging"

"papering"

"papers"

"paperweight"

"paperweights"

"paperwork"

"papery"

"papier"

"papilla"

"papillae"

"papillary"

"papillate"

"papillons"

"papist"

"papistries"

"papistry"

"papists"

"papoose"

"papooses"

"pappies"

"pappooses"

"pappy"

"paprika"

"paprikas"

"paps"

"papua"

"papuan"

"papuans"

"papular"

"papule"

"papules"

"papyral"

"papyri"

"papyrology"

"papyrus"

"papyruses"

"par"

"para"

"parable"

"parables"

"parabola"

"parabolas"

"parabolic"

"parachute"

"parachuted"

"parachutes"

"parachuting"

"parachutist"

"parachutists"

"parade"

"paraded"

"parader"

"paraders"

"parades"

"paradigm"

"paradigms"

"parading"

"paradisal"

"paradise"

"paradises"

"paradisiacal"

"paradisiacally"

"paradox"

"paradoxes"

"paradoxical"

"paradoxically"

"paraffin"

"paraffine"

"paraffined"

"paraffinic"

"paraffins"

"parafoil"

"paragon"

"paragoning"

"paragons"

"paragraph"

"paragraphed"

"paragraphing"

"paragraphs"

"paraguay"

"paraguayan"

"paraguayans"

"parakeet"

"parakeets"

"paralegal"

"parallax"

"parallaxes"

"parallel"

"paralleled"

"paralleling"

"parallelism"

"parallelled"

"parallelling"

"parallelogram"

"parallelograms"

"parallels"

"paralyse"

"paralyses"

"paralysis"

"paralytic"

"paralytica"

"paralytical"

"paralyzant"

"paralyzation"

"paralyze"

"paralyzed"

"paralyzer"

"paralyzers"

"paralyzes"

"paralyzing"

"paralyzingly"

"paramecia"

"paramecium"

"parameciums"

"paramedic"

"paramedical"

"paramedics"

"parameter"

"parameterization"

"parameters"

"parametric"

"paramilitary"

"paramount"

"paramountly"

"paramour"

"paramours"

"paranoia"

"paranoiac"

"paranoiacs"

"paranoias"

"paranoid"

"paranoids"

"paranormal"

"paranormality"

"paranormally"

"parapet"

"parapets"

"paraphernalia"

"paraphrase"

"paraphrased"

"paraphraser"

"paraphrasers"

"paraphrases"

"paraphrasing"

"paraplegia"

"paraplegic"

"paraplegics"

"paraprofessional"

"paraprofessionals"

"parapsychologies"

"parapsychologist"

"parapsychologists"

"parapsychology"

"paraquat"

"paraquats"

"paras"

"parasite"

"parasites"

"parasitic"

"parasitical"

"parasitically"

"parasiticidal"

"parasiticide"

"parasiticidic"

"parasitism"

"parasitization"

"parasitize"

"parasitized"

"parasitizes"

"parasitizing"

"parasitologic"

"parasitological"

"parasitologies"

"parasitologist"

"parasitology"

"parasol"

"parasols"

"parasympathetic"

"parathion"

"parathyroid"

"parathyroidal"

"parathyroids"

"paratroop"

"paratrooper"

"paratroopers"

"paratroops"

"paratyphoid"

"paratypic"

"parboil"

"parboiled"

"parboiling"

"parboils"

"parcel"

"parceled"

"parceling"

"parcelled"

"parcelling"

"parcels"

"parch"

"parched"

"parches"

"parching"

"parchment"

"parchments"

"pard"

"pardner"

"pardners"

"pardon"

"pardonable"

"pardonably"

"pardoned"

"pardoner"

"pardoners"

"pardoning"

"pardons"

"pare"

"pared"

"paregoric"

"parent"

"parentage"

"parental"

"parented"

"parentheses"

"parenthesis"

"parenthesize"

"parenthetic"

"parenthetical"

"parenthetically"

"parenthood"

"parenticide"

"parenting"

"parentis"

"parents"

"parer"

"parers"

"pares"

"paresis"

"paretic"

"paretics"

"pareve"

"parfait"

"parfaits"

"pargetting"

"pariah"

"pariahs"

"parietal"

"parietals"

"parimutuel"

"parimutuels"

"paring"

"parings"

"paris"

"parises"

"parish"

"parishes"

"parishioner"

"parishioners"

"parisian"

"parisians"

"parities"

"parity"

"park"

"parka"

"parkas"

"parked"

"parker"

"parkers"

"parking"

"parking timer"

"parkings"

"parkinson"

"parkinsonian"

"parkinsonism"

"parkland"

"parklands"

"parks"

"parkway"

"parkways"

"parlance"

"parlances"

"parlay"

"parlayed"

"parlayer"

"parlayers"

"parlaying"

"parlays"

"parley"

"parleyed"

"parleyer"

"parleyers"

"parleying"

"parleys"

"parliament"

"parliamentarian"

"parliamentarians"

"parliamentary"

"parliaments"

"parlor"

"parlors"

"parlour"

"parlours"

"parlous"

"parlously"

"parmesan"

"parmigiana"

"parochial"

"parochialism"

"parochially"

"parodic"

"parodied"

"parodies"

"parodist"

"parodists"

"parody"

"parodying"

"paroemiology"

"parolable"

"parole"

"paroled"

"parolee"

"parolees"

"paroler"

"parolers"

"paroles"

"paroling"

"parols"

"paroquets"

"parousiamania"

"paroxysm"

"paroxysmal"

"paroxysmic"

"paroxysms"

"parquet"

"parqueted"

"parqueting"

"parquetry"

"parquets"

"parrakeet"

"parrakeets"

"parred"

"parricidal"

"parricide"

"parricides"

"parried"

"parries"

"parring"

"parrot"

"parroted"

"parroter"

"parroters"

"parroting"

"parrots"

"parroty"

"parry"

"parrying"

"pars"

"parsable"

"parse"

"parsec"

"parsecs"

"parsed"

"parser"

"parsers"

"parses"

"parsimonious"

"parsimoniously"

"parsimoniousness"

"parsimony"

"parsing"

"parsley"

"parsleys"

"parsnip"

"parsnips"

"parson"

"parsonage"

"parsonages"

"parsons"

"part"

"partake"

"partaken"

"partaker"

"partakers"

"partakes"

"partaking"

"parte"

"parted"

"parterre"

"parterres"

"parthenogeneses"

"parthenogenesis"

"parthenogenetic"

"parthenogenic"

"parthenology"

"parthenon"

"parthophobia"

"parti"

"partial"

"partialities"

"partiality"

"partially"

"partials"

"partible"

"participant"

"participants"

"participate"

"participated"

"participates"

"participating"

"participating pharmacies"

"participation"

"participator"

"participators"

"participatory"

"participial"

"participle"

"participles"

"particle"

"particles"

"particular"

"particularities"

"particularity"

"particularize"

"particularized"

"particularizes"

"particularizing"

"particularly"

"particulars"

"particulate"

"partied"

"parties"

"partim"

"parting"

"partings"

"partisan"

"partisans"

"partisanship"

"partita"

"partitas"

"partition"

"partitioned"

"partitioning"

"partitions"

"partitive"

"partizans"

"partly"

"partner"

"partnered"

"partnering"

"partners"

"partnership"

"partnerships"

"partook"

"partridge"

"partridges"

"parts"

"parturition"

"parturitions"

"partway"

"party"

"partying"

"parve"

"parvenu"

"parvenue"

"parvenus"

"pas"

"pasadena"

"pascal"

"paschal"

"paseo"

"paseos"

"pasha"

"pashas"

"pashes"

"paso"

"pasquinade"

"pasquinades"

"pass"

"passable"

"passably"

"passage"

"passaged"

"passages"

"passageway"

"passageways"

"passaging"

"passant"

"passbook"

"passbooks"

"passe"

"passed"

"passee"

"passel"

"passels"

"passenger"

"passengers"

"passer"

"passerby"

"passerine"

"passers"

"passersby"

"passes"

"passible"

"passim"

"passimeter"

"passing"

"passingly"

"passings"

"passion"

"passionate"

"passionately"

"passionless"

"passions"

"passive"

"passively"

"passiveness"

"passives"

"passivity"

"passkey"

"passkeys"

"passover"

"passovers"

"passport"

"passports"

"passway"

"password"

"Password protected"

"Password protection"

"passwords"

"past"

"pasta"

"pastas"

"paste"

"pasteboard"

"pasteboards"

"pasted"

"pastel"

"pastelist"

"pastelists"

"pastellist"

"pastellists"

"pastels"

"paster"

"pastern"

"pasterns"

"pasters"

"pastes"

"pasteur"

"pasteurization"

"pasteurize"

"pasteurized"

"pasteurizer"

"pasteurizers"

"pasteurizes"

"pasteurizing"

"pastiche"

"pastiches"

"pastier"

"pasties"

"pastiest"

"pastille"

"pastilles"

"pastils"

"pastime"

"pastimes"

"pastina"

"pastinas"

"pastiness"

"pasting"

"pastor"

"pastoral"

"pastorale"

"pastorales"

"pastoralism"

"pastoralist"

"pastorals"

"pastorate"

"pastorates"

"pastored"

"pastoring"

"pastors"

"pastorship"

"pastrami"

"pastramis"

"pastries"

"pastry"

"pasts"

"pasturage"

"pastural"

"pasture"

"pastured"

"pasturer"

"pasturers"

"pastures"

"pasturing"

"pasty"

"pat"

"pataphysics"

"patch"

"patchable"

"patched"

"patcher"

"patchers"

"patches"

"patchier"

"patchiest"

"patchily"

"patchiness"

"patching"

"patchwork"

"patchy"

"pate"

"pated"

"patella"

"patellae"

"patellar"

"patellas"

"patellate"

"paten"

"patencies"

"patency"

"patens"

"patent"

"patentability"

"patentable"

"patentably"

"patented"

"patentee"

"patentees"

"patenting"

"patently"

"patentor"

"patentors"

"patents"

"pater"

"paterfamilias"

"paterfamiliases"

"paternal"

"paternalism"

"paternalistic"

"paternally"

"paternities"

"paternity"

"paternoster"

"paternosters"

"paters"

"pates"

"path"

"pathetic"

"pathetically"

"pathfinder"

"pathfinders"

"pathless"

"pathogen"

"pathogeneses"

"pathogenesis"

"pathogenetic"

"pathogenic"

"pathogenically"

"pathogenicity"

"pathogens"

"pathogeny"

"pathologic"

"pathological"

"pathologically"

"pathologies"

"pathologist"

"pathologists"

"pathology"

"pathomania"

"pathophobia"

"pathophysiology"

"pathos"

"paths"

"pathway"

"pathways"

"patience"

"patiences"

"patient"

"patient care"

"patient data"

"patient medical record"

"patient photo"

"patient record"

"Patient-centered"

"patient-centered care"

"patienter"

"patientest"

"patiently"

"patients"

"patina"

"patinas"

"patio"

"patios"

"patly"

"patness"

"patnesses"

"patois"

"patriarch"

"patriarchal"

"patriarchate"

"patriarchates"

"patriarchies"

"patriarchs"

"patriarchy"

"patricia"

"patrician"

"patricians"

"patricidal"

"patricide"

"patricides"

"patrick"

"patrilineal"

"patrilinear"

"patrilinies"

"patriliny"

"patrimonial"

"patrimonially"

"patrimonies"

"patrimonium"

"patrimony"

"patriot"

"patriotic"

"patriotically"

"patriotism"

"patriots"

"patristic"

"patrol"

"patrolled"

"patroller"

"patrollers"

"patrolling"

"patrolman"

"patrolmen"

"patrology"

"patrols"

"patrolwoman"

"patrolwomen"

"patron"

"patronage"

"patronal"

"patroness"

"patronesses"

"patronize"

"patronized"

"patronizer"

"patronizers"

"patronizes"

"patronizing"

"patronly"

"patrons"

"patronymic"

"patronymically"

"patronymics"

"patroon"

"pats"

"patsies"

"patsy"

"patted"

"pattee"

"patter"

"pattered"

"patterer"

"patterers"

"pattering"

"pattern"

"patterned"

"patterning"

"patterns"

"patters"

"pattie"

"patties"

"patting"

"patty"

"pattypan"

"pattypans"

"patulous"

"paucities"

"paucity"

"paul"

"pauline"

"paunch"

"paunches"

"paunchier"

"paunchiest"

"paunchiness"

"paunchy"

"pauper"

"paupered"

"paupering"

"pauperism"

"pauperization"

"pauperize"

"pauperized"

"pauperizes"

"pauperizing"

"paupers"

"pause"

"paused"

"pauser"

"pausers"

"pauses"

"pausing"

"pavan"

"pavane"

"pavanes"

"pavans"

"pave"

"paved"

"pavement"

"pavements"

"paver"

"pavers"

"paves"

"pavilion"

"pavilioned"

"pavilions"

"paving"

"pavings"

"pavlov"

"pavlovian"

"paw"

"pawed"

"pawer"

"pawers"

"pawing"

"pawky"

"pawl"

"pawls"

"pawn"

"pawnable"

"pawnbroker"

"pawnbrokers"

"pawnbroking"

"pawned"

"pawnee"

"pawnees"

"pawner"

"pawners"

"pawning"

"pawnor"

"pawns"

"pawnshop"

"pawnshops"

"pawpaw"

"pawpaws"

"paws"

"pax"

"paxes"

"pay"

"payability"

"payable"

"payably"

"payback"

"paycheck"

"paychecks"

"payday"

"paydays"

"payed"

"payee"

"payees"

"payer"

"payers"

"paying"

"payload"

"payloads"

"paymaster"

"paymasters"

"payment"

"payment innovation"

"payments"

"paynim"

"paynims"

"payoff"

"payoffs"

"payola"

"payolas"

"payors"

"payout"

"payroll"

"payrolls"

"pays"

"pbx"

"pc"

"pct"

"PDF"

"PDR Network"

"pea"

"peace"

"Peace Night"

"peace of mind"

"peaceable"

"peaceably"

"peaced"

"peaceful"

"peaceful mood"

"peacefully"

"peacefulness"

"peacekeeper"

"peacekeepers"

"peacekeeping"

"peacemaker"

"peacemakers"

"peacemaking"

"peaces"

"peacetime"

"peach"

"peached"

"peacher"

"peaches"

"peachier"

"peachiest"

"peachy"

"peacing"

"peacoat"

"peacoats"

"peacock"

"peacocked"

"peacockier"

"peacocking"

"peacocks"

"peafowl"

"peafowls"

"peahen"

"peahens"

"peak"

"peaked"

"peakedness"

"peakier"

"peakiest"

"peaking"

"peakish"

"peaks"

"peaky"

"peal"

"pealed"

"pealing"

"peals"

"pean"

"peanut"

"peanuts"

"pear"

"pearl"

"pearled"

"pearler"

"pearlers"

"pearlier"

"pearliest"

"pearling"

"pearlite"

"pearlites"

"pearls"

"pearly"

"pears"

"peart"

"pearter"

"peartly"

"peas"

"peasant"

"peasantry"

"peasants"

"pease"

"peases"

"peashooter"

"peat"

"peatier"

"peatiest"

"peats"

"peaty"

"peavey"

"peaveys"

"peavies"

"peavy"

"pebble"

"pebbled"

"pebbles"

"pebblier"

"pebbliest"

"pebbling"

"pebbly"

"pecan"

"pecans"

"peccable"

"peccadillo"

"peccadilloes"

"peccadillos"

"peccaries"

"peccary"

"peccavi"

"peccavis"

"peck"

"pecked"

"pecker"

"peckers"

"peckier"

"pecking"

"pecks"

"pecky"

"pectic"

"pectin"

"pectinous"

"pectins"

"pectoral"

"pectorals"

"pectoris"

"peculate"

"peculated"

"peculates"

"peculating"

"peculation"

"peculations"

"peculator"

"peculators"

"peculiar"

"peculiarities"

"peculiarity"

"peculiarly"

"peculiars"

"pecuniarily"

"pecuniary"

"ped"

"pedagog"

"pedagogic"

"pedagogical"

"pedagogically"

"pedagogics"

"pedagogies"

"pedagogs"

"pedagogue"

"pedagogues"

"pedagogy"

"pedal"

"pedaled"

"pedaling"

"pedalled"

"pedalling"

"pedals"

"pedant"

"pedantic"

"pedantically"

"pedantries"

"pedantry"

"pedants"

"peddlar"

"peddle"

"peddled"

"peddler"

"peddlers"

"peddlery"

"peddles"

"peddling"

"pederast"

"pederastic"

"pederastically"

"pederasties"

"pederasts"

"pederasty"

"pedes"

"pedestal"

"pedestaled"

"pedestals"

"pedestrian"

"pedestrianism"

"pedestrians"

"pediatric"

"pediatric algorithm"

"pediatric cardiologist"

"pediatric ECG"

"pediatric EKG"

"pediatric electrocardiogram"

"pediatric emergency medicine"

"pediatric ICU"

"pediatric information"

"pediatric patients"

"pediatrician"

"pediatricians"

"pediatrics"

"pedicab"

"pedicabs"

"pediculophobia"

"pedicure"

"pedicured"

"pedicures"

"pedicuring"

"pedicurist"

"pedicurists"

"pedigree"

"pedigreed"

"pedigrees"

"pediment"

"pediments"

"pedlar"

"pedler"

"pedologies"

"pedology"

"pedometer"

"pedometers"

"pedophile"

"pedophilia"

"pedophiliac"

"pedophilic"

"pedro"

"pedros"

"peds"

"peduncle"

"peduncles"

"pedunculated"

"pee"

"peed"

"peeing"

"peek"

"peekaboo"

"peekaboos"

"peeked"

"peeking"

"peeks"

"peel"

"peelable"

"peeled"

"peeler"

"peelers"

"peeling"

"peelings"

"peels"

"peen"

"peened"

"peening"

"peens"

"peep"

"peeped"

"peeper"

"peepers"

"peephole"

"peepholes"

"peeping"

"peeps"

"peepshow"

"peepshows"

"peer"

"peerage"

"peerages"

"peered"

"peeress"

"peeresses"

"peering"

"peerless"

"peerlessly"

"peers"

"peery"

"pees"

"peeve"

"peeved"

"peeves"

"peeving"

"peevish"

"peevishly"

"peevishness"

"peewee"

"peewees"

"peewit"

"peewits"

"peg"

"pegasus"

"pegboard"

"pegboards"

"pegbox"

"pegboxes"

"pegged"

"pegging"

"peggy"

"pegless"

"pegmatite"

"pegmatitic"

"pegs"

"peignoir"

"peignoirs"

"peins"

"peiping"

"peirameter"

"pejoration"

"pejorative"

"pejoratively"

"pejoratives"

"pekans"

"peke"

"pekes"

"pekin"

"pekinese"

"peking"

"pekingese"

"pekins"

"pekoe"

"pekoes"

"pelage"

"pelagic"

"pelf"

"pelfs"

"pelican"

"pelicans"

"pellagra"

"pellagras"

"pellagrous"

"pellet"

"pelleted"

"pelleting"

"pelletize"

"pelletized"

"pelletizes"

"pelletizing"

"pellets"

"pellmell"

"pellmells"

"pellucid"

"pellucidly"

"pelology"

"pelorias"

"pelt"

"pelted"

"pelter"

"pelters"

"pelting"

"pelts"

"pelves"

"pelvic"

"pelvics"

"pelvimeter"

"pelvis"

"pelvises"

"pemmican"

"pemmicans"

"pen"

"penal"

"penalities"

"penalization"

"penalize"

"penalized"

"penalizes"

"penalizing"

"penally"

"penalties"

"penalty"

"penance"

"penances"

"penancing"

"penang"

"penates"

"pence"

"penchant"

"penchants"

"pencil"

"penciled"

"penciler"

"pencilers"

"penciling"

"pencilled"

"pencilling"

"pencils"

"pend"

"pendant"

"pendants"

"pended"

"pendency"

"pendent"

"pendently"

"pendents"

"pending"

"pends"

"pendular"

"pendulous"

"pendulum"

"pendulums"

"peneplain"

"peneplains"

"penes"

"penetrable"

"penetrably"

"penetrate"

"penetrated"

"penetrates"

"penetrating"

"penetratingly"

"penetration"

"penetrations"

"penetrative"

"penetrator"

"penetrators"

"penetrometer"

"penguin"

"penguins"

"penholder"

"penicillin"

"penicillinic"

"penicillium"

"penile"

"peninsula"

"peninsular"

"peninsulas"

"penis"

"penises"

"penitence"

"penitent"

"penitential"

"penitentiaries"

"penitentiary"

"penitently"

"penitents"

"penknife"

"penknives"

"penlight"

"penlights"

"penlite"

"penlites"

"penman"

"penmanship"

"penmen"

"penna"

"pennae"

"penname"

"pennames"

"pennant"

"pennants"

"pennate"

"penned"

"penner"

"penners"

"penney"

"pennies"

"penniless"

"pennilessness"

"pennines"

"penning"

"pennon"

"pennoned"

"pennons"

"pennsylvania"

"pennsylvanian"

"pennsylvanians"

"penny"

"pennyroyal"

"pennyroyals"

"pennyweight"

"pennyweights"

"penologies"

"penologist"

"penologists"

"penology"

"penpoint"

"penpoints"

"pens"

"pense"

"pensees"

"pension"

"pensionable"

"pensionary"

"pensione"

"pensioned"

"pensioner"

"pensioners"

"pensiones"

"pensioning"

"pensionless"

"pensions"

"pensive"

"pensively"

"pensiveness"

"penstock"

"penstocks"

"pent"

"pentacle"

"pentacles"

"pentad"

"pentadactyl"

"pentadactylate"

"pentadactylism"

"pentads"

"pentagon"

"pentagonal"

"pentagonally"

"pentagons"

"pentalogies"

"pentameter"

"pentameters"

"pentarch"

"pentateuchal"

"pentathlon"

"pentathlons"

"pentecost"

"pentecostal"

"pentheraphobia"

"penthouse"

"penthouses"

"pentobarbital"

"pentobarbitone"

"pentothal"

"penuche"

"penuches"

"penult"

"penultimate"

"penults"

"penumbra"

"penumbrae"

"penumbras"

"penuries"

"penurious"

"penuriously"

"penuriousness"

"penury"

"peon"

"peonage"

"peonages"

"peones"

"peonies"

"peonism"

"peonisms"

"peons"

"peony"

"people"

"peopled"

"peopler"

"peoplers"

"peoples"

"peopling"

"peotillomania"

"pep"

"peplum"

"pepped"

"pepper"

"pepperbox"

"peppercorn"

"peppercorns"

"peppered"

"pepperer"

"pepperers"

"pepperiness"

"peppering"

"peppermint"

"peppermints"

"pepperoni"

"peppers"

"peppertree"

"peppery"

"peppier"

"peppiest"

"peppily"

"peppiness"

"pepping"

"peppy"

"peps"

"pepsi"

"pepsin"

"pepsine"

"pepsines"

"pepsins"

"peptic"

"peptics"

"peptide"

"peptids"

"per"

"peradventure"

"perambulate"

"perambulated"

"perambulates"

"perambulating"

"perambulation"

"perambulations"

"perambulator"

"perambulators"

"percale"

"percales"

"perceivable"

"perceivably"

"perceive"

"perceived"

"perceiver"

"perceivers"

"perceives"

"perceiving"

"percent"

"percentage"

"percentaged"

"percentages"

"percenter"

"percentile"

"percentiles"

"percents"

"percept"

"perceptibility"

"perceptible"

"perceptibly"

"perception"

"perceptions"

"perceptive"

"perceptively"

"perceptiveness"

"perceptivity"

"percepts"

"perceptual"

"perceptually"

"perch"

"perchance"

"perched"

"percher"

"perchers"

"perches"

"perching"

"percipience"

"percipient"

"percolate"

"percolated"

"percolates"

"percolating"

"percolation"

"percolator"

"percolators"

"percussed"

"percusses"

"percussing"

"percussion"

"percussional"

"percussionist"

"percussionists"

"percussions"

"percussor"

"perdition"

"perdu"

"perdue"

"perdues"

"perdurability"

"perdurable"

"perdus"

"perdy"

"pere"

"peregrinate"

"peregrination"

"peregrinations"

"peregrins"

"peremption"

"peremptorily"

"peremptoriness"

"peremptory"

"perennial"

"perennially"

"perennials"

"peres"

"perfect"

"perfectability"

"perfectas"

"perfected"

"perfecter"

"perfecters"

"perfectest"

"perfectibility"

"perfectible"

"perfecting"

"perfection"

"perfectionism"

"perfectionist"

"perfectionists"

"perfections"

"perfectly"

"perfectness"

"perfecto"

"perfectos"

"perfects"

"perfidies"

"perfidious"

"perfidiously"

"perfidy"

"perforate"

"perforated"

"perforates"

"perforating"

"perforation"

"perforations"

"perforator"

"perforators"

"perforce"

"perform"

"performable"

"performance"

"performances"

"performed"

"performer"

"performers"

"performing"

"performs"

"perfume"

"perfumed"

"perfumer"

"perfumeries"

"perfumers"

"perfumery"

"perfumes"

"perfuming"

"perfunctorily"

"perfunctoriness"

"perfunctory"

"perfusing"

"perfusion"

"pergola"

"pergolas"

"perhaps"

"perhapses"

"peri-menopause"

"pericardia"

"pericardial"

"pericarditis"

"pericardium"

"pericarps"

"pericles"

"pericynthion"

"peridot"

"peridots"

"perigee"

"perigees"

"perihelia"

"perihelial"

"perihelion"

"peril"

"periled"

"periling"

"perilled"

"perilling"

"perilous"

"perilously"

"perilousness"

"perils"

"perilune"

"perilunes"

"perimeter"

"perimeters"

"perimetry"

"perinea"

"perineal"

"perineum"

"period"

"periodic"

"periodical"

"periodically"

"periodicals"

"periodicity"

"periodontal"

"periodontia"

"periodontic"

"periodontics"

"periodontist"

"periodontitis"

"periodontology"

"periodontoses"

"periodontosis"

"periods"

"peripatetic"

"peripheral"

"peripherally"

"peripherals"

"peripheries"

"periphery"

"periphrases"

"periphrasis"

"perique"

"peris"

"periscope"

"periscopes"

"perish"

"perishability"

"perishable"

"perishableness"

"perishables"

"perishably"

"perished"

"perishes"

"perishing"

"peristalses"

"peristalsis"

"peristaltic"

"peristaltically"

"peristerophily"

"peristylar"

"peristyle"

"peristyles"

"peritonea"

"peritoneal"

"peritoneally"

"peritoneum"

"peritoneums"

"peritonital"

"peritonitic"

"peritonitis"

"periwig"

"periwigs"

"periwinkle"

"periwinkles"

"perjure"

"perjured"

"perjurer"

"perjurers"

"perjures"

"perjuries"

"perjuring"

"perjurious"

"perjuriously"

"perjury"

"perk"

"perked"

"perkier"

"perkiest"

"perkily"

"perkiness"

"perking"

"perkish"

"perks"

"perky"

"perlites"

"perlitic"

"perm"

"permafrost"

"permanence"

"permanencies"

"permanency"

"permanent"

"permanently"

"permanents"

"permeability"

"permeable"

"permeably"

"permeameter"

"permeate"

"permeated"

"permeates"

"permeating"

"permeation"

"permeations"

"permian"

"permissable"

"permissibility"

"permissible"

"permissibleness"

"permissibly"

"permission"

"permissions"

"permissive"

"permissively"

"permissiveness"

"permit"

"permits"

"permitted"

"permittee"

"permitting"

"perms"

"permutation"

"permutational"

"permutationist"

"permutationists"

"permutations"

"permute"

"permuted"

"permutes"

"permuting"

"pernicious"

"perniciously"

"perniciousness"

"peroration"

"perorations"

"peroxide"

"peroxided"

"peroxides"

"peroxiding"

"perpendicular"

"perpendicularity"

"perpendicularly"

"perpendiculars"

"perpetrate"

"perpetrated"

"perpetrates"

"perpetrating"

"perpetration"

"perpetrations"

"perpetrator"

"perpetrators"

"perpetual"

"perpetually"

"perpetualness"

"perpetuate"

"perpetuated"

"perpetuates"

"perpetuating"

"perpetuation"

"perpetuator"

"perpetuators"

"perpetuities"

"perpetuity"

"perpetuum"

"perplex"

"perplexed"

"perplexedly"

"perplexes"

"perplexing"

"perplexities"

"perplexity"

"perquisite"

"perquisites"

"perry"

"persecute"

"persecuted"

"persecutee"

"persecutes"

"persecuting"

"persecution"

"persecutions"

"persecutor"

"persecutors"

"perseverance"

"persevere"

"persevered"

"perseveres"

"persevering"

"persia"

"persian"

"persians"

"persiflage"

"persimmon"

"persimmons"

"persist"

"persistance"

"persisted"

"persistence"

"persistency"

"persistent"

"persistently"

"persister"

"persisters"

"persisting"

"persists"

"persnicketiness"

"persnickety"

"person"

"persona"

"personable"

"personableness"

"personably"

"personae"

"personage"

"personages"

"personal"

"personal business"

"Personal business app"

"Personal Health Record"

"Personal medical record"

"personal tracking"

"personal trainer"

"personalis"

"personalism"

"personalities"

"personality"

"personalization"

"personalize"

"personalized"

"personalized assistance"

"personalizes"

"personalizing"

"personally"

"personals"

"personalties"

"personalty"

"personas"

"personate"

"personation"

"personative"

"personator"

"personification"

"personifications"

"personified"

"personifier"

"personifies"

"personify"

"personifying"

"personnel"

"persons"

"perspective"

"perspectives"

"perspicacious"

"perspicaciously"

"perspicaciousness"

"perspicacity"

"perspicuity"

"perspicuous"

"perspicuously"

"perspicuousness"

"perspiration"

"perspiratory"

"perspire"

"perspired"

"perspires"

"perspiring"

"perspiry"

"persuadable"

"persuadably"

"persuade"

"persuaded"

"persuader"

"persuaders"

"persuades"

"persuading"

"persuasion"

"persuasions"

"persuasive"

"persuasively"

"persuasiveness"

"pert"

"pertain"

"pertained"

"pertaining"

"pertains"

"perter"

"pertest"

"pertinacious"

"pertinacity"

"pertinence"

"pertinencies"

"pertinency"

"pertinent"

"pertinently"

"pertly"

"pertness"

"perturb"

"perturbable"

"perturbation"

"perturbational"

"perturbations"

"perturbed"

"perturbing"

"perturbs"

"pertussis"

"peru"

"peruke"

"perukes"

"perusal"

"perusals"

"peruse"

"perused"

"peruser"

"perusers"

"peruses"

"perusing"

"peruvian"

"peruvians"

"pervade"

"pervaded"

"pervader"

"pervaders"

"pervades"

"pervading"

"pervasion"

"pervasive"

"pervasively"

"pervasiveness"

"perverse"

"perversely"

"perverseness"

"perversion"

"perversions"

"perversities"

"perversity"

"perversive"

"pervert"

"perverted"

"pervertedly"

"pervertedness"

"perverter"

"perverting"

"perverts"

"pervious"

"perviousness"

"peseta"

"pesetas"

"peskier"

"peskiest"

"peskily"

"peskiness"

"pesky"

"peso"

"pesos"

"pessimism"

"pessimist"

"pessimistic"

"pessimistically"

"pessimists"

"pest"

"pester"

"pestered"

"pesterer"

"pesterers"

"pestering"

"pesters"

"pesthole"

"pestholes"

"pesticidal"

"pesticide"

"pesticides"

"pestiferous"

"pestiferously"

"pestilence"

"pestilences"

"pestilent"

"pestilential"

"pestilentially"

"pestilently"

"pestle"

"pestled"

"pestles"

"pestology"

"pests"

"pet"

"petal"

"petaled"

"petalled"

"petals"

"petard"

"petards"

"petcock"

"petcocks"

"peter"

"petered"

"petering"

"peters"

"petersburg"

"petiolate"

"petiole"

"petioles"

"petit"

"petite"

"petites"

"petition"

"petitional"

"petitioned"

"petitionee"

"petitioner"

"petitioners"

"petitioning"

"petitions"

"petits"

"petnapping"

"petnappings"

"petrel"

"petrels"

"petri"

"petrifaction"

"petrification"

"petrified"

"petrifies"

"petrify"

"petrifying"

"petro"

"petrochemical"

"petrochemicals"

"petrochemistry"

"petrographer"

"petrographers"

"petrographic"

"petrographical"

"petrography"

"petrol"

"petrolatum"

"petroleous"

"petroleum"

"petrologic"

"petrological"

"petrologically"

"petrologist"

"petrologists"

"petrology"

"petrols"

"petrous"

"pets"

"petted"

"pettedly"

"petter"

"petters"

"petticoat"

"petticoats"

"pettier"

"pettiest"

"pettifog"

"pettifogged"

"pettifogger"

"pettifoggers"

"pettifoggery"

"pettifogging"

"pettifogs"

"pettily"

"pettiness"

"petting"

"pettish"

"pettishly"

"pettishness"

"petty"

"petulance"

"petulancy"

"petulant"

"petulantly"

"petunia"

"petunias"

"peugeot"

"pew"

"pewee"

"pewees"

"pewit"

"pewits"

"pews"

"pewter"

"pewterer"

"pewterers"

"pewters"

"peyote"

"peyotes"

"peyotl"

"peyotyl"

"peyotyls"

"pf"

"pfennig"

"pfennigs"

"phacometer"

"phaeton"

"phaetons"

"phage"

"phages"

"phagocyte"

"phagomania"

"phagophobia"

"phagosome"

"phalange"

"phalanges"

"phalanx"

"phalanxes"

"phalarope"

"phalaropes"

"phalli"

"phallic"

"phallically"

"phallism"

"phallist"

"phalloid"

"phallus"

"phalluses"

"phaneromania"

"phantasied"

"phantasies"

"phantasm"

"phantasmagoria"

"phantasmagorias"

"phantasmagoric"

"phantasmagorical"

"phantasmagories"

"phantasmagory"

"phantasms"

"phantast"

"phantasts"

"phantasy"

"phantom"

"phantomlike"

"phantoms"

"phaometer"

"pharaoh"

"pharaohs"

"pharisaic"

"pharisaical"

"pharisaically"

"pharisee"

"pharisees"

"pharm"

"pharmaceutic"

"pharmaceutical"

"pharmaceutically"

"pharmaceuticals"

"pharmaceutics"

"pharmacies"

"pharmacist"

"pharmacists"

"pharmacognosy"

"pharmacokinetic dosing"

"pharmacologic"

"pharmacological"

"pharmacologies"

"pharmacologist"

"pharmacologists"

"pharmacology"

"pharmacomania"

"pharmacometer"

"pharmacopeia"

"pharmacopeias"

"pharmacopoeia"

"pharmacopoeias"

"Pharmacovigilance"

"pharmacy"

"Pharmacy doctor benefits"

"pharology"

"pharyngal"

"pharyngeal"

"pharyngectomies"

"pharyngectomy"

"pharynges"

"pharyngitis"

"pharyngology"

"pharyngoscope"

"pharynx"

"pharynxes"

"phase"

"phaseal"

"phased"

"phaseout"

"phaseouts"

"phaser"

"phasers"

"phases"

"phasic"

"phasing"

"pheasant"

"pheasants"

"phenacetin"

"phengophobia"

"phenix"

"phenobarbital"

"phenocopies"

"phenocopy"

"phenol"

"phenolic"

"phenolics"

"phenological"

"phenologically"

"phenology"

"phenolphthalein"

"phenols"

"phenomena"

"phenomenal"

"phenomenology"

"phenomenon"

"phenomenons"

"phenothiazine"

"phenotype"

"phenotypes"

"phenotypic"

"phenotypical"

"phenotypically"

"phenylketonuria"

"phenylketonuric"

"pheromonal"

"pheromone"

"pheromones"

"phew"

"phi"

"phial"

"phials"

"philadelphia"

"philadelphian"

"philadelphians"

"philander"

"philandered"

"philanderer"

"philanderers"

"philandering"

"philanders"

"philanthropic"

"philanthropies"

"philanthropist"

"philanthropists"

"philanthropy"

"philatelic"

"philatelist"

"philatelists"

"philately"

"philematology"

"philharmonic"

"philharmonics"

"philip"

"philippic"

"philippics"

"philippine"

"philippines"

"philistine"

"philistines"

"phillumeny"

"philodendron"

"philodendrons"

"philol"

"philological"

"philologist"

"philologists"

"philology"

"philomel"

"philomels"

"philoprogenitive"

"philos"

"philosopher"

"philosophers"

"philosophic"

"philosophical"

"philosophically"

"philosophies"

"philosophize"

"philosophized"

"philosophizes"

"philosophizing"

"philosophy"

"philter"

"philtered"

"philtering"

"philters"

"philtre"

"philtred"

"philtres"

"phiz"

"phlebitis"

"phlebotomies"

"phlebotomy"

"Phlebotomy Certification exam"

"phlegm"

"phlegmatic"

"phlegmatical"

"phlegmatically"

"phlegmier"

"phlegmiest"

"phlegms"

"phlegmy"

"phloem"

"phlox"

"phloxes"

"phobia"

"phobias"

"phobic"

"phocomeli"

"phoebe"

"phoebes"

"phoenician"

"phoenicians"

"phoenix"

"phoenixes"

"phon"

"phonal"

"phonautograph"

"phone"

"phoned"

"phoneme"

"phonemes"

"phonemic"

"phonemically"

"phonendoscope"

"phones"

"phonetic"

"phonetically"

"phonetician"

"phoneticians"

"phonetics"

"phoney"

"phoneys"

"phoniatrics"

"phonic"

"phonically"

"phonics"

"phonier"

"phonies"

"phoniest"

"phonily"

"phoniness"

"phoning"

"phono"

"phonogram"

"phonogramically"

"phonogrammic"

"phonogrammically"

"phonograph"

"phonographic"

"phonographically"

"phonographs"

"phonological"

"phonologist"

"phonologists"

"phonology"

"phonomania"

"phonometer"

"phonons"

"phonophobia"

"phonophotography"

"phonoreception"

"phonoreceptor"

"phonos"

"phons"

"phony"

"phooey"

"phorometer"

"phosgene"

"phosgenes"

"phosphate"

"phosphates"

"phosphatic"

"phosphene"

"phosphor"

"phosphorescence"

"phosphorescent"

"phosphorescently"

"phosphoric"

"phosphorous"

"phosphors"

"phosphorus"

"photic"

"photics"

"photo"

"photobiology"

"photocatalyst"

"photocell"

"photocells"

"photochemical"

"photochemist"

"photochemistry"

"photocompose"

"photocomposed"

"photocomposes"

"photocomposing"

"photocomposition"

"photocopied"

"photocopier"

"photocopiers"

"photocopies"

"photocopy"

"photocopying"

"photoed"

"photoelectric"

"photoelectrically"

"photoelectricity"

"photoelectron"

"photoengrave"

"photoengraved"

"photoengraver"

"photoengravers"

"photoengraves"

"photoengraving"

"photoengravings"

"photoflash"

"photog"

"photogenic"

"photogenically"

"photograph"

"photographed"

"photographer"

"photographers"

"photographic"

"photographically"

"photographing"

"photographs"

"photography"

"photogs"

"photoinduced"

"photoing"

"photojournalism"

"photojournalist"

"photojournalists"

"photoluminescent"

"photoluminescently"

"photoluminescents"

"photomania"

"photomap"

"photomaps"

"photomechanical"

"photometer"

"photometers"

"photometric"

"photometry"

"photomicrogram"

"photomicrograph"

"photomicrographic"

"photomicrographs"

"photomicrography"

"photomural"

"photomurals"

"photon"

"photonegative"

"photonic"

"photons"

"photophilic"

"photophobia"

"photophobic"

"photoplay"

"photoplays"

"photopolarimeter"

"photoreception"

"photoreceptive"

"photoreceptor"

"photoreduction"

"photos"

"photosensitive"

"photosensitivity"

"photosensitization"

"photosensitize"

"photosensitized"

"photosensitizer"

"photosensitizes"

"photosensitizing"

"photosphere"

"photospheres"

"photospheric"

"photospherically"

"photostat"

"photostated"

"photostatic"

"photostating"

"photostats"

"photosyntheses"

"photosynthesis"

"photosynthesize"

"photosynthesized"

"photosynthesizes"

"photosynthesizing"

"photosynthetic"

"photosynthetically"

"phototachometer"

"phototelegraph"

"phototherapies"

"phototherapy"

"phototrophic"

"phototropic"

"phototropically"

"phototropism"

"photovoltaic"

"phr"

"phrasal"

"phrase"

"phrased"

"phraseology"

"phrases"

"phrasing"

"phrasings"

"phren"

"phrenetic"

"phrenic"

"phrenologic"

"phrenological"

"phrenologically"

"phrenologies"

"phrenologist"

"phrenologists"

"phrenology"

"phrensy"

"phthongometer"

"phycology"

"phycomycete"

"phycomycetes"

"phyla"

"phylacteries"

"phylactery"

"phylae"

"phyllomania"

"phylogeny"

"phylum"

"phys"

"physic"

"physical"

"physical alarm"

"physical capacity"

"physical health"

"physical information"

"physically"

"physicals"

"physician"

"physicianly"

"physicians"

"physicist"

"physicists"

"physicked"

"physicks"

"physicochemical"

"physics"

"physiognomic"

"physiognomical"

"physiognomically"

"physiognomies"

"physiognomist"

"physiognomy"

"physiographic"

"physiography"

"physiologic"

"physiological"

"physiologically"

"physiologies"

"physiologist"

"physiologists"

"physiology"

"physiopathologic"

"physiopathological"

"physiopathologically"

"physiotherapies"

"physiotherapist"

"physiotherapists"

"physiotherapy"

"physique"

"physiques"

"physis"

"phytology"

"phytomania"

"pi"

"pianic"

"pianissimo"

"pianist"

"pianists"

"piano"

"pianoforte"

"pianofortes"

"pianos"

"piaster"

"piasters"

"piastre"

"piastres"

"piazadora"

"piazza"

"piazzas"

"piazze"

"pibroch"

"pibrochs"

"pica"

"picador"

"picadores"

"picadors"

"picaresque"

"picaro"

"picaroon"

"picarooned"

"picaroons"

"picaros"

"picas"

"picasso"

"picayune"

"picayunes"

"piccalilli"

"piccalillis"

"piccolo"

"piccolos"

"pick"

"pickaback"

"pickaninnies"

"pickaninny"

"pickax"

"pickaxe"

"pickaxed"

"pickaxes"

"pickaxing"

"picked"

"picker"

"pickerel"

"pickerels"

"pickers"

"picket"

"picketed"

"picketer"

"picketers"

"picketing"

"pickets"

"pickier"

"pickiest"

"picking"

"pickings"

"pickle"

"pickled"

"pickles"

"pickling"

"picklock"

"picklocks"

"pickpocket"

"pickpockets"

"picks"

"pickup"

"pickups"

"pickwickian"

"pickwicks"

"picky"

"picnic"

"picnicked"

"picnicker"

"picnickers"

"picnicking"

"picnicky"

"picnics"

"picograms"

"picosecond"

"picoseconds"

"picot"

"picots"

"picquet"

"picquets"

"pics"

"pictograph"

"pictographic"

"pictographs"

"pictorial"

"pictorially"

"pictorials"

"picture"

"pictured"

"picturephone"

"picturephones"

"picturer"

"picturers"

"pictures"

"picturesque"

"picturesquely"

"picturesqueness"

"picturing"

"piddle"

"piddled"

"piddler"

"piddlers"

"piddles"

"piddling"

"pidgin"

"pidgins"

"pie"

"piebald"

"piebalds"

"piece"

"pieced"

"piecemeal"

"piecer"

"piecers"

"pieces"

"piecework"

"pieceworker"

"pieceworkers"

"piecing"

"piecings"

"piecrust"

"piecrusts"

"pied"

"piedmont"

"piedmonts"

"pieing"

"pieplant"

"pieplants"

"pier"

"pierce"

"pierced"

"piercer"

"piercers"

"pierces"

"piercing"

"piercingly"

"pierre"

"pierrot"

"pierrots"

"piers"

"pies"

"pieta"

"pietas"

"pieties"

"pietism"

"pietisms"

"pietist"

"pietists"

"piety"

"piezochemistries"

"piezochemistry"

"piezoelectric"

"piezoelectricity"

"piezometer"

"piezometric"

"piffle"

"piffled"

"piffles"

"piffling"

"pig"

"pigeon"

"pigeonhole"

"pigeonholed"

"pigeonholes"

"pigeonholing"

"pigeons"

"pigged"

"piggeries"

"piggery"

"piggie"

"piggier"

"piggies"

"piggiest"

"piggin"

"pigging"

"piggins"

"piggish"

"piggishness"

"piggy"

"piggyback"

"piggybacks"

"pigheaded"

"pigheadedness"

"piglet"

"piglets"

"pigment"

"pigmentation"

"pigmentations"

"pigmented"

"pigmenting"

"pigments"

"pigmies"

"pigmy"

"pignet"

"pignorated"

"pignut"

"pignuts"

"pigpen"

"pigpens"

"pigs"

"pigskin"

"pigskins"

"pigsticked"

"pigsties"

"pigsty"

"pigtail"

"pigtails"

"pigweed"

"PII"

"pike"

"piked"

"pikeman"

"pikemen"

"piker"

"pikers"

"pikes"

"pikestaff"

"pikestaves"

"piking"

"pilaf"

"pilaff"

"pilaffs"

"pilafs"

"pilar"

"pilaster"

"pilastered"

"pilasters"

"pilate"

"pilchard"

"pilchards"

"pile"

"pileate"

"piled"

"piles"

"pileup"

"pileups"

"pilfer"

"pilferage"

"pilfered"

"pilferer"

"pilferers"

"pilfering"

"pilfers"

"pilgrim"

"pilgrimage"

"pilgrimages"

"pilgrims"

"piling"

"pilings"

"pill"

"Pill Reminder"

"pillage"

"pillaged"

"pillager"

"pillagers"

"pillages"

"pillaging"

"pillar"

"pillared"

"pillaring"

"pillars"

"pillbox"

"pillboxes"

"pilled"

"pilling"

"pillion"

"pillions"

"pilloried"

"pillories"

"pillory"

"pillorying"

"pillow"

"pillowcase"

"pillowcases"

"pillowed"

"pillowing"

"pillows"

"pillowslip"

"pillowslips"

"pillowy"

"pills"

"pilose"

"pilot"

"pilotage"

"pilotages"

"piloted"

"pilothouse"

"pilothouses"

"piloting"

"pilotings"

"pilotless"

"pilots"

"pilous"

"pilsener"

"pilseners"

"pilsner"

"pilsners"

"pima"

"pimas"

"pimento"

"pimentos"

"pimiento"

"pimientos"

"pimp"

"pimped"

"pimpernel"

"pimpernels"

"pimping"

"pimple"

"pimpled"

"pimples"

"pimplier"

"pimpliest"

"pimpling"

"pimply"

"pimps"

"pin"

"pinafore"

"pinafores"

"pinata"

"pinatas"

"pinball"

"pinballs"

"pincer"

"pincers"

"pinch"

"pinchbugs"

"pinched"

"pincher"

"pinchers"

"pinches"

"pinching"

"pinchpenny"

"pincushion"

"pincushions"

"pine"

"pineal"

"pineapple"

"pineapples"

"pinecone"

"pinecones"

"pined"

"pineries"

"pines"

"pinesap"

"pinesaps"

"pinewood"

"pinewoods"

"piney"

"pinfeather"

"pinfeathers"

"pinfold"

"pinfolded"

"pinfolding"

"ping"

"pinged"

"pinger"

"pingers"

"pinging"

"pingrasses"

"pings"

"pinhead"

"pinheaded"

"pinheadedness"

"pinheads"

"pinhole"

"pinholes"

"pinier"

"piniest"

"pining"

"pinion"

"pinioned"

"pinioning"

"pinions"

"pink"

"pinked"

"pinker"

"pinkest"

"pinkeye"

"pinkie"

"pinkies"

"pinking"

"pinkings"

"pinkish"

"pinkishness"

"pinkly"

"pinkness"

"pinko"

"pinkoes"

"pinkos"

"pinks"

"pinky"

"pinna"

"pinnace"

"pinnaces"

"pinnacle"

"pinnacled"

"pinnacles"

"pinnacling"

"pinnae"

"pinnal"

"pinnas"

"pinnate"

"pinnated"

"pinnately"

"pinned"

"pinner"

"pinners"

"pinning"

"pinnings"

"pinocchio"

"pinochle"

"pinochles"

"pinocle"

"pinole"

"pinoles"

"pinon"

"pinones"

"pinons"

"pinpoint"

"pinpointed"

"pinpointing"

"pinpoints"

"pinprick"

"pinpricked"

"pinpricks"

"pins"

"pinscher"

"pinschers"

"pinsetter"

"pinsetters"

"pinspotter"

"pinspotters"

"pinstripe"

"pinstriped"

"pinstripes"

"pint"

"pinta"

"pintail"

"pintas"

"pinto"

"pintoes"

"pintos"

"pints"

"pintsize"

"pinup"

"pinups"

"pinwheel"

"pinwheels"

"pinworm"

"pinworms"

"piny"

"pinyon"

"pinyons"

"pion"

"pioneer"

"pioneered"

"pioneering"

"pioneers"

"pionic"

"pions"

"piosity"

"pious"

"piously"

"piousness"

"pip"

"pipage"

"pipe"

"pipeages"

"piped"

"pipedream"

"pipefish"

"pipeful"

"pipefuls"

"pipeline"

"pipelined"

"pipelines"

"pipelining"

"piper"

"pipers"

"pipes"

"pipestem"

"pipestems"

"pipet"

"pipets"

"pipette"

"pipetted"

"pipettes"

"pipetting"

"pipier"

"piping"

"pipingly"

"pipings"

"pipit"

"pipits"

"pipkin"

"pipkins"

"pipped"

"pippin"

"pippins"

"pips"

"pipsqueak"

"pipsqueaks"

"pipy"

"piquancies"

"piquancy"

"piquant"

"piquantly"

"pique"

"piqued"

"piques"

"piquet"

"piquing"

"piracies"

"piracy"

"pirana"

"piranas"

"piranha"

"piranhas"

"pirate"

"pirated"

"pirates"

"piratic"

"piratical"

"pirating"

"pirog"

"piroghi"

"pirogi"

"pirogue"

"pirogues"

"pirojki"

"piroshki"

"pirouette"

"pirouetted"

"pirouettes"

"pirouetting"

"pirozhki"

"pisa"

"piscatology"

"piscatorial"

"piscators"

"pisces"

"piscicide"

"piscine"

"pish"

"pished"

"pishes"

"pishing"

"pismire"

"pismires"

"piss"

"pissant"

"pissants"

"pissed"

"pisses"

"pissing"

"pissoir"

"pissoirs"

"pistache"

"pistachio"

"pistachios"

"pisteology"

"pistil"

"pistillate"

"pistils"

"pistol"

"pistole"

"pistoling"

"pistolled"

"pistolling"

"pistols"

"piston"

"pistons"

"pit"

"pita"

"pitapat"

"pitapats"

"pitas"

"pitch"

"pitchblende"

"pitched"

"pitcher"

"pitchers"

"pitches"

"pitchfork"

"pitchforks"

"pitchier"

"pitchiest"

"pitchily"

"pitching"

"pitchman"

"pitchmen"

"pitchometer"

"pitchouts"

"pitchy"

"piteous"

"piteously"

"piteousness"

"pitfall"

"pitfalls"

"pith"

"pitheads"

"pithecanthropus"

"pithed"

"pithier"

"pithiest"

"pithily"

"pithiness"

"pithing"

"piths"

"pithy"

"pitiable"

"pitiableness"

"pitiably"

"pitied"

"pitier"

"pitiers"

"pities"

"pitiful"

"pitifuller"

"pitifully"

"pitifulness"

"pitiless"

"pitilessly"

"pitilessness"

"pitman"

"pitmen"

"piton"

"pitons"

"pits"

"pitsaw"

"pittance"

"pittances"

"pitted"

"pitter"

"pitting"

"pittings"

"pituitaries"

"pituitary"

"pity"

"pitying"

"pityingly"

"pius"

"pivot"

"pivotal"

"pivotally"

"pivoted"

"pivoting"

"pivots"

"pix"

"pixel"

"pixels"

"pixes"

"pixie"

"pixieish"

"pixies"

"pixy"

"pixyish"

"pizazz"

"pizazzes"

"pizza"

"pizzas"

"pizzazz"

"pizzeria"

"pizzerias"

"pizzicato"

"pizzle"

"pkg"

"pkwy"

"pl"

"placability"

"placable"

"placably"

"placard"

"placarded"

"placarder"

"placarders"

"placarding"

"placards"

"placate"

"placated"

"placater"

"placaters"

"placates"

"placating"

"placation"

"place"

"placeable"

"placebo"

"placeboes"

"placebos"

"placed"

"placeholder"

"placement"

"placements"

"placenta"

"placentae"

"placental"

"placentas"

"placentation"

"placentography"

"placentomata"

"placer"

"placers"

"places"

"placid"

"placidity"

"placidly"

"placidness"

"placing"

"plack"

"placket"

"plackets"

"placks"

"placoid"

"placque"

"plagal"

"plages"

"plagiaries"

"plagiarism"

"plagiarisms"

"plagiarist"

"plagiaristic"

"plagiarists"

"plagiarize"

"plagiarized"

"plagiarizer"

"plagiarizers"

"plagiarizes"

"plagiarizing"

"plagiary"

"plague"

"plagued"

"plaguer"

"plaguers"

"plagues"

"plaguey"

"plaguily"

"plaguing"

"plaguy"

"plaice"

"plaid"

"plaids"

"plain"

"plainclothes"

"plainclothesman"

"plainclothesmen"

"plainer"

"plainest"

"plaining"

"plainly"

"plainness"

"plains"

"plainsman"

"plainsmen"

"plainsong"

"plainspoken"

"plainspokenness"

"plaint"

"plaintiff"

"plaintiffs"

"plaintive"

"plaintively"

"plaints"

"plait"

"plaited"

"plaiter"

"plaiters"

"plaiting"

"plaitings"

"plaits"

"plan"

"planar"

"planaria"

"planarian"

"planarias"

"planarity"

"plane"

"planed"

"planeload"

"planer"

"planers"

"planes"

"planet"

"planetaria"

"planetarium"

"planetariums"

"planetary"

"planetesimal"

"planetesimals"

"planetoid"

"planetoids"

"planetologist"

"planetologists"

"planetology"

"planets"

"plangency"

"plangent"

"planigraph"

"planigraphy"

"planimeter"

"planing"

"planish"

"planishing"

"plank"

"planked"

"planking"

"plankings"

"planks"

"plankton"

"planktonic"

"planktons"

"planless"

"planned"

"planner"

"planners"

"planning"

"plannings"

"planomania"

"plans"

"plant"

"plantain"

"plantains"

"plantar"

"plantation"

"plantations"

"planted"

"planter"

"planters"

"planting"

"plantings"

"plants"

"plaque"

"plaques"

"plash"

"plashed"

"plasher"

"plashes"

"plashiest"

"plashy"

"plasm"

"plasma"

"plasmaphereses"

"plasmapheresis"

"plasmas"

"plasmatic"

"plasmic"

"plasmoids"

"plasms"

"plaster"

"plasterboard"

"plastered"

"plasterer"

"plasterers"

"plastering"

"plasters"

"plasterwork"

"plastery"

"plastic"

"plastic wheels"

"plasticity"

"plasticize"

"plasticized"

"plasticizer"

"plasticizes"

"plasticizing"

"plastics"

"plastron"

"plastrons"

"plat"

"plate"

"plateau"

"plateaued"

"plateauing"

"plateaus"

"plateaux"

"plated"

"plateful"

"platefuls"

"platelet"

"platelets"

"platen"

"platens"

"plater"

"platers"

"plates"

"platesful"

"platform"

"platforms"

"platier"

"platies"

"plating"

"platings"

"platinic"

"platinum"

"platinums"

"platitude"

"platitudes"

"platitudinous"

"platitudinously"

"plato"

"platometer"

"platonic"

"platonically"

"platoon"

"platooned"

"platooning"

"platoons"

"plats"

"platted"

"platter"

"platters"

"platting"

"platy"

"platypi"

"platypus"

"platypuses"

"platys"

"plaudit"

"plaudits"

"plausibility"

"plausible"

"plausibleness"

"plausibly"

"plausive"

"play"

"playa"

"playable"

"playact"

"playacted"

"playacting"

"playacts"

"playas"

"playback"

"playbacks"

"playbill"

"playbills"

"playbook"

"playbooks"

"playboy"

"playboys"

"played"

"player"

"players"

"playfellow"

"playfellows"

"playful"

"playfully"

"playfulness"

"playgirl"

"playgirls"

"playgoer"

"playgoers"

"playground"

"playgrounds"

"playhouse"

"playhouses"

"playing"

"playland"

"playlands"

"playlet"

"playlets"

"playlist"

"playmate"

"playmates"

"playoff"

"playoffs"

"playpen"

"playpens"

"playroom"

"playrooms"

"plays"

"playsuit"

"playsuits"

"plaything"

"playthings"

"playtime"

"playtimes"

"playwear"

"playwears"

"playwright"

"playwrights"

"plaza"

"plazas"

"plea"

"plead"

"pleadable"

"pleaded"

"pleader"

"pleaders"

"pleading"

"pleadings"

"pleads"

"pleas"

"pleasant"

"pleasanter"

"pleasantly"

"pleasantness"

"pleasantries"

"pleasantry"

"please"

"pleased"

"pleaser"

"pleasers"

"pleases"

"pleasing"

"pleasingly"

"pleasingness"

"pleasurable"

"pleasurably"

"pleasure"

"pleasured"

"pleasureful"

"pleasures"

"pleasuring"

"pleat"

"pleated"

"pleater"

"pleaters"

"pleating"

"pleats"

"plebe"

"plebeian"

"plebeians"

"plebes"

"plebescite"

"plebian"

"plebiscite"

"plebiscites"

"plebs"

"plectra"

"plectrum"

"plectrums"

"pled"

"pledge"

"pledged"

"pledgee"

"pledgees"

"pledgeholder"

"pledger"

"pledgers"

"pledges"

"pledging"

"plegometer"

"pleiades"

"pleistocene"

"plemyrameter"

"plena"

"plenarily"

"plenary"

"plenipotentiaries"

"plenipotentiary"

"plenished"

"plenishes"

"plenitude"

"plenteous"

"plenteousness"

"plenties"

"plentiful"

"plentifully"

"plentifulness"

"plentitude"

"plenty"

"plenum"

"plenums"

"plethora"

"plethoras"

"plethoric"

"plethysmograph"

"pleura"

"pleural"

"pleuras"

"pleurisies"

"pleurisy"

"pleuritis"

"plexiglas"

"plexiglass"

"plexus"

"plexuses"

"pliability"

"pliable"

"pliably"

"pliancies"

"pliancy"

"pliant"

"pliantly"

"plied"

"plier"

"pliers"

"plies"

"plight"

"plighted"

"plighter"

"plighters"

"plighting"

"plights"

"plink"

"plinked"

"plinker"

"plinks"

"plinth"

"plinths"

"pliocene"

"plisse"

"plod"

"plodded"

"plodder"

"plodders"

"plodding"

"ploddingly"

"plods"

"plonk"

"plonked"

"plonking"

"plonks"

"plop"

"plopped"

"plopping"

"plops"

"plosions"

"plosive"

"plosives"

"plot"

"plotless"

"plots"

"plottage"

"plottages"

"plotted"

"plotter"

"plotters"

"plottier"

"plotties"

"plottiest"

"plotting"

"plough"

"ploughed"

"plougher"

"ploughers"

"ploughing"

"ploughman"

"ploughs"

"plover"

"plovers"

"plow"

"plowable"

"plowboy"

"plowboys"

"plowed"

"plower"

"plowers"

"plowing"

"plowlands"

"plowman"

"plowmen"

"plows"

"plowshare"

"plowshares"

"ploy"

"ployed"

"ploying"

"ploys"

"pluck"

"plucked"

"plucker"

"pluckers"

"pluckier"

"pluckiest"

"pluckily"

"pluckiness"

"plucking"

"plucks"

"plucky"

"plug"

"plugged"

"plugger"

"pluggers"

"plugging"

"plugin"

"plugs"

"pluguglies"

"plugugly"

"plum"

"plumage"

"plumaged"

"plumages"

"plumb"

"plumbable"

"plumbed"

"plumber"

"plumberies"

"plumbers"

"plumbery"

"plumbing"

"plumbings"

"plumbism"

"plumbs"

"plume"

"plumed"

"plumelet"

"plumelets"

"plumes"

"plumier"

"plumiest"

"pluming"

"plummet"

"plummeted"

"plummeting"

"plummets"

"plummier"

"plummiest"

"plummy"

"plump"

"plumped"

"plumpened"

"plumpening"

"plumpens"

"plumper"

"plumpers"

"plumpest"

"plumping"

"plumpish"

"plumply"

"plumpness"

"plumps"

"plums"

"plumy"

"plunder"

"plunderable"

"plunderage"

"plundered"

"plunderer"

"plunderers"

"plundering"

"plunders"

"plunge"

"plunged"

"plunger"

"plungers"

"plunges"

"plunging"

"plunk"

"plunked"

"plunker"

"plunkers"

"plunking"

"plunks"

"pluperfect"

"pluperfects"

"plural"

"pluralism"

"pluralities"

"plurality"

"pluralization"

"pluralize"

"pluralized"

"pluralizes"

"pluralizing"

"plurally"

"plurals"

"pluribus"

"plus"

"pluses"

"plush"

"plusher"

"plushes"

"plushest"

"plushier"

"plushiest"

"plushily"

"plushly"

"plushy"

"plussages"

"plusses"

"plutarch"

"pluto"

"plutocracies"

"plutocracy"

"plutocrat"

"plutocratic"

"plutocrats"

"plutology"

"plutomania"

"pluton"

"plutonic"

"plutonism"

"plutonium"

"plutons"

"pluvial"

"pluvially"

"pluviograph"

"pluviometer"

"ply"

"plyer"

"plyers"

"plying"

"plymouth"

"plymouths"

"plywood"

"plywoods"

"pm"

"PM&R"

"pneometer"

"pneuma"

"pneumas"

"pneumatic"

"pneumatically"

"pneumaticity"

"pneumatics"

"pneumatometer"

"pneumococcal"

"pneumococci"

"pneumococcic"

"pneumococcus"

"pneumoconiosis"

"pneumograph"

"pneumonia"

"pneumonic"

"pneumonitis"

"po"

"poach"

"poached"

"poacher"

"poachers"

"poaches"

"poachier"

"poachiest"

"poaching"

"poachy"

"pock"

"pocked"

"pocket"

"pocketbook"

"pocketbooks"

"pocketed"

"pocketer"

"pocketers"

"pocketful"

"pocketfuls"

"pocketing"

"pocketknife"

"pocketknives"

"pockets"

"pockier"

"pockily"

"pocking"

"pockmark"

"pockmarked"

"pockmarks"

"pocks"

"pocky"

"poco"

"pod"

"podded"

"podgier"

"podgily"

"podgy"

"podia"

"podiatric"

"podiatries"

"podiatrist"

"podiatrists"

"podiatry"

"podium"

"podiums"

"podology"

"pods"

"poem"

"poems"

"poesies"

"poesy"

"poet"

"poetaster"

"poetasters"

"poetess"

"poetesses"

"poetic"

"poetical"

"poetically"

"poetics"

"poetise"

"poetize"

"poetized"

"poetizer"

"poetizers"

"poetizes"

"poetizing"

"poetries"

"poetry"

"poets"

"pogonophobia"

"pogrom"

"pogromed"

"pogroming"

"pogroms"

"poi"

"poignancy"

"poignant"

"poignantly"

"poilu"

"poilus"

"poinciana"

"poincianas"

"poinsettia"

"poinsettias"

"point"

"pointblank"

"pointe"

"pointed"

"pointedly"

"pointedness"

"pointer"

"pointers"

"pointes"

"pointier"

"pointiest"

"pointillism"

"pointillist"

"pointillists"

"pointing"

"pointless"

"pointlessly"

"pointlessness"

"pointman"

"pointmen"

"points"

"pointy"

"poise"

"poised"

"poiser"

"poisers"

"poises"

"poising"

"poison"

"poisoned"

"poisoner"

"poisoners"

"poisoning"

"poisonings"

"poisonous"

"poisonously"

"poisonousness"

"poisons"

"poke"

"poked"

"poker"

"pokers"

"pokes"

"pokeweed"

"pokeweeds"

"pokey"

"pokeys"

"pokier"

"pokies"

"pokiest"

"pokily"

"pokiness"

"poking"

"poky"

"pol"

"poland"

"polar"

"polarimeter"

"polarimetric"

"polarimetries"

"polarimetry"

"polaris"

"polariscope"

"polariscopic"

"polarities"

"polarity"

"polarization"

"polarizations"

"polarize"

"polarized"

"polarizer"

"polarizes"

"polarizing"

"polarographic"

"polarography"

"polaroid"

"polaroids"

"polars"

"polder"

"polders"

"pole"

"poleax"

"poleaxe"

"poleaxed"

"poleaxes"

"poleaxing"

"polecat"

"polecats"

"poled"

"polemic"

"polemical"

"polemically"

"polemicist"

"polemicists"

"polemics"

"polemist"

"polemists"

"polemize"

"polemized"

"polemizes"

"polemizing"

"polemology"

"polemomania"

"polenta"

"polentas"

"poler"

"polers"

"poles"

"polestar"

"polestars"

"poleward"

"police"

"policed"

"policeman"

"policemen"

"polices"

"policewoman"

"policewomen"

"policies"

"policing"

"policy"

"policyholder"

"policyholders"

"poling"

"polio"

"poliomyelitic"

"poliomyelitis"

"polios"

"polis"

"polish"

"polished"

"polisher"

"polishers"

"polishes"

"polishing"

"polit"

"politburo"

"polite"

"politely"

"politeness"

"politer"

"politesse"

"politest"

"politic"

"political"

"politically"

"politician"

"politicians"

"politicize"

"politicized"

"politicizes"

"politicizing"

"politick"

"politicked"

"politicking"

"politicks"

"politico"

"politicoes"

"politicomania"

"politicos"

"politics"

"polities"

"polity"

"polk"

"polka"

"polkaed"

"polkaing"

"polkamania"

"polkas"

"poll"

"pollack"

"pollacks"

"pollard"

"pollarding"

"pollards"

"pollbook"

"polled"

"pollee"

"pollees"

"pollen"

"pollened"

"pollens"

"poller"

"pollers"

"pollinate"

"pollinated"

"pollinates"

"pollinating"

"pollination"

"pollinator"

"pollinators"

"polling"

"pollist"

"polliwog"

"polliwogs"

"polloi"

"polls"

"pollster"

"pollsters"

"pollutant"

"pollutants"

"pollute"

"polluted"

"polluter"

"polluters"

"pollutes"

"polluting"

"pollution"

"pollywog"

"pollywogs"

"polo"

"poloist"

"poloists"

"polonaise"

"polonaises"

"polonium"

"poloniums"

"pols"

"poltergeist"

"poltergeists"

"poltroon"

"poltroonery"

"poltroons"

"poly"

"polyandric"

"polyandries"

"polyandrist"

"polyandrous"

"polyandry"

"polychromatic"

"polychromia"

"polyclinic"

"polyclinics"

"polydactylies"

"polydactylism"

"polydactylous"

"polydactyly"

"polyester"

"polyesters"

"polyethylene"

"polygamic"

"polygamies"

"polygamist"

"polygamists"

"polygamous"

"polygamy"

"polyglot"

"polyglots"

"polygon"

"polygonal"

"polygonally"

"polygons"

"polygony"

"polygram"

"polygraph"

"polygraphic"

"polygraphically"

"polygraphs"

"polyhedra"

"polyhedral"

"polyhedron"

"polyhedrons"

"polymania"

"polymath"

"polymaths"

"polymer"

"polymeric"

"polymerically"

"polymerization"

"polymerize"

"polymerized"

"polymerizes"

"polymerizing"

"polymers"

"polymorph"

"polymorphic"

"polymorphically"

"polymorphism"

"polymorphous"

"polymorphously"

"polynesia"

"polynesian"

"polynesians"

"polynomial"

"polynomials"

"polyp"

"polyphonic"

"polyphonically"

"polyphony"

"polyploid"

"polypod"

"polypoid"

"polypous"

"polyps"

"polypus"

"polys"

"polysaccharide"

"polysorbate"

"polystyrene"

"polysyllabic"

"polysyllable"

"polysyllables"

"polytechnic"

"polytheism"

"polytheist"

"polytheistic"

"polytheists"

"polyunsaturated"

"polyvinyl"

"pomade"

"pomaded"

"pomades"

"pomading"

"pomander"

"pomanders"

"pomatums"

"pome"

"pomegranate"

"pomegranates"

"pomeranian"

"pomeranians"

"pomes"

"pommel"

"pommeled"

"pommeling"

"pommelled"

"pommelling"

"pommels"

"pomology"

"pomp"

"pompadour"

"pompadours"

"pompano"

"pompanos"

"pompeii"

"pompom"

"pompoms"

"pompon"

"pompons"

"pomposity"

"pompous"

"pompously"

"pompousness"

"pomps"

"ponce"

"ponces"

"poncho"

"ponchos"

"pond"

"ponder"

"ponderable"

"pondered"

"ponderer"

"ponderers"

"pondering"

"ponderosa"

"ponderous"

"ponderously"

"ponderousness"

"ponders"

"ponds"

"pondweed"

"pondweeds"

"pone"

"pones"

"pong"

"pongee"

"pongees"

"pongid"

"poniard"

"poniarded"

"poniards"

"ponied"

"ponies"

"pons"

"pontes"

"pontiac"

"pontiacs"

"pontiff"

"pontiffs"

"pontifical"

"pontifically"

"pontificate"

"pontificated"

"pontificates"

"pontificating"

"pontificator"

"pontius"

"ponton"

"pontons"

"pontoon"

"pontoons"

"pony"

"ponying"

"ponytail"

"ponytails"

"pooch"

"pooches"

"poodle"

"poodles"

"pooh"

"poohed"

"poohing"

"poohs"

"pool"

"pooled"

"poolhall"

"poolhalls"

"pooling"

"poolroom"

"poolrooms"

"pools"

"poop"

"pooped"

"pooping"

"poops"

"poopsie"

"poor"

"poorer"

"poorest"

"poorhouse"

"poorhouses"

"poorish"

"poorly"

"poorness"

"pop"

"pop-up"

"popcorn"

"popcorns"

"pope"

"popedom"

"popedoms"

"poperies"

"popery"

"popes"

"popeye"

"popeyed"

"popgun"

"popguns"

"popinjay"

"popinjays"

"popish"

"popishly"

"poplar"

"poplars"

"poplin"

"poplins"

"popover"

"popovers"

"poppa"

"poppas"

"popped"

"popper"

"poppers"

"poppet"

"poppets"

"poppied"

"poppies"

"popping"

"poppy"

"poppycock"

"pops"

"populace"

"populaces"

"popular"

"popular recipes"

"popularity"

"popularization"

"popularizations"

"popularize"

"popularized"

"popularizes"

"popularizing"

"popularly"

"populate"

"populated"

"populates"

"populating"

"population"

"populations"

"populi"

"populism"

"populisms"

"populist"

"populists"

"populous"

"populousness"

"porcelain"

"porcelains"

"porch"

"porches"

"porcine"

"porcupine"

"porcupines"

"pore"

"pored"

"pores"

"porgies"

"porgy"

"poring"

"poriomania"

"pork"

"porker"

"porkers"

"porkier"

"porkies"

"porkiest"

"porkpie"

"porkpies"

"porks"

"porky"

"porn"

"porno"

"pornographer"

"pornographic"

"pornographically"

"pornographies"

"pornography"

"pornomania"

"pornos"

"porns"

"porometer"

"poroscope"

"porose"

"porosities"

"porosity"

"porous"

"porously"

"porousness"

"porphyries"

"porphyritic"

"porphyry"

"porpoise"

"porpoises"

"porridge"

"porridges"

"porringer"

"porringers"

"port"

"portability"

"portable"

"portables"

"portably"

"portage"

"portaged"

"portages"

"portaging"

"portal"

"portaled"

"portalled"

"portals"

"portcullis"

"portcullises"

"ported"

"portend"

"portended"

"portending"

"portends"

"portent"

"portentous"

"portentously"

"portentousness"

"portents"

"porter"

"porterhouse"

"porters"

"portfolio"

"portfolios"

"porthole"

"portholes"

"portico"

"porticoed"

"porticoes"

"porticos"

"portiere"

"portiered"

"portieres"

"porting"

"portion"

"portioned"

"portioner"

"portioners"

"portiones"

"portioning"

"portionless"

"portions"

"portland"

"portless"

"portlier"

"portliest"

"portliness"

"portly"

"portmanteau"

"portmanteaus"

"portmanteaux"

"portrait"

"portraitist"

"portraitists"

"portraits"

"portraiture"

"portray"

"portrayal"

"portrayals"

"portrayed"

"portraying"

"portrays"

"portress"

"portresses"

"ports"

"portugal"

"portuguese"

"portulaca"

"portulacas"

"pose"

"posed"

"poseidon"

"poser"

"posers"

"poses"

"poseur"

"poseurs"

"posh"

"posher"

"poshest"

"poshly"

"poshness"

"posies"

"posing"

"posingly"

"posit"

"posited"

"positing"

"position"

"positional"

"positioned"

"positioning"

"positions"

"positive"

"positive feelings"

"positive manner"

"positively"

"positiveness"

"positiver"

"positives"

"positivest"

"positron"

"positrons"

"posits"

"posology"

"posse"

"posses"

"possess"

"possessable"

"possessed"

"possesses"

"possessible"

"possessing"

"possession"

"possessions"

"possessive"

"possessively"

"possessiveness"

"possessives"

"possessor"

"possessors"

"possessory"

"possets"

"possibilities"

"possibility"

"possible"

"possibler"

"possiblest"

"possibly"

"possum"

"possums"

"post"

"postage"

"postages"

"postal"

"postally"

"postals"

"postaxial"

"postbag"

"postbags"

"postbellum"

"postbox"

"postboxes"

"postboy"

"postboys"

"postcard"

"postcardinal"

"postcards"

"postclassical"

"postcoital"

"postconsonantal"

"postconvalescent"

"postconvalescents"

"postdate"

"postdated"

"postdates"

"postdating"

"postdigestive"

"postdoctoral"

"posted"

"postelection"

"poster"

"posterior"

"posteriority"

"posteriorly"

"posteriors"

"posterities"

"posterity"

"postern"

"posterns"

"posters"

"postfaces"

"postfix"

"postfixed"

"postfixes"

"postfixing"

"postformed"

"postforms"

"postglacial"

"postgraduate"

"postgraduates"

"posthaste"

"posthole"

"postholes"

"posthumous"

"posthumously"

"posthypnotic"

"posthypnotically"

"postilion"

"postilions"

"posting"

"postings"

"postlude"

"postludes"

"postman"

"postmark"

"postmarked"

"postmarking"

"postmarks"

"postmaster"

"postmasters"

"postmen"

"postmenopausal"

"postmenstrual"

"postmillennial"

"postmistress"

"postmistresses"

"postmortem"

"postmortems"

"postnasal"

"postnatal"

"postnatally"

"postnuptial"

"postoffice"

"postoperative"

"postoperatively"

"postorbital"

"postpaid"

"postpartum"

"postpone"

"postponed"

"postponement"

"postponements"

"postpones"

"postponing"

"postprandial"

"postprandially"

"postprocessing"

"posts"

"postscript"

"postscripts"

"postseason"

"postseasonal"

"posttraumatic"

"posttreatment"

"postulant"

"postulants"

"postulate"

"postulated"

"postulates"

"postulating"

"postulation"

"postulations"

"postulator"

"postural"

"posture"

"postured"

"posturer"

"posturers"

"postures"

"posturing"

"postwar"

"posy"

"pot"

"potability"

"potable"

"potables"

"potage"

"potages"

"potamology"

"potash"

"potashes"

"potassium"

"potation"

"potations"

"potato"

"potatoes"

"potbellied"

"potbellies"

"potbelly"

"potboiled"

"potboiler"

"potboilers"

"potboiling"

"potboy"

"potboys"

"poteen"

"poteens"

"potence"

"potences"

"potencies"

"potency"

"potent"

"potentate"

"potentates"

"potential"

"potentialities"

"potentiality"

"potentially"

"potentials"

"potentiate"

"potentiated"

"potentiates"

"potentiating"

"potentiation"

"potentiator"

"potentiometer"

"potentiometers"

"potentiometric"

"potently"

"potful"

"potfuls"

"pothead"

"potheads"

"pother"

"potherb"

"potherbs"

"potholder"

"potholders"

"pothole"

"potholed"

"potholes"

"pothook"

"pothooks"

"pothouse"

"pothouses"

"potichomania"

"potion"

"potions"

"potlach"

"potlatch"

"potluck"

"potlucks"

"potman"

"potmen"

"potomac"

"potomania"

"potometer"

"potpie"

"potpies"

"potpourri"

"potpourris"

"pots"

"potshard"

"potsherd"

"potsherds"

"potshot"

"potshots"

"potsie"

"potsies"

"potsy"

"pottage"

"pottages"

"potted"

"potteen"

"potter"

"pottered"

"potterer"

"potterers"

"potteries"

"pottering"

"potters"

"pottery"

"pottier"

"potties"

"potting"

"pottle"

"potty"

"pouch"

"pouched"

"pouches"

"pouchiest"

"pouching"

"pouchy"

"pouf"

"poufed"

"pouff"

"pouffe"

"pouffed"

"pouffes"

"pouffs"

"poufs"

"poult"

"poultice"

"poulticed"

"poultices"

"poulticing"

"poultries"

"poultry"

"poults"

"pounce"

"pounced"

"pouncer"

"pouncers"

"pounces"

"pouncing"

"pound"

"poundage"

"poundages"

"poundal"

"poundals"

"pounded"

"pounder"

"pounders"

"pounding"

"poundkeeper"

"pounds"

"pour"

"pourable"

"pourboire"

"pourboires"

"poured"

"pourer"

"pourers"

"pouring"

"pours"

"pout"

"pouted"

"pouter"

"pouters"

"poutier"

"poutiest"

"pouting"

"pouts"

"pouty"

"poverties"

"poverty"

"pow"

"powder"

"powdered"

"powderer"

"powderers"

"powdering"

"powders"

"powdery"

"power"

"powerboat"

"powerboats"

"powered"

"powerful"

"powerful pattern"

"powerfully"

"powerfulness"

"powerhouse"

"powerhouses"

"powering"

"powerless"

"powerlessly"

"powerplants"

"powers"

"pows"

"powwow"

"powwowed"

"powwowing"

"powwows"

"pox"

"poxed"

"poxes"

"poxing"

"pp"

"ppd"

"practicabilities"

"practicability"

"practicable"

"practicably"

"practical"

"practicality"

"practically"

"practice"

"practiced"

"practices"

"practicing"

"practising"

"practitioner"

"practitioners"

"praecoces"

"praecox"

"praesidia"

"praetor"

"praetorian"

"praetors"

"pragmatic"

"pragmatical"

"pragmatically"

"pragmatism"

"pragmatist"

"pragmatists"

"prague"

"prairie"

"prairies"

"praise"

"praised"

"praiser"

"praisers"

"praises"

"praiseworthily"

"praiseworthiness"

"praiseworthy"

"praising"

"praline"

"pralines"

"pram"

"prams"

"prana"

"prance"

"pranced"

"prancer"

"prancers"

"prances"

"prancing"

"prancingly"

"prandial"

"prank"

"pranked"

"prankish"

"pranks"

"prankster"

"pranksters"

"praos"

"praseodymium"

"prat"

"prate"

"prated"

"prater"

"praters"

"prates"

"pratfall"

"pratfalls"

"prating"

"pratique"

"pratiques"

"prats"

"prattle"

"prattled"

"prattler"

"prattlers"

"prattles"

"prattling"

"praus"

"prawn"

"prawned"

"prawner"

"prawners"

"prawning"

"prawns"

"praxeological"

"praxeology"

"praxes"

"praxis"

"praxises"

"pray"

"prayed"

"prayer"

"prayerful"

"prayerfully"

"prayerfulness"

"prayers"

"praying"

"prayingly"

"prays"

"pre"

"preaccept"

"preacceptance"

"preacceptances"

"preaccepted"

"preaccepting"

"preaccepts"

"preaccustom"

"preaccustomed"

"preaccustoming"

"preaccustoms"

"preach"

"preached"

"preacher"

"preachers"

"preaches"

"preachier"

"preachiest"

"preaching"

"preachings"

"preachment"

"preachments"

"preachy"

"preadapt"

"preadapted"

"preadapting"

"preadapts"

"preadjust"

"preadjustable"

"preadjusted"

"preadjusting"

"preadjustment"

"preadjustments"

"preadjusts"

"preadmit"

"preadolescence"

"preadolescent"

"preadolescents"

"preadult"

"preadults"

"preaffirm"

"preaffirmation"

"preaffirmed"

"preaffirming"

"preaffirms"

"preallot"

"preallots"

"preallotted"

"preallotting"

"preamble"

"preambles"

"preamp"

"preamplifier"

"preamplifiers"

"preamps"

"preanesthetic"

"preannounce"

"preannounced"

"preannouncement"

"preannouncements"

"preannounces"

"preannouncing"

"preappearance"

"preappearances"

"preapplication"

"preapplications"

"preappoint"

"preappointed"

"preappointing"

"preappoints"

"prearm"

"prearmed"

"prearming"

"prearms"

"prearrange"

"prearranged"

"prearrangement"

"prearranges"

"prearranging"

"preascertain"

"preascertained"

"preascertaining"

"preascertainment"

"preascertains"

"preassemble"

"preassembled"

"preassembles"

"preassembling"

"preassembly"

"preassign"

"preassigned"

"preassigning"

"preassigns"

"preaxial"

"preaxially"

"prebend"

"prebendaries"

"prebendary"

"prebends"

"prebill"

"prebilled"

"prebilling"

"prebills"

"prebless"

"preblessed"

"preblesses"

"preblessing"

"preboil"

"preboiled"

"preboiling"

"preboils"

"precalculate"

"precalculated"

"precalculates"

"precalculating"

"precalculation"

"precalculations"

"precambrian"

"precancel"

"precanceled"

"precanceling"

"precancelled"

"precancelling"

"precancels"

"precancerous"

"precapitalistic"

"precarious"

"precariously"

"precariousness"

"precast"

"precaution"

"precautionary"

"precautions"

"precedable"

"precede"

"preceded"

"precedence"

"precedent"

"precedentless"

"precedents"

"precedes"

"preceding"

"preceeding"

"precelebration"

"precelebrations"

"precented"

"precentor"

"precentors"

"precept"

"preceptor"

"preceptors"

"preceptress"

"preceptresses"

"precepts"

"precess"

"precessed"

"precesses"

"precessing"

"precession"

"precessional"

"precessions"

"prechill"

"prechilled"

"prechilling"

"prechills"

"precinct"

"precincts"

"preciosities"

"preciosity"

"precious"

"preciously"

"preciousness"

"precipice"

"precipiced"

"precipices"

"precipitability"

"precipitable"

"precipitancy"

"precipitant"

"precipitate"

"precipitated"

"precipitately"

"precipitateness"

"precipitates"

"precipitating"

"precipitation"

"precipitations"

"precipitous"

"precipitously"

"precipitousness"

"precis"

"precise"

"precised"

"precisely"

"preciseness"

"preciser"

"precises"

"precisest"

"precisian"

"precisians"

"precising"

"precision"

"precivilization"

"preclean"

"precleaned"

"precleaning"

"precleans"

"preclude"

"precluded"

"precludes"

"precluding"

"preclusion"

"preclusively"

"precocious"

"precociously"

"precociousness"

"precocity"

"precognition"

"precognitions"

"precognitive"

"precollege"

"precollegiate"

"preconceal"

"preconcealed"

"preconcealing"

"preconcealment"

"preconceals"

"preconceive"

"preconceived"

"preconceives"

"preconceiving"

"preconception"

"preconceptions"

"preconcession"

"preconcessions"

"precondemn"

"precondemnation"

"precondemned"

"precondemning"

"precondemns"

"precondition"

"preconditioned"

"preconditioning"

"preconditions"

"preconscious"

"preconsideration"

"preconsiderations"

"preconstruct"

"preconstructed"

"preconstructing"

"preconstruction"

"preconstructs"

"preconsultation"

"preconsultations"

"precontrive"

"precontrived"

"precontrives"

"precontriving"

"precook"

"precooked"

"precooking"

"precooks"

"precooled"

"precooling"

"precools"

"precox"

"precursor"

"precursors"

"precursory"

"precut"

"predacious"

"predaciousness"

"predacity"

"predate"

"predated"

"predates"

"predating"

"predation"

"predations"

"predator"

"predatorial"

"predatoriness"

"predators"

"predatory"

"predawn"

"predawns"

"predecease"

"predeceased"

"predeceases"

"predeceasing"

"predecessor"

"predecessors"

"predefined"

"predefining"

"predepression"

"predesignate"

"predesignated"

"predesignates"

"predesignating"

"predesignation"

"predestinarian"

"predestinate"

"predestinated"

"predestinates"

"predestinating"

"predestination"

"predestine"

"predestined"

"predestines"

"predestining"

"predetermination"

"predeterminations"

"predetermine"

"predetermined"

"predetermines"

"predetermining"

"prediagnostic"

"predicable"

"predicament"

"predicaments"

"predicate"

"predicated"

"predicates"

"predicating"

"predication"

"predications"

"predicative"

"predicator"

"predicatory"

"predict"

"predictability"

"predictable"

"predictably"

"predicted"

"predicting"

"prediction"

"predictions"

"predictive"

"predictively"

"predictiveness"

"predictor"

"predictors"

"predicts"

"predigest"

"predigested"

"predigesting"

"predigestion"

"predigests"

"predilection"

"predilections"

"predispose"

"predisposed"

"predisposes"

"predisposing"

"predisposition"

"predispositions"

"predominance"

"predominant"

"predominantly"

"predominate"

"predominated"

"predominately"

"predominates"

"predominating"

"predomination"

"predusks"

"preelection"

"preemie"

"preemies"

"preeminence"

"preeminent"

"preeminently"

"preempt"

"preempted"

"preempting"

"preemption"

"preemptions"

"preemptive"

"preemptively"

"preemptory"

"preempts"

"preen"

"preened"

"preener"

"preeners"

"preengage"

"preengaged"

"preengages"

"preengaging"

"preening"

"preenlistment"

"preenlistments"

"preens"

"preestablish"

"preestablished"

"preestablishes"

"preestablishing"

"preestimate"

"preestimated"

"preestimates"

"preestimating"

"preexamination"

"preexaminations"

"preexamine"

"preexamined"

"preexamines"

"preexamining"

"preexist"

"preexisted"

"preexisting"

"preexists"

"preexpose"

"preexposed"

"preexposes"

"preexposing"

"preexposure"

"preexposures"

"prefab"

"prefabbed"

"prefabbing"

"prefabricate"

"prefabricated"

"prefabricates"

"prefabricating"

"prefabrication"

"prefabs"

"preface"

"prefaced"

"prefacer"

"prefacers"

"prefaces"

"prefacing"

"prefatory"

"prefect"

"prefects"

"prefecture"

"prefectures"

"prefer"

"preferability"

"preferable"

"preferably"

"preference"

"preferences"

"preferential"

"preferentially"

"preferment"

"preferments"

"preferred"

"preferrer"

"preferrers"

"preferring"

"prefers"

"prefigure"

"prefigured"

"prefigures"

"prefiguring"

"prefix"

"prefixal"

"prefixally"

"prefixed"

"prefixes"

"prefixing"

"prefixion"

"prefixions"

"preform"

"preformed"

"preforming"

"preforms"

"pregame"

"preglacial"

"pregnancies"

"pregnancy"

"pregnancy facts"

"pregnancy test"

"pregnancy tips"

"pregnancy wheel"

"pregnant"

"pregnantly"

"preharden"

"prehardened"

"prehardening"

"prehardens"

"preheat"

"preheated"

"preheating"

"preheats"

"prehensile"

"prehensility"

"prehistoric"

"prehistorical"

"prehistorically"

"prehistory"

"prehuman"

"preinaugural"

"preindustrial"

"preinsert"

"preinserted"

"preinserting"

"preinserts"

"preinstruct"

"preinstructed"

"preinstructing"

"preinstruction"

"preinstructs"

"preintimation"

"prejudge"

"prejudged"

"prejudger"

"prejudges"

"prejudging"

"prejudgment"

"prejudgments"

"prejudice"

"prejudiced"

"prejudicedly"

"prejudices"

"prejudicial"

"prejudicially"

"prejudicing"

"prekindergarten"

"prekindergartens"

"prelacies"

"prelacy"

"prelate"

"prelates"

"prelatic"

"prelim"

"preliminaries"

"preliminarily"

"preliminary"

"prelimit"

"prelimited"

"prelimiting"

"prelimits"

"prelims"

"preliterate"

"prelude"

"preluded"

"preluder"

"preludes"

"premarital"

"premature"

"prematurely"

"prematureness"

"prematurities"

"premed"

"premedical"

"premedics"

"premeditate"

"premeditated"

"premeditatedly"

"premeditatedness"

"premeditates"

"premeditating"

"premeditation"

"premeditative"

"premeditator"

"premeditators"

"premeds"

"premenstrual"

"premenstrually"

"premie"

"premier"

"premiere"

"premiered"

"premieres"

"premiering"

"premiers"

"premiership"

"premierships"

"premies"

"premise"

"premised"

"premises"

"premising"

"premiss"

"premisses"

"premium"

"Premium version"

"premiums"

"premix"

"premixed"

"premixes"

"premixing"

"premolar"

"premolars"

"premonition"

"premonitions"

"premonitory"

"prename"

"prenames"

"prenatal"

"Prenatal Lullabies"

"Prenatal music"

"prenatally"

"prentice"

"prenticed"

"prentices"

"prenticing"

"prenuptial"

"preoccupation"

"preoccupations"

"preoccupied"

"preoccupies"

"preoccupy"

"preoccupying"

"preoperative"

"preordain"

"preordained"

"preordaining"

"preordains"

"preordination"

"preorganization"

"prep"

"prepack"

"prepackage"

"prepackaged"

"prepackages"

"prepackaging"

"prepacked"

"prepacking"

"prepacks"

"prepaid"

"preparation"

"Preparation study"

"preparations"

"preparatorily"

"preparatory"

"prepare"

"prepared"

"preparedness"

"preparer"

"preparers"

"prepares"

"preparing"

"prepay"

"prepaying"

"prepayment"

"prepayments"

"prepays"

"preplan"

"preplanned"

"preplanning"

"preplans"

"preponderance"

"preponderant"

"preponderantly"

"preponderate"

"preponderated"

"preponderates"

"preponderating"

"preposition"

"prepositional"

"prepositions"

"prepossess"

"prepossessed"

"prepossesses"

"prepossessing"

"prepossessingly"

"prepossessingness"

"prepossession"

"prepossessions"

"preposterous"

"preposterously"

"preposterousness"

"prepped"

"preppie"

"preppies"

"prepping"

"preprint"

"preprinted"

"preprints"

"preprocessing"

"preprocessor"

"preprogrammed"

"preps"

"prepsychotic"

"prepubescence"

"prepubescent"

"prepublication"

"prepuce"

"prepuces"

"prepunch"

"prerecord"

"prerecorded"

"prerecording"

"prerecords"

"preregister"

"preregistered"

"preregistering"

"preregisters"

"preregistration"

"prereproductive"

"prerequisite"

"prerequisites"

"prerogative"

"prerogatives"

"pres"

"presage"

"presaged"

"presager"

"presagers"

"presages"

"presaging"

"presanctified"

"presbyope"

"presbyopia"

"presbyopic"

"presbyter"

"presbyterian"

"presbyterianism"

"presbyterians"

"presbyters"

"preschool"

"preschooler"

"preschoolers"

"prescience"

"prescient"

"prescientific"

"prescore"

"prescored"

"prescores"

"prescoring"

"prescribable"

"prescribe"

"prescribed"

"prescriber"

"prescribes"

"prescribing"

"prescript"

"prescription"

"prescriptions"

"prescriptive"

"prescripts"

"preseason"

"preselect"

"preselected"

"preselecting"

"preselects"

"presell"

"presells"

"presence"

"presences"

"present"

"presentability"

"presentable"

"presentably"

"presentation"

"presentations"

"presented"

"presentence"

"presenter"

"presenters"

"presentiment"

"presentiments"

"presenting"

"presently"

"presentment"

"presents"

"preservable"

"preservation"

"preservations"

"preservative"

"preservatives"

"preserve"

"preserved"

"preserver"

"preservers"

"preserves"

"preserving"

"preset"

"presets"

"presetting"

"preshape"

"preshaped"

"preshapes"

"preshrunk"

"preside"

"presided"

"presidencies"

"presidency"

"president"

"presidential"

"presidents"

"presider"

"presiders"

"presides"

"presiding"

"presidio"

"presidios"

"presidium"

"presidiums"

"presift"

"presifted"

"presifting"

"presifts"

"preslavery"

"presley"

"presoak"

"presoaked"

"presoaking"

"presoaks"

"presold"

"press"

"pressed"

"presser"

"pressers"

"presses"

"pressing"

"pressingly"

"pressingness"

"pressings"

"pressman"

"pressmark"

"pressmen"

"pressor"

"pressoreceptor"

"pressosensitive"

"pressroom"

"pressrooms"

"pressrun"

"pressruns"

"pressure"

"pressured"

"pressures"

"pressuring"

"pressurization"

"pressurize"

"pressurized"

"pressurizer"

"pressurizers"

"pressurizes"

"pressurizing"

"presswork"

"prest"

"prestamp"

"prestidigitation"

"prestidigitator"

"prestidigitators"

"prestige"

"prestigeful"

"prestiges"

"prestigious"

"prestigiously"

"prestigiousness"

"presto"

"prestos"

"prestressed"

"presumable"

"presumably"

"presume"

"presumed"

"presumer"

"presumers"

"presumes"

"presuming"

"presumption"

"presumptions"

"presumptive"

"presumptively"

"presumptuous"

"presumptuously"

"presumptuousness"

"presuppose"

"presupposed"

"presupposes"

"presupposing"

"presupposition"

"presuppositions"

"presurgical"

"presynaptically"

"pretaste"

"preteen"

"preteens"

"pretence"

"pretences"

"pretend"

"pretended"

"pretendedly"

"pretender"

"pretenders"

"pretending"

"pretends"

"pretense"

"pretensed"

"pretenses"

"pretension"

"pretensions"

"pretention"

"pretentious"

"pretentiously"

"pretentiousness"

"preterit"

"preterits"

"preterminal"

"preternatural"

"preternaturally"

"pretest"

"pretested"

"pretesting"

"pretests"

"pretext"

"pretexts"

"pretor"

"pretoria"

"pretors"

"pretrial"

"prettied"

"prettier"

"pretties"

"prettiest"

"prettification"

"prettified"

"prettifier"

"prettifiers"

"prettifies"

"prettify"

"prettifying"

"prettily"

"prettiness"

"pretty"

"prettying"

"pretzel"

"pretzels"

"preunion"

"prevail"

"prevailed"

"prevailer"

"prevailers"

"prevailing"

"prevailingly"

"prevails"

"prevalence"

"prevalent"

"prevalently"

"prevaricate"

"prevaricated"

"prevaricates"

"prevaricating"

"prevarication"

"prevarications"

"prevaricator"

"prevaricators"

"prevent"

"preventability"

"preventable"

"preventative"

"preventatives"

"prevented"

"preventible"

"preventing"

"prevention"

"preventions"

"preventive"

"preventively"

"preventiveness"

"preventives"

"preventorium"

"prevents"

"preview"

"previewed"

"previewing"

"previews"

"previous"

"previously"

"prevocational"

"prevue"

"prevued"

"prevues"

"prevuing"

"prewar"

"prewarm"

"prewarmed"

"prewarming"

"prewarms"

"prewarned"

"prewash"

"prewashed"

"prewashes"

"prewashing"

"prexies"

"prexy"

"prey"

"preyed"

"preyer"

"preyers"

"preying"

"preys"

"priapic"

"priapism"

"priapisms"

"priapuses"

"price"

"priced"

"priceless"

"pricelessness"

"pricer"

"pricers"

"prices"

"pricey"

"pricier"

"priciest"

"pricing"

"prick"

"pricked"

"pricker"

"prickers"

"prickier"

"prickiest"

"pricking"

"prickle"

"prickled"

"prickles"

"pricklier"

"prickliest"

"prickliness"

"prickling"

"prickly"

"pricks"

"pricky"

"pricy"

"pride"

"prided"

"prideful"

"pridefully"

"prides"

"priding"

"pried"

"priedieus"

"priedieux"

"prier"

"priers"

"pries"

"priest"

"priested"

"priestess"

"priestesses"

"priesthood"

"priesting"

"priestlier"

"priestliness"

"priestly"

"priests"

"prig"

"priggery"

"priggish"

"priggishly"

"priggishness"

"prigs"

"prim"

"prima"

"primacies"

"primacy"

"primal"

"primaries"

"primarily"

"primariness"

"primary"

"primas"

"primate"

"primates"

"primatial"

"primatology"

"prime"

"primed"

"primely"

"primer"

"primero"

"primeros"

"primers"

"primes"

"primeval"

"primevally"

"primigenial"

"priming"

"primings"

"primitive"

"primitively"

"primitiveness"

"primitives"

"primitivism"

"primitivity"

"primly"

"primmed"

"primmer"

"primmest"

"primming"

"primness"

"primo"

"primogenitor"

"primogenitors"

"primogeniture"

"primordial"

"primordially"

"primos"

"primp"

"primped"

"primping"

"primps"

"primrose"

"primroses"

"prims"

"primulas"

"primus"

"primuses"

"prince"

"princedom"

"princedoms"

"princelier"

"princeliness"

"princeling"

"princelings"

"princely"

"princes"

"princess"

"princesses"

"princeton"

"principal"

"principalities"

"principality"

"principally"

"principals"

"principle"

"principled"

"principles"

"prink"

"prinked"

"prinking"

"prinks"

"print"

"printable"

"printed"

"printer"

"printers"

"printery"

"printing"

"printings"

"printout"

"printouts"

"prints"

"prior"

"priorate"

"priorates"

"prioress"

"prioresses"

"priori"

"priories"

"priorities"

"priority"

"priors"

"priory"

"prise"

"prised"

"prises"

"prism"

"prismatic"

"prismoid"

"prismoids"

"prisms"

"prison"

"prisoned"

"prisoner"

"prisoners"

"prisoning"

"prisons"

"prisoptometer"

"priss"

"prisses"

"prissier"

"prissies"

"prissiest"

"prissily"

"prissiness"

"prissy"

"pristine"

"prithee"

"privacies"

"privacy"

"private"

"private clinic"

"privateer"

"privateers"

"privately"

"privateness"

"privater"

"privates"

"privatest"

"privation"

"privations"

"privatized"

"privatizing"

"privet"

"privets"

"privier"

"privies"

"priviest"

"privilege"

"privileged"

"privileges"

"privileging"

"privily"

"privities"

"privity"

"privy"

"prix"

"prize"

"prized"

"prizefight"

"prizefighter"

"prizefighters"

"prizefighting"

"prizefights"

"prizer"

"prizers"

"prizes"

"prizewinner"

"prizewinners"

"prizewinning"

"prizing"

"pro"

"Pro version"

"proabortion"

"proadministration"

"proadoption"

"proalliance"

"proamendment"

"proapproval"

"proas"

"probabilities"

"probability"

"probable"

"probably"

"probate"

"probated"

"probates"

"probating"

"probation"

"probational"

"probationary"

"probationer"

"probationers"

"probations"

"probative"

"probatively"

"probe"

"probeable"

"probed"

"prober"

"probers"

"probes"

"probing"

"probit"

"probities"

"probity"

"problem"

"problem solving"

"problematic"

"problematical"

"problems"

"proboscides"

"proboscis"

"proboscises"

"proboycott"

"probusiness"

"proc"

"procaine"

"procapitalist"

"procapitalists"

"procathedral"

"procathedrals"

"procedural"

"procedurally"

"procedurals"

"procedure"

"procedures"

"proceed"

"proceeded"

"proceeder"

"proceeders"

"proceeding"

"proceedings"

"proceeds"

"process"

"processed"

"processes"

"processing"

"procession"

"processional"

"processionally"

"processionals"

"processions"

"processor"

"processors"

"prochurch"

"proclaim"

"proclaimed"

"proclaimer"

"proclaimers"

"proclaiming"

"proclaims"

"proclamation"

"proclamations"

"proclerical"

"proclivities"

"proclivity"

"procommunism"

"procommunist"

"procommunists"

"procompromise"

"proconservation"

"proconsul"

"proconsular"

"proconsulate"

"proconsulates"

"proconsuls"

"proconsulship"

"proconsulships"

"procrastinate"

"procrastinated"

"procrastinates"

"procrastinating"

"procrastination"

"procrastinator"

"procrastinators"

"procreate"

"procreated"

"procreates"

"procreating"

"procreation"

"procreative"

"procreativity"

"procreator"

"procreators"

"procrustean"

"proctologic"

"proctological"

"proctologies"

"proctologist"

"proctologists"

"proctology"

"proctor"

"proctored"

"proctorial"

"proctoring"

"proctors"

"proctorship"

"proctoscope"

"proctoscopes"

"proctoscopic"

"proctoscopically"

"proctoscopies"

"proctoscopy"

"procurable"

"procural"

"procurals"

"procuration"

"procurator"

"procurators"

"procure"

"procured"

"procurement"

"procurer"

"procurers"

"procures"

"procuress"

"procuresses"

"procuring"

"prod"

"prodded"

"prodder"

"prodders"

"prodding"

"prodemocratic"

"prodigal"

"prodigality"

"prodigally"

"prodigals"

"prodigies"

"prodigious"

"prodigiously"

"prodigiousness"

"prodigy"

"prodisarmament"

"prods"

"produce"

"produced"

"producer"

"producers"

"produces"

"producible"

"producing"

"product"

"production"

"productions"

"productive"

"productively"

"productiveness"

"productivity"

"products"

"proem"

"proems"

"proenforcement"

"prof"

"profanation"

"profanations"

"profanatory"

"profane"

"profaned"

"profanely"

"profaneness"

"profaner"

"profaners"

"profanes"

"profaning"

"profanities"

"profanity"

"profascist"

"profascists"

"profeminist"

"profeminists"

"profess"

"professed"

"professedly"

"professes"

"professing"

"profession"

"professional"

"professionalism"

"professionalist"

"professionalists"

"professionalize"

"professionally"

"professionals"

"professions"

"professor"

"professorate"

"professorial"

"professoriate"

"professors"

"professorship"

"professorships"

"proffer"

"proffered"

"profferer"

"profferers"

"proffering"

"proffers"

"proficiency"

"proficient"

"proficiently"

"profile"

"profiled"

"profiler"

"profilers"

"profiles"

"profiling"

"profit"

"profitability"

"profitable"

"profitableness"

"profitably"

"profited"

"profiteer"

"profiteered"

"profiteering"

"profiteers"

"profiter"

"profiters"

"profiting"

"profitless"

"profits"

"profligacy"

"profligate"

"profligately"

"profligates"

"proforma"

"profound"

"profounder"

"profoundest"

"profoundly"

"profoundness"

"profounds"

"profs"

"profundities"

"profundity"

"profuse"

"profusely"

"profuseness"

"profusion"

"progenies"

"progenitive"

"progenitor"

"progenitors"

"progeny"

"prognathous"

"prognose"

"prognosed"

"prognoses"

"prognosis"

"prognostic"

"prognosticate"

"prognosticated"

"prognosticates"

"prognosticating"

"prognostication"

"prognostications"

"prognosticator"

"prognosticators"

"progovernment"

"program"

"programable"

"programed"

"programer"

"programers"

"programing"

"programmability"

"programmable"

"programmata"

"programmatic"

"programmatically"

"programme"

"programmed"

"programmer"

"programmers"

"programmes"

"programming"

"programs"

"progress"

"progressed"

"progresses"

"progressing"

"progression"

"progressional"

"progressionist"

"progressions"

"progressive"

"progressively"

"progressiveness"

"progressives"

"progs"

"prohibit"

"prohibited"

"prohibiting"

"prohibition"

"prohibitionist"

"prohibitionists"

"prohibitions"

"prohibitive"

"prohibitively"

"prohibitor"

"prohibitory"

"prohibits"

"proindustry"

"prointegration"

"prointervention"

"project"

"projected"

"projectile"

"projectiles"

"projecting"

"projection"

"projectionist"

"projectionists"

"projections"

"projector"

"projectors"

"projects"

"prolabor"

"prolapse"

"prolapsed"

"prolapses"

"prolapsing"

"prolate"

"prole"

"prolegomena"

"prolegomenon"

"proles"

"proletarian"

"proletarianize"

"proletarians"

"proletariat"

"proletariate"

"proliferate"

"proliferated"

"proliferates"

"proliferating"

"proliferation"

"proliferations"

"proliferative"

"proliferous"

"proliferously"

"prolific"

"prolifically"

"prolificness"

"prolix"

"prolixity"

"prolixly"

"prolog"

"prologed"

"prologing"

"prologs"

"prologue"

"prologued"

"prologues"

"prologuing"

"prolong"

"prolongation"

"prolongations"

"prolonged"

"prolonges"

"prolonging"

"prolongs"

"prom"

"promenade"

"promenaded"

"promenader"

"promenaders"

"promenades"

"promenading"

"promethean"

"prometheus"

"promethium"

"promilitary"

"prominence"

"prominences"

"prominent"

"prominently"

"promiscuities"

"promiscuity"

"promiscuous"

"promiscuously"

"promiscuousness"

"promise"

"promised"

"promisee"

"promisees"

"promiser"

"promisers"

"promises"

"promising"

"promisingly"

"promisor"

"promisors"

"promissory"

"promodern"

"promonarchist"

"promonarchists"

"promontories"

"promontory"

"promotable"

"promote"

"promote dendrite"

"promoted"

"promoter"

"promoters"

"promotes"

"promoting"

"promotion"

"promotional"

"promotions"

"prompt"

"promptbook"

"promptbooks"

"prompted"

"prompter"

"prompters"

"promptest"

"prompting"

"promptitude"

"promptly"

"promptness"

"prompts"

"proms"

"promulgate"

"promulgated"

"promulgates"

"promulgating"

"promulgation"

"promulgations"

"promulgator"

"promulgators"

"promulged"

"promulges"

"promulging"

"pron"

"pronate"

"pronating"

"pronation"

"pronationalist"

"pronators"

"prone"

"pronely"

"proneness"

"prong"

"pronged"

"pronghorn"

"pronghorns"

"pronging"

"prongs"

"pronominal"

"pronoun"

"pronounce"

"pronounceable"

"pronounced"

"pronouncedly"

"pronouncement"

"pronouncements"

"pronounces"

"pronouncing"

"pronouns"

"pronto"

"pronuclear"

"pronunciamento"

"pronunciamentos"

"pronunciation"

"pronunciations"

"proof"

"proofed"

"proofer"

"proofers"

"proofing"

"proofread"

"proofreader"

"proofreaders"

"proofreading"

"proofreads"

"proofs"

"prop"

"propaganda"

"propagandist"

"propagandistic"

"propagandists"

"propagandize"

"propagandized"

"propagandizes"

"propagandizing"

"propagate"

"propagated"

"propagates"

"propagating"

"propagation"

"propagational"

"propagative"

"propagator"

"propagators"

"propane"

"propanes"

"propanol"

"propel"

"propellant"

"propellants"

"propelled"

"propellent"

"propeller"

"propellers"

"propelling"

"propels"

"propended"

"propensities"

"propensity"

"proper"

"properer"

"properest"

"properitoneal"

"properly"

"properness"

"propers"

"propertied"

"properties"

"property"

"propertyless"

"prophase"

"prophases"

"prophecies"

"prophecy"

"prophesied"

"prophesier"

"prophesiers"

"prophesies"

"prophesy"

"prophesying"

"prophet"

"prophetess"

"prophetesses"

"prophetic"

"prophetical"

"prophetically"

"prophets"

"prophylactic"

"prophylactically"

"prophylactics"

"prophylaxis"

"propinquity"

"propitiate"

"propitiated"

"propitiates"

"propitiating"

"propitiation"

"propitiatory"

"propitious"

"propitiously"

"propjet"

"propjets"

"propman"

"propmen"

"propmistress"

"propmistresses"

"proponent"

"proponents"

"proponing"

"proportion"

"proportional"

"proportionality"

"proportionally"

"proportionate"

"proportionately"

"proportioned"

"proportioning"

"proportions"

"proposal"

"proposals"

"propose"

"proposed"

"proposer"

"proposers"

"proposes"

"proposing"

"proposition"

"propositional"

"propositions"

"propound"

"propounded"

"propounder"

"propounders"

"propounding"

"propounds"

"propped"

"propping"

"propranolol"

"proprietaries"

"proprietary"

"proprieties"

"proprietor"

"proprietorial"

"proprietors"

"proprietorship"

"proprietorships"

"proprietress"

"proprietresses"

"propriety"

"proprioception"

"proprioceptive"

"proprioceptor"

"props"

"propulsion"

"propulsive"

"propyl"

"propylene"

"prorate"

"prorated"

"prorater"

"prorates"

"prorating"

"proration"

"proreform"

"prorestoration"

"prorevolutionary"

"prorogation"

"prorogations"

"prorogue"

"prorogued"

"prorogues"

"proroguing"

"pros"

"prosaic"

"prosaically"

"prosaisms"

"prosaists"

"proscenia"

"proscenium"

"prosceniums"

"proscribe"

"proscribed"

"proscribes"

"proscribing"

"proscription"

"proscriptions"

"proscriptive"

"prose"

"prosecutable"

"prosecute"

"prosecuted"

"prosecutes"

"prosecuting"

"prosecution"

"prosecutions"

"prosecutive"

"prosecutor"

"prosecutorial"

"prosecutors"

"prosecutory"

"prosecutrices"

"prosecutrix"

"prosecutrixes"

"prosed"

"proselyte"

"proselyted"

"proselytes"

"proselyting"

"proselytism"

"proselytize"

"proselytized"

"proselytizer"

"proselytizers"

"proselytizes"

"proselytizing"

"prosequi"

"proser"

"prosers"

"proses"

"prosier"

"prosiest"

"prosily"

"prosing"

"prosit"

"proslavery"

"prosodic"

"prosodies"

"prosody"

"prospect"

"prospected"

"prospecting"

"prospective"

"prospectively"

"prospector"

"prospectors"

"prospects"

"prospectus"

"prospectuses"

"prosper"

"prospered"

"prospering"

"prosperity"

"prosperous"

"prosperously"

"prosperousness"

"prospers"

"prostaglandin"

"prostate"

"prostatectomies"

"prostatectomy"

"prostates"

"prostatic"

"prostatitis"

"prostheses"

"prosthesis"

"prosthetic"

"prosthetically"

"prosthetics"

"prosthetist"

"prosthodontia"

"prosthodontics"

"prosthodontist"

"prostitute"

"prostituted"

"prostitutes"

"prostituting"

"prostitution"

"prostrate"

"prostrated"

"prostrates"

"prostrating"

"prostration"

"prostrations"

"prostyle"

"prostyles"

"prosuffrage"

"prosy"

"protactinium"

"protagonist"

"protagonists"

"protea"

"protean"

"proteas"

"protect"

"protected"

"protecting"

"protection"

"protectional"

"protectionism"

"protectionist"

"protectionists"

"protective"

"protectively"

"protectiveness"

"protector"

"protectorate"

"protectorates"

"protectors"

"protectress"

"protectresses"

"protects"

"protege"

"protegee"

"protegees"

"proteges"

"protein"

"proteinaceous"

"proteins"

"protest"

"protestable"

"protestant"

"protestantism"

"protestants"

"protestation"

"protestations"

"protested"

"protester"

"protesters"

"protesting"

"protestor"

"protestors"

"protests"

"proteus"

"prothalamia"

"prothalamion"

"protist"

"protista"

"protistology"

"protists"

"protoactinium"

"protocol"

"protocols"

"proton"

"protonic"

"protons"

"protoplasm"

"protoplasmal"

"protoplasmatic"

"protoplasmic"

"prototype"

"prototypes"

"prototypic"

"prototypical"

"protozoa"

"protozoal"

"protozoan"

"protozoans"

"protozoic"

"protozoology"

"protozoon"

"protract"

"protracted"

"protractile"

"protracting"

"protraction"

"protractor"

"protractors"

"protracts"

"protrude"

"protruded"

"protrudes"

"protruding"

"protrusile"

"protrusion"

"protrusions"

"protrusive"

"protuberance"

"protuberances"

"protuberant"

"proud"

"prouder"

"proudest"

"proudly"

"proudness"

"prounion"

"provability"

"provable"

"provably"

"prove"

"proved"

"proven"

"provenance"

"provenances"

"provencal"

"provence"

"provender"

"provenly"

"prover"

"proverb"

"proverbed"

"proverbial"

"proverbing"

"proverbs"

"provers"

"proves"

"provide"

"provided"

"providence"

"provident"

"providential"

"providentially"

"providently"

"provider"

"providers"

"provides"

"providing"

"province"

"provinces"

"provincial"

"provincialism"

"provinciality"

"provincially"

"proving"

"provision"

"provisional"

"provisionally"

"provisions"

"proviso"

"provisoes"

"provisos"

"provocateur"

"provocateurs"

"provocation"

"provocations"

"provocative"

"provocatively"

"provocativeness"

"provoke"

"provoked"

"provoker"

"provokers"

"provokes"

"provoking"

"provokingly"

"provolone"

"provost"

"provosts"

"prow"

"prowar"

"prowess"

"prowesses"

"prowl"

"prowled"

"prowler"

"prowlers"

"prowling"

"prowls"

"prows"

"proxemics"

"proxies"

"proxima"

"proximal"

"proximate"

"proximately"

"proximity"

"proximo"

"proxy"

"prs"

"prude"

"prudence"

"prudences"

"prudent"

"prudential"

"prudentially"

"prudently"

"pruderies"

"prudery"

"prudes"

"prudish"

"prudishly"

"prudishness"

"prunable"

"prune"

"pruned"

"pruner"

"pruners"

"prunes"

"pruning"

"prurience"

"prurient"

"pruriently"

"prussia"

"prussian"

"prussians"

"prussic"

"pry"

"pryer"

"pryers"

"prying"

"pryingly"

"prythee"

"psalligraphy"

"psalm"

"psalmed"

"psalmic"

"psalming"

"psalmist"

"psalmists"

"psalmody"

"psalms"

"psalter"

"psalteries"

"psalters"

"psaltery"

"psaltries"

"psaltry"

"pschent"

"pschents"

"psephology"

"pseud"

"pseudo"

"pseudoaristocratic"

"pseudoartistic"

"pseudobiographical"

"pseudoclassic"

"pseudoclassical"

"pseudoclassicism"

"pseudoephedrine"

"pseudohistoric"

"pseudohistorical"

"pseudointellectual"

"pseudointellectuals"

"pseudolegendary"

"pseudoliberal"

"pseudoliterary"

"pseudology"

"pseudomania"

"pseudomodern"

"pseudonym"

"pseudonymous"

"pseudonyms"

"pseudoparalyses"

"pseudoparalysis"

"pseudophilosophical"

"pseudopod"

"pseudopodia"

"pseudopodium"

"pseudoprofessional"

"pseudoptics"

"pseudoscholarly"

"pseudoscientific"

"pseudoscientifically"

"psf"

"pshaw"

"pshawed"

"pshawing"

"pshaws"

"psi"

"psilocybin"

"psophometer"

"psoriases"

"psoriasis"

"psst"

"psych"

"psyche"

"psyched"

"psychedelic"

"psychedelically"

"psychedelics"

"psyches"

"psychiatric"

"psychiatric codes"

"psychiatrical"

"psychiatrically"

"psychiatries"

"psychiatrist"

"psychiatrists"

"psychiatry"

"psychic"

"psychical"

"psychically"

"psychics"

"psyching"

"psycho"

"psycho health"

"psychoactive"

"psychoanalyses"

"psychoanalysis"

"psychoanalyst"

"psychoanalysts"

"psychoanalytic"

"psychoanalytical"

"psychoanalytically"

"psychoanalyze"

"psychoanalyzed"

"psychoanalyzes"

"psychoanalyzing"

"psychobiology"

"psychodrama"

"psychodramas"

"psychodynamic"

"psychodynamics"

"psychogenetics"

"psychogenic"

"psychogenically"

"psychognosy"

"psychograph"

"psychokineses"

"psychokinesia"

"psychokinesis"

"psychol"

"psychologic"

"psychological"

"psychologically"

"psychologies"

"psychologism"

"psychologist"

"psychologists"

"psychologize"

"psychologized"

"psychologizing"

"psychology"

"psychometrics"

"psychometries"

"psychometry"

"psychomotor"

"psychoneuroses"

"psychoneurosis"

"psychoneurotic"

"psychopath"

"psychopathia"

"psychopathic"

"psychopathically"

"psychopathies"

"psychopathologic"

"psychopathological"

"psychopathologically"

"psychopathology"

"psychopaths"

"psychopathy"

"psychophysical"

"psychophysically"

"psychophysics"

"psychophysiology"

"psychoquackeries"

"psychos"

"psychosensory"

"psychoses"

"psychosexual"

"psychosexuality"

"psychosexually"

"psychosis"

"psychosocial"

"psychosocially"

"psychosomatic"

"psychosomatics"

"psychosyntheses"

"psychosynthesis"

"psychotherapies"

"psychotherapist"

"psychotherapists"

"psychotherapy"

"psychotic"

"psychotically"

"psychotics"

"psychotogen"

"psychotogenic"

"psychotomimetic"

"psychotoxic"

"psychotropic"

"psychrometer"

"psychrophobia"

"psychrotherapies"

"psychs"

"ptarmigan"

"ptarmigans"

"pteridology"

"pteridomania"

"pterodactyl"

"pterodactyls"

"pteronophobia"

"pterylology"

"ptolemaic"

"ptolemy"

"ptomain"

"ptomaine"

"ptomaines"

"ptomainic"

"pts"

"pub"

"pubertal"

"puberties"

"puberty"

"pubes"

"pubescence"

"pubescent"

"pubic"

"pubis"

"public"

"publican"

"publicans"

"publication"

"publications"

"publicist"

"publicists"

"publicity"

"publicize"

"publicized"

"publicizes"

"publicizing"

"publicly"

"publics"

"publish"

"publishable"

"published"

"publisher"

"publishers"

"publishes"

"publishing"

"pubs"

"puccini"

"puce"

"puces"

"puck"

"pucker"

"puckered"

"puckerer"

"puckerers"

"puckerier"

"puckering"

"puckers"

"puckery"

"puckish"

"pucks"

"pud"

"pudding"

"puddings"

"puddle"

"puddled"

"puddler"

"puddlers"

"puddles"

"puddlier"

"puddliest"

"puddling"

"puddlings"

"puddly"

"pudenda"

"pudendum"

"pudgier"

"pudgiest"

"pudgily"

"pudginess"

"pudgy"

"puds"

"pueblo"

"pueblos"

"puerile"

"puerilely"

"puerilities"

"puerility"

"puerperal"

"puerto"

"Puerto Rico"

"puff"

"puffball"

"puffballs"

"puffed"

"puffer"

"pufferies"

"puffers"

"puffery"

"puffier"

"puffiest"

"puffily"

"puffin"

"puffiness"

"puffing"

"puffins"

"puffs"

"puffy"

"pug"

"pugged"

"puggish"

"puggy"

"pugilism"

"pugilisms"

"pugilist"

"pugilistic"

"pugilists"

"pugnacious"

"pugnaciously"

"pugnaciousness"

"pugnacity"

"pugs"

"puissance"

"puissant"

"puissantly"

"puke"

"puked"

"pukes"

"puking"

"pukka"

"pulchritude"

"pulchritudinous"

"pule"

"puled"

"puler"

"pulers"

"pules"

"puling"

"pulingly"

"pulings"

"pulitzer"

"pull"

"pullback"

"pullbacks"

"pulldown"

"pulled"

"puller"

"pullers"

"pullet"

"pullets"

"pulley"

"pulleys"

"pulling"

"pullman"

"pullmans"

"pullout"

"pullouts"

"pullover"

"pullovers"

"pulls"

"pulmonary"

"pulmonectomies"

"pulmonic"

"pulmotor"

"pulmotors"

"pulp"

"pulped"

"pulper"

"pulpers"

"pulpier"

"pulpiest"

"pulpily"

"pulping"

"pulpit"

"pulpital"

"pulpits"

"pulps"

"pulpwood"

"pulpwoods"

"pulpy"

"pulque"

"pulques"

"pulsar"

"pulsars"

"pulsate"

"pulsated"

"pulsates"

"pulsating"

"pulsation"

"pulsations"

"pulsator"

"pulsators"

"pulsatory"

"pulse"

"pulsed"

"pulsejet"

"pulsejets"

"pulser"

"pulsers"

"pulses"

"pulsimeter"

"pulsing"

"pulsions"

"pulverization"

"pulverize"

"pulverized"

"pulverizes"

"pulverizing"

"puma"

"pumas"

"pumice"

"pumiced"

"pumicer"

"pumicers"

"pumices"

"pumicing"

"pumicites"

"pummel"

"pummeled"

"pummeling"

"pummelled"

"pummelling"

"pummels"

"pump"

"pumped"

"pumper"

"pumpernickel"

"pumpers"

"pumping"

"pumpkin"

"pumpkins"

"pumps"

"pun"

"punch"

"punched"

"puncheon"

"puncheons"

"puncher"

"punchers"

"punches"

"punchier"

"punchiest"

"punching"

"punchy"

"punctatim"

"punctilio"

"punctilios"

"punctilious"

"punctiliously"

"punctiliousness"

"punctual"

"punctuality"

"punctually"

"punctualness"

"punctuate"

"punctuated"

"punctuates"

"punctuating"

"punctuation"

"puncture"

"punctured"

"punctures"

"puncturing"

"pundit"

"punditic"

"punditry"

"pundits"

"pungencies"

"pungency"

"pungent"

"pungently"

"punier"

"puniest"

"punily"

"puniness"

"punish"

"punishability"

"punishable"

"punishably"

"punished"

"punisher"

"punishers"

"punishes"

"punishing"

"punishment"

"punishments"

"punitions"

"punitive"

"punitively"

"punk"

"punker"

"punkest"

"punkey"

"punkie"

"punkier"

"punkin"

"punkins"

"punks"

"punky"

"punned"

"punner"

"punners"

"punnier"

"punning"

"punny"

"puns"

"punster"

"punsters"

"punt"

"punted"

"punter"

"punters"

"punting"

"punts"

"punty"

"puny"

"pup"

"pupa"

"pupae"

"pupal"

"pupas"

"pupate"

"pupated"

"pupates"

"pupating"

"pupation"

"pupations"

"pupfish"

"pupfishes"

"pupil"

"pupilar"

"pupillary"

"pupillometries"

"pupils"

"pupped"

"puppet"

"puppeteer"

"puppeteers"

"puppetries"

"puppetry"

"puppets"

"puppies"

"pupping"

"puppy"

"puppydoms"

"puppyish"

"pups"

"purblind"

"purblindness"

"purchasable"

"purchase"

"purchaseable"

"purchased"

"purchaser"

"purchasers"

"purchases"

"purchasing"

"purdah"

"purdahs"

"purdas"

"pure"

"Pure sounds"

"purebred"

"purebreds"

"puree"

"pureed"

"pureeing"

"purees"

"purely"

"pureness"

"purer"

"purest"

"purgation"

"purgations"

"purgative"

"purgatively"

"purgatives"

"purgatorial"

"purgatories"

"purgatory"

"purge"

"purged"

"purger"

"purgers"

"purges"

"purging"

"purgings"

"purification"

"purifications"

"purificatory"

"purified"

"purifier"

"purifiers"

"purifies"

"purify"

"purifying"

"purim"

"purine"

"purins"

"purism"

"purisms"

"purist"

"puristic"

"purists"

"puritan"

"puritanical"

"puritanically"

"puritanism"

"puritans"

"purities"

"purity"

"purl"

"purled"

"purlieu"

"purlieus"

"purling"

"purloin"

"purloined"

"purloiner"

"purloiners"

"purloining"

"purloins"

"purls"

"purple"

"purpled"

"purpler"

"purples"

"purplest"

"purpling"

"purplish"

"purply"

"purport"

"purported"

"purportedly"

"purporting"

"purports"

"purpose"

"purposed"

"purposeful"

"purposefully"

"purposefulness"

"purposeless"

"purposelessly"

"purposelessness"

"purposely"

"purposes"

"purposing"

"purposive"

"purpresture"

"purr"

"purred"

"purring"

"purrs"

"purse"

"pursed"

"purser"

"pursers"

"purses"

"pursier"

"pursily"

"pursing"

"purslane"

"purslanes"

"pursuable"

"pursuance"

"pursuant"

"pursue"

"pursued"

"pursuer"

"pursuers"

"pursues"

"pursuing"

"pursuit"

"pursuits"

"pursy"

"purulence"

"purulences"

"purulencies"

"purulency"

"purulent"

"purulently"

"puruloid"

"purvey"

"purveyance"

"purveyed"

"purveying"

"purveyor"

"purveyors"

"purveys"

"purview"

"purviews"

"pus"

"push"

"pushcart"

"pushcarts"

"pushed"

"pusher"

"pushers"

"pushes"

"pushier"

"pushiest"

"pushily"

"pushiness"

"pushing"

"pushover"

"pushovers"

"pushpin"

"pushpins"

"pushup"

"pushups"

"pushy"

"pusillanimity"

"pusillanimous"

"pusillanimously"

"puslike"

"puss"

"pusses"

"pussier"

"pussies"

"pussiest"

"pussy"

"pussycat"

"pussycats"

"pussyfoot"

"pussyfooted"

"pussyfooting"

"pussyfoots"

"pustular"

"pustulating"

"pustulation"

"pustule"

"pustuled"

"pustules"

"pustuliform"

"put"

"put on a headphone"

"putative"

"putatively"

"putdown"

"putdowns"

"putoff"

"putoffs"

"puton"

"putons"

"putout"

"putouts"

"putrefaction"

"putrefactive"

"putrefied"

"putrefies"

"putrefy"

"putrefying"

"putrescence"

"putrescent"

"putrid"

"putridity"

"putridly"

"putridness"

"puts"

"putsch"

"putsches"

"putt"

"putted"

"puttee"

"puttees"

"putter"

"puttered"

"putterer"

"putterers"

"puttering"

"putters"

"puttied"

"puttier"

"puttiers"

"putties"

"putting"

"putts"

"putty"

"puttying"

"puy"

"puzzle"

"puzzled"

"puzzlement"

"puzzler"

"puzzlers"

"puzzles"

"puzzling"

"puzzlingly"

"pycnometer"

"pyelonephritis"

"pygmalionism"

"pygmies"

"pygmoid"

"pygmy"

"pygmyish"

"pygmyism"

"pygmyisms"

"pyjamas"

"pyknometer"

"pylon"

"pylons"

"pylori"

"pyloric"

"pylorous"

"pylorouses"

"pylorus"

"pyloruses"

"pyongyang"

"pyorrhea"

"pyorrheas"

"pyorrhoea"

"pyramid"

"pyramidal"

"pyramided"

"pyramiding"

"pyramids"

"pyranometer"

"pyre"

"pyrenees"

"pyrenes"

"pyres"

"pyrethrin"

"pyrethrum"

"pyretology"

"pyrex"

"pyrgeometer"

"pyrgology"

"pyrheliometer"

"pyric"

"pyrimidine"

"pyrite"

"pyrites"

"pyritic"

"pyroballogy"

"pyrogen"

"pyrography"

"pyrolysis"

"pyromania"

"pyromaniac"

"pyromaniacal"

"pyromaniacs"

"pyrometer"

"pyrometers"

"pyrophobia"

"pyroscope"

"pyrostats"

"pyrotechnic"

"pyrotechnical"

"pyrotechnics"

"pyrrhic"

"pyruvic"

"pythagoras"

"pythagorean"

"pythagoreans"

"python"

"pythons"

"pyx"

"pyxes"

"pyxie"

"pyxies"

"pyxis"

"QA of health"

"QA of health collection"

"qaid"

"qaids"

"qat"

"qatar"

"qed"

"qiana"

"qindars"

"qintars"

"qoph"

"qophs"

"QTc CALCULATOR"

"qts"

"qty"

"qua"

"quaalude"

"quaaludes"

"quack"

"quacked"

"quackeries"

"quackery"

"quackier"

"quackiest"

"quacking"

"quackish"

"quackishly"

"quackishness"

"quackism"

"quackisms"

"quacks"

"quacksalver"

"quackster"

"quacky"

"quad"

"quadded"

"quadrangle"

"quadrangles"

"quadrangular"

"quadrant"

"quadrantal"

"quadrants"

"quadraphonic"

"quadrat"

"quadrate"

"quadrated"

"quadrates"

"quadratic"

"quadratics"

"quadrennial"

"quadrennials"

"quadrennium"

"quadrenniums"

"quadric"

"quadricentennial"

"quadricentennials"

"quadriceps"

"quadrics"

"quadriennium"

"quadrigamist"

"quadrilateral"

"quadrilaterals"

"quadrille"

"quadrilles"

"quadrillion"

"quadrillions"

"quadrillionth"

"quadrillionths"

"quadripartite"

"quadriplegia"

"quadriplegic"

"quadrivium"

"quadroon"

"quadroons"

"quadrumvirate"

"quadruped"

"quadrupedal"

"quadrupeds"

"quadruple"

"quadrupled"

"quadruples"

"quadruplet"

"quadruplets"

"quadruplicate"

"quadruplicated"

"quadruplicates"

"quadruplicating"

"quadruplication"

"quadruplications"

"quadrupling"

"quads"

"quae"

"quaestor"

"quaff"

"quaffed"

"quaffer"

"quaffers"

"quaffing"

"quaffs"

"quag"

"quagga"

"quaggas"

"quaggier"

"quaggiest"

"quaggy"

"quagmire"

"quagmires"

"quagmiry"

"quags"

"quahaug"

"quahaugs"

"quahog"

"quahogs"

"quai"

"quail"

"quailed"

"quailing"

"quails"

"quaint"

"quainter"

"quaintest"

"quaintly"

"quaintness"

"quais"

"quake"

"quaked"

"quaker"

"quakerism"

"quakers"

"quakes"

"quakier"

"quakiest"

"quakily"

"quaking"

"quakingly"

"quaky"

"qual"

"quale"

"qualification"

"qualifications"

"qualified"

"qualifiedly"

"qualifier"

"qualifiers"

"qualifies"

"qualify"

"qualifying"

"qualimeter"

"qualitative"

"qualitatively"

"qualities"

"quality"

"qualm"

"qualmier"

"qualmiest"

"qualmish"

"qualmishly"

"qualmishness"

"qualms"

"qualmy"

"quam"

"quandaries"

"quandary"

"quando"

"quant"

"quanta"

"quantal"

"quanted"

"quanti"

"quantic"

"quantified"

"quantifies"

"quantify"

"quantifying"

"quantimeter"

"quantitative"

"quantitatively"

"quantities"

"quantity"

"quantize"

"quantized"

"quantizes"

"quantizing"

"quantometer"

"quants"

"quantum"

"quarantinable"

"quarantine"

"quarantined"

"quarantines"

"quarantining"

"quark"

"quarks"

"quarrel"

"quarreled"

"quarreler"

"quarrelers"

"quarreling"

"quarrelled"

"quarreller"

"quarrellers"

"quarrelling"

"quarrels"

"quarrelsome"

"quarried"

"quarrier"

"quarriers"

"quarries"

"quarry"

"quarrying"

"quarrymen"

"quart"

"quartan"

"quarte"

"quarter"

"quarterback"

"quarterbacks"

"quarterdeck"

"quarterdecks"

"quartered"

"quarterfinal"

"quarterfinalist"

"quartering"

"quarterings"

"quarterlies"

"quarterly"

"quartermaster"

"quartermasters"

"quarters"

"quarterstaff"

"quarterstaves"

"quartes"

"quartet"

"quartets"

"quartic"

"quartics"

"quartile"

"quartiles"

"quarto"

"quartos"

"quarts"

"quartz"

"quartzes"

"quartzite"

"quasar"

"quasars"

"quash"

"quashed"

"quashes"

"quashing"

"quasi"

"quat"

"quatenus"

"quaternary"

"quatorze"

"quatorzes"

"quatrain"

"quatrains"

"quatre"

"quatrefoil"

"quatrefoils"

"quatres"

"quaver"

"quavered"

"quaverer"

"quaverers"

"quavering"

"quaveringly"

"quavers"

"quavery"

"quay"

"quayage"

"quayages"

"quays"

"quayside"

"quaysides"

"que"

"quean"

"queans"

"queasier"

"queasiest"

"queasily"

"queasiness"

"queasy"

"queaziest"

"queazy"

"quebec"

"queen"

"queened"

"queening"

"queenlier"

"queenliest"

"queenliness"

"queenly"

"queens"

"queer"

"queered"

"queerer"

"queerest"

"queering"

"queerish"

"queerly"

"queerness"

"queers"

"quell"

"quelled"

"queller"

"quellers"

"quelling"

"quells"

"quem"

"quench"

"quenchable"

"quenched"

"quencher"

"quenchers"

"quenches"

"quenching"

"quenchless"

"queried"

"querier"

"queriers"

"queries"

"querist"

"querists"

"quern"

"querns"

"querulous"

"querulously"

"querulousness"

"query"

"querying"

"ques"

"quest"

"quested"

"quester"

"questers"

"questing"

"question"

"questionability"

"questionable"

"questionableness"

"questionably"

"questioned"

"questioner"

"questioners"

"questioning"

"questionnaire"

"questionnaire System"

"questionnaires"

"questions"

"questor"

"questors"

"quests"

"quetzal"

"quetzales"

"quetzals"

"queue"

"queued"

"queueing"

"queuer"

"queuers"

"queues"

"queuing"

"quey"

"queys"

"quezal"

"quezals"

"qui"

"quia"

"quibble"

"quibbled"

"quibbler"

"quibblers"

"quibbles"

"quibbling"

"quiche"

"quiches"

"quick"

"quicken"

"quickened"

"quickening"

"quickens"

"quicker"

"quickest"

"quickie"

"quickies"

"quicklime"

"quickly"

"quickness"

"quicks"

"quicksand"

"quicksets"

"quicksilver"

"quickstep"

"quicksteps"

"quid"

"quiddities"

"quiddity"

"quidnunc"

"quids"

"quiescence"

"quiescency"

"quiescent"

"quiet"

"quieta"

"quieted"

"quieten"

"quietened"

"quietening"

"quietens"

"quieter"

"quieters"

"quietest"

"quieti"

"quieting"

"quietism"

"quietisms"

"quietist"

"quietists"

"quietly"

"quietness"

"quiets"

"quietude"

"quietudes"

"quietus"

"quietuses"

"quill"

"quilled"

"quills"

"quilt"

"quilted"

"quilter"

"quilters"

"quilting"

"quiltings"

"quilts"

"quince"

"quinces"

"quincunx"

"quincunxes"

"quinic"

"quinin"

"quinine"

"quinines"

"quinins"

"quinology"

"quinols"

"quinone"

"quinquina"

"quinsies"

"quinsy"

"quint"

"quintain"

"quintains"

"quintal"

"quintals"

"quintan"

"quintar"

"quintessence"

"quintessential"

"quintet"

"quintets"

"quintette"

"quintic"

"quintics"

"quintile"

"quintiles"

"quintillion"

"quintillions"

"quintillionth"

"quintillionths"

"quintin"

"quints"

"quintuple"

"quintupled"

"quintuples"

"quintuplet"

"quintuplets"

"quintuplicate"

"quintuplicated"

"quintuplicates"

"quintuplicating"

"quintupling"

"quip"

"quipped"

"quipping"

"quippish"

"quips"

"quipster"

"quipsters"

"quipu"

"quipus"

"quire"

"quired"

"quires"

"quiring"

"quirk"

"quirked"

"quirkier"

"quirkiest"

"quirkily"

"quirkiness"

"quirking"

"quirks"

"quirky"

"quirt"

"quirted"

"quirts"

"quisling"

"quislings"

"quit"

"quitclaim"

"quitclaimed"

"quitclaiming"

"quitclaims"

"quite"

"quito"

"quitrents"

"quits"

"quittance"

"quittances"

"quitted"

"quitter"

"quitters"

"quitting"

"quittors"

"quiver"

"quivered"

"quiverer"

"quiverers"

"quivering"

"quiveringly"

"quivers"

"quivery"

"quixote"

"quixotes"

"quixotic"

"quixotically"

"quixotries"

"quixotry"

"quiz"

"quizzed"

"quizzer"

"quizzers"

"quizzes"

"quizzical"

"quizzicality"

"quizzically"

"quizzicalness"

"quizzing"

"quo"

"quoad"

"quod"

"quods"

"quoin"

"quoined"

"quoins"

"quoit"

"quoited"

"quoits"

"quondam"

"quonset"

"quorum"

"quorums"

"quos"

"quota"

"quotable"

"quotably"

"quotas"

"quotation"

"quotational"

"quotationally"

"quotations"

"quote"

"quoted"

"quoter"

"quoters"

"quotes"

"quoth"

"quotha"

"quotidian"

"quotient"

"quotients"

"quoting"

"qursh"

"qurush"

"r"

"rab"

"rabbet"

"rabbeted"

"rabbeting"

"rabbets"

"rabbi"

"rabbinate"

"rabbinates"

"rabbinic"

"rabbinical"

"rabbis"

"rabbit"

"rabbiters"

"rabbiting"

"rabbits"

"rabble"

"rabbles"

"rabelais"

"rabelaisian"

"rabic"

"rabid"

"rabidities"

"rabidity"

"rabidly"

"rabidness"

"rabies"

"raccoon"

"raccoons"

"race"

"racecourse"

"racecourses"

"raced"

"racehorse"

"racehorses"

"raceme"

"racemes"

"racemose"

"racer"

"racers"

"races"

"racetrack"

"racetracks"

"raceway"

"raceways"

"rachets"

"rachiometer"

"rachitic"

"rachitis"

"racial"

"racialism"

"racialist"

"racialistic"

"racialists"

"racially"

"racier"

"raciest"

"racily"

"raciness"

"racing"

"racings"

"raciology"

"racism"

"racisms"

"racist"

"racists"

"rack"

"racked"

"racker"

"rackers"

"racket"

"racketed"

"racketeer"

"racketeering"

"racketeers"

"racketier"

"racketiest"

"racketing"

"rackets"

"rackety"

"racking"

"racks"

"raconteur"

"raconteurs"

"racoon"

"racoons"

"racquet"

"racquetball"

"racquets"

"racy"

"rad"

"radar"

"radarman"

"radars"

"radarscope"

"radarscopes"

"raddle"

"raddled"

"raddles"

"raddling"

"radial"

"radially"

"radials"

"radian"

"radiance"

"radiances"

"radiancies"

"radiancy"

"radians"

"radiant"

"radiantly"

"radiants"

"radiate"

"radiated"

"radiates"

"radiating"

"radiation"

"radiations"

"radiative"

"radiator"

"radiators"

"radical"

"radicalism"

"radicalization"

"radicalize"

"radicalized"

"radicalizes"

"radicalizing"

"radically"

"radicalness"

"radicals"

"radicands"

"radicated"

"radicates"

"radii"

"radio"

"radioactive"

"radioactively"

"radioactivities"

"radioactivity"

"radiobiologic"

"radiobiology"

"radiobroadcast"

"radiobroadcaster"

"radiobroadcasters"

"radiocarbon"

"radiocast"

"radiocaster"

"radiochemical"

"radiochemist"

"radiochemistry"

"radioed"

"radioelement"

"radiogenic"

"radiogoniometer"

"radiogram"

"radiograms"

"radiograph"

"radiographer"

"radiographic"

"radiographically"

"radiographies"

"radiographs"

"radiography"

"radioing"

"radioisotope"

"radioisotopes"

"radioisotopic"

"radiologic"

"radiological"

"radiologically"

"radiologies"

"radiologist"

"radiologists"

"radiology"

"radiolucencies"

"radiolucency"

"radioman"

"radiomen"

"radiometeorograph"

"radiometer"

"radiometers"

"radiometric"

"radiometrically"

"radiometries"

"radiometry"

"radiophone"

"radiophones"

"radios"

"radioscope"

"radioscopic"

"radioscopical"

"radioscopy"

"radiosensitive"

"radiosensitivities"

"radiosensitivity"

"radiosonde"

"radiosondes"

"radiosurgeries"

"radiotelegraph"

"radiotelegraphic"

"radiotelegraphically"

"radiotelegraphs"

"radiotelegraphy"

"radiotelemetric"

"radiotelemetries"

"radiotelemetry"

"radiotelephone"

"radiotelephones"

"radiotelephonic"

"radiotelephony"

"radiotherapies"

"radiotherapist"

"radiotherapists"

"radiotherapy"

"radish"

"radishes"

"radium"

"radiums"

"radius"

"radiuses"

"radix"

"radixes"

"radome"

"radomes"

"radon"

"radons"

"rads"

"raffia"

"raffias"

"raffish"

"raffishly"

"raffishness"

"raffle"

"raffled"

"raffler"

"rafflers"

"raffles"

"raffling"

"raft"

"raftage"

"rafted"

"rafter"

"rafters"

"rafting"

"rafts"

"raftsman"

"raftsmen"

"rag"

"raga"

"ragamuffin"

"ragamuffins"

"ragas"

"ragbag"

"ragbags"

"rage"

"raged"

"rages"

"ragged"

"raggeder"

"raggedest"

"raggedly"

"raggedness"

"raggedy"

"ragging"

"raggle"

"raggy"

"raging"

"ragingly"

"raglan"

"raglans"

"ragman"

"ragmen"

"ragout"

"ragouting"

"ragouts"

"rags"

"ragtag"

"ragtags"

"ragtime"

"ragtimes"

"ragweed"

"ragweeds"

"ragwort"

"ragworts"

"rah"

"raid"

"raided"

"raider"

"raiders"

"raiding"

"raids"

"rail"

"railbird"

"railed"

"railer"

"railers"

"railhead"

"railheads"

"railing"

"railings"

"railleries"

"raillery"

"railroad"

"railroaded"

"railroader"

"railroaders"

"railroading"

"railroads"

"rails"

"railside"

"railway"

"railways"

"raiment"

"raiments"

"rain"

"rainbow"

"rainbows"

"raincoat"

"raincoats"

"raindrop"

"raindrops"

"rained"

"rainfall"

"rainfalls"

"rainier"

"rainiest"

"rainily"

"raininess"

"raining"

"rainmaker"

"rainmakers"

"rainmaking"

"rainout"

"rainproof"

"rains"

"rainstorm"

"rainstorms"

"rainwater"

"rainwear"

"rainwears"

"rainy"

"raisable"

"raise"

"raised"

"raiser"

"raisers"

"raises"

"raisin"

"raising"

"raisings"

"raisins"

"raisiny"

"raison"

"raisons"

"raja"

"rajah"

"rajahs"

"rajas"

"rake"

"raked"

"rakehell"

"rakehells"

"rakeoff"

"rakeoffs"

"raker"

"rakers"

"rakes"

"raking"

"rakish"

"rakishly"

"rakishness"

"rale"

"rales"

"rallied"

"rallier"

"ralliers"

"rallies"

"rally"

"rallye"

"rallyes"

"rallying"

"rallyings"

"rallyist"

"rallyists"

"ralph"

"ram"

"ramble"

"rambled"

"rambler"

"ramblers"

"rambles"

"rambling"

"rambunctious"

"rambunctiousness"

"ramekin"

"ramekins"

"ramie"

"ramies"

"ramification"

"ramifications"

"ramified"

"ramifies"

"ramify"

"ramifying"

"ramjet"

"ramjets"

"rammed"

"rammer"

"rammers"

"ramming"

"rammish"

"ramp"

"rampage"

"rampaged"

"rampageous"

"rampager"

"rampagers"

"rampages"

"rampaging"

"rampancies"

"rampancy"

"rampant"

"rampart"

"ramparted"

"ramparting"

"ramparts"

"ramped"

"ramping"

"rampion"

"ramps"

"ramrod"

"ramrods"

"rams"

"ramshackle"

"ramshorn"

"ramshorns"

"ran"

"ranch"

"ranched"

"rancher"

"ranchero"

"rancheros"

"ranchers"

"ranches"

"ranching"

"ranchman"

"ranchmen"

"rancho"

"ranchos"

"rancid"

"rancidification"

"rancidified"

"rancidifying"

"rancidities"

"rancidity"

"rancidness"

"rancor"

"rancored"

"rancorous"

"rancorously"

"rancors"

"rancour"

"rancours"

"rand"

"randier"

"randiest"

"random"

"randomization"

"randomize"

"randomized"

"randomizes"

"randomizing"

"randomly"

"randomness"

"randoms"

"rands"

"randy"

"ranee"

"ranees"

"rang"

"range"

"ranged"

"rangelands"

"ranger"

"rangers"

"ranges"

"rangier"

"rangiest"

"ranginess"

"ranging"

"rangoon"

"rangy"

"rani"

"ranis"

"rank"

"ranked"

"ranker"

"rankers"

"rankest"

"ranking"

"rankings"

"rankish"

"rankle"

"rankled"

"rankles"

"rankling"

"ranklingly"

"rankly"

"rankness"

"ranks"

"ransack"

"ransacked"

"ransacker"

"ransackers"

"ransacking"

"ransacks"

"ransom"

"ransomable"

"ransomed"

"ransomer"

"ransomers"

"ransoming"

"ransoms"

"rant"

"ranted"

"ranter"

"ranters"

"ranting"

"rantingly"

"rants"

"rap"

"rapacious"

"rapaciously"

"rapaciousness"

"rapacities"

"rapacity"

"rape"

"raped"

"raper"

"rapers"

"rapes"

"rapeseed"

"rapeseeds"

"raphael"

"rapid"

"rapider"

"rapidest"

"rapidities"

"rapidity"

"rapidly"

"rapidness"

"rapids"

"rapier"

"rapiered"

"rapiers"

"rapine"

"rapines"

"raping"

"rapist"

"rapists"

"rapped"

"rappel"

"rappelled"

"rappelling"

"rappels"

"rapper"

"rappers"

"rapping"

"rapport"

"rapporteur"

"rapports"

"rapprochement"

"rapprochements"

"raps"

"rapscallion"

"rapscallions"

"rapt"

"rapter"

"raptest"

"raptly"

"raptness"

"raptor"

"raptorial"

"raptors"

"rapture"

"raptured"

"raptures"

"rapturing"

"rapturous"

"rapturously"

"rapturousness"

"raqi"

"rara"

"rare"

"rarebit"

"rarebits"

"rarefaction"

"rarefied"

"rarefier"

"rarefiers"

"rarefies"

"rarefy"

"rarefying"

"rarely"

"rareness"

"rarer"

"rarest"

"rarified"

"rarify"

"rarifying"

"raring"

"rarities"

"rarity"

"ras"

"rascal"

"rascality"

"rascally"

"rascals"

"rase"

"rased"

"raser"

"rasers"

"rases"

"rash"

"rasher"

"rashers"

"rashes"

"rashest"

"rashly"

"rashness"

"rasing"

"rasp"

"raspberries"

"raspberry"

"rasped"

"rasper"

"raspers"

"raspier"

"raspiest"

"rasping"

"raspingly"

"raspish"

"rasps"

"raspy"

"rassle"

"rassled"

"rassles"

"rassling"

"rastafarian"

"raster"

"rasters"

"rat"

"rata"

"ratability"

"ratable"

"ratably"

"ratatat"

"ratch"

"ratchet"

"ratchets"

"rate"

"rate of breathing"

"rateable"

"rateably"

"rated"

"ratemeter"

"ratepayer"

"rater"

"raters"

"rates"

"ratfink"

"ratfinks"

"ratfish"

"rather"

"rathole"

"ratholes"

"rathskeller"

"rathskellers"

"raticides"

"ratification"

"ratified"

"ratifier"

"ratifiers"

"ratifies"

"ratify"

"ratifying"

"rating"

"ratings"

"ratio"

"ratiocinate"

"ratiocinated"

"ratiocinates"

"ratiocinating"

"ratiocination"

"ratiocinations"

"ratiocinative"

"ratiocinator"

"ratiocinators"

"ration"

"rational"

"rationale"

"rationales"

"rationalism"

"rationalist"

"rationalistic"

"rationalistically"

"rationalists"

"rationalities"

"rationality"

"rationalization"

"rationalizations"

"rationalize"

"rationalized"

"rationalizer"

"rationalizers"

"rationalizes"

"rationalizing"

"rationally"

"rationalness"

"rationals"

"rationed"

"rationing"

"rations"

"ratios"

"ratline"

"ratlines"

"rats"

"ratsbane"

"ratsbanes"

"rattail"

"rattails"

"rattan"

"rattans"

"ratted"

"ratter"

"ratters"

"rattier"

"rattiest"

"ratting"

"rattish"

"rattle"

"rattlebrain"

"rattlebrained"

"rattlebrains"

"rattled"

"rattler"

"rattlers"

"rattles"

"rattlesnake"

"rattlesnakes"

"rattletrap"

"rattletraps"

"rattling"

"rattlings"

"rattly"

"rattooning"

"rattrap"

"rattraps"

"rattus"

"ratty"

"raucous"

"raucously"

"raucousness"

"raunchier"

"raunchiest"

"raunchiness"

"raunchy"

"rauwolfia"

"ravage"

"ravaged"

"ravager"

"ravagers"

"ravages"

"ravaging"

"rave"

"raved"

"ravel"

"raveled"

"raveler"

"ravelers"

"raveling"

"ravelings"

"ravelled"

"raveller"

"ravellers"

"ravelling"

"ravellings"

"ravelly"

"ravels"

"raven"

"ravened"

"ravener"

"raveners"

"ravening"

"ravenings"

"ravenous"

"ravenously"

"ravenousness"

"ravens"

"raver"

"ravers"

"raves"

"ravine"

"ravined"

"ravines"

"raving"

"ravingly"

"ravings"

"ravioli"

"raviolis"

"ravish"

"ravished"

"ravisher"

"ravishers"

"ravishes"

"ravishing"

"ravishingly"

"ravishment"

"ravishments"

"raw"

"rawboned"

"rawer"

"rawest"

"rawhide"

"rawhided"

"rawhides"

"rawhiding"

"rawish"

"rawly"

"rawness"

"rawnesses"

"raws"

"ray"

"rayed"

"raygrasses"

"raying"

"rayless"

"rayon"

"rayons"

"rays"

"raze"

"razed"

"razee"

"razer"

"razers"

"razes"

"razing"

"razor"

"razorback"

"razorbill"

"razored"

"razoring"

"razors"

"razz"

"razzed"

"razzes"

"razzing"

"razzmatazz"

"rcpt"

"rd"

"re"

"reabandon"

"reabandoned"

"reabandoning"

"reabandons"

"reabsorb"

"reabsorbed"

"reabsorbing"

"reabsorbs"

"reabsorption"

"reaccede"

"reacceded"

"reaccedes"

"reacceding"

"reaccent"

"reaccented"

"reaccenting"

"reaccents"

"reaccept"

"reaccepted"

"reaccepting"

"reaccepts"

"reaccession"

"reacclimate"

"reacclimated"

"reacclimates"

"reacclimating"

"reaccommodate"

"reaccommodated"

"reaccommodates"

"reaccommodating"

"reaccompanied"

"reaccompanies"

"reaccompany"

"reaccompanying"

"reaccredit"

"reaccredited"

"reaccrediting"

"reaccredits"

"reaccuse"

"reaccused"

"reaccuses"

"reaccusing"

"reaccustom"

"reaccustomed"

"reaccustoming"

"reaccustoms"

"reach"

"reachable"

"reached"

"reacher"

"reachers"

"reaches"

"reaching"

"reacquaint"

"reacquaintance"

"reacquainted"

"reacquainting"

"reacquaints"

"reacquire"

"reacquired"

"reacquires"

"reacquiring"

"reacquisition"

"reacquisitions"

"react"

"reactance"

"reactant"

"reactants"

"reacted"

"reacting"

"reaction"

"reactionaries"

"reactionary"

"reactions"

"reactivate"

"reactivated"

"reactivates"

"reactivating"

"reactivation"

"reactive"

"reactively"

"reactivities"

"reactivity"

"reactor"

"reactors"

"reacts"

"read"

"readability"

"readable"

"readableness"

"readably"

"readapt"

"readaptation"

"readapted"

"readapting"

"readapts"

"readd"

"readdicted"

"readdress"

"readdressed"

"readdresses"

"readdressing"

"readds"

"reader"

"readers"

"readership"

"readerships"

"readied"

"readier"

"readies"

"readiest"

"readily"

"readiness"

"reading"

"readings"

"readjourn"

"readjourned"

"readjourning"

"readjournment"

"readjournments"

"readjourns"

"readjust"

"readjustable"

"readjusted"

"readjusting"

"readjustment"

"readjustments"

"readjusts"

"readmission"

"readmissions"

"readmit"

"readmits"

"readmittance"

"readmitted"

"readmitting"

"readopt"

"readopted"

"readopting"

"readopts"

"readout"

"readouts"

"reads"

"ready"

"readying"

"reaffirm"

"reaffirmation"

"reaffirmations"

"reaffirmed"

"reaffirming"

"reaffirms"

"reagan"

"reaganomics"

"reagent"

"reagents"

"real"

"real sound"

"real sounds"

"realer"

"realest"

"realign"

"realigned"

"realigning"

"realignment"

"realignments"

"realigns"

"realise"

"realisers"

"realising"

"realism"

"realisms"

"realist"

"realistic"

"realistically"

"realists"

"realities"

"reality"

"realizability"

"realizable"

"realization"

"realizations"

"realize"

"realized"

"realizer"

"realizers"

"realizes"

"realizing"

"reallocate"

"reallocated"

"reallocates"

"reallocating"

"reallocation"

"reallocations"

"reallotment"

"reallotting"

"really"

"realm"

"realms"

"realness"

"realpolitik"

"reals"

"realties"

"realtor"

"realtors"

"realty"

"ream"

"reamed"

"reamer"

"reamers"

"reaming"

"reams"

"reanalyses"

"reanalysis"

"reanalyze"

"reanalyzed"

"reanalyzes"

"reanalyzing"

"reanimate"

"reanimated"

"reanimates"

"reanimating"

"reanimation"

"reanimations"

"reannex"

"reannexed"

"reannexes"

"reannexing"

"reap"

"reapable"

"reaped"

"reaper"

"reapers"

"reaping"

"reappear"

"reappearance"

"reappearances"

"reappeared"

"reappearing"

"reappears"

"reapplication"

"reapplied"

"reapplier"

"reapplies"

"reapply"

"reapplying"

"reappoint"

"reappointed"

"reappointing"

"reappointment"

"reappointments"

"reappoints"

"reapportion"

"reapportioned"

"reapportioning"

"reapportionment"

"reapportionments"

"reapportions"

"reappraisal"

"reappraisals"

"reappraise"

"reappraised"

"reappraisement"

"reappraiser"

"reappraises"

"reappraising"

"reappropriated"

"reappropriating"

"reappropriation"

"reaps"

"rear"

"reared"

"rearer"

"rearers"

"reargue"

"reargued"

"reargues"

"rearguing"

"rearing"

"rearm"

"rearmament"

"rearmed"

"rearming"

"rearmost"

"rearms"

"rearousal"

"rearouse"

"rearoused"

"rearouses"

"rearousing"

"rearrange"

"rearranged"

"rearrangement"

"rearrangements"

"rearranges"

"rearranging"

"rearrest"

"rearrested"

"rearresting"

"rearrests"

"rears"

"rearward"

"rearwards"

"reascend"

"reascended"

"reascending"

"reascends"

"reascent"

"reascents"

"reason"

"reasonability"

"reasonable"

"reasonableness"

"reasonably"

"reasoned"

"reasoner"

"reasoners"

"reasoning"

"reasonless"

"reasons"

"reassemble"

"reassembled"

"reassembles"

"reassemblies"

"reassembling"

"reassembly"

"reassert"

"reasserted"

"reasserting"

"reassertion"

"reasserts"

"reassess"

"reassessed"

"reassesses"

"reassessing"

"reassessment"

"reassessments"

"reassign"

"reassigned"

"reassigning"

"reassignment"

"reassignments"

"reassigns"

"reassimilate"

"reassimilated"

"reassimilates"

"reassimilating"

"reassimilation"

"reassociation"

"reassort"

"reassorted"

"reassorting"

"reassortment"

"reassortments"

"reassorts"

"reassume"

"reassumed"

"reassumes"

"reassuming"

"reassumption"

"reassumptions"

"reassurance"

"reassurances"

"reassure"

"reassured"

"reassures"

"reassuring"

"reassuringly"

"reattach"

"reattached"

"reattaches"

"reattaching"

"reattachment"

"reattachments"

"reattain"

"reattained"

"reattaining"

"reattainment"

"reattains"

"reattempt"

"reattempted"

"reattempting"

"reattempts"

"reave"

"reaved"

"reaver"

"reaves"

"reavow"

"reavowed"

"reavowing"

"reavows"

"reawake"

"reawaked"

"reawaken"

"reawakened"

"reawakening"

"reawakenings"

"reawakens"

"reawakes"

"reawaking"

"reawoke"

"reb"

"rebait"

"rebaptism"

"rebaptize"

"rebaptized"

"rebaptizes"

"rebaptizing"

"rebate"

"rebated"

"rebater"

"rebaters"

"rebates"

"rebating"

"rebbe"

"rebbes"

"rebec"

"rebeck"

"rebecks"

"rebecs"

"rebel"

"rebelled"

"rebelling"

"rebellion"

"rebellions"

"rebellious"

"rebelliously"

"rebelliousness"

"rebels"

"rebid"

"rebidding"

"rebids"

"rebill"

"rebilled"

"rebilling"

"rebills"

"rebind"

"rebinding"

"rebinds"

"rebirth"

"rebirths"

"reblooming"

"reboarding"

"reboil"

"reboiled"

"reboiling"

"reboils"

"reboot"

"rebop"

"rebops"

"reborn"

"rebound"

"rebounded"

"rebounding"

"rebounds"

"rebroadcast"

"rebroadcasted"

"rebroadcasting"

"rebroadcasts"

"rebroaden"

"rebroadened"

"rebroadening"

"rebroadens"

"rebs"

"rebuff"

"rebuffed"

"rebuffing"

"rebuffs"

"rebuild"

"rebuilding"

"rebuilds"

"rebuilt"

"rebuke"

"rebuked"

"rebuker"

"rebukers"

"rebukes"

"rebuking"

"rebukingly"

"reburial"

"reburials"

"reburied"

"reburies"

"rebury"

"reburying"

"rebus"

"rebuses"

"rebut"

"rebuts"

"rebuttable"

"rebuttably"

"rebuttal"

"rebuttals"

"rebutted"

"rebutter"

"rebutters"

"rebutting"

"rebutton"

"rebuttoned"

"rebuttoning"

"rebuttons"

"rec"

"recalcitrance"

"recalcitrances"

"recalcitrancies"

"recalcitrancy"

"recalcitrant"

"recalculate"

"recalculated"

"recalculates"

"recalculating"

"recalculation"

"recalculations"

"recalibrates"

"recall"

"recallable"

"recalled"

"recaller"

"recallers"

"recalling"

"recalls"

"recane"

"recaning"

"recant"

"recantation"

"recantations"

"recanted"

"recanter"

"recanters"

"recanting"

"recantingly"

"recants"

"recap"

"recapitalize"

"recapitalized"

"recapitalizes"

"recapitalizing"

"recapitulate"

"recapitulated"

"recapitulates"

"recapitulating"

"recapitulation"

"recapitulations"

"recapitulative"

"recappable"

"recapped"

"recapping"

"recaps"

"recapture"

"recaptured"

"recaptures"

"recapturing"

"recast"

"recasting"

"recasts"

"recd"

"recede"

"receded"

"recedes"

"receding"

"receipt"

"receipted"

"receipting"

"receiptor"

"receipts"

"receivability"

"receivable"

"receivables"

"receive"

"received"

"receiver"

"receivers"

"receivership"

"receiverships"

"receives"

"receiving"

"recelebrate"

"recelebrated"

"recelebrates"

"recelebrating"

"recencies"

"recency"

"recension"

"recent"

"recent recipes"

"recenter"

"recentest"

"recently"

"recentness"

"recept"

"receptacle"

"receptacles"

"reception"

"receptionist"

"receptionists"

"receptions"

"receptive"

"receptively"

"receptiveness"

"receptivity"

"receptor"

"receptors"

"recess"

"recessed"

"recesses"

"recessing"

"recession"

"recessional"

"recessionals"

"recessionary"

"recessions"

"recessive"

"recessively"

"recessiveness"

"recharge"

"rechargeable"

"recharged"

"recharges"

"recharging"

"rechart"

"recharted"

"recharter"

"rechartered"

"rechartering"

"recharters"

"recharting"

"recharts"

"recheck"

"rechecked"

"rechecking"

"rechecks"

"recherche"

"rechristen"

"rechristened"

"rechristening"

"rechristenings"

"rechristens"

"recidivism"

"recidivist"

"recidivistic"

"recidivists"

"recidivous"

"recipe"

"Recipe calculator"

"recipes"

"recipiangle"

"recipient"

"recipients"

"reciprocal"

"reciprocality"

"reciprocally"

"reciprocals"

"reciprocate"

"reciprocated"

"reciprocates"

"reciprocating"

"reciprocation"

"reciprocative"

"reciprocator"

"reciprocatory"

"reciprocities"

"reciprocity"

"recirculate"

"recirculated"

"recirculates"

"recirculating"

"recirculation"

"recirculations"

"recital"

"recitalist"

"recitalists"

"recitals"

"recitation"

"recitations"

"recitative"

"recitatives"

"recite"

"recited"

"reciter"

"reciters"

"recites"

"reciting"

"recked"

"reckless"

"recklessly"

"recklessness"

"reckon"

"reckoned"

"reckoner"

"reckoners"

"reckoning"

"reckonings"

"reckons"

"recks"

"reclad"

"reclaim"

"reclaimable"

"reclaimant"

"reclaimed"

"reclaiming"

"reclaims"

"reclamation"

"reclamations"

"reclassification"

"reclassifications"

"reclassified"

"reclassifies"

"reclassify"

"reclassifying"

"reclean"

"recleaned"

"recleaning"

"recleans"

"recline"

"reclined"

"recliner"

"recliners"

"reclines"

"reclining"

"reclothe"

"reclothed"

"reclothes"

"reclothing"

"recluse"

"recluses"

"reclusive"

"recognition"

"recognitions"

"recognitive"

"recognitory"

"recognizability"

"recognizable"

"recognizably"

"recognizance"

"recognize"

"recognized"

"recognizer"

"recognizes"

"recognizing"

"recoil"

"recoiled"

"recoiler"

"recoilers"

"recoiling"

"recoilless"

"recoils"

"recoin"

"recoinage"

"recoined"

"recoining"

"recoins"

"recollect"

"recollected"

"recollecting"

"recollection"

"recollections"

"recollects"

"recolonization"

"recolonize"

"recolonized"

"recolonizes"

"recolonizing"

"recolor"

"recoloration"

"recolored"

"recoloring"

"recolors"

"recomb"

"recombed"

"recombinant"

"recombination"

"recombine"

"recombined"

"recombines"

"recombing"

"recombining"

"recombs"

"recommence"

"recommenced"

"recommencement"

"recommences"

"recommencing"

"recommend"

"recommendable"

"recommendation"

"recommendations"

"recommendatory"

"recommended"

"recommender"

"recommenders"

"recommending"

"recommends"

"recommission"

"recommissioned"

"recommissioning"

"recommissions"

"recommit"

"recommits"

"recommitted"

"recommitting"

"recomparison"

"recompensable"

"recompensation"

"recompensatory"

"recompense"

"recompensed"

"recompenser"

"recompenses"

"recompensing"

"recompensive"

"recompilation"

"recompilations"

"recompiled"

"recompiling"

"recompose"

"recomposed"

"recomposes"

"recomposing"

"recomposition"

"recompound"

"recompounded"

"recompounding"

"recompounds"

"recompression"

"recompute"

"recon"

"reconcentrate"

"reconcentrated"

"reconcentrates"

"reconcentrating"

"reconcentration"

"reconcilability"

"reconcilable"

"reconcilably"

"reconcile"

"reconciled"

"reconcilement"

"reconcilements"

"reconciler"

"reconcilers"

"reconciles"

"reconciliate"

"reconciliated"

"reconciliating"

"reconciliation"

"reconciliations"

"reconciliator"

"reconciliatory"

"reconciling"

"recondensation"

"recondense"

"recondensed"

"recondenses"

"recondensing"

"recondite"

"reconditely"

"reconditeness"

"recondition"

"reconditioned"

"reconditioning"

"reconditions"

"reconfigurable"

"reconfiguration"

"reconfigure"

"reconfirm"

"reconfirmation"

"reconfirmations"

"reconfirmed"

"reconfirming"

"reconfirms"

"reconfiscated"

"reconfiscating"

"reconfiscation"

"reconnaissance"

"reconnaissances"

"reconnect"

"reconnected"

"reconnecting"

"reconnects"

"reconnoiter"

"reconnoitered"

"reconnoitering"

"reconnoiters"

"reconquer"

"reconquered"

"reconquering"

"reconquers"

"reconquest"

"recons"

"reconsecrate"

"reconsecrated"

"reconsecrates"

"reconsecrating"

"reconsecration"

"reconsecrations"

"reconsider"

"reconsideration"

"reconsidered"

"reconsidering"

"reconsiders"

"reconsign"

"reconsigned"

"reconsigning"

"reconsignment"

"reconsigns"

"reconsolidate"

"reconsolidated"

"reconsolidates"

"reconsolidating"

"reconsolidation"

"reconsolidations"

"reconstitute"

"reconstituted"

"reconstitutes"

"reconstituting"

"reconstitution"

"reconstruct"

"reconstructed"

"reconstructible"

"reconstructing"

"reconstruction"

"reconstructions"

"reconstructive"

"reconstructs"

"recontamination"

"recontest"

"recontested"

"recontesting"

"recontests"

"recontinuance"

"recontract"

"recontracted"

"recontracting"

"recontracts"

"recontrolling"

"reconvene"

"reconvened"

"reconvenes"

"reconvening"

"reconversion"

"reconversions"

"reconvert"

"reconverted"

"reconverting"

"reconverts"

"reconvey"

"reconveyance"

"reconveyed"

"reconveying"

"reconveys"

"reconviction"

"recook"

"recooked"

"recooking"

"recooks"

"recopied"

"recopies"

"recopy"

"recopying"

"record"

"recordable"

"recorded"

"recorder"

"recorders"

"recordership"

"recording"

"recordings"

"recordist"

"recordists"

"records"

"recount"

"recounted"

"recounting"

"recounts"

"recoup"

"recouped"

"recouping"

"recoups"

"recourse"

"recourses"

"recover"

"recoverability"

"recoverable"

"recovered"

"recoveree"

"recoverer"

"recoveries"

"recovering"

"recovers"

"recovery"

"recrate"

"recrated"

"recrates"

"recrating"

"recreance"

"recreancy"

"recreant"

"recreantly"

"recreants"

"recreate"

"recreated"

"recreates"

"recreating"

"recreation"

"recreational"

"recreations"

"recreative"

"recriminate"

"recriminated"

"recriminates"

"recriminating"

"recrimination"

"recriminations"

"recriminative"

"recriminator"

"recriminatory"

"recross"

"recrossed"

"recrosses"

"recrossing"

"recrown"

"recrowned"

"recrowning"

"recrowns"

"recrudesce"

"recrudesced"

"recrudescence"

"recrudescent"

"recrudesces"

"recrudescing"

"recruit"

"recruited"

"recruiter"

"recruiters"

"recruiting"

"recruitment"

"recruits"

"recrystallize"

"recrystallized"

"recrystallizes"

"recrystallizing"

"recs"

"recta"

"rectal"

"rectally"

"rectangle"

"rectangles"

"rectangular"

"rectangularity"

"rectangularly"

"recti"

"rectifiable"

"rectification"

"rectifications"

"rectified"

"rectifier"

"rectifiers"

"rectifies"

"rectify"

"rectifying"

"rectilinear"

"rectitude"

"recto"

"rector"

"rectorate"

"rectorates"

"rectorial"

"rectories"

"rectors"

"rectory"

"rectos"

"rectum"

"rectums"

"recumbencies"

"recumbent"

"recuperate"

"recuperated"

"recuperates"

"recuperating"

"recuperation"

"recuperative"

"recur"

"recurred"

"recurrence"

"recurrences"

"recurrent"

"recurrently"

"recurring"

"recurs"

"recursively"

"recurve"

"recurving"

"recusants"

"recusator"

"recuse"

"recused"

"recuses"

"recusing"

"recut"

"recutting"

"recyclability"

"recyclable"

"recycle"

"recycled"

"recycles"

"recycling"

"red"

"RED FLAGS"

"redact"

"redacted"

"redacting"

"redactional"

"redactor"

"redactors"

"redacts"

"redbird"

"redbirds"

"redbreast"

"redbreasts"

"redbud"

"redbuds"

"redbug"

"redbugs"

"redcap"

"redcaps"

"redcoat"

"redcoats"

"redded"

"redden"

"reddened"

"reddening"

"reddens"

"redder"

"reddest"

"reddish"

"reddishness"

"reddle"

"redecorate"

"redecorated"

"redecorates"

"redecorating"

"redecoration"

"reded"

"rededicate"

"rededicated"

"rededicates"

"rededicating"

"rededication"

"redeem"

"redeemability"

"redeemable"

"redeemed"

"redeemer"

"redeemers"

"redeeming"

"redeems"

"redefine"

"redefined"

"redefines"

"redefining"

"redefinition"

"redefinitions"

"redeliberation"

"redeliver"

"redelivered"

"redeliveries"

"redelivering"

"redelivers"

"redemand"

"redemanded"

"redemanding"

"redemands"

"redemonstrate"

"redemonstrated"

"redemonstrates"

"redemonstrating"

"redemonstration"

"redemptible"

"redemption"

"redemptional"

"redemptioner"

"redemptions"

"redemptive"

"redemptory"

"redeploy"

"redeployed"

"redeploying"

"redeploys"

"redeposit"

"redeposited"

"redepositing"

"redeposits"

"redes"

"redescribe"

"redescribed"

"redescribes"

"redescribing"

"redesign"

"redesignated"

"redesigned"

"redesigning"

"redesigns"

"redetermination"

"redetermine"

"redetermined"

"redetermines"

"redetermining"

"redevelop"

"redeveloped"

"redeveloper"

"redevelopers"

"redeveloping"

"redevelopment"

"redevelopments"

"redevelops"

"redeye"

"redeyes"

"redfin"

"redhead"

"redheaded"

"redheads"

"redid"

"redigest"

"redigested"

"redigesting"

"redigestion"

"redigests"

"reding"

"redip"

"redirect"

"redirected"

"redirecting"

"redirection"

"redirects"

"rediscount"

"rediscounted"

"rediscounting"

"rediscounts"

"rediscover"

"rediscovered"

"rediscoveries"

"rediscovering"

"rediscovers"

"rediscovery"

"redissolve"

"redissolved"

"redissolves"

"redissolving"

"redistill"

"redistilled"

"redistilling"

"redistills"

"redistribute"

"redistributed"

"redistributes"

"redistributing"

"redistribution"

"redistributions"

"redistrict"

"redistricted"

"redistricting"

"redistricts"

"redivide"

"redivided"

"redivides"

"redividing"

"redline"

"redlined"

"redlines"

"redlining"

"redly"

"redneck"

"rednecks"

"redness"

"rednesses"

"redo"

"redoes"

"redoing"

"redolence"

"redolency"

"redolent"

"redolently"

"redone"

"redos"

"redouble"

"redoubled"

"redoubles"

"redoubling"

"redoubt"

"redoubtable"

"redoubtably"

"redoubts"

"redound"

"redounded"

"redounding"

"redounds"

"redout"

"redox"

"redraft"

"redrafted"

"redrafting"

"redrafts"

"redraw"

"redrawing"

"redrawn"

"redraws"

"redress"

"redressed"

"redresser"

"redresses"

"redressing"

"redressment"

"redrew"

"redried"

"redries"

"redrill"

"redrilled"

"redrilling"

"redrills"

"redry"

"redrying"

"reds"

"redskin"

"redskins"

"reduce"

"reduced"

"reducer"

"reducers"

"reduces"

"reducibilities"

"reducibility"

"reducible"

"reducibly"

"reducing"

"reductio"

"reduction"

"reductional"

"reductionism"

"reductionist"

"reductions"

"reductive"

"redundance"

"redundances"

"redundancies"

"redundancy"

"redundant"

"redundantly"

"reduplicate"

"reduplicated"

"reduplicating"

"reduplication"

"reduplicative"

"reduplicatively"

"redux"

"redwing"

"redwings"

"redwood"

"redwoods"

"redye"

"redyed"

"redyeing"

"redyes"

"reecho"

"reechoed"

"reechoes"

"reechoing"

"reed"

"reeded"

"reedier"

"reediest"

"reediness"

"reeding"

"reedit"

"reedited"

"reediting"

"reedits"

"reeds"

"reeducate"

"reeducated"

"reeducates"

"reeducating"

"reeducation"

"reedy"

"reef"

"reefed"

"reefer"

"reefers"

"reefier"

"reefing"

"reefs"

"reefy"

"reek"

"reeked"

"reeker"

"reekers"

"reekier"

"reeking"

"reeks"

"reeky"

"reel"

"reelect"

"reelected"

"reelecting"

"reelection"

"reelections"

"reelects"

"reeled"

"reeler"

"reelers"

"reeling"

"reels"

"reembark"

"reembarkation"

"reembarked"

"reembarking"

"reembarks"

"reembodied"

"reembodies"

"reembody"

"reembodying"

"reemerge"

"reemerged"

"reemergence"

"reemerges"

"reemerging"

"reemphases"

"reemphasis"

"reemphasize"

"reemphasized"

"reemphasizes"

"reemphasizing"

"reemploy"

"reemployed"

"reemploying"

"reemployment"

"reemploys"

"reenact"

"reenacted"

"reenacting"

"reenactment"

"reenactments"

"reenacts"

"reenclose"

"reenclosed"

"reencloses"

"reenclosing"

"reencounter"

"reencountered"

"reencountering"

"reencounters"

"reendow"

"reendowed"

"reendowing"

"reendows"

"reenforce"

"reenforced"

"reenforces"

"reenforcing"

"reengage"

"reengaged"

"reengages"

"reengaging"

"reenjoy"

"reenjoyed"

"reenjoying"

"reenjoys"

"reenlarge"

"reenlarged"

"reenlargement"

"reenlarges"

"reenlarging"

"reenlighted"

"reenlighten"

"reenlightened"

"reenlightening"

"reenlightens"

"reenlist"

"reenlisted"

"reenlisting"

"reenlistment"

"reenlistments"

"reenlists"

"reenslave"

"reenslaved"

"reenslaves"

"reenslaving"

"reenter"

"reentered"

"reentering"

"reenters"

"reentrance"

"reentrances"

"reentrant"

"reentries"

"reentry"

"reenunciation"

"reequip"

"reequipped"

"reequipping"

"reequips"

"reerect"

"reerected"

"reerecting"

"reerects"

"reestablish"

"reestablished"

"reestablishes"

"reestablishing"

"reestablishment"

"reevaluate"

"reevaluated"

"reevaluates"

"reevaluating"

"reevaluation"

"reevaluations"

"reeve"

"reeved"

"reeves"

"reeving"

"reexamination"

"reexaminations"

"reexamine"

"reexamined"

"reexamines"

"reexamining"

"reexchange"

"reexchanged"

"reexchanges"

"reexchanging"

"reexhibit"

"reexhibited"

"reexhibiting"

"reexhibits"

"reexperience"

"reexperienced"

"reexperiences"

"reexperiencing"

"reexport"

"reexported"

"reexporting"

"reexports"

"reexpress"

"reexpressed"

"reexpresses"

"reexpressing"

"reexpression"

"ref"

"refashion"

"refashioned"

"refashioning"

"refashions"

"refasten"

"refastened"

"refastening"

"refastens"

"refection"

"refectories"

"refectory"

"refed"

"refer"

"referable"

"referee"

"refereed"

"refereeing"

"referees"

"reference"

"Reference app"

"reference material"

"reference tool"

"referenced"

"references"

"referencing"

"referenda"

"referendum"

"referendums"

"referent"

"referents"

"referral"

"referrals"

"referred"

"referrer"

"referrers"

"referring"

"refers"

"reffed"

"reffing"

"refigure"

"refigured"

"refigures"

"refiguring"

"refile"

"refiled"

"refiles"

"refiling"

"refill"

"refillable"

"refilled"

"refilling"

"refills"

"refilm"

"refilmed"

"refilming"

"refilms"

"refilter"

"refiltered"

"refiltering"

"refilters"

"refinance"

"refinanced"

"refinances"

"refinancing"

"refine"

"refined"

"refinement"

"refinements"

"refiner"

"refineries"

"refiners"

"refinery"

"refines"

"refining"

"refinish"

"refinished"

"refinishes"

"refinishing"

"refire"

"refired"

"refires"

"refiring"

"refit"

"refits"

"refitted"

"refitting"

"refix"

"reflect"

"reflected"

"reflecting"

"reflection"

"reflections"

"reflective"

"reflectively"

"reflectometer"

"reflector"

"reflectors"

"reflects"

"reflex"

"reflexed"

"reflexes"

"reflexive"

"reflexively"

"reflexiveness"

"reflexives"

"reflexologically"

"reflexologies"

"reflexologist"

"reflexology"

"reflow"

"reflowed"

"reflower"

"reflowered"

"reflowering"

"reflowers"

"reflowing"

"reflows"

"reflux"

"refly"

"refocus"

"refocused"

"refocuses"

"refocusing"

"refocussed"

"refocussing"

"refold"

"refolded"

"refolding"

"refolds"

"reforest"

"reforestation"

"reforested"

"reforesting"

"reforests"

"reforge"

"reforged"

"reforges"

"reforging"

"reform"

"reformability"

"reformable"

"reformat"

"reformated"

"reformating"

"reformation"

"reformational"

"reformations"

"reformative"

"reformatories"

"reformatory"

"reformats"

"reformatted"

"reformatting"

"reformed"

"reformer"

"reformers"

"reforming"

"reforms"

"reformulate"

"reformulated"

"reformulates"

"reformulating"

"reformulation"

"reformulations"

"refortified"

"refortifies"

"refortify"

"refortifying"

"refract"

"refracted"

"refracting"

"refraction"

"refractionist"

"refractions"

"refractive"

"refractiveness"

"refractivities"

"refractivity"

"refractometer"

"refractometry"

"refractor"

"refractorily"

"refractoriness"

"refractors"

"refractory"

"refracts"

"refracture"

"refractured"

"refractures"

"refracturing"

"refrain"

"refrained"

"refraining"

"refrainment"

"refrains"

"reframe"

"reframed"

"reframes"

"reframing"

"refrangibilities"

"refrangibility"

"refreeze"

"refreezes"

"refreezing"

"refresh"

"refreshed"

"refreshed mind"

"refresher"

"refreshers"

"refreshes"

"refreshing"

"refreshing mentally"

"refreshingly"

"refreshment"

"refreshments"

"refried"

"refries"

"refrigerant"

"refrigerants"

"refrigerate"

"refrigerated"

"refrigerates"

"refrigerating"

"refrigeration"

"refrigerator"

"refrigerators"

"refroze"

"refrozen"

"refry"

"refrying"

"refs"

"reft"

"refuel"

"refueled"

"refueling"

"refuelled"

"refuelling"

"refuels"

"refuge"

"refuged"

"refugee"

"refugees"

"refuges"

"refuging"

"refulgence"

"refulgent"

"refulgently"

"refund"

"refundable"

"refunded"

"refunder"

"refunders"

"refunding"

"refunds"

"refurbish"

"refurbished"

"refurbishes"

"refurbishing"

"refurbishment"

"refurnish"

"refurnished"

"refurnishes"

"refurnishing"

"refusal"

"refusals"

"refuse"

"refused"

"refuser"

"refusers"

"refuses"

"refusing"

"refutability"

"refutable"

"refutably"

"refutals"

"refutation"

"refutations"

"refutatory"

"refute"

"refuted"

"refuter"

"refuters"

"refutes"

"refuting"

"reg"

"regain"

"regained"

"regainer"

"regainers"

"regaining"

"regains"

"regal"

"regale"

"regaled"

"regalement"

"regales"

"regalia"

"regaling"

"regalities"

"regality"

"regally"

"regard"

"regarded"

"regardful"

"regarding"

"regardless"

"regards"

"regather"

"regathered"

"regathering"

"regathers"

"regatta"

"regattas"

"regauge"

"regauged"

"regauges"

"regauging"

"regear"

"regeared"

"regearing"

"regears"

"regencies"

"regency"

"regeneracy"

"regenerate"

"regenerated"

"regenerates"

"regenerating"

"regeneration"

"regenerative"

"regenerator"

"regenerators"

"regent"

"regents"

"regerminate"

"regerminated"

"regerminates"

"regerminating"

"regermination"

"regerminative"

"regerminatively"

"reges"

"reggae"

"regia"

"regicidal"

"regicide"

"regicides"

"regild"

"regilded"

"regilding"

"regilds"

"regilt"

"regime"

"regimen"

"regimens"

"regiment"

"regimental"

"regimentally"

"regimentals"

"regimentation"

"regimented"

"regimenting"

"regiments"

"regimes"

"regina"

"reginal"

"reginas"

"region"

"regional"

"regionalism"

"regionalist"

"regionalistic"

"regionally"

"regionals"

"regions"

"register"

"registerable"

"registered"

"Registered Medical Transcriptionist"

"registerer"

"registering"

"registers"

"registership"

"registrability"

"registrable"

"registrant"

"registrants"

"registrar"

"registrars"

"registrarship"

"registration"

"registrational"

"registrations"

"registries"

"registry"

"reglaze"

"reglazed"

"reglazes"

"reglazing"

"regloss"

"reglossed"

"reglosses"

"reglossing"

"reglue"

"reglued"

"reglues"

"regluing"

"regnal"

"regnancy"

"regnant"

"regnum"

"regrade"

"regraded"

"regrades"

"regrading"

"regrafting"

"regranting"

"regress"

"regressed"

"regresses"

"regressing"

"regression"

"regressions"

"regressive"

"regressively"

"regressiveness"

"regressor"

"regressors"

"regret"

"regretful"

"regretfully"

"regretfulness"

"regrets"

"regrettable"

"regrettably"

"regretted"

"regretter"

"regretters"

"regretting"

"regrew"

"regrooved"

"regrooves"

"regroup"

"regrouped"

"regrouping"

"regroups"

"regrow"

"regrowing"

"regrown"

"regrows"

"regrowth"

"regulable"

"regular"

"regularities"

"regularity"

"regularization"

"regularize"

"regularized"

"regularizer"

"regularizes"

"regularizing"

"regularly"

"regulars"

"regulatable"

"regulate"

"regulated"

"regulates"

"regulating"

"regulation"

"regulations"

"regulative"

"regulatively"

"regulator"

"regulators"

"regulatory"

"regulus"

"regurgitant"

"regurgitate"

"regurgitated"

"regurgitates"

"regurgitating"

"regurgitation"

"regurgitations"

"regurgitative"

"rehabilitant"

"rehabilitate"

"rehabilitated"

"rehabilitates"

"rehabilitating"

"rehabilitation"

"rehabilitations"

"rehabilitative"

"rehabilitator"

"rehabilitee"

"rehammered"

"rehandle"

"rehandled"

"rehandles"

"rehandling"

"rehang"

"rehanged"

"rehanging"

"rehangs"

"reharden"

"rehardened"

"rehardening"

"rehardens"

"reharmonization"

"rehash"

"rehashed"

"rehashes"

"rehashing"

"rehear"

"reheard"

"rehearing"

"rehearings"

"rehears"

"rehearsal"

"rehearsals"

"rehearse"

"rehearsed"

"rehearser"

"rehearsers"

"rehearses"

"rehearsing"

"reheat"

"reheated"

"reheater"

"reheaters"

"reheating"

"reheats"

"reheel"

"reheeled"

"reheeling"

"reheels"

"rehem"

"rehemmed"

"rehemming"

"rehems"

"rehinge"

"rehinged"

"rehinges"

"rehinging"

"rehire"

"rehired"

"rehires"

"rehiring"

"rehung"

"rehydrate"

"rehydrating"

"rehydration"

"reich"

"reified"

"reifier"

"reifiers"

"reifies"

"reify"

"reifying"

"reign"

"reigned"

"reigning"

"reignite"

"reignited"

"reignites"

"reigniting"

"reigns"

"Reiki"

"reimbursable"

"reimburse"

"reimburseable"

"reimbursed"

"reimbursement"

"reimbursements"

"reimburses"

"reimbursing"

"reimported"

"reimpose"

"reimposed"

"reimposes"

"reimposing"

"reimprison"

"reimprisoned"

"reimprisoning"

"reimprisons"

"rein"

"reincarnate"

"reincarnated"

"reincarnates"

"reincarnating"

"reincarnation"

"reincarnationist"

"reincarnationists"

"reincarnations"

"reinciting"

"reincorporate"

"reincorporated"

"reincorporates"

"reincorporating"

"reincur"

"reincurred"

"reincurring"

"reincurs"

"reindeer"

"reindeers"

"reindexed"

"reindexes"

"reinduce"

"reinduced"

"reinduces"

"reinducing"

"reinduct"

"reinducted"

"reinducting"

"reinduction"

"reinducts"

"reined"

"reinfect"

"reinfected"

"reinfecting"

"reinfection"

"reinfections"

"reinfects"

"reinflame"

"reinflamed"

"reinflames"

"reinflaming"

"reinforce"

"reinforced"

"reinforcement"

"reinforcements"

"reinforcer"

"reinforcers"

"reinforces"

"reinforcing"

"reinform"

"reinformed"

"reinforming"

"reinforms"

"reinfuse"

"reinfused"

"reinfuses"

"reinfusing"

"reinfusion"

"reining"

"reinjured"

"reinjures"

"reinjuring"

"reinoculate"

"reinoculated"

"reinoculates"

"reinoculating"

"reinoculation"

"reinoculations"

"reins"

"reinscribe"

"reinscribed"

"reinscribes"

"reinscribing"

"reinsert"

"reinserted"

"reinserting"

"reinsertion"

"reinserts"

"reinsman"

"reinsmen"

"reinspect"

"reinspected"

"reinspecting"

"reinspection"

"reinspects"

"reinstall"

"reinstallation"

"reinstallations"

"reinstalled"

"reinstalling"

"reinstallment"

"reinstallments"

"reinstalls"

"reinstate"

"reinstated"

"reinstatement"

"reinstatements"

"reinstates"

"reinstating"

"reinstitution"

"reinstruct"

"reinstructed"

"reinstructing"

"reinstructs"

"reinsure"

"reinsured"

"reinsures"

"reinsuring"

"reintegrate"

"reintegrated"

"reintegrates"

"reintegrating"

"reintegration"

"reinter"

"reinterpret"

"reinterpretation"

"reinterpretations"

"reinterpreted"

"reinterpreting"

"reinterprets"

"reinterred"

"reinterring"

"reinterrogate"

"reinterrogated"

"reinterrogates"

"reinterrogating"

"reinterrogation"

"reinterrogations"

"reinters"

"reintrench"

"reintrenched"

"reintrenches"

"reintrenching"

"reintrenchment"

"reintroduce"

"reintroduced"

"reintroduces"

"reintroducing"

"reintroduction"

"reinvent"

"reinvented"

"reinventing"

"reinvents"

"reinvest"

"reinvested"

"reinvestigate"

"reinvestigated"

"reinvestigates"

"reinvestigating"

"reinvestigation"

"reinvestigations"

"reinvesting"

"reinvestment"

"reinvests"

"reinvigorate"

"reinvigorated"

"reinvigorates"

"reinvigorating"

"reinvigoration"

"reinvitation"

"reinvite"

"reinvited"

"reinvites"

"reinviting"

"reinvoke"

"reinvoked"

"reinvokes"

"reinvoking"

"reinvolve"

"reinvolved"

"reinvolvement"

"reinvolves"

"reinvolving"

"reissue"

"reissued"

"reissuer"

"reissuers"

"reissues"

"reissuing"

"reiterate"

"reiterated"

"reiterates"

"reiterating"

"reiteration"

"reiterations"

"reiterative"

"reivers"

"reiving"

"reject"

"rejectable"

"rejected"

"rejectee"

"rejectees"

"rejecter"

"rejecters"

"rejecting"

"rejection"

"rejections"

"rejector"

"rejectors"

"rejects"

"rejoice"

"rejoiced"

"rejoicer"

"rejoicers"

"rejoices"

"rejoicing"

"rejoin"

"rejoinder"

"rejoinders"

"rejoined"

"rejoining"

"rejoins"

"rejudge"

"rejudged"

"rejudges"

"rejudging"

"rejuvenate"

"rejuvenated"

"rejuvenates"

"rejuvenating"

"rejuvenation"

"rejuvenations"

"rejuvenescence"

"rejuvenescent"

"rekey"

"rekeyed"

"rekeying"

"rekeys"

"rekindle"

"rekindled"

"rekindles"

"rekindling"

"relabel"

"relabeled"

"relabeling"

"relabelled"

"relabelling"

"relabels"

"relapse"

"relapsed"

"relapser"

"relapsers"

"relapses"

"relapsing"

"relatable"

"relate"

"related"

"relatedness"

"relater"

"relaters"

"relates"

"relating"

"relation"

"relational"

"relatione"

"relations"

"relationship"

"relationships"

"relative"

"relatively"

"relativeness"

"relatives"

"relativistic"

"relativistically"

"relativity"

"relator"

"relaunder"

"relaundered"

"relaundering"

"relaunders"

"relax"

"Relax state"

"Relax state of mind"

"relaxant"

"relaxants"

"relaxation"

"relaxations"

"relaxed"

"relaxer"

"relaxers"

"relaxes"

"relaxing"

"relaxing sound"

"relaxing state"

"relaxing way"

"relay"

"relayed"

"relaying"

"relays"

"relearn"

"relearned"

"relearning"

"relearns"

"relearnt"

"releasability"

"releasable"

"release"

"released"

"releaser"

"releasers"

"releases"

"releasibility"

"releasible"

"releasing"

"relegable"

"relegate"

"relegated"

"relegates"

"relegating"

"relegation"

"relent"

"relented"

"relenting"

"relentless"

"relentlessly"

"relentlessness"

"relents"

"relet"

"relets"

"reletter"

"relettered"

"relettering"

"reletters"

"reletting"

"relevance"

"relevances"

"relevancies"

"relevancy"

"relevant"

"relevantly"

"reliability"

"reliable"

"reliableness"

"reliably"

"reliance"

"reliances"

"reliant"

"reliantly"

"relic"

"relicense"

"relicensed"

"relicenses"

"relicensing"

"relics"

"relict"

"relicts"

"relied"

"relief"

"reliefs"

"reliers"

"relies"

"relieve"

"relieved"

"reliever"

"relievers"

"relieves"

"relieving"

"relight"

"relighted"

"relighting"

"relights"

"religion"

"religionist"

"religionists"

"religions"

"religiosity"

"religious"

"religiously"

"religiousness"

"reline"

"relined"

"relines"

"relining"

"relinked"

"relinquish"

"relinquished"

"relinquisher"

"relinquishers"

"relinquishes"

"relinquishing"

"relinquishment"

"relinquishments"

"reliquaries"

"reliquary"

"relique"

"reliques"

"reliquidate"

"reliquidated"

"reliquidates"

"reliquidating"

"reliquidation"

"relish"

"relishable"

"relished"

"relishes"

"relishing"

"relist"

"relisted"

"relisting"

"relists"

"relit"

"relive"

"relived"

"relives"

"reliving"

"reload"

"reloaded"

"reloader"

"reloaders"

"reloading"

"reloads"

"reloan"

"reloaned"

"reloaning"

"reloans"

"relocate"

"relocated"

"relocates"

"relocating"

"relocation"

"relocations"

"reluctance"

"reluctancy"

"reluctant"

"reluctantly"

"rely"

"relying"

"rem"

"remade"

"remail"

"remailed"

"remailing"

"remails"

"remain"

"remainder"

"remaindered"

"remaindering"

"remainders"

"remained"

"remaining"

"remains"

"remake"

"remakes"

"remaking"

"reman"

"remand"

"remanded"

"remanding"

"remandment"

"remands"

"remanufacture"

"remanufactured"

"remanufactures"

"remanufacturing"

"remap"

"remark"

"remarkable"

"remarkableness"

"remarkably"

"remarked"

"remarker"

"remarkers"

"remarking"

"remarks"

"remarque"

"remarques"

"remarriage"

"remarriages"

"remarried"

"remarries"

"remarry"

"remarrying"

"rematch"

"rematched"

"rematches"

"rematching"

"rembrandt"

"remeasure"

"remeasured"

"remeasurement"

"remeasurements"

"remeasures"

"remeasuring"

"remediable"

"remedial"

"remedially"

"remedied"

"remedies"

"remediless"

"remedy"

"remedying"

"remeets"

"remelt"

"remelted"

"remelting"

"remelts"

"remember"

"rememberable"

"remembered"

"rememberer"

"rememberers"

"remembering"

"remembers"

"remembrance"

"remembrances"

"remend"

"remended"

"remending"

"remends"

"remet"

"remigrate"

"remigrated"

"remigrates"

"remigrating"

"remigration"

"remigrations"

"remilitarization"

"remilitarize"

"remilitarized"

"remilitarizes"

"remilitarizing"

"remind"

"reminded"

"reminder"

"reminders"

"reminding"

"reminds"

"reminisce"

"reminisced"

"reminiscence"

"reminiscences"

"reminiscent"

"reminiscently"

"reminisces"

"reminiscing"

"remiss"

"remission"

"remissions"

"remissly"

"remissness"

"remit"

"remits"

"remittable"

"remittal"

"remittals"

"remittance"

"remittances"

"remitted"

"remittee"

"remittent"

"remittently"

"remitter"

"remitters"

"remitting"

"remittor"

"remittors"

"remix"

"remixed"

"remixes"

"remixing"

"remnant"

"remnants"

"remodel"

"remodeled"

"remodeler"

"remodelers"

"remodeling"

"remodelled"

"remodelling"

"remodels"

"remodification"

"remodified"

"remodifies"

"remodify"

"remodifying"

"remolades"

"remold"

"remolded"

"remolding"

"remolds"

"remonetization"

"remonetize"

"remonetized"

"remonetizes"

"remonetizing"

"remonstrance"

"remonstrances"

"remonstrant"

"remonstrantly"

"remonstrate"

"remonstrated"

"remonstrates"

"remonstrating"

"remonstration"

"remonstrations"

"remonstrative"

"remonstrator"

"remonstrators"

"remora"

"remoras"

"remorse"

"remorseful"

"remorsefully"

"remorsefulness"

"remorseless"

"remorselessly"

"remorselessness"

"remorses"

"remortgage"

"remortgaged"

"remortgages"

"remortgaging"

"remote"

"remotely"

"remoteness"

"remoter"

"remotest"

"remount"

"remounted"

"remounting"

"remounts"

"removable"

"removal"

"removals"

"remove"

"remove ads"

"removed"

"remover"

"removers"

"removes"

"removing"

"rems"

"remunerate"

"remunerated"

"remunerates"

"remunerating"

"remuneration"

"remunerations"

"remunerative"

"remuneratively"

"remunerativeness"

"remunerator"

"remunerators"

"remuneratory"

"renaissance"

"renal"

"rename"

"renamed"

"renames"

"renaming"

"renascence"

"renascences"

"renascent"

"rench"

"rencounter"

"rencounters"

"rend"

"rended"

"render"

"rendered"

"renderer"

"renderers"

"rendering"

"renderings"

"renders"

"rendezvous"

"rendezvoused"

"rendezvouses"

"rendezvousing"

"rending"

"rendition"

"renditions"

"rends"

"renegade"

"renegades"

"renegading"

"renege"

"reneged"

"reneger"

"renegers"

"reneges"

"reneging"

"renegotiable"

"renegotiate"

"renegotiated"

"renegotiates"

"renegotiating"

"renegotiation"

"renegotiations"

"renegotiator"

"renew"

"renewability"

"renewable"

"renewal"

"renewals"

"renewed"

"renewer"

"renewers"

"renewing"

"renews"

"renig"

"renigs"

"rennet"

"rennin"

"reno"

"renograms"

"renoir"

"renominate"

"renominated"

"renominates"

"renominating"

"renomination"

"renominations"

"renotification"

"renotified"

"renotifies"

"renotify"

"renotifying"

"renounce"

"renounceable"

"renounced"

"renouncement"

"renouncements"

"renouncer"

"renouncers"

"renounces"

"renouncing"

"renovate"

"renovated"

"renovates"

"renovating"

"renovation"

"renovations"

"renovator"

"renovators"

"renown"

"renowned"

"renowns"

"rent"

"rentability"

"rentable"

"rentage"

"rental"

"rentals"

"rented"

"renter"

"renters"

"renting"

"rents"

"renumber"

"renumbered"

"renumbering"

"renumbers"

"renunciation"

"renunciations"

"renunciatory"

"reobtain"

"reobtainable"

"reobtained"

"reobtaining"

"reobtains"

"reoccupation"

"reoccupations"

"reoccupied"

"reoccupies"

"reoccupy"

"reoccupying"

"reoccur"

"reoccurred"

"reoccurrence"

"reoccurrences"

"reoccurring"

"reoccurs"

"reoil"

"reopen"

"reopened"

"reopener"

"reopening"

"reopenings"

"reopens"

"reordain"

"reorder"

"reordered"

"reordering"

"reorders"

"reorganization"

"reorganizations"

"reorganize"

"reorganized"

"reorganizer"

"reorganizers"

"reorganizes"

"reorganizing"

"reorient"

"reorientation"

"reorientations"

"reoriented"

"reorienting"

"reorients"

"rep"

"repacified"

"repacifies"

"repacify"

"repacifying"

"repack"

"repackage"

"repackaged"

"repackages"

"repackaging"

"repacked"

"repacking"

"repacks"

"repaginate"

"repaginated"

"repaginates"

"repaginating"

"repagination"

"repaid"

"repaint"

"repainted"

"repainting"

"repaints"

"repair"

"repairable"

"repaired"

"repairer"

"repairers"

"repairing"

"repairman"

"repairmen"

"repairs"

"repapered"

"repapering"

"reparable"

"reparation"

"reparations"

"reparative"

"reparatory"

"repartee"

"repartees"

"repartition"

"repass"

"repassed"

"repasses"

"repassing"

"repast"

"repasted"

"repasting"

"repasts"

"repatriate"

"repatriated"

"repatriates"

"repatriating"

"repatriation"

"repatriations"

"repave"

"repaved"

"repaves"

"repaving"

"repay"

"repayable"

"repaying"

"repayment"

"repayments"

"repays"

"repeal"

"repealable"

"repealed"

"repealer"

"repealers"

"repealing"

"repeals"

"repeat"

"repeatability"

"repeatable"

"repeated"

"repeatedly"

"repeater"

"repeaters"

"repeating"

"repeats"

"repel"

"repellant"

"repelled"

"repellency"

"repellent"

"repellently"

"repellents"

"repeller"

"repellers"

"repelling"

"repels"

"repent"

"repentance"

"repentant"

"repentantly"

"repented"

"repenter"

"repenters"

"repenting"

"repents"

"repeople"

"repeopled"

"repeoples"

"repeopling"

"repercussion"

"repercussions"

"repercussive"

"repercussiveness"

"repertoire"

"repertoires"

"repertorial"

"repertories"

"repertory"

"repetition"

"repetitions"

"repetitious"

"repetitiously"

"repetitiousness"

"repetitive"

"repetitively"

"repetitiveness"

"rephrase"

"rephrased"

"rephrases"

"rephrasing"

"repin"

"repine"

"repined"

"repiner"

"repiners"

"repines"

"repining"

"repinned"

"repinning"

"repins"

"replace"

"replaceable"

"replaced"

"replacement"

"replacements"

"replacer"

"replacers"

"replaces"

"replacing"

"replan"

"replanned"

"replanning"

"replans"

"replant"

"replanted"

"replanting"

"replants"

"replated"

"replates"

"replay"

"replayed"

"replaying"

"replays"

"replenish"

"replenished"

"replenisher"

"replenishers"

"replenishes"

"replenishing"

"replenishment"

"replete"

"repleteness"

"repletion"

"replica"

"replicas"

"replicate"

"replicated"

"replicates"

"replicating"

"replication"

"replications"

"replicative"

"replied"

"replier"

"repliers"

"replies"

"reply"

"replying"

"repopulate"

"repopulated"

"repopulates"

"repopulating"

"repopulation"

"report"

"reportable"

"reportage"

"reportages"

"reported"

"reportedly"

"reporter"

"reporters"

"reporting"

"reportorial"

"reports"

"repose"

"reposed"

"reposeful"

"reposer"

"reposers"

"reposes"

"reposing"

"reposition"

"repositioned"

"repositioning"

"repositories"

"repository"

"repossess"

"repossessed"

"repossesses"

"repossessing"

"repossession"

"repossessions"

"repossessor"

"repousses"

"repowering"

"repped"

"reprehend"

"reprehended"

"reprehending"

"reprehends"

"reprehensible"

"reprehensibly"

"reprehension"

"represent"

"representable"

"representation"

"representational"

"representations"

"representative"

"representatively"

"representatives"

"represented"

"representee"

"representing"

"representor"

"represents"

"repress"

"repressed"

"represses"

"repressibilities"

"repressibility"

"repressible"

"repressing"

"repression"

"repressions"

"repressive"

"repressively"

"repressiveness"

"repressor"

"reprice"

"repriced"

"reprices"

"repricing"

"reprieval"

"reprieve"

"reprieved"

"repriever"

"reprievers"

"reprieves"

"reprieving"

"reprimand"

"reprimanded"

"reprimanding"

"reprimands"

"reprint"

"reprinted"

"reprinter"

"reprinting"

"reprintings"

"reprints"

"reprisal"

"reprisals"

"reprise"

"reprised"

"reprises"

"reprising"

"repro"

"reproach"

"reproachable"

"reproached"

"reproacher"

"reproaches"

"reproachful"

"reproachfully"

"reproachfulness"

"reproaching"

"reproachingly"

"reprobate"

"reprobated"

"reprobates"

"reprobating"

"reprobation"

"reprobative"

"reprobe"

"reprobed"

"reprobes"

"reprobing"

"reprocess"

"reprocessed"

"reprocesses"

"reprocessing"

"reproduce"

"reproduced"

"reproducer"

"reproducers"

"reproduces"

"reproducible"

"reproducing"

"reproduction"

"reproductions"

"reproductive"

"reproductive health"

"Reproductive System"

"reproductively"

"reproductiveness"

"reproductivity"

"reprogram"

"reprogrammed"

"reprogramming"

"reprography"

"reproof"

"reproofs"

"reproval"

"reprove"

"reproved"

"reprover"

"reprovers"

"reproves"

"reproving"

"reprovingly"

"reps"

"reptile"

"reptiles"

"reptilian"

"reptilians"

"republic"

"republica"

"republican"

"republicanism"

"republicans"

"republication"

"republics"

"republish"

"republished"

"republishes"

"republishing"

"repudiate"

"repudiated"

"repudiates"

"repudiating"

"repudiation"

"repudiations"

"repudiator"

"repudiators"

"repugnance"

"repugnancy"

"repugnant"

"repugnantly"

"repugned"

"repulse"

"repulsed"

"repulser"

"repulsers"

"repulses"

"repulsing"

"repulsion"

"repulsions"

"repulsive"

"repulsively"

"repulsiveness"

"repurchase"

"repurchased"

"repurchases"

"repurchasing"

"reputability"

"reputable"

"reputably"

"reputation"

"reputations"

"repute"

"reputed"

"reputedly"

"reputes"

"reputing"

"req"

"request"

"requested"

"requester"

"requesters"

"requesting"

"requestor"

"requestors"

"requests"

"requiem"

"requiems"

"requiescat"

"require"

"required"

"requirement"

"requirements"

"requirer"

"requirers"

"requires"

"requiring"

"requisite"

"requisitely"

"requisiteness"

"requisites"

"requisition"

"requisitioned"

"requisitioner"

"requisitioners"

"requisitioning"

"requisitions"

"requital"

"requitals"

"requite"

"requited"

"requiter"

"requiters"

"requites"

"requiting"

"reradiate"

"reradiated"

"reradiates"

"reradiating"

"reran"

"reread"

"rereading"

"rereads"

"rerecord"

"rerecorded"

"rerecording"

"rerecords"

"reredos"

"reredoses"

"reroll"

"rerolled"

"rerolling"

"rerolls"

"reroute"

"rerouted"

"reroutes"

"rerouting"

"rerun"

"rerunning"

"reruns"

"resalable"

"resale"

"resales"

"resaw"

"resay"

"reschedule"

"rescheduled"

"reschedules"

"rescheduling"

"rescind"

"rescindable"

"rescinded"

"rescinder"

"rescinding"

"rescindment"

"rescinds"

"rescission"

"rescissions"

"rescript"

"rescripts"

"rescue"

"rescued"

"rescuer"

"rescuers"

"rescues"

"rescuing"

"reseal"

"resealable"

"resealed"

"resealing"

"reseals"

"research"

"researched"

"researcher"

"researchers"

"researches"

"researching"

"reseat"

"reseated"

"reseating"

"reseats"

"resectabilities"

"resection"

"resections"

"resee"

"reseed"

"reseeded"

"reseeding"

"reseeds"

"resell"

"reseller"

"resellers"

"reselling"

"resells"

"resemblance"

"resemblances"

"resemble"

"resembled"

"resembles"

"resembling"

"resent"

"resented"

"resentful"

"resentfully"

"resentfulness"

"resenting"

"resentment"

"resentments"

"resents"

"reserpine"

"reservation"

"reservations"

"reserve"

"reserved"

"reservedly"

"reservedness"

"reserver"

"reservers"

"reserves"

"reserving"

"reservist"

"reservists"

"reservoir"

"reservoirs"

"reset"

"resets"

"resetter"

"resetters"

"resetting"

"resettings"

"resettle"

"resettled"

"resettlement"

"resettlements"

"resettles"

"resettling"

"resew"

"resewing"

"reshape"

"reshaped"

"reshaper"

"reshapers"

"reshapes"

"reshaping"

"resharpen"

"resharpened"

"resharpening"

"resharpens"

"reship"

"reshipment"

"reshipments"

"reshipped"

"reshipper"

"reshipping"

"reships"

"reshooting"

"reshowed"

"reshowing"

"reshuffle"

"reshuffled"

"reshuffles"

"reshuffling"

"reside"

"resided"

"residence"

"residences"

"residencies"

"residency"

"resident"

"residential"

"residentially"

"residents"

"resider"

"residers"

"resides"

"residing"

"residua"

"residual"

"residually"

"residuals"

"residuary"

"residue"

"residues"

"residuum"

"residuums"

"resifted"

"resifting"

"resign"

"resignation"

"resignations"

"resigned"

"resignedly"

"resignee"

"resigner"

"resigners"

"resigning"

"resigns"

"resilience"

"resiliency"

"resilient"

"resiliently"

"resin"

"resinoids"

"resinous"

"resins"

"resist"

"resistably"

"resistance"

"resistances"

"resistant"

"resistantly"

"resisted"

"resistent"

"resister"

"resisters"

"resistibility"

"resistible"

"resisting"

"resistive"

"resistivity"

"resistless"

"resistor"

"resistors"

"resists"

"resituate"

"resituated"

"resituates"

"resituating"

"resizing"

"resold"

"resolder"

"resole"

"resoled"

"resoles"

"resoling"

"resolute"

"resolutely"

"resoluteness"

"resolutes"

"resolution"

"resolutions"

"resolutive"

"resolutory"

"resolvable"

"resolve"

"resolved"

"resolver"

"resolvers"

"resolves"

"resolving"

"resonance"

"resonances"

"resonant"

"resonantly"

"resonants"

"resonate"

"resonated"

"resonates"

"resonating"

"resonation"

"resonations"

"resonator"

"resonators"

"resorbed"

"resort"

"resorted"

"resorter"

"resorters"

"resorting"

"resorts"

"resound"

"resounded"

"resounding"

"resoundingly"

"resounds"

"resource"

"resourceful"

"resourcefully"

"resourcefulness"

"resources"

"resow"

"resowed"

"resowing"

"resown"

"resows"

"resp"

"respect"

"respectability"

"respectable"

"respectably"

"respected"

"respecter"

"respecters"

"respectful"

"respectfully"

"respectfulness"

"respecting"

"respective"

"respectively"

"respects"

"respell"

"respelled"

"respelling"

"respells"

"respirability"

"respirable"

"respirating"

"respiration"

"respirational"

"respirations"

"respirator"

"respirators"

"respiratory"

"Respiratory System"

"respire"

"respired"

"respires"

"respiring"

"respirometer"

"respite"

"respited"

"respites"

"respiting"

"resplendence"

"resplendent"

"resplendently"

"respond"

"responded"

"respondences"

"respondencies"

"respondent"

"respondents"

"responder"

"responders"

"responding"

"responds"

"response"

"responses"

"responsibilities"

"responsibility"

"responsible"

"responsibleness"

"responsibly"

"responsive"

"responsively"

"responsiveness"

"rest"

"restack"

"restacked"

"restacking"

"restacks"

"restaff"

"restaffed"

"restaffing"

"restaffs"

"restage"

"restaged"

"restages"

"restaging"

"restamp"

"restamped"

"restamping"

"restamps"

"restart"

"restartable"

"restarted"

"restarting"

"restarts"

"restate"

"restated"

"restatement"

"restatements"

"restates"

"restating"

"restaurant"

"restaurant chains"

"restaurants"

"restaurateur"

"restaurateurs"

"rested"

"rester"

"resters"

"restful"

"restful sleep"

"restfully"

"restfulness"

"resting"

"restituted"

"restitution"

"restitutions"

"restitutive"

"restitutory"

"restive"

"restively"

"restiveness"

"restless"

"restlessly"

"restlessness"

"restock"

"restocked"

"restocking"

"restocks"

"restorability"

"restorable"

"restorals"

"restoration"

"restorations"

"restorative"

"restoratively"

"restorativeness"

"restoratives"

"restore"

"restored"

"restorer"

"restorers"

"restores"

"restoring"

"restraighten"

"restraightened"

"restraightening"

"restraightens"

"restrain"

"restrainable"

"restrained"

"restrainedly"

"restrainer"

"restrainers"

"restraining"

"restrains"

"restraint"

"restraints"

"restrengthen"

"restrengthened"

"restrengthening"

"restrengthens"

"restrict"

"restricted"

"restricting"

"restriction"

"restrictionism"

"restrictionist"

"restrictions"

"restrictive"

"restrictively"

"restrictiveness"

"restricts"

"restring"

"restringing"

"restrings"

"restructure"

"restructured"

"restructures"

"restructuring"

"restrung"

"rests"

"restudied"

"restudies"

"restudy"

"restudying"

"restuff"

"restuffed"

"restuffing"

"restuffs"

"restyle"

"restyled"

"restyles"

"restyling"

"resubmission"

"resubmissions"

"resubmit"

"resubmits"

"resubmitted"

"resubmitting"

"resubscribe"

"resubscribed"

"resubscribes"

"resubscribing"

"resubscription"

"result"

"resultant"

"resultants"

"resulted"

"resulting"

"results"

"resume"

"resumed"

"resumer"

"resumers"

"resumes"

"resuming"

"resummon"

"resummoned"

"resummoning"

"resummons"

"resumption"

"resumptions"

"resupplied"

"resupplies"

"resupply"

"resupplying"

"resurface"

"resurfaced"

"resurfaces"

"resurfacing"

"resurged"

"resurgence"

"resurgences"

"resurgent"

"resurges"

"resurging"

"resurrect"

"resurrected"

"resurrecting"

"resurrection"

"resurrectionism"

"resurrectionist"

"resurrections"

"resurrects"

"resurvey"

"resurveyed"

"resurveying"

"resurveys"

"resuscitate"

"resuscitated"

"resuscitates"

"resuscitating"

"resuscitation"

"resuscitative"

"resuscitator"

"resuscitators"

"ret"

"retail"

"retailed"

"retailer"

"retailers"

"retailing"

"retailor"

"retailors"

"retails"

"retain"

"retainable"

"retained"

"retainer"

"retainers"

"retaining"

"retainment"

"retains"

"retake"

"retaken"

"retaker"

"retakers"

"retakes"

"retaking"

"retaliate"

"retaliated"

"retaliates"

"retaliating"

"retaliation"

"retaliations"

"retaliator"

"retaliators"

"retaliatory"

"retard"

"retardant"

"retardants"

"retardate"

"retardates"

"retardation"

"retarded"

"retarder"

"retarders"

"retarding"

"retards"

"retaught"

"retch"

"retched"

"retches"

"retching"

"retd"

"reteach"

"reteaches"

"reteaching"

"retell"

"retelling"

"retells"

"retention"

"retentive"

"retentiveness"

"retest"

"retested"

"retesting"

"retests"

"rethink"

"rethinking"

"rethinks"

"rethought"

"rethread"

"rethreaded"

"rethreading"

"rethreads"

"reticence"

"reticent"

"reticently"

"reticula"

"reticular"

"reticulated"

"reticulation"

"reticule"

"reticules"

"reticulum"

"retie"

"retied"

"reties"

"retina"

"retinal"

"retinal damage"

"retinals"

"retinas"

"retinoscope"

"retinoscopies"

"retinoscopy"

"retinted"

"retinue"

"retinued"

"retinues"

"retire"

"retired"

"retiree"

"retirees"

"retirement"

"retirements"

"retirer"

"retirers"

"retires"

"retiring"

"retiringly"

"retitle"

"retitled"

"retitles"

"retitling"

"retold"

"retook"

"retool"

"retooled"

"retooling"

"retort"

"retorted"

"retorter"

"retorters"

"retorting"

"retorts"

"retouch"

"retouchable"

"retouched"

"retoucher"

"retouchers"

"retouches"

"retouching"

"retrace"

"retraceable"

"retraced"

"retraces"

"retracing"

"retract"

"retractable"

"retracted"

"retractile"

"retracting"

"retraction"

"retractions"

"retractor"

"retractors"

"retracts"

"retrain"

"retrained"

"retraining"

"retrains"

"retransfer"

"retransferred"

"retransferring"

"retransfers"

"retranslate"

"retranslated"

"retranslates"

"retranslating"

"retranslation"

"retranslations"

"retransmissions"

"retransmit"

"retransmits"

"retransmitted"

"retransmitting"

"retread"

"retreaded"

"retreading"

"retreads"

"retreat"

"retreated"

"retreating"

"retreats"

"retrench"

"retrenched"

"retrenches"

"retrenching"

"retrenchment"

"retrenchments"

"retrial"

"retrials"

"retribute"

"retributed"

"retributing"

"retribution"

"retributive"

"retributor"

"retributory"

"retried"

"retries"

"retrievable"

"retrieval"

"retrievals"

"retrieve"

"retrieved"

"retriever"

"retrievers"

"retrieves"

"retrieving"

"retrimmed"

"retro"

"retroact"

"retroacted"

"retroaction"

"retroactive"

"retroactively"

"retroactivity"

"retroacts"

"retrocede"

"retrofire"

"retrofired"

"retrofires"

"retrofiring"

"retrofit"

"retrofits"

"retrograde"

"retrograded"

"retrogradely"

"retrogrades"

"retrograding"

"retrogress"

"retrogressed"

"retrogresses"

"retrogressing"

"retrogression"

"retrogressions"

"retrogressive"

"retrogressively"

"retrorocket"

"retrorockets"

"retrospect"

"retrospection"

"retrospective"

"retrospectively"

"retrospectives"

"retry"

"retrying"

"retsina"

"retsinas"

"retuning"

"return"

"returnability"

"returnable"

"returned"

"returnee"

"returnees"

"returner"

"returners"

"returning"

"returns"

"retying"

"retype"

"retyped"

"retypes"

"retyping"

"reunification"

"reunifications"

"reunified"

"reunifies"

"reunify"

"reunifying"

"reunion"

"reunions"

"reunite"

"reunited"

"reuniter"

"reuniters"

"reunites"

"reuniting"

"reupholster"

"reupholstered"

"reupholstering"

"reupholsters"

"reusability"

"reusable"

"reusableness"

"reuse"

"reuseable"

"reuseableness"

"reused"

"reuses"

"reusing"

"reutilization"

"reutilizations"

"reutilize"

"reutilized"

"reutilizes"

"reutilizing"

"rev"

"revalidate"

"revalidated"

"revalidating"

"revalidation"

"revaluate"

"revaluated"

"revaluates"

"revaluating"

"revaluation"

"revaluations"

"revalue"

"revalued"

"revalues"

"revaluing"

"revamp"

"revamped"

"revamper"

"revampers"

"revamping"

"revamps"

"revarnish"

"revarnished"

"revarnishes"

"revarnishing"

"reveal"

"revealed"

"revealer"

"revealing"

"revealingly"

"revealment"

"reveals"

"reveille"

"reveilles"

"revel"

"revelation"

"revelational"

"revelations"

"revelator"

"revelatory"

"reveled"

"reveler"

"revelers"

"reveling"

"revelled"

"reveller"

"revellers"

"revelling"

"revellings"

"revelries"

"revelry"

"revels"

"revenant"

"revenants"

"revenge"

"revenged"

"revengeful"

"revengefully"

"revenger"

"revengers"

"revenges"

"revenging"

"revenual"

"revenue"

"revenued"

"revenuer"

"revenuers"

"revenues"

"reverb"

"reverberant"

"reverberate"

"reverberated"

"reverberates"

"reverberating"

"reverberation"

"reverberations"

"reverberator"

"reverberators"

"reverbs"

"revere"

"revered"

"reverence"

"reverenced"

"reverencer"

"reverencers"

"reverences"

"reverencing"

"reverend"

"reverends"

"reverent"

"reverential"

"reverently"

"reverer"

"reverers"

"reveres"

"reverie"

"reveries"

"reverification"

"reverifications"

"reverified"

"reverifies"

"reverify"

"reverifying"

"revering"

"revers"

"reversal"

"reversals"

"reverse"

"reversed"

"reversely"

"reverser"

"reversers"

"reverses"

"reversibility"

"reversible"

"reversibleness"

"reversibly"

"reversing"

"reversion"

"reversionary"

"reversionist"

"reversions"

"revert"

"reverted"

"reverter"

"reverters"

"revertible"

"reverting"

"reverts"

"revery"

"revested"

"revetment"

"revetments"

"revetted"

"revetting"

"revictual"

"revictualed"

"revictualing"

"revictuals"

"review"

"review articles"

"review questions"

"reviewability"

"reviewable"

"reviewal"

"reviewed"

"reviewer"

"reviewers"

"reviewing"

"reviews"

"revile"

"reviled"

"revilement"

"reviler"

"revilers"

"reviles"

"reviling"

"revindicate"

"revindicated"

"revindicates"

"revindicating"

"revindication"

"revisable"

"revisal"

"revisals"

"revise"

"revised"

"reviser"

"revisers"

"revises"

"revising"

"revision"

"revisionary"

"revisionism"

"revisionist"

"revisionists"

"revisions"

"revisit"

"revisited"

"revisiting"

"revisits"

"revisor"

"revisors"

"revisory"

"revitalization"

"revitalize"

"revitalized"

"revitalizes"

"revitalizing"

"revival"

"revivalism"

"revivalist"

"revivalistic"

"revivalists"

"revivals"

"revive"

"revived"

"reviver"

"revivers"

"revives"

"revivification"

"revivified"

"revivifies"

"revivify"

"revivifying"

"reviving"

"revocability"

"revocable"

"revocation"

"revocations"

"revocative"

"revocatory"

"revoir"

"revokable"

"revoke"

"revoked"

"revoker"

"revokers"

"revokes"

"revoking"

"revolt"

"revolted"

"revolter"

"revolters"

"revolting"

"revoltingly"

"revolts"

"revolution"

"revolutionaries"

"revolutionary"

"revolutionist"

"revolutionists"

"revolutionize"

"revolutionized"

"revolutionizer"

"revolutionizes"

"revolutionizing"

"revolutions"

"revolvable"

"revolve"

"revolved"

"revolver"

"revolvers"

"revolves"

"revolving"

"revs"

"revue"

"revues"

"revulsion"

"revulsions"

"revulsive"

"revved"

"revving"

"rewakened"

"rewakening"

"reward"

"rewardable"

"rewarded"

"rewarder"

"rewarders"

"rewarding"

"rewardingly"

"rewards"

"rewarm"

"rewarmed"

"rewarming"

"rewarms"

"rewash"

"rewashed"

"rewashes"

"rewashing"

"rewax"

"rewaxing"

"reweave"

"reweaved"

"reweaves"

"reweaving"

"rewed"

"rewedded"

"rewedding"

"reweds"

"reweigh"

"reweighed"

"reweighing"

"reweighs"

"reweld"

"rewelded"

"rewelding"

"rewelds"

"rewidening"

"rewin"

"rewind"

"rewinder"

"rewinders"

"rewinding"

"rewinds"

"rewire"

"rewired"

"rewires"

"rewiring"

"rewon"

"reword"

"reworded"

"rewording"

"rewords"

"rework"

"reworked"

"reworking"

"reworks"

"rewound"

"rewove"

"rewoven"

"rewrap"

"rewrapped"

"rewrapping"

"rewraps"

"rewrite"

"rewriter"

"rewriters"

"rewrites"

"rewriting"

"rewritten"

"rewrote"

"rewrought"

"rex"

"rexes"

"reykjavik"

"rezone"

"rezoned"

"rezones"

"rezoning"

"rf"

"rh"

"rhabdology"

"rhapsodic"

"rhapsodical"

"rhapsodically"

"rhapsodies"

"rhapsodist"

"rhapsodists"

"rhapsodize"

"rhapsodized"

"rhapsodizes"

"rhapsodizing"

"rhapsody"

"rhea"

"rheas"

"rhebok"

"rhenium"

"rheniums"

"rheologic"

"rheological"

"rheologist"

"rheologists"

"rheology"

"rheometer"

"rheometers"

"rheostat"

"rheostatic"

"rheostats"

"rhesus"

"rhesuses"

"rhetoric"

"rhetorical"

"rhetorically"

"rhetorician"

"rhetoricians"

"rhetorics"

"rhetors"

"rheum"

"rheumatic"

"rheumatically"

"rheumatism"

"rheumatogenic"

"rheumatoid"

"rheumatology"

"rheumic"

"rheumier"

"rheumiest"

"rheums"

"rheumy"

"rhine"

"rhinestone"

"rhinestones"

"rhinitis"

"rhino"

"rhinoceros"

"rhinoceroses"

"rhinology"

"rhinos"

"rhinoscope"

"rhinotillexomania"

"rhizomatous"

"rhizome"

"rhizomes"

"rho"

"rhochrematics"

"rhodes"

"rhodesia"

"rhodesian"

"rhodesians"

"rhodium"

"rhodiums"

"rhododendron"

"rhododendrons"

"rhodopsin"

"rhomb"

"rhombi"

"rhombic"

"rhomboid"

"rhomboids"

"rhombs"

"rhombus"

"rhombuses"

"rhonchi"

"rhubarb"

"rhubarbs"

"rhumb"

"rhumba"

"rhumbaed"

"rhumbaing"

"rhumbas"

"rhumbs"

"rhyme"

"rhymed"

"rhymer"

"rhymers"

"rhymes"

"rhymester"

"rhymesters"

"rhyming"

"rhyolite"

"rhyta"

"rhythm"

"rhythmic"

"rhythmical"

"rhythmically"

"rhythmicities"

"rhythmicity"

"rhythmics"

"rhythmometer"

"rhythms"

"ria"

"rial"

"rials"

"rialto"

"rib"

"ribald"

"ribaldly"

"ribaldries"

"ribaldry"

"ribalds"

"riband"

"ribands"

"ribbed"

"ribber"

"ribbers"

"ribbier"

"ribbing"

"ribbon"

"ribboned"

"ribboning"

"ribbons"

"ribbony"

"ribby"

"ribgrasses"

"ribless"

"riblet"

"riblets"

"riboflavin"

"ribonucleic"

"ribonucleotide"

"ribose"

"ribosomal"

"ribosome"

"ribosomes"

"ribs"

"rice"

"riced"

"ricer"

"ricercar"

"ricercars"

"ricers"

"rices"

"rich"

"richard"

"richardson"

"riche"

"richened"

"richening"

"richens"

"richer"

"riches"

"richest"

"richfield"

"richly"

"richmond"

"richness"

"richter"

"ricing"

"rick"

"ricked"

"ricketier"

"ricketiest"

"ricketiness"

"rickets"

"rickettsia"

"rickettsiae"

"rickettsial"

"rickettsias"

"rickety"

"rickey"

"rickeys"

"ricking"

"rickrack"

"rickracks"

"ricks"

"ricksha"

"rickshas"

"rickshaw"

"rickshaws"

"ricochet"

"ricocheted"

"ricocheting"

"ricochets"

"ricochetted"

"ricochetting"

"ricotta"

"ricottas"

"ricrac"

"ricracs"

"rictus"

"rictuses"

"rid"

"ridable"

"riddance"

"riddances"

"ridded"

"ridden"

"ridder"

"ridders"

"ridding"

"riddle"

"riddled"

"riddles"

"riddling"

"ride"

"rideable"

"rider"

"riderless"

"riders"

"ridership"

"riderships"

"rides"

"ridge"

"ridged"

"ridgepole"

"ridgepoles"

"ridges"

"ridgier"

"ridging"

"ridgy"

"ridicule"

"ridiculed"

"ridicules"

"ridiculing"

"ridiculous"

"ridiculously"

"ridiculousness"

"riding"

"ridings"

"ridley"

"rids"

"riel"

"riels"

"rife"

"rifely"

"rifeness"

"rifer"

"rifest"

"riff"

"riffed"

"riffing"

"riffle"

"riffled"

"riffler"

"rifflers"

"riffles"

"riffling"

"riffraff"

"riffraffs"

"riffs"

"rifle"

"rifled"

"rifleman"

"riflemen"

"rifler"

"rifleries"

"riflers"

"riflery"

"rifles"

"rifling"

"riflings"

"rift"

"rifted"

"rifting"

"riftless"

"rifts"

"rig"

"rigadoon"

"rigamarole"

"rigatoni"

"rigatonis"

"rigged"

"rigger"

"riggers"

"rigging"

"riggings"

"right"

"right brain functions"

"righted"

"righteous"

"righteously"

"righteousness"

"righter"

"righters"

"rightest"

"rightful"

"rightfully"

"rightfulness"

"righting"

"rightism"

"rightisms"

"rightist"

"rightists"

"rightly"

"rightness"

"righto"

"rights"

"rightward"

"righty"

"rigid"

"rigidified"

"rigidifies"

"rigidify"

"rigidities"

"rigidity"

"rigidly"

"rigidness"

"rigmarole"

"rigmaroles"

"rigor"

"rigorism"

"rigorisms"

"rigorist"

"rigorists"

"rigorous"

"rigorously"

"rigorousness"

"rigors"

"rigour"

"rigs"

"rigueur"

"rikshas"

"rikshaw"

"rile"

"riled"

"riles"

"riling"

"rill"

"rilled"

"rilling"

"rills"

"rim"

"rime"

"rimed"

"rimers"

"rimes"

"rimester"

"rimier"

"rimiest"

"riming"

"rimland"

"rimlands"

"rimless"

"rimmed"

"rimmer"

"rimmers"

"rimming"

"rimrock"

"rimrocks"

"rims"

"rimy"

"rind"

"rinded"

"rinds"

"ring"

"ringbolt"

"ringbolts"

"ringdove"

"ringdoves"

"ringed"

"ringer"

"ringers"

"ringing"

"ringleader"

"ringleaders"

"ringlet"

"ringlets"

"ringlike"

"ringmaster"

"ringmasters"

"ringneck"

"ringnecks"

"rings"

"ringside"

"ringsides"

"ringtail"

"ringtails"

"ringtone"

"ringtoss"

"ringtosses"

"ringworm"

"ringworms"

"rink"

"rinkomania"

"rinks"

"rinsable"

"rinse"

"rinsed"

"rinser"

"rinsers"

"rinses"

"rinsible"

"rinsing"

"rinsings"

"riometer"

"riot"

"rioted"

"rioter"

"rioters"

"rioting"

"riotous"

"riotously"

"riotousness"

"riots"

"rip"

"riparian"

"ripcord"

"ripcords"

"ripe"

"ripely"

"ripen"

"ripened"

"ripener"

"ripeners"

"ripeness"

"ripening"

"ripens"

"riper"

"ripes"

"ripest"

"riping"

"ripoff"

"ripoffs"

"ripost"

"riposte"

"riposted"

"ripostes"

"riposting"

"riposts"

"rippable"

"ripped"

"ripper"

"rippers"

"ripping"

"ripple"

"rippled"

"rippler"

"ripplers"

"ripples"

"ripplets"

"ripplier"

"rippliest"

"rippling"

"ripply"

"riprap"

"riprapped"

"riprapping"

"ripraps"

"rips"

"ripsaw"

"ripsaws"

"riptide"

"riptides"

"rise"

"risen"

"riser"

"risers"

"rises"

"rish"

"rishi"

"rishis"

"risibility"

"risible"

"risibles"

"risibly"

"rising"

"risings"

"risk"

"risk assessment"

"risked"

"risker"

"riskers"

"riskier"

"riskiest"

"riskily"

"riskiness"

"risking"

"risks"

"risky"

"risotto"

"risottos"

"risque"

"rissole"

"risus"

"ritard"

"ritards"

"rite"

"rites"

"ritish"

"ritual"

"ritualism"

"ritualist"

"ritualistic"

"ritualistically"

"ritualists"

"ritualization"

"ritualize"

"ritualized"

"ritually"

"rituals"

"ritz"

"ritzes"

"ritzier"

"ritziest"

"ritzily"

"ritziness"

"ritzy"

"rival"

"rivaled"

"rivaling"

"rivalled"

"rivalling"

"rivalries"

"rivalry"

"rivals"

"rive"

"rived"

"rivederci"

"riven"

"river"

"riverbank"

"riverbanks"

"riverbed"

"riverbeds"

"riverine"

"rivers"

"riverside"

"rives"

"rivet"

"riveted"

"riveter"

"riveters"

"riveting"

"rivets"

"rivetted"

"rivetting"

"riviera"

"rivieras"

"riving"

"rivulet"

"rivulets"

"riyal"

"riyals"

"RMT"

"rn"

"roach"

"roached"

"roaches"

"roaching"

"road"

"roadability"

"roadbed"

"roadbeds"

"roadblock"

"roadblocks"

"roader"

"roaders"

"roadhouse"

"roadhouses"

"roadless"

"roadrunner"

"roadrunners"

"roads"

"roadside"

"roadsides"

"roadstead"

"roadsteads"

"roadster"

"roadsters"

"roadway"

"roadways"

"roadwork"

"roadworks"

"roam"

"roamed"

"roamer"

"roamers"

"roaming"

"roams"

"roan"

"roans"

"roar"

"roared"

"roarer"

"roarers"

"roaring"

"roarings"

"roars"

"roast"

"roasted"

"roaster"

"roasters"

"roasting"

"roasts"

"rob"

"robbed"

"robber"

"robberies"

"robbers"

"robbery"

"robbing"

"robbins"

"robe"

"robed"

"robert"

"roberts"

"robes"

"robin"

"robing"

"robins"

"robinson"

"roble"

"robles"

"robot"

"robotics"

"robotism"

"robotisms"

"robotization"

"robotize"

"robotized"

"robotizes"

"robotizing"

"robotry"

"robots"

"robs"

"robust"

"robuster"

"robustest"

"robustly"

"robustness"

"roc"

"rochester"

"rock"

"rockabies"

"rockaby"

"rockabye"

"rockabyes"

"rockaways"

"rocked"

"rocker"

"rockeries"

"rockers"

"rockery"

"rocket"

"rocketed"

"rocketer"

"rocketers"

"rocketing"

"rocketlike"

"rocketries"

"rocketry"

"rockets"

"rockfall"

"rockfalls"

"rockfish"

"rockfishes"

"rockier"

"rockies"

"rockiest"

"rockiness"

"rocking"

"rockless"

"rocklike"

"rockroses"

"rocks"

"rockworks"

"rocky"

"rococo"

"rococos"

"rocs"

"rod"

"rodded"

"rodder"

"rodders"

"rodding"

"rode"

"rodent"

"rodenticide"

"rodents"

"rodeo"

"rodeos"

"rodless"

"rodman"

"rodmen"

"rodriguez"

"rods"

"roe"

"roebuck"

"roebucks"

"roentgen"

"roentgenize"

"roentgenogram"

"roentgenograms"

"roentgenographic"

"roentgenography"

"roentgenologic"

"roentgenological"

"roentgenologies"

"roentgenologist"

"roentgenologists"

"roentgenology"

"roentgenometer"

"roentgenometries"

"roentgenometry"

"roentgenoscope"

"roentgenoscopic"

"roentgenoscopies"

"roentgenoscopy"

"roentgens"

"roes"

"roger"

"rogers"

"rogue"

"rogued"

"rogueing"

"rogueries"

"roguery"

"rogues"

"roguing"

"roguish"

"roguishly"

"roguishness"

"roil"

"roiled"

"roilier"

"roiling"

"roils"

"roily"

"roister"

"roistered"

"roisterer"

"roisterers"

"roistering"

"roisterous"

"roisters"

"role"

"roleplayed"

"roleplaying"

"roles"

"roll"

"rollaway"

"rollback"

"rollbacks"

"rolled"

"roller"

"rollers"

"rollick"

"rollicked"

"rollicking"

"rollickingly"

"rollicks"

"rolling"

"rollings"

"rollout"

"rollover"

"rollovers"

"rolls"

"rolltop"

"rollway"

"rom"

"romaine"

"romaines"

"roman"

"romance"

"romanced"

"romancer"

"romancers"

"romances"

"romancing"

"romanesque"

"romanian"

"romanies"

"romanism"

"romanist"

"romanistic"

"romanize"

"romanized"

"romanizes"

"romanizing"

"romano"

"romans"

"romantic"

"romantically"

"romanticism"

"romanticist"

"romanticists"

"romanticization"

"romanticize"

"romanticized"

"romanticizes"

"romanticizing"

"romantics"

"romany"

"rome"

"romeo"

"romp"

"romped"

"romper"

"rompers"

"romping"

"rompish"

"romps"

"roms"

"ronald"

"rondeau"

"rondeaux"

"rondelle"

"rondo"

"rondos"

"rondure"

"rondures"

"rontgen"

"rood"

"roods"

"roof"

"roofed"

"roofer"

"roofers"

"roofing"

"roofings"

"roofless"

"roofline"

"rooflines"

"roofs"

"rooftop"

"rooftops"

"rooftree"

"rooftrees"

"rook"

"rooked"

"rookeries"

"rookery"

"rookie"

"rookier"

"rookies"

"rooking"

"rooks"

"rooky"

"room"

"roomed"

"roomer"

"roomers"

"roomette"

"roomettes"

"roomful"

"roomfuls"

"roomier"

"roomiest"

"roomily"

"roominess"

"rooming"

"roommate"

"roommates"

"rooms"

"roomy"

"roosevelt"

"roost"

"roosted"

"rooster"

"roosters"

"roosting"

"roosts"

"root"

"rooted"

"rooter"

"rooters"

"rootier"

"rooting"

"rootless"

"rootlet"

"rootlets"

"rootlike"

"roots"

"rootstock"

"rootstocks"

"rooty"

"ropable"

"rope"

"roped"

"roper"

"roperies"

"ropers"

"ropery"

"ropes"

"ropewalk"

"ropewalks"

"ropeway"

"ropeways"

"ropier"

"ropiest"

"ropily"

"ropiness"

"roping"

"ropy"

"roquefort"

"rorschach"

"rosa"

"rosalind"

"rosalyn"

"rosarian"

"rosaries"

"rosarium"

"rosary"

"roscoe"

"roscoes"

"rose"

"roseate"

"rosebay"

"rosebud"

"rosebuds"

"rosebush"

"rosebushes"

"rosed"

"rosemaries"

"rosemary"

"rosery"

"roses"

"rosets"

"rosette"

"rosettes"

"rosewater"

"rosewood"

"rosewoods"

"roshi"

"rosier"

"rosiest"

"rosily"

"rosin"

"rosined"

"rosiness"

"rosing"

"rosining"

"rosinous"

"rosins"

"rosiny"

"roster"

"rosters"

"rostra"

"rostral"

"rostrum"

"rostrums"

"rosy"

"rot"

"rotameter"

"rotaries"

"rotary"

"rotatable"

"rotate"

"rotated"

"rotates"

"rotating"

"rotation"

"rotational"

"rotational views"

"rotationally"

"rotations"

"rotative"

"rotatively"

"rotator"

"rotators"

"rotatory"

"rote"

"rotes"

"rotgut"

"rotguts"

"rotifer"

"rotifers"

"rotisserie"

"rotisseries"

"roto"

"rotogravure"

"rotogravures"

"rotor"

"rotors"

"rototill"

"rototilled"

"rototiller"

"rototills"

"rots"

"rotted"

"rotten"

"rottener"

"rottenest"

"rottenly"

"rottenness"

"rotter"

"rotterdam"

"rotters"

"rotting"

"rotund"

"rotunda"

"rotundas"

"rotundity"

"rotundly"

"rotundness"

"rouble"

"roubles"

"roue"

"rouens"

"roues"

"rouge"

"rouged"

"rouges"

"rough"

"roughage"

"roughages"

"roughcast"

"roughed"

"roughen"

"roughened"

"roughening"

"roughens"

"rougher"

"roughers"

"roughest"

"roughhew"

"roughhewed"

"roughhewing"

"roughhewn"

"roughhews"

"roughhouse"

"roughhoused"

"roughhouses"

"roughhousing"

"roughing"

"roughish"

"roughly"

"roughneck"

"roughnecks"

"roughness"

"roughnesses"

"roughs"

"roughshod"

"rouging"

"roulade"

"rouleau"

"roulette"

"rouletted"

"roulettes"

"rouletting"

"round"

"roundabout"

"rounded"

"roundel"

"roundelay"

"roundelays"

"rounder"

"rounders"

"roundest"

"roundhouse"

"roundhouses"

"rounding"

"roundish"

"roundly"

"roundness"

"rounds"

"roundup"

"roundups"

"roundworm"

"roundworms"

"rouse"

"roused"

"rouser"

"rousers"

"rouses"

"rousing"

"rousseau"

"rousseaus"

"roust"

"roustabout"

"roustabouts"

"rousted"

"rouster"

"rousters"

"rousting"

"rousts"

"rout"

"route"

"routed"

"routeman"

"routemen"

"router"

"routers"

"routes"

"routeway"

"routeways"

"routine"

"routinely"

"routines"

"routing"

"routings"

"routinize"

"routinized"

"routinizes"

"routinizing"

"routs"

"roux"

"rove"

"roved"

"rover"

"rovers"

"roves"

"roving"

"rovingly"

"rovings"

"row"

"rowable"

"rowan"

"rowans"

"rowboat"

"rowboats"

"rowdier"

"rowdies"

"rowdiest"

"rowdily"

"rowdiness"

"rowdy"

"rowdyish"

"rowdyism"

"rowdyisms"

"rowed"

"rowel"

"rowels"

"rower"

"rowers"

"rowing"

"rowings"

"rows"

"royal"

"royalism"

"royalisms"

"royalist"

"royalists"

"royally"

"royals"

"royalties"

"royalty"

"roystered"

"rpm"

"rs"

"rte"

"rub"

"rubaiyat"

"rubato"

"rubatos"

"rubbed"

"rubber"

"rubberize"

"rubberized"

"rubberizes"

"rubberizing"

"rubberneck"

"rubbernecked"

"rubbernecking"

"rubbernecks"

"rubbers"

"rubbery"

"rubbing"

"rubbings"

"rubbish"

"rubbishes"

"rubbishy"

"rubble"

"rubbled"

"rubbles"

"rubblier"

"rubbliest"

"rubbling"

"rubbly"

"rubdown"

"rubdowns"

"rube"

"rubella"

"rubellas"

"rubens"

"rubes"

"rubicund"

"rubicundity"

"rubidium"

"rubidiums"

"rubied"

"rubier"

"rubies"

"rubiest"

"ruble"

"rubles"

"rubric"

"rubrical"

"rubrics"

"rubs"

"ruby"

"rubying"

"ruck"

"rucked"

"rucks"

"rucksack"

"rucksacks"

"ruckus"

"ruckuses"

"ructions"

"ructious"

"rudder"

"rudderless"

"rudders"

"ruddier"

"ruddiest"

"ruddily"

"ruddiness"

"ruddle"

"ruddy"

"rude"

"rudely"

"rudeness"

"ruder"

"rudest"

"rudiment"

"rudimentary"

"rudiments"

"rue"

"rued"

"rueful"

"ruefully"

"ruefulness"

"ruer"

"ruers"

"rues"

"ruff"

"ruffed"

"ruffes"

"ruffian"

"ruffianly"

"ruffians"

"ruffing"

"ruffle"

"ruffled"

"ruffler"

"rufflers"

"ruffles"

"rufflike"

"ruffling"

"ruffly"

"ruffs"

"rufous"

"rug"

"rugbies"

"rugby"

"rugged"

"ruggeder"

"ruggedest"

"ruggedly"

"ruggedness"

"rugger"

"ruggers"

"rugging"

"ruglike"

"rugs"

"ruin"

"ruinable"

"ruinate"

"ruinated"

"ruinates"

"ruinating"

"ruination"

"ruined"

"ruiner"

"ruiners"

"ruing"

"ruining"

"ruinous"

"ruinously"

"ruinousness"

"ruins"

"rulable"

"rule"

"ruled"

"ruleless"

"ruler"

"rulers"

"rulership"

"rules"

"ruling"

"rulings"

"rum"

"rumania"

"rumanian"

"rumanians"

"rumba"

"rumbaed"

"rumbaing"

"rumbas"

"rumble"

"rumbled"

"rumbler"

"rumblers"

"rumbles"

"rumbling"

"rumblingly"

"rumblings"

"rumbly"

"ruminant"

"ruminants"

"ruminate"

"ruminated"

"ruminates"

"ruminating"

"ruminatingly"

"rumination"

"ruminations"

"ruminative"

"ruminator"

"ruminators"

"rummage"

"rummaged"

"rummager"

"rummagers"

"rummages"

"rummaging"

"rummer"

"rummers"

"rummest"

"rummier"

"rummies"

"rummiest"

"rummy"

"rumor"

"rumored"

"rumoring"

"rumormonger"

"rumors"

"rumour"

"rumoured"

"rumouring"

"rumours"

"rump"

"rumpelstiltskin"

"rumple"

"rumpled"

"rumples"

"rumpless"

"rumpliest"

"rumpling"

"rumply"

"rumps"

"rumpus"

"rumpuses"

"rumrunner"

"rumrunners"

"rumrunning"

"rums"

"run"

"runabout"

"runabouts"

"runagates"

"runaround"

"runaway"

"runaways"

"runback"

"rundlet"

"rundown"

"rundowns"

"rune"

"runes"

"rung"

"rungless"

"rungs"

"runic"

"runless"

"runlet"

"runlets"

"runnel"

"runnels"

"runner"

"runners"

"runnier"

"runniest"

"running"

"runnings"

"runny"

"runoff"

"runoffs"

"runology"

"runout"

"runouts"

"runover"

"runrounds"

"runs"

"runt"

"runtier"

"runtiest"

"runtiness"

"runtish"

"runts"

"runty"

"runway"

"runways"

"rupee"

"rupees"

"rupiah"

"rupiahs"

"rupturable"

"rupture"

"ruptured"

"ruptures"

"rupturing"

"rural"

"ruralism"

"ruralisms"

"ruralist"

"ruralists"

"ruralite"

"ruralites"

"ruralities"

"rurality"

"ruralize"

"ruralized"

"ruralizes"

"ruralizing"

"rurally"

"ruse"

"ruses"

"rush"

"rushed"

"rushee"

"rusher"

"rushers"

"rushes"

"rushier"

"rushing"

"rushingly"

"rushings"

"rushy"

"rusk"

"rusks"

"russe"

"russell"

"russet"

"russets"

"russety"

"russia"

"russian"

"russians"

"russified"

"russifies"

"russify"

"russifying"

"Russophobia"

"rust"

"rustable"

"rusted"

"rustic"

"rustical"

"rustically"

"rusticate"

"rusticated"

"rusticates"

"rusticating"

"rustication"

"rusticator"

"rusticators"

"rusticity"

"rusticly"

"rustics"

"rustier"

"rustiest"

"rustily"

"rustiness"

"rusting"

"rustle"

"rustled"

"rustler"

"rustlers"

"rustles"

"rustless"

"rustling"

"rustlingly"

"rustproof"

"rusts"

"rusty"

"rut"

"rutabaga"

"rutabagas"

"ruth"

"ruthenium"

"rutherford"

"rutherfordium"

"ruthless"

"ruthlessly"

"ruthlessness"

"ruths"

"ruts"

"rutted"

"ruttier"

"ruttiest"

"ruttily"

"ruttiness"

"rutting"

"ruttish"

"rutty"

"rya"

"ryas"

"rye"

"ryegrass"

"ryegrasses"

"ryes"

"s"

"sa"

"sabbat"

"sabbath"

"sabbaths"

"sabbatic"

"sabbatical"

"sabbaticals"

"sabbats"

"saber"

"sabered"

"sabering"

"sabers"

"sabin"

"sabine"

"sabines"

"sable"

"sables"

"sabot"

"sabotage"

"sabotaged"

"sabotages"

"sabotaging"

"saboteur"

"saboteurs"

"sabots"

"sabra"

"sabras"

"sabred"

"sabres"

"sabring"

"sac"

"sacbut"

"sacbuts"

"saccharated"

"saccharification"

"saccharimeter"

"saccharin"

"saccharine"

"saccharinely"

"saccharinity"

"sacerdotal"

"sacerdotalism"

"sacerdotally"

"sachem"

"sachemic"

"sachems"

"sachet"

"sacheted"

"sachets"

"sack"

"sackbut"

"sackbuts"

"sackcloth"

"sackclothed"

"sacked"

"sacker"

"sackers"

"sackful"

"sackfuls"

"sacking"

"sackings"

"sacks"

"sacksful"

"saclike"

"sacra"

"sacral"

"sacrals"

"sacrament"

"sacramental"

"sacramentally"

"sacramento"

"sacraments"

"sacred"

"sacredly"

"sacredness"

"sacrifice"

"sacrificed"

"sacrificer"

"sacrificers"

"sacrifices"

"sacrificial"

"sacrificially"

"sacrificing"

"sacrilege"

"sacrilegious"

"sacrilegiously"

"sacrilegiousness"

"sacrist"

"sacristan"

"sacristans"

"sacristies"

"sacristry"

"sacrists"

"sacristy"

"sacroiliac"

"sacroiliacs"

"sacrolumbar"

"sacrosanct"

"sacrosanctness"

"sacrovertebral"

"sacrum"

"sacrums"

"sacs"

"sad"

"sadden"

"saddened"

"saddening"

"saddens"

"sadder"

"saddest"

"saddhu"

"saddhus"

"saddle"

"saddlebag"

"saddlebags"

"saddlebow"

"saddlebows"

"saddlecloth"

"saddled"

"saddler"

"saddleries"

"saddlers"

"saddlery"

"saddles"

"saddletree"

"saddling"

"sadducee"

"sadhu"

"sadhus"

"sadiron"

"sadirons"

"sadism"

"sadisms"

"sadist"

"sadistic"

"sadistically"

"sadists"

"sadly"

"sadness"

"sadnesses"

"sadomasochism"

"sadomasochist"

"sadomasochistic"

"sadomasochists"

"safari"

"safaried"

"safariing"

"safaris"

"safe"

"safecracker"

"safecracking"

"safegaurds"

"safeguard"

"safeguarded"

"safeguarding"

"safeguards"

"safekeeping"

"safelight"

"safely"

"safeness"

"safer"

"safes"

"safest"

"safetied"

"safeties"

"safety"

"safety sake"

"safetying"

"safeway"

"safflower"

"safflowers"

"saffron"

"saffrons"

"sag"

"saga"

"sagacious"

"sagaciously"

"sagacities"

"sagacity"

"sagamore"

"sagamores"

"sagas"

"sage"

"sagebrush"

"sagebrushes"

"sagely"

"sageness"

"sager"

"sages"

"sagest"

"sagged"

"sagger"

"saggers"

"saggier"

"saggiest"

"sagging"

"saggy"

"sagier"

"sagiest"

"sagittal"

"sagittarius"

"sago"

"sagos"

"sags"

"saguaro"

"saguaros"

"sagy"

"sahara"

"saharan"

"sahib"

"sahibs"

"sahuaros"

"said"

"saids"

"saigon"

"sail"

"sailable"

"sailboat"

"sailboats"

"sailcloth"

"sailed"

"sailer"

"sailers"

"sailfish"

"sailfishes"

"sailing"

"sailings"

"sailor"

"sailorly"

"sailors"

"sails"

"saint"

"saintdom"

"saintdoms"

"sainted"

"sainthood"

"sainting"

"saintlier"

"saintliest"

"saintliness"

"saintly"

"saints"

"saintship"

"saith"

"sake"

"sakes"

"sakis"

"sal"

"salaam"

"salaamed"

"salaaming"

"salaams"

"salabilities"

"salability"

"salable"

"salably"

"salacious"

"salaciously"

"salaciousness"

"salacity"

"salad"

"salads"

"salamander"

"salamanders"

"salami"

"salamis"

"salaried"

"salaries"

"salary"

"salarying"

"sale"

"saleable"

"saleably"

"salem"

"saleroom"

"salerooms"

"sales"

"salesclerk"

"salesclerks"

"salesgirl"

"salesgirls"

"salesladies"

"saleslady"

"salesman"

"salesmanship"

"salesmen"

"salespeople"

"salesperson"

"salespersons"

"salesroom"

"salesrooms"

"saleswoman"

"saleswomen"

"saleyard"

"salicylic"

"salience"

"saliences"

"saliencies"

"saliency"

"salient"

"saliently"

"salients"

"salinas"

"saline"

"salines"

"salinities"

"salinity"

"salinize"

"salinized"

"salinizes"

"salinizing"

"salinometer"

"salisbury"

"saliva"

"salivary"

"salivas"

"salivate"

"salivated"

"salivates"

"salivating"

"salivation"

"sallied"

"sallier"

"sallies"

"sallow"

"sallower"

"sallowest"

"sallowing"

"sallowly"

"sallowness"

"sallows"

"sallowy"

"sally"

"sallying"

"salmagundi"

"salmagundis"

"salmon"

"salmonella"

"salmonellas"

"salmons"

"salon"

"salons"

"saloon"

"saloons"

"salsa"

"salsify"

"salt"

"saltation"

"saltatory"

"saltbox"

"saltboxes"

"saltbush"

"saltbushes"

"saltcellar"

"saltcellars"

"salted"

"salter"

"salters"

"saltest"

"saltier"

"salties"

"saltiest"

"saltily"

"saltine"

"saltines"

"saltiness"

"salting"

"saltires"

"saltish"

"saltless"

"saltness"

"saltpans"

"saltpeter"

"saltpetre"

"salts"

"saltshaker"

"saltwater"

"saltworks"

"saltworts"

"salty"

"salubrious"

"salubriously"

"salubriousness"

"salubrities"

"salubrity"

"salutarily"

"salutariness"

"salutary"

"salutation"

"salutations"

"salutatory"

"salute"

"saluted"

"saluter"

"saluters"

"salutes"

"saluting"

"salvable"

"salvably"

"salvador"

"salvage"

"salvageability"

"salvageable"

"salvaged"

"salvagee"

"salvagees"

"salvager"

"salvagers"

"salvages"

"salvaging"

"salvation"

"salvational"

"salvations"

"salve"

"salved"

"salver"

"salvers"

"salves"

"salvia"

"salvias"

"salving"

"salvo"

"salvoed"

"salvoes"

"salvoing"

"salvos"

"sam"

"samadhi"

"samaritan"

"samaritans"

"samarium"

"samariums"

"samba"

"sambaed"

"sambaing"

"sambas"

"sambo"

"sambos"

"same"

"samechs"

"samekhs"

"sameness"

"samisen"

"samisens"

"samite"

"samites"

"samizdat"

"samlet"

"samoa"

"samoan"

"samoans"

"samovar"

"samovars"

"sampan"

"sampans"

"samphires"

"sample"

"sampled"

"sampler"

"samplers"

"samples"

"sampling"

"samplings"

"samsara"

"samsaras"

"samuel"

"samurai"

"samurais"

"san"

"sanatarium"

"sanatoria"

"sanatorium"

"sanatoriums"

"sanatory"

"sancta"

"sanctification"

"sanctifications"

"sanctified"

"sanctifier"

"sanctifiers"

"sanctifies"

"sanctify"

"sanctifying"

"sanctimonious"

"sanctimoniously"

"sanctimoniousness"

"sanctimony"

"sanction"

"sanctioned"

"sanctioner"

"sanctioners"

"sanctioning"

"sanctions"

"sanctities"

"sanctity"

"sanctuaries"

"sanctuary"

"sanctum"

"sanctums"

"sand"

"sandal"

"sandaled"

"sandaling"

"sandalled"

"sandalling"

"sandals"

"sandalwood"

"sandalwoods"

"sandbag"

"sandbagged"

"sandbagger"

"sandbaggers"

"sandbagging"

"sandbags"

"sandbank"

"sandbanks"

"sandbar"

"sandbars"

"sandblast"

"sandblasted"

"sandblaster"

"sandblasters"

"sandblasting"

"sandblasts"

"sandbox"

"sandboxes"

"sandburrs"

"sanded"

"sander"

"sanders"

"sandfishes"

"sandflies"

"sandfly"

"sandhog"

"sandhogs"

"sandier"

"sandiest"

"sandiness"

"sanding"

"sandlot"

"sandlots"

"sandlotter"

"sandlotters"

"sandman"

"sandmen"

"sandpaper"

"sandpapered"

"sandpapering"

"sandpapers"

"sandpile"

"sandpiper"

"sandpipers"

"sandpit"

"sandpits"

"sandra"

"sands"

"sandsoaps"

"sandstone"

"sandstones"

"sandstorm"

"sandwich"

"sandwiched"

"sandwiches"

"sandwiching"

"sandworms"

"sandwort"

"sandy"

"sane"

"saned"

"sanely"

"saneness"

"saner"

"sanes"

"sanest"

"sanforized"

"sang"

"sanga"

"sanger"

"sangfroid"

"sangh"

"sangha"

"sangria"

"sangrias"

"sanguification"

"sanguinarily"

"sanguinary"

"sanguine"

"sanguinely"

"sanguineness"

"sanguines"

"sanicles"

"sanitaria"

"sanitarian"

"sanitarians"

"sanitaries"

"sanitarily"

"sanitarium"

"sanitariums"

"sanitary"

"sanitated"

"sanitates"

"sanitating"

"sanitation"

"sanitationist"

"sanities"

"sanitization"

"sanitize"

"sanitized"

"sanitizer"

"sanitizes"

"sanitizing"

"sanitoria"

"sanitorium"

"sanity"

"sank"

"sanka"

"sannyasi"

"sans"

"sansei"

"sanseis"

"sanserif"

"sanserifs"

"sanskrit"

"santa"

"santee"

"santiago"

"santonins"

"sanzen"

"sap"

"saphead"

"sapheads"

"sapid"

"sapidity"

"sapience"

"sapiences"

"sapiencies"

"sapiency"

"sapiens"

"sapient"

"sapiently"

"sapless"

"sapling"

"saplings"

"saponify"

"saponine"

"sapor"

"sapped"

"sapper"

"sappers"

"sapphic"

"sapphics"

"sapphire"

"sapphires"

"sapphism"

"sapphisms"

"sapphist"

"sapphists"

"sappier"

"sappiest"

"sappily"

"sappiness"

"sapping"

"sappy"

"saprophagous"

"saprophyte"

"saprophytes"

"saprophytic"

"saprophytically"

"saps"

"sapsucker"

"sapsuckers"

"sapwood"

"sapwoods"

"saraband"

"sarabands"

"saracen"

"saracenic"

"saracens"

"sarah"

"saran"

"sarape"

"sarapes"

"sarcasm"

"sarcasms"

"sarcastic"

"sarcastically"

"sarcology"

"sarcoma"

"sarcomas"

"sarcomata"

"sarcophagi"

"sarcophagus"

"sarcophaguses"

"sardine"

"sardines"

"sardinia"

"sardinian"

"sardinians"

"sardonic"

"sardonically"

"sardonyx"

"sardonyxes"

"saree"

"sarees"

"sargasso"

"sargassos"

"sarge"

"sarges"

"sari"

"saris"

"sarod"

"sarong"

"sarongs"

"sarsaparilla"

"sarsaparillas"

"sartor"

"sartorial"

"sartorially"

"sash"

"sashay"

"sashayed"

"sashaying"

"sashays"

"sashed"

"sashes"

"sashimi"

"sashimis"

"sashing"

"saskatchewan"

"sass"

"sassafras"

"sassafrases"

"sassed"

"sasses"

"sassier"

"sassies"

"sassiest"

"sassily"

"sassing"

"sassy"

"sat"

"satan"

"satanic"

"satanical"

"satanically"

"satanism"

"satanisms"

"satanist"

"satanists"

"satanology"

"satanophobia"

"satchel"

"satchels"

"sate"

"sated"

"sateen"

"sateens"

"satellite"

"satellites"

"sates"

"satiable"

"satiably"

"satiate"

"satiated"

"satiates"

"satiating"

"satiation"

"satieties"

"satiety"

"satin"

"sating"

"satinpods"

"satins"

"satinwood"

"satinwoods"

"satiny"

"satire"

"satires"

"satiric"

"satirical"

"satirically"

"satirist"

"satirists"

"satirize"

"satirized"

"satirizer"

"satirizers"

"satirizes"

"satirizing"

"satisfaction"

"satisfactions"

"satisfactorily"

"satisfactoriness"

"satisfactory"

"satisfiable"

"satisfied"

"satisfier"

"satisfiers"

"satisfies"

"satisfy"

"satisfying"

"satisfyingly"

"sativa"

"satori"

"satoris"

"satrap"

"satrapies"

"satraps"

"satrapy"

"saturable"

"saturants"

"saturate"

"saturated"

"saturates"

"saturating"

"saturation"

"saturations"

"saturday"

"saturdays"

"saturn"

"saturnine"

"saturninity"

"saturnism"

"satyr"

"satyriases"

"satyriasis"

"satyric"

"satyrid"

"satyromania"

"satyrs"

"sauce"

"saucebox"

"sauceboxes"

"sauced"

"saucepan"

"saucepans"

"saucer"

"saucerize"

"saucerized"

"saucers"

"sauces"

"saucier"

"sauciest"

"saucily"

"sauciness"

"saucing"

"saucy"

"saudi"

"saudis"

"sauerbraten"

"sauerkraut"

"sauls"

"sault"

"saults"

"sauna"

"saunas"

"saunter"

"sauntered"

"saunterer"

"saunterers"

"sauntering"

"saunters"

"saurian"

"saurians"

"sauropod"

"sauropods"

"sausage"

"sausages"

"saute"

"sauted"

"sauteed"

"sauteing"

"sauterne"

"sauternes"

"sautes"

"savable"

"savage"

"savaged"

"savagely"

"savageness"

"savager"

"savageries"

"savagery"

"savages"

"savagest"

"savaging"

"savagism"

"savagisms"

"savanna"

"savannah"

"savannahs"

"savannas"

"savant"

"savants"

"savate"

"savates"

"save"

"saveable"

"saved"

"saver"

"savers"

"saves"

"saving"

"savingly"

"savings"

"savior"

"saviors"

"saviour"

"saviours"

"savor"

"savored"

"savorer"

"savorers"

"savorier"

"savories"

"savoriest"

"savorily"

"savoriness"

"savoring"

"savorous"

"savors"

"savory"

"savour"

"savoured"

"savourer"

"savourers"

"savourier"

"savouries"

"savouriest"

"savouring"

"savours"

"savoury"

"savoy"

"savoys"

"savvied"

"savvies"

"savvy"

"savvying"

"saw"

"sawbills"

"sawbones"

"sawboneses"

"sawbuck"

"sawbucks"

"sawdust"

"sawdusts"

"sawed"

"sawer"

"sawers"

"sawfish"

"sawfishes"

"sawflies"

"sawfly"

"sawhorse"

"sawhorses"

"sawing"

"sawmill"

"sawmills"

"sawn"

"saws"

"sawteeth"

"sawtooth"

"sawyer"

"sawyers"

"sax"

"saxes"

"saxhorn"

"saxhorns"

"saxon"

"saxonies"

"saxons"

"saxony"

"saxophone"

"saxophones"

"saxophonist"

"saxophonists"

"say"

"sayable"

"sayee"

"sayer"

"sayers"

"sayest"

"saying"

"sayings"

"sayonara"

"sayonaras"

"says"

"sayst"

"sc"

"scab"

"scabbard"

"scabbarded"

"scabbards"

"scabbed"

"scabbier"

"scabbiest"

"scabbily"

"scabbiness"

"scabbing"

"scabby"

"scabies"

"scabiosa"

"scabious"

"scabrous"

"scabrously"

"scabrousness"

"scabs"

"scad"

"scads"

"scaffold"

"scaffoldage"

"scaffolded"

"scaffolding"

"scaffolds"

"scag"

"scags"

"scalable"

"scalably"

"scalar"

"scalars"

"scalawag"

"scalawags"

"scald"

"scalded"

"scaldic"

"scalding"

"scalds"

"scale"

"scaled"

"scaleless"

"scalelike"

"scalene"

"scalepan"

"scalepans"

"scaler"

"scalers"

"scales"

"scalesman"

"scalesmen"

"scalier"

"scaliest"

"scaliness"

"scaling"

"scallion"

"scallions"

"scallop"

"scalloped"

"scalloper"

"scallopers"

"scalloping"

"scallops"

"scalls"

"scallywag"

"scalp"

"scalped"

"scalpel"

"scalpels"

"scalper"

"scalpers"

"scalping"

"scalps"

"scaly"

"scam"

"scammonies"

"scamp"

"scamped"

"scamper"

"scampered"

"scampering"

"scampers"

"scampi"

"scamping"

"scampish"

"scamps"

"scams"

"scan"

"scandal"

"scandaled"

"scandaling"

"scandalization"

"scandalize"

"scandalized"

"scandalizer"

"scandalizers"

"scandalizes"

"scandalizing"

"scandalled"

"scandalmonger"

"scandalous"

"scandalously"

"scandalousness"

"scandals"

"scandia"

"scandic"

"scandinavia"

"scandinavian"

"scandinavians"

"scandium"

"scandiums"

"scanned"

"scanner"

"scanners"

"scanning"

"scannings"

"scans"

"scansion"

"scansions"

"scant"

"scanted"

"scanter"

"scantest"

"scantier"

"scanties"

"scantiest"

"scantily"

"scantiness"

"scanting"

"scantling"

"scantlings"

"scantly"

"scantness"

"scants"

"scanty"

"scape"

"scaped"

"scapegoat"

"scapegoater"

"scapegoatism"

"scapegoats"

"scapegrace"

"scapegraces"

"scapes"

"scaping"

"scapula"

"scapulae"

"scapular"

"scapulars"

"scapulas"

"scar"

"scarab"

"scarabs"

"scarce"

"scarcely"

"scarceness"

"scarcer"

"scarcest"

"scarcities"

"scarcity"

"scare"

"scarecrow"

"scarecrows"

"scared"

"scarer"

"scarers"

"scares"

"scarey"

"scarf"

"scarfed"

"scarfing"

"scarfpin"

"scarfpins"

"scarfs"

"scarier"

"scariest"

"scarification"

"scarificator"

"scarified"

"scarifier"

"scarifies"

"scarify"

"scarifying"

"scariness"

"scaring"

"scarless"

"scarlet"

"scarletina"

"scarlets"

"scarp"

"scarped"

"scarper"

"scarpering"

"scarps"

"scarred"

"scarrier"

"scarriest"

"scarring"

"scarry"

"scars"

"scarting"

"scarves"

"scary"

"scat"

"scathe"

"scathed"

"scathes"

"scathing"

"scathingly"

"scatologic"

"scatological"

"scatologies"

"scatology"

"scatophagies"

"scatophagous"

"scats"

"scatted"

"scatter"

"scatterbrain"

"scatterbrained"

"scatterbrains"

"scattered"

"scatterer"

"scatterers"

"scattering"

"scatters"

"scattersite"

"scattier"

"scattiest"

"scatting"

"scavenge"

"scavenged"

"scavenger"

"scavengers"

"scavengery"

"scavenges"

"scavenging"

"scenario"

"scenarios"

"scenarist"

"scenarists"

"scene"

"sceneries"

"scenery"

"scenes"

"scenic"

"scenically"

"scent"

"scented"

"scenting"

"scentless"

"scents"

"scepter"

"sceptered"

"sceptering"

"scepters"

"sceptic"

"sceptics"

"sceptral"

"sceptre"

"sceptred"

"sceptres"

"sceptring"

"schedular"

"schedule"

"scheduled"

"scheduler"

"schedulers"

"schedules"

"scheduling"

"scheelite"

"schema"

"schemata"

"schematic"

"schematically"

"schematics"

"schematonics"

"scheme"

"schemed"

"schemer"

"schemers"

"schemery"

"schemes"

"scheming"

"scherzi"

"scherzo"

"scherzos"

"schick"

"schilling"

"schillings"

"schism"

"schismatic"

"schismatically"

"schismatics"

"schismatize"

"schismatized"

"schisms"

"schist"

"schistose"

"schistous"

"schists"

"schizo"

"schizoid"

"schizoidism"

"schizoids"

"schizomanic"

"schizophrenia"

"schizophrenic"

"schizophrenics"

"schizos"

"schlemiel"

"schlemiels"

"schlep"

"schlepp"

"schlepped"

"schlepping"

"schlepps"

"schleps"

"schlock"

"schlocks"

"schmaltz"

"schmaltzes"

"schmaltzier"

"schmaltziest"

"schmaltzy"

"schmalz"

"schmalzes"

"schmalzier"

"schmalzy"

"schmeer"

"schmeered"

"schmeering"

"schmeers"

"schmelze"

"schmo"

"schmoe"

"schmoes"

"schmoos"

"schmooze"

"schmoozed"

"schmoozes"

"schmoozing"

"schmuck"

"schmucks"

"schnapps"

"schnaps"

"schnauzer"

"schnauzers"

"schnook"

"schnooks"

"schnozzle"

"scholar"

"scholarliness"

"scholarly"

"scholars"

"scholarship"

"scholarships"

"scholastic"

"scholastically"

"scholastics"

"scholium"

"school"

"schoolbag"

"schoolbook"

"schoolbooks"

"schoolboy"

"schoolboys"

"schoolchild"

"schoolchildren"

"schooldays"

"schooled"

"schoolers"

"schoolfellow"

"schoolfellows"

"schoolgirl"

"schoolgirlish"

"schoolgirls"

"schoolhouse"

"schoolhouses"

"schooling"

"schoolmarm"

"schoolmarms"

"schoolmaster"

"schoolmasters"

"schoolmate"

"schoolmates"

"schoolmistress"

"schoolmistresses"

"schoolroom"

"schoolrooms"

"schools"

"schoolteacher"

"schoolteachers"

"schoolteaching"

"schoolwork"

"schoolyard"

"schoolyards"

"schooner"

"schooners"

"schtick"

"schticks"

"schubert"

"schul"

"schultz"

"schuss"

"schussboomer"

"schussboomers"

"schussed"

"schusses"

"schussing"

"schwa"

"schwas"

"sci"

"sciagraphy"

"sciaphobia"

"sciatic"

"sciatica"

"sciaticas"

"sciatics"

"science"

"science of life"

"sciences"

"scientific"

"scientifically"

"scientist"

"scientistic"

"scientists"

"scil"

"scilicet"

"scimitar"

"scimitars"

"scintilla"

"scintillas"

"scintillate"

"scintillated"

"scintillates"

"scintillating"

"scintillatingly"

"scintillation"

"scintillations"

"scintillator"

"scintillometer"

"scintilloscope"

"scion"

"scions"

"scirocco"

"sciroccos"

"scission"

"scissor"

"scissored"

"scissoring"

"scissors"

"sclera"

"scleral"

"scleras"

"scleroid"

"scleroma"

"sclerometer"

"scleroses"

"sclerosis"

"sclerotic"

"sclerotomy"

"scoff"

"scoffed"

"scoffer"

"scoffers"

"scoffing"

"scoffingly"

"scofflaw"

"scofflaws"

"scoffs"

"scold"

"scolded"

"scolder"

"scolders"

"scolding"

"scoldingly"

"scoldings"

"scolds"

"scoliometer"

"scoliosis"

"scollop"

"scolloped"

"scollops"

"sconce"

"sconced"

"sconces"

"sconcing"

"scone"

"scones"

"scoop"

"scooped"

"scooper"

"scoopers"

"scoopful"

"scoopfuls"

"scooping"

"scoops"

"scoopsful"

"scoot"

"scooted"

"scooter"

"scooters"

"scooting"

"scoots"

"scop"

"scope"

"scopes"

"scoping"

"scopolamine"

"scopophobia"

"scoptophobia"

"scorbutic"

"scorch"

"scorched"

"scorcher"

"scorchers"

"scorches"

"scorching"

"scorchingly"

"score"

"scoreboard"

"scoreboards"

"scorecard"

"scored"

"scorekeeper"

"scoreless"

"scorepad"

"scorepads"

"scorer"

"scorers"

"scores"

"scoria"

"scoriae"

"scorified"

"scorifies"

"scorify"

"scorifying"

"scoring"

"scorn"

"scorned"

"scorner"

"scorners"

"scornful"

"scornfully"

"scorning"

"scorns"

"scorpio"

"scorpion"

"scorpions"

"scorpios"

"scot"

"scotch"

"scotched"

"scotches"

"scotching"

"scotchman"

"scotchmen"

"scotia"

"scotland"

"scotograph"

"scotophobia"

"scotoscope"

"scots"

"scotsman"

"scotsmen"

"scott"

"scottie"

"scotties"

"scottish"

"scoundrel"

"scoundrelly"

"scoundrels"

"scour"

"scoured"

"scourer"

"scourers"

"scourge"

"scourged"

"scourger"

"scourgers"

"scourges"

"scourging"

"scouring"

"scourings"

"scours"

"scout"

"scouted"

"scouter"

"scouters"

"scouting"

"scoutings"

"scoutmaster"

"scoutmasters"

"scouts"

"scow"

"scowed"

"scowl"

"scowled"

"scowler"

"scowlers"

"scowling"

"scowlingly"

"scowls"

"scows"

"scrabble"

"scrabbled"

"scrabbler"

"scrabblers"

"scrabbles"

"scrabbling"

"scrabbly"

"scrag"

"scragged"

"scraggier"

"scraggiest"

"scragging"

"scragglier"

"scraggliest"

"scraggly"

"scraggy"

"scrags"

"scram"

"scramble"

"scrambled"

"scrambler"

"scramblers"

"scrambles"

"scrambling"

"scrammed"

"scramming"

"scrams"

"scrap"

"scrapbook"

"scrapbooks"

"scrape"

"scraped"

"scraper"

"scrapers"

"scrapes"

"scraping"

"scrapings"

"scrappage"

"scrapped"

"scrapper"

"scrappers"

"scrappier"

"scrappiest"

"scrappiness"

"scrapping"

"scrapple"

"scrapples"

"scrappy"

"scraps"

"scratch"

"scratched"

"scratcher"

"scratches"

"scratchier"

"scratchiest"

"scratchily"

"scratchiness"

"scratching"

"scratchpad"

"scratchy"

"scrawl"

"scrawled"

"scrawler"

"scrawlers"

"scrawlier"

"scrawliest"

"scrawling"

"scrawls"

"scrawly"

"scrawnier"

"scrawniest"

"scrawniness"

"scrawny"

"scream"

"screamed"

"screamer"

"screamers"

"screaming"

"screamingly"

"screams"

"scree"

"screech"

"screeched"

"screecher"

"screeches"

"screechier"

"screechiest"

"screeching"

"screechy"

"screed"

"screen"

"Screen Dimmer"

"screened"

"screener"

"screeners"

"screening"

"screenings"

"screenplay"

"screenplays"

"screens"

"screenwriter"

"screes"

"screw"

"screwball"

"screwballs"

"screwdriver"

"screwdrivers"

"screwed"

"screwer"

"screwers"

"screwier"

"screwiest"

"screwing"

"screws"

"screwworm"

"screwy"

"scribal"

"scribble"

"scribbled"

"scribbleomania"

"scribbler"

"scribblers"

"scribbles"

"scribbling"

"scribe"

"scribed"

"scriber"

"scribers"

"scribes"

"scribing"

"scrim"

"scrimmage"

"scrimmaged"

"scrimmages"

"scrimmaging"

"scrimp"

"scrimped"

"scrimpier"

"scrimpiest"

"scrimping"

"scrimps"

"scrimpy"

"scrims"

"scrimshaw"

"scrimshaws"

"scrip"

"scripophily"

"scrips"

"script"

"scripted"

"scripting"

"scripts"

"scriptural"

"scripturally"

"scripture"

"scriptures"

"scriptwriter"

"scrive"

"scrived"

"scrivener"

"scriveners"

"scrivenery"

"scrives"

"scriving"

"scrod"

"scrods"

"scrofula"

"scrofulas"

"scrofulous"

"scroggiest"

"scroll"

"scrolled"

"scrolling"

"scrolls"

"scrollwork"

"scrooge"

"scrooges"

"scrota"

"scrotal"

"scrotum"

"scrotums"

"scrounge"

"scrounged"

"scrounger"

"scroungers"

"scrounges"

"scroungier"

"scrounging"

"scroungy"

"scrub"

"scrubbed"

"scrubber"

"scrubbers"

"scrubbier"

"scrubbiest"

"scrubbing"

"scrubby"

"scrubs"

"scrubwoman"

"scruff"

"scruffier"

"scruffiest"

"scruffs"

"scruffy"

"scrumptious"

"scrumptiously"

"scrumptiousness"

"scrunch"

"scrunched"

"scrunches"

"scrunching"

"scruple"

"scrupled"

"scruples"

"scrupling"

"scrupulosities"

"scrupulosity"

"scrupulous"

"scrupulously"

"scrupulousness"

"scrutable"

"scrutator"

"scrutinies"

"scrutinise"

"scrutinising"

"scrutinize"

"scrutinized"

"scrutinizer"

"scrutinizers"

"scrutinizes"

"scrutinizing"

"scrutinizingly"

"scrutiny"

"scuba"

"scubas"

"scud"

"scudded"

"scudding"

"scuds"

"scuff"

"scuffed"

"scuffing"

"scuffle"

"scuffled"

"scuffler"

"scufflers"

"scuffles"

"scuffling"

"scuffs"

"sculk"

"sculked"

"sculker"

"sculks"

"scull"

"sculled"

"sculler"

"sculleries"

"scullers"

"scullery"

"sculling"

"scullion"

"scullions"

"sculls"

"sculp"

"sculpt"

"sculpted"

"sculpting"

"sculptor"

"sculptors"

"sculptress"

"sculptresses"

"sculpts"

"sculptural"

"sculpture"

"sculptured"

"sculptures"

"sculpturing"

"scum"

"scummers"

"scummier"

"scummiest"

"scumming"

"scummy"

"scums"

"scupper"

"scuppered"

"scuppering"

"scuppers"

"scups"

"scurf"

"scurfier"

"scurfiest"

"scurfs"

"scurfy"

"scurried"

"scurries"

"scurrilities"

"scurrility"

"scurrilous"

"scurrilously"

"scurry"

"scurrying"

"scurvier"

"scurvies"

"scurviest"

"scurvily"

"scurvy"

"scut"

"scuta"

"scutcheon"

"scutcheons"

"scute"

"scuts"

"scuttle"

"scuttlebutt"

"scuttled"

"scuttler"

"scuttles"

"scuttling"

"scythe"

"scythed"

"scythes"

"scything"

"sd"

"se"

"sea"

"seabag"

"seabags"

"seabeaches"

"seabed"

"seabeds"

"seabird"

"seabirds"

"seaboard"

"seaboards"

"seaboots"

"seaborne"

"seacoast"

"seacoasts"

"seacraft"

"seadog"

"seadogs"

"seafarer"

"seafarers"

"seafaring"

"seafloor"

"seafloors"

"seafood"

"seafoods"

"seafowls"

"seafront"

"seafronts"

"seagoing"

"seahorse"

"seakeeping"

"seal"

"sealable"

"sealant"

"sealants"

"sealed"

"sealer"

"sealeries"

"sealers"

"sealery"

"sealing"

"seals"

"sealskin"

"sealskins"

"seam"

"seaman"

"seamanly"

"seamanship"

"seamed"

"seamen"

"seamer"

"seamers"

"seamier"

"seamiest"

"seaminess"

"seaming"

"seamless"

"seamount"

"seamounts"

"seams"

"seamster"

"seamsters"

"seamstress"

"seamstresses"

"seamy"

"seance"

"seances"

"seaplane"

"seaplanes"

"seaport"

"seaports"

"seaquake"

"seaquakes"

"sear"

"search"

"Search app about my health"

"search function"

"searchable"

"searched"

"searcher"

"searchers"

"searches"

"searching"

"searchingly"

"searchings"

"searchlight"

"searchlights"

"seared"

"searer"

"searing"

"sears"

"seas"

"seascape"

"seascapes"

"seascout"

"seascouts"

"seashell"

"seashells"

"seashore"

"seashores"

"seasick"

"seasickness"

"seaside"

"seasider"

"seasides"

"season"

"seasonable"

"seasonableness"

"seasonably"

"seasonal"

"seasonality"

"seasonally"

"seasoned"

"seasoner"

"seasoners"

"seasoning"

"seasonings"

"seasons"

"seat"

"seated"

"seater"

"seaters"

"seating"

"seatings"

"seatless"

"seatmate"

"seatmates"

"seatrain"

"seatrains"

"seats"

"seattle"

"seatwork"

"seawall"

"seawalls"

"seaward"

"seawards"

"seawater"

"seawaters"

"seaway"

"seaways"

"seaweed"

"seaweeds"

"seaworthiness"

"seaworthy"

"sebaceous"

"sebastomania"

"seborrhea"

"seborrhoeic"

"sec"

"secant"

"secants"

"secede"

"seceded"

"seceder"

"seceders"

"secedes"

"seceding"

"secession"

"secessionist"

"secessionists"

"secessions"

"seclude"

"secluded"

"secludedly"

"secludedness"

"secludes"

"secluding"

"seclusion"

"seclusionist"

"seclusive"

"secobarbital"

"seconal"

"second"

"secondaries"

"secondarily"

"secondary"

"seconde"

"seconded"

"seconder"

"seconders"

"secondes"

"secondhand"

"secondines"

"seconding"

"secondly"

"seconds"

"secrecies"

"secrecy"

"secret"

"secretarial"

"secretariat"

"secretariats"

"secretaries"

"secretary"

"secretaryship"

"secretaryships"

"secrete"

"secreted"

"secreter"

"secretes"

"secretest"

"secreting"

"secretion"

"secretions"

"secretive"

"secretively"

"secretiveness"

"secretly"

"secretness"

"secretor"

"secretors"

"secretory"

"secrets"

"secs"

"sect"

"sectarian"

"sectarianism"

"sectarians"

"sectaries"

"sectary"

"sectile"

"sectility"

"section"

"sectional"

"sectionalism"

"sectionally"

"sectioned"

"sectioning"

"sectionize"

"sectionized"

"sectionizing"

"sections"

"sector"

"sectoral"

"sectored"

"sectoring"

"sectors"

"sects"

"secular"

"secularism"

"secularist"

"secularistic"

"secularists"

"secularity"

"secularization"

"secularize"

"secularized"

"secularizer"

"secularizers"

"secularizes"

"secularizing"

"secularly"

"seculars"

"secunda"

"secundines"

"secundogeniture"

"secundum"

"securable"

"securance"

"secure"

"secured"

"securely"

"securement"

"secureness"

"securer"

"securers"

"secures"

"securest"

"securing"

"securities"

"security"

"secus"

"sedan"

"sedans"

"sedate"

"sedated"

"sedately"

"sedateness"

"sedater"

"sedates"

"sedatest"

"sedating"

"sedation"

"sedations"

"sedative"

"sedatives"

"sedentariness"

"sedentary"

"seder"

"seders"

"sedge"

"sedges"

"sedgier"

"sedgy"

"sediment"

"sedimentary"

"sedimentation"

"sedimented"

"sedimentology"

"sediments"

"sedition"

"seditionary"

"seditionist"

"seditionists"

"seditions"

"seditious"

"seditiousness"

"seduce"

"seduceable"

"seduced"

"seducee"

"seducement"

"seducer"

"seducers"

"seduces"

"seducible"

"seducing"

"seducingly"

"seducive"

"seduction"

"seductions"

"seductive"

"seductively"

"seductiveness"

"seductress"

"seductresses"

"sedulous"

"sedulously"

"sedulousness"

"sedum"

"sedums"

"see"

"seeable"

"seed"

"seedbed"

"seedbeds"

"seedcake"

"seedcakes"

"seedcase"

"seedcases"

"seeded"

"seeder"

"seeders"

"seedier"

"seediest"

"seedily"

"seediness"

"seeding"

"seedings"

"seedless"

"seedling"

"seedlings"

"seedman"

"seedmen"

"seedpod"

"seedpods"

"seeds"

"seedsman"

"seedsmen"

"seedtime"

"seedy"

"seeing"

"seeings"

"seek"

"seeker"

"seekers"

"seeking"

"seeks"

"seels"

"seem"

"seemed"

"seemer"

"seemers"

"seeming"

"seemingly"

"seemingness"

"seemings"

"seemlier"

"seemliest"

"seemliness"

"seemly"

"seems"

"seen"

"seep"

"seepage"

"seepages"

"seeped"

"seepier"

"seeping"

"seeps"

"seepy"

"seer"

"seeress"

"seeresses"

"seers"

"seersucker"

"sees"

"seesaw"

"seesawed"

"seesawing"

"seesaws"

"seethe"

"seethed"

"seethes"

"seething"

"seethingly"

"seg"

"segment"

"segmental"

"segmentary"

"segmentation"

"segmented"

"segmenter"

"segmenting"

"segments"

"segno"

"segnos"

"segos"

"segregant"

"segregate"

"segregated"

"segregates"

"segregating"

"segregation"

"segregationist"

"segregationists"

"segregative"

"segue"

"segued"

"segues"

"seguing"

"seidlitz"

"seige"

"seigneur"

"seigneurage"

"seigneurs"

"seignior"

"seigniorage"

"seigniorial"

"seigniors"

"seignorage"

"seignories"

"seignory"

"seine"

"seined"

"seiner"

"seiners"

"seines"

"seining"

"seism"

"seismal"

"seismic"

"seismically"

"seismicity"

"seismism"

"seismisms"

"seismogram"

"seismograms"

"seismograph"

"seismographer"

"seismographers"

"seismographic"

"seismographs"

"seismography"

"seismological"

"seismologist"

"seismologists"

"seismology"

"seismometer"

"seismometers"

"seismometric"

"seismoscope"

"seisms"

"seisure"

"seizable"

"seize"

"seized"

"seizer"

"seizers"

"seizes"

"seizing"

"seizings"

"seizins"

"seizor"

"seizors"

"seizure"

"seizures"

"seldom"

"seldomly"

"seldomness"

"select"

"selected"

"selectee"

"selectees"

"selecting"

"selection"

"selection and administration"

"selectional"

"selections"

"selective"

"selectively"

"selectiveness"

"selectivity"

"selectly"

"selectman"

"selectmen"

"selectness"

"selector"

"selectors"

"selects"

"selectus"

"selenide"

"selenite"

"selenites"

"selenium"

"seleniums"

"selenodesy"

"selenographer"

"selenographers"

"selenography"

"selenology"

"selenoscope"

"selenous"

"self"

"self contained"

"self control"

"self diagnose"

"self hypnosis"

"self infliction"

"self-confidence"

"Self-Hypnosis"

"self-sabotaging"

"Self-Treatment"

"selfdom"

"selfdoms"

"selfed"

"selfesteem"

"selfheal"

"selfheals"

"selfhood"

"selfhoods"

"selfing"

"selfish"

"selfishly"

"selfishness"

"selfless"

"selflessly"

"selflessness"

"selfness"

"selfs"

"selfsame"

"selfward"

"sell"

"sellable"

"seller"

"sellers"

"selling"

"sellout"

"sellouts"

"sells"

"selsyn"

"selsyns"

"seltzer"

"seltzers"

"selvage"

"selvaged"

"selvages"

"selvedge"

"selvedges"

"selves"

"semantic"

"semantical"

"semantically"

"semanticist"

"semanticists"

"semantics"

"semantology"

"semaphore"

"semaphores"

"semasiology"

"semblance"

"semblances"

"sembling"

"semen"

"semens"

"semester"

"semesters"

"semestral"

"semestrial"

"semi"

"semiactive"

"semiagricultural"

"semiannual"

"semiannually"

"semiaquatic"

"semiarid"

"semiautomatic"

"semiautomatically"

"semiautomatics"

"semiautonomous"

"semibiographical"

"semibiographically"

"semicircle"

"semicircles"

"semicircular"

"semicivilized"

"semiclassical"

"semiclassically"

"semicolon"

"semicolons"

"semicomatose"

"semiconducting"

"semiconductor"

"semiconductors"

"semiconscious"

"semiconsciously"

"semiconsciousness"

"semicrystalline"

"semidaily"

"semidependence"

"semidependent"

"semidependently"

"semidesert"

"semideserts"

"semidetached"

"semidivine"

"semidomesticated"

"semidomestication"

"semidry"

"semierect"

"semifictional"

"semifictionally"

"semifinal"

"semifinals"

"semifinished"

"semiformal"

"semiformed"

"semigraphic"

"semigraphics"

"semilegal"

"semilegendary"

"semiliterate"

"semilunar"

"semimature"

"semimonthly"

"semimystical"

"semimythical"

"seminal"

"seminally"

"seminar"

"seminarian"

"seminarians"

"seminaries"

"seminars"

"seminary"

"seminated"

"semination"

"seminole"

"seminoles"

"seminormal"

"seminude"

"seminudity"

"semioblivious"

"semiofficial"

"semiology"

"semiopaque"

"semioses"

"semiosis"

"semiotic"

"semiotics"

"semipermanent"

"semipermeability"

"semipermeable"

"semipetrified"

"semipolitical"

"semiprecious"

"semiprimitive"

"semiprivate"

"semipro"

"semiprofessional"

"semiprofessionally"

"semiprofessionals"

"semipros"

"semipublic"

"semirefined"

"semiresolute"

"semirespectability"

"semirespectable"

"semiretired"

"semiretirement"

"semirigid"

"semirural"

"semis"

"semisacred"

"semisatirical"

"semisatirically"

"semiserious"

"semiskilled"

"semisocialistic"

"semisoft"

"semisolid"

"semisweet"

"semite"

"semites"

"semitic"

"semitism"

"semitist"

"semitists"

"semitone"

"semitones"

"semitraditional"

"semitrailer"

"semitrailers"

"semitranslucent"

"semitransparent"

"semitropical"

"semitruthful"

"semiurban"

"semivoluntary"

"semivowel"

"semivowels"

"semiweekly"

"semiyearly"

"semolina"

"semolinas"

"semper"

"semplice"

"sempre"

"senate"

"senates"

"senator"

"senatorial"

"senatorian"

"senators"

"senatorship"

"send"

"sendable"

"sendee"

"sender"

"senders"

"sending"

"sendoff"

"sendoffs"

"sends"

"seneca"

"senecas"

"senegal"

"senegalese"

"senescence"

"senescent"

"seneschal"

"senhor"

"senhora"

"senhoras"

"senhores"

"senhors"

"senile"

"senilely"

"seniles"

"senilities"

"senility"

"senior"

"seniorities"

"seniority"

"seniors"

"senna"

"sennas"

"sennets"

"sennits"

"senor"

"senora"

"senoras"

"senores"

"senorita"

"senoritas"

"senors"

"sensate"

"sensated"

"sensates"

"sensating"

"sensation"

"sensational"

"sensationalism"

"sensationalist"

"sensationalists"

"sensationally"

"sensations"

"sense"

"sensed"

"senseful"

"senseless"

"senselessly"

"senselessness"

"senses"

"sensibilities"

"sensibility"

"sensible"

"sensibleness"

"sensibler"

"sensibles"

"sensiblest"

"sensibly"

"sensing"

"sensitive"

"sensitively"

"sensitiveness"

"sensitivities"

"sensitivity"

"sensitization"

"sensitize"

"sensitized"

"sensitizes"

"sensitizing"

"sensitometer"

"sensitometers"

"sensitometric"

"sensor"

"sensoria"

"sensorial"

"sensorially"

"sensorimotor"

"sensorium"

"sensoriums"

"sensors"

"sensory"

"sensu"

"sensual"

"sensualism"

"sensualist"

"sensualistic"

"sensualists"

"sensualities"

"sensuality"

"sensualization"

"sensualize"

"sensually"

"sensualness"

"sensuous"

"sensuously"

"sensuousness"

"sent"

"sentence"

"sentenced"

"sentences"

"sentencing"

"sententious"

"sententiously"

"sententiousness"

"senti"

"sentient"

"sentiently"

"sentients"

"sentiment"

"sentimental"

"sentimentalism"

"sentimentalist"

"sentimentalists"

"sentimentality"

"sentimentalization"

"sentimentalize"

"sentimentalized"

"sentimentalizes"

"sentimentalizing"

"sentimentally"

"sentiments"

"sentinel"

"sentineled"

"sentinels"

"sentried"

"sentries"

"sentry"

"sentrying"

"seoul"

"sepal"

"sepaled"

"sepalled"

"sepaloid"

"sepalous"

"sepals"

"separability"

"separable"

"separableness"

"separably"

"separate"

"separated"

"separately"

"separateness"

"separates"

"separating"

"separation"

"separations"

"separatism"

"separatist"

"separatists"

"separative"

"separator"

"separators"

"sepia"

"sepias"

"sepometer"

"sepoy"

"seppuku"

"seppukus"

"sepsis"

"sept"

"septa"

"septal"

"septaugintal"

"september"

"septet"

"septets"

"septette"

"septettes"

"septic"

"septical"

"septicemia"

"septics"

"septime"

"septimes"

"septs"

"septuagenarian"

"septuagenarians"

"septum"

"septums"

"septuple"

"septupled"

"septuples"

"septuplet"

"septupling"

"sepulcher"

"sepulchered"

"sepulchering"

"sepulchers"

"sepulchral"

"sepulchrally"

"sepulchre"

"sepulture"

"seq"

"sequel"

"sequelae"

"sequels"

"sequence"

"sequenced"

"sequencer"

"sequences"

"sequencies"

"sequencing"

"sequencings"

"sequency"

"sequent"

"sequential"

"sequentiality"

"sequentially"

"sequents"

"sequester"

"sequestered"

"sequestering"

"sequesters"

"sequestrable"

"sequestrate"

"sequestrated"

"sequestrates"

"sequestrating"

"sequestration"

"sequestrations"

"sequestrator"

"sequestratrices"

"sequestratrix"

"sequin"

"sequined"

"sequinned"

"sequins"

"sequitur"

"sequiturs"

"sequoia"

"sequoias"

"sera"

"seraglio"

"seraglios"

"seral"

"serape"

"serapes"

"seraph"

"seraphic"

"seraphically"

"seraphim"

"seraphims"

"seraphs"

"serb"

"serbia"

"serbian"

"serbians"

"sere"

"sered"

"serenade"

"serenaded"

"serenader"

"serenaders"

"serenades"

"serenading"

"serendipitous"

"serendipity"

"serene"

"serenely"

"sereneness"

"serener"

"serenes"

"serenest"

"serenities"

"serenity"

"serer"

"seres"

"serest"

"serf"

"serfage"

"serfages"

"serfdom"

"serfdoms"

"serfhood"

"serfhoods"

"serfish"

"serfs"

"serge"

"sergeancies"

"sergeancy"

"sergeant"

"sergeantcies"

"sergeantcy"

"sergeants"

"sergeantship"

"sergeantships"

"serges"

"serging"

"sergings"

"serial"

"serialist"

"serialists"

"seriality"

"serialization"

"serializations"

"serialize"

"serialized"

"serializes"

"serializing"

"serially"

"serials"

"seriated"

"seriates"

"seriatim"

"seriating"

"seriation"

"series"

"serif"

"serifs"

"serigraph"

"serigrapher"

"serigraphers"

"serigraphs"

"serigraphy"

"serimeter"

"serin"

"serine"

"sering"

"serins"

"serious"

"seriously"

"seriousness"

"sermon"

"sermonic"

"sermonize"

"sermonized"

"sermonizer"

"sermonizes"

"sermonizing"

"sermons"

"serologic"

"serological"

"serologically"

"serology"

"serotonin"

"serotype"

"serotypes"

"serous"

"serow"

"serpent"

"serpentine"

"serpents"

"serrate"

"serrated"

"serrates"

"serrating"

"serration"

"serried"

"serries"

"serrying"

"serum"

"serum levels"

"serumal"

"serums"

"servable"

"serval"

"servals"

"servant"

"servants"

"servantship"

"serve"

"served"

"server"

"servers"

"serves"

"service"

"serviceability"

"serviceable"

"serviceableness"

"serviceably"

"serviced"

"serviceman"

"servicemen"

"servicer"

"servicers"

"services"

"servicewoman"

"servicewomen"

"servicing"

"serviette"

"serviettes"

"servile"

"servilely"

"servilities"

"servility"

"serving"

"servings"

"servitor"

"servitors"

"servitude"

"servo"

"servomechanism"

"servomechanisms"

"servomotor"

"servomotors"

"servos"

"sesame"

"sesames"

"sesquicentennial"

"sesquicentennially"

"sesquicentennials"

"sesquipedalian"

"sessile"

"session"

"sessional"

"sessions"

"sesterce"

"sesterces"

"sestet"

"sestets"

"sestina"

"sestinas"

"sestine"

"sestines"

"set"

"setae"

"setal"

"setback"

"setbacks"

"setlines"

"setoff"

"setoffs"

"seton"

"setons"

"setout"

"sets"

"setscrew"

"setscrews"

"settee"

"settees"

"setter"

"setters"

"setting"

"settings"

"settle"

"settleability"

"settled"

"settlement"

"settlements"

"settler"

"settlers"

"settles"

"settling"

"settlings"

"setup"

"setups"

"seven"

"sevens"

"seventeen"

"seventeens"

"seventeenth"

"seventeenths"

"seventh"

"sevenths"

"seventies"

"seventieth"

"seventieths"

"seventy"

"sever"

"severability"

"severable"

"several"

"severalized"

"severalizing"

"severally"

"severals"

"severalties"

"severance"

"severation"

"severe"

"severed"

"severely"

"severeness"

"severer"

"severers"

"severest"

"severing"

"severities"

"severity"

"severs"

"seville"

"sew"

"sewage"

"sewages"

"sewed"

"sewer"

"sewerage"

"sewerages"

"sewers"

"sewing"

"sewings"

"sewn"

"sews"

"sex"

"sex drive"

"sexagenarian"

"sexagenarians"

"sexed"

"sexes"

"sexier"

"sexiest"

"sexily"

"sexiness"

"sexing"

"sexism"

"sexisms"

"sexist"

"sexists"

"sexless"

"sexlessly"

"sexlessness"

"sexological"

"sexologies"

"sexologist"

"sexology"

"sexpot"

"sexpots"

"sextan"

"sextant"

"sextants"

"sextet"

"sextets"

"sextette"

"sextettes"

"sextile"

"sextiles"

"sexto"

"sexton"

"sextons"

"sextos"

"sexts"

"sextuple"

"sextupled"

"sextuples"

"sextuplet"

"sextuplets"

"sextupling"

"sextuply"

"sexual"

"sexualities"

"sexuality"

"sexualization"

"sexualize"

"sexualized"

"sexualizing"

"sexually"

"sexy"

"sforzato"

"sforzatos"

"sh"

"shabbier"

"shabbiest"

"shabbily"

"shabbiness"

"shabby"

"shack"

"shacked"

"shacker"

"shacking"

"shackle"

"shackled"

"shackler"

"shacklers"

"shackles"

"shackling"

"shacks"

"shad"

"shade"

"shaded"

"shadeless"

"shader"

"shaders"

"shades"

"shadier"

"shadiest"

"shadily"

"shadiness"

"shading"

"shadings"

"shadow"

"shadowbox"

"shadowboxed"

"shadowboxes"

"shadowboxing"

"shadowed"

"shadower"

"shadowers"

"shadowgraph"

"shadowier"

"shadowiest"

"shadowiness"

"shadowing"

"shadowless"

"shadows"

"shadowy"

"shads"

"shady"

"shaft"

"shafted"

"shafting"

"shaftings"

"shafts"

"shag"

"shagbark"

"shagbarks"

"shagged"

"shaggier"

"shaggiest"

"shaggily"

"shagginess"

"shagging"

"shaggy"

"shagreen"

"shags"

"shah"

"shahdom"

"shahdoms"

"shahs"

"shaitan"

"shaitans"

"shakable"

"shake"

"shakeable"

"shakedown"

"shakedowns"

"shaken"

"shakeout"

"shakeouts"

"shaker"

"shakers"

"shakes"

"shakespeare"

"shakespearean"

"shakespeareans"

"shakeup"

"shakeups"

"shakier"

"shakiest"

"shakily"

"shakiness"

"shaking"

"shako"

"shakoes"

"shakos"

"shaky"

"shale"

"shaled"

"shales"

"shalier"

"shall"

"shallop"

"shallops"

"shallot"

"shallots"

"shallow"

"shallowed"

"shallower"

"shallowest"

"shallowing"

"shallowness"

"shallows"

"shalom"

"shalt"

"shaly"

"sham"

"shamable"

"shaman"

"shamanic"

"shamans"

"shamble"

"shambled"

"shambles"

"shambling"

"shame"

"shamed"

"shamefaced"

"shamefacedly"

"shamefacedness"

"shameful"

"shamefully"

"shamefulness"

"shameless"

"shamelessly"

"shamelessness"

"shames"

"shaming"

"shammed"

"shammer"

"shammers"

"shammes"

"shammied"

"shammies"

"shamming"

"shammy"

"shamois"

"shampoo"

"shampooed"

"shampooer"

"shampooers"

"shampooing"

"shampoos"

"shamrock"

"shamrocks"

"shams"

"shamus"

"shamuses"

"shandies"

"shanghai"

"shanghaied"

"shanghaiing"

"shanghais"

"shank"

"shanked"

"shanking"

"shanks"

"shantey"

"shanteys"

"shanti"

"shanties"

"shantis"

"shantung"

"shanty"

"shapable"

"shape"

"shapeable"

"shaped"

"shapeless"

"shapelessly"

"shapelessness"

"shapelier"

"shapeliest"

"shapeliness"

"shapely"

"shaper"

"shapers"

"shapes"

"shapeup"

"shapeups"

"shaping"

"sharable"

"shard"

"shards"

"share"

"shareability"

"shareable"

"sharecrop"

"sharecropped"

"sharecropper"

"sharecroppers"

"sharecropping"

"sharecrops"

"shared"

"shareholder"

"shareholders"

"shareowner"

"sharer"

"sharers"

"shares"

"sharesman"

"sharesmen"

"sharif"

"sharifs"

"sharing"

"sharing a peaceful moment"

"shark"

"sharked"

"sharker"

"sharkers"

"sharking"

"sharks"

"sharkskin"

"sharkskins"

"sharp"

"sharped"

"sharpen"

"sharpened"

"sharpener"

"sharpeners"

"sharpening"

"sharpens"

"sharper"

"sharpers"

"sharpest"

"sharpie"

"sharpies"

"sharping"

"sharply"

"sharpness"

"sharps"

"sharpshooter"

"sharpshooters"

"sharpshooting"

"sharpy"

"shashlik"

"shat"

"shatter"

"shattered"

"shattering"

"shatteringly"

"shatterproof"

"shatters"

"shavable"

"shave"

"shaveable"

"shaved"

"shaven"

"shaver"

"shavers"

"shaves"

"shavians"

"shaving"

"shavings"

"shawed"

"shawl"

"shawled"

"shawling"

"shawls"

"shawm"

"shawms"

"shawn"

"shawnee"

"shawnees"

"shaws"

"shay"

"shays"

"she"

"sheaf"

"sheafed"

"sheafing"

"sheafs"

"shear"

"sheared"

"shearer"

"shearers"

"shearing"

"shears"

"sheath"

"sheathe"

"sheathed"

"sheather"

"sheathers"

"sheathes"

"sheathing"

"sheaths"

"sheave"

"sheaved"

"sheaves"

"sheaving"

"shebang"

"shebangs"

"shebeen"

"shebeens"

"shed"

"shedable"

"shedded"

"shedder"

"shedders"

"shedding"

"sheds"

"sheen"

"sheened"

"sheeney"

"sheeneys"

"sheenful"

"sheenie"

"sheenier"

"sheenies"

"sheeniest"

"sheening"

"sheens"

"sheeny"

"sheep"

"sheepdog"

"sheepdogs"

"sheepfold"

"sheepfolds"

"sheepherder"

"sheepherding"

"sheepish"

"sheepishly"

"sheepishness"

"sheepman"

"sheepmen"

"sheepshank"

"sheepshearer"

"sheepshearing"

"sheepskin"

"sheepskins"

"sheer"

"sheered"

"sheerer"

"sheerest"

"sheering"

"sheerly"

"sheerness"

"sheers"

"sheet"

"sheeted"

"sheeter"

"sheeters"

"sheetfed"

"sheeting"

"sheetrock"

"sheets"

"shegetz"

"sheik"

"sheikdom"

"sheikdoms"

"sheikh"

"sheikhs"

"sheiks"

"sheila"

"shekel"

"shekels"

"shelf"

"shelfful"

"shelffuls"

"shell"

"shellac"

"shellack"

"shellacked"

"shellacker"

"shellackers"

"shellacking"

"shellackings"

"shellacks"

"shellacs"

"shelled"

"sheller"

"shellers"

"shelley"

"shellfire"

"shellfish"

"shellfishes"

"shellier"

"shelling"

"shells"

"shelly"

"shelter"

"sheltered"

"shelterer"

"sheltering"

"shelterless"

"shelters"

"shelties"

"shelve"

"shelved"

"shelver"

"shelvers"

"shelves"

"shelvier"

"shelviest"

"shelving"

"shelvings"

"shelvy"

"shenanigan"

"shenanigans"

"sheol"

"sheols"

"shepherd"

"shepherded"

"shepherdess"

"shepherdesses"

"shepherding"

"shepherds"

"sherbert"

"sherberts"

"sherbet"

"sherbets"

"sherd"

"sherds"

"sherif"

"sheriff"

"sheriffalty"

"sheriffcies"

"sheriffdom"

"sheriffs"

"sherifs"

"sherlock"

"sherlocks"

"sherpa"

"sherpas"

"sherries"

"sherry"

"shes"

"shetland"

"shetlands"

"shew"

"shewed"

"shewer"

"shewers"

"shewing"

"shewn"

"shews"

"shiatsu"

"shibboleth"

"shibboleths"

"shicksa"

"shicksas"

"shied"

"shield"

"shielded"

"shielder"

"shielders"

"shielding"

"shields"

"shier"

"shiers"

"shies"

"shiest"

"shift"

"shiftability"

"shiftable"

"shifted"

"shifter"

"shifters"

"shiftier"

"shiftiest"

"shiftily"

"shiftiness"

"shifting"

"shiftless"

"shiftlessly"

"shiftlessness"

"shifts"

"shifty"

"shikaris"

"shiksa"

"shiksas"

"shikses"

"shill"

"shilled"

"shillelagh"

"shillelaghs"

"shilling"

"shillings"

"shills"

"shily"

"shim"

"shimmed"

"shimmer"

"shimmered"

"shimmering"

"shimmeringly"

"shimmers"

"shimmery"

"shimmied"

"shimmies"

"shimming"

"shimmy"

"shimmying"

"shims"

"shin"

"shinbone"

"shinbones"

"shindies"

"shindig"

"shindigs"

"shindy"

"shindys"

"shine"

"shined"

"shiner"

"shiners"

"shines"

"shingle"

"shingled"

"shingler"

"shinglers"

"shingles"

"shingling"

"shingly"

"shinier"

"shiniest"

"shinily"

"shininess"

"shining"

"shiningly"

"shinleaf"

"shinleafs"

"shinned"

"shinney"

"shinnied"

"shinnies"

"shinning"

"shinny"

"shinnying"

"shins"

"shinsplints"

"shinto"

"shintoism"

"shintoist"

"shintoists"

"shiny"

"ship"

"shipboard"

"shipbuilder"

"shipbuilders"

"shipbuilding"

"shipkeeper"

"shipload"

"shiploads"

"shipman"

"shipmaster"

"shipmate"

"shipmates"

"shipmen"

"shipment"

"shipments"

"shipowner"

"shippable"

"shippage"

"shipped"

"shipper"

"shippers"

"shipping"

"shippings"

"ships"

"shipshape"

"shipside"

"shipt"

"shipway"

"shipways"

"shipworm"

"shipworms"

"shipwreck"

"shipwrecked"

"shipwrecking"

"shipwrecks"

"shipwright"

"shipwrights"

"shipyard"

"shipyards"

"shire"

"shires"

"shirk"

"shirked"

"shirker"

"shirkers"

"shirking"

"shirks"

"shirley"

"shirr"

"shirred"

"shirring"

"shirrings"

"shirrs"

"shirt"

"shirtfront"

"shirtier"

"shirtiest"

"shirting"

"shirtings"

"shirtmaker"

"shirts"

"shirtsleeve"

"shirttail"

"shirtwaist"

"shirty"

"shish"

"shist"

"shists"

"shit"

"shits"

"shitted"

"shitting"

"shiv"

"shiva"

"shivah"

"shivaree"

"shivareed"

"shivarees"

"shivas"

"shive"

"shiver"

"shivered"

"shiverer"

"shiverers"

"shivering"

"shiveringly"

"shivers"

"shivery"

"shivs"

"shlemiel"

"shlep"

"shlock"

"shlocks"

"shmo"

"shmoes"

"shnaps"

"shoal"

"shoaled"

"shoaler"

"shoalier"

"shoaliest"

"shoaling"

"shoals"

"shoaly"

"shoat"

"shoats"

"shock"

"shocked"

"shocker"

"shockers"

"shocking"

"shockingly"

"shockproof"

"shocks"

"shockwave"

"shod"

"shodden"

"shoddier"

"shoddies"

"shoddiest"

"shoddily"

"shoddiness"

"shoddy"

"shoe"

"shoeblack"

"shoed"

"shoehorn"

"shoehorned"

"shoehorns"

"shoeing"

"shoelace"

"shoelaces"

"shoemaker"

"shoemakers"

"shoer"

"shoers"

"shoes"

"shoestring"

"shoestrings"

"shoetree"

"shoetrees"

"shogged"

"shogun"

"shogunal"

"shoguns"

"shoji"

"shojis"

"sholom"

"shone"

"shoo"

"shooed"

"shooflies"

"shoofly"

"shooing"

"shook"

"shooks"

"shoos"

"shoot"

"shooter"

"shooters"

"shooting"

"shootings"

"shootout"

"shootouts"

"shoots"

"shop"

"shopboy"

"shopboys"

"shopbreaker"

"shope"

"shopgirl"

"shopgirls"

"shopkeeper"

"shopkeepers"

"shoplift"

"shoplifted"

"shoplifter"

"shoplifters"

"shoplifting"

"shoplifts"

"shopman"

"shopmen"

"shoppe"

"shopped"

"shopper"

"shoppers"

"shoppes"

"shopping"

"shoppings"

"shops"

"shoptalk"

"shoptalks"

"shopworn"

"shore"

"shorebird"

"shorebirds"

"shored"

"shoreless"

"shoreline"

"shorelines"

"shores"

"shoring"

"shorings"

"shorn"

"short"

"shortage"

"shortages"

"shortbread"

"shortcake"

"shortcakes"

"shortchange"

"shortchanged"

"shortchanges"

"shortchanging"

"shortcoming"

"shortcomings"

"shortcut"

"shortcuts"

"shorted"

"shorten"

"shortened"

"shortener"

"shorteners"

"shortening"

"shortenings"

"shortens"

"shorter"

"shortest"

"shortfall"

"shortfalls"

"shorthand"

"shorthanded"

"shorthorn"

"shorthorns"

"shortie"

"shorties"

"shorting"

"shortish"

"shortly"

"shortness"

"shorts"

"shortsighted"

"shortsightedly"

"shortsightedness"

"shortstop"

"shortstops"

"shortterm"

"shortwave"

"shortwaves"

"shorty"

"shoshone"

"shoshonean"

"shoshonis"

"shot"

"shote"

"shotes"

"shotgun"

"shotgunned"

"shotguns"

"shots"

"shotted"

"shotting"

"should"

"shoulder"

"shouldered"

"shouldering"

"shoulders"

"shouldst"

"shout"

"shouted"

"shouter"

"shouters"

"shouting"

"shouts"

"shove"

"shoved"

"shovel"

"shoveled"

"shoveler"

"shovelers"

"shovelful"

"shovelfuls"

"shovelhead"

"shoveling"

"shovelled"

"shoveller"

"shovelling"

"shovelman"

"shovels"

"shovelsful"

"shover"

"shovers"

"shoves"

"shoving"

"show"

"showboat"

"showboats"

"showcase"

"showcased"

"showcases"

"showcasing"

"showdown"

"showdowns"

"showed"

"shower"

"showered"

"showerhead"

"showering"

"showers"

"showery"

"showgirl"

"showgirls"

"showier"

"showiest"

"showily"

"showiness"

"showing"

"showings"

"showman"

"showmanship"

"showmen"

"shown"

"showoff"

"showoffs"

"showpiece"

"showpieces"

"showplace"

"showplaces"

"showroom"

"showrooms"

"shows"

"showup"

"showy"

"shrank"

"shrapnel"

"shred"

"shredded"

"shredder"

"shredders"

"shredding"

"shreds"

"shreveport"

"shrew"

"shrewd"

"shrewder"

"shrewdest"

"shrewdly"

"shrewdness"

"shrewed"

"shrewing"

"shrewish"

"shrewishness"

"shrews"

"shriek"

"shrieked"

"shrieker"

"shriekers"

"shriekier"

"shriekiest"

"shrieking"

"shrieks"

"shrieky"

"shrift"

"shrifts"

"shrike"

"shrikes"

"shrill"

"shrilled"

"shriller"

"shrillest"

"shrilling"

"shrillness"

"shrills"

"shrilly"

"shrimp"

"shrimped"

"shrimper"

"shrimpers"

"shrimpier"

"shrimpiest"

"shrimping"

"shrimps"

"shrimpy"

"shrine"

"shrined"

"shrines"

"shrining"

"shrink"

"shrinkable"

"shrinkage"

"shrinkages"

"shrinker"

"shrinkers"

"shrinking"

"shrinks"

"shrive"

"shrived"

"shrivel"

"shriveled"

"shriveling"

"shrivelled"

"shrivelling"

"shrivels"

"shriven"

"shriver"

"shrivers"

"shrives"

"shriving"

"shroud"

"shrouded"

"shrouding"

"shrouds"

"shrove"

"shrub"

"shrubberies"

"shrubbery"

"shrubbier"

"shrubbiest"

"shrubby"

"shrubs"

"shrug"

"shrugged"

"shrugging"

"shrugs"

"shrunk"

"shrunken"

"shtetel"

"shtetl"

"shtick"

"shticks"

"shuck"

"shucked"

"shucker"

"shuckers"

"shucking"

"shuckings"

"shucks"

"shudder"

"shuddered"

"shuddering"

"shudderingly"

"shudders"

"shuddery"

"shuffle"

"shuffleboard"

"shuffled"

"shuffler"

"shufflers"

"shuffles"

"shuffling"

"shuftiscope"

"shul"

"shuls"

"shun"

"shunned"

"shunner"

"shunners"

"shunning"

"shunpike"

"shunpiked"

"shunpiker"

"shunpikers"

"shunpikes"

"shunpiking"

"shuns"

"shunt"

"shunted"

"shunter"

"shunters"

"shunting"

"shunts"

"shush"

"shushed"

"shushes"

"shushing"

"shut"

"shutdown"

"shutdowns"

"shute"

"shuted"

"shutes"

"shuteye"

"shuteyes"

"shuting"

"shutoff"

"shutoffs"

"shutout"

"shutouts"

"shuts"

"shutter"

"shutterbug"

"shutterbugs"

"shuttered"

"shuttering"

"shutters"

"shutting"

"shuttle"

"shuttlecock"

"shuttlecocks"

"shuttled"

"shuttles"

"shuttling"

"shy"

"shyer"

"shyers"

"shyest"

"shying"

"shylock"

"shylocked"

"shylocking"

"shylocks"

"shyly"

"shyness"

"shynesses"

"shyster"

"shysters"

"si"

"siam"

"siamese"

"siameses"

"sian"

"sib"

"siberia"

"siberian"

"siberians"

"sibilance"

"sibilant"

"sibilantly"

"sibilants"

"sibilate"

"sibilated"

"sibilates"

"sibilating"

"sibilation"

"sibling"

"siblings"

"sibs"

"sibyl"

"sibylic"

"sibyllic"

"sibylline"

"sibyls"

"sic"

"sicced"

"siccimeter"

"siccing"

"sicilian"

"sicilians"

"sicily"

"sick"

"sick symptom"

"sickbay"

"sickbays"

"sickbed"

"sickbeds"

"sicked"

"sicken"

"sickened"

"sickener"

"sickeners"

"sickening"

"sickeningly"

"sickens"

"sicker"

"sickest"

"sicking"

"sickish"

"sickle"

"sickled"

"sickles"

"sicklier"

"sickliest"

"sicklily"

"sickliness"

"sickling"

"sickly"

"sickness"

"sicknesses"

"sickout"

"sickouts"

"sickroom"

"sickrooms"

"sicks"

"sics"

"side"

"side effect"

"side effects"

"sidearm"

"sidearms"

"sideband"

"sidebands"

"sideboard"

"sideboards"

"sideburn"

"sideburns"

"sidecar"

"sidecars"

"sidechairs"

"sided"

"sidedness"

"sidehill"

"sidekick"

"sidekicks"

"sidelight"

"sidelights"

"sideline"

"sidelined"

"sideliner"

"sidelines"

"sidelining"

"sidelong"

"sideman"

"sidemen"

"sidepiece"

"sidepieces"

"sidereal"

"siderite"

"siderography"

"sideroscope"

"sides"

"sidesaddle"

"sidesaddles"

"sideshow"

"sideshows"

"sideslip"

"sideslipped"

"sideslipping"

"sideslips"

"sidespin"

"sidesplitting"

"sidestep"

"sidestepped"

"sidestepper"

"sidesteppers"

"sidestepping"

"sidesteps"

"sidestroke"

"sidestrokes"

"sideswipe"

"sideswiped"

"sideswiper"

"sideswipers"

"sideswipes"

"sideswiping"

"sidetrack"

"sidetracked"

"sidetracking"

"sidetracks"

"sidewalk"

"sidewalks"

"sidewall"

"sidewalls"

"sideward"

"sideway"

"sideways"

"sidewinder"

"sidewinders"

"sidewise"

"siding"

"sidings"

"sidle"

"sidled"

"sidler"

"sidlers"

"sidles"

"sidling"

"sidlingly"

"sidney"

"siecle"

"siege"

"sieged"

"sieges"

"sieging"

"siemens"

"sienna"

"siennas"

"sierra"

"sierran"

"sierras"

"siesta"

"siestas"

"sieur"

"sieurs"

"sieve"

"sieved"

"sieves"

"sieving"

"sift"

"sifted"

"sifter"

"sifters"

"sifting"

"siftings"

"sifts"

"sigh"

"sighed"

"sigher"

"sighers"

"sighing"

"sighs"

"sight"

"sighted"

"sighter"

"sighters"

"sighting"

"sightings"

"sightless"

"sightlessness"

"sightlier"

"sightliest"

"sightliness"

"sightly"

"sights"

"sightsaw"

"sightsee"

"sightseeing"

"sightseen"

"sightseer"

"sightseers"

"sightsees"

"sigil"

"sigillography"

"sigils"

"siglos"

"sigma"

"sigmas"

"sigmoid"

"sigmoidal"

"sigmoidoscope"

"sigmoids"

"sign"

"signable"

"signal"

"signaled"

"signaler"

"signalers"

"signaling"

"signalization"

"signalize"

"signalized"

"signalizes"

"signalizing"

"signalled"

"signaller"

"signalling"

"signally"

"signalman"

"signalmen"

"signals"

"signatary"

"signatories"

"signatory"

"signatural"

"signature"

"signatured"

"signatureless"

"signatures"

"signboard"

"signboards"

"signed"

"signee"

"signer"

"signers"

"signet"

"signeted"

"signets"

"significance"

"significant"

"significantly"

"significate"

"signification"

"significations"

"significs"

"signified"

"signifier"

"signifies"

"signify"

"signifying"

"signing"

"signiori"

"signiories"

"signiors"

"signiory"

"signor"

"signora"

"signoras"

"signore"

"signori"

"signories"

"signorina"

"signorinas"

"signorine"

"signors"

"signory"

"signpost"

"signposted"

"signposts"

"signs"

"sikh"

"sikhism"

"sikhs"

"silage"

"silages"

"silence"

"silenced"

"silencer"

"silencers"

"silences"

"silencing"

"silent"

"silenter"

"silentest"

"silently"

"silentness"

"silents"

"silesia"

"silex"

"silhouette"

"silhouetted"

"silhouettes"

"silhouetting"

"silica"

"silicas"

"silicate"

"silicates"

"siliceous"

"silicon"

"silicone"

"silicones"

"silicons"

"silicoses"

"silicosis"

"silk"

"silked"

"silken"

"silkier"

"silkiest"

"silkily"

"silkiness"

"silking"

"silks"

"silkscreen"

"silkscreened"

"silkscreening"

"silkscreens"

"silkweed"

"silkworm"

"silkworms"

"silky"

"sill"

"sillers"

"sillier"

"sillies"

"silliest"

"sillily"

"silliness"

"sillometer"

"sills"

"silly"

"silo"

"siloed"

"siloing"

"silos"

"silt"

"siltation"

"silted"

"siltier"

"siltiest"

"silting"

"silts"

"silty"

"silurian"

"silva"

"silvan"

"silvans"

"silvas"

"silver"

"silvered"

"silverer"

"silverers"

"silverfish"

"silverfishes"

"silveriness"

"silvering"

"silvern"

"silvers"

"silversmith"

"silversmiths"

"silverware"

"silvery"

"silvester"

"silvics"

"simian"

"simians"

"similar"

"similarities"

"similarity"

"similarly"

"simile"

"similes"

"similiter"

"similitude"

"simitar"

"simmer"

"simmered"

"simmering"

"simmers"

"simoleon"

"simoleons"

"simon"

"simoniac"

"simoniacs"

"simonies"

"simonist"

"simonists"

"simonize"

"simonized"

"simonizes"

"simonizing"

"simony"

"simp"

"simpatico"

"simper"

"simpered"

"simperer"

"simperers"

"simpering"

"simperingly"

"simpers"

"simple"

"simpleminded"

"simplemindedly"

"simplemindedness"

"simpleness"

"simpler"

"simples"

"simplest"

"simpleton"

"simpletons"

"simplex"

"simplexes"

"simplices"

"simplicities"

"simplicity"

"simplification"

"simplifications"

"simplified"

"simplifier"

"simplifiers"

"simplifies"

"simplify"

"simplifying"

"simplism"

"simplisms"

"simplistic"

"simplistically"

"simply"

"simps"

"simulant"

"simulants"

"simulate"

"simulated"

"simulates"

"simulating"

"simulation"

"simulations"

"simulative"

"simulator"

"simulators"

"simulcast"

"simulcasting"

"simulcasts"

"simultaneity"

"simultaneous"

"simultaneously"

"simultaneousness"

"sin"

"sinatra"

"since"

"sincere"

"sincerely"

"sincerer"

"sincerest"

"sincerity"

"sindonology"

"sine"

"sinecure"

"sinecures"

"sines"

"sinew"

"sinewed"

"sinewing"

"sinews"

"sinewy"

"sinfonia"

"sinful"

"sinfully"

"sinfulness"

"sing"

"singable"

"singapore"

"singe"

"singed"

"singeing"

"singer"

"singers"

"singes"

"singhalese"

"singillatim"

"singing"

"single"

"singled"

"singlehandedly"

"singleness"

"singles"

"singlet"

"singleton"

"singletons"

"singletree"

"singletrees"

"singlets"

"singling"

"singly"

"sings"

"singsong"

"singsongs"

"singular"

"singularities"

"singularity"

"singularly"

"singulars"

"sinh"

"sinhalese"

"sinhs"

"sinicize"

"sinicized"

"sinicizes"

"sinicizing"

"sinister"

"sinisterly"

"sinistrality"

"sinistrally"

"sink"

"sinkable"

"sinkage"

"sinkages"

"sinker"

"sinkers"

"sinkhole"

"sinkholes"

"sinking"

"sinks"

"sinless"

"sinlessly"

"sinlessness"

"sinned"

"sinner"

"sinners"

"sinning"

"sinologies"

"Sinology"

"sins"

"sinter"

"sintered"

"sintering"

"sinters"

"sinuate"

"sinuated"

"sinuates"

"sinuating"

"sinuosity"

"sinuous"

"sinuously"

"sinus"

"sinuses"

"sinusitis"

"sinusoid"

"sinusoidally"

"sinusoids"

"sioux"

"sip"

"siphon"

"siphonage"

"siphonal"

"siphoned"

"siphonic"

"siphoning"

"siphons"

"sipped"

"sipper"

"sippers"

"sippets"

"sipping"

"sippy"

"sips"

"sir"

"sire"

"sired"

"siree"

"sireless"

"siren"

"sirenomelus"

"sirens"

"sires"

"siring"

"sirloin"

"sirloins"

"sirocco"

"siroccos"

"sirrah"

"sirrahs"

"sirree"

"sirrees"

"sirs"

"sirup"

"sirups"

"sirupy"

"sis"

"sisal"

"sisals"

"sissier"

"sissies"

"sissified"

"sissy"

"sissyish"

"sister"

"sistered"

"sisterhood"

"sisterhoods"

"sistering"

"sisterly"

"sisters"

"sistrum"

"sistrums"

"sisyphus"

"sit"

"sit-up"

"sitar"

"sitarist"

"sitarists"

"sitars"

"sitcom"

"sitcoms"

"site"

"sited"

"sites"

"siting"

"sitiomania"

"sitiophobia"

"sitology"

"sitophobia"

"sits"

"sitter"

"sitters"

"sitting"

"sittings"

"situ"

"situate"

"situated"

"situates"

"situating"

"situation"

"situational"

"situations"

"situp"

"situps"

"situs"

"sitz"

"sitzmark"

"sitzmarks"

"six"

"sixes"

"sixfold"

"sixing"

"sixpence"

"sixpences"

"sixpenny"

"sixte"

"sixteen"

"sixteens"

"sixteenth"

"sixteenths"

"sixtes"

"sixth"

"sixthly"

"sixths"

"sixties"

"sixtieth"

"sixtieths"

"sixty"

"sizable"

"sizably"

"size"

"sizeable"

"sizeably"

"sized"

"sizer"

"sizers"

"sizes"

"sizier"

"siziest"

"siziness"

"sizing"

"sizings"

"sizy"

"sizzle"

"sizzled"

"sizzler"

"sizzlers"

"sizzles"

"sizzling"

"skag"

"skags"

"skald"

"skaldic"

"skalds"

"skate"

"skateboard"

"skateboarded"

"skateboarder"

"skateboarders"

"skateboarding"

"skateboards"

"skated"

"skater"

"skaters"

"skates"

"skating"

"skatings"

"skean"

"skeans"

"skeeing"

"skeet"

"skeeter"

"skeeters"

"skeets"

"skein"

"skeined"

"skeining"

"skeins"

"skeletal"

"skeletally"

"skeletomuscular"

"skeleton"

"skeletons"

"skelter"

"skeltering"

"skepsis"

"skepsises"

"skeptic"

"skeptical"

"skeptically"

"skepticism"

"skeptics"

"sketch"

"sketchbook"

"sketched"

"sketcher"

"sketchers"

"sketches"

"sketchier"

"sketchiest"

"sketchily"

"sketchiness"

"sketching"

"sketchy"

"skew"

"skewed"

"skewer"

"skewered"

"skewering"

"skewers"

"skewing"

"skewness"

"skews"

"ski"

"skiable"

"skiascope"

"skid"

"skidded"

"skidder"

"skidders"

"skiddier"

"skiddiest"

"skidding"

"skiddoo"

"skiddooed"

"skiddooing"

"skiddoos"

"skiddy"

"skidoo"

"skidooed"

"skidooing"

"skidoos"

"skids"

"skidways"

"skied"

"skier"

"skiers"

"skies"

"skiey"

"skiff"

"skiffs"

"skiing"

"skiings"

"skiis"

"skilful"

"skill"

"skilled"

"skillet"

"skillets"

"skillful"

"skillfully"

"skillfulness"

"skilling"

"skills"

"skim"

"skimmed"

"skimmer"

"skimmers"

"skimming"

"skimmings"

"skimp"

"skimped"

"skimpier"

"skimpiest"

"skimpily"

"skimpiness"

"skimping"

"skimps"

"skimpy"

"skims"

"skin"

"skindive"

"skindiving"

"skinflint"

"skinflints"

"skinful"

"skinfuls"

"skinhead"

"skinheads"

"skink"

"skinks"

"skinless"

"skinned"

"skinner"

"skinners"

"skinnier"

"skinniest"

"skinniness"

"skinning"

"skinny"

"skins"

"skintight"

"skip"

"skipjack"

"skipjacks"

"skiplane"

"skiplanes"

"skipped"

"skipper"

"skipperage"

"skippered"

"skippering"

"skippers"

"skipping"

"skips"

"skirl"

"skirled"

"skirling"

"skirls"

"skirmish"

"skirmished"

"skirmisher"

"skirmishers"

"skirmishes"

"skirmishing"

"skirt"

"skirted"

"skirter"

"skirters"

"skirting"

"skirtings"

"skirts"

"skis"

"skit"

"skits"

"skitter"

"skittered"

"skitterier"

"skittering"

"skitters"

"skittery"

"skittish"

"skittishness"

"skittle"

"skittles"

"skivvies"

"skivvy"

"skiwear"

"skiwears"

"skoal"

"skoaled"

"skoaling"

"skoals"

"skuas"

"skulduggery"

"skulk"

"skulked"

"skulker"

"skulkers"

"skulking"

"skulks"

"skull"

"skullcap"

"skullcaps"

"skullduggeries"

"skullduggery"

"skulled"

"skulls"

"skunk"

"skunked"

"skunking"

"skunks"

"sky"

"skyborne"

"skycap"

"skycaps"

"skycoach"

"skydive"

"skydived"

"skydiver"

"skydivers"

"skydives"

"skydiving"

"skydove"

"skyed"

"skyey"

"skyhook"

"skyhooks"

"skying"

"skyjack"

"skyjacked"

"skyjacker"

"skyjackers"

"skyjacking"

"skyjacks"

"skylab"

"skylark"

"skylarked"

"skylarker"

"skylarkers"

"skylarking"

"skylarks"

"skylight"

"skylights"

"skyline"

"skylines"

"skyman"

"skymen"

"skyrocket"

"skyrocketed"

"skyrocketing"

"skyrockets"

"skys"

"skyscraper"

"skyscrapers"

"skyscraping"

"skyward"

"skywards"

"skyway"

"skyways"

"skywrite"

"skywriter"

"skywriters"

"skywrites"

"skywriting"

"skywritten"

"skywrote"

"slab"

"slabbed"

"slabber"

"slabbering"

"slabbers"

"slabbery"

"slabbing"

"slabs"

"slack"

"slackage"

"slacked"

"slacken"

"slackened"

"slackening"

"slackens"

"slacker"

"slackers"

"slackest"

"slacking"

"slackly"

"slackness"

"slacks"

"slag"

"slagged"

"slaggier"

"slaggiest"

"slagging"

"slaggy"

"slags"

"slain"

"slakable"

"slake"

"slaked"

"slaker"

"slakers"

"slakes"

"slaking"

"slalom"

"slalomed"

"slaloming"

"slaloms"

"slam"

"slamic"

"slammed"

"slamming"

"slams"

"slander"

"slandered"

"slanderer"

"slanderers"

"slandering"

"slanderous"

"slanderously"

"slanders"

"slang"

"slanged"

"slangier"

"slangiest"

"slanginess"

"slanging"

"slangs"

"slangy"

"slant"

"slanted"

"slanting"

"slantingly"

"slants"

"slantwise"

"slap"

"slapdash"

"slapdashes"

"slaphappier"

"slaphappiest"

"slaphappy"

"slapjack"

"slapjacks"

"slapped"

"slapper"

"slappers"

"slapping"

"slaps"

"slapstick"

"slapsticks"

"slash"

"slashed"

"slasher"

"slashers"

"slashes"

"slashing"

"slashingly"

"slashings"

"slat"

"slate"

"slated"

"slater"

"slaters"

"slates"

"slather"

"slathered"

"slathering"

"slathers"

"slatier"

"slating"

"slatings"

"slats"

"slatted"

"slattern"

"slatternly"

"slatterns"

"slatting"

"slaty"

"slaughter"

"slaughtered"

"slaughterer"

"slaughterers"

"slaughterhouse"

"slaughterhouses"

"slaughtering"

"slaughters"

"slav"

"slave"

"slaved"

"slaver"

"slavered"

"slaverer"

"slaverers"

"slaveries"

"slavering"

"slavers"

"slavery"

"slaves"

"slavey"

"slaveys"

"slavic"

"slaving"

"slavish"

"slavishly"

"slavishness"

"slavs"

"slaw"

"slaws"

"slay"

"slayer"

"slayers"

"slaying"

"slays"

"sleave"

"sleazier"

"sleaziest"

"sleazily"

"sleaziness"

"sleazy"

"sled"

"sledded"

"sledder"

"sledders"

"sledding"

"sleddings"

"sledge"

"sledged"

"sledgehammer"

"sledgehammers"

"sledges"

"sledging"

"sleds"

"sleek"

"sleekened"

"sleekening"

"sleekens"

"sleeker"

"sleekest"

"sleekier"

"sleeking"

"sleekly"

"sleekness"

"sleeks"

"sleep"

"sleep activity"

"Sleep Better"

"sleep quality"

"Sleep Timer"

"sleeper"

"sleepers"

"sleepier"

"sleepiest"

"sleepily"

"sleepiness"

"sleeping"

"sleepings"

"sleepless"

"sleeplessness"

"sleeps"

"sleepwalk"

"sleepwalker"

"sleepwalkers"

"sleepwalking"

"sleepy"

"sleepyhead"

"sleepyheads"

"sleet"

"sleeted"

"sleetier"

"sleetiest"

"sleeting"

"sleets"

"sleety"

"sleeve"

"sleeved"

"sleeveless"

"sleeves"

"sleeving"

"sleigh"

"sleighed"

"sleigher"

"sleighers"

"sleighing"

"sleighs"

"sleight"

"sleights"

"slender"

"slenderer"

"slenderest"

"slenderize"

"slenderized"

"slenderizes"

"slenderizing"

"slenderly"

"slenderness"

"slept"

"sleuth"

"sleuthed"

"sleuthing"

"sleuths"

"slew"

"slewed"

"slewing"

"slews"

"slice"

"sliceable"

"sliced"

"slicer"

"slicers"

"slices"

"slicing"

"slick"

"slicked"

"slicker"

"slickered"

"slickers"

"slickest"

"slicking"

"slickly"

"slickness"

"slicks"

"slid"

"slidable"

"slidden"

"slide"

"slider"

"sliders"

"slides"

"slideshow"

"slideway"

"slideways"

"sliding"

"slier"

"sliest"

"slight"

"slighted"

"slighter"

"slightest"

"slighting"

"slightly"

"slightness"

"slights"

"slily"

"slim"

"slime"

"slimed"

"slimes"

"slimier"

"slimiest"

"slimily"

"sliming"

"slimly"

"slimmed"

"slimmer"

"slimmest"

"slimming"

"slimness"

"slims"

"slimy"

"sling"

"slinger"

"slingers"

"slinging"

"slings"

"slingshot"

"slingshots"

"slink"

"slinkier"

"slinkiest"

"slinkily"

"slinking"

"slinks"

"slinky"

"slip"

"slipcase"

"slipcases"

"slipcover"

"slipcovers"

"slipforms"

"slipknot"

"slipknots"

"slipover"

"slipovers"

"slippage"

"slippages"

"slipped"

"slipper"

"slipperier"

"slipperiest"

"slipperiness"

"slippers"

"slippery"

"slippier"

"slippiest"

"slipping"

"slippy"

"slips"

"slipshod"

"slipshodness"

"slipslop"

"slipslops"

"slipsoles"

"slipt"

"slipup"

"slipups"

"slipways"

"slit"

"slither"

"slithered"

"slithering"

"slithers"

"slithery"

"slitless"

"slits"

"slitted"

"slitter"

"slitters"

"slitting"

"sliver"

"slivered"

"sliverer"

"sliverers"

"slivering"

"slivers"

"slivovic"

"slob"

"slobber"

"slobbered"

"slobbering"

"slobbers"

"slobbery"

"slobbish"

"slobs"

"sloe"

"sloes"

"slog"

"slogan"

"slogans"

"slogged"

"slogger"

"sloggers"

"slogging"

"slogs"

"sloop"

"sloops"

"slop"

"slope"

"sloped"

"sloper"

"slopers"

"slopes"

"sloping"

"slopped"

"sloppier"

"sloppiest"

"sloppily"

"sloppiness"

"slopping"

"sloppy"

"slops"

"slopwork"

"slopworks"

"slosh"

"sloshed"

"sloshes"

"sloshier"

"sloshiest"

"sloshing"

"sloshy"

"slot"

"slotbacks"

"sloth"

"slothful"

"slothfulness"

"sloths"

"slots"

"slotted"

"slotting"

"slouch"

"slouched"

"sloucher"

"slouchers"

"slouches"

"slouchier"

"slouchiest"

"slouching"

"slouchingly"

"slouchy"

"slough"

"sloughed"

"sloughier"

"sloughiest"

"sloughing"

"sloughs"

"sloughy"

"slovak"

"slovaks"

"sloven"

"slovenlier"

"slovenliness"

"slovenly"

"slovens"

"slow"

"slowdown"

"slowdowns"

"slowed"

"slower"

"slowest"

"slowing"

"Slowing down"

"slowish"

"slowly"

"slowness"

"slowpoke"

"slowpokes"

"slows"

"slowwitted"

"slowworm"

"slowworms"

"slubbered"

"slubbering"

"slubbings"

"slubs"

"sludge"

"sludges"

"sludgier"

"sludgiest"

"sludgy"

"slue"

"slued"

"slues"

"slug"

"slugabed"

"slugabeds"

"slugfest"

"slugfests"

"sluggard"

"sluggardly"

"sluggards"

"slugged"

"slugger"

"sluggers"

"slugging"

"sluggish"

"sluggishly"

"sluggishness"

"slugs"

"sluice"

"sluiced"

"sluices"

"sluiceway"

"sluicing"

"sluicy"

"sluing"

"slum"

"slumber"

"slumbered"

"slumberer"

"slumberers"

"slumbering"

"slumberous"

"slumbers"

"slumbery"

"slumlord"

"slumlords"

"slummed"

"slummer"

"slummers"

"slummier"

"slummiest"

"slumming"

"slummy"

"slump"

"slumped"

"slumping"

"slumps"

"slums"

"slung"

"slunk"

"slur"

"slurp"

"slurped"

"slurping"

"slurps"

"slurred"

"slurried"

"slurries"

"slurring"

"slurry"

"slurrying"

"slurs"

"slush"

"slushed"

"slushes"

"slushier"

"slushiest"

"slushily"

"slushiness"

"slushing"

"slushy"

"slut"

"sluts"

"sluttish"

"sluttishness"

"sly"

"slyboots"

"slyer"

"slyest"

"slyly"

"slyness"

"slynesses"

"smack"

"smacked"

"smacker"

"smackers"

"smacking"

"smacks"

"small"

"smaller"

"smallest"

"smallholder"

"smallish"

"smallness"

"smallpox"

"smallpoxes"

"smalls"

"smarmier"

"smarmiest"

"smarmy"

"smart"

"smarted"

"smarten"

"smartened"

"smartening"

"smartens"

"smarter"

"smartest"

"smartie"

"smarties"

"smarting"

"smartingly"

"smartly"

"smartness"

"smarts"

"smarty"

"smash"

"smashable"

"smashed"

"smasher"

"smashers"

"smashes"

"smashing"

"smashingly"

"smashup"

"smashups"

"smatter"

"smattered"

"smattering"

"smatterings"

"smatters"

"smear"

"smearcase"

"smeared"

"smearer"

"smearers"

"smearier"

"smeariest"

"smearing"

"smears"

"smeary"

"smegma"

"smegmas"

"smell"

"smelled"

"smeller"

"smellers"

"smellier"

"smelliest"

"smelliness"

"smelling"

"smells"

"smelly"

"smelt"

"smelted"

"smelter"

"smelteries"

"smelters"

"smeltery"

"smelting"

"smelts"

"smidgen"

"smidgens"

"smidgeon"

"smidgeons"

"smidgins"

"smilax"

"smilaxes"

"smile"

"smiled"

"smiler"

"smilers"

"smiles"

"smiling"

"smilingly"

"smirch"

"smirched"

"smirches"

"smirching"

"smirk"

"smirked"

"smirker"

"smirkers"

"smirkier"

"smirkiest"

"smirking"

"smirkingly"

"smirks"

"smirky"

"smit"

"smite"

"smiter"

"smiters"

"smites"

"smith"

"smithereens"

"smitheries"

"smithies"

"smiths"

"smithy"

"smiting"

"smitten"

"smock"

"smocked"

"smocking"

"smockings"

"smocks"

"smog"

"smoggier"

"smoggiest"

"smoggy"

"smogless"

"smokable"

"smoke"

"smoked"

"smokehouse"

"smokehouses"

"smokeless"

"smokepot"

"smokepots"

"smoker"

"smokers"

"smokes"

"smokestack"

"smokestacks"

"smokey"

"smokier"

"smokiest"

"smokily"

"smokiness"

"smoking"

"smoky"

"smolder"

"smoldered"

"smoldering"

"smolders"

"smooch"

"smooched"

"smooches"

"smooching"

"smoochy"

"smooth"

"smoothed"

"smoothen"

"smoothened"

"smoothens"

"smoother"

"smoothers"

"smoothest"

"smoothie"

"smoothies"

"smoothing"

"smoothly"

"smoothness"

"smooths"

"smoothy"

"smorgasbord"

"smorgasbords"

"smote"

"smother"

"smothered"

"smothering"

"smothers"

"smothery"

"smoulder"

"smouldered"

"smoulders"

"smudge"

"smudged"

"smudges"

"smudgier"

"smudgiest"

"smudgily"

"smudging"

"smudgy"

"smug"

"smugger"

"smuggest"

"smuggle"

"smuggled"

"smuggler"

"smugglers"

"smuggles"

"smuggling"

"smugly"

"smugness"

"smut"

"smutch"

"smuts"

"smutted"

"smuttier"

"smuttiest"

"smuttily"

"smuttiness"

"smutting"

"smutty"

"sn"

"snack"

"snacked"

"snacking"

"snacks"

"snaffle"

"snaffled"

"snaffles"

"snafu"

"snafued"

"snafuing"

"snafus"

"snag"

"snagged"

"snaggier"

"snaggiest"

"snagging"

"snaggy"

"snags"

"snail"

"snailed"

"snailing"

"snaillike"

"snails"

"snake"

"snakebite"

"snaked"

"snakelike"

"snakes"

"snakier"

"snakiest"

"snakily"

"snaking"

"snaky"

"snap"

"snapback"

"snapdragon"

"snapdragons"

"snapless"

"snapped"

"snapper"

"snappers"

"snappier"

"snappiest"

"snappily"

"snappiness"

"snapping"

"snappish"

"snappy"

"snaps"

"snapshot"

"snapshots"

"snapweed"

"snare"

"snared"

"snarer"

"snarers"

"snares"

"snaring"

"snark"

"snarks"

"snarl"

"snarled"

"snarler"

"snarlers"

"snarlier"

"snarliest"

"snarling"

"snarlingly"

"snarls"

"snarly"

"snatch"

"snatched"

"snatcher"

"snatchers"

"snatches"

"snatchier"

"snatchiest"

"snatching"

"snatchy"

"snazzier"

"snazziest"

"snazzy"

"sneak"

"sneaked"

"sneaker"

"sneakers"

"sneakier"

"sneakiest"

"sneakily"

"sneakiness"

"sneaking"

"sneakingly"

"sneaks"

"sneaky"

"sneer"

"sneered"

"sneerer"

"sneerers"

"sneerful"

"sneering"

"sneeringly"

"sneers"

"sneeze"

"sneezed"

"sneezer"

"sneezers"

"sneezes"

"sneezier"

"sneeziest"

"sneezing"

"sneezy"

"snick"

"snicked"

"snicker"

"snickered"

"snickering"

"snickeringly"

"snickers"

"snickery"

"snicking"

"snicks"

"snide"

"snidely"

"snideness"

"snider"

"snidest"

"sniff"

"sniffed"

"sniffer"

"sniffers"

"sniffier"

"sniffily"

"sniffing"

"sniffingly"

"sniffish"

"sniffle"

"sniffled"

"sniffler"

"snifflers"

"sniffles"

"sniffling"

"sniffs"

"sniffy"

"snifter"

"snifters"

"snigger"

"sniggered"

"sniggering"

"sniggeringly"

"sniggers"

"sniggle"

"snigglers"

"sniggling"

"snip"

"snipe"

"sniped"

"sniper"

"snipers"

"snipes"

"sniping"

"snipped"

"snipper"

"snippers"

"snippet"

"snippets"

"snippety"

"snippier"

"snippiest"

"snippily"

"snippiness"

"snipping"

"snippy"

"snips"

"snit"

"snitch"

"snitched"

"snitcher"

"snitchers"

"snitches"

"snitching"

"snits"

"snivel"

"sniveled"

"sniveler"

"snivelers"

"sniveling"

"snivelled"

"snivelling"

"snivels"

"snob"

"snobberies"

"snobbery"

"snobbier"

"snobbiest"

"snobbily"

"snobbish"

"snobbishly"

"snobbishness"

"snobbism"

"snobbisms"

"snobby"

"snobs"

"snood"

"snoods"

"snooker"

"snookers"

"snooking"

"snoop"

"snooped"

"snooper"

"snoopers"

"snooperscope"

"snoopier"

"snoopiest"

"snoopily"

"snooping"

"snoops"

"snoopy"

"snoot"

"snooted"

"snootier"

"snootiest"

"snootily"

"snootiness"

"snooting"

"snoots"

"snooty"

"snooze"

"snooze alarm"

"snooze alarm clock"

"Snooze Time"

"snoozed"

"snoozer"

"snoozers"

"snoozes"

"snoozier"

"snoozing"

"snoozy"

"snore"

"snored"

"snorer"

"snorers"

"snores"

"snoring"

"snorkel"

"snorkeled"

"snorkeling"

"snorkels"

"snort"

"snorted"

"snorter"

"snorters"

"snorting"

"snorts"

"snot"

"snots"

"snottier"

"snottiest"

"snottily"

"snotty"

"snout"

"snouted"

"snoutier"

"snoutiest"

"snouting"

"snoutish"

"snouts"

"snouty"

"snow"

"snowball"

"snowballed"

"snowballing"

"snowballs"

"snowbank"

"snowbanks"

"snowbarding"

"snowbelt"

"snowbirds"

"snowbound"

"snowbushes"

"snowcap"

"snowcapped"

"snowcaps"

"snowdrift"

"snowdrifts"

"snowdrop"

"snowdrops"

"snowed"

"snowfall"

"snowfalls"

"snowfield"

"snowflake"

"snowflakes"

"snowier"

"snowiest"

"snowily"

"snowing"

"snowman"

"snowmelt"

"snowmelts"

"snowmen"

"snowmobile"

"snowmobiler"

"snowmobilers"

"snowmobiles"

"snowmobiling"

"snowpack"

"snowpacks"

"snowplow"

"snowplowed"

"snowplows"

"snows"

"snowshoe"

"snowshoed"

"snowshoes"

"snowslide"

"snowstorm"

"snowstorms"

"snowsuit"

"snowsuits"

"snowy"

"snub"

"snubbed"

"snubber"

"snubbers"

"snubbier"

"snubbiest"

"snubbing"

"snubby"

"snubness"

"snubs"

"snuck"

"snuff"

"snuffbox"

"snuffboxes"

"snuffed"

"snuffer"

"snuffers"

"snuffier"

"snuffiest"

"snuffily"

"snuffing"

"snuffle"

"snuffled"

"snuffler"

"snufflers"

"snuffles"

"snufflier"

"snuffliest"

"snuffling"

"snuffly"

"snuffs"

"snuffy"

"snug"

"snugged"

"snugger"

"snuggeries"

"snuggery"

"snuggest"

"snugging"

"snuggle"

"snuggled"

"snuggles"

"snuggling"

"snugly"

"snugness"

"snugs"

"so"

"soak"

"soaked"

"soaker"

"soakers"

"soaking"

"soaks"

"soap"

"soapbark"

"soapbox"

"soapboxes"

"soaped"

"soaper"

"soapers"

"soapier"

"soapiest"

"soapily"

"soapiness"

"soaping"

"soapless"

"soapmaking"

"soaps"

"soapstone"

"soapstones"

"soapsuds"

"soapwort"

"soapworts"

"soapy"

"soar"

"soared"

"soarer"

"soarers"

"soaring"

"soarings"

"soars"

"soave"

"sob"

"sobbed"

"sobber"

"sobbers"

"sobbing"

"sobbingly"

"sobeit"

"sober"

"sobered"

"soberer"

"soberest"

"sobering"

"soberize"

"soberizing"

"soberly"

"soberness"

"sobers"

"sobful"

"sobrieties"

"sobriety"

"sobriquet"

"sobriquets"

"sobs"

"soc"

"socalled"

"soccer"

"soccers"

"sociability"

"sociable"

"sociables"

"sociably"

"social"

"socialism"

"socialist"

"socialistic"

"socialists"

"socialite"

"socialites"

"socialization"

"socialize"

"socialized"

"socializer"

"socializers"

"socializes"

"socializing"

"socially"

"socials"

"societal"

"societies"

"society"

"sociobiology"

"sociocentricity"

"sociocentrism"

"socioeconomic"

"sociologic"

"sociological"

"sociologically"

"sociologies"

"sociologist"

"sociologists"

"sociology"

"sociometric"

"sociopath"

"sociopathic"

"sociopathies"

"sociopaths"

"sociopathy"

"sociopolitical"

"sociosexual"

"sociosexualities"

"sociosexuality"

"sock"

"socked"

"socket"

"socketed"

"socketing"

"sockets"

"sockeye"

"sockeyes"

"socking"

"sockman"

"sockmen"

"socks"

"socrates"

"socratic"

"sod"

"soda"

"sodalist"

"sodalite"

"sodalities"

"sodality"

"sodas"

"sodded"

"sodden"

"soddened"

"soddening"

"soddenly"

"soddenness"

"soddens"

"soddies"

"sodding"

"soddy"

"sodium"

"sodiums"

"sodom"

"sodomies"

"sodomite"

"sodomites"

"sodomy"

"sods"

"soever"

"sofa"

"sofar"

"sofars"

"sofas"

"soffit"

"soffits"

"sofia"

"soft"

"softbacks"

"softball"

"softballs"

"softbound"

"soften"

"softened"

"softener"

"softeners"

"softening"

"softens"

"softer"

"softest"

"softheads"

"softhearted"

"softheartedly"

"softheartedness"

"softie"

"softies"

"softly"

"softness"

"softs"

"software"

"softwares"

"softwood"

"softwoods"

"softy"

"sogged"

"soggier"

"soggiest"

"soggily"

"sogginess"

"soggy"

"soigne"

"soil"

"soilage"

"soilages"

"soilborne"

"soiled"

"soiling"

"soilless"

"soils"

"soiree"

"soirees"

"sojourn"

"sojourned"

"sojourner"

"sojourners"

"sojourning"

"sojournment"

"sojourns"

"sol"

"solace"

"solaced"

"solacer"

"solacers"

"solaces"

"solacing"

"solanums"

"solar"

"solaria"

"solarimeter"

"solarism"

"solarisms"

"solarium"

"solariums"

"solarization"

"solarize"

"solarized"

"solarizes"

"solarizing"

"sold"

"solder"

"soldered"

"solderer"

"solderers"

"soldering"

"solders"

"soldier"

"soldiered"

"soldiering"

"soldierly"

"soldiers"

"soldiery"

"sole"

"solecism"

"solecisms"

"solecist"

"solecists"

"solecize"

"solecized"

"solecizes"

"soled"

"soleless"

"solely"

"solemn"

"solemner"

"solemnest"

"solemnity"

"solemnization"

"solemnize"

"solemnized"

"solemnizes"

"solemnizing"

"solemnly"

"solemnness"

"soleness"

"solenoid"

"solenoidal"

"solenoids"

"soleplate"

"soleprint"

"soles"

"solfege"

"solfeges"

"solfeggi"

"solfeggio musical"

"soli"

"solicit"

"solicitation"

"solicitations"

"solicited"

"soliciting"

"solicitor"

"solicitors"

"solicitorship"

"solicitous"

"solicitously"

"solicitousness"

"solicitress"

"solicits"

"solicitude"

"solid"

"solidarities"

"solidarity"

"solidary"

"solider"

"solidest"

"solidi"

"solidification"

"solidified"

"solidifies"

"solidify"

"solidifying"

"solidities"

"solidity"

"solidly"

"solidness"

"solido"

"solids"

"solidus"

"soliloquies"

"soliloquize"

"soliloquized"

"soliloquizes"

"soliloquizing"

"soliloquy"

"soling"

"solipsism"

"solipsist"

"solipsistic"

"solipsists"

"soliquid"

"solitaire"

"solitaires"

"solitaries"

"solitariness"

"solitary"

"solitude"

"solitudes"

"solo"

"soloed"

"soloing"

"soloist"

"soloists"

"solomon"

"solos"

"solstice"

"solstices"

"solstitial"

"solubilities"

"solubility"

"solubilization"

"solubilized"

"solubilizing"

"soluble"

"solubles"

"solubly"

"solus"

"solute"

"solutes"

"solution"

"solutions"

"solvability"

"solvable"

"solvate"

"solvated"

"solvates"

"solvating"

"solvation"

"solve"

"solved"

"solvencies"

"solvency"

"solvent"

"solvently"

"solvents"

"solver"

"solvers"

"solves"

"solving"

"soma"

"somalia"

"somas"

"somatic"

"somatically"

"somatological"

"somatology"

"somatopsychic"

"somatotypically"

"somatotypology"

"somber"

"somberly"

"somberness"

"sombre"

"sombrely"

"sombrero"

"sombreros"

"some"

"somebodies"

"somebody"

"someday"

"somehow"

"someone"

"someplace"

"somersault"

"somersaulted"

"somersaulting"

"somersaults"

"somesthesises"

"something"

"sometime"

"sometimes"

"someway"

"someways"

"somewhat"

"somewhats"

"somewhen"

"somewhere"

"somewise"

"somnambulant"

"somnambular"

"somnambulate"

"somnambulated"

"somnambulating"

"somnambulation"

"somnambulator"

"somnambulism"

"somnambulist"

"somnambulistic"

"somnambulists"

"somnific"

"somniloquies"

"somniloquist"

"somnolence"

"somnolences"

"somnolencies"

"somnolency"

"somnolent"

"somnolently"

"son"

"sonants"

"sonar"

"sonarman"

"sonarmen"

"sonars"

"sonata"

"sonatas"

"sonatina"

"sonatinas"

"sonatine"

"sonde"

"sone"

"sones"

"song"

"songbird"

"songbirds"

"songbook"

"songbooks"

"songfest"

"songfests"

"songful"

"songfully"

"songs"

"songster"

"songsters"

"songstress"

"songstresses"

"songwriter"

"songwriters"

"sonic"

"sonics"

"sonless"

"sonnet"

"sonneted"

"sonneting"

"sonnets"

"sonnetted"

"sonnetting"

"sonnies"

"sonny"

"sonograph"

"sonorant"

"sonorants"

"sonorities"

"sonority"

"sonorous"

"sonorously"

"sons"

"sonships"

"sooey"

"soon"

"sooner"

"sooners"

"soonest"

"soot"

"sooted"

"sooth"

"soothe"

"soothed"

"soother"

"soothers"

"soothes"

"soothest"

"soothing"

"soothing backgrounds"

"soothingly"

"soothly"

"sooths"

"soothsaid"

"soothsay"

"soothsayer"

"soothsayers"

"soothsaying"

"soothsays"

"sootier"

"sootiest"

"sootily"

"sooting"

"soots"

"sooty"

"sop"

"soph"

"sophies"

"sophiology"

"sophism"

"sophisms"

"sophist"

"sophistic"

"sophistical"

"sophisticate"

"sophisticated"

"sophisticatedly"

"sophisticates"

"sophisticating"

"sophistication"

"sophisticator"

"sophistries"

"sophistry"

"sophists"

"sophoclean"

"sophocles"

"sophomania"

"sophomore"

"sophomores"

"sophomoric"

"sophomorically"

"sophs"

"sophy"

"sopor"

"soporific"

"soporifically"

"soporifics"

"soporose"

"sopors"

"sopped"

"soppier"

"soppiest"

"sopping"

"soppy"

"soprani"

"soprano"

"sopranos"

"sops"

"sorbate"

"sorbates"

"sorbed"

"sorbet"

"sorbets"

"sorbic"

"sorbitol"

"sorbitols"

"sorcerer"

"sorcerers"

"sorceress"

"sorceresses"

"sorceries"

"sorcery"

"sordid"

"sordidly"

"sordidness"

"sore"

"sorehead"

"soreheads"

"sorel"

"sorels"

"sorely"

"soreness"

"sorer"

"sores"

"sorest"

"sorghum"

"sorghums"

"sororities"

"sorority"

"sorption"

"sorptive"

"sorrel"

"sorrels"

"sorrier"

"sorriest"

"sorrily"

"sorriness"

"sorrow"

"sorrowed"

"sorrower"

"sorrowers"

"sorrowful"

"sorrowfully"

"sorrowfulness"

"sorrowing"

"sorrows"

"sorry"

"sort"

"sortable"

"sortably"

"sorted"

"sorter"

"sorters"

"sortie"

"sortied"

"sortieing"

"sorties"

"sorting"

"sorts"

"sos"

"sot"

"soteriology"

"sots"

"sotted"

"sottish"

"sottishly"

"soubrette"

"soubrettes"

"soubriquet"

"souchong"

"soudan"

"souffle"

"souffles"

"sough"

"soughed"

"soughing"

"soughs"

"sought"

"soul"

"souled"

"soulful"

"soulfully"

"soulfulness"

"soulless"

"soullessness"

"souls"

"sound"

"sound alarm"

"sound loops and samples"

"Sound Sleep"

"sound tracks"

"sound visualizations"

"soundboard"

"soundboards"

"soundbox"

"soundboxes"

"sounded"

"sounder"

"sounders"

"soundest"

"sounding"

"soundings"

"soundless"

"soundlessly"

"soundly"

"soundness"

"soundproof"

"soundproofed"

"soundproofing"

"soundproofs"

"sounds"

"soundtrack"

"soundtracks"

"soup"

"soupcon"

"soupcons"

"souped"

"soupier"

"soupiest"

"souping"

"soups"

"soupy"

"sour"

"sourball"

"sourballs"

"source"

"sources"

"sourdough"

"sourdoughs"

"soured"

"sourer"

"sourest"

"souring"

"sourish"

"sourly"

"sourness"

"sourpuss"

"sourpusses"

"sours"

"soursops"

"sourwood"

"souse"

"soused"

"souses"

"sousing"

"south"

"southbound"

"southeast"

"southeaster"

"southeasterly"

"southeastern"

"southeasters"

"southeastward"

"southeastwardly"

"southed"

"souther"

"southerly"

"southern"

"southerner"

"southerners"

"southernmost"

"southerns"

"southers"

"southing"

"southings"

"southpaw"

"southpaws"

"southron"

"southrons"

"souths"

"southward"

"southwardly"

"southwest"

"southwester"

"southwesterly"

"southwestern"

"southwesterner"

"southwesterners"

"southwesters"

"southwestward"

"southwestwardly"

"souvenir"

"souvenirs"

"sovereign"

"sovereignly"

"sovereigns"

"sovereignties"

"sovereignty"

"soviet"

"sovietism"

"sovietize"

"sovietized"

"sovietizes"

"sovietizing"

"soviets"

"sovran"

"sovrans"

"sow"

"sowable"

"sowbellies"

"sowbelly"

"sowbread"

"sowed"

"sower"

"sowers"

"sowing"

"sown"

"sows"

"sox"

"soy"

"soya"

"soyas"

"soybean"

"soybeans"

"soys"

"sp"

"spa"

"space"

"spacecraft"

"spaced"

"spaceflight"

"spaceflights"

"spaceless"

"spaceman"

"spacemen"

"spaceport"

"spacer"

"spacers"

"spaces"

"spaceship"

"spaceships"

"spacesuit"

"spacesuits"

"spacewalk"

"spacewalked"

"spacewalker"

"spacewalkers"

"spacewalking"

"spacewalks"

"spaceward"

"spacewoman"

"spacewomen"

"spacial"

"spacing"

"spacings"

"spacious"

"spaciously"

"spaciousness"

"spade"

"spaded"

"spadeful"

"spadefuls"

"spader"

"spaders"

"spades"

"spadework"

"spadices"

"spading"

"spadix"

"spadixes"

"spaghetti"

"spain"

"spake"

"spale"

"spalled"

"spaller"

"spalls"

"spalpeen"

"span"

"spangle"

"spangled"

"spangles"

"spanglier"

"spangliest"

"spangling"

"spangly"

"spaniard"

"spaniards"

"spaniel"

"spaniels"

"spank"

"spanked"

"spanker"

"spankers"

"spanking"

"spankings"

"spanks"

"spanless"

"spanned"

"spanner"

"spanners"

"spanning"

"spans"

"spar"

"sparable"

"spare"

"spared"

"sparely"

"spareness"

"sparer"

"sparerib"

"spareribs"

"sparers"

"spares"

"sparest"

"sparge"

"sparing"

"sparingly"

"spark"

"sparked"

"sparker"

"sparkers"

"sparkier"

"sparkiest"

"sparkily"

"sparking"

"sparkish"

"sparkle"

"sparkled"

"sparkler"

"sparklers"

"sparkles"

"sparkling"

"sparkplug"

"sparks"

"sparky"

"sparred"

"sparriest"

"sparring"

"sparrow"

"sparrows"

"sparry"

"spars"

"sparse"

"sparsely"

"sparseness"

"sparser"

"sparsest"

"sparsim"

"sparsities"

"sparsity"

"sparta"

"spartan"

"spartans"

"spas"

"spasm"

"spasmodic"

"spasmodical"

"spasmodically"

"spasms"

"spastic"

"spastically"

"spasticities"

"spasticity"

"spastics"

"spat"

"spate"

"spates"

"spathal"

"spathe"

"spathed"

"spathes"

"spathic"

"spatial"

"spatially"

"spats"

"spatted"

"spatter"

"spattered"

"spattering"

"spatteringly"

"spatters"

"spatting"

"spatula"

"spatular"

"spatulas"

"spatulate"

"spavin"

"spavined"

"spavins"

"spawn"

"spawned"

"spawner"

"spawners"

"spawning"

"spawns"

"spay"

"spayed"

"spaying"

"spays"

"speak"

"speakable"

"speakeasies"

"speakeasy"

"speaker"

"speakers"

"speaking"

"speakings"

"speaks"

"spear"

"speared"

"spearer"

"spearers"

"spearfish"

"spearhead"

"spearheaded"

"spearheading"

"spearheads"

"spearing"

"spearman"

"spearmen"

"spearmint"

"spearmints"

"spears"

"spec"

"special"

"specialer"

"specialist"

"specialists"

"specialization"

"specializations"

"specialize"

"specialized"

"specializes"

"specializing"

"specially"

"specials"

"specialties"

"specialty"

"speciating"

"specie"

"species"

"specific"

"specifically"

"specificated"

"specification"

"specifications"

"specificities"

"specificity"

"specificized"

"specificizing"

"specifics"

"specified"

"specifier"

"specifiers"

"specifies"

"specify"

"specifying"

"specimen"

"specimens"

"speciosities"

"speciosity"

"specious"

"speciously"

"speciousness"

"speck"

"specked"

"specking"

"speckle"

"speckled"

"speckles"

"speckling"

"specks"

"specs"

"spectacle"

"spectacles"

"spectacular"

"spectacularly"

"spectaculars"

"spectate"

"spectated"

"spectates"

"spectating"

"spectator"

"spectators"

"specter"

"specters"

"spectra"

"spectral"

"spectre"

"spectres"

"spectrochemical"

"spectrochemistry"

"spectrofluorimeter"

"spectrogram"

"spectrograms"

"spectrograph"

"spectrographer"

"spectrographic"

"spectrographically"

"spectrographies"

"spectrographs"

"spectrography"

"spectroheliograph"

"spectroheliokinematograph"

"spectrohelioscope"

"spectrology"

"spectrometer"

"spectrometers"

"spectrometric"

"spectrometries"

"spectrometry"

"spectrophobia"

"spectrophotometer"

"spectroscope"

"spectroscopes"

"spectroscopic"

"spectroscopical"

"spectroscopically"

"spectroscopies"

"spectroscopist"

"spectroscopists"

"spectroscopy"

"spectrum"

"spectrums"

"specula"

"specular"

"speculate"

"speculated"

"speculates"

"speculating"

"speculation"

"speculations"

"speculative"

"speculatively"

"speculator"

"speculators"

"speculum"

"speculums"

"sped"

"speech"

"speeches"

"speechless"

"speechlessly"

"speechlessness"

"speed"

"speedboat"

"speedboating"

"speedboats"

"speeded"

"speeder"

"speeders"

"speedier"

"speediest"

"speedily"

"speediness"

"speeding"

"speedings"

"speedometer"

"speedometers"

"speeds"

"speedster"

"speedup"

"speedups"

"speedway"

"speedways"

"speedwell"

"speedwells"

"speedy"

"speiled"

"speleologist"

"speleologists"

"speleology"

"spell"

"spellbind"

"spellbinder"

"spellbinders"

"spellbinding"

"spellbinds"

"spellbound"

"spelldown"

"spelldowns"

"spelled"

"speller"

"spellers"

"spelling"

"spellings"

"spells"

"spelt"

"spelunk"

"spelunked"

"spelunker"

"spelunkers"

"spelunking"

"spelunks"

"spence"

"spencer"

"spences"

"spend"

"spendable"

"spender"

"spenders"

"spending"

"spends"

"spendthrift"

"spendthriftiness"

"spendthrifts"

"spendthrifty"

"spent"

"sperm"

"spermary"

"spermatic"

"spermatocidal"

"spermatocide"

"spermatozoa"

"spermatozoan"

"spermatozoon"

"spermic"

"spermicidal"

"spermicide"

"spermology"

"spermous"

"sperms"

"spew"

"spewed"

"spewer"

"spewers"

"spewing"

"spews"

"sphagnology"

"sphagnum"

"sphagnums"

"sphenoid"

"spheral"

"sphere"

"sphered"

"spheres"

"spheric"

"spherical"

"spherically"

"sphericity"

"spherics"

"spherier"

"sphering"

"spheroid"

"spheroidal"

"spheroids"

"spherometer"

"spherule"

"sphincter"

"sphincteral"

"sphincters"

"sphinges"

"sphinx"

"sphinxes"

"sphragistics"

"sphygmogram"

"sphygmograph"

"sphygmographic"

"sphygmographies"

"sphygmography"

"sphygmology"

"sphygmomanometer"

"sphygmomanometers"

"sphygmomanometry"

"sphygmometer"

"sphygmoscope"

"spic"

"spica"

"spicas"

"spice"

"spiced"

"spicer"

"spicers"

"spicery"

"spices"

"spicey"

"spicier"

"spiciest"

"spicily"

"spiciness"

"spicing"

"spick"

"spics"

"spicular"

"spiculate"

"spicule"

"spicules"

"spicy"

"spider"

"spiderier"

"spideriest"

"spiders"

"spidery"

"spied"

"spiegel"

"spiegels"

"spiel"

"spieled"

"spieler"

"spielers"

"spieling"

"spiels"

"spier"

"spiers"

"spies"

"spiff"

"spiffier"

"spiffiest"

"spiffily"

"spiffing"

"spiffy"

"spigot"

"spigots"

"spike"

"spiked"

"spikelet"

"spikelets"

"spiker"

"spikers"

"spikes"

"spikier"

"spikiest"

"spikily"

"spiking"

"spiky"

"spill"

"spillable"

"spillage"

"spilled"

"spiller"

"spillers"

"spilling"

"spills"

"spillway"

"spillways"

"spilt"

"spilth"

"spilths"

"spin"

"spinach"

"spinaches"

"spinage"

"spinal"

"spinally"

"spinals"

"spinate"

"spindle"

"spindled"

"spindler"

"spindlers"

"spindles"

"spindlier"

"spindliest"

"spindling"

"spindly"

"spine"

"spined"

"spinel"

"spineless"

"spinelessly"

"spinelessness"

"spinels"

"spines"

"spinet"

"spinets"

"spinier"

"spiniest"

"spinless"

"spinnaker"

"spinnakers"

"spinner"

"spinneret"

"spinneries"

"spinners"

"spinnery"

"spinney"

"spinneys"

"spinnies"

"spinning"

"spinnings"

"spinny"

"spinocerebellar"

"spinoff"

"spinoffs"

"spinosely"

"spinout"

"spinouts"

"spins"

"spinster"

"spinsterhood"

"spinsters"

"spinthariscope"

"spiny"

"spiracle"

"spiracles"

"spiraea"

"spiraeas"

"spiral"

"spiraled"

"spiraling"

"spiralled"

"spiralling"

"spirally"

"spirals"

"spirant"

"spire"

"spirea"

"spireas"

"spired"

"spires"

"spiring"

"spirit"

"spirited"

"spiritedly"

"spiritedness"

"spiriting"

"spiritless"

"spiritlessly"

"spirits"

"spiritual"

"Spiritual Regression Hypnotherapy"

"Spiritual RegressionÃƒâ€šÃ‚Â Hypnotherapy"

"Spiritual RegressionÃ‚Â Hypnotherapy"

"spiritual unification"

"spiritualism"

"spiritualist"

"spiritualistic"

"spiritualists"

"spirituality"

"spiritualize"

"spiritualized"

"spiritualizes"

"spiritualizing"

"spiritually"

"spirituals"

"spirituous"

"spiritus"

"spirochetal"

"spirochete"

"spirochetes"

"spirogram"

"spirograph"

"spiroid"

"spirometer"

"spirted"

"spirts"

"spiry"

"spit"

"spital"

"spitball"

"spitballs"

"spite"

"spited"

"spiteful"

"spitefully"

"spitefulness"

"spites"

"spitfire"

"spitfires"

"spiting"

"spits"

"spitted"

"spitter"

"spitters"

"spitting"

"spittle"

"spittles"

"spittoon"

"spittoons"

"spitz"

"splanchnology"

"splash"

"splashdown"

"splashdowns"

"splashed"

"splasher"

"splashers"

"splashes"

"splashier"

"splashiest"

"splashily"

"splashiness"

"splashing"

"splashy"

"splat"

"splats"

"splatter"

"splattered"

"splattering"

"splatters"

"splay"

"splayed"

"splayfeet"

"splayfoot"

"splayfooted"

"splaying"

"splays"

"spleen"

"spleenier"

"spleeniest"

"spleenish"

"spleens"

"spleeny"

"splendid"

"splendider"

"splendidly"

"splendor"

"splendorous"

"splendors"

"splenectomies"

"splenectomize"

"splenectomized"

"splenectomizing"

"splenectomy"

"splenetic"

"splenetically"

"splenic"

"splenification"

"splenitises"

"splent"

"splice"

"spliced"

"splicer"

"splicers"

"splices"

"splicing"

"spline"

"splined"

"splines"

"splining"

"splint"

"splinted"

"splinter"

"splintered"

"splintering"

"splinters"

"splintery"

"splinting"

"splints"

"split"

"splits"

"splitter"

"splitters"

"splitting"

"splosh"

"sploshed"

"sploshes"

"splotch"

"splotched"

"splotches"

"splotchier"

"splotchiest"

"splotching"

"splotchy"

"splurge"

"splurged"

"splurges"

"splurgiest"

"splurging"

"splurgy"

"splutter"

"spluttered"

"spluttering"

"splutters"

"spoil"

"spoilable"

"spoilage"

"spoilages"

"spoiled"

"spoiler"

"spoilers"

"spoiling"

"spoils"

"spoilsman"

"spoilsmen"

"spoilsport"

"spoilsports"

"spoilt"

"spokane"

"spoke"

"spoked"

"spoken"

"spokes"

"spokesman"

"spokesmen"

"spokeswoman"

"spokeswomen"

"spoking"

"spoliation"

"spoliator"

"spoliators"

"spondaic"

"spondaics"

"spondee"

"spondees"

"spondylosis"

"sponge"

"sponged"

"sponger"

"spongers"

"sponges"

"spongier"

"spongiest"

"spongily"

"sponging"

"spongins"

"spongology"

"spongy"

"sponsor"

"sponsored"

"sponsorial"

"sponsoring"

"sponsors"

"sponsorship"

"sponsorships"

"spontaneity"

"spontaneous"

"spontaneously"

"spontaneousness"

"spoof"

"spoofed"

"spoofing"

"spoofs"

"spook"

"spooked"

"spookier"

"spookiest"

"spookily"

"spooking"

"spookish"

"spooks"

"spooky"

"spool"

"spooled"

"spooler"

"spoolers"

"spooling"

"spools"

"spoon"

"spoonbill"

"spoonbills"

"spooned"

"spoonerism"

"spoonerisms"

"spoonful"

"spoonfuls"

"spoonier"

"spoonies"

"spooniest"

"spoonily"

"spooning"

"spoons"

"spoonsful"

"spoony"

"spoor"

"spoored"

"spooring"

"spoors"

"sporadic"

"sporadically"

"spore"

"spored"

"spores"

"sporing"

"sporozoa"

"sporozoan"

"sporozoon"

"sporran"

"sporrans"

"sport"

"sported"

"sporter"

"sporters"

"sportful"

"sportier"

"sportiest"

"sportily"

"sporting"

"sportive"

"sportively"

"sports"

"sports timer"

"sportscast"

"sportscaster"

"sportscasters"

"sportscasts"

"sportsman"

"sportsmanlike"

"sportsmanship"

"sportsmen"

"sportswear"

"sportswoman"

"sportswomen"

"sportswriter"

"sportswriters"

"sporty"

"sporulate"

"sporule"

"spot"

"spotless"

"spotlessly"

"spotlight"

"spotlights"

"spots"

"spotted"

"spotter"

"spotters"

"spottier"

"spottiest"

"spottily"

"spottiness"

"spotting"

"spotty"

"spousal"

"spouse"

"spoused"

"spouseless"

"spouses"

"spout"

"spouted"

"spouter"

"spouters"

"spouting"

"spouts"

"spraddle"

"sprain"

"sprained"

"spraining"

"sprains"

"sprang"

"sprat"

"sprats"

"sprattle"

"sprawl"

"sprawled"

"sprawler"

"sprawlers"

"sprawlier"

"sprawliest"

"sprawling"

"sprawls"

"sprawly"

"spray"

"sprayed"

"sprayer"

"sprayers"

"spraying"

"sprays"

"spread"

"spreadable"

"spreader"

"spreaders"

"spreading"

"spreads"

"spreadsheet"

"spreadsheets"

"spree"

"sprees"

"sprier"

"spriest"

"sprig"

"sprigged"

"sprigger"

"spriggy"

"spright"

"sprightlier"

"sprightliest"

"sprightliness"

"sprightly"

"sprights"

"sprigs"

"spring"

"springboard"

"springboards"

"springed"

"springer"

"springers"

"springes"

"springfield"

"springier"

"springiest"

"springiness"

"springing"

"springs"

"springtime"

"springy"

"sprinkle"

"sprinkled"

"sprinkler"

"sprinklers"

"sprinkles"

"sprinkling"

"sprinklings"

"sprint"

"sprinted"

"sprinter"

"sprinters"

"sprinting"

"sprints"

"sprit"

"sprite"

"sprites"

"sprits"

"sprocket"

"sprockets"

"sprout"

"sprouted"

"sprouting"

"sprouts"

"spruce"

"spruced"

"sprucer"

"spruces"

"sprucest"

"sprucing"

"sprucy"

"sprung"

"spry"

"spryer"

"spryest"

"spryly"

"spryness"

"spud"

"spuds"

"spued"

"spues"

"spuing"

"spumante"

"spume"

"spumed"

"spumes"

"spumier"

"spuming"

"spumone"

"spumones"

"spumoni"

"spumonis"

"spumous"

"spumy"

"spun"

"spunk"

"spunked"

"spunkier"

"spunkies"

"spunkiest"

"spunkily"

"spunkiness"

"spunks"

"spunky"

"spur"

"spurge"

"spurious"

"spuriously"

"spuriousness"

"spurn"

"spurned"

"spurner"

"spurners"

"spurning"

"spurns"

"spurred"

"spurrer"

"spurrers"

"spurrey"

"spurreys"

"spurrier"

"spurries"

"spurring"

"spurry"

"spurs"

"spurt"

"spurted"

"spurting"

"spurtles"

"spurts"

"sputa"

"sputnik"

"sputniks"

"sputter"

"sputtered"

"sputterer"

"sputterers"

"sputtering"

"sputters"

"sputum"

"spy"

"spyglass"

"spyglasses"

"spying"

"squab"

"squabbier"

"squabbiest"

"squabble"

"squabbled"

"squabbler"

"squabblers"

"squabbles"

"squabbling"

"squabby"

"squabs"

"squad"

"squadded"

"squadron"

"squadroned"

"squadrons"

"squads"

"squalid"

"squalider"

"squalidest"

"squalidly"

"squalidness"

"squall"

"squalled"

"squaller"

"squallers"

"squallier"

"squalliest"

"squalling"

"squalls"

"squally"

"squalor"

"squalors"

"squamous"

"squander"

"squandered"

"squanderer"

"squanderers"

"squandering"

"squandermania"

"squanders"

"square"

"squared"

"squarely"

"squareness"

"squarer"

"squarers"

"squares"

"squarest"

"squaring"

"squarish"

"squash"

"squashed"

"squasher"

"squashers"

"squashes"

"squashier"

"squashiest"

"squashing"

"squashy"

"squat"

"squatly"

"squatness"

"squats"

"squatted"

"squatter"

"squatters"

"squattest"

"squattier"

"squattiest"

"squatting"

"squatty"

"squaw"

"squawk"

"squawked"

"squawker"

"squawkers"

"squawking"

"squawks"

"squaws"

"squeak"

"squeaked"

"squeaker"

"squeakers"

"squeakier"

"squeakiest"

"squeaking"

"squeaks"

"squeaky"

"squeal"

"squealed"

"squealer"

"squealers"

"squealing"

"squeals"

"squeamish"

"squeamishly"

"squeamishness"

"squeegee"

"squeegeed"

"squeegees"

"squeeze"

"squeezed"

"squeezer"

"squeezers"

"squeezes"

"squeezing"

"squelch"

"squelched"

"squelcher"

"squelchers"

"squelches"

"squelchier"

"squelching"

"squelchy"

"squib"

"squibs"

"squid"

"squidded"

"squidding"

"squids"

"squiffed"

"squiggle"

"squiggled"

"squiggles"

"squigglier"

"squiggling"

"squiggly"

"squinch"

"squinched"

"squinches"

"squinching"

"squint"

"squinted"

"squinter"

"squinters"

"squintier"

"squintiest"

"squinting"

"squints"

"squinty"

"squire"

"squired"

"squires"

"squiring"

"squirish"

"squirm"

"squirmed"

"squirmer"

"squirmers"

"squirmier"

"squirmiest"

"squirming"

"squirms"

"squirmy"

"squirrel"

"squirreled"

"squirreling"

"squirrelled"

"squirrelling"

"squirrels"

"squirt"

"squirted"

"squirter"

"squirters"

"squirting"

"squirts"

"squish"

"squished"

"squishes"

"squishier"

"squishiest"

"squishing"

"squishy"

"squooshed"

"squooshes"

"squooshing"

"squushing"

"sr"

"sraeli"

"sri"

"ss"

"st"

"stab"

"stabbed"

"stabber"

"stabbers"

"stabbing"

"stabile"

"stabiles"

"stabilities"

"stability"

"stabilization"

"stabilize"

"stabilized"

"stabilizer"

"stabilizers"

"stabilizes"

"stabilizing"

"stable"

"stabled"

"stableman"

"stableness"

"stabler"

"stablers"

"stables"

"stabling"

"stablings"

"stably"

"stabs"

"staccato"

"staccatos"

"stack"

"stacked"

"stacker"

"stackers"

"stacking"

"stacks"

"stactometer"

"stadia"

"stadiometer"

"stadium"

"stadiums"

"staff"

"staffed"

"staffer"

"staffers"

"staffing"

"staffs"

"stag"

"stage"

"stagecoach"

"stagecoaches"

"staged"

"stagehand"

"stagehands"

"stager"

"stagers"

"stages"

"stagestruck"

"stagey"

"stagflation"

"stagger"

"staggered"

"staggerer"

"staggerers"

"staggering"

"staggeringly"

"staggers"

"staggery"

"staggier"

"staggy"

"stagier"

"stagiest"

"stagily"

"staging"

"stagings"

"stagmometer"

"stagnancy"

"stagnant"

"stagnantly"

"stagnate"

"stagnated"

"stagnates"

"stagnating"

"stagnation"

"stags"

"stagy"

"staid"

"staider"

"staidest"

"staidly"

"stain"

"stainabilities"

"stainability"

"stainable"

"stained"

"stainer"

"stainers"

"staining"

"stainless"

"stains"

"stair"

"staircase"

"staircases"

"stairs"

"stairway"

"stairways"

"stairwell"

"stairwells"

"stake"

"staked"

"stakeholder"

"stakeout"

"stakeouts"

"stakes"

"staking"

"stalactite"

"stalactites"

"stalag"

"stalagmite"

"stalagmites"

"stalagmometer"

"stalags"

"stale"

"staled"

"stalely"

"stalemate"

"stalemated"

"stalemates"

"stalemating"

"staleness"

"staler"

"stales"

"stalest"

"stalin"

"staling"

"stalingrad"

"stalinism"

"stalinist"

"stalinists"

"stalk"

"stalked"

"stalker"

"stalkers"

"stalkier"

"stalkiest"

"stalkily"

"stalking"

"stalkless"

"stalks"

"stalky"

"stall"

"stalled"

"stalling"

"stallion"

"stallions"

"stalls"

"stalwart"

"stalwartly"

"stalwarts"

"stamen"

"stamens"

"stamina"

"staminal"

"staminas"

"staminate"

"stammels"

"stammer"

"stammered"

"stammerer"

"stammerers"

"stammering"

"stammeringly"

"stammers"

"stamp"

"stamped"

"stampede"

"stampeded"

"stampedes"

"stampeding"

"stamper"

"stampers"

"stamping"

"stampomania"

"stamps"

"stance"

"stances"

"stanch"

"stanched"

"stancher"

"stanchers"

"stanches"

"stanchest"

"stanching"

"stanchion"

"stanchions"

"stanchly"

"stand"

"standard"

"standardbearer"

"standardbearers"

"standardizable"

"standardization"

"standardize"

"standardized"

"standardizes"

"standardizing"

"standards"

"standby"

"standbys"

"standee"

"standees"

"stander"

"standers"

"standing"

"standings"

"standish"

"standishes"

"standoff"

"standoffish"

"standoffs"

"standout"

"standouts"

"standpat"

"standpipe"

"standpipes"

"standpoint"

"standpoints"

"stands"

"standstill"

"standup"

"stanford"

"stank"

"stanley"

"stannic"

"stannous"

"stannum"

"stanza"

"stanzaed"

"stanzaic"

"stanzas"

"stapedes"

"stapes"

"staph"

"staphs"

"staphylococcal"

"staphylococcemia"

"staphylococcemic"

"staphylococci"

"staphylococcic"

"staphylococcus"

"staple"

"stapled"

"stapler"

"staplers"

"staples"

"stapling"

"star"

"starboard"

"starch"

"starched"

"starches"

"starchier"

"starchiest"

"starchiness"

"starching"

"starchy"

"stardom"

"stardoms"

"stardust"

"stardusts"

"stare"

"stared"

"starer"

"starers"

"stares"

"starfish"

"starfishes"

"stargaze"

"stargazed"

"stargazer"

"stargazers"

"stargazes"

"stargazing"

"staring"

"stark"

"starker"

"starkest"

"starkly"

"starkness"

"starless"

"starlet"

"starlets"

"starlight"

"starlike"

"starling"

"starlings"

"starlit"

"starred"

"starrier"

"starriest"

"starring"

"starry"

"stars"

"starship"

"start"

"started"

"starter"

"starters"

"starting"

"startle"

"startled"

"startler"

"startlers"

"startles"

"startling"

"startlingly"

"starts"

"starvation"

"starve"

"starved"

"starveling"

"starvelings"

"starver"

"starvers"

"starves"

"starving"

"stases"

"stash"

"stashed"

"stashes"

"stashing"

"stasiology"

"stasis"

"stat"

"statable"

"statal"

"state"

"stateable"

"statecraft"

"stated"

"statedly"

"statehood"

"statehouse"

"statehouses"

"stateless"

"statelessness"

"statelier"

"stateliest"

"stateliness"

"stately"

"statement"

"statements"

"stater"

"stateroom"

"staterooms"

"staters"

"states"

"stateside"

"statesman"

"statesmanlike"

"statesmanship"

"statesmen"

"stateswoman"

"stateswomen"

"statewide"

"static"

"statically"

"statice"

"statices"

"statics"

"statim"

"stating"

"station"

"stationary"

"stationed"

"stationer"

"stationeries"

"stationers"

"stationery"

"stationing"

"stations"

"statism"

"statisms"

"statist"

"statistc"

"statistic"

"Statistic report"

"statistical"

"statistically"

"statistician"

"statisticians"

"statistics"

"statists"

"stator"

"stators"

"statoscope"

"stats"

"statuaries"

"statuary"

"statue"

"statued"

"statues"

"statuesque"

"statuette"

"statuettes"

"stature"

"statures"

"status"

"statuses"

"statutable"

"statutableness"

"statutably"

"statute"

"statuted"

"statutes"

"statuting"

"statutorily"

"statutory"

"staunch"

"staunched"

"stauncher"

"staunches"

"staunchest"

"staunching"

"staunchly"

"staunchness"

"stauroscope"

"stave"

"staved"

"staves"

"staving"

"stay"

"stay motivated"

"stayed"

"stayer"

"stayers"

"staying"

"stays"

"staysail"

"staysails"

"stead"

"steaded"

"steadfast"

"steadfastly"

"steadfastness"

"steadied"

"steadier"

"steadiers"

"steadies"

"steadiest"

"steadily"

"steadiness"

"steading"

"steadings"

"steads"

"steady"

"steadying"

"steak"

"steaks"

"steal"

"stealable"

"stealer"

"stealers"

"stealing"

"stealings"

"steals"

"stealth"

"stealthier"

"stealthiest"

"stealthily"

"stealthiness"

"stealths"

"stealthy"

"steam"

"steamboat"

"steamboats"

"steamed"

"steamer"

"steamered"

"steamering"

"steamers"

"steamier"

"steamiest"

"steamily"

"steaming"

"steamroller"

"steamrollered"

"steamrollering"

"steamrollers"

"steams"

"steamship"

"Steamship Agent"

"steamships"

"steamy"

"stearic"

"stearin"

"steatite"

"steatopygia"

"steatopygic"

"steatopygous"

"stedhorses"

"steed"

"steeds"

"steel"

"steeled"

"steelie"

"steelier"

"steelies"

"steeliest"

"steeling"

"steels"

"steely"

"steelyard"

"steelyards"

"steep"

"steeped"

"steepen"

"steepened"

"steepening"

"steepens"

"steeper"

"steepers"

"steepest"

"steeping"

"steeple"

"steeplechase"

"steeplechases"

"steepled"

"steeplejack"

"steeplejacks"

"steeples"

"steeply"

"steepness"

"steeps"

"steer"

"steerable"

"steerage"

"steerages"

"steered"

"steerer"

"steerers"

"steering"

"steers"

"steersman"

"steersmen"

"steeve"

"stegosaur"

"stegosaurs"

"stein"

"steins"

"stele"

"stella"

"stellar"

"stellas"

"stellate"

"stellify"

"stem"

"stemless"

"stemmatology"

"stemmed"

"stemmer"

"stemmers"

"stemmier"

"stemmiest"

"stemming"

"stemmy"

"stems"

"stemware"

"stemwares"

"stench"

"stenches"

"stenchier"

"stenchiest"

"stenchy"

"stencil"

"stenciled"

"stenciling"

"stencilled"

"stencilling"

"stencils"

"steno"

"stenographer"

"stenographers"

"stenographic"

"stenographically"

"stenography"

"stenometer"

"stenos"

"stentor"

"stentorian"

"stentors"

"step"

"step by step"

"step counter"

"stepbrother"

"stepbrothers"

"stepchild"

"stepchildren"

"stepdames"

"stepdaughter"

"stepdaughters"

"stepdown"

"stepdowns"

"stepfather"

"stepfathers"

"stephen"

"stepladder"

"stepladders"

"stepmother"

"stepmothers"

"stepparent"

"stepparents"

"steppe"

"stepped"

"stepper"

"steppers"

"steppes"

"stepping"

"steppingstone"

"steppingstones"

"steps"

"stepsister"

"stepsisters"

"stepson"

"stepsons"

"stepup"

"stepups"

"stepwise"

"steradian"

"stere"

"stereo"

"stereochemical"

"stereochemistry"

"stereoed"

"stereograph"

"stereoing"

"stereoisomer"

"stereoisomeric"

"stereoisomerism"

"stereometer"

"stereophonic"

"stereophonically"

"stereos"

"stereoscope"

"stereoscopes"

"stereoscopic"

"stereoscopical"

"stereoscopically"

"stereoscopies"

"stereoscopy"

"stereospecific"

"stereotape"

"stereotapes"

"stereotype"

"stereotyped"

"stereotyper"

"stereotypers"

"stereotypes"

"stereotypical"

"stereotypies"

"stereotyping"

"steres"

"sterile"

"sterilely"

"sterilities"

"sterility"

"sterilization"

"sterilizations"

"sterilize"

"sterilized"

"sterilizer"

"sterilizers"

"sterilizes"

"sterilizing"

"sterling"

"sterlings"

"stern"

"sterna"

"sternal"

"sterner"

"sternest"

"sternly"

"sternness"

"sterns"

"sternum"

"sternums"

"sternutate"

"steroid"

"steroidal"

"steroids"

"sterols"

"stertorous"

"stertorously"

"stet"

"stethometer"

"stethoscope"

"stethoscopes"

"stethoscopic"

"stethoscopical"

"stethoscopically"

"stethoscopies"

"stethoscopy"

"stets"

"stetson"

"stetsons"

"stetted"

"stetting"

"steuben"

"steve"

"stevedore"

"stevedored"

"stevedores"

"stevedoring"

"steven"

"stew"

"steward"

"stewarded"

"stewardess"

"stewardesses"

"stewarding"

"stewards"

"stewardship"

"stewart"

"stewbum"

"stewbums"

"stewed"

"stewing"

"stewpan"

"stewpans"

"stews"

"stibium"

"stick"

"sticked"

"sticker"

"stickers"

"stickier"

"stickiest"

"stickily"

"stickiness"

"sticking"

"stickle"

"stickleback"

"stickled"

"stickler"

"sticklers"

"stickles"

"stickling"

"stickman"

"stickmen"

"stickouts"

"stickpin"

"stickpins"

"sticks"

"stickum"

"stickums"

"stickup"

"stickups"

"sticky"

"stied"

"sties"

"stiff"

"stiffed"

"stiffen"

"stiffened"

"stiffener"

"stiffeners"

"stiffening"

"stiffens"

"stiffer"

"stiffest"

"stiffing"

"stiffish"

"stiffly"

"stiffness"

"stiffs"

"stifle"

"stifled"

"stifler"

"stiflers"

"stifles"

"stifling"

"stiflingly"

"stigma"

"stigmas"

"stigmata"

"stigmatic"

"stigmatization"

"stigmatize"

"stigmatized"

"stigmatizes"

"stigmatizing"

"stilbestrol"

"stile"

"stiles"

"stiletted"

"stiletto"

"stilettoed"

"stilettoes"

"stilettos"

"still"

"stillatim"

"stillbirth"

"stillbirths"

"stillborn"

"stilled"

"stiller"

"stillest"

"stillier"

"stilliest"

"stilling"

"stillness"

"stills"

"stilly"

"stilt"

"stilted"

"stilting"

"stilton"

"stilts"

"stimied"

"stimies"

"stimulant"

"stimulants"

"stimulate"

"stimulated"

"stimulates"

"stimulating"

"stimulatingly"

"stimulation"

"stimulations"

"stimulative"

"stimulatives"

"stimulator"

"stimulatory"

"stimuli"

"stimulus"

"stimy"

"sting"

"stinger"

"stingers"

"stingier"

"stingiest"

"stingily"

"stinginess"

"stinging"

"stingingly"

"stingo"

"stingos"

"stingray"

"stingrays"

"stings"

"stingy"

"stink"

"stinkard"

"stinkards"

"stinkbug"

"stinkbugs"

"stinker"

"stinkers"

"stinkier"

"stinkiest"

"stinking"

"stinko"

"stinkpot"

"stinkpots"

"stinks"

"stinky"

"stint"

"stinted"

"stinter"

"stinters"

"stinting"

"stintingly"

"stints"

"stipend"

"stipendless"

"stipends"

"stipes"

"stipple"

"stippled"

"stippler"

"stipplers"

"stipples"

"stippling"

"stipulable"

"stipulate"

"stipulated"

"stipulates"

"stipulating"

"stipulation"

"stipulations"

"stipulator"

"stipulators"

"stipulatory"

"stir"

"stirred"

"stirrer"

"stirrers"

"stirring"

"stirringly"

"stirrup"

"stirrups"

"stirs"

"stitch"

"stitched"

"stitcher"

"stitchers"

"stitchery"

"stitches"

"stitching"

"stiver"

"stoa"

"stoas"

"stoat"

"stoats"

"stock"

"stockade"

"stockades"

"stockading"

"stockateer"

"stockbroker"

"stockbrokerage"

"stockbrokers"

"stockbroking"

"stockcar"

"stockcars"

"stocked"

"stocker"

"stockers"

"stockholder"

"stockholders"

"stockholding"

"stockholm"

"stockier"

"stockiest"

"stockily"

"stockiness"

"stockinet"

"stockinets"

"stockinette"

"stocking"

"stockings"

"stockish"

"stockists"

"stockjobber"

"stockjobbing"

"stockkeeper"

"stockman"

"stockmen"

"stockpile"

"stockpiled"

"stockpiles"

"stockpiling"

"stockpot"

"stockpots"

"stockroom"

"stockrooms"

"stocks"

"stocktaking"

"stocky"

"stockyard"

"stockyards"

"stodge"

"stodged"

"stodges"

"stodgier"

"stodgiest"

"stodgily"

"stodginess"

"stodging"

"stodgy"

"stogey"

"stogeys"

"stogie"

"stogies"

"stogy"

"stoic"

"stoical"

"stoically"

"stoichiology"

"stoicism"

"stoicisms"

"stoics"

"stoke"

"stoked"

"stoker"

"stokers"

"stokes"

"stoking"

"stole"

"stolen"

"stoles"

"stolid"

"stolider"

"stolidest"

"stolidity"

"stolidly"

"stollen"

"stollens"

"stolonic"

"stolons"

"stomach"

"stomach ache"

"stomachache"

"stomachaches"

"stomached"

"stomacher"

"stomachers"

"stomachic"

"stomachical"

"stomachically"

"stomaching"

"stomachs"

"stomachy"

"stomatology"

"stomp"

"stomped"

"stomper"

"stompers"

"stomping"

"stomps"

"stonable"

"stone"

"stonecutter"

"stonecutting"

"stoned"

"stoneflies"

"stonefly"

"stoner"

"stoners"

"stones"

"stonewall"

"stonewalled"

"stonewalling"

"stonewalls"

"stoneware"

"stonework"

"stoneworks"

"stoney"

"stonier"

"stoniest"

"stonily"

"stoniness"

"stoning"

"stonish"

"stonishing"

"stony"

"stood"

"stooge"

"stooged"

"stooges"

"stooging"

"stool"

"stooled"

"stoolie"

"stoolies"

"stooling"

"stools"

"stoop"

"stooped"

"stooper"

"stoopers"

"stooping"

"stoopingly"

"stoops"

"stop"

"Stop smoking"

"stopcock"

"stopcocks"

"stopgap"

"stopgaps"

"stoplight"

"stoplights"

"stopover"

"stopovers"

"stoppage"

"stoppages"

"stopped"

"stopper"

"stoppered"

"stoppering"

"stoppers"

"stopping"

"stopple"

"stoppled"

"stopples"

"stoppling"

"stops"

"stopt"

"stopwatch"

"stopwatches"

"storable"

"storables"

"storage"

"storage space"

"storages"

"store"

"Store multiple"

"stored"

"storefront"

"storefronts"

"storehouse"

"storehouses"

"storekeeper"

"storekeepers"

"storeroom"

"storerooms"

"stores"

"storewide"

"storey"

"storeyed"

"storeys"

"storied"

"stories"

"storing"

"storiology"

"stork"

"storks"

"storm"

"stormed"

"stormier"

"stormiest"

"stormily"

"storminess"

"storming"

"storms"

"stormy"

"story"

"storybook"

"storybooks"

"storying"

"storyline"

"storylines"

"storyteller"

"storytellers"

"storytelling"

"stoup"

"stoups"

"stout"

"stouten"

"stoutened"

"stoutening"

"stoutens"

"stouter"

"stoutest"

"stouthearted"

"stoutish"

"stoutly"

"stoutness"

"stouts"

"stove"

"stovepipe"

"stovepipes"

"stover"

"stovers"

"stoves"

"stow"

"stowable"

"stowage"

"stowages"

"stowaway"

"stowaways"

"stowed"

"stowing"

"stows"

"strabismally"

"strabismometer"

"strabismus"

"strabometer"

"straddle"

"straddled"

"straddler"

"straddlers"

"straddles"

"straddling"

"strafe"

"strafed"

"strafer"

"strafers"

"strafes"

"strafing"

"straggle"

"straggled"

"straggler"

"stragglers"

"straggles"

"stragglier"

"straggliest"

"straggling"

"straggly"

"straight"

"straightaway"

"straighted"

"straightedge"

"straightedges"

"straighten"

"straightened"

"straightener"

"straighteners"

"straightening"

"straightens"

"straighter"

"straightest"

"straightforward"

"straightforwardly"

"straightforwardness"

"straightjacket"

"straightly"

"straightness"

"straights"

"straightway"

"strain"

"strained"

"strainer"

"strainers"

"straining"

"strains"

"strait"

"straiten"

"straitened"

"straitening"

"straitens"

"straiter"

"straitest"

"straitjacket"

"straitlaced"

"straitly"

"straits"

"strand"

"stranded"

"strandedness"

"strander"

"stranders"

"stranding"

"strands"

"strange"

"strangely"

"strangeness"

"stranger"

"strangered"

"strangers"

"strangest"

"strangle"

"strangled"

"strangler"

"stranglers"

"strangles"

"strangling"

"stranglings"

"strangulate"

"strangulated"

"strangulates"

"strangulating"

"strangulation"

"strangulations"

"strap"

"strapless"

"strapped"

"strapper"

"strappers"

"strapping"

"straps"

"strata"

"stratagem"

"stratagems"

"stratas"

"strate"

"strategic"

"strategically"

"strategies"

"strategist"

"strategists"

"strategy"

"strath"

"stratification"

"stratifications"

"stratified"

"stratifies"

"stratify"

"stratifying"

"stratigraphic"

"stratigraphy"

"stratocumuli"

"stratocumulus"

"stratography"

"stratosphere"

"stratospheric"

"stratous"

"stratum"

"stratums"

"stratus"

"strauss"

"stravinsky"

"straw"

"strawberries"

"strawberry"

"strawed"

"strawhat"

"strawier"

"strawing"

"straws"

"strawy"

"stray"

"strayed"

"strayer"

"strayers"

"straying"

"strays"

"streak"

"streaked"

"streaker"

"streakers"

"streakier"

"streakiest"

"streakiness"

"streaking"

"streaks"

"streaky"

"stream"

"streamed"

"streamer"

"streamers"

"streamier"

"streamiest"

"streaming"

"streamlet"

"streamlets"

"streamline"

"streamlined"

"streamliner"

"streamliners"

"streamlines"

"streamlining"

"streams"

"streamy"

"street"

"streetcar"

"streetcars"

"streetlight"

"streets"

"streetwalker"

"streetwalkers"

"streetwalking"

"strenghten"

"strength"

"strengthen"

"strengthened"

"strengthener"

"strengtheners"

"strengthening"

"strengthens"

"strengths"

"strenuous"

"strenuously"

"strenuousness"

"strep"

"streps"

"streptobacilli"

"streptobacillus"

"streptococcal"

"streptococci"

"streptococcic"

"streptococcus"

"streptomycin"

"stress"

"stress disorder"

"Stress Relief"

"stress relief movement positions"

"stress symptom"

"stress symptoms"

"stress tested"

"stressed"

"stresses"

"stressful"

"stressing"

"stressor"

"stressors"

"stretch"

"Stretch Exercises"

"stretchable"

"stretched"

"stretcher"

"stretchers"

"stretches"

"stretchier"

"stretchiest"

"stretching"

"stretching program"

"stretchy"

"stretti"

"stretto"

"strettos"

"streusel"

"strew"

"strewed"

"strewer"

"strewers"

"strewing"

"strewn"

"strews"

"stria"

"striae"

"striate"

"striated"

"striates"

"striating"

"striation"

"striations"

"stricken"

"strickenly"

"strickled"

"strickles"

"strict"

"stricter"

"strictest"

"strictly"

"strictness"

"stricture"

"strictured"

"strictures"

"stridden"

"stride"

"stridency"

"strident"

"stridently"

"strider"

"striders"

"strides"

"striding"

"stridor"

"strife"

"strifes"

"strike"

"strikebreaker"

"strikebreakers"

"strikebreaking"

"strikeout"

"strikeouts"

"strikeover"

"striker"

"strikers"

"strikes"

"striking"

"strikingly"

"string"

"stringed"

"stringency"

"stringent"

"stringently"

"stringer"

"stringers"

"stringier"

"stringiest"

"stringiness"

"stringing"

"stringless"

"strings"

"stringy"

"strip"

"stripe"

"striped"

"striper"

"stripers"

"stripes"

"stripier"

"stripiest"

"striping"

"stripings"

"stripling"

"striplings"

"stripped"

"stripper"

"strippers"

"stripping"

"strips"

"stript"

"striptease"

"stripteased"

"stripteaser"

"stripteasers"

"stripteases"

"stripteasing"

"stripy"

"strive"

"strived"

"striven"

"striver"

"strivers"

"strives"

"striving"

"strobe"

"strobes"

"strobic"

"strobilization"

"stroboscope"

"stroboscopes"

"stroboscopic"

"stroboscopically"

"strode"

"stroganoff"

"stroke"

"stroked"

"stroker"

"strokers"

"strokes"

"stroking"

"stroll"

"Stroll Along the Creek"

"strolled"

"stroller"

"strollers"

"strolling"

"strolls"

"strong"

"strongarmer"

"strongbox"

"strongboxes"

"stronger"

"strongest"

"stronghold"

"strongholds"

"strongly"

"strongman"

"strongmen"

"strongroom"

"strongrooms"

"strongyle"

"strontium"

"strop"

"strophe"

"strophes"

"strophic"

"stropped"

"stropping"

"strops"

"strove"

"struck"

"structural"

"structurally"

"structure"

"structured"

"structures"

"structuring"

"strudel"

"strudels"

"struggle"

"struggled"

"struggler"

"strugglers"

"struggles"

"struggling"

"strum"

"strummed"

"strummer"

"strummers"

"strumming"

"strumpet"

"strumpets"

"strums"

"strung"

"strut"

"struts"

"strutted"

"strutter"

"strutters"

"strutting"

"strychnine"

"strychninism"

"strychninization"

"stub"

"stubbed"

"stubbier"

"stubbiest"

"stubbily"

"stubbiness"

"stubbing"

"stubble"

"stubbled"

"stubbles"

"stubblier"

"stubbliest"

"stubbly"

"stubborn"

"stubborner"

"stubbornest"

"stubbornly"

"stubbornness"

"stubby"

"stubs"

"stucco"

"stuccoed"

"stuccoer"

"stuccoers"

"stuccoes"

"stuccoing"

"stuccos"

"stuccowork"

"stuck"

"stud"

"studbook"

"studbooks"

"studded"

"studding"

"studdings"

"student"

"students"

"studhorse"

"studhorses"

"studied"

"studiedly"

"studier"

"studiers"

"studies"

"studio"

"studios"

"studious"

"studiously"

"studiousness"

"studs"

"study"

"studying"

"stuff"

"stuffed"

"stuffer"

"stuffers"

"stuffier"

"stuffiest"

"stuffily"

"stuffiness"

"stuffing"

"stuffings"

"stuffs"

"stuffy"

"stultification"

"stultified"

"stultifies"

"stultify"

"stultifying"

"stumble"

"stumbled"

"stumbler"

"stumblers"

"stumbles"

"stumbling"

"stumblingly"

"stump"

"stumped"

"stumper"

"stumpers"

"stumpier"

"stumpiest"

"stumping"

"stumps"

"stumpy"

"stun"

"stung"

"stunk"

"stunned"

"stunner"

"stunners"

"stunning"

"stuns"

"stunsail"

"stunt"

"stunted"

"stuntedness"

"stunting"

"stunts"

"stupa"

"stupas"

"stupe"

"stupefacient"

"stupefaction"

"stupefactive"

"stupefied"

"stupefies"

"stupefy"

"stupefying"

"stupendous"

"stupendously"

"stupes"

"stupid"

"stupider"

"stupidest"

"stupidity"

"stupidly"

"stupids"

"stupor"

"stuporous"

"stupors"

"sturdier"

"sturdiest"

"sturdily"

"sturdiness"

"sturdy"

"sturgeon"

"sturgeons"

"stutter"

"stuttered"

"stutterer"

"stutterers"

"stuttering"

"stutteringly"

"stutters"

"sty"

"stye"

"styed"

"styes"

"stygian"

"stylar"

"stylate"

"style"

"stylebook"

"stylebooks"

"styled"

"styleless"

"styler"

"stylers"

"styles"

"stylets"

"styli"

"styling"

"stylings"

"stylise"

"stylish"

"stylishly"

"stylishness"

"stylist"

"stylistic"

"stylistically"

"stylists"

"stylite"

"stylize"

"stylized"

"stylizer"

"stylizers"

"stylizes"

"stylizing"

"stylometer"

"stylometry"

"stylus"

"styluses"

"stymie"

"stymied"

"stymieing"

"stymies"

"stymy"

"stymying"

"stypsis"

"styptic"

"styptics"

"styrene"

"styrofoam"

"styx"

"suability"

"suable"

"suably"

"suasion"

"suasions"

"suasive"

"suasively"

"suave"

"suavely"

"suaveness"

"suaver"

"suavest"

"suavities"

"suavity"

"sub"

"subabbot"

"subabbots"

"subacute"

"subacutely"

"subagencies"

"subagency"

"subagent"

"subagents"

"subahdars"

"suballiance"

"suballiances"

"subalpine"

"subaltern"

"subalterns"

"subaqueous"

"subarea"

"subareas"

"subassemblies"

"subassembly"

"subassociation"

"subassociations"

"subatomic"

"subaverage"

"subbasement"

"subbasements"

"subbass"

"subbed"

"subbing"

"subbings"

"subbranch"

"subbranches"

"subbreed"

"subbreeds"

"subcategories"

"subcategory"

"subcauses"

"subcell"

"subcellar"

"subcellars"

"subcells"

"subcellular"

"subchapter"

"subchapters"

"subchief"

"subchiefs"

"subcivilization"

"subcivilizations"

"subclan"

"subclass"

"subclassed"

"subclasses"

"subclassification"

"subclassifications"

"subclassified"

"subclassifies"

"subclassify"

"subclassifying"

"subclause"

"subclauses"

"subclerks"

"subclinical"

"subclinically"

"subcommander"

"subcommanders"

"subcommission"

"subcommissioner"

"subcommissioners"

"subcommissions"

"subcommittee"

"subcommittees"

"subcompact"

"subcompacts"

"subconscious"

"subconsciously"

"subconsciousness"

"subcontinent"

"subcontinental"

"subcontinents"

"subcontract"

"subcontracted"

"subcontracting"

"subcontractor"

"subcontractors"

"subcontracts"

"subcouncil"

"subcouncils"

"subcranial"

"Subcription service"

"subculture"

"subcultures"

"subcurator"

"subcurators"

"subcutaneous"

"subcutaneously"

"subdeacon"

"subdeacons"

"subdeb"

"subdebs"

"subdebutante"

"subdebutantes"

"subdefinition"

"subdefinitions"

"subdepartment"

"subdepartmental"

"subdepartments"

"subdepot"

"subdepots"

"subdermal"

"subdialect"

"subdialects"

"subdirector"

"subdirectories"

"subdirectors"

"subdirectory"

"subdiscipline"

"subdisciplines"

"subdistinction"

"subdistinctions"

"subdistrict"

"subdistricts"

"subdividable"

"subdivide"

"subdivided"

"subdivider"

"subdivides"

"subdividing"

"subdivisible"

"subdivision"

"subdivisions"

"subdual"

"subduals"

"subdue"

"subdued"

"subduer"

"subduers"

"subdues"

"subduing"

"subeditor"

"subeditors"

"subendorsed"

"subendorsing"

"subentries"

"subentry"

"subfamilies"

"subfamily"

"subfloor"

"subfloors"

"subfraction"

"subfractional"

"subfractions"

"subfreezing"

"subfunction"

"subfunctions"

"subgenera"

"subgenus"

"subgenuses"

"subglacial"

"subgrades"

"subgroup"

"subgroups"

"subgum"

"subhead"

"subheading"

"subheadings"

"subheads"

"subhuman"

"subhumans"

"subindexes"

"subindices"

"subitem"

"subitems"

"subito"

"subjacent"

"subject"

"subjected"

"subjecting"

"subjection"

"subjective"

"subjectively"

"subjectiveness"

"subjectivity"

"subjects"

"subjoin"

"subjoined"

"subjoining"

"subjoins"

"subjugate"

"subjugated"

"subjugates"

"subjugating"

"subjugation"

"subjugator"

"subjugators"

"subjunctive"

"subjunctives"

"subkingdom"

"subkingdoms"

"sublease"

"subleased"

"subleases"

"subleasing"

"sublessee"

"sublessor"

"sublet"

"sublethal"

"sublets"

"subletting"

"sublevel"

"sublevels"

"sublicensed"

"sublicensee"

"sublicenses"

"sublimate"

"sublimated"

"sublimates"

"sublimating"

"sublimation"

"sublimations"

"sublime"

"sublimed"

"sublimely"

"sublimeness"

"sublimer"

"sublimers"

"sublimes"

"sublimest"

"subliminal"

"subliminally"

"subliming"

"sublimities"

"sublimity"

"sublunar"

"sublunary"

"subluxation"

"submachine"

"submarginal"

"submarine"

"submarines"

"submember"

"submembers"

"submental"

"submerge"

"submerged"

"submergence"

"submergences"

"submerges"

"submergibility"

"submergible"

"submerging"

"submerse"

"submersed"

"submerses"

"submersibility"

"submersible"

"submersibles"

"submersing"

"submersion"

"submersions"

"submicroscopic"

"subminiature"

"subminiaturization"

"subminiaturize"

"subminiaturized"

"subminiaturizes"

"subminiaturizing"

"submiss"

"submission"

"submissions"

"submissive"

"submissively"

"submissiveness"

"submit"

"submits"

"submittal"

"submittance"

"submitted"

"submitter"

"submitting"

"submolecular"

"submontane"

"subnormal"

"subnormality"

"subnormally"

"subnuclei"

"subnucleus"

"subnucleuses"

"suboffice"

"subofficer"

"subofficers"

"suboffices"

"suborbital"

"suborder"

"suborders"

"subordinate"

"subordinated"

"subordinately"

"subordinates"

"subordinating"

"subordination"

"subordinations"

"suborn"

"subornation"

"subornations"

"suborned"

"suborner"

"suborners"

"suborning"

"suborns"

"suboxides"

"subparagraph"

"subparagraphs"

"subpartnership"

"subparts"

"subpena"

"subpenaing"

"subpenas"

"subphyla"

"subphylum"

"subplot"

"subplots"

"subpoena"

"subpoenaed"

"subpoenaing"

"subpoenal"

"subpoenas"

"subprincipal"

"subprincipals"

"subprocess"

"subprogram"

"subprovince"

"subprovinces"

"subrace"

"subraces"

"subregion"

"subregions"

"subrents"

"subroutine"

"subroutines"

"subrule"

"subrules"

"subs"

"subschedule"

"subschedules"

"subscribe"

"subscribed"

"subscriber"

"subscribers"

"subscribes"

"subscribing"

"subscript"

"subscripted"

"subscripting"

"subscription"

"subscriptions"

"subscripts"

"subsection"

"subsections"

"subsegment"

"subsegments"

"subsequent"

"subsequential"

"subsequently"

"subseries"

"subservience"

"subserviency"

"subservient"

"subserviently"

"subserving"

"subset"

"subsets"

"subside"

"subsided"

"subsidence"

"subsider"

"subsiders"

"subsides"

"subsidiaries"

"subsidiary"

"subsidies"

"subsiding"

"subsidizable"

"subsidization"

"subsidizations"

"subsidize"

"subsidized"

"subsidizes"

"subsidizing"

"subsidy"

"subsist"

"subsisted"

"subsistence"

"subsisting"

"subsists"

"subsoil"

"subsoiling"

"subsoils"

"subsonic"

"subspace"

"subspecies"

"subspecific"

"subspecifically"

"substage"

"substance"

"substanceless"

"substances"

"substandard"

"substantiable"

"substantiae"

"substantial"

"substantiality"

"substantialize"

"substantialized"

"substantializing"

"substantially"

"substantialness"

"substantiate"

"substantiated"

"substantiates"

"substantiating"

"substantiation"

"substantiations"

"substantiator"

"substantival"

"substantive"

"substantively"

"substantiveness"

"substantives"

"substation"

"substations"

"substitutabilities"

"substitutability"

"substitute"

"substituted"

"substituter"

"substitutes"

"substituting"

"substitution"

"substitutional"

"substitutionary"

"substitutions"

"substitutive"

"substrata"

"substrate"

"substratum"

"substratums"

"substring"

"substructure"

"substructures"

"subsumable"

"subsume"

"subsumed"

"subsumes"

"subsuming"

"subsurface"

"subsurfaces"

"subsystem"

"subsystems"

"subtask"

"subtasks"

"subteen"

"subteens"

"subtenancies"

"subtenancy"

"subtenant"

"subtenants"

"subtend"

"subtended"

"subtending"

"subtends"

"subterfuge"

"subterfuges"

"subterranean"

"subterraneously"

"subthreshold"

"subtile"

"subtilest"

"subtitle"

"subtitled"

"subtitles"

"subtitling"

"subtle"

"subtleness"

"subtler"

"subtlest"

"subtleties"

"subtlety"

"subtly"

"subtones"

"subtonic"

"subtopic"

"subtopics"

"subtotal"

"subtotaled"

"subtotaling"

"subtotalled"

"subtotalling"

"subtotals"

"subtract"

"subtracted"

"subtracting"

"subtraction"

"subtractions"

"subtracts"

"subtrahend"

"subtrahends"

"subtreasuries"

"subtreasury"

"subtribe"

"subtropical"

"subtype"

"subtypes"

"subunit"

"subunits"

"suburb"

"suburban"

"suburbanite"

"suburbanites"

"suburbans"

"suburbed"

"suburbia"

"suburbias"

"suburbs"

"subvaluation"

"subvarieties"

"subvariety"

"subvention"

"subventions"

"subversion"

"subversions"

"subversive"

"subversively"

"subversives"

"subvert"

"subverted"

"subverter"

"subverters"

"subvertible"

"subverting"

"subverts"

"subvocal"

"subway"

"subways"

"succeed"

"succeeded"

"succeeder"

"succeeders"

"succeeding"

"succeeds"

"success"

"successes"

"successful"

"successfully"

"succession"

"successional"

"successions"

"successive"

"successively"

"successor"

"successors"

"successorship"

"succinct"

"succinctly"

"succinctness"

"succor"

"succored"

"succorer"

"succorers"

"succories"

"succoring"

"succors"

"succotash"

"succour"

"succoured"

"succouring"

"succours"

"succuba"

"succubi"

"succubus"

"succubuses"

"succulence"

"succulency"

"succulent"

"succulently"

"succulents"

"succumb"

"succumbed"

"succumber"

"succumbers"

"succumbing"

"succumbs"

"such"

"suchlike"

"suchness"

"suck"

"sucked"

"sucker"

"suckered"

"suckering"

"suckers"

"sucking"

"suckle"

"suckled"

"suckler"

"sucklers"

"suckles"

"suckling"

"sucklings"

"sucks"

"sucre"

"sucres"

"sucrose"

"sucroses"

"suction"

"suctional"

"suctions"

"suctorial"

"sudan"

"sudanese"

"sudden"

"suddenly"

"suddenness"

"suddens"

"sudor"

"sudoral"

"sudorific"

"sudors"

"suds"

"sudsed"

"sudser"

"sudsers"

"sudses"

"sudsier"

"sudsiest"

"sudsing"

"sudsless"

"sudsy"

"sue"

"sued"

"suede"

"sueded"

"suedes"

"sueding"

"suer"

"suers"

"sues"

"suet"

"suets"

"suety"

"suey"

"suez"

"suffer"

"sufferable"

"sufferance"

"suffered"

"sufferer"

"sufferers"

"suffering"

"sufferingly"

"sufferings"

"suffers"

"suffice"

"sufficed"

"sufficer"

"sufficers"

"suffices"

"sufficiencies"

"sufficiency"

"sufficient"

"sufficiently"

"sufficing"

"suffix"

"suffixal"

"suffixed"

"suffixes"

"suffixing"

"suffixion"

"sufflated"

"sufflates"

"suffocate"

"suffocated"

"suffocates"

"suffocating"

"suffocatingly"

"suffocation"

"suffragan"

"suffragans"

"suffrage"

"suffrages"

"suffragette"

"suffragettes"

"suffragist"

"suffragists"

"suffuse"

"suffused"

"suffuses"

"suffusing"

"suffusion"

"suffusions"

"sugar"

"sugarcane"

"sugarcoat"

"sugarcoated"

"sugarcoating"

"sugarcoats"

"sugared"

"sugarier"

"sugariest"

"sugariness"

"sugaring"

"sugarless"

"sugarplum"

"sugarplums"

"sugars"

"sugary"

"suggest"

"suggested"

"suggestibility"

"suggestible"

"suggesting"

"suggestion"

"suggestions"

"suggestive"

"suggestively"

"suggestiveness"

"suggests"

"sui"

"suicidal"

"suicidally"

"suicide"

"suicided"

"suicides"

"suiciding"

"suicidology"

"suing"

"suit"

"suitability"

"suitable"

"suitableness"

"suitably"

"suitcase"

"suitcases"

"suite"

"suited"

"suites"

"suiting"

"suitings"

"suitor"

"suitors"

"suits"

"sukiyaki"

"sukiyakis"

"sulfa"

"sulfanilamide"

"sulfas"

"sulfate"

"sulfates"

"sulfating"

"sulfide"

"sulfides"

"sulfids"

"sulfite"

"sulfites"

"sulfur"

"sulfured"

"sulfureous"

"sulfuric"

"sulfuring"

"sulfurize"

"sulfurized"

"sulfurous"

"sulfurs"

"sulfury"

"sulfuryls"

"sulk"

"sulked"

"sulker"

"sulkers"

"sulkier"

"sulkies"

"sulkiest"

"sulkily"

"sulkiness"

"sulking"

"sulks"

"sulky"

"sullen"

"sullener"

"sullenest"

"sullenly"

"sullenness"

"sullied"

"sullies"

"sully"

"sullying"

"sulpha"

"sulphas"

"sulphate"

"sulphates"

"sulphid"

"sulphide"

"sulphur"

"sulphured"

"sulphuring"

"sulphurize"

"sulphurizing"

"sulphurs"

"sulphury"

"sultan"

"sultana"

"sultanas"

"sultanate"

"sultanates"

"sultanic"

"sultans"

"sultrier"

"sultriest"

"sultrily"

"sultriness"

"sultry"

"sum"

"sumac"

"sumach"

"sumachs"

"sumacs"

"sumatra"

"sumatran"

"sumatrans"

"summa"

"summable"

"summaries"

"summarily"

"summarization"

"summarizations"

"summarize"

"summarized"

"summarizes"

"summarizing"

"summary"

"summating"

"summation"

"summations"

"summed"

"summer"

"summered"

"summerhouse"

"summerhouses"

"summerier"

"summeriest"

"summering"

"summerly"

"summers"

"summertime"

"summery"

"summing"

"summings"

"summit"

"summital"

"summitry"

"summits"

"summon"

"summoned"

"summoner"

"summoners"

"summoning"

"summons"

"summonsed"

"summonses"

"sumo"

"sumos"

"sump"

"sumps"

"sumpter"

"sumpters"

"sumptuous"

"sumptuously"

"sumptuousness"

"sums"

"sun"

"sunback"

"sunbaked"

"sunbath"

"sunbathe"

"sunbathed"

"sunbather"

"sunbathers"

"sunbathes"

"sunbathing"

"sunbaths"

"sunbeam"

"sunbeams"

"sunbelt"

"sunbird"

"sunbirds"

"sunbonnet"

"sunbonnets"

"sunbow"

"sunbows"

"sunburn"

"sunburned"

"sunburning"

"sunburns"

"sunburnt"

"sunburst"

"sunbursts"

"sundae"

"sundaes"

"sunday"

"sundays"

"sunder"

"sundered"

"sunderer"

"sunderers"

"sundering"

"sunders"

"sundew"

"sundews"

"sundial"

"sundials"

"sundog"

"sundogs"

"sundown"

"sundowns"

"sundries"

"sundrops"

"sundry"

"sunfish"

"sunfishes"

"sunflower"

"sunflowers"

"sung"

"sunglass"

"sunglasses"

"sunglow"

"sunk"

"sunken"

"sunlamp"

"sunlamps"

"sunless"

"sunlight"

"sunlights"

"sunlit"

"sunned"

"sunnier"

"sunniest"

"sunnily"

"sunniness"

"sunning"

"sunny"

"Sunny Beach"

"sunrise"

"sunrises"

"sunroof"

"sunroofs"

"sunroom"

"sunrooms"

"suns"

"sunset"

"sunsets"

"sunshade"

"sunshades"

"sunshine"

"sunshines"

"sunshiny"

"sunspot"

"sunspots"

"sunstones"

"sunstroke"

"sunstrokes"

"sunstruck"

"sunsuit"

"sunsuits"

"suntan"

"suntanned"

"suntans"

"sunup"

"sunups"

"sunward"

"sunwards"

"sunwise"

"sup"

"supe"

"super"

"superabundance"

"superabundant"

"superabundantly"

"superannuate"

"superannuated"

"superannuating"

"superannuation"

"superannuity"

"superb"

"superber"

"superbly"

"supercargo"

"supercargoes"

"supercargos"

"supercede"

"superceded"

"supercedes"

"superceding"

"supercharge"

"supercharged"

"supercharger"

"superchargers"

"supercharges"

"supercharging"

"supercilious"

"superciliously"

"superciliousness"

"supercomputer"

"supercomputers"

"superconductivity"

"superconductor"

"superconductors"

"supered"

"superego"

"superegos"

"supereminent"

"supererogation"

"supererogatory"

"superficial"

"superficialities"

"superficiality"

"superficially"

"superficialness"

"superficiary"

"superficies"

"superfluities"

"superfluity"

"superfluous"

"superfluously"

"superfluousness"

"superhighway"

"superhighways"

"superhuman"

"superimpose"

"superimposed"

"superimposes"

"superimposing"

"superimposition"

"superimpositions"

"supering"

"superintend"

"superintended"

"superintendence"

"superintendency"

"superintendent"

"superintendents"

"superintending"

"superintends"

"superior"

"superiorities"

"superiority"

"superiorly"

"superiors"

"superjets"

"superlative"

"superlatively"

"superlativeness"

"superlatives"

"superman"

"supermarket"

"supermarkets"

"Supermarkt"

"supermen"

"supermini"

"superminis"

"supermolecular"

"supermolecule"

"supernal"

"supernational"

"supernationalism"

"supernationalisms"

"supernatural"

"supernaturally"

"supernaturalness"

"supernormal"

"supernova"

"supernovas"

"supernumeraries"

"supernumerary"

"superposable"

"superpose"

"superposed"

"superposes"

"superposing"

"superposition"

"superpositions"

"superpower"

"superpowers"

"supers"

"supersaturate"

"supersaturated"

"supersaturates"

"supersaturating"

"supersaturation"

"superscribe"

"superscribed"

"superscribes"

"superscribing"

"superscript"

"superscripted"

"superscripting"

"superscription"

"superscriptions"

"superscripts"

"supersecret"

"supersede"

"superseded"

"supersedence"

"superseder"

"supersedes"

"superseding"

"supersedure"

"supersensitive"

"supersession"

"supersessive"

"supersex"

"supersexes"

"supersonic"

"supersonically"

"supersonics"

"superstition"

"superstitions"

"superstitious"

"superstitiously"

"superstructure"

"superstructures"

"supertanker"

"supertaxes"

"supervene"

"supervened"

"supervenes"

"supervening"

"supervention"

"supervisal"

"supervise"

"supervised"

"supervisee"

"supervises"

"supervising"

"supervision"

"supervisor"

"supervisorial"

"supervisors"

"supervisorship"

"supervisory"

"supes"

"supinate"

"supinated"

"supinates"

"supinating"

"supinator"

"supine"

"supinely"

"supineness"

"supines"

"suporvisory"

"supped"

"supper"

"supperless"

"suppers"

"suppertime"

"supping"

"supplant"

"supplantation"

"supplanted"

"supplanter"

"supplanters"

"supplanting"

"supplants"

"supple"

"supplely"

"supplement"

"supplemental"

"supplementally"

"supplementals"

"supplementarily"

"supplementary"

"supplementation"

"supplemented"

"supplementer"

"supplementing"

"supplements"

"suppleness"

"suppler"

"supplest"

"suppliable"

"suppliance"

"suppliant"

"suppliants"

"supplicant"

"supplicants"

"supplicate"

"supplicated"

"supplicates"

"supplicating"

"supplication"

"supplications"

"supplied"

"supplier"

"suppliers"

"supplies"

"supply"

"supplying"

"support"

"support tool"

"supportable"

"supportance"

"supported"

"supporter"

"supporters"

"supporting"

"supportive"

"supportless"

"supports"

"Supports All Screen Orientations"

"suppose"

"supposed"

"supposedly"

"supposer"

"supposers"

"supposes"

"supposing"

"supposition"

"suppositional"

"suppositions"

"suppositive"

"suppositories"

"suppository"

"suppress"

"suppressant"

"suppressants"

"suppressed"

"suppresses"

"suppressible"

"suppressing"

"suppression"

"suppressions"

"suppressive"

"suppurate"

"suppurated"

"suppurates"

"suppurating"

"suppuration"

"suppurations"

"suppurative"

"supra"

"supraliminal"

"supraliminally"

"supramental"

"supranational"

"supraorbital"

"supremacist"

"supremacists"

"supremacy"

"supreme"

"supremely"

"supremeness"

"supremer"

"supremest"

"sups"

"supt"

"suq"

"surcease"

"surceased"

"surceases"

"surceasing"

"surcharge"

"surcharged"

"surcharger"

"surchargers"

"surcharges"

"surcharging"

"surcingle"

"surcingles"

"surcoat"

"surcoats"

"surds"

"sure"

"surefire"

"surefooted"

"surefootedness"

"surely"

"sureness"

"surer"

"surest"

"sureties"

"surety"

"surf"

"surfable"

"surface"

"surfaced"

"surfacer"

"surfacers"

"surfaces"

"surfacing"

"surfboard"

"surfboards"

"surfed"

"surfeit"

"surfeited"

"surfeiting"

"surfeits"

"surfer"

"surfers"

"surffish"

"surffishes"

"surfier"

"surfiest"

"surfing"

"surfings"

"surfs"

"surfy"

"surge"

"surged"

"surgeon"

"surgeons"

"surger"

"surgeries"

"surgers"

"surgery"

"surges"

"surgical"

"surgically"

"surging"

"surgy"

"surinam"

"surlier"

"surliest"

"surlily"

"surliness"

"surly"

"surmisable"

"surmise"

"surmised"

"surmiser"

"surmisers"

"surmises"

"surmising"

"surmount"

"surmountable"

"surmounted"

"surmounting"

"surmounts"

"surname"

"surnamed"

"surnamer"

"surnamers"

"surnames"

"surnaming"

"surpass"

"surpassable"

"surpassed"

"surpasses"

"surpassing"

"surpassingly"

"surplice"

"surplices"

"surplus"

"surplusage"

"surpluses"

"surprints"

"surprise"

"surprised"

"surpriser"

"surprisers"

"surprises"

"surprising"

"surprisingly"

"surprize"

"surprized"

"surprizes"

"surprizing"

"surreal"

"surrealism"

"surrealist"

"surrealistic"

"surrealistically"

"surrealists"

"surrejoinder"

"surrejoinders"

"surrender"

"surrendered"

"surrenderee"

"surrendering"

"surrenderor"

"surrenders"

"surreptitious"

"surreptitiously"

"surreptitiousness"

"surrey"

"surreys"

"surrogacies"

"surrogacy"

"surrogate"

"surrogates"

"surround"

"surrounded"

"surrounding"

"surroundings"

"surrounds"

"surtax"

"surtaxed"

"surtaxes"

"surtaxing"

"surveil"

"surveiled"

"surveiling"

"surveillance"

"surveillant"

"surveils"

"survey"

"surveyable"

"surveyance"

"surveyed"

"surveying"

"surveyor"

"surveyors"

"surveys"

"survivability"

"survivable"

"survival"

"survivals"

"survive"

"survived"

"surviver"

"survivers"

"survives"

"surviving"

"survivor"

"survivors"

"survivorship"

"susan"

"susans"

"susceptibilities"

"susceptibility"

"susceptible"

"susceptibleness"

"susceptibly"

"susceptiveness"

"suspect"

"suspectable"

"suspected"

"suspectedly"

"suspectedness"

"suspecter"

"suspecting"

"suspects"

"suspend"

"suspended"

"suspender"

"suspenders"

"suspending"

"suspends"

"suspense"

"suspenseful"

"suspenses"

"suspension"

"suspensions"

"suspensive"

"suspensory"

"suspicion"

"suspicions"

"suspicious"

"suspiciously"

"suspiciousness"

"suspire"

"sustain"

"sustainable"

"sustained"

"sustaining"

"sustainment"

"sustains"

"sustenance"

"sustenant"

"susurration"

"susurrations"

"susurrus"

"susurruses"

"sutler"

"sutlers"

"sutra"

"sutras"

"sutta"

"suttas"

"suttee"

"suttees"

"sutural"

"suture"

"sutured"

"sutures"

"suturing"

"suzanne"

"suzerain"

"suzerains"

"suzerainty"

"suzette"

"suzettes"

"suzuki"

"svedberg"

"svelte"

"sveltely"

"svelter"

"sveltest"

"swab"

"swabbed"

"swabber"

"swabbers"

"swabbie"

"swabbies"

"swabbing"

"swabby"

"swabs"

"swaddle"

"swaddled"

"swaddles"

"swaddling"

"swag"

"swage"

"swaged"

"swages"

"swagged"

"swagger"

"swaggered"

"swaggerer"

"swaggerers"

"swaggering"

"swaggers"

"swagging"

"swaging"

"swagman"

"swagmen"

"swahili"

"swahilian"

"swail"

"swain"

"swainish"

"swains"

"swale"

"swallow"

"swallowed"

"swallowing"

"swallows"

"swallowtail"

"swallowtails"

"swam"

"swami"

"swamies"

"swamis"

"swamp"

"swamped"

"swamper"

"swampers"

"swampier"

"swampiest"

"swampiness"

"swamping"

"swampish"

"swampland"

"swamps"

"swampy"

"swan"

"swang"

"swanherd"

"swanherds"

"swank"

"swanked"

"swanker"

"swankest"

"swankier"

"swankiest"

"swankily"

"swanking"

"swanks"

"swanky"

"swanned"

"swannery"

"swanning"

"swans"

"swansdown"

"swanskins"

"swap"

"swapped"

"swapper"

"swappers"

"swapping"

"swaps"

"sward"

"swards"

"swarm"

"swarmed"

"swarmer"

"swarmers"

"swarming"

"swarms"

"swart"

"swarth"

"swarthier"

"swarthiest"

"swarthiness"

"swarthy"

"swarty"

"swash"

"swashbuckler"

"swashbucklers"

"swashbuckling"

"swashed"

"swasher"

"swashers"

"swashes"

"swashing"

"swastika"

"swastikas"

"swat"

"swatch"

"swatches"

"swath"

"swathe"

"swathed"

"swather"

"swathers"

"swathes"

"swathing"

"swaths"

"swats"

"swatted"

"swatter"

"swatters"

"swatting"

"sway"

"swayable"

"swayback"

"swaybacked"

"swaybacks"

"swayed"

"swayer"

"swayers"

"swaying"

"sways"

"swaziland"

"swear"

"swearer"

"swearers"

"swearing"

"swears"

"swearword"

"sweat"

"sweatband"

"sweatbox"

"sweatboxes"

"sweated"

"sweater"

"sweaters"

"sweatier"

"sweatiest"

"sweatily"

"sweating"

"sweats"

"sweatshirt"

"sweatshop"

"sweatshops"

"sweaty"

"swede"

"sweden"

"swedes"

"sweep"

"sweeper"

"sweepers"

"sweepier"

"sweepiest"

"sweeping"

"sweepingly"

"sweepings"

"sweeps"

"sweepstake"

"sweepstakes"

"sweepy"

"sweet"

"sweetbread"

"sweetbreads"

"sweetbrier"

"sweetbriers"

"sweeten"

"sweetened"

"sweetener"

"sweeteners"

"sweetening"

"sweetens"

"sweeter"

"sweetest"

"sweetheart"

"sweethearts"

"sweetie"

"sweeties"

"sweeting"

"sweetings"

"sweetish"

"sweetly"

"sweetmeat"

"sweetmeats"

"sweetness"

"sweets"

"sweetsop"

"sweetsops"

"swell"

"swelled"

"sweller"

"swellest"

"swellhead"

"swellheaded"

"swellheads"

"swelling"

"swellings"

"swells"

"swelter"

"sweltered"

"sweltering"

"swelters"

"sweltrier"

"sweltriest"

"swept"

"sweptback"

"swerve"

"swerved"

"swerver"

"swervers"

"swerves"

"swerving"

"swift"

"swifter"

"swifters"

"swiftest"

"swiftian"

"swiftly"

"swiftness"

"swifts"

"swig"

"swigged"

"swigger"

"swiggers"

"swigging"

"swigs"

"swill"

"swilled"

"swiller"

"swillers"

"swilling"

"swills"

"swim"

"swimmable"

"swimmer"

"swimmers"

"swimmier"

"swimmiest"

"swimmily"

"swimming"

"swimmingly"

"swimmings"

"swimmy"

"swims"

"swimsuit"

"swimsuits"

"swindle"

"swindleable"

"swindled"

"swindler"

"swindlers"

"swindles"

"swindling"

"swine"

"swing"

"swinge"

"swinged"

"swingeing"

"swinger"

"swingers"

"swinges"

"swingier"

"swingiest"

"swinging"

"swingometer"

"swings"

"swingy"

"swinish"

"swinks"

"swipe"

"swiped"

"swipes"

"swiping"

"swirl"

"swirled"

"swirlier"

"swirliest"

"swirling"

"swirls"

"swirly"

"swish"

"swished"

"swisher"

"swishers"

"swishes"

"swishier"

"swishiest"

"swishing"

"swishy"

"swiss"

"swisses"

"switch"

"switchable"

"switchback"

"switchbacks"

"switchblade"

"switchblades"

"switchboard"

"switchboards"

"switched"

"switcher"

"switchers"

"switches"

"switching"

"switchman"

"switchmen"

"switchyard"

"swithers"

"switzerland"

"swivel"

"swiveled"

"swiveling"

"swivelled"

"swivelling"

"swivels"

"swivet"

"swivets"

"swizzle"

"swizzled"

"swizzler"

"swizzlers"

"swizzles"

"swizzling"

"swob"

"swobbed"

"swobber"

"swollen"

"swoon"

"swooned"

"swooner"

"swooners"

"swooning"

"swooningly"

"swoons"

"swoop"

"swooped"

"swooper"

"swoopers"

"swooping"

"swoops"

"swoosh"

"swooshed"

"swooshes"

"swooshing"

"swop"

"swopped"

"swops"

"sword"

"swordfish"

"swordfishes"

"swordman"

"swordmen"

"swordplay"

"swords"

"swordsman"

"swordsmanship"

"swordsmen"

"swore"

"sworn"

"swounds"

"swum"

"swung"

"sybarite"

"sybarites"

"sybaritic"

"sycamore"

"sycamores"

"sycophancy"

"sycophant"

"sycophantic"

"sycophants"

"sydney"

"syllabatim"

"syllabi"

"syllabic"

"syllabicate"

"syllabics"

"syllabification"

"syllabifications"

"syllabified"

"syllabifies"

"syllabify"

"syllabifying"

"syllable"

"syllabled"

"syllables"

"syllabub"

"syllabubs"

"syllabus"

"syllabuses"

"syllogism"

"syllogisms"

"syllogistic"

"syllogistically"

"sylph"

"sylphic"

"sylphid"

"sylphids"

"sylphish"

"sylphs"

"sylphy"

"sylvan"

"sylvans"

"sylvas"

"sylvia"

"sylvian"

"sylvius"

"symbion"

"symbiont"

"symbionts"

"symbioses"

"symbiosis"

"symbiot"

"symbiote"

"symbiotes"

"symbiotic"

"symbiotical"

"symbiotically"

"symblepharon"

"symbol"

"symboled"

"symbolic"

"symbolical"

"symbolically"

"symboling"

"symbolism"

"symbolisms"

"symbolization"

"symbolizations"

"symbolize"

"symbolized"

"symbolizes"

"symbolizing"

"symbology"

"symbols"

"symmetric"

"symmetrical"

"symmetrically"

"symmetries"

"symmetrophobia"

"symmetry"

"sympathetic"

"sympathetically"

"sympathies"

"sympathize"

"sympathized"

"sympathizer"

"sympathizers"

"sympathizes"

"sympathizing"

"sympathy"

"symphonic"

"symphonies"

"symphony"

"sympiesometer"

"symposia"

"symposium"

"symposiums"

"sympto-thermal"

"symptom"

"symptomatic"

"symptomatically"

"symptomatological"

"symptomatologically"

"symptomatologies"

"symptomatology"

"symptomless"

"symptoms"

"symptothermal method"

"synaesthesia"

"synaesthetic"

"synagog"

"synagogal"

"synagogs"

"synagogue"

"synagogues"

"synapse"

"synapse growth"

"synapsed"

"synapses"

"synapsing"

"synapsis"

"synaptic"

"synaptically"

"sync"

"synced"

"synch"

"synched"

"synching"

"synchro"

"synchronies"

"synchronism"

"synchronization"

"synchronize"

"synchronized"

"synchronizer"

"synchronizers"

"synchronizes"

"synchronizing"

"synchronous"

"synchronously"

"synchrony"

"synchros"

"synchroscope"

"synchrotron"

"synchs"

"syncing"

"syncline"

"synclines"

"syncom"

"syncoms"

"syncopal"

"syncopate"

"syncopated"

"syncopates"

"syncopating"

"syncopation"

"syncopations"

"syncope"

"syncopes"

"syncopic"

"syncs"

"syndic"

"syndical"

"syndicate"

"syndicated"

"syndicates"

"syndicating"

"syndication"

"syndications"

"syndicator"

"syndics"

"syndrome"

"syndromes"

"syne"

"synecology"

"synectics"

"synergetic"

"synergically"

"synergies"

"synergism"

"synergist"

"synergistic"

"synergistical"

"synergistically"

"synergists"

"synergy"

"synesthesia"

"synesthetic"

"synfuel"

"synfuels"

"synod"

"synodal"

"synodic"

"synodical"

"synods"

"synonym"

"synonymicon"

"synonymous"

"synonyms"

"synonymy"

"synopses"

"synopsis"

"synoptic"

"synoptical"

"synovial"

"synovias"

"syntactic"

"syntactical"

"syntactically"

"syntalities"

"syntax"

"syntaxes"

"syntheses"

"synthesis"

"synthesize"

"synthesized"

"synthesizer"

"synthesizers"

"synthesizes"

"synthesizing"

"synthetic"

"synthetical"

"synthetically"

"synthetics"

"sypher"

"syphilis"

"syphilises"

"syphilitic"

"syphilitics"

"syphilized"

"syphilizing"

"syphiloid"

"syphilology"

"syphilomania"

"syphilophobia"

"syphon"

"syphoned"

"syphoning"

"syphons"

"syracuse"

"syren"

"syrens"

"syria"

"syrian"

"syrians"

"syringe"

"syringed"

"syringes"

"syringing"

"syrinx"

"syrinxes"

"syrup"

"syrups"

"syrupy"

"system"

"systematic"

"systematical"

"systematically"

"systematization"

"systematize"

"systematized"

"systematizes"

"systematizing"

"systematology"

"systemic"

"systemically"

"systemics"

"systemize"

"systemized"

"systemizes"

"systemizing"

"systemless"

"systems"

"systole"

"systoles"

"systolic"

"syzygal"

"syzygial"

"syzygies"

"syzygy"

"tab"

"tabard"

"tabarded"

"tabards"

"tabaret"

"tabasco"

"tabbed"

"tabbies"

"tabbing"

"tabby"

"tabernacle"

"tabernacles"

"tabers"

"tabla"

"tablas"

"table"

"tableau"

"tableaus"

"tableaux"

"tablecloth"

"tablecloths"

"tabled"

"tableful"

"tablefuls"

"tableland"

"tablelands"

"tables"

"tablesful"

"tablespoon"

"tablespoonful"

"tablespoonfuls"

"tablespoons"

"tablespoonsful"

"tablet"

"tabletop"

"tabletops"

"tablets"

"tabletted"

"tabletting"

"tableware"

"tabling"

"tabloid"

"tabloids"

"taboo"

"tabooed"

"tabooing"

"taboos"

"tabor"

"taboret"

"taborets"

"tabors"

"tabour"

"tabourers"

"tabouret"

"tabourets"

"tabs"

"tabstop"

"tabstops"

"tabu"

"tabued"

"tabuing"

"tabula"

"tabulable"

"tabular"

"tabularly"

"tabulate"

"tabulated"

"tabulates"

"tabulating"

"tabulation"

"tabulations"

"tabulator"

"tabulators"

"tacet"

"tach"

"tacheometer"

"tachistoscope"

"tachograph"

"tachometer"

"tachometers"

"tachs"

"tachycardia"

"tachycardiac"

"tacit"

"tacitly"

"tacitness"

"taciturn"

"taciturnities"

"taciturnity"

"taciturnly"

"tack"

"tacked"

"tacker"

"tackers"

"tackets"

"tackey"

"tackier"

"tackiest"

"tackified"

"tackifies"

"tackify"

"tackifying"

"tackily"

"tackiness"

"tacking"

"tackle"

"tackled"

"tackler"

"tacklers"

"tackles"

"tackless"

"tackling"

"tacklings"

"tacks"

"tacksman"

"tacksmen"

"tacky"

"taco"

"tacoma"

"taconite"

"tacos"

"tact"

"tactful"

"tactfully"

"tactfulness"

"tactic"

"tactical"

"tactically"

"tactician"

"tacticians"

"tactics"

"tactile"

"tactility"

"taction"

"tactless"

"tactlessly"

"tactoid"

"tacts"

"tactual"

"tactually"

"tad"

"tadpole"

"tadpoles"

"tads"

"taels"

"taffeta"

"taffetas"

"taffies"

"taffrail"

"taffrails"

"taffy"

"tag"

"tagalog"

"tagalogs"

"tagalong"

"tagalongs"

"tagboard"

"tagboards"

"tagged"

"tagger"

"taggers"

"tagging"

"tags"

"tahiti"

"tahitian"

"tahitians"

"tai"

"taiga"

"tail"

"tailbacks"

"tailbone"

"tailbones"

"tailcoat"

"tailcoats"

"tailed"

"tailer"

"tailers"

"tailgate"

"tailgated"

"tailgates"

"tailgating"

"tailing"

"tailings"

"tailless"

"taillight"

"taillights"

"tailor"

"tailored"

"tailoress"

"tailoring"

"tailors"

"tailpiece"

"tailpipe"

"tailpipes"

"tails"

"tailskids"

"tailspin"

"tailspins"

"tailwind"

"tailwinds"

"taint"

"tainted"

"tainting"

"taints"

"taipei"

"taiwan"

"taiwanese"

"taj"

"takable"

"take"

"takeable"

"takedown"

"takedowns"

"takeing"

"taken"

"takeoff"

"takeoffs"

"takeout"

"takeouts"

"takeover"

"takeovers"

"taker"

"takers"

"takes"

"taketh"

"taking"

"takingly"

"takings"

"talc"

"talced"

"talcky"

"talcs"

"talcum"

"talcums"

"tale"

"talebearer"

"talebearers"

"talebearing"

"talent"

"talented"

"talented artists"

"talents"

"taler"

"talers"

"tales"

"talesman"

"talesmen"

"talian"

"talisman"

"talismans"

"talk"

"talkable"

"talkative"

"talkatively"

"talkativeness"

"talked"

"talker"

"talkers"

"talkie"

"talkier"

"talkies"

"talkiest"

"talking"

"talkings"

"talks"

"talky"

"tall"

"tallahassee"

"tallboys"

"taller"

"tallest"

"tallied"

"tallier"

"talliers"

"tallies"

"tallish"

"tallness"

"tallow"

"tallowed"

"tallowing"

"tallows"

"tallowy"

"tally"

"tallyho"

"tallyhoed"

"tallyhoing"

"tallyhos"

"tallying"

"tallyman"

"tallymen"

"talmud"

"talmudic"

"talmudist"

"talmudists"

"talon"

"taloned"

"talons"

"talus"

"taluses"

"tam"

"tamable"

"tamale"

"tamales"

"tamals"

"tamarack"

"tamaracks"

"tamarind"

"tamarinds"

"tamarisk"

"tamarisks"

"tambour"

"tamboura"

"tambourine"

"tambourines"

"tambouring"

"tambours"

"tambur"

"tambura"

"tamburas"

"tamburs"

"tame"

"tameable"

"tamed"

"tamely"

"tameness"

"tamer"

"tamers"

"tames"

"tamest"

"taming"

"tammie"

"tammies"

"tammy"

"tamp"

"tampa"

"tamped"

"tamper"

"tampered"

"tamperer"

"tamperers"

"tampering"

"tampers"

"tamping"

"tampon"

"tamponed"

"tampons"

"tamps"

"tams"

"tan"

"tanager"

"tanagers"

"tanbark"

"tanbarks"

"tandem"

"tandems"

"tang"

"tanged"

"tangelo"

"tangelos"

"tangence"

"tangences"

"tangencies"

"tangency"

"tangent"

"tangential"

"tangentiality"

"tangentially"

"tangents"

"tangerine"

"tangerines"

"tangibility"

"tangible"

"tangibleness"

"tangibles"

"tangibly"

"tangier"

"tangiest"

"tangle"

"tangled"

"tangler"

"tanglers"

"tangles"

"tanglier"

"tangliest"

"tangling"

"tangly"

"tango"

"tangoed"

"tangoing"

"tangos"

"tangram"

"tangrams"

"tangs"

"tangy"

"tank"

"tanka"

"tankage"

"tankages"

"tankard"

"tankards"

"tankas"

"tanked"

"tanker"

"tankers"

"tankful"

"tankfuls"

"tanking"

"tanks"

"tankship"

"tankships"

"tannable"

"tannages"

"tannates"

"tanned"

"tanner"

"tanneries"

"tanners"

"tannery"

"tannest"

"tannic"

"tannin"

"tanning"

"tannings"

"tannins"

"tannish"

"tans"

"tansies"

"tansy"

"tantalic"

"tantalization"

"tantalize"

"tantalized"

"tantalizer"

"tantalizers"

"tantalizes"

"tantalizing"

"tantalizingly"

"tantalum"

"tantalums"

"tantalus"

"tantaluses"

"tantamount"

"tantara"

"tantaras"

"tanti"

"tanto"

"tantra"

"tantras"

"tantric"

"tantrum"

"tantrums"

"tanyard"

"tanyards"

"tanzania"

"tanzanian"

"tanzanians"

"tao"

"taoism"

"taoist"

"taoists"

"taos"

"tap"

"tape"

"taped"

"tapeline"

"tapelines"

"taper"

"tapered"

"taperer"

"taperers"

"tapering"

"taperingly"

"tapers"

"tapes"

"tapestried"

"tapestries"

"tapestry"

"tapeworm"

"tapeworms"

"taphephobia"

"taphole"

"tapholes"

"taphouse"

"taphouses"

"taping"

"tapioca"

"tapiocas"

"tapir"

"tapirs"

"tapped"

"tapper"

"tappers"

"tappet"

"tappets"

"tapping"

"tappings"

"taproom"

"taprooms"

"taproot"

"taproots"

"taps"

"Taps per minute"

"tapster"

"tapsters"

"tar"

"tarantula"

"tarantulae"

"tarantulas"

"tarboosh"

"tarbush"

"tarbushes"

"tarde"

"tardier"

"tardies"

"tardiest"

"tardily"

"tardiness"

"tardo"

"tardy"

"tare"

"tared"

"tares"

"target"

"targeted"

"targeting"

"targets"

"tariff"

"tariffed"

"tariffing"

"tariffless"

"tariffs"

"taring"

"tarmac"

"tarmacs"

"tarn"

"tarnal"

"tarnally"

"tarnish"

"tarnishable"

"tarnished"

"tarnishes"

"tarnishing"

"tarns"

"taro"

"taros"

"tarot"

"tarots"

"tarp"

"tarpaper"

"tarpapered"

"tarpapers"

"tarpaulin"

"tarpaulins"

"tarpon"

"tarpons"

"tarps"

"tarragon"

"tarragons"

"tarred"

"tarried"

"tarrier"

"tarriers"

"tarries"

"tarriest"

"tarriness"

"tarring"

"tarry"

"tarrying"

"tars"

"tarsal"

"tarsals"

"tarsi"

"tarsier"

"tarsiers"

"tarsus"

"tart"

"tartan"

"tartans"

"tartar"

"tartare"

"tartaric"

"tartars"

"tarted"

"tarter"

"tartest"

"tarting"

"tartish"

"tartlet"

"tartlets"

"tartly"

"tartness"

"tartrate"

"tartrated"

"tartrates"

"tarts"

"tartufe"

"tartuffe"

"tartuffes"

"tarweed"

"tarweeds"

"tarzan"

"tarzans"

"taseometer"

"tasimeter"

"task"

"tasked"

"tasking"

"taskmaster"

"taskmasters"

"tasks"

"tasksetter"

"taskwork"

"taskworks"

"tass"

"tassel"

"tasseled"

"tasseling"

"tasselled"

"tasselling"

"tassels"

"tasses"

"tastable"

"taste"

"tasted"

"tasteful"

"tastefully"

"tastefulness"

"tasteless"

"tastelessly"

"tastelessness"

"taster"

"tasters"

"tastes"

"tastier"

"tastiest"

"tastily"

"tastiness"

"tasting"

"tasty"

"tat"

"tatami"

"tatamis"

"tatar"

"tate"

"tater"

"taters"

"tatoo"

"tatoos"

"tats"

"tatted"

"tatter"

"tatterdemalion"

"tatterdemalions"

"tattered"

"tattering"

"tatters"

"tattersall"

"tattersalls"

"tattier"

"tattiest"

"tatting"

"tattings"

"tattle"

"tattled"

"tattler"

"tattlers"

"tattles"

"tattletale"

"tattletales"

"tattling"

"tattoo"

"tattooed"

"tattooer"

"tattooers"

"tattooing"

"tattooist"

"tattooists"

"tattoos"

"tatty"

"tau"

"taught"

"taunt"

"taunted"

"taunter"

"taunters"

"taunting"

"tauntingly"

"taunts"

"taupe"

"taupes"

"taurine"

"taurines"

"taurus"

"tauruses"

"taut"

"tauten"

"tautened"

"tautening"

"tautens"

"tauter"

"tautest"

"tauting"

"tautly"

"tautness"

"tautogs"

"tautological"

"tautologically"

"tautologies"

"tautologous"

"tautology"

"tautonym"

"tautonyms"

"tauts"

"tavern"

"taverner"

"taverners"

"taverns"

"taw"

"tawdrier"

"tawdries"

"tawdriest"

"tawdrily"

"tawdriness"

"tawdry"

"tawers"

"tawing"

"tawney"

"tawnier"

"tawnies"

"tawniest"

"tawnily"

"tawniness"

"tawny"

"taws"

"tax"

"taxability"

"taxable"

"taxables"

"taxably"

"taxation"

"taxational"

"taxations"

"taxed"

"taxer"

"taxers"

"taxes"

"taxi"

"taxicab"

"taxicabs"

"taxidermist"

"taxidermists"

"taxidermy"

"taxied"

"taxies"

"taxiing"

"taximan"

"taximen"

"taximeter"

"taxing"

"taxingly"

"taxiplane"

"taxis"

"taxistand"

"taxiway"

"taxiways"

"taxless"

"taxman"

"taxmen"

"taxonomic"

"taxonomical"

"taxonomically"

"taxonomies"

"taxonomist"

"taxonomists"

"taxonomy"

"taxpayer"

"taxpayers"

"taxpaying"

"taxying"

"tazza"

"tazzas"

"tazze"

"tbs"

"tbsp"

"tchaikovsky"

"tea"

"teaberries"

"teaberry"

"teaboard"

"teaboards"

"teabowl"

"teabowls"

"teabox"

"teaboxes"

"teacake"

"teacakes"

"teacart"

"teacarts"

"teach"

"teachability"

"teachable"

"teacher"

"teacherage"

"teachers"

"teaches"

"teaching"

"teachings"

"teacup"

"teacupful"

"teacupfuls"

"teacups"

"teahouse"

"teahouses"

"teak"

"teakettle"

"teakettles"

"teaks"

"teakwood"

"teakwoods"

"teal"

"teals"

"team"

"teamaker"

"teamakers"

"teamed"

"teamer"

"teaming"

"teammate"

"teammates"

"teams"

"teamster"

"teamsters"

"teamwork"

"teamworks"

"teapot"

"teapots"

"tear"

"tearable"

"teardown"

"teardrop"

"teardrops"

"teared"

"tearer"

"tearers"

"tearful"

"tearfully"

"teargas"

"teargases"

"teargassed"

"teargasses"

"teargassing"

"tearier"

"teariest"

"tearing"

"tearjerker"

"tearjerkers"

"tearoom"

"tears"

"tearstain"

"tearstained"

"teary"

"teas"

"tease"

"teased"

"teasel"

"teasels"

"teaser"

"teasers"

"teases"

"teashop"

"teashops"

"teasing"

"teasingly"

"teaspoon"

"teaspoonful"

"teaspoonfuls"

"teaspoons"

"teaspoonsful"

"teat"

"teated"

"teatime"

"teatimes"

"teats"

"teaware"

"teawares"

"teazel"

"teazeled"

"teazelling"

"teazels"

"teazle"

"teazled"

"teazles"

"teazling"

"tech"

"techie"

"techies"

"technetium"

"technic"

"technical"

"technicalities"

"technicality"

"technically"

"technicalness"

"technician"

"technicians"

"technicolor"

"technics"

"technique"

"techniques"

"technocracy"

"technocrat"

"technocratic"

"technocrats"

"technological"

"technologically"

"technologies"

"technologist"

"technologists"

"technology"

"technomania"

"technophobia"

"techy"

"tectonic"

"tectonics"

"tecum"

"ted"

"teddies"

"teddy"

"tedious"

"tediously"

"tediousness"

"tedium"

"tediums"

"teds"

"tee"

"teed"

"teeing"

"teem"

"teemed"

"teemer"

"teemers"

"teeming"

"teems"

"teen"

"teenage"

"teenaged"

"teenager"

"teenagers"

"teener"

"teeners"

"teenful"

"teenier"

"teeniest"

"teens"

"teensier"

"teensiest"

"teensy"

"teentsier"

"teentsiest"

"teentsy"

"teeny"

"teenybopper"

"teenyboppers"

"teepee"

"teepees"

"tees"

"teeter"

"teetered"

"teetering"

"teeters"

"teeth"

"teethe"

"teethed"

"teether"

"teethers"

"teethes"

"teething"

"teethings"

"teetotal"

"teetotaled"

"teetotaler"

"teetotalers"

"teetotalism"

"teetotals"

"teetotum"

"teetotums"

"teflon"

"teg"

"tegestology"

"tegument"

"teheran"

"tej"

"tektite"

"tektites"

"tektitic"

"telecast"

"telecasted"

"telecaster"

"telecasters"

"telecasting"

"telecasts"

"telecommunication"

"telecommunications"

"telefilms"

"telegenic"

"telegram"

"telegrams"

"telegraph"

"telegraphed"

"telegrapher"

"telegraphers"

"telegraphic"

"telegraphically"

"telegraphing"

"telegraphist"

"telegraphists"

"telegraphs"

"telegraphy"

"telekineses"

"telekinesis"

"telemeter"

"telemeters"

"telemetric"

"telemetrically"

"telemetries"

"telemetry"

"teleological"

"teleologies"

"teleology"

"telepathic"

"telepathically"

"telepathies"

"telepathist"

"telepathy"

"telephone"

"telephoned"

"telephoner"

"telephoners"

"telephones"

"telephonic"

"telephonically"

"telephoning"

"telephonist"

"telephonists"

"telephony"

"telephoto"

"telephotograph"

"telephotographed"

"telephotographic"

"telephotographing"

"telephotographs"

"telephotography"

"teleplay"

"teleplays"

"teleport"

"teleported"

"teleports"

"teleprinter"

"teleprinters"

"teleradiography"

"telescope"

"telescoped"

"telescopes"

"telescopic"

"telescopically"

"telescoping"

"telesis"

"telespectroscope"

"telestereoscope"

"telethon"

"telethons"

"teletype"

"teletypes"

"teletypewriter"

"teletypewriters"

"teletypist"

"teletypists"

"teleview"

"televiewed"

"televiewer"

"televiews"

"televise"

"televised"

"televises"

"televising"

"television"

"televisional"

"televisionally"

"televisionary"

"televisions"

"telex"

"telexed"

"telexes"

"telexing"

"tell"

"tellable"

"teller"

"tellers"

"tellership"

"tellies"

"telling"

"tellingly"

"tells"

"telltale"

"telltales"

"telluric"

"tellurium"

"tellurometer"

"telly"

"telmatology"

"tem"

"temblor"

"temblors"

"temerities"

"temerity"

"temp"

"tempeh"

"tempehs"

"temper"

"tempera"

"temperament"

"temperamental"

"temperamentally"

"temperaments"

"temperance"

"temperas"

"temperate"

"temperately"

"temperateness"

"temperature"

"Temperature method"

"Temperature Reminder"

"temperatures"

"tempered"

"temperer"

"temperers"

"tempering"

"tempers"

"tempest"

"tempested"

"tempesting"

"tempests"

"tempestuous"

"tempestuously"

"tempestuousness"

"tempi"

"templar"

"templars"

"template"

"templates"

"temple"

"templed"

"temples"

"tempo"

"temporal"

"temporalities"

"temporality"

"temporally"

"temporals"

"temporalties"

"temporalty"

"temporaries"

"temporarily"

"temporariness"

"temporary"

"tempore"

"temporization"

"temporize"

"temporized"

"temporizer"

"temporizers"

"temporizes"

"temporizing"

"tempos"

"temps"

"tempt"

"temptable"

"temptation"

"temptations"

"tempted"

"tempter"

"tempters"

"tempting"

"temptingly"

"temptress"

"temptresses"

"tempts"

"tempura"

"tempuras"

"tempus"

"ten"

"tenability"

"tenable"

"tenableness"

"tenably"

"tenacious"

"tenaciously"

"tenacities"

"tenacity"

"tenancies"

"tenancy"

"tenant"

"tenantable"

"tenanted"

"tenanting"

"tenantless"

"tenantry"

"tenants"

"tenantship"

"tench"

"tenches"

"tend"

"tended"

"tendencies"

"tendency"

"tendentious"

"tendentiously"

"tendentiousness"

"tender"

"tenderability"

"tenderable"

"tendered"

"tenderer"

"tenderers"

"tenderest"

"tenderfeet"

"tenderfoot"

"tenderfoots"

"tenderhearted"

"tenderheartedly"

"tenderheartedness"

"tendering"

"tenderize"

"tenderized"

"tenderizer"

"tenderizers"

"tenderizes"

"tenderizing"

"tenderloin"

"tenderloins"

"tenderly"

"tenderness"

"tenderometer"

"tenders"

"tending"

"tendon"

"tendonitis"

"tendons"

"tendril"

"tendrils"

"tends"

"tenebrous"

"tenement"

"tenemental"

"tenemented"

"tenements"

"tenet"

"tenets"

"tenfold"

"tenfolds"

"tenner"

"tenners"

"tennessean"

"tennesseans"

"tennessee"

"tennesseeans"

"tennis"

"tennises"

"tennists"

"tennyson"

"tenon"

"tenoned"

"tenoner"

"tenoners"

"tenoning"

"tenons"

"tenor"

"tenors"

"tenours"

"tenpence"

"tenpences"

"tenpenny"

"tenpin"

"tenpins"

"tens"

"tense"

"tensed"

"tensely"

"tenseness"

"tenser"

"tenses"

"tensest"

"tensible"

"tensibly"

"tensile"

"tensimeter"

"tensing"

"tensiometer"

"tension"

"tensional"

"tensioned"

"tensioning"

"tensionless"

"tensions"

"tensities"

"tensity"

"tensive"

"tensor"

"tensors"

"tent"

"tentacle"

"tentacled"

"tentacles"

"tentacular"

"tentage"

"tentages"

"tentative"

"tentatively"

"tentativeness"

"tented"

"tenter"

"tentered"

"tenterhook"

"tenterhooks"

"tentering"

"tenters"

"tenth"

"tenthly"

"tenths"

"tentier"

"tenting"

"tentless"

"tentmaker"

"tents"

"tenty"

"tenuity"

"tenuous"

"tenuously"

"tenuousness"

"tenure"

"tenured"

"tenures"

"tenuto"

"tenutos"

"tepee"

"tepees"

"tepid"

"tepidities"

"tepidity"

"tepidly"

"tepidness"

"tequila"

"tequilas"

"teraphim"

"teratism"

"teratisms"

"teratogen"

"teratogenetic"

"teratogenic"

"teratoid"

"teratologic"

"teratological"

"teratologies"

"teratologist"

"teratology"

"teratoma"

"teratomas"

"teratophobia"

"teratosis"

"terbium"

"terbiums"

"terce"

"tercel"

"tercels"

"tercentenaries"

"tercentenary"

"tercentennial"

"tercentennials"

"teriyaki"

"teriyakis"

"term"

"termagant"

"termagants"

"termed"

"termer"

"termers"

"terminability"

"terminable"

"terminal"

"terminally"

"terminals"

"terminate"

"terminated"

"terminates"

"terminating"

"termination"

"terminations"

"terminative"

"terminator"

"terminators"

"terminatory"

"terming"

"termini"

"terminological"

"terminologically"

"terminologies"

"terminologist"

"terminologists"

"terminology"

"terminus"

"terminuses"

"termite"

"termites"

"termitic"

"termly"

"terms"

"tern"

"ternaries"

"ternary"

"ternate"

"terne"

"terns"

"terpsichorean"

"terr"

"terra"

"terrace"

"terraced"

"terraces"

"terracing"

"terrain"

"terrains"

"terrane"

"terrapin"

"terrapins"

"terraqueous"

"terraria"

"terrarium"

"terrariums"

"terras"

"terrazzo"

"terrazzos"

"terre"

"terrene"

"terrenes"

"terrestrial"

"terrestrially"

"terrible"

"terribleness"

"terribles"

"terribly"

"terrier"

"terriers"

"terrific"

"terrifically"

"terrified"

"terrifier"

"terrifiers"

"terrifies"

"terrify"

"terrifying"

"terrifyingly"

"terrines"

"territorial"

"territorialize"

"territorialized"

"territorializing"

"territories"

"territory"

"terror"

"terrorism"

"terrorist"

"terrorists"

"terrorization"

"terrorize"

"terrorized"

"terrorizes"

"terrorizing"

"terrors"

"terry"

"terse"

"tersely"

"terseness"

"terser"

"tersest"

"tertial"

"tertials"

"tertian"

"tertians"

"tertiaries"

"tertiary"

"tesla"

"teslas"

"tessellate"

"tessellated"

"tessellates"

"tessellating"

"tessellation"

"tessellations"

"test"

"testability"

"testable"

"testacies"

"testacy"

"testament"

"testamental"

"testamentary"

"testaments"

"testate"

"testation"

"testator"

"testators"

"testatrix"

"testatrixes"

"testatum"

"tested"

"testee"

"testees"

"tester"

"testers"

"testes"

"testicle"

"testicles"

"testicular"

"testier"

"testiest"

"testified"

"testifier"

"testifiers"

"testifies"

"testify"

"testifying"

"testily"

"testimonial"

"testimonials"

"testimonies"

"testimony"

"testiness"

"testing"

"testings"

"testis"

"testosterone"

"tests"

"testy"

"tetanal"

"tetanic"

"tetanization"

"tetanized"

"tetanizes"

"tetanus"

"tetanuses"

"tetany"

"tetched"

"tetchier"

"tetchiest"

"tetchily"

"tetchy"

"tether"

"tetherball"

"tethered"

"tethering"

"tethers"

"tetotum"

"tetra"

"tetrachloride"

"tetrachlorides"

"tetracycline"

"tetrad"

"tetradic"

"tetrads"

"tetraethyl"

"tetragon"

"tetragons"

"tetrahedra"

"tetrahedral"

"tetrahedron"

"tetrahedrons"

"tetralogies"

"tetralogy"

"tetrameter"

"tetrameters"

"tetrapod"

"tetrapods"

"tetrarch"

"tetrarchs"

"tetras"

"tetrasaccharide"

"tetravalent"

"tetryl"

"teuthology"

"Teutomania"

"teuton"

"teutonic"

"teutons"

"tex"

"texaco"

"texan"

"texans"

"texas"

"texases"

"text"

"textbook"

"textbooks"

"textile"

"textiles"

"textology"

"texts"

"textual"

"textually"

"textuaries"

"textural"

"texture"

"textured"

"textures"

"texturing"

"th"

"thaddeus"

"thai"

"thailand"

"thalami"

"thalamic"

"thalamically"

"thalamocortical"

"thalamus"

"thalassography"

"thalassometer"

"thalassophobia"

"thalers"

"thalidomide"

"thallium"

"thalliums"

"thallophyte"

"thallophytic"

"thames"

"than"

"thanatoid"

"thanatologies"

"thanatology"

"thanatomania"

"thanatophobia"

"thanatos"

"thanatoses"

"thane"

"thanes"

"thank"

"thanked"

"thanker"

"thankers"

"thankful"

"thankfully"

"thankfulness"

"thanking"

"thankless"

"thanklessly"

"thanks"

"thanksgiving"

"thanksgivings"

"thankyou"

"that"

"thataway"

"thatch"

"thatched"

"thatcher"

"thatchers"

"thatches"

"thatching"

"thats"

"thaumatology"

"thaw"

"thawed"

"thawing"

"thawless"

"thaws"

"the"

"The Orange Book"

"thearchies"

"thearchy"

"theater"

"theatergoer"

"theatergoers"

"theaters"

"theatre"

"theatres"

"theatric"

"theatrical"

"theatricality"

"theatrically"

"theatricals"

"theatrics"

"theatromania"

"thee"

"theft"

"theftproof"

"thefts"

"their"

"theirs"

"theism"

"theisms"

"theist"

"theistic"

"theistically"

"theists"

"them"

"thematic"

"thematically"

"theme"

"themes"

"themselves"

"then"

"thence"

"thenceforth"

"thens"

"theobromine"

"theocracies"

"theocracy"

"theocrat"

"theocratic"

"theocratically"

"theocrats"

"theodicy"

"theodore"

"theologian"

"theologians"

"theological"

"theologically"

"theologies"

"theologs"

"theology"

"theomania"

"theophobia"

"theorem"

"theorems"

"theoretic"

"theoretical"

"theoretically"

"theoretician"

"theoreticians"

"theories"

"theorising"

"theorist"

"theorists"

"theorization"

"theorize"

"theorized"

"theorizer"

"theorizers"

"theorizes"

"theorizing"

"theory"

"theosophic"

"theosophical"

"theosophically"

"theosophist"

"theosophists"

"theosophy"

"therapeutic"

"therapeutic biologicals"

"therapeutical"

"therapeutically"

"therapeutically equivalent"

"therapeutics"

"therapeutist"

"therapies"

"therapist"

"therapists"

"therapy"

"therblig"

"there"

"thereabout"

"thereabouts"

"thereafter"

"thereamong"

"thereat"

"thereby"

"therefor"

"therefore"

"therefrom"

"therein"

"thereinafter"

"theremin"

"theremins"

"thereof"

"thereon"

"thereout"

"theres"

"thereto"

"theretofore"

"thereunder"

"thereuntil"

"thereunto"

"thereupon"

"therewith"

"therewithal"

"theriatrics"

"theriogenology"

"therm"

"thermal"

"thermally"

"thermistor"

"thermistors"

"thermite"

"thermites"

"thermocauteries"

"thermochemistry"

"thermocouple"

"thermocurrent"

"thermodynamic"

"thermodynamically"

"thermodynamics"

"thermoelectric"

"thermoelectron"

"thermograph"

"thermography"

"thermokinematics"

"thermology"

"thermometer"

"thermometers"

"thermometric"

"thermometrical"

"thermometrically"

"thermometrograph"

"thermometry"

"thermonuclear"

"thermoplastic"

"thermoplasticity"

"thermoplastics"

"thermoreceptor"

"thermoregulation"

"thermoregulatory"

"thermos"

"thermoscope"

"thermoses"

"thermosetting"

"thermosphere"

"thermospheres"

"thermostable"

"thermostat"

"thermostatic"

"thermostatically"

"thermostats"

"thermotropic"

"therms"

"therology"

"thersitical"

"thesauri"

"thesaurus"

"thesauruses"

"these"

"theses"

"thesis"

"thespian"

"thespians"

"thessalonians"

"theta"

"thetas"

"theurgic"

"theurgies"

"theurgy"

"thew"

"thewless"

"thews"

"thewy"

"they"

"thiabendazole"

"thiamin"

"thiamine"

"thiamines"

"thiamins"

"thick"

"thicken"

"thickened"

"thickener"

"thickeners"

"thickening"

"thickens"

"thicker"

"thickest"

"thicket"

"thickets"

"thickety"

"thickish"

"thickly"

"thickness"

"thicknesses"

"thicks"

"thickset"

"thicksets"

"thief"

"thieftaker"

"thieve"

"thieved"

"thieveries"

"thievery"

"thieves"

"thieving"

"thievish"

"thigh"

"thighbone"

"thighbones"

"thighed"

"thighs"

"thimble"

"thimbleful"

"thimblefuls"

"thimbles"

"thin"

"thinclad"

"thinclads"

"thine"

"thing"

"things"

"think"

"thinkable"

"thinkably"

"thinker"

"thinkers"

"thinking"

"thinkings"

"thinks"

"thinly"

"thinned"

"thinner"

"thinners"

"thinness"

"thinnest"

"thinning"

"thinnish"

"thins"

"thiosulfate"

"thiosulfates"

"third"

"thirdendeal"

"thirdly"

"thirds"

"thirst"

"thirsted"

"thirster"

"thirsters"

"thirstier"

"thirstiest"

"thirstily"

"thirstiness"

"thirsting"

"thirsts"

"thirsty"

"thirteen"

"thirteens"

"thirteenth"

"thirteenths"

"thirties"

"thirtieth"

"thirtieths"

"thirty"

"this"

"thistle"

"thistledown"

"thistles"

"thistly"

"thither"

"thitherward"

"tho"

"thole"

"tholes"

"thomas"

"thompson"

"thong"

"thonged"

"thongs"

"thor"

"thoraces"

"thoracic"

"thoracoscope"

"thorax"

"thoraxes"

"thorium"

"thoriums"

"thorn"

"thornbush"

"thorned"

"thornier"

"thorniest"

"thornily"

"thorning"

"thorns"

"thorny"

"thoro"

"thorough"

"thoroughbred"

"thoroughbreds"

"thorougher"

"thoroughfare"

"thoroughfares"

"thoroughgoing"

"thoroughly"

"thoroughness"

"thorp"

"thorpe"

"thorpes"

"thorps"

"those"

"thou"

"thoued"

"though"

"thought"

"thoughtful"

"thoughtfully"

"thoughtfulness"

"thoughtless"

"thoughtlessly"

"thoughtlessness"

"thoughts"

"thougth"

"thouing"

"thous"

"thousand"

"thousands"

"thousandth"

"thousandths"

"thraldom"

"thrall"

"thralldom"

"thralled"

"thralling"

"thralls"

"thrash"

"thrashed"

"thrasher"

"thrashers"

"thrashes"

"thrashing"

"thraves"

"thrawed"

"thread"

"threadbare"

"threaded"

"threader"

"threaders"

"threadier"

"threadiest"

"threading"

"threads"

"threadworm"

"thready"

"threaped"

"threaper"

"threapers"

"threat"

"threated"

"threaten"

"threatened"

"threatener"

"threateners"

"threatening"

"threateningly"

"threatens"

"threatful"

"threating"

"threats"

"three"

"threefold"

"threeping"

"threes"

"threescore"

"threesome"

"threesomes"

"thremmatology"

"threnodes"

"threnodies"

"threnody"

"threpsology"

"thresh"

"threshed"

"thresher"

"threshers"

"threshes"

"threshing"

"threshold"

"thresholds"

"threw"

"thrice"

"thrift"

"thriftier"

"thriftiest"

"thriftily"

"thriftiness"

"thriftless"

"thriftlessness"

"thrifts"

"thrifty"

"thrill"

"thrilled"

"thriller"

"thrillers"

"thrilling"

"thrillingly"

"thrills"

"thrip"

"thrips"

"thrive"

"thrived"

"thriven"

"thriver"

"thrivers"

"thrives"

"thriving"

"thro"

"throat"

"throated"

"throatier"

"throatiest"

"throatily"

"throatiness"

"throating"

"throats"

"throaty"

"throb"

"throbbed"

"throbber"

"throbbers"

"throbbing"

"throbs"

"throe"

"throes"

"thrombi"

"thromboses"

"thrombosis"

"thrombotic"

"thrombus"

"throne"

"throned"

"thrones"

"throng"

"thronged"

"thronging"

"throngs"

"throning"

"throstle"

"throstles"

"throttle"

"throttled"

"throttler"

"throttlers"

"throttles"

"throttling"

"through"

"throughout"

"throughput"

"throughway"

"throughways"

"throve"

"throw"

"throwaway"

"throwaways"

"throwback"

"throwbacks"

"thrower"

"throwers"

"throwing"

"thrown"

"throws"

"thru"

"thrum"

"thrummed"

"thrummer"

"thrummers"

"thrummier"

"thrummiest"

"thrumming"

"thrummy"

"thrums"

"thruput"

"thruputs"

"thrush"

"thrushes"

"thrust"

"thrusted"

"thruster"

"thrusters"

"thrusting"

"thrustor"

"thrustors"

"thrustpush"

"thrusts"

"thruway"

"thruways"

"thud"

"thudded"

"thudding"

"thuddingly"

"thuds"

"thug"

"thuggee"

"thuggees"

"thuggeries"

"thuggery"

"thuggish"

"thugs"

"thulium"

"thumb"

"thumbed"

"thumbhole"

"thumbing"

"thumbkins"

"thumbnail"

"thumbnails"

"thumbnuts"

"thumbprint"

"thumbs"

"thumbscrew"

"thumbscrews"

"thumbtack"

"thumbtacked"

"thumbtacking"

"thumbtacks"

"thump"

"thumped"

"thumper"

"thumpers"

"thumping"

"thumps"

"thunder"

"thunderbird"

"thunderbolt"

"thunderbolts"

"thunderclap"

"thunderclaps"

"thundercloud"

"thunderclouds"

"thundered"

"thunderhead"

"thunderheads"

"thundering"

"thunderingly"

"thunderous"

"thunderously"

"thunders"

"thundershower"

"thundershowers"

"thunderstorm"

"thunderstorms"

"thunderstruck"

"thundery"

"thurible"

"thuribles"

"thurifer"

"thurifers"

"thursday"

"thursdays"

"thus"

"thusly"

"thwack"

"thwacked"

"thwacker"

"thwackers"

"thwacking"

"thwacks"

"thwart"

"thwarted"

"thwarter"

"thwarters"

"thwarting"

"thwartly"

"thwarts"

"thy"

"thyme"

"thymes"

"thymey"

"thymi"

"thymier"

"thymine"

"thymines"

"thymol"

"thymus"

"thymuses"

"thymy"

"thyroid"

"thyroidal"

"thyroidectomies"

"thyroidectomize"

"thyroidectomized"

"thyroidectomy"

"thyroids"

"thyrse"

"thyself"

"ti"

"tiara"

"tiaraed"

"tiaras"

"tiber"

"tibet"

"tibetan"

"tibetans"

"tibia"

"tibiae"

"tibial"

"tibias"

"tic"

"tick"

"ticked"

"ticker"

"tickers"

"ticket"

"ticketed"

"ticketing"

"tickets"

"ticking"

"tickings"

"tickle"

"tickled"

"tickler"

"ticklers"

"tickles"

"tickling"

"ticklish"

"ticklishly"

"ticklishness"

"ticks"

"ticktock"

"ticktocked"

"ticktocks"

"tics"

"tictac"

"tictacs"

"tictoc"

"tictocked"

"tictocking"

"tictocs"

"tidal"

"tidally"

"tidbit"

"tidbits"

"tiddly"

"tiddlywinks"

"tide"

"tided"

"tideland"

"tidelands"

"tideless"

"tidemark"

"tidemarks"

"tiderips"

"tides"

"tidewater"

"tidewaters"

"tideways"

"tidied"

"tidier"

"tidies"

"tidiest"

"tidily"

"tidiness"

"tiding"

"tidings"

"tidology"

"tidy"

"tidying"

"tidytips"

"tie"

"tieback"

"tiebacks"

"tieclasp"

"tieclasps"

"tied"

"tieing"

"tiepins"

"tier"

"tiercel"

"tiercels"

"tierces"

"tiered"

"tiering"

"tiers"

"ties"

"tiff"

"tiffanies"

"tiffany"

"tiffed"

"tiffin"

"tiffined"

"tiffing"

"tiffins"

"tiffs"

"tiger"

"tigereye"

"tigereyes"

"tigerish"

"tigers"

"tight"

"tighten"

"tightened"

"tightener"

"tighteners"

"tightening"

"tightens"

"tighter"

"tightest"

"tightfisted"

"tightly"

"tightness"

"tightrope"

"tightropes"

"tights"

"tightwad"

"tightwads"

"tightwire"

"tiglon"

"tiglons"

"tigress"

"tigresses"

"tigris"

"tigrish"

"tigroid"

"tike"

"tikes"

"tikis"

"til"

"tilde"

"tildes"

"tile"

"tiled"

"tiler"

"tilers"

"tiles"

"tiling"

"till"

"tillable"

"tillage"

"tillages"

"tilled"

"tiller"

"tillered"

"tillering"

"tillers"

"tilling"

"tills"

"tilt"

"tiltable"

"tilted"

"tilter"

"tilters"

"tilth"

"tilths"

"tilting"

"tiltmeter"

"tilts"

"tiltyard"

"tiltyards"

"tim"

"timbal"

"timbale"

"timbales"

"timbals"

"timber"

"timbered"

"timberhead"

"timbering"

"timberland"

"timberlands"

"timberline"

"timberlines"

"timbers"

"timbre"

"timbrel"

"timbrels"

"timbres"

"timbrology"

"timbromania"

"time"

"Time-setting control"

"timecard"

"timecards"

"timed"

"timekeeper"

"timekeepers"

"timekeeping"

"timeless"

"timelessly"

"timelessness"

"timelier"

"timeliest"

"timeliness"

"timely"

"timeout"

"timeouts"

"timepiece"

"timepieces"

"timer"

"timers"

"times"

"timesaver"

"timesavers"

"timesaving"

"timeserver"

"timeservers"

"timeserving"

"timesharing"

"timetable"

"timetables"

"timework"

"timeworker"

"timeworks"

"timeworn"

"timid"

"timider"

"timidest"

"timidities"

"timidity"

"timidly"

"timidness"

"timing"

"timings"

"timorous"

"timorously"

"timorousness"

"timothies"

"timothy"

"timpani"

"timpanist"

"timpanists"

"timpanum"

"timpanums"

"tin"

"tinct"

"tincted"

"tincting"

"tincts"

"tincture"

"tinctured"

"tinctures"

"tincturing"

"tinder"

"tinderbox"

"tinderboxes"

"tinders"

"tindery"

"tine"

"tined"

"tines"

"tinfoil"

"tinfoils"

"tinfuls"

"ting"

"tinge"

"tinged"

"tingeing"

"tinges"

"tinging"

"tingle"

"tingled"

"tingler"

"tinglers"

"tingles"

"tinglier"

"tingliest"

"tingling"

"tingly"

"tings"

"tinhorn"

"tinhorns"

"tinier"

"tiniest"

"tinily"

"tininess"

"tining"

"tinker"

"tinkered"

"tinkerer"

"tinkerers"

"tinkering"

"tinkers"

"tinkle"

"tinkled"

"tinkles"

"tinklier"

"tinkliest"

"tinkling"

"tinklings"

"tinkly"

"tinman"

"tinmen"

"tinned"

"tinner"

"tinners"

"tinnier"

"tinniest"

"tinnily"

"tinniness"

"tinning"

"tinny"

"tinplate"

"tinplates"

"tins"

"tinsel"

"tinseled"

"tinseling"

"tinselled"

"tinselly"

"tinsels"

"tinsmith"

"tinsmiths"

"tinstone"

"tinstones"

"tint"

"tinted"

"tinter"

"tinters"

"tinting"

"tintings"

"tintinnabulation"

"tintinnabulations"

"tintless"

"tints"

"tintype"

"tintypes"

"tinware"

"tinwares"

"tinwork"

"tinworks"

"tiny"

"tip"

"tipcart"

"tipcarts"

"tipcat"

"tipcats"

"tipi"

"tipis"

"tipless"

"tipoff"

"tipoffs"

"tippable"

"tipped"

"tipper"

"tippers"

"tippet"

"tippets"

"tippier"

"tippiest"

"tipping"

"tipple"

"tippled"

"tippler"

"tipplers"

"tipples"

"tippling"

"tippy"

"tips"

"tipsier"

"tipsiest"

"tipsily"

"tipsiness"

"tipstaff"

"tipster"

"tipsters"

"tipsy"

"tiptoe"

"tiptoed"

"tiptoeing"

"tiptoes"

"tiptop"

"tiptops"

"tirade"

"tirades"

"tire"

"tired"

"tireder"

"tiredest"

"tiredly"

"tiredness"

"tireless"

"tirelessly"

"tirelessness"

"tires"

"tiresome"

"tiresomely"

"tiresomeness"

"tiring"

"tiro"

"tiros"

"tis"

"tisane"

"tisanes"

"tissue"

"tissued"

"tissues"

"tissuey"

"tissuing"

"tit"

"titan"

"titaness"

"titania"

"titanias"

"titanic"

"titanism"

"titanisms"

"titanium"

"titaniums"

"titans"

"titbit"

"titbits"

"titer"

"titers"

"tithable"

"tithe"

"tithed"

"tither"

"tithers"

"tithes"

"tithing"

"tithings"

"titian"

"titians"

"titillate"

"titillated"

"titillates"

"titillating"

"titillatingly"

"titillation"

"titillations"

"titillative"

"titivate"

"titivated"

"titivates"

"titivating"

"title"

"titled"

"titleholder"

"titles"

"titling"

"titlists"

"titmice"

"titmouse"

"titrant"

"titrate"

"titrated"

"titrates"

"titrating"

"titration"

"titrator"

"titrators"

"titre"

"tits"

"titter"

"tittered"

"titterer"

"titterers"

"tittering"

"titteringly"

"titters"

"tittie"

"titties"

"tittle"

"tittles"

"titty"

"titular"

"titularies"

"titulars"

"titulary"

"tizzies"

"tizzy"

"tm"

"tmh"

"TNM"

"tnpk"

"tnt"

"to"

"toad"

"toadfish"

"toadflax"

"toadflaxes"

"toadied"

"toadies"

"toadish"

"toads"

"toadstool"

"toadstools"

"toady"

"toadying"

"toadyish"

"toadyism"

"toadyisms"

"toast"

"toasted"

"toaster"

"toasters"

"toastier"

"toastiest"

"toasting"

"toastmaster"

"toastmasters"

"toastmistress"

"toastmistresses"

"toasts"

"toasty"

"tobacco"

"tobaccoes"

"tobacconist"

"tobacconists"

"tobaccos"

"toboggan"

"tobogganed"

"tobogganist"

"tobogganists"

"toboggans"

"toccata"

"toccatas"

"tocodynamometer"

"tocology"

"tocophobia"

"tocsin"

"tocsins"

"tod"

"today"

"todays"

"toddies"

"toddle"

"toddled"

"toddler"

"toddlers"

"toddles"

"toddling"

"toddy"

"toe"

"toecap"

"toecaps"

"toed"

"toehold"

"toeholds"

"toeing"

"toeless"

"toenail"

"toenailed"

"toenailing"

"toenails"

"toepiece"

"toepieces"

"toeplate"

"toeplates"

"toes"

"toeshoe"

"toeshoes"

"toff"

"toffee"

"toffees"

"toffies"

"toffs"

"toffy"

"tofts"

"tofu"

"tofus"

"tog"

"toga"

"togae"

"togaed"

"togas"

"together"

"togetherness"

"togethers"

"togged"

"toggery"

"togging"

"toggle"

"toggled"

"toggler"

"togglers"

"toggles"

"toggling"

"togo"

"togs"

"toil"

"toiled"

"toiler"

"toilers"

"toilet"

"toileted"

"toileting"

"toiletries"

"toiletry"

"toilets"

"toilette"

"toilettes"

"toilful"

"toiling"

"toils"

"toilsome"

"toilworn"

"toited"

"tokay"

"tokays"

"toke"

"toked"

"token"

"tokened"

"tokening"

"tokenism"

"tokenisms"

"tokenize"

"tokens"

"tokes"

"toking"

"tokonoma"

"tokonomas"

"tokyo"

"tokyoite"

"tokyoites"

"tolbutamide"

"told"

"tole"

"toledo"

"toledos"

"tolerable"

"tolerably"

"tolerance"

"tolerances"

"tolerant"

"tolerantly"

"tolerate"

"tolerated"

"tolerates"

"tolerating"

"toleration"

"tolerative"

"tolerator"

"tolerators"

"toll"

"tollage"

"tollages"

"tollbars"

"tollbooth"

"tollbooths"

"tolled"

"toller"

"tollers"

"tollgate"

"tollgates"

"tollgatherer"

"tollhouse"

"tolling"

"tollman"

"tollmen"

"tolls"

"tollway"

"tollways"

"tolstoy"

"toluene"

"toluol"

"toluyl"

"tolyls"

"tom"

"tomahawk"

"tomahawked"

"tomahawks"

"tomato"

"tomatoes"

"tomb"

"tombed"

"tombing"

"tomboy"

"tomboys"

"tombs"

"tombstone"

"tombstones"

"tomcat"

"tomcats"

"tome"

"tomes"

"tomfool"

"tomfoolery"

"tomfools"

"tommies"

"tommy"

"tommyrot"

"tommyrots"

"tomo"

"tomogram"

"tomograms"

"tomograph"

"tomographic"

"tomographies"

"tomomania"

"tomorrow"

"tomorrows"

"toms"

"tomtit"

"tomtits"

"ton"

"tonal"

"tonalities"

"tonality"

"tonally"

"tone"

"toned"

"toneless"

"toner"

"toners"

"tones"

"tonetics"

"tonettes"

"tong"

"tongas"

"tonged"

"tonger"

"tongers"

"tonging"

"tongs"

"tongue"

"tongued"

"tongueless"

"tongues"

"tonguing"

"tonguings"

"tonic"

"tonically"

"tonicity"

"tonics"

"tonier"

"tonies"

"toniest"

"tonight"

"tonights"

"toning"

"tonishly"

"tonitrophobia"

"tonnage"

"tonnages"

"tonne"

"tonneau"

"tonneaus"

"tonneaux"

"tonner"

"tonners"

"tonnes"

"tonnish"

"tonometer"

"tons"

"tonsil"

"tonsilar"

"tonsillar"

"tonsillectomies"

"tonsillectomy"

"tonsillitis"

"tonsillotomies"

"tonsillotomy"

"tonsils"

"tonsorial"

"tonsure"

"tonsured"

"tonsures"

"tonsuring"

"tonuses"

"tony"

"too"

"took"

"tool"

"toolbox"

"toolboxes"

"tooled"

"tooler"

"toolers"

"toolhead"

"toolholder"

"tooling"

"toolings"

"toolless"

"toolmaker"

"toolmakers"

"toolmaking"

"toolroom"

"toolrooms"

"tools"

"toolshed"

"toolsheds"

"toot"

"tooted"

"tooter"

"tooters"

"tooth"

"toothache"

"toothaches"

"toothbrush"

"toothbrushes"

"toothed"

"toothier"

"toothiest"

"toothily"

"toothing"

"toothless"

"toothpaste"

"toothpastes"

"toothpick"

"toothpicks"

"tooths"

"toothsome"

"toothsomeness"

"toothy"

"tooting"

"tootle"

"tootled"

"tootler"

"tootlers"

"tootles"

"tootling"

"toots"

"tootsie"

"tootsies"

"tootsy"

"top"

"topaz"

"topazes"

"topcoat"

"topcoats"

"topcrosses"

"tope"

"toped"

"topeka"

"toper"

"topers"

"topes"

"topflight"

"topful"

"topfull"

"topiaries"

"topiary"

"topic"

"topical"

"topicality"

"topically"

"topics"

"toping"

"topkick"

"topkicks"

"topknot"

"topknots"

"topless"

"toplessness"

"toploftier"

"topmast"

"topmasts"

"topmost"

"topnotch"

"topographer"

"topographers"

"topographic"

"topographical"

"topographically"

"topographies"

"topography"

"topological"

"topologically"

"topologies"

"topology"

"toponymics"

"topophobia"

"topophone"

"topos"

"topotypes"

"topped"

"topper"

"toppers"

"topping"

"toppings"

"topple"

"toppled"

"topples"

"toppling"

"tops"

"topsail"

"topsails"

"topside"

"topsider"

"topsiders"

"topsides"

"topsoil"

"topsoiled"

"topsoiling"

"topsoils"

"topstitch"

"topstone"

"topwork"

"toque"

"toques"

"tor"

"tora"

"torah"

"torahs"

"toras"

"torc"

"torch"

"torchbearer"

"torchbearers"

"torched"

"torchere"

"torcheres"

"torches"

"torchier"

"torchiers"

"torching"

"torchlight"

"torcs"

"tore"

"toreador"

"toreadors"

"torero"

"toreros"

"tores"

"toreutics"

"tories"

"torii"

"torment"

"tormented"

"tormentedly"

"tormenter"

"tormenters"

"tormenting"

"tormentingly"

"tormentor"

"tormentors"

"torments"

"torn"

"tornadic"

"tornado"

"tornadoes"

"tornados"

"toro"

"toroid"

"toroidal"

"toroids"

"toronto"

"toros"

"torpedo"

"torpedoed"

"torpedoes"

"torpedoing"

"torpedolike"

"torpid"

"torpidity"

"torpidly"

"torpids"

"torpor"

"torpors"

"torque"

"torqued"

"torquer"

"torquers"

"torques"

"torquing"

"torr"

"torrent"

"torrential"

"torrents"

"torrid"

"torrider"

"torridest"

"torridity"

"torridly"

"torridness"

"tors"

"torsi"

"torsiograph"

"torsion"

"torsional"

"torsionally"

"torsions"

"torso"

"torsoes"

"torsos"

"tort"

"torte"

"tortes"

"torticollis"

"tortilla"

"tortillas"

"tortoise"

"tortoises"

"tortoiseshell"

"tortoni"

"tortrix"

"torts"

"tortuosities"

"tortuosity"

"tortuous"

"tortuously"

"tortuousness"

"torture"

"tortured"

"torturedly"

"torturer"

"torturers"

"tortures"

"torturing"

"torturous"

"torturously"

"torus"

"tory"

"tosh"

"toshes"

"toss"

"tossed"

"tosser"

"tossers"

"tosses"

"tossing"

"tosspot"

"tosspots"

"tossup"

"tossups"

"tost"

"tot"

"totable"

"total"

"totaled"

"totaling"

"totalism"

"totalisms"

"totalitarian"

"totalitarianism"

"totalitarians"

"totalities"

"totality"

"totalizator"

"totalizators"

"totalize"

"totalized"

"totalizer"

"totalizes"

"totalizing"

"totalled"

"totalling"

"totally"

"totals"

"tote"

"toted"

"totem"

"totemic"

"totemism"

"totemisms"

"totemist"

"totemists"

"totemites"

"totems"

"toter"

"toters"

"totes"

"tother"

"toting"

"totipotencies"

"totipotency"

"totipotential"

"totipotentiality"

"toto"

"tots"

"totted"

"totter"

"tottered"

"totterer"

"totterers"

"tottering"

"totters"

"tottery"

"totting"

"toucan"

"toucans"

"touch"

"touchable"

"touchback"

"touchdown"

"touchdowns"

"touche"

"touched"

"toucher"

"touchers"

"touches"

"touchier"

"touchiest"

"touchily"

"touchiness"

"touching"

"touchingly"

"touchstone"

"touchstones"

"touchup"

"touchups"

"touchy"

"tough"

"toughen"

"toughened"

"toughener"

"tougheners"

"toughening"

"toughens"

"tougher"

"toughest"

"toughie"

"toughies"

"toughish"

"toughly"

"toughness"

"toughs"

"toughy"

"toupee"

"toupees"

"tour"

"toured"

"tourer"

"tourers"

"touring"

"tourings"

"tourism"

"tourisms"

"tourist"

"tourists"

"touristy"

"tourmaline"

"tournament"

"tournaments"

"tourney"

"tourneyed"

"tourneying"

"tourneys"

"tourniquet"

"tourniquets"

"tours"

"tousle"

"tousled"

"tousles"

"tousling"

"tout"

"touted"

"touter"

"touters"

"touting"

"touts"

"touzle"

"touzled"

"touzles"

"tov"

"tovarich"

"tovariches"

"tovarish"

"tovarishes"

"tow"

"towability"

"towable"

"towage"

"towages"

"toward"

"towardly"

"towards"

"towaway"

"towaways"

"towboat"

"towboats"

"towed"

"towel"

"toweled"

"toweling"

"towelings"

"towelled"

"towelling"

"towels"

"tower"

"towered"

"towerier"

"toweriest"

"towering"

"toweringly"

"towers"

"towery"

"towhead"

"towheaded"

"towheads"

"towhee"

"towhees"

"towies"

"towing"

"towline"

"towlines"

"town"

"townfolk"

"townhouse"

"townhouses"

"townie"

"townies"

"townish"

"townless"

"townlet"

"townlets"

"towns"

"townsfolk"

"township"

"townships"

"townsite"

"townsman"

"townsmen"

"townspeople"

"townswoman"

"townswomen"

"townwear"

"townwears"

"towny"

"towpath"

"towpaths"

"towrope"

"towropes"

"tows"

"toxaemia"

"toxaemic"

"toxemia"

"toxemias"

"toxemic"

"toxic"

"toxical"

"toxically"

"toxicant"

"toxicants"

"toxicities"

"toxicity"

"toxicoid"

"toxicologic"

"toxicological"

"toxicologically"

"toxicologist"

"toxicologists"

"toxicology"

"toxicomania"

"toxicophobia"

"toxified"

"toxify"

"toxifying"

"toxigenicities"

"toxin"

"toxins"

"toxiphobia"

"toxoids"

"toxophily"

"toy"

"toyed"

"toyer"

"toyers"

"toying"

"toyish"

"toyon"

"toyons"

"toyos"

"toyota"

"toyotas"

"toys"

"tpk"

"TPM"

"trace"

"traceability"

"traceable"

"traceableness"

"traceably"

"traced"

"tracer"

"traceries"

"tracers"

"tracery"

"traces"

"trachea"

"tracheae"

"tracheal"

"tracheas"

"tracheids"

"tracheobronchial"

"tracheotomies"

"tracheotomize"

"tracheotomized"

"tracheotomizing"

"tracheotomy"

"trachoma"

"trachomas"

"tracing"

"tracings"

"track"

"track weight"

"trackable"

"trackage"

"trackages"

"tracked"

"tracker"

"tracker womenâ€™s health app"

"trackers"

"tracking"

"tracking tools"

"trackings"

"trackless"

"trackman"

"trackmen"

"tracks"

"trackway"

"tract"

"tractability"

"tractable"

"tractably"

"tractate"

"traction"

"tractional"

"tractions"

"tractive"

"tractor"

"tractors"

"tracts"

"tradable"

"trade"

"tradeable"

"traded"

"trademark"

"trademarks"

"tradename"

"tradeoff"

"tradeoffs"

"trader"

"traders"

"tradership"

"trades"

"tradesfolk"

"tradesman"

"tradesmen"

"tradespeople"

"trading"

"tradition"

"traditional"

"traditionalism"

"traditionalist"

"traditionalistic"

"traditionalists"

"traditionalize"

"traditionalized"

"traditionally"

"traditionary"

"traditionless"

"traditions"

"traditores"

"traduce"

"traduced"

"traducement"

"traducements"

"traducer"

"traducers"

"traduces"

"traducing"

"traduction"

"traffic"

"trafficable"

"traffick"

"trafficked"

"trafficker"

"traffickers"

"trafficking"

"trafficks"

"traffics"

"trafficway"

"tragedian"

"tragedians"

"tragedienne"

"tragediennes"

"tragedies"

"tragedy"

"tragic"

"tragical"

"tragically"

"tragicomedies"

"tragicomedy"

"tragicomic"

"trail"

"trail data"

"trailblazer"

"trailblazers"

"trailblazing"

"trailed"

"trailer"

"trailered"

"trailering"

"trailers"

"trailing"

"trails"

"train"

"trainable"

"trained"

"trainee"

"trainees"

"trainer"

"trainers"

"trainful"

"trainfuls"

"training"

"training diary"

"trainings"

"trainload"

"trainman"

"trainmaster"

"trainmen"

"trains"

"trainsick"

"trainsickness"

"trainway"

"trainways"

"traipse"

"traipsed"

"traipses"

"traipsing"

"trait"

"traitor"

"traitoress"

"traitorism"

"traitorous"

"traitorously"

"traitorousness"

"traitors"

"traitress"

"traitresses"

"traits"

"trajected"

"trajectories"

"trajectory"

"trajects"

"tram"

"tramcar"

"tramcars"

"trameled"

"trameling"

"tramell"

"tramelled"

"tramelling"

"tramells"

"tramels"

"tramless"

"tramline"

"tramlines"

"trammed"

"trammel"

"trammeled"

"trammeling"

"trammelled"

"trammelling"

"trammels"

"tramming"

"tramp"

"tramped"

"tramper"

"trampers"

"tramping"

"trampish"

"trample"

"trampled"

"trampler"

"tramplers"

"tramples"

"trampling"

"trampoline"

"trampoliner"

"trampoliners"

"trampolines"

"trampolinist"

"trampolinists"

"tramps"

"tramroad"

"tramroads"

"trams"

"tramway"

"tramways"

"trance"

"tranced"

"trances"

"trancing"

"tranquil"

"tranquil mind"

"tranquiler"

"tranquility"

"tranquilize"

"tranquilized"

"tranquilizer"

"tranquilizers"

"tranquilizes"

"tranquilizing"

"tranquillity"

"tranquillize"

"tranquillized"

"tranquillizer"

"tranquillizing"

"tranquilly"

"transact"

"transacted"

"transacting"

"transaction"

"transactional"

"transactions"

"transactor"

"transacts"

"transalpine"

"transatlantic"

"transborder"

"transceiver"

"transceivers"

"transcend"

"transcendant"

"transcended"

"transcendence"

"transcendency"

"transcendent"

"transcendental"

"transcendentalism"

"transcendentalist"

"transcendentalists"

"transcendentalizm"

"transcendentally"

"transcendently"

"transcending"

"transcends"

"transcontinental"

"transcribe"

"transcribed"

"transcriber"

"transcribers"

"transcribes"

"transcribing"

"transcript"

"transcription"

"transcriptions"

"transcripts"

"transdesert"

"transduce"

"transducer"

"transducers"

"transducing"

"transect"

"transected"

"transects"

"transept"

"transepts"

"transequatorial"

"transfer"

"transferability"

"transferable"

"transferal"

"transferals"

"transferee"

"transference"

"transferer"

"transferrable"

"transferral"

"transferrals"

"transferred"

"transferrer"

"transferrers"

"transferring"

"transferror"

"transfers"

"transfiguration"

"transfigurations"

"transfigure"

"transfigured"

"transfigures"

"transfiguring"

"transfix"

"transfixed"

"transfixes"

"transfixing"

"transfixion"

"transfixt"

"transform"

"transformation"

"transformations"

"transformed"

"transformer"

"transformers"

"transforming"

"transforms"

"transfrontier"

"transfusable"

"transfuse"

"transfused"

"transfuser"

"transfusers"

"transfuses"

"transfusing"

"transfusion"

"transfusional"

"transfusions"

"transgress"

"transgressed"

"transgresses"

"transgressing"

"transgression"

"transgressions"

"transgressive"

"transgressor"

"transgressors"

"tranship"

"transhipment"

"transhipping"

"tranships"

"transience"

"transiencies"

"transiency"

"transient"

"transiently"

"transients"

"transisthmian"

"transistor"

"transistorize"

"transistorized"

"transistorizes"

"transistorizing"

"transistors"

"transit"

"transited"

"transiting"

"transition"

"transitional"

"transitionally"

"transitions"

"transitive"

"transitively"

"transitiveness"

"transitivity"

"transitorily"

"transitoriness"

"transitory"

"transits"

"translatable"

"translate"

"translated"

"translates"

"translating"

"translation"

"translations"

"translative"

"translator"

"translators"

"transliterate"

"transliterated"

"transliterates"

"transliterating"

"transliteration"

"transliterations"

"translucence"

"translucencies"

"translucency"

"translucent"

"translucently"

"translucid"

"transmarine"

"transmen"

"transmigrate"

"transmigrated"

"transmigrates"

"transmigrating"

"transmigration"

"transmigrations"

"transmigrator"

"transmigrators"

"transmigratory"

"transmissibility"

"transmissible"

"transmission"

"transmissions"

"transmissive"

"transmissometer"

"transmit"

"transmits"

"transmittable"

"transmittal"

"transmittals"

"transmittance"

"transmittances"

"transmitted"

"transmitter"

"transmitters"

"transmittible"

"transmitting"

"transmogrification"

"transmogrifications"

"transmogrified"

"transmogrifies"

"transmogrify"

"transmogrifying"

"transmutable"

"transmutation"

"transmutations"

"transmute"

"transmuted"

"transmutes"

"transmuting"

"transnational"

"transoceanic"

"transom"

"transoms"

"transonic"

"transorbital"

"transpacific"

"transparencies"

"transparency"

"transparent"

"transparently"

"transpiration"

"transpire"

"transpired"

"transpires"

"transpiring"

"transplant"

"transplantation"

"transplantations"

"transplanted"

"transplanter"

"transplanters"

"transplanting"

"transplants"

"transpolar"

"transponder"

"transponders"

"transport"

"transportability"

"transportable"

"transportables"

"transportal"

"transportation"

"transportational"

"transported"

"transportee"

"transporter"

"transporters"

"transporting"

"transports"

"transpose"

"transposed"

"transposes"

"transposing"

"transposition"

"transpositions"

"transsexual"

"transsexualism"

"transsexuals"

"transship"

"transshipment"

"transshipped"

"transshipping"

"transships"

"transubstantiate"

"transubstantiation"

"transverse"

"transversely"

"transverses"

"transvestism"

"transvestite"

"transvestites"

"transvestitism"

"trap"

"trapdoor"

"trapdoors"

"trapeze"

"trapezes"

"trapezium"

"trapeziums"

"trapezoid"

"trapezoidal"

"trapezoids"

"trapped"

"trapper"

"trappers"

"trapping"

"trappings"

"traps"

"trapshooting"

"trapt"

"trash"

"trashed"

"trashes"

"trashier"

"trashiest"

"trashily"

"trashing"

"trashman"

"trashmen"

"trashy"

"trauma"

"traumas"

"traumata"

"traumatic"

"traumatically"

"traumatism"

"traumatization"

"traumatize"

"traumatized"

"traumatizes"

"traumatizing"

"traumatologies"

"traumatology"

"travail"

"travailed"

"travailing"

"travails"

"trave"

"travel"

"travelable"

"traveled"

"traveler"

"travelers"

"traveling"

"travellable"

"travelled"

"traveller"

"travellers"

"travelling"

"travelog"

"travelogs"

"travelogue"

"travelogues"

"travels"

"traversable"

"traversal"

"traversals"

"traverse"

"traversed"

"traverser"

"traverses"

"traversing"

"travertine"

"travestied"

"travesties"

"travesty"

"travestying"

"travois"

"travoise"

"travoises"

"trawl"

"trawled"

"trawler"

"trawlers"

"trawleys"

"trawling"

"trawls"

"tray"

"trayful"

"trayfuls"

"trays"

"treacheries"

"treacherous"

"treacherously"

"treacherousness"

"treachery"

"treacle"

"treacles"

"treacly"

"tread"

"treaded"

"treader"

"treaders"

"treading"

"treadle"

"treadled"

"treadler"

"treadles"

"treadmill"

"treadmill run"

"treadmills"

"treads"

"treason"

"treasonable"

"treasonably"

"treasonous"

"treasons"

"treasurable"

"treasure"

"treasured"

"treasurer"

"treasurers"

"treasurership"

"treasures"

"treasuries"

"treasuring"

"treasury"

"treasuryship"

"treat"

"treatabilities"

"treatability"

"treatable"

"treated"

"treater"

"treaters"

"treaties"

"treating"

"treatise"

"treatises"

"treatment"

"treatments"

"treats"

"treaty"

"treble"

"trebled"

"trebles"

"trebling"

"trebly"

"trechometer"

"tree"

"treed"

"treeing"

"treeless"

"trees"

"treetop"

"treetops"

"tref"

"trefoil"

"trefoils"

"trek"

"trekked"

"trekker"

"trekkers"

"trekking"

"treks"

"trellis"

"trellised"

"trellises"

"trellising"

"trematode"

"trematodes"

"tremble"

"trembled"

"trembler"

"tremblers"

"trembles"

"tremblier"

"trembliest"

"trembling"

"tremblingly"

"trembly"

"tremendous"

"tremendously"

"tremendousness"

"tremens"

"tremograph"

"tremolo"

"tremolos"

"tremor"

"tremors"

"tremulous"

"tremulously"

"tremulousness"

"trench"

"trenchancy"

"trenchant"

"trenchantly"

"trenched"

"trencher"

"trencherman"

"trenchermen"

"trenchers"

"trenches"

"trenching"

"trend"

"trended"

"trendier"

"trendiest"

"trendily"

"trending"

"trends"

"trendy"

"trenton"

"trepan"

"trepanned"

"trepans"

"trephination"

"trephine"

"trephined"

"trephines"

"trephining"

"trepid"

"trepidation"

"trepidations"

"trespass"

"trespassed"

"trespasser"

"trespassers"

"trespasses"

"trespassing"

"trespassory"

"tress"

"tressed"

"tresses"

"tressier"

"tressiest"

"tressy"

"trestle"

"trestles"

"trets"

"trews"

"trey"

"treys"

"triable"

"triad"

"triadic"

"triadics"

"triadism"

"triadisms"

"triads"

"triage"

"triages"

"trial"

"trial version"

"trials"

"triangle"

"triangles"

"triangular"

"triangularly"

"triangulate"

"triangulated"

"triangulates"

"triangulating"

"triangulation"

"triangulations"

"triangulator"

"triarchy"

"triassic"

"triatomic"

"triaxial"

"tribade"

"tribades"

"tribadic"

"tribadism"

"tribal"

"tribally"

"tribe"

"tribes"

"tribesman"

"tribesmen"

"tribeswoman"

"tribeswomen"

"tribology"

"tribometer"

"tribulation"

"tribulations"

"tribunal"

"tribunals"

"tribunate"

"tribune"

"tribunes"

"tribuneship"

"tributaries"

"tributary"

"tribute"

"tributes"

"trice"

"triced"

"tricentennial"

"tricentennials"

"triceps"

"tricepses"

"triceratops"

"triceratopses"

"trices"

"trichinella"

"trichiniasis"

"trichinoses"

"trichinosis"

"trichinous"

"trichlorethylene"

"trichlorethylenes"

"trichloromethane"

"trichloromethanes"

"trichology"

"trichotillomania"

"trichroic"

"trichrome"

"trick"

"tricked"

"tricker"

"trickeries"

"trickers"

"trickery"

"trickie"

"trickier"

"trickiest"

"trickily"

"trickiness"

"tricking"

"trickingly"

"trickish"

"trickishly"

"trickishness"

"trickle"

"trickled"

"trickles"

"tricklier"

"trickling"

"trickly"

"tricks"

"tricksier"

"tricksiest"

"trickster"

"tricksters"

"tricksy"

"tricky"

"tricolor"

"tricolors"

"tricorn"

"tricorne"

"tricornes"

"tricorns"

"tricot"

"tricots"

"tricuspid"

"tricycle"

"tricycles"

"trident"

"tridents"

"tried"

"triennial"

"triennially"

"trier"

"triers"

"tries"

"trifacial"

"trifid"

"trifle"

"trifled"

"trifler"

"triflers"

"trifles"

"trifling"

"triflingly"

"triflings"

"trifocal"

"trifocals"

"trifold"

"trifoliate"

"trifolium"

"triform"

"trifurcated"

"trifurcating"

"trifurcation"

"trig"

"trigamist"

"trigamy"

"trigger"

"triggered"

"triggering"

"triggers"

"triggest"

"trigging"

"triglyceride"

"triglycerides"

"trigon"

"trigonal"

"trigonometer"

"trigonometric"

"trigonometrical"

"trigonometrically"

"trigonometry"

"trigons"

"trigraph"

"trigraphs"

"trihedra"

"trihybrid"

"trijet"

"trijets"

"trilateral"

"triliteral"

"trill"

"trilled"

"triller"

"trillers"

"trilling"

"trillion"

"trillions"

"trillionth"

"trillionths"

"trillium"

"trilliums"

"trills"

"trilobal"

"trilobate"

"trilobed"

"trilogies"

"trilogy"

"trim"

"trimaran"

"trimarans"

"trimester"

"trimesters"

"trimeter"

"trimly"

"trimmed"

"trimmer"

"trimmers"

"trimmest"

"trimming"

"trimmings"

"trimness"

"trimonthly"

"trimorph"

"trimorphs"

"trimotor"

"trimotors"

"trims"

"trinal"

"trinary"

"trine"

"trined"

"trines"

"trinidad"

"trining"

"trinitarian"

"trinitarianism"

"trinitarians"

"trinities"

"trinitrotoluene"

"trinity"

"trinket"

"trinketed"

"trinketing"

"trinkets"

"trinodal"

"trio"

"triode"

"triodes"

"triolet"

"triolets"

"trios"

"trioxide"

"trioxides"

"trip"

"tripart"

"tripartite"

"tripe"

"tripedal"

"tripes"

"triphase"

"triplane"

"triplanes"

"triple"

"tripled"

"triples"

"triplet"

"triplets"

"triplex"

"triplexes"

"triplicate"

"triplicated"

"triplicates"

"triplicating"

"triplication"

"triplications"

"tripling"

"triploid"

"triply"

"tripod"

"tripodal"

"tripodic"

"tripods"

"tripoli"

"tripped"

"tripper"

"trippers"

"trippets"

"tripping"

"trippings"

"trips"

"triptych"

"triptychs"

"trireme"

"triremes"

"trisaccharide"

"triscele"

"trisect"

"trisected"

"trisecting"

"trisection"

"trisections"

"trisects"

"triskaidekaphobe"

"triskaidekaphobes"

"triskaidekaphobia"

"triskeles"

"tristate"

"triste"

"tristezas"

"trite"

"tritely"

"triteness"

"triter"

"tritest"

"triticale"

"tritium"

"tritiums"

"triton"

"tritone"

"tritones"

"tritons"

"triturable"

"triturate"

"triturated"

"triturates"

"triturating"

"trituration"

"triturator"

"triturators"

"triumph"

"triumphal"

"triumphant"

"triumphantly"

"triumphed"

"triumphing"

"triumphs"

"triumvir"

"triumviral"

"triumvirate"

"triumvirates"

"triumviri"

"triumvirs"

"triune"

"triunes"

"triunities"

"triunity"

"trivalent"

"trivalve"

"trivalves"

"trivet"

"trivets"

"trivia"

"trivial"

"trivialities"

"triviality"

"trivially"

"trivium"

"trochaic"

"trochaics"

"troche"

"trocheameter"

"trochee"

"trochees"

"troches"

"trochoid"

"trochoids"

"trod"

"trodden"

"trode"

"troglodyte"

"troglodytes"

"troika"

"troikas"

"troilus"

"trois"

"trojan"

"trojans"

"troll"

"trolled"

"troller"

"trollers"

"trolley"

"trolleyed"

"trolleying"

"trolleys"

"trollied"

"trollies"

"trolling"

"trollings"

"trollop"

"trollops"

"trollopy"

"trolls"

"trolly"

"trollying"

"trombone"

"trombones"

"trombonist"

"trombonists"

"tromometer"

"tromp"

"trompe"

"tromped"

"trompes"

"tromping"

"tromps"

"troop"

"trooped"

"trooper"

"troopers"

"trooping"

"troops"

"troopship"

"troopships"

"trop"

"trope"

"tropes"

"trophic"

"trophied"

"trophies"

"trophism"

"trophology"

"trophy"

"trophying"

"tropia"

"tropic"

"tropical"

"tropically"

"tropics"

"tropin"

"tropine"

"tropins"

"tropism"

"tropisms"

"tropometer"

"troposphere"

"tropospheric"

"troppo"

"trot"

"troth"

"trothed"

"trothing"

"troths"

"trotlines"

"trots"

"trotted"

"trotter"

"trotters"

"trotting"

"troubadour"

"troubadours"

"trouble"

"troubled"

"troublemaker"

"troublemakers"

"troubler"

"troublers"

"troubles"

"troubleshoot"

"troubleshooter"

"troubleshooters"

"troubleshooting"

"troubleshoots"

"troubleshot"

"troublesome"

"troublesomely"

"troubling"

"troublous"

"trough"

"troughs"

"trounce"

"trounced"

"trouncer"

"trouncers"

"trounces"

"trouncing"

"troupe"

"trouped"

"trouper"

"troupers"

"troupes"

"trouping"

"trouser"

"trousers"

"trousseau"

"trousseaus"

"trousseaux"

"trout"

"troutier"

"troutiest"

"trouts"

"trouty"

"trove"

"trover"

"trovers"

"troves"

"trow"

"trowed"

"trowel"

"troweled"

"troweler"

"trowelers"

"troweling"

"trowelled"

"trowelling"

"trowels"

"trowing"

"trows"

"trowsers"

"troy"

"troys"

"truancies"

"truancy"

"truant"

"truanted"

"truanting"

"truantries"

"truantry"

"truants"

"truce"

"truced"

"truces"

"trucing"

"truck"

"truckage"

"truckdriver"

"trucked"

"trucker"

"truckers"

"trucking"

"truckings"

"truckle"

"truckled"

"truckler"

"trucklers"

"truckles"

"truckling"

"truckload"

"truckloads"

"truckman"

"truckmaster"

"truckmen"

"trucks"

"truculence"

"truculency"

"truculent"

"truculently"

"trudge"

"trudged"

"trudger"

"trudgers"

"trudges"

"trudging"

"true"

"trueblue"

"trueblues"

"trueborn"

"trued"

"trueing"

"truelove"

"trueloves"

"trueness"

"truer"

"trues"

"truest"

"truffle"

"truffled"

"truffles"

"truing"

"truism"

"truisms"

"truistic"

"trull"

"trulls"

"truly"

"truman"

"trump"

"trumped"

"trumperies"

"trumpery"

"trumpet"

"trumpeted"

"trumpeter"

"trumpeters"

"trumpeting"

"trumpets"

"trumping"

"trumps"

"truncate"

"truncated"

"truncates"

"truncating"

"truncation"

"truncations"

"truncheon"

"truncheons"

"trundle"

"trundled"

"trundler"

"trundlers"

"trundles"

"trundling"

"trunk"

"trunked"

"trunks"

"trunkway"

"trunnels"

"trunnion"

"trunnions"

"truss"

"trussed"

"trusser"

"trussers"

"trusses"

"trussing"

"trussings"

"trust"

"trustability"

"trustable"

"trustbuster"

"trustbusting"

"trusted"

"trustee"

"trusteed"

"trusteeing"

"trustees"

"trusteeship"

"trusteeships"

"truster"

"trusters"

"trustful"

"trustfully"

"trustfulness"

"trustier"

"trusties"

"trustiest"

"trustified"

"trustifying"

"trustily"

"trusting"

"trusts"

"trustwoman"

"trustwomen"

"trustworthily"

"trustworthiness"

"trustworthy"

"trusty"

"truth"

"truthful"

"truthfully"

"truthfulness"

"truthless"

"truths"

"try"

"trying"

"tryingly"

"tryout"

"tryouts"

"trypsin"

"tryptic"

"tryptophane"

"tryst"

"trysted"

"tryster"

"trysters"

"trystes"

"trysting"

"trysts"

"tsar"

"tsardom"

"tsardoms"

"tsarevna"

"tsarevnas"

"tsarina"

"tsarinas"

"tsarism"

"tsarisms"

"tsarist"

"tsarists"

"tsaritza"

"tsaritzas"

"tsars"

"tsetse"

"tsetses"

"tsiganology"

"tsimmes"

"tsked"

"tsking"

"tsktsked"

"tsktsking"

"tsp"

"tsuba"

"tsunami"

"tsunamic"

"tsunamis"

"tsuris"

"TTE"

"tty"

"tuataras"

"tub"

"tuba"

"tubal"

"tubas"

"tubbable"

"tubbed"

"tubber"

"tubbers"

"tubbier"

"tubbiest"

"tubbiness"

"tubbing"

"tubby"

"tube"

"tubectomies"

"tubectomy"

"tubed"

"tubeless"

"tuber"

"tubercle"

"tubercled"

"tubercles"

"tubercular"

"tuberculin"

"tuberculoid"

"tuberculoses"

"tuberculosis"

"tuberculous"

"tuberculously"

"tuberoid"

"tuberose"

"tuberoses"

"tuberosity"

"tuberous"

"tubers"

"tubes"

"tubework"

"tubful"

"tubifexes"

"tubiform"

"tubing"

"tubings"

"tubs"

"tubular"

"tubularly"

"tubulate"

"tubule"

"tubules"

"tuck"

"tuckahoes"

"tucked"

"tucker"

"tuckered"

"tuckering"

"tuckers"

"tucket"

"tuckets"

"tucking"

"tucks"

"tucson"

"tudor"

"tuesday"

"tuesdays"

"tufa"

"tufaceous"

"tufas"

"tuff"

"tuffet"

"tuffets"

"tuffs"

"tuft"

"tufted"

"tufter"

"tufters"

"tuftier"

"tuftiest"

"tuftily"

"tufting"

"tufts"

"tufty"

"tug"

"tugboat"

"tugboats"

"tugged"

"tugger"

"tuggers"

"tugging"

"tugs"

"tuition"

"tuitions"

"tularemia"

"tularemic"

"tules"

"tulip"

"tulipomania"

"tulips"

"tulle"

"tulles"

"tulsa"

"tumble"

"tumbled"

"tumbledown"

"tumbler"

"tumblers"

"tumbles"

"tumbleweed"

"tumbleweeds"

"tumbling"

"tumblings"

"tumbrel"

"tumbrels"

"tumbrils"

"tumefied"

"tumefies"

"tumeric"

"tumescence"

"tumescent"

"tumid"

"tumidity"

"tummies"

"tummy"

"tumor"

"tumoral"

"tumorous"

"tumors"

"tumour"

"tumours"

"tumps"

"tumult"

"tumults"

"tumultuous"

"tumultuousness"

"tumultus"

"tumulus"

"tumuluses"

"tun"

"tuna"

"tunability"

"tunable"

"tunably"

"tunas"

"tundra"

"tundras"

"tune"

"tuneable"

"tuneably"

"tuned"

"tuneful"

"tunefully"

"tunefulness"

"tuneless"

"tunelessly"

"tuner"

"tuners"

"tunes"

"tuneup"

"tuneups"

"tungsten"

"tungstenic"

"tungstens"

"tunic"

"tunics"

"tuning"

"tunings"

"tunis"

"tunisia"

"tunisian"

"tunisians"

"tunned"

"tunnel"

"tunneled"

"tunneler"

"tunnelers"

"tunneling"

"tunnelled"

"tunneller"

"tunnellers"

"tunnelling"

"tunnels"

"tunney"

"tunnies"

"tunning"

"tunny"

"tuns"

"tup"

"tupelo"

"tupelos"

"tupped"

"tuppence"

"tuppences"

"tuppenny"

"tupping"

"tups"

"tuque"

"tuques"

"turban"

"turbaned"

"turbans"

"turbeths"

"turbid"

"turbidimeter"

"turbidities"

"turbidity"

"turbidly"

"turbidness"

"turbinate"

"turbine"

"turbines"

"turbit"

"turbo"

"turbocar"

"turbocars"

"turbocharger"

"turbofan"

"turbofans"

"turbojet"

"turbojets"

"turboprop"

"turboprops"

"turbos"

"turbot"

"turbots"

"turbulence"

"turbulency"

"turbulent"

"turbulently"

"turd"

"turds"

"tureen"

"tureens"

"turf"

"turfed"

"turfier"

"turfing"

"turfless"

"turfs"

"turfy"

"turgencies"

"turgescence"

"turgid"

"turgidities"

"turgidity"

"turgidly"

"turgometer"

"turgors"

"turk"

"turkey"

"turkeys"

"turkois"

"turks"

"turmeric"

"turmerics"

"turmoil"

"turmoiled"

"turmoiling"

"turmoils"

"turn"

"turnable"

"turnabout"

"turnabouts"

"turnaround"

"turnarounds"

"turnbuckle"

"turnbuckles"

"turncoat"

"turncoats"

"turndown"

"turndowns"

"turned"

"turner"

"turneries"

"turners"

"turnery"

"turnhall"

"turning"

"turnings"

"turnip"

"turnips"

"turnkey"

"turnkeys"

"turnoff"

"turnoffs"

"turnout"

"turnouts"

"turnover"

"turnovers"

"turnpike"

"turnpikes"

"turns"

"turnspit"

"turnspits"

"turnstile"

"turnstiles"

"turntable"

"turntables"

"turnup"

"turnups"

"turpentine"

"turpitude"

"turps"

"turquoise"

"turquoises"

"turret"

"turreted"

"turrets"

"turtle"

"turtled"

"turtledove"

"turtledoves"

"turtleneck"

"turtlenecks"

"turtler"

"turtlers"

"turtles"

"turtling"

"tusche"

"tusches"

"tush"

"tushed"

"tushes"

"tushing"

"tusk"

"tusked"

"tusker"

"tuskers"

"tusking"

"tuskless"

"tusks"

"tussle"

"tussled"

"tussles"

"tussling"

"tussock"

"tussocks"

"tussocky"

"tussuck"

"tut"

"tutankhamen"

"tutee"

"tutees"

"tutelage"

"tutelages"

"tutelar"

"tutelaries"

"tutelary"

"tutor"

"tutorage"

"tutorages"

"tutored"

"tutoress"

"tutoresses"

"tutorhood"

"tutorial"

"tutorials"

"tutoring"

"tutors"

"tutorship"

"tutoyered"

"tutrix"

"tuts"

"tutted"

"tutti"

"tutting"

"tutu"

"tutus"

"tux"

"tuxedo"

"tuxedoes"

"tuxedos"

"tuxes"

"tv"

"twaddle"

"twaddled"

"twaddler"

"twaddlers"

"twaddles"

"twaddling"

"twain"

"twains"

"twang"

"twanged"

"twangier"

"twangiest"

"twanging"

"twangle"

"twangled"

"twangler"

"twangles"

"twangs"

"twangy"

"twas"

"twat"

"twats"

"twattle"

"tweak"

"tweaked"

"tweakier"

"tweakiest"

"tweaking"

"tweaks"

"tweaky"

"tweed"

"tweedier"

"tweediest"

"tweedle"

"tweedled"

"tweedles"

"tweeds"

"tweedy"

"tween"

"tweet"

"tweeted"

"tweeter"

"tweeters"

"tweeting"

"tweets"

"tweeze"

"tweezed"

"tweezer"

"tweezers"

"tweezes"

"tweezing"

"twelfth"

"twelfths"

"twelve"

"twelvemo"

"twelvemonth"

"twelvemonths"

"twelvemos"

"twelves"

"twenties"

"twentieth"

"twentieths"

"twenty"

"twerp"

"twerps"

"twice"

"twiddle"

"twiddled"

"twiddler"

"twiddlers"

"twiddles"

"twiddling"

"twier"

"twig"

"twigged"

"twiggier"

"twiggiest"

"twigging"

"twiggy"

"twigless"

"twigs"

"twilight"

"twilights"

"twilit"

"twill"

"twilled"

"twilling"

"twills"

"twin"

"twinborn"

"twine"

"twined"

"twiner"

"twiners"

"twines"

"twinge"

"twinged"

"twingeing"

"twinges"

"twinging"

"twinier"

"twinight"

"twinighter"

"twinighters"

"twining"

"twinkle"

"twinkled"

"twinkler"

"twinklers"

"twinkles"

"twinkling"

"twinkly"

"twinned"

"twinning"

"twinnings"

"twins"

"twinship"

"twinships"

"twiny"

"twirl"

"twirled"

"twirler"

"twirlers"

"twirlier"

"twirliest"

"twirling"

"twirls"

"twirly"

"twirp"

"twirps"

"twist"

"twistable"

"twisted"

"twister"

"twisters"

"twisting"

"twistings"

"twists"

"twit"

"twitch"

"twitched"

"twitcher"

"twitchers"

"twitches"

"twitchier"

"twitchiest"

"twitching"

"twitchingly"

"twitchy"

"twits"

"twitted"

"twitter"

"twittered"

"twittering"

"twitters"

"twittery"

"twitting"

"twixt"

"two"

"twofer"

"twofers"

"twofold"

"twofolds"

"twopence"

"twopences"

"twopenny"

"twos"

"twosome"

"twosomes"

"twothirds"

"tx"

"tycoon"

"tycoons"

"tye"

"tying"

"tyke"

"tykes"

"tyler"

"tymbal"

"tympan"

"tympana"

"tympani"

"tympanic"

"tympanies"

"tympans"

"tympanum"

"tympanums"

"tympany"

"typal"

"type"

"typeable"

"typebar"

"typebars"

"typecase"

"typecast"

"typecasting"

"typecasts"

"typed"

"typeface"

"typefaces"

"types"

"types of diabetes"

"typescript"

"typescripts"

"typeset"

"typesets"

"typesetter"

"typesetters"

"typesetting"

"typewrite"

"typewriter"

"typewriters"

"typewrites"

"typewriting"

"typewritten"

"typewrote"

"typhlograph"

"typhlology"

"typhoid"

"typhoidal"

"typhoids"

"typhomania"

"typhon"

"typhons"

"typhoon"

"typhoons"

"typhous"

"typhus"

"typhuses"

"typic"

"typical"

"typicality"

"typically"

"typicalness"

"typier"

"typiest"

"typification"

"typified"

"typifier"

"typifiers"

"typifies"

"typify"

"typifying"

"typing"

"typist"

"typists"

"typo"

"typographer"

"typographers"

"typographic"

"typographical"

"typographically"

"typographies"

"typography"

"typology"

"typomania"

"typos"

"tyrannic"

"tyrannical"

"tyrannically"

"tyrannicalness"

"tyrannies"

"tyrannis"

"tyrannize"

"tyrannized"

"tyrannizer"

"tyrannizers"

"tyrannizes"

"tyrannizing"

"tyrannosaur"

"tyrannosaurs"

"tyrannosaurus"

"tyrannosauruses"

"tyrannous"

"tyrannously"

"tyranny"

"tyrant"

"tyrants"

"tyre"

"tyred"

"tyres"

"tyro"

"tyros"

"tything"

"tzar"

"tzardom"

"tzardoms"

"tzarevna"

"tzarevnas"

"tzarina"

"tzarinas"

"tzarism"

"tzarisms"

"tzarist"

"tzarists"

"tzaritza"

"tzaritzas"

"tzars"

"tzetze"

"tzetzes"

"tzigane"

"tzimmes"

"tzitzis"

"tzuris"

"uban"

"uberrima"

"uberties"

"ubique"

"ubiquities"

"ubiquitous"

"ubiquitously"

"ubiquity"

"udder"

"udders"

"udometer"

"ufo"

"ufology"

"ufos"

"uganda"

"ugandan"

"ugandans"

"ugh"

"ughs"

"ugli"

"uglier"

"ugliest"

"uglified"

"uglifier"

"uglifiers"

"uglifies"

"uglify"

"uglifying"

"uglily"

"ugliness"

"uglis"

"ugly"

"ugsome"

"uh"

"uhs"

"ukase"

"ukases"

"uke"

"ukelele"

"ukeleles"

"ukes"

"ukraine"

"ukrainian"

"ukrainians"

"ukulele"

"ukuleles"

"ulcer"

"ulcerate"

"ulcerated"

"ulcerates"

"ulcerating"

"ulceration"

"ulcerations"

"ulcerative"

"ulcered"

"ulcering"

"ulcerous"

"ulcers"

"ullage"

"ullages"

"ulna"

"ulnae"

"ulnar"

"ulnas"

"ulster"

"ulsters"

"ult"

"ulterior"

"ulteriorly"

"ultima"

"ultimacies"

"ultimacy"

"ultimas"

"ultimata"

"ultimate"

"ultimately"

"ultimateness"

"ultimates"

"ultimation"

"ultimatum"

"ultimatums"

"ultimo"

"ultra"

"ultracentrifuge"

"ultraconservative"

"ultraconservatives"

"ultrafiche"

"ultrafiches"

"ultrafiltration"

"ultrahazardous"

"ultrahigh"

"ultraism"

"ultraist"

"ultramarine"

"ultramicroscope"

"ultramicroscopic"

"ultramicroscopically"

"ultramicroscopy"

"ultramicrotome"

"ultramodern"

"ultramundane"

"ultrared"

"ultras"

"ultrasonic"

"ultrasonically"

"ultrasonics"

"ultrasonogram"

"ultrasonography"

"ultrasound"

"ultrasound technician"

"ultrastructural"

"ultrastructure"

"ultrasuede"

"ultraviolet"

"ululate"

"ululated"

"ululates"

"ululating"

"ululation"

"ululations"

"ulva"

"ulvas"

"ulysses"

"umbel"

"umbeled"

"umbellate"

"umbels"

"umber"

"umbered"

"umbers"

"umbilical"

"umbilici"

"umbilicus"

"umbilicuses"

"umbles"

"umbra"

"umbrae"

"umbrage"

"umbrageous"

"umbrages"

"umbral"

"umbras"

"umbrella"

"umbrellaed"

"umbrellas"

"umiak"

"umiaks"

"umlaut"

"umlauted"

"umlauting"

"umlauts"

"ump"

"umped"

"umping"

"umpire"

"umpired"

"umpires"

"umpireship"

"umpiring"

"umps"

"umpteen"

"umpteenth"

"umteenth"

"un"

"unabashed"

"unabashedly"

"unabated"

"unabating"

"unabbreviated"

"unable"

"unabridged"

"unabsentmindedness"

"unabsolved"

"unabsorbed"

"unabsorbent"

"unacademic"

"unaccented"

"unaccentuated"

"unacceptable"

"unacceptably"

"unacceptance"

"unaccepted"

"unaccessible"

"unaccidental"

"unacclaimate"

"unacclaimed"

"unacclimated"

"unacclimatized"

"unaccommodating"

"unaccompanied"

"unaccomplished"

"unaccountability"

"unaccountable"

"unaccountably"

"unaccounted"

"unaccredited"

"unaccustomed"

"unacknowledged"

"unacknowledging"

"unacquainted"

"unactionable"

"unactuated"

"unadapted"

"unaddressed"

"unadjourned"

"unadjudicated"

"unadjustable"

"unadjusted"

"unadorned"

"unadulterate"

"unadulterated"

"unadvantageous"

"unadventurous"

"unadvertised"

"unadvisable"

"unadvised"

"unadvisedly"

"unaesthetic"

"unaffected"

"unaffectedly"

"unaffiliated"

"unafraid"

"unaged"

"unaging"

"unaided"

"unaimed"

"unaired"

"unalarmed"

"unalarming"

"unalienable"

"unalienated"

"unaligned"

"unalike"

"unallayed"

"unalleviated"

"unallied"

"unallowable"

"unalloyed"

"unalphabetized"

"unalterable"

"unalterably"

"unaltered"

"unambidextrousness"

"unambiguous"

"unambiguously"

"unambitious"

"unamortized"

"unamplified"

"unamused"

"unamusing"

"unanimated"

"unanimities"

"unanimity"

"unanimous"

"unanimously"

"unannounced"

"unanswerable"

"unanswered"

"unanticipated"

"unapologetic"

"unapologetically"

"unapparent"

"unappealing"

"unappeasable"

"unappeased"

"unappetizing"

"unappetizingly"

"unapplicable"

"unapplied"

"unappointed"

"unapportioned"

"unappreciated"

"unappreciative"

"unapprehensive"

"unapproachable"

"unappropriated"

"unapproved"

"unapproving"

"unapt"

"unarm"

"unarmed"

"unarmored"

"unarms"

"unarrested"

"unartful"

"unartfully"

"unartfulness"

"unarticulate"

"unarticulated"

"unarticulately"

"unartistic"

"unary"

"unascertainable"

"unashamed"

"unasked"

"unaspirated"

"unaspiring"

"unassailable"

"unassailably"

"unassertive"

"unassessed"

"unassigned"

"unassimilated"

"unassisted"

"unassorted"

"unassuming"

"unassumingly"

"unassured"

"unattached"

"unattackable"

"unattainable"

"unattempted"

"unattended"

"unattested"

"unattracted"

"unattractive"

"unauspicious"

"unauthentic"

"unauthenticated"

"unauthorized"

"unavailability"

"unavailable"

"unavailing"

"unavailingly"

"unavenged"

"unavoidability"

"unavoidable"

"unavoidableness"

"unavoidably"

"unavowed"

"unawaked"

"unawakened"

"unaware"

"unawareness"

"unawares"

"unawed"

"unbacked"

"unbailable"

"unbaked"

"unbalance"

"unbalanced"

"unbalancing"

"unbaptized"

"unbar"

"unbarred"

"unbarring"

"unbars"

"unbear"

"unbearable"

"unbearably"

"unbearing"

"unbeatable"

"unbeaten"

"unbecoming"

"unbecomingly"

"unbefitting"

"unbeholden"

"unbeknown"

"unbeknownst"

"unbelief"

"unbeliefs"

"unbelievable"

"unbelievably"

"unbeliever"

"unbelievers"

"unbelieving"

"unbeloved"

"unbend"

"unbendable"

"unbended"

"unbending"

"unbends"

"unbent"

"unbiased"

"unbiasedly"

"unbid"

"unbidden"

"unbigoted"

"unbind"

"unbinding"

"unbinds"

"unbleached"

"unblemished"

"unblessed"

"unblessedness"

"unblinking"

"unblock"

"unblocked"

"unblocking"

"unblocks"

"unblushing"

"unblushingly"

"unbodied"

"unbolt"

"unbolted"

"unbolting"

"unbolts"

"unborn"

"unborn baby"

"unbosom"

"unbosomed"

"unbosoming"

"unbosoms"

"unbound"

"unbounded"

"unboundedly"

"unbowed"

"unbox"

"unbraiding"

"unbranded"

"unbreakable"

"unbred"

"unbribable"

"unbridgeable"

"unbridged"

"unbridle"

"unbridled"

"unbridledly"

"unbridles"

"unbroken"

"unbrotherly"

"unbruised"

"unbrushed"

"unbuckle"

"unbuckled"

"unbuckles"

"unbuckling"

"unbudgeted"

"unbudging"

"unbuilding"

"unburden"

"unburdened"

"unburdening"

"unburdens"

"unburied"

"unburned"

"unburnt"

"unbutton"

"unbuttoned"

"unbuttoning"

"unbuttons"

"uncage"

"uncanceled"

"uncancelled"

"uncannier"

"uncanniest"

"uncannily"

"uncanny"

"uncap"

"uncapitalized"

"uncapped"

"uncapping"

"uncaps"

"uncaring"

"uncarpeted"

"uncase"

"uncashed"

"uncaught"

"unceasing"

"unceasingly"

"uncelebrated"

"uncensored"

"uncensured"

"unceremonious"

"unceremoniously"

"unceremoniousness"

"uncertain"

"uncertainly"

"uncertainties"

"uncertainty"

"uncertified"

"unchain"

"unchained"

"unchaining"

"unchains"

"unchallengeable"

"unchallenged"

"unchangeable"

"unchanged"

"unchanging"

"unchaperoned"

"uncharacteristic"

"uncharged"

"uncharges"

"uncharging"

"uncharitable"

"uncharitableness"

"uncharitably"

"uncharted"

"unchaste"

"unchastely"

"unchastened"

"unchasteness"

"unchastised"

"unchastities"

"unchastity"

"unchecked"

"uncheerful"

"uncheerfully"

"uncherished"

"unchilled"

"unchivalrous"

"unchosen"

"unchristened"

"unchristian"

"unchurched"

"uncial"

"uncials"

"unciforms"

"uncircumcised"

"uncircumstantial"

"uncircumstantialy"

"uncivil"

"uncivilized"

"uncivilly"

"unclad"

"unclaimed"

"unclamped"

"unclamps"

"unclarified"

"unclasp"

"unclasped"

"unclasping"

"unclasps"

"unclassifiable"

"unclassified"

"uncle"

"unclean"

"uncleaned"

"uncleanliness"

"uncleanly"

"uncleanness"

"unclear"

"uncleared"

"unclearer"

"unclehood"

"unclench"

"unclenched"

"unclenches"

"unclenching"

"unclerical"

"uncles"

"uncloak"

"uncloaked"

"uncloaking"

"uncloaks"

"unclog"

"unclogged"

"unclogging"

"unclogs"

"unclose"

"unclosed"

"uncloses"

"unclosing"

"unclothe"

"unclothed"

"unclothes"

"unclothing"

"unclouded"

"unclouding"

"uncluttered"

"unco"

"uncoagulated"

"uncoated"

"uncoffined"

"uncoil"

"uncoiled"

"uncoiling"

"uncoils"

"uncollected"

"uncolored"

"uncombed"

"uncombined"

"uncomfortable"

"uncomfortableness"

"uncomfortably"

"uncomforted"

"uncomforting"

"uncommendable"

"uncommercial"

"uncommitted"

"uncommon"

"uncommoner"

"uncommonly"

"uncommonness"

"uncommunicative"

"uncompartmentalize"

"uncompartmentalized"

"uncompartmentalizes"

"uncompassionate"

"uncompensated"

"uncompetitive"

"uncomplaining"

"uncomplainingly"

"uncompleted"

"uncompliant"

"uncomplicated"

"uncomplimentary"

"uncompounded"

"uncomprehended"

"uncomprehending"

"uncomprehendingly"

"uncomprehened"

"uncompressed"

"uncompromising"

"uncompromisingly"

"unconcealed"

"unconceded"

"unconcern"

"unconcerned"

"unconcernedly"

"unconcluded"

"uncondensed"

"unconditional"

"unconditionality"

"unconditionally"

"unconditioned"

"uncondoned"

"unconfessed"

"unconfined"

"unconfirmed"

"unconformable"

"unconforming"

"unconfused"

"uncongenial"

"unconnected"

"unconquerable"

"unconquerably"

"unconquered"

"unconscientious"

"unconscientiously"

"unconscionable"

"unconscionably"

"unconscious"

"unconsciously"

"unconsciousness"

"unconsecrated"

"unconsenting"

"unconsidered"

"unconsoled"

"unconsolidated"

"unconstitutional"

"unconstitutionality"

"unconstitutionally"

"unconstrained"

"unconstricted"

"unconsumed"

"unconsummated"

"uncontaminated"

"uncontestable"

"uncontested"

"uncontradicted"

"uncontrite"

"uncontrollable"

"uncontrollably"

"uncontrolled"

"uncontrovertible"

"unconventional"

"unconventionality"

"unconventionalized"

"unconventionalizes"

"unconventionally"

"unconversant"

"unconverted"

"unconvertible"

"unconvinced"

"unconvincing"

"unconvincingly"

"uncooked"

"uncool"

"uncooperative"

"uncoordinated"

"uncordial"

"uncork"

"uncorked"

"uncorking"

"uncorks"

"uncorrected"

"uncorroborated"

"uncorrupted"

"uncos"

"uncountable"

"uncounted"

"uncouple"

"uncoupled"

"uncouples"

"uncoupling"

"uncourageous"

"uncourteous"

"uncouth"

"uncouthness"

"uncover"

"uncovered"

"uncovering"

"uncovers"

"uncrate"

"uncrated"

"uncrates"

"uncrating"

"uncreated"

"uncreates"

"uncreating"

"uncritical"

"uncritically"

"uncross"

"uncrossed"

"uncrosses"

"uncrossing"

"uncrowded"

"uncrowned"

"uncrowning"

"uncrystallized"

"unction"

"unctions"

"unctuosity"

"unctuous"

"unctuously"

"unctuousness"

"uncultivated"

"uncultured"

"uncurbed"

"uncured"

"uncurious"

"uncurl"

"uncurled"

"uncurling"

"uncurls"

"uncurtained"

"uncustomary"

"uncut"

"undamaged"

"undamped"

"undated"

"undaunted"

"undauntedly"

"undebatable"

"undecayed"

"undeceive"

"undeceived"

"undeceives"

"undeceiving"

"undecidable"

"undecided"

"undecipherable"

"undeclared"

"undecorated"

"undefeated"

"undefended"

"undefensible"

"undefiled"

"undefinable"

"undefinably"

"undefined"

"undeliverable"

"undelivered"

"undemanding"

"undemocratic"

"undemocratically"

"undemonstrable"

"undemonstrably"

"undemonstrative"

"undemonstratively"

"undemonstrativeness"

"undeniable"

"undeniably"

"undenied"

"undenominational"

"undependable"

"under"

"underachieve"

"underachieved"

"underachiever"

"underachievers"

"underachieves"

"underachieving"

"underact"

"underacted"

"underacting"

"underacts"

"underage"

"underages"

"underarm"

"underarms"

"underassessed"

"underassessment"

"underate"

"underbellies"

"underbelly"

"underbid"

"underbidder"

"underbidders"

"underbidding"

"underbids"

"underbred"

"underbrush"

"undercapitalize"

"undercapitalized"

"undercarriage"

"undercarriages"

"undercharge"

"undercharged"

"undercharges"

"undercharging"

"underclad"

"underclassman"

"underclassmen"

"underclerk"

"underclerks"

"underclothed"

"underclothes"

"underclothing"

"undercoat"

"undercoated"

"undercoating"

"undercoatings"

"undercoats"

"undercook"

"undercooked"

"undercooking"

"undercooks"

"undercover"

"undercurrent"

"undercurrents"

"undercut"

"undercuts"

"undercutting"

"underdeveloped"

"underdevelopment"

"underdoes"

"underdog"

"underdogs"

"underdone"

"underdrawers"

"underdress"

"underdressed"

"underdresses"

"underdressing"

"undereat"

"undereducated"

"underemphasize"

"underemphasized"

"underemphasizes"

"underemphasizing"

"underemployed"

"underemployment"

"underestimate"

"underestimated"

"underestimates"

"underestimating"

"underestimation"

"underestimations"

"underexpose"

"underexposed"

"underexposes"

"underexposing"

"underexposure"

"underexposures"

"underfed"

"underfeed"

"underfeeding"

"underfeeds"

"underfinance"

"underfinanced"

"underfinances"

"underfinancing"

"underflow"

"underfoot"

"underfur"

"undergarment"

"undergarments"

"undergird"

"undergirded"

"undergirding"

"undergirds"

"undergo"

"undergoes"

"undergoing"

"undergone"

"undergraduate"

"undergraduates"

"underground"

"undergrounder"

"undergrounds"

"undergrowth"

"underhand"

"underhanded"

"underhandedly"

"underhandedness"

"underlaid"

"underlain"

"underlaps"

"underlay"

"underlayer"

"underlayers"

"underlays"

"underlie"

"underlier"

"underlies"

"underline"

"underlined"

"underlines"

"underling"

"underlings"

"underlining"

"underlip"

"underlips"

"underlying"

"undermanned"

"undermentioned"

"undermine"

"undermined"

"underminer"

"undermines"

"undermining"

"undermost"

"underneath"

"undernourished"

"undernourishment"

"underofficial"

"underofficials"

"underpaid"

"underpants"

"underpart"

"underparts"

"underpass"

"underpasses"

"underpay"

"underpaying"

"underpayment"

"underpays"

"underpeopled"

"underpin"

"underpinned"

"underpinning"

"underpinnings"

"underpins"

"underplay"

"underplayed"

"underplaying"

"underplays"

"underpopulated"

"underpowered"

"underprice"

"underpriced"

"underprices"

"underpricing"

"underprivileged"

"underproduce"

"underproduced"

"underproduces"

"underproducing"

"underproduction"

"underran"

"underrate"

"underrated"

"underrates"

"underrating"

"underripened"

"underrun"

"underrunning"

"underruns"

"underscore"

"underscored"

"underscores"

"underscoring"

"undersea"

"underseas"

"undersecretary"

"undersell"

"underselling"

"undersells"

"underset"

"undersexed"

"undersheriff"

"undershirt"

"undershirts"

"undershorts"

"undershot"

"underside"

"undersides"

"undersign"

"undersigned"

"undersize"

"undersized"

"underskirt"

"underskirts"

"underslung"

"undersold"

"underspend"

"underspending"

"underspends"

"underspent"

"understaffed"

"understand"

"understandable"

"understandably"

"understanding"

"understandingly"

"understandings"

"understands"

"understate"

"understated"

"understatement"

"understatements"

"understates"

"understating"

"understood"

"understructure"

"understructures"

"understudied"

"understudies"

"understudy"

"understudying"

"undersupplied"

"undersupplies"

"undersupply"

"undersupplying"

"undersurface"

"undertake"

"undertaken"

"undertaker"

"undertakers"

"undertakes"

"undertaking"

"undertakings"

"underthings"

"undertone"

"undertones"

"undertook"

"undertow"

"undertows"

"undertrained"

"undervalue"

"undervalued"

"undervalues"

"undervaluing"

"underwaist"

"underwaists"

"underwater"

"underway"

"underwear"

"underweight"

"underwent"

"underwind"

"underwinding"

"underwinds"

"underworld"

"underwound"

"underwrite"

"underwriter"

"underwriters"

"underwrites"

"underwriting"

"underwritten"

"underwrote"

"undescribable"

"undescribably"

"undeserved"

"undeserving"

"undesigned"

"undesigning"

"undesirability"

"undesirable"

"undesired"

"undestroyed"

"undetachable"

"undetached"

"undetectable"

"undetected"

"undeterminable"

"undetermined"

"undeterred"

"undeveloped"

"undeviating"

"undeviatingly"

"undiagnosed"

"undid"

"undies"

"undifferentiated"

"undiffused"

"undigested"

"undignified"

"undiluted"

"undiminished"

"undimmed"

"undine"

"undines"

"undiplomatic"

"undirected"

"undiscerned"

"undiscernible"

"undiscernibly"

"undiscerning"

"undischarged"

"undisciplinable"

"undisciplined"

"undisclosed"

"undiscouraged"

"undiscoverable"

"undiscovered"

"undiscriminating"

"undiscriminatingly"

"undisguised"

"undismayed"

"undispelled"

"undisplayed"

"undisposed"

"undisproved"

"undisputable"

"undisputed"

"undissolved"

"undistilled"

"undistinguishable"

"undistinguished"

"undistinguishing"

"undistressed"

"undistributed"

"undisturbed"

"undiversified"

"undivided"

"undivulged"

"undo"

"undocking"

"undocks"

"undocumented"

"undoer"

"undoers"

"undoes"

"undogmatic"

"undoing"

"undoings"

"undomesticated"

"undone"

"undoubted"

"undoubtedly"

"undoubting"

"undramatic"

"undrape"

"undraped"

"undrapes"

"undraping"

"undreamed"

"undreamt"

"undress"

"undressed"

"undresses"

"undressing"

"undrest"

"undrinkable"

"undue"

"undulance"

"undulant"

"undulate"

"undulated"

"undulates"

"undulating"

"undulation"

"undulations"

"undulatory"

"unduly"

"undutiful"

"undutifully"

"undy"

"undyed"

"undying"

"undyingly"

"unearned"

"unearth"

"unearthed"

"unearthing"

"unearthly"

"unearths"

"unease"

"uneasier"

"uneasiest"

"uneasily"

"uneasiness"

"uneasy"

"uneatable"

"uneated"

"uneaten"

"uneconomic"

"uneconomical"

"uneconomically"

"unedible"

"unedifying"

"unedited"

"uneducable"

"uneducated"

"unemancipated"

"unembarrassed"

"unembellished"

"unemotional"

"unemotionally"

"unemphatic"

"unemployability"

"unemployable"

"unemployed"

"unemployment"

"unenclosed"

"unencumbered"

"unendangered"

"unended"

"unending"

"unendingly"

"unendorsed"

"unendurable"

"unendurably"

"unenforceable"

"unenforced"

"unenfranchised"

"unengaged"

"unenjoyable"

"unenlightened"

"unenriched"

"unenrolled"

"unentangled"

"unentered"

"unenterprising"

"unentertaining"

"unenthusiastic"

"unenthusiastically"

"unenviable"

"unenvious"

"unenviously"

"unequal"

"unequaled"

"unequalled"

"unequally"

"unequals"

"unequipped"

"unequivocal"

"unequivocally"

"unequivocalness"

"unerased"

"unerring"

"unerringly"

"unescapable"

"unescapably"

"unesco"

"unescorted"

"unessential"

"unestablished"

"unesthetic"

"unethical"

"unethically"

"uneven"

"unevener"

"unevenest"

"unevenly"

"unevenness"

"uneventful"

"uneventfully"

"unexaggerated"

"unexampled"

"unexcavated"

"unexcelled"

"unexceptionable"

"unexceptionably"

"unexceptional"

"unexchangeable"

"unexcited"

"unexciting"

"unexcusable"

"unexcusably"

"unexcused"

"unexecuted"

"unexercised"

"unexpected"

"unexpectedly"

"unexpectedness"

"unexpended"

"unexperienced"

"unexpired"

"unexplainable"

"unexplainably"

"unexplained"

"unexplicit"

"unexploded"

"unexploited"

"unexplored"

"unexposed"

"unexpressed"

"unexpressive"

"unexpurgated"

"unextended"

"unextinguished"

"unextravagant"

"unfaded"

"unfading"

"unfailing"

"unfailingly"

"unfailingness"

"unfair"

"unfairer"

"unfairest"

"unfairly"

"unfairness"

"unfaithful"

"unfaithfully"

"unfaithfulness"

"unfaltering"

"unfalteringly"

"unfamiliar"

"unfamiliarity"

"unfamiliarly"

"unfashionable"

"unfashionably"

"unfasten"

"unfastened"

"unfastening"

"unfastens"

"unfathomable"

"unfathomed"

"unfavorable"

"unfavorably"

"unfavored"

"unfazed"

"unfeared"

"unfearing"

"unfeasible"

"unfed"

"unfederated"

"unfeeling"

"unfeelingly"

"unfeigned"

"unfelt"

"unfeminine"

"unfenced"

"unfences"

"unfermented"

"unfertile"

"unfertilized"

"unfestive"

"unfetter"

"unfettered"

"unfetters"

"unfilial"

"unfilled"

"unfiltered"

"unfinished"

"unfit"

"unfitly"

"unfitness"

"unfits"

"unfitted"

"unfitting"

"unfittingly"

"unfix"

"unfixed"

"unfixes"

"unfixing"

"unflagging"

"unflaggingly"

"unflappability"

"unflappable"

"unflappably"

"unflattering"

"unflavored"

"unfledged"

"unflinching"

"unflinchingly"

"unfocused"

"unfocussed"

"unfold"

"unfolded"

"unfolder"

"unfolders"

"unfolding"

"unfolds"

"unforbidded"

"unforbidden"

"unforbidding"

"unforced"

"unforeseeable"

"unforeseen"

"unforested"

"unforetold"

"unforgettable"

"unforgettably"

"unforgivable"

"unforgivably"

"unforgiven"

"unforgiving"

"unforgotten"

"unformatted"

"unformed"

"unformulated"

"unforsaken"

"unforseen"

"unfortified"

"unfortunate"

"unfortunately"

"unfortunateness"

"unfortunates"

"unfought"

"unfounded"

"unframed"

"unfree"

"unfreeze"

"unfreezes"

"unfreezing"

"unfrequented"

"unfriendliness"

"unfriendly"

"unfrock"

"unfrocked"

"unfrocking"

"unfrocks"

"unfroze"

"unfrozen"

"unfruitful"

"unfulfilled"

"unfunny"

"unfurl"

"unfurled"

"unfurling"

"unfurls"

"unfurnished"

"ungainlier"

"ungainliness"

"ungainly"

"ungallant"

"ungallantly"

"ungathered"

"ungenerous"

"ungenial"

"ungenially"

"ungenteel"

"ungentle"

"ungentlemanly"

"ungently"

"unglazed"

"unglue"

"ungodlier"

"ungodliness"

"ungodly"

"ungot"

"ungovernability"

"ungovernable"

"ungoverned"

"ungraceful"

"ungracefully"

"ungracious"

"ungraciously"

"ungraciousness"

"ungraded"

"ungrammatical"

"ungrammatically"

"ungrateful"

"ungratefully"

"ungratefulness"

"ungratifying"

"ungrounded"

"ungrudging"

"ungrudgingly"

"unguarded"

"unguent"

"unguentary"

"unguents"

"unguided"

"unguiltily"

"ungulate"

"ungulates"

"unhabituated"

"unhackneyed"

"unhallowed"

"unhampered"

"unhand"

"unhanded"

"unhandicapped"

"unhandier"

"unhandiest"

"unhanding"

"unhands"

"unhandy"

"unhanged"

"unhappier"

"unhappiest"

"unhappily"

"unhappiness"

"unhappy"

"unhardened"

"unharmed"

"unharmful"

"unharmonious"

"unharness"

"unharnessed"

"unharnesses"

"unharnessing"

"unharvested"

"unhat"

"unhatched"

"unhats"

"unhatted"

"unhealed"

"unhealthful"

"unhealthier"

"unhealthiest"

"unhealthiness"

"unhealthy"

"unhealthy eating"

"unhealthy eating patterns"

"unheard"

"unheated"

"unheeded"

"unheedful"

"unheedfully"

"unheeding"

"unhelm"

"unhelpful"

"unheralded"

"unheroic"

"unhesitating"

"unhesitatingly"

"unhindered"

"unhinge"

"unhinged"

"unhinges"

"unhinging"

"unhip"

"unhitch"

"unhitched"

"unhitches"

"unhitching"

"unholier"

"unholiest"

"unholily"

"unholiness"

"unholy"

"unhonored"

"unhooded"

"unhook"

"unhooked"

"unhooking"

"unhooks"

"unhorse"

"unhorsed"

"unhorses"

"unhorsing"

"unhoused"

"unhuman"

"unhung"

"unhurried"

"unhurriedly"

"unhurt"

"unhygienic"

"unhyphenated"

"uniaxial"

"unicameral"

"unicamerally"

"unicef"

"unicellular"

"unicolor"

"unicorn"

"unicorns"

"unicycle"

"unicycles"

"unicyclist"

"unidentifiable"

"unidentified"

"unidiomatic"

"unidiomatically"

"unidirectional"

"unific"

"unification"

"unified"

"unifier"

"unifiers"

"unifies"

"uniform"

"uniformed"

"uniformer"

"uniformest"

"uniforming"

"uniformities"

"uniformity"

"uniformly"

"uniformness"

"uniforms"

"unify"

"unifying"

"unilateral"

"unilaterally"

"unilluminated"

"unillustrated"

"unimaginable"

"unimaginably"

"unimaginative"

"unimaginatively"

"unimpaired"

"unimpassioned"

"unimpeachability"

"unimpeachable"

"unimpeachably"

"unimpeached"

"unimpeded"

"unimportance"

"unimportant"

"unimposing"

"unimpressed"

"unimpressible"

"unimpressive"

"unimpressively"

"unimproved"

"uninclosed"

"unincorporated"

"unincumbered"

"unindemnified"

"unindorsed"

"uninfected"

"uninflammable"

"uninfluenced"

"uninfluential"

"uninformative"

"uninformed"

"uninhabitable"

"uninhabited"

"uninhibited"

"uninhibitedly"

"uninitiated"

"uninjured"

"uninspired"

"uninspiring"

"uninspiringly"

"uninstructed"

"uninsurable"

"uninsured"

"uninsured/underinsured customers"

"unintellectual"

"unintelligent"

"unintelligently"

"unintelligible"

"unintelligibly"

"unintended"

"unintendedly"

"unintentional"

"unintentionally"

"uninterested"

"uninterestedly"

"uninteresting"

"uninterestingly"

"uninterrupted"

"uninterruptedly"

"unintoxicated"

"uninvested"

"uninvited"

"uninviting"

"uninvitingly"

"uninvolved"

"union"

"unionism"

"unionisms"

"unionist"

"unionistic"

"unionists"

"unionization"

"unionize"

"unionized"

"unionizes"

"unionizing"

"unions"

"unipod"

"unipolar"

"unique"

"uniquely"

"uniqueness"

"uniquer"

"uniques"

"uniquest"

"unironed"

"unisex"

"unisexes"

"unisexual"

"unison"

"unisonal"

"unisons"

"unit"

"unitarian"

"unitarianism"

"unitarians"

"unitary"

"unite"

"united"

"unitedly"

"uniter"

"uniters"

"unites"

"unities"

"uniting"

"unitive"

"unitize"

"unitized"

"unitizes"

"unitizing"

"units"

"unity"

"univ"

"univalent"

"univalve"

"univalves"

"universal"

"universalism"

"universalist"

"universalists"

"universality"

"universalization"

"universalize"

"universalized"

"universalizes"

"universalizing"

"universally"

"universals"

"universe"

"universes"

"universities"

"university"

"univocal"

"univocals"

"unix"

"unjoined"

"unjointed"

"unjudicial"

"unjudicially"

"unjust"

"unjustifiable"

"unjustifiably"

"unjustification"

"unjustified"

"unjustly"

"unjustness"

"unkempt"

"unkennel"

"unkenneled"

"unkennels"

"unkept"

"unkind"

"unkinder"

"unkindest"

"unkindlier"

"unkindly"

"unkindness"

"unkingly"

"unkissed"

"unknits"

"unknitting"

"unknot"

"unknots"

"unknotted"

"unknotting"

"unknowable"

"unknowing"

"unknowingly"

"unknown"

"unknowns"

"unkosher"

"unlabeled"

"unlabelled"

"unlabored"

"unlaboured"

"unlace"

"unlaced"

"unlaces"

"unlacing"

"unlading"

"unlamented"

"unlanded"

"unlashing"

"unlatch"

"unlatched"

"unlatches"

"unlatching"

"unlaw"

"unlawful"

"unlawfully"

"unlawfulness"

"unlay"

"unlaying"

"unleaded"

"unlearn"

"unlearned"

"unlearning"

"unlearns"

"unlearnt"

"unleash"

"unleashed"

"unleashes"

"unleashing"

"unleavened"

"unled"

"unless"

"unlet"

"unlettable"

"unlettered"

"unleveling"

"unlevelled"

"unlicensed"

"unlifelike"

"unlighted"

"unlikable"

"unlike"

"unlikelier"

"unlikeliest"

"unlikelihood"

"unlikeliness"

"unlikely"

"unlikeness"

"unlimber"

"unlimbered"

"unlimbering"

"unlimbers"

"unlimited"

"unlimited scalability"

"unlimitedness"

"unlined"

"unlink"

"unlinked"

"unlinking"

"unlinks"

"unliquidated"

"unlisted"

"unlit"

"unlivable"

"unliveable"

"unliveries"

"unload"

"unloaded"

"unloader"

"unloaders"

"unloading"

"unloads"

"unlocated"

"unlock"

"unlocked"

"unlocking"

"unlocks"

"unlooked"

"unloose"

"unloosed"

"unloosen"

"unloosened"

"unloosening"

"unloosens"

"unlooses"

"unloosing"

"unlovable"

"unloved"

"unlovelier"

"unlovely"

"unloving"

"unlubricated"

"unluckier"

"unluckiest"

"unluckily"

"unlucky"

"unmade"

"unmagnified"

"unmailable"

"unmaintainable"

"unmake"

"unman"

"unmanageable"

"unmanageably"

"unmanful"

"unmanliness"

"unmanly"

"unmanned"

"unmannerliness"

"unmannerly"

"unmanning"

"unmans"

"unmanufactured"

"unmapped"

"unmarked"

"unmarketable"

"unmarred"

"unmarriageable"

"unmarried"

"unmarrying"

"unmask"

"unmasked"

"unmasker"

"unmaskers"

"unmasking"

"unmasks"

"unmastered"

"unmatched"

"unmeaning"

"unmeant"

"unmeasured"

"unmechanical"

"unmedicated"

"unmelodious"

"unmelted"

"unmemorized"

"unmended"

"unmentionable"

"unmentionables"

"unmentioned"

"unmerchantable"

"unmerciful"

"unmercifully"

"unmerged"

"unmerited"

"unmet"

"unmethodical"

"unmilitary"

"unmindful"

"unmingled"

"unmingling"

"unmistakable"

"unmistakably"

"unmistaken"

"unmitering"

"unmitigated"

"unmitigatedly"

"unmixed"

"unmixt"

"unmodified"

"unmold"

"unmolded"

"unmolested"

"unmollified"

"unmoored"

"unmooring"

"unmoral"

"unmorality"

"unmortgaged"

"unmotivated"

"unmounted"

"unmourned"

"unmovable"

"unmoved"

"unmoving"

"unmown"

"unmuffle"

"unmuffled"

"unmuffles"

"unmuffling"

"unmusical"

"unmuzzle"

"unmuzzled"

"unmuzzles"

"unmuzzling"

"unnameable"

"unnamed"

"unnatural"

"unnaturally"

"unnaturalness"

"unnavigable"

"unnecessarily"

"unnecessariness"

"unnecessary"

"unneeded"

"unneedful"

"unneedfully"

"unnegotiable"

"unneighborly"

"unnerve"

"unnerved"

"unnerves"

"unnerving"

"unnoted"

"unnoticeable"

"unnoticeably"

"unnoticed"

"unnourished"

"unnumbered"

"unobjectionable"

"unobjectionably"

"unobliged"

"unobliging"

"unobnoxious"

"unobscured"

"unobservant"

"unobserved"

"unobserving"

"unobstructed"

"unobtainable"

"unobtruding"

"unobtrusive"

"unobtrusively"

"unobtrusiveness"

"unoccupied"

"unoffended"

"unoffending"

"unoffensive"

"unoffensively"

"unoffered"

"unofficial"

"unofficially"

"unofficiously"

"unopened"

"unopposed"

"unoppressed"

"unordained"

"unorganized"

"unoriginal"

"unornamented"

"unorthodox"

"unorthodoxly"

"unostentatious"

"unostentatiously"

"unowned"

"unpacified"

"unpack"

"unpacked"

"unpacker"

"unpackers"

"unpacking"

"unpacks"

"unpaid"

"unpainted"

"unpaired"

"unpalatable"

"unpalatably"

"unparalleled"

"unpardonable"

"unpardonably"

"unpardoned"

"unpasteurized"

"unpatentable"

"unpatented"

"unpatriotic"

"unpatriotically"

"unpaved"

"unpaying"

"unpedigreed"

"unpeg"

"unpen"

"unpenetrated"

"unpenned"

"unpens"

"unpent"

"unpeople"

"unpeopled"

"unpeoples"

"unpeopling"

"unperceived"

"unperceiving"

"unperceptive"

"unperceptively"

"unperfected"

"unperformed"

"unperjured"

"unperson"

"unpersons"

"unpersuaded"

"unpersuasive"

"unpersuasively"

"unperturbable"

"unperturbably"

"unperturbed"

"unphotographic"

"unpicked"

"unpile"

"unpiled"

"unpiles"

"unpiling"

"unpin"

"unpinned"

"unpinning"

"unpins"

"unpited"

"unpitied"

"unpitying"

"unpityingly"

"unplaced"

"unplaiting"

"unplanned"

"unplanted"

"unplayable"

"unplayed"

"unpleasant"

"unpleasantly"

"unpleasantness"

"unpleased"

"unpleasing"

"unpledged"

"unplowed"

"unplug"

"unplugged"

"unplugging"

"unplugs"

"unplumbed"

"unpoetic"

"unpoetical"

"unpoetically"

"unpointed"

"unpoised"

"unpolarized"

"unpolished"

"unpolitic"

"unpolitical"

"unpolled"

"unpolluted"

"unpopular"

"unpopularity"

"unpopularly"

"unpopulated"

"unposed"

"unpossessive"

"unpossessively"

"unpracticable"

"unpractical"

"unpracticed"

"unprecedented"

"unpredictability"

"unpredictabilness"

"unpredictable"

"unpredictably"

"unpredicted"

"unprejudiced"

"unpremeditated"

"unprepared"

"unpreparedness"

"unprepossessing"

"unprescribed"

"unpresentable"

"unpresentably"

"unpreserved"

"unpressed"

"unpresumptuous"

"unpretending"

"unpretentious"

"unpretentiously"

"unpretentiousness"

"unpreventable"

"unpriced"

"unprimed"

"unprincipled"

"unprintable"

"unprized"

"unprocessed"

"unproclaimed"

"unprocurable"

"unproductive"

"unproductively"

"unproductiveness"

"unprofessed"

"unprofessional"

"unprofessionally"

"unprofitable"

"unprofitably"

"unprogressive"

"unprogressively"

"unprohibited"

"unprolific"

"unpromising"

"unpromisingly"

"unprompted"

"unpronounceable"

"unpronounced"

"unpropitious"

"unpropitiously"

"unproportionate"

"unproportionately"

"unproposed"

"unprotected"

"unprotesting"

"unprotestingly"

"unprovable"

"unproved"

"unproven"

"unprovided"

"unprovoked"

"unpublished"

"unpuckered"

"unpunctual"

"unpunished"

"unpurified"

"unpuzzling"

"unqualified"

"unqualifiedly"

"unquenchable"

"unquenched"

"unquestionable"

"unquestionably"

"unquestioned"

"unquestioning"

"unquestioningly"

"unquiet"

"unquieter"

"unquietest"

"unquiets"

"unquotable"

"unquote"

"unquoted"

"unquotes"

"unraised"

"unrated"

"unravel"

"unraveled"

"unraveling"

"unravelled"

"unravelling"

"unravels"

"unread"

"unreadable"

"unreadier"

"unreadiest"

"unready"

"unreal"

"unrealistic"

"unrealistically"

"unreality"

"unrealized"

"unreally"

"unreason"

"unreasonable"

"unreasonableness"

"unreasonably"

"unreasoned"

"unreasoning"

"unrebuked"

"unreceptive"

"unreceptively"

"unreckoned"

"unreclaimed"

"unrecognizable"

"unrecognizably"

"unrecognized"

"unrecommended"

"unrecompensed"

"unreconcilable"

"unreconcilably"

"unreconciled"

"unreconstructed"

"unrecorded"

"unrecoverable"

"unrectified"

"unredeemed"

"unreel"

"unreeled"

"unreeler"

"unreelers"

"unreeling"

"unreels"

"unrefined"

"unreflecting"

"unreflectingly"

"unreflective"

"unreformed"

"unrefreshed"

"unregenerate"

"unregimented"

"unregistered"

"unregulated"

"unrehearsed"

"unrelated"

"unrelenting"

"unrelentingly"

"unreliable"

"unreliably"

"unrelieved"

"unrelinquished"

"unremembered"

"unremitted"

"unremitting"

"unremittingly"

"unremorseful"

"unremorsefully"

"unremovable"

"unremoved"

"unremunerated"

"unremunerative"

"unrenewed"

"unrentable"

"unrented"

"unrepaid"

"unrepealed"

"unrepentant"

"unrepenting"

"unrepentingly"

"unreplaceable"

"unreplaced"

"unreported"

"unrepresentative"

"unrepresented"

"unrepressed"

"unreprieved"

"unreprimanded"

"unreproved"

"unrequitable"

"unrequited"

"unresentful"

"unresentfully"

"unreserved"

"unreservedly"

"unresigned"

"unresistant"

"unresisting"

"unresolved"

"unrespectful"

"unrespectfully"

"unresponsive"

"unresponsively"

"unresponsiveness"

"unrest"

"unrested"

"unrestored"

"unrestrained"

"unrestrainedly"

"unrestricted"

"unrestrictedly"

"unrests"

"unretracted"

"unreturned"

"unrevealed"

"unrevenged"

"unrevised"

"unrevoked"

"unrewarded"

"unrewarding"

"unrhymed"

"unrhythmic"

"unriddle"

"unriddling"

"unrig"

"unrighteous"

"unrighteously"

"unrighteousness"

"unrightful"

"unrip"

"unripe"

"unripely"

"unripened"

"unriper"

"unripest"

"unrisen"

"unrivaled"

"unrivalled"

"unrobe"

"unrobed"

"unrobes"

"unrobing"

"unroll"

"unrolled"

"unrolling"

"unrolls"

"unromantic"

"unromantically"

"unroof"

"unroofed"

"unroofing"

"unroofs"

"unrounding"

"unruffled"

"unrule"

"unruled"

"unrulier"

"unruliest"

"unruliness"

"unruly"

"unsaddle"

"unsaddled"

"unsaddles"

"unsaddling"

"unsafe"

"unsafely"

"unsafeties"

"unsafety"

"unsaid"

"unsalability"

"unsalable"

"unsalaried"

"unsalted"

"unsanctified"

"unsanctioned"

"unsanitary"

"unsatiable"

"unsatiably"

"unsatisfactorily"

"unsatisfactory"

"unsatisfiable"

"unsatisfied"

"unsatisfying"

"unsaturate"

"unsaturated"

"unsaturates"

"unsaved"

"unsavoriness"

"unsavory"

"unsay"

"unsays"

"unscaled"

"unscarred"

"unscathed"

"unscented"

"unscheduled"

"unscholarly"

"unschooled"

"unscientific"

"unscientifically"

"unscramble"

"unscrambled"

"unscrambles"

"unscrambling"

"unscratched"

"unscreened"

"unscrew"

"unscrewed"

"unscrewing"

"unscrews"

"unscriptural"

"unscrupulous"

"unscrupulously"

"unscrupulousness"

"unseal"

"unsealed"

"unsealing"

"unseals"

"unseaming"

"unseams"

"unseasonable"

"unseasonably"

"unseasoned"

"unseat"

"unseated"

"unseating"

"unseats"

"unseaworthiness"

"unseaworthy"

"unsecluded"

"unsecured"

"unseduced"

"unseeing"

"unseeingly"

"unseemlier"

"unseemly"

"unseen"

"unsegmented"

"unsegregated"

"unselective"

"unselfconscious"

"unselfish"

"unselfishly"

"unselfishness"

"unsensible"

"unsensitive"

"unsent"

"unsentimental"

"unsentimentally"

"unserved"

"unserviceable"

"unserviceably"

"unset"

"unsettle"

"unsettled"

"unsettlement"

"unsettles"

"unsettling"

"unsew"

"unsex"

"unsexing"

"unsexual"

"unshackle"

"unshackled"

"unshackles"

"unshackling"

"unshaded"

"unshakable"

"unshakably"

"unshaken"

"unshamed"

"unshapely"

"unshared"

"unshaved"

"unshaven"

"unsheathe"

"unsheathed"

"unsheathes"

"unsheathing"

"unshed"

"unshelled"

"unshelling"

"unsheltered"

"unshielded"

"unshifting"

"unship"

"unshipped"

"unshipping"

"unships"

"unshod"

"unshorn"

"unshrinkable"

"unshut"

"unsifted"

"unsighted"

"unsighting"

"unsightliness"

"unsightly"

"unsigned"

"unsilenced"

"unsinful"

"unsinkable"

"unskilled"

"unskillful"

"unskillfully"

"unskillfulness"

"unslaked"

"unsling"

"unslinging"

"unslings"

"unslung"

"unsmiling"

"unsmilingly"

"unsnap"

"unsnapped"

"unsnapping"

"unsnaps"

"unsnarl"

"unsnarled"

"unsnarling"

"unsnarls"

"unsociable"

"unsociably"

"unsocial"

"unsocially"

"unsoiled"

"unsold"

"unsolder"

"unsoldered"

"unsolders"

"unsolicited"

"unsolicitous"

"unsolvable"

"unsolved"

"unsoothed"

"unsophisticated"

"unsophisticatedly"

"unsorted"

"unsought"

"unsound"

"unsoundest"

"unsoundly"

"unsoundness"

"unsparing"

"unsparingly"

"unsparingness"

"unspeakable"

"unspeakably"

"unspeaking"

"unspecialized"

"unspecific"

"unspecifically"

"unspecified"

"unspectacular"

"unspent"

"unsphering"

"unspiritual"

"unspoiled"

"unspoilt"

"unspoken"

"unsportsmanlike"

"unspotted"

"unsprung"

"unstable"

"unstableness"

"unstabler"

"unstablest"

"unstably"

"unstack"

"unstacked"

"unstacking"

"unstacks"

"unstained"

"unstamped"

"unstandardized"

"unstapled"

"unstarched"

"unstated"

"unstates"

"unsteadier"

"unsteadies"

"unsteadiest"

"unsteadily"

"unsteadiness"

"unsteady"

"unsteeling"

"unstemmed"

"unstepping"

"unsterile"

"unsterilized"

"unsticking"

"unsticks"

"unstinted"

"unstirred"

"unstop"

"unstoppable"

"unstopped"

"unstopping"

"unstops"

"unstrained"

"unstrap"

"unstrapped"

"unstraps"

"unstressed"

"unstresses"

"unstring"

"unstructured"

"unstrung"

"unstuck"

"unstudied"

"unsubdued"

"unsubmissive"

"unsubstantial"

"unsubstantially"

"unsubstantiated"

"unsubtle"

"unsubtly"

"unsuccessful"

"unsuccessfully"

"unsuccessfulness"

"unsuggestive"

"unsuitability"

"unsuitable"

"unsuitableness"

"unsuitably"

"unsuited"

"unsullied"

"unsung"

"unsupervised"

"unsupported"

"unsupportedly"

"unsuppressed"

"unsuppressible"

"unsure"

"unsurely"

"unsureness"

"unsurmountable"

"unsurmountably"

"unsurpassable"

"unsurpassably"

"unsurpassed"

"unsurprised"

"unsurveyed"

"unsusceptible"

"unsusceptibly"

"unsuspected"

"unsuspecting"

"unsuspectingly"

"unsuspicious"

"unsuspiciously"

"unsustainable"

"unsustained"

"unswathe"

"unswathing"

"unswayed"

"unswearing"

"unsweetened"

"unswept"

"unswerving"

"unswervingly"

"unsymmetrical"

"unsymmetrically"

"unsympathetic"

"unsympathetically"

"unsystematic"

"unsystematical"

"unsystematically"

"untactful"

"untactfully"

"untainted"

"untalented"

"untamed"

"untangle"

"untangled"

"untangles"

"untangling"

"untanned"

"untapped"

"untarnished"

"untasted"

"untasteful"

"untastefully"

"untaught"

"untaxed"

"unteachable"

"unteaching"

"untempted"

"untempting"

"untenable"

"untenanted"

"untended"

"unterminated"

"unterrified"

"untested"

"untether"

"untethered"

"untethers"

"unthankful"

"unthawed"

"unthinkable"

"unthinkably"

"unthinking"

"unthought"

"unthoughtful"

"unthoughtfully"

"unthreaded"

"unthriftily"

"unthrifty"

"unthroning"

"untidied"

"untidier"

"untidies"

"untidiest"

"untidily"

"untidiness"

"untidy"

"untidying"

"untie"

"untied"

"unties"

"until"

"untillable"

"untilled"

"untimelier"

"untimeliness"

"untimely"

"untired"

"untiring"

"untiringly"

"untitled"

"unto"

"untold"

"untouchable"

"untouchably"

"untouched"

"untoward"

"untraceable"

"untraced"

"untractable"

"untrained"

"untrammeled"

"untrammelled"

"untranscendentally"

"untransferable"

"untransferred"

"untransformed"

"untranslatable"

"untranslated"

"untraveled"

"untravelled"

"untraversed"

"untreading"

"untreated"

"untried"

"untrimmed"

"untrimming"

"untrod"

"untrodden"

"untroubled"

"untrue"

"untruer"

"untruest"

"untruly"

"untrussing"

"untrustful"

"untrusting"

"untrustworthy"

"untrusty"

"untruth"

"untruthful"

"untruthfulness"

"untruths"

"untucked"

"unturned"

"untutored"

"untwist"

"untwisted"

"untwisting"

"untwists"

"untying"

"untypical"

"untypically"

"unum"

"unusable"

"unused"

"unusual"

"unusually"

"unusualness"

"unutilized"

"unutterable"

"unutterably"

"unuttered"

"unvaccinated"

"unvanquishable"

"unvanquished"

"unvaried"

"unvarnished"

"unvarying"

"unvaryingly"

"unveil"

"unveiled"

"unveiling"

"unveils"

"unvendible"

"unventilated"

"unventuresome"

"unverifiable"

"unverifiably"

"unverified"

"unversed"

"unvexed"

"unvisited"

"unvoiced"

"unvoices"

"unwanted"

"unwarier"

"unwariest"

"unwarily"

"unwariness"

"unwarmed"

"unwarned"

"unwarrantable"

"unwarranted"

"unwary"

"unwashed"

"unwatched"

"unwavering"

"unwaveringly"

"unwaxed"

"unweakened"

"unweaned"

"unwearable"

"unwearably"

"unweary"

"unwearying"

"unweave"

"unweaves"

"unweaving"

"unwed"

"unweeded"

"unweighted"

"unwelcome"

"unwelded"

"unwell"

"unwept"

"unwholesome"

"unwholesomely"

"unwholesomeness"

"unwieldier"

"unwieldiness"

"unwieldy"

"unwifely"

"unwilled"

"unwilling"

"unwillingly"

"unwillingness"

"unwind"

"unwinder"

"unwinders"

"unwinding"

"unwinds"

"unwise"

"unwisely"

"unwiser"

"unwisest"

"unwished"

"unwishes"

"unwit"

"unwitnessed"

"unwitted"

"unwitting"

"unwittingly"

"unwomanly"

"unwon"

"unwonted"

"unwontedly"

"unworkable"

"unworkably"

"unworked"

"unworldly"

"unworn"

"unworried"

"unworthier"

"unworthies"

"unworthily"

"unworthiness"

"unworthy"

"unwound"

"unwove"

"unwoven"

"unwrap"

"unwrapped"

"unwrapping"

"unwraps"

"unwrinkle"

"unwrinkled"

"unwrinkles"

"unwrinkling"

"unwritten"

"unyielding"

"unyieldingly"

"unyoke"

"unyoked"

"unyokes"

"unyoking"

"unzealous"

"unzealously"

"unzip"

"unzipped"

"unzipping"

"unzips"

"up"

"up to date"

"upbearer"

"upbeat"

"upbeats"

"upboiling"

"upbraid"

"upbraided"

"upbraider"

"upbraiders"

"upbraiding"

"upbraids"

"upbringing"

"upchuck"

"upchucked"

"upchucking"

"upchucks"

"upcoiling"

"upcoming"

"upcountry"

"upcurve"

"upcurved"

"upcurves"

"upcurving"

"updatable"

"update"

"updated"

"updater"

"updaters"

"updates"

"updating"

"updraft"

"updrafts"

"upend"

"upended"

"upending"

"upends"

"upgrade"

"upgraded"

"upgrades"

"upgrading"

"upheaval"

"upheavals"

"upheave"

"upheaved"

"upheaver"

"upheavers"

"upheaves"

"upheaving"

"upheld"

"uphill"

"uphills"

"uphold"

"upholder"

"upholders"

"upholding"

"upholds"

"upholster"

"upholstered"

"upholsterer"

"upholsterers"

"upholsteries"

"upholstering"

"upholsters"

"upholstery"

"upkeep"

"upkeeps"

"upland"

"uplander"

"uplanders"

"uplands"

"upleaping"

"uplift"

"uplifted"

"uplifter"

"uplifters"

"uplifting"

"upliftment"

"uplifts"

"uplink"

"uplinked"

"uplinking"

"uplinks"

"upload"

"uploadable"

"uploaded"

"uploading"

"uploads"

"upmost"

"upon"

"upped"

"upper"

"uppercase"

"upperclassman"

"upperclassmen"

"uppercut"

"uppercuts"

"uppermost"

"uppers"

"upping"

"uppish"

"uppity"

"upraise"

"upraised"

"upraiser"

"upraisers"

"upraises"

"upraising"

"upreached"

"upreaches"

"uprear"

"upreared"

"uprearing"

"uprears"

"upreme"

"upright"

"uprighted"

"uprightly"

"uprightness"

"uprights"

"uprise"

"uprisen"

"upriser"

"uprisers"

"uprises"

"uprising"

"uprisings"

"upriver"

"uprivers"

"uproar"

"uproarious"

"uproariously"

"uproariousness"

"uproars"

"uproot"

"uprootals"

"uprooted"

"uprooter"

"uprooters"

"uprooting"

"uproots"

"uprose"

"uprousing"

"ups"

"upscale"

"upsending"

"upset"

"upsets"

"upsetter"

"upsetters"

"upsetting"

"upshift"

"upshifted"

"upshifting"

"upshifts"

"upshot"

"upshots"

"upside"

"upsilon"

"upsilons"

"upstage"

"upstaged"

"upstages"

"upstaging"

"upstairs"

"upstanding"

"upstart"

"upstarts"

"upstate"

"upstream"

"upstroke"

"upstrokes"

"upsurge"

"upsurged"

"upsurges"

"upsurging"

"upsweep"

"upsweeps"

"upswell"

"upswelled"

"upswells"

"upswept"

"upswing"

"upswings"

"upswollen"

"upswung"

"uptake"

"uptakes"

"uptight"

"uptightness"

"uptilts"

"uptime"

"uptimes"

"uptown"

"uptowner"

"uptowners"

"uptowns"

"upturn"

"upturned"

"upturning"

"upturns"

"upward"

"upwardly"

"upwardness"

"upwards"

"upwelled"

"upwelling"

"upwells"

"upwind"

"uracil"

"ural"

"uranian"

"uranic"

"uranium"

"uraniums"

"uranography"

"uranology"

"uranomania"

"uranophobia"

"uranous"

"uranus"

"urb"

"urban"

"urbana"

"urbane"

"urbanely"

"urbaner"

"urbanest"

"urbanism"

"urbanisms"

"urbanist"

"urbanists"

"urbanite"

"urbanites"

"urbanities"

"urbanity"

"urbanization"

"urbanize"

"urbanized"

"urbanizes"

"urbanizing"

"urbanologist"

"urbanologists"

"urbanology"

"urbs"

"urchin"

"urchins"

"urds"

"ure"

"urea"

"ureal"

"ureas"

"ureic"

"uremia"

"uremic"

"urenology"

"ureter"

"ureters"

"urethanes"

"urethra"

"urethrae"

"urethral"

"urethras"

"Urethritis"

"urethroscope"

"uretic"

"urge"

"urge to urinate"

"urged"

"urgencies"

"urgency"

"urgent"

"urgently"

"urger"

"urgers"

"urges"

"urging"

"urgingly"

"uric"

"urinal"

"urinals"

"urinalyses"

"urinalysis"

"urinaries"

"urinary"

"Urinary System"

"urinary tract"

"Urinary tract infection"

"urinate"

"urinated"

"urinates"

"urinating"

"urination"

"urine"

"urines"

"urinogenital"

"urinometer"

"urn"

"urns"

"urogenital"

"urogram"

"urolith"

"urolithic"

"uroliths"

"urologic"

"urological"

"urologies"

"urologist"

"urologists"

"urology"

"uropean"

"uroscopic"

"ursa"

"ursae"

"ursiform"

"ursine"

"urticaria"

"uruguay"

"uruguayan"

"uruguayans"

"urushiol"

"urushiols"

"us"

"usa"

"usability"

"usable"

"usableness"

"usably"

"usage"

"usages"

"use"

"useability"

"useable"

"useably"

"used"

"usee"

"useful"

"usefully"

"usefulness"

"useless"

"uselessly"

"uselessness"

"user"

"user-friendly"

"users"

"uses"

"usher"

"ushered"

"usherette"

"usherettes"

"ushering"

"ushers"

"using"

"uslim"

"ussian"

"ussr"

"usual"

"usually"

"usualness"

"usuals"

"usufruct"

"usufructs"

"usufructuary"

"usurer"

"usurers"

"usuries"

"usurious"

"usuriously"

"usurp"

"usurpation"

"usurpations"

"usurpative"

"usurpatory"

"usurped"

"usurper"

"usurpers"

"usurping"

"usurps"

"usury"

"ut"

"utah"

"utahan"

"utahans"

"utch"

"utensil"

"utensils"

"uteri"

"uterine"

"utero"

"uterus"

"uteruses"

"UTI"

"utile"

"utilise"

"utilitarian"

"utilitarianism"

"utilitarians"

"utilities"

"utility"

"utilizable"

"utilization"

"utilizations"

"utilize"

"utilized"

"utilizer"

"utilizers"

"utilizes"

"utilizing"

"utmost"

"utmosts"

"utopia"

"utopian"

"utopians"

"utopias"

"utopisms"

"utopists"

"utter"

"utterance"

"utterances"

"uttered"

"utterer"

"utterers"

"uttering"

"utterly"

"uttermost"

"utters"

"uveal"

"uveas"

"uvula"

"uvulae"

"uvular"

"uvularly"

"uvulars"

"uvulas"

"uxorial"

"uxorious"

"uxoriously"

"uxoriousness"

"va"

"vacancies"

"vacancy"

"vacant"

"vacantly"

"vacatable"

"vacate"

"vacated"

"vacates"

"vacating"

"vacation"

"vacationed"

"vacationer"

"vacationers"

"vacationing"

"vacationist"

"vacationists"

"vacationland"

"vacations"

"vaccinable"

"vaccinal"

"vaccinate"

"vaccinated"

"vaccinates"

"vaccinating"

"vaccination"

"vaccinations"

"vaccinator"

"vaccinators"

"vaccine"

"vaccinee"

"vaccines"

"vaccinia"

"vaccinial"

"vaccinotherapy"

"vacillate"

"vacillated"

"vacillates"

"vacillating"

"vacillation"

"vacillations"

"vacillator"

"vacillators"

"vacua"

"vacuities"

"vacuity"

"vacuo"

"vacuolar"

"vacuolate"

"vacuole"

"vacuoles"

"vacuous"

"vacuously"

"vacuousness"

"vacuum"

"vacuumed"

"vacuuming"

"vacuums"

"vade"

"vadis"

"vagabond"

"vagabondage"

"vagabonded"

"vagabondism"

"vagabonds"

"vagal"

"vagaries"

"vagarious"

"vagary"

"vagina"

"vaginae"

"vaginal"

"vaginal swab"

"vaginally"

"vaginas"

"vaginate"

"vaginated"

"vaginitis"

"vagrance"

"vagrancies"

"vagrancy"

"vagrant"

"vagrantly"

"vagrants"

"vagrom"

"vague"

"vaguely"

"vagueness"

"vaguer"

"vaguest"

"vagus"

"vail"

"vailing"

"vain"

"vainer"

"vainest"

"vainglorious"

"vainglory"

"vainly"

"vainness"

"val"

"valance"

"valanced"

"valances"

"valancing"

"vale"

"valediction"

"valedictions"

"valedictorian"

"valedictorians"

"valedictories"

"valedictory"

"valence"

"valences"

"valencia"

"valencias"

"valency"

"valentine"

"valentines"

"valerian"

"valerians"

"vales"

"valet"

"valeted"

"valeting"

"valets"

"valetudinarian"

"valetudinarianism"

"valetudinarians"

"valhalla"

"valiance"

"valiancies"

"valiancy"

"valiant"

"valiantly"

"valiants"

"valid"

"validate"

"validated"

"validates"

"validating"

"validation"

"validations"

"validatory"

"validities"

"validity"

"validly"

"validness"

"valise"

"valises"

"valium"

"valkyrie"

"valkyries"

"valkyrs"

"valley"

"valleys"

"valor"

"valorem"

"valorization"

"valorizations"

"valorize"

"valorized"

"valorizes"

"valorizing"

"valorous"

"valorously"

"valors"

"valour"

"valours"

"valse"

"valses"

"valuable"

"valuable resource"

"valuableness"

"valuables"

"valuably"

"valuate"

"valuated"

"valuates"

"valuating"

"valuation"

"valuational"

"valuations"

"valuative"

"valuator"

"valuators"

"value"

"valued"

"valueless"

"valuer"

"valuers"

"values"

"valuing"

"valuta"

"valutas"

"valva"

"valval"

"valvar"

"valvate"

"valve"

"valved"

"valveless"

"valvelet"

"valvelets"

"valves"

"valving"

"valvular"

"valvules"

"vamoose"

"vamoosed"

"vamooses"

"vamoosing"

"vamp"

"vamped"

"vamper"

"vampers"

"vamping"

"vampire"

"vampires"

"vampiric"

"vampirism"

"vampish"

"vamps"

"van"

"vanadium"

"vanadiums"

"vancomycin"

"vancouver"

"vandal"

"vandalic"

"vandalism"

"vandalistic"

"vandalization"

"vandalize"

"vandalized"

"vandalizes"

"vandalizing"

"vandals"

"vandyke"

"vandykes"

"vane"

"vaned"

"vanes"

"vanguard"

"vanguards"

"vanilla"

"vanillas"

"vanillic"

"vanillin"

"vanillins"

"vanish"

"vanished"

"vanisher"

"vanishers"

"vanishes"

"vanishing"

"vanitied"

"vanities"

"vanity"

"vanman"

"vanmen"

"vanquish"

"vanquished"

"vanquisher"

"vanquishers"

"vanquishes"

"vanquishing"

"vanquishment"

"vans"

"vantage"

"vantages"

"vanward"

"vapid"

"vapidities"

"vapidity"

"vapidly"

"vapidness"

"vapor"

"vapored"

"vaporer"

"vaporers"

"vaporimeter"

"vaporing"

"vaporings"

"vaporise"

"vaporish"

"vaporishness"

"vaporization"

"vaporize"

"vaporized"

"vaporizer"

"vaporizers"

"vaporizes"

"vaporizing"

"vaporous"

"vaporously"

"vaporousness"

"vapors"

"vapory"

"vapotherapy"

"vapour"

"vapoured"

"vapourer"

"vapourers"

"vapouring"

"vapours"

"vapoury"

"vaquero"

"vaqueros"

"vara"

"variabilities"

"variability"

"variable"

"variableness"

"variables"

"variably"

"variance"

"variances"

"variant"

"variants"

"variated"

"variates"

"variating"

"variation"

"variational"

"variations"

"varicolored"

"varicose"

"varicosities"

"varicosity"

"varied"

"variedly"

"variegate"

"variegated"

"variegates"

"variegating"

"variegation"

"variegations"

"varier"

"variers"

"varies"

"varietal"

"varietally"

"varietals"

"varieties"

"variety"

"variform"

"variometer"

"variorum"

"variorums"

"various"

"variously"

"variousness"

"varistor"

"varistors"

"varlet"

"varletry"

"varlets"

"varment"

"varments"

"varmint"

"varmints"

"varnish"

"varnished"

"varnishes"

"varnishing"

"varnishy"

"varsities"

"varsity"

"vary"

"varying"

"varyingly"

"vascular"

"vascularities"

"vascularly"

"vasculums"

"vase"

"vasectomies"

"vasectomize"

"vasectomized"

"vasectomizing"

"vasectomy"

"vaseline"

"vases"

"vasoconstriction"

"vasoconstrictive"

"vasoconstrictor"

"vasoconstrictors"

"vasodepressor"

"vasodilatation"

"vasodilation"

"vasodilator"

"vasoinhibitor"

"vasoinhibitory"

"vasomotor"

"vasopressin"

"vasopressor"

"vassal"

"vassalage"

"vassals"

"vassar"

"vast"

"vaster"

"vastest"

"vastier"

"vastiest"

"vastity"

"vastly"

"vastness"

"vasts"

"vasty"

"vat"

"vatful"

"vatfuls"

"vatic"

"vatican"

"vats"

"vatted"

"vatting"

"vaudeville"

"vaudevillian"

"vaudevillians"

"vault"

"vaulted"

"vaulter"

"vaulters"

"vaultier"

"vaultiest"

"vaulting"

"vaultings"

"vaults"

"vaulty"

"vaunt"

"vaunted"

"vaunter"

"vaunters"

"vauntful"

"vaunting"

"vaunts"

"vaunty"

"vc"

"veal"

"vealier"

"veals"

"vealy"

"vector"

"vectored"

"vectorial"

"vectoring"

"vectors"

"veda"

"vedanta"

"vedantic"

"vedic"

"vee"

"veep"

"veepee"

"veepees"

"veeps"

"veer"

"veered"

"veeries"

"veering"

"veeringly"

"veers"

"veery"

"vees"

"vegan"

"veganism"

"veganisms"

"vegans"

"vegas"

"vegetable"

"vegetables"

"vegetal"

"vegetarian"

"vegetarianism"

"vegetarians"

"vegetate"

"vegetated"

"vegetates"

"vegetating"

"vegetation"

"vegetational"

"vegetative"

"vegetist"

"vegetists"

"vegetive"

"vehemence"

"vehemency"

"vehement"

"vehemently"

"vehicle"

"vehicles"

"vehicular"

"veil"

"veiled"

"veiledly"

"veiler"

"veilers"

"veiling"

"veilings"

"veils"

"vein"

"veinal"

"veined"

"veiner"

"veiners"

"veinier"

"veining"

"veinings"

"veinless"

"veinlet"

"veinlets"

"veins"

"veinule"

"veiny"

"vela"

"velar"

"velcro"

"veld"

"velds"

"veldt"

"veldts"

"velleities"

"velleity"

"vellicate"

"vellicating"

"vellication"

"vellum"

"vellums"

"velocimeter"

"velocipede"

"velocipedes"

"velocities"

"velocity"

"velocity statistics"

"velometer"

"velour"

"velours"

"velum"

"velure"

"velured"

"velures"

"veluring"

"velvet"

"velveted"

"velveteen"

"velvets"

"velvety"

"venal"

"venalities"

"venality"

"venally"

"venalness"

"venatic"

"venation"

"venations"

"vend"

"vendable"

"vended"

"vendee"

"vendees"

"vender"

"venders"

"vendetta"

"vendettas"

"vendibility"

"vendible"

"vendibles"

"vendibly"

"vending"

"vendor"

"vendors"

"vends"

"veneer"

"veneered"

"veneerer"

"veneerers"

"veneering"

"veneers"

"venerability"

"venerable"

"venerably"

"venerate"

"venerated"

"venerates"

"venerating"

"veneration"

"venereal"

"venereology"

"veneris"

"venerology"

"venery"

"venetian"

"venetians"

"venezuela"

"venezuelan"

"venezuelans"

"vengeance"

"vengeant"

"venged"

"vengeful"

"vengefully"

"vengefulness"

"venges"

"venging"

"venial"

"venially"

"venice"

"venin"

"venine"

"venins"

"venipuncture"

"venire"

"venireman"

"veniremen"

"venires"

"venison"

"venisons"

"venom"

"venomed"

"venomer"

"venomers"

"venoming"

"venomous"

"venomously"

"venoms"

"venose"

"venosities"

"venous"

"vent"

"ventage"

"vented"

"venter"

"venters"

"ventilate"

"ventilated"

"ventilates"

"ventilating"

"ventilation"

"ventilator"

"ventilators"

"ventilatory"

"venting"

"ventless"

"ventral"

"ventrally"

"ventrals"

"ventricle"

"ventricles"

"ventricular"

"ventricular hypertrophy"

"ventriloquism"

"ventriloquist"

"ventriloquists"

"ventriloquy"

"vents"

"venture"

"ventured"

"venturer"

"venturers"

"ventures"

"venturesome"

"venturesomely"

"venturesomeness"

"venturi"

"venturing"

"venturis"

"venturous"

"venturously"

"venturousness"

"venue"

"venues"

"venular"

"venules"

"venus"

"venusian"

"venusians"

"veracious"

"veraciously"

"veraciousness"

"veracities"

"veracity"

"veranda"

"verandah"

"verandahs"

"verandas"

"verb"

"verbal"

"verbalization"

"verbalizations"

"verbalize"

"verbalized"

"verbalizes"

"verbalizing"

"verbally"

"verbals"

"verbatim"

"verbena"

"verbenas"

"verbiage"

"verbiages"

"verbid"

"verbids"

"verbified"

"verbifies"

"verbify"

"verbile"

"verbless"

"verbomania"

"verbose"

"verbosely"

"verboseness"

"verbosity"

"verboten"

"verbs"

"verdancies"

"verdancy"

"verdant"

"verdantly"

"verde"

"verdi"

"verdict"

"verdicts"

"verdigris"

"verdure"

"verdured"

"verdures"

"verge"

"verged"

"vergences"

"verger"

"vergers"

"verges"

"verging"

"veridic"

"verier"

"veriest"

"verifiability"

"verifiable"

"verifiableness"

"verification"

"verifications"

"verificatory"

"verified"

"verifier"

"verifiers"

"verifies"

"verify"

"verifying"

"verily"

"verisimilitude"

"verisms"

"verists"

"veritable"

"veritably"

"veritas"

"verite"

"verities"

"verity"

"vermeil"

"vermeology"

"vermicelli"

"vermicide"

"vermiculite"

"vermiculites"

"vermiform"

"vermifuge"

"vermifuges"

"vermilion"

"vermin"

"verminous"

"verminously"

"vermont"

"vermonter"

"vermonters"

"vermouth"

"vermouths"

"vermuth"

"vernacular"

"vernacularly"

"vernaculars"

"vernal"

"vernalization"

"vernalize"

"vernalized"

"vernalizes"

"vernalizing"

"vernally"

"vernier"

"verniers"

"veronica"

"veronicas"

"vers"

"versa"

"versailles"

"versal"

"versant"

"versatile"

"versatilely"

"versatileness"

"versatility"

"verse"

"versed"

"verseman"

"versemen"

"verser"

"versers"

"verses"

"versicle"

"versicles"

"versicolored"

"versification"

"versifications"

"versified"

"versifier"

"versifiers"

"versifies"

"versify"

"versifying"

"versine"

"versing"

"version"

"versional"

"versions"

"verso"

"versos"

"verst"

"versus"

"vert"

"vertebra"

"vertebra diseases"

"vertebrae"

"vertebral"

"vertebrally"

"vertebras"

"vertebrate"

"vertebrated"

"vertebrates"

"vertex"

"vertexes"

"vertical"

"verticality"

"vertically"

"verticalness"

"verticals"

"vertices"

"verticillate"

"vertigines"

"vertiginous"

"vertiginously"

"vertigo"

"vertigoes"

"vertigos"

"vervain"

"vervains"

"verve"

"verves"

"vervet"

"vervets"

"very"

"vesicant"

"vesicants"

"vesicle"

"vesicles"

"vesicular"

"vesiculate"

"vesper"

"vesperal"

"vesperals"

"vespers"

"vespertine"

"vespucci"

"vessel"

"vesseled"

"vessels"

"vest"

"vestal"

"vestally"

"vestals"

"vestas"

"vested"

"vestee"

"vestees"

"vestibular"

"vestibule"

"vestibules"

"vestige"

"vestiges"

"vestigial"

"vestigially"

"vesting"

"vestings"

"vestless"

"vestment"

"vestments"

"vestries"

"vestry"

"vestryman"

"vestrymen"

"vests"

"vestural"

"vesture"

"vestured"

"vestures"

"vesuvians"

"vesuvius"

"vet"

"vetch"

"vetches"

"veteran"

"veteran solution"

"veterans"

"veterinarian"

"veterinarians"

"veterinaries"

"veterinary"

"veterinary medicine"

"veto"

"vetoed"

"vetoer"

"vetoers"

"vetoes"

"vetoing"

"vets"

"vetted"

"vetting"

"vex"

"vexation"

"vexations"

"vexatious"

"vexatiously"

"vexatiousness"

"vexed"

"vexedly"

"vexer"

"vexers"

"vexes"

"vexillology"

"vexing"

"vexingly"

"via"

"viabilities"

"viability"

"viable"

"viably"

"viaduct"

"viaducts"

"vial"

"vialed"

"vialing"

"vialled"

"vialling"

"vials"

"viameter"

"viand"

"viands"

"vias"

"viatica"

"viaticum"

"viaticums"

"viators"

"vibes"

"vibists"

"vibraharp"

"vibraharps"

"vibrance"

"vibrances"

"vibrancies"

"vibrancy"

"vibrant"

"vibrantly"

"vibrants"

"vibraphone"

"vibraphones"

"vibrate"

"vibrated"

"vibrates"

"vibrating"

"vibration"

"vibrational"

"vibrational energy"

"vibrations"

"vibrato"

"vibrator"

"vibrators"

"vibratory"

"vibratos"

"vibrograph"

"vibrometer"

"viburnum"

"viburnums"

"vicar"

"vicarage"

"vicarages"

"vicarate"

"vicarates"

"vicarial"

"vicariate"

"vicariates"

"vicarious"

"vicariously"

"vicariousness"

"vicarly"

"vicars"

"vice"

"viced"

"vicegerencies"

"vicegerency"

"vicegerent"

"vicegerents"

"viceless"

"vicennial"

"viceregal"

"viceregally"

"viceregent"

"viceregents"

"viceroy"

"viceroyalty"

"viceroys"

"vices"

"vichies"

"vichy"

"vichyssoise"

"vicinage"

"vicinal"

"vicing"

"vicinities"

"vicinity"

"vicious"

"viciously"

"viciousness"

"vicissitude"

"vicissitudes"

"vicomte"

"victim"

"victimization"

"victimizations"

"victimize"

"victimized"

"victimizer"

"victimizers"

"victimizes"

"victimizing"

"victimless"

"victimology"

"victims"

"victor"

"victoria"

"victorian"

"victorianism"

"victorians"

"victorias"

"victories"

"victorious"

"victoriously"

"victoriousness"

"victors"

"victory"

"victress"

"victresses"

"victual"

"victualed"

"victualer"

"victualers"

"victualing"

"victualled"

"victualler"

"victuallers"

"victualling"

"victuals"

"vicuna"

"vicunas"

"vide"

"videlicet"

"video"

"videocassette"

"videocassettes"

"videodisc"

"videodiscs"

"videos"

"videotape"

"videotaped"

"videotapes"

"videotaping"

"videotext"

"vidkid"

"vidkids"

"vie"

"vied"

"vienna"

"viennese"

"vier"

"viers"

"vies"

"vietcong"

"vietnam"

"vietnamese"

"view"

"viewable"

"viewed"

"viewer"

"viewers"

"viewfinder"

"viewfinders"

"viewier"

"viewing"

"viewings"

"viewless"

"viewpoint"

"viewpoints"

"views"

"viewy"

"vigesimal"

"vigil"

"vigilance"

"vigilant"

"vigilante"

"vigilantes"

"vigilantism"

"vigilantly"

"vigilantness"

"vigils"

"vignette"

"vignetted"

"vignettes"

"vignetting"

"vignettist"

"vignettists"

"vigor"

"vigorish"

"vigorous"

"vigorously"

"vigorousness"

"vigors"

"vigour"

"vigours"

"viking"

"vikings"

"vile"

"vilely"

"vileness"

"viler"

"vilest"

"vilification"

"vilified"

"vilifier"

"vilifiers"

"vilifies"

"vilify"

"vilifying"

"villa"

"villadom"

"villadoms"

"village"

"villager"

"villagers"

"villages"

"villain"

"villainess"

"villainesses"

"villainies"

"villainous"

"villainously"

"villainousness"

"villains"

"villainy"

"villas"

"villein"

"villeinage"

"villi"

"villous"

"villus"

"vim"

"vims"

"vin"

"vinaigrette"

"vinaigrettes"

"vinal"

"vinas"

"vinca"

"vincas"

"vincent"

"vincible"

"vinculum"

"vindicable"

"vindicate"

"vindicated"

"vindicates"

"vindicating"

"vindication"

"vindications"

"vindicative"

"vindicator"

"vindicators"

"vindicatory"

"vindictive"

"vindictively"

"vindictiveness"

"vine"

"vineal"

"vined"

"vinegar"

"vinegars"

"vinegary"

"vineries"

"vinery"

"vines"

"vineyard"

"vineyards"

"vinic"

"vinier"

"viniest"

"vining"

"vino"

"vinology"

"vinos"

"vinosities"

"vinosity"

"vinous"

"vinously"

"vins"

"vintage"

"vintagers"

"vintages"

"vintner"

"vintners"

"viny"

"vinyl"

"vinylic"

"vinyls"

"viol"

"viola"

"violability"

"violable"

"violably"

"violas"

"violate"

"violated"

"violater"

"violaters"

"violates"

"violating"

"violation"

"violations"

"violative"

"violator"

"violators"

"violence"

"violences"

"violent"

"violently"

"violet"

"violets"

"violin"

"violinist"

"violinists"

"violins"

"violist"

"violists"

"violoncellist"

"violoncellists"

"violoncello"

"violoncellos"

"viols"

"vip"

"viper"

"viperidae"

"viperine"

"viperish"

"viperous"

"vipers"

"vips"

"virago"

"viragoes"

"viragos"

"viral"

"virally"

"vireo"

"vireos"

"virgate"

"virgil"

"virgin"

"virginal"

"virginally"

"virginals"

"virginia"

"virginian"

"virginians"

"virginities"

"virginity"

"virginium"

"virgins"

"virgo"

"virgos"

"virgule"

"virgules"

"viricidal"

"viricides"

"virid"

"viridescent"

"viridian"

"viridians"

"virile"

"virilities"

"virility"

"virilization"

"virilize"

"virilizing"

"virological"

"virologies"

"virologist"

"virologists"

"virology"

"virtu"

"virtual"

"virtually"

"virtue"

"virtues"

"virtuosas"

"virtuosi"

"virtuosities"

"virtuosity"

"virtuoso"

"virtuosos"

"virtuous"

"virtuously"

"virtuousness"

"virucide"

"virulence"

"virulences"

"virulencies"

"virulency"

"virulent"

"virulently"

"virus"

"viruses"

"vis"

"visa"

"visaed"

"visage"

"visaged"

"visages"

"visaing"

"visard"

"visards"

"visas"

"viscera"

"visceral"

"viscerally"

"visceromotor"

"viscid"

"viscidities"

"viscidity"

"viscidly"

"viscoid"

"viscometer"

"viscose"

"viscoses"

"viscosimeter"

"viscosimetry"

"viscosities"

"viscosity"

"viscount"

"viscountess"

"viscountesses"

"viscounts"

"viscous"

"viscously"

"viscousness"

"viscus"

"vise"

"vised"

"viseing"

"viselike"

"vises"

"vishnu"

"visibility"

"visible"

"visible trail"

"visibly"

"vising"

"vision"

"visional"

"visionaries"

"visionary"

"visioned"

"visioning"

"visions"

"visit"

"visitable"

"visitant"

"visitants"

"visitation"

"visitational"

"visitations"

"visitatorial"

"visited"

"visiter"

"visiters"

"visiting"

"visitor"

"visitorial"

"visitors"

"visitress"

"visits"

"visometer"

"visor"

"visored"

"visoring"

"visorless"

"visors"

"vista"

"vistaed"

"vistas"

"visual"

"Visualisation"

"visualization"

"visualizations"

"visualize"

"visualized"

"visualizer"

"visualizers"

"visualizes"

"visualizing"

"visually"

"visuals"

"vita"

"vitae"

"vital"

"vital information"

"vital medical data"

"vitalising"

"vitalism"

"vitalisms"

"vitalist"

"vitalists"

"vitalities"

"vitality"

"vitalization"

"vitalize"

"vitalized"

"vitalizer"

"vitalizers"

"vitalizes"

"vitalizing"

"vitally"

"vitals"

"vitamin"

"vitamine"

"vitamines"

"vitaminization"

"vitaminized"

"vitaminizing"

"vitaminology"

"vitamins"

"vitiate"

"vitiated"

"vitiates"

"vitiating"

"vitiation"

"vitiator"

"vitiators"

"viticultural"

"viticulture"

"viticulturist"

"viticulturists"

"vitreous"

"vitric"

"vitrics"

"vitrifiable"

"vitrification"

"vitrified"

"vitrifies"

"vitrify"

"vitrifying"

"vitrine"

"vitrines"

"vitriol"

"vitrioled"

"vitriolic"

"vitriols"

"vitro"

"vittle"

"vittled"

"vittles"

"vittling"

"vituperate"

"vituperated"

"vituperates"

"vituperating"

"vituperation"

"vituperations"

"vituperative"

"vituperatively"

"viva"

"vivace"

"vivacious"

"vivaciously"

"vivaciousness"

"vivacities"

"vivacity"

"vivant"

"vivants"

"vivaria"

"vivaries"

"vivarium"

"vivariums"

"vive"

"vivendi"

"vivid"

"vivider"

"vividest"

"vividly"

"vividness"

"vivific"

"vivification"

"vivified"

"vivifier"

"vivifiers"

"vivifies"

"vivify"

"vivifying"

"viviparities"

"viviparity"

"viviparous"

"viviparously"

"vivisect"

"vivisected"

"vivisecting"

"vivisection"

"vivisectional"

"vivisectionist"

"vivisectionists"

"vivisects"

"vivo"

"vivre"

"vixen"

"vixenish"

"vixenishly"

"vixenly"

"vixens"

"viz"

"vizard"

"vizarded"

"vizards"

"vizier"

"viziers"

"vizir"

"vizirs"

"vizor"

"vizored"

"vizors"

"vocable"

"vocables"

"vocably"

"vocabularies"

"vocabulary"

"vocal"

"vocalic"

"vocalism"

"vocalisms"

"vocalist"

"vocalists"

"vocalities"

"vocality"

"vocalization"

"vocalizations"

"vocalize"

"vocalized"

"vocalizer"

"vocalizers"

"vocalizes"

"vocalizing"

"vocally"

"vocals"

"vocation"

"vocational"

"vocations"

"vocative"

"vocatively"

"vocatives"

"voce"

"voces"

"vociferate"

"vociferated"

"vociferates"

"vociferating"

"vociferation"

"vociferations"

"vociferous"

"vociferously"

"vociferousness"

"vocoder"

"vocoders"

"vodka"

"vodkas"

"vogue"

"vogues"

"voguish"

"voice"

"voiced"

"voicedness"

"voiceful"

"voiceless"

"voicelessly"

"voicelessness"

"voiceprint"

"voiceprints"

"voicer"

"voicers"

"voices"

"voicing"

"void"

"voidable"

"voidableness"

"voidance"

"voidances"

"voided"

"voider"

"voiders"

"voiding"

"voidness"

"voids"

"voila"

"voile"

"voiles"

"vol"

"volante"

"volatile"

"volatiles"

"volatility"

"volatilization"

"volatilize"

"volatilized"

"volatilizes"

"volatilizing"

"volcanic"

"volcanically"

"volcanics"

"volcanism"

"volcano"

"volcanoes"

"volcanological"

"volcanologist"

"volcanologists"

"volcanology"

"volcanos"

"vole"

"volente"

"voles"

"volga"

"volition"

"volitional"

"volitionally"

"volitions"

"volkswagen"

"volkswagens"

"volley"

"volleyball"

"volleyballs"

"volleyed"

"volleyer"

"volleyers"

"volleying"

"volleys"

"volplane"

"volplaned"

"volplanes"

"volplaning"

"volt"

"volta"

"voltage"

"voltages"

"voltaic"

"voltaire"

"voltameter"

"voltes"

"voltmeter"

"voltmeters"

"volts"

"volubility"

"voluble"

"volubly"

"volume"

"volumed"

"volumenometer"

"volumes"

"volumeter"

"volumetric"

"volumetrically"

"voluminosity"

"voluminous"

"voluminously"

"voluminousness"

"voluntarily"

"voluntary"

"voluntaryism"

"volunteer"

"volunteered"

"volunteering"

"volunteers"

"voluptuaries"

"voluptuary"

"voluptuous"

"voluptuously"

"voluptuousness"

"volute"

"voluted"

"volutes"

"volution"

"volvox"

"volvoxes"

"vomit"

"vomited"

"vomiter"

"vomiters"

"vomiting"

"vomitive"

"vomitory"

"vomitous"

"vomits"

"vomitus"

"von"

"voodoo"

"voodooed"

"voodooing"

"voodooism"

"voodoos"

"voracious"

"voraciously"

"voraciousness"

"voracities"

"voracity"

"vortex"

"vortexes"

"vortical"

"vortices"

"votable"

"votaries"

"votarist"

"votarists"

"votary"

"vote"

"voteable"

"voted"

"voteless"

"voter"

"voters"

"votes"

"voting"

"votive"

"votively"

"vouch"

"vouched"

"vouchee"

"vouchees"

"voucher"

"voucherable"

"vouchered"

"vouchering"

"vouchers"

"vouches"

"vouching"

"vouchsafe"

"vouchsafed"

"vouchsafes"

"vouchsafing"

"vow"

"vowed"

"vowel"

"vowelize"

"vowelized"

"vowelizes"

"vowels"

"vower"

"vowers"

"vowing"

"vowless"

"vows"

"vox"

"voyage"

"voyaged"

"voyager"

"voyagers"

"voyages"

"voyageur"

"voyageurs"

"voyaging"

"voyeur"

"voyeurism"

"voyeuristic"

"voyeurs"

"vroom"

"vroomed"

"vrooming"

"vrooms"

"vrouw"

"vrouws"

"vrow"

"vrows"

"vs"

"vt"

"vug"

"vugg"

"vuggs"

"vuggy"

"vugh"

"vughs"

"vugs"

"vulcan"

"vulcanic"

"vulcanism"

"vulcanite"

"vulcanization"

"vulcanize"

"vulcanized"

"vulcanizer"

"vulcanizers"

"vulcanizes"

"vulcanizing"

"vulcanology"

"vulgar"

"vulgarer"

"vulgarest"

"vulgarian"

"vulgarians"

"vulgarism"

"vulgarisms"

"vulgarities"

"vulgarity"

"vulgarization"

"vulgarizations"

"vulgarize"

"vulgarized"

"vulgarizer"

"vulgarizers"

"vulgarizes"

"vulgarizing"

"vulgarly"

"vulgarness"

"vulgars"

"vulgate"

"vulgates"

"vulgo"

"vulguses"

"vulnerabilities"

"vulnerability"

"vulnerable"

"vulnerably"

"vulpine"

"vulture"

"vultures"

"vulturous"

"vulva"

"vulvae"

"vulval"

"vulvar"

"vulvas"

"vulvate"

"vying"

"vyingly"

"wa"

"wabble"

"wabbled"

"wabbler"

"wabbles"

"wabbly"

"wack"

"wackier"

"wackiest"

"wackily"

"wackiness"

"wacks"

"wacky"

"wacs"

"wad"

"wadable"

"wadded"

"wadder"

"wadders"

"waddied"

"waddies"

"wadding"

"waddings"

"waddle"

"waddled"

"waddler"

"waddlers"

"waddles"

"waddling"

"waddly"

"waddy"

"wade"

"wadeable"

"waded"

"wader"

"waders"

"wades"

"wadi"

"wadies"

"wading"

"wadis"

"wads"

"wafer"

"wafered"

"wafers"

"wafery"

"waffle"

"waffled"

"waffles"

"waffling"

"waft"

"waftage"

"wafted"

"wafter"

"wafters"

"wafting"

"wafts"

"wag"

"wage"

"waged"

"wageless"

"wager"

"wagered"

"wagerer"

"wagerers"

"wagering"

"wagers"

"wages"

"wagged"

"wagger"

"waggeries"

"waggers"

"waggery"

"wagging"

"waggish"

"waggle"

"waggled"

"waggles"

"waggling"

"waggly"

"waggon"

"waggoned"

"waggoner"

"waggoners"

"waggoning"

"waggons"

"waging"

"wagner"

"wagnerian"

"wagnerians"

"wagon"

"wagonage"

"wagoned"

"wagoner"

"wagoners"

"wagonette"

"wagonettes"

"wagoning"

"wagons"

"wags"

"wagtail"

"wagtails"

"wahine"

"wahines"

"wahoo"

"wahoos"

"waif"

"waifing"

"waifs"

"wail"

"wailed"

"wailer"

"wailers"

"wailful"

"wailfully"

"wailing"

"wails"

"wain"

"wains"

"wainscot"

"wainscoted"

"wainscoting"

"wainscots"

"wainscotted"

"wainscotting"

"wainwright"

"wainwrights"

"waist"

"waist pains"

"waistband"

"waistbands"

"waistcoat"

"waistcoats"

"waisted"

"waister"

"waisters"

"waisting"

"waistings"

"waistline"

"waistlines"

"waists"

"wait"

"waited"

"waiter"

"waiters"

"waiting"

"waitings"

"waitress"

"waitresses"

"waits"

"waive"

"waived"

"waiver"

"waivers"

"waives"

"waiving"

"wake"

"wake up"

"waked"

"wakeful"

"wakefulness"

"wakeless"

"waken"

"wakened"

"wakener"

"wakeners"

"wakening"

"wakenings"

"wakens"

"waker"

"wakers"

"wakes"

"wakiki"

"wakikis"

"waking"

"waldorf"

"wale"

"waled"

"waler"

"wales"

"walgreen app"

"Walgreens "

"waling"

"walk"

"walkable"

"walkaway"

"walkaways"

"walked"

"walker"

"walkers"

"walking"

"walkings"

"walkout"

"walkouts"

"walkover"

"walkovers"

"walks"

"walkup"

"walkups"

"walkway"

"walkways"

"wall"

"walla"

"wallabies"

"wallaby"

"wallah"

"wallahs"

"wallas"

"wallboard"

"walled"

"wallet"

"wallets"

"walleye"

"walleyed"

"walleyes"

"wallflower"

"wallflowers"

"walling"

"walloon"

"wallop"

"walloped"

"walloper"

"wallopers"

"walloping"

"wallops"

"wallow"

"wallowed"

"wallower"

"wallowers"

"wallowing"

"wallows"

"wallpaper"

"wallpapered"

"wallpapering"

"wallpapers"

"walls"

"wally"

"walnut"

"walnuts"

"walrus"

"walruses"

"walt"

"walter"

"waltz"

"waltzed"

"waltzer"

"waltzers"

"waltzes"

"waltzing"

"wammus"

"wampum"

"wampums"

"wan"

"wand"

"wander"

"wandered"

"wanderer"

"wanderers"

"wandering"

"wanderings"

"wanderlust"

"wanders"

"wands"

"wane"

"waned"

"wanes"

"wang"

"wangle"

"wangled"

"wangler"

"wanglers"

"wangles"

"wangling"

"waning"

"wankel"

"wanly"

"wanner"

"wanness"

"wannesses"

"wannest"

"wanning"

"want"

"wantage"

"wanted"

"wanter"

"wanters"

"wanting"

"wanton"

"wantoned"

"wantoner"

"wantoners"

"wantoning"

"wantonly"

"wantonness"

"wantons"

"wants"

"wapiti"

"wapitis"

"wapping"

"war"

"warble"

"warbled"

"warbler"

"warblers"

"warbles"

"warbling"

"warcraft"

"warcrafts"

"ward"

"warded"

"warden"

"wardens"

"wardenship"

"warder"

"warders"

"wardership"

"warding"

"wardress"

"wardresses"

"wardrobe"

"wardrobes"

"wardroom"

"wardrooms"

"wards"

"wardship"

"wardships"

"ware"

"wared"

"warehouse"

"warehoused"

"warehouseman"

"warehousemen"

"warehouser"

"warehousers"

"warehouses"

"warehousing"

"wareroom"

"wares"

"warfare"

"warfares"

"warfarin"

"warfarins"

"warhead"

"warheads"

"warhorse"

"warhorses"

"warier"

"wariest"

"warily"

"wariness"

"waring"

"wark"

"warks"

"warless"

"warlike"

"warlock"

"warlocks"

"warlord"

"warlords"

"warm"

"warmaker"

"warmakers"

"warmed"

"warmer"

"warmers"

"warmest"

"warmhearted"

"warmheartedly"

"warmheartedness"

"warming"

"warmish"

"warmly"

"warmness"

"warmonger"

"warmongering"

"warmongers"

"warms"

"warmth"

"warmths"

"warmup"

"warmups"

"warn"

"warned"

"warner"

"warners"

"warning"

"warningly"

"warnings"

"warns"

"warp"

"warpage"

"warpath"

"warpaths"

"warped"

"warper"

"warpers"

"warping"

"warplane"

"warplanes"

"warpower"

"warpowers"

"warps"

"warrant"

"warrantable"

"warranted"

"warrantee"

"warrantees"

"warranter"

"warranties"

"warranting"

"warrantless"

"warrantor"

"warrantors"

"warrants"

"warranty"

"warred"

"warren"

"warreners"

"warrens"

"warring"

"warrior"

"warriors"

"wars"

"warsaw"

"warsaws"

"warship"

"warships"

"wart"

"warted"

"warthog"

"warthogs"

"wartier"

"wartiest"

"wartime"

"wartimes"

"warts"

"warty"

"warwork"

"warworks"

"warworn"

"wary"

"was"

"wash"

"washability"

"washable"

"washbasin"

"washbasins"

"washboard"

"washboards"

"washbowl"

"washbowls"

"washcloth"

"washcloths"

"washday"

"washdays"

"washed"

"washer"

"washers"

"washerwoman"

"washerwomen"

"washes"

"washier"

"washiest"

"washiness"

"washing"

"washings"

"washington"

"washingtonian"

"washingtonians"

"washout"

"washouts"

"washrag"

"washrags"

"washroom"

"washrooms"

"washstand"

"washstands"

"washtub"

"washtubs"

"washwoman"

"washwomen"

"washy"

"wasp"

"waspier"

"waspily"

"waspish"

"waspishly"

"waspishness"

"wasps"

"waspy"

"wassail"

"wassailed"

"wassailer"

"wassailers"

"wassailing"

"wassails"

"wast"

"wastable"

"wastage"

"wastages"

"waste"

"wastebasket"

"wastebaskets"

"wasted"

"wasteful"

"wastefully"

"wastefulness"

"wasteland"

"wastelands"

"wastepaper"

"waster"

"wasters"

"wastery"

"wastes"

"wastier"

"wasting"

"wastrel"

"wastrels"

"wasts"

"watch"

"watchband"

"watchbands"

"watchdog"

"watchdogs"

"watched"

"watcher"

"watchers"

"watches"

"watchful"

"watchfully"

"watchfulness"

"watching"

"watchmaker"

"watchmakers"

"watchmaking"

"watchman"

"watchmen"

"watchout"

"watchtower"

"watchtowers"

"watchwoman"

"watchwomen"

"watchword"

"watchwords"

"water"

"Water Fall"

"waterbed"

"waterbeds"

"waterborne"

"waterbury"

"watercolor"

"watercolors"

"watercourse"

"watercourses"

"watercraft"

"watercress"

"watercresses"

"watered"

"waterer"

"waterers"

"waterfall"

"waterfalls"

"waterfowl"

"waterfowls"

"waterfront"

"waterfronts"

"watergate"

"waterier"

"wateriest"

"waterily"

"watering"

"waterings"

"waterish"

"waterlog"

"waterlogged"

"waterlogging"

"waterlogs"

"waterloo"

"waterloos"

"waterman"

"watermark"

"watermarked"

"watermarking"

"watermarks"

"watermelon"

"watermelons"

"watermen"

"waterpower"

"waterproof"

"waterproofed"

"waterproofer"

"waterproofing"

"waterproofs"

"waters"

"watershed"

"watersheds"

"waterside"

"waterskiing"

"waterspout"

"waterspouts"

"watertight"

"waterway"

"waterways"

"waterwheel"

"waterworks"

"waterworthy"

"watery"

"wats"

"watson"

"watt"

"wattage"

"wattages"

"wattest"

"watthour"

"watthours"

"wattle"

"wattled"

"wattles"

"wattling"

"wattmeter"

"watts"

"waugh"

"waul"

"wauls"

"wave"

"waveband"

"wavebands"

"waved"

"waveform"

"waveforms"

"wavelength"

"wavelengths"

"waveless"

"wavelet"

"wavelets"

"wavelike"

"wavemeter"

"waveoff"

"waveoffs"

"waver"

"wavered"

"waverer"

"waverers"

"wavering"

"waveringly"

"wavers"

"wavery"

"waves"

"wavey"

"waveys"

"wavier"

"waviest"

"wavily"

"waviness"

"waving"

"wavy"

"wawls"

"wax"

"waxbill"

"waxed"

"waxen"

"waxer"

"waxers"

"waxes"

"waxier"

"waxiest"

"waxily"

"waxiness"

"waxing"

"waxings"

"waxwing"

"waxwings"

"waxwork"

"waxworks"

"waxy"

"way"

"waybill"

"waybills"

"wayfarer"

"wayfarers"

"wayfaring"

"wayfarings"

"waylaid"

"waylay"

"waylayer"

"waylayers"

"waylaying"

"waylays"

"wayless"

"wayne"

"ways"

"wayside"

"waysides"

"wayward"

"waywardly"

"waywardness"

"wayworn"

"we"

"weak"

"weaken"

"weakened"

"weakener"

"weakeners"

"weakening"

"weakens"

"weaker"

"weakest"

"weakfish"

"weakfishes"

"weakhearted"

"weakish"

"weaklier"

"weakliest"

"weakling"

"weaklings"

"weakly"

"weakness"

"weaknesses"

"weal"

"weald"

"weals"

"wealth"

"wealthier"

"wealthiest"

"wealthiness"

"wealths"

"wealthy"

"wean"

"weaned"

"weaner"

"weaners"

"weaning"

"weanling"

"weanlings"

"weans"

"weapon"

"weaponed"

"weaponing"

"weaponless"

"weaponries"

"weaponry"

"weapons"

"wear"

"wearable"

"wearables"

"wearer"

"wearers"

"wearied"

"wearier"

"wearies"

"weariest"

"weariful"

"weariless"

"wearily"

"weariness"

"wearing"

"wearish"

"wearisome"

"wearisomely"

"wearisomeness"

"wears"

"weary"

"wearying"

"weasand"

"weasands"

"weasel"

"weaseled"

"weaseling"

"weaselly"

"weasels"

"weather"

"weather channel"

"weather channel pollen"

"weatherability"

"weatherboard"

"weatherbound"

"weathercock"

"weathercocks"

"weathered"

"weatherglass"

"weatherglasses"

"weathering"

"weatherman"

"weathermen"

"weatherometer"

"weatherproof"

"weatherproofed"

"weatherproofing"

"weatherproofs"

"weathers"

"weatherstrip"

"weatherstripped"

"weatherstrippers"

"weatherstripping"

"weatherstrips"

"weatherwise"

"weatherworn"

"weave"

"weaved"

"weaver"

"weavers"

"weaves"

"weaving"

"weazand"

"weazands"

"web"

"web app"

"webbed"

"webbier"

"webbing"

"webbings"

"webby"

"weber"

"webers"

"webfeet"

"webfoot"

"webfooted"

"webless"

"WebMD"

"webpages"

"webs"

"website"

"webster"

"websters"

"webworm"

"wed"

"wedded"

"wedder"

"wedders"

"wedding"

"weddings"

"wedge"

"wedged"

"wedges"

"wedgie"

"wedgier"

"wedgies"

"wedging"

"wedgy"

"wedlock"

"wedlocks"

"wednesday"

"wednesdays"

"weds"

"wee"

"weed"

"weeded"

"weeder"

"weeders"

"weedier"

"weediest"

"weedily"

"weediness"

"weeding"

"weedless"

"weeds"

"weedy"

"week"

"weekday"

"weekdays"

"weekend"

"weekended"

"weekender"

"weekending"

"weekends"

"weeklies"

"weeklong"

"weekly"

"weekly alarms"

"weeks"

"Weeks gestational age"

"ween"

"weened"

"weenie"

"weenier"

"weenies"

"weeniest"

"weening"

"weens"

"weensier"

"weensiest"

"weensy"

"weeny"

"weep"

"weeper"

"weepers"

"weepier"

"weepiest"

"weeping"

"weeps"

"weepy"

"weest"

"weevil"

"weeviled"

"weevilly"

"weevils"

"weevily"

"weewee"

"weeweed"

"weeweeing"

"weewees"

"weft"

"wefts"

"wehner"

"weigh"

"weighage"

"weighed"

"weigher"

"weighers"

"weighing"

"weighman"

"weighmaster"

"weighmen"

"weighs"

"weight"

"Weight Control"

"weight goal"

"weight loos"

"weight loss"

"weight loss goal"

"Weight Lost"

"weight training"

"weight warchers"

"weight watchers"

"Weight Watchers Points Plus"

"weight you've lost"

"Weight-loss"

"weighted"

"weighter"

"weighters"

"weightier"

"weightiest"

"weightily"

"weightiness"

"weighting"

"weightless"

"weightlessly"

"weightlessness"

"weights"

"weighty"

"weiner"

"weiners"

"weir"

"weird"

"weirder"

"weirdest"

"weirdie"

"weirdies"

"weirdly"

"weirdness"

"weirdo"

"weirdoes"

"weirdos"

"weirds"

"weirdy"

"weirs"

"welch"

"welched"

"welcher"

"welchers"

"welches"

"welching"

"welcome"

"welcomed"

"welcomer"

"welcomers"

"welcomes"

"welcoming"

"weld"

"weldable"

"welded"

"welder"

"welders"

"welding"

"weldless"

"welds"

"welfare"

"welfares"

"welkin"

"welkins"

"well"

"well being"

"well-being"

"welladay"

"welladays"

"wellbeing"

"wellborn"

"wellbred"

"welled"

"wellhead"

"wellheads"

"wellhole"

"wellholes"

"welling"

"wellington"

"wellknown"

"wellness"

"wells"

"wellsite"

"wellspring"

"wellsprings"

"welsh"

"welshed"

"welsher"

"welshers"

"welshes"

"welshing"

"welshman"

"welshmen"

"welshwoman"

"welshwomen"

"welt"

"weltanschauung"

"welted"

"welter"

"weltered"

"weltering"

"welters"

"welterweight"

"welterweights"

"welting"

"weltings"

"welts"

"wen"

"wench"

"wenched"

"wencher"

"wenchers"

"wenches"

"wenching"

"wend"

"wended"

"wending"

"wends"

"wennier"

"wennish"

"wenny"

"wens"

"went"

"wept"

"were"

"weregild"

"weregilds"

"werewolf"

"werewolves"

"wergeld"

"wergelt"

"wergild"

"wert"

"werwolf"

"werwolves"

"weskit"

"weskits"

"wesley"

"wesleyans"

"west"

"westbound"

"wester"

"westered"

"westering"

"westerlies"

"westerly"

"western"

"westerner"

"westerners"

"westernize"

"westernized"

"westernizes"

"westernizing"

"westerns"

"westers"

"westing"

"westinghouse"

"westings"

"westminster"

"westmost"

"wests"

"westward"

"westwardly"

"westwards"

"wet"

"wetback"

"wetbacks"

"wether"

"wethers"

"wetland"

"wetlands"

"wetly"

"wetness"

"wetnesses"

"wetproof"

"wets"

"wetsuit"

"wettable"

"wetted"

"wetter"

"wetters"

"wettest"

"wetting"

"wettings"

"wettish"

"wey"

"WGA"

"wha"

"whack"

"whacked"

"whacker"

"whackers"

"whackier"

"whackiest"

"whacking"

"whacks"

"whacky"

"whale"

"whaleboat"

"whaleboats"

"whalebone"

"whalebones"

"whaled"

"whaler"

"whalers"

"whales"

"whaling"

"whalings"

"wham"

"whammed"

"whammies"

"whamming"

"whammy"

"whams"

"whang"

"whanged"

"whangers"

"whanging"

"whangs"

"whap"

"whapped"

"whapper"

"whappers"

"whapping"

"whaps"

"wharf"

"wharfage"

"wharfages"

"wharfed"

"wharfing"

"wharfinger"

"wharfingers"

"wharfmaster"

"wharfs"

"wharve"

"wharves"

"what"

"whatever"

"whatnot"

"whatnots"

"whats"

"whatsoever"

"wheal"

"wheals"

"wheat"

"wheaten"

"wheaties"

"wheats"

"whee"

"wheedle"

"wheedled"

"wheedler"

"wheedlers"

"wheedles"

"wheedling"

"wheel"

"wheelbarrow"

"wheelbarrows"

"wheelbase"

"wheelbases"

"wheelchair"

"wheelchairs"

"wheeled"

"wheeler"

"wheelers"

"wheelie"

"wheelies"

"wheeling"

"wheelings"

"wheelless"

"wheelman"

"wheelmen"

"wheels"

"wheelwright"

"wheelwrights"

"wheeze"

"wheezed"

"wheezer"

"wheezers"

"wheezes"

"wheezier"

"wheeziest"

"wheezily"

"wheeziness"

"wheezing"

"wheezy"

"whelk"

"whelks"

"whelky"

"whelm"

"whelmed"

"whelming"

"whelms"

"whelp"

"whelped"

"whelping"

"whelps"

"when"

"whenas"

"whence"

"whencesoever"

"whenever"

"whens"

"whensoever"

"where"

"whereabouts"

"whereafter"

"whereas"

"whereases"

"whereat"

"whereby"

"wherefor"

"wherefore"

"wherefores"

"wherefrom"

"wherein"

"whereinsoever"

"whereof"

"whereon"

"wheres"

"wheresoever"

"whereto"

"whereunder"

"whereunto"

"whereupon"

"wherever"

"wherewith"

"wherewithal"

"wherries"

"wherry"

"wherrying"

"whet"

"whether"

"whets"

"whetstone"

"whetstones"

"whetted"

"whetter"

"whetters"

"whetting"

"whew"

"whews"

"whey"

"wheyey"

"wheyface"

"wheyfaces"

"wheyish"

"wheys"

"which"

"whichever"

"whichsoever"

"whicker"

"whickered"

"whickering"

"whickers"

"whiff"

"whiffed"

"whiffer"

"whiffers"

"whiffing"

"whiffle"

"whiffled"

"whiffler"

"whifflers"

"whiffles"

"whiffletree"

"whiffletrees"

"whiffling"

"whiffs"

"whig"

"whigs"

"while"

"whiled"

"whiles"

"whiling"

"whilom"

"whilst"

"whim"

"whimper"

"whimpered"

"whimpering"

"whimperingly"

"whimpers"

"whims"

"whimsey"

"whimsical"

"whimsicality"

"whimsically"

"whimsied"

"whimsies"

"whimsy"

"whine"

"whined"

"whiner"

"whiners"

"whines"

"whiney"

"whinier"

"whiniest"

"whining"

"whiningly"

"whinnied"

"whinnier"

"whinnies"

"whinniest"

"whinny"

"whinnying"

"whiny"

"whip"

"whipcord"

"whipcords"

"whiplash"

"whiplashes"

"whipped"

"whipper"

"whippers"

"whippersnapper"

"whippersnappers"

"whippet"

"whippets"

"whippier"

"whippiest"

"whipping"

"whippings"

"whippletree"

"whippoorwill"

"whippoorwills"

"whippy"

"whips"

"whipsaw"

"whipsawed"

"whipsawing"

"whipsawn"

"whipsaws"

"whipt"

"whiptail"

"whiptails"

"whipworm"

"whipworms"

"whir"

"whirl"

"whirled"

"whirler"

"whirlers"

"whirlier"

"whirlies"

"whirliest"

"whirligig"

"whirligigs"

"whirling"

"whirlpool"

"whirlpools"

"whirls"

"whirlwind"

"whirlwinds"

"whirly"

"whirlybird"

"whirlybirds"

"whirr"

"whirred"

"whirring"

"whirrs"

"whirry"

"whirs"

"whish"

"whished"

"whishes"

"whishing"

"whisht"

"whishted"

"whishts"

"whisk"

"whisked"

"whisker"

"whiskered"

"whiskers"

"whiskery"

"whiskey"

"whiskeys"

"whiskies"

"whisking"

"whisks"

"whisky"

"whisper"

"whispered"

"whispering"

"whisperings"

"whispers"

"whispery"

"whist"

"whisted"

"whisting"

"whistle"

"whistled"

"whistler"

"whistlers"

"whistles"

"whistling"

"whists"

"whit"

"white"

"whitecap"

"whitecapper"

"whitecapping"

"whitecaps"

"whitecomb"

"whited"

"whitefish"

"whitefishes"

"whitehall"

"whitehead"

"whiteheads"

"whitely"

"whiten"

"whitened"

"whitener"

"whiteners"

"whiteness"

"whitening"

"whitens"

"whiteout"

"whiteouts"

"whiter"

"whites"

"whitest"

"whitewall"

"whitewalls"

"whitewash"

"whitewashed"

"whitewashes"

"whitewashing"

"whitey"

"whiteys"

"whitfield"

"whither"

"whithersoever"

"whities"

"whiting"

"whitings"

"whitish"

"whitishness"

"whitlow"

"whitlows"

"whitman"

"whitney"

"whits"

"whitsunday"

"whitter"

"whittle"

"whittled"

"whittler"

"whittlers"

"whittles"

"whittling"

"whity"

"whiz"

"whizbang"

"whizbangs"

"whizz"

"whizzed"

"whizzer"

"whizzers"

"whizzes"

"whizzing"

"who"

"whoa"

"whodunit"

"whodunits"

"whoever"

"whole"

"wholehearted"

"wholeheartedly"

"wholeheartedness"

"wholely"

"wholeness"

"wholes"

"wholesale"

"wholesaled"

"wholesaler"

"wholesalers"

"wholesales"

"wholesaling"

"wholesome"

"wholesomely"

"wholesomeness"

"wholewheat"

"wholism"

"wholisms"

"wholly"

"whom"

"whomever"

"whomp"

"whomped"

"whomping"

"whomps"

"whomso"

"whomsoever"

"whoop"

"whooped"

"whoopee"

"whoopees"

"whooper"

"whoopers"

"whooping"

"whoopla"

"whooplas"

"whoops"

"whoosh"

"whooshed"

"whooshes"

"whooshing"

"whoosis"

"whopped"

"whopper"

"whoppers"

"whopping"

"whops"

"whore"

"whored"

"whoredom"

"whoredoms"

"whorehouse"

"whoremaster"

"whores"

"whoreson"

"whoresons"

"whoring"

"whorish"

"whorl"

"whorled"

"whorls"

"whortle"

"whose"

"whosis"

"whoso"

"whosoever"

"whump"

"whumped"

"whumping"

"whumps"

"why"

"whys"

"wichita"

"wick"

"wicked"

"wickeder"

"wickedest"

"wickedly"

"wickedness"

"wicker"

"wickers"

"wickerwork"

"wicket"

"wickets"

"wicking"

"wickings"

"wickiup"

"wickiups"

"wicks"

"wickyup"

"widder"

"widders"

"widdies"

"widdle"

"widdled"

"widdles"

"widdling"

"wide"

"widely"

"widemouthed"

"widen"

"widened"

"widener"

"wideners"

"wideness"

"widening"

"widens"

"wider"

"wides"

"widespread"

"widest"

"widgeon"

"widgeons"

"widget"

"widgets"

"widish"

"widow"

"widowed"

"widower"

"widowered"

"widowerhood"

"widowers"

"widowhood"

"widowing"

"widows"

"width"

"widths"

"widthway"

"wiedersehen"

"wield"

"wielded"

"wielder"

"wielders"

"wieldier"

"wieldiest"

"wielding"

"wields"

"wieldy"

"wiener"

"wieners"

"wienie"

"wienies"

"wierd"

"wife"

"wifed"

"wifedom"

"wifedoms"

"wifehood"

"wifehoods"

"wifeless"

"wifelier"

"wifeliest"

"wifely"

"wifes"

"wifi"

"wifing"

"wig"

"wigeon"

"wigeons"

"wigged"

"wiggeries"

"wiggery"

"wigging"

"wiggle"

"wiggled"

"wiggler"

"wigglers"

"wiggles"

"wigglier"

"wiggliest"

"wiggling"

"wiggly"

"wight"

"wight loss"

"wights"

"wigless"

"wiglet"

"wiglets"

"wiglike"

"wigmaker"

"wigmakers"

"wigs"

"wigwag"

"wigwagged"

"wigwagging"

"wigwags"

"wigwam"

"wigwams"

"Wiki"

"wikiups"

"wilco"

"wild"

"wildcard"

"wildcat"

"wildcats"

"wildcatted"

"wildcatter"

"wildcatting"

"wildebeest"

"wildebeests"

"wilder"

"wildering"

"wilderness"

"wildernesses"

"wilders"

"wildest"

"wildfire"

"wildfires"

"wildfowl"

"wildfowls"

"wilding"

"wildish"

"wildlife"

"wildling"

"wildlings"

"wildly"

"wildness"

"wilds"

"wildwood"

"wildwoods"

"wile"

"wiled"

"wiles"

"wilful"

"wilfully"

"wilfulness"

"wilier"

"wiliest"

"wilily"

"wiliness"

"wiling"

"will"

"willable"

"willed"

"willer"

"willers"

"willets"

"willful"

"willfully"

"willfulness"

"william"

"williams"

"willied"

"willies"

"willing"

"willinger"

"willingest"

"willingly"

"willingness"

"williwaw"

"williwaws"

"willow"

"willowed"

"willowers"

"willowier"

"willowiest"

"willowing"

"willows"

"willowy"

"willpower"

"wills"

"willy"

"wilson"

"wilt"

"wilted"

"wilting"

"wilts"

"wily"

"wimble"

"wimbles"

"wimple"

"wimpled"

"wimples"

"win"

"wince"

"winced"

"wincer"

"wincers"

"winces"

"winceys"

"winch"

"winched"

"wincher"

"winchers"

"winches"

"winching"

"wincing"

"wind"

"wind-up"

"windable"

"windage"

"windages"

"windbag"

"windbags"

"windblown"

"windbreak"

"windbreaks"

"windburn"

"windburned"

"windburns"

"windburnt"

"windchill"

"winded"

"winder"

"winders"

"windfall"

"windfalls"

"windflower"

"windflowers"

"windier"

"windiest"

"windily"

"windiness"

"winding"

"windings"

"windjammer"

"windjammers"

"windlass"

"windlassed"

"windlasses"

"windless"

"windmill"

"windmilled"

"windmills"

"window"

"windowed"

"windowing"

"windowless"

"windowpane"

"windowpanes"

"windows"

"windowsill"

"windpipe"

"windpipes"

"windproof"

"windrow"

"windrowing"

"windrows"

"winds"

"windscreen"

"windshield"

"windshields"

"windsock"

"windsocks"

"windsor"

"windstorm"

"windstorms"

"windsurf"

"windswept"

"windup"

"windups"

"windward"

"windwards"

"windy"

"wine"

"wined"

"winegrower"

"winepress"

"wineries"

"winery"

"wines"

"wineshop"

"wineshops"

"wineskin"

"wineskins"

"winesop"

"winesops"

"winey"

"wing"

"wingback"

"wingbacks"

"wingding"

"wingdings"

"winged"

"wingedly"

"wingers"

"wingier"

"winging"

"wingless"

"winglet"

"winglets"

"wingman"

"wingmen"

"wingover"

"wingovers"

"wings"

"wingspan"

"wingspans"

"wingspread"

"wingspreads"

"wingy"

"winier"

"winiest"

"wining"

"winish"

"wink"

"winked"

"winker"

"winkers"

"winking"

"winkle"

"winkled"

"winkles"

"winkling"

"winks"

"winless"

"winnable"

"winned"

"winner"

"winners"

"winning"

"winningly"

"winnings"

"winnipeg"

"winnow"

"winnowed"

"winnower"

"winnowers"

"winnowing"

"winnows"

"wino"

"winoes"

"winos"

"wins"

"winslow"

"winsome"

"winsomely"

"winsomeness"

"winsomer"

"winsomest"

"winter"

"wintered"

"winterer"

"winterers"

"wintergreen"

"wintergreens"

"winterier"

"winteriest"

"wintering"

"winterization"

"winterize"

"winterized"

"winterizes"

"winterizing"

"winterkill"

"winterkilled"

"winterkilling"

"winterkills"

"winterly"

"winters"

"wintertide"

"wintertime"

"wintery"

"wintling"

"wintrier"

"wintriest"

"wintrily"

"wintry"

"winy"

"wipe"

"wiped"

"wipeout"

"wipeouts"

"wiper"

"wipers"

"wipes"

"wiping"

"wirable"

"wire"

"wired"

"wiredraw"

"wiredrawn"

"wiredraws"

"wiredrew"

"wirehair"

"wirehaired"

"wirehairs"

"wireless"

"wirelessed"

"wirelesses"

"wireman"

"wiremen"

"wirephoto"

"wirephotos"

"wirepuller"

"wirepullers"

"wirepulling"

"wirer"

"wirers"

"wires"

"wiretap"

"wiretapped"

"wiretapper"

"wiretappers"

"wiretapping"

"wiretaps"

"wireway"

"wireways"

"wirework"

"wireworks"

"wireworm"

"wireworms"

"wirier"

"wiriest"

"wirily"

"wiriness"

"wiring"

"wirings"

"wiry"

"wis"

"wisconsin"

"wisconsinite"

"wisconsinites"

"wisdom"

"wisdoms"

"wise"

"wiseacre"

"wiseacres"

"wisecrack"

"wisecracked"

"wisecracker"

"wisecrackers"

"wisecracking"

"wisecracks"

"wised"

"wiseliest"

"wisely"

"wiseness"

"wiser"

"wises"

"wisest"

"wish"

"wishbone"

"wishbones"

"wished"

"wisher"

"wishers"

"wishes"

"wishful"

"wishfully"

"wishfulness"

"wishing"

"wishless"

"wishy"

"wising"

"wisp"

"wisped"

"wispier"

"wispiest"

"wispily"

"wisping"

"wispish"

"wisps"

"wispy"

"wisteria"

"wisterias"

"wistful"

"wistfully"

"wistfulness"

"wisting"

"wists"

"wit"

"witch"

"witchcraft"

"witched"

"witcheries"

"witchery"

"witches"

"witchier"

"witchiest"

"witching"

"witchings"

"witchy"

"with"

"withal"

"withdraw"

"withdrawable"

"withdrawal"

"withdrawals"

"withdrawer"

"withdrawing"

"withdrawn"

"withdrawnness"

"withdraws"

"withdrew"

"withe"

"withed"

"wither"

"withered"

"witherer"

"witherers"

"withering"

"witheringly"

"withers"

"withes"

"withheld"

"withhold"

"withholder"

"withholders"

"withholding"

"withholdings"

"withholds"

"withier"

"withies"

"within"

"withing"

"withins"

"without"

"without needing a network"

"withouts"

"withstand"

"withstanding"

"withstands"

"withstood"

"withy"

"witless"

"witlessly"

"witlessness"

"witling"

"witlings"

"witness"

"witnessable"

"witnessed"

"witnesser"

"witnessers"

"witnesses"

"witnessing"

"wits"

"witted"

"wittedness"

"witticism"

"witticisms"

"wittier"

"wittiest"

"wittily"

"wittiness"

"witting"

"wittingly"

"wittings"

"witty"

"wive"

"wived"

"wiver"

"wivern"

"wiverns"

"wivers"

"wives"

"wiving"

"wiz"

"wizard"

"wizardly"

"wizardries"

"wizardry"

"wizards"

"wizen"

"wizened"

"wizening"

"wizens"

"wizes"

"wk"

"wkly"

"woad"

"woaded"

"woads"

"woald"

"woalds"

"wobble"

"wobbled"

"wobbler"

"wobblers"

"wobbles"

"wobblier"

"wobblies"

"wobbliest"

"wobbliness"

"wobbling"

"wobbly"

"wobegone"

"woe"

"woebegone"

"woeful"

"woefuller"

"woefullest"

"woefully"

"woefulness"

"woeness"

"woenesses"

"woes"

"woesome"

"woful"

"wofully"

"wok"

"woke"

"woken"

"woks"

"wold"

"wolds"

"wolf"

"wolfed"

"wolfer"

"wolfers"

"wolfhound"

"wolfhounds"

"wolfing"

"wolfish"

"wolfram"

"wolframs"

"wolfs"

"wolfsbane"

"wolfsbanes"

"wolver"

"wolverine"

"wolverines"

"wolvers"

"wolves"

"woman"

"womaned"

"womanhood"

"womanish"

"womanize"

"womanized"

"womanizer"

"womanizers"

"womanizes"

"womanizing"

"womankind"

"womanlier"

"womanliest"

"womanlike"

"womanliness"

"womanly"

"womans"

"womb"

"wombat"

"wombats"

"wombed"

"wombier"

"wombs"

"womby"

"women"

"women's health"

"womenfolk"

"won"

"wonder"

"wondered"

"wonderer"

"wonderers"

"wonderful"

"wonderfully"

"wonderfulness"

"wondering"

"wonderingly"

"wonderland"

"wonderlands"

"wonderment"

"wonders"

"wondrous"

"wondrously"

"wondrousness"

"wonkier"

"wonky"

"wont"

"wonted"

"wontedly"

"wonting"

"wonton"

"wontons"

"wonts"

"woo"

"wood"

"woodbin"

"woodbine"

"woodbines"

"woodbins"

"woodblock"

"woodblocks"

"woodbox"

"woodcarver"

"woodcarvers"

"woodcarving"

"woodcarvings"

"woodchopper"

"woodchuck"

"woodchucks"

"woodcock"

"woodcocks"

"woodcraft"

"woodcut"

"woodcuts"

"woodcutter"

"woodcutters"

"woodcutting"

"wooded"

"wooden"

"woodener"

"woodenest"

"woodenly"

"woodenness"

"woodenware"

"woodgraining"

"woodhen"

"woodier"

"woodiest"

"woodiness"

"wooding"

"woodland"

"woodlander"

"woodlands"

"woodlore"

"woodlot"

"woodlots"

"woodman"

"woodmen"

"woodnote"

"woodnotes"

"woodpecker"

"woodpeckers"

"woodpile"

"woodpiles"

"woodruff"

"woodruffs"

"woods"

"woodshed"

"woodsheds"

"woodsier"

"woodsiest"

"woodsman"

"woodsmen"

"woodsy"

"woodward"

"woodwax"

"woodwind"

"woodwinds"

"woodwork"

"woodworker"

"woodworking"

"woodworks"

"woodworm"

"woodworms"

"woody"

"wooed"

"wooer"

"wooers"

"woof"

"woofed"

"woofer"

"woofers"

"woofing"

"woofs"

"wooing"

"wooingly"

"wool"

"wooled"

"woolen"

"woolens"

"wooler"

"woolers"

"woolgathering"

"woolie"

"woolier"

"woolies"

"wooliest"

"woollen"

"woollens"

"woollier"

"woollies"

"woolliest"

"woolliness"

"woolly"

"woolman"

"woolmen"

"woolpack"

"wools"

"woolsack"

"woolshed"

"woolskin"

"woolsorter"

"woolworth"

"wooly"

"woomera"

"woops"

"woos"

"woosh"

"wooshed"

"wooshes"

"wooshing"

"woozier"

"wooziest"

"woozily"

"wooziness"

"woozy"

"wop"

"wops"

"worcester"

"word"

"wordage"

"wordages"

"wordbook"

"wordbooks"

"worded"

"wordier"

"wordiest"

"wordily"

"wordiness"

"wording"

"wordings"

"wordless"

"wordlessly"

"wordperfect"

"wordplay"

"wordplays"

"wordprocessors"

"words"

"wordstar"

"wordy"

"wore"

"work"

"work timer"

"workability"

"workable"

"workableness"

"workaday"

"workaholic"

"workaholics"

"workaholism"

"workbag"

"workbags"

"workbench"

"workbenches"

"workboat"

"workbook"

"workbooks"

"workbox"

"workboxes"

"workday"

"workdays"

"worked"

"worker"

"workers"

"workfolk"

"workhand"

"workhorse"

"workhorses"

"workhouse"

"workhouses"

"working"

"workingman"

"workingmen"

"workings"

"workingwoman"

"workingwomen"

"workless"

"workload"

"workloads"

"workman"

"workmanlike"

"workmanship"

"workmaster"

"workmen"

"workout"

"workouts"

"workplace"

"workroom"

"workrooms"

"works"

"workshop"

"workshops"

"workstation"

"workstations"

"worktable"

"worktables"

"workup"

"workups"

"workweek"

"workweeks"

"workwoman"

"workwomen"

"world"

"worldbeater"

"worldbeaters"

"worldlier"

"worldliest"

"worldliness"

"worldling"

"worldlings"

"worldly"

"worlds"

"worldwide"

"worm"

"wormed"

"wormer"

"wormers"

"wormhole"

"wormholes"

"wormier"

"wormiest"

"worming"

"wormish"

"worms"

"wormwood"

"wormwoods"

"wormy"

"worn"

"wornness"

"wornout"

"worried"

"worriedly"

"worrier"

"worriers"

"worries"

"worriment"

"worriments"

"worrisome"

"worrisomely"

"worrit"

"worry"

"worrying"

"worrywart"

"worrywarts"

"worse"

"worsen"

"worsened"

"worsening"

"worsens"

"worser"

"worses"

"worship"

"worshiped"

"worshiper"

"worshipers"

"worshipful"

"worshipfully"

"worshiping"

"worshipped"

"worshipper"

"worshippers"

"worshipping"

"worships"

"worst"

"worsted"

"worsteds"

"worsting"

"worsts"

"wort"

"worth"

"worthed"

"worthful"

"worthier"

"worthies"

"worthiest"

"worthily"

"worthiness"

"worthing"

"worthless"

"worthlessly"

"worthlessness"

"worths"

"worthwhile"

"worthy"

"worts"

"wots"

"wotted"

"wotting"

"would"

"wouldest"

"wouldst"

"wound"

"wounded"

"wounding"

"woundingly"

"wounds"

"wove"

"woven"

"wow"

"wowed"

"wowing"

"wows"

"wowser"

"wowsers"

"wpm"

"wrack"

"wracked"

"wrackful"

"wracking"

"wracks"

"wraith"

"wraiths"

"wrang"

"wrangle"

"wrangled"

"wrangler"

"wranglers"

"wrangles"

"wrangling"

"wrap"

"wraparound"

"wraparounds"

"wrapped"

"wrapper"

"wrappers"

"wrapping"

"wrappings"

"wraps"

"wrapt"

"wrasse"

"wrasses"

"wrastle"

"wrastled"

"wrastles"

"wrath"

"wrathed"

"wrathful"

"wrathfully"

"wrathfulness"

"wrathier"

"wrathiest"

"wrathily"

"wrathing"

"wraths"

"wrathy"

"wreak"

"wreaked"

"wreaker"

"wreakers"

"wreaking"

"wreaks"

"wreath"

"wreathe"

"wreathed"

"wreathes"

"wreathing"

"wreaths"

"wreathy"

"wreck"

"wreckage"

"wreckages"

"wrecked"

"wrecker"

"wreckers"

"wreckful"

"wrecking"

"wreckings"

"wrecks"

"wren"

"wrench"

"wrenched"

"wrenches"

"wrenching"

"wrens"

"wrest"

"wrested"

"wrester"

"wresters"

"wresting"

"wrestle"

"wrestled"

"wrestler"

"wrestlers"

"wrestles"

"wrestling"

"wrests"

"wretch"

"wretched"

"wretcheder"

"wretchedly"

"wretchedness"

"wretches"

"wried"

"wrier"

"wries"

"wriest"

"wriggle"

"wriggled"

"wriggler"

"wrigglers"

"wriggles"

"wrigglier"

"wriggliest"

"wriggling"

"wriggly"

"wright"

"wrights"

"wrigley"

"wring"

"wringed"

"wringer"

"wringers"

"wringing"

"wrings"

"wrinkle"

"wrinkled"

"wrinkles"

"wrinklier"

"wrinkliest"

"wrinkling"

"wrinkly"

"wrist"

"wristband"

"wristbands"

"wristdrop"

"wristiest"

"wristlet"

"wristlets"

"wrists"

"wristwatch"

"wristwatches"

"wristy"

"writ"

"writable"

"write"

"writeoff"

"writeoffs"

"writer"

"writers"

"writes"

"writhe"

"writhed"

"writher"

"writhers"

"writhes"

"writhing"

"writhingly"

"writing"

"writings"

"writs"

"written"

"wrong"

"wrongdoer"

"wrongdoers"

"wrongdoing"

"wronged"

"wronger"

"wrongers"

"wrongest"

"wrongful"

"wrongfully"

"wrongfulness"

"wrongheaded"

"wrongheadedly"

"wrongheadedness"

"wronging"

"wrongly"

"wrongness"

"wrongs"

"wrote"

"wroth"

"wrothful"

"wrought"

"wrung"

"wry"

"wryer"

"wryest"

"wrying"

"wryly"

"wryneck"

"wrynecks"

"wryness"

"wrynesses"

"wurst"

"wursts"

"wurzel"

"WW points"

"wye"

"wyes"

"wyn"

"wyoming"

"wyomingite"

"wyvern"

"wyverns"

"xanthate"

"xanthates"

"xanthic"

"xanthin"

"xanthine"

"xanthippe"

"xanthochroid"

"xanthoma"

"xanthometer"

"xanthophyll"

"xanthous"

"xebec"

"xebecs"

"xenia"

"xenic"

"xenobiologies"

"xenobiology"

"xenocryst"

"xenogamy"

"xenograft"

"xenolith"

"xenolithic"

"xenoliths"

"xenomania"

"xenon"

"xenons"

"xenophobe"

"xenophobes"

"xenophobia"

"xenophobic"

"xeric"

"xeroderma"

"xerographic"

"xerography"

"xerophilous"

"xerophthalmia"

"xerophyte"

"xerosis"

"xerox"

"xeroxed"

"xeroxes"

"xeroxing"

"xii"

"xiii"

"xiphoid"

"xiphoids"

"xiphosuran"

"xiv"

"xix"

"xmas"

"xmases"

"XML"

"xvi"

"xvii"

"xviii"

"xx"

"xxi"

"xxii"

"xxiii"

"xxiv"

"xxv"

"xxx"

"xylan"

"xylem"

"xylems"

"xylene"

"xylidine"

"xylitol"

"xylograph"

"xylography"

"xyloid"

"xylology"

"xylometer"

"xylophagous"

"xylophone"

"xylophones"

"xylophonist"

"xylophonists"

"xylose"

"xylotomy"

"xyster"

"xysters"

"xysts"

"xystus"

"yabber"

"yabbers"

"yacht"

"yachted"

"yachter"

"yachters"

"yachting"

"yachtings"

"yachtman"

"yachtmen"

"yachts"

"yachtsman"

"yachtsmanship"

"yachtsmen"

"yachtswoman"

"yachtswomen"

"yack"

"yacked"

"yacking"

"yacks"

"yad"

"yag"

"yahoo"

"yahooism"

"yahooisms"

"yahoos"

"yahweh"

"yak"

"yakked"

"yakking"

"yaks"

"yale"

"yam"

"yamen"

"yamens"

"yammer"

"yammered"

"yammerer"

"yammerers"

"yammering"

"yammers"

"yams"

"yamun"

"yamuns"

"yang"

"yangtze"

"yank"

"yanked"

"yankee"

"yankees"

"yanking"

"yanks"

"yanqui"

"yanquis"

"yap"

"yapped"

"yapper"

"yappers"

"yapping"

"yaps"

"yard"

"yardage"

"yardages"

"yardarm"

"yardarms"

"yardbird"

"yardbirds"

"yarded"

"yarding"

"yardland"

"yardman"

"yardmaster"

"yardmasters"

"yardmen"

"yards"

"yardstick"

"yardsticks"

"yare"

"yarely"

"yarer"

"yarest"

"yarmulke"

"yarmulkes"

"yarn"

"yarned"

"yarning"

"yarns"

"yarrow"

"yarrows"

"yashmac"

"yashmak"

"yashmaks"

"yasmaks"

"yaw"

"yawed"

"yawing"

"yawl"

"yawled"

"yawling"

"yawls"

"yawn"

"yawned"

"yawner"

"yawners"

"yawning"

"yawns"

"yawp"

"yawped"

"yawper"

"yawpers"

"yawping"

"yawps"

"yaws"

"yay"

"ycleped"

"yclept"

"yds"

"ye"

"yea"

"yeah"

"year"

"yearbook"

"yearbooks"

"yearlies"

"yearling"

"yearlings"

"yearlong"

"yearly"

"yearn"

"yearned"

"yearner"

"yearners"

"yearning"

"yearningly"

"yearnings"

"yearns"

"years"

"yeas"

"yeast"

"yeasted"

"yeastier"

"yeastiest"

"yeastily"

"yeasting"

"yeasts"

"yeasty"

"yegg"

"yeggman"

"yeggmen"

"yeggs"

"yell"

"yelled"

"yeller"

"yellers"

"yelling"

"yellow"

"yellowbellied"

"yellowbellies"

"yellowbelly"

"yellowed"

"yellower"

"yellowest"

"yellowing"

"yellowish"

"yellowknife"

"yellowly"

"yellows"

"yellowy"

"yells"

"yelp"

"yelped"

"yelper"

"yelpers"

"yelping"

"yelps"

"yemen"

"yemenite"

"yemenites"

"yen"

"yenned"

"yenning"

"yens"

"yenta"

"yentas"

"yeo"

"yeoman"

"yeomanly"

"yeomanry"

"yeomen"

"yep"

"yerba"

"yerbas"

"yes"

"yeses"

"yeshiva"

"yeshivah"

"yeshivahs"

"yeshivas"

"yeshivoth"

"yessed"

"yesses"

"yessing"

"yester"

"yesterday"

"yesterdays"

"yesteryear"

"yesteryears"

"yet"

"yeti"

"yetis"

"yew"

"yews"

"yex"

"yid"

"yids"

"yield"

"yielded"

"yielder"

"yielders"

"yielding"

"yields"

"yin"

"yins"

"yip"

"yipe"

"yipes"

"yipped"

"yippee"

"yippie"

"yippies"

"yipping"

"yips"

"ymca"

"yod"

"yodel"

"yodeled"

"yodeler"

"yodelers"

"yodeling"

"yodelled"

"yodeller"

"yodellers"

"yodelling"

"yodels"

"yodhs"

"yodle"

"yodled"

"yodler"

"yodlers"

"yodles"

"yodling"

"yoga"

"yoga nidra"

"Yoga poses"

"yoga positions"

"Yoga teacher"

"yoga tunes"

"yogas"

"yogee"

"yogees"

"yoghourts"

"yoghs"

"yoghurt"

"yoghurts"

"yogi"

"yogic"

"yogin"

"yogini"

"yoginis"

"yogins"

"yogis"

"yogurt"

"yogurts"

"yoicks"

"yok"

"yoke"

"yoked"

"yokel"

"yokeless"

"yokelish"

"yokels"

"yokemate"

"yokemates"

"yokes"

"yoking"

"yokohama"

"yolk"

"yolked"

"yolkier"

"yolks"

"yolky"

"yon"

"yond"

"yonder"

"yoni"

"yonis"

"yonker"

"yonkers"

"yore"

"yores"

"york"

"yorker"

"yorkers"

"yosemite"

"you"

"young"

"younger"

"youngers"

"youngest"

"youngish"

"youngling"

"younglings"

"youngs"

"youngster"

"youngsters"

"youngstown"

"younker"

"younkers"

"your"

"your dream"

"yourn"

"yours"

"yourself"

"yourselves"

"youse"

"youth"

"youthen"

"youthened"

"youthening"

"youthens"

"youthful"

"youthfully"

"youthfulness"

"youths"

"yow"

"yowed"

"yowie"

"yowies"

"yowing"

"yowl"

"yowled"

"yowler"

"yowlers"

"yowling"

"yowls"

"yows"

"yr"

"yrneh"

"yrs"

"ytterbic"

"ytterbium"

"yttria"

"yttric"

"yttrium"

"yttriums"

"yuan"

"yucca"

"yuccas"

"yugoslav"

"yugoslavia"

"yugoslavian"

"yugoslavians"

"yugoslavs"

"yuk"

"yukked"

"yukking"

"yukon"

"yuks"

"yule"

"yules"

"yuletide"

"yuletides"

"yummier"

"yummies"

"yummiest"

"yummy"

"yup"

"yuppie"

"yurt"

"yurts"

"ywca"

"zabaione"

"zabaiones"

"zachariah"

"zaftig"

"zag"

"zagged"

"zagging"

"zags"

"zaire"

"zairian"

"zairians"

"zambezi"

"zambia"

"zambian"

"zambians"

"zanier"

"zanies"

"zaniest"

"zanily"

"zaniness"

"zany"

"zanyish"

"zanzibar"

"zap"

"zapped"

"zapping"

"zaps"

"zarfs"

"zazen"

"zeal"

"zealand"

"zealander"

"zealanders"

"zealot"

"zealotries"

"zealotry"

"zealots"

"zealous"

"zealously"

"zealousness"

"zeals"

"zebeck"

"zebecks"

"zebecs"

"zebra"

"zebraic"

"zebras"

"zebrass"

"zebrasses"

"zebrine"

"zebroid"

"zebu"

"zebus"

"zed"

"zeds"

"zee"

"zees"

"zeins"

"zeiss"

"zeitgeist"

"zek"

"zel"

"zelophobia"

"zemstvos"

"zen"

"Zen music"

"zenana"

"zenanas"

"zendo"

"zenith"

"zenithal"

"zeniths"

"zenography"

"zeolite"

"zephyr"

"zephyrs"

"zeppelin"

"zeppelins"

"zero"

"zeroed"

"zeroes"

"zeroing"

"zeros"

"zest"

"zested"

"zestful"

"zestfully"

"zestfulness"

"zestier"

"zestiest"

"zesting"

"zests"

"zesty"

"zeta"

"zetas"

"zeus"

"zho"

"zig"

"zigged"

"zigging"

"ziggurat"

"ziggurats"

"zigs"

"zigzag"

"zigzagged"

"zigzagging"

"zigzags"

"zikurat"

"zilch"

"zilches"

"zillion"

"zillions"

"zillionth"

"zillionths"

"zimbabwe"

"zinc"

"zincate"

"zinced"

"zincic"

"zincified"

"zincifies"

"zincify"

"zincing"

"zincite"

"zincked"

"zincking"

"zincky"

"zincoid"

"zincous"

"zincs"

"zincy"

"zing"

"zinged"

"zinger"

"zingers"

"zingier"

"zingiest"

"zinging"

"zings"

"zingy"

"zinkify"

"zinky"

"zinnia"

"zinnias"

"zion"

"zionism"

"zionist"

"zionists"

"zip"

"zipped"

"zipper"

"zippered"

"zippering"

"zippers"

"zippier"

"zippiest"

"zipping"

"zippy"

"zips"

"zircon"

"zirconic"

"zirconium"

"zircons"

"zither"

"zitherist"

"zitherists"

"zithern"

"zitherns"

"zithers"

"zitis"

"zizzle"

"zizzled"

"zizzles"

"zizzling"

"zloty"

"zlotys"

"zn"

"zodiac"

"zodiacal"

"zodiacs"

"zoeas"

"zoftig"

"zoiatrics"

"zolotnik"

"zombi"

"zombie"

"zombies"

"zombiism"

"zombiisms"

"zombis"

"zonal"

"zonally"

"zonated"

"zonation"

"zone"

"zoned"

"zoneless"

"zoner"

"zoners"

"zones"

"zonetime"

"zonetimes"

"zoning"

"zonked"

"zoo"

"zooarchaeology"

"zoochemistry"

"zoogenous"

"zoogeographic"

"zoogeographical"

"zoogeographies"

"zoogeography"

"zoogeology"

"zoography"

"zooid"

"zooids"

"zooks"

"zoologic"

"zoological"

"zoologically"

"zoologies"

"zoologist"

"zoologists"

"zoology"

"zoom"

"zoomania"

"zoomanias"

"zoomed"

"zooming"

"zoomorphs"

"zooms"

"zoonomy"

"zoonosology"

"zoons"

"zooparasitic"

"zoopathologies"

"zoopathology"

"zoophiles"

"zoophobia"

"zoophysics"

"zoophysiology"

"zoophyte"

"zoophytes"

"zoophytology"

"zooplankton"

"zoos"

"zoosemiotics"

"zoospores"

"zootaxy"

"zootechnics"

"zori"

"zoroaster"

"zoroastrian"

"zoroastrianism"

"zoroastrians"

"zoster"

"zouave"

"zouaves"

"zounds"

"zowie"

"zoysia"

"zoysias"

"zucchetto"

"zucchettos"

"zucchini"

"zucchinis"

"zug"

"zulu"

"zulus"

"zuni"

"zunis"

"zurich"

"zwieback"

"zwiebacks"

"zygology"

"zygote"

"zygotes"

"zygotic"

"zymase"

"zymogenic"

"zymology"

"zymolysis"

"zymometer"

"zymoplastic"

"zymoscope"

"zymosimeter"

"zymurgy"

"zyzzyva"

"zyzzyvas"
